# A Polychaete’s Powerful Punch: Venom Gland Transcriptomics of *Glycera* Reveals a Complex Cocktail of Toxin Homologs

**DOI:** 10.1093/gbe/evu190

**Published:** 2014-09-05

**Authors:** Björn M. von Reumont, Lahcen I. Campbell, Sandy Richter, Lars Hering, Dan Sykes, Jörg Hetmank, Ronald A. Jenner, Christoph Bleidorn

**Affiliations:** ^1^Department of Life Sciences, The Natural History Museum, London, United Kingdom; ^2^Molecular Evolution and Systematics of Animals, Institute of Biology, University of Leipzig, Germany; ^3^Animal Evolution & Development, Institute of Biology, University of Leipzig, Germany; ^4^Imaging and Analysis Centre, The Natural History Museum, London, United Kingdom; ^5^German Centre for Integrative Biodiversity Research (iDiv) Halle-Jena-Leipzig, Leipzig, Germany

**Keywords:** venomics, venom evolution, Annelida, bloodworms, *Glycera*, ShKT toxin

## Abstract

Glycerids are marine annelids commonly known as bloodworms. Bloodworms have an eversible proboscis adorned with jaws connected to venom glands. Bloodworms prey on invertebrates, and it is known that the venom glands produce compounds that can induce toxic effects in animals. Yet, none of these putative toxins has been characterized on a molecular basis. Here we present the transcriptomic profiles of the venom glands of three species of bloodworm, *Glycera dibranchiata*, *Glycera fallax* and *Glycera tridactyla*, as well as the body tissue of *G. tridactyla*. The venom glands express a complex mixture of transcripts coding for putative toxin precursors. These transcripts represent 20 known toxin classes that have been convergently recruited into animal venoms, as well as transcripts potentially coding for *Glycera-*specific toxins. The toxins represent five functional categories: Pore-forming and membrane-disrupting toxins, neurotoxins, protease inhibitors, other enzymes, and CAP domain toxins. Many of the transcripts coding for putative *Glycera* toxins belong to classes that have been widely recruited into venoms, but some are homologs of toxins previously only known from the venoms of scorpaeniform fish and monotremes (stonustoxin-like toxin), turrid gastropods (turripeptide-like peptides), and sea anemones (gigantoxin I-like neurotoxin). This complex mixture of toxin homologs suggests that bloodworms employ venom while predating on macroscopic prey, casting doubt on the previously widespread opinion that *G. dibranchiata* is a detritivore. Our results further show that researchers should be aware that different assembly methods, as well as different methods of homology prediction, can influence the transcriptomic profiling of venom glands.

## Introduction

Complex proteinaceous venoms have evolved convergently in many animal groups, with primary roles in defense, predation, and competition (Fry et al. 2009; Casewell et al. 2013). Individual venoms can have an extraordinary range of physiological effects depending on their specific composition. The diverse bioactivities of venom cocktails do not only play central roles in securing the ecological and evolutionary success of venomous taxa as different as arthropods, jellyfish, and cone snails, they are also being exploited for important applied uses, such as the development of new drugs and insect-resistant crops (King 2011; King and Hardy 2013). Venomics, the scientific study of venoms, is also yielding insights into general biological questions, such as the evolutionary importance of orphan genes, the evolution of neofunctionalization of duplicate genes, and the evolutionary pressures regulating the optimal use of metabolically expensive resources such as venoms (Khalturin et al. 2009; Chang and Duda 2012; Morgenstern and King 2013).

Rapid technological advances brought by next generation sequencing (NGS) platforms and improvements in mass spectrometry techniques are currently unlocking transcriptomic, genomic, and proteomic resources for venomics research at an unparalleled rate and in unprecedented depth (Favreau et al. 2006; Terrat et al. 2012; Aird et al. 2013; Francischetti et al. 2013; Vonk et al. 2013; von Reumont et al. 2014). These approaches are generating a host of new insights into the venom composition of poorly studied taxa such as bats, echidnas, and remipede crustaceans, while at the same time providing more detailed data on venom complexity in better studied taxa (Terrat et al. 2012; Francischetti et al. 2013; He et al. 2013; Wong et al. 2013; von Reumont et al. 2014). All these studies facilitate further insights into venomics’ central theme: The convergent recruitment of protein families into venoms (Fry et al. 2009; Casewell et al. 2013).

However, it is important that these technological advances are based on a broad and solid empirical foundation so that taxon- and method-specific peculiarities can be distinguished from general insights into the composition and biology of venoms. In this respect Annelida is a promising group for gaining new insights into the composition, biology, and evolution of venoms.

Annelida currently comprise around 17,000 described species that are classified into more than a hundred families, reflecting the huge morphological diversity of this animal phylum (Zhang 2011). Given the extreme disparity of annelid life styles it may be no surprise that several annelid taxa use noxious substances to defend themselves against predators and parasites, or to secure prey. For instance, fireworms (Amphinomidae) bear irritating calcareous chaetae that upon breaking can release a trimethylammonium compound (complanine) that can cause serious skin inflammation in humans (Nakamura et al. 2008; Borda et al. 2012). Another defensive annelid toxin is a protein called Lysenin that is found in the coelomic fluid of the earthworm *Eisenia fetida* (Sukumwang and Umezawa 2013)*. Eisenia fetida* expels its coelomic fluid when attacked, and the fluid is known to be toxic to vertebrates, probably as a result of the presence of Lysenin (Kobayashi et al. 2001). Lysenin is hemolytic and can lyse cells by inserting into cell membranes, an ability which probably also allows it to play a role in innate immunity as it is able to attack the cell membranes of parasites (De Colibus et al. 2012).

**Fig. 1.—:**
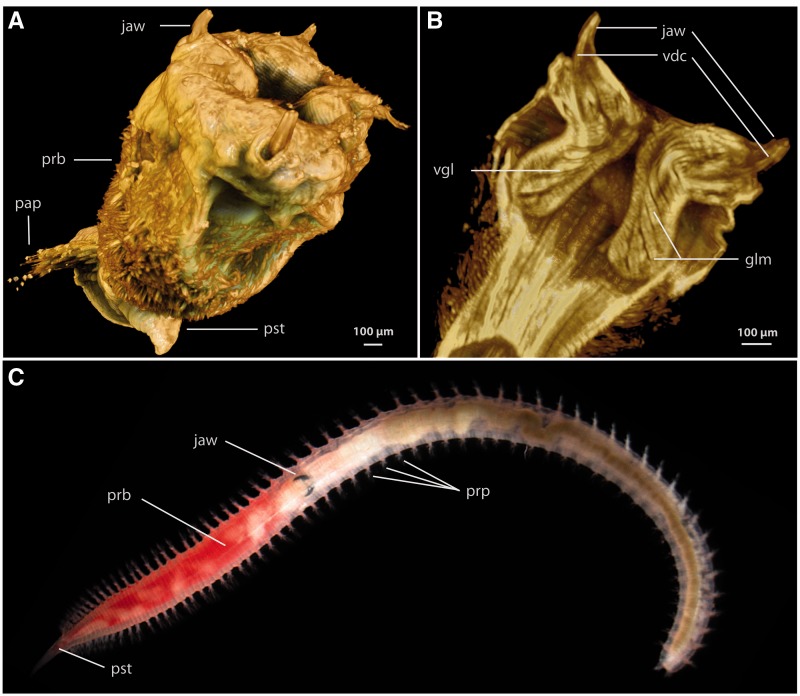
Morphology of *Glycera* and its proboscis and venom apparatus. (*A*) Rendered Micro-CT image of everted proboscis of *Glycera tesselata* showing the four jaws surrounding the terminal mouth. (*B*) Rendered Micro-CT saggital section through the proboscis of *G. tesselata* showing the outlines of two venom glands associated with the jaws. (*C*) General bloodworm morphology illustrated by an unidentified *Glycera* specimen with an inverted proboscis. glm, muscles associated with the venom glands; pap, parapodium; prb, proboscis; pst, prostomium; vgl, venom gland.

In addition to these defensive uses of toxins, two annelid taxa are known to employ toxins for predatory and parasitic purposes. Parasitic leeches express a complex mixture of anticoagulant polypeptides in their salivary glands to assist in blood feeding and to prevent coagulation of blood inside the animal’s crop (Min et al. 2010; Kvist et al. 2014). The glycerids, also known as bloodworms, are the only annelids known to use a complex venom for overwhelming prey. There are at least 42 described species of glycerids (Böggemann 2002) that are characterized by a uniform morphology. All Glyceridae possess a pharynx equipped with four strong jaws which are connected to venom glands (fig. 1). The jaws are largely composed of a melanin-like network, making them highly resistant to abrasion, and each jaw bears a channel and pores for venom release (Moses et al. 2006).

The biological activity of *Glycera* venom has been described on the protein level for two species, *Glycera tridactyla* (formerly *Glycera convoluta*) and *Glycera dibranchiata* (Michel and Robin 1972; Michel and Keil 1975; Manaranche et al. 1980; Bon et al. 1985; Ungerer 2002), revealing both neurotoxic and enzymatic activities. A severe local inflammatory response has been described for humans bitten by these worms (Klawe and Dickie 1957). Moreover, *Glycera* venom can cause cardiac arrest, progressive paralysis, convulsions, and death when injected into crustaceans (Michel and Keil 1975; Manaranche et al. 1980; Bon et al. 1985; Ungerer 2002).

Several interesting neurotoxins have been identified in the venom cocktail of bloodworms. Meunier et al. (2002) demonstrated that one neurotoxin of *G. tridactyla*, which is called glycerotoxin, acts selectively on Ca_v_2.2 channels (N-type Ca^2+^ channels) and is able to stimulate an increase in miniature potentials that is fully reversible. Due to its properties, glycerotoxin has been used as a research tool in several recent studies (Schenning et al. 2006; Meunier et al. 2010). At least one additional component with high toxicity to crustaceans was found in the venom of *G. tridactyla*, but it has not yet been studied further (Bon et al. 1985). An initial study of the venom of *G. dibranchiata* revealed the existence of another toxin that differs in its mode of action from glycerotoxin. Intriguingly, this protein is able to form pores in plasma membranes in a manner similar to α-latrotoxin (Kagan et al. 1982), the potent vertebrate-specific neurotoxin from black widow spider venom (Garb and Hayashi 2013). However, all this work has been conducted on the protein level, often on isolated protein fractions. There is no toxin profile available for any polychaete venom on either the proteomic or transcriptomic level, which hinders identifying and characterizing the toxins responsible for envenomation symptoms.

In this article, we investigate glycerid venom composition by providing the first Illumina-based NGS transcriptomic analyses of glycerid venom glands and body tissue obtained from three species of *Glycera*. We perform phylogenetic analyses of individual toxin families including bloodworm homologs to illuminate their evolutionary relationships and possible evolutionary origins. Additionally, we present a methodological investigation of the difficulties of profiling the expression of putative toxin transcripts with transcriptomic techniques. We pay special attention to the effects of using different methods of transcriptome assembly and different strategies to identify putative venom toxins.

Readers should keep in mind that our transcriptomic approach identifies putative toxin precursor transcripts, and that proteomic and functional data need to be collected in order to validate whether the transcripts indeed code for active venom toxins.

## Materials and Methods

### Specimen Collection and Dissection

Several specimens of *G. tridactyla* Schmarda, 1861 and *Glycera fallax* Quatrefages, 1850 were collected from sandy intertidal flats at Roscoff, France. *Glycera dibranchiata* Ehlers, 1868 specimens were obtained from the Marine Biological Laboratory in Woods Hole, USA. Venom glands were dissected from individuals of all three species. Samples of gland tissue of *G. tridactyla* and *G. fallax* were preserved in RNAlater (Applied Biosystems, Warrington, UK). Additionally, a sample of whole pharynx tissue without venom glands, but composed of epidermal, muscle, nervous system and digestive system tissues was preserved in RNAlater (Applied Biosystems) from *G. tridactyla* to cover also nonvenom gland-related transcripts. Venom glands of one specimen of *G. dibranchiata* were dissected from fresh material and directly used for total RNA extraction. All RNAlater preserved samples were stored for a few days at 4 °C and transferred then to −80 °C.

### RNA Extraction, Library Reconstruction, and Illumina Sequencing

Total RNA was extracted from all samples by homogenization and cell lysis using TRIzol (Invitrogen, Carlsbad, CA). Further sample processing, library preparation and TruSeq RNA sequencing (one lane) of the *G. dibranchiata* sample, as well as quality control and base calling were performed at the GenePool genomics facility of the University of Edinburgh by dedicated technical staff. For the other two species, total RNA was purified using the RNeasy MinElute Cleanup Kit (Qiagen, Hilden, Germany). Afterwards, mRNA was isolated from total RNA using oligo(dT) beads and then fragmented using divalent cationic ions. First and second strand cDNA syntheses were performed by applying SuperScript II, RNase H and DNA polymerase I reactions, respectively, according to the manufacturer’s protocols (Invitrogen). The library preparations of *G. tridactyla* and *G. fallax* were performed according to the Illumina multiplex protocol starting with the blunt end repair described by Meyer and Kircher (2010) with one modification. Instead of the unmodified P7 Illumina adapter mentioned therein, a biotinylated P7 adapter was used to immobilize the libraries by binding to streptavidin beads. The libraries were sequenced (101 bp, paired-end) on the Genome Analyzer IIx platform (Illumina, San Diego) at the Max Planck Institute for Evolutionary Anthropology (Leipzig, Germany).

### Sequence Assembly and Processing

The raw sequencing data were processed with IBIS 1.1.2 (Kircher et al. 2009) to split up the reads according to their indices and to clip off adapter sequences. Reads below a certain threshold (quality filter 15: 95% of the nucleotides of the read with a Phred score above 15; quality filter 20: 95% of the nucleotides of the read with a Phred score above 20) were removed by using the program ConDeTri (Smeds and Kunstner 2011). To retain the highest transcript diversity, the most thorough sequence analyses were conducted using filter 15 settings. Subsequently, sequence reads were assembled de novo using IDBA-tran v1.1.1 (Peng et al. 2013) and CLC Genomics Workbench v5.5.x (CLC bio, Aarhus, Denmark). IDBA-tran assemblies were performed using an initial kmer size of 20, an iteration size of 5, and a maximum *k*-mer size of 60. The option to keep more than one putative isoform per transcript was switched off. CLC assemblies were conducted using the following parameters: mismatch cost = 3, insertion cost = 3, deletion cost = 3, length fraction = 0.5, similarity fraction = 0.8, minimum contig length = 200, automatic bubble size = yes, automatic word size = yes, perform scaffolding = yes. N50 weighted median statistics were calculated for all assemblies (Earl et al. 2011), see supplementary figures S6 and S7, Supplementary Material online. Comparing the assemblies, IDBA-tran assemblies were chosen as the basis for our analyses, based on both the generally longer average and maximum contig lengths.

### Identification and Annotation of Putative Venom Protein Contigs

To identify transcripts for candidate venom proteins, we used an updated customized bioinformatic pipeline as described in von Reumont et al. (2014). This updated bioinformatic pipeline was implemented in Perl, Python, and shell scripts (scripts and pipeline are provided in the supplementary material, Supplementary Material online) and was used to integrate sequence translation, Basic Local Alignment Search Tool (BLAST) analyses, duplicate BLAST hit identification and sequence retrieval. Secreted proteins (UniProt, location_sl_0243) were used as baits and searched against every 6-frame translated transcriptome by using local BLAST (*e* value ≤ 1e-5). The resulting sequence contig-files were subsequently loaded into BLAST2GO (Conesa and Götz 2008) to obtain BLAST annotation, GO-term annotation, and InterPro ID’s for all of the contigs (see supplementary tables S3–S6, Supplementary Material online). Signal peptides for all sequences were predicted separately from InterPro’s signal peptide prediction feature implemented in Geneious R6 (Biomatters, http://www.geneious.com/, last accessed September 10, 2014) as well as using SignalP 4.1 (applying sensitivity setting of 3.0; see Petersen et al. 2011). Disulfide bridge patterns were predicted with the DBCP web server (Lin and Tseng 2010).

Contigs of all libraries were analyzed for the presence of homologs of venom proteins in a 2-fold strategy. First, matches for venom protein classes identified by InterProScan in BLAST2GO were extracted utilizing a Perl script. Second, HMMER 3 (http://hmmer.org/, last accessed September 10, 2014) was used to train hidden Markov models (HMMs) using -hmmbuild on alignments of known venom protein classes, which included annotated UniProt sequences of all non-*Glycera* sequences (both venomous and nonvenomous species) that were included in our trees. The alignments used to train the HMMER searches can be found in the supplementary material, Supplementary Material online. HMMER searches were then performed using –hmmsearch (default parameters with *e* value threshold of 10e^−^^4^) to identify venom protein classes in our contig-files. Two different strategies were applied to build HMMER profiles for the final HMMER search: 1) Complete sequence alignments and 2) only specific domain regions of the particular proteins were used. Domains, chains, and signalpeptides were identified by the InterProScan function of Geneious R6 and additionally checked by the UniProt annotation. Upon completion of each HMMER analysis, hit sequences identified by the HMMER for both complete and domain profiles were extracted from the respective contig libraries using a Perl script. Transcripts with sequences coding for signal peptides, but for which neither InterProScan nor HMMER provided any annotation may represent novel venom toxins unique for the *Glycera* lineage (see supplementary table S2, Supplementary Material online).

It should be noted that we did not attempt to distinguish between allelic variation and paralogy as causes of the transcript diversity we report here. Our primary interest is to estimate the diversity of putative toxin precursor transcripts as this is likely to be informative about the diversity of toxin proteins in crude venom.

### Phylogenetic Reconstruction of Putative Venom Protein Transcripts

Prior to generating multiple sequence alignments of venom proteins, redundant contig sequences identified by the three independent search strategies (InterProScan, HMMER full alignment, HMMER Domain) were removed. Redundant contigs were removed using an in-house coded Perl script, that according to contig fasta headers, collated identical sequence contigs, and summarized those identical contigs under a single fasta header comprised the search method(s) that identified a particular contig. Sequences were aligned using MAFFT L-INS-i (Katoh and Standley 2013), including UniProt reviewed manually curated venom protein structure constraints (Jungo et al. 2012), if available. Using Geneious, contigs were annotated and sequences in which stop-codons interrupted the open reading frames (potentially representing sequencing artifacts or pseudogenes), or sequence fractions with stop codons located external to the venom domain of interest were removed from downstream alignments. Note that based on available sequence annotations divergent N-terminal and C-terminal regions were clipped from the alignments. This has in some cases resulted in the inclusion of seemingly identical sequences in several of our trees as in those cases distinct and unique sequences were represented only by their conserved domain regions. After choosing the best fitting model for each protein with ProtTest 3 (Darriba et al. 2011), a maximum-likelihood analysis was conducted with raxmlHPC-PTHREADS-SSE3 (Stamatakis and Alachiotis 2010) (-f a, 1,000 bootstrap pseudoreplicates, see figure legends of reconstructed trees and supplementary table S7, Supplementary Material online, for the chosen evolutionary model for each protein). Being most interested in the relationships of the *Glycera* sequences and their nearest relatives, we rooted all but two trees with a sequence from a nonvenomous vertebrate, one tree with a cnidarian (supplementary fig. S14, Supplementary Material online) and one tree with a tunicate (supplementary fig. S15, Supplementary Material online). It should be noted that although our chosen rootings are not ad hoc, they are provisional, and may need to be revised as more taxa are sampled and a more complete sampling of paralogs becomes available. All alignments used for tree reconstructions of putative venom proteins are provided in the supplementary material, Supplementary Material online.

Additional sequences from venomous and nonvenomous taxa were predominantly obtained from UniProt in order to maximize the number of annotated sequences in our analyses. We strove to broadly represent the phylogenetic breadth of Metazoa, including nonbilaterians, deuterostomes, ecdysozoans, and especially lophotrochozoans, including both venomous and nonvenomous taxa to represent transcripts coding for putative toxins and nontoxin homologs.

## Results and Discussion

### Diversity and Molecular Evolution of *Glycera* Venom Toxin Homologs

**Fig. 2.—:**
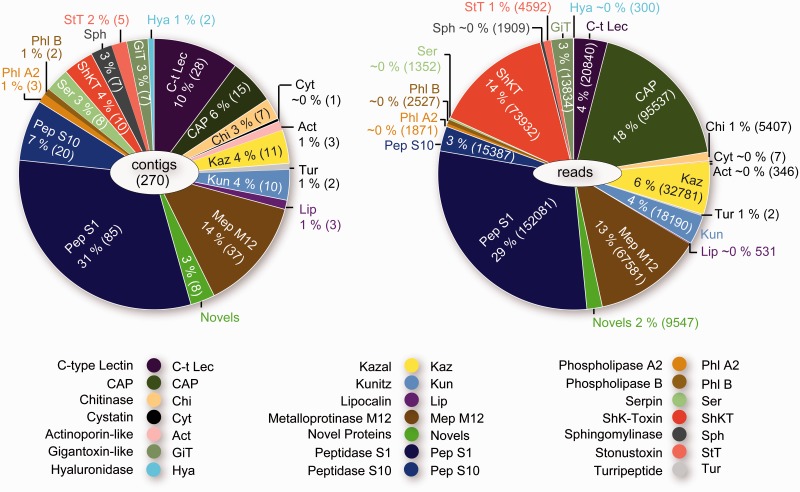
Transcriptomic profile of toxin genes expressed in the venom glands of *Glycera dibranchiata*. (*A*) Contig diversity for the different toxins. (*B*) Abundance of sequence reads for the different toxins. Relative contig diversity and relative abundance of reads are expressed as percentages followed by the numbers of contigs and reads in parentheses. See supplementary figure S1, Supplementary Material online, for the transcriptomic profiles of *G. fallax* and *G. tridactyla*.

The transcriptomes of bloodworm venom glands reveal an unexpectedly complex cocktail of transcripts coding for putative venom protein precursors (fig. 2). The most deeply sequenced library (*G. dibranchiata*) expresses the greatest diversity of putative venom toxin transcripts, representing 20 toxin classes that have been convergently recruited into animal venoms, as well as 12 putative toxins that are possibly unique for bloodworms (see fig. 2 and supplementary tables S1 and S2, Supplementary Material online). For convenience the identifiable putative *Glycera* toxins are classified into five functional categories: 1) Pore-forming and membrane-disrupting toxins: Actinoporin-like toxin, stonustoxin (SNTX)-like toxin, and sphingomyelinase; 2) neurotoxins: ShKT domain neurotoxin, gigantoxin-like neurotoxin, and turripeptide-like neurotoxin; 3) protease inhibitors: Cystatin, Kazal domain protease inhibitor, Kunitz domain protease inhibitor, lipocalin, and serpin; 4) other enzymes: C-type lectin, chitinase, hyaluronidase, phospholipases, peptidase S1, peptidase S10, and metalloproteinase M12; and 5) CAP domain proteins.

The five most abundant toxin homologs transcribed in the venom glands of *G. dibranchiata* are peptidase S1, CAP, ShKT domain neurotoxin, metalloproteinase M12, and Kazal domain protease inhibitor, together representing 80% of putative toxin sequence reads. The venom gland libraries of *G. fallax* and *G. tridactyla* are sequenced at a shallower depth than that of *G. dibranchiata*, and consequently we detected a lower diversity of expressed toxin homologs in these species. The relative abundance of toxin homologs also differs between the species. Although peptidase S1 and Kazal domain protease inhibitors are among the most abundantly expressed toxins homologs in the venom glands of all species, the second most abundantly expressed type of toxin homolog in *G. fallax* is lipocalin, whereas in *G. tridactyla* the second most abundantly expressed toxin category represents a diversity of potentially lineage-specific toxin precursor transcripts without known homologs in other taxa. Potentially novel toxin precursor transcripts are also expressed in the venom glands of the other two species, albeit representing smaller percentages of the total number of toxin transcripts (supplementary fig. S1, Supplementary Material online). In the following, we discuss a selection of the transcripts coding for putative *Glycera* toxins from the major categories defined above. Further discussion is provided in the supplementary material, Supplementary Material online.

#### Pore-Forming and Membrane-Disrupting Toxins: Actinoporin-Like Toxin, SNTX-Like Toxin, and Sphingomyelinase

The intense pain and rapid and prolonged swelling and redness that can result from bloodworm envenomation (Klawe and Dickie 1957) may be mediated by a mix of putative pore-forming and membrane-disrupting toxins expressed in the venom glands of *G. dibranchiata*. These potential toxins may similarly account for the results of activity assays of *G. dibranchiata* venom (Kagan et al. 1982), which have shown that bloodworm venom can induce ion-permeable pores in lipid bilayers. The glands express a diversity of transcripts coding for precursors of toxins that are known to be able to cause cytolysis and hemolysis, and to induce severe pain. Three transcripts are homologous to actinoporins, which are highly conserved 20 kDa cytolytic proteins that belong to the large family of pore-forming toxins (Anderluh and Macek 2003; Garcia-Ortega et al. 2011). They lack cysteine residues and are known to exhibit cytolytic, hemolytic, and nerve stimulatory activities (Bakrac et al. 2008; Bakrac and Anderluh 2010). Although they were initially discovered and known to be highly abundant in sea anemones (Anderluh and Macek 2003), actinoporins have recently also been reported from mollusks (Shiomi et al. 2002; Violette et al. 2012) and chordates (Warren et al. 2008; Garcia-Ortega et al. 2011). A search of GenBank and UniProt also revealed homologs in arthropods. Actinoporins have been shown to be toxic to fish, small mammals, mollusks, and crustaceans (Giese et al. 1996; Garcia et al. 2009; Garcia-Ortega et al. 2011), the latter two of which are known glycerid prey.

**Fig. 3.—:**
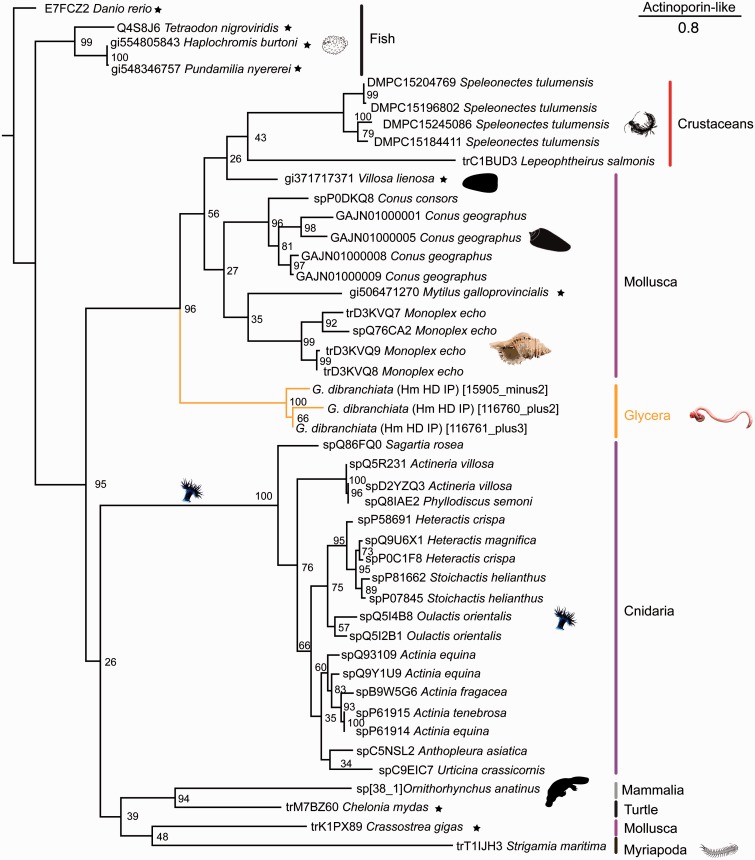
Phylogenetic tree of actinoporin-like sequences. Phylogenetic reconstruction was performed with RAxML (7.4.2 SSE3-PTHREADS) (Stamatakis and Alachiotis 2010), -f a, PROTGAMMAIWAG, 1,000 bootstrap pseudoreplicates. Bootstrap support values given for all nodes. Clade names are indicated by colored vertical bars, common names are given for clades with disputed monophyletic status. *Glycera* and polychaete transcripts are highlighted in orange. Sequences obtained from UniProt are denoted by “tr” or “sp” prefixes. Search strategies that identified a given *Glycera* sequence are labeled behind the species names as follows: Hm, HMMER; HD, HMMERDomain; IP, Interpro. The tree is rooted with a nonvenomous taxon (indicated by a star).

A phylogenetic analysis shows that the three *Glycera* actinoporin-like transcripts group together in a clade with strong support that is sister group to a weakly supported clade of mollusk and arthropod sequences (fig. 3). The bloodworm actinoporin-like sequences contain a conserved “Tryptophan rich region” motif: Ser-x-Pro-Tyr-Asn-x-x-x-Tyr-Ser-Asn-Trp-x-x-Val (Kawashima et al. 2003; Kristan et al. 2009). This motif is known to mediate the binding of actinoporins to cell membranes and facilitate subsequent cytolysis through the recognition of sphingolipids to which they bind preferentially (Mancheno et al. 2003; Bakrac et al. 2008; Garcia-Ortega et al. 2011). Although the venom glands of *G. dibranchiata* express these actinoporin-like toxin transcripts at a relatively low level (fig. 2), actinoporins have been shown to be extremely toxic even at low concentrations (Barlic et al. 2004).

**Fig. 4.—:**
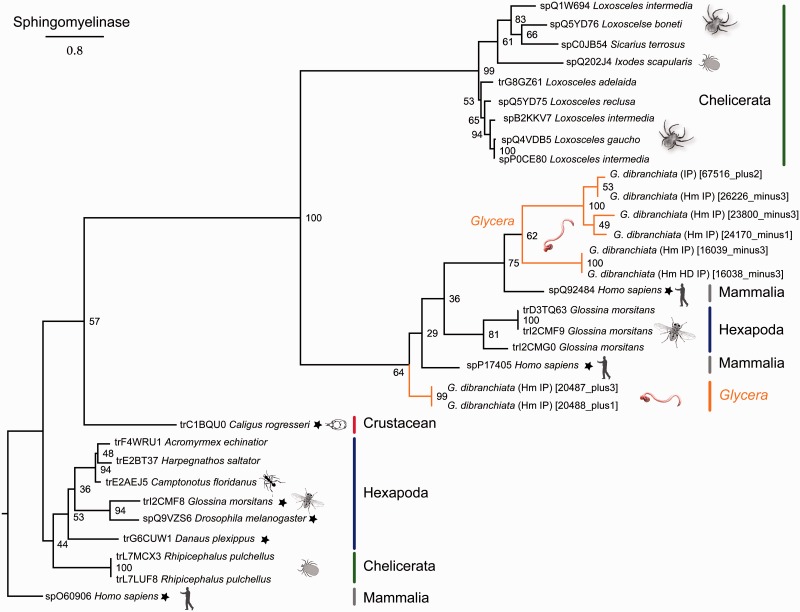
Phylogenetic tree of sphingomyelinase sequences. Phylogenetic reconstruction was performed with RAxML (7.4.2 SSE3-PTHREADS) (Stamatakis and Alachiotis 2010), -f a, PROTGAMMAIWAGF, 1,000 bootstrap pseudoreplicates. Bootstrap support values given for all nodes. See the legend of figure 3 for further information.

Pore formation by actinoporins may be enhanced by the presence of sphingomyelinase, a membrane-disrupting toxin that has an affinity for the sphingomyelin in cell membranes. *Glycera dibranchiata* venom glands express eight distinct sphingomyelinase transcripts, which are placed in two clades inside a large clade of acidic sphingomyelinases (fig. 4). Acidic sphingomyelinases function optimally at lower pH, and they disrupt cell membranes by hydrolyzing sphingolipids. Interestingly, sphingomyelinases have not been widely recruited into animal venoms. The best known example is sphingomyelinase D, which is found in the venom of sicariid spiders (Binford and Wells 2003; Fernandes-Pedrosa et al. 2008; Binford et al. 2009). It is insecticidal (Zobel-Thropp et al. 2012) and responsible for dermonecrosis in envenomed mammals. However, sphingomyelinases have also been described from the sialome of ticks and the tsetse fly, and sphingomyelinase B has recently been detected in the venom of a hydrozoan jellyfish (Alarcon-Chaidez et al. 2009; Alves-Silva et al. 2010; Weston et al. 2013).

The third and most highly expressed pore-forming toxin homolog in *Glycera* venom glands is represented by SNTX-like transcripts. SNTXs and the related verrucotoxins, neoverrucotoxins, and trachynilysin are nonenzymatic cytolytic toxins that have been identified in the venom of a diversity of scorpaeniform fish (Low et al. 1994; Chen et al. 1997; Ueda et al. 2006; Kiriake et al. 2013). Homologs have recently also been found to be expressed in the venoms of monotremes (Whittington et al. 2010; Wong et al. 2013). The fish SNTX can cause lethal hemolysis through the formation of pores in the cell membrane, as well as a variety of other effects that disturb normal function of the circulatory and neuromuscular systems.

**Fig. 5.—:**
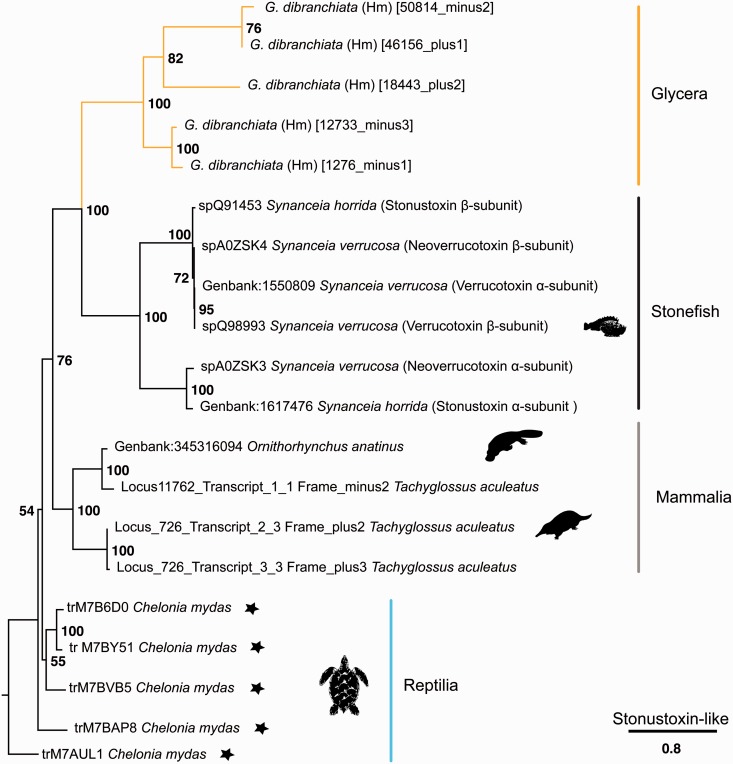
Phylogenetic tree of SNTX-like sequences. Phylogenetic reconstruction was performed with RAxML (7.4.2 SSE3-PTHREADS) (Stamatakis and Alachiotis 2010), -f a, PROTGAMMAIJTTF, 1,000 bootstrap pseudoreplicates. Bootstrap support values given for all nodes. See the legend of figure 3 for further information.

The venom glands of *G. dibranchiata* express five different transcripts homologous to SNTX-like toxins. The *Glycera* sequences are remarkably conserved with respect to the SNTX-like sequences from scorpaeniform fish and monotremes, although the pattern of cysteine residues may differ between some of these taxa. Major differences, however, occur at the C-terminal end of the sequences. In particular none of the *Glycera* sequences contains a B30.2/SPRY domain, which is present in the vertebrate SNTX-like toxins, and thought to be involved in mediating protein–protein interactions. Interestingly, our BLAST searches identified SNTX-like toxin homologs in the genome of the green sea-turtle, and these similarly lack B30.2/SPRY domains. In our tree of selected SNTX-like toxins and bloodworm homologs, we find that the bloodworm sequences fall into two clades (fig. 5). These clades might correspond to different SNTX-like subunits, although proteomic work is needed to confirm this. SNTX-like toxins are known to be active as either heterodimers or tetramers.

The *Glycera* sequences appear not to have N-terminal signal peptides. Intriguingly, the absence of signal peptides has also been noted for stonefish SNTX (Ghadessy et al. 1996; Ueda et al. 2006), where it correlates with the absence of Golgi and rough endoplasmic reticulum from venom gland cells.

#### Neurotoxins: ShKT Domain Neurotoxin and Gigantoxin-Like Neurotoxin

The venom of bloodworms can produce a variety of neurotoxic effects in both vertebrates and invertebrates (Michel and Robin 1972; Michel and Keil 1975; Manaranche et al. 1980; Bon et al. 1985; Meunier et al. 2002, 2010). These effects include cardiac arrest, progressive paralysis, and sudden convulsions followed by death in crustaceans, which are known bloodworm prey (Fauchald and Jumars 1979). Our results suggest that *Glycera* venom glands express precursors of different putative neurotoxins that could explain these observations.

The venom glands of both *G. dibranchiata* and *G. tridactyla* (as well as the body tissue of *G. tridactyla*) express 13 different transcripts containing ShKT domains. ShKT domains code for two peptide toxins, ShK and BgK, originally described from sea anemones, which can block voltage-gated potassium channels (Castaneda et al. 1995; Castaneda and Harvey 2009). ShK and BgK peptides are, respectively, 35 and 37 amino acids long, and they contain six conserved cysteine residues that form three disulfide bridges that play important roles in facilitating their ion channel blocking activities (Pennington et al. 1999). ShKT domains have been incorporated into a wide diversity of animal proteins, including venom toxins, which also contain a variety of other domains (Rangaraju et al. 2010).

**Fig. 6.—:**
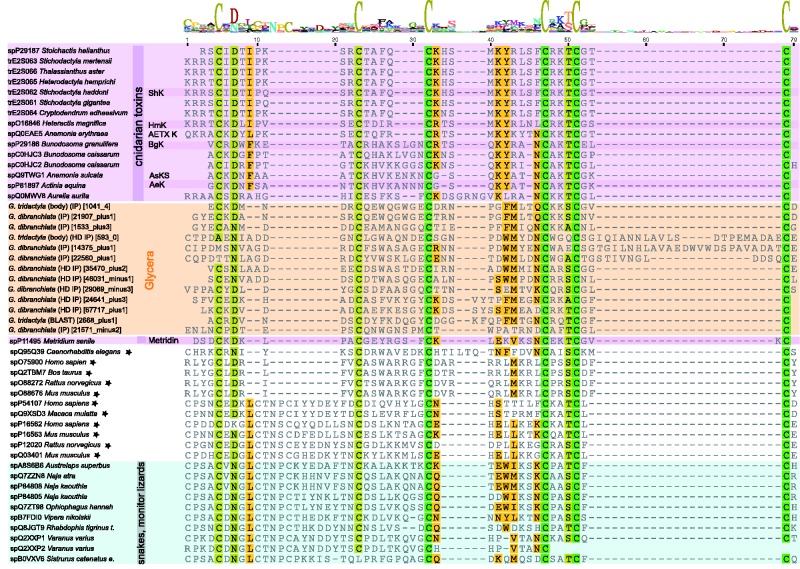
Multiple sequence alignment of ShKT toxin domains generated by MAFFT-L-INS-i (Katoh and Standley 2013). Conserved residues are highlighted. Cnidarian sequences are highlighted in purple, *Glycera* sequences in orange, squamates in blue, and nonvenomous taxa are indicated by stars. See supplementary figure S2, Supplementary Material online, for an overview of the different domain arrangement patterns found in ShKT transcripts.

The *Glycera* ShKT domain transcripts contain the six conserved cysteine residues that are characteristic for this domain (fig. 6). The transcripts show three basic domain arrangements: Metalloproteinase M12 + ShKT, SUEL-like Lectin +ShKT, and CAP + ShKT (see supplementary fig. S2, Supplementary Material online). However, within and between these three basic types the transcripts vary widely in the presence of putative cleavage sites, signal peptides, transmembrane regions, tandem repeats of ShKT domain variants, and the presence of additional domain types.

**Fig. 7.—:**
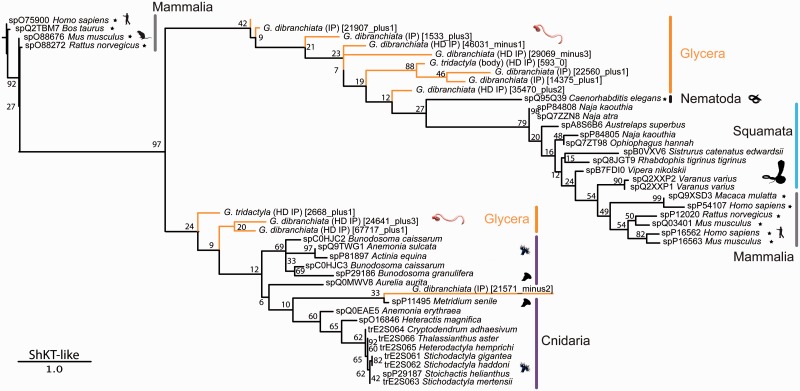
Phylogenetic tree of ShKT domain sequences. Phylogenetic reconstruction was performed with RAxML (7.4.2 SSE3-PTHREADS) (Stamatakis and Alachiotis 2010), -f a, PROTGAMMAIVTF, 1,000 bootstrap pseudoreplicates. Bootstrap support values given for all nodes. See the legend of figure 3 for further information.

We performed a phylogenetic analysis on an alignment of the ShKT domains present in our sequences and those in a range of outgroups. This produced a tree with two distinct, but poorly supported, clades (fig. 7). Nine *Glycera* sequences group together with the sequences from other bilaterians, whereas four sequences group together exclusively with cnidarian sequences. Each of these two clades contains a transcript that together are the two most highly expressed putative toxin transcripts in the venom gland library of *G. dibranchiata*, but apart from their shared ShKT domains they have a very different overall organization. The transcript that groups with cnidarian sequences (21571_minus2) has a CAP domain upstream of the ShKT domain, whereas the other (14375_plus1) is preceded by two lectin domains, and also has a string of ShKT-like domains (supplementary fig. S2, Supplementary Material online).

Given these very different structures it is nearly impossible to predict the effects of these putative toxins. Most of the cnidarian peptides are known to be potassium channel blockers, with the exception of metridin. Metridin does not have ion channel modulating activities, but instead has a hemolytic effect (Krebs and Habermehl 1987). This may be correlated with the absence of the lysine residue (located in position 41 in the alignment of fig. 6) that is present in the other cnidarian sequences, and which is needed for blocking the pore of the potassium ion channel (Rangaraju et al. 2010). Substitution of this lysine in ShKT peptides into other amino acids diminishes or abolishes potassium channel blocking activity. None of the bloodworm sequences have this lysine residue, but this does not necessarily imply that they cannot function as neurotoxins. Most of the snake ShKT domains also lack this lysine residue, yet many of them have the ability to block potassium or calcium channels. This is probably related to the fact that as in the bloodworms, snake ShKT domains are part of larger proteins that often contain an N-terminal CAP domain (supplementary fig. S2, Supplementary Material online), just as one of the two most highly expressed *Glycera* ShKT domain proteins.

**Fig. 8.—:**
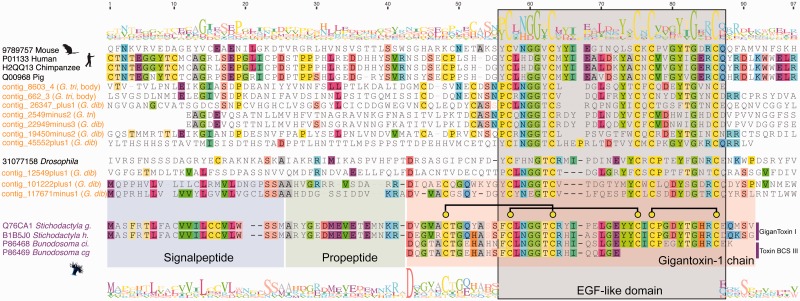
Multiple sequence alignment of gigantoxin domain sequences generated by MAFFT-L-INS-i (Katoh and Standley 2013). The signal peptide, propeptide, and domain regions of the cnidarian sequences and the two *Glycera* peptides that most strongly resemble them are highlighted in colored boxes. *Glycera* contig 117671minus1 has a leucine residue in position 91 of the alignment, which is thought to be involved in mediating the high-affinity binding of the EGF domain to the TRPV1 receptor, triggering the associated pain pathway.

Another putative *Glycera* neurotoxin is represented by transcripts that show strong sequence similarity to gigantoxin I, a neurotoxic peptide first found to be expressed in the nematocysts of the giant sea anemone *Stichodactyla gigantea* (Honma et al. 2003; Shiomi et al. 2003). Gigantoxin I is potently paralytic to crustaceans, but its epidermal growth factor (EGF)-like domain also gives it EGF activity. The presence of EGF domains in many transcripts expressed in the venom glands of all three investigated species of *Glycera* is probably the reason why significant BLAST matches (*e* value ≤ 0.00001) for gigantoxin I are found in all of our libraries. Most of these transcripts, however, code for large proteins. In contrast, two transcripts expressed in the venom glands of *G. dibranchiata* code for peptides of about the same length as gigantoxin I (117671_minus1 and 101222_plus1 are 88 and 89 amino acids long, respectively) (fig. 8). These peptides are also predicted to have signal peptides and propeptides with remarkable similarity to the gigantoxin I-like neurotoxins found in sea anemones, and the annelid and cnidarian peptide precursors also have an identical cysteine scaffold. Interestingly, transcript 117671_minus1 is predicted to form the same three disulfide bridges as the cnidarian toxins, whereas transcript 101222_plus1 is predicted to form two of these. The homology of the gigantoxin I-like peptide sequences in cnidarians and *Glycera* is limited to the EGF domains of longer proteins. Because of the very short and conserved alignment we did not perform a phylogenetic analysis of these sequences. However, we did explore whether the EGF-like domains of the gigantoxin I-like sequences of *Glycera* are more similar to the EGF domains of proteins from other bilaterians (reflecting the phylogenetic position of *Glycera*) or whether they are more similar to those of cnidarians (possibly reflecting changes associated with being expressed as peptides rather than larger proteins). To do this, we constructed a neighbor-joining network with Splitstree. Neighbor-joining networks graphically summarize the presence of conflicting signals in aligned sequence data. The results (supplementary fig. S3, Supplementary Material online) show that the sea anemone peptides and bloodworm homologs are indeed similar, and that the two *Glycera* peptide transcripts group more closely with the cnidarian peptide sequences than with the *Glycera* transcripts that code for larger proteins.

Cnidarian gigantoxin I-like peptides are able to elicit acute pain by indirect activation of the TRPV1 channel, a cation channel also known as the capsaicin receptor because it is targeted by the active component of chilli peppers. The toxin peptides do this by binding to EGF receptors with their EGF domain, which results in the activation of phospholipase A_2_ and the production of metabolites that activate the TRPV1 channel (Cuypers et al. 2011). We expect that their similar structure, including the presence of an EGF domain, allows the two putative *Glycera* peptides to exert a similar effect, which may cause the sharp pain induced by bloodworm bites (Klawe and Dickie 1957). We expect that one of the *Glycera* peptides will be an especially potent activator of the TRPV1 pain pathway as it possesses a leucine residue in alignment position 91 (fig. 8) that is essential for high-affinity binding to the EGF receptor, and which the cnidarian toxins lack (Shiomi et al. 2003).

*Glycera* venom glands also express transcripts coding for the precursors of a third putative type of neurotoxin that shows high similarity to the turripeptides of turrid gastropods. These peptides are dominated by a Kazal domain and we therefore discuss them in the section on Kazal protease inhibitors in the supplementary material, Supplementary Material online (see also supplementary fig. S12, Supplementary Material online).

There is no indication that any of the candidate neurotoxins identified here represents glycerotoxin, the calcium channel activating neurotoxin known from the venom of *G. tridactyla* (Bon et al. 1985; Meunier et al. 2002, 2010). Glycerotoxin is expected to be much larger than the putative neurotoxins identified here. Identifying and characterizing glycerotoxin in future studies will require our approach to be complemented by protein sequencing of purified fractions of *Glycera* venom.

#### Enzymes: Phospholipase A_2_ and Phospholipase B

The venom glands of *G. dibranchiata* express PLA_2_ and phospolipase B (PLB) transcripts, which is consistent with earlier biochemical studies that have shown phospholipase activity of *G. tridactyla* venom (Bon et al. 1985). The expression of phospholipases might, among other things, enhance the neurotoxicity of the *Glycera* venom. PLA_2_ is an enzyme that has been widely recruited into animal venoms, from cnidarians to cone snails, snakes, and arthropods (Fry et al. 2009; Terrat et al. 2012). PLA_2_ catalyzes the hydrolysis of phospholipids, and as a result its venom effects are varied, including cytotoxicity, myotoxicity, neurotoxicity, antiplatelet activity, and inflammation. *Glycera dibranchiata* venom glands express three different PLA_2_ transcripts at a low abundance, whereas the body tissues of *G. tridactyla* express two transcripts (of unequal length and only differing by a single amino acid). All these sequences have the two typical active sites (histidine and aspartic acid) for PLA_2_. The clade formed of two of the *G. dibranchiata* sequences and the two *G. tridactyla* sequences represents group XII secreted PLA_2_ (supplementary fig. S4, Supplementary Material online).

We also found two transcripts of PLB expressed in the *G. dibranchiata* gland library. Although PLA_2_ has been frequently recruited into venom cocktails, this is more rarely the case for PLB. As far as we know PLB activity has been reported from some hymenopteran venoms (Rosenberg et al. 1977; Watala and Kowalczyk 1990), and PLB transcripts have only been found to be expressed in the venom glands of several species of snakes (Rokyta et al. 2011; Fry et al. 2012; Margres et al. 2013).

PLA_2_ is known to be responsible for neurotoxic activity at the presynaptic membrane, where it hydrolyzes phospholipids into fatty acids and lysophospholipids (Rigoni et al. 2005; Zimmerberg and Chernomordik 2005). These products cannot form a lipid bilayer. Fatty acids are able to change the conformation of the membrane into a hemifusion state, thereby reducing the threshold for synaptic vesicle release. PLB does not generate lysophospholipids, but instead it hydrolyzes twice the amount of fatty acids compared with PLA_2_, which might help to increase the stimulatory effect of glycerotoxin at the presynaptic membrane.

#### Bloodworm Feeding Biology and the Unexpected Diversity of Expressed Toxin Homologs

In addition to the components discussed above, bloodworm venom glands express transcripts coding for another dozen different putative toxins, as discussed in the supplementary material, Supplementary Material online. Bloodworm venom is clearly not dominated by toxin classes that are more or less variations on a single theme, such as the neurotoxic peptides of cone snails and their relatives (Olivera et al. 2012), which represent the nearest venomous outgroups of bloodworms outside Annelida. The different components of *Glycera* venom are most likely involved in different aspects of prey capture and processing, although conclusions can only be tentative until more comprehensive functional assays have been performed.

Based on the results of available experiments that assessed the effects of bloodworm venom on crustacean prey, and supplemented by the known effects of homologs of the putative bloodworm toxins in other taxa, we speculate that the various pore-forming toxins and neurotoxin transcripts identified in *Glycera* venom glands code for proteins that can rapidly paralyze or kill invertebrate prey, especially crustaceans. The various highly expressed CAP domain proteins might contribute to overwhelming the prey. Enzymes such as the phospholipases probably enhance the neurotoxicity of the venom, whereas others, such as the various peptidases, and especially hyaluronidase, may act as spreading factors. Enzymes, including the various proteases and chitinase, may act predominantly as aides in the digestion of prey. Although bloodworms can inflict painful bites in defense, we do not know how effective these are in deterring predation by their natural predators, such as fish and birds.

The toxin category that remains most mysterious is that of the protease inhibitors. The homologs of five different types of protease inhibitors expressed in bloodworm venom glands are particularly prominent in the oral secretions of blood-feeders, such as ticks, mosquitos, leeches, and vampire bats (Anatriello et al. 2010; Chagas et al. 2013; Francischetti et al. 2013; Low et al. 2013; Kvist et al. 2014). But despite the fact that these protease inhibitors may have antihemostatic effects, no evidence exists suggesting that bloodworms are blood feeders.

It is possible that the observed differences in the complement of putative toxin transcripts expressed by the three species of bloodworm investigated here reflect different dietary specializations, but not enough is known about the prey preferences of the different species of *Glycera* to address this issue at this time. Our results do contradict, however, the long held belief that *G. dibranchiata* is chiefly a detritivore (Klawe and Dickie 1957; Fauchald and Jumars 1979; Rouse and Pleijel 2001). The anatomy of the venom apparatus and the diverse mix of toxin homologs transcribed in the venom glands of *G. dibranchiata* leave little doubt that it is a capable predator of macroscopic prey that likely uses potent venom to catch its prey. This conclusion is supported by the presence of the remains of other polychaetes, mollusks, and crustaceans in the guts and feces of this species (Ambrose 1984; Ungerer 2002), as well as by predation experiments in which it consumed enteropneusts (Giray and King 1997).

Two other factors need to be kept in mind as well when considering the issue of differences in the toxin mix of different species. First, the library of *G. dibrachiata* was sequenced more deeply than those of the other two species, which may have led to an underestimate of toxin homolog diversity in these species. Second, the venom gland libraries for all three species were based on tissue from single individuals, and they therefore only provide a snapshot of possible toxin variation. It is known that substantial intraspecific variation in the toxin composition of venom can result from differences in the physiological state of the venom glands (active or resting), as well as sex differences, ontogenetic differences, and geographical differences (Abdel-Rahman et al. 2009; Morgenstern et al. 2011; Safavi-Hemami et al. 2011; Sunagar et al. 2014). Consequently, to achieve a more detailed understanding of species differences in toxin composition future studies need to take these different factors into account.

Interestingly, the venom glands and body tissue of *G. tridactyla* express transcripts coding for the same convergently recruited types of putative toxins. Indeed, as indicated in supplementary table S8, Supplementary Material online, several *G. tridactyla* transcripts expressed in both the venom gland and body tissue appear to be identical. However, *G. tridactyla* venom glands express several unique putative toxin transcripts (supplementary table S2, Supplementary Material online) not expressed in the body tissue, and which do not correspond to any known convergently recruited toxin types. In fact, this class of potentially novel toxins is the most highly expressed class of putative toxins in the venom glands of *G. tridactyla*. Interestingly, the widespread expression of putative toxins in nonvenom gland tissues has recently also been found in snakes (Hargreaves et al. 2014), and has been taken to suggest that the expression of venom toxins may become restricted to the venom glands after gene duplication and subfunctionalization events.

### Identifying Toxin Homologs: Comparison of Transcriptome Assembly Methods

All transcriptome-based studies rely on the assembly of sequencing reads, and different kinds of assembly software have been published for both 454 and Illumina data. Different transcriptome de novo assemblies are generally difficult to compare (Grabherr et al. 2011; Martin and Wang 2011; O’Neil and Emrich 2013). Assembly software can differ in the number, length, and quality of the contigs they produce, as well as their sensitivity for finding low expressed transcripts (Bradnam et al. 2013; Peng et al. 2013), especially during de novo assembly. In our analyses, we compared assemblies produced by the commonly used licensed software suite CLC Genomics Workbench (CLC bio) and IDBA-tran (Peng et al. 2013). IDBA-tran is a de novo transcriptome assembler based on de Bruijn graphs for Illumina platform based data that uses an iterative *k*-mer optimization approach. IDBA-tran outperforms widely used software such as Trinity (Grabherr et al. 2011) with respect to sensitivity, specificity, and the number of correctly assembled contigs (Peng et al. 2013). Moreover, it is specifically designed to assemble low abundance transcripts, which is important as highly potent toxins may be expressed at lower levels.

Our results confirm that the choice of transcriptome assembly method can substantially influence the results (see supplementary table S1, Supplementary Material online). We found that overall IDBA was more sensitive than CLC. For several putative toxins, only IDBA was able to generate contigs. For instance, putative actinoporin-like toxins were never identified in the CLC assemblies, and only IDBA produced for the *G. tridactyla* library contigs of the ShKT domain toxin, and for the *G. fallax* library contigs of CAP domain toxins. However, IDBA did not generate a larger number of contigs than CLC for every toxin. The assemblers also differed in the average length of contigs produced. This is especially important for identifying N-terminal signal peptides. With some exceptions, such as the absence of signal peptides from many secreted cnidarian peptides and stonefish SNTXs (Ghadessy et al. 1996; Balasubramanian et al. 2012; Brinkman et al. 2012), most venom proteins are secreted and should therefore have a signal peptide (Fry et al. 2009). For our data IDBA generally produced assemblies comprising longer transcripts including signal peptides, with the exception of hyaluronidase, serpin and sphingomyelinase, for which only CLC produced contigs with predicted signal peptides. We therefore based our analyses on the IDBA-tran assembled data.

#### Multipronged Approach to Toxin Homolog Identification

Many transcriptomic studies of venom composition rely either on BLAST (Altschul et al. 1990) or on InterProScan (Zdobnov and Apweiler 2001) based identification of putative venom proteins. Instead, our approach for annotating and identifying all putative toxin families of *Glycera* consists of three independent search strategies: 1) BLAST2GO based InterPro annotation (Conesa et al. 2005), 2) profile HMMs (Eddy 1998) based on profiles of full-length toxin sequences, and 3) profile HMM based on single protein domains (for details see Materials and Methods and supplementary fig. S5, Supplementary Material online).

BLAST identification is especially useful for analyses of large numbers of sequences. However, BLAST is typically less sensitive than model-based approaches when it comes to identifying protein homologs showing a wide evolutionary diversity (Koua et al. 2013). Moreover, a lot of manual editing of BLAST-outputs is needed if toxins are composed of domain regions that are found also in proteins with normal physiological functions unrelated to venom proteins. In contrast, BLAST2GO utilizes an automated model-based approach and scans whole transcript sequences for domains through HMM profiles based on InterProScan. Thereby, it searches against protein databases such as PFAM, PROSITE, and SUPERFAMILY (Conesa and Götz 2008). The protein family IDs and related protein or domain names are output into a “.xml” file that can be searched by text or script-based strategies. However, with this approach obviously only toxins which are curated in these databases can be discovered. This is true, for instance, for glycerotoxin, the activity of which has been characterized, but for which no sequence data are available (Meunier et al. 2002, 2010). Additionally, searching toxins in.xml files is hindered by the lack of a consistent vocabulary and high numbers of synonyms for names of protein families in these databases (Koua et al. 2013).

The identification of gigantoxin I-like transcripts in our data represents a good example of the difficulty of identifying new toxins that were neither revealed by InterProScan (domain IDs) nor by our HMMER approaches. The use of probabilistic models of sequence composition in HMMER is beneficial as these are based on large multiple sequence alignments of specific protein families. HMMs use position-specific insertion/deletion probabilities instead of the arbitrary, position-invariant gap costs of the more traditional BLAST Smith–Waterman algorithm, and this allows profile HMMs to model a varying frequency of indels (Eddy 1998; Koua et al. 2013). One downside is that HMMER requires alignments of a minimum number of homologous sequences to train HMM profiles. Because only a small number of gigantoxin I-like sequences is present in public databases we could not train a HMM profile to indentify this toxin in our assemblies. Moreover, the domain of gigantoxin I strongly resembles an EGF domain, which is present in many proteins that are abundant in nonvenom gland tissue. Hence, a search for gigantoxin through InterProScan would lead to many false positives. In the case of gigantoxin, it was the BLAST output from BlAST2GO (against NCBI [National Center for Biotechnology Information] nr) that gave the correct match for one of our transcripts. The same was true for the identification of the two turripeptide-like toxins. InterProScan and HMMER annotated their Kazal domains. However, only careful study of our BLAST results provided the seed for further investigation that resulted in our identification of the first nonmollusk turripeptide-like toxin transcripts.

Generally, the different search strategies produced similar results for our data. There was a high degree of overlap in identification between the methods, yet a number of transcripts could have been overlooked if only one of the search strategies would have been applied. Our methodological conclusions are therefore 2-fold. First, only a combination of the above mentioned strategies is able to exhaustively identify putative toxins. Second, unusual or novel toxins require off-pipeline attention due to the lack of information present in public databases.

## Conclusion

In summary, we present the first transcriptomic profiles of polychaete venom glands. *Glycera* venom glands express a complex cocktail of putative toxin precursor transcripts, including neurotoxic peptides, pore-forming proteins, and enzymes. Our study further expands the number and taxonomic range of protein types known to have been convergently recruited into animal venoms (Casewell et al. 2013). Notably, bloodworm venom glands express homologs of toxins previously only known from venoms of scorpaeniform fish and monotremes (SNTX-like toxin), turrid gastropods (turripeptide-like peptides), and sea anemones (gigantoxin I-like neurotoxin), as well as many toxins with a broader phylogenetic distribution. Given this unexpected diversity of new putative bloodworm toxins, it will be interesting to profile more species of bloodworms.

The deeply sequenced venom gland transcriptome of *G. dibranchiata* shows that with the single exception of the protease inhibitor cystatin, all identified venom toxin transcripts have undergone varying degrees of lineage-specific diversification. Highly expressed putative toxins, such as the enzymes peptidase S1 and metalloprotease M12, as well as CAP and Kazal domain proteins, have diversified into large numbers of species- or genus-specific paralogs. Although proteomic and functional studies are needed to confirm our transcriptomic predictions of venom composition and activities, our results clearly suggest that bloodworms are effective predators that likely use complex toxin cocktails to subdue and process macroscopic prey. These predatory habits are confirmed by the anatomy of their venom apparatus, as well as field observations of their feeding habits, even for *G. dibranchiata*, which had long been thought to be a detritivore.

Moreover, our results suggest that in order to produce exhaustive transcriptomic profiles of venom glands it is important to be aware that different transcriptome assembly methods, as well as different methods of homology prediction, can yield different results. Although our analyses benefit from the use of a customized bioinformatic pipeline, several putative toxins would not have been identified without manually scrutinizing the results. But when a bioinformatic pipeline is combined with a thorough inspection of the results, deep transcriptomic profiling of venom glands of poorly studied taxa is one of the most effective ways to broaden the empirical foundation of venomics.

## Supplementary Material

Supplementary files S1–S3 are available at *Genome Biology and Evolution* online (http://www.gbe.oxfordjournals.org/).

Supplementary Data
