# Supplementary material for: A Polychaete’s Powerful Punch: Venom Gland Transcriptomics of Glycera Reveals a Complex Cocktail of Toxin Homologs
Source: Genome Biol Evol. 2014 Sep 5;6(9):2406–23. doi: 10.1093/gbe/evu190 (PMC4202326; doi:10.1093/gbe/evu190)
Supplement: Supplementary Data [file supp_6_9_2406__index.html]

A polychaete's powerful punch: venom gland transcriptomics of Glycera reveals a complex cocktail of toxin homologs — A Polychaete’s Powerful Punch: Venom Gland Transcriptomics of Glycera Reveals a Complex Cocktail of Toxin Homologs — Supplementary Data 

# A Polychaete’s Powerful Punch: Venom Gland Transcriptomics of *Glycera* Reveals a Complex Cocktail of Toxin Homologs

## Supplementary Data

files

**Files in this Data Supplement:**

- Supplementary Data - zip file
