# Supplementary material for: A Polychaete’s Powerful Punch: Venom Gland Transcriptomics of Glycera Reveals a Complex Cocktail of Toxin Homologs
Source: Genome Biol Evol. 2014 Sep 5;6(9):2406–23. doi: 10.1093/gbe/evu190 (PMC4202326; doi:10.1093/gbe/evu190)
Supplement: Supplementary Data [file supp_evu190_suppl_data.zip › Supp_file2_final.pdf]

## Supplementary Material

# A polychaete's powerful punch: venom gland transcriptomics of *Glycera* reveals a complex cocktail of toxin homologs

Björn M. von Reumont, Lahcen Campbell, Sandy Richter, Lars Hering, Dan Sykes, Jörg Hetmank, Ronald A. Jenner, Christoph Bleidorn

## Supplementary Material includes:

Supplementary_file1: Supplementary Text, References, Supplementary Figures S1-S20

**Supplementary _file2: Supplementary Tables S1-8**

Supplementary_file3: Alignments, datasets, scripts

### Supplementary Tables description

**Supplementary table S1.** Comparison of assembly and search strategies and overview of all venom protein matches. Matches for venom proteins are given for each library, each search strategy, and the two different assembly methods relying on IDBA-tran and CLC Genomic workbench. Numbers given indicate total number of non-redundant matches for toxin proteins for each species across all three search strategies (Interpro, Hmmer, Hmmer Domain). Numbers shown in parenthesis indicate valid contigs retained for final phylogenetic analysis following manual inspection of GO annotation and preliminary

phylogenetic reconstruction. Gigantoxin I was a special case and only one match each was recovered in the CLC and IDBA-tran assemblies of *G. dibranchiata* via BLAST search based on BLAST2GO (data not shown). NA indicates search strategy not applicable for a given toxin.

**Supplementary table S2.** Summary of contigs with signal peptides and open reading frames identified in our transcriptome libaries for which we could not identify convincing homologues in other taxa. Sequences with short open reading frames due to the presence of stopcodons, multiple transmembrane regions, and suspicious BLAST hits are indicated as putative sequence artifacts.

**Supplementary table S3.** All hits for secreted proteins found in the venom gland library of *Glycera tridactyla.*

**Supplementary table S4.** All hits for secreted proteins found in the body tissue library of *Glycera tridactyla.*

**Supplementary table S5.** All hits for secreted proteins found in the venom gland library of *Glycera fallax.*

**Supplementary table S6.** All hits for secreted proteins found in the venom gland library of *Glycera dibranchiata.*

**Supplementary table S7.** Best fitting models selected by ProtTest 3 (Darriba et al. 2011) for each dataset used for phylogenetic analysis.

**Supplementary table S8.** Venom gland and body tissue sequences of *G. tridactyla* that appear identical (zero-length branches) in several toxin trees are indicated in the same colour, with comments indicating if the full length contigs are identical or not.

Supplementary table S1

| | | InterproScan Hits | | | | | | | | Hmmer Hits - Full Alignment | | | | | | | | Hmmer Hits - Domains only | | | | | | | |
|---|---|---|---|---|---|---|---|---|---|---|---|---|---|---|---|---|---|---|---|---|---|---|---|---|---|
| Proteins | InterPro Ids / Searchterms | *G. dibranchiata* | | *G. fallax* | | *G. tridactyla* | | *G. tridactyla, Body* | | *G. dibranchiata* | | *G. fallax* | | *G. tridactyla* | | *G. tridactyla, Body* | | *G. dibranchiata* | | *G. fallax* | | *G. tridactyla* | | *G. tridactyla, Body* | |
| | | CLC | IDBA | CLC | IDBA | CLC | IDBA | CLC | IDBA | CLC | IDBA | CLC | IDBA | CLC | IDBA | CLC | IDBA | CLC | IDBA | CLC | IDBA | CLC | IDBA | CLC | IDBA |
| Actinoporin | Sea anemone actinoporin (IPR009104) | -- | **3** | -- | -- | -- | -- | -- | -- | -- | **3** | -- | -- | -- | -- | -- | -- | -- | **3** | -- | -- | -- | -- | -- | -- |
| C-type Lectin | C-type Lectin (IPR001304) | **22** | **26 (25)** | **1** | **2** | **2** | **3** | -- | **1** | **24** | **28 (27)** | **1** | **2** | **2** | **3** | **2** | **1** | **24** | **29 (28)** | **1** | **2** | **2** | **3** | **2** | **1** |
| CAP | CAP doamin (IPR014044), Cysteine rich secretory protein, allergen V5/Tpx-1-related (IPR001283) | **20** | **20 (15)** | -- | **1** | **5** | **5** | **2** | **2** | **20** | **20 (15)** | -- | **1** | **5** | **5** | **2** | **2** | **17** | **20 (15)** | -- | **1** | **5** | **5** | **2** | **2** |
| Chitinase | Glycoside hydrolase, family 18, catalytic domain (IPR001223), chitinase active site (IPR001579) | **14** | **11 (7)** | -- | -- | -- | -- | -- | -- | **13** | **11 (7)** | -- | -- | -- | -- | -- | -- | **13** | **10 (7)** | -- | -- | -- | -- | -- | -- |
| Cystatin | Proteinase inhibitor I25, cystatin (IPR000010) | **1** | **1 (0)** | **1** | **1** | **1** | **1** | **1** | **1** | **1** | **1 (0)** | **1** | **1** | **2** | **1** | **1** | **1** | **1** | **1 (0)** | **1** | **1** | **1** | **1** | **1** | **1** |
| Hyaluronidase | Hyaluronidase (IPR018155) | **3** | **2** | -- | -- | -- | -- | -- | -- | **3** | **2** | -- | -- | -- | -- | -- | -- | **3** | **2** | -- | -- | -- | -- | -- | -- |
| Kazal inhibitors | Kazal domain (IPR002350) | **20** | **14 (11)** | **4** | **5** | **6** | **6** | **7** | **8** | **18** | **13 (11)** | **7** | **6** | **6** | **6** | **7** | **9** | **18** | **13 (11)** | **5** | **5** | **5** | **6** | **7** | **8** |
| Kunitz | Proteinase inhibitor I2, Kunitz metazoa (IPR002223) | **8** | **10** | -- | -- | -- | -- | **2** | **2** | **7** | **9** | -- | -- | -- | -- | **2** | **2** | **7** | **9** | -- | -- | -- | -- | **2** | **2** |
| Lipocalin | Lipocalin/cytosolic fatty-acid binding domain (IPR000566) | **1** | **3** | **1** | **1** | -- | -- | ***1*** | -- | **1** | **1** | -- | -- | -- | -- | -- | -- | **1** | **1** | -- | -- | -- | -- | -- | -- |
| Metallopeptidase M12 | "M12" | **41** | **42 (36)** | -- | -- | **2** | **2** | **2** | **2** | **32** | **36** | -- | -- | **2** | **1** | **2** | **2** | **28** | **40 (35)** | -- | -- | **2** | **1** | **2** | **3 (2)** |
| Peptidase S1 | Peptidase S1 domain (IPR001254), Peptidase S1, trypsin family, active site (IPR018114) | **81** | **86 (85)** | **7** | **5** | **11** | **8** | **11** | **13** | **80** | **87 (85)** | **7** | **5** | **11** | **9** | **16** | **13** | **79** | **86 (85)** | **7** | **5** | **11** | **8** | **16** | **13** |
| Peptidase S10 - Venom Serine carboxypeptidase | Peptidase S10 (IPR001563) | **28** | **21 (20)** | -- | -- | **2** | **3** | **4** | **4** | **26** | **21 (20)** | **1** | -- | **2** | **3** | **4** | **4** | **26** | **21 (20)** | **1** | -- | **2** | **3** | **4** | **4** |
| Phospholipase A2 | Phospholipase A2 (IPR001211), Phospholipase A2 | **4** | **5 (3)** | -- | -- | -- | -- | **1** | **2** | **4** | **3 (2)** | -- | -- | -- | -- | **1** | **2** | **2** | **3** | -- | -- | -- | -- | **1** | **2** |
| Serpin | Serpin family (IPR000215), Serpin domain (IPR023796), Serpin, conserved site (IPR023795) | **9** | **9 (8)** | **1** | -- | **1** | **2** | **3** | **1** | **9** | **9 (8)** | **1** | -- | **1** | **2** | **5** | **1** | **9** | **9 (8)** | **1** | -- | **1** | **2** | **5** | **1** |
| ShK | ShKT domain (IPR003582) | **7** | **10** | -- | -- | -- | **1** | **2** | **2** | **NA** | **NA** | **NA** | **NA** | **NA** | **NA** | **NA** | **NA** | **2** | **5** | -- | -- | -- | **1** | **1** | **1** |
| Sphingomyelinase | Sphingomyelinase C/phospholipase C (IPR017766), "PTHR10340" | **9** | **8** | -- | -- | -- | -- | -- | -- | **5** | **7** | -- | -- | -- | -- | -- | -- | -- | -- | -- | -- | -- | -- | -- | -- |
| Stonustoxin | "stonu" keyword | -- | -- | -- | -- | -- | -- | -- | -- | **6** | **5** | -- | -- | -- | -- | -- | -- | **NA** | **NA** | **NA** | **NA** | **NA** | **NA** | **NA** | **NA** |

Supplementary table S2

| Nr. | Contig sequence name | Number of reads | Seq Length | % Assembly Mismatch | Putative sequence artifacts | Top BLAST hit Uniprot Secreted proteins | :E-value | Top BLAST hit NCBI ESTs | :E-value | Top BLAST hit NCBI TSA | :E-value | Interpro scan results (BLAST2GO) |
|---|---|---|---|---|---|---|---|---|---|---|---|---|
| 1 | **lib_Dibranchiata_Filter15_IDBA_contig_37118_plus1** | 106 | 1538 | 0.728 | -- | sp\|Q26264\|SM41_HEMPU 41 kDa spicule matrix protein OS=Hemicentrotus pulcherrimus PE=2 SV=1 | **8.00E-05** | No hits found | N\A | No hits found | N\A | SignalP-NN(euk) (SIGNALP), tmhmm (TMHMM) |
| 2 | **lib_Dibranchiata_Filter15_IDBA_contig_4347_minus2** | 8428 | 7865 | 0.601 | -- | No hits found | N\A | No hits found | N\A | No hits found | N\A | PTHR24023 (PANTHER), PTHR24023:SF106 (PANTHER), SignalP-NN(euk) (SIGNALP) |
| 3 | **lib_Dibranchiata_Filter15_IDBA_contig_48769_minus1** | 344 | 2287 | 1.461 | x | tr\|F9HQ33\|F9HQ33_STRMT Gram positive anchor OS=Streptococcus mitis SK1080 GN=HMPREF9957_0196 PE=3 SV=1 | **1.00E-06** | No hits found | N\A | No hits found | N\A | SignalP-NN(euk) (SIGNALP), tmhmm (TMHMM) |
| 4 | **lib_Dibranchiata_Filter15_IDBA_contig_65879_minus3** | 35 | 497 | 1.131 | -- | sp\|B3A0Q9\|PLSLP_LOTGI Perlustrin-like protein OS=Lottia gigantea PE=1 SV=1 | **2.00E-10** | No hits found | N\A | No hits found | N\A | SignalP-NN(euk) (SIGNALP), tmhmm (TMHMM) |
| 5 | **lib_Dibranchiata_Filter15_IDBA_contig_77124_minus3** | 495 | 688 | 3.264 | x | No hits found | N\A | No hits found | N\A | No hits found | N\A | PTHR11905 (PANTHER), PTHR11905:SF48 (PANTHER), SignalP-NN(euk) (SIGNALP) |
| 6 | **lib_Dibranchiata_Filter15_IDBA_contig_78707_minus1** | 47 | 770 | 0.863 | x | No hits found | N\A | No hits found | N\A | No hits found | N\A | SignalP-NN(euk) (SIGNALP) |
| 7 | **lib_Dibranchiata_Filter15_IDBA_contig_78707_minus2** | 47 | 770 | 0.863 | x | No hits found | N\A | No hits found | N\A | No hits found | N\A | SignalP-NN(euk) (SIGNALP), tmhmm (TMHMM) |
| 8 | **lib_Dibranchiata_Filter15_IDBA_contig_78708_minus1** | 45 | 770 | 0.682 | x | No hits found | N\A | No hits found | N\A | No hits found | N\A | SignalP-NN(euk) (SIGNALP) |
| 9 | **lib_Fallax_Filter15_IDBA_contig_509_minus1** | 309 | 2100 | 0.505 | -- | No hits found | N\A | No hits found | N\A | No hits found | N\A | SignalP-NN(euk) (SIGNALP) |
| 10 | **lib_Tridactyla_Body_Filter15_IDBA_contig_1175_0** | 517 | 1141 | 0.791 | x | tr\|E6MB43\|E6MB43_STALU Serine-aspartate repeat-containing protein F OS=Staphylococcus lugdunensis M23590 GN=HMPREF0790_1723 PE=3 SV=1 | **2.00E-13** | No hits found | N\A | No hits found | N\A | SignalP-NN(euk) (SIGNALP) |
| 11 | **lib_Tridactyla_Body_Filter15_IDBA_contig_1269_2** | 2484 | 1079 | 0.271 | x | tr\|Q3MUG3\|Q3MUG3_STRAG C protein beta antigen OS=Streptococcus agalactiae GN=bac PE=3 SV=1 | **4.00E-15** | No hits found | N\A | No hits found | N\A | SignalP-NN(euk) (SIGNALP), tmhmm (TMHMM) |
| 12 | **lib_Tridactyla_Body_Filter15_IDBA_contig_3926_4** | 3555 | 1538 | 0.759 | -- | tr\|D5J9P5\|D5J9P5_9SAUR Non-conventional three finger toxin isoform 1 OS=Bungarus flaviceps PE=3 SV=1 | **5.00E-04** | CAGA Alvinella pompejana Normalized library RN04 posterior end Alvinella pompejana cDNA clone CAGA19428 5', mRNA sequence. | **3.00E-25** | Brachionus calyciflorus contig40640_876_1631 mRNA sequence GACQ01015522.1 | **4.00E-09** | G3DSA:2.10.60.10 (GENE3D), SignalP-NN(euk) (SIGNALP), tmhmm (TMHMM), SSF57302 (SUPERFAMILY) |
| 13 | **lib_Tridactyla_Body_Filter15_IDBA_contig_4416_0** | 107 | 763 | 0.841 | -- | sp\|P02784\|SFP1_BOVIN Seminal plasma protein PDC-109 OS=Bos taurus PE=1 SV=2 | **7.00E-06** | No hits found | N\A | Acropora cervicornis comp30190_c0_seq1 transcribed RNA sequence GASU01012422.1 | **7.00E-05** | IPR000562 (G3DSA:2.10.10.GENE3D); IPR013806 (SUPERFAMILY); SignalP-NN(euk) (SIGNALP) |
| 14 | **lib_Tridactyla_Body_Filter15_IDBA_contig_60_4** | 751 | 1338 | 1.115 | -- | sp\|Q9JHB3\|TIMP4_MOUSE Metalloproteinase inhibitor 4 OS=Mus musculus GN=Timp4 PE=2 SV=1 | **2.00E-05** | FPM_M_03461 Mantle of freshwater pearl mussel cDNA library Hyriopsis cumingii cDNA 5', mRNA sequence | **1.00E-07** | No hits found | N\A | IPR001820 (PFAM); IPR008993 (SUPERFAMILY); G3DSA:2.40.50.120 (GENE3D), SignalP-NN(euk) (SIGNALP), tmhmm (TMHMM) |
| 15 | **lib_Tridactyla_Body_Filter15_IDBA_contig_9410_3** | 46 | 614 | 0.925 | -- | tr\|A7S6C3\|A7S6C3_NEMVE Predicted protein OS=Nematostella vectensis GN=v1g207484 PE=3 SV=1 | **3.00E-06** | No hits found | N\A | No hits found | N\A | SignalP-NN(euk) (SIGNALP), tmhmm (TMHMM) |
| 16 | **lib_Tridactyla_Filter15_IDBA_contig_1556_minus2** | 906 | 1460 | 1.678 | -- | tr\|D5J9P5\|D5J9P5_9SAUR Non-conventional three finger toxin isoform 1 OS=Bungarus flaviceps PE=3 SV=1 | **2.00E-04** | CAGA19428.fwd CAGA Alvinella pompejana Normalized library RN04 posterior end Alvinella pompejana cDNA clone CAGA19428 5', mRNA sequence. | **2.00E-25** | Brachionus calyciflorus contig40640_876_1631 mRNA sequence GACQ01015522.1 | **1.00E-09** | G3DSA:2.10.60.10 (GENE3D), SignalP-NN(euk) (SIGNALP), SSF57302 (SUPERFAMILY) |
| 17 | **lib_Tridactyla_Filter15_IDBA_contig_1700_minus2** | 129 | 966 | 0.719 | -- | sp\|P83472\|ICI_LUMTE Chymotrypsin inhibitor OS=Lumbricus terrestris PE=1 SV=2 | **2.00E-11** | FR755362 Arenicola marina chloragog and foregut adult Arenicola marina cDNA clone dmp023cmP0006P20, mRNA sequence. | **7.00E-18** | Enchytraeus crypticus contig19688 transcribed RNA sequence GALF01017306.1 | **6.00E-17** | IPR000864 (PRODOM); G3DSA:3.30.10.10 (GENE3D), SignalP-NN(euk) (SIGNALP), tmhmm (TMHMM) |
| 18 | **lib_Tridactyla_Filter15_IDBA_contig_320_minus2** | 1044 | 1591 | 0.644 | -- | sp\|A8TX70\|CO6A5_HUMAN Collagen alpha-5(VI) chain OS=Homo sapiens GN=COL6A5 PE=1 SV=1 | **9.00E-22** | No hits found | N\A | No hits found | N\A | IPR008160 (PFAM); PTHR24023 (PANTHER), SignalP-NN(euk) (SIGNALP), tmhmm (TMHMM) |
| 19 | **lib_Tridactyla_Filter15_IDBA_contig_964_plus3** | 414 | 1280 | 0.847 | -- | sp\|Q64425\|LIPP_MYOCO Pancreatic triacylglycerol lipase (Fragment) OS=Myocastor coypus GN=PNLIP PE=2 SV=1 | **2.00E-47** | FR766474 Pectinaria koreni whole animal adult Pectinaria koreni cDNA clone dmp057P0002N19, mRNA sequence. | **1.00E-49** | Hynobius chinensis comp6981_c0_seq1 transcribed RNA sequence GAQK01103903.1 | **3.00E-74** | IPR003599 (SMART); IPR007110 (PROFILE); IPR013098 (PFAM); IPR013783 (G3DSA:2.60.40.GENE3D); IPR020675 (PANTHER); PTHR22964:SF3 (PANTHER), SSF48726 (SUPERFAMILY) |

## Supplementary Table 3

| Nr. | Contig sequence name | Number of reads | Sequence description (BLAST nr, E-value= > 0.0001) | Length (aa pos.) | E-Value | mean Similarity | Nr. of GO terms | Gene ontology terms and annotation (GO's) | Interpro scan results (BLAST2GO) | SignalP 4.1 (3.0) |
|---|---|---|---|---|---|---|---|---|---|---|
| 1 | lib_tri_Filter15_IDBA_contig_10_minus2 | 7071 | | 785 | 0 | 100% | 1 | F:DNA binding | IPR002059 (PRINTS); IPR011129 (SMART); IPR012340 (G3DSA:2.40.50.GENE3D); IPR019844 (PROSITE); PTHR11544 (PANTHER), PTHR11544:SF11 (PANTHER), tmhmm (TMHMM) | |
| 2 | lib_tri_Filter15_IDBA_contig_1003_plus2 | 291 | | 702 | 0 | 100% | 2 | P:carbohydrate metabolic process; F:catalytic activity | IPR001701 (PFAM); IPR008928 (SUPERFAMILY); IPR012341 (G3DSA:1.50.10.GENE3D); PTHR22298 (PANTHER), PTHR22298:SF3 (PANTHER) | |
| 3 | lib_tri_Filter15_IDBA_contig_1027_minus1 | 91 | | 380 | 0 | 100% | 1 | F:protein binding | IPR003599 (SMART); IPR007110 (PROFILE); IPR013098 (PFAM); IPR013783 (G3DSA:2.60.40.GENE3D); IPR020675 (PANTHER); IPR020682 (PTHR22964:PANTHER); SSF48726 (SUPERFAMILY) | |
| 4 | lib_tri_Filter15_IDBA_contig_1028_plus1 | 3574 | guanine nucleotide-binding protein subunit beta-2-like 1 | 380 | 0 | 93.75% | 25 | P:regulation of biological process; P:cell death; C:cytoskeleton; F:protein binding; C:cell; C:ribosome; F:receptor activity; F:receptor binding; P:biological_process; P:cell cycle; P:cellular component organization; C:plasma membrane; P:translation; P:cellular protein modification process; P:transport; P:signal transduction; C:cytoplasm; F:molecular_function; P:protein metabolic process; P:catabolic process; F:enzyme regulator activity; F:kinase activity; C:nucleus; P:cell growth; P:metabolic process | IPR001680 (PFAM); IPR015943 (G3DSA:2.130.10.GENE3D); IPR017986 (PROFILE); IPR019775 (PROSITE); IPR020472 (PRINTS); PTHR19868 (PANTHER), PTHR19868:SF0 (PANTHER) | |
| 5 | lib_tri_Filter15_IDBA_contig_1046_minus3 | 247 | nicotinic acetylcholine receptor alpha 6 isoform i | 375 | 5.87E-32 | 52.85% | 4 | C:plasma membrane; F:ion channel activity; C:cell; P:ion transport | IPR006201 (PANTHER); IPR006202 (G3DSA:2.70.170.GENE3D); PTHR18945:SF66 (PANTHER) | |
| 6 | lib_tri_Filter15_IDBA_contig_1052_plus3 | 331 | a chain solution structure of component iv glycera dibranchiata monomeric hemoglobin-co | 372 | 1.82E-52 | 56.60% | 3 | F:binding; P:transport; F:oxygen binding | IPR000971 (PFAM); IPR009050 (SUPERFAMILY); IPR012292 (G3DSA:1.10.490.GENE3D); IPR013316 (PRINTS); PTHR22924 (PANTHER), tmhmm (TMHMM) | |
| 7 | lib_tri_Filter15_IDBA_contig_1066_plus3 | 1351 | transmembrane protease serine 11d | 853 | 8.33E-51 | 55.80% | 3 | F:peptidase activity; P:protein metabolic process; P:catabolic process | IPR001254 (PFAM); IPR001314 (PRINTS); IPR009003 (SUPERFAMILY); IPR018114 (PROSITE); G3DSA:2.40.10.10 (GENE3D), PTHR24265 (PANTHER), SignalP-NN(euk) (SIGNALP) | Yes |
| 8 | lib_tri_Filter15_IDBA_contig_1067_plus1 | 50 | | 110 | 0 | 100% | 1 | F:catalytic activity | IPR009003 (SUPERFAMILY); G3DSA:2.40.10.10 (GENE3D), PTHR24265 (PANTHER) | |
| 9 | lib_tri_Filter15_IDBA_contig_1094_plus3 | 98 | neurotrypsin | 359 | 8.94E-65 | 58.50% | 3 | F:peptidase activity; F:receptor activity; C:cell | IPR001190 (PRINTS); IPR017448 (SMART); G3DSA:3.10.250.10 (GENE3D), PTHR19331 (PANTHER) | |
| 10 | lib_tri_Filter15_IDBA_contig_1104_plus1 | 99 | | 358 | 0 | 100% | 1 | F:protein binding | IPR006652 (PFAM); IPR011705 (PFAM); IPR015915 (G3DSA:2.120.10.GENE3D); PTHR24412 (PANTHER), SSF117281 (SUPERFAMILY) | |
| 11 | lib_tri_Filter15_IDBA_contig_1145_plus2 | 56 | selectin p | 375 | 2.50E-76 | 47.10% | 1 | F:calcium ion binding | IPR000152 (PROSITE); IPR000436 (PFAM); IPR001881 (PFAM); IPR002919 (SUPERFAMILY); IPR009030 (SUPERFAMILY); IPR011641 (PFAM); IPR018097 (PROSITE); G3DSA:2.10.25.10 (GENE3D), G3DSA:2.10.70.10 (GENE3D), PTHR19325 (PANTHER), SSF57196 (SUPERFAMILY) | |

| | | | | | | | | | |
|---|---|---|---|---|---|---|---|---|---|
| 12 | lib_tri_Filter15_IDBA_contig_1161_minus1 | 52 | protein-glutamine gamma-glutamyltransferase k | 342 | 1.06E-148 | 62.80% | 1 | P:cellular protein modification process | IPR001102 (PFAM); IPR002931 (G3DSA:3.90.260.GENE3D); IPR013783 (G3DSA:2.60.40.GENE3D); IPR013808 (PROSITE); IPR014756 (SUPERFAMILY); IPR023608 (PANTHER); SSF54001 (SUPERFAMILY) |
| 13 | lib_tri_Filter15_IDBA_contig_118_minus2 | 2327 | ubiquitin-40s ribosomal protein s27a | 204 | 1.31E-78 | 96.95% | 12 | C:nucleoplasm; P:signal transduction; P:response to stress; P:DNA metabolic process; P:translation; F:binding; P:metabolic process; F:structural molecule activity; C:endosome; C:ribosome; C:cytosol; F:protein binding | IPR000626 (PFAM); IPR002906 (PFAM); IPR019954 (PROSITE); IPR019955 (PROFILE); IPR019956 (PRINTS); G3DSA:3.10.20.90 (GENE3D), PTHR10666 (PANTHER), SSF54236 (SUPERFAMILY) |
| 14 | lib_tri_Filter15_IDBA_contig_1202_plus2 | 58 | muscle m-line assembly protein unc-89 | 327 | 3.58E-64 | 49.55% | 1 | F:protein binding | IPR003598 (SMART); IPR003599 (SMART); IPR003961 (PROFILE); IPR007110 (PROFILE); IPR013098 (PFAM); IPR013783 (G3DSA:2.60.40.GENE3D); PTHR10489 (PANTHER), SSF48726 (SUPERFAMILY) |
| 15 | lib_tri_Filter15_IDBA_contig_1213_minus3 | 3564 | | 644 | | | | | no IPS match |
| 16 | lib_tri_Filter15_IDBA_contig_1238_plus3 | 155 | | 500 | 0 | 100% | 2 | F:cytoskeletal protein binding; F:protein binding | IPR024836 (PANTHER); PTHR18935:SF4 (PANTHER) |
| 17 | lib_tri_Filter15_IDBA_contig_1240_minus2 | 114 | retinol dehydrogenase 11-like | 320 | 8.41E-48 | 68.30% | 2 | F:catalytic activity; P:metabolic process | IPR002424 (PRINTS); IPR016040 (G3DSA:3.40.50.GENE3D); PTHR24320 (PANTHER), PTHR24320:SF0 (PANTHER), tmhmm (TMHMM), SSF51735 (SUPERFAMILY) |
| 18 | lib_tri_Filter15_IDBA_contig_1248_plus1 | 694 | kazal-type serine protease inhibitor domain-containing protein 1 precursor | 705 | 9.03E-88 | 58.75% | 5 | C:proteinaceous extracellular matrix; P:cellular component organization; P:regulation of biological process; P:cell growth; F:protein binding | IPR000867 (PFAM); IPR002350 (PFAM); IPR003598 (SMART); IPR003599 (SMART); IPR007110 (PROFILE); IPR011390 (PANTHER); IPR013098 (PFAM); IPR013783 (G3DSA:2.60.40.GENE3D); G3DSA:3.30.60.30 (GENE3D), PTHR14186:SF7 (PANTHER), tmhmm (TMHMM), SSF100895 (SUPERFAMILY), SSF48726 (SUPERFAMILY) |
| 19 | lib_tri_Filter15_IDBA_contig_1256_plus2 | 62 | | 317 | 0 | 100% | 1 | F:protein binding | IPR003961 (PFAM); IPR013783 (G3DSA:2.60.40.GENE3D); PR00014 (PRINTS) |
| 20 | lib_tri_Filter15_IDBA_contig_1333_plus2 | 72 | | 306 | 0 | 100% | 1 | F:protein binding | IPR003961 (PFAM); IPR013783 (G3DSA:2.60.40.GENE3D); PR00014 (PRINTS), SSF48726 (SUPERFAMILY) |
| 21 | lib_tri_Filter15_IDBA_contig_1344_plus1 | 112 | heat shock protein 70 | 305 | 4.64E-123 | 95.45% | 25 | C:cell; F:receptor binding; P:cellular component organization; P:transport; P:cell-cell signaling; C:cytoplasmic membrane-bounded vesicle; F:nucleotide binding; P:regulation of biological process; ; C:nucleolus; C:cytosol; P:cell cycle; F:protein binding; C:organelle; C:extracellular region; P:protein metabolic process; C:protein complex; F:hydrolase activity; P:nucleobase-containing compound metabolic process; P:catabolic process; C:plasma membrane; C:intracellular; P:response to stress; P:response to biotic stimulus; P:biological_process | IPR013126 (PRINTS); G3DSA:1.20.1270.10 (GENE3D), G3DSA:2.60.34.10 (GENE3D), PTHR19375 (PANTHER), SignalP-NN(euk) (SIGNALP), SSF100920 (SUPERFAMILY), SSF100934 (SUPERFAMILY) |
| 22 | lib_tri_Filter15_IDBA_contig_1345_minus1 | 58 | | 305 | 0 | 100% | 1 | F:protein binding | IPR003599 (SMART); IPR003961 (PFAM); IPR007110 (PROFILE); IPR013098 (PFAM); IPR013783 (G3DSA:2.60.40.GENE3D); SSF48726 (SUPERFAMILY) |

| | | | | | | | | | |
|---|---|---|---|---|---|---|---|---|---|
| 23 | lib_tri_Filter15_IDBA_contig_1361_minus1 | 85 | | 414 | 0 | 100% | 1 | F:protein binding | IPR003598 (SMART); IPR003599 (SMART); IPR003961 (PFAM); IPR007110 (PROFILE); IPR013098 (PFAM); IPR013783 (G3DSA:2.60.40.GENE3D); PR00014 (PRINTS), SSF48726 (SUPERFAMILY) |
| 24 | lib_tri_Filter15_IDBA_contig_1363_plus2 | 59 | | 301 | 0 | 100% | 1 | F:protein binding | IPR003961 (PFAM); IPR013098 (PFAM); IPR013783 (G3DSA:2.60.40.GENE3D); PR00014 (PRINTS), SSF48726 (SUPERFAMILY) |
| 25 | lib_tri_Filter15_IDBA_contig_1367_minus1 | 67 | immunoglobulin i-set domain containing protein | 300 | 4.88E-43 | 44.10% | 4 | F:transferase activity; C:cell; P:biological_process; F:protein binding | IPR003598 (SMART); IPR003599 (SMART); IPR007110 (PROFILE); IPR013098 (PFAM); IPR013783 (G3DSA:2.60.40.GENE3D); PTHR10489 (PANTHER), PF13895 (PFAM), SSF48726 (SUPERFAMILY) |
| 26 | lib_tri_Filter15_IDBA_contig_1381_minus2 | 89 | | 297 | 0 | 100% | 1 | F:carbohydrate binding | IPR001304 (PFAM); IPR016186 (G3DSA:3.10.100.GENE3D); IPR016187 (SUPERFAMILY); PTHR22802 (PANTHER), tmhmm (TMHMM) |
| 27 | lib_tri_Filter15_IDBA_contig_1423_plus3 | 76 | | 292 | 0 | 100% | 1 | F:protein binding | IPR003599 (SMART); IPR003961 (PFAM); IPR007110 (PROFILE); IPR013098 (PFAM); IPR013783 (G3DSA:2.60.40.GENE3D); PR00014 (PRINTS), PF13895 (PFAM), SSF48726 (SUPERFAMILY) |
| 28 | lib_tri_Filter15_IDBA_contig_1425_minus1 | 65 | muscle m-line assembly protein unc-89 | 292 | 2.12E-30 | 49.05% | 2 | P:biological_process; F:protein binding | IPR003598 (SMART); IPR003599 (SMART); IPR007110 (PROFILE); IPR013098 (PFAM); IPR013783 (G3DSA:2.60.40.GENE3D); PTHR10489 (PANTHER), SSF48726 (SUPERFAMILY) |
| 29 | lib_tri_Filter15_IDBA_contig_1448_plus1 | 32 | tolloid-like protein 1-like | 288 | 2.39E-36 | 46.95% | 2 | F:binding; F:hydrolase activity | IPR000859 (G3DSA:2.60.120.GENE3D); PTHR10127 (PANTHER) |
| 30 | lib_tri_Filter15_IDBA_contig_1469_plus3 | 57 | | 308 | 0 | 100% | 1 | F:protein binding | IPR003961 (PFAM); IPR013783 (G3DSA:2.60.40.GENE3D); PR00014 (PRINTS) |
| 31 | lib_tri_Filter15_IDBA_contig_1481_plus3 | 81 | | 283 | 0 | 100% | 1 | F:protein binding | IPR003961 (PFAM); IPR013098 (PFAM); IPR013783 (G3DSA:2.60.40.GENE3D); PR00014 (PRINTS), SSF48726 (SUPERFAMILY) |
| 32 | lib_tri_Filter15_IDBA_contig_1486_plus1 | 2808 | enolase | 856 | 0 | 85.85% | 21 | P:response to biotic stimulus; C:cytoplasm; P:regulation of biological process; P:cell growth; C:cytosol; C:protein complex; F:DNA binding; ; F:protein binding; F:transcription regulator activity; F:binding; C:cell; F:sequence-specific DNA binding transcription factor activity; P:carbohydrate metabolic process; P:generation of precursor metabolites and energy; P:catabolic process; P:biosynthetic process; F:hydrolase activity; F:catalytic activity; C:nucleus; C:plasma membrane | IPR000941 (PRINTS); IPR020809 (PROSITE); IPR020810 (PFAM); IPR020811 (PFAM); G3DSA:3.20.20.120 (GENE3D), G3DSA:3.30.390.10 (GENE3D), tmhmm (TMHMM), SSF51604 (SUPERFAMILY), SSF54826 (SUPERFAMILY) |
| 33 | lib_tri_Filter15_IDBA_contig_1504_minus2 | 127 | tyrosyl-trna cytoplasmic | 386 | 2.12E-153 | 75.75% | 7 | P:translation; P:nucleobase-containing compound metabolic process; ; F:nucleotide binding; C:cytoplasm; F:RNA binding; F:catalytic activity | IPR002305 (PFAM); IPR002547 (PFAM); IPR012340 (G3DSA:2.40.50.GENE3D); IPR014729 (G3DSA:3.40.50.GENE3D); IPR023617 (PTHR11946:PANTHER); G3DSA:1.10.240.10 (GENE3D), PTHR11946 (PANTHER), SSF52374 (SUPERFAMILY) |
| 34 | lib_tri_Filter15_IDBA_contig_1512_minus3 | 117 | | 334 | 0 | 100% | 1 | F:protein binding | IPR003599 (SMART); IPR003961 (PFAM); IPR007110 (PROFILE); IPR013098 (PFAM); IPR013783 (G3DSA:2.60.40.GENE3D); PR00014 (PRINTS), SSF48726 (SUPERFAMILY) |

| | | | | | | | | | | |
|---|---|---|---|---|---|---|---|---|---|---|
| 35 | lib_tri_Filter15_IDBA_contig_1513_minus2 | 95 | | 307 | 0 | 100% | 1 | F:protein binding | IPR003599 (SMART); IPR003961 (PFAM); IPR013098 (PFAM); IPR013783 (G3DSA:2.60.40.GENE3D); PR00014 (PRINTS), SSF48726 (SUPERFAMILY) | |
| 36 | lib_tri_Filter15_IDBA_contig_1514_minus1 | 41 | | 163 | 0 | 100% | 1 | F:protein binding | IPR003961 (PFAM); IPR013783 (G3DSA:2.60.40.GENE3D); PR00014 (PRINTS) | |
| 37 | lib_tri_Filter15_IDBA_contig_1524_minus1 | 58 | aael005317- partial | 352 | 2.94E-146 | 56.20% | 11 | P:cell cycle; P:anatomical structure morphogenesis; P:organelle organization; P:biological_process; P:cell differentiation; P:multicellular organismal development; C:organelle; C:intracellular; F:structural molecule activity; C:cytoplasm; F:protein binding | IPR003598 (SMART); IPR003599 (SMART); IPR007110 (PROFILE); IPR013098 (PFAM); IPR013783 (G3DSA:2.60.40.GENE3D); SSF48726 (SUPERFAMILY) | |
| 38 | lib_tri_Filter15_IDBA_contig_1556_minus2 | 906 | ---NA--- | 487 | | | 0 | - | G3DSA:2.10.60.10 (GENE3D), SignalP-NN(euk) (SIGNALP), SSF57302 (SUPERFAMILY) | Yes |
| 39 | lib_tri_Filter15_IDBA_contig_1574_minus3 | 121 | transforming growth factor-beta-induced protein ig-h3 | 273 | 7.71E-15 | 51.75% | 3 | P:multicellular organismal development; P:biological_process; C:proteinaceous extracellular matrix | IPR000782 (G3DSA:2.30.180.GENE3D); PTHR10900 (PANTHER) | |
| 40 | lib_tri_Filter15_IDBA_contig_1615_minus2 | 41 | titin | 269 | 1.32E-32 | 48.40% | 3 | F:transferase activity; P:biological_process; F:protein binding | IPR003599 (SMART); IPR003961 (SUPERFAMILY); IPR007110 (PROFILE); IPR013098 (PFAM); IPR013783 (G3DSA:2.60.40.GENE3D); PTHR25964 (PANTHER), PTHR25964:SF5 (PANTHER), SSF48726 (SUPERFAMILY) | |
| 41 | lib_tri_Filter15_IDBA_contig_1628_minus2 | 246 | von willebrand factor type egf and pentraxin domain-containing protein 1 | 424 | 2.83E-24 | 48.30% | 3 | F:calcium ion binding; F:chromatin binding; F:carbohydrate binding | IPR000436 (PFAM); IPR000884 (SMART); IPR013032 (PROSITE); G3DSA:2.10.70.10 (GENE3D), G3DSA:2.20.100.10 (GENE3D), PTHR19325 (PANTHER), SignalP-NN(euk) (SIGNALP), tmhmm (TMHMM) | |
| 42 | lib_tri_Filter15_IDBA_contig_1638_plus3 | 80 | neuroendocrine convertase 1 | 338 | 7.22E-43 | 63.30% | 3 | F:peptidase activity; P:protein metabolic process; P:catabolic process | IPR002884 (PFAM); IPR008979 (G3DSA:2.60.120.GENE3D); IPR015500 (PANTHER); PTHR10795:SF11 (PANTHER) | |
| 43 | lib_tri_Filter15_IDBA_contig_1639_plus3 | 727 | globin x | 315 | 2.16E-38 | 53.95% | 3 | F:binding; P:transport; F:oxygen binding | IPR000971 (PFAM); IPR009050 (SUPERFAMILY); IPR012292 (G3DSA:1.10.490.GENE3D); IPR013316 (PRINTS); PTHR22924 (PANTHER) | |
| 44 | lib_tri_Filter15_IDBA_contig_1700_minus2 | 129 | protein | 322 | 9.51E-19 | 64.65% | 3 | F:enzyme regulator activity; P:response to external stimulus; P:response to stress | IPR000864 (PRODOM); G3DSA:3.30.10.10 (GENE3D), SignalP-NN(euk) (SIGNALP), tmhmm (TMHMM) | Yes |
| 45 | lib_tri_Filter15_IDBA_contig_1711_minus3 | 118 | ---NA--- | 256 | | | 0 | - | no IPS match | |
| 46 | lib_tri_Filter15_IDBA_contig_1711_plus2 | 118 | ---NA--- | 257 | | | 0 | - | IPR008160 (PFAM); PTHR24023 (PANTHER) | |
| 47 | lib_tri_Filter15_IDBA_contig_1737_minus2 | 387 | type alpha partial | 379 | 7.74E-117 | 68.85% | 9 | P:anatomical structure morphogenesis; P:cellular component organization; P:cell differentiation; P:multicellular organismal development; P:biological_process; C:proteinaceous extracellular matrix; F:protein binding; P:embryo development; F:structural molecule activity | IPR000885 (PRODOM); PTHR24637 (PANTHER), PTHR24637:SF84 (PANTHER) | |
| 48 | lib_tri_Filter15_IDBA_contig_1738_minus1 | 131 | fibrillar collagen chain fap1 alpha | 255 | 1.12E-13 | 72.00% | 2 | F:extracellular matrix structural constituent; C:collagen | IPR008160 (PFAM); PTHR24023 (PANTHER) | |
| 49 | lib_tri_Filter15_IDBA_contig_1738_plus1 | 131 | ---NA--- | 255 | | | 0 | - | no IPS match | |

| | | | | | | | | | | |
|---|---|---|---|---|---|---|---|---|---|---|
| 50 | lib_tri_Filter15_IDBA_contig_174_minus3 | 83 | cysteine-rich secretory protein mr30 | 216 | 2.85E-30 | 51.50% | 6 | P:proteolysis; F:hydrolase activity; F:peptidase activity; C:extracellular region; F:ion channel inhibitor activity; F:calcium channel inhibitor activity | IPR001283 (PRINTS); IPR014044 (G3DSA:3.40.33.GENE3D); SignalP-NN(euk) (SIGNALP), tmhmm (TMHMM) | Yes |
| 51 | lib_tri_Filter15_IDBA_contig_1748_plus2 | 46 | collagen alpha-2 chain | 253 | 4.79E-09 | 52.55% | 4 | P:biological_process; P:multicellular organismal development; C:proteinaceous extracellular matrix; F:protein binding | IPR001007 (PFAM); IPR008160 (PFAM); G3DSA:2.10.70.10 (GENE3D), PTHR24023 (PANTHER), PTHR24023:SF54 (PANTHER), SignalP-NN(euk) (SIGNALP), tmhmm (TMHMM), SSF57603 (SUPERFAMILY) | Yes |
| 52 | lib_tri_Filter15_IDBA_contig_1782_plus1 | 64 | | 251 | 0 | 100% | 1 | F:protein binding | IPR003599 (SMART); IPR007110 (PROFILE); IPR013098 (PFAM); IPR013783 (G3DSA:2.60.40.GENE3D); IPR020675 (PANTHER); IPR020682 (PTHR22964:PANTHER); SSF48726 (SUPERFAMILY) | |
| 53 | lib_tri_Filter15_IDBA_contig_1794_minus3 | 38 | | 249 | 0 | 100% | 1 | F:protein binding | IPR003961 (PFAM); IPR013783 (G3DSA:2.60.40.GENE3D); PR00014 (PRINTS), SSF48726 (SUPERFAMILY) | |
| 54 | lib_tri_Filter15_IDBA_contig_1808_minus2 | 47 | | 248 | 0 | 100% | 1 | F:protein binding | IPR003599 (SMART); IPR007110 (PROFILE); IPR013098 (PFAM); IPR013783 (G3DSA:2.60.40.GENE3D); PTHR13817 (PANTHER), PTHR13817:SF2 (PANTHER), SSF48726 (SUPERFAMILY) | |
| 55 | lib_tri_Filter15_IDBA_contig_1823_minus1 | 83 | immunoglobulin i-set domain protein | 247 | 2.86E-19 | 52.75% | 4 | F:transferase activity; C:intracellular; P:biological_process; F:protein binding | IPR003598 (SMART); IPR003599 (SMART); IPR007110 (PROFILE); IPR013098 (PFAM); IPR013783 (G3DSA:2.60.40.GENE3D); PTHR10489 (PANTHER), SSF48726 (SUPERFAMILY) | |
| 56 | lib_tri_Filter15_IDBA_contig_1834_minus2 | 376 | ---NA--- | 327 | | | 0 | - | IPR008160 (PFAM); PTHR24022 (PANTHER), PTHR24022:SF54 (PANTHER), tmhmm (TMHMM) | |
| 57 | lib_tri_Filter15_IDBA_contig_1834_plus2 | 376 | ---NA--- | 327 | | | 0 | - | SignalP-NN(euk) (SIGNALP) | |
| 58 | lib_tri_Filter15_IDBA_contig_1901_minus3 | 45 | vascular endothelial growth factor receptor 1 precursor | 239 | 2.24E-14 | 49.90% | 14 | P:anatomical structure morphogenesis; P:multicellular organismal development; P:signal transduction; F:protein binding; F:nucleotide binding; C:organelle; C:intracellular; P:regulation of biological process; P:cell proliferation; P:metabolic process; F:protein kinase activity; F:receptor activity; C:plasma membrane; P:biological_process | IPR003598 (SMART); IPR003599 (SMART); IPR007110 (PROFILE); IPR013098 (PFAM); IPR013783 (G3DSA:2.60.40.GENE3D); PTHR10489 (PANTHER), SSF48726 (SUPERFAMILY) | |
| 59 | lib_tri_Filter15_IDBA_contig_1911_plus1 | 33 | cathepsin z precursor | 239 | 5.82E-33 | 81.75% | 7 | P:anatomical structure morphogenesis; P:multicellular organismal development; C:extracellular space; F:peptidase activity; P:protein metabolic process; P:catabolic process; C:endoplasmic reticulum | IPR000668 (PFAM); IPR013128 (PANTHER); IPR025661 (PROSITE); G3DSA:3.90.70.10 (GENE3D), PTHR12411:SF14 (PANTHER), tmhmm (TMHMM), SSF54001 (SUPERFAMILY) | |
| 60 | lib_tri_Filter15_IDBA_contig_1924_plus3 | 8585 | globin x | 345 | 1.05E-44 | 55.10% | 3 | F:binding; P:transport; F:oxygen binding | IPR000971 (PFAM); IPR009050 (SUPERFAMILY); IPR012292 (G3DSA:1.10.490.GENE3D); IPR013316 (PRINTS); PTHR22924 (PANTHER), tmhmm (TMHMM) | |
| 61 | lib_tri_Filter15_IDBA_contig_2040_plus1 | 49 | histone h2av | 229 | 2.09E-65 | 99.30% | 6 | C:chromosome; F:DNA binding; P:organelle organization; P:multicellular organismal development; C:nucleus; F:protein binding | IPR002119 (PRINTS); IPR007125 (PFAM); IPR009072 (G3DSA:1.10.20.GENE3D); PTHR23430 (PANTHER) | |

| | | | | | | | | | | |
|---|---|---|---|---|---|---|---|---|---|---|
| 62 | lib_tri_Filter15_IDBA_contig_2043_plus2 | 327 | nidogen 2 precursor | 228 | 9.14E-12 | 48.35% | 2 | P:cell-matrix adhesion; F:calcium ion binding | IPR000716 (G3DSA:4.10.800.GENE3D); IPR022339 (PANTHER); SignalP-NN(euk) (SIGNALP), tmhmm (TMHMM) | |
| 63 | lib_tri_Filter15_IDBA_contig_2090_minus2 | 426 | | 435 | 0 | 100% | 1 | F:protein binding | IPR007110 (PROFILE); IPR013098 (PFAM); IPR013783 (G3DSA:2.60.40.GENE3D); PTHR10489 (PANTHER), SSF48726 (SUPERFAMILY) | |
| 64 | lib_tri_Filter15_IDBA_contig_2108_plus3 | 49 | camp-dependent protein kinase catalytic | 223 | 4.99E-102 | 73.15% | 7 | P:cellular protein modification process; F:nucleotide binding; P:anatomical structure morphogenesis; P:multicellular organismal development; F:protein kinase activity; C:intracellular; C:protein complex | IPR000719 (PFAM); IPR000961 (SMART); IPR002290 (SMART); IPR011009 (SUPERFAMILY); G3DSA:1.10.510.10 (GENE3D), PTHR24353 (PANTHER) | |
| 65 | lib_tri_Filter15_IDBA_contig_2110_minus3 | 48 | neuronal acetylcholine receptor subunit alpha-9 | 223 | 5.61E-53 | 60.30% | 10 | P:response to external stimulus; P:response to abiotic stimulus; P:anatomical structure morphogenesis; P:embryo development; P:cellular homeostasis; F:protein binding; F:ion channel activity; P:ion transport; C:plasma membrane; C:cell | IPR006201 (PRINTS); IPR006202 (G3DSA:2.70.170.GENE3D); IPR018000 (PROSITE); PTHR18945:SF127 (PANTHER), tmhmm (TMHMM) | |
| 66 | lib_tri_Filter15_IDBA_contig_2134_minus2 | 96 | | 222 | 0 | 100% | 1 | F:protein binding | IPR003961 (PFAM); IPR013098 (PFAM); IPR013783 (G3DSA:2.60.40.GENE3D); PR00014 (PRINTS), SSF48726 (SUPERFAMILY) | |
| 67 | lib_tri_Filter15_IDBA_contig_2143_minus2 | 46 | protein | 221 | 1.37E-42 | 58.30% | 12 | F:receptor binding; C:extracellular space; P:signal transduction; P:platelet activation; P:protein polymerization; C:external side of plasma membrane; C:fibrinogen complex; P:response to calcium ion; F:eukaryotic cell surface binding; C:cell cortex; C:platelet alpha granule; F:protein binding, bridging | IPR002181 (PFAM); IPR014715 (G3DSA:4.10.530.GENE3D); IPR014716 (G3DSA:3.90.215.GENE3D); IPR020837 (PROSITE); PTHR19143 (PANTHER) | |
| 68 | lib_tri_Filter15_IDBA_contig_2184_plus3 | 271 | nadh dehydrogenase isoform 1 | 300 | 1.83E-49 | 79.25% | 6 | P:generation of precursor metabolites and energy; P:biosynthetic process; P:lipid metabolic process; F:transporter activity; F:binding; C:mitochondrion | IPR003231 (PRODOM); IPR009081 (G3DSA:1.10.1200.GENE3D); PTHR20863 (PANTHER), PTHR20863:SF5 (PANTHER), tmhmm (TMHMM) | |
| 69 | lib_tri_Filter15_IDBA_contig_2233_plus2 | 85 | deleted in malignant brain tumors 1 | 225 | 7.68E-30 | 65.55% | 10 | P:regulation of biological process; P:protein transport; F:protein binding; P:transport; P:cell differentiation; C:cytoplasm; C:extracellular region; P:biological_process; C:cell; F:receptor activity | IPR001190 (PRINTS); IPR017448 (SMART); G3DSA:3.10.250.10 (GENE3D), PTHR19331 (PANTHER), SignalP-NN(euk) (SIGNALP), tmhmm (TMHMM) | Yes |
| 70 | lib_tri_Filter15_IDBA_contig_225_minus2 | 113 | deleted in malignant brain tumors 1 | 255 | 3.50E-59 | 58.55% | 2 | F:receptor activity; C:cell | IPR001190 (PRINTS); IPR017448 (SMART); G3DSA:3.10.250.10 (GENE3D), PTHR19331 (PANTHER), PTHR19331:SF118 (PANTHER) | |
| 71 | lib_tri_Filter15_IDBA_contig_2281_plus3 | 101 | deleted in malignant brain tumors 1 | 260 | 8.37E-24 | 65.10% | 2 | F:receptor activity; C:cell | IPR001190 (PRINTS); IPR017448 (SMART); G3DSA:3.10.250.10 (GENE3D), PTHR19331 (PANTHER), PTHR19331:SF118 (PANTHER) | |
| 72 | lib_tri_Filter15_IDBA_contig_2286_plus1 | 270 | | 428 | 0 | 100% | 1 | C:cell | IPR000998 (PFAM); IPR008985 (SUPERFAMILY); PTHR23282 (PANTHER) | |
| 73 | lib_tri_Filter15_IDBA_contig_2299_plus3 | 62 | | 295 | | | | | IPR013126 (PRINTS); IPR018181 (PROSITE); G3DSA:3.30.30.30 (GENE3D), G3DSA:3.30.420.40 (GENE3D), G3DSA:3.90.640.10 (GENE3D), PTHR19375 (PANTHER), SSF53067 (SUPERFAMILY) | |

| | | | | | | | | | |
|---|---|---|---|---|---|---|---|---|---|
| 74 | lib_tri_Filter15_IDBA_contig_2307_minus2 | 52 | | 209 | 0 | 100% | 1 | F:protein binding | IPR003599 (SMART); IPR003961 (PFAM); IPR007110 (PROFILE); IPR013098 (PFAM); IPR013783 (G3DSA:2.60.40.GENE3D); PR00014 (PRINTS), PTHR25964 (PANTHER), PTHR25964:SF5 (PANTHER), PF13895 (PFAM), SSF48726 (SUPERFAMILY) |
| 75 | lib_tri_Filter15_IDBA_contig_2326_plus1 | 60 | | 208 | 0 | 100% | 1 | F:protein binding | IPR003961 (PFAM); IPR013098 (PFAM); IPR013783 (G3DSA:2.60.40.GENE3D); PR00014 (PRINTS), SSF48726 (SUPERFAMILY) |
| 76 | lib_tri_Filter15_IDBA_contig_2336_plus1 | 30 | protein-glutamine gamma-glutamyltransferase k-like | 208 | 7.57E-58 | 56.45% | 4 | F:metal ion binding; P:peptide cross-linking; F:protein-glutamine gamma-glutamyltransferase activity; F:transferase activity | IPR002931 (G3DSA:3.90.260.GENE3D); IPR013783 (G3DSA:2.60.40.GENE3D); IPR023608 (PANTHER); PTHR11590:SF17 (PANTHER), SSF54001 (SUPERFAMILY) |
| 77 | lib_tri_Filter15_IDBA_contig_235_minus2 | 2298 | annexin a4 | 1041 | 6.35E-110 | 70.70% | 2 | F:calcium ion binding; F:lipid binding | IPR001464 (PRINTS); IPR018252 (PROSITE); IPR018502 (G3DSA:1.10.220.GENE3D); tmhmm (TMHMM) |
| 78 | lib_tri_Filter15_IDBA_contig_2384_plus1 | 140 | cysteine-rich secretory protein mr30 | 294 | 9.05E-59 | 53.85% | 7 | P:proteolysis; F:hydrolase activity; F:peptidase activity; C:extracellular region; F:ion channel inhibitor activity; F:calcium channel inhibitor activity; F:potassium channel inhibitor activity | IPR001283 (PRINTS); IPR002413 (PRINTS); IPR014044 (G3DSA:3.40.33.GENE3D) |
| 79 | lib_tri_Filter15_IDBA_contig_2386_plus1 | 25 | | 205 | 0 | 100% | 1 | F:protein binding | IPR003599 (SMART); IPR007110 (PROFILE); IPR013098 (PFAM); IPR013783 (G3DSA:2.60.40.GENE3D); IPR020675 (PANTHER); IPR020682 (PTHR22964:PANTHER); SSF48726 (SUPERFAMILY) |
| 80 | lib_tri_Filter15_IDBA_contig_241_minus1 | 6256 | ferritin | 329 | 1.39E-73 | 82.65% | 6 | C:cytoplasm; P:cellular homeostasis; F:binding; F:catalytic activity; P:ion transport; P:metabolic process | IPR001519 (PANTHER); IPR008331 (PFAM); IPR009040 (PROFILE); IPR009078 (SUPERFAMILY); IPR012347 (G3DSA:1.20.1260.GENE3D) |
| 81 | lib_tri_Filter15_IDBA_contig_2431_plus3 | 61 | fbn8 | 202 | 6.54E-21 | 55.80% | 3 | F:receptor binding; C:extracellular space; P:signal transduction | IPR002181 (PFAM); IPR014715 (G3DSA:4.10.530.GENE3D); IPR014716 (G3DSA:3.90.215.GENE3D); PTHR19143 (PANTHER) |
| 82 | lib_tri_Filter15_IDBA_contig_2441_minus1 | 61 | cre-unc-22 protein | 256 | 7.09E-75 | 59.95% | 7 | F:protein kinase activity; C:cytoplasm; P:multicellular organismal development; P:regulation of biological process; P:biological_process; P:growth; F:protein binding | IPR003599 (SMART); IPR003961 (PFAM); IPR007110 (PROFILE); IPR013098 (PFAM); IPR013783 (G3DSA:2.60.40.GENE3D); PR00014 (PRINTS), SSF48726 (SUPERFAMILY) |
| 83 | lib_tri_Filter15_IDBA_contig_2456_minus3 | 55 | adenosine deaminase cecr1 | 200 | 5.96E-66 | 74.65% | 10 | F:hydrolase activity; F:binding; P:biosynthetic process; P:nucleobase-containing compound metabolic process; C:extracellular space; F:protein binding; P:catabolic process; F:receptor binding; P:multicellular organismal development; C:Golgi apparatus | IPR001365 (PFAM); G3DSA:3.20.20.140 (GENE3D), PTHR11409 (PANTHER), PTHR11409:SF5 (PANTHER), SSF51556 (SUPERFAMILY) |
| 84 | lib_tri_Filter15_IDBA_contig_246_plus2 | 101 | carcinolectin 5a isoform | 190 | 3.89E-21 | 62.50% | 2 | P:biological_process; F:carbohydrate binding | IPR002181 (PFAM); IPR014715 (G3DSA:4.10.530.GENE3D); IPR020837 (PROSITE); PTHR19143 (PANTHER) |
| 85 | lib_tri_Filter15_IDBA_contig_2468_minus2 | 57 | | 239 | 0 | 100% | 1 | F:protein binding | IPR003961 (PFAM); IPR013783 (G3DSA:2.60.40.GENE3D); PR00014 (PRINTS) |
| 86 | lib_tri_Filter15_IDBA_contig_2469_minus1 | 45 | | 200 | 0 | 100% | 1 | F:protein binding | IPR003961 (PFAM); IPR007110 (PROFILE); IPR013098 (PFAM); IPR013783 (G3DSA:2.60.40.GENE3D); PR00014 (PRINTS), SSF48726 (SUPERFAMILY) |

| | | | | | | | | | | |
|---|---|---|---|---|---|---|---|---|---|---|
| 87 | lib_tri_Filter15_IDBA_contig_2477_minus3 | 554 | carbonic anhydrase | 527 | 2.69E-105 | 64.25% | 3 | F:catalytic activity; P:metabolic process; F:binding | IPR001148 (G3DSA:3.10.200.GENE3D); IPR018338 (PROSITE); IPR018440 (PTHR18952:PANTHER); IPR023561 (PANTHER) | |
| 88 | lib_tri_Filter15_IDBA_contig_2506_minus2 | 35 | muscle m-line assembly protein unc-89 | 198 | 6.01E-57 | 49.70% | 1 | F:protein binding | IPR003598 (SMART); IPR003599 (SMART); IPR007110 (PROFILE); IPR013098 (PFAM); IPR013783 (G3DSA:2.60.40.GENE3D); PTHR10489 (PANTHER), SSF48726 (SUPERFAMILY) | |
| 89 | lib_tri_Filter15_IDBA_contig_2530_minus2 | 52 | projectin short variant | 243 | 3.18E-106 | 58.15% | 2 | F:transferase activity; F:protein binding | IPR007110 (PROFILE); IPR013098 (PFAM); IPR013783 (G3DSA:2.60.40.GENE3D); SSF48726 (SUPERFAMILY) | |
| 90 | lib_tri_Filter15_IDBA_contig_2535_plus2 | 35 | carboxypeptidase b | 196 | 2.00E-28 | 55.30% | 4 | F:peptidase activity; P:protein metabolic process; P:catabolic process; F:binding | IPR000834 (PFAM); G3DSA:3.40.630.10 (GENE3D), PTHR11705 (PANTHER), SSF53187 (SUPERFAMILY) | |
| 91 | lib_tri_Filter15_IDBA_contig_2549_minus2 | 33 | sushi repeat-containing protein srpx2 | 195 | 4.11E-14 | 57.70% | 1 | F:protein binding | IPR000742 (PFAM); IPR000884 (PFAM); IPR013032 (PROSITE); PR01705 (PRINTS), G3DSA:2.10.25.10 (GENE3D), G3DSA:2.20.100.10 (GENE3D), PTHR12582 (PANTHER), SSF57196 (SUPERFAMILY) | |
| 92 | lib_tri_Filter15_IDBA_contig_2636_minus2 | 46 | | 192 | 0 | 100% | 3 | F:enzyme regulator activity; P:response to external stimulus; P:response to stress | IPR000864 (PRODOM); G3DSA:3.30.10.10 (GENE3D) | |
| 93 | lib_tri_Filter15_IDBA_contig_2668_plus1 | 35 | blastula protease 10-like | 191 | 5.96E-17 | 52.85% | 3 | F:peptidase activity; P:protein metabolic process; P:catabolic process | IPR001506 (PRINTS); IPR003582 (PFAM); IPR024079 (G3DSA:3.40.390.GENE3D); PTHR10127 (PANTHER), PTHR10127:SF334 (PANTHER), SSF55486 (SUPERFAMILY) | |
| 94 | lib_tri_Filter15_IDBA_contig_2686_minus1 | 24 | cathepsin l-like cysteine proteinase | 190 | 3.96E-46 | 86.50% | 3 | F:peptidase activity; P:protein metabolic process; P:catabolic process | IPR000668 (PFAM); IPR013128 (PANTHER); IPR025660 (PROSITE); IPR025661 (PROSITE); G3DSA:3.90.70.10 (GENE3D), PTHR12411:SF149 (PANTHER), SSF54001 (SUPERFAMILY) | |
| 95 | lib_tri_Filter15_IDBA_contig_2703_minus3 | 371 | protein acr-11 | 393 | 1.36E-30 | 49.95% | 3 | P:ion transport; C:cell; F:ion channel activity | IPR006201 (PANTHER); IPR006202 (G3DSA:2.70.170.GENE3D); PTHR18945:SF89 (PANTHER), SignalP-NN(euk) (SIGNALP) | Yes |
| 96 | lib_tri_Filter15_IDBA_contig_2752_minus3 | 29 | transmembrane protease serine 3 | 187 | 2.92E-32 | 59.50% | 3 | F:peptidase activity; P:protein metabolic process; P:catabolic process | IPR001254 (PFAM); IPR009003 (SUPERFAMILY); G3DSA:2.40.10.10 (GENE3D), PTHR24256 (PANTHER) | |
| 97 | lib_tri_Filter15_IDBA_contig_2781_plus1 | 45 | microfibril-associated glycoprotein 4-like | 186 | 7.71E-38 | 57.40% | 3 | F:receptor binding; P:signal transduction; C:extracellular space | IPR002181 (PFAM); IPR014715 (G3DSA:4.10.530.GENE3D); IPR014716 (G3DSA:3.90.215.GENE3D); PTHR19143 (PANTHER) | |
| 98 | lib_tri_Filter15_IDBA_contig_2793_plus1 | 24 | granulin- partial | 185 | 6.80E-44 | 65.20% | 8 | C:mitochondrion; P:embryo implantation; P:blastocyst hatching; P:positive regulation of epithelial cell proliferation; C:intracellular membrane-bounded organelle; C:extracellular space; F:cytokine activity; C:extracellular region | IPR000118 (PFAM); PTHR12274 (PANTHER), PTHR12274:SF0 (PANTHER), SSF57277 (SUPERFAMILY) | |
| 99 | lib_tri_Filter15_IDBA_contig_2802_minus3 | 53 | | 182 | 0 | 100% | 1 | F:protein binding | IPR003599 (SMART); IPR007110 (PROFILE); IPR013098 (PFAM); IPR013783 (G3DSA:2.60.40.GENE3D); IPR020675 (PANTHER); IPR020682 (PTHR22964:PANTHER); SSF48726 (SUPERFAMILY) | |
| 100 | lib_tri_Filter15_IDBA_contig_2803_minus3 | 81 | | 284 | 0 | 100% | 1 | F:protein binding | IPR003598 (SMART); IPR003599 (SMART); IPR007110 (PROFILE); IPR013098 (PFAM); IPR013783 (G3DSA:2.60.40.GENE3D); IPR020675 (PANTHER); IPR020682 (PTHR22964:PANTHER); SSF48726 (SUPERFAMILY) | |

| | | | | | | | | | | |
|---|---|---|---|---|---|---|---|---|---|---|
| 101 | lib_tri_Filter15_IDBA_contig_2804_minus2 | 57 | isoform q | 169 | 5.96E-26 | 55.40% | 1 | F:protein binding | IPR003599 (SMART); IPR007110 (PROFILE); IPR013098 (PFAM); IPR013783 (G3DSA:2.60.40.GENE3D); IPR020675 (PANTHER); IPR020682 (PTHR22964:PANTHER); SSF48726 (SUPERFAMILY) | |
| 102 | lib_tri_Filter15_IDBA_contig_2805_minus1 | 44 | | 185 | 0 | 100% | 1 | F:protein binding | IPR003599 (SMART); IPR007110 (PROFILE); IPR013098 (PFAM); IPR013783 (G3DSA:2.60.40.GENE3D); IPR020675 (PANTHER); IPR020682 (PTHR22964:PANTHER); SSF48726 (SUPERFAMILY) | |
| 103 | lib_tri_Filter15_IDBA_contig_2824_minus2 | 34 | a disintegrin and metalloproteinase with thrombospondin motifs 2-like | 184 | 8.08E-09 | 47.95% | 1 | F:peptidase activity | IPR024079 (G3DSA:3.40.390.GENE3D); PTHR13723 (PANTHER) | |
| 104 | lib_tri_Filter15_IDBA_contig_2833_plus3 | 36 | neuronal acetylcholine receptor subunit alpha-7-like | 183 | 1.88E-36 | 59.45% | 3 | P:ion transport; C:cell; F:ion channel activity | IPR006201 (PRINTS); IPR006202 (G3DSA:2.70.170.GENE3D); IPR018000 (PROSITE); PTHR18945:SF127 (PANTHER), tmhmm (TMHMM) | |
| 105 | lib_tri_Filter15_IDBA_contig_2846_minus2 | 39 | fibroleukin precursor | 183 | 1.36E-27 | 58.80% | 1 | F:binding | IPR002181 (PFAM); IPR014715 (G3DSA:4.10.530.GENE3D); IPR014716 (G3DSA:3.90.215.GENE3D); PTHR19143 (PANTHER) | |
| 106 | lib_tri_Filter15_IDBA_contig_2901_plus3 | 22 | | 181 | 0 | 100% | 1 | F:calcium ion binding | IPR002048 (SMART); IPR011992 (G3DSA:1.10.238.GENE3D); IPR018247 (PROSITE); PTHR19972 (PANTHER), SSF47473 (SUPERFAMILY) | |
| 107 | lib_tri_Filter15_IDBA_contig_2908_minus1 | 26 | | 181 | 0 | 100% | 1 | F:protein binding | IPR003961 (PFAM); IPR007110 (PROFILE); IPR013098 (PFAM); IPR013783 (G3DSA:2.60.40.GENE3D); PTHR10489 (PANTHER), SSF48726 (SUPERFAMILY) | |
| 108 | lib_tri_Filter15_IDBA_contig_2931_minus2 | 267 | | 287 | | | | | PTHR21301 (PANTHER), tmhmm (TMHMM) | |
| 109 | lib_tri_Filter15_IDBA_contig_2953_minus1 | 21 | deleted in malignant brain tumors 1 | 179 | 2.53E-20 | 61.50% | 2 | F:receptor activity; C:cell | IPR001190 (PRINTS); IPR017448 (SMART); G3DSA:3.10.250.10 (GENE3D), PTHR19331 (PANTHER), PTHR19331:SF118 (PANTHER), SignalP-NN(euk) (SIGNALP), tmhmm (TMHMM) | Yes |
| 110 | lib_tri_Filter15_IDBA_contig_298_minus3 | 4799 | glucose dehydrogenase | 1567 | 1.08E-158 | 60.25% | 2 | F:nucleotide binding; P:metabolic process | IPR000172 (PFAM); IPR007867 (PFAM); G3DSA:3.50.50.60 (GENE3D), PTHR11552 (PANTHER), PTHR11552:SF10 (PANTHER), SSF51905 (SUPERFAMILY), SSF54373 (SUPERFAMILY) | |
| 111 | lib_tri_Filter15_IDBA_contig_2982_minus2 | 35 | galectin-3-binding protein precursor | 199 | 1.87E-25 | 61.20% | 2 | F:receptor activity; C:cell | IPR001190 (PRINTS); IPR017448 (SMART); G3DSA:3.10.250.10 (GENE3D), PTHR19331 (PANTHER), SignalP-NN(euk) (SIGNALP), tmhmm (TMHMM) | Yes |
| 112 | lib_tri_Filter15_IDBA_contig_2998_minus1 | 24 | | 169 | 0 | 100% | 3 | F:catalytic activity; F:antioxidant activity; P:metabolic process | IPR012336 (G3DSA:3.40.30.GENE3D); IPR019479 (PFAM); PTHR10681 (PANTHER), PTHR10681:SF45 (PANTHER) | |
| 113 | lib_tri_Filter15_IDBA_contig_3016_plus3 | 51 | | 177 | 0 | 100% | 1 | F:protein binding | IPR003961 (PFAM); IPR013783 (G3DSA:2.60.40.GENE3D); PR00014 (PRINTS) | |
| 114 | lib_tri_Filter15_IDBA_contig_3025_plus3 | 29 | ankyrin repeat-containing protein | 176 | 4.36E-19 | 55.75% | 1 | F:protein binding | IPR002110 (SMART); IPR020683 (G3DSA:1.25.40.GENE3D); PTHR24166 (PANTHER) | |
| 115 | lib_tri_Filter15_IDBA_contig_309_minus1 | 6672 | ferritin | 282 | 9.84E-74 | 89.10% | 6 | C:cytoplasm; P:cellular homeostasis; F:binding; F:catalytic activity; P:ion transport; P:metabolic process | IPR001519 (PANTHER); IPR008331 (PFAM); IPR009040 (PROFILE); IPR009078 (SUPERFAMILY); IPR012347 (G3DSA:1.20.1260.GENE3D); IPR014034 (PROSITE); tmhmm (TMHMM) | |
| 116 | lib_tri_Filter15_IDBA_contig_3095_plus1 | 47 | isoform a | 174 | 1.02E-15 | 44.80% | 1 | F:protein binding | IPR002172 (G3DSA:4.10.400.GENE3D); IPR023415 (PROSITE) | |

| | | | | | | | | | | |
|---|---|---|---|---|---|---|---|---|---|---|
| 117 | lib_tri_Filter15_IDBA_contig_310_minus1 | 2929 | ribosomal protein ubq l40e | 194 | 2.61E-82 | 97.65% | 11 | C:cytoskeleton; C:protein complex; F:structural molecule activity; C:lipid particle; P:cellular protein modification process; P:protein metabolic process; P:catabolic process; C:ribosome; C:cytosol; P:translation; F:protein binding | IPR000626 (PFAM); IPR001975 (PFAM); IPR019954 (PROSITE); IPR019955 (PROFILE); IPR019956 (PRINTS); G3DSA:3.10.20.90 (GENE3D), PTHR10666 (PANTHER), SSF54236 (SUPERFAMILY) | |
| 118 | lib_tri_Filter15_IDBA_contig_3105_minus2 | 33 | serine-threonine kinase receptor-associated | 130 | 7.67E-51 | 84.95% | 8 | F:receptor activity; C:cytoskeleton; C:protein complex; F:kinase activity; P:metabolic process; F:receptor binding; P:anatomical structure morphogenesis; P:multicellular organismal development | IPR001680 (PFAM); IPR011047 (SUPERFAMILY); IPR015943 (G3DSA:2.130.10.GENE3D); IPR017986 (PROFILE); PTHR19877 (PANTHER), PTHR19877:SF2 (PANTHER) | |
| 119 | lib_tri_Filter15_IDBA_contig_3138_minus2 | 35 | | 172 | | | | | IPR013126 (PRINTS); IPR018181 (PROSITE); G3DSA:2.60.34.10 (GENE3D), G3DSA:3.30.420.40 (GENE3D), G3DSA:3.90.640.10 (GENE3D), PTHR19375 (PANTHER), PTHR19375:SF1 (PANTHER), SSF100920 (SUPERFAMILY), SSF53067 (SUPERFAMILY) | |
| 120 | lib_tri_Filter15_IDBA_contig_320_minus2 | 1044 | ---NA--- | 530 | | | 0 | - | IPR008160 (PFAM); PTHR24023 (PANTHER), SignalP-NN(euk) (SIGNALP), tmhmm (TMHMM) | Yes |
| 121 | lib_tri_Filter15_IDBA_contig_320_plus2 | 1044 | ---NA--- | 530 | | | 0 | - | PTHR24023 (PANTHER), PTHR24023:SF238 (PANTHER), SignalP-NN(euk) (SIGNALP), tmhmm (TMHMM) | |
| 122 | lib_tri_Filter15_IDBA_contig_3210_plus1 | 51 | novel protein human titin | 188 | 3.25E-14 | 56.30% | 5 | P:anatomical structure morphogenesis; P:cell differentiation; P:multicellular organismal development; P:cytoskeleton organization; F:protein binding | IPR003599 (SMART); IPR007110 (PROFILE); IPR013098 (PFAM); IPR013783 (G3DSA:2.60.40.GENE3D); PTHR25963 (PANTHER), SSF48726 (SUPERFAMILY) | |
| 123 | lib_tri_Filter15_IDBA_contig_3234_minus2 | 22 | protein unc- isoform f | 169 | 4.56E-26 | 52.95% | 19 | C:cytoplasm; F:protein kinase activity; F:structural molecule activity; P:cytoskeleton organization; F:binding; C:cytoskeleton; P:multicellular organismal development; P:regulation of biological process; P:primary metabolic process; P:metabolic process; P:biological_process; F:enzyme regulator activity; P:anatomical structure morphogenesis; P:nucleobase-containing compound metabolic process; P:cell differentiation; F:protein binding; C:nucleus; P:cellular protein modification process; F:cytoskeletal protein binding | IPR003599 (SMART); IPR007110 (PROFILE); IPR013098 (PFAM); IPR013783 (G3DSA:2.60.40.GENE3D); IPR020675 (PANTHER); PTHR22964:SF3 (PANTHER), SSF48726 (SUPERFAMILY) | |
| 124 | lib_tri_Filter15_IDBA_contig_3381_minus1 | 22 | protein-glutamine gamma-glutamyltransferase k | 165 | 5.49E-60 | 72.80% | 8 | C:cytoskeleton; P:cell differentiation; P:multicellular organismal development; F:protein binding; P:cellular protein modification process; P:cellular component organization; C:cell; F:transferase activity | IPR002931 (G3DSA:3.90.260.GENE3D); IPR023608 (PANTHER); SSF54001 (SUPERFAMILY) | |
| 125 | lib_tri_Filter15_IDBA_contig_3398_plus3 | 33 | serine protease partial | 164 | 1.02E-23 | 55.50% | 3 | F:peptidase activity; P:protein metabolic process; P:catabolic process | IPR001254 (PFAM); IPR009003 (SUPERFAMILY); IPR018114 (PROSITE); G3DSA:2.40.10.10 (GENE3D), PTHR24265 (PANTHER) | |

| | | | | | | | | | | |
|---|---|---|---|---|---|---|---|---|---|---|
| 126 | lib_tri_Filter15_IDBA_contig_3432_plus1 | 14 | | 163 | 0 | 100% | 1 | F:enzyme regulator activity | IPR001820 (PANTHER); IPR008993 (SUPERFAMILY); IPR027465 (G3DSA:3.90.370.GENE3D); PTHR11844:SF9 (PANTHER) | |
| 127 | lib_tri_Filter15_IDBA_contig_3532_plus1 | 519 | serine protease | 377 | 2.17E-62 | 52.30% | 3 | F:peptidase activity; P:protein metabolic process; P:catabolic process | IPR001254 (PFAM); IPR001314 (PRINTS); IPR009003 (SUPERFAMILY); G3DSA:2.40.10.10 (GENE3D), PTHR24256 (PANTHER), SignalP-NN(euk) (SIGNALP), tmhmm (TMHMM) | Yes |
| 128 | lib_tri_Filter15_IDBA_contig_3535_minus1 | 78 | deleted in malignant brain tumors 1 partial | 206 | 1.28E-28 | 65.95% | 2 | F:receptor activity; C:cell | IPR001190 (PRINTS); IPR017448 (SMART); G3DSA:3.10.250.10 (GENE3D), PTHR19331 (PANTHER) | |
| 129 | lib_tri_Filter15_IDBA_contig_3536_minus1 | 68 | deleted in malignant brain tumors 1 partial | 193 | 1.32E-28 | 65.75% | 2 | F:receptor activity; C:cell | IPR001190 (PRINTS); IPR017448 (SMART); G3DSA:3.10.250.10 (GENE3D), PTHR19331 (PANTHER) | |
| 130 | lib_tri_Filter15_IDBA_contig_354_minus1 | 1797 | | 144 | 0 | 100% | 3 | F:binding; P:transport; F:oxygen binding | IPR000971 (PFAM); IPR009050 (SUPERFAMILY); IPR012292 (G3DSA:1.10.490.GENE3D); IPR013316 (PRINTS); PTHR22924 (PANTHER) | |
| 131 | lib_tri_Filter15_IDBA_contig_3554_minus2 | 208 | cysteine-rich secretory protein mr30 | 276 | 2.22E-40 | 50.25% | 6 | P:proteolysis; F:hydrolase activity; F:peptidase activity; C:extracellular region; F:ion channel inhibitor activity; F:calcium channel inhibitor activity | IPR001283 (PANTHER); IPR014044 (G3DSA:3.40.33.GENE3D) | |
| 132 | lib_tri_Filter15_IDBA_contig_3559_plus1 | 31 | protein unc- isoform g | 182 | 1.20E-12 | 59.30% | 10 | F:transferase activity; P:biological_process; C:cytoplasm; F:protein binding; P:signal transduction; P:anatomical structure morphogenesis; P:cell differentiation; P:multicellular organismal development; P:cytoskeleton organization; F:enzyme regulator activity | IPR003598 (SMART); IPR003599 (SMART); IPR007110 (PROFILE); IPR013098 (PFAM); IPR013783 (G3DSA:2.60.40.GENE3D); IPR020675 (PANTHER); PTHR22964:SF3 (PANTHER), SSF48726 (SUPERFAMILY) | |
| 133 | lib_tri_Filter15_IDBA_contig_3560_plus1 | 26 | | 134 | 0 | 100% | 1 | F:protein binding | IPR003598 (SMART); IPR003599 (SMART); IPR007110 (PROFILE); IPR013098 (PFAM); IPR013783 (G3DSA:2.60.40.GENE3D); PTHR10489 (PANTHER), SSF48726 (SUPERFAMILY) | |
| 134 | lib_tri_Filter15_IDBA_contig_3582_plus2 | 20 | | 138 | 0 | 100% | 1 | F:protein binding | IPR003961 (PFAM); IPR013783 (G3DSA:2.60.40.GENE3D); PR00014 (PRINTS) | |
| 135 | lib_tri_Filter15_IDBA_contig_3583_plus1 | 37 | | 160 | 0 | 100% | 1 | F:protein binding | IPR003961 (PFAM); IPR013783 (G3DSA:2.60.40.GENE3D); PR00014 (PRINTS) | |
| 136 | lib_tri_Filter15_IDBA_contig_3606_plus2 | 21 | cathepsin l | 159 | 1.23E-38 | 82.85% | 3 | F:peptidase activity; P:protein metabolic process; P:catabolic process | IPR000668 (PFAM); IPR013128 (PANTHER); IPR025660 (PROSITE); IPR025661 (PROSITE); G3DSA:3.90.70.10 (GENE3D), PTHR12411:SF149 (PANTHER), SSF54001 (SUPERFAMILY) | |
| 137 | lib_tri_Filter15_IDBA_contig_3615_minus1 | 16 | upf0764 protein c16orf89 homolog | 159 | 2.24E-23 | 49.90% | 4 | F:molecular_function; P:biological_process; C:extracellular region; C:cellular_component | no IPS match | |
| 138 | lib_tri_Filter15_IDBA_contig_3678_minus2 | 76 | aldehyde mitochondrial-like isoform 2 | 202 | 2.71E-74 | 86.05% | 9 | P:lipid metabolic process; P:secondary metabolic process; C:cytosol; F:enzyme regulator activity; P:metabolic process; P:signal transduction; F:catalytic activity; P:catabolic process; F:lipid binding | IPR015590 (PFAM); IPR016161 (SUPERFAMILY); IPR016163 (G3DSA:3.40.309.GENE3D); PTHR11699 (PANTHER), PTHR11699:SF46 (PANTHER) | |
| 139 | lib_tri_Filter15_IDBA_contig_3773_plus1 | 25 | cathepsin l | 155 | 6.57E-67 | 78.20% | 3 | F:peptidase activity; P:protein metabolic process; P:catabolic process | IPR000169 (PROSITE); IPR000668 (PFAM); IPR013128 (PANTHER); IPR013201 (PFAM); G3DSA:3.90.70.10 (GENE3D), PTHR12411:SF149 (PANTHER), SSF54001 (SUPERFAMILY) | |

| | | | | | | | | | | |
|---|---|---|---|---|---|---|---|---|---|---|
| 140 | lib_tri_Filter15_IDBA_contig_3901_plus2 | 72 | gelsolin-like protein 2-like | 151 | 3.83E-49 | 86.50% | 8 | C:cytoskeleton; F:calcium ion binding; P:biological_process; P:cytoskeleton organization; F:actin binding; P:multicellular organismal development; C:cytoplasm; P:regulation of biological process | IPR007122 (PANTHER); G3DSA:3.40.20.10 (GENE3D), PTHR11977:SF19 (PANTHER), SSF55753 (SUPERFAMILY) | |
| 141 | lib_tri_Filter15_IDBA_contig_3908_plus2 | 59 | | 210 | 0 | 100% | 3 | P:protein metabolic process; P:catabolic process; F:peptidase activity | IPR007484 (PFAM); G3DSA:3.40.630.10 (GENE3D), PTHR12283 (PANTHER), PTHR12283:SF0 (PANTHER), SSF53187 (SUPERFAMILY) | |
| 142 | lib_tri_Filter15_IDBA_contig_3927_minus1 | 62 | | 189 | 0 | 100% | 1 | F:protein binding | IPR003961 (PFAM); IPR013783 (G3DSA:2.60.40.GENE3D); PR00014 (PRINTS) | |
| 143 | lib_tri_Filter15_IDBA_contig_3928_minus2 | 34 | | 118 | 0 | 100% | 1 | F:protein binding | IPR003961 (PFAM); IPR013783 (G3DSA:2.60.40.GENE3D); PR00014 (PRINTS) | |
| 144 | lib_tri_Filter15_IDBA_contig_394_minus1 | 1020 | | 103 | 0 | 100% | 5 | P:ion transport; P:cellular homeostasis; F:binding; F:catalytic activity; P:metabolic process | IPR001519 (PANTHER); IPR009040 (PROFILE); IPR009078 (SUPERFAMILY); IPR012347 (G3DSA:1.20.1260.GENE3D) | |
| 145 | lib_tri_Filter15_IDBA_contig_400_minus2 | 4648 | 14-3-3 zeta | 1363 | 1.96E-116 | 89.25% | 22 | P:signal transduction; P:anatomical structure morphogenesis; P:cell differentiation; P:multicellular organismal development; P:reproduction; P:cytoskeleton organization; P:protein metabolic process; P:behavior; P:cell proliferation; P:metabolic process; P:regulation of biological process; F:protein binding; C:extracellular region; F:enzyme regulator activity; P:biological_process; C:cytoskeleton; C:protein complex; P:embryo development; P:cell cycle; C:nucleus; P:biosynthetic process; | IPR000308 (PRINTS); IPR023409 (PROSITE); IPR023410 (G3DSA:1.20.190.GENE3D); PTHR18860:SF0 (PANTHER), tmhmm (TMHMM) | |
| 146 | lib_tri_Filter15_IDBA_contig_4006_plus2 | 35 | perlucin 1 | 241 | 1.91E-17 | 52.15% | 1 | F:carbohydrate binding | IPR001304 (PFAM); IPR016186 (G3DSA:3.10.100.GENE3D); IPR016187 (SUPERFAMILY); IPR018378 (PROSITE); PTHR22802 (PANTHER), tmhmm (TMHMM) | |
| 147 | lib_tri_Filter15_IDBA_contig_4060_plus3 | 262 | protease inhibitor epi11 | 246 | 2.44E-29 | 44.10% | 1 | F:protein binding | IPR002350 (PFAM); G3DSA:1.10.1890.10 (GENE3D), G3DSA:3.30.60.30 (GENE3D), PTHR21312 (PANTHER), PTHR21312:SF1 (PANTHER), SignalP-NN(euk) (SIGNALP), SSF100895 (SUPERFAMILY) | Yes |
| 148 | lib_tri_Filter15_IDBA_contig_4081_minus2 | 25 | | 147 | 0 | 100% | 1 | F:protein binding | IPR003961 (PFAM); IPR013783 (G3DSA:2.60.40.GENE3D); PR00014 (PRINTS) | |
| 149 | lib_tri_Filter15_IDBA_contig_4107_minus3 | 428 | papilin-like partial | 305 | 3.01E-11 | 48.80% | 11 | P:regulation of myelination; P:thyroid hormone metabolic process; P:iodide transport; C:extracellular space; P:thyroid gland development; P:cell-matrix adhesion; F:calcium ion binding; F:peptidase inhibitor activity; F:serine-type endopeptidase inhibitor activity; C:extracellular region; P:negative regulation of peptidase activity | IPR000716 (G3DSA:4.10.800.GENE3D); PTHR12352 (PANTHER), tmhmm (TMHMM) | |
| 150 | lib_tri_Filter15_IDBA_contig_4108_minus3 | 563 | papilin-like partial | 327 | 4.72E-11 | 49.00% | 5 | P:regulation of myelination; P:thyroid hormone metabolic process; P:iodide transport; C:extracellular space; P:thyroid gland development | IPR000716 (G3DSA:4.10.800.GENE3D); PTHR12352 (PANTHER) | |

| | | | | | | | | | |
|---|---|---|---|---|---|---|---|---|---|
| 151 | lib_tri_Filter15_IDBA_contig_4109_minus3 | 579 | papilin-like partial | 327 | 4.63E-11 | 48.45% | 26 | P:regulation of myelination; P:thyroid hormone metabolic process; P:iodide transport; C:extracellular space; P:thyroid gland development; P:cell-matrix adhesion; F:calcium ion binding; C:cell surface; F:neurexin family protein binding; P:hormone biosynthetic process; C:perinuclear region of cytoplasm; C:synapse; C:endoplasmic reticulum; F:protein complex binding; P:response to lipopolysaccharide; P:response to pH; F:receptor activity; P:synapse assembly; F:hormone activity; C:Golgi apparatus; F:carboxylesterase activity; P:thyroid hormone generation; C:integral to plasma membrane; F:protein binding; P:transcytosis; | IPR000716 (G3DSA:4.10.800.GENE3D); PTHR12352 (PANTHER) |
| 152 | lib_tri_Filter15_IDBA_contig_4117_minus3 | 27 | aael005317- partial | 146 | 4.76E-22 | 50.50% | 1 | F:protein binding | IPR007110 (PROFILE); IPR013098 (PFAM); IPR013783 (G3DSA:2.60.40.GENE3D); IPR020675 (PANTHER); PTHR22964:SF3 (PANTHER), SSF48726 (SUPERFAMILY) |
| 153 | lib_tri_Filter15_IDBA_contig_4165_minus2 | 17 | isoform b | 145 | 1.62E-20 | 69.45% | 17 | P:anatomical structure morphogenesis; P:cellular component organization; P:cell differentiation; P:multicellular organismal development; P:metabolic process; P:regulation of biological process; P:cytoskeleton organization; P:biological_process; F:protein binding; C:plasma membrane; F:receptor activity; P:signal transduction; P:reproduction; P:transport; C:cytoplasm; C:endosome; | IPR015526 (PANTHER); IPR020067 (G3DSA:1.10.2000.GENE3D); PTHR11309:SF26 (PANTHER) |
| 154 | lib_tri_Filter15_IDBA_contig_4168_plus2 | 2117 | | 145 | 0 | 100% | 1 | P:metabolic process | IPR016040 (G3DSA:3.40.50.GENE3D); IPR020828 (PFAM); IPR020831 (PANTHER); SSF51735 (SUPERFAMILY) |
| 155 | lib_tri_Filter15_IDBA_contig_4180_minus2 | 24 | low quality protein: hemicentin-2 | 145 | 1.93E-08 | 46.45% | 2 | P:multicellular organismal development; F:protein binding | IPR003598 (SMART); IPR003599 (SMART); IPR007110 (PROFILE); IPR013783 (G3DSA:2.60.40.GENE3D); PTHR10489 (PANTHER), PF13895 (PFAM), SSF48726 (SUPERFAMILY) |
| 156 | lib_tri_Filter15_IDBA_contig_4185_plus2 | 306 | macrophage migration inhibitory factor | 358 | 1.27E-09 | 51.40% | 14 | P:cellular protein modification process; P:regulation of biological process; P:response to external stimulus; P:response to stress; P:multicellular organismal development; P:biological_process; P:cell death; P:transport; P:response to endogenous stimulus; C:cell; P:cell proliferation; F:catalytic activity; P:signal transduction; F:receptor binding | IPR001398 (PRODOM); IPR014347 (SUPERFAMILY); G3DSA:3.30.429.10 (GENE3D), tmhmm (TMHMM) |
| 157 | lib_tri_Filter15_IDBA_contig_419_plus2 | 73 | transmembrane protease serine 5 | 136 | 7.41E-21 | 65.00% | 3 | F:peptidase activity; P:protein metabolic process; P:catabolic process | IPR001254 (PFAM); IPR009003 (SUPERFAMILY); IPR018114 (PROSITE); G3DSA:2.40.10.10 (GENE3D), PTHR24265 (PANTHER) |

| | | | | | | | | | |
|---|---|---|---|---|---|---|---|---|---|
| 158 | lib_tri_Filter15_IDBA_contig_4195_minus2 | 55 | collagen alpha-1 chain | 246 | 7.22E-12 | 52.30% | 1 | F:protein binding | IPR002035 (G3DSA:3.40.50.GENE3D); PTHR22992 (PANTHER), SSF53300 (SUPERFAMILY) |
| 159 | lib_tri_Filter15_IDBA_contig_4196_minus2 | 62 | collagen alpha-1 chain | 267 | 6.45E-12 | 52.25% | 1 | F:protein binding | IPR002035 (G3DSA:3.40.50.GENE3D); PTHR22992 (PANTHER), SSF53300 (SUPERFAMILY) |
| 160 | lib_tri_Filter15_IDBA_contig_4205_minus2 | 33 | fibril-forming collagen alpha chain-like | 144 | 6.18E-10 | 61.33% | 30 | F:extracellular matrix structural constituent; C:collagen; C:chromosome; F:ATP binding; P:chromosome organization; P:cartilage condensation; C:basement membrane; P:cartilage development involved in endochondral bone morphogenesis; P:otic vesicle development; C:collagen type II; P:embryonic skeletal joint morphogenesis; P:sensory perception of sound; P:collagen fibril organization; C:extracellular space; P:chondrocyte differentiation; P:heart morphogenesis; P:inner ear morphogenesis; P:visual perception; P:palate development; P:proteoglycan metabolic process; P:central nervous system development; C:cytoplasm; P:limb bud formation; P:tissue homeostasis; P:cellular response to BMP stimulus; P:endochondral ossification; P:regulation of gene expression; F:platelet-derived growth factor binding; P:notochord development; P:negative regulation of apoptotic | IPR008160 (PFAM); PTHR24023 (PANTHER), PTHR24023:SF164 (PANTHER) |
| 161 | lib_tri_Filter15_IDBA_contig_4229_plus1 | 19 | cysteine-rich secretory protein 2-like | 144 | 5.24E-28 | 56.45% | 1 | C:extracellular region | IPR001283 (PRINTS); IPR002413 (PRINTS); IPR014044 (G3DSA:3.40.33.GENE3D) |
| 162 | lib_tri_Filter15_IDBA_contig_4232_plus2 | 32 | ttn partial | 144 | 7.36E-46 | 65.40% | 26 | F:cytoskeletal protein binding; F:protein kinase activity; P:anatomical structure morphogenesis; P:cellular component organization; P:embryo development; C:cytoskeleton; C:cytoplasm; P:cell differentiation; P:multicellular organismal development; P:cytoskeleton organization; P:biological_process; C:cytosol; F:structural molecule activity; P:response to external stimulus; P:response to stress; P:transport; F:protein binding; P:metabolic process; P:regulation of biological process; C:protein complex; C:nuclear chromosome; F:actin binding; F:calcium ion binding; P:organelle organization; P:cell cycle; | IPR003598 (SMART); IPR003599 (SMART); IPR007110 (PROFILE); IPR013098 (PFAM); IPR013783 (G3DSA:2.60.40.GENE3D); IPR020675 (PANTHER); SSF48726 (SUPERFAMILY) |
| 163 | lib_tri_Filter15_IDBA_contig_4238_minus1 | 221 | ---NA--- | 144 | | | 0 | - | no IPS match |

| | | | | | | | | | | |
|---|---|---|---|---|---|---|---|---|---|---|
| 164 | lib_tri_Filter15_IDBA_contig_4243_plus1 | 4191 | nicotinic acetylcholine receptor alpha1 subunit | 326 | 9.22E-19 | 47.80% | 3 | P:ion transport; C:cell; F:ion channel activity | IPR006201 (PANTHER); IPR006202 (G3DSA:2.70.170.GENE3D); PTHR18945:SF127 (PANTHER), SignalP-NN(euk) (SIGNALP), tmhmm (TMHMM) | |
| 165 | lib_tri_Filter15_IDBA_contig_4244_plus3 | 4134 | nicotinic acetylcholine receptor subunit alpha 3 | 245 | 8.19E-19 | 46.40% | 3 | P:ion transport; C:cell; F:ion channel activity | IPR006201 (PANTHER); IPR006202 (G3DSA:2.70.170.GENE3D); PTHR18945:SF127 (PANTHER) | |
| 166 | lib_tri_Filter15_IDBA_contig_4282_minus2 | 24 | titin | 143 | 6.34E-26 | 54.95% | 1 | F:protein binding | IPR003598 (SMART); IPR003599 (SMART); IPR007110 (PROFILE); IPR013098 (PFAM); IPR013783 (G3DSA:2.60.40.GENE3D); PTHR10489 (PANTHER), SSF48726 (SUPERFAMILY) | |
| 167 | lib_tri_Filter15_IDBA_contig_4399_plus2 | 11 | ectonucleotide pyrophosphatase phosphodiesterase family member 5 | 141 | 1.70E-29 | 54.70% | 3 | C:cell; F:catalytic activity; P:metabolic process | IPR002591 (PFAM); IPR017849 (G3DSA:3.40.720.GENE3D); IPR017850 (SUPERFAMILY); IPR024873 (PANTHER); PTHR10151:SF17 (PANTHER) | |
| 168 | lib_tri_Filter15_IDBA_contig_4407_minus1 | 203 | deleted in malignant brain tumors 1 | 196 | 3.30E-37 | 58.75% | 2 | F:receptor activity; C:cell | IPR001190 (PRINTS); IPR017448 (SMART); G3DSA:3.10.250.10 (GENE3D), PTHR19331 (PANTHER), PTHR19331:SF118 (PANTHER) | |
| 169 | lib_tri_Filter15_IDBA_contig_4408_minus1 | 100 | protein | 141 | 5.38E-31 | 57.50% | 2 | F:receptor activity; C:cell | IPR001190 (PRINTS); IPR017448 (SMART); G3DSA:3.10.250.10 (GENE3D), PTHR19331 (PANTHER), PTHR19331:SF118 (PANTHER) | |
| 170 | lib_tri_Filter15_IDBA_contig_4409_minus2 | 22 | protein | 119 | 1.85E-28 | 64.35% | 2 | F:receptor activity; C:cell | IPR001190 (PRINTS); IPR017448 (SMART); G3DSA:3.10.250.10 (GENE3D), PTHR19331 (PANTHER), PTHR19331:SF118 (PANTHER) | |
| 171 | lib_tri_Filter15_IDBA_contig_4436_plus2 | 281 | cysteine-rich secretory protein mr30 | 312 | 1.85E-54 | 50.85% | 6 | C:extracellular region; P:proteolysis; F:hydrolase activity; F:peptidase activity; F:ion channel inhibitor activity; F:calcium channel inhibitor activity | IPR001283 (PRINTS); IPR002413 (PRINTS); IPR014044 (G3DSA:3.40.33.GENE3D) | |
| 172 | lib_tri_Filter15_IDBA_contig_448_minus1 | 3505 | | 695 | 0 | 100% | 1 | F:structural molecule activity | IPR001664 (PANTHER); IPR016044 (PFAM); IPR016451 (PIR); IPR018039 (PROSITE); G3DSA:1.20.5.170 (GENE3D), G3DSA:2.60.40.1260 (GENE3D), SSF74853 (SUPERFAMILY) | |
| 173 | lib_tri_Filter15_IDBA_contig_4648_minus1 | 29 | glucose dehydrogenase | 137 | 5.69E-50 | 68.10% | 2 | F:nucleotide binding; P:metabolic process | IPR000172 (PFAM); G3DSA:3.50.50.60 (GENE3D), PTHR11552 (PANTHER), PTHR11552:SF10 (PANTHER), SSF51905 (SUPERFAMILY) | |
| 174 | lib_tri_Filter15_IDBA_contig_4689_plus1 | 73 | ferritin peptide | 262 | 3.54E-37 | 61.00% | 5 | P:ion transport; P:cellular homeostasis; F:binding; F:catalytic activity; P:metabolic process | IPR001519 (PANTHER); IPR008331 (PFAM); IPR009040 (PROFILE); IPR009078 (SUPERFAMILY); IPR012347 (G3DSA:1.20.1260.GENE3D); SignalP-NN(euk) (SIGNALP) | Yes |
| 175 | lib_tri_Filter15_IDBA_contig_4711_minus2 | 10 | sonic partial | 135 | 1.37E-18 | 62.75% | 3 | P:protein metabolic process; P:catabolic process; F:peptidase activity | IPR001767 (PFAM); IPR003586 (SMART); G3DSA:2.170.16.10 (GENE3D), PTHR11889 (PANTHER), tmhmm (TMHMM), SSF51294 (SUPERFAMILY) | |
| 176 | lib_tri_Filter15_IDBA_contig_4712_plus2 | 19 | deleted in malignant brain tumors 1 | 135 | 2.63E-19 | 53.10% | 2 | F:receptor activity; C:cell | IPR001190 (PRINTS); IPR017448 (SMART); G3DSA:3.10.250.10 (GENE3D), PTHR19331 (PANTHER), PTHR19331:SF118 (PANTHER) | |
| 177 | lib_tri_Filter15_IDBA_contig_4720_minus3 | 35 | | 134 | 0 | 100% | 1 | F:protein binding | IPR001478 (PFAM); G3DSA:2.30.42.10 (GENE3D), PTHR24214 (PANTHER) | |
| 178 | lib_tri_Filter15_IDBA_contig_4761_minus3 | 19 | kazal-type proteinase inhibitor | 134 | 1.35E-14 | 43.85% | 1 | F:protein binding | IPR002350 (PFAM); G3DSA:3.30.60.30 (GENE3D), PTHR10913 (PANTHER), PTHR10913:SF21 (PANTHER), SSF100895 (SUPERFAMILY) | |
| 179 | lib_tri_Filter15_IDBA_contig_479_minus1 | 137 | | 138 | 0 | 100% | 3 | F:calcium ion binding; C:proteinaceous extracellular matrix; P:signal transduction | IPR011992 (G3DSA:1.10.238.GENE3D); IPR018247 (PROSITE); IPR019577 (PFAM); PTHR13866 (PANTHER), PTHR13866:SF5 (PANTHER), SSF47473 (SUPERFAMILY) | |

| | | | | | | | | | |
|---|---|---|---|---|---|---|---|---|---|
| 180 | lib_tri_Filter15_IDBA_contig_481_minus1 | 9543 | ferritin | 393 | 7.59E-73 | 83.95% | 6 | C:cytoplasm; P:cellular homeostasis; F:binding; F:catalytic activity; P:ion transport; P:metabolic process | IPR001519 (PANTHER); IPR008331 (PFAM); IPR009040 (PROFILE); IPR009078 (SUPERFAMILY); IPR012347 (G3DSA:1.20.1260.GENE3D); IPR014034 (PROSITE); tmhmm (TMHMM) |
| 181 | lib_tri_Filter15_IDBA_contig_4959_plus1 | 11 | serpin b4-like | 131 | 1.28E-14 | 57.95% | 1 | F:enzyme regulator activity | IPR000215 (PANTHER); IPR023796 (PFAM); G3DSA:3.30.497.10 (GENE3D) |
| 182 | lib_tri_Filter15_IDBA_contig_4967_minus2 | 24 | deleted in malignant brain tumors 1 | 131 | 3.44E-28 | 60.00% | 2 | F:receptor activity; C:cell | IPR001190 (PRINTS); IPR017448 (SMART); G3DSA:3.10.250.10 (GENE3D), PTHR19331 (PANTHER), PTHR19331:SF118 (PANTHER) |
| 183 | lib_tri_Filter15_IDBA_contig_504_minus2 | 10584 | protein unc- isoform d | 2213 | 0 | 69.75% | 9 | P:cellular protein modification process; F:protein kinase activity; C:cytoplasm; P:multicellular organismal development; P:regulation of biological process; F:nucleotide binding; P:biological_process; P:growth; F:protein binding | IPR000719 (PFAM); IPR002290 (SMART); IPR003598 (SMART); IPR003599 (SMART); IPR007110 (PROFILE); IPR008271 (PROSITE); IPR011009 (SUPERFAMILY); IPR013098 (PFAM); IPR013783 (G3DSA:2.60.40.GENE3D); IPR020635 (SMART); IPR020675 (PANTHER); G3DSA:1.10.510.10 (GENE3D), G3DSA:3.30.200.20 (GENE3D), PTHR22964:SF3 (PANTHER), tmhmm (TMHMM), SSF48726 (SUPERFAMILY) |
| 184 | lib_tri_Filter15_IDBA_contig_5058_minus2 | 29 | serine carboxypeptidase-like 51 | 129 | 6.40E-14 | 60.80% | 3 | F:peptidase activity; P:protein metabolic process; P:catabolic process | IPR001563 (PANTHER); G3DSA:3.40.50.1820 (GENE3D), PTHR11802:SF3 (PANTHER), SSF53474 (SUPERFAMILY) |
| 185 | lib_tri_Filter15_IDBA_contig_5066_minus3 | 13 | fcn3-a protein | 129 | 2.11E-15 | 63.05% | 3 | F:receptor binding; C:extracellular space; P:signal transduction | IPR002181 (PFAM); IPR014715 (G3DSA:4.10.530.GENE3D); IPR020837 (PROSITE); PTHR19143 (PANTHER) |
| 186 | lib_tri_Filter15_IDBA_contig_5111_plus1 | 26 | thioredoxin-dependent peroxide reductase | 181 | 1.13E-91 | 83.50% | 3 | P:metabolic process; F:catalytic activity; F:antioxidant activity | IPR000866 (PFAM); IPR012336 (G3DSA:3.40.30.GENE3D); IPR024706 (PIR); PTHR10681 (PANTHER), PTHR10681:SF45 (PANTHER) |
| 187 | lib_tri_Filter15_IDBA_contig_5207_minus3 | 25 | lysyl oxidase like protein 2 | 127 | 1.81E-33 | 68.00% | 11 | C:cytosol; P:regulation of biological process; P:protein transport; F:protein binding; P:transport; C:organelle; C:cytoplasm; P:cell differentiation; P:biological_process; C:plasma membrane; F:receptor activity | IPR001190 (PRINTS); IPR017448 (SMART); G3DSA:3.10.250.10 (GENE3D), PTHR19331 (PANTHER) |
| 188 | lib_tri_Filter15_IDBA_contig_521_minus1 | 6979 | glyceraldehyde 3 phosphate dehydrogenase 2 | 275 | 2.42E-106 | 85.75% | 15 | P:cell death; C:cytosol; F:nucleotide binding; F:cytoskeletal protein binding; C:cytoskeleton; C:protein complex; P:metabolic process; P:regulation of biological process; P:cytoskeleton organization; F:catalytic activity; P:carbohydrate metabolic process; P:generation of precursor metabolites and energy; P:catabolic process; C:lipid particle; C:nucleus | IPR020829 (PFAM); IPR020830 (PROSITE); IPR020831 (PRINTS); G3DSA:3.30.360.10 (GENE3D), SSF55347 (SUPERFAMILY) |
| 189 | lib_tri_Filter15_IDBA_contig_5222_plus3 | 24 | ficolin 3 | 127 | 1.92E-42 | 60.10% | 3 | F:receptor binding; C:extracellular space; P:signal transduction | IPR002181 (PFAM); IPR014716 (G3DSA:3.90.215.GENE3D); PTHR19143 (PANTHER) |
| 190 | lib_tri_Filter15_IDBA_contig_5238_minus2 | 14 | suppressor of lurcher protein 1-like | 127 | 1.14E-44 | 64.20% | 0 | - | IPR000859 (G3DSA:2.60.120.GENE3D); PTHR10127 (PANTHER), PTHR10127:SF464 (PANTHER) |

| | | | | | | | | | |
|---|---|---|---|---|---|---|---|---|---|
| 191 | lib_tri_Filter15_IDBA_contig_524_minus1 | 5750 | ubiquitin c | 110 | 6.08E-71 | 100.00% | 27 | P:cell cycle; P:signal transduction; F:structural molecule activity; P:biological_process; P:response to stress; P:DNA metabolic process; P:reproduction; P:viral reproduction; P:translation; F:binding; C:nucleoplasm; P:protein metabolic process; P:catabolic process; P:regulation of biological process; P:cell death; F:protein binding; P:cellular component organization; C:plasma membrane; P:protein transport; ; C:endosome; C:cytoplasmic membrane-bounded vesicle; P:cellular protein modification process; P:nucleobase-containing compound metabolic process; C:ribosome; | IPR000626 (PFAM); IPR019954 (PROSITE); IPR019955 (PROFILE); IPR019956 (PRINTS); G3DSA:3.10.20.90 (GENE3D), PTHR10666 (PANTHER), SSF54236 (SUPERFAMILY) |
| 192 | lib_tri_Filter15_IDBA_contig_546_minus2 | 24263 | paramyosin | 1355 | 0 | 70.85% | 4 | C:cytoskeleton; C:protein complex; C:cytoplasm; F:motor activity | IPR002928 (PFAM); IPR009053 (SUPERFAMILY); G3DSA:1.20.5.340 (GENE3D), PTHR13140 (PANTHER), PTHR13140:SF106 (PANTHER), tmhmm (TMHMM) |
| 193 | lib_tri_Filter15_IDBA_contig_5518_minus1 | 27 | | 123 | 0 | 100% | 1 | F:carbohydrate binding | IPR000922 (PFAM); PTHR25100 (PANTHER), PTHR25100:SF14 (PANTHER) |
| 194 | lib_tri_Filter15_IDBA_contig_5549_minus3 | 11 | macrophage receptor marco | 122 | 6.49E-16 | 58.60% | 2 | F:receptor activity; C:cell | IPR001190 (PRINTS); IPR017448 (SMART); G3DSA:3.10.250.10 (GENE3D), PTHR19331 (PANTHER), PTHR19331:SF118 (PANTHER) |
| 195 | lib_tri_Filter15_IDBA_contig_557_minus2 | 11213 | | 597 | 0 | 100% | 1 | F:protein binding | IPR000557 (PFAM); IPR001715 (G3DSA:1.10.418.GENE3D); IPR003096 (PRINTS); PTHR18959 (PANTHER), PTHR18959:SF3 (PANTHER) |
| 196 | lib_tri_Filter15_IDBA_contig_5576_plus3 | 16 | lysyl oxidase homolog 4-like | 121 | 2.05E-24 | 64.30% | 2 | F:receptor activity; C:cell | IPR001190 (PRINTS); IPR017448 (SMART); G3DSA:3.10.250.10 (GENE3D), PTHR19331 (PANTHER), PTHR19331:SF118 (PANTHER) |
| 197 | lib_tri_Filter15_IDBA_contig_558_minus1 | 397 | | 312 | 0 | 100% | 1 | F:protein binding | IPR003599 (SMART); IPR007110 (PROFILE); IPR013098 (PFAM); IPR013783 (G3DSA:2.60.40.GENE3D); SSF48726 (SUPERFAMILY) |
| 198 | lib_tri_Filter15_IDBA_contig_559_minus1 | 15538 | myosin heavy chain | 134 | 3.53E-47 | 87.95% | 7 | C:cytoskeleton; C:protein complex; F:actin binding; F:nucleotide binding; C:cytoplasm; F:motor activity; F:protein binding | IPR002928 (PFAM); PTHR13140 (PANTHER), PTHR13140:SF106 (PANTHER) |
| 199 | lib_tri_Filter15_IDBA_contig_561_minus2 | 10693 | myosin heavy chain | 231 | 3.37E-79 | 88.25% | 7 | C:cytoskeleton; C:protein complex; F:nucleotide binding; C:cytoplasm; F:actin binding; F:motor activity; F:protein binding | IPR002928 (PFAM); PTHR13140 (PANTHER), PTHR13140:SF106 (PANTHER) |
| 200 | lib_tri_Filter15_IDBA_contig_564_minus1 | 3532 | | 192 | 0 | 100% | 3 | F:motor activity; C:cytoskeleton; C:protein complex | IPR002928 (PFAM); PTHR13140 (PANTHER), PTHR13140:SF106 (PANTHER) |
| 201 | lib_tri_Filter15_IDBA_contig_5643_plus1 | 66 | | 143 | | | | | IPR000716 (G3DSA:4.10.800.GENE3D); PTHR12352 (PANTHER) |
| 202 | lib_tri_Filter15_IDBA_contig_566_minus1 | 10634 | myosin heavy chain | 1183 | 0 | 80.15% | 7 | C:cytoskeleton; C:protein complex; F:nucleotide binding; C:cytoplasm; F:actin binding; F:motor activity; F:protein binding | IPR000048 (PFAM); IPR001609 (PRINTS); IPR004009 (PFAM); IPR009053 (SUPERFAMILY); IPR027401 (G3DSA:4.10.270.GENE3D); IPR027417 (SUPERFAMILY); G3DSA:1.20.5.340 (GENE3D), PTHR13140 (PANTHER), PTHR13140:SF106 (PANTHER) |

| | | | | | | | | | | |
|---|---|---|---|---|---|---|---|---|---|---|
| 203 | lib_tri_Filter15_IDBA_contig_569_plus3 | 1920 | | 1625 | 0 | 100% | 1 | F:protein binding | IPR003598 (SMART); IPR003599 (SMART); IPR003961 (PFAM); IPR007110 (PROFILE); IPR013098 (PFAM); IPR013783 (G3DSA:2.60.40.GENE3D); PR00014 (PRINTS), SSF48726 (SUPERFAMILY) | |
| 204 | lib_tri_Filter15_IDBA_contig_5710_plus1 | 85 | sparc | 120 | 2.09E-23 | 63.15% | 1 | F:protein binding | IPR002350 (PFAM); G3DSA:3.30.60.30 (GENE3D), PTHR13866 (PANTHER), SSF100895 (SUPERFAMILY) | |
| 205 | lib_tri_Filter15_IDBA_contig_5719_minus2 | 15 | ankyrin unc44 | 120 | 4.06E-61 | 84.10% | 7 | P:transport; C:extracellular region; P:metabolic process; P:signal transduction; F:hydrolase activity; F:lipid binding; F:protein binding | IPR002110 (PRINTS); IPR020683 (G3DSA:1.25.40.GENE3D); PTHR24123 (PANTHER), PTHR24123:SF0 (PANTHER) | |
| 206 | lib_tri_Filter15_IDBA_contig_579_minus2 | 563 | | 1430 | 0 | 100% | 1 | F:protein binding | IPR003598 (SMART); IPR003599 (SMART); IPR007110 (PROFILE); IPR013098 (PFAM); IPR013783 (G3DSA:2.60.40.GENE3D); PTHR13817 (PANTHER), SSF48726 (SUPERFAMILY) | |
| 207 | lib_tri_Filter15_IDBA_contig_582_plus2 | 1736 | myosin light chain kinase-like protein | 1522 | 0 | 58.10% | 4 | F:nucleotide binding; P:cellular protein modification process; F:protein binding; F:protein kinase activity | IPR000719 (PFAM); IPR002290 (SMART); IPR003598 (SMART); IPR003961 (PFAM); IPR007110 (PROFILE); IPR011009 (SUPERFAMILY); IPR013098 (PFAM); IPR013783 (G3DSA:2.60.40.GENE3D); IPR020635 (SMART); IPR020675 (PANTHER); G3DSA:1.10.510.10 (GENE3D), G3DSA:3.30.200.20 (GENE3D), tmhmm (TMHMM), SSF48726 (SUPERFAMILY) | |
| 208 | lib_tri_Filter15_IDBA_contig_583_plus3 | 401 | | 1190 | 0 | 100% | 1 | F:protein binding | IPR003598 (SMART); IPR003599 (SMART); IPR003961 (PFAM); IPR007110 (PROFILE); IPR013098 (PFAM); IPR013783 (G3DSA:2.60.40.GENE3D); PR00014 (PRINTS), SSF48726 (SUPERFAMILY) | |
| 209 | lib_tri_Filter15_IDBA_contig_5836_plus2 | 17 | alpha amylase | 118 | 7.47E-51 | 79.50% | 3 | F:hydrolase activity; P:carbohydrate metabolic process; F:binding | IPR006047 (PFAM); IPR013781 (G3DSA:3.20.20.GENE3D); IPR015902 (PANTHER); IPR017853 (SUPERFAMILY); PTHR10357:SF73 (PANTHER) | |
| 210 | lib_tri_Filter15_IDBA_contig_584_plus2 | 4897 | | 2107 | | | | | IPR009114 (PRINTS); IPR024646 (PFAM); tmhmm (TMHMM) | |
| 211 | lib_tri_Filter15_IDBA_contig_5928_plus3 | 23 | obscurin-like protein 1 | 117 | 1.65E-21 | 57.80% | 6 | P:anatomical structure morphogenesis; P:cell differentiation; P:multicellular organismal development; P:cytoskeleton organization; C:cytoplasm; F:protein binding | IPR007110 (PROFILE); IPR013098 (PFAM); IPR013783 (G3DSA:2.60.40.GENE3D); IPR020675 (PANTHER); PTHR22964:SF3 (PANTHER), SSF48726 (SUPERFAMILY) | |
| 212 | lib_tri_Filter15_IDBA_contig_5981_plus2 | 12 | matrix metalloproteinase-9 | 116 | 1.31E-10 | 68.55% | 15 | P:regulation of biological process; P:biological_process; P:reproduction; P:multicellular organismal development; F:binding; C:extracellular space; P:cell death; F:peptidase activity; P:protein metabolic process; P:catabolic process; F:protein binding; C:extracellular region; P:cell communication; P:metabolic process; P:cellular component organization | IPR000001 (PROFILE); IPR000562 (G3DSA:2.10.10.GENE3D); IPR013806 (SUPERFAMILY); PR00013 (PRINTS), PTHR22918 (PANTHER), PTHR22918:SF0 (PANTHER) | |
| 213 | lib_tri_Filter15_IDBA_contig_604_minus1 | 442 | calreticulin | 887 | 6.48E-158 | 84.80% | 4 | P:protein metabolic process; C:endoplasmic reticulum; F:protein binding; F:calcium ion binding | IPR001580 (PRINTS); IPR008985 (SUPERFAMILY); IPR013320 (G3DSA:2.60.120.GENE3D); IPR018124 (PROSITE); PTHR11073:SF2 (PANTHER), SignalP-NN(euk) (SIGNALP), tmhmm (TMHMM) | Yes |

| | | | | | | | | | |
|---|---|---|---|---|---|---|---|---|---|
| 214 | lib_tri_Filter15_IDBA_contig_605_plus1 | 4767 | enolase | 1125 | 0 | 85.95% | 9 | C:cytosol; C:protein complex; C:cell; F:catalytic activity; F:hydrolase activity; F:binding; P:carbohydrate metabolic process; P:generation of precursor metabolites and energy; P:catabolic process | IPR000941 (PRINTS); IPR020809 (PROSITE); IPR020810 (PFAM); IPR020811 (PFAM); G3DSA:3.20.20.120 (GENE3D), G3DSA:3.30.390.10 (GENE3D), tmhmm (TMHMM), SSF51604 (SUPERFAMILY), SSF54826 (SUPERFAMILY) |
| 215 | lib_tri_Filter15_IDBA_contig_6090_plus2 | 31 | cre-ttn-1 protein | 132 | 6.47E-20 | 51.75% | 3 | P:biological_process; F:transferase activity; F:protein binding | IPR003598 (SMART); IPR003599 (SMART); IPR007110 (PROFILE); IPR013098 (PFAM); IPR013783 (G3DSA:2.60.40.GENE3D); PTHR10489 (PANTHER), PF13895 (PFAM), SSF48726 (SUPERFAMILY) |
| 216 | lib_tri_Filter15_IDBA_contig_612_plus1 | 281 | fibronectin type iii domain protein | 818 | 8.65E-142 | 50.65% | 3 | P:biological_process; F:transferase activity; F:protein binding | IPR003596 (SMART); IPR003598 (SMART); IPR003599 (SMART); IPR003961 (PFAM); IPR007110 (PROFILE); IPR013098 (PFAM); IPR013783 (G3DSA:2.60.40.GENE3D); SSF48726 (SUPERFAMILY) |
| 217 | lib_tri_Filter15_IDBA_contig_6120_minus2 | 98 | galectin-8-like isoform 1 | 115 | 5.09E-16 | 66.65% | 2 | C:extracellular space; F:carbohydrate binding | IPR001079 (PFAM); IPR008985 (SUPERFAMILY); IPR013320 (G3DSA:2.60.120.GENE3D); PTHR11346 (PANTHER) |
| 218 | lib_tri_Filter15_IDBA_contig_6144_plus2 | 22 | serine-threonine kinase receptor-associated | 115 | 2.18E-46 | 74.65% | 2 | F:kinase activity; F:protein binding | IPR001680 (PFAM); IPR015943 (G3DSA:2.130.10.GENE3D); IPR017986 (PROFILE); IPR019775 (PROSITE); PTHR19877 (PANTHER), PTHR19877:SF2 (PANTHER), SSF50960 (SUPERFAMILY) |
| 219 | lib_tri_Filter15_IDBA_contig_6176_plus2 | 18 | beta-ig-h3 fasciclin | 114 | 1.20E-14 | 53.10% | 0 | - | IPR000782 (G3DSA:2.30.180.GENE3D); PTHR10900 (PANTHER) |
| 220 | lib_tri_Filter15_IDBA_contig_6228_minus3 | 31 | epidermal growth factor receptor | 156 | 5.26E-14 | 53.00% | 6 | C:cell; F:protein kinase activity; F:receptor activity; F:nucleotide binding; P:cellular protein modification process; P:signal transduction | IPR000494 (G3DSA:3.80.20.GENE3D); IPR006211 (PFAM); IPR006212 (SMART); IPR009030 (SUPERFAMILY); G3DSA:2.10.220.10 (GENE3D), PTHR24416 (PANTHER), PTHR24416:SF95 (PANTHER), SSF52058 (SUPERFAMILY) |
| 221 | lib_tri_Filter15_IDBA_contig_6289_minus1 | 15 | | 113 | 0 | 100% | 1 | F:carbohydrate binding | IPR001304 (PFAM); IPR016186 (G3DSA:3.10.100.GENE3D); IPR016187 (SUPERFAMILY); SignalP-NN(euk) (SIGNALP) |
| 222 | lib_tri_Filter15_IDBA_contig_6305_minus1 | 32 | prostasin isoform 2 | 156 | 7.13E-18 | 61.90% | 6 | C:plasma membrane; F:protein binding; C:extracellular region; F:peptidase activity; P:protein metabolic process; P:catabolic process | IPR001254 (PFAM); IPR009003 (SUPERFAMILY); G3DSA:2.40.10.10 (GENE3D), PTHR24264 (PANTHER) |
| 223 | lib_tri_Filter15_IDBA_contig_631_minus1 | 1780 | | 1056 | 0 | 100% | 4 | F:nucleotide binding; P:signal transduction; C:intracellular; P:protein transport | IPR003579 (SMART); IPR005225 (TIGRFAMs); IPR006687 (SMART); IPR006689 (PRINTS); IPR024156 (SMART); IPR027417 (SUPERFAMILY); G3DSA:3.40.50.300 (GENE3D), PTHR11711 (PANTHER), tmhmm (TMHMM) |
| 224 | lib_tri_Filter15_IDBA_contig_634_plus1 | 514 | | 968 | 0 | 100% | 1 | F:protein binding | IPR003598 (SMART); IPR003599 (SMART); IPR007110 (PROFILE); IPR013098 (PFAM); IPR013783 (G3DSA:2.60.40.GENE3D); IPR020675 (PANTHER); SSF48726 (SUPERFAMILY) |
| 225 | lib_tri_Filter15_IDBA_contig_636_minus1 | 768 | endothelin-converting enzyme 2 | 822 | 3.79E-18 | 41.50% | 3 | F:peptidase activity; P:protein metabolic process; P:catabolic process | IPR000718 (PANTHER); IPR008753 (PFAM); IPR024079 (G3DSA:3.40.390.GENE3D); SSF55486 (SUPERFAMILY) |
| 226 | lib_tri_Filter15_IDBA_contig_6362_minus1 | 10 | leukocyte elastase inhibitor-like | 112 | 2.52E-23 | 65.25% | 3 | P:biological_process; F:binding; F:enzyme regulator activity | IPR000215 (PANTHER); IPR023796 (PFAM); G3DSA:2.30.39.10 (GENE3D), G3DSA:3.30.497.10 (GENE3D) |
| 227 | lib_tri_Filter15_IDBA_contig_638_minus2 | 302 | hemocyte protein-glutamine gamma-glutamyltransferase-like | 754 | 8.10E-126 | 59.50% | 2 | F:transferase activity; P:cellular protein modification process | IPR002931 (G3DSA:3.90.260.GENE3D); IPR008958 (PFAM); IPR013783 (G3DSA:2.60.40.GENE3D); IPR023608 (PANTHER); SSF54001 (SUPERFAMILY) |

| | | | | | | | | | |
|---|---|---|---|---|---|---|---|---|---|
| 228 | lib_tri_Filter15_IDBA_contig_6386_minus3 | 44 | protein unc- isoform e | 220 | 1.13E-21 | 46.05% | 4 | C:intracellular; P:biological_process; F:catalytic activity; F:protein binding | IPR003598 (SMART); IPR003599 (SMART); IPR007110 (PROFILE); IPR013098 (PFAM); IPR013783 (G3DSA:2.60.40.GENE3D); PTHR10489 (PANTHER), SSF48726 (SUPERFAMILY) |
| 229 | lib_tri_Filter15_IDBA_contig_6405_minus1 | 15 | ---NA--- | 112 | | | 0 | - | PTHR24023 (PANTHER), PTHR24023:SF164 (PANTHER) |
| 230 | lib_tri_Filter15_IDBA_contig_6405_plus3 | 15 | | 111 | | | | | SignalP-NN(euk) (SIGNALP) |
| 231 | lib_tri_Filter15_IDBA_contig_6424_plus1 | 11 | ---NA--- | 112 | | | 0 | - | SignalP-NN(euk) (SIGNALP) |
| 232 | lib_tri_Filter15_IDBA_contig_6444_minus1 | 16 | | 111 | 0 | 100% | 1 | F:protein binding | IPR003961 (PFAM); IPR013783 (G3DSA:2.60.40.GENE3D); PTHR10489 (PANTHER) |
| 233 | lib_tri_Filter15_IDBA_contig_652_minus2 | 247 | | 699 | 0 | 100% | 1 | F:protein binding | IPR003598 (SMART); IPR003599 (SMART); IPR007110 (PROFILE); IPR013098 (PFAM); IPR013783 (G3DSA:2.60.40.GENE3D); SSF48726 (SUPERFAMILY) |
| 234 | lib_tri_Filter15_IDBA_contig_654_plus2 | 238 | nexilin isoform 1 | 692 | 2.59E-29 | 59.80% | 4 | P:regulation of biological process; F:structural molecule activity; P:cytoskeleton organization; F:actin binding | IPR003598 (SMART); IPR003599 (SMART); IPR007110 (PROFILE); IPR013098 (PFAM); IPR013783 (G3DSA:2.60.40.GENE3D); PTHR25963 (PANTHER), SSF48726 (SUPERFAMILY) |
| 235 | lib_tri_Filter15_IDBA_contig_662_minus2 | 376 | neuronal acetylcholine receptor subunit alpha- partial | 669 | 7.06E-79 | 54.10% | 10 | P:biological_process; C:cell; P:behavior; P:signal transduction; P:cell-cell signaling; P:multicellular organismal development; F:receptor activity; C:plasma membrane; F:ion channel activity; P:ion transport | IPR002394 (PRINTS); IPR006029 (SUPERFAMILY); IPR006201 (PRINTS); IPR006202 (G3DSA:2.70.170.GENE3D); IPR018000 (PROSITE); IPR027361 (G3DSA:1.20.120.GENE3D); tmhmm (TMHMM) |
| 236 | lib_tri_Filter15_IDBA_contig_6634_minus2 | 16 | disulfide isomerase | 109 | 2.20E-44 | 80.50% | 8 | F:transferase activity; F:electron carrier activity; P:cellular homeostasis; P:regulation of biological process; P:metabolic process; F:catalytic activity; C:endoplasmic reticulum; F:nucleotide binding | IPR005746 (PRINTS); IPR005788 (TIGRFAMs); IPR008254 (PROFILE); IPR012336 (G3DSA:3.40.30.GENE3D); IPR013766 (PFAM); IPR017937 (PROSITE); PTHR18929 (PANTHER), PTHR18929:SF32 (PANTHER) |
| 237 | lib_tri_Filter15_IDBA_contig_6638_minus3 | 10 | | 108 | | | | | IPR000118 (PFAM); PTHR12274 (PANTHER), PTHR12274:SF0 (PANTHER), SSF57277 (SUPERFAMILY) |
| 238 | lib_tri_Filter15_IDBA_contig_667_plus1 | 202 | protein unc- isoform c | 669 | 0 | 61.40% | 7 | F:protein kinase activity; C:cytoplasm; P:multicellular organismal development; P:regulation of biological process; P:biological_process; P:growth; F:protein binding | IPR003599 (SMART); IPR003961 (PFAM); IPR007110 (PROFILE); IPR013098 (PFAM); IPR013783 (G3DSA:2.60.40.GENE3D); PR00014 (PRINTS), SSF48726 (SUPERFAMILY) |
| 239 | lib_tri_Filter15_IDBA_contig_6697_minus1 | 11 | deleted in malignant brain tumors 1 | 108 | 7.93E-11 | 60.05% | 2 | F:receptor activity; C:cell | IPR001190 (PFAM); IPR017448 (SMART); G3DSA:3.10.250.10 (GENE3D), PTHR19331 (PANTHER), PTHR19331:SF118 (PANTHER), SignalP-NN(euk) (SIGNALP) |
| 240 | lib_tri_Filter15_IDBA_contig_673_minus1 | 293 | protein | 660 | 1.80E-43 | 39.65% | 1 | F:protein binding | IPR002350 (PFAM); G3DSA:1.10.1890.10 (GENE3D), G3DSA:3.30.60.30 (GENE3D), PTHR10913 (PANTHER), PTHR10913:SF21 (PANTHER), SSF100895 (SUPERFAMILY) |
| 241 | lib_tri_Filter15_IDBA_contig_6740_plus1 | 10 | neuronal acetylcholine receptor subunit alpha-7-like | 108 | 1.44E-24 | 64.25% | 3 | F:ion channel activity; P:ion transport; C:cell | IPR002394 (PRINTS); IPR006201 (PANTHER); IPR006202 (G3DSA:2.70.170.GENE3D); IPR018000 (PROSITE); PTHR18945:SF92 (PANTHER) |
| 242 | lib_tri_Filter15_IDBA_contig_6757_plus2 | 21 | | 107 | 0 | 100% | 2 | F:catalytic activity; P:lipid metabolic process | IPR000734 (PANTHER); IPR013818 (PFAM); G3DSA:3.40.50.1820 (GENE3D), SSF53474 (SUPERFAMILY) |

| | | | | | | | | | |
|---|---|---|---|---|---|---|---|---|---|
| 243 | lib_tri_Filter15_IDBA_contig_6773_plus1 | 58 | disintegrin and metalloproteinase domain-containing protein 23 | 147 | 3.75E-05 | 55.55% | 13 | P:proteolysis; F:zinc ion binding; F:metalloendopeptidase activity; C:integral to membrane; P:spermatogenesis; F:metallopeptidase activity; C:extracellular region; F:integrin binding; F:protein binding; C:integral to plasma membrane; C:plasma membrane; P:central nervous system development; P:cell adhesion | PTHR11905 (PANTHER) |
| 244 | lib_tri_Filter15_IDBA_contig_68_minus1 | 1847 | peptidyl-prolyl cis-trans isomerase | 923 | 7.22E-81 | 79.05% | 8 | C:extracellular space; P:protein metabolic process; C:cytoskeleton; C:protein complex; P:reproduction; C:lipid particle; P:cellular protein modification process; F:catalytic activity | IPR002130 (PRINTS); IPR020892 (PROSITE); G3DSA:2.40.100.10 (GENE3D), PTHR11071 (PANTHER), tmhmm (TMHMM) |
| 245 | lib_tri_Filter15_IDBA_contig_6828_plus2 | 27 | ---NA--- | 107 | | | 0 | - | IPR008160 (PFAM); PTHR24023 (PANTHER) |
| 246 | lib_tri_Filter15_IDBA_contig_6834_minus1 | 7 | thioester-containing protein | 107 | 1.25E-20 | 62.80% | 1 | F:enzyme regulator activity | IPR002890 (PFAM); PTHR11412 (PANTHER) |
| 247 | lib_tri_Filter15_IDBA_contig_6870_minus3 | 9 | thrombospondin-3 isoform 3 | 106 | 1.93E-55 | 86.45% | 16 | F:receptor binding; P:cell proliferation; P:regulation of biological process; P:anatomical structure morphogenesis; P:multicellular organismal development; C:proteinaceous extracellular matrix; P:biological_process; P:cell differentiation; P:behavior; P:response to external stimulus; C:extracellular space; F:calcium ion binding; F:carbohydrate binding; P:cellular protein modification process; P:growth; C:cytoplasm | IPR008859 (PFAM); IPR008985 (SUPERFAMILY); IPR013320 (G3DSA:2.60.120.GENE3D); PTHR10199 (PANTHER) |
| 248 | lib_tri_Filter15_IDBA_contig_6917_minus3 | 21 | superoxide dismutase extracellular | 105 | 8.49E-14 | 57.45% | 5 | C:cytoplasm; F:binding; F:antioxidant activity; F:catalytic activity; P:metabolic process | IPR001424 (G3DSA:2.60.40.GENE3D); IPR024134 (PANTHER) |
| 249 | lib_tri_Filter15_IDBA_contig_694_minus3 | 2182 | | 873 | 0 | 100% | 4 | F:kinase activity; P:carbohydrate metabolic process; P:generation of precursor metabolites and energy; P:catabolic process | IPR001576 (PRINTS); IPR015824 (G3DSA:3.40.50.GENE3D); IPR015901 (G3DSA:3.40.50.GENE3D); IPR015911 (PROSITE) |
| 250 | lib_tri_Filter15_IDBA_contig_6988_minus1 | 14 | low quality protein: hemicentin-2 | 105 | 3.12E-13 | 52.05% | 5 | P:anatomical structure morphogenesis; P:cellular component organization; P:metabolic process; P:organelle organization; P:primary metabolic process | IPR013098 (PFAM); IPR013783 (G3DSA:2.60.40.GENE3D); PTHR10489 (PANTHER), SSF48726 (SUPERFAMILY) |
| 251 | lib_tri_Filter15_IDBA_contig_7017_plus2 | 20 | ef hand family protein | 125 | 1.05E-21 | 60.70% | 3 | F:protein binding; C:endoplasmic reticulum; F:calcium ion binding | IPR002048 (PROFILE); IPR011992 (G3DSA:1.10.238.GENE3D); IPR018247 (PROSITE); IPR027241 (PTHR10827:PANTHER); PTHR10827 (PANTHER), SSF47473 (SUPERFAMILY) |
| 252 | lib_tri_Filter15_IDBA_contig_7081_minus3 | 42 | macrophage receptor marco | 163 | 2.85E-29 | 58.50% | 2 | F:receptor activity; C:cell | IPR001190 (PRINTS); IPR017448 (SMART); G3DSA:3.10.250.10 (GENE3D), PTHR19331 (PANTHER), PTHR19331:SF118 (PANTHER) |
| 253 | lib_tri_Filter15_IDBA_contig_7154_plus2 | 12 | | 104 | 0 | 100% | 3 | F:peptidase activity; P:protein metabolic process; P:catabolic process | IPR000718 (PANTHER); SignalP-NN(euk) (SIGNALP), tmhmm (TMHMM), SSF55486 (SUPERFAMILY) |

| | | | | | | | | | | |
|---|---|---|---|---|---|---|---|---|---|---|
| 254 | lib_tri_Filter15_IDBA_contig_7191_minus2 | 6 | speract scavenger transmembrane glycoprotein | 103 | 3.08E-11 | 67.00% | 2 | C:cell; F:receptor activity | IPR001190 (PFAM); IPR017448 (SUPERFAMILY); G3DSA:3.10.250.10 (GENE3D), PTHR19331 (PANTHER), PTHR19331:SF118 (PANTHER), SignalP-NN(euk) (SIGNALP) | |
| 255 | lib_tri_Filter15_IDBA_contig_720_plus1 | 107 | neuropeptide y | 581 | 3.14E-07 | 72.50% | 2 | F:receptor binding; C:extracellular region | IPR001955 (PFAM); IPR020392 (PROSITE); G3DSA:1.20.5.570 (GENE3D), SignalP-NN(euk) (SIGNALP), tmhmm (TMHMM) | Yes |
| 256 | lib_tri_Filter15_IDBA_contig_7380_plus1 | 12 | angiotensin-converting enzyme | 102 | 3.94E-27 | 63.35% | 15 | F:peptidase activity; P:biological_process; P:catabolic process; F:binding; P:multicellular organismal development; C:cell; F:receptor binding; P:regulation of biological process; P:transport; C:extracellular space; F:actin binding; C:plasma membrane; P:cell differentiation; C:endosome; P:protein metabolic process | IPR001548 (PRINTS); SSF55486 (SUPERFAMILY) | |
| 257 | lib_tri_Filter15_IDBA_contig_742_minus3 | 195 | isoform a | 580 | 3.73E-28 | 47.30% | 1 | F:protein binding | IPR000742 (SMART); IPR009030 (SUPERFAMILY); IPR013032 (PFAM); G3DSA:2.10.25.10 (GENE3D), PTHR24035 (PANTHER), PTHR24035:SF4 (PANTHER) | |
| 258 | lib_tri_Filter15_IDBA_contig_7467_plus2 | 26 | cystatin c | 101 | 6.33E-10 | 60.35% | 1 | F:enzyme regulator activity | IPR000010 (PFAM); IPR018073 (PROSITE); IPR027214 (PANTHER); G3DSA:3.10.450.10 (GENE3D), PTHR11413:SF23 (PANTHER), SSF54403 (SUPERFAMILY) | |
| 259 | lib_tri_Filter15_IDBA_contig_7489_plus3 | 10 | ankyrin unc44 | 100 | 6.31E-36 | 67.30% | 11 | P:anatomical structure morphogenesis; P:cellular component organization; P:cell differentiation; P:multicellular organismal development; C:cell; P:cytoskeleton organization; C:cellular_component; P:signal transduction; P:cellular homeostasis; C:plasma membrane; F:protein binding | IPR002110 (SMART); IPR020683 (G3DSA:1.25.40.GENE3D); PTHR24123 (PANTHER), PTHR24123:SF0 (PANTHER) | |
| 260 | lib_tri_Filter15_IDBA_contig_7497_minus2 | 14 | endothelin-converting enzyme 1-like | 101 | 1.09E-10 | 56.60% | 3 | F:peptidase activity; P:protein metabolic process; P:catabolic process | IPR000718 (PANTHER); IPR008753 (PFAM); IPR024079 (G3DSA:3.40.390.GENE3D); SSF55486 (SUPERFAMILY) | |
| 261 | lib_tri_Filter15_IDBA_contig_7540_minus3 | 16 | retinoid-inducible serine carboxypeptidase isoform 2 | 100 | 3.78E-22 | 64.35% | 6 | F:peptidase activity; P:lipid metabolic process; P:secondary metabolic process; C:cytosol; P:protein metabolic process; P:catabolic process | IPR001563 (PANTHER); G3DSA:3.40.50.1820 (GENE3D), PTHR11802:SF3 (PANTHER), SSF53474 (SUPERFAMILY) | |
| 262 | lib_tri_Filter15_IDBA_contig_7567_plus3 | 6 | bone morphogenic protein 1 | 100 | 8.50E-22 | 61.70% | 4 | F:peptidase activity; P:protein metabolic process; P:catabolic process; F:binding | IPR001506 (PRINTS); IPR006026 (SMART); IPR024079 (G3DSA:3.40.390.GENE3D); PTHR10127 (PANTHER), SSF55486 (SUPERFAMILY) | |
| 263 | lib_tri_Filter15_IDBA_contig_7573_minus1 | 62 | trypsin- partial | 137 | 4.58E-26 | 67.90% | 3 | F:peptidase activity; P:protein metabolic process; P:catabolic process | IPR001254 (PFAM); IPR009003 (SUPERFAMILY); IPR018114 (PROSITE); G3DSA:2.40.10.10 (GENE3D), PTHR24264 (PANTHER) | |
| 264 | lib_tri_Filter15_IDBA_contig_7649_plus1 | 30 | deleted in malignant brain tumors 1 | 103 | 5.19E-12 | 54.10% | 2 | F:receptor activity; C:cell | IPR001190 (PRINTS); IPR017448 (SMART); G3DSA:3.10.250.10 (GENE3D), PTHR19331 (PANTHER), PTHR19331:SF118 (PANTHER) | |
| 265 | lib_tri_Filter15_IDBA_contig_7681_minus1 | 41 | tail fiber partial | 116 | 1.49E-09 | 53.30% | 1 | C:extracellular region | IPR008160 (PFAM); PTHR24023 (PANTHER) | |
| 266 | lib_tri_Filter15_IDBA_contig_78_minus2 | 858 | ---NA--- | 494 | | | 0 | - | IPR008160 (PFAM); PTHR24023 (PANTHER), PTHR24023:SF140 (PANTHER), tmhmm (TMHMM) | |
| 267 | lib_tri_Filter15_IDBA_contig_78_plus3 | 858 | | 493 | | | | | SignalP-NN(euk) (SIGNALP), tmhmm (TMHMM) | |

| | | | | | | | | | | |
|---|---|---|---|---|---|---|---|---|---|---|
| 268 | lib_tri_Filter15_IDBA_contig_7810_plus1 | 50 | protein | 138 | 1.84E-13 | 53.75% | 1 | F:protein binding | IPR002350 (PFAM); G3DSA:1.10.1890.10 (GENE3D), G3DSA:3.30.60.30 (GENE3D), PTHR10913 (PANTHER), SSF100895 (SUPERFAMILY) | |
| 269 | lib_tri_Filter15_IDBA_contig_7883_plus3 | 24 | | 125 | 0 | 100% | 1 | F:protein binding | IPR003961 (PFAM); IPR013783 (G3DSA:2.60.40.GENE3D) | |
| 270 | lib_tri_Filter15_IDBA_contig_790_plus3 | 116 | | 548 | | | | | IPR009053 (SUPERFAMILY); PTHR18937 (PANTHER), PTHR18937:SF52 (PANTHER) | |
| 271 | lib_tri_Filter15_IDBA_contig_7903_plus2 | 28 | deleted in malignant brain tumors 1 | 121 | 5.96E-15 | 66.85% | 2 | F:receptor activity; C:cell | IPR001190 (PRINTS); IPR017448 (SMART); G3DSA:3.10.250.10 (GENE3D), PTHR19331 (PANTHER) | |
| 272 | lib_tri_Filter15_IDBA_contig_7914_minus3 | 201 | | 169 | 0 | 100% | 1 | F:transferase activity | IPR011004 (SUPERFAMILY); G3DSA:2.160.10.10 (GENE3D) | |
| 273 | lib_tri_Filter15_IDBA_contig_7956_plus3 | 26 | transmembrane serine protease 8 | 101 | 1.09E-12 | 70.30% | 13 | F:protein binding; P:protein metabolic process; F:peptidase activity; P:biological_process; C:extracellular region; C:cytoplasm; C:cell; P:cell communication; P:regulation of biological process; P:catabolic process; P:response to external stimulus; P:response to stress; P:cellular protein modification process | IPR001254 (PFAM); IPR009003 (SUPERFAMILY); IPR018114 (PROSITE); G3DSA:2.40.10.10 (GENE3D), PTHR24265 (PANTHER) | |
| 274 | lib_tri_Filter15_IDBA_contig_808_minus1 | 221 | egf-like domain-containing protein | 623 | 6.38E-16 | 49.90% | 2 | F:protein binding; F:calcium ion binding | IPR000742 (SMART); IPR001881 (PFAM); IPR013032 (PROSITE); IPR018097 (PROSITE); G3DSA:2.10.25.10 (GENE3D), PTHR24035 (PANTHER), PTHR24035:SF19 (PANTHER), tmhmm (TMHMM), SSF57196 (SUPERFAMILY) | |
| 275 | lib_tri_Filter15_IDBA_contig_813_plus1 | 306 | retinoid-inducible serine carboxypeptidase | 593 | 3.90E-69 | 53.10% | 3 | F:peptidase activity; P:protein metabolic process; P:catabolic process | IPR001563 (PRINTS); G3DSA:3.40.50.1820 (GENE3D), PTHR11802:SF3 (PANTHER), SSF53474 (SUPERFAMILY) | |
| 276 | lib_tri_Filter15_IDBA_contig_816_minus3 | 1407 | pdz domain protein | 1240 | 6.92E-32 | 57.70% | 1 | F:protein binding | IPR001478 (PFAM); IPR006643 (SMART); G3DSA:2.30.42.10 (GENE3D), PTHR24214 (PANTHER), PTHR24214:SF8 (PANTHER) | |
| 277 | lib_tri_Filter15_IDBA_contig_818_minus1 | 2724 | | 889 | | | | | IPR013126 (PRINTS); IPR018181 (PROSITE); G3DSA:1.20.1270.10 (GENE3D), G3DSA:2.60.34.10 (GENE3D), G3DSA:3.30.30.30 (GENE3D), G3DSA:3.30.420.40 (GENE3D), G3DSA:3.90.640.10 (GENE3D), PTHR19375 (PANTHER), SSF100920 (SUPERFAMILY), SSF100934 (SUPERFAMILY), SSF53067 (SUPERFAMILY) | |
| 278 | lib_tri_Filter15_IDBA_contig_826_plus1 | 120 | nadh-ubiquinone oxidoreductase 75 kda mitochondrial-like | 507 | 0 | 82.10% | 10 | P:nucleobase-containing compound metabolic process; F:binding; F:catalytic activity; C:protein complex; C:mitochondrion; P:cell death; F:electron carrier activity; P:cellular homeostasis; P:generation of precursor metabolites and energy; F:protein binding | IPR006656 (PFAM); IPR015405 (PFAM); G3DSA:3.40.50.740 (GENE3D), PTHR11615 (PANTHER), PTHR11615:SF10 (PANTHER), SSF53706 (SUPERFAMILY) | |
| 279 | lib_tri_Filter15_IDBA_contig_841_minus2 | 162 | acyl- -binding protein | 498 | 1.38E-34 | 80.55% | 1 | F:lipid binding | IPR000582 (PRINTS); IPR014352 (G3DSA:1.20.80.GENE3D); PTHR23310 (PANTHER), tmhmm (TMHMM) | |
| 280 | lib_tri_Filter15_IDBA_contig_849_plus3 | 700 | thioredoxin peroxidase | 616 | 9.62E-119 | 87.95% | 3 | P:metabolic process; F:catalytic activity; F:antioxidant activity | IPR000866 (PFAM); IPR012336 (G3DSA:3.40.30.GENE3D); IPR019479 (PFAM); PTHR10681 (PANTHER), PTHR10681:SF44 (PANTHER), tmhmm (TMHMM) | |
| 281 | lib_tri_Filter15_IDBA_contig_853_minus3 | 397 | superoxide dismutase | 541 | 9.65E-75 | 79.45% | 4 | F:binding; C:cytoplasm; P:metabolic process; F:catalytic activity | IPR001424 (PRINTS); IPR018152 (PROSITE); IPR024134 (PANTHER); SignalP-NN(euk) (SIGNALP) | Yes |

| | | | | | | | | | | |
|---|---|---|---|---|---|---|---|---|---|---|
| 282 | lib_tri_Filter15_IDBA_contig_855_minus2 | 2471 | gelsolin-like protein 2-like | 794 | 8.33E-112 | 67.75% | 6 | F:calcium ion binding; P:biological_process; P:cytoskeleton organization; F:actin binding; C:intracellular; P:regulation of biological process | IPR007122 (PRINTS); IPR007123 (PFAM); G3DSA:3.40.20.10 (GENE3D), PTHR11977:SF19 (PANTHER), tmhmm (TMHMM), SSF55753 (SUPERFAMILY) | |
| 283 | lib_tri_Filter15_IDBA_contig_886_plus1 | 418 | | 738 | | | | | no IPS match | |
| 284 | lib_tri_Filter15_IDBA_contig_889_plus1 | 1090 | na subunit | 657 | 1.90E-141 | 68.30% | 3 | F:ion channel activity; P:ion transport; C:cell | IPR002394 (PRINTS); IPR006029 (PFAM); IPR006201 (PRINTS); IPR006202 (G3DSA:2.70.170.GENE3D); IPR018000 (PROSITE); IPR027361 (G3DSA:1.20.120.GENE3D); PTHR18945:SF92 (PANTHER), SignalP-NN(euk) (SIGNALP), tmhmm (TMHMM) | |
| 285 | lib_tri_Filter15_IDBA_contig_895_plus2 | 92 | protein disulfide isomerase | 444 | 7.28E-52 | 86.40% | 15 | P:metabolic process; P:lipid metabolic process; F:catalytic activity; C:cytoplasm; P:cellular homeostasis; P:regulation of biological process; F:electron carrier activity; C:cell; C:cytoplasmic membrane-bounded vesicle; C:endoplasmic reticulum; P:cellular protein modification process; ; F:protein binding; C:extracellular region; C:plasma membrane | IPR005746 (PRINTS); IPR005788 (TIGRFAMs); IPR012336 (G3DSA:3.40.30.GENE3D); IPR013766 (PFAM); IPR017937 (PROSITE); PTHR18929 (PANTHER), PTHR18929:SF32 (PANTHER), tmhmm (TMHMM) | |
| 286 | lib_tri_Filter15_IDBA_contig_896_minus2 | 1035 | kielin chordin-like protein | 517 | 3.85E-34 | 38.00% | 2 | F:protein binding; F:enzyme regulator activity | IPR001007 (PFAM); IPR006552 (SMART); IPR008037 (PFAM); G3DSA:2.10.70.10 (GENE3D), PTHR11339 (PANTHER), PTHR11339:SF46 (PANTHER), SSF57603 (SUPERFAMILY) | |
| 287 | lib_tri_Filter15_IDBA_contig_922_minus3 | 672 | retinal dehydrogenase 2 | 714 | 4.10E-176 | 83.45% | 20 | P:anatomical structure morphogenesis; P:multicellular organismal development; P:metabolic process; P:regulation of biological process; F:catalytic activity; P:embryo development; P:signal transduction; P:cell differentiation; P:lipid metabolic process; P:secondary metabolic process; P:biological_process; C:cytosol; P:cell proliferation; P:response to external stimulus; P:cell communication; P:biosynthetic process; F:lipid binding; P:cell death; C:nucleus; P:response to endogenous stimulus | IPR015590 (PFAM); IPR016160 (PROSITE); IPR016161 (SUPERFAMILY); IPR016162 (G3DSA:3.40.605.GENE3D); IPR016163 (G3DSA:3.40.309.GENE3D); PTHR11699 (PANTHER), PTHR11699:SF46 (PANTHER), SignalP-NN(euk) (SIGNALP) | Yes |
| 288 | lib_tri_Filter15_IDBA_contig_926_minus2 | 797 | | 825 | 0 | 100% | 1 | F:protein binding | IPR003599 (SMART); IPR003961 (PFAM); IPR007110 (PROFILE); IPR013098 (PFAM); IPR013783 (G3DSA:2.60.40.GENE3D); PR00014 (PRINTS), SSF48726 (SUPERFAMILY) | |
| 289 | lib_tri_Filter15_IDBA_contig_93_minus1 | 8488 | cyclophilin a | 295 | 3.52E-99 | 89.60% | 13 | C:cytosol; F:binding; C:cytoskeleton; C:protein complex; P:cellular protein modification process; P:protein metabolic process; F:enzyme regulator activity; P:anatomical structure morphogenesis; P:multicellular organismal development; P:cell death; C:nucleus; F:catalytic activity; C:nucleoplasm | IPR002130 (PRINTS); IPR020892 (PROSITE); G3DSA:2.40.100.10 (GENE3D), PTHR11071 (PANTHER), SignalP-NN(euk) (SIGNALP) | |

| | | | | | | | | | | |
|---|---|---|---|---|---|---|---|---|---|---|
| 290 | lib_tri_Filter15_IDBA_contig_964_plus3 | 414 | | 426 | 0 | 100% | 2 | F:catalytic activity; P:lipid metabolic process | IPR000734 (PRINTS); IPR013818 (PFAM); G3DSA:3.40.50.1820 (GENE3D), SignalP-NN(euk) (SIGNALP), SSF53474 (SUPERFAMILY) | Yes |
| 291 | lib_tri_Filter15_IDBA_contig_967_plus2 | 94 | map kinase-interacting serine threonine-protein kinase 1 | 402 | 0 | 75.70% | 3 | F:nucleotide binding; P:cellular protein modification process; F:protein kinase activity | IPR000719 (PFAM); IPR002290 (SMART); IPR008271 (PROSITE); IPR011009 (SUPERFAMILY); IPR017441 (PROSITE); IPR020635 (SMART); G3DSA:1.10.510.10 (GENE3D), G3DSA:3.30.200.20 (GENE3D), PTHR24349 (PANTHER), PTHR24349:SF0 (PANTHER), tmhmm (TMHMM) | |
| 292 | lib_tri_Filter15_IDBA_contig_972_minus3 | 141 | | 485 | 0 | 100% | 2 | C:extracellular region; F:enzyme regulator activity | IPR008197 (PROFILE); PTHR14308 (PANTHER), tmhmm (TMHMM) | |
| 293 | lib_tri_Filter15_IDBA_contig_984_plus1 | 94 | | 430 | 0 | 100% | 1 | F:protein binding | IPR002110 (PRINTS); IPR020683 (G3DSA:1.25.40.GENE3D); PTHR24168 (PANTHER), PF13637 (PFAM) | |

## Supplementary Table 4

| Nr. | Contig sequence name | Number of reads | Sequence description (BLAST nr, E-value= > 0.0001) | Length (aa pos.) | E-Value | mean Similarity | Nr. of GO terms | Gene ontology terms and annotation (GO's) | Interpro scan results (BLAST2GO) | SignalP 4.1 (3.0) |
|---|---|---|---|---|---|---|---|---|---|---|
| 1 | lib3_tri_Filter15_IDBA_contig_0_0 | 6499 | ---NA--- | 2893 | | | 0 | - | PTHR24023 (PANTHER), PTHR24023:SF106 (PANTHER), SignalP-NN(euk) (SIGNALP), tmhmm (TMHMM) | |
| 2 | lib3_tri_Filter15_IDBA_contig_0_4 | 6499 | collagen triple helix repeat family partial | 2893 | 9.15E-07 | 69.00% | 0 | - | IPR008160 (PFAM); PTHR24023 (PANTHER), tmhmm (TMHMM) | |
| 3 | lib3_tri_Filter15_IDBA_contig_0_5 | 6499 | ---NA--- | 2893 | | | 0 | - | tmhmm (TMHMM) | |
| 4 | lib3_tri_Filter15_IDBA_contig_1000 2_1 | 16 | cathepsin l1-like | 121 | 8.24E-41 | 73.60% | 1 | F:peptidase activity | IPR000169 (PROSITE); IPR000668 (PFAM); IPR013128 (PANTHER); G3DSA:3.90.70.10 (GENE3D), PTHR12411:SF149 (PANTHER), SSF54001 (SUPERFAMILY) | |
| 5 | lib3_tri_Filter15_IDBA_contig_1003 _5 | 121 | pregnancy zone protein | 439 | 1.70E-74 | 57.30% | 4 | P:negative regulation of endopeptidase activity; C:extracellular space; C:extracellular region; F:endopeptidase inhibitor activity | IPR009048 (G3DSA:2.60.40.GENE3D); PTHR11412 (PANTHER), PTHR11412:SF36 (PANTHER) | |
| 6 | lib3_tri_Filter15_IDBA_contig_1004 0_4 | 40 | ---NA--- | 102 | | | 0 | - | no IPS match | |
| 7 | lib3_tri_Filter15_IDBA_contig_1005 _0 | 340 | serine-threonine kinase receptor-associated | 568 | 7.36E-78 | 77.85% | 3 | F:receptor activity; F:kinase activity; P:metabolic process | IPR001680 (PFAM); IPR011047 (SUPERFAMILY); IPR015943 (G3DSA:2.130.10.GENE3D); IPR017986 (PROFILE); IPR019775 (PROSITE); PTHR19877 (PANTHER), PTHR19877:SF2 (PANTHER) | |
| 8 | lib3_tri_Filter15_IDBA_contig_1006 2_1 | 340 | isoform cra_b | 109 | 6.30E-69 | 99.00% | 2 | C:nucleus; C:cytoplasm | IPR000626 (PFAM); IPR019954 (PROSITE); IPR019955 (PROFILE); IPR019956 (PRINTS); G3DSA:3.10.20.90 (GENE3D), PTHR10666 (PANTHER), SSF54236 (SUPERFAMILY) | |
| 9 | lib3_tri_Filter15_IDBA_contig_1023 _2 | 158 | cytosolic non-specific dipeptidase isoform 2 | 482 | 2.74E-134 | 82.70% | 5 | C:cytosol; F:binding; F:peptidase activity; P:protein metabolic process; P:catabolic process | IPR002933 (PFAM); IPR011650 (G3DSA:3.30.70.GENE3D); G3DSA:3.40.630.10 (GENE3D), PTHR11014 (PANTHER), PTHR11014:SF15 (PANTHER), tmhmm (TMHMM), SSF53187 (SUPERFAMILY) | |
| 10 | lib3_tri_Filter15_IDBA_contig_1032 _4 | 85 | ---NA--- | 425 | | | 0 | - | IPR011992 (G3DSA:1.10.238.GENE3D); PTHR19972 (PANTHER), PTHR19972:SF1 (PANTHER), SSF47473 (SUPERFAMILY) | |
| 11 | lib3_tri_Filter15_IDBA_contig_1032 _5 | 85 | calretinin | 425 | 3.34E-24 | 65.05% | 1 | C:plasma membrane | IPR002048 (SMART); IPR011992 (G3DSA:1.10.238.GENE3D); IPR018247 (PROSITE); PTHR19972 (PANTHER), SSF47473 (SUPERFAMILY) | |
| 12 | lib3_tri_Filter15_IDBA_contig_1035 _4 | 172 | enolase | 423 | 0 | 87.20% | 12 | P:reproduction; P:multicellular organismal development; P:embryo development; C:cytosol; C:protein complex; P:regulation of biological process; P:growth; F:catalytic activity; F:binding; P:carbohydrate metabolic process; P:generation of precursor metabolites and energy; P:catabolic process | IPR000941 (PRINTS); IPR020809 (PROSITE); IPR020810 (PFAM); G3DSA:3.20.20.120 (GENE3D), SSF51604 (SUPERFAMILY) | |
| 13 | lib3_tri_Filter15_IDBA_contig_1041 _4 | 1511 | protein nas-15 | 447 | 1.50E-58 | 52.90% | 1 | F:hydrolase activity | IPR001506 (PRINTS); IPR003582 (PFAM); IPR006026 (SMART); IPR024079 (G3DSA:3.40.390.GENE3D); PTHR10127 (PANTHER), PTHR10127:SF334 (PANTHER), SSF55486 (SUPERFAMILY) | |
| 14 | lib3_tri_Filter15_IDBA_contig_1053 _4 | 389 | PREDICTED: alpha-enolase-like | 417 | 2.42E-09 | 97.00% | 0 | - | IPR000941 (PANTHER); IPR020810 (PFAM); G3DSA:3.20.20.120 (GENE3D), tmhmm (TMHMM), SSF51604 (SUPERFAMILY) | |

| | | | | | | | | | |
|---|---|---|---|---|---|---|---|---|---|
| 15 | lib3_tri_Filter15_IDBA_contig_1057_0 | 1033 | cellular retinoic acid-binding protein 1 | 456 | 2.15E-14 | 54.00% | 4 | F:lipid binding; P:biological_process; P:regulation of biological process; P:multicellular organismal development | IPR000463 (PRINTS); IPR011038 (SUPERFAMILY); IPR012674 (G3DSA:2.40.128.GENE3D); PTHR11955 (PANTHER), PTHR11955:SF47 (PANTHER), PF14651 (PFAM) |
| 16 | lib3_tri_Filter15_IDBA_contig_1075_3 | 52 | protein-glutamine gamma-glutamyltransferase k | 410 | 0 | 66.95% | 3 | F:metal ion binding; P:peptide cross-linking; F:protein-glutamine gamma-glutamyltransferase activity | IPR002931 (G3DSA:3.90.260.GENE3D); IPR008958 (PFAM); IPR013783 (G3DSA:2.60.40.GENE3D); IPR013808 (PROSITE); IPR023608 (PANTHER); SSF54001 (SUPERFAMILY) |
| 17 | lib3_tri_Filter15_IDBA_contig_1077_1 | 589 | ficolin (collagen fibrinogen domain containing) 3 (hakata antigen) precursor | 620 | 1.37E-85 | 63.00% | 6 | F:receptor binding; C:extracellular space; P:signal transduction; F:signal transducer activity; C:membrane; C:collagen | IPR002181 (PFAM); IPR014715 (G3DSA:4.10.530.GENE3D); IPR014716 (G3DSA:3.90.215.GENE3D); IPR020837 (PROSITE); PTHR19143 (PANTHER) |
| 18 | lib3_tri_Filter15_IDBA_contig_107_3 | 105 | fibrinogen-like protein 1 | 326 | 1.13E-15 | 56.30% | 4 | F:receptor binding; C:extracellular space; P:signal transduction; P:response to stilbenoid | IPR002181 (PFAM); IPR014715 (G3DSA:4.10.530.GENE3D); PTHR19143 (PANTHER), tmhmm (TMHMM) |
| 19 | lib3_tri_Filter15_IDBA_contig_1093_1 | 436 | neuroglobin | 405 | 1.07E-47 | 55.45% | 2 | F:binding; P:transport | IPR000971 (PFAM); IPR009050 (SUPERFAMILY); IPR012292 (G3DSA:1.10.490.GENE3D); IPR013316 (PRINTS); PTHR22924 (PANTHER) |
| 20 | lib3_tri_Filter15_IDBA_contig_1097_3 | 1204 | myosin heavy chain | 702 | 1.11E-124 | 85.65% | 7 | C:cytoskeleton; C:protein complex; F:actin binding; F:nucleotide binding; C:cytoplasm; F:motor activity; F:protein binding | IPR002928 (PFAM); PTHR13140 (PANTHER), PTHR13140:SF106 (PANTHER), tmhmm (TMHMM) |
| 21 | lib3_tri_Filter15_IDBA_contig_1099_2 | 192 | paramyosin | 401 | 4.29E-22 | 72.15% | 3 | C:cytoskeleton; C:protein complex; F:motor activity | IPR002928 (PFAM); PTHR13140 (PANTHER), PTHR13140:SF106 (PANTHER) |
| 22 | lib3_tri_Filter15_IDBA_contig_1105_3 | 299 | hypothetical protein CAPTEDRAFT_212422 | 422 | 2.06E-24 | 49.17% | 0 | - | IPR000436 (PFAM); IPR000884 (SMART); IPR013032 (PROSITE); G3DSA:2.10.70.10 (GENE3D), G3DSA:2.20.100.10 (GENE3D), PTHR19325 (PANTHER), SignalP-NN(euk) (SIGNALP), tmhmm (TMHMM) |
| 23 | lib3_tri_Filter15_IDBA_contig_1109_0 | 297 | af312826_1sea star regeneration-associated protease srap | 823 | 4.81E-93 | 59.50% | 1 | F:peptidase activity | IPR000859 (G3DSA:2.60.120.GENE3D); IPR001254 (PFAM); IPR001314 (PRINTS); IPR009003 (SUPERFAMILY); IPR018114 (PROSITE); G3DSA:2.40.10.10 (GENE3D), PTHR24265 (PANTHER) |
| 24 | lib3_tri_Filter15_IDBA_contig_1136_5 | 312 | si:dkey- protein | 390 | 1.70E-13 | 46.30% | 10 | F:carbohydrate binding; P:biological_process; F:transmembrane signaling receptor activity; C:integral to membrane; C:membrane; F:G-protein coupled receptor activity; P:neuropeptide signaling pathway; P:cell surface receptor signaling pathway; P:G-protein coupled receptor signaling pathway; C:plasma membrane | IPR000922 (PFAM); PTHR12011 (PANTHER), PTHR12011:SF4 (PANTHER), tmhmm (TMHMM) |
| 25 | lib3_tri_Filter15_IDBA_contig_1140_4 | 2812 | guanine nucleotide-binding protein subunit beta-2-like 1 | 390 | 0 | 93.80% | 25 | P:regulation of biological process; P:cell death; C:cytoskeleton; F:protein binding; C:cell; C:ribosome; F:receptor activity; F:receptor binding; P:biological_process; P:cell cycle; P:cellular component organization; C:plasma membrane; P:translation; P:cellular protein modification process; P:transport; P:signal transduction; C:cytoplasm; F:molecular_function; P:protein metabolic process; P:catabolic process; F:enzyme regulator activity; F:kinase activity; C:nucleus; P:cell growth; P:metabolic process | IPR001680 (PFAM); IPR015943 (G3DSA:2.130.10.GENE3D); IPR017986 (PROFILE); IPR019775 (PROSITE); IPR020472 (PRINTS); PTHR19868 (PANTHER), PTHR19868:SF0 (PANTHER) |

| | | | | | | | | | | |
|---|---|---|---|---|---|---|---|---|---|---|
| 26 | lib3_tri_Filter15_IDBA_contig_1145_2 | 127 | ---NA--- | 388 | | | 0 | - | no IPS match | |
| 27 | lib3_tri_Filter15_IDBA_contig_1145_3 | 127 | ---NA--- | 389 | | | 0 | - | IPR008160 (PFAM); PTHR24023 (PANTHER) | |
| 28 | lib3_tri_Filter15_IDBA_contig_1154_2 | 72 | proactivator polypeptide | 386 | 2.93E-100 | 53.00% | 3 | P:lipid metabolic process; P:sphingolipid metabolic process; C:lysosome | IPR007856 (PFAM); IPR008138 (PFAM); IPR008139 (SMART); IPR008373 (PRINTS); IPR011001 (G3DSA:1.10.225.GENE3D); PTHR11480 (PANTHER), PTHR11480:SF3 (PANTHER) | |
| 29 | lib3_tri_Filter15_IDBA_contig_1164_3 | 285 | histone -like | 383 | 2.00E-64 | 99.40% | 4 | C:chromosome; F:DNA binding; P:organelle organization; C:nucleus | IPR002119 (PRINTS); IPR007125 (PFAM); IPR009072 (G3DSA:1.10.20.GENE3D); PTHR23430 (PANTHER) | |
| 30 | lib3_tri_Filter15_IDBA_contig_1172_1 | 171 | aldehyde mitochondrial-like isoform 1 | 413 | 0 | 81.80% | 6 | C:cytoplasm; F:catalytic activity; P:lipid metabolic process; P:secondary metabolic process; P:catabolic process; P:metabolic process | IPR015590 (PFAM); IPR016160 (PROSITE); IPR016161 (SUPERFAMILY); IPR016162 (G3DSA:3.40.605.GENE3D); IPR016163 (G3DSA:3.40.309.GENE3D); PTHR11699 (PANTHER), PTHR11699:SF46 (PANTHER) | |
| 31 | lib3_tri_Filter15_IDBA_contig_1175_0 | 517 | ---NA--- | 381 | | | 0 | - | SignalP-NN(euk) (SIGNALP) | Yes |
| 32 | lib3_tri_Filter15_IDBA_contig_1175_5 | 517 | signal cub egf-like 1 | 380 | 7.76E-23 | 52.10% | 3 | C:plasma membrane; C:cell; F:protein binding | IPR000152 (PROSITE); IPR000742 (SMART); IPR001881 (SMART); IPR008160 (PFAM); IPR013032 (PROSITE); IPR018097 (PROSITE); G3DSA:2.10.25.10 (GENE3D), PTHR24046 (PANTHER), PF14670 (PFAM), SSF57196 (SUPERFAMILY) | |
| 33 | lib3_tri_Filter15_IDBA_contig_1188_4 | 78 | kettin protein | 378 | 2.17E-96 | 58.00% | 6 | P:intracellular signal transduction; P:cyclic nucleotide biosynthetic process; F:phosphorus-oxygen lyase activity; F:Rho guanyl-nucleotide exchange factor activity; P:regulation of Rho protein signal transduction; C:intracellular | IPR003599 (SMART); IPR007110 (PROFILE); IPR013098 (PFAM); IPR013783 (G3DSA:2.60.40.GENE3D); SSF48726 (SUPERFAMILY) | |
| 34 | lib3_tri_Filter15_IDBA_contig_1213_0 | 382 | protease inhibitor | 377 | 5.08E-14 | 64.40% | 3 | C:cell wall; P:response to stress; P:response to biotic stimulus | IPR000864 (PRODOM); G3DSA:3.30.10.10 (GENE3D), tmhmm (TMHMM) | |
| 35 | lib3_tri_Filter15_IDBA_contig_1224_0 | 172 | fibrinogen-like protein 1-like | 370 | 2.53E-55 | 56.55% | 3 | F:receptor binding; C:extracellular space; P:signal transduction | IPR002181 (PFAM); IPR014715 (G3DSA:4.10.530.GENE3D); IPR014716 (G3DSA:3.90.215.GENE3D); PTHR19143 (PANTHER), tmhmm (TMHMM) | |
| 36 | lib3_tri_Filter15_IDBA_contig_1235_5 | 274 | kazal-type serine protease inhibitor domain-containing protein 1 | 535 | 8.34E-53 | 62.70% | 2 | C:proteinaceous extracellular matrix; P:cellular component organization | IPR003598 (SMART); IPR003599 (SMART); IPR007110 (PROFILE); IPR011390 (PANTHER); IPR013098 (PFAM); IPR013783 (G3DSA:2.60.40.GENE3D); PTHR14186:SF7 (PANTHER), tmhmm (TMHMM), SSF48726 (SUPERFAMILY) | |
| 37 | lib3_tri_Filter15_IDBA_contig_1267_3 | 109 | hypothetical protein BRAFLDRAFT_105827 | 360 | 3.53E-07 | 40.00% | 1 | F:carbohydrate binding | IPR000562 (G3DSA:2.10.10.GENE3D); IPR013806 (SUPERFAMILY); PTHR22918 (PANTHER), PTHR22918:SF0 (PANTHER), SignalP-NN(euk) (SIGNALP), tmhmm (TMHMM) | |
| 38 | lib3_tri_Filter15_IDBA_contig_1269_2 | 2484 | ---NA--- | 359 | | | 0 | - | SignalP-NN(euk) (SIGNALP), tmhmm (TMHMM) | yes |
| 39 | lib3_tri_Filter15_IDBA_contig_1269_4 | 2484 | ---NA--- | 360 | | | 0 | - | IPR008160 (PFAM); PTHR24022 (PANTHER), PTHR24022:SF54 (PANTHER), tmhmm (TMHMM) | |
| 40 | lib3_tri_Filter15_IDBA_contig_1284_4 | 138 | af355375_1 reverse transcriptase | 357 | 1.27E-42 | 51.20% | 5 | F:RNA binding; P:RNA-dependent DNA replication; F:RNA-directed DNA polymerase activity; F:zinc ion binding; C:intracellular | PTHR21301 (PANTHER) | |
| 41 | lib3_tri_Filter15_IDBA_contig_1330_3 | 6299 | ---NA--- | 795 | | | 0 | - | SignalP-NN(euk) (SIGNALP), tmhmm (TMHMM) | |
| 42 | lib3_tri_Filter15_IDBA_contig_1331_3 | 9156 | ---NA--- | 905 | | | 0 | - | SignalP-NN(euk) (SIGNALP), tmhmm (TMHMM) | |

| | | | | | | | | | | |
|---|---|---|---|---|---|---|---|---|---|---|
| 43 | lib3_tri_Filter15_IDBA_contig_1331_4 | 9156 | ---NA--- | 905 | | | 0 | - | IPR011004 (SUPERFAMILY); G3DSA:2.160.10.10 (GENE3D) | |
| 44 | lib3_tri_Filter15_IDBA_contig_1363_2 | 164 | glyceraldehyde-3-phosphate dehydrogenase | 342 | 3.09E-21 | 88.25% | 7 | C:cytoplasm; F:nucleotide binding; P:carbohydrate metabolic process; P:generation of precursor metabolites and energy; P:catabolic process; F:catalytic activity; P:metabolic process | IPR020829 (PFAM); IPR020831 (PANTHER); G3DSA:3.30.360.10 (GENE3D), tmhmm (TMHMM), SSF55347 (SUPERFAMILY) | |
| 45 | lib3_tri_Filter15_IDBA_contig_1406_2 | 71 | briggsae cbr-clec-86 protein | 334 | 4.31E-18 | 50.33% | 1 | F:carbohydrate binding | IPR001304 (PFAM); IPR016186 (G3DSA:3.10.100.GENE3D); IPR016187 (SUPERFAMILY); PTHR22803 (PANTHER) | |
| 46 | lib3_tri_Filter15_IDBA_contig_1502_1 | 279 | dorsal-ventral patterning protein tolloid-like | 416 | 1.26E-29 | 47.10% | 1 | F:catalytic activity | IPR000859 (G3DSA:2.60.120.GENE3D); PTHR10127 (PANTHER) | |
| 47 | lib3_tri_Filter15_IDBA_contig_1507_5 | 45 | | 317 | | | | | IPR003961 (PFAM); IPR013783 (G3DSA:2.60.40.GENE3D); PR00014 (PRINTS) | |
| 48 | lib3_tri_Filter15_IDBA_contig_1512_2 | 64 | low quality protein: titin-like | 316 | 3.41E-64 | 53.85% | 9 | P:lipid transport; F:nucleic acid binding; F:lipid transporter activity; P:intracellular signal transduction; P:cyclic nucleotide biosynthetic process; F:phosphorus-oxygen lyase activity; F:Rho guanyl-nucleotide exchange factor activity; P:regulation of Rho protein signal transduction; C:intracellular | IPR003598 (SMART); IPR003599 (SMART); IPR007110 (PROFILE); IPR013098 (PFAM); IPR013783 (G3DSA:2.60.40.GENE3D); IPR020675 (PANTHER); PTHR22964:SF3 (PANTHER), SSF48726 (SUPERFAMILY) | |
| 49 | lib3_tri_Filter15_IDBA_contig_1522_0 | 71 | receptor tyrosine-protein kinase erbb-4 | 316 | 1.25E-21 | 51.00% | 16 | P:cell differentiation; P:multicellular organismal development; P:primary metabolic process; P:metabolic process; P:biological_process; P:signal transduction; P:response to external stimulus; P:anatomical structure morphogenesis; P:response to stress; P:growth; P:cellular homeostasis; P:cellular component organization; P:cell communication; P:regulation of biological process; F:kinase activity; F:binding | IPR000494 (G3DSA:3.80.20.GENE3D); IPR006212 (SMART); IPR009030 (SUPERFAMILY); G3DSA:2.10.220.10 (GENE3D), PTHR24416 (PANTHER), PTHR24416:SF95 (PANTHER), PF14843 (PFAM), SSF52058 (SUPERFAMILY) | |
| 50 | lib3_tri_Filter15_IDBA_contig_1528_3 | 53 | stress-70 mitochondrial | 315 | 0 | 92.95% | 11 | P:protein metabolic process; C:nucleolus; P:protein transport; C:mitochondrion; P:mitochondrion organization; C:cell; P:regulation of biological process; P:cell death; F:protein binding; F:nucleotide binding; P:response to stress | IPR013126 (PRINTS); IPR018181 (PROSITE); G3DSA:3.30.30.30 (GENE3D), G3DSA:3.30.420.40 (GENE3D), G3DSA:3.90.640.10 (GENE3D), PTHR19375 (PANTHER), PTHR19375:SF1 (PANTHER), SSF53067 (SUPERFAMILY) | |
| 51 | lib3_tri_Filter15_IDBA_contig_1536_3 | 288 | neuronal acetylcholine receptor subunit alpha-6 isoform 1 | 313 | 1.65E-22 | 47.90% | 4 | P:biological_process; P:ion transport; F:ion channel activity; C:cell | IPR006201 (PANTHER); IPR006202 (G3DSA:2.70.170.GENE3D); PTHR18945:SF127 (PANTHER), SignalP-NN(euk) (SIGNALP), tmhmm (TMHMM) | Yes |
| 52 | lib3_tri_Filter15_IDBA_contig_1548_0 | 58 | epididymal sperm-binding protein 1-like | 311 | 8.50E-10 | 45.00% | 0 | - | IPR000562 (G3DSA:2.10.10.GENE3D); IPR013806 (SUPERFAMILY); PTHR22918 (PANTHER), PTHR22918:SF0 (PANTHER), SignalP-NN(euk) (SIGNALP) | Yes |
| 53 | lib3_tri_Filter15_IDBA_contig_1567_1 | 601 | carbonic anhydrase | 454 | 4.34E-106 | 64.15% | 10 | F:metal ion binding; F:zinc ion binding; P:one-carbon metabolic process; F:lyase activity; F:carbonate dehydratase activity; P:secretion; C:apical part of cell; P:carbon dioxide transport; C:cytosol; P:morphogenesis of an epithelium | IPR001148 (G3DSA:3.10.200.GENE3D); IPR018338 (PROSITE); IPR023561 (PANTHER) | |
| 54 | lib3_tri_Filter15_IDBA_contig_1576_1 | 48 | unc- isoform c | 305 | 1.33E-32 | 42.15% | 3 | P:biological_process; C:intracellular; F:transferase activity | IPR003598 (SMART); IPR003599 (SMART); IPR007110 (PROFILE); IPR013098 (PFAM); IPR013783 (G3DSA:2.60.40.GENE3D); PTHR10489 (PANTHER), SSF48726 (SUPERFAMILY) | |

| | | | | | | | | | | |
|---|---|---|---|---|---|---|---|---|---|---|
| 55 | lib3_tri_Filter15_IDBA_contig_1660_3 | 210 | viral a-type inclusion protein | 552 | 2.99E-08 | 49.00% | 0 | - | IPR009053 (SUPERFAMILY) | |
| 56 | lib3_tri_Filter15_IDBA_contig_1663_1 | 58 | myopalladin isoform 1 | 292 | 3.07E-19 | 46.25% | 16 | C:cleavage furrow; C:membrane; P:positive regulation of wound healing; C:lamellipodium; P:aorta smooth muscle tissue morphogenesis; C:cytoplasm; F:ATP binding; P:bleb assembly; P:positive regulation of cell migration; F:myosin light chain kinase activity; F:calmodulin binding; P:positive regulation of calcium ion transport; F:metal ion binding; P:cellular hypotonic response; C:cytosol; C:stress fiber | IPR003598 (SMART); IPR003599 (SMART); IPR007110 (PROFILE); IPR013098 (PFAM); IPR013783 (G3DSA:2.60.40.GENE3D); PTHR10489 (PANTHER), SSF48726 (SUPERFAMILY) | |
| 57 | lib3_tri_Filter15_IDBA_contig_1686_1 | 61 | peptidyl-prolyl cis-trans isomerase b | 291 | 1.71E-29 | 78.40% | 8 | C:extracellular space; P:protein metabolic process; C:cytoskeleton; C:protein complex; P:reproduction; C:lipid particle; P:cellular protein modification process; F:catalytic activity | IPR002130 (PRINTS); G3DSA:2.40.100.10 (GENE3D), PTHR11071 (PANTHER) | |
| 58 | lib3_tri_Filter15_IDBA_contig_1715_4 | 1392 | ---NA--- | 287 | | | 0 | - | IPR001134 (PROFILE); IPR001820 (PANTHER); IPR008993 (SUPERFAMILY); G3DSA:2.40.50.120 (GENE3D), SignalP-NN(euk) (SIGNALP) | |
| 59 | lib3_tri_Filter15_IDBA_contig_1726_0 | 64 | ---NA--- | 286 | | | 0 | - | IPR008160 (PFAM); PTHR24023 (PANTHER) | |
| 60 | lib3_tri_Filter15_IDBA_contig_1726_3 | 64 | ---NA--- | 286 | | | 0 | - | no IPS match | |
| 61 | lib3_tri_Filter15_IDBA_contig_1744_3 | 117 | galectin-3-binding protein precursor | 325 | 6.29E-28 | 65.80% | 2 | F:scavenger receptor activity; C:membrane | IPR001190 (PRINTS); IPR017448 (SMART); G3DSA:3.10.250.10 (GENE3D), PTHR19331 (PANTHER) | |
| 62 | lib3_tri_Filter15_IDBA_contig_1747_0 | 112 | neurotrypsin isoform x2 | 284 | 2.47E-53 | 61.30% | 1 | F:hydrolase activity | IPR001190 (PRINTS); IPR017448 (SMART); G3DSA:3.10.250.10 (GENE3D), PTHR19331 (PANTHER) | |
| 63 | lib3_tri_Filter15_IDBA_contig_1780_5 | 63 | neuronal acetylcholine receptor subunit alpha-7 | 280 | 1.23E-60 | 70.35% | 2 | C:cell; F:ion channel activity | IPR006029 (PFAM); IPR006201 (PANTHER); IPR006202 (G3DSA:2.70.170.GENE3D); IPR027361 (G3DSA:1.20.120.GENE3D); PTHR18945:SF127 (PANTHER), tmhmm (TMHMM) | |
| 64 | lib3_tri_Filter15_IDBA_contig_1798_5 | 120 | hypothetical protein CAPTEDRAFT_223820 | 334 | 1.18E-25 | 65.00% | 0 | - | IPR021712 (PFAM); PD936484 (PRODOM), SignalP-NN(euk) (SIGNALP), tmhmm (TMHMM) | Yes |
| 65 | lib3_tri_Filter15_IDBA_contig_1829_2 | 53 | ---NA--- | 275 | | | 0 | - | no IPS match | |
| 66 | lib3_tri_Filter15_IDBA_contig_1829_5 | 53 | ---NA--- | 275 | | | 0 | - | IPR008160 (PFAM); PTHR24023 (PANTHER) | |
| 67 | lib3_tri_Filter15_IDBA_contig_1833_1 | 94 | deleted in malignant brain tumors 1 | 275 | 2.70E-29 | 65.65% | 8 | P:regulation of biological process; P:protein transport; F:protein binding; P:transport; P:cell differentiation; C:cytoplasm; P:biological_process; C:cell | IPR001190 (PRINTS); IPR017448 (SMART); G3DSA:3.10.250.10 (GENE3D), PTHR19331 (PANTHER) | |
| 68 | lib3_tri_Filter15_IDBA_contig_1839_4 | 457 | pro protein convertase subtilisin kexintype 5 partial | 514 | 5.05E-36 | 42.15% | 6 | P:proteolysis; P:cell adhesion; C:membrane; F:zinc ion binding; F:metalloendopeptidase activity; C:intracellular | IPR000742 (SMART); IPR001577 (PANTHER); IPR006212 (SMART); IPR009030 (SUPERFAMILY); G3DSA:2.10.220.10 (GENE3D), PTHR10942:SF0 (PANTHER), SignalP-NN(euk) (SIGNALP) | Yes |
| 69 | lib3_tri_Filter15_IDBA_contig_1840_4 | 93 | high cysteine membrane protein group 2 | 218 | 7.88E-19 | 44.20% | 10 | P:proteolysis; P:cell adhesion; C:membrane; F:zinc ion binding; F:metalloendopeptidase activity; F:copper ion binding; F:sequence-specific DNA binding transcription factor activity; F:DNA binding; C:nucleus; P:regulation of transcription, DNA-dependent | IPR001577 (PANTHER); IPR009030 (SUPERFAMILY); PTHR10942:SF0 (PANTHER), SignalP-NN(euk) (SIGNALP) | Yes |

| | | | | | | | | | | |
|---|---|---|---|---|---|---|---|---|---|---|
| 70 | lib3_tri_Filter15_IDBA_contig_1848_0 | 54 | elastase 4 precursor | 274 | 2.92E-41 | 54.45% | 1 | F:catalytic activity | IPR001254 (PFAM); IPR001314 (PRINTS); IPR009003 (SUPERFAMILY); IPR018114 (PROSITE); G3DSA:2.40.10.10 (GENE3D), PTHR24265 (PANTHER) | |
| 71 | lib3_tri_Filter15_IDBA_contig_1878_5 | 59 | ---NA--- | 270 | | | 0 | - | IPR001190 (PFAM); IPR017448 (SUPERFAMILY); G3DSA:3.10.250.10 (GENE3D), PTHR19331 (PANTHER), PTHR19331:SF13 (PANTHER) | |
| 72 | lib3_tri_Filter15_IDBA_contig_1886_0 | 156 | serine protease 44-like | 309 | 6.38E-33 | 50.90% | 1 | F:catalytic activity | IPR001254 (PFAM); IPR001314 (PRINTS); IPR009003 (SUPERFAMILY); IPR018114 (PROSITE); G3DSA:2.40.10.10 (GENE3D), PTHR24265 (PANTHER) | |
| 73 | lib3_tri_Filter15_IDBA_contig_1891_5 | 349 | protease inhibitor epi11 | 459 | 3.15E-27 | 48.50% | 2 | P:proteolysis; F:peptidase activity | IPR002350 (PFAM); G3DSA:3.30.60.30 (GENE3D), PTHR10913 (PANTHER), SignalP-NN(euk) (SIGNALP), tmhmm (TMHMM), SSF100895 (SUPERFAMILY) | Yes |
| 74 | lib3_tri_Filter15_IDBA_contig_1892_5 | 106 | protease inhibitor epi11 | 239 | 2.05E-28 | 48.50% | 2 | P:proteolysis; F:peptidase activity | IPR002350 (PFAM); G3DSA:3.30.60.30 (GENE3D), PTHR10913 (PANTHER), SignalP-NN(euk) (SIGNALP), tmhmm (TMHMM), SSF100895 (SUPERFAMILY) | Yes |
| 75 | lib3_tri_Filter15_IDBA_contig_1893_3 | 35 | protein | 122 | 1.11E-13 | 49.55% | 5 | P:proteolysis; F:peptidase activity; F:peptidase inhibitor activity; F:serine-type endopeptidase inhibitor activity; P:negative regulation of peptidase activity | IPR002350 (PFAM); G3DSA:3.30.60.30 (GENE3D), PTHR21312 (PANTHER), SignalP-NN(euk) (SIGNALP), SSF100895 (SUPERFAMILY) | Yes |
| 76 | lib3_tri_Filter15_IDBA_contig_1895_2 | 29 | von willebrand factor type egf and pentraxin domain-containing protein 1 | 268 | 8.69E-46 | 49.45% | 3 | F:carbohydrate binding; F:calcium ion binding; F:chromatin binding | IPR000436 (PFAM); G3DSA:2.10.70.10 (GENE3D), PTHR19325 (PANTHER) | |
| 77 | lib3_tri_Filter15_IDBA_contig_1905_5 | 125 | anionic trypsin-2-like | 267 | 4.74E-47 | 57.70% | 1 | F:hydrolase activity | IPR001254 (PFAM); IPR001314 (PRINTS); IPR009003 (SUPERFAMILY); IPR018114 (PROSITE); G3DSA:2.40.10.10 (GENE3D), PTHR24265 (PANTHER) | |
| 78 | lib3_tri_Filter15_IDBA_contig_191_2 | 136 | hep_hag family protein | 145 | 9.83E-11 | 64.67% | 2 | C:outer membrane; P:pathogenesis | no IPS match | |
| 79 | lib3_tri_Filter15_IDBA_contig_191_5 | 136 | ---NA--- | 145 | | | 0 | - | IPR008160 (PFAM); PTHR24023 (PANTHER) | |
| 80 | lib3_tri_Filter15_IDBA_contig_1940_1 | 3425 | kielin chordin-like protein | 578 | 3.54E-34 | 37.60% | 3 | F:peptidase inhibitor activity; P:negative regulation of peptidase activity; C:extracellular region | IPR001007 (PFAM); IPR006552 (SMART); IPR008037 (PFAM); G3DSA:2.10.70.10 (GENE3D), PTHR11339 (PANTHER), PTHR11339:SF46 (PANTHER), SignalP-NN(euk) (SIGNALP), tmhmm (TMHMM), SSF57603 (SUPERFAMILY) | Yes |
| 81 | lib3_tri_Filter15_IDBA_contig_1945_1 | 1169 | a chain solution structure of component iv glycera dibranchiata monomeric hemoglobin-co | 333 | 2.95E-38 | 54.70% | 1 | F:binding | IPR000971 (PFAM); IPR009050 (SUPERFAMILY); IPR012292 (G3DSA:1.10.490.GENE3D); IPR013316 (PRINTS); PTHR22924 (PANTHER), tmhmm (TMHMM) | |
| 82 | lib3_tri_Filter15_IDBA_contig_1948_4 | 57 | collagen alpha-3 chain | 263 | 1.62E-72 | 58.15% | 0 | - | IPR000742 (PROFILE); IPR002035 (G3DSA:3.40.50.GENE3D); IPR013032 (PROSITE); PR00453 (PRINTS), G3DSA:2.10.25.10 (GENE3D), PTHR22992 (PANTHER), SSF53300 (SUPERFAMILY) | |
| 83 | lib3_tri_Filter15_IDBA_contig_1950_2 | 67 | | 263 | | | | | IPR003961 (PFAM); IPR013783 (G3DSA:2.60.40.GENE3D); PR00014 (PRINTS) | |
| 84 | lib3_tri_Filter15_IDBA_contig_1955_2 | 33 | antistasin-like protein | 262 | 5.22E-44 | 51.05% | 3 | P:negative regulation of catalytic activity; F:enzyme inhibitor activity; F:serine-type endopeptidase inhibitor activity | IPR004094 (PFAM); IPR011061 (SUPERFAMILY); IPR018112 (G3DSA:2.10.22.GENE3D); PTHR11339 (PANTHER), PTHR11339:SF40 (PANTHER) | |
| 85 | lib3_tri_Filter15_IDBA_contig_1964_0 | 60 | low quality protein: titin | 262 | 4.10E-46 | 49.20% | 1 | F:binding | IPR003598 (SMART); IPR003599 (SMART); IPR007110 (PROFILE); IPR013098 (PFAM); IPR013783 (G3DSA:2.60.40.GENE3D); SSF48726 (SUPERFAMILY) | |

| | | | | | | | | | |
|---|---|---|---|---|---|---|---|---|---|
| 86 | lib3_tri_Filter15_IDBA_contig_1969_4 | 41 | cathepsin l-like | 262 | 5.44E-120 | 80.35% | 3 | F:peptidase activity; P:protein metabolic process; P:catabolic process | IPR000169 (PROSITE); IPR000668 (PRINTS); IPR013128 (PANTHER); IPR025660 (PROSITE); IPR025661 (PROSITE); G3DSA:3.90.70.10 (GENE3D), PTHR12411:SF149 (PANTHER), SignalP-NN(euk) (SIGNALP), SSF54001 (SUPERFAMILY) |
| 87 | lib3_tri_Filter15_IDBA_contig_1974_4 | 93 | gamma polypeptide | 261 | 1.00E-43 | 53.90% | 3 | P:biological_process; C:cytoplasm; C:extracellular region | IPR002181 (PFAM); IPR014715 (G3DSA:4.10.530.GENE3D); IPR014716 (G3DSA:3.90.215.GENE3D); IPR020837 (PROSITE); PTHR19143 (PANTHER) |
| 88 | lib3_tri_Filter15_IDBA_contig_1977_0 | 85 | ---NA--- | 261 | | | 0 | - | IPR008160 (PFAM); PTHR24023 (PANTHER) |
| 89 | lib3_tri_Filter15_IDBA_contig_1977_4 | 85 | ---NA--- | 261 | | | 0 | - | no IPS match |
| 90 | lib3_tri_Filter15_IDBA_contig_1978_3 | 73 | matrix metalloproteinase-9 | 261 | 8.00E-20 | 57.85% | 1 | F:hydrolase activity | IPR000562 (G3DSA:2.10.10.GENE3D); IPR013806 (SUPERFAMILY); PTHR22918 (PANTHER), PTHR22918:SF0 (PANTHER) |
| 91 | lib3_tri_Filter15_IDBA_contig_2032_3 | 91 | b chain acetylcholine binding protein as template for hierarchical in silico screening procedures to identify structurally novel ligands for the nicotinic receptors | 283 | 2.40E-13 | 51.15% | 6 | F:receptor activity; C:integral to membrane; C:membrane; F:extracellular ligand-gated ion channel activity; P:ion transport; P:transport | IPR006201 (PANTHER); IPR006202 (G3DSA:2.70.170.GENE3D); PTHR18945:SF71 (PANTHER), tmhmm (TMHMM) |
| 92 | lib3_tri_Filter15_IDBA_contig_2038_2 | 41 | fibronectin type iii domain protein | 256 | 1.02E-79 | 53.30% | 4 | P:regulation of biological process; F:transferase activity; C:cytoplasm; P:growth | IPR003598 (SMART); IPR003599 (SMART); IPR007110 (PROFILE); IPR013098 (PFAM); IPR013783 (G3DSA:2.60.40.GENE3D); IPR020675 (PANTHER); IPR020682 (PTHR22964:PANTHER); SSF48726 (SUPERFAMILY) |
| 93 | lib3_tri_Filter15_IDBA_contig_2039_5 | 75 | contactin | 265 | 4.36E-27 | 57.10% | 6 | P:cellular homeostasis; P:multicellular organismal development; P:cell communication; P:cellular component organization; C:plasma membrane; P:cell differentiation | IPR003961 (PFAM); IPR013783 (G3DSA:2.60.40.GENE3D); PTHR10489 (PANTHER), PTHR10489:SF48 (PANTHER) |
| 94 | lib3_tri_Filter15_IDBA_contig_2055_0 | 59 | phosphoglycerate kinase 1 | 255 | 2.69E-144 | 87.05% | 9 | C:cell; P:biosynthetic process; P:carbohydrate metabolic process; F:nucleotide binding; F:kinase activity; P:metabolic process; P:generation of precursor metabolites and energy; P:catabolic process; C:cytosol | IPR001576 (PRINTS); IPR015824 (G3DSA:3.40.50.GENE3D); IPR015901 (G3DSA:3.40.50.GENE3D); PTHR11406:SF1 (PANTHER) |
| 95 | lib3_tri_Filter15_IDBA_contig_2089_4 | 114 | 78 kda glucose-regulated protein | 483 | 1.68E-145 | 92.80% | 30 | C:cell; C:endoplasmic reticulum; C:protein complex; F:protein binding; P:signal transduction; P:anatomical structure morphogenesis; P:multicellular organismal development; P:regulation of biological process; P:embryo development; P:nucleobase-containing compound metabolic process; P:catabolic process; P:response to external stimulus; P:response to stress; P:cellular protein modification process; F:enzyme regulator activity; P:protein metabolic process; P:cell death; P:response to biotic stimulus; C:nucleus; C:cytosol; P:transport; C:cytoplasm; F:binding; P:cell communication; P:metabolic process; F:nucleotide binding; F:hydrolase activity; C:cytoplasmic membrane-bounded vesicle; F:calcium ion | IPR013126 (PRINTS); G3DSA:1.20.1270.10 (GENE3D), G3DSA:2.60.34.10 (GENE3D), PTHR19375 (PANTHER), SSF100920 (SUPERFAMILY), SSF100934 (SUPERFAMILY), SSF53067 (SUPERFAMILY) |

| | | | | | | | | | | |
|---|---|---|---|---|---|---|---|---|---|---|
| 96 | lib3_tri_Filter15_IDBA_contig_2098_0 | 1199 | cysteine-rich secretory protein mr30 | 355 | 3.92E-59 | 52.30% | 6 | F:ion channel inhibitor activity; F:potassium channel inhibitor activity; C:extracellular region; F:calcium channel inhibitor activity; P:defense response; F:molecular_function | IPR001283 (PRINTS); IPR014044 (G3DSA:3.40.33.GENE3D); SignalP-NN(euk) (SIGNALP), tmhmm (TMHMM) | Yes |
| 97 | lib3_tri_Filter15_IDBA_contig_2124_0 | 74 | tyrosine--trna cytoplasmic-like | 248 | 4.09E-83 | 74.10% | 8 | C:cytoplasm; F:catalytic activity; F:RNA binding; P:translation; P:nucleobase-containing compound metabolic process; ; C:nucleus; F:nucleotide binding | IPR002547 (PFAM); IPR012340 (G3DSA:2.40.50.GENE3D); IPR023617 (PTHR11946:PANTHER); PTHR11946 (PANTHER) | |
| 98 | lib3_tri_Filter15_IDBA_contig_2125_3 | 488 | protein | 355 | 2.76E-18 | 64.45% | 1 | F:enzyme regulator activity | IPR000864 (PRODOM); G3DSA:3.30.10.10 (GENE3D), tmhmm (TMHMM) | |
| 99 | lib3_tri_Filter15_IDBA_contig_2137_2 | 59 | galactose soluble 8-like | 190 | 4.88E-39 | 57.80% | 1 | F:carbohydrate binding | IPR001079 (PFAM); IPR008985 (SUPERFAMILY); IPR013320 (G3DSA:2.60.120.GENE3D); PTHR11346 (PANTHER) | |
| 100 | lib3_tri_Filter15_IDBA_contig_2138_0 | 60 | galectin-9 isoform x4 | 248 | 2.24E-34 | 55.35% | 1 | F:carbohydrate binding | IPR001079 (PFAM); IPR008985 (SUPERFAMILY); IPR013320 (G3DSA:2.60.120.GENE3D); PTHR11346 (PANTHER) | |
| 101 | lib3_tri_Filter15_IDBA_contig_2143_4 | 61 | kunitz-type protease inhibitor 2 | 246 | 5.78E-15 | 71.35% | 4 | P:proteolysis; F:peptidase activity; F:serine-type endopeptidase inhibitor activity; C:cytoplasm | IPR002223 (PRINTS); IPR004094 (PFAM); IPR011061 (SUPERFAMILY); IPR018112 (G3DSA:2.10.22.GENE3D); IPR020901 (PROSITE); PTHR10083 (PANTHER), PTHR10083:SF19 (PANTHER) | |
| 102 | lib3_tri_Filter15_IDBA_contig_2159_1 | 66 | carboxypeptidase b-like | 245 | 2.24E-35 | 54.70% | 4 | P:proteolysis; F:metallocarboxypeptidase activity; F:carboxypeptidase activity; F:zinc ion binding | IPR000834 (PFAM); G3DSA:3.40.630.10 (GENE3D), PTHR11705 (PANTHER), SSF53187 (SUPERFAMILY) | |
| 103 | lib3_tri_Filter15_IDBA_contig_2166_3 | 194 | group xiia secretory phospholipase a2 | 277 | 7.00E-24 | 54.90% | 8 | F:phospholipase A2 activity; F:calcium ion binding; P:lipid catabolic process; P:phospholipid metabolic process; C:extracellular region; F:hydrolase activity; C:endoplasmic reticulum; C:Golgi apparatus | IPR010711 (PANTHER); IPR013090 (PROSITE); IPR016090 (G3DSA:1.20.90.GENE3D) | |
| 104 | lib3_tri_Filter15_IDBA_contig_2167_3 | 824 | group xiia secretory phospholipase a2 | 658 | 1.76E-24 | 55.35% | 4 | P:lipid metabolic process; F:hydrolase activity; C:Golgi apparatus; C:endoplasmic reticulum | IPR010711 (PANTHER); IPR013090 (PROSITE); IPR016090 (G3DSA:1.20.90.GENE3D); tmhmm (TMHMM) | |
| 105 | lib3_tri_Filter15_IDBA_contig_2224_1 | 36 | low-density lipoprotein receptor-related protein 6 | 242 | 3.02E-39 | 52.30% | 18 | P:anatomical structure morphogenesis; P:embryo development; P:multicellular organismal development; P:regulation of biological process; F:receptor activity; P:biological_process; P:growth; P:cellular component organization; P:cell differentiation; C:organelle; C:intracellular; P:cell proliferation; P:cell death; C:plasma membrane; P:transport; F:protein binding; ; C:cytoplasm | IPR000033 (SMART); IPR011042 (G3DSA:2.120.10.GENE3D); PTHR10529 (PANTHER), SSF63825 (SUPERFAMILY) | |
| 106 | lib3_tri_Filter15_IDBA_contig_2226_2 | 98 | acyl- -binding protein | 241 | 1.92E-34 | 80.70% | 1 | F:lipid binding | IPR000582 (PRINTS); IPR014352 (G3DSA:1.20.80.GENE3D); PTHR23310 (PANTHER) | |
| 107 | lib3_tri_Filter15_IDBA_contig_2242_2 | 58 | ---NA--- | 240 | | | 0 | - | IPR008160 (PFAM); PTHR24023 (PANTHER) | |
| 108 | lib3_tri_Filter15_IDBA_contig_2244_3 | 62 | serine carboxypeptidase-like 51 | 240 | 6.41E-18 | 59.30% | 5 | P:proteolysis; F:serine-type carboxypeptidase activity; P:response to endoplasmic reticulum stress; P:systemic acquired resistance; F:carboxypeptidase activity | IPR001563 (PANTHER); G3DSA:3.40.50.1820 (GENE3D), PTHR11802:SF3 (PANTHER), SSF53474 (SUPERFAMILY) | |

| | | | | | | | | | | |
|---|---|---|---|---|---|---|---|---|---|---|
| 109 | lib3_tri_Filter15_IDBA_contig_2248_1 | 333 | neuronal acetylcholine receptor subunit alpha-10 | 239 | 2.41E-18 | 48.50% | 16 | F:acetylcholine-activated cation-selective channel activity; F:ion channel activity; C:integral to membrane; C:membrane; C:synapse; P:ion transport; C:cell junction; P:transport; F:extracellular ligand-gated ion channel activity; F:receptor activity; C:postsynaptic membrane; C:plasma membrane; P:inner ear morphogenesis; P:synaptic transmission, cholinergic; P:detection of mechanical stimulus involved in sensory perception of sound; P:elevation of cytosolic calcium ion concentration | IPR006201 (PANTHER); IPR006202 (G3DSA:2.70.170.GENE3D); PTHR18945:SF66 (PANTHER), tmhmm (TMHMM) | |
| 110 | lib3_tri_Filter15_IDBA_contig_229_5 | 3839 | glyceraldehyde-3-phosphate dehydrogenase | 637 | 0 | 85.15% | 15 | P:cell death; C:cytosol; F:nucleotide binding; F:cytoskeletal protein binding; P:metabolic process; C:cytoskeleton; P:regulation of biological process; P:cytoskeleton organization; P:cellular component organization; F:catalytic activity; P:carbohydrate metabolic process; P:generation of precursor metabolites and energy; P:catabolic process; P:biosynthetic process; C:nucleus | IPR006424 (TIGRFAMs); IPR016040 (G3DSA:3.40.50.GENE3D); IPR020828 (PFAM); IPR020829 (PFAM); IPR020830 (PROSITE); IPR020831 (PRINTS); G3DSA:3.30.360.10 (GENE3D), SSF51735 (SUPERFAMILY), SSF55347 (SUPERFAMILY) | |
| 111 | lib3_tri_Filter15_IDBA_contig_2312_1 | 45 | cysteine-rich secretory protein mr30 | 236 | 9.84E-34 | 51.15% | 2 | P:behavior; P:response to external stimulus | IPR001283 (PANTHER); IPR014044 (G3DSA:3.40.33.GENE3D); PTHR10334:SF13 (PANTHER) | |
| 112 | lib3_tri_Filter15_IDBA_contig_2334_4 | 31 | masquerade-like serine proteinase homologue | 234 | 5.30E-13 | 70.35% | 1 | F:peptidase activity | IPR001254 (PFAM); IPR009003 (SUPERFAMILY); G3DSA:2.40.10.10 (GENE3D), PTHR24268 (PANTHER) | |
| 113 | lib3_tri_Filter15_IDBA_contig_2346_2 | 84 | matrix metalloproteinase isoform j | 303 | 2.58E-81 | 65.35% | 11 | P:cellular component organization; P:anatomical structure morphogenesis; P:multicellular organismal development; C:cell; F:binding; P:cell death; F:peptidase activity; P:biological_process; P:growth; P:response to external stimulus; P:response to stress | IPR001818 (PFAM); IPR002477 (G3DSA:1.10.101.GENE3D); IPR006026 (SMART); IPR021158 (PROSITE); IPR021190 (PRINTS); IPR024079 (G3DSA:3.40.390.GENE3D); PTHR10201 (PANTHER), SignalP-NN(euk) (SIGNALP), SSF55486 (SUPERFAMILY) | Yes |
| 114 | lib3_tri_Filter15_IDBA_contig_2347_3 | 75 | myosin heavy chain | 234 | 1.07E-58 | 79.50% | 7 | C:cytoskeleton; C:protein complex; F:nucleotide binding; C:cytoplasm; F:actin binding; F:motor activity; F:protein binding | IPR002928 (PFAM); PTHR13140 (PANTHER), PTHR13140:SF106 (PANTHER) | |
| 115 | lib3_tri_Filter15_IDBA_contig_2365_1 | 1131 | protein acr-11 | 411 | 4.68E-30 | 50.05% | 14 | F:acetylcholine-activated cation-selective channel activity; F:ion channel activity; C:integral to membrane; C:membrane; C:synapse; P:ion transport; C:cell junction; P:transport; F:extracellular ligand-gated ion channel activity; F:receptor activity; C:postsynaptic membrane; C:plasma membrane; P:synaptic transmission; F:acetylcholine receptor activity | IPR006201 (PANTHER); IPR006202 (G3DSA:2.70.170.GENE3D); PTHR18945:SF89 (PANTHER) | |
| 116 | lib3_tri_Filter15_IDBA_contig_2421_0 | 45 | ---NA--- | 229 | | | 0 | - | IPR008160 (PFAM); PTHR24023 (PANTHER) | |
| 117 | lib3_tri_Filter15_IDBA_contig_2421_5 | 45 | ---NA--- | 228 | | | 0 | - | no IPS match | |
| 118 | lib3_tri_Filter15_IDBA_contig_2462_2 | 40 | hypothetical protein CAPTEDRAFT_52911, partial | 226 | 6.53E-10 | 53.00% | 0 | - | IPR003598 (SMART); IPR007110 (PROFILE); IPR013098 (PFAM); IPR013783 (G3DSA:2.60.40.GENE3D); PTHR10489 (PANTHER), tmhmm (TMHMM), SSF48726 (SUPERFAMILY) | |

| | | | | | | | | | | |
|---|---|---|---|---|---|---|---|---|---|---|
| 119 | lib3_tri_Filter15_IDBA_contig_2478_4 | 42 | nop56 protein | 226 | 1.31E-74 | 93.85% | 8 | C:cytoplasm; C:nucleolus; F:RNA binding; F:protein binding; P:nucleobase-containing compound metabolic process; P:multicellular organismal development; P:cell death; C:intracellular | IPR002687 (PFAM); PTHR10894 (PANTHER), SSF89124 (SUPERFAMILY) | |
| 120 | lib3_tri_Filter15_IDBA_contig_2526_0 | 122 | retinoid-inducible serine carboxypeptidase-like | 322 | 3.81E-23 | 45.05% | 1 | F:hydrolase activity | IPR001563 (PANTHER); G3DSA:3.40.50.1820 (GENE3D), PTHR11802:SF3 (PANTHER), SSF53474 (SUPERFAMILY) | |
| 121 | lib3_tri_Filter15_IDBA_contig_2528_1 | 70 | ---NA--- | 222 | | | 0 | - | IPR008160 (PFAM); PTHR24023 (PANTHER) | |
| 122 | lib3_tri_Filter15_IDBA_contig_2548_4 | 47 | fibrinogen c domain-containing protein 1-like | 221 | 2.68E-34 | 60.55% | 2 | P:biological_process; F:carbohydrate binding | IPR002181 (PFAM); IPR014715 (G3DSA:4.10.530.GENE3D); IPR014716 (G3DSA:3.90.215.GENE3D); PTHR19143 (PANTHER) | |
| 123 | lib3_tri_Filter15_IDBA_contig_2567_5 | 54 | retinoid-inducible serine carboxypeptidase-like | 219 | 8.38E-38 | 62.10% | 1 | F:hydrolase activity | IPR001563 (PRINTS); G3DSA:3.40.50.1820 (GENE3D), PTHR11802:SF3 (PANTHER), SignalP-NN(euk) (SIGNALP), tmhmm (TMHMM), SSF53474 (SUPERFAMILY) | Yes |
| 124 | lib3_tri_Filter15_IDBA_contig_2579_0 | 1046 | protein | 271 | 1.76E-27 | 48.95% | 5 | P:proteolysis; F:peptidase activity; F:peptidase inhibitor activity; F:serine-type endopeptidase inhibitor activity; P:negative regulation of peptidase activity | IPR002350 (PFAM); G3DSA:1.10.1890.10 (GENE3D), G3DSA:3.30.60.30 (GENE3D), PTHR10913 (PANTHER), PTHR10913:SF21 (PANTHER), SignalP-NN(euk) (SIGNALP), tmhmm (TMHMM), SSF100895 (SUPERFAMILY) | Yes |
| 125 | lib3_tri_Filter15_IDBA_contig_2590_3 | 160 | ---NA--- | 338 | | | 0 | - | IPR003599 (SMART); IPR007110 (PROFILE); IPR013098 (PFAM); IPR013783 (G3DSA:2.60.40.GENE3D); PTHR10489 (PANTHER), SSF48726 (SUPERFAMILY) | |
| 126 | lib3_tri_Filter15_IDBA_contig_2613_0 | 44 | nerve growth factor receptor-like | 218 | 6.83E-11 | 54.00% | 9 | F:transmembrane signaling receptor activity; C:integral to membrane; C:membrane; F:G-protein coupled receptor activity; F:zinc ion binding; P:cell surface receptor signaling pathway; F:receptor activity; F:metal ion binding; P:G-protein coupled receptor signaling pathway | IPR001368 (PFAM); G3DSA:2.10.50.10 (GENE3D), PTHR23097 (PANTHER), SignalP-NN(euk) (SIGNALP), tmhmm (TMHMM), SSF57586 (SUPERFAMILY) | |
| 127 | lib3_tri_Filter15_IDBA_contig_262_3 | 4097 | soma ferritin | 180 | 2.19E-69 | 91.65% | 6 | C:cytoplasm; P:cellular homeostasis; F:binding; F:catalytic activity; P:ion transport; P:metabolic process | IPR001519 (PANTHER); IPR008331 (PFAM); IPR009040 (PROFILE); IPR009078 (SUPERFAMILY); IPR012347 (G3DSA:1.20.1260.GENE3D); IPR014034 (PROSITE) | |
| 128 | lib3_tri_Filter15_IDBA_contig_2640_0 | 437 | transmembrane protease serine 3 | 250 | 1.21E-29 | 52.10% | 3 | F:peptidase activity; C:endoplasmic reticulum; C:cell | IPR001254 (PFAM); IPR001314 (PRINTS); IPR009003 (SUPERFAMILY); IPR018114 (PROSITE); G3DSA:2.40.10.10 (GENE3D), PTHR24265 (PANTHER) | Yes |
| 129 | lib3_tri_Filter15_IDBA_contig_265_4 | 36 | ferric-chelate reductase 1 precursor | 168 | 2.99E-12 | 46.91% | 11 | P:oxidation-reduction process; F:oxidoreductase activity; C:integral to membrane; C:membrane; P:electron transport chain; P:transport; F:molecular_function; P:defense response to protozoan; C:extracellular region; P:innate immune response; P:defense response to bacterium | IPR002861 (PFAM); PTHR23130 (PANTHER), PTHR23130:SF6 (PANTHER), SignalP-NN(euk) (SIGNALP) | |
| 130 | lib3_tri_Filter15_IDBA_contig_266_3 | 66 | filaggrin-2-like isoform x12 | 200 | 1.89E-10 | 45.20% | 0 | - | IPR002861 (PFAM); PTHR23130 (PANTHER), PTHR23130:SF6 (PANTHER), SignalP-NN(euk) (SIGNALP) | |
| 131 | lib3_tri_Filter15_IDBA_contig_2699_5 | 46 | ---NA--- | 213 | | | 0 | - | IPR008160 (PFAM); PTHR24023 (PANTHER) | |

| | | | | | | | | | | |
|---|---|---|---|---|---|---|---|---|---|---|
| 132 | lib3_tri_Filter15_IDBA_contig_2710_2 | 922 | protease inhibitor epi11 | 265 | 5.12E-29 | 44.25% | 2 | P:proteolysis; F:peptidase activity | IPR002350 (PFAM); G3DSA:1.10.1890.10 (GENE3D), G3DSA:3.30.60.30 (GENE3D), PTHR21312 (PANTHER), PTHR21312:SF1 (PANTHER), SignalP-NN(euk) (SIGNALP), SSF100895 (SUPERFAMILY) | Yes |
| 133 | lib3_tri_Filter15_IDBA_contig_271_3 | 1543 | hypothetical protein KGM_22095 | 347 | 1.05E-10 | 49.47% | 0 | - | IPR000716 (G3DSA:4.10.800.GENE3D); IPR022339 (PANTHER) | |
| 134 | lib3_tri_Filter15_IDBA_contig_2734_0 | 107 | ---NA--- | 306 | | | 0 | - | IPR008160 (PFAM); PTHR24023 (PANTHER), tmhmm (TMHMM) | |
| 135 | lib3_tri_Filter15_IDBA_contig_2734_3 | 107 | ---NA--- | 306 | | | 0 | - | tmhmm (TMHMM) | |
| 136 | lib3_tri_Filter15_IDBA_contig_2751_1 | 70 | ---NA--- | 211 | | | 0 | - | no IPS match | |
| 137 | lib3_tri_Filter15_IDBA_contig_2751_4 | 70 | ---NA--- | 211 | | | 0 | - | IPR008160 (PFAM); PTHR24023 (PANTHER) | |
| 138 | lib3_tri_Filter15_IDBA_contig_280_3 | 992 | transmembrane protease serine 3 | 381 | 1.28E-39 | 51.95% | 3 | F:peptidase activity; C:endoplasmic reticulum; C:cell | IPR001254 (PFAM); IPR001314 (PRINTS); IPR009003 (SUPERFAMILY); IPR018114 (PROSITE); G3DSA:2.40.10.10 (GENE3D), PTHR24265 (PANTHER) | |
| 139 | lib3_tri_Filter15_IDBA_contig_2836_4 | 198 | nadh dehydrogenase isoform 1 | 230 | 5.16E-40 | 94.65% | 6 | P:generation of precursor metabolites and energy; P:biosynthetic process; P:lipid metabolic process; F:transporter activity; F:binding; C:mitochondrion | IPR003231 (PRODOM); IPR009081 (G3DSA:1.10.1200.GENE3D); PTHR20863 (PANTHER), PTHR20863:SF5 (PANTHER), tmhmm (TMHMM) | |
| 140 | lib3_tri_Filter15_IDBA_contig_2852_5 | 495 | ---NA--- | 207 | | | 0 | - | IPR008160 (PFAM); PTHR24023 (PANTHER) | |
| 141 | lib3_tri_Filter15_IDBA_contig_2860_1 | 37 | cathepsin d | 207 | 7.76E-39 | 84.25% | 9 | C:lysosome; C:cytoskeleton; C:protein complex; P:cell death; P:anatomical structure morphogenesis; P:multicellular organismal development; P:protein metabolic process; P:catabolic process; F:peptidase activity | IPR001461 (PANTHER); IPR021109 (G3DSA:2.40.70.GENE3D); PTHR13683:SF84 (PANTHER), tmhmm (TMHMM) | |
| 142 | lib3_tri_Filter15_IDBA_contig_2866_4 | 27 | ---NA--- | 207 | | | 0 | - | IPR000884 (PFAM); G3DSA:2.20.100.10 (GENE3D), PTHR13723 (PANTHER), PTHR13723:SF9 (PANTHER) | |
| 143 | lib3_tri_Filter15_IDBA_contig_2868_2 | 39 | cubilin precursor | 206 | 1.58E-17 | 52.55% | 11 | P:protein transport; C:cell; C:cytoplasmic membrane-bounded vesicle; C:endosome; F:protein binding; C:plasma membrane; P:transport; P:cellular component organization; F:catalytic activity; C:Golgi apparatus; C:endoplasmic reticulum | IPR000859 (G3DSA:2.60.120.GENE3D); PTHR10127 (PANTHER) | |
| 144 | lib3_tri_Filter15_IDBA_contig_2872_3 | 24 | leucine-rich transmembrane | 207 | 4.97E-35 | 53.20% | 4 | C:integral to membrane; F:hydrolase activity; F:phosphoprotein phosphatase activity; F:carbohydrate binding | IPR001611 (PFAM); IPR003591 (SMART); IPR026906 (PFAM); G3DSA:3.80.10.10 (GENE3D), PTHR24367 (PANTHER), SSF52058 (SUPERFAMILY) | |
| 145 | lib3_tri_Filter15_IDBA_contig_2874_0 | 42 | ---NA--- | 207 | | | 0 | - | no IPS match | |
| 146 | lib3_tri_Filter15_IDBA_contig_2874_5 | 42 | ---NA--- | 206 | | | 0 | - | IPR008160 (PFAM); PTHR24023 (PANTHER) | |
| 147 | lib3_tri_Filter15_IDBA_contig_2903_5 | 32 | peptidyl-dipeptidase a | 205 | 1.84E-34 | 71.10% | 1 | F:peptidase activity | IPR001548 (PANTHER); PTHR10514:SF16 (PANTHER), SSF55486 (SUPERFAMILY) | |

| | | | | | | | | | |
|---|---|---|---|---|---|---|---|---|---|
| 148 | lib3_tri_Filter15_IDBA_contig_293_3 | 5295 | y chain e2~ubiquitin-hect | 118 | 7.24E-29 | 100.00% | 27 | P:cell cycle; P:signal transduction; F:structural molecule activity; P:biological_process; P:response to stress; P:DNA metabolic process; P:reproduction; P:viral reproduction; P:translation; F:binding; C:nucleoplasm; P:protein metabolic process; P:catabolic process; P:regulation of biological process; P:cell death; F:protein binding; P:cellular component organization; C:plasma membrane; P:protein transport; ; C:endosome; C:cytoplasmic membrane-bounded vesicle; P:cellular protein modification process; P:nucleobase-containing compound metabolic process; C:ribosome; C:cytosol; P:transport | IPR000626 (PFAM); IPR019954 (PROSITE); IPR019955 (PROFILE); IPR019956 (PRINTS); G3DSA:3.10.20.90 (GENE3D), PTHR10666 (PANTHER), SSF54236 (SUPERFAMILY) |
| 149 | lib3_tri_Filter15_IDBA_contig_2_4 | 1994 | thioester-containing protein | 1751 | 2.46E-179 | 55.25% | 4 | P:negative regulation of endopeptidase activity; C:extracellular space; C:extracellular region; F:endopeptidase inhibitor activity | IPR001599 (PFAM); IPR002890 (PFAM); IPR008930 (SUPERFAMILY); IPR009048 (G3DSA:2.60.40.GENE3D); IPR011625 (PFAM); IPR011626 (PFAM); IPR019565 (PFAM); IPR019742 (PROSITE); G3DSA:1.50.10.20 (GENE3D), PTHR11412 (PANTHER), tmhmm (TMHMM) |
| 150 | lib3_tri_Filter15_IDBA_contig_3006_2 | 38 | annexin a7 | 199 | 7.18E-76 | 75.05% | 5 | P:biological_process; F:binding; P:cellular homeostasis; C:cell; C:nucleus | IPR001464 (PRINTS); IPR018252 (PROSITE); IPR018502 (G3DSA:1.10.220.GENE3D) |
| 151 | lib3_tri_Filter15_IDBA_contig_300_5 | 496 | heat shock protein 70 | 721 | 0 | 95.60% | 2 | F:nucleotide binding; P:response to stress | IPR013126 (PRINTS); IPR018181 (PROSITE); G3DSA:1.20.1270.10 (GENE3D), G3DSA:2.60.34.10 (GENE3D), G3DSA:3.30.30.30 (GENE3D), G3DSA:3.30.420.40 (GENE3D), G3DSA:3.90.640.10 (GENE3D), PTHR19375 (PANTHER), SignalP-NN(euk) (SIGNALP), SSF100920 (SUPERFAMILY), SSF100934 (SUPERFAMILY), SSF53067 (SUPERFAMILY) |
| 152 | lib3_tri_Filter15_IDBA_contig_3050_0 | 30 | ---NA--- | 198 | | | 0 | - | IPR008160 (PFAM); PTHR24023 (PANTHER) |
| 153 | lib3_tri_Filter15_IDBA_contig_3050_3 | 30 | ---NA--- | 198 | | | 0 | - | no IPS match |
| 154 | lib3_tri_Filter15_IDBA_contig_3072_4 | 72 | leukocyte elastase inhibitor | 197 | 1.73E-07 | 80.00% | 0 | - | IPR000215 (PANTHER); IPR023795 (PROSITE); IPR023796 (PFAM); G3DSA:2.30.39.10 (GENE3D), tmhmm (TMHMM) |
| 155 | lib3_tri_Filter15_IDBA_contig_3111_1 | 57 | ---NA--- | 196 | | | 0 | - | IPR008160 (PFAM); PTHR24023 (PANTHER) |
| 156 | lib3_tri_Filter15_IDBA_contig_3111_5 | 57 | ---NA--- | 195 | | | 0 | - | no IPS match |
| 157 | lib3_tri_Filter15_IDBA_contig_311_3 | 2789 | annexin a7-like | 872 | 6.54E-108 | 70.55% | 1 | F:binding | IPR001464 (PRINTS); IPR002392 (PRINTS); IPR018252 (PROSITE); IPR018502 (G3DSA:1.10.220.GENE3D); tmhmm (TMHMM) |
| 158 | lib3_tri_Filter15_IDBA_contig_3173_1 | 17 | cathepsin b-like | 193 | 9.82E-48 | 85.35% | 4 | P:protein metabolic process; P:catabolic process; P:biological_process; F:peptidase activity | IPR000668 (PFAM); IPR013128 (PANTHER); IPR015643 (PTHR12411:PANTHER); IPR025660 (PROSITE); IPR025661 (PROSITE); G3DSA:3.90.70.10 (GENE3D), SSF54001 (SUPERFAMILY) |
| 159 | lib3_tri_Filter15_IDBA_contig_3216_5 | 28 | phospholipid-hydroperoxide glutathione peroxidase | 191 | 1.59E-83 | 79.05% | 4 | F:antioxidant activity; F:catalytic activity; P:response to stress; P:metabolic process | IPR000889 (PRINTS); IPR012336 (G3DSA:3.40.30.GENE3D); PTHR11592:SF0 (PANTHER) |

| | | | | | | | | | | |
|---|---|---|---|---|---|---|---|---|---|---|
| 160 | lib3_tri_Filter15_IDBA_contig_3257_0 | 20 | ferric-chelate reductase 1-like protein | 190 | 2.98E-25 | 46.50% | 8 | F:molecular_function; P:defense response to protozoan; C:extracellular region; P:innate immune response; P:defense response to bacterium; C:integral to membrane; P:proteolysis; F:peptidase activity | IPR002861 (PFAM); PTHR23130 (PANTHER), PTHR23130:SF6 (PANTHER), SignalP-NN(euk) (SIGNALP) | Yes |
| 161 | lib3_tri_Filter15_IDBA_contig_332_5 | 2500 | 14-3-3 zeta | 1360 | 2.63E-116 | 89.25% | 22 | P:signal transduction; P:anatomical structure morphogenesis; P:cell differentiation; P:multicellular organismal development; P:reproduction; P:cytoskeleton organization; P:protein metabolic process; P:behavior; P:cell proliferation; P:metabolic process; P:regulation of biological process; F:protein binding; C:extracellular region; F:enzyme regulator activity; P:biological_process; C:cytoskeleton; C:protein complex; P:embryo development; P:cell cycle; C:nucleus; P:biosynthetic process; | IPR000308 (PRINTS); IPR023409 (PROSITE); IPR023410 (G3DSA:1.20.190.GENE3D); PTHR18860:SF0 (PANTHER), tmhmm (TMHMM) | |
| 162 | lib3_tri_Filter15_IDBA_contig_3349_3 | 108 | gelsolin-like protein 2-like | 343 | 1.08E-158 | 74.00% | 6 | F:calcium ion binding; P:biological_process; P:cytoskeleton organization; F:actin binding; C:intracellular; P:regulation of biological process | IPR007122 (PRINTS); IPR007123 (PFAM); G3DSA:3.40.20.10 (GENE3D), PTHR11977:SF19 (PANTHER), SSF55753 (SUPERFAMILY) | |
| 163 | lib3_tri_Filter15_IDBA_contig_3351_0 | 12811 | protease inhibitor epi11 | 678 | 1.15E-62 | 46.10% | 2 | P:proteolysis; F:peptidase activity | IPR002350 (PFAM); G3DSA:1.10.1890.10 (GENE3D), G3DSA:3.30.60.30 (GENE3D), PTHR10913 (PANTHER), PTHR10913:SF11 (PANTHER), SignalP-NN(euk) (SIGNALP), SSF100895 (SUPERFAMILY) | Yes |
| 164 | lib3_tri_Filter15_IDBA_contig_3353_0 | 1179 | protease inhibitor epi11 | 186 | 2.18E-28 | 48.80% | 5 | P:proteolysis; F:peptidase activity; F:peptidase inhibitor activity; F:serine-type endopeptidase inhibitor activity; P:negative regulation of peptidase activity | IPR002350 (PFAM); G3DSA:3.30.60.30 (GENE3D), PTHR10913 (PANTHER), PTHR10913:SF21 (PANTHER), SSF100895 (SUPERFAMILY) | |
| 165 | lib3_tri_Filter15_IDBA_contig_3392_5 | 36 | phosphoglycerate kinase | 185 | 1.18E-56 | 85.60% | 5 | P:carbohydrate metabolic process; P:generation of precursor metabolites and energy; P:catabolic process; P:metabolic process; F:kinase activity | IPR001576 (PRINTS); IPR015901 (G3DSA:3.40.50.GENE3D) | |
| 166 | lib3_tri_Filter15_IDBA_contig_3399_5 | 29 | cathepsin d | 185 | 1.71E-91 | 79.50% | 3 | P:protein metabolic process; P:catabolic process; F:peptidase activity | IPR001461 (PRINTS); IPR001969 (PROSITE); IPR021109 (G3DSA:2.40.70.GENE3D); PTHR13683:SF84 (PANTHER) | |
| 167 | lib3_tri_Filter15_IDBA_contig_3425_1 | 48 | deleted in malignant brain tumors 1 | 184 | 9.40E-29 | 63.35% | 3 | F:scavenger receptor activity; C:membrane; F:receptor activity | IPR001190 (PRINTS); IPR017448 (SMART); G3DSA:3.10.250.10 (GENE3D), PTHR19331 (PANTHER) | |
| 168 | lib3_tri_Filter15_IDBA_contig_346_4 | 2829 | ferritin | 150 | 6.96E-65 | 82.45% | 6 | C:cytoplasm; P:cellular homeostasis; F:binding; F:catalytic activity; P:ion transport; P:metabolic process | IPR001519 (PANTHER); IPR008331 (PFAM); IPR009040 (PROFILE); IPR009078 (SUPERFAMILY); IPR012347 (G3DSA:1.20.1260.GENE3D) | |
| 169 | lib3_tri_Filter15_IDBA_contig_3492_4 | 78 | collagen alpha-1 chain | 182 | 5.28E-07 | 55.00% | 0 | - | IPR002035 (G3DSA:3.40.50.GENE3D); PTHR22992 (PANTHER), SSF53300 (SUPERFAMILY) | |
| 170 | lib3_tri_Filter15_IDBA_contig_3495_0 | 25 | cathepsin d | 182 | 1.77E-85 | 78.55% | 9 | C:lysosome; C:cytoskeleton; C:protein complex; P:cell death; P:anatomical structure morphogenesis; P:multicellular organismal development; P:protein metabolic process; P:catabolic process; F:peptidase activity | IPR001461 (PANTHER); IPR001969 (PROSITE); IPR012848 (PFAM); IPR021109 (G3DSA:2.40.70.GENE3D); PTHR13683:SF84 (PANTHER), SignalP-NN(euk) (SIGNALP), tmhmm (TMHMM) | |

| | | | | | | | | | | |
|---|---|---|---|---|---|---|---|---|---|---|
| 171 | lib3_tri_Filter15_IDBA_contig_34_5 | 1990 | a chain solution structure of component iv glycera dibranchiata monomeric hemoglobin-co | 314 | 1.13E-38 | 54.35% | 1 | F:binding | IPR000971 (PFAM); IPR009050 (SUPERFAMILY); IPR012292 (G3DSA:1.10.490.GENE3D); IPR013316 (PRINTS); PTHR22924 (PANTHER) | |
| 172 | lib3_tri_Filter15_IDBA_contig_3579_3 | 29 | titin | 180 | 2.01E-66 | 52.80% | 3 | P:lipid transport; F:nucleic acid binding; F:lipid transporter activity | IPR007110 (PROFILE); IPR013098 (PFAM); IPR013783 (G3DSA:2.60.40.GENE3D); IPR020675 (PANTHER); SSF48726 (SUPERFAMILY) | |
| 173 | lib3_tri_Filter15_IDBA_contig_3611_4 | 62 | hypothetical protein CAPTEDRAFT_223820 | 179 | 3.24E-25 | 57.00% | 0 | - | IPR021712 (PFAM); PD936484 (PRODOM), SignalP-NN(euk) (SIGNALP) | |
| 174 | lib3_tri_Filter15_IDBA_contig_3680_3 | 26 | twitchin-like protein | 177 | 1.95E-92 | 85.35% | 3 | F:nucleotide binding; P:cellular protein modification process; F:protein kinase activity | IPR000719 (PFAM); IPR002290 (SMART); IPR008271 (PROSITE); IPR011009 (SUPERFAMILY); IPR020675 (PANTHER); G3DSA:1.10.510.10 (GENE3D) | |
| 175 | lib3_tri_Filter15_IDBA_contig_3700_2 | 37 | ---NA--- | 176 | | | 0 | - | IPR007110 (PROFILE); IPR013783 (G3DSA:2.60.40.GENE3D); PTHR12035 (PANTHER), PTHR12035:SF5 (PANTHER), PF13895 (PFAM), SSF48726 (SUPERFAMILY) | |
| 176 | lib3_tri_Filter15_IDBA_contig_3729_2 | 278 | ---NA--- | 326 | | | 0 | - | no IPS match | |
| 177 | lib3_tri_Filter15_IDBA_contig_3769_0 | 44 | ---NA--- | 175 | | | 0 | - | no IPS match | |
| 178 | lib3_tri_Filter15_IDBA_contig_3769_5 | 44 | ---NA--- | 174 | | | 0 | - | IPR008160 (PFAM); PTHR24023 (PANTHER) | |
| 179 | lib3_tri_Filter15_IDBA_contig_3815_5 | 37 | isoform d | 173 | 2.31E-25 | 57.10% | 13 | P:multicellular organismal development; C:cytoskeleton; C:protein complex; C:cytoplasm; F:structural molecule activity; P:anatomical structure morphogenesis; P:cell differentiation; P:cytoskeleton organization; C:nuclear chromosome; P:organelle organization; P:cell cycle; F:actin binding; P:biological_process | IPR003599 (SMART); IPR007110 (PROFILE); IPR013098 (PFAM); IPR013783 (G3DSA:2.60.40.GENE3D); PTHR10489 (PANTHER), SSF48726 (SUPERFAMILY) | |
| 180 | lib3_tri_Filter15_IDBA_contig_3853_1 | 28 | ---NA--- | 172 | | | 0 | - | no IPS match | |
| 181 | lib3_tri_Filter15_IDBA_contig_3853_4 | 28 | ---NA--- | 172 | | | 0 | - | IPR008160 (PFAM); PTHR24023 (PANTHER), PTHR24023:SF164 (PANTHER) | |
| 182 | lib3_tri_Filter15_IDBA_contig_3926_4 | 3555 | ---NA--- | 513 | | | 0 | - | G3DSA:2.10.60.10 (GENE3D), SignalP-NN(euk) (SIGNALP), tmhmm (TMHMM), SSF57302 (SUPERFAMILY) | Yes |
| 183 | lib3_tri_Filter15_IDBA_contig_3943_0 | 114 | aldehyde mitochondrial isoform x1 | 299 | 1.61E-161 | 82.00% | 3 | C:mitochondrion; F:catalytic activity; P:metabolic process | IPR015590 (PFAM); IPR016160 (PROSITE); IPR016161 (SUPERFAMILY); IPR016162 (G3DSA:3.40.605.GENE3D); IPR016163 (G3DSA:3.40.309.GENE3D); PTHR11699 (PANTHER), PTHR11699:SF46 (PANTHER) | |
| 184 | lib3_tri_Filter15_IDBA_contig_3948_3 | 38 | ---NA--- | 170 | | | 0 | - | IPR008160 (PFAM); PTHR24023 (PANTHER) | |
| 185 | lib3_tri_Filter15_IDBA_contig_397_4 | 538 | epididymal secretory protein e1 precursor | 659 | 4.22E-22 | 66.00% | 1 | C:extracellular region | IPR003172 (G3DSA:2.60.40.GENE3D); IPR014756 (SUPERFAMILY); PTHR11306 (PANTHER), tmhmm (TMHMM) | |
| 186 | lib3_tri_Filter15_IDBA_contig_4025_3 | 24 | 3-oxoacyl-acyl-carrier-protein reductase | 168 | 2.89E-44 | 63.65% | 4 | P:oxidation-reduction process; F:oxidoreductase activity; P:metabolic process; F:nucleotide binding | IPR002198 (PRINTS); IPR002347 (PRINTS); IPR016040 (G3DSA:3.40.50.GENE3D); PTHR24322 (PANTHER), PTHR24322:SF0 (PANTHER), SSF51735 (SUPERFAMILY) | |
| 187 | lib3_tri_Filter15_IDBA_contig_4030_0 | 26 | von willebrand factor type egf and pentraxin domain-containing protein 1 | 168 | 4.39E-21 | 48.30% | 2 | F:carbohydrate binding; F:calcium ion binding | IPR000436 (PFAM); IPR011641 (PFAM); G3DSA:2.10.70.10 (GENE3D), PTHR19325 (PANTHER) | |

| | | | | | | | | | | |
|---|---|---|---|---|---|---|---|---|---|---|
| 188 | lib3_tri_Filter15_IDBA_contig_4055_0 | 22 | isoform i | 167 | 1.18E-58 | 73.45% | 8 | P:multicellular organismal development; P:cellular protein modification process; C:cytoplasm; F:structural molecule activity; P:anatomical structure morphogenesis; P:cell differentiation; P:cytoskeleton organization; F:protein kinase activity | IPR003599 (SMART); IPR007110 (PROFILE); IPR013098 (PFAM); IPR013783 (G3DSA:2.60.40.GENE3D); SSF48726 (SUPERFAMILY) | |
| 189 | lib3_tri_Filter15_IDBA_contig_4071_4 | 24 | collagen alpha-5 chain | 166 | 1.71E-42 | 62.15% | 0 | - | IPR002035 (G3DSA:3.40.50.GENE3D); PTHR22992 (PANTHER), SSF53300 (SUPERFAMILY) | |
| 190 | lib3_tri_Filter15_IDBA_contig_4178_0 | 36 | paramyosin | 164 | 3.10E-42 | 73.75% | 4 | C:cytoplasm; C:cytoskeleton; C:protein complex; F:motor activity | IPR002928 (PFAM); PTHR13140 (PANTHER), PTHR13140:SF106 (PANTHER), SignalP-NN(euk) (SIGNALP) | Yes |
| 191 | lib3_tri_Filter15_IDBA_contig_4180_1 | 16 | epidermal retinol dehydrogenase 2-like | 164 | 4.21E-47 | 70.55% | 5 | F:oxidoreductase activity; F:nucleotide binding; P:oxidation-reduction process; P:metabolic process; C:cellular_component | IPR002198 (PFAM); IPR002347 (PRINTS); IPR016040 (G3DSA:3.40.50.GENE3D); PTHR24316 (PANTHER), PTHR24316:SF1 (PANTHER), SignalP-NN(euk) (SIGNALP), tmhmm (TMHMM), SSF51735 (SUPERFAMILY) | |
| 192 | lib3_tri_Filter15_IDBA_contig_419_4 | 2161 | ferritin | 115 | 2.95E-62 | 86.90% | 6 | C:cytoplasm; P:cellular homeostasis; F:binding; F:catalytic activity; P:ion transport; P:metabolic process | IPR001519 (PANTHER); IPR008331 (PFAM); IPR009040 (PROFILE); IPR009078 (SUPERFAMILY); IPR012347 (G3DSA:1.20.1260.GENE3D); IPR014034 (PROSITE) | |
| 193 | lib3_tri_Filter15_IDBA_contig_4294_2 | 15 | 15-hydroxyprostaglandin dehydrogenase | 161 | 6.06E-24 | 57.45% | 4 | P:oxidation-reduction process; F:oxidoreductase activity; P:metabolic process; F:nucleotide binding | IPR002198 (PFAM); IPR002347 (PRINTS); IPR016040 (G3DSA:3.40.50.GENE3D); PTHR24310 (PANTHER), PTHR24310:SF0 (PANTHER), SSF51735 (SUPERFAMILY) | |
| 194 | lib3_tri_Filter15_IDBA_contig_4328_1 | 43 | ---NA--- | 160 | | | 0 | - | IPR008160 (PFAM); PTHR24023 (PANTHER) | |
| 195 | lib3_tri_Filter15_IDBA_contig_4328_2 | 43 | ---NA--- | 160 | | | 0 | - | no IPS match | |
| 196 | lib3_tri_Filter15_IDBA_contig_4328_4 | 43 | ---NA--- | 160 | | | 0 | - | no IPS match | |
| 197 | lib3_tri_Filter15_IDBA_contig_4361_0 | 35 | low quality protein: titin | 160 | 1.86E-22 | 54.75% | 6 | F:nucleic acid binding; F:ATP binding; F:Rho guanyl-nucleotide exchange factor activity; F:protein tyrosine kinase activity; P:regulation of Rho protein signal transduction; C:intracellular | IPR003599 (SMART); IPR007110 (PROFILE); IPR013098 (PFAM); IPR013783 (G3DSA:2.60.40.GENE3D); PTHR10489 (PANTHER), PF13895 (PFAM), SSF48726 (SUPERFAMILY) | |
| 198 | lib3_tri_Filter15_IDBA_contig_4381_0 | 16 | endothelin-converting enzyme 1-like | 160 | 2.03E-19 | 58.70% | 8 | P:metabolic process; F:peptidase activity; F:binding; C:cell; C:cytoplasmic membrane-bounded vesicle; P:biological_process; P:protein metabolic process; P:catabolic process | IPR000718 (PANTHER); IPR008753 (PFAM); G3DSA:1.10.1380.10 (GENE3D), SignalP-NN(euk) (SIGNALP), tmhmm (TMHMM), SSF55486 (SUPERFAMILY) | |
| 199 | lib3_tri_Filter15_IDBA_contig_4382_1 | 18 | cre-unc-22 protein | 159 | 4.58E-54 | 60.85% | 6 | F:protein kinase activity; C:cytoplasm; P:multicellular organismal development; P:regulation of biological process; P:biological_process; P:growth | IPR003961 (PFAM); IPR013783 (G3DSA:2.60.40.GENE3D); PR00014 (PRINTS), PTHR10489 (PANTHER), SSF48726 (SUPERFAMILY) | |
| 200 | lib3_tri_Filter15_IDBA_contig_4416_0 | 107 | ---NA--- | 255 | | | 0 | - | IPR000562 (G3DSA:2.10.10.GENE3D); IPR013806 (SUPERFAMILY); SignalP-NN(euk) (SIGNALP) | Yes |
| 201 | lib3_tri_Filter15_IDBA_contig_4419_4 | 35 | type alpha partial | 159 | 2.98E-66 | 68.45% | 9 | P:anatomical structure morphogenesis; P:cellular component organization; P:cell differentiation; P:multicellular organismal development; P:biological_process; C:proteinaceous extracellular matrix; F:protein binding; P:embryo development; F:structural molecule activity | IPR000885 (PRODOM); PTHR24637 (PANTHER), PTHR24637:SF25 (PANTHER) | |

| | | | | | | | | | | |
|---|---|---|---|---|---|---|---|---|---|---|
| 202 | lib3_tri_Filter15_IDBA_contig_4474_2 | 16 | aael005317- partial | 157 | 5.85E-65 | 66.25% | 13 | P:multicellular organismal development; C:cytoskeleton; C:protein complex; C:cytoplasm; F:structural molecule activity; P:anatomical structure morphogenesis; P:cell differentiation; P:cytoskeleton organization; C:nuclear chromosome; P:organelle organization; P:cell cycle; F:actin binding; P:biological_process | IPR003598 (SMART); IPR003599 (SMART); IPR007110 (PROFILE); IPR013098 (PFAM); IPR013783 (G3DSA:2.60.40.GENE3D); IPR020675 (PANTHER); PTHR22964:SF3 (PANTHER), SSF48726 (SUPERFAMILY) | |
| 203 | lib3_tri_Filter15_IDBA_contig_4520_3 | 16 | heat shock | 157 | 2.49E-56 | 92.10% | 29 | C:cell; C:endoplasmic reticulum; C:protein complex; F:protein binding; P:signal transduction; P:anatomical structure morphogenesis; P:multicellular organismal development; P:nucleobase-containing compound metabolic process; P:catabolic process; P:response to external stimulus; P:response to stress; P:cellular protein modification process; P:regulation of biological process; F:enzyme regulator activity; P:protein metabolic process; P:cell death; P:response to biotic stimulus; C:nucleus; C:cytosol; P:transport; C:cytoplasm; F:binding; P:cell communication; P:metabolic process; F:nucleotide binding; F:hydrolase activity; C:cytoplasmic membrane-bounded vesicle; | IPR013126 (PRINTS); IPR018181 (PROSITE); G3DSA:3.30.30.30 (GENE3D), G3DSA:3.30.420.40 (GENE3D), PTHR19375 (PANTHER), SSF53067 (SUPERFAMILY) | Yes |
| 204 | lib3_tri_Filter15_IDBA_contig_4521_1 | 28 | nexilin isoform x7 | 157 | 1.80E-27 | 59.50% | 5 | P:regulation of biological process; F:structural molecule activity; P:cytoskeleton organization; C:cell; F:actin binding | IPR003598 (SMART); IPR003599 (SMART); IPR007110 (PROFILE); IPR013098 (PFAM); IPR013783 (G3DSA:2.60.40.GENE3D); IPR020675 (PANTHER); SSF48726 (SUPERFAMILY) | |
| 205 | lib3_tri_Filter15_IDBA_contig_4565_1 | 19 | protein | 156 | 7.02E-27 | 53.00% | 1 | F:carbohydrate binding | IPR000922 (PFAM); PTHR12011 (PANTHER), PTHR12011:SF4 (PANTHER) | |
| 206 | lib3_tri_Filter15_IDBA_contig_4570_2 | 21 | neurogenic locus notch homolog protein 1 | 155 | 5.20E-11 | 49.65% | 18 | P:cell differentiation; P:multicellular organismal development; P:regulation of biological process; P:cell proliferation; P:anatomical structure morphogenesis; P:signal transduction; P:embryo development; P:cellular component organization; C:organelle; C:intracellular; ; P:cell death; C:cytoplasm; P:cell communication; F:DNA binding; P:biological_process; P:reproduction; C:cell | IPR000742 (PFAM); IPR001881 (SMART); IPR013032 (PFAM); PR00010 (PRINTS), G3DSA:2.10.25.10 (GENE3D), PTHR24838 (PANTHER), PTHR24838:SF112 (PANTHER), SSF57196 (SUPERFAMILY) | |
| 207 | lib3_tri_Filter15_IDBA_contig_4574_0 | 15 | versican core | 156 | 2.51E-09 | 66.55% | 3 | C:proteinaceous extracellular matrix; C:organelle; C:cytoplasm | IPR000152 (PROSITE); IPR000742 (PFAM); IPR001881 (SMART); IPR013032 (PROSITE); IPR018097 (PROSITE); PR00010 (PRINTS), G3DSA:2.10.25.10 (GENE3D), PTHR24838 (PANTHER), tmhmm (TMHMM), SSF57196 (SUPERFAMILY) | |
| 208 | lib3_tri_Filter15_IDBA_contig_4633_4 | 632 | hypothetical protein AaeL_AAEL002257 | 379 | 2.05E-10 | 50.17% | 0 | - | IPR000716 (G3DSA:4.10.800.GENE3D); PTHR12352 (PANTHER), SignalP-NN(euk) (SIGNALP), tmhmm (TMHMM) | Yes |
| 209 | lib3_tri_Filter15_IDBA_contig_4634_4 | 572 | hypothetical protein AaeL_AAEL002257 | 386 | 3.16E-10 | 52.20% | 0 | - | IPR000716 (G3DSA:4.10.800.GENE3D); PTHR12352 (PANTHER), SignalP-NN(euk) (SIGNALP), tmhmm (TMHMM) | Yes |

| | | | | | | | | | | |
|---|---|---|---|---|---|---|---|---|---|---|
| 210 | lib3_tri_Filter15_IDBA_contig_4643_4 | 924 | a chain solution structure of component iv glycera dibranchiata monomeric hemoglobin-co | 343 | 3.43E-31 | 56.64% | 1 | F:binding | IPR000971 (PFAM); IPR009050 (SUPERFAMILY); IPR012292 (G3DSA:1.10.490.GENE3D); IPR013316 (PRINTS); PTHR11442 (PANTHER) | |
| 211 | lib3_tri_Filter15_IDBA_contig_4644_4 | 1308 | a chain solution structure of component iv glycera dibranchiata monomeric hemoglobin-co | 320 | 3.60E-31 | 55.83% | 1 | F:binding | IPR000971 (PFAM); IPR009050 (SUPERFAMILY); IPR012292 (G3DSA:1.10.490.GENE3D); IPR013316 (PRINTS); PTHR11442 (PANTHER), tmhmm (TMHMM) | |
| 212 | lib3_tri_Filter15_IDBA_contig_4710_0 | 25 | ---NA--- | 154 | | | 0 | - | no IPS match | |
| 213 | lib3_tri_Filter15_IDBA_contig_4710_5 | 25 | ---NA--- | 153 | | | 0 | - | IPR008160 (PFAM); PTHR24023 (PANTHER), PTHR24023:SF164 (PANTHER) | |
| 214 | lib3_tri_Filter15_IDBA_contig_4741_0 | 978 | ubiquitin-40s ribosomal protein s27a | 153 | 1.48E-69 | 97.50% | 11 | C:nucleoplasm; P:signal transduction; P:response to stress; P:DNA metabolic process; P:translation; F:binding; P:metabolic process; F:structural molecule activity; C:endosome; C:ribosome; C:cytosol | IPR000626 (PFAM); IPR002906 (PFAM); IPR019954 (PROSITE); IPR019955 (PROFILE); IPR019956 (PRINTS); G3DSA:3.10.20.90 (GENE3D), PTHR10666 (PANTHER), SSF54236 (SUPERFAMILY) | |
| 215 | lib3_tri_Filter15_IDBA_contig_4804_3 | 24 | protein unc-22 | 152 | 4.47E-34 | 59.80% | 1 | F:transferase activity | IPR003961 (PFAM); IPR013098 (PFAM); IPR013783 (G3DSA:2.60.40.GENE3D); PR00014 (PRINTS), PTHR10489 (PANTHER), SSF48726 (SUPERFAMILY) | |
| 216 | lib3_tri_Filter15_IDBA_contig_4876_2 | 20 | ---NA--- | 150 | | | 0 | - | IPR008160 (PFAM); PTHR24023 (PANTHER) | |
| 217 | lib3_tri_Filter15_IDBA_contig_4886_0 | 56 | ---NA--- | 150 | | | 0 | - | IPR008160 (PFAM); PTHR24023 (PANTHER) | |
| 218 | lib3_tri_Filter15_IDBA_contig_4892_0 | 17 | glutamyl aminopeptidase (aminopeptidase a) | 150 | 3.32E-38 | 62.10% | 4 | P:biological_process; C:plasma membrane; F:peptidase activity; P:cell proliferation | IPR001930 (PANTHER); G3DSA:1.10.390.10 (GENE3D), PTHR11533:SF59 (PANTHER), SSF55486 (SUPERFAMILY) | |
| 219 | lib3_tri_Filter15_IDBA_contig_4903_2 | 24 | | 149 | | | | | IPR003961 (PFAM); IPR013783 (G3DSA:2.60.40.GENE3D); PR00014 (PRINTS), PTHR10489 (PANTHER) | |
| 220 | lib3_tri_Filter15_IDBA_contig_491_3 | 546 | tropomyosin | 234 | 3.84E-80 | 79.75% | 0 | - | IPR000533 (PRINTS); G3DSA:1.20.5.340 (GENE3D), PTHR19269 (PANTHER), PTHR19269:SF11 (PANTHER) | |
| 221 | lib3_tri_Filter15_IDBA_contig_4942_4 | 13 | lysyl oxidase homolog 4 | 149 | 5.59E-14 | 67.90% | 2 | P:metabolic process; F:catalytic activity | IPR001190 (PFAM); IPR017448 (SMART); G3DSA:3.10.250.10 (GENE3D), PTHR19331 (PANTHER), PTHR19331:SF118 (PANTHER), tmhmm (TMHMM) | |
| 222 | lib3_tri_Filter15_IDBA_contig_4949_1 | 33 | cartilage matrix protein | 148 | 9.07E-18 | 49.42% | 5 | P:regulation of bone mineralization; F:calcium ion binding; P:growth plate cartilage chondrocyte morphogenesis; C:proteinaceous extracellular matrix; P:chondrocyte differentiation | IPR002035 (G3DSA:3.40.50.GENE3D); PTHR22992 (PANTHER), SSF53300 (SUPERFAMILY) | |
| 223 | lib3_tri_Filter15_IDBA_contig_4961_1 | 21 | leucine-rich repeat-containing g protein coupled receptor 5 | 148 | 1.22E-19 | 54.10% | 3 | C:integral to membrane; P:G-protein coupled receptor signaling pathway; F:receptor activity | IPR001611 (PROFILE); IPR003591 (SMART); IPR025875 (PFAM); G3DSA:3.80.10.10 (GENE3D), PTHR24365 (PANTHER), PF13855 (PFAM), SSF52058 (SUPERFAMILY) | |
| 224 | lib3_tri_Filter15_IDBA_contig_4967_1 | 22 | sulfated glycoprotein 1 | 148 | 1.83E-16 | 54.00% | 3 | P:sphingolipid metabolic process; C:lysosome; P:lipid metabolic process | IPR007856 (PFAM); IPR008139 (PROFILE); IPR011001 (G3DSA:1.10.225.GENE3D); PTHR11480 (PANTHER), SignalP-NN(euk) (SIGNALP), tmhmm (TMHMM) | Yes |
| 225 | lib3_tri_Filter15_IDBA_contig_5097_1 | 114 | deleted in malignant brain tumors 1 | 146 | 1.06E-25 | 64.80% | 2 | F:scavenger receptor activity; C:membrane | IPR001190 (PRINTS); IPR017448 (SMART); G3DSA:3.10.250.10 (GENE3D), PTHR19331 (PANTHER) | |
| 226 | lib3_tri_Filter15_IDBA_contig_518_5 | 9807 | intermediate filament protein | 517 | 6.52E-167 | 77.90% | 5 | P:biological_process; F:protein binding; C:cytoskeleton; C:protein complex; F:structural molecule activity | IPR001664 (PANTHER); IPR016451 (PIR); IPR018039 (PROSITE); G3DSA:1.20.5.170 (GENE3D), SignalP-NN(euk) (SIGNALP) | Yes |

| | | | | | | | | | |
|---|---|---|---|---|---|---|---|---|---|
| 227 | lib3_tri_Filter15_IDBA_contig_5232_1 | 18 | ---NA--- | 144 | | | 0 | - | no IPS match |
| 228 | lib3_tri_Filter15_IDBA_contig_5232_5 | 18 | ---NA--- | 143 | | | 0 | - | IPR008160 (PFAM); PTHR24023 (PANTHER) |
| 229 | lib3_tri_Filter15_IDBA_contig_5241_1 | 10 | endothelin-converting enzyme 1 | 143 | 9.12E-09 | 54.10% | 6 | F:peptidase activity; F:binding; P:primary metabolic process; C:cell; P:biological_process; P:metabolic process | IPR000718 (PANTHER); IPR018497 (PFAM); IPR024079 (G3DSA:3.40.390.GENE3D); SSF55486 (SUPERFAMILY) |
| 230 | lib3_tri_Filter15_IDBA_contig_5298_0 | 24 | ---NA--- | 143 | | | 0 | - | IPR008160 (PFAM); PTHR24023 (PANTHER) |
| 231 | lib3_tri_Filter15_IDBA_contig_52_3 | 6487 | peptidyl-prolyl cis-trans isomerase-like protein | 221 | 5.61E-74 | 92.45% | 3 | P:protein metabolic process; P:cellular protein modification process; F:catalytic activity | IPR002130 (PRINTS); IPR020892 (PROSITE); G3DSA:2.40.100.10 (GENE3D), PTHR11071 (PANTHER), PTHR11071:SF116 (PANTHER) |
| 232 | lib3_tri_Filter15_IDBA_contig_5304_5 | 29 | low quality protein: titin | 142 | 8.31E-22 | 54.90% | 11 | F:Rho guanyl-nucleotide exchange factor activity; P:regulation of Rho protein signal transduction; F:protein kinase activity; P:protein phosphorylation; F:ATP binding; F:protein tyrosine kinase activity; P:positive regulation of Rab GTPase activity; C:intracellular; F:Rab GTPase activator activity; F:transferase activity, transferring phosphorus-containing groups; P:regulation of Rab GTPase activity | IPR003599 (SMART); IPR007110 (PROFILE); IPR013098 (PFAM); IPR013783 (G3DSA:2.60.40.GENE3D); IPR020675 (PANTHER); IPR020682 (PTHR22964:PANTHER); SSF48726 (SUPERFAMILY) |
| 233 | lib3_tri_Filter15_IDBA_contig_5327_2 | 17 | ---NA--- | 141 | | | 0 | - | IPR008160 (PFAM); PTHR24023 (PANTHER) |
| 234 | lib3_tri_Filter15_IDBA_contig_5327_4 | 17 | ---NA--- | 142 | | | 0 | - | PTHR24637 (PANTHER), PTHR24637:SF91 (PANTHER) |
| 235 | lib3_tri_Filter15_IDBA_contig_5345_4 | 28 | aminopeptidase n | 141 | 2.28E-39 | 67.40% | 10 | C:integral to membrane; P:interspecies interaction between organisms; F:zinc ion binding; P:angiogenesis; C:endoplasmic reticulum-Golgi intermediate compartment; F:metallopeptidase activity; P:cell differentiation; P:proteolysis; F:receptor activity; F:aminopeptidase activity | IPR001930 (PANTHER); IPR014782 (PRINTS); PTHR11533:SF38 (PANTHER), SSF55486 (SUPERFAMILY), SSF63737 (SUPERFAMILY) |
| 236 | lib3_tri_Filter15_IDBA_contig_5408_2 | 32 | ---NA--- | 140 | | | 0 | - | IPR008160 (PFAM); PTHR24023 (PANTHER) |
| 237 | lib3_tri_Filter15_IDBA_contig_5440_3 | 733 | 14-3-3 protein epsilon | 140 | 2.14E-44 | 88.40% | 21 | P:multicellular organismal development; P:signal transduction; P:reproduction; P:cell differentiation; P:cytoskeleton organization; P:response to stress; P:cell cycle; P:response to abiotic stimulus; C:cytoplasm; F:protein binding; C:extracellular region; P:regulation of biological process; P:organelle organization; P:growth; P:behavior; C:chromosome; F:enzyme regulator activity; C:cytoskeleton; C:protein complex; C:nucleus; P:anatomical structure morphogenesis | IPR000308 (PRINTS); IPR023409 (PROSITE); IPR023410 (G3DSA:1.20.190.GENE3D); PTHR18860:SF0 (PANTHER) |
| 238 | lib3_tri_Filter15_IDBA_contig_5497_0 | 21 | ---NA--- | 139 | | | 0 | - | IPR008160 (PFAM); PTHR24023 (PANTHER) |
| 239 | lib3_tri_Filter15_IDBA_contig_5497_4 | 21 | ---NA--- | 139 | | | 0 | - | no IPS match |
| 240 | lib3_tri_Filter15_IDBA_contig_5560_2 | 17 | deleted in malignant brain tumors 1 | 138 | 5.39E-17 | 51.70% | 3 | F:scavenger receptor activity; F:receptor activity; C:membrane | IPR001190 (PRINTS); IPR017448 (SMART); G3DSA:3.10.250.10 (GENE3D), PTHR19331 (PANTHER), PTHR19331:SF118 (PANTHER) |

| | | | | | | | | | | |
|---|---|---|---|---|---|---|---|---|---|---|
| 241 | lib3_tri_Filter15_IDBA_contig_5564_2 | 13 | protein | 138 | 2.73E-16 | 55.35% | 7 | F:hydrolase activity; P:regulation of biological process; ; P:organelle organization; P:cellular protein modification process; C:nucleoplasm; C:protein complex | IPR016133 (G3DSA:2.160.20.GENE3D) | |
| 242 | lib3_tri_Filter15_IDBA_contig_5564_4 | 13 | interspersed repeat antigen | 138 | 3.71E-17 | 47.44% | 1 | F:transferase activity | no IPS match | |
| 243 | lib3_tri_Filter15_IDBA_contig_5565_3 | 755 | retinal dehydrogenase 1 isoform x2 | 512 | 2.14E-73 | 87.00% | 16 | F:catalytic activity; P:anatomical structure morphogenesis; P:embryo development; F:binding; F:nucleotide binding; P:signal transduction; P:lipid metabolic process; P:secondary metabolic process; P:biological_process; C:cytoplasm; P:catabolic process; P:regulation of biological process; P:cell death; P:multicellular organismal development; P:metabolic process; F:protein binding | IPR015590 (PFAM); IPR016161 (SUPERFAMILY); IPR016163 (G3DSA:3.40.309.GENE3D); PTHR11699 (PANTHER), PTHR11699:SF46 (PANTHER) | |
| 244 | lib3_tri_Filter15_IDBA_contig_5569_3 | 10 | alpha-2-macroglobulin- partial | 138 | 4.79E-20 | 55.60% | 4 | P:negative regulation of endopeptidase activity; C:extracellular space; C:extracellular region; F:endopeptidase inhibitor activity | IPR008930 (SUPERFAMILY); IPR011626 (PFAM); G3DSA:1.50.10.20 (GENE3D), PTHR11412 (PANTHER) | |
| 245 | lib3_tri_Filter15_IDBA_contig_5583_2 | 15 | ---NA--- | 137 | | | 0 | - | PTHR24023 (PANTHER) | |
| 246 | lib3_tri_Filter15_IDBA_contig_5593_1 | 23 | titin- partial | 138 | 9.60E-52 | 57.65% | 6 | P:intracellular signal transduction; P:cyclic nucleotide biosynthetic process; F:phosphorus-oxygen lyase activity; F:Rho guanyl-nucleotide exchange factor activity; P:regulation of Rho protein signal transduction; C:intracellular | IPR003598 (SMART); IPR007110 (PROFILE); IPR013098 (PFAM); IPR013783 (G3DSA:2.60.40.GENE3D); PTHR10489 (PANTHER), SSF48726 (SUPERFAMILY) | |
| 247 | lib3_tri_Filter15_IDBA_contig_5602_3 | 28 | ---NA--- | 138 | | | 0 | - | IPR008160 (PFAM); PTHR24023 (PANTHER) | |
| 248 | lib3_tri_Filter15_IDBA_contig_5610_1 | 26 | sparc | 138 | 5.93E-20 | 60.10% | 4 | F:calcium ion binding; C:extracellular space; P:signal transduction; C:proteinaceous extracellular matrix | G3DSA:3.30.60.30 (GENE3D), PTHR13866 (PANTHER), SignalP-NN(euk) (SIGNALP), SSF100895 (SUPERFAMILY) | Yes |
| 249 | lib3_tri_Filter15_IDBA_contig_5611_5 | 19 | low quality protein: titin | 137 | 3.52E-19 | 54.65% | 6 | F:transferase activity; P:anatomical structure morphogenesis; P:cellular component organization; P:metabolic process; P:organelle organization; P:primary metabolic process | IPR003598 (SMART); IPR003599 (SMART); IPR007110 (PROFILE); IPR013098 (PFAM); IPR013783 (G3DSA:2.60.40.GENE3D); PTHR10489 (PANTHER), SSF48726 (SUPERFAMILY) | |
| 250 | lib3_tri_Filter15_IDBA_contig_5630_4 | 61 | cub domain protein | 253 | 6.86E-12 | 54.55% | 9 | P:transport; P:biosynthetic process; P:lipid metabolic process; C:lysosome; C:vacuole; P:metabolic process; F:protein binding; F:catalytic activity; C:plasma membrane | IPR000859 (G3DSA:2.60.120.GENE3D); PTHR10127 (PANTHER), PTHR10127:SF310 (PANTHER), SignalP-NN(euk) (SIGNALP) | Yes |
| 251 | lib3_tri_Filter15_IDBA_contig_5631_4 | 67 | cubilin precursor | 236 | 4.39E-23 | 52.10% | 4 | F:binding; C:organelle; C:intracellular; C:cytoplasm | IPR000859 (G3DSA:2.60.120.GENE3D); PTHR10127 (PANTHER), SignalP-NN(euk) (SIGNALP) | Yes |
| 252 | lib3_tri_Filter15_IDBA_contig_5635_0 | 22 | paramyosin | 137 | 2.17E-27 | 71.15% | 3 | C:cytoskeleton; C:protein complex; C:cytoplasm | IPR002928 (PFAM); PTHR13140 (PANTHER), PTHR13140:SF106 (PANTHER) | |
| 253 | lib3_tri_Filter15_IDBA_contig_5648_4 | 201 | ---NA--- | 331 | | | 0 | - | no IPS match | |
| 254 | lib3_tri_Filter15_IDBA_contig_5649_4 | 201 | ---NA--- | 309 | | | 0 | - | no IPS match | |

| | | | | | | | | | | |
|---|---|---|---|---|---|---|---|---|---|---|
| 255 | lib3_tri_Filter15_IDBA_contig_5663_4 | 20 | 72 kda type iv collagenase | 137 | 1.78E-17 | 46.90% | 10 | F:peptidase activity; P:biological_process; C:intracellular; P:anatomical structure morphogenesis; P:multicellular organismal development; P:response to endogenous stimulus; P:response to stress; C:extracellular region; P:reproduction; P:metabolic process | IPR000562 (G3DSA:2.10.10.GENE3D); IPR013806 (SUPERFAMILY); PTHR22803 (PANTHER) | |
| 256 | lib3_tri_Filter15_IDBA_contig_5680_2 | 12 | ankyrin repeat domain-containing | 136 | 3.10E-69 | 90.75% | 1 | F:hydrolase activity | IPR002110 (PRINTS); IPR020683 (G3DSA:1.25.40.GENE3D); PTHR24123 (PANTHER), PTHR24123:SF0 (PANTHER) | |
| 257 | lib3_tri_Filter15_IDBA_contig_5686_1 | 38 | histone | 136 | 4.15E-58 | 99.75% | 8 | C:chromosome; P:response to stress; P:response to biotic stimulus; F:DNA binding; C:nucleoplasm; C:protein complex; P:organelle organization; C:lipid particle | IPR000558 (PRINTS); IPR007125 (PFAM); IPR009072 (G3DSA:1.10.20.GENE3D) | |
| 258 | lib3_tri_Filter15_IDBA_contig_5706_5 | 42 | serine partial | 136 | 8.37E-28 | 65.45% | 1 | F:catalytic activity | IPR001190 (PRINTS); IPR017448 (SMART); G3DSA:3.10.250.10 (GENE3D), PTHR19331 (PANTHER), PTHR19331:SF118 (PANTHER) | |
| 259 | lib3_tri_Filter15_IDBA_contig_5709_1 | 15 | serine carboxypeptidase 1 | 136 | 1.03E-34 | 66.00% | 4 | F:peptidase activity; P:lipid metabolic process; P:secondary metabolic process; C:cytosol | IPR001563 (PANTHER); G3DSA:3.40.50.1820 (GENE3D), PTHR11802:SF3 (PANTHER), SSF53474 (SUPERFAMILY) | |
| 260 | lib3_tri_Filter15_IDBA_contig_5736_1 | 1797 | ribosomal protein ubq l40e | 135 | 1.56E-85 | 98.30% | 10 | C:cytoskeleton; C:protein complex; F:structural molecule activity; C:lipid particle; P:cellular protein modification process; P:protein metabolic process; P:catabolic process; C:ribosome; C:cytosol; P:translation | IPR000626 (PFAM); IPR001975 (PFAM); IPR019954 (PROSITE); IPR019955 (PROFILE); IPR019956 (PRINTS); G3DSA:3.10.20.90 (GENE3D), PTHR10666 (PANTHER), SSF54236 (SUPERFAMILY) | |
| 261 | lib3_tri_Filter15_IDBA_contig_5739_0 | 19 | kinase related protein | 136 | 1.09E-33 | 56.60% | 6 | P:biological_process; F:protein binding; P:regulation of biological process; C:cytoplasm; F:protein kinase activity; P:response to stress | IPR003598 (SMART); IPR003599 (SMART); IPR007110 (PROFILE); IPR013098 (PFAM); IPR013783 (G3DSA:2.60.40.GENE3D); PTHR25963 (PANTHER), SSF48726 (SUPERFAMILY) | |
| 262 | lib3_tri_Filter15_IDBA_contig_5771_4 | 16 | bpti kunitz domain-containing protein 4-like | 135 | 2.07E-20 | 50.80% | 9 | P:negative regulation of catalytic activity; F:enzyme inhibitor activity; F:serine-type endopeptidase inhibitor activity; F:peptidase inhibitor activity; F:heparin binding; P:hemostasis; P:blood coagulation; C:extracellular region; P:negative regulation of peptidase activity | IPR004094 (PFAM); IPR011061 (SUPERFAMILY); IPR018112 (G3DSA:2.10.22.GENE3D) | |
| 263 | lib3_tri_Filter15_IDBA_contig_5805_3 | 13 | neuroendocrine convertase 1 | 135 | 2.18E-38 | 64.55% | 1 | F:peptidase activity | IPR002884 (PFAM); IPR008979 (G3DSA:2.60.120.GENE3D); IPR015500 (PANTHER); PTHR10795:SF11 (PANTHER) | |
| 264 | lib3_tri_Filter15_IDBA_contig_5844_5 | 24 | apolipoprotein o-like | 134 | 7.76E-13 | 54.33% | 0 | - | PTHR14564 (PANTHER), PTHR14564:SF0 (PANTHER), tmhmm (TMHMM) | |
| 265 | lib3_tri_Filter15_IDBA_contig_5848_3 | 25 | fibrinogen c domain-containing protein 1-a-like | 134 | 2.09E-23 | 62.20% | 3 | F:receptor binding; C:extracellular space; P:signal transduction | IPR002181 (PFAM); IPR014715 (G3DSA:4.10.530.GENE3D); IPR020837 (PROSITE); PTHR19143 (PANTHER) | |
| 266 | lib3_tri_Filter15_IDBA_contig_584_3 | 4953 | latrophilin-2-like protein | 1358 | 5.82E-09 | 48.05% | 7 | P:neuropeptide signaling pathway; F:latrotoxin receptor activity; F:carbohydrate binding; C:integral to membrane; F:G-protein coupled receptor activity; P:G-protein coupled receptor signaling pathway; C:plasma membrane | IPR000922 (PFAM); PTHR25100 (PANTHER), PTHR25100:SF14 (PANTHER), SignalP-NN(euk) (SIGNALP), tmhmm (TMHMM) | Yes |
| 267 | lib3_tri_Filter15_IDBA_contig_5869_4 | 20 | ---NA--- | 134 | | | 0 | - | IPR001190 (PFAM); IPR017448 (SUPERFAMILY); G3DSA:3.10.250.10 (GENE3D), PTHR19331 (PANTHER), PTHR19331:SF118 (PANTHER) | |

| | | | | | | | | | | |
|---|---|---|---|---|---|---|---|---|---|---|
| 268 | lib3_tri_Filter15_IDBA_contig_5877_3 | 9 | thioredoxin domain-containing protein 5 | 134 | 8.89E-33 | 63.70% | 1 | C:endoplasmic reticulum | IPR012336 (G3DSA:3.40.30.GENE3D); IPR013766 (PFAM); IPR017937 (PROSITE); PTHR18929 (PANTHER), PTHR18929:SF32 (PANTHER) | |
| 269 | lib3_tri_Filter15_IDBA_contig_5879_5 | 25 | lysosomal alpha-glucosidase-like | 133 | 7.68E-17 | 52.25% | 1 | F:hydrolase activity | IPR000322 (PANTHER) | |
| 270 | lib3_tri_Filter15_IDBA_contig_587_0 | 5988 | hypothetical protein, partial | 1664 | 5.70E-09 | 57.00% | 0 | - | PTHR24023 (PANTHER), PTHR24023:SF196 (PANTHER), SignalP-NN(euk) (SIGNALP), tmhmm (TMHMM) | |
| 271 | lib3_tri_Filter15_IDBA_contig_587_3 | 5988 | triple helix repeat-containing collagen | 1664 | 4.72E-09 | 74.50% | 1 | C:collagen | IPR008160 (PFAM); PTHR24023 (PANTHER), tmhmm (TMHMM) | |
| 272 | lib3_tri_Filter15_IDBA_contig_587_4 | 5988 | ---NA--- | 1664 | | | 0 | - | tmhmm (TMHMM) | |
| 273 | lib3_tri_Filter15_IDBA_contig_589_1 | 1632 | map kinase-interacting serine threonine-protein kinase 1 | 1653 | 0 | 74.35% | 3 | F:protein kinase activity; P:metabolic process; F:nucleotide binding | IPR000719 (PFAM); IPR002290 (SMART); IPR008271 (PROSITE); IPR011009 (SUPERFAMILY); IPR017441 (PROSITE); IPR020635 (SMART); G3DSA:1.10.510.10 (GENE3D), G3DSA:3.30.200.20 (GENE3D), PTHR24349 (PANTHER), PTHR24349:SF0 (PANTHER), tmhmm (TMHMM) | |
| 274 | lib3_tri_Filter15_IDBA_contig_592_1 | 3326 | | 1086 | | | | | IPR003961 (PFAM); IPR013783 (G3DSA:2.60.40.GENE3D); PR00014 (PRINTS), tmhmm (TMHMM) | |
| 275 | lib3_tri_Filter15_IDBA_contig_593_0 | 4104 | astacin-like protein | 1030 | 1.26E-67 | 45.50% | 8 | F:carbohydrate binding; F:metal ion binding; P:proteolysis; F:metallopeptidase activity; F:hydrolase activity; F:zinc ion binding; F:peptidase activity; F:metalloendopeptidase activity | IPR000922 (PFAM); IPR003582 (PFAM); PTHR12011 (PANTHER), PTHR12011:SF4 (PANTHER), SignalP-NN(euk) (SIGNALP) | Yes |
| 276 | lib3_tri_Filter15_IDBA_contig_5982_0 | 30 | ---NA--- | 133 | | | 0 | - | IPR008160 (PFAM); PTHR24023 (PANTHER) | |
| 277 | lib3_tri_Filter15_IDBA_contig_598_5 | 1069 | protein-glutamine gamma-glutamyltransferase k | 1011 | 0 | 58.60% | 3 | F:metal ion binding; P:peptide cross-linking; F:protein-glutamine gamma-glutamyltransferase activity | IPR001102 (PFAM); IPR002931 (G3DSA:3.90.260.GENE3D); IPR008958 (PFAM); IPR013783 (G3DSA:2.60.40.GENE3D); IPR014756 (SUPERFAMILY); IPR023608 (PANTHER); SSF54001 (SUPERFAMILY) | |
| 278 | lib3_tri_Filter15_IDBA_contig_5_1 | 1048 | ---NA--- | 1385 | | | 0 | - | no IPS match | |
| 279 | lib3_tri_Filter15_IDBA_contig_5_3 | 1048 | ---NA--- | 1385 | | | 0 | - | IPR008160 (PFAM); PTHR24023 (PANTHER) | |
| 280 | lib3_tri_Filter15_IDBA_contig_6015_2 | 14 | matrix metalloproteinase-9 | 131 | 2.63E-10 | 61.70% | 5 | F:peptidase activity; P:multicellular organismal development; P:cellular component organization; C:extracellular space; P:metabolic process | IPR000001 (PROFILE); IPR000562 (G3DSA:2.10.10.GENE3D); IPR013806 (SUPERFAMILY); PR00013 (PRINTS), PTHR22803 (PANTHER) | |
| 281 | lib3_tri_Filter15_IDBA_contig_605_3 | 717 | ---NA--- | 1085 | | | 0 | - | IPR000716 (G3DSA:4.10.800.GENE3D); IPR022339 (PANTHER); tmhmm (TMHMM) | |
| 282 | lib3_tri_Filter15_IDBA_contig_606_3 | 2254 | ---NA--- | 1468 | | | 0 | - | IPR000716 (G3DSA:4.10.800.GENE3D); IPR022339 (PANTHER); tmhmm (TMHMM) | |
| 283 | lib3_tri_Filter15_IDBA_contig_6077_1 | 34 | ---NA--- | 131 | | | 0 | - | no IPS match | |
| 284 | lib3_tri_Filter15_IDBA_contig_6077_3 | 34 | ---NA--- | 131 | | | 0 | - | IPR008160 (PFAM); PTHR24023 (PANTHER) | |
| 285 | lib3_tri_Filter15_IDBA_contig_60_4 | 751 | ---NA--- | 446 | | | 0 | - | IPR001820 (PFAM); IPR008993 (SUPERFAMILY); G3DSA:2.40.50.120 (GENE3D), SignalP-NN(euk) (SIGNALP), tmhmm (TMHMM) | Yes |
| 286 | lib3_tri_Filter15_IDBA_contig_611_1 | 1846 | paramyosin | 1236 | 0 | 70.85% | 3 | C:cytoskeleton; C:protein complex; C:cytoplasm | IPR002928 (PFAM); IPR009053 (SUPERFAMILY); G3DSA:1.20.5.340 (GENE3D), PTHR13140 (PANTHER), PTHR13140:SF106 (PANTHER), tmhmm (TMHMM) | |
| 287 | lib3_tri_Filter15_IDBA_contig_6129_3 | 23 | immunoglobulin superfamily member 22 | 131 | 2.30E-20 | 63.80% | 1 | F:transferase activity | IPR003961 (PFAM); IPR013783 (G3DSA:2.60.40.GENE3D); PR00014 (PRINTS), PTHR10489 (PANTHER) | |

| | | | | | | | | | | |
|---|---|---|---|---|---|---|---|---|---|---|
| 288 | lib3_tri_Filter15_IDBA_contig_6143_5 | 19 | alpha-galactosidase alpha-n-acetylgalactosaminidase | 130 | 5.13E-46 | 68.80% | 1 | F:hydrolase activity | IPR002241 (PRINTS); IPR013780 (G3DSA:2.60.40.GENE3D); IPR013785 (G3DSA:3.20.20.GENE3D); IPR017853 (SUPERFAMILY); PTHR11452 (PANTHER), SSF51011 (SUPERFAMILY) | |
| 289 | lib3_tri_Filter15_IDBA_contig_6183_2 | 22 | fkbp-type peptidylprolyl cis-trans isomerase | 130 | 1.41E-28 | 86.80% | 3 | P:protein metabolic process; P:cellular protein modification process; F:catalytic activity | IPR001179 (PFAM); IPR023566 (PANTHER); G3DSA:3.10.50.40 (GENE3D), SSF54534 (SUPERFAMILY) | Yes |
| 290 | lib3_tri_Filter15_IDBA_contig_6188_1 | 25 | ---NA--- | 130 | | | 0 | - | IPR008160 (PFAM); PTHR24023 (PANTHER) | |
| 291 | lib3_tri_Filter15_IDBA_contig_618_5 | 667 | calreticulin | 902 | 1.12E-157 | 84.80% | 4 | P:protein metabolic process; C:endoplasmic reticulum; F:protein binding; F:calcium ion binding | IPR001580 (PRINTS); IPR008985 (SUPERFAMILY); IPR013320 (G3DSA:2.60.120.GENE3D); IPR018124 (PROSITE); PTHR11073:SF2 (PANTHER), SignalP-NN(euk) (SIGNALP), tmhmm (TMHMM) | |
| 292 | lib3_tri_Filter15_IDBA_contig_61_3 | 3369 | ybx1 protein | 777 | 1.29E-49 | 90.25% | 24 | P:regulation of biological process; C:plasma membrane; C:nucleoplasm; ; F:transcription regulator activity; F:protein binding; C:nucleus; P:cell death; C:intracellular; F:RNA binding; P:nucleobase-containing compound metabolic process; P:anatomical structure morphogenesis; P:multicellular organismal development; P:reproduction; F:DNA binding; C:extracellular region; P:growth; C:cytoplasm; P:cell differentiation; P:response to stress; P:response to abiotic stimulus; F:sequence-specific DNA binding transcription factor activity; C:protein complex; P:embryo development | IPR002059 (PRINTS); IPR011129 (SMART); IPR012340 (G3DSA:2.40.50.GENE3D); IPR019844 (PROSITE); PTHR11544 (PANTHER), PTHR11544:SF11 (PANTHER), tmhmm (TMHMM) | |
| 293 | lib3_tri_Filter15_IDBA_contig_6209_0 | 20 | ---NA--- | 130 | | | 0 | - | IPR008160 (PFAM); PTHR24023 (PANTHER) | |
| 294 | lib3_tri_Filter15_IDBA_contig_620_5 | 3181 | heat shock protein 70 | 887 | 0 | 95.50% | 2 | F:nucleotide binding; P:response to stress | IPR013126 (PRINTS); IPR018181 (PROSITE); G3DSA:1.20.1270.10 (GENE3D), G3DSA:2.60.34.10 (GENE3D), G3DSA:3.30.30.30 (GENE3D), G3DSA:3.30.420.40 (GENE3D), G3DSA:3.90.640.10 (GENE3D), PTHR19375 (PANTHER), tmhmm (TMHMM), SSF100920 (SUPERFAMILY), SSF100934 (SUPERFAMILY), SSF53067 (SUPERFAMILY) | |
| 295 | lib3_tri_Filter15_IDBA_contig_6230_2 | 20 | ---NA--- | 129 | | | 0 | - | IPR008160 (PFAM); PTHR24023 (PANTHER) | |
| 296 | lib3_tri_Filter15_IDBA_contig_6244_4 | 14 | antistasin-like protein | 129 | 1.55E-22 | 60.30% | 1 | P:biological_process | IPR004094 (PFAM); IPR011061 (SUPERFAMILY); IPR018112 (G3DSA:2.10.22.GENE3D); PTHR11339 (PANTHER), PTHR11339:SF40 (PANTHER) | |
| 297 | lib3_tri_Filter15_IDBA_contig_628_1 | 4811 | myosin heavy chain | 1935 | 0 | 80.25% | 7 | C:cytoskeleton; C:protein complex; F:nucleotide binding; C:cytoplasm; F:actin binding; F:motor activity; F:protein binding | IPR000048 (PFAM); IPR001609 (PRINTS); IPR002928 (PFAM); IPR004009 (PFAM); IPR009053 (SUPERFAMILY); IPR010978 (SUPERFAMILY); IPR027401 (G3DSA:4.10.270.GENE3D); IPR027417 (SUPERFAMILY); G3DSA:1.20.5.340 (GENE3D), PTHR13140 (PANTHER), PTHR13140:SF106 (PANTHER) | |
| 298 | lib3_tri_Filter15_IDBA_contig_6294_3 | 23 | tissue factor pathway inhibitor isoform x7 | 104 | 2.88E-14 | 60.95% | 2 | C:extracellular region; F:enzyme regulator activity | IPR002223 (PRINTS); IPR020901 (PROSITE); PTHR10083 (PANTHER) | |
| 299 | lib3_tri_Filter15_IDBA_contig_629_1 | 3459 | myosin heavy chain | 846 | 0 | 81.70% | 7 | C:cytoskeleton; C:protein complex; F:nucleotide binding; C:cytoplasm; F:actin binding; F:motor activity; F:protein binding | IPR002928 (PFAM); IPR009053 (SUPERFAMILY); IPR010978 (SUPERFAMILY); G3DSA:1.20.1170.10 (GENE3D), PTHR13140 (PANTHER), PTHR13140:SF106 (PANTHER) | |

| | | | | | | | | | | |
|---|---|---|---|---|---|---|---|---|---|---|
| 300 | lib3_tri_Filter15_IDBA_contig_632_4 | 8328 | isoform a | 1048 | 2.81E-57 | 75.80% | 1 | F:actin binding | IPR000557 (PFAM); IPR001715 (G3DSA:1.10.418.GENE3D); IPR003096 (PRINTS); PTHR18959 (PANTHER), PTHR18959:SF3 (PANTHER), tmhmm (TMHMM) | |
| 301 | lib3_tri_Filter15_IDBA_contig_6339_4 | 13 | protocadherin gamma-a11 | 128 | 4.47E-28 | 60.20% | 0 | - | IPR002126 (PRINTS); IPR015919 (SUPERFAMILY); IPR020894 (PROSITE); PTHR24028 (PANTHER), PTHR24028:SF12 (PANTHER) | |
| 302 | lib3_tri_Filter15_IDBA_contig_6351_0 | 15 | aminoacyl trna synthase complex-interacting multifunctional protein 1-like | 128 | 1.42E-14 | 67.10% | 17 | C:cytosol; P:cell proliferation; P:regulation of biological process; C:organelle; C:intracellular; C:protein complex; P:cell-cell signaling; P:response to external stimulus; P:response to stress; P:behavior; P:biological_process; P:translation; P:nucleobase-containing compound metabolic process; ; F:binding; F:RNA binding; F:protein binding | PTHR11946 (PANTHER), PTHR11946:SF3 (PANTHER), tmhmm (TMHMM) | |
| 303 | lib3_tri_Filter15_IDBA_contig_6398_2 | 24 | aminopeptidase n | 127 | 2.70E-35 | 62.45% | 1 | F:peptidase activity | IPR001930 (PANTHER); G3DSA:1.10.390.10 (GENE3D), PTHR11533:SF38 (PANTHER), SSF55486 (SUPERFAMILY) | |
| 304 | lib3_tri_Filter15_IDBA_contig_641_2 | 508 | calmodulin | 1288 | 4.70E-96 | 98.70% | 45 | P:regulation of biological process; P:ion transport; P:biological_process; P:carbohydrate metabolic process; P:protein transport; F:protein binding; P:cytoskeleton organization; P:cell cycle; P:transport; P:cellular component organization; P:cell-cell signaling; C:cell; P:response to external stimulus; P:signal transduction; P:response to abiotic stimulus; F:receptor binding; F:cytoskeletal protein binding; C:cytosol; C:nucleoplasm; P:reproduction; P:behavior; P:organelle organization; P:response to stress; P:growth; C:cytoskeleton; C:protein complex; C:microtubule organizing center; F:molecular_function; P:cell death; C:plasma membrane; P:anatomical structure morphogenesis; F:protein kinase activity; P:embryo development; C:cellular_component; F:nucleotide binding; P:metabolic process; P:cell differentiation; P:multicellular organismal development; P:cellular homeostasis; C:intracellular; P:generation of precursor metabolites and energy; P:catabolic process; F:calcium ion binding; P:cellular protein modification process; | IPR002048 (SMART); IPR011992 (G3DSA:1.10.238.GENE3D); IPR018247 (PROSITE); PTHR23050 (PANTHER), tmhmm (TMHMM), SSF47473 (SUPERFAMILY) | |
| 305 | lib3_tri_Filter15_IDBA_contig_643_0 | 390 | ---NA--- | 810 | | | 0 | - | IPR008160 (PFAM); PTHR24023 (PANTHER) | |
| 306 | lib3_tri_Filter15_IDBA_contig_643_4 | 390 | ---NA--- | 810 | | | 0 | - | no IPS match | |
| 307 | lib3_tri_Filter15_IDBA_contig_6455_2 | 17 | cathepsin l | 127 | 1.34E-20 | 66.00% | 1 | F:peptidase activity | IPR013128 (PANTHER); IPR013201 (PFAM); G3DSA:3.90.70.10 (GENE3D), PTHR12411:SF149 (PANTHER), SignalP-NN(euk) (SIGNALP), tmhmm (TMHMM), SSF54001 (SUPERFAMILY) | Yes |
| 308 | lib3_tri_Filter15_IDBA_contig_6457_2 | 19 | ---NA--- | 127 | | | 0 | - | IPR008160 (PFAM); PTHR24023 (PANTHER) | |

| | | | | | | | | | | |
|---|---|---|---|---|---|---|---|---|---|---|
| 309 | lib3_tri_Filter15_IDBA_contig_6484_2 | 5129 | globin x | 350 | 1.63E-44 | 55.15% | 1 | F:binding | IPR000971 (PFAM); IPR009050 (SUPERFAMILY); IPR012292 (G3DSA:1.10.490.GENE3D); IPR013316 (PRINTS); PTHR22924 (PANTHER) | |
| 310 | lib3_tri_Filter15_IDBA_contig_6490_3 | 12 | dbh-like monooxygenase protein 1 | 127 | 1.90E-19 | 56.75% | 1 | F:catalytic activity | IPR000323 (G3DSA:2.60.120.GENE3D); IPR000945 (PRINTS); IPR008977 (SUPERFAMILY); PTHR10157:SF17 (PANTHER) | |
| 311 | lib3_tri_Filter15_IDBA_contig_652_3 | 333 | protease inhibitor epi11 | 779 | 3.81E-43 | 40.10% | 7 | P:proteolysis; F:peptidase activity; C:protein complex; F:protease binding; F:potassium channel inhibitor activity; F:serine-type endopeptidase inhibitor activity; C:extracellular region | IPR002350 (PFAM); G3DSA:1.10.1890.10 (GENE3D), G3DSA:3.30.60.30 (GENE3D), PTHR10913 (PANTHER), PTHR10913:SF21 (PANTHER), SSF100895 (SUPERFAMILY) | |
| 312 | lib3_tri_Filter15_IDBA_contig_6532_4 | 11 | protocadherin fat 4-like | 126 | 2.60E-14 | 56.82% | 8 | P:cell-cell signaling; F:calcium ion binding; P:homophilic cell adhesion; C:integral to membrane; P:multicellular organismal development; P:cell adhesion; C:membrane; C:plasma membrane | IPR002035 (G3DSA:3.40.50.GENE3D); PTHR22992 (PANTHER), SignalP-NN(euk) (SIGNALP), tmhmm (TMHMM), SSF53300 (SUPERFAMILY) | Yes |
| 313 | lib3_tri_Filter15_IDBA_contig_6559_1 | 10 | guanylate cyclase | 126 | 2.21E-27 | 50.50% | 13 | C:cell; P:transport; P:regulation of biological process; C:organelle; C:intracellular; C:cellular_component; C:plasma membrane; P:cell communication; P:biological_process; P:biosynthetic process; P:nucleobase-containing compound metabolic process; C:cytoplasm; F:binding | IPR001828 (PFAM); IPR028082 (SUPERFAMILY); G3DSA:3.40.50.2300 (GENE3D), PTHR11920 (PANTHER), PTHR11920:SF50 (PANTHER) | |
| 314 | lib3_tri_Filter15_IDBA_contig_6597_4 | 66 | a chain elaborate manifold of short hydrogen bond arrays mediating binding of active site-directed serine protease inhibitors | 176 | 7.44E-32 | 63.60% | 2 | F:protein binding; F:peptidase activity | IPR001254 (PFAM); IPR009003 (SUPERFAMILY); IPR018114 (PROSITE); G3DSA:2.40.10.10 (GENE3D), PTHR24264 (PANTHER), PTHR24264:SF0 (PANTHER) | |
| 315 | lib3_tri_Filter15_IDBA_contig_661_2 | 873 | troponin t | 741 | 2.98E-53 | 62.00% | 9 | P:multicellular organismal development; P:anatomical structure morphogenesis; P:cell differentiation; P:cytoskeleton organization; F:calcium ion binding; P:cellular homeostasis; C:cytoskeleton; C:cytoplasm; P:mitochondrion organization | IPR001978 (PFAM); IPR027707 (PANTHER); G3DSA:1.20.5.350 (GENE3D), PTHR11521:SF1 (PANTHER), tmhmm (TMHMM) | |
| 316 | lib3_tri_Filter15_IDBA_contig_662_3 | 200 | collagen alpha-6 chain-like isoform x2 | 877 | 1.66E-104 | 57.65% | 0 | - | IPR000152 (PROSITE); IPR000742 (SMART); IPR001881 (SMART); IPR002035 (G3DSA:3.40.50.GENE3D); IPR013032 (PROSITE); IPR018097 (PROSITE); PR00453 (PRINTS), G3DSA:2.10.25.10 (GENE3D), PTHR22992 (PANTHER), tmhmm (TMHMM), SSF53300 (SUPERFAMILY) | |
| 317 | lib3_tri_Filter15_IDBA_contig_663_3 | 429 | aminopeptidase n | 776 | 3.12E-80 | 54.10% | 6 | P:proteolysis; F:metallopeptidase activity; F:aminopeptidase activity; F:zinc ion binding; C:integral to membrane; C:plasma membrane | IPR001930 (PANTHER); IPR024571 (PFAM); PTHR11533:SF38 (PANTHER), tmhmm (TMHMM) | |
| 318 | lib3_tri_Filter15_IDBA_contig_6674_2 | 16 | hhip-like protein partial | 124 | 4.25E-07 | 55.00% | 0 | - | IPR001190 (PFAM); IPR017448 (SUPERFAMILY); G3DSA:3.10.250.10 (GENE3D), PTHR19331 (PANTHER), PTHR19331:SF118 (PANTHER) | |
| 319 | lib3_tri_Filter15_IDBA_contig_6688_5 | 20 | furin | 124 | 5.29E-16 | 58.80% | 4 | P:regulation of biological process; F:peptidase activity; P:biological_process; P:protein metabolic process | IPR002884 (PFAM); IPR008979 (G3DSA:2.60.120.GENE3D); IPR015500 (PANTHER); PTHR10795:SF155 (PANTHER) | |
| 320 | lib3_tri_Filter15_IDBA_contig_6710_3 | 12 | superoxide dismutase | 125 | 1.01E-34 | 71.75% | 2 | P:metabolic process; F:catalytic activity | IPR001189 (PANTHER); IPR019832 (PFAM); IPR019833 (PROSITE); PTHR11404:SF9 (PANTHER) | |
| 321 | lib3_tri_Filter15_IDBA_contig_6735_3 | 19 | ---NA--- | 124 | | | 0 | - | IPR008160 (PFAM); PTHR24023 (PANTHER) | |

| | | | | | | | | | |
|---|---|---|---|---|---|---|---|---|---|
| 322 | lib3_tri_Filter15_IDBA_contig_6735_4 | 19 | ---NA--- | 124 | | | 0 | - | no IPS match |
| 323 | lib3_tri_Filter15_IDBA_contig_675_3 | 2653 | calmodulin | 1551 | 1.86E-96 | 99.60% | 34 | P:biological_process; P:regulation of biological process; P:protein transport; P:cytoskeleton organization; P:cell cycle; P:transport; P:cellular component organization; P:cell-cell signaling; C:cell; P:response to external stimulus; P:signal transduction; P:response to abiotic stimulus; F:cytoskeletal protein binding; P:reproduction; P:behavior; P:organelle organization; P:ion transport; P:growth; C:cytoskeleton; C:protein complex; C:microtubule organizing center; P:cell death; C:plasma membrane; P:anatomical structure morphogenesis; F:protein kinase activity; P:embryo development; C:cellular_component; F:nucleotide binding; P:cell differentiation; P:multicellular organismal development; F:protein binding; C:intracellular; F:calcium ion binding; P:cellular protein | IPR002048 (SMART); IPR011992 (G3DSA:1.10.238.GENE3D); IPR018247 (PROSITE); PTHR23050 (PANTHER), tmhmm (TMHMM), SSF47473 (SUPERFAMILY) |
| 324 | lib3_tri_Filter15_IDBA_contig_684_3 | 440 | chymotrypsin-like elastase family member 2a-like | 674 | 1.60E-83 | 51.10% | 1 | F:hydrolase activity | IPR001254 (PFAM); IPR001314 (PRINTS); IPR009003 (SUPERFAMILY); IPR018114 (PROSITE); G3DSA:2.40.10.10 (GENE3D), PTHR24260 (PANTHER), SignalP-NN(euk) (SIGNALP), tmhmm (TMHMM) |
| 325 | lib3_tri_Filter15_IDBA_contig_6869_4 | 15 | 72 kda type iv collagenase | 122 | 8.96E-13 | 49.90% | 14 | P:anatomical structure morphogenesis; P:multicellular organismal development; F:protein binding; P:biological_process; P:response to external stimulus; P:regulation of biological process; P:cell communication; P:response to abiotic stimulus; F:peptidase activity; P:response to endogenous stimulus; P:response to stress; P:metabolic process; C:extracellular region; P:reproduction | IPR000562 (G3DSA:2.10.10.GENE3D); IPR013806 (SUPERFAMILY); PTHR22918 (PANTHER), PTHR22918:SF0 (PANTHER) |
| 326 | lib3_tri_Filter15_IDBA_contig_6879_4 | 17 | ---NA--- | 122 | | | 0 | - | no IPS match |
| 327 | lib3_tri_Filter15_IDBA_contig_6972_0 | 18 | ---NA--- | 122 | | | 0 | - | IPR008160 (PFAM); PTHR24023 (PANTHER) |
| 328 | lib3_tri_Filter15_IDBA_contig_6972_5 | 18 | ---NA--- | 121 | | | 0 | - | no IPS match |

| | | | | | | | | | | |
|---|---|---|---|---|---|---|---|---|---|---|
| 329 | lib3_tri_Filter15_IDBA_contig_699_3 | 1635 | adp-ribosylation factor 1 | 1046 | 2.87E-122 | 98.30% | 25 | F:signal transducer activity; P:anatomical structure morphogenesis; P:cellular component organization; P:regulation of biological process; C:Golgi apparatus; F:protein binding; P:biological_process; P:viral reproduction; P:transport; P:cell-cell signaling; F:nucleotide binding; P:protein transport; P:cellular homeostasis; P:nucleobase-containing compound metabolic process; P:catabolic process; C:cytosol; P:response to stress; P:response to biotic stimulus; P:signal transduction; P:multicellular organismal development; C:cytoplasm; F:hydrolase activity; C:plasma membrane; P:organelle organization; P:cellular protein modification process | IPR003579 (SMART); IPR005225 (TIGRFAMs); IPR006687 (SMART); IPR006689 (PRINTS); IPR024156 (SMART); IPR027417 (SUPERFAMILY); G3DSA:3.40.50.300 (GENE3D), PTHR11711 (PANTHER), tmhmm (TMHMM) | |
| 330 | lib3_tri_Filter15_IDBA_contig_700_5 | 2433 | angiotensin-converting enzyme-like isoform 1 | 1356 | 1.19E-86 | 53.90% | 10 | F:peptidase activity; P:biological_process; F:protein binding; C:extracellular region; P:regulation of biological process; P:multicellular organismal development; F:binding; C:cell; P:catabolic process; C:plasma membrane | IPR001548 (PRINTS); PTHR10514:SF16 (PANTHER), tmhmm (TMHMM), SSF55486 (SUPERFAMILY) | |
| 331 | lib3_tri_Filter15_IDBA_contig_7014_0 | 16 | tumor necrosis factor-inducible gene 6 protein | 134 | 1.64E-09 | 52.45% | 3 | F:calcium ion binding; P:cell adhesion; F:hyaluronic acid binding | IPR000859 (G3DSA:2.60.120.GENE3D); PTHR10127 (PANTHER) | |
| 332 | lib3_tri_Filter15_IDBA_contig_7046_4 | 15 | ---NA--- | 121 | | | 0 | - | IPR008160 (PFAM); PTHR24023 (PANTHER) | |
| 333 | lib3_tri_Filter15_IDBA_contig_705_4 | 567 | matrix metalloproteinase-16-like | 845 | 1.55E-37 | 55.15% | 10 | P:cellular component organization; P:anatomical structure morphogenesis; P:multicellular organismal development; C:cell; P:cell death; F:peptidase activity; P:biological_process; P:growth; P:response to external stimulus; P:response to stress | IPR000585 (G3DSA:2.110.10.GENE3D); IPR018486 (PROSITE); IPR018487 (PFAM); PTHR10201 (PANTHER), tmhmm (TMHMM) | |
| 334 | lib3_tri_Filter15_IDBA_contig_7080_0 | 20 | protein | 121 | 4.33E-16 | 62.10% | 3 | F:scavenger receptor activity; C:membrane; P:receptor-mediated endocytosis | IPR001190 (PRINTS); IPR017448 (SMART); G3DSA:3.10.250.10 (GENE3D), PTHR19331 (PANTHER), PTHR19331:SF118 (PANTHER) | |
| 335 | lib3_tri_Filter15_IDBA_contig_7094_4 | 13 | bone morphogenetic protein 1 | 120 | 1.31E-11 | 63.15% | 2 | F:peptidase activity; F:binding | IPR000859 (G3DSA:2.60.120.GENE3D); PTHR10127 (PANTHER) | |
| 336 | lib3_tri_Filter15_IDBA_contig_7096_5 | 13 | aminopeptidase n | 120 | 3.13E-22 | 57.30% | 1 | F:hydrolase activity | IPR001930 (PANTHER); IPR014782 (PFAM); SSF63737 (SUPERFAMILY) | |
| 337 | lib3_tri_Filter15_IDBA_contig_711_0 | 526 | thioredoxin peroxidase | 621 | 2.29E-118 | 87.60% | 3 | P:metabolic process; F:catalytic activity; F:antioxidant activity | IPR000866 (PFAM); IPR012336 (G3DSA:3.40.30.GENE3D); IPR019479 (PFAM); PTHR10681 (PANTHER), PTHR10681:SF44 (PANTHER), tmhmm (TMHMM) | |
| 338 | lib3_tri_Filter15_IDBA_contig_7126_2 | 20 | | 119 | | | | | IPR003961 (PFAM); IPR013783 (G3DSA:2.60.40.GENE3D); PR00014 (PRINTS) | |
| 339 | lib3_tri_Filter15_IDBA_contig_7221_1 | 59 | ---NA--- | 131 | | | 0 | - | no IPS match | |
| 340 | lib3_tri_Filter15_IDBA_contig_7281_5 | 12 | carbonic anhydrase 2 | 118 | 1.28E-16 | 53.40% | 5 | F:metal ion binding; F:zinc ion binding; P:one-carbon metabolic process; F:lyase activity; F:carbonate dehydratase activity | IPR001148 (G3DSA:3.10.200.GENE3D); IPR023561 (PANTHER) | |
| 341 | lib3_tri_Filter15_IDBA_contig_7341_1 | 13 | immunoglobulin i-set domain protein | 118 | 2.82E-30 | 58.65% | 7 | P:multicellular organismal development; C:cytoplasm; F:structural molecule activity; P:anatomical structure morphogenesis; P:cell differentiation; P:cytoskeleton organization; F:protein kinase activity | IPR003598 (SMART); IPR007110 (PROFILE); IPR013098 (PFAM); IPR013783 (G3DSA:2.60.40.GENE3D); PTHR25963 (PANTHER), SSF48726 (SUPERFAMILY) | Yes |

| | | | | | | | | | |
|---|---|---|---|---|---|---|---|---|---|
| 342 | lib3_tri_Filter15_IDBA_contig_734_0 | 387 | calmodulin | 589 | 1.94E-101 | 98.35% | 34 | P:biological_process; P:regulation of biological process; P:protein transport; P:cytoskeleton organization; P:cell cycle; P:transport; P:cellular component organization; P:cell-cell signaling; C:cell; P:response to external stimulus; P:signal transduction; P:response to abiotic stimulus; F:cytoskeletal protein binding; P:reproduction; P:behavior; P:organelle organization; P:ion transport; P:growth; C:cytoskeleton; C:protein complex; C:microtubule organizing center; P:cell death; C:plasma membrane; P:anatomical structure morphogenesis; F:protein kinase activity; P:embryo development; C:cellular_component; F:nucleotide binding; P:cell differentiation; P:multicellular organismal development; F:protein binding; C:intracellular; F:calcium ion binding; P:cellular protein | IPR002048 (SMART); IPR011992 (G3DSA:1.10.238.GENE3D); IPR018247 (PROSITE); PTHR23050 (PANTHER), PTHR23050:SF84 (PANTHER), tmhmm (TMHMM), SSF47473 (SUPERFAMILY) |
| 343 | lib3_tri_Filter15_IDBA_contig_738_0 | 234 | protein disulfide isomerase | 585 | 0 | 77.80% | 6 | F:electron carrier activity; P:cellular homeostasis; P:regulation of biological process; P:metabolic process; F:catalytic activity; C:endoplasmic reticulum | IPR005746 (PRINTS); IPR005788 (TIGRFAMs); IPR005792 (TIGRFAMs); IPR012336 (G3DSA:3.40.30.GENE3D); IPR013766 (PFAM); IPR017937 (PROSITE); PTHR18929 (PANTHER), PTHR18929:SF32 (PANTHER), PF13848 (PFAM), SignalP-NN(euk) (SIGNALP), tmhmm (TMHMM) |
| 344 | lib3_tri_Filter15_IDBA_contig_7412_0 | 11 | | 117 | | | | | IPR003961 (PFAM); IPR013783 (G3DSA:2.60.40.GENE3D); PR00014 (PRINTS) |
| 345 | lib3_tri_Filter15_IDBA_contig_7419_5 | 17 | ---NA--- | 117 | | | 0 | - | IPR000859 (G3DSA:2.60.120.GENE3D); PTHR10127 (PANTHER) |
| 346 | lib3_tri_Filter15_IDBA_contig_7420_1 | 14 | ---NA--- | 117 | | | 0 | - | no IPS match |
| 347 | lib3_tri_Filter15_IDBA_contig_7420_3 | 14 | ---NA--- | 117 | | | 0 | - | PTHR24023 (PANTHER), PTHR24023:SF164 (PANTHER) |
| 348 | lib3_tri_Filter15_IDBA_contig_7450_0 | 15 | low quality protein: titin | 117 | 2.41E-17 | 56.45% | 7 | C:organelle; F:protein binding; P:anatomical structure morphogenesis; P:cell differentiation; P:multicellular organismal development; P:cytoskeleton organization; C:intracellular | IPR003598 (SMART); IPR003599 (SMART); IPR007110 (PROFILE); IPR013098 (PFAM); IPR013783 (G3DSA:2.60.40.GENE3D); PTHR10489 (PANTHER), SSF48726 (SUPERFAMILY) |
| 349 | lib3_tri_Filter15_IDBA_contig_7460_1 | 15 | ---NA--- | 117 | | | 0 | - | IPR008160 (PFAM); PTHR24023 (PANTHER) |
| 350 | lib3_tri_Filter15_IDBA_contig_7471_2 | 16 | ---NA--- | 116 | | | 0 | - | IPR001254 (PFAM); IPR009003 (SUPERFAMILY); G3DSA:2.40.10.10 (GENE3D), PTHR24273 (PANTHER), PTHR24273:SF0 (PANTHER) |
| 351 | lib3_tri_Filter15_IDBA_contig_7570_2 | 24 | low quality protein: titin | 115 | 9.33E-20 | 54.80% | 1 | F:transferase activity | IPR003599 (SMART); IPR013098 (PFAM); IPR013783 (G3DSA:2.60.40.GENE3D); PTHR25963 (PANTHER), SSF48726 (SUPERFAMILY) |
| 352 | lib3_tri_Filter15_IDBA_contig_7594_1 | 8 | anionic trypsin-like | 115 | 9.10E-31 | 69.20% | 1 | F:peptidase activity | IPR001254 (PFAM); IPR009003 (SUPERFAMILY); IPR018114 (PROSITE); G3DSA:2.40.10.10 (GENE3D), PTHR24264 (PANTHER) |
| 353 | lib3_tri_Filter15_IDBA_contig_7664_1 | 10 | ---NA--- | 115 | | | 0 | - | IPR013032 (PROSITE) |
| 354 | lib3_tri_Filter15_IDBA_contig_7696_5 | 15 | ---NA--- | 114 | | | 0 | - | IPR008160 (PFAM); PTHR24023 (PANTHER) |

| | | | | | | | | | |
|---|---|---|---|---|---|---|---|---|---|
| 355 | lib3_tri_Filter15_IDBA_contig_7701_3 | 11 | cathepsin l | 115 | 3.10E-34 | 77.85% | 4 | P:protein metabolic process; P:catabolic process; P:biological_process; F:peptidase activity | IPR000668 (PFAM); IPR013128 (PANTHER); IPR025660 (PROSITE); IPR025661 (PROSITE); G3DSA:3.90.70.10 (GENE3D), PTHR12411:SF149 (PANTHER), SSF54001 (SUPERFAMILY) |
| 356 | lib3_tri_Filter15_IDBA_contig_7703_1 | 18 | protocadherin gamma-b7 | 114 | 1.65E-20 | 53.50% | 6 | F:calcium ion binding; P:homophilic cell adhesion; C:integral to membrane; P:cell adhesion; C:membrane; C:plasma membrane | IPR002126 (PRINTS); IPR015919 (SUPERFAMILY); PTHR24027 (PANTHER), PTHR24027:SF110 (PANTHER) |
| 357 | lib3_tri_Filter15_IDBA_contig_7787_3 | 9 | paraoxonase 1 | 114 | 9.25E-15 | 54.60% | 9 | P:transport; P:regulation of biological process; P:lipid metabolic process; P:catabolic process; F:binding; F:hydrolase activity; P:metabolic process; P:biological_process; C:extracellular space | IPR002640 (PFAM); IPR011042 (G3DSA:2.120.10.GENE3D); PTHR11799 (PANTHER), PTHR11799:SF0 (PANTHER) |
| 358 | lib3_tri_Filter15_IDBA_contig_7813_0 | 22 | ---NA--- | 114 | | | 0 | - | IPR008160 (PFAM); PTHR24023 (PANTHER) |
| 359 | lib3_tri_Filter15_IDBA_contig_7815_2 | 142 | lysyl oxidase like protein 2 | 222 | 5.34E-33 | 66.80% | 10 | C:cytosol; P:regulation of biological process; P:protein transport; F:protein binding; P:transport; C:organelle; C:cytoplasm; P:cell differentiation; P:biological_process; C:plasma membrane | IPR001190 (PRINTS); IPR017448 (SMART); G3DSA:3.10.250.10 (GENE3D), PTHR19331 (PANTHER) |
| 360 | lib3_tri_Filter15_IDBA_contig_782_0 | 103 | superfamily ii dna rna partial | 236 | 7.31E-13 | 64.07% | 0 | - | no IPS match |
| 361 | lib3_tri_Filter15_IDBA_contig_7919_5 | 12 | hypothetical protein CAPTEDRAFT_226305 | 112 | 7.65E-33 | 58.25% | 0 | - | IPR000152 (PROSITE); IPR001881 (PFAM); IPR018097 (PROSITE); G3DSA:2.10.25.10 (GENE3D), PTHR24035 (PANTHER), PTHR24035:SF4 (PANTHER), SSF57196 (SUPERFAMILY) |
| 362 | lib3_tri_Filter15_IDBA_contig_7938_4 | 19 | mitochondrial manganese superoxide dismutase | 113 | 3.12E-52 | 86.40% | 3 | F:binding; P:metabolic process; F:catalytic activity | IPR001189 (PRINTS); IPR019832 (PFAM); IPR019833 (PROSITE); PTHR11404:SF5 (PANTHER) |
| 363 | lib3_tri_Filter15_IDBA_contig_7_0 | 4597 | ---NA--- | 786 | | | 0 | - | no IPS match |
| 364 | lib3_tri_Filter15_IDBA_contig_7_5 | 4597 | ---NA--- | 785 | | | 0 | - | IPR008160 (PFAM); PTHR24023 (PANTHER), tmhmm (TMHMM) |
| 365 | lib3_tri_Filter15_IDBA_contig_8093_4 | 9 | counting factor associated protein d-like | 111 | 3.72E-41 | 81.60% | 3 | F:peptidase activity; P:protein metabolic process; P:catabolic process | IPR000169 (PROSITE); IPR000668 (PFAM); IPR013128 (PANTHER); G3DSA:3.90.70.10 (GENE3D), PTHR12411:SF47 (PANTHER), SSF54001 (SUPERFAMILY) |
| 366 | lib3_tri_Filter15_IDBA_contig_8097_2 | 25 | connectin titin-like | 111 | 1.33E-27 | 71.40% | 25 | P:cell differentiation; P:multicellular organismal development; P:biological_process; F:cytoskeletal protein binding; P:response to external stimulus; P:response to stress; P:metabolic process; P:regulation of biological process; P:anatomical structure morphogenesis; P:cytoskeleton organization; F:protein binding; P:organelle organization; P:cell cycle; P:transport; F:calcium ion binding; C:cytoskeleton; C:cytoplasm; C:extracellular region; C:cytosol; F:structural molecule activity; F:protein kinase activity; F:actin binding; C:nuclear chromosome; P:embryo development; C:protein complex | IPR003598 (SMART); IPR007110 (PROFILE); IPR013098 (PFAM); IPR013783 (G3DSA:2.60.40.GENE3D); IPR020675 (PANTHER); SSF48726 (SUPERFAMILY) |
| 367 | lib3_tri_Filter15_IDBA_contig_8114_1 | 10 | camk mlck protein kinase | 111 | 1.03E-33 | 55.75% | 4 | P:regulation of biological process; F:kinase activity; C:cytoplasm; P:growth | IPR003598 (SMART); IPR007110 (PROFILE); IPR013098 (PFAM); IPR013783 (G3DSA:2.60.40.GENE3D); PTHR10489 (PANTHER), SSF48726 (SUPERFAMILY) |

| | | | | | | | | | | |
|---|---|---|---|---|---|---|---|---|---|---|
| 368 | lib3_tri_Filter15_IDBA_contig_8187_3 | 10 | kinesin-related motor protein eg5 2 | 111 | 2.28E-08 | 51.83% | 0 | - | IPR000742 (SMART); IPR001774 (PFAM); IPR013032 (PROSITE); G3DSA:2.10.25.10 (GENE3D), PTHR24044 (PANTHER), PTHR24044:SF0 (PANTHER), SSF57196 (SUPERFAMILY) | |
| 369 | lib3_tri_Filter15_IDBA_contig_8198_1 | 14 | ---NA--- | 111 | | | 0 | - | IPR001254 (PFAM); IPR009003 (SUPERFAMILY); G3DSA:2.40.10.10 (GENE3D), PTHR24265 (PANTHER) | |
| 370 | lib3_tri_Filter15_IDBA_contig_8199_4 | 18 | cell surface protein precursor | 110 | 1.38E-10 | 48.14% | 2 | F:calcium ion binding; F:calcium-dependent phospholipid binding | no IPS match | |
| 371 | lib3_tri_Filter15_IDBA_contig_8209_1 | 13 | | 110 | | | | | IPR003961 (PFAM); IPR013783 (G3DSA:2.60.40.GENE3D); PR00014 (PRINTS), PTHR10489 (PANTHER) | |
| 372 | lib3_tri_Filter15_IDBA_contig_8223_2 | 11 | ---NA--- | 110 | | | 0 | - | IPR002181 (PFAM); IPR014715 (G3DSA:4.10.530.GENE3D); PTHR19143 (PANTHER) | |
| 373 | lib3_tri_Filter15_IDBA_contig_8244_3 | 163 | thyroglobulin-like isoform 1 | 167 | 1.08E-10 | 48.95% | 0 | - | IPR000716 (G3DSA:4.10.800.GENE3D); PTHR12352 (PANTHER) | |
| 374 | lib3_tri_Filter15_IDBA_contig_8281_2 | 12 | ferritin heavy subunit | 110 | 3.67E-12 | 66.90% | 1 | F:binding | IPR001519 (PANTHER); IPR008331 (PFAM); IPR009040 (PROFILE); IPR009078 (SUPERFAMILY); IPR012347 (G3DSA:1.20.1260.GENE3D); SignalP-NN(euk) (SIGNALP) | Yes |
| 375 | lib3_tri_Filter15_IDBA_contig_8298_3 | 11 | plasma alpha-l-fucosidase | 110 | 3.84E-38 | 84.05% | 3 | P:carbohydrate metabolic process; F:hydrolase activity; F:binding | IPR000933 (PANTHER); IPR013781 (G3DSA:3.20.20.GENE3D); IPR016286 (PRINTS); IPR017853 (SUPERFAMILY); PTHR10030:SF2 (PANTHER) | |
| 376 | lib3_tri_Filter15_IDBA_contig_8356_4 | 13 | granulin-like protein | 109 | 2.33E-14 | 57.65% | 3 | C:organelle; C:intracellular; F:receptor binding | IPR000118 (PFAM); PTHR12274 (PANTHER), PTHR12274:SF0 (PANTHER) | |
| 377 | lib3_tri_Filter15_IDBA_contig_836_3 | 474 | arylacetamide deacetylase-like 4-like | 684 | 1.45E-51 | 51.05% | 4 | F:hydrolase activity; P:metabolic process; C:integral to membrane; F:carboxylesterase activity | IPR013094 (PFAM); G3DSA:3.40.50.1820 (GENE3D), PTHR23024 (PANTHER), SignalP-NN(euk) (SIGNALP), SSF53474 (SUPERFAMILY) | Yes |
| 378 | lib3_tri_Filter15_IDBA_contig_8388_1 | 8 | protein-glutamine gamma-glutamyltransferase k | 109 | 1.87E-19 | 62.75% | 0 | - | IPR008958 (SUPERFAMILY); IPR013783 (G3DSA:2.60.40.GENE3D); IPR023608 (PANTHER) | |
| 379 | lib3_tri_Filter15_IDBA_contig_8406_1 | 19 | cytosolic non-specific dipeptidase | 109 | 5.34E-51 | 86.15% | 4 | F:peptidase activity; P:protein metabolic process; P:catabolic process; F:hydrolase activity | IPR001261 (PROSITE); IPR002933 (PFAM); G3DSA:3.40.630.10 (GENE3D), PTHR11014 (PANTHER), PTHR11014:SF15 (PANTHER), SSF53187 (SUPERFAMILY) | |
| 380 | lib3_tri_Filter15_IDBA_contig_8441_2 | 3026 | hypothetical protein CAPTEDRAFT_223820 | 297 | 1.52E-26 | 54.00% | 0 | - | IPR021712 (PFAM); SignalP-NN(euk) (SIGNALP), tmhmm (TMHMM) | Yes |
| 381 | lib3_tri_Filter15_IDBA_contig_8449_2 | 10 | aael008062- partial | 108 | 5.17E-15 | 71.90% | 4 | P:biological_process; C:extracellular region; P:regulation of biological process; F:binding | IPR003367 (PFAM); IPR017897 (PROFILE); G3DSA:4.10.1080.10 (GENE3D), PTHR10199 (PANTHER), SSF103647 (SUPERFAMILY) | |
| 382 | lib3_tri_Filter15_IDBA_contig_845_3 | 604 | thioredoxin-1 | 582 | 3.77E-26 | 71.50% | 4 | P:cell redox homeostasis; F:electron carrier activity; P:glycerol ether metabolic process; F:protein disulfide oxidoreductase activity | IPR005746 (PRINTS); IPR012336 (G3DSA:3.40.30.GENE3D); IPR013766 (PFAM); IPR017937 (PROSITE) | |
| 383 | lib3_tri_Filter15_IDBA_contig_846_0 | 262 | signal cub and egf-like domain-containing protein 1 | 627 | 2.12E-77 | 43.40% | 1 | F:calcium ion binding | IPR000436 (PROFILE); IPR011641 (PFAM); G3DSA:2.10.25.10 (GENE3D), G3DSA:2.10.50.10 (GENE3D), PTHR22727 (PANTHER), SSF57196 (SUPERFAMILY), SSF57586 (SUPERFAMILY) | |
| 384 | lib3_tri_Filter15_IDBA_contig_849_1 | 3596 | ---NA--- | 546 | | | 0 | - | IPR008160 (PFAM); PTHR24023 (PANTHER), PTHR24023:SF210 (PANTHER), tmhmm (TMHMM) | |
| 385 | lib3_tri_Filter15_IDBA_contig_849_4 | 3596 | ---NA--- | 546 | | | 0 | - | SignalP-NN(euk) (SIGNALP), tmhmm (TMHMM) | |
| 386 | lib3_tri_Filter15_IDBA_contig_852_2 | 1150 | ---NA--- | 742 | | | 0 | - | IPR000859 (G3DSA:2.60.120.GENE3D); PTHR10127 (PANTHER) | |
| 387 | lib3_tri_Filter15_IDBA_contig_854_1 | 3562 | ---NA--- | 542 | | | 0 | - | IPR008160 (PFAM); PTHR24023 (PANTHER), tmhmm (TMHMM) | |

| | | | | | | | | | | |
|---|---|---|---|---|---|---|---|---|---|---|
| 388 | lib3_tri_Filter15_IDBA_contig_854_5 | 3562 | ---NA--- | 541 | | | 0 | - | SignalP-NN(euk) (SIGNALP), tmhmm (TMHMM) | |
| 389 | lib3_tri_Filter15_IDBA_contig_8603_4 | 6 | collagen alpha-3 chain | 107 | 4.12E-34 | 62.25% | 0 | - | IPR000152 (PROSITE); IPR000742 (PFAM); IPR001881 (SMART); IPR002035 (G3DSA:3.40.50.GENE3D); IPR013032 (PROSITE); PR00010 (PRINTS), G3DSA:2.10.25.10 (GENE3D), PTHR24048 (PANTHER), PTHR24048:SF15 (PANTHER), SSF53300 (SUPERFAMILY), SSF57196 (SUPERFAMILY) | |
| 390 | lib3_tri_Filter15_IDBA_contig_8618_1 | 11 | stromal cell-derived factor 2-like 1 | 107 | 1.84E-25 | 81.80% | 2 | C:cell; C:extracellular region | IPR016093 (SMART); IPR027005 (PANTHER); G3DSA:2.80.10.50 (GENE3D), PTHR10050:SF9 (PANTHER), SignalP-NN(euk) (SIGNALP) | |
| 391 | lib3_tri_Filter15_IDBA_contig_8624_2 | 9 | srcrb4d protein | 107 | 6.06E-11 | 62.80% | 10 | P:oxidation-reduction process; F:scavenger receptor activity; F:copper ion binding; C:membrane; F:oxidoreductase activity, acting on the CH-NH2 group of donors, oxygen as acceptor; P:cell adhesion; C:proteinaceous extracellular matrix; P:receptor-mediated endocytosis; C:extracellular region; P:biological_process | IPR001190 (PRINTS); IPR017448 (SUPERFAMILY); G3DSA:3.10.250.10 (GENE3D), PTHR19331 (PANTHER), PTHR19331:SF118 (PANTHER), SignalP-NN(euk) (SIGNALP), tmhmm (TMHMM) | |
| 392 | lib3_tri_Filter15_IDBA_contig_8629_4 | 11 | collagen type i alpha 1 | 107 | 1.03E-45 | 69.40% | 13 | P:anatomical structure morphogenesis; P:multicellular organismal development; P:biological_process; P:regulation of biological process; P:cell differentiation; C:proteinaceous extracellular matrix; P:metabolic process; P:cell death; F:structural molecule activity; C:extracellular space; P:embryo development; C:cytoplasm; P:cellular component organization | IPR000885 (PRODOM); PTHR24637 (PANTHER), PTHR24637:SF84 (PANTHER) | |
| 393 | lib3_tri_Filter15_IDBA_contig_8654_2 | 17 | 72 kda type iv collagenase | 107 | 3.98E-10 | 66.20% | 20 | P:multicellular organismal development; P:reproduction; P:cellular component organization; P:response to stress; P:biological_process; F:peptidase activity; P:response to external stimulus; P:response to abiotic stimulus; P:anatomical structure morphogenesis; C:extracellular space; P:regulation of biological process; P:cell differentiation; P:cell death; P:metabolic process; P:protein metabolic process; P:catabolic process; P:response to endogenous stimulus; F:protein binding; C:plasma membrane; C:cytoplasm [illegible] | IPR000562 (G3DSA:2.10.10.GENE3D); IPR013806 (SUPERFAMILY); PR00013 (PRINTS), PTHR22918 (PANTHER), PTHR22918:SF0 (PANTHER) | |
| 394 | lib3_tri_Filter15_IDBA_contig_869_5 | 512 | neuronal acetylcholine receptor subunit alpha-9 | 529 | 1.99E-37 | 47.85% | 16 | F:acetylcholine-activated cation-selective channel activity; F:ion channel activity; C:integral to membrane; C:membrane; C:synapse; P:ion transport; C:cell junction; P:transport; F:extracellular ligand-gated ion channel activity; F:receptor activity; C:postsynaptic membrane; C:plasma membrane; P:inner ear morphogenesis; P:cation transport; P:detection of mechanical stimulus involved in sensory perception of sound; P:elevation of cytosolic calcium ion concentration [illegible] | IPR006029 (SUPERFAMILY); IPR006201 (PRINTS); IPR006202 (G3DSA:2.70.170.GENE3D); IPR018000 (PROSITE); IPR027361 (G3DSA:1.20.120.GENE3D); SignalP-NN(euk) (SIGNALP), tmhmm (TMHMM) | Yes |

| | | | | | | | | | | |
|---|---|---|---|---|---|---|---|---|---|---|
| 395 | lib3_tri_Filter15_IDBA_contig_870_1 | 422 | cathepsin partial | 596 | 6.54E-66 | 82.85% | 3 | F:peptidase activity; P:protein metabolic process; P:catabolic process | IPR000668 (PFAM); IPR013128 (PANTHER); IPR025660 (PROSITE); IPR025661 (PROSITE); G3DSA:3.90.70.10 (GENE3D), PTHR12411:SF149 (PANTHER), tmhmm (TMHMM), SSF54001 (SUPERFAMILY) | |
| 396 | lib3_tri_Filter15_IDBA_contig_8767_4 | 11 | | 106 | | | | | IPR003961 (PFAM); IPR013783 (G3DSA:2.60.40.GENE3D); PR00014 (PRINTS), PTHR10489 (PANTHER) | |
| 397 | lib3_tri_Filter15_IDBA_contig_8771_0 | 25 | ---NA--- | 106 | | | 0 | - | IPR008160 (PFAM); PTHR24023 (PANTHER) | |
| 398 | lib3_tri_Filter15_IDBA_contig_8771_4 | 25 | ---NA--- | 106 | | | 0 | - | no IPS match | |
| 399 | lib3_tri_Filter15_IDBA_contig_8825_5 | 19 | paramyosin | 105 | 1.18E-45 | 87.75% | 5 | C:cytoplasm; C:extracellular region; C:cytoskeleton; C:protein complex; F:motor activity | IPR002928 (PFAM); PTHR13140 (PANTHER), PTHR13140:SF106 (PANTHER) | |
| 400 | lib3_tri_Filter15_IDBA_contig_891_5 | 236 | a chain structural analysis of neprilysin with various specific and potent inhibitors | 512 | 2.32E-92 | 59.85% | 13 | P:biological_process; C:cell; F:binding; C:cytoplasmic membrane-bounded vesicle; P:response to abiotic stimulus; F:peptidase activity; P:protein metabolic process; P:catabolic process; ; P:metabolic process; F:protein binding; P:multicellular organismal development; C:plasma membrane | IPR000718 (PANTHER); IPR008753 (PFAM); IPR018497 (PRINTS); IPR024079 (G3DSA:3.40.390.GENE3D); SSF55486 (SUPERFAMILY) | |
| 401 | lib3_tri_Filter15_IDBA_contig_8966_4 | 26 | deleted in malignant brain tumors 1 | 105 | 3.63E-10 | 69.53% | 9 | F:scavenger receptor activity; C:membrane; C:extracellular matrix; P:plasma lipoprotein particle clearance; P:lipoprotein transport; P:positive regulation of macrophage derived foam cell differentiation; P:cholesterol transport; P:positive regulation of cholesterol storage; F:low-density lipoprotein particle binding | IPR001190 (PRINTS); IPR017448 (SUPERFAMILY); G3DSA:3.10.250.10 (GENE3D), PTHR19331 (PANTHER), PTHR19331:SF118 (PANTHER), SignalP-NN(euk) (SIGNALP), tmhmm (TMHMM) | Yes |
| 402 | lib3_tri_Filter15_IDBA_contig_8981_5 | 12 | cathepsin l-like | 104 | 1.89E-46 | 84.45% | 9 | C:cytoplasm; F:structural molecule activity; C:lysosome; P:protein metabolic process; P:catabolic process; P:anatomical structure morphogenesis; P:multicellular organismal development; P:cell death; F:peptidase activity | IPR000169 (PROSITE); IPR000668 (PFAM); IPR013128 (PANTHER); G3DSA:3.90.70.10 (GENE3D), PTHR12411:SF149 (PANTHER), SSF54001 (SUPERFAMILY) | |
| 403 | lib3_tri_Filter15_IDBA_contig_8992_4 | 11 | contactin-3 | 104 | 9.84E-14 | 49.00% | 3 | P:nervous system development; P:cell-cell adhesion; C:anchored to plasma membrane | IPR003599 (SMART); IPR007110 (PROFILE); IPR013098 (PFAM); IPR013783 (G3DSA:2.60.40.GENE3D); PTHR10489 (PANTHER), PTHR10489:SF54 (PANTHER), SSF48726 (SUPERFAMILY) | |
| 404 | lib3_tri_Filter15_IDBA_contig_8995_0 | 20 | ---NA--- | 105 | | | 0 | - | no IPS match | |
| 405 | lib3_tri_Filter15_IDBA_contig_8995_5 | 20 | ---NA--- | 104 | | | 0 | - | IPR008160 (PFAM); PTHR24023 (PANTHER) | |
| 406 | lib3_tri_Filter15_IDBA_contig_9063_0 | 8 | PREDICTED: hemicentin-1 | 104 | 1.89E-07 | 70.00% | 0 | - | IPR001881 (PFAM); IPR018097 (PROSITE); G3DSA:2.10.25.10 (GENE3D), PTHR24034 (PANTHER), tmhmm (TMHMM), SSF57196 (SUPERFAMILY) | |
| 407 | lib3_tri_Filter15_IDBA_contig_9065_1 | 15 | ---NA--- | 104 | | | 0 | - | no IPS match | |
| 408 | lib3_tri_Filter15_IDBA_contig_9065_3 | 15 | ---NA--- | 104 | | | 0 | - | IPR008160 (PFAM); PTHR24023 (PANTHER) | |

| | | | | | | | | | |
|---|---|---|---|---|---|---|---|---|---|
| 409 | lib3_tri_Filter15_IDBA_contig_9177_4 | 15 | protein unc- isoform f | 103 | 7.45E-11 | 60.20% | 10 | F:transferase activity; P:biological_process; C:cytoplasm; F:protein binding; P:signal transduction; P:anatomical structure morphogenesis; P:cell differentiation; P:multicellular organismal development; P:cytoskeleton organization; F:enzyme regulator activity | IPR003598 (SMART); IPR007110 (PROFILE); IPR013098 (PFAM); IPR013783 (G3DSA:2.60.40.GENE3D); IPR015726 (PTHR22964:PANTHER); IPR020675 (PANTHER); SSF48726 (SUPERFAMILY) |
| 410 | lib3_tri_Filter15_IDBA_contig_9182_5 | 7 | peptidyl-prolyl cis-trans isomerase b-like | 103 | 1.15E-22 | 58.10% | 5 | F:peptidyl-prolyl cis-trans isomerase activity; P:protein folding; P:protein peptidyl-prolyl isomerization; F:isomerase activity; C:Golgi apparatus | IPR002130 (PRINTS); G3DSA:2.40.100.10 (GENE3D), PTHR11071 (PANTHER), PTHR11071:SF11 (PANTHER) |
| 411 | lib3_tri_Filter15_IDBA_contig_9204_4 | 15 | matrix metalloproteinase-9 precursor | 103 | 2.60E-08 | 56.60% | 16 | P:extracellular matrix organization; P:positive regulation of apoptotic process; P:positive regulation of keratinocyte migration; F:metalloendopeptidase activity; P:skeletal system development; F:hydrolase activity; P:embryo implantation; F:zinc ion binding; F:metallopeptidase activity; C:extracellular matrix; F:peptidase activity; P:proteolysis; P:collagen catabolic process; F:metal ion binding; P:metabolic process; C:extracellular space | IPR000562 (G3DSA:2.10.10.GENE3D); IPR013806 (SUPERFAMILY); PR00013 (PRINTS), PTHR22803 (PANTHER) |
| 412 | lib3_tri_Filter15_IDBA_contig_9275_3 | 9 | cartilage oligomeric matrix protein | 103 | 2.23E-16 | 71.05% | 13 | F:protein binding; P:anatomical structure morphogenesis; P:multicellular organismal development; P:growth; F:calcium ion binding; C:extracellular space; P:regulation of biological process; P:cell death; F:lipid binding; F:carbohydrate binding; C:proteinaceous extracellular matrix; F:structural molecule activity; P:cellular component organization | IPR003367 (PFAM); IPR017897 (PROFILE); G3DSA:4.10.1080.10 (GENE3D), PTHR10199 (PANTHER), SSF103647 (SUPERFAMILY) |
| 413 | lib3_tri_Filter15_IDBA_contig_9282_0 | 12 | puromycin-sensitive aminopeptidase | 103 | 9.89E-37 | 81.00% | 9 | P:response to stress; P:cellular protein modification process; F:peptidase activity; P:biological_process; P:protein metabolic process; P:catabolic process; C:nucleus; C:cytosol; F:binding | IPR001930 (PANTHER); IPR015568 (PTHR11533:PANTHER); IPR024571 (PFAM) |
| 414 | lib3_tri_Filter15_IDBA_contig_9289_4 | 14 | 78 kda glucose-regulated partial | 103 | 2.18E-58 | 94.00% | 30 | C:cell; C:endoplasmic reticulum; C:protein complex; F:protein binding; P:signal transduction; P:anatomical structure morphogenesis; P:multicellular organismal development; P:regulation of biological process; P:embryo development; P:nucleobase-containing compound metabolic process; P:catabolic process; P:response to external stimulus; P:response to stress; P:cellular protein modification process; F:enzyme regulator activity; P:protein metabolic process; P:cell death; P:response to biotic stimulus; C:nucleus; C:cytosol; P:transport; C:cytoplasm; F:binding; P:cell communication; F:nucleotide binding; F:hydrolase activity; C:cytoplasmic membrane-bounded vesicle; P:metabolic process; F:calcium ion binding; | IPR013126 (PRINTS); G3DSA:3.30.420.40 (GENE3D), PTHR19375 (PANTHER), PTHR19375:SF1 (PANTHER), SSF53067 (SUPERFAMILY) |

| | | | | | | | | | | |
|---|---|---|---|---|---|---|---|---|---|---|
| 415 | lib3_tri_Filter15_IDBA_contig_928_0 | 598 | histone h2a | 798 | 6.84E-62 | 93.10% | 7 | C:chromosome; P:DNA metabolic process; P:cell cycle; P:response to stress; F:DNA binding; P:organelle organization; C:nucleus | IPR002119 (PRINTS); IPR007125 (PFAM); IPR009072 (G3DSA:1.10.20.GENE3D); PTHR23430 (PANTHER), tmhmm (TMHMM) | |
| 416 | lib3_tri_Filter15_IDBA_contig_9309_3 | 5 | c3 and pzp-like alpha-2-macroglobulin domain-containing protein 8 | 103 | 8.11E-21 | 56.95% | 2 | C:extracellular space; F:endopeptidase inhibitor activity | IPR022041 (PFAM) | |
| 417 | lib3_tri_Filter15_IDBA_contig_9321_2 | 40 | transmembrane protease serine 5 | 102 | 2.38E-23 | 66.90% | 1 | F:peptidase activity | IPR001254 (PFAM); IPR009003 (SUPERFAMILY); G3DSA:2.40.10.10 (GENE3D), PTHR24264 (PANTHER) | |
| 418 | lib3_tri_Filter15_IDBA_contig_9340_5 | 39 | | 131 | | | | | IPR003961 (PFAM); IPR013783 (G3DSA:2.60.40.GENE3D); IPR020675 (PANTHER); PR00014 (PRINTS) | |
| 419 | lib3_tri_Filter15_IDBA_contig_938_3 | 404 | deleted in malignant brain tumors 1 | 475 | 9.16E-91 | 54.50% | 2 | F:scavenger receptor activity; C:membrane | IPR001190 (PRINTS); IPR017448 (SMART); G3DSA:3.10.250.10 (GENE3D), PTHR19331 (PANTHER) | |
| 420 | lib3_tri_Filter15_IDBA_contig_9410_3 | 46 | ---NA--- | 205 | | | 0 | - | SignalP-NN(euk) (SIGNALP), tmhmm (TMHMM) | Yes |
| 421 | lib3_tri_Filter15_IDBA_contig_947_0 | 1348 | cystatin-c-like | 467 | 9.19E-21 | 52.40% | 7 | P:proteolysis; F:peptidase inhibitor activity; F:cysteine-type endopeptidase inhibitor activity; F:peptidase activity; P:negative regulation of peptidase activity; P:negative regulation of endopeptidase activity; C:cellular_component | IPR000010 (PFAM); IPR018073 (PROSITE); IPR027214 (PANTHER); G3DSA:3.10.450.10 (GENE3D), PTHR11413:SF23 (PANTHER), SSF54403 (SUPERFAMILY) | |
| 422 | lib3_tri_Filter15_IDBA_contig_9576_2 | 10 | ---NA--- | 100 | | | 0 | - | IPR008160 (PFAM); PTHR24023 (PANTHER) | |
| 423 | lib3_tri_Filter15_IDBA_contig_9576_4 | 10 | ---NA--- | 101 | | | 0 | - | PTHR24023 (PANTHER), PTHR24023:SF106 (PANTHER) | |
| 424 | lib3_tri_Filter15_IDBA_contig_9592_1 | 10 | myosin-binding protein cardiac-type | 100 | 1.36E-25 | 58.55% | 1 | F:transferase activity | IPR003598 (SMART); IPR003599 (SMART); IPR007110 (PROFILE); IPR013098 (PFAM); IPR013783 (G3DSA:2.60.40.GENE3D); IPR015621 (PANTHER); SSF48726 (SUPERFAMILY) | |
| 425 | lib3_tri_Filter15_IDBA_contig_959_3 | 337 | superoxide dismutase | 537 | 9.75E-74 | 79.55% | 4 | F:binding; C:cytoplasm; P:metabolic process; F:catalytic activity | IPR001424 (PRINTS); IPR018152 (PROSITE); IPR024134 (PANTHER); SignalP-NN(euk) (SIGNALP), tmhmm (TMHMM) | Yes |
| 426 | lib3_tri_Filter15_IDBA_contig_9609_5 | 6 | trans-l-3-hydroxyproline dehydratase | 100 | 1.29E-22 | 72.50% | 1 | F:catalytic activity | IPR008794 (PFAM); G3DSA:3.10.310.10 (GENE3D), PTHR32298 (PANTHER), PTHR32298:SF0 (PANTHER), SSF54506 (SUPERFAMILY) | |
| 427 | lib3_tri_Filter15_IDBA_contig_9611_2 | 23 | ppib protein | 100 | 1.77E-47 | 82.45% | 8 | C:extracellular space; P:protein metabolic process; C:cytoskeleton; C:protein complex; P:reproduction; C:lipid particle; P:cellular protein modification process; F:catalytic activity | IPR002130 (PRINTS); IPR020892 (PROSITE); G3DSA:2.40.100.10 (GENE3D), PTHR11071 (PANTHER) | |
| 428 | lib3_tri_Filter15_IDBA_contig_961_3 | 4014 | peroxiredoxin 6 | 461 | 8.60E-112 | 79.70% | 3 | P:metabolic process; F:catalytic activity; F:antioxidant activity | IPR000866 (PFAM); IPR012336 (G3DSA:3.40.30.GENE3D); IPR019479 (PFAM); G3DSA:3.30.1020.10 (GENE3D), PTHR10681 (PANTHER), tmhmm (TMHMM) | |
| 429 | lib3_tri_Filter15_IDBA_contig_9634_5 | 12 | kex2-like endoprotease | 100 | 6.05E-22 | 73.45% | 1 | F:peptidase activity | IPR000209 (G3DSA:3.40.50.GENE3D); IPR015500 (PANTHER); IPR023828 (PROSITE); PTHR10795:SF93 (PANTHER) | |
| 430 | lib3_tri_Filter15_IDBA_contig_9640_0 | 13 | multiple epidermal growth factor-like domains protein 10-like | 101 | 3.02E-07 | 50.50% | 0 | - | PTHR24035 (PANTHER), PTHR24035:SF4 (PANTHER) | |
| 431 | lib3_tri_Filter15_IDBA_contig_9737_3 | 19 | puromycin-sensitive aminopeptidase | 125 | 1.61E-39 | 74.55% | 1 | F:peptidase activity | IPR001930 (PANTHER); IPR015568 (PTHR11533:PANTHER); IPR024571 (PFAM) | |

| | | | | | | | | | | |
|---|---|---|---|---|---|---|---|---|---|---|
| 432 | lib3_tri_Filter15_IDBA_contig_9745_0 | 25 | muscle m-line assembly protein unc-89 | 155 | 5.16E-14 | 45.00% | 1 | P:biological_process | IPR013098 (PFAM); IPR013783 (G3DSA:2.60.40.GENE3D); PTHR10489 (PANTHER), SSF48726 (SUPERFAMILY) | |
| 433 | lib3_tri_Filter15_IDBA_contig_975_2 | 122 | ---NA--- | 453 | | | 0 | - | IPR008160 (PFAM); PTHR24023 (PANTHER) | |
| 434 | lib3_tri_Filter15_IDBA_contig_975_3 | 122 | ---NA--- | 454 | | | 0 | - | no IPS match | |
| 435 | lib3_tri_Filter15_IDBA_contig_978_0 | 93 | ---NA--- | 451 | | | 0 | - | IPR008160 (PFAM); PTHR24023 (PANTHER) | |
| 436 | lib3_tri_Filter15_IDBA_contig_978_1 | 93 | ---NA--- | 450 | | | 0 | - | no IPS match | |
| 437 | lib3_tri_Filter15_IDBA_contig_978_5 | 93 | ---NA--- | 450 | | | 0 | - | no IPS match | |
| 438 | lib3_tri_Filter15_IDBA_contig_979_5 | 538 | von willebrand factor type egf and pentraxin domain-containing protein 1 precursor | 699 | 5.60E-37 | 52.00% | 2 | P:cell-matrix adhesion; F:calcium ion binding | IPR000742 (SMART); IPR009030 (SUPERFAMILY); IPR013032 (PROSITE); PTHR24035 (PANTHER), PTHR24035:SF4 (PANTHER) | |
| 439 | lib3_tri_Filter15_IDBA_contig_97_3 | 506 | stress-70 mitochondrial | 939 | 9.99E-120 | 92.70% | 11 | P:protein metabolic process; C:nucleolus; P:protein transport; C:mitochondrion; P:mitochondrion organization; C:cell; P:regulation of biological process; P:cell death; F:nucleotide binding; F:protein binding; P:response to stress | IPR013126 (PRINTS); G3DSA:1.20.1270.10 (GENE3D), G3DSA:2.60.34.10 (GENE3D), PTHR19375 (PANTHER), PTHR19375:SF1 (PANTHER), tmhmm (TMHMM), SSF100920 (SUPERFAMILY), SSF100934 (SUPERFAMILY) | |
| 440 | lib3_tri_Filter15_IDBA_contig_9815_2 | 1815 | hypothetical protein CAPTEDRAFT_228840 | 190 | 5.49E-08 | 52.67% | 6 | F:calcium ion binding; P:homophilic cell adhesion; C:integral to membrane; P:cell adhesion; C:membrane; C:plasma membrane | IPR000742 (PFAM); IPR013032 (PROSITE); G3DSA:2.10.25.10 (GENE3D), PTHR24044 (PANTHER), PTHR24044:SF67 (PANTHER), SignalP-NN(euk) (SIGNALP), tmhmm (TMHMM), SSF57196 (SUPERFAMILY) | Yes |
| 441 | lib3_tri_Filter15_IDBA_contig_9816_0 | 1784 | hypothetical protein CAPTEDRAFT_228840 | 189 | 5.47E-08 | 52.67% | 6 | F:calcium ion binding; P:homophilic cell adhesion; C:integral to membrane; P:cell adhesion; C:membrane; C:plasma membrane | IPR000742 (PFAM); IPR013032 (PROSITE); G3DSA:2.10.25.10 (GENE3D), PTHR24044 (PANTHER), PTHR24044:SF67 (PANTHER), SignalP-NN(euk) (SIGNALP), tmhmm (TMHMM), SSF57196 (SUPERFAMILY) | Yes |
| 442 | lib3_tri_Filter15_IDBA_contig_982_1 | 601 | adam family mig-17 | 500 | 1.48E-98 | 46.85% | 8 | P:proteolysis; F:metallopeptidase activity; F:hydrolase activity; C:extracellular matrix; F:zinc ion binding; F:peptidase activity; C:proteinaceous extracellular matrix; F:metalloendopeptidase activity | IPR001590 (PROFILE); IPR024079 (G3DSA:3.40.390.GENE3D); PTHR13723 (PANTHER), PF13574 (PFAM), SSF55486 (SUPERFAMILY) | |
| 443 | lib3_tri_Filter15_IDBA_contig_9835_0 | 38 | | 159 | | | | | IPR007110 (PROFILE); IPR013098 (PFAM); IPR013783 (G3DSA:2.60.40.GENE3D); IPR020675 (PANTHER); IPR020682 (PTHR22964:PANTHER); SSF48726 (SUPERFAMILY) | |

## Supplementary Table 5

| Nr. | Contig sequence name | Number of reads | Sequence description (BLAST nr, E-value= > 0.0001) | Length (aa pos.) | E-Value | mean Similarity | Nr. of GO terms | Gene ontology terms and annotation (GO's) | Interpro scan results (BLAST2GO) | SignalP 4.1 (3.0) |
|---|---|---|---|---|---|---|---|---|---|---|
| 1 | lib_fal_Filter15_IDBA_contig_0_minus3 | 7589 | | 4189 | 0 | 100% | 1 | F:protein binding | IPR003598 (SMART); IPR003599 (SMART); IPR007110 (PROFILE); IPR013098 (PFAM); IPR013783 (G3DSA:2.60.40.GENE3D); tmhmm (TMHMM), SSF48726 (SUPERFAMILY) | |
| 2 | lib_fal_Filter15_IDBA_contig_1_plus1 | 5336 | | 3314 | 0 | 100% | 4 | F:nucleotide binding; P:cellular protein modification process; F:protein binding; F:protein kinase activity | IPR000719 (PFAM); IPR002290 (SMART); IPR003598 (SMART); IPR003599 (SMART); IPR003961 (PFAM); IPR007110 (PROFILE); IPR008271 (PROSITE); IPR011009 (SUPERFAMILY); IPR013098 (PFAM); IPR013783 (G3DSA:2.60.40.GENE3D); IPR017441 (PROSITE); IPR020635 (SMART); IPR020675 (PANTHER); PR00014 (PRINTS), G3DSA:1.10.510.10 (GENE3D), G3DSA:3.30.200.20 (GENE3D), tmhmm (TMHMM), SSF48726 (SUPERFAMILY) | |
| 3 | lib_fal_Filter15_IDBA_contig_1028_plus3 | 82 | kn motif and ankyrin repeat domain-containing protein 1 | 352 | 4.91E-94 | 75.75% | 11 | P:regulation of biological process; C:plasma membrane; P:signal transduction; C:cytoplasm; P:response to endogenous stimulus; P:cellular component organization; P:cell differentiation; P:multicellular organismal development; F:protein binding; P:cytoskeleton organization; C:nucleus | IPR002110 (PRINTS); IPR020683 (G3DSA:1.25.40.GENE3D); PTHR24168 (PANTHER) | |
| 4 | lib_fal_Filter15_IDBA_contig_1086_plus1 | 171 | phosphoglycerate kinase 1 | 341 | 0 | 86.00% | 9 | C:cell; P:biosynthetic process; P:carbohydrate metabolic process; F:nucleotide binding; F:kinase activity; P:metabolic process; P:generation of precursor metabolites and energy; P:catabolic process; C:cytosol | IPR001576 (PRINTS); IPR015824 (G3DSA:3.40.50.GENE3D); IPR015901 (G3DSA:3.40.50.GENE3D); PTHR11406:SF1 (PANTHER) | |
| 5 | lib_fal_Filter15_IDBA_contig_1092_minus2 | 58 | tyrosine-protein phosphatase non-receptor type 13 | 339 | 1.85E-59 | 65.15% | 7 | C:cytoplasm; F:phosphoprotein phosphatase activity; C:cell; C:plasma membrane; F:protein binding; C:nucleus; C:cytoskeleton | IPR000299 (PROFILE); IPR001478 (PFAM); IPR011993 (G3DSA:2.30.29.GENE3D); IPR018980 (PFAM); G3DSA:2.30.42.10 (GENE3D), PTHR19964 (PANTHER), PTHR19964:SF7 (PANTHER), SSF50729 (SUPERFAMILY) | |
| 6 | lib_fal_Filter15_IDBA_contig_1097_minus1 | 78 | tyrosine-protein phosphatase non-receptor type 13 | 338 | 8.58E-27 | 44.15% | 2 | C:intracellular; F:protein binding | IPR001478 (PFAM); G3DSA:2.30.42.10 (GENE3D), PTHR19964 (PANTHER), PTHR19964:SF7 (PANTHER), tmhmm (TMHMM) | |
| 7 | lib_fal_Filter15_IDBA_contig_1174_minus2 | 86 | | 208 | 0 | 100% | 1 | F:protein binding | IPR003599 (SMART); IPR003961 (PFAM); IPR007110 (PROFILE); IPR013098 (PFAM); IPR013783 (G3DSA:2.60.40.GENE3D); IPR020675 (PANTHER); PR00014 (PRINTS), SSF48726 (SUPERFAMILY) | |
| 8 | lib_fal_Filter15_IDBA_contig_1175_minus2 | 59 | | 325 | 0 | 100% | 1 | F:protein binding | IPR003961 (PFAM); IPR007110 (PROFILE); IPR013098 (PFAM); IPR013783 (G3DSA:2.60.40.GENE3D); PR00014 (PRINTS), SSF48726 (SUPERFAMILY) | |
| 9 | lib_fal_Filter15_IDBA_contig_1208_plus2 | 989 | carbonic anhydrase 2 | 787 | 1.48E-97 | 63.75% | 3 | F:catalytic activity; P:metabolic process; F:binding | IPR001148 (G3DSA:3.10.200.GENE3D); IPR018338 (PROSITE); IPR023561 (PANTHER) | |
| 10 | lib_fal_Filter15_IDBA_contig_1214_plus1 | 72 | ---NA--- | 317 | | | 0 | - | no IPS match | |
| 11 | lib_fal_Filter15_IDBA_contig_1214_plus3 | 72 | ---NA--- | 317 | | | 0 | - | IPR008160 (PFAM); PTHR24023 (PANTHER) | |
| 12 | lib_fal_Filter15_IDBA_contig_1218_plus1 | 1134 | peptidyl-prolyl cis-trans isomerase | 915 | 6.77E-83 | 75.25% | 3 | P:protein metabolic process; P:cellular protein modification process; F:catalytic activity | IPR002130 (PRINTS); IPR020892 (PROSITE); G3DSA:2.40.100.10 (GENE3D), PTHR11071 (PANTHER), SignalP-NN(euk) (SIGNALP), tmhmm (TMHMM) | Yes |

| | | | | | | | | | | |
|---|---|---|---|---|---|---|---|---|---|---|
| 13 | lib_fal_Filter15_IDBA_contig_1272_plus1 | 48 | c-type mannose receptor 2-like | 307 | 1.33E-20 | 42.60% | 1 | F:carbohydrate binding | IPR001304 (PFAM); IPR016186 (G3DSA:3.10.100.GENE3D); IPR016187 (SUPERFAMILY); IPR018378 (PROSITE); PTHR22803 (PANTHER), SignalP-NN(euk) (SIGNALP) | Yes |
| 14 | lib_fal_Filter15_IDBA_contig_13_minus1 | 8597 | beta-galactosidase ubiquitin fusion partial | 257 | 6.33E-48 | 95.50% | 3 | C:nucleus; F:protein binding; C:cytoplasm | IPR000626 (PFAM); IPR019954 (PROSITE); IPR019955 (PROFILE); IPR019956 (PRINTS); G3DSA:3.10.20.90 (GENE3D), PTHR10666 (PANTHER), SSF54236 (SUPERFAMILY) | |
| 15 | lib_fal_Filter15_IDBA_contig_1315_plus1 | 47 | kelch-like protein 26 | 302 | 2.80E-48 | 51.55% | 1 | F:protein binding | IPR000210 (SMART); IPR011333 (G3DSA:3.30.710.GENE3D); IPR011705 (PFAM); IPR013069 (PFAM); PTHR24412 (PANTHER) | |
| 16 | lib_fal_Filter15_IDBA_contig_1357_plus1 | 44 | fibrinogen c domain-containing protein 1 | 294 | 1.40E-97 | 59.50% | 7 | F:receptor binding; C:extracellular space; P:signal transduction; F:metal ion binding; F:chitin binding; C:integral to membrane; C:membrane | IPR002181 (PFAM); IPR014715 (G3DSA:4.10.530.GENE3D); IPR014716 (G3DSA:3.90.215.GENE3D); PTHR19143 (PANTHER) | |
| 17 | lib_fal_Filter15_IDBA_contig_1411_minus3 | 345 | kazal-type serine protease inhibitor domain-containing protein 1-like | 342 | 2.09E-86 | 57.45% | 4 | C:proteinaceous extracellular matrix; P:regulation of biological process; P:cell growth; F:protein binding | IPR000867 (PFAM); IPR002350 (PFAM); IPR003598 (SMART); IPR003599 (SMART); IPR007110 (PROFILE); IPR011390 (PANTHER); IPR013098 (PFAM); IPR013783 (G3DSA:2.60.40.GENE3D); G3DSA:3.30.60.30 (GENE3D), PTHR14186:SF7 (PANTHER), SSF100895 (SUPERFAMILY), SSF48726 (SUPERFAMILY) | |
| 18 | lib_fal_Filter15_IDBA_contig_1413_plus2 | 83 | plasma kallikrein | 286 | 2.42E-73 | 53.35% | 7 | P:protein metabolic process; P:regulation of biological process; P:response to external stimulus; P:response to stress; C:extracellular region; F:peptidase activity; P:catabolic process | IPR001254 (PFAM); IPR001314 (PRINTS); IPR009003 (SUPERFAMILY); IPR018114 (PROSITE); G3DSA:2.40.10.10 (GENE3D), PTHR24259 (PANTHER) | |
| 19 | lib_fal_Filter15_IDBA_contig_1431_plus2 | 67 | maltase a4 | 283 | 2.82E-79 | 57.70% | 3 | F:catalytic activity; P:carbohydrate metabolic process; F:binding | IPR006047 (PFAM); IPR006589 (SMART); IPR013781 (G3DSA:3.20.20.GENE3D); IPR015902 (PANTHER); IPR017853 (SUPERFAMILY); G3DSA:3.90.400.10 (GENE3D), PTHR10357:SF76 (PANTHER) | |
| 20 | lib_fal_Filter15_IDBA_contig_1439_plus2 | 52 | annexin a7 | 281 | 1.59E-112 | 72.90% | 17 | C:cytosol; P:cellular homeostasis; P:anatomical structure morphogenesis; P:cellular component organization; P:regulation of biological process; P:response to stress; P:response to abiotic stimulus; P:behavior; F:lipid binding; C:organelle; P:biological_process; F:protein binding; C:cell; C:nuclear envelope; P:cell proliferation; C:plasma membrane; F:calcium ion binding | IPR001464 (PRINTS); IPR018252 (PROSITE); IPR018502 (G3DSA:1.10.220.GENE3D) | |
| 21 | lib_fal_Filter15_IDBA_contig_1467_minus2 | 45 | proactivator polypeptide | 277 | 6.75E-70 | 63.00% | 2 | C:lysosome; P:lipid metabolic process | IPR003119 (PFAM); IPR007856 (PFAM); IPR008138 (PFAM); IPR008139 (SMART); IPR008373 (PRINTS); IPR011001 (G3DSA:1.10.225.GENE3D); PTHR11480 (PANTHER), PTHR11480:SF3 (PANTHER) | |
| 22 | lib_fal_Filter15_IDBA_contig_1484_plus1 | 304 | kielin chordin-like protein | 434 | 1.24E-35 | 38.75% | 2 | F:protein binding; F:enzyme regulator activity | IPR001007 (PROFILE); IPR006552 (SMART); IPR008037 (PFAM); IPR025615 (PFAM); PTHR11339 (PANTHER), PTHR11339:SF44 (PANTHER), SSF57603 (SUPERFAMILY) | |
| 23 | lib_fal_Filter15_IDBA_contig_1491_minus1 | 142 | peroxiredoxin 6 | 303 | 4.00E-107 | 78.20% | 3 | F:catalytic activity; F:antioxidant activity; P:metabolic process | IPR000866 (PFAM); IPR012336 (G3DSA:3.40.30.GENE3D); IPR019479 (PFAM); G3DSA:3.30.1020.10 (GENE3D), PTHR10681 (PANTHER) | |
| 24 | lib_fal_Filter15_IDBA_contig_1505_plus2 | 1196 | retinol-binding protein 1 | 414 | 2.21E-12 | 50.50% | 6 | F:lipid binding; F:binding; P:lipid metabolic process; P:secondary metabolic process; F:transporter activity; P:transport | IPR000463 (PRINTS); IPR011038 (SUPERFAMILY); IPR012674 (G3DSA:2.40.128.GENE3D); PTHR11955 (PANTHER), PTHR11955:SF47 (PANTHER) | |

| | | | | | | | | | | |
|---|---|---|---|---|---|---|---|---|---|---|
| 25 | lib_fal_Filter15_IDBA_contig_1519_minus2 | 130 | thioredoxin peroxidase | 336 | 2.49E-121 | 87.80% | 3 | P:metabolic process; F:catalytic activity; F:antioxidant activity | IPR000866 (PFAM); IPR012336 (G3DSA:3.40.30.GENE3D); IPR019479 (PFAM); PTHR10681 (PANTHER), PTHR10681:SF44 (PANTHER), SignalP-NN(euk) (SIGNALP) | Yes |
| 26 | lib_fal_Filter15_IDBA_contig_1528_plus3 | 261 | hypothetical protein AaeL_AAEL002257 | 393 | 1.54E-12 | 50.10% | 0 | - | IPR000716 (G3DSA:4.10.800.GENE3D); PTHR12352 (PANTHER), SignalP-NN(euk) (SIGNALP), tmhmm (TMHMM) | Yes |
| 27 | lib_fal_Filter15_IDBA_contig_1542_minus3 | 67 | | 270 | 0 | 100% | 1 | F:protein binding | IPR003598 (SMART); IPR003599 (SMART); IPR007110 (PROFILE); IPR013098 (PFAM); IPR013783 (G3DSA:2.60.40.GENE3D); IPR020675 (PANTHER); PTHR22964:SF3 (PANTHER), SSF48726 (SUPERFAMILY) | |
| 28 | lib_fal_Filter15_IDBA_contig_159_minus2 | 406 | ---NA--- | 578 | | | 0 | - | SignalP-NN(euk) (SIGNALP), tmhmm (TMHMM) | |
| 29 | lib_fal_Filter15_IDBA_contig_1594_plus1 | 76 | glucose-6-phosphate isomerase | 288 | 3.84E-164 | 86.30% | 12 | C:cytoplasm; P:anatomical structure morphogenesis; P:multicellular organismal development; P:biosynthetic process; P:carbohydrate metabolic process; C:extracellular space; F:catalytic activity; C:plasma membrane; F:receptor binding; P:generation of precursor metabolites and energy; P:catabolic process; C:nucleus | IPR001672 (PRINTS); IPR018189 (PROSITE); G3DSA:3.40.50.10490 (GENE3D), PTHR11469:SF0 (PANTHER), tmhmm (TMHMM), SSF53697 (SUPERFAMILY) | |
| 30 | lib_fal_Filter15_IDBA_contig_1595_plus3 | 18 | glucose-6-phosphate isomerase-like | 109 | 5.79E-12 | 87.15% | 6 | P:generation of precursor metabolites and energy; P:carbohydrate metabolic process; P:catabolic process; C:cytoplasm; F:catalytic activity; P:biosynthetic process | IPR001672 (PANTHER); IPR023096 (G3DSA:1.10.1390.GENE3D); PTHR11469:SF0 (PANTHER), SSF53697 (SUPERFAMILY) | |
| 31 | lib_fal_Filter15_IDBA_contig_1596_plus3 | 20 | glucose-6-phosphate isomerase | 102 | 6.75E-44 | 88.90% | 12 | C:cytoplasm; P:anatomical structure morphogenesis; P:multicellular organismal development; P:biosynthetic process; P:carbohydrate metabolic process; C:extracellular space; F:catalytic activity; C:plasma membrane; F:receptor binding; P:generation of precursor metabolites and energy; P:catabolic process; C:nucleus | IPR001672 (PRINTS); IPR018189 (PROSITE); IPR023096 (G3DSA:1.10.1390.GENE3D); G3DSA:3.40.50.10490 (GENE3D), PTHR11469:SF0 (PANTHER), SSF53697 (SUPERFAMILY) | |
| 32 | lib_fal_Filter15_IDBA_contig_1610_minus2 | 154 | heat shock protein 70 | 262 | 2.25E-112 | 95.40% | 25 | [illegible] component organization; P:transport; P:cell-cell signaling; C:cytoplasmic membrane-bounded vesicle; F:nucleotide binding; P:regulation of biological process; ; C:nucleolus; C:cytosol; P:cell cycle; F:protein binding; C:organelle; C:extracellular region; P:protein metabolic process; C:protein complex; F:hydrolase activity; P:nucleobase-containing compound metabolic process; P:catabolic process; C:plasma membrane; C:intracellular; P:response to stress; P:response to biotic stimulus; [illegible] | IPR013126 (PFAM); G3DSA:1.20.1270.10 (GENE3D), G3DSA:2.60.34.10 (GENE3D), PTHR19375 (PANTHER), SSF100920 (SUPERFAMILY), SSF100934 (SUPERFAMILY) | |

| | | | | | | | | | | |
|---|---|---|---|---|---|---|---|---|---|---|
| 33 | lib_fal_Filter15_IDBA_contig_162_minus1 | 6451 | y-box binding protein | 821 | 2.28E-53 | 88.90% | 19 | C:plasma membrane; P:regulation of biological process; ; F:protein binding; F:transcription regulator activity; P:cell death; C:intracellular; F:RNA binding; P:anatomical structure morphogenesis; P:multicellular organismal development; P:reproduction; F:DNA binding; C:cytoplasm; P:growth; P:response to stress; P:response to abiotic stimulus; F:sequence-specific DNA binding transcription factor activity; C:nucleus; P:embryo development | IPR002059 (PRINTS); IPR011129 (SMART); IPR012340 (G3DSA:2.40.50.GENE3D); IPR019844 (PROSITE); PTHR11544 (PANTHER), PTHR11544:SF8 (PANTHER), tmhmm (TMHMM) | |
| 34 | lib_fal_Filter15_IDBA_contig_163_minus3 | 269 | cysteine-rich secretory protein mr30 | 479 | 3.02E-43 | 44.75% | 1 | F:protein binding | IPR000742 (SMART); IPR001283 (PRINTS); IPR002413 (PRINTS); IPR013032 (PROSITE); IPR014044 (G3DSA:3.40.33.GENE3D); SSF57196 (SUPERFAMILY) | |
| 35 | lib_fal_Filter15_IDBA_contig_1654_plus1 | 61 | ---NA--- | 257 | | | 0 | - | G3DSA:2.10.60.10 (GENE3D), SSF57302 (SUPERFAMILY) | |
| 36 | lib_fal_Filter15_IDBA_contig_1743_minus1 | 111 | chymotrypsinogen 2-like | 351 | 2.84E-58 | 57.45% | 3 | F:peptidase activity; P:protein metabolic process; P:catabolic process | IPR001254 (PFAM); IPR001314 (PRINTS); IPR009003 (SUPERFAMILY); IPR018114 (PROSITE); G3DSA:2.40.10.10 (GENE3D), PTHR24265 (PANTHER) | |
| 37 | lib_fal_Filter15_IDBA_contig_1747_plus1 | 947 | phosphoglycerate kinase | 732 | 2.05E-09 | 95.33% | 5 | P:generation of precursor metabolites and energy; P:carbohydrate metabolic process; P:catabolic process; P:metabolic process; F:kinase activity | IPR001576 (PANTHER); PTHR11406:SF1 (PANTHER) | |
| 38 | lib_fal_Filter15_IDBA_contig_1769_plus1 | 61 | protein-glutamine gamma-glutamyltransferase k | 248 | 9.22E-100 | 69.55% | 3 | F:metal ion binding; P:peptide cross-linking; F:protein-glutamine gamma-glutamyltransferase activity | IPR002931 (G3DSA:3.90.260.GENE3D); IPR023608 (PANTHER); PTHR11590:SF17 (PANTHER), SSF54001 (SUPERFAMILY) | |
| 39 | lib_fal_Filter15_IDBA_contig_1793_minus1 | 91 | nadh dehydrogenase isoform 1 | 246 | 1.15E-32 | 90.60% | 6 | P:generation of precursor metabolites and energy; P:biosynthetic process; P:lipid metabolic process; F:transporter activity; F:binding; C:mitochondrion | IPR003231 (PRODOM); IPR009081 (G3DSA:1.10.1200.GENE3D); PTHR20863 (PANTHER), PTHR20863:SF5 (PANTHER), tmhmm (TMHMM) | |
| 40 | lib_fal_Filter15_IDBA_contig_1837_minus2 | 25 | kelch-like protein 38 | 242 | 2.01E-50 | 58.95% | 1 | F:protein binding | IPR000210 (SMART); IPR011333 (G3DSA:3.30.710.GENE3D); IPR011705 (PFAM); IPR013069 (PFAM); PTHR24412 (PANTHER) | |
| 41 | lib_fal_Filter15_IDBA_contig_1838_minus1 | 140 | protease inhibitor epi11 | 268 | 7.77E-18 | 41.25% | 1 | F:protein binding | IPR002350 (PFAM); G3DSA:3.30.60.30 (GENE3D), PTHR10913 (PANTHER), tmhmm (TMHMM), SSF100895 (SUPERFAMILY) | |
| 42 | lib_fal_Filter15_IDBA_contig_1848_minus1 | 37 | fibrillin-1- partial | 241 | 5.98E-14 | 50.00% | 2 | F:protein binding; F:calcium ion binding | IPR000152 (PROSITE); IPR000742 (SMART); IPR001881 (PFAM); IPR013032 (PROSITE); IPR018097 (PROSITE); G3DSA:2.10.25.10 (GENE3D), PTHR24039 (PANTHER), SSF57196 (SUPERFAMILY) | |
| 43 | lib_fal_Filter15_IDBA_contig_1858_minus1 | 129 | neuronal acetylcholine receptor subunit alpha-7-like | 291 | 4.15E-32 | 49.00% | 3 | P:ion transport; C:cell; F:ion channel activity | IPR006201 (PANTHER); IPR006202 (G3DSA:2.70.170.GENE3D); PTHR18945:SF127 (PANTHER), SignalP-NN(euk) (SIGNALP), tmhmm (TMHMM) | Yes |
| 44 | lib_fal_Filter15_IDBA_contig_1887_plus3 | 42 | collagen alpha-1 chain | 237 | 1.09E-16 | 50.65% | 1 | F:protein binding | IPR002035 (G3DSA:3.40.50.GENE3D); PTHR22992 (PANTHER), SSF53300 (SUPERFAMILY) | |
| 45 | lib_fal_Filter15_IDBA_contig_1915_plus2 | 695 | angiopoietin-related protein 1 | 389 | 4.05E-36 | 59.45% | 4 | F:receptor binding; P:signal transduction; P:transmembrane receptor protein tyrosine kinase signaling pathway; C:extracellular space | IPR002181 (PFAM); IPR014715 (G3DSA:4.10.530.GENE3D); IPR014716 (G3DSA:3.90.215.GENE3D); PTHR19143 (PANTHER), SignalP-NN(euk) (SIGNALP) | Yes |
| 46 | lib_fal_Filter15_IDBA_contig_196_minus1 | 25320 | paramyosin | 832 | 0 | 72.10% | 4 | C:cytoskeleton; C:protein complex; C:cytoplasm; F:motor activity | IPR002928 (PFAM); PTHR13140 (PANTHER), PTHR13140:SF106 (PANTHER) | |

| | | | | | | | | | | |
|---|---|---|---|---|---|---|---|---|---|---|
| 47 | lib_fal_Filter15_IDBA_contig_2_minus2 | 2569 | unc- isoform b | 2627 | 0 | 53.30% | 4 | F:nucleotide binding; P:cellular protein modification process; F:protein binding; F:protein kinase activity | IPR000719 (PFAM); IPR002290 (SMART); IPR003598 (SMART); IPR003599 (SMART); IPR003961 (PFAM); IPR007110 (PROFILE); IPR011009 (SUPERFAMILY); IPR013098 (PFAM); IPR013783 (G3DSA:2.60.40.GENE3D); IPR020635 (SMART); IPR020675 (PANTHER); G3DSA:1.10.510.10 (GENE3D), G3DSA:3.30.200.20 (GENE3D), PTHR22964:SF6 (PANTHER), tmhmm (TMHMM), SSF48726 (SUPERFAMILY) | |
| 48 | lib_fal_Filter15_IDBA_contig_2021_minus3 | 351 | protease inhibitor epi11 | 449 | 5.53E-29 | 41.20% | 7 | P:metabolic process; P:regulation of biological process; P:primary metabolic process; C:extracellular region; P:cellular component organization; P:biological_process; F:protein binding | IPR002350 (PFAM); G3DSA:1.10.1890.10 (GENE3D), G3DSA:3.30.60.30 (GENE3D), PTHR10913 (PANTHER), PTHR10913:SF21 (PANTHER), SSF100895 (SUPERFAMILY) | |
| 49 | lib_fal_Filter15_IDBA_contig_2126_plus2 | 25 | hypothetical protein CAPTEDRAFT_224948 | 220 | 3.19E-10 | 67.50% | 0 | - | no IPS match | |
| 50 | lib_fal_Filter15_IDBA_contig_2131_plus1 | 67 | aael008062- partial | 219 | 4.87E-76 | 85.65% | 5 | C:extracellular region; F:receptor binding; P:biological_process; P:regulation of biological process; F:calcium ion binding | IPR008859 (PFAM); IPR008985 (SUPERFAMILY); IPR013320 (G3DSA:2.60.120.GENE3D); PTHR10199 (PANTHER) | |
| 51 | lib_fal_Filter15_IDBA_contig_217_minus1 | 4306 | paramyosin | 260 | 3.07E-63 | 69.70% | 4 | C:cytoskeleton; C:protein complex; C:cytoplasm; F:motor activity | IPR002928 (PFAM); G3DSA:1.20.5.340 (GENE3D), PTHR13140 (PANTHER), PTHR13140:SF106 (PANTHER) | |
| 52 | lib_fal_Filter15_IDBA_contig_2193_plus2 | 146 | dipeptidyl peptidase 1 | 256 | 7.15E-79 | 73.00% | 11 | C:lysosome; F:protein binding; F:binding; P:protein metabolic process; P:catabolic process; F:peptidase activity; P:regulation of biological process; P:cell death; P:biological_process; C:endoplasmic reticulum; C:Golgi apparatus | IPR000169 (PROSITE); IPR000668 (PRINTS); IPR013128 (PANTHER); IPR025661 (PROSITE); G3DSA:3.90.70.10 (GENE3D), PTHR12411:SF17 (PANTHER), SSF54001 (SUPERFAMILY) | |
| 53 | lib_fal_Filter15_IDBA_contig_2194_plus1 | 152 | dipeptidyl peptidase 1 | 275 | 1.34E-88 | 72.90% | 11 | C:lysosome; F:protein binding; F:binding; P:protein metabolic process; P:catabolic process; F:peptidase activity; P:regulation of biological process; P:cell death; P:biological_process; C:endoplasmic reticulum; C:Golgi apparatus | IPR000169 (PROSITE); IPR000668 (PRINTS); IPR013128 (PANTHER); IPR025661 (PROSITE); G3DSA:3.90.70.10 (GENE3D), PTHR12411:SF17 (PANTHER), SSF54001 (SUPERFAMILY) | |
| 54 | lib_fal_Filter15_IDBA_contig_2199_minus3 | 414 | cystatin precursor | 330 | 3.32E-25 | 53.00% | 24 | C:proteinaceous extracellular matrix; P:regulation of biological process; P:cell death; C:organelle; C:nuclear envelope; P:response to stress; C:extracellular space; C:lysosome; P:behavior; P:cell proliferation; P:multicellular organismal development; C:cell; P:biological_process; P:response to external stimulus; C:endosome; P:reproduction; P:cell differentiation; F:protein binding; C:cytoplasm; C:endoplasmic reticulum; P:DNA metabolic process; P:biosynthetic process; P:response to endogenous stimulus; F:enzyme regulator activity | IPR000010 (PFAM); IPR027214 (PANTHER); G3DSA:3.10.450.10 (GENE3D), PTHR11413:SF23 (PANTHER), SignalP-NN(euk) (SIGNALP), tmhmm (TMHMM), SSF54403 (SUPERFAMILY) | Yes |
| 55 | lib_fal_Filter15_IDBA_contig_2226_plus1 | 112 | serine proteinase inhibitor | 348 | 7.35E-30 | 48.35% | 1 | F:protein binding | IPR002350 (PFAM); G3DSA:1.10.1890.10 (GENE3D), G3DSA:3.30.60.30 (GENE3D), PTHR10913 (PANTHER), PTHR10913:SF21 (PANTHER), SignalP-NN(euk) (SIGNALP), SSF100895 (SUPERFAMILY) | Yes |
| 56 | lib_fal_Filter15_IDBA_contig_2283_minus3 | 71 | protein-glutamine gamma-glutamyltransferase k | 208 | 3.84E-33 | 51.75% | 5 | F:binding; P:cell differentiation; P:multicellular organismal development; F:transferase activity; P:cellular protein modification process | IPR008958 (PFAM); IPR013783 (G3DSA:2.60.40.GENE3D); IPR023608 (PANTHER) | |

| | | | | | | | | | |
|---|---|---|---|---|---|---|---|---|---|
| 57 | lib_fal_Filter15_IDBA_contig_2313_minus2 | 22 | nicotinic acetylcholine receptor alpha 3 subunit | 207 | 9.89E-31 | 71.80% | 4 | F:ion channel activity; C:cellular_component; P:ion transport; C:cell | IPR006201 (PANTHER); IPR006202 (G3DSA:2.70.170.GENE3D); PTHR18945:SF88 (PANTHER) |
| 58 | lib_fal_Filter15_IDBA_contig_2332_plus2 | 35 | | 206 | 0 | 100% | 1 | F:protein binding | IPR003961 (PFAM); IPR013783 (G3DSA:2.60.40.GENE3D); PR00014 (PRINTS) |
| 59 | lib_fal_Filter15_IDBA_contig_2342_minus1 | 28 | mam domain-containing protein 2 | 206 | 3.95E-24 | 49.20% | 1 | C:cell | IPR000998 (PFAM); IPR008985 (SUPERFAMILY); PTHR23282 (PANTHER) |
| 60 | lib_fal_Filter15_IDBA_contig_2344_plus3 | 93 | pdlim7 protein | 233 | 2.66E-21 | 58.80% | 12 | C:plasma membrane; F:protein binding; C:cytoskeleton; C:protein complex; P:transport; P:cellular component organization; P:embryo development; P:anatomical structure morphogenesis; P:multicellular organismal development; P:regulation of biological process; P:cell differentiation; C:cytoplasm | IPR001478 (PFAM); G3DSA:2.30.42.10 (GENE3D), PTHR24214 (PANTHER), SignalP-NN(euk) (SIGNALP) |
| 61 | lib_fal_Filter15_IDBA_contig_2376_plus3 | 39 | von willebrand factor type egf and pentraxin domain-containing protein 1 | 204 | 6.65E-12 | 53.75% | 6 | F:calcium ion binding; F:chromatin binding; F:carbohydrate binding; F:G-protein coupled receptor activity; C:membrane; P:G-protein coupled receptor signaling pathway | IPR000436 (PFAM); G3DSA:2.10.70.10 (GENE3D), PTHR19325 (PANTHER) |
| 62 | lib_fal_Filter15_IDBA_contig_242_minus1 | 215 | paramyosin- partial | 164 | 4.71E-05 | 68.00% | 5 | C:myosin filament; C:myosin complex; F:motor activity; C:cytoplasm; C:myofibril | no IPS match |
| 63 | lib_fal_Filter15_IDBA_contig_2467_plus2 | 46 | thioredoxin-dependent peroxide mitochondrial-like isoform 2 | 200 | 2.38E-57 | 89.85% | 20 | C:cytosol; C:protein complex; P:response to biotic stimulus; P:response to stress; P:catabolic process; F:protein binding; F:catalytic activity; F:antioxidant activity; P:metabolic process; P:regulation of biological process; F:enzyme regulator activity; P:cell proliferation; P:multicellular organismal development; P:cell death; P:cellular homeostasis; P:cell differentiation; C:endosome; P:mitochondrion organization; ; C:mitochondrion | IPR000866 (PFAM); IPR012336 (G3DSA:3.40.30.GENE3D); IPR019479 (PFAM); PTHR10681 (PANTHER), PTHR10681:SF45 (PANTHER) |
| 64 | lib_fal_Filter15_IDBA_contig_2537_minus1 | 39 | neurotransmitter-gated ion-channel ligand binding domain protein | 198 | 5.59E-68 | 62.45% | 3 | F:ion channel activity; P:ion transport; C:cell | IPR002394 (PRINTS); IPR006201 (PRINTS); IPR006202 (G3DSA:2.70.170.GENE3D); IPR018000 (PROSITE); PTHR18945:SF92 (PANTHER), tmhmm (TMHMM) |
| 65 | lib_fal_Filter15_IDBA_contig_254_minus1 | 1315 | antimicrobial-like peptide pp-1 | 646 | 4.90E-18 | 71.36% | 3 | P:response to stress; P:response to biotic stimulus; P:biological_process | no IPS match |
| 66 | lib_fal_Filter15_IDBA_contig_2562_plus1 | 161 | deleted in malignant brain tumors 1 | 251 | 2.40E-22 | 65.25% | 2 | F:receptor activity; C:cell | IPR001190 (PRINTS); IPR017448 (SMART); G3DSA:3.10.250.10 (GENE3D), PTHR19331 (PANTHER), PTHR19331:SF118 (PANTHER), SignalP-NN(euk) (SIGNALP), tmhmm (TMHMM) |

| | | | | | | | | | | |
|---|---|---|---|---|---|---|---|---|---|---|
| 67 | lib_fal_Filter15_IDBA_contig_2589_minus2 | 46 | paraoxonase 1 | 195 | 3.20E-09 | 53.15% | 22 | F:aryldialkylphosphatase activity; F:hydrolase activity; F:arylesterase activity; P:negative regulation of plasma lipoprotein particle oxidation; P:phosphatidylcholine metabolic process; P:carboxylic acid catabolic process; P:aromatic compound catabolic process; C:spherical high-density lipoprotein particle; C:high-density lipoprotein particle; P:positive regulation of binding; F:protein homodimerization activity; P:cholesterol metabolic process; C:extracellular space; F:calcium ion binding; P:positive regulation of cholesterol efflux; P:organophosphate catabolic process; P:response to external stimulus; P:response to toxin; C:microsome; P:positive regulation of transporter activity; C:extracellular region; | IPR011042 (G3DSA:2.120.10.GENE3D); PTHR11799 (PANTHER), PTHR11799:SF0 (PANTHER) | |
| 68 | lib_fal_Filter15_IDBA_contig_2619_plus1 | 24 | | 193 | 0 | 100% | 1 | F:protein binding | IPR003961 (PFAM); IPR013783 (G3DSA:2.60.40.GENE3D); PR00014 (PRINTS) | |
| 69 | lib_fal_Filter15_IDBA_contig_2627_plus1 | 44 | deleted in malignant brain tumors 1 | 193 | 2.71E-33 | 66.75% | 2 | F:receptor activity; C:cell | IPR001190 (PRINTS); IPR017448 (SMART); G3DSA:3.10.250.10 (GENE3D), PTHR19331 (PANTHER) | |
| 70 | lib_fal_Filter15_IDBA_contig_2653_plus2 | 29 | oligo- -glucosidase | 192 | 1.08E-65 | 75.40% | 3 | F:hydrolase activity; P:carbohydrate metabolic process; F:binding | IPR006047 (PFAM); IPR006589 (SMART); IPR013781 (G3DSA:3.20.20.GENE3D); IPR015902 (PANTHER); IPR017853 (SUPERFAMILY); G3DSA:3.90.400.10 (GENE3D), PTHR10357:SF11 (PANTHER), SignalP-NN(euk) (SIGNALP), tmhmm (TMHMM) | |
| 71 | lib_fal_Filter15_IDBA_contig_2689_minus3 | 807 | ribosomal protein ubq l40e | 190 | 2.67E-85 | 98.15% | 11 | C:cytoskeleton; C:protein complex; F:structural molecule activity; C:lipid particle; P:cellular protein modification process; P:protein metabolic process; P:catabolic process; C:ribosome; C:cytosol; P:translation; F:protein binding | IPR000626 (PFAM); IPR001975 (PFAM); IPR019954 (PROSITE); IPR019955 (PROFILE); IPR019956 (PRINTS); G3DSA:3.10.20.90 (GENE3D), PTHR10666 (PANTHER), SSF54236 (SUPERFAMILY) | |
| 72 | lib_fal_Filter15_IDBA_contig_269_minus3 | 3827 | glyceraldehyde-3-phosphate dehydrogenase | 148 | 2.78E-33 | 89.40% | 7 | C:cytoplasm; F:nucleotide binding; P:generation of precursor metabolites and energy; P:carbohydrate metabolic process; P:catabolic process; F:catalytic activity; P:metabolic process | IPR020829 (PFAM); IPR020831 (PANTHER); G3DSA:3.30.360.10 (GENE3D), SSF55347 (SUPERFAMILY) | |
| 73 | lib_fal_Filter15_IDBA_contig_2705_minus2 | 50 | protein disulfide isomerase | 201 | 5.24E-72 | 76.00% | 14 | C:extracellular space; P:protein metabolic process; C:cell; C:endoplasmic reticulum; C:cytoplasm; C:lipid particle; F:electron carrier activity; P:regulation of biological process; P:cellular homeostasis; P:metabolic process; C:nuclear envelope; C:organelle; C:intracellular; F:catalytic activity | IPR005746 (PRINTS); IPR005788 (TIGRFAMs); IPR012336 (G3DSA:3.40.30.GENE3D); IPR013766 (PFAM); IPR017937 (PROSITE); PTHR18929 (PANTHER), PTHR18929:SF32 (PANTHER), SignalP-NN(euk) (SIGNALP), tmhmm (TMHMM) | Yes |
| 74 | lib_fal_Filter15_IDBA_contig_2764_minus2 | 33 | | 186 | 0 | 100% | 1 | F:protein binding | IPR003961 (PFAM); IPR013783 (G3DSA:2.60.40.GENE3D); PR00014 (PRINTS) | |
| 75 | lib_fal_Filter15_IDBA_contig_2885_minus1 | 56 | h-2 class ii histocompatibility antigen gamma chain | 181 | 3.59E-04 | 52.50% | 2 | P:proteolysis; F:peptidase activity | IPR000716 (G3DSA:4.10.800.GENE3D); IPR022339 (PANTHER); PTHR14093:SF6 (PANTHER), SignalP-NN(euk) (SIGNALP), tmhmm (TMHMM) | Yes |
| 76 | lib_fal_Filter15_IDBA_contig_291_minus1 | 3882 | calponin transgelin | 281 | 1.25E-51 | 76.85% | 1 | F:actin binding | IPR001715 (G3DSA:1.10.418.GENE3D); IPR003096 (PRINTS); PTHR18959 (PANTHER), PTHR18959:SF3 (PANTHER) | |

| | | | | | | | | | |
|---|---|---|---|---|---|---|---|---|---|
| 77 | lib_fal_Filter15_IDBA_contig_2920_minus3 | 29 | transforming growth factor-beta-induced protein ig-h3 | 179 | 2.70E-19 | 51.85% | 1 | P:multicellular organismal development | IPR000782 (G3DSA:2.30.180.GENE3D); PTHR10900 (PANTHER) |
| 78 | lib_fal_Filter15_IDBA_contig_2932_minus3 | 16 | collagen alpha-1 chain | 179 | 1.60E-05 | 55.33% | 0 | - | IPR008160 (PFAM); PTHR24023 (PANTHER), PTHR24023:SF164 (PANTHER) |
| 79 | lib_fal_Filter15_IDBA_contig_2932_plus3 | 16 | ---NA--- | 179 | | | 0 | - | no IPS match |
| 80 | lib_fal_Filter15_IDBA_contig_2983_plus3 | 33 | immunoglobulin i-set domain protein | 177 | 2.96E-12 | 58.60% | 11 | F:protein kinase activity; P:biological_process; C:cytoplasm; F:protein binding; P:signal transduction; P:anatomical structure morphogenesis; P:cell differentiation; P:multicellular organismal development; P:cytoskeleton organization; F:enzyme regulator activity; P:metabolic process | IPR003598 (SMART); IPR003599 (SMART); IPR007110 (PROFILE); IPR013098 (PFAM); IPR013783 (G3DSA:2.60.40.GENE3D); PTHR25963 (PANTHER), SSF48726 (SUPERFAMILY) |
| 81 | lib_fal_Filter15_IDBA_contig_2998_minus1 | 25 | proactivator polypeptide | 177 | 1.12E-24 | 51.90% | 2 | C:lysosome; P:lipid metabolic process | IPR007856 (PFAM); IPR008138 (PFAM); IPR008139 (SMART); IPR008373 (PRINTS); IPR011001 (G3DSA:1.10.225.GENE3D); PTHR11480 (PANTHER) |
| 82 | lib_fal_Filter15_IDBA_contig_3033_minus3 | 33 | | 184 | 0 | 100% | 1 | F:protein binding | IPR003961 (PFAM); IPR007110 (PROFILE); IPR013098 (PFAM); IPR013783 (G3DSA:2.60.40.GENE3D); PR00014 (PRINTS), PTHR10489 (PANTHER), SSF48726 (SUPERFAMILY) |
| 83 | lib_fal_Filter15_IDBA_contig_304_minus2 | 5478 | glyceraldehyde-3-phosphate dehydrogenase | 307 | 5.65E-150 | 87.55% | 15 | P:cell death; C:cytosol; F:nucleotide binding; F:cytoskeletal protein binding; P:metabolic process; C:cytoskeleton; P:regulation of biological process; P:cytoskeleton organization; P:cellular component organization; F:catalytic activity; P:generation of precursor metabolites and energy; P:carbohydrate metabolic process; P:catabolic process; P:biosynthetic process; C:nucleus | IPR006424 (TIGRFAMs); IPR016040 (G3DSA:3.40.50.GENE3D); IPR020828 (PFAM); IPR020829 (PFAM); IPR020830 (PROSITE); IPR020831 (PRINTS); G3DSA:3.30.360.10 (GENE3D), SSF51735 (SUPERFAMILY), SSF55347 (SUPERFAMILY) |
| 84 | lib_fal_Filter15_IDBA_contig_3172_plus2 | 27 | ---NA--- | 171 | | | 0 | - | IPR008160 (PFAM); PTHR24023 (PANTHER) |
| 85 | lib_fal_Filter15_IDBA_contig_3196_minus3 | 25 | periostin isoform 1 | 169 | 7.73E-25 | 59.40% | 7 | F:heparin binding; P:extracellular matrix organization; P:cell adhesion; C:extracellular matrix; P:tissue development; P:regulation of Notch signaling pathway; C:proteinaceous extracellular matrix | IPR000782 (G3DSA:2.30.180.GENE3D); PTHR10900 (PANTHER) |

| | | | | | | | | | |
|---|---|---|---|---|---|---|---|---|---|
| 86 | lib_fal_Filter15_IDBA_contig_3203_minus3 | 26 | calmodulin | 169 | 8.92E-83 | 99.65% | 34 | P:biological_process; P:regulation of biological process; P:protein transport; F:protein binding; P:cytoskeleton organization; P:cell cycle; P:transport; P:cellular component organization; P:cell-cell signaling; C:cell; P:response to external stimulus; P:signal transduction; P:response to abiotic stimulus; F:cytoskeletal protein binding; P:reproduction; P:behavior; P:organelle organization; P:ion transport; P:growth; C:cytoskeleton; C:protein complex; C:microtubule organizing center; P:cell death; C:plasma membrane; P:anatomical structure morphogenesis; F:protein kinase activity; P:embryo development; C:cellular_component; F:nucleotide binding; P:cell differentiation; P:multicellular organismal development; C:intracellular; F:calcium ion binding; | IPR002048 (SMART); IPR011992 (G3DSA:1.10.238.GENE3D); IPR018247 (PROSITE); PTHR23050 (PANTHER), SSF47473 (SUPERFAMILY) |
| 87 | lib_fal_Filter15_IDBA_contig_3214_plus2 | 24 | matrix metalloproteinase-14-like | 169 | 7.73E-29 | 60.65% | 10 | P:cellular component organization; P:anatomical structure morphogenesis; P:multicellular organismal development; C:cell; P:cell death; F:peptidase activity; P:biological_process; P:growth; P:response to external stimulus; P:response to stress | IPR000585 (G3DSA:2.110.10.GENE3D); IPR018486 (PROSITE); IPR018487 (PFAM); PTHR10201 (PANTHER) |
| 88 | lib_fal_Filter15_IDBA_contig_3248_minus1 | 28 | low quality protein: titin | 169 | 2.52E-20 | 53.45% | 14 | P:anatomical structure morphogenesis; P:cell differentiation; P:multicellular organismal development; P:cytoskeleton organization; P:biological_process; F:cytoskeletal protein binding; C:cytoplasm; P:regulation of biological process; C:organelle; C:intracellular; F:protein binding; F:protein kinase activity; F:nucleotide binding; P:cellular protein modification process | IPR003961 (PFAM); IPR007110 (PROFILE); IPR013098 (PFAM); IPR013783 (G3DSA:2.60.40.GENE3D); IPR015726 (PTHR22964:PANTHER); IPR020675 (PANTHER); SSF48726 (SUPERFAMILY) |
| 89 | lib_fal_Filter15_IDBA_contig_3264_plus3 | 140 | 3-oxoacyl- | 345 | 8.96E-41 | 56.00% | 2 | P:metabolic process; F:catalytic activity | IPR002198 (PRINTS); IPR002347 (PRINTS); IPR016040 (G3DSA:3.40.50.GENE3D); IPR020904 (PROSITE); PTHR24322 (PANTHER), PTHR24322:SF0 (PANTHER), PF13561 (PFAM), SSF51735 (SUPERFAMILY) |
| 90 | lib_fal_Filter15_IDBA_contig_3267_plus3 | 18 | matrix metalloproteinase 9 | 167 | 3.34E-20 | 53.90% | 11 | P:multicellular organismal development; F:protein binding; P:cellular component organization; P:biological_process; C:extracellular space; F:binding; F:peptidase activity; P:response to stress; P:protein metabolic process; P:catabolic process; P:regulation of biological process | IPR000001 (PROFILE); IPR000562 (G3DSA:2.10.10.GENE3D); IPR013806 (SUPERFAMILY); PTHR22918 (PANTHER), PTHR22918:SF0 (PANTHER) |
| 91 | lib_fal_Filter15_IDBA_contig_3273_minus2 | 21 | | 167 | 0 | 100% | 1 | F:protein binding | IPR003599 (SMART); IPR007110 (PROFILE); IPR013098 (PFAM); IPR013783 (G3DSA:2.60.40.GENE3D); PTHR13817 (PANTHER), PTHR13817:SF2 (PANTHER), SSF48726 (SUPERFAMILY) |
| 92 | lib_fal_Filter15_IDBA_contig_3332_plus1 | 27 | ---NA--- | 166 | | | 0 | - | no IPS match |

| | | | | | | | | | | |
|---|---|---|---|---|---|---|---|---|---|---|
| 93 | lib_fal_Filter15_IDBA_contig_3332_plus3 | 27 | ---NA--- | 165 | | | 0 | - | IPR008160 (PFAM); PTHR24023 (PANTHER) | |
| 94 | lib_fal_Filter15_IDBA_contig_3342_minus1 | 28 | glucose dehydrogenase | 165 | 5.07E-57 | 58.75% | 2 | F:nucleotide binding; P:metabolic process | IPR000172 (PFAM); G3DSA:3.50.50.60 (GENE3D), PTHR11552 (PANTHER), PTHR11552:SF10 (PANTHER), SSF51905 (SUPERFAMILY) | |
| 95 | lib_fal_Filter15_IDBA_contig_3379_minus3 | 18 | triacylglycerol pancreatic | 164 | 1.34E-15 | 46.85% | 3 | F:catalytic activity; P:lipid metabolic process; F:protein binding | IPR000734 (PANTHER); IPR001024 (G3DSA:2.60.60.GENE3D); IPR013818 (PFAM); G3DSA:3.40.50.1820 (GENE3D), SSF53474 (SUPERFAMILY) | |
| 96 | lib_fal_Filter15_IDBA_contig_3392_plus1 | 17 | | 164 | 0 | 100% | 1 | F:protein binding | IPR003961 (PFAM); IPR013783 (G3DSA:2.60.40.GENE3D); PR00014 (PRINTS) | |
| 97 | lib_fal_Filter15_IDBA_contig_3400_minus3 | 52 | phosphoglucose isomerase | 226 | 8.65E-109 | 81.20% | 6 | P:generation of precursor metabolites and energy; P:carbohydrate metabolic process; P:catabolic process; C:cytoplasm; F:catalytic activity; P:biosynthetic process | IPR001672 (PANTHER); G3DSA:3.40.50.10490 (GENE3D), PTHR11469:SF0 (PANTHER), SSF53697 (SUPERFAMILY) | |
| 98 | lib_fal_Filter15_IDBA_contig_3413_minus3 | 26 | deleted in malignant brain tumors 1 | 169 | 1.31E-27 | 60.95% | 2 | F:receptor activity; C:cell | IPR001190 (PRINTS); IPR017448 (SMART); G3DSA:3.10.250.10 (GENE3D), PTHR19331 (PANTHER), PTHR19331:SF118 (PANTHER) | |
| 99 | lib_fal_Filter15_IDBA_contig_3424_minus2 | 20 | von willebrand factor type egf and pentraxin domain-containing protein 1 | 162 | 3.80E-06 | 58.70% | 7 | C:cytoplasm; F:calcium ion binding; P:cell adhesion; C:membrane; F:chromatin binding; C:extracellular region; P:biological process | IPR000436 (PFAM); G3DSA:2.10.70.10 (GENE3D), PTHR19325 (PANTHER), PTHR19325:SF43 (PANTHER) | |
| 100 | lib_fal_Filter15_IDBA_contig_3469_minus1 | 19 | lysyl oxidase homolog 2-partial | 161 | 1.01E-20 | 61.70% | 2 | F:receptor activity; C:cell | IPR001190 (PRINTS); IPR017448 (SMART); G3DSA:3.10.250.10 (GENE3D), PTHR19331 (PANTHER), PTHR19331:SF118 (PANTHER) | |
| 101 | lib_fal_Filter15_IDBA_contig_3503_minus3 | 2408 | protein | 340 | 1.71E-90 | 90.45% | 6 | P:protein metabolic process; P:cellular protein modification process; F:binding; C:cytoplasm; F:hydrolase activity; F:catalytic activity | IPR002130 (PRINTS); IPR020892 (PROSITE); G3DSA:2.40.100.10 (GENE3D), PTHR11071 (PANTHER), tmhmm (TMHMM) | |
| 102 | lib_fal_Filter15_IDBA_contig_3504_minus1 | 187 | cyclophilin ovcyp-2 | 101 | 1.69E-32 | 89.70% | 5 | P:protein metabolic process; P:cellular protein modification process; F:binding; C:cytoplasm; F:catalytic activity | IPR002130 (PRINTS); G3DSA:2.40.100.10 (GENE3D), PTHR11071 (PANTHER), tmhmm (TMHMM) | |
| 103 | lib_fal_Filter15_IDBA_contig_3516_plus1 | 36 | von willebrand factor type egf and pentraxin domain-containing protein 1-like | 160 | 2.40E-27 | 50.60% | 3 | P:cell adhesion; C:membrane; F:carbohydrate binding | IPR000436 (PFAM); IPR011641 (PFAM); G3DSA:2.10.70.10 (GENE3D), PTHR19325 (PANTHER) | |
| 104 | lib_fal_Filter15_IDBA_contig_3561_minus1 | 719 | sparc | 337 | 2.24E-66 | 60.60% | 7 | P:embryo development; P:multicellular organismal development; P:regulation of biological process; P:growth; F:calcium ion binding; C:proteinaceous extracellular matrix; P:signal transduction | IPR011992 (G3DSA:1.10.238.GENE3D); IPR018247 (PROSITE); IPR019577 (PFAM); G3DSA:3.30.60.30 (GENE3D), PTHR13866 (PANTHER), PTHR13866:SF5 (PANTHER), SignalP-NN(euk) (SIGNALP), SSF47473 (SUPERFAMILY) | Yes |
| 105 | lib_fal_Filter15_IDBA_contig_3568_plus2 | 26 | ---NA--- | 159 | | | 0 | - | IPR008160 (PFAM); PTHR24023 (PANTHER) | |
| 106 | lib_fal_Filter15_IDBA_contig_3592_minus3 | 17 | cyclophilin a | 157 | 7.50E-43 | 92.25% | 3 | P:protein metabolic process; P:cellular protein modification process; F:catalytic activity | IPR002130 (PRINTS); G3DSA:2.40.100.10 (GENE3D), PTHR11071 (PANTHER), PTHR11071:SF116 (PANTHER) | |
| 107 | lib_fal_Filter15_IDBA_contig_3594_minus3 | 22 | thioester containing protein partial | 157 | 8.26E-39 | 66.10% | 1 | C:extracellular space | IPR008930 (SUPERFAMILY); IPR011626 (PFAM); G3DSA:1.50.10.20 (GENE3D), PTHR11412 (PANTHER) | |
| 108 | lib_fal_Filter15_IDBA_contig_3666_plus3 | 19 | | 156 | 0 | 100% | 1 | F:protein binding | IPR003961 (PFAM); IPR013783 (G3DSA:2.60.40.GENE3D); PR00014 (PRINTS) | |
| 109 | lib_fal_Filter15_IDBA_contig_3692_plus2 | 39 | kettin protein | 155 | 4.13E-53 | 57.20% | 1 | F:protein binding | IPR007110 (PROFILE); IPR013098 (PFAM); IPR013783 (G3DSA:2.60.40.GENE3D); SSF48726 (SUPERFAMILY) | |

| | | | | | | | | | |
|---|---|---|---|---|---|---|---|---|---|
| 110 | lib_fal_Filter15_IDBA_contig_3698_plus1 | 22 | basement membrane-specific heparan sulfate proteoglycan core | 155 | 3.59E-29 | 58.00% | 11 | P:biological_process; P:regulation of biological process; P:growth; P:multicellular organismal development; P:cellular component organization; C:proteinaceous extracellular matrix; P:anatomical structure morphogenesis; P:cell differentiation; P:cytoskeleton organization; P:embryo development; P:signal transduction | IPR001791 (PFAM); IPR008985 (SUPERFAMILY); IPR013320 (G3DSA:2.60.120.GENE3D); PTHR10574 (PANTHER), PTHR10574:SF20 (PANTHER), tmhmm (TMHMM) |
| 111 | lib_fal_Filter15_IDBA_contig_370_minus2 | 3282 | ---NA--- | 1163 | | | 0 | - | PTHR24023 (PANTHER), PTHR24023:SF196 (PANTHER) |
| 112 | lib_fal_Filter15_IDBA_contig_370_plus3 | 3282 | fibrillar collagen | 1162 | 4.24E-122 | 66.20% | 14 | P:regulation of biological process; P:multicellular organismal development; P:biological_process; P:anatomical structure morphogenesis; P:cellular component organization; P:cell differentiation; P:response to endogenous stimulus; F:protein binding; P:protein transport; P:embryo development; C:proteinaceous extracellular matrix; F:structural molecule activity; C:cytoplasm; P:metabolic process | IPR000885 (PRODOM); IPR008160 (PFAM); PTHR24023 (PANTHER) |
| 113 | lib_fal_Filter15_IDBA_contig_371_plus1 | 453 | ankyrin isoform x | 215 | 7.29E-49 | 89.10% | 13 | F:cytoskeletal protein binding; P:anatomical structure morphogenesis; P:cellular component organization; P:cell differentiation; P:multicellular organismal development; P:cytoskeleton organization; C:cell; C:cellular_component; P:signal transduction; P:cellular homeostasis; F:structural molecule activity; F:hydrolase activity; C:plasma membrane | IPR002110 (PRINTS); IPR020683 (G3DSA:1.25.40.GENE3D); PTHR24123 (PANTHER), PTHR24123:SF0 (PANTHER), SignalP-NN(euk) (SIGNALP) |
| 114 | lib_fal_Filter15_IDBA_contig_3754_minus2 | 23 | collagen alpha-6 chain | 154 | 5.57E-55 | 76.25% | 8 | P:anatomical structure morphogenesis; P:regulation of biological process; P:multicellular organismal development; P:response to endogenous stimulus; ; F:structural molecule activity; P:cellular component organization; C:proteinaceous extracellular matrix | IPR001442 (G3DSA:2.170.240.GENE3D); IPR008160 (PFAM); IPR016187 (SUPERFAMILY); PTHR24637 (PANTHER), PTHR24637:SF12 (PANTHER) |
| 115 | lib_fal_Filter15_IDBA_contig_3754_plus3 | 23 | ---NA--- | 153 | | | 0 | - | no IPS match |
| 116 | lib_fal_Filter15_IDBA_contig_377_plus1 | 50 | ---NA--- | 112 | | | 0 | - | PTHR24023 (PANTHER), PTHR24023:SF164 (PANTHER) |
| 117 | lib_fal_Filter15_IDBA_contig_378_plus1 | 77 | ---NA--- | 112 | | | 0 | - | PTHR24023 (PANTHER), PTHR24023:SF164 (PANTHER) |
| 118 | lib_fal_Filter15_IDBA_contig_3788_minus1 | 12 | protein lethal giant larvae-like | 153 | 6.00E-35 | 81.25% | 0 | - | IPR000664 (PRINTS); PTHR10241 (PANTHER), PTHR10241:SF20 (PANTHER) |
| 119 | lib_fal_Filter15_IDBA_contig_3802_minus1 | 19 | cytosolic non-specific dipeptidase isoform 2 | 153 | 2.13E-58 | 84.80% | 8 | F:binding; P:metabolic process; F:peptidase activity; P:protein metabolic process; P:catabolic process; C:cytosol; P:biosynthetic process; | IPR002933 (PFAM); G3DSA:3.40.630.10 (GENE3D), PTHR11014 (PANTHER), PTHR11014:SF15 (PANTHER), SSF53187 (SUPERFAMILY) |
| 120 | lib_fal_Filter15_IDBA_contig_383_minus1 | 642 | ---NA--- | 511 | | | 0 | - | no IPS match |
| 121 | lib_fal_Filter15_IDBA_contig_383_plus2 | 642 | chordin-like protein 2 | 511 | 1.82E-07 | 51.45% | 1 | F:protein binding | IPR001007 (PFAM); IPR008160 (PFAM); G3DSA:2.10.70.10 (GENE3D), PTHR24023 (PANTHER), PTHR24023:SF58 (PANTHER), SSF57603 (SUPERFAMILY) |

| | | | | | | | | | | |
|---|---|---|---|---|---|---|---|---|---|---|
| 122 | lib_fal_Filter15_IDBA_contig_3846_plus1 | 7493 | ferritin | 528 | 1.16E-88 | 85.30% | 6 | C:cytoplasm; P:cellular homeostasis; F:binding; F:catalytic activity; P:ion transport; P:metabolic process | IPR001519 (PANTHER); IPR008331 (PFAM); IPR009040 (PROFILE); IPR009078 (SUPERFAMILY); IPR012347 (G3DSA:1.20.1260.GENE3D); IPR014034 (PROSITE) | |
| 123 | lib_fal_Filter15_IDBA_contig_386_minus2 | 36497 | myosin heavy chain | 602 | 0 | 81.60% | 7 | C:cytoskeleton; C:protein complex; F:nucleotide binding; C:cytoplasm; F:actin binding; F:motor activity; F:protein binding | IPR002928 (PFAM); IPR009053 (SUPERFAMILY); G3DSA:1.20.1170.10 (GENE3D), PTHR13140 (PANTHER), PTHR13140:SF106 (PANTHER) | |
| 124 | lib_fal_Filter15_IDBA_contig_3888_minus2 | 26 | membrane metallo-endopeptidase-like 1 | 151 | 7.87E-10 | 51.95% | 5 | C:organelle; C:intracellular; F:peptidase activity; P:protein metabolic process; P:catabolic process | IPR000718 (PANTHER); IPR018497 (PFAM); IPR024079 (G3DSA:3.40.390.GENE3D); SSF55486 (SUPERFAMILY) | |
| 125 | lib_fal_Filter15_IDBA_contig_3914_minus3 | 21 | calreticulin | 150 | 1.70E-40 | 82.95% | 4 | P:protein metabolic process; C:endoplasmic reticulum; F:protein binding; F:calcium ion binding | IPR001580 (PRINTS); IPR006861 (PFAM); IPR008985 (SUPERFAMILY); IPR009033 (G3DSA:2.10.250.GENE3D); PTHR11073:SF2 (PANTHER) | |
| 126 | lib_fal_Filter15_IDBA_contig_392_minus1 | 3086 | myosin heavy fast skeletal muscle | 104 | 2.70E-20 | 88.80% | 7 | C:cytoskeleton; C:protein complex; F:actin binding; P:response to stress; F:nucleotide binding; F:motor activity; P:metabolic process | IPR002928 (PFAM); PTHR13140 (PANTHER), PTHR13140:SF109 (PANTHER) | |
| 127 | lib_fal_Filter15_IDBA_contig_3937_minus1 | 33 | endo-b- -glucanase | 150 | 1.95E-34 | 61.95% | 2 | F:hydrolase activity; P:carbohydrate metabolic process | IPR001701 (PFAM); IPR008928 (SUPERFAMILY); IPR012341 (G3DSA:1.50.10.GENE3D); PTHR22298 (PANTHER) | |
| 128 | lib_fal_Filter15_IDBA_contig_402_minus1 | 27483 | myosin heavy chain | 1170 | 0 | 80.45% | 7 | C:cytoskeleton; C:protein complex; F:nucleotide binding; C:cytoplasm; F:actin binding; F:motor activity; F:protein binding | IPR000048 (PFAM); IPR001609 (PRINTS); IPR004009 (PFAM); IPR009053 (SUPERFAMILY); IPR027401 (G3DSA:4.10.270.GENE3D); IPR027417 (SUPERFAMILY); G3DSA:1.20.5.340 (GENE3D), PTHR13140 (PANTHER), PTHR13140:SF106 (PANTHER), SignalP-NN(euk) (SIGNALP) | Yes |
| 129 | lib_fal_Filter15_IDBA_contig_4048_plus1 | 17 | isoform h | 147 | 2.83E-29 | 50.15% | 3 | P:biological_process; C:intracellular; F:protein binding | IPR003598 (SMART); IPR003599 (SMART); IPR003961 (SUPERFAMILY); IPR007110 (PROFILE); IPR013098 (PFAM); IPR013783 (G3DSA:2.60.40.GENE3D); PTHR10489 (PANTHER), SSF48726 (SUPERFAMILY) | |
| 130 | lib_fal_Filter15_IDBA_contig_409_plus1 | 1659 | stretchin- isoform u | 2171 | 1.01E-105 | 43.65% | 6 | C:organelle; F:protein kinase activity; P:multicellular organismal development; C:intracellular; P:biological_process; F:protein binding | IPR003598 (SMART); IPR003599 (SMART); IPR007110 (PROFILE); IPR013098 (PFAM); IPR013783 (G3DSA:2.60.40.GENE3D); IPR020675 (PANTHER); PTHR22964:SF3 (PANTHER), SSF48726 (SUPERFAMILY) | |
| 131 | lib_fal_Filter15_IDBA_contig_411_minus3 | 4016 | angiomotin | 2094 | 3.22E-73 | 63.95% | 5 | P:anatomical structure morphogenesis; P:embryo development; C:cytoskeleton; P:regulation of biological process; P:biological_process | IPR009114 (PRINTS); IPR024646 (PFAM); tmhmm (TMHMM) | |
| 132 | lib_fal_Filter15_IDBA_contig_412_minus3 | 8625 | protein unc- isoform c | 1886 | 0 | 58.60% | 7 | F:transferase activity; C:cytoplasm; P:regulation of biological process; P:multicellular organismal development; P:biological_process; P:growth; F:protein binding | IPR003598 (SMART); IPR003599 (SMART); IPR003961 (PFAM); IPR007110 (PROFILE); IPR013098 (PFAM); IPR013783 (G3DSA:2.60.40.GENE3D); PR00014 (PRINTS), SSF48726 (SUPERFAMILY) | |
| 133 | lib_fal_Filter15_IDBA_contig_4127_plus2 | 11 | thioester-containing protein | 145 | 8.07E-11 | 59.25% | 4 | P:negative regulation of endopeptidase activity; F:endopeptidase inhibitor activity; C:extracellular space; C:extracellular region | IPR011625 (PFAM); PTHR11412 (PANTHER) | |
| 134 | lib_fal_Filter15_IDBA_contig_413_minus2 | 3415 | projectin short variant | 691 | 6.82E-143 | 54.15% | 2 | F:transferase activity; F:protein binding | IPR003598 (SMART); IPR003599 (SMART); IPR007110 (PROFILE); IPR013098 (PFAM); IPR013783 (G3DSA:2.60.40.GENE3D); IPR020675 (PANTHER); PF13895 (PFAM), SSF48726 (SUPERFAMILY) | |
| 135 | lib_fal_Filter15_IDBA_contig_4148_plus1 | 53 | granulin- partial | 145 | 1.16E-17 | 55.80% | 0 | - | IPR000118 (PFAM); PTHR12274 (PANTHER), PTHR12274:SF0 (PANTHER) | |

| | | | | | | | | | |
|---|---|---|---|---|---|---|---|---|---|
| 136 | lib_fal_Filter15_IDBA_contig_4197_plus2 | 14 | | 143 | 0 | 100% | 1 | F:protein binding | IPR003961 (PFAM); IPR013783 (G3DSA:2.60.40.GENE3D); PR00014 (PRINTS) |
| 137 | lib_fal_Filter15_IDBA_contig_422_minus2 | 119 | ---NA--- | 284 | | | 0 | - | no IPS match |
| 138 | lib_fal_Filter15_IDBA_contig_427_plus1 | 2173 | 14-3-3 zeta | 1334 | 1.82E-116 | 89.25% | 22 | P:signal transduction; P:anatomical structure morphogenesis; P:cell differentiation; P:multicellular organismal development; P:reproduction; P:cytoskeleton organization; P:protein metabolic process; P:behavior; P:cell proliferation; P:metabolic process; P:regulation of biological process; F:protein binding; C:extracellular region; F:enzyme regulator activity; P:biological_process; C:cytoskeleton; C:protein complex; P:embryo development; P:cell cycle; C:nucleus; P:biosynthetic process; | IPR000308 (PRINTS); IPR023409 (PROSITE); IPR023410 (G3DSA:1.20.190.GENE3D); PTHR18860:SF0 (PANTHER), tmhmm (TMHMM) |
| 139 | lib_fal_Filter15_IDBA_contig_4284_plus2 | 30 | wap four-disulfide core domain protein 1 | 163 | 1.25E-17 | 57.95% | 6 | C:extracellular space; P:cell proliferation; P:regulation of biological process; P:biological_process; P:response to endogenous stimulus; P:cell growth | PTHR14308 (PANTHER), SignalP-NN(euk) (SIGNALP) |
| 140 | lib_fal_Filter15_IDBA_contig_4303_plus2 | 45 | fibrillin-1- partial | 141 | 1.47E-16 | 52.70% | 2 | F:protein binding; F:calcium ion binding | IPR000152 (PROSITE); IPR000742 (SMART); IPR001881 (PFAM); IPR018097 (PROSITE); G3DSA:2.10.25.10 (GENE3D), PTHR24046 (PANTHER), SSF57196 (SUPERFAMILY) |
| 141 | lib_fal_Filter15_IDBA_contig_432_minus3 | 365 | map kinase-interacting serine threonine-protein kinase 1 | 1198 | 0 | 76.35% | 5 | P:response to stress; P:cellular protein modification process; F:nucleotide binding; P:signal transduction; F:protein kinase activity | IPR000719 (PFAM); IPR002290 (SMART); IPR008271 (PROSITE); IPR011009 (SUPERFAMILY); IPR017441 (PROSITE); IPR020635 (SMART); G3DSA:1.10.510.10 (GENE3D), G3DSA:3.30.200.20 (GENE3D), PTHR24349 (PANTHER), PTHR24349:SF0 (PANTHER), tmhmm (TMHMM) |
| 142 | lib_fal_Filter15_IDBA_contig_4324_plus2 | 26 | svil partial | 145 | 3.29E-53 | 73.10% | 7 | C:cytoplasm; P:multicellular organismal development; C:plasma membrane; F:actin binding; C:nucleus; C:cytoskeleton; C:cell | IPR007122 (PRINTS); IPR007123 (PFAM); IPR015628 (PTHR11977:PANTHER); G3DSA:3.40.20.10 (GENE3D), SSF55753 (SUPERFAMILY) |
| 143 | lib_fal_Filter15_IDBA_contig_4359_plus3 | 20 | glucose dehydrogenase | 140 | 7.53E-29 | 79.55% | 6 | F:nucleotide binding; C:mitochondrion; P:biosynthetic process; ; F:catalytic activity; P:metabolic process | IPR007867 (PFAM); G3DSA:3.50.50.60 (GENE3D), PTHR11552 (PANTHER), PTHR11552:SF10 (PANTHER), SSF51905 (SUPERFAMILY) |
| 144 | lib_fal_Filter15_IDBA_contig_4368_minus1 | 18 | isoform y | 140 | 7.53E-32 | 52.35% | 3 | F:protein kinase activity; F:nucleotide binding; P:cellular protein modification process | IPR013098 (PFAM); IPR013783 (G3DSA:2.60.40.GENE3D); IPR015726 (PTHR22964:PANTHER); IPR020675 (PANTHER); SSF48726 (SUPERFAMILY) |
| 145 | lib_fal_Filter15_IDBA_contig_4376_minus2 | 23 | thioredoxin | 140 | 6.73E-33 | 73.20% | 16 | ; C:nucleoplasm; P:regulation of biological process; P:protein transport; P:cell-cell signaling; F:protein binding; P:biological_process; P:cell proliferation; C:cytosol; P:nucleobase-containing compound metabolic process; P:metabolic process; P:response to abiotic stimulus; P:response to stress; F:electron carrier activity; F:catalytic activity; P:cellular homeostasis | IPR005746 (PRINTS); IPR012336 (G3DSA:3.40.30.GENE3D); IPR013766 (PFAM); IPR017937 (PROSITE) |

| | | | | | | | | | | |
|---|---|---|---|---|---|---|---|---|---|---|
| 146 | lib_fal_Filter15_IDBA_contig_4385_minus1 | 12 | cre-unc-22 protein | 140 | 3.68E-41 | 56.75% | 7 | F:transferase activity; C:cytoplasm; P:regulation of biological process; P:multicellular organismal development; P:biological_process; P:growth; F:protein binding | IPR003598 (SMART); IPR003961 (SUPERFAMILY); IPR007110 (PROFILE); IPR013098 (PFAM); IPR013783 (G3DSA:2.60.40.GENE3D); PTHR25963 (PANTHER), SSF48726 (SUPERFAMILY) | |
| 147 | lib_fal_Filter15_IDBA_contig_4423_plus2 | 87 | protein cal-8 | 204 | 5.71E-45 | 57.90% | 1 | F:calcium ion binding | IPR002048 (SMART); IPR011992 (G3DSA:1.10.238.GENE3D); IPR018247 (PROSITE); PTHR10891 (PANTHER), SSF47473 (SUPERFAMILY) | |
| 148 | lib_fal_Filter15_IDBA_contig_4435_plus3 | 30 | palladin isoform 2 | 139 | 1.03E-16 | 60.20% | 5 | F:cytoskeletal protein binding; F:transferase activity; C:cytoskeleton; C:protein complex; C:nucleus | IPR003598 (SMART); IPR003599 (SMART); IPR007110 (PROFILE); IPR013098 (PFAM); IPR013783 (G3DSA:2.60.40.GENE3D); PTHR10489 (PANTHER), SSF48726 (SUPERFAMILY) | |
| 149 | lib_fal_Filter15_IDBA_contig_451_plus3 | 638 | protein-glutamine gamma-glutamyltransferase k | 1083 | 0 | 60.40% | 2 | P:cellular protein modification process; F:transferase activity | IPR001102 (PFAM); IPR002931 (G3DSA:3.90.260.GENE3D); IPR008958 (PFAM); IPR013783 (G3DSA:2.60.40.GENE3D); IPR013808 (PROSITE); IPR014756 (SUPERFAMILY); IPR023608 (PANTHER); SSF54001 (SUPERFAMILY) | |
| 150 | lib_fal_Filter15_IDBA_contig_4558_plus3 | 22 | low-density lipoprotein receptor-related protein 2 | 136 | 1.06E-06 | 63.25% | 1 | F:protein binding | IPR002172 (G3DSA:4.10.400.GENE3D); PR00261 (PRINTS), PTHR10529 (PANTHER) | |
| 151 | lib_fal_Filter15_IDBA_contig_4612_minus2 | 26 | lysyl oxidase homolog 4 | 136 | 2.41E-28 | 69.55% | 3 | F:protein binding; F:receptor activity; C:cell | IPR001190 (PRINTS); IPR017448 (SMART); G3DSA:3.10.250.10 (GENE3D), PTHR19331 (PANTHER) | |
| 152 | lib_fal_Filter15_IDBA_contig_462_minus1 | 3615 | heat shock protein 70 | 889 | 0 | 95.40% | 2 | F:nucleotide binding; P:response to stress | IPR013126 (PRINTS); IPR018181 (PROSITE); G3DSA:1.20.1270.10 (GENE3D), G3DSA:2.60.34.10 (GENE3D), G3DSA:3.30.30.30 (GENE3D), G3DSA:3.30.420.40 (GENE3D), G3DSA:3.90.640.10 (GENE3D), PTHR19375 (PANTHER), tmhmm (TMHMM), SSF100920 (SUPERFAMILY), SSF100934 (SUPERFAMILY), SSF53067 (SUPERFAMILY) | |
| 153 | lib_fal_Filter15_IDBA_contig_4651_minus1 | 98 | ---NA--- | 204 | | | 0 | - | no IPS match | |
| 154 | lib_fal_Filter15_IDBA_contig_4651_plus1 | 98 | trans- partial | 204 | 1.90E-06 | 53.75% | 1 | F:transferase activity | PTHR12392 (PANTHER) | |
| 155 | lib_fal_Filter15_IDBA_contig_476_minus1 | 497 | lysyl oxidase like protein 3 | 124 | 2.64E-32 | 72.80% | 13 | F:binding; P:anatomical structure morphogenesis; P:cellular component organization; P:cell differentiation; P:multicellular organismal development; F:catalytic activity; F:protein binding; C:extracellular region; P:regulation of biological process; ; C:nucleus; P:embryo development; P:metabolic process | IPR001695 (PRINTS); PTHR19331 (PANTHER) | |
| 156 | lib_fal_Filter15_IDBA_contig_4787_minus2 | 41 | | 204 | 0 | 100% | 1 | F:protein binding | IPR003961 (PFAM); IPR007110 (PROFILE); IPR013098 (PFAM); IPR013783 (G3DSA:2.60.40.GENE3D); PR00014 (PRINTS), PTHR10489 (PANTHER), SSF48726 (SUPERFAMILY) | |
| 157 | lib_fal_Filter15_IDBA_contig_484_plus3 | 224 | calbindin | 801 | 5.44E-21 | 61.55% | 11 | P:behavior; F:protein binding; P:multicellular organismal development; P:ion transport; P:regulation of biological process; P:cell-cell signaling; P:biological_process; C:cytosol; C:cell; C:nucleus; F:calcium ion binding | IPR002048 (PFAM); IPR011992 (G3DSA:1.10.238.GENE3D); IPR018247 (PROSITE); PTHR19972 (PANTHER), SignalP-NN(euk) (SIGNALP), tmhmm (TMHMM), SSF47473 (SUPERFAMILY) | Yes |
| 158 | lib_fal_Filter15_IDBA_contig_4862_minus3 | 46 | multiple epidermal growth factor-like domains protein 6-like | 131 | 4.13E-47 | 68.30% | 1 | F:protein binding | IPR000742 (PROFILE); IPR002049 (PFAM); IPR013032 (PROSITE); PR00011 (PRINTS), G3DSA:2.170.300.10 (GENE3D), PTHR24035 (PANTHER), PTHR24035:SF4 (PANTHER), SSF57196 (SUPERFAMILY) | |

| | | | | | | | | | | |
|---|---|---|---|---|---|---|---|---|---|---|
| 159 | lib_fal_Filter15_IDBA_contig_4909_minus3 | 25 | protein-glutamine gamma-glutamyltransferase k | 130 | 1.18E-39 | 69.25% | 8 | C:cytoskeleton; P:cell differentiation; P:multicellular organismal development; F:protein binding; P:cellular protein modification process; P:cellular component organization; C:cell; F:transferase activity | IPR002931 (G3DSA:3.90.260.GENE3D); IPR013783 (G3DSA:2.60.40.GENE3D); IPR014756 (SUPERFAMILY); IPR023608 (PANTHER); PTHR11590:SF13 (PANTHER), SSF54001 (SUPERFAMILY) | |
| 160 | lib_fal_Filter15_IDBA_contig_493_plus2 | 316 | mitochondrial manganese superoxide dismutase | 878 | 9.52E-47 | 83.65% | 3 | F:binding; P:metabolic process; F:catalytic activity | IPR001189 (PANTHER); IPR019832 (PFAM); IPR019833 (PROSITE); PTHR11404:SF5 (PANTHER), tmhmm (TMHMM) | |
| 161 | lib_fal_Filter15_IDBA_contig_4933_plus1 | 16 | membrane metallo isoform cra_a | 131 | 5.41E-19 | 72.25% | 12 | P:metabolic process; F:binding; P:biological_process; C:cell; C:cytoplasmic membrane-bounded vesicle; P:response to abiotic stimulus; F:peptidase activity; P:protein metabolic process; P:catabolic process; ; P:multicellular organismal development; C:plasma membrane | IPR000718 (PANTHER); IPR018497 (PFAM); IPR024079 (G3DSA:3.40.390.GENE3D); SSF55486 (SUPERFAMILY) | |
| 162 | lib_fal_Filter15_IDBA_contig_4999_plus3 | 16 | serine-threonine kinase receptor-associated protein | 129 | 1.40E-73 | 89.25% | 11 | C:plasma membrane; C:nucleus; P:signal transduction; C:mitochondrion; F:receptor activity; P:nucleobase-containing compound metabolic process; P:regulation of biological process; ; F:receptor binding; P:metabolic process; F:kinase activity | IPR001680 (PFAM); IPR015943 (G3DSA:2.130.10.GENE3D); IPR017986 (PROFILE); IPR019775 (PROSITE); PTHR19877 (PANTHER) | |
| 163 | lib_fal_Filter15_IDBA_contig_5_minus1 | 2098 | | 2116 | 0 | 100% | 1 | F:protein binding | IPR003598 (SMART); IPR003599 (SMART); IPR003961 (PFAM); IPR007110 (PROFILE); IPR013098 (PFAM); IPR013783 (G3DSA:2.60.40.GENE3D); PR00014 (PRINTS), SSF48726 (SUPERFAMILY) | |
| 164 | lib_fal_Filter15_IDBA_contig_500_minus3 | 944 | collagen-like exosporium surface protein | 1209 | 1.63E-08 | 53.11% | 1 | F:binding | IPR007527 (PROFILE); IPR008160 (PFAM); PTHR24023 (PANTHER), tmhmm (TMHMM) | |
| 165 | lib_fal_Filter15_IDBA_contig_500_plus1 | 944 | ---NA--- | 1210 | | | 0 | - | tmhmm (TMHMM) | |
| 166 | lib_fal_Filter15_IDBA_contig_5057_plus3 | 11 | titin | 128 | 3.42E-17 | 50.30% | 12 | P:anatomical structure morphogenesis; P:cell differentiation; P:multicellular organismal development; P:cytoskeleton organization; F:protein kinase activity; P:biological_process; F:cytoskeletal protein binding; C:cytoplasm; P:cellular protein modification process; C:organelle; C:intracellular; F:protein binding | IPR003598 (SMART); IPR003599 (SMART); IPR007110 (PROFILE); IPR013098 (PFAM); IPR013783 (G3DSA:2.60.40.GENE3D); PTHR10489 (PANTHER), SSF48726 (SUPERFAMILY) | |
| 167 | lib_fal_Filter15_IDBA_contig_509_minus1 | 309 | ---NA--- | 700 | | | 0 | - | SignalP-NN(euk) (SIGNALP) | Yes |
| 168 | lib_fal_Filter15_IDBA_contig_509_plus2 | 309 | hypothetical protein IG3_03228, partial | 700 | 2.26E-04 | 62.00% | 0 | - | IPR008160 (PFAM); PTHR24023 (PANTHER), tmhmm (TMHMM) | |
| 169 | lib_fal_Filter15_IDBA_contig_509_plus3 | 309 | ---NA--- | 700 | | | 0 | - | no IPS match | |
| 170 | lib_fal_Filter15_IDBA_contig_5121_minus2 | 26 | membrane metallo-endopeptidase-like 1-like | 152 | 3.01E-09 | 71.55% | 3 | F:peptidase activity; P:protein metabolic process; P:catabolic process | IPR000718 (PANTHER); tmhmm (TMHMM), SSF55486 (SUPERFAMILY) | |

| | | | | | | | | | |
|---|---|---|---|---|---|---|---|---|---|
| 171 | lib_fal_Filter15_IDBA_contig_5138_plus2 | 20 | cartilage oligomeric matrix protein | 127 | 4.95E-53 | 80.35% | 17 | F:receptor binding; P:regulation of biological process; P:cell proliferation; P:anatomical structure morphogenesis; P:multicellular organismal development; C:proteinaceous extracellular matrix; P:biological_process; P:cell differentiation; P:behavior; P:response to external stimulus; C:extracellular space; F:calcium ion binding; P:cell death; F:carbohydrate binding; P:cellular protein modification process; P:growth; C:cytoplasm | IPR003367 (PFAM); IPR008859 (PFAM); IPR008985 (SUPERFAMILY); IPR013320 (G3DSA:2.60.120.GENE3D); G3DSA:4.10.1080.10 (GENE3D), PTHR10199 (PANTHER), SSF103647 (SUPERFAMILY) |
| 172 | lib_fal_Filter15_IDBA_contig_516_minus1 | 502 | neuroblast differentiation-associated protein ahnak | 334 | 1.05E-06 | 41.20% | 0 | - | PTHR23348 (PANTHER) |
| 173 | lib_fal_Filter15_IDBA_contig_5162_minus1 | 27 | sushi repeat-containing protein srpx2 | 189 | 3.30E-13 | 60.00% | 1 | F:protein binding | IPR000742 (PFAM); IPR000884 (PFAM); IPR013032 (PROSITE); IPR027231 (PANTHER); G3DSA:2.10.25.10 (GENE3D), G3DSA:2.20.100.10 (GENE3D), PTHR11036:SF39 (PANTHER), SSF57196 (SUPERFAMILY) |
| 174 | lib_fal_Filter15_IDBA_contig_5174_minus1 | 18 | 14-3-3 protein epsilon | 127 | 6.01E-48 | 87.55% | 21 | P:multicellular organismal development; P:signal transduction; P:reproduction; P:cell differentiation; P:cytoskeleton organization; P:response to stress; P:cell cycle; P:response to abiotic stimulus; C:cytoplasm; F:protein binding; C:extracellular region; P:regulation of biological process; P:organelle organization; P:growth; P:behavior; C:chromosome; F:enzyme regulator activity; C:cytoskeleton; C:protein complex; C:nucleus; P:anatomical structure morphogenesis | IPR000308 (PRINTS); IPR023409 (PROSITE); IPR023410 (G3DSA:1.20.190.GENE3D); PTHR18860:SF0 (PANTHER) |
| 175 | lib_fal_Filter15_IDBA_contig_519_plus3 | 258 | signal cub and egf-like domain-containing protein 1-like isoform 1 | 687 | 1.28E-140 | 48.50% | 2 | F:protein binding; F:calcium ion binding | IPR000152 (PROSITE); IPR000436 (SMART); IPR000742 (SMART); IPR001881 (SMART); IPR002919 (SUPERFAMILY); IPR009030 (SUPERFAMILY); IPR011641 (PFAM); IPR013032 (PROSITE); IPR018097 (PROSITE); G3DSA:2.10.25.10 (GENE3D), G3DSA:2.10.70.10 (GENE3D), PTHR24838 (PANTHER), SSF57196 (SUPERFAMILY), SSF57586 (SUPERFAMILY) |
| 176 | lib_fal_Filter15_IDBA_contig_5268_plus1 | 21 | titin | 125 | 4.85E-19 | 53.85% | 15 | C:organelle; C:intracellular; F:protein kinase activity; P:multicellular organismal development; P:biological_process; P:regulation of biological process; P:nucleobase-containing compound metabolic process; P:anatomical structure morphogenesis; P:cell differentiation; P:cytoskeleton organization; P:transport; F:protein binding; C:cytoplasm; P:cellular protein modification process; F:cytoskeletal protein binding | IPR007110 (PROFILE); IPR013098 (PFAM); IPR013783 (G3DSA:2.60.40.GENE3D); PTHR10489 (PANTHER), SSF48726 (SUPERFAMILY) |
| 177 | lib_fal_Filter15_IDBA_contig_528_plus1 | 395 | signal cub egf-like 2 | 674 | 6.46E-81 | 44.70% | 1 | F:calcium ion binding | IPR000152 (PROSITE); IPR000436 (PROFILE); IPR001881 (SMART); IPR002919 (SUPERFAMILY); IPR011641 (PFAM); IPR018097 (PROSITE); G3DSA:2.10.25.10 (GENE3D), PTHR24039 (PANTHER), SSF57196 (SUPERFAMILY), SSF57586 (SUPERFAMILY) |

| | | | | | | | | | |
|---|---|---|---|---|---|---|---|---|---|
| 178 | lib_fal_Filter15_IDBA_contig_5324_minus2 | 28 | | 205 | 0 | 100% | 1 | F:protein binding | IPR003961 (PFAM); IPR013783 (G3DSA:2.60.40.GENE3D); PR00014 (PRINTS), SSF48726 (SUPERFAMILY) |
| 179 | lib_fal_Filter15_IDBA_contig_5348_plus2 | 21 | ttn partial | 124 | 6.66E-36 | 67.85% | 28 | F:cytoskeletal protein binding; F:protein kinase activity; P:anatomical structure morphogenesis; P:cellular component organization; P:embryo development; C:cytoskeleton; C:cytoplasm; P:cell differentiation; P:multicellular organismal development; P:cytoskeleton organization; P:biological_process; C:cytosol; F:structural molecule activity; P:response to external stimulus; P:response to stress; P:signal transduction; P:transport; P:nucleobase-containing compound metabolic process; F:protein binding; P:metabolic process; P:regulation of biological process; C:protein complex; C:nuclear chromosome; F:actin binding; F:calcium ion binding; P:organelle organization; P:cell cycle; C:extracellular | IPR003598 (SMART); IPR003599 (SMART); IPR007110 (PROFILE); IPR013098 (PFAM); IPR013783 (G3DSA:2.60.40.GENE3D); IPR020675 (PANTHER); SSF48726 (SUPERFAMILY) |
| 180 | lib_fal_Filter15_IDBA_contig_537_plus1 | 2069 | annexin a13 isoform 2 | 1264 | 2.48E-121 | 70.35% | 3 | F:calcium ion binding; F:lipid binding; F:binding | IPR001464 (PRINTS); IPR007087 (PROSITE); IPR018252 (PROSITE); IPR018502 (G3DSA:1.10.220.GENE3D); tmhmm (TMHMM) |
| 181 | lib_fal_Filter15_IDBA_contig_538_plus3 | 1647 | annexin a7-like | 690 | 4.28E-10 | 76.95% | 10 | P:multicellular organismal development; F:calcium ion binding; P:cellular homeostasis; P:behavior; F:lipid binding; C:organelle; F:actin binding; F:protein binding; C:cell; C:intracellular | IPR001464 (PANTHER); IPR018502 (G3DSA:1.10.220.GENE3D); tmhmm (TMHMM) |
| 182 | lib_fal_Filter15_IDBA_contig_55_minus2 | 6509 | intermediate filament protein | 516 | 2.38E-169 | 77.95% | 10 | P:biological_process; P:regulation of biological process; P:growth; P:multicellular organismal development; F:protein binding; F:structural molecule activity; P:embryo development; C:cytoskeleton; C:protein complex; F:nucleotide binding | IPR001664 (PANTHER); IPR010978 (SUPERFAMILY); IPR016044 (PFAM); IPR016451 (PIR); IPR018039 (PROSITE); G3DSA:1.20.5.170 (GENE3D) |
| 183 | lib_fal_Filter15_IDBA_contig_554_minus1 | 166 | myosin light chain smooth partial | 622 | 4.18E-12 | 54.15% | 4 | F:transferase activity; C:cell; P:biological_process; F:protein binding | IPR003598 (SMART); IPR003599 (SMART); IPR007110 (PROFILE); IPR013098 (PFAM); IPR013783 (G3DSA:2.60.40.GENE3D); PTHR10489 (PANTHER), SSF48726 (SUPERFAMILY) |
| 184 | lib_fal_Filter15_IDBA_contig_5567_minus2 | 16 | loc733382 protein | 121 | 1.77E-12 | 53.60% | 1 | F:carbohydrate binding | IPR000922 (PFAM); PTHR12011 (PANTHER), PTHR12011:SF4 (PANTHER) |
| 185 | lib_fal_Filter15_IDBA_contig_5580_plus2 | 20 | | 121 | 0 | 100% | 1 | F:protein binding | IPR003961 (PFAM); IPR013783 (G3DSA:2.60.40.GENE3D); IPR020675 (PANTHER); PR00014 (PRINTS), PTHR22964:SF14 (PANTHER) |
| 186 | lib_fal_Filter15_IDBA_contig_5602_minus2 | 14 | paraoxonase 1-like | 120 | 5.48E-17 | 56.00% | 9 | F:arylesterase activity; F:metal ion binding; F:aryldialkylphosphatase activity; F:hydrolase activity; C:extracellular space; C:extracellular region; P:aromatic compound catabolic process; F:protein homodimerization activity; P:carboxylic acid catabolic process | IPR011042 (G3DSA:2.120.10.GENE3D); PTHR11799 (PANTHER), PTHR11799:SF0 (PANTHER) |
| 187 | lib_fal_Filter15_IDBA_contig_5638_plus1 | 10 | adamts-like protein 5-like | 120 | 1.78E-13 | 53.55% | 1 | F:protein binding | IPR001134 (PROFILE); IPR008993 (SUPERFAMILY); IPR018933 (PFAM); G3DSA:2.40.50.120 (GENE3D) |

| | | | | | | | | | | |
|---|---|---|---|---|---|---|---|---|---|---|
| 188 | lib_fal_Filter15_IDBA_contig_5652_plus2 | 24 | glucose dehydrogenase | 120 | 2.84E-44 | 57.00% | 3 | P:cell differentiation; P:cell-cell signaling; P:metabolic process | IPR007867 (PFAM); G3DSA:3.30.560.10 (GENE3D), PTHR11552 (PANTHER), PTHR11552:SF10 (PANTHER), SSF54373 (SUPERFAMILY) | |
| 189 | lib_fal_Filter15_IDBA_contig_5703_minus2 | 17 | | 139 | 0 | 100% | 1 | F:protein binding | IPR007110 (PROFILE); IPR013098 (PFAM); IPR013783 (G3DSA:2.60.40.GENE3D); IPR020675 (PANTHER); IPR020682 (PTHR22964:PANTHER); SSF48726 (SUPERFAMILY) | |
| 190 | lib_fal_Filter15_IDBA_contig_573_plus3 | 170 | ankyrin repeat protein | 598 | 2.11E-16 | 50.60% | 1 | F:protein binding | IPR002110 (SMART); IPR020683 (G3DSA:1.25.40.GENE3D); PTHR24198 (PANTHER) | |
| 191 | lib_fal_Filter15_IDBA_contig_5734_plus1 | 14 | | 119 | 0 | 100% | 1 | F:protein binding | IPR003961 (PFAM); IPR013783 (G3DSA:2.60.40.GENE3D); PR00014 (PRINTS), PTHR10489 (PANTHER) | |
| 192 | lib_fal_Filter15_IDBA_contig_5806_plus3 | 27 | ---NA--- | 117 | | | 0 | - | IPR008160 (PFAM); PTHR24023 (PANTHER), tmhmm (TMHMM) | |
| 193 | lib_fal_Filter15_IDBA_contig_586_plus2 | 280 | metalloproteinase inhibitor 3 | 611 | 1.26E-08 | 39.65% | 2 | F:protein binding; F:enzyme regulator activity | IPR001134 (PROFILE); IPR001820 (PANTHER); IPR008993 (SUPERFAMILY); G3DSA:2.40.50.120 (GENE3D), PTHR11844:SF9 (PANTHER), SignalP-NN(euk) (SIGNALP), tmhmm (TMHMM) | Yes |
| 194 | lib_fal_Filter15_IDBA_contig_589_minus2 | 170 | annexin a7 | 602 | 2.56E-16 | 83.30% | 2 | F:calcium ion binding; F:lipid binding | IPR001464 (PANTHER); IPR018502 (G3DSA:1.10.220.GENE3D); tmhmm (TMHMM) | |
| 195 | lib_fal_Filter15_IDBA_contig_590_minus2 | 219 | annexin a7 | 599 | 2.53E-16 | 83.60% | 2 | F:calcium ion binding; F:lipid binding | IPR001464 (PANTHER); IPR018502 (G3DSA:1.10.220.GENE3D); tmhmm (TMHMM) | |
| 196 | lib_fal_Filter15_IDBA_contig_5932_plus2 | 13 | von willebrand factor type egf and pentraxin domain-containing protein 1 | 116 | 3.10E-22 | 55.15% | 5 | F:calcium ion binding; F:G-protein coupled receptor activity; C:membrane; P:G-protein coupled receptor signaling pathway; F:carbohydrate binding | IPR000436 (PFAM); G3DSA:2.10.70.10 (GENE3D), PTHR19325 (PANTHER) | |
| 197 | lib_fal_Filter15_IDBA_contig_6046_minus1 | 15 | glucose dehydrogenase | 115 | 3.13E-39 | 70.05% | 2 | F:nucleotide binding; P:metabolic process | IPR000172 (PFAM); IPR027424 (G3DSA:4.10.450.GENE3D); G3DSA:3.30.560.10 (GENE3D), PTHR11552 (PANTHER), PTHR11552:SF10 (PANTHER), SSF51905 (SUPERFAMILY) | |
| 198 | lib_fal_Filter15_IDBA_contig_6054_plus3 | 18 | cartilage oligomeric matrix | 114 | 1.53E-22 | 62.10% | 1 | F:protein binding | IPR000742 (SMART); G3DSA:2.10.25.10 (GENE3D), PTHR10199 (PANTHER), PTHR10199:SF4 (PANTHER) | |
| 199 | lib_fal_Filter15_IDBA_contig_607_plus1 | 314 | neurofilament medium polypeptide | 558 | 2.82E-10 | 52.55% | 2 | C:organelle; C:intracellular | no IPS match | |
| 200 | lib_fal_Filter15_IDBA_contig_607_plus3 | 314 | ---NA--- | 557 | | | 0 | - | no IPS match | |
| 201 | lib_fal_Filter15_IDBA_contig_609_plus2 | 155 | pdz domain-containing protein 2 | 542 | 4.51E-19 | 49.65% | 4 | P:metabolic process; F:hydrolase activity; C:intracellular; F:protein binding | IPR001478 (PFAM); G3DSA:2.30.42.10 (GENE3D), PTHR19964 (PANTHER) | |
| 202 | lib_fal_Filter15_IDBA_contig_6099_minus2 | 11 | angiopoietin-related protein 7 | 114 | 6.88E-24 | 58.80% | 2 | P:signal transduction; F:receptor binding | IPR002181 (PFAM); IPR014715 (G3DSA:4.10.530.GENE3D); IPR014716 (G3DSA:3.90.215.GENE3D); PTHR19143 (PANTHER) | |
| 203 | lib_fal_Filter15_IDBA_contig_611_minus1 | 8932 | | 1128 | 0 | 100% | 1 | F:protein binding | IPR003961 (PFAM); IPR013783 (G3DSA:2.60.40.GENE3D); PR00014 (PRINTS), tmhmm (TMHMM) | |
| 204 | lib_fal_Filter15_IDBA_contig_6127_plus1 | 32 | 3-oxoacyl-acyl-carrier-protein reductase | 114 | 4.09E-27 | 64.05% | 2 | P:metabolic process; F:catalytic activity | IPR002198 (PRINTS); IPR002347 (PRINTS); IPR016040 (G3DSA:3.40.50.GENE3D); PTHR24322 (PANTHER), PTHR24322:SF0 (PANTHER), SSF51735 (SUPERFAMILY) | |
| 205 | lib_fal_Filter15_IDBA_contig_6178_plus1 | 45 | 3-oxoacyl- | 114 | 1.03E-22 | 69.05% | 2 | P:metabolic process; F:catalytic activity | IPR002198 (PFAM); IPR016040 (G3DSA:3.40.50.GENE3D); PTHR24322 (PANTHER), PTHR24322:SF0 (PANTHER), SSF51735 (SUPERFAMILY) | |

| | | | | | | | | | |
|---|---|---|---|---|---|---|---|---|---|
| 206 | lib_fal_Filter15_IDBA_contig_6221_minus1 | 9 | cathepsin l | 113 | 2.34E-46 | 76.70% | 3 | F:peptidase activity; P:protein metabolic process; P:catabolic process | IPR000169 (PROSITE); IPR000668 (PFAM); IPR013128 (PANTHER); IPR013201 (PFAM); G3DSA:3.90.70.10 (GENE3D), PTHR12411:SF149 (PANTHER), SSF54001 (SUPERFAMILY) |
| 207 | lib_fal_Filter15_IDBA_contig_6229_plus3 | 10 | | 112 | 0 | 100% | 1 | F:protein binding | IPR003961 (PFAM); IPR013783 (G3DSA:2.60.40.GENE3D) |
| 208 | lib_fal_Filter15_IDBA_contig_6232_plus2 | 7 | basement membrane-specific heparan sulfate proteoglycan core | 113 | 5.36E-27 | 64.90% | 16 | P:regulation of biological process; P:cellular protein modification process; P:signal transduction; F:signal transducer activity; P:cellular component organization; C:extracellular space; C:cell; C:proteinaceous extracellular matrix; ; P:cell differentiation; P:cell-cell signaling; P:multicellular organismal development; P:growth; P:metabolic process; P:cell death; C:cellular_component | IPR001791 (PFAM); IPR008985 (SUPERFAMILY); IPR013320 (G3DSA:2.60.120.GENE3D); PTHR10574 (PANTHER) |
| 209 | lib_fal_Filter15_IDBA_contig_6239_minus3 | 28 | isoform b | 112 | 1.23E-04 | 66.00% | 1 | F:protein binding | IPR002172 (G3DSA:4.10.400.GENE3D); PTHR10529 (PANTHER) |
| 210 | lib_fal_Filter15_IDBA_contig_6266_plus1 | 18 | ---NA--- | 113 | | | 0 | - | IPR008160 (PFAM); PTHR24023 (PANTHER) |
| 211 | lib_fal_Filter15_IDBA_contig_6266_plus2 | 18 | ---NA--- | 112 | | | 0 | - | no IPS match |
| 212 | lib_fal_Filter15_IDBA_contig_6291_plus1 | 6 | limbic system-associated membrane protein | 112 | 3.88E-28 | 54.95% | 1 | F:protein binding | IPR007110 (PROFILE); IPR013098 (PFAM); IPR013783 (G3DSA:2.60.40.GENE3D); PTHR10489 (PANTHER), PF13895 (PFAM), SSF48726 (SUPERFAMILY) |
| 213 | lib_fal_Filter15_IDBA_contig_6296_minus3 | 7 | macrophage migration inhibitory factor variant 1 | 112 | 2.29E-05 | 49.91% | 30 | P:inflammatory response; F:dopachrome isomerase activity; C:cytoplasm; F:phenylpyruvate tautomerase activity; F:cytokine activity; C:extracellular region; P:embryonic morphogenesis; F:isomerase activity; P:innate immune response; P:cell proliferation; C:extracellular space; P:negative regulation of DNA damage response, signal transduction by p53 class mediator; F:cytokine receptor binding; C:cell surface; P:positive regulation of peptidyl-tyrosine phosphorylation; F:cell surface binding; P:prostaglandin biosynthetic process; P:positive regulation of cytokine secretion; P:positive regulation of ERK1 and ERK2 cascade; P:protein homotrimerization; P:positive regulation of protein kinase A signaling cascade; F:chemoattractant activity; P:positive regulation of B cell proliferation; P:negative regulation of gene expression; P:positive regulation of fibroblast proliferation; P:negative regulation of cell aging; P:cell surface receptor signaling pathway; P:negative regulation of cell cycle arrest; P:positive regulation of | IPR001398 (PRODOM); IPR014347 (SUPERFAMILY); G3DSA:3.30.429.10 (GENE3D) |
| 214 | lib_fal_Filter15_IDBA_contig_6330_plus1 | 14 | protein nep-2 | 112 | 1.20E-09 | 51.50% | 3 | F:peptidase activity; P:protein metabolic process; P:catabolic process | IPR000718 (PANTHER); IPR008753 (PFAM); IPR024079 (G3DSA:3.40.390.GENE3D); SSF55486 (SUPERFAMILY) |

| | | | | | | | | | | |
|---|---|---|---|---|---|---|---|---|---|---|
| 215 | lib_fal_Filter15_IDBA_contig_6336_plus3 | 11 | endothelin-converting enzyme 1-like | 111 | 2.08E-29 | 66.10% | 6 | C:cytoplasmic membrane-bounded vesicle; P:cell-cell signaling; P:multicellular organismal development; P:protein metabolic process; F:peptidase activity; P:catabolic process | IPR000718 (PANTHER); IPR018497 (PFAM); IPR024079 (G3DSA:3.40.390.GENE3D); SSF55486 (SUPERFAMILY) | |
| 216 | lib_fal_Filter15_IDBA_contig_6340_minus2 | 8 | cathepsin l | 112 | 1.88E-34 | 69.85% | 3 | P:protein metabolic process; P:catabolic process; F:peptidase activity | IPR000668 (PFAM); IPR013128 (PANTHER); G3DSA:3.90.70.10 (GENE3D), PTHR12411:SF149 (PANTHER), SSF54001 (SUPERFAMILY) | |
| 217 | lib_fal_Filter15_IDBA_contig_644_minus3 | 113 | sushi repeat-containing protein srpx2 | 508 | 2.17E-50 | 46.40% | 7 | F:ATP binding; F:protein kinase activity; P:protein phosphorylation; F:protein tyrosine kinase activity; F:transferase activity, transferring phosphorus-containing groups; F:calcium ion binding; F:carbohydrate binding | IPR011641 (PFAM); G3DSA:2.10.50.10 (GENE3D), PTHR22727 (PANTHER), PTHR22727:SF4 (PANTHER), SignalP-NN(euk) (SIGNALP), SSF57586 (SUPERFAMILY) | Yes |
| 218 | lib_fal_Filter15_IDBA_contig_6514_plus1 | 901 | ubiquitin-40s ribosomal protein s27a | 192 | 1.46E-77 | 97.00% | 28 | P:cell cycle; P:metabolic process; P:signal transduction; F:structural molecule activity; P:biological_process; P:response to stress; P:DNA metabolic process; P:reproduction; P:viral reproduction; P:translation; F:binding; C:nucleoplasm; P:protein metabolic process; P:catabolic process; P:regulation of biological process; P:cell death; P:cellular component organization; C:plasma membrane; P:protein transport; ; C:endosome; C:cytoplasmic membrane-bounded vesicle; P:nucleobase-containing compound metabolic process; C:ribosome; C:cytosol; P:cellular protein modification process; P:transport; F:protein binding | IPR000626 (PFAM); IPR002906 (PFAM); IPR019954 (PROSITE); IPR019955 (PROFILE); IPR019956 (PRINTS); G3DSA:3.10.20.90 (GENE3D), PTHR10666 (PANTHER), SSF54236 (SUPERFAMILY) | |
| 219 | lib_fal_Filter15_IDBA_contig_6534_plus2 | 12 | deleted in malignant brain tumors 1 | 110 | 5.00E-10 | 65.15% | 2 | F:receptor activity; C:cell | IPR000519 (G3DSA:4.10.110.GENE3D); IPR001190 (PRINTS); IPR017448 (SUPERFAMILY); G3DSA:3.10.250.10 (GENE3D), PTHR19331 (PANTHER), PTHR19331:SF118 (PANTHER) | |
| 220 | lib_fal_Filter15_IDBA_contig_6554_plus1 | 38 | ---NA--- | 169 | | | 0 | - | no IPS match | |
| 221 | lib_fal_Filter15_IDBA_contig_6561_plus2 | 14 | ankyrin-3 isoform 2 | 109 | 1.38E-18 | 71.90% | 20 | P:anatomical structure morphogenesis; P:cellular component organization; P:cell differentiation; P:multicellular organismal development; F:cytoskeletal protein binding; P:metabolic process; P:regulation of biological process; C:cytoskeleton; C:cellular_component; C:endoplasmic reticulum; P:cell communication; F:protein binding; C:cell; C:cytoplasm; P:cellular homeostasis; P:ion transport; C:plasma membrane; P:cell cycle; P:protein transport; F:structural molecule activity | IPR002110 (SMART); IPR020683 (G3DSA:1.25.40.GENE3D); PTHR24149 (PANTHER), PTHR24149:SF1 (PANTHER) | |
| 222 | lib_fal_Filter15_IDBA_contig_657_plus2 | 690 | gelsolin-like protein 2-like | 497 | 9.56E-171 | 71.45% | 6 | F:calcium ion binding; P:biological_process; P:cytoskeleton organization; F:actin binding; C:intracellular; P:regulation of biological process | IPR007122 (PRINTS); IPR007123 (PFAM); G3DSA:3.40.20.10 (GENE3D), PTHR11977:SF19 (PANTHER), SSF55753 (SUPERFAMILY) | |

| | | | | | | | | | | |
|---|---|---|---|---|---|---|---|---|---|---|
| 223 | lib_fal_Filter15_IDBA_contig_6579_plus1 | 41 | phosphoglycerate kinase | 110 | 3.00E-36 | 85.80% | 6 | F:kinase activity; P:metabolic process; F:nucleotide binding; P:generation of precursor metabolites and energy; P:carbohydrate metabolic process; P:catabolic process | IPR001576 (PRINTS); IPR015824 (G3DSA:3.40.50.GENE3D); IPR015911 (PROSITE) | |
| 224 | lib_fal_Filter15_IDBA_contig_6623_plus1 | 14 | isoform a | 109 | 3.66E-14 | 54.05% | 1 | P:biological_process | IPR003591 (SMART); G3DSA:3.80.10.10 (GENE3D), PTHR24367 (PANTHER), PF13855 (PFAM), SSF52058 (SUPERFAMILY) | |
| 225 | lib_fal_Filter15_IDBA_contig_6669_plus1 | 16 | ---NA--- | 109 | | | 0 | - | IPR008160 (PFAM); PTHR24023 (PANTHER) | |
| 226 | lib_fal_Filter15_IDBA_contig_669_plus1 | 123 | | 486 | 0 | 100% | 1 | F:protein binding | IPR003599 (SMART); IPR007110 (PROFILE); IPR013098 (PFAM); IPR013783 (G3DSA:2.60.40.GENE3D); SSF48726 (SUPERFAMILY) | |
| 227 | lib_fal_Filter15_IDBA_contig_6703_plus2 | 15 | leg5_rat ame: full=galectin-5 short=gal-5 ame: full=rl-18 | 108 | 3.07E-11 | 58.30% | 1 | F:carbohydrate binding | IPR001079 (PFAM); IPR008985 (SUPERFAMILY); IPR013320 (G3DSA:2.60.120.GENE3D); PTHR11346 (PANTHER) | |
| 228 | lib_fal_Filter15_IDBA_contig_6853_plus3 | 19 | immunoglobulin i-set domain protein | 106 | 5.36E-18 | 51.70% | 3 | F:protein kinase activity; F:nucleotide binding; P:cellular protein modification process | IPR003599 (SMART); IPR013098 (PFAM); IPR013783 (G3DSA:2.60.40.GENE3D); IPR015726 (PTHR22964:PANTHER); IPR020675 (PANTHER); SSF48726 (SUPERFAMILY) | |
| 229 | lib_fal_Filter15_IDBA_contig_6880_minus3 | 21 | | 177 | 0 | 100% | 1 | F:protein binding | IPR003961 (PFAM); IPR013783 (G3DSA:2.60.40.GENE3D); PR00014 (PRINTS) | |
| 230 | lib_fal_Filter15_IDBA_contig_6943_minus1 | 9 | 3-oxoacyl-acyl-carrier-protein reductase | 106 | 1.33E-27 | 65.45% | 2 | P:metabolic process; F:catalytic activity | IPR002198 (PRINTS); IPR002347 (PRINTS); IPR016040 (G3DSA:3.40.50.GENE3D); PTHR24322 (PANTHER), PTHR24322:SF0 (PANTHER), SSF51735 (SUPERFAMILY) | |
| 231 | lib_fal_Filter15_IDBA_contig_6952_plus3 | 23 | cub and sushi domain-containing protein 1- partial | 105 | 9.06E-19 | 53.80% | 1 | C:integral to membrane | IPR000436 (PFAM); G3DSA:2.10.70.10 (GENE3D), PTHR19325 (PANTHER) | |
| 232 | lib_fal_Filter15_IDBA_contig_696_minus3 | 569 | superoxide dismutase | 742 | 4.77E-74 | 80.20% | 3 | F:binding; P:metabolic process; F:catalytic activity | IPR001424 (PRINTS); IPR018152 (PROSITE); IPR024134 (PANTHER); SignalP-NN(euk) (SIGNALP) | Yes |
| 233 | lib_fal_Filter15_IDBA_contig_699_plus2 | 308 | low quality protein: obscurin-like | 890 | 3.62E-170 | 49.70% | 6 | F:transferase activity; C:intracellular; F:enzyme regulator activity; P:signal transduction; F:protein binding; F:lipid binding | IPR000219 (G3DSA:1.20.900.GENE3D); IPR001452 (SUPERFAMILY); IPR001849 (PROFILE); IPR003598 (SMART); IPR003599 (SMART); IPR007110 (PROFILE); IPR011993 (G3DSA:2.30.29.GENE3D); IPR013098 (PFAM); IPR013783 (G3DSA:2.60.40.GENE3D); G3DSA:2.30.30.40 (GENE3D), PTHR22826 (PANTHER), PTHR22826:SF5 (PANTHER), SSF48726 (SUPERFAMILY), SSF50729 (SUPERFAMILY) | |
| 234 | lib_fal_Filter15_IDBA_contig_6990_plus3 | 10 | protein lethal giant larvae-like | 105 | 2.08E-38 | 74.05% | 1 | F:protein binding | IPR015943 (G3DSA:2.130.10.GENE3D); IPR017986 (SUPERFAMILY); PTHR10241 (PANTHER) | |
| 235 | lib_fal_Filter15_IDBA_contig_70_minus3 | 4118 | soma ferritin | 421 | 1.02E-100 | 89.70% | 6 | C:cytoplasm; P:cellular homeostasis; F:binding; F:catalytic activity; P:ion transport; P:metabolic process | IPR001519 (PANTHER); IPR008331 (PFAM); IPR009040 (PROFILE); IPR009078 (SUPERFAMILY); IPR012347 (G3DSA:1.20.1260.GENE3D); IPR014034 (PROSITE); tmhmm (TMHMM) | |
| 236 | lib_fal_Filter15_IDBA_contig_7052_minus2 | 16 | deleted in malignant brain tumors 1 | 105 | 1.86E-10 | 68.50% | 5 | F:receptor activity; P:response to external stimulus; P:response to stress; C:cell; F:catalytic activity | IPR001190 (PFAM); IPR017448 (SUPERFAMILY); G3DSA:3.10.250.10 (GENE3D), PTHR19331 (PANTHER), PTHR19331:SF118 (PANTHER) | |
| 237 | lib_fal_Filter15_IDBA_contig_7053_plus2 | 6 | retinal dehydrogenase 1 | 105 | 1.76E-52 | 89.40% | 9 | F:catalytic activity; P:signal transduction; P:lipid metabolic process; P:secondary metabolic process; P:metabolic process; C:cytosol; P:catabolic process; F:lipid binding; F:enzyme regulator activity | IPR015590 (PFAM); IPR016161 (SUPERFAMILY); IPR016163 (G3DSA:3.40.309.GENE3D); PTHR11699 (PANTHER), PTHR11699:SF46 (PANTHER) | |
| 238 | lib_fal_Filter15_IDBA_contig_7139_plus3 | 6 | peptidyl-prolyl cis-trans isomerase fkbp3-like | 104 | 4.19E-25 | 72.80% | 6 | P:protein metabolic process; F:binding; P:cellular protein modification process; C:cell; C:nucleus; F:catalytic activity | IPR001179 (PFAM); IPR023566 (PANTHER); G3DSA:3.10.50.40 (GENE3D), PTHR10516:SF115 (PANTHER), SSF54534 (SUPERFAMILY) | |

| | | | | | | | | | |
|---|---|---|---|---|---|---|---|---|---|
| 239 | lib_fal_Filter15_IDBA_contig_7170_plus3 | 28 | multiple egf-like-domains 6 | 103 | 1.77E-30 | 64.55% | 1 | F:protein binding | IPR000742 (PROFILE); IPR013032 (PFAM); PD936484 (PRODOM), G3DSA:2.170.300.10 (GENE3D), PTHR24035 (PANTHER), PTHR24035:SF4 (PANTHER), SSF57196 (SUPERFAMILY) |
| 240 | lib_fal_Filter15_IDBA_contig_7271_plus1 | 18 | inhibitor of trypsin and hageman factor-like | 103 | 3.94E-14 | 63.45% | 3 | F:enzyme regulator activity; P:response to external stimulus; P:response to stress | IPR000864 (PRODOM); G3DSA:3.30.10.10 (GENE3D) |
| 241 | lib_fal_Filter15_IDBA_contig_730_plus3 | 136 | protein disulfide isomerase | 455 | 8.72E-148 | 79.45% | 13 | P:metabolic process; P:lipid metabolic process; F:catalytic activity; C:cytoplasm; P:regulation of biological process; P:cellular homeostasis; F:electron carrier activity; C:cell; C:endoplasmic reticulum; F:protein binding; P:cellular protein modification process; ; C:extracellular region | IPR005746 (PRINTS); IPR005788 (TIGRFAMs); IPR012336 (G3DSA:3.40.30.GENE3D); IPR013766 (PFAM); IPR017937 (PROSITE); PTHR18929 (PANTHER), PTHR18929:SF32 (PANTHER), PF13848 (PFAM) |
| 242 | lib_fal_Filter15_IDBA_contig_7321_plus2 | 14 | leucine-rich repeat-containing protein 4b | 102 | 1.15E-13 | 54.70% | 3 | P:regulation of biological process; P:multicellular organismal development; P:cellular component organization | G3DSA:3.80.10.10 (GENE3D), PTHR24365 (PANTHER), PF13855 (PFAM), SSF52058 (SUPERFAMILY) |
| 243 | lib_fal_Filter15_IDBA_contig_739_plus1 | 5063 | enolase | 950 | 1.89E-143 | 86.20% | 12 | P:reproduction; P:multicellular organismal development; P:embryo development; C:cytosol; C:protein complex; P:regulation of biological process; P:growth; F:catalytic activity; F:binding; P:generation of precursor metabolites and energy; P:carbohydrate metabolic process; P:catabolic process | IPR000941 (PRINTS); IPR020809 (PROSITE); IPR020810 (PFAM); G3DSA:3.20.20.120 (GENE3D), SSF51604 (SUPERFAMILY) |
| 244 | lib_fal_Filter15_IDBA_contig_740_plus2 | 5771 | enolase | 1159 | 0 | 85.85% | 12 | P:reproduction; P:multicellular organismal development; P:embryo development; C:cytosol; C:protein complex; P:regulation of biological process; P:growth; F:catalytic activity; F:binding; P:generation of precursor metabolites and energy; P:carbohydrate metabolic process; P:catabolic process | IPR000941 (PRINTS); IPR020809 (PROSITE); IPR020810 (PFAM); IPR020811 (PFAM); G3DSA:3.20.20.120 (GENE3D), G3DSA:3.30.390.10 (GENE3D), SSF51604 (SUPERFAMILY), SSF54826 (SUPERFAMILY) |
| 245 | lib_fal_Filter15_IDBA_contig_742_plus2 | 114 | alpha-enolase-like isoform 1 | 205 | 6.66E-97 | 85.25% | 18 | P:response to biotic stimulus; C:cell; P:regulation of biological process; P:cell growth; P:biological_process; C:cytosol; C:protein complex; F:binding; P:generation of precursor metabolites and energy; P:carbohydrate metabolic process; P:catabolic process; P:biosynthetic process; F:protein binding; ; C:plasma membrane; F:hydrolase activity; P:embryo development; F:catalytic activity | IPR000941 (PRINTS); IPR020810 (PFAM); IPR020811 (PFAM); G3DSA:3.20.20.120 (GENE3D), G3DSA:3.30.390.10 (GENE3D), SSF51604 (SUPERFAMILY), SSF54826 (SUPERFAMILY) |
| 246 | lib_fal_Filter15_IDBA_contig_743_plus1 | 141 | enolase 3-2 | 190 | 2.03E-83 | 89.90% | 12 | P:response to biotic stimulus; P:regulation of biological process; P:cell growth; C:cytosol; C:protein complex; F:binding; P:generation of precursor metabolites and energy; P:carbohydrate metabolic process; P:catabolic process; P:biosynthetic process; ; F:catalytic activity | IPR000941 (PRINTS); IPR020809 (PROSITE); IPR020810 (PFAM); G3DSA:3.20.20.120 (GENE3D), SSF51604 (SUPERFAMILY) |
| 247 | lib_fal_Filter15_IDBA_contig_753_plus2 | 370 | macrophage mannose receptor 1 | 538 | 1.66E-12 | 39.10% | 1 | F:carbohydrate binding | IPR001304 (PFAM); IPR016186 (G3DSA:3.10.100.GENE3D); IPR016187 (SUPERFAMILY); IPR018378 (PROSITE); PTHR22803 (PANTHER) |

| | | | | | | | | | | |
|---|---|---|---|---|---|---|---|---|---|---|
| 248 | lib_fal_Filter15_IDBA_contig_759_minus2 | 109 | deleted in malignant brain tumors 1 | 443 | 2.46E-41 | 57.35% | 2 | F:receptor activity; C:cell | IPR001190 (PRINTS); IPR017448 (SMART); G3DSA:3.10.250.10 (GENE3D), PTHR19331 (PANTHER) | |
| 249 | lib_fal_Filter15_IDBA_contig_7634_plus1 | 110 | hypothetical protein CAPTEDRAFT_223820 | 248 | 1.43E-28 | 66.00% | 0 | - | IPR021712 (PFAM); SignalP-NN(euk) (SIGNALP), tmhmm (TMHMM) | Yes |
| 250 | lib_fal_Filter15_IDBA_contig_7635_plus2 | 107 | hypothetical protein CAPTEDRAFT_223820 | 242 | 1.45E-28 | 66.00% | 0 | - | IPR021712 (PFAM); SignalP-NN(euk) (SIGNALP), tmhmm (TMHMM) | Yes |
| 251 | lib_fal_Filter15_IDBA_contig_7637_minus2 | 34 | granulin precursor | 120 | 4.45E-27 | 60.00% | 3 | C:organelle; C:intracellular; F:receptor binding | IPR000118 (PFAM); PTHR12274 (PANTHER), PTHR12274:SF0 (PANTHER), SSF57277 (SUPERFAMILY) | |
| 252 | lib_fal_Filter15_IDBA_contig_7659_minus1 | 60 | granulin- partial | 165 | 4.15E-44 | 59.35% | 3 | F:protein binding; C:organelle; C:intracellular | IPR000118 (PFAM); PTHR12274 (PANTHER), PTHR12274:SF0 (PANTHER), SSF57277 (SUPERFAMILY) | |
| 253 | lib_fal_Filter15_IDBA_contig_7665_minus1 | 27 | titin | 183 | 6.56E-19 | 49.00% | 14 | C:organelle; C:intracellular; F:protein kinase activity; C:cytoplasm; P:anatomical structure morphogenesis; P:cell differentiation; P:multicellular organismal development; P:cytoskeleton organization; P:metabolic process; P:regulation of biological process; P:primary metabolic process; P:biological_process; F:cytoskeletal protein binding; P:nucleobase-containing compound metabolic process | IPR007110 (PROFILE); IPR013098 (PFAM); IPR013783 (G3DSA:2.60.40.GENE3D); PTHR10489 (PANTHER), SSF48726 (SUPERFAMILY) | |
| 254 | lib_fal_Filter15_IDBA_contig_7666_minus1 | 34 | titin | 202 | 3.15E-20 | 49.40% | 14 | C:cytoplasm; C:organelle; C:intracellular; F:protein kinase activity; P:anatomical structure morphogenesis; P:cell differentiation; P:multicellular organismal development; P:cytoskeleton organization; P:metabolic process; P:regulation of biological process; P:primary metabolic process; P:biological_process; F:cytoskeletal protein binding; P:nucleobase-containing compound metabolic process | IPR007110 (PROFILE); IPR013098 (PFAM); IPR013783 (G3DSA:2.60.40.GENE3D); PTHR10489 (PANTHER), SSF48726 (SUPERFAMILY) | |
| 255 | lib_fal_Filter15_IDBA_contig_7714_plus1 | 46 | fibrillin-1- partial | 116 | 1.20E-05 | 50.94% | 2 | F:protein binding; F:calcium ion binding | IPR000152 (PROSITE); IPR000742 (SMART); IPR001881 (PFAM); G3DSA:2.10.25.10 (GENE3D), PTHR24032 (PANTHER), PTHR24032:SF0 (PANTHER), SSF57196 (SUPERFAMILY) | |
| 256 | lib_fal_Filter15_IDBA_contig_7895_minus3 | 22 | beta- -endoglucanase | 114 | 3.83E-11 | 48.85% | 2 | F:catalytic activity; P:carbohydrate metabolic process | IPR001701 (PFAM); IPR008928 (SUPERFAMILY); IPR012341 (G3DSA:1.50.10.GENE3D); PTHR22298 (PANTHER), PTHR22298:SF3 (PANTHER) | |
| 257 | lib_fal_Filter15_IDBA_contig_7968_plus3 | 111 | four-domain proteases inhibitor | 125 | 6.22E-13 | 49.90% | 1 | F:protein binding | IPR002350 (PFAM); G3DSA:1.10.1890.10 (GENE3D), G3DSA:3.30.60.30 (GENE3D), PTHR10913 (PANTHER), PTHR10913:SF21 (PANTHER), SignalP-NN(euk) (SIGNALP), tmhmm (TMHMM), SSF100895 (SUPERFAMILY) | Yes |
| 258 | lib_fal_Filter15_IDBA_contig_7972_plus2 | 97 | alpha beta hydrolase fold-1 domain-containing protein | 107 | 5.71E-13 | 57.22% | 10 | P:glucan biosynthetic process; P:biosynthetic process; F:transferase activity; F:transferase activity, transferring glycosyl groups; F:starch synthase activity; F:zinc ion binding; C:intracellular; P:carbohydrate metabolic process; F:cation binding; F:catalytic activity | no IPS match | |

| | | | | | | | | | | |
|---|---|---|---|---|---|---|---|---|---|---|
| 259 | lib_fal_Filter15_IDBA_contig_7975_minus1 | 39 | multiple epidermal growth factor-like domains protein 6-like | 137 | 3.60E-26 | 60.00% | 1 | F:protein binding | IPR000742 (SMART); IPR002049 (SMART); IPR013032 (PFAM); PR00011 (PRINTS), G3DSA:2.170.300.10 (GENE3D), PTHR24035 (PANTHER), PTHR24035:SF4 (PANTHER), SSF57196 (SUPERFAMILY) | |
| 260 | lib_fal_Filter15_IDBA_contig_7976_minus1 | 34 | multiple epidermal growth factor-like domains protein 6-like | 126 | 2.12E-26 | 61.15% | 1 | F:protein binding | IPR000742 (SMART); IPR002049 (SMART); IPR013032 (PFAM); PR00011 (PRINTS), G3DSA:2.170.300.10 (GENE3D), PTHR24035 (PANTHER), PTHR24035:SF4 (PANTHER), SSF57196 (SUPERFAMILY) | |
| 261 | lib_fal_Filter15_IDBA_contig_805_minus1 | 152 | collagen triple helix repeat domain protein | 418 | 7.38E-05 | 58.00% | 1 | C:collagen | IPR008160 (PFAM); PTHR24023 (PANTHER), PTHR24023:SF164 (PANTHER), tmhmm (TMHMM) | |
| 262 | lib_fal_Filter15_IDBA_contig_805_plus3 | 152 | ---NA--- | 417 | | | 0 | - | no IPS match | |
| 263 | lib_fal_Filter15_IDBA_contig_824_plus3 | 346 | protein | 409 | 2.25E-40 | 52.25% | 3 | F:peptidase activity; P:protein metabolic process; P:catabolic process | IPR001254 (PFAM); IPR001314 (PRINTS); IPR009003 (SUPERFAMILY); IPR018114 (PROSITE); G3DSA:2.40.10.10 (GENE3D), PTHR24265 (PANTHER), SignalP-NN(euk) (SIGNALP), tmhmm (TMHMM) | Yes |
| 264 | lib_fal_Filter15_IDBA_contig_850_plus2 | 1036 | | 695 | 0 | 100% | 1 | F:protein binding | IPR003599 (SMART); IPR003961 (PFAM); IPR007110 (PROFILE); IPR013098 (PFAM); IPR013783 (G3DSA:2.60.40.GENE3D); PR00014 (PRINTS), SSF48726 (SUPERFAMILY) | |
| 265 | lib_fal_Filter15_IDBA_contig_860_plus3 | 85 | protein unc- isoform b | 393 | 1.22E-49 | 46.55% | 3 | C:intracellular; P:biological_process; F:protein binding | IPR003598 (SMART); IPR003599 (SMART); IPR007110 (PROFILE); IPR013098 (PFAM); IPR013783 (G3DSA:2.60.40.GENE3D); SSF48726 (SUPERFAMILY) | |
| 266 | lib_fal_Filter15_IDBA_contig_881_minus1 | 9144 | flavohemoprotein | 494 | 3.67E-50 | 55.15% | 3 | F:binding; P:transport; F:oxygen binding | IPR000971 (PFAM); IPR009050 (SUPERFAMILY); IPR012292 (G3DSA:1.10.490.GENE3D); IPR013316 (PRINTS); PTHR22924 (PANTHER), tmhmm (TMHMM) | |
| 267 | lib_fal_Filter15_IDBA_contig_900_plus3 | 85 | histone h2av | 382 | 3.18E-64 | 99.00% | 6 | C:chromosome; F:DNA binding; P:organelle organization; P:multicellular organismal development; C:nucleus; F:protein binding | IPR002119 (PRINTS); IPR007125 (PFAM); IPR009072 (G3DSA:1.10.20.GENE3D); PTHR23430 (PANTHER) | |
| 268 | lib_fal_Filter15_IDBA_contig_935_plus1 | 134 | adp-ribosylation factor 1 | 375 | 1.11E-125 | 98.25% | 25 | F:signal transducer activity; P:anatomical structure morphogenesis; P:cellular component organization; P:regulation of biological process; C:Golgi apparatus; F:protein binding; P:biological_process; P:viral reproduction; P:transport; P:cell-cell signaling; F:nucleotide binding; P:cellular homeostasis; P:nucleobase-containing compound metabolic process; P:catabolic process; C:cytosol; P:response to stress; P:response to biotic stimulus; P:signal transduction; P:multicellular organismal development; C:cytoplasm; F:hydrolase activity; C:plasma membrane; P:organelle organization; P:cellular protein modification process; P:protein transport | IPR003579 (SMART); IPR005225 (TIGRFAMs); IPR006687 (SMART); IPR006689 (PRINTS); IPR024156 (SMART); IPR027417 (SUPERFAMILY); G3DSA:3.40.50.300 (GENE3D), PTHR11711 (PANTHER) | |

| | | | | | | | | | | |
|---|---|---|---|---|---|---|---|---|---|---|
| 269 | lib_fal_Filter15_IDBA_contig_937_minus1 | 1661 | guanine nucleotide-binding protein subunit beta-2-like 1 | 375 | 0 | 93.70% | 25 | P:regulation of biological process; P:cell death; C:cytoskeleton; F:protein binding; C:cell; C:ribosome; F:receptor activity; F:receptor binding; P:biological_process; P:cell cycle; P:cellular component organization; C:plasma membrane; P:translation; P:cellular protein modification process; P:transport; P:signal transduction; C:cytoplasm; F:molecular_function; P:protein metabolic process; P:catabolic process; F:enzyme regulator activity; F:kinase activity; C:nucleus; P:cell growth; P:metabolic process | IPR001680 (PFAM); IPR015943 (G3DSA:2.130.10.GENE3D); IPR017986 (PROFILE); IPR019775 (PROSITE); IPR020472 (PRINTS); PTHR19868 (PANTHER), PTHR19868:SF0 (PANTHER) | |
| 270 | lib_fal_Filter15_IDBA_contig_950_minus1 | 470 | cationic trypsin-like | 285 | 4.59E-38 | 60.80% | 3 | F:peptidase activity; P:protein metabolic process; P:catabolic process | IPR001254 (PFAM); IPR001314 (PRINTS); IPR009003 (SUPERFAMILY); IPR018114 (PROSITE); G3DSA:2.40.10.10 (GENE3D), PTHR24256 (PANTHER) | |
| 271 | lib_fal_Filter15_IDBA_contig_952_minus2 | 169 | vacuolar sorting protein | 372 | 1.96E-23 | 53.30% | 4 | F:peptidase activity; P:protein metabolic process; P:catabolic process; F:protein binding | IPR001254 (PFAM); IPR002172 (G3DSA:4.10.400.GENE3D); IPR009003 (SUPERFAMILY); IPR018114 (PROSITE); PR00261 (PRINTS), G3DSA:2.40.10.10 (GENE3D), PTHR24275 (PANTHER), SignalP-NN(euk) (SIGNALP) | Yes |
| 272 | lib_fal_Filter15_IDBA_contig_96_minus1 | 1290 | ubiquitin family protein | 226 | 6.59E-42 | 99.90% | 27 | P:cell cycle; P:signal transduction; F:structural molecule activity; P:biological_process; P:response to stress; P:DNA metabolic process; P:reproduction; P:viral reproduction; P:translation; F:binding; C:nucleoplasm; P:protein metabolic process; P:catabolic process; P:regulation of biological process; P:cell death; F:protein binding; P:cellular component organization; C:plasma membrane; P:protein transport; ; C:endosome; C:cytoplasmic membrane-bounded vesicle; P:cellular protein modification process; P:nucleobase-containing compound metabolic process; C:ribosome; C:cytosol; P:transport | IPR000626 (PFAM); IPR019954 (PROSITE); IPR019955 (PROFILE); IPR019956 (PRINTS); G3DSA:3.10.20.90 (GENE3D), PTHR10666 (PANTHER), tmhmm (TMHMM), SSF54236 (SUPERFAMILY) | |
| 273 | lib_fal_Filter15_IDBA_contig_993_plus2 | 162 | a chain crystal structures of hsc70bag1 in complex with small molecule inhibitors | 364 | 0 | 96.20% | 23 | P:cellular component organization; P:transport; P:cell-cell signaling; C:cytoplasmic membrane-bounded vesicle; P:regulation of biological process; ; C:nucleolus; C:cytosol; C:cell; P:cell cycle; F:protein binding; P:protein metabolic process; C:organelle; C:extracellular region; C:protein complex; F:hydrolase activity; C:plasma membrane; P:nucleobase-containing compound metabolic process; F:nucleotide binding; C:intracellular; P:response to stress; P:response to biotic stimulus; P:biological_process | IPR013126 (PRINTS); IPR018181 (PROSITE); G3DSA:3.30.30.30 (GENE3D), G3DSA:3.30.420.40 (GENE3D), G3DSA:3.90.640.10 (GENE3D), PTHR19375 (PANTHER), SSF53067 (SUPERFAMILY) | |

## Supplementary Table 6

| Nr. | Contig sequence name | Read numbers | Sequence description (BLAST nr, E-value= > 0.0001) | Length (aa pos.) | E-Value | mean Similarity | Nr. of GO terms | Gene ontology terms and annotation (GO's) | Interpro scan results (BLAST2GO) | SignalP 4.1 (3.0) |
|---|---|---|---|---|---|---|---|---|---|---|
| 1 | lib_dib_Filter15_IDBA_contig_0_minus 2 | 6373 | ---NA--- | 6585 | | | 0 | - | IPR008160 (PFAM); PTHR24023 (PANTHER) | |
| 2 | lib_dib_Filter15_IDBA_contig_0_minus 3 | 6373 | multiple banded antigen | 6585 | 2.91E-06 | 43.50% | 2 | P:proteolysis; F:cysteine-type peptidase activity | no IPS match | |
| 3 | lib_dib_Filter15_IDBA_contig_0_plus1 | 6373 | ---NA--- | 6585 | | | 0 | - | no IPS match | |
| 4 | lib_dib_Filter15_IDBA_contig_1_minus 2 | 20 | collagen alpha-5 chain | 217 | 9.31E-33 | 55.85% | 1 | F:protein binding | IPR002035 (PFAM); PR00453 (PRINTS), G3DSA:3.40.50.410 (GENE3D), PTHR22992 (PANTHER), SSF53300 (SUPERFAMILY) | |
| 5 | lib_dib_Filter15_IDBA_contig_100033_ minus2 | 10 | arylsulfatase | 115 | 1.17E-19 | 60.70% | 2 | F:catalytic activity; P:metabolic process | IPR017850 (SUPERFAMILY); G3DSA:3.30.1120.10 (GENE3D), PTHR10342 (PANTHER), PTHR10342:SF19 (PANTHER) | |
| 6 | lib_dib_Filter15_IDBA_contig_100057_ minus2 | 170 | cholinesterase precursor | 275 | 1.71E-27 | 61.05% | 7 | F:binding; C:organelle; C:intracellular; F:hydrolase activity; P:biological_process; P:metabolic process; C:extracellular region | IPR000997 (PRINTS); IPR002018 (PFAM); G3DSA:3.40.50.1820 (GENE3D), PTHR11559 (PANTHER), PTHR11559:SF33 (PANTHER), SSF53474 (SUPERFAMILY) | |
| 7 | lib_dib_Filter15_IDBA_contig_100058_ minus2 | 227 | cholinesterase precursor | 260 | 6.33E-28 | 60.25% | 7 | F:binding; C:organelle; C:intracellular; F:hydrolase activity; P:biological_process; P:metabolic process; C:extracellular region | IPR000997 (PRINTS); IPR002018 (PFAM); G3DSA:3.40.50.1820 (GENE3D), PTHR11559 (PANTHER), PTHR11559:SF33 (PANTHER), SSF53474 (SUPERFAMILY) | |
| 8 | lib_dib_Filter15_IDBA_contig_100059_ minus1 | 55 | cholinesterase precursor | 105 | 1.03E-20 | 68.70% | 8 | C:extracellular space; F:protein binding; P:cell-cell signaling; ; P:biological_process; C:endoplasmic reticulum; F:hydrolase activity; P:secondary metabolic process | IPR000997 (PRINTS); IPR002018 (PFAM); G3DSA:3.40.50.1820 (GENE3D), PTHR11559 (PANTHER), PTHR11559:SF33 (PANTHER), SSF53474 (SUPERFAMILY) | |
| 9 | lib_dib_Filter15_IDBA_contig_100095_ plus3 | 10033 | tryptase gamma precursor | 386 | 2.26E-65 | 52.80% | 3 | F:peptidase activity; P:protein metabolic process; P:catabolic process | IPR001254 (PFAM); IPR001314 (PRINTS); IPR009003 (SUPERFAMILY); G3DSA:2.40.10.10 (GENE3D), PTHR24265 (PANTHER), SignalP-NN(euk) (SIGNALP), tmhmm (TMHMM) | Yes |
| 10 | lib_dib_Filter15_IDBA_contig_100178_ plus3 | 13 | zinc finger protein 366 | 114 | 1.58E-50 | 71.05% | 3 | F:nucleic acid binding; C:intracellular; F:binding | IPR007087 (PFAM); IPR013087 (G3DSA:3.30.160.GENE3D); IPR015880 (SMART); PTHR11389 (PANTHER), PF13465 (PFAM), SSF57667 (SUPERFAMILY) | |
| 11 | lib_dib_Filter15_IDBA_contig_100335_ plus3 | 13 | ga-binding protein subunit beta-1 isoform 2 | 114 | 1.37E-54 | 83.55% | 5 | P:regulation of biological process; ; F:DNA binding; F:protein binding; C:nucleus | IPR002110 (PRINTS); IPR020683 (G3DSA:1.25.40.GENE3D); PTHR24193 (PANTHER), PTHR24193:SF0 (PANTHER) | |
| 12 | lib_dib_Filter15_IDBA_contig_100337_ minus1 | 6 | oocyte zinc finger protein 6-partial | 115 | 1.22E-21 | 62.70% | 3 | F:binding; F:nucleic acid binding; C:intracellular | IPR007087 (PROSITE); IPR013087 (G3DSA:3.30.160.GENE3D); IPR015880 (SMART); PTHR24382 (PANTHER), PTHR24382:SF0 (PANTHER), PF13465 (PFAM), SSF57667 (SUPERFAMILY) | |
| 13 | lib_dib_Filter15_IDBA_contig_100362_ minus2 | 13 | multiple epidermal growth factor-like domains protein 6 precursor | 114 | 4.77E-19 | 58.80% | 2 | F:protein binding; F:calcium ion binding | IPR000152 (PROSITE); IPR000742 (SMART); IPR001881 (PFAM); IPR013032 (PROSITE); IPR018097 (PROSITE); G3DSA:2.10.25.10 (GENE3D), PTHR24046 (PANTHER), SSF57196 (SUPERFAMILY) | |
| 14 | lib_dib_Filter15_IDBA_contig_100746_ minus2 | 27 | lysosomal pro-x carboxypeptidase | 114 | 8.93E-47 | 77.05% | 7 | F:peptidase activity; P:response to external stimulus; P:response to stress; C:plasma membrane; F:protein binding; P:protein metabolic process; P:catabolic process | IPR008758 (PANTHER); G3DSA:3.40.50.1820 (GENE3D), PTHR11010:SF9 (PANTHER), tmhmm (TMHMM), SSF53474 (SUPERFAMILY) | |

| | | | | | | | | | |
|---|---|---|---|---|---|---|---|---|---|
| 15 | lib_dib_Filter15_IDBA_contig_100783_minus1 | 7 | signal cub egf-like isoform cra_c | 114 | 5.45E-14 | 62.60% | 6 | P:biological_process; C:plasma membrane; P:cellular component organization; F:protein binding; C:extracellular space; F:calcium ion binding | IPR000152 (PROSITE); IPR000742 (SMART); IPR001881 (SMART); IPR013032 (PROSITE); IPR023413 (G3DSA:2.40.155.GENE3D); IPR024731 (PFAM); G3DSA:2.10.25.10 (GENE3D), PTHR24046 (PANTHER), SSF57196 (SUPERFAMILY) |
| 16 | lib_dib_Filter15_IDBA_contig_100848_plus2 | 18 | protein sel-1 homolog 1 | 114 | 5.68E-11 | 56.65% | 8 | P:multicellular organismal development; P:cellular component organization; C:extracellular space; P:metabolic process; C:extracellular region; F:peptidase activity; P:anatomical structure morphogenesis; C:cell | IPR000562 (G3DSA:2.10.10.GENE3D); IPR013806 (SUPERFAMILY); PR00013 (PRINTS), PTHR22918 (PANTHER), PTHR22918:SF0 (PANTHER) |
| 17 | lib_dib_Filter15_IDBA_contig_100870_minus2 | 12 | pr domain zinc finger protein partial | 114 | 1.61E-21 | 55.15% | 8 | P:regulation of biological process; P:nucleobase-containing compound metabolic process; P:DNA metabolic process; C:intracellular; P:metabolic process; P:cell cycle; F:binding; F:nucleic acid binding | IPR007087 (PROSITE); IPR013087 (G3DSA:3.30.160.GENE3D); IPR015880 (SMART); PTHR24380 (PANTHER), PTHR24380:SF0 (PANTHER), PF13465 (PFAM), SSF57667 (SUPERFAMILY) |
| 18 | lib_dib_Filter15_IDBA_contig_100987_plus1 | 6 | isoform a | 114 | 4.85E-11 | 52.90% | 6 | P:multicellular organismal development; P:regulation of biological process; P:response to stress; C:extracellular region; F:carbohydrate binding; P:biological_process | IPR000421 (PFAM); IPR006585 (SMART); IPR008979 (SUPERFAMILY); G3DSA:2.60.120.260 (GENE3D), PTHR19277 (PANTHER), PTHR19277:SF6 (PANTHER), SignalP-NN(euk) (SIGNALP) |
| 19 | lib_dib_Filter15_IDBA_contig_10111_plus3 | 671 | translocation protein sec63-like protein | 921 | 0 | 73.10% | 2 | F:protein binding; F:transporter activity | IPR001623 (PRINTS); IPR004179 (PFAM); IPR027137 (PANTHER); G3DSA:1.10.150.20 (GENE3D), G3DSA:1.10.3380.10 (GENE3D), G3DSA:2.60.40.150 (GENE3D), SignalP-NN(euk) (SIGNALP), tmhmm (TMHMM) |
| 20 | lib_dib_Filter15_IDBA_contig_101144_minus2 | 12 | zinc finger protein 271-like | 114 | 3.30E-27 | 62.00% | 3 | F:binding; F:nucleic acid binding; C:intracellular | IPR007087 (PROSITE); IPR013087 (G3DSA:3.30.160.GENE3D); IPR015880 (SMART); PTHR24402 (PANTHER), PF13465 (PFAM), SSF57667 (SUPERFAMILY) |
| 21 | lib_dib_Filter15_IDBA_contig_101189_plus1 | 20 | fibrinogen c domain-containing protein 1-like | 134 | 6.45E-36 | 64.20% | 3 | F:receptor binding; C:extracellular space; P:signal transduction | IPR002181 (PFAM); IPR014715 (G3DSA:4.10.530.GENE3D); IPR014716 (G3DSA:3.90.215.GENE3D); PTHR19143 (PANTHER) |
| 22 | lib_dib_Filter15_IDBA_contig_101190_plus1 | 23 | fibrinogen c domain-containing protein 1-like | 147 | 1.62E-40 | 63.90% | 3 | F:receptor binding; C:extracellular space; P:signal transduction | IPR002181 (PFAM); IPR014715 (G3DSA:4.10.530.GENE3D); IPR014716 (G3DSA:3.90.215.GENE3D); PTHR19143 (PANTHER) |
| 23 | lib_dib_Filter15_IDBA_contig_101222_plus1 | 46 | gig1_stigi ame: full=gigantoxin-1 ame: full=egf-like peptide toxin ame: full=gigantoxin i short=gigt i flags: precursor | 183 | 7.86E-04 | 59.00% | 1 | F:protein binding | IPR000742 (PROFILE); IPR013032 (PROSITE); G3DSA:2.10.25.10 (GENE3D), PTHR10529 (PANTHER), PTHR10529:SF134 (PANTHER), SignalP-NN(euk) (SIGNALP), SSF57196 (SUPERFAMILY) |
| 24 | lib_dib_Filter15_IDBA_contig_10127_plus2 | 1396 | immunoglobulin i-set domain protein | 254 | 1.68E-31 | 57.95% | 9 | F:protein kinase activity; P:biological_process; C:cytoplasm; F:protein binding; P:anatomical structure morphogenesis; P:cell differentiation; P:multicellular organismal development; P:cytoskeleton organization; F:enzyme regulator activity | IPR003598 (SMART); IPR003599 (SMART); IPR007110 (PROFILE); IPR013098 (PFAM); IPR013783 (G3DSA:2.60.40.GENE3D); PTHR10489 (PANTHER), SSF48726 (SUPERFAMILY) |
| 25 | lib_dib_Filter15_IDBA_contig_10133_plus1 | 215 | class b secretin-like g-protein coupled receptor | 590 | 1.98E-59 | 49.05% | 6 | P:signal transduction; F:receptor activity; C:cell; P:anatomical structure morphogenesis; P:regulation of biological process; P:multicellular organismal development | IPR000203 (PFAM); IPR000832 (PRINTS); IPR008077 (PRINTS); IPR017981 (PROFILE); IPR022624 (PFAM); PTHR12011 (PANTHER), tmhmm (TMHMM), SSF81321 (SUPERFAMILY) |

| | | | | | | | | |
|---|---|---|---|---|---|---|---|---|
| 26 | lib_dib_Filter15_IDBA_contig_101386_minus2 | 6 | protocadherin fat 4 | 113 | 8.41E-20 | 60.60% | 3 | C:cell; F:calcium ion binding; P:biological_process | IPR002126 (PRINTS); IPR015919 (SUPERFAMILY); PTHR24027 (PANTHER) |
| 27 | lib_dib_Filter15_IDBA_contig_101505_minus1 | 9 | udp-n-acetyl-alpha-d-galactosamine:polypeptide n-acetylgalactosaminyltransferase isoform a | 114 | 4.50E-59 | 78.80% | 6 | F:transferase activity; C:Golgi apparatus; F:carbohydrate binding; P:biosynthetic process; P:carbohydrate metabolic process; C:cell | IPR001173 (PFAM); G3DSA:3.90.550.10 (GENE3D), PTHR11675 (PANTHER), SSF53448 (SUPERFAMILY) |
| 28 | lib_dib_Filter15_IDBA_contig_101518_minus2 | 8 | probable beta-d-xylosidase 2-like | 113 | 2.15E-54 | 77.00% | 2 | P:carbohydrate metabolic process; F:hydrolase activity | IPR001764 (G3DSA:3.20.20.GENE3D); IPR017853 (SUPERFAMILY); IPR026892 (PANTHER); PTHR30620:SF0 (PANTHER) |
| 29 | lib_dib_Filter15_IDBA_contig_101526_minus1 | 9 | acidic mammalian chitinase-like | 114 | 3.54E-31 | 62.60% | 2 | P:carbohydrate metabolic process; F:hydrolase activity | IPR001223 (PFAM); IPR013781 (G3DSA:3.20.20.GENE3D); IPR017853 (SUPERFAMILY); G3DSA:3.10.50.10 (GENE3D), PTHR11177 (PANTHER), PTHR11177:SF82 (PANTHER), SSF54556 (SUPERFAMILY) |
| 30 | lib_dib_Filter15_IDBA_contig_101552_minus2 | 13 | stan | 113 | 3.82E-40 | 68.40% | 6 | F:receptor activity; F:signal transducer activity; P:signal transduction; F:calcium ion binding; P:biological_process; C:plasma membrane | IPR002126 (PRINTS); IPR015919 (SUPERFAMILY); IPR020894 (PROSITE); PTHR24026 (PANTHER), PTHR24026:SF3 (PANTHER) |
| 31 | lib_dib_Filter15_IDBA_contig_1016_minus1 | 15 | myosin heavy chain | 161 | 3.05E-46 | 81.95% | 5 | C:cytoskeleton; C:protein complex; F:nucleotide binding; F:actin binding; F:motor activity | IPR002928 (PFAM); PTHR13140 (PANTHER), PTHR13140:SF109 (PANTHER) |
| 32 | lib_dib_Filter15_IDBA_contig_101680_plus2 | 6 | exostosin-1 | 113 | 2.89E-30 | 68.90% | 12 | P:anatomical structure morphogenesis; P:cellular component organization; P:cell differentiation; P:multicellular organismal development; P:embryo development; C:Golgi apparatus; C:endoplasmic reticulum; P:biosynthetic process; P:carbohydrate metabolic process; F:protein binding; F:transferase activity; P:signal transduction | PTHR11062 (PANTHER), PTHR11062:SF7 (PANTHER) |
| 33 | lib_dib_Filter15_IDBA_contig_101780_minus2 | 11 | btb poz domain-containing protein 17-like | 113 | 1.97E-43 | 63.20% | 1 | F:protein binding | IPR011333 (G3DSA:3.30.710.GENE3D); IPR013069 (PFAM); PTHR24410 (PANTHER) |
| 34 | lib_dib_Filter15_IDBA_contig_101854_minus2 | 27 | isoform b | 146 | 1.01E-29 | 53.95% | 10 | C:plasma membrane; F:protein binding; P:regulation of biological process; P:cellular component organization; P:multicellular organismal development; P:anatomical structure morphogenesis; P:embryo development; P:biological_process; P:signal transduction; P:cell differentiation | IPR000742 (PFAM); IPR008985 (SUPERFAMILY); IPR013032 (PROSITE); IPR013320 (G3DSA:2.60.120.GENE3D); G3DSA:2.10.25.10 (GENE3D), PTHR24044 (PANTHER), SSF57196 (SUPERFAMILY) |
| 35 | lib_dib_Filter15_IDBA_contig_101956_minus2 | 8 | apolipoprotein a-i-binding | 113 | 7.62E-44 | 71.95% | 6 | C:extracellular space; F:binding; F:protein binding; C:cell; F:catalytic activity; F:nucleotide binding | IPR004443 (G3DSA:3.40.50.GENE3D); PTHR13232 (PANTHER) |

| | | | | | | | | | |
|---|---|---|---|---|---|---|---|---|---|
| 36 | lib_dib_Filter15_IDBA_contig_102079_plus1 | 25189 | ubiquitin-40s ribosomal protein s27a | 191 | 9.30E-77 | 97.20% | 28 | P:cell cycle; P:metabolic process; P:signal transduction; F:structural molecule activity; P:biological_process; P:response to stress; P:DNA metabolic process; P:reproduction; P:viral reproduction; P:translation; F:binding; C:nucleoplasm; P:protein metabolic process; P:catabolic process; P:regulation of biological process; P:cell death; P:cellular component organization; C:plasma membrane; P:protein transport; ; C:endosome; C:cytoplasmic membrane-bounded vesicle; P:nucleobase-containing compound metabolic process; C:ribosome; C:cytosol; P:cellular protein modification process; P:transport; F:protein binding | IPR000626 (PFAM); IPR002906 (PFAM); IPR019954 (PROSITE); IPR019955 (PROFILE); IPR019956 (PRINTS); G3DSA:3.10.20.90 (GENE3D), PTHR10666 (PANTHER), SSF54236 (SUPERFAMILY) |
| 37 | lib_dib_Filter15_IDBA_contig_102173_plus3 | 9 | ---NA--- | 112 | | | 4 | F:receptor binding; C:extracellular region; P:multicellular organismal development; P:signal transduction | IPR005817 (PANTHER); SignalP-NN(euk) (SIGNALP) |
| 38 | lib_dib_Filter15_IDBA_contig_102217_plus2 | 8 | pr domain zinc finger protein 14-like | 113 | 4.51E-70 | 97.25% | 3 | F:binding; F:nucleic acid binding; C:intracellular | IPR007087 (PROSITE); IPR013087 (G3DSA:3.30.160.GENE3D); IPR015880 (SMART); PTHR11389 (PANTHER), PTHR11389:SF354 (PANTHER), PF13465 (PFAM), PF13894 (PFAM), SSF57667 (SUPERFAMILY) |
| 39 | lib_dib_Filter15_IDBA_contig_102414_minus3 | 6 | arylsulfatase b | 112 | 4.15E-38 | 77.00% | 12 | P:multicellular organismal development; F:binding; P:response to endogenous stimulus; P:response to abiotic stimulus; P:response to external stimulus; C:Golgi apparatus; C:lysosome; C:endoplasmic reticulum; F:hydrolase activity; P:biological_process; C:mitochondrion; P:catabolic process | IPR000917 (PFAM); IPR017849 (G3DSA:3.40.720.GENE3D); IPR017850 (SUPERFAMILY); PTHR10342 (PANTHER), PTHR10342:SF19 (PANTHER) |
| 40 | lib_dib_Filter15_IDBA_contig_102570_minus1 | 51 | carbohydrate sulfotransferase 11-like | 262 | 2.36E-10 | 52.05% | 4 | F:transferase activity; C:cell; P:biosynthetic process; P:carbohydrate metabolic process | IPR005331 (PFAM); IPR018011 (PANTHER); PTHR12137:SF14 (PANTHER) |
| 41 | lib_dib_Filter15_IDBA_contig_102578_minus1 | 9 | galactosamine (n-acetyl)-6-sulfate sulfatase | 113 | 1.23E-45 | 68.65% | 4 | F:hydrolase activity; P:metabolic process; F:peptidase activity; C:cell | IPR000917 (PFAM); IPR017849 (G3DSA:3.40.720.GENE3D); IPR017850 (SUPERFAMILY); IPR019756 (PROSITE); G3DSA:3.30.1120.10 (GENE3D), PTHR10342 (PANTHER), PTHR10342:SF27 (PANTHER) |
| 42 | lib_dib_Filter15_IDBA_contig_102591_plus3 | 14 | wingless | 112 | 8.81E-15 | 76.25% | 6 | F:binding; P:multicellular organismal development; C:proteinaceous extracellular matrix; F:electron carrier activity; F:receptor binding; P:signal transduction | IPR005817 (PANTHER) |
| 43 | lib_dib_Filter15_IDBA_contig_102647_minus2 | 12 | oocyte zinc finger protein 6-like | 112 | 1.30E-16 | 62.10% | 3 | F:binding; F:nucleic acid binding; C:intracellular | IPR007087 (PROSITE); IPR013087 (G3DSA:3.30.160.GENE3D); IPR015880 (SMART); PTHR11389 (PANTHER), PF13465 (PFAM), SSF57667 (SUPERFAMILY) |
| 44 | lib_dib_Filter15_IDBA_contig_102656_minus1 | 10 | serine threonine-protein kinase sik3 | 113 | 9.74E-67 | 92.25% | 5 | C:cytoplasm; P:cellular protein modification process; F:nucleotide binding; F:binding; F:protein kinase activity | IPR000719 (PFAM); IPR002290 (SMART); IPR008271 (PROSITE); IPR011009 (SUPERFAMILY); G3DSA:1.10.510.10 (GENE3D), G3DSA:3.30.200.20 (GENE3D), PTHR24343 (PANTHER), PTHR24343:SF26 (PANTHER) |
| 45 | lib_dib_Filter15_IDBA_contig_102679_plus2 | 23 | achain structural determinants for improved thermal stability of designed ankyrin repeat proteins with a redesigned c-capping | 112 | 1.27E-28 | 63.60% | 1 | F:protein binding | IPR002110 (PRINTS); IPR020683 (G3DSA:1.25.40.GENE3D); PTHR24198 (PANTHER) |

| | | | | | | | | | |
|---|---|---|---|---|---|---|---|---|---|
| 46 | lib_dib_Filter15_IDBA_contig_102984_plus3 | 19 | low quality protein: protocadherin-16-like | 128 | 4.10E-18 | 54.85% | 3 | C:cell; F:calcium ion binding; P:biological_process | IPR002126 (PRINTS); IPR015919 (SUPERFAMILY); PTHR24027 (PANTHER) |
| 47 | lib_dib_Filter15_IDBA_contig_103097_minus3 | 18 | thioredoxin domain-containing protein 11-like | 111 | 8.06E-25 | 61.20% | 2 | P:cellular homeostasis; P:regulation of biological process | IPR012336 (G3DSA:3.40.30.GENE3D); IPR013766 (PFAM); PTHR18929 (PANTHER), PTHR18929:SF32 (PANTHER), tmhmm (TMHMM) |
| 48 | lib_dib_Filter15_IDBA_contig_103236_plus2 | 13 | cubilin precursor | 112 | 1.13E-41 | 71.90% | 20 | P:protein transport; P:cellular component organization; F:RNA binding; C:protein complex; F:binding; P:metabolic process; P:response to external stimulus; C:plasma membrane; C:lysosome; F:receptor activity; F:protein binding; C:endosome; C:cell; C:cytoplasmic membrane-bounded vesicle; P:transport; P:biosynthetic process; P:lipid metabolic process; P:embryo development; C:Golgi apparatus; C:endoplasmic reticulum | IPR000859 (G3DSA:2.60.120.GENE3D); PTHR10127 (PANTHER), PTHR10127:SF310 (PANTHER) |
| 49 | lib_dib_Filter15_IDBA_contig_103292_plus1 | 20 | ---NA--- | 112 | | | 0 | - | no IPS match |
| 50 | lib_dib_Filter15_IDBA_contig_103292_plus3 | 20 | ---NA--- | 111 | | | 0 | - | no IPS match |
| 51 | lib_dib_Filter15_IDBA_contig_103446_minus1 | 10 | ---NA--- | 112 | | | 0 | - | no IPS match |
| 52 | lib_dib_Filter15_IDBA_contig_103448_minus2 | 10 | serine proteinase | 111 | 3.56E-25 | 56.75% | 3 | F:peptidase activity; P:protein metabolic process; P:catabolic process | IPR001254 (PFAM); IPR009003 (SUPERFAMILY); G3DSA:2.40.10.10 (GENE3D), PTHR24250 (PANTHER) |
| 53 | lib_dib_Filter15_IDBA_contig_103566_plus2 | 11 | oocyte zinc finger protein 6-like | 111 | 4.91E-31 | 67.20% | 3 | F:binding; F:nucleic acid binding; C:intracellular | IPR007087 (PFAM); IPR013087 (G3DSA:3.30.160.GENE3D); IPR015880 (SMART); PTHR24402 (PANTHER), PF13465 (PFAM), SSF57667 (SUPERFAMILY) |
| 54 | lib_dib_Filter15_IDBA_contig_103567_minus3 | 8 | protein wnt-10a | 111 | 1.09E-17 | 65.55% | 4 | P:multicellular organismal development; C:extracellular region; F:receptor binding; P:signal transduction | IPR005817 (PANTHER); IPR013302 (PTHR12027:PANTHER) |
| 55 | lib_dib_Filter15_IDBA_contig_103624_plus3 | 9 | metabotropic gaba-b receptor | 111 | 3.85E-51 | 80.55% | 3 | C:cell; F:receptor activity; P:signal transduction | IPR002455 (PRINTS); IPR002456 (PRINTS); IPR017978 (PFAM); PTHR10519 (PANTHER), PTHR10519:SF3 (PANTHER), SignalP-NN(euk) (SIGNALP), tmhmm (TMHMM) |
| 56 | lib_dib_Filter15_IDBA_contig_10379_plus3 | 772 | integral membrane protein 2b-like | 331 | 2.98E-74 | 50.10% | 0 | - | IPR007084 (PFAM); PTHR10962 (PANTHER), tmhmm (TMHMM) |
| 57 | lib_dib_Filter15_IDBA_contig_103797_minus2 | 7 | protein | 111 | 4.59E-17 | 48.65% | 0 | - | no IPS match |
| 58 | lib_dib_Filter15_IDBA_contig_103903_minus1 | 6 | bone morphogenetic protein 3-like | 111 | 1.38E-28 | 67.05% | 6 | C:organelle; P:regulation of biological process; ; C:extracellular space; P:biological_process; F:receptor binding | IPR001839 (PFAM); IPR002405 (PRINTS); IPR015615 (PANTHER); IPR017948 (PROSITE); G3DSA:2.10.90.10 (GENE3D), SSF57501 (SUPERFAMILY) |
| 59 | lib_dib_Filter15_IDBA_contig_104055_minus2 | 20 | ankyrin repeat domain protein | 111 | 1.93E-15 | 57.70% | 1 | F:protein binding | IPR002110 (PRINTS); IPR020683 (G3DSA:1.25.40.GENE3D); PTHR24198 (PANTHER) |
| 60 | lib_dib_Filter15_IDBA_contig_104140_plus1 | 635 | nidogen 2 precursor | 260 | 2.36E-10 | 47.70% | 4 | P:cell-matrix adhesion; F:calcium ion binding; P:proteolysis; F:peptidase activity | IPR000716 (G3DSA:4.10.800.GENE3D); PTHR12352 (PANTHER), SignalP-NN(euk) (SIGNALP), tmhmm (TMHMM) |
| 61 | lib_dib_Filter15_IDBA_contig_104184_minus2 | 7 | a disintegrin and metalloproteinase with thrombospondin motifs 10 | 111 | 1.33E-15 | 57.55% | 1 | F:hydrolase activity | IPR000884 (PFAM); G3DSA:2.20.100.10 (GENE3D), PTHR13723 (PANTHER) |
| 62 | lib_dib_Filter15_IDBA_contig_1042_minus1 | 821 | cathepsin f precursor | 123 | 1.48E-45 | 75.70% | 4 | F:enzyme regulator activity; P:protein metabolic process; P:catabolic process; F:peptidase activity | IPR000169 (PROSITE); IPR000668 (PFAM); IPR013128 (PANTHER); G3DSA:3.90.70.10 (GENE3D), PTHR12411:SF43 (PANTHER), SSF54001 (SUPERFAMILY) |

| | | | | | | | | | |
|---|---|---|---|---|---|---|---|---|---|
| 63 | lib_dib_Filter15_IDBA_contig_104228_minus3 | 10 | ---NA--- | 110 | | | 0 | - | IPR008160 (PFAM); PTHR24023 (PANTHER) |
| 64 | lib_dib_Filter15_IDBA_contig_104259_minus3 | 6 | zinc finger protein 271 (zinc finger protein 7) (zinc finger protein znfphex133) (epstein-barr virus-induced zinc finger protein) (znf-eb) (ct-zfp48) (zinc finger protein | 110 | 2.63E-29 | 73.45% | 3 | F:binding; F:nucleic acid binding; C:intracellular | IPR007087 (PROSITE); IPR013087 (G3DSA:3.30.160.GENE3D); IPR015880 (SMART); PTHR24387 (PANTHER), PF13465 (PFAM), SSF57667 (SUPERFAMILY) |
| 65 | lib_dib_Filter15_IDBA_contig_104281_plus1 | 27 | frizzled-4 | 145 | 1.00E-56 | 81.30% | 18 | P:behavior; P:signal transduction; C:plasma membrane; P:biological_process; F:protein binding; F:receptor activity; P:anatomical structure morphogenesis; P:cellular component organization; P:cell differentiation; P:regulation of biological process; ; C:cell; C:cytoplasm; P:reproduction; P:transport; P:cell-cell signaling; P:multicellular organismal development; P:embryo development | IPR015526 (PANTHER); IPR020067 (G3DSA:1.10.2000.GENE3D); IPR026551 (PTHR11309:PANTHER) |
| 66 | lib_dib_Filter15_IDBA_contig_104287_plus1 | 30 | calcium binding egf domain protein | 179 | 3.98E-36 | 60.95% | 1 | F:protein binding | IPR000742 (SMART); IPR002049 (PFAM); IPR013032 (PFAM); PR00011 (PRINTS), G3DSA:2.170.300.10 (GENE3D), PTHR24035 (PANTHER), PTHR24035:SF4 (PANTHER), SSF57196 (SUPERFAMILY) |
| 67 | lib_dib_Filter15_IDBA_contig_1043_minus1 | 949 | cathepsin f precursor | 123 | 2.60E-45 | 76.10% | 4 | F:enzyme regulator activity; P:protein metabolic process; P:catabolic process; F:peptidase activity | IPR000169 (PROSITE); IPR000668 (PFAM); IPR013128 (PANTHER); G3DSA:3.90.70.10 (GENE3D), PTHR12411:SF43 (PANTHER), SSF54001 (SUPERFAMILY) |
| 68 | lib_dib_Filter15_IDBA_contig_104331_plus1 | 12 | e3 ubiquitin-protein ligase hace1 | 111 | 6.22E-51 | 86.25% | 14 | F:protein binding; F:enzyme regulator activity; C:Golgi apparatus; P:cell cycle; P:regulation of biological process; ; P:cellular protein modification process; F:catalytic activity; P:catabolic process; P:cellular component organization; P:organelle organization; P:signal transduction; C:endoplasmic reticulum; C:nucleus | IPR002110 (SMART); IPR020683 (G3DSA:1.25.40.GENE3D); PTHR11254 (PANTHER), PTHR11254:SF51 (PANTHER) |
| 69 | lib_dib_Filter15_IDBA_contig_104478_plus1 | 15 | leukocyte elastase inhibitor | 111 | 1.99E-20 | 61.05% | 2 | C:extracellular space; F:enzyme regulator activity | IPR000215 (PANTHER); IPR023796 (PFAM); G3DSA:3.30.497.10 (GENE3D), tmhmm (TMHMM) |
| 70 | lib_dib_Filter15_IDBA_contig_104558_minus3 | 11 | zinc finger protein 271 (zinc finger protein 7) (zinc finger protein znfphex133) (epstein-barr virus-induced zinc finger protein) (znf-eb) (ct-zfp48) (zinc finger protein | 110 | 1.24E-32 | 66.30% | 3 | F:binding; F:nucleic acid binding; C:intracellular | IPR007087 (PROSITE); IPR013087 (G3DSA:3.30.160.GENE3D); IPR015880 (SMART); PTHR24375 (PANTHER), PTHR24375:SF0 (PANTHER), PF13465 (PFAM), SSF57667 (SUPERFAMILY) |
| 71 | lib_dib_Filter15_IDBA_contig_104728_minus2 | 20 | scavenger receptor cysteine-rich type 1 protein m130-partial | 110 | 2.92E-22 | 60.25% | 2 | F:receptor activity; C:cell | IPR001190 (PRINTS); IPR017448 (SMART); G3DSA:3.10.250.10 (GENE3D), PTHR19331 (PANTHER), PTHR19331:SF118 (PANTHER) |
| 72 | lib_dib_Filter15_IDBA_contig_104777_minus2 | 8 | ds_drome ame: full=protein dachsous ame: full=adherin flags: precursor | 110 | 1.17E-38 | 75.75% | 3 | F:calcium ion binding; P:biological_process; C:plasma membrane | IPR002126 (PRINTS); IPR015919 (SUPERFAMILY); IPR020894 (PROSITE); PTHR24027 (PANTHER), PTHR24027:SF64 (PANTHER) |
| 73 | lib_dib_Filter15_IDBA_contig_104793_plus2 | 9 | thrombospondin type-1 domain-containing protein 7a | 110 | 7.54E-18 | 53.25% | 0 | - | IPR000884 (SUPERFAMILY); G3DSA:2.20.100.10 (GENE3D), PTHR11311 (PANTHER), PTHR11311:SF2 (PANTHER) |
| 74 | lib_dib_Filter15_IDBA_contig_104809_plus1 | 8 | serpin b4 | 110 | 1.16E-21 | 63.80% | 3 | P:metabolic process; P:regulation of biological process; F:enzyme regulator activity | IPR000215 (PANTHER); IPR023796 (PFAM); G3DSA:2.30.39.10 (GENE3D), G3DSA:3.30.497.10 (GENE3D) |

| | | | | | | | | | |
|---|---|---|---|---|---|---|---|---|---|
| 75 | lib_dib_Filter15_IDBA_contig_104868_plus2 | 9 | threonine synthase-like 1 | 110 | 1.20E-43 | 78.30% | 6 | P:biosynthetic process; ; F:binding; F:catalytic activity; F:kinase activity; F:nucleotide binding | IPR001926 (SUPERFAMILY); G3DSA:3.40.50.1100 (GENE3D), PTHR10314 (PANTHER), PTHR10314:SF45 (PANTHER) |
| 76 | lib_dib_Filter15_IDBA_contig_104898_minus1 | 8 | protocadherin fat 4 | 110 | 6.85E-19 | 59.05% | 4 | F:catalytic activity; F:calcium ion binding; P:biological_process; C:plasma membrane | IPR002126 (PRINTS); IPR010221 (TIGRFAMs); IPR015919 (SUPERFAMILY); IPR020894 (PROSITE); PTHR24027 (PANTHER) |
| 77 | lib_dib_Filter15_IDBA_contig_104952_plus1 | 14 | zinc finger protein 426 | 110 | 1.39E-35 | 68.00% | 3 | F:nucleic acid binding; C:intracellular; F:binding | IPR007087 (PROSITE); IPR013087 (G3DSA:3.30.160.GENE3D); IPR015880 (SMART); PTHR24381 (PANTHER), PF13465 (PFAM), SSF57667 (SUPERFAMILY) |
| 78 | lib_dib_Filter15_IDBA_contig_104962_plus3 | 7 | zinc finger protein 271-like | 110 | 3.46E-32 | 63.75% | 3 | F:binding; F:nucleic acid binding; C:intracellular | IPR007087 (PROSITE); IPR013087 (G3DSA:3.30.160.GENE3D); IPR015880 (SMART); PTHR24402 (PANTHER), PF13465 (PFAM), SSF57667 (SUPERFAMILY) |
| 79 | lib_dib_Filter15_IDBA_contig_105066_plus1 | 16 | galectin-3 | 110 | 4.00E-13 | 69.20% | 4 | P:biological_process; C:organelle; C:intracellular; F:carbohydrate binding | IPR001079 (PFAM); IPR008985 (SUPERFAMILY); IPR013320 (G3DSA:2.60.120.GENE3D); PTHR11346 (PANTHER) |
| 80 | lib_dib_Filter15_IDBA_contig_10508_plus1 | 1000 | matrix metalloproteinase-14-like | 467 | 2.95E-79 | 67.10% | 14 | P:cellular component organization; P:anatomical structure morphogenesis; P:multicellular organismal development; C:cell; P:cell death; F:peptidase activity; P:biological_process; P:growth; P:response to external stimulus; P:response to stress; P:protein metabolic process; P:catabolic process; F:binding; C:extracellular region | IPR001818 (PFAM); IPR002477 (PFAM); IPR006026 (SMART); IPR021190 (PRINTS); IPR024079 (G3DSA:3.40.390.GENE3D); PTHR10201 (PANTHER), tmhmm (TMHMM), SSF55486 (SUPERFAMILY) |
| 81 | lib_dib_Filter15_IDBA_contig_105091_minus1 | 10 | membrane frizzled-related protein | 110 | 6.43E-12 | 54.45% | 1 | F:protein binding | IPR000859 (G3DSA:2.60.120.GENE3D); IPR002172 (G3DSA:4.10.400.GENE3D); IPR023415 (PROSITE); PTHR10127 (PANTHER), PTHR10127:SF310 (PANTHER) |
| 82 | lib_dib_Filter15_IDBA_contig_105153_plus3 | 9 | zinc finger protein 271 (zinc finger protein 7) (zinc finger protein znfphex133) (epstein-barr virus-induced zinc finger protein) (znf-eb) (ct-zfp48) (zinc finger protein | 109 | 9.64E-23 | 61.20% | 3 | F:binding; F:nucleic acid binding; C:intracellular | IPR007087 (PROSITE); IPR013087 (G3DSA:3.30.160.GENE3D); IPR015880 (SMART); PTHR11389 (PANTHER), PF13465 (PFAM), SSF57667 (SUPERFAMILY) |
| 83 | lib_dib_Filter15_IDBA_contig_105155_minus1 | 13 | ankyrin repeat and sam domain-containing protein 6 | 110 | 1.93E-28 | 73.60% | 1 | F:protein binding | IPR002110 (PRINTS); IPR020683 (G3DSA:1.25.40.GENE3D); PTHR10627 (PANTHER), PTHR10627:SF2 (PANTHER) |
| 84 | lib_dib_Filter15_IDBA_contig_10528_plus2 | 429 | protein lin-7 homolog c | 617 | 2.49E-105 | 91.25% | 8 | F:cytoskeletal protein binding; C:plasma membrane; P:transport; F:protein binding; P:cell-cell signaling; C:cell; C:cytoskeleton; P:protein transport | IPR001478 (PFAM); IPR004172 (SMART); IPR014775 (PFAM); G3DSA:2.30.42.10 (GENE3D), PTHR14063 (PANTHER), PTHR14063:SF2 (PANTHER), tmhmm (TMHMM), SSF101288 (SUPERFAMILY) |
| 85 | lib_dib_Filter15_IDBA_contig_105536_plus3 | 9 | zinc finger protein 271-like | 109 | 8.08E-25 | 70.85% | 3 | F:binding; F:nucleic acid binding; C:intracellular | IPR007087 (PROSITE); IPR013087 (G3DSA:3.30.160.GENE3D); IPR015880 (SMART); PTHR24382 (PANTHER), PTHR24382:SF0 (PANTHER), PF13465 (PFAM), SSF57667 (SUPERFAMILY) |
| 86 | lib_dib_Filter15_IDBA_contig_105599_minus1 | 7 | plasma alpha-l-fucosidase | 110 | 5.15E-32 | 69.65% | 2 | P:carbohydrate metabolic process; F:hydrolase activity | IPR000933 (PANTHER); IPR013780 (G3DSA:2.60.40.GENE3D); IPR013781 (G3DSA:3.20.20.GENE3D); IPR017853 (SUPERFAMILY); PTHR10030:SF3 (PANTHER) |
| 87 | lib_dib_Filter15_IDBA_contig_105603_plus2 | 9 | low-density lipoprotein receptor-related protein 2 | 109 | 3.10E-29 | 60.15% | 2 | F:catalytic activity; F:protein binding | IPR002172 (G3DSA:4.10.400.GENE3D); IPR023415 (PROSITE); PR00261 (PRINTS), PTHR10529 (PANTHER) |

| | | | | | | | | | |
|---|---|---|---|---|---|---|---|---|---|
| 88 | lib_dib_Filter15_IDBA_contig_105638_minus1 | 16 | dehydrogenase reductase sdr family member 11-like | 110 | 4.10E-23 | 68.15% | 3 | P:metabolic process; F:catalytic activity; F:nucleotide binding | IPR002198 (PFAM); IPR016040 (G3DSA:3.40.50.GENE3D); PTHR24322 (PANTHER), PTHR24322:SF7 (PANTHER), SSF51735 (SUPERFAMILY) |
| 89 | lib_dib_Filter15_IDBA_contig_105654_plus2 | 10 | discoidin domain-containing receptor 2 | 109 | 9.04E-59 | 81.45% | 16 | P:reproduction; P:multicellular organismal development; P:biological_process; P:regulation of biological process; P:anatomical structure morphogenesis; P:cell growth; C:plasma membrane; ; C:extracellular region; P:cell proliferation; P:cellular component organization; P:metabolic process; P:growth; P:cell differentiation; F:protein binding; F:nucleotide binding | IPR000719 (PROFILE); IPR001245 (PFAM); IPR011009 (SUPERFAMILY); IPR020635 (SMART); G3DSA:1.10.510.10 (GENE3D), PTHR24416 (PANTHER), PTHR24416:SF64 (PANTHER) |
| 90 | lib_dib_Filter15_IDBA_contig_1057_plus1 | 2288 | eosinophil peroxidase | 128 | 1.18E-44 | 70.20% | 5 | F:antioxidant activity; F:catalytic activity; P:response to stress; F:binding; P:metabolic process | IPR002007 (G3DSA:1.10.640.GENE3D); IPR010255 (SUPERFAMILY); PTHR11475 (PANTHER) |
| 91 | lib_dib_Filter15_IDBA_contig_105746_minus3 | 232 | defense protein 3-like | 333 | 6.65E-16 | 50.35% | 3 | P:carbohydrate metabolic process; F:cation binding; F:catalytic activity | IPR002861 (PFAM); PTHR23130 (PANTHER), tmhmm (TMHMM) |
| 92 | lib_dib_Filter15_IDBA_contig_106019_minus2 | 201 | low quality protein: multiple epidermal growth factor-like domains protein 6 | 455 | 6.56E-173 | 63.55% | 1 | F:protein binding | IPR000742 (SMART); IPR002049 (PFAM); IPR013032 (PROSITE); PD936484 (PRODOM), PR00011 (PRINTS), G3DSA:2.170.300.10 (GENE3D), PTHR24035 (PANTHER), PTHR24035:SF4 (PANTHER), SSF57196 (SUPERFAMILY) |
| 93 | lib_dib_Filter15_IDBA_contig_106064_plus1 | 12 | isoform a | 109 | 2.42E-29 | 65.05% | 33 | P:multicellular organismal development; P:biological_process; P:reproduction; P:cell differentiation; P:embryo development; P:anatomical structure morphogenesis; P:cytoskeleton organization; P:cellular component organization; F:nucleotide binding; C:plasma membrane; F:receptor activity; P:signal transduction; P:cell death; P:regulation of biological process; P:cell-cell signaling; P:growth; P:behavior; C:protein complex; P:cell proliferation; ; C:organelle; C:cytoplasm; F:hydrolase activity; C:nucleus; P:response to biotic stimulus; P:cell cycle; F:chromatin binding; F:protein binding; P:response to external stimulus; P:response to stress; C:cell; F:DNA binding; F:calcium ion binding | IPR000152 (PROSITE); IPR000742 (PFAM); IPR001881 (SMART); IPR013032 (PROSITE); IPR018097 (PROSITE); PR00010 (PRINTS), G3DSA:2.10.25.10 (GENE3D), PTHR24044 (PANTHER), SSF57196 (SUPERFAMILY) |
| 94 | lib_dib_Filter15_IDBA_contig_106309_minus2 | 17 | low-density lipoprotein receptor-related protein 2 | 149 | 2.52E-12 | 62.60% | 1 | F:protein binding | IPR002172 (G3DSA:4.10.400.GENE3D); IPR023415 (PROSITE); PR00261 (PRINTS), PTHR10529 (PANTHER) |
| 95 | lib_dib_Filter15_IDBA_contig_106527_minus3 | 7 | natriuretic peptide receptor 1-like | 108 | 3.80E-18 | 53.95% | 4 | F:catalytic activity; F:receptor activity; P:signal transduction; C:cell | IPR000337 (PRINTS); IPR001828 (PFAM); G3DSA:3.40.50.2300 (GENE3D), PTHR11920 (PANTHER), PTHR11920:SF50 (PANTHER), SSF53822 (SUPERFAMILY) |
| 96 | lib_dib_Filter15_IDBA_contig_106656_minus1 | 10 | variable lymphocyte receptor a | 109 | 6.58E-13 | 62.25% | 1 | F:protein binding | IPR001611 (PROFILE); IPR003591 (SMART); IPR026906 (PFAM); G3DSA:3.80.10.10 (GENE3D), PTHR24365 (PANTHER), SSF52058 (SUPERFAMILY) |
| 97 | lib_dib_Filter15_IDBA_contig_106664_minus3 | 7 | cd163 antigen | 108 | 4.00E-16 | 65.75% | 4 | F:binding; P:biological_process; C:cell; F:receptor activity | IPR001190 (PRINTS); IPR017448 (SMART); G3DSA:3.10.250.10 (GENE3D), PTHR19331 (PANTHER), PTHR19331:SF118 (PANTHER) |

| | | | | | | | | | | |
|---|---|---|---|---|---|---|---|---|---|---|
| 98 | lib_dib_Filter15_IDBA_contig_106781_minus1 | 17 | protein | 109 | 5.25E-11 | 60.80% | 2 | F:kinase activity; F:protein binding | IPR001611 (PROFILE); G3DSA:3.80.10.10 (GENE3D), PTHR24365 (PANTHER), PF13855 (PFAM), SSF52058 (SUPERFAMILY) | |
| 99 | lib_dib_Filter15_IDBA_contig_106947_plus2 | 887 | retinoid-inducible serine carboxypeptidase isoform 1 | 167 | 1.62E-28 | 62.70% | 3 | F:peptidase activity; P:protein metabolic process; P:catabolic process | IPR001563 (PANTHER); G3DSA:3.40.50.1820 (GENE3D), PTHR11802:SF3 (PANTHER), SignalP-NN(euk) (SIGNALP), tmhmm (TMHMM), SSF53474 (SUPERFAMILY) | Yes |
| 100 | lib_dib_Filter15_IDBA_contig_106952_minus2 | 6 | oocyte zinc finger protein 6-like | 108 | 1.06E-24 | 61.95% | 3 | F:binding; F:nucleic acid binding; C:intracellular | IPR007087 (PFAM); IPR013087 (G3DSA:3.30.160.GENE3D); IPR015880 (SMART); IPR022755 (PFAM); PTHR11389 (PANTHER), PF13465 (PFAM), SSF57667 (SUPERFAMILY) | |
| 101 | lib_dib_Filter15_IDBA_contig_106958_plus2 | 49 | carbohydrate sulfotransferase 8 | 283 | 1.35E-07 | 52.70% | 4 | F:transferase activity; C:cell; P:biosynthetic process; P:carbohydrate metabolic process | IPR005331 (PFAM); IPR018011 (PANTHER); PTHR12137:SF6 (PANTHER) | |
| 102 | lib_dib_Filter15_IDBA_contig_106977_minus2 | 8 | zinc finger protein | 108 | 4.54E-21 | 59.90% | 3 | F:binding; F:nucleic acid binding; C:intracellular | IPR007087 (PROSITE); IPR013087 (G3DSA:3.30.160.GENE3D); IPR015880 (SMART); PTHR24402 (PANTHER), PF13465 (PFAM), SSF57667 (SUPERFAMILY) | |
| 103 | lib_dib_Filter15_IDBA_contig_106990_minus1 | 13 | alpha-mannosidase 2x | 108 | 4.15E-21 | 72.35% | 7 | P:organelle organization; F:hydrolase activity; F:binding; P:carbohydrate metabolic process; P:multicellular organismal development; C:cell; C:Golgi apparatus | IPR000602 (PFAM); IPR011330 (SUPERFAMILY); IPR027291 (G3DSA:3.20.110.GENE3D); PTHR11607 (PANTHER), PTHR11607:SF4 (PANTHER) | |
| 104 | lib_dib_Filter15_IDBA_contig_107215_minus2 | 7 | von willebrand factor type egf and pentraxin domain-containing protein 1 | 108 | 1.22E-18 | 55.45% | 1 | F:chromatin binding | IPR000436 (PFAM); IPR011641 (PFAM); G3DSA:2.10.70.10 (GENE3D), PTHR19325 (PANTHER) | |
| 105 | lib_dib_Filter15_IDBA_contig_107402_minus3 | 8 | low quality protein: titin | 107 | 8.93E-13 | 56.20% | 13 | P:anatomical structure morphogenesis; P:multicellular organismal development; P:biological_process; F:cytoskeletal protein binding; F:structural molecule activity; C:cytoskeleton; P:cell differentiation; P:cytoskeleton organization; C:cytoplasm; P:embryo development; F:protein kinase activity; F:nucleotide binding; P:cellular protein modification process | IPR003598 (SMART); IPR003599 (SMART); IPR007110 (PROFILE); IPR013098 (PFAM); IPR013783 (G3DSA:2.60.40.GENE3D); IPR015726 (PTHR22964:PANTHER); IPR020675 (PANTHER); SSF48726 (SUPERFAMILY) | |
| 106 | lib_dib_Filter15_IDBA_contig_107436_minus3 | 10 | trypsin | 107 | 2.91E-21 | 61.85% | 3 | F:peptidase activity; P:protein metabolic process; P:catabolic process | IPR001254 (PFAM); IPR009003 (SUPERFAMILY); IPR018114 (PROSITE); G3DSA:2.40.10.10 (GENE3D), PTHR24256 (PANTHER) | |
| 107 | lib_dib_Filter15_IDBA_contig_107510_plus1 | 5 | carbohydrate sulfotransferase 11 | 108 | 2.71E-18 | 59.00% | 14 | P:biological_process; P:anatomical structure morphogenesis; P:embryo development; P:regulation of biological process; P:cell proliferation; P:growth; P:signal transduction; P:cell differentiation; P:multicellular organismal development; P:cell death; F:transferase activity; P:biosynthetic process; P:carbohydrate metabolic process; C:cell | IPR005331 (PFAM); IPR018011 (PANTHER) | |
| 108 | lib_dib_Filter15_IDBA_contig_10768_plus2 | 1279 | matrix metalloproteinase-16-like | 636 | 2.46E-43 | 56.90% | 6 | F:peptidase activity; P:multicellular organismal development; P:anatomical structure morphogenesis; P:growth; P:cell death; P:cellular component organization | IPR000585 (G3DSA:2.110.10.GENE3D); IPR018486 (PROSITE); IPR018487 (PFAM); PTHR10201 (PANTHER) | |
| 109 | lib_dib_Filter15_IDBA_contig_107892_plus2 | 14 | arylacetamide deacetylase-like 4-like | 107 | 3.99E-09 | 56.45% | 2 | P:metabolic process; F:hydrolase activity | IPR013094 (PFAM); G3DSA:3.40.50.1820 (GENE3D), PTHR23024 (PANTHER), SSF53474 (SUPERFAMILY) | |

| | | | | | | | | | |
|---|---|---|---|---|---|---|---|---|---|
| 110 | lib_dib_Filter15_IDBA_contig_107895_minus1 | 17 | nardilysin isoform 1 | 143 | 8.40E-27 | 66.90% | 14 | P:biological_process; C:cytosol; P:cellular component organization; P:cell differentiation; P:multicellular organismal development; C:mitochondrion; P:regulation of biological process; P:protein metabolic process; P:catabolic process; C:cell; F:peptidase activity; F:protein binding; P:cell proliferation; F:binding | IPR011237 (G3DSA:3.30.830.GENE3D); IPR011249 (SUPERFAMILY); PTHR11851 (PANTHER), PTHR11851:SF65 (PANTHER) |
| 111 | lib_dib_Filter15_IDBA_contig_107896_minus2 | 12 | nardilysin isoform 1 | 107 | 2.21E-29 | 65.05% | 2 | F:catalytic activity; F:binding | IPR011237 (G3DSA:3.30.830.GENE3D); IPR011249 (SUPERFAMILY); PTHR11851 (PANTHER), PTHR11851:SF65 (PANTHER) |
| 112 | lib_dib_Filter15_IDBA_contig_107954_minus3 | 8 | ---NA--- | 107 | | | 0 | - | IPR008160 (PFAM); PTHR24023 (PANTHER) |
| 113 | lib_dib_Filter15_IDBA_contig_10831_plus3 | 1428 | collagen alpha-3 chain | 1393 | 0 | 52.55% | 5 | P:cell differentiation; P:multicellular organismal development; C:extracellular region; F:protein binding; F:calcium ion binding | IPR000152 (PROSITE); IPR000742 (PFAM); IPR001881 (SMART); IPR002035 (PFAM); IPR013032 (PROSITE); IPR018097 (PROSITE); PR00453 (PRINTS), G3DSA:2.10.25.10 (GENE3D), G3DSA:3.40.50.410 (GENE3D), PTHR22992 (PANTHER), SSF53300 (SUPERFAMILY) |
| 114 | lib_dib_Filter15_IDBA_contig_108321_minus3 | 10 | cadherin egf lag seven-pass g-type receptor 2-like | 106 | 1.07E-39 | 67.95% | 8 | P:anatomical structure morphogenesis; P:embryo development; F:receptor activity; P:multicellular organismal development; P:signal transduction; P:biological_process; C:plasma membrane; F:calcium ion binding | IPR002126 (PRINTS); IPR015919 (SUPERFAMILY); IPR020894 (PROSITE); PTHR24026 (PANTHER), PTHR24026:SF3 (PANTHER) |
| 115 | lib_dib_Filter15_IDBA_contig_108406_plus2 | 384 | superoxide dismutase | 434 | 5.00E-74 | 62.45% | 3 | F:catalytic activity; P:metabolic process; F:binding | IPR001189 (PRINTS); IPR019831 (PFAM); IPR019832 (PFAM); IPR019833 (PROSITE); G3DSA:1.10.287.990 (GENE3D), PTHR11404:SF9 (PANTHER) |
| 116 | lib_dib_Filter15_IDBA_contig_108407_plus3 | 384 | superoxide dismutase | 428 | 3.59E-74 | 62.40% | 3 | F:catalytic activity; P:metabolic process; F:binding | IPR001189 (PRINTS); IPR019831 (PFAM); IPR019832 (PFAM); IPR019833 (PROSITE); G3DSA:1.10.287.990 (GENE3D), PTHR11404:SF9 (PANTHER), SignalP-NN(euk) (SIGNALP) |
| 117 | lib_dib_Filter15_IDBA_contig_108792_plus1 | 6 | zinc finger protein 271-like | 107 | 5.91E-38 | 71.15% | 5 | P:regulation of biological process; ; F:nucleic acid binding; C:nucleus; F:binding | IPR007087 (PROSITE); IPR013087 (G3DSA:3.30.160.GENE3D); IPR015880 (SMART); PTHR24384 (PANTHER), PF13465 (PFAM), SSF57667 (SUPERFAMILY) |
| 118 | lib_dib_Filter15_IDBA_contig_108819_plus1 | 7 | oocyte zinc finger protein 6-partial | 107 | 1.65E-37 | 69.10% | 3 | F:binding; F:nucleic acid binding; C:intracellular | IPR007087 (PROSITE); IPR013087 (G3DSA:3.30.160.GENE3D); IPR015880 (SMART); PTHR24402 (PANTHER), PF13465 (PFAM), SSF57667 (SUPERFAMILY) |
| 119 | lib_dib_Filter15_IDBA_contig_108910_minus2 | 15 | low quality protein: protocadherin-16-like | 106 | 5.97E-30 | 61.10% | 3 | F:calcium ion binding; P:biological_process; C:plasma membrane | IPR002126 (PRINTS); IPR015919 (SUPERFAMILY); IPR020894 (PROSITE); PTHR24027 (PANTHER) |
| 120 | lib_dib_Filter15_IDBA_contig_108963_plus3 | 8 | spermatogenesis-associated protein 6-like | 106 | 3.41E-29 | 65.40% | 7 | F:molecular_function; P:biological_process; C:cellular_component; P:cell differentiation; P:multicellular organismal development; C:extracellular region; P:spermatogenesis | PTHR16435 (PANTHER), PTHR16435:SF0 (PANTHER) |

| | | | | | | | | | |
|---|---|---|---|---|---|---|---|---|---|
| 121 | lib_dib_Filter15_IDBA_contig_109023_minus2 | 15 | matrix metalloproteinase-9 | 106 | 1.19E-15 | 53.90% | 14 | P:anatomical structure morphogenesis; P:multicellular organismal development; P:response to endogenous stimulus; F:protein binding; P:biological_process; P:response to external stimulus; P:regulation of biological process; P:cell communication; P:response to abiotic stimulus; F:peptidase activity; P:response to stress; P:metabolic process; C:extracellular region; P:reproduction | IPR000562 (G3DSA:2.10.10.GENE3D); IPR013806 (SUPERFAMILY); PR00013 (PRINTS), PTHR22918 (PANTHER), PTHR22918:SF0 (PANTHER) |
| 122 | lib_dib_Filter15_IDBA_contig_109167_minus1 | 1372 | chitinase 1 | 182 | 4.30E-23 | 62.85% | 9 | P:biological_process; F:binding; P:response to external stimulus; P:response to stress; C:extracellular region; F:hydrolase activity; C:cytoplasm; P:carbohydrate metabolic process; P:catabolic process | IPR001223 (PFAM); IPR013781 (G3DSA:3.20.20.GENE3D); IPR017853 (SUPERFAMILY); PTHR11177 (PANTHER), PTHR11177:SF37 (PANTHER) |
| 123 | lib_dib_Filter15_IDBA_contig_109299_plus3 | 11 | collagen triple helix repeat protein | 106 | 7.60E-05 | 65.38% | 1 | C:collagen | IPR008160 (PFAM); PTHR24023 (PANTHER), PTHR24023:SF164 (PANTHER) |
| 124 | lib_dib_Filter15_IDBA_contig_109525_minus1 | 6 | multiple epidermal growth factor-like domains protein 6-like | 106 | 4.52E-10 | 55.25% | 1 | F:calcium ion binding | G3DSA:2.170.300.10 (GENE3D), PTHR24035 (PANTHER), PTHR24035:SF4 (PANTHER) |
| 125 | lib_dib_Filter15_IDBA_contig_109642_minus3 | 5 | chordin | 105 | 5.66E-25 | 55.35% | 6 | P:regulation of biological process; P:multicellular organismal development; P:signal transduction; F:protein binding; P:cell differentiation; P:embryo development | IPR001007 (PFAM); G3DSA:2.10.70.10 (GENE3D), PTHR11339 (PANTHER), PTHR11339:SF39 (PANTHER), SSF57603 (SUPERFAMILY) |
| 126 | lib_dib_Filter15_IDBA_contig_109838_minus3 | 10 | matrix metallopeptidase 9 (gelatinase 92kda 92kda type iv collagenase) | 105 | 3.94E-09 | 50.15% | 51 | F:carbohydrate binding; F:metalloendopeptidase activity; F:hydrolase activity; F:zinc ion binding; F:metallopeptidase activity; C:extracellular matrix; F:peptidase activity; P:proteolysis; F:metal ion binding; P:metabolic process; C:membrane; P:anatomical structure regression; F:protein complex binding; P:response to lipopolysaccharide; P:response to mechanical stimulus; P:response to hypoxia; P:response to retinoic acid; P:response to hyperoxia; P:transformation of host cell by virus; P:cellular response to interleukin-1; P:response to tumor necrosis factor; P:cell growth; P:ossification; P:skeletal system development; C:protein complex; P:response to estradiol stimulus; C:extracellular space; P:response to radiation; P:cellular response to low-density lipoprotein particle stimulus; P:extracellular matrix organization; P:response to oxidative stress; P:positive regulation of keratinocyte migration; F:collagen binding; P:cellular response to cell-matrix adhesion; P:tissue remodeling; F:protein binding; C:proteinaceous extracellular matrix; | IPR000562 (G3DSA:2.10.10.GENE3D); IPR013806 (SUPERFAMILY); PTHR22918 (PANTHER), PTHR22918:SF0 (PANTHER) |

| | | | | | | | | | |
|---|---|---|---|---|---|---|---|---|---|
| 127 | lib_dib_Filter15_IDBA_contig_109859_plus1 | 10 | er degradation-enhancing alpha-mannosidase-like 2 | 106 | 5.92E-47 | 84.75% | 9 | P:signal transduction; P:response to stress; P:response to biotic stimulus; F:hydrolase activity; F:calcium ion binding; P:multicellular organismal development; P:protein metabolic process; P:catabolic process; C:cell | IPR001382 (G3DSA:1.50.10.GENE3D); PTHR11742:SF10 (PANTHER) | |
| 128 | lib_dib_Filter15_IDBA_contig_109888_minus2 | 8 | ankyrin repeat protein | 105 | 9.58E-16 | 58.85% | 1 | F:protein binding | IPR002110 (SMART); IPR020683 (G3DSA:1.25.40.GENE3D); PTHR24198 (PANTHER), SignalP-NN(euk) (SIGNALP) | |
| 129 | lib_dib_Filter15_IDBA_contig_109898_minus2 | 12 | retinol dehydrogenase 13-like | 105 | 3.36E-21 | 79.90% | 3 | F:catalytic activity; P:metabolic process; F:nucleotide binding | IPR002198 (PFAM); IPR016040 (G3DSA:3.40.50.GENE3D); PTHR24320 (PANTHER), PTHR24320:SF0 (PANTHER), tmhmm (TMHMM), SSF51735 (SUPERFAMILY) | |
| 130 | lib_dib_Filter15_IDBA_contig_110020_minus2 | 15 | heat shock protein partial | 105 | 4.85E-53 | 96.50% | 2 | F:nucleotide binding; P:response to stress | IPR013126 (PFAM); IPR018181 (PROSITE); G3DSA:3.30.420.40 (GENE3D), G3DSA:3.90.640.10 (GENE3D), PTHR19375 (PANTHER), SSF53067 (SUPERFAMILY) | |
| 131 | lib_dib_Filter15_IDBA_contig_110063_minus3 | 28 | cub domain protein | 181 | 5.21E-15 | 61.35% | 3 | F:molecular_function; P:biological_process; C:cellular_component | IPR000859 (G3DSA:2.60.120.GENE3D); PTHR10127 (PANTHER) | |
| 132 | lib_dib_Filter15_IDBA_contig_110095_plus3 | 12 | multiple egf-like-domains 10 | 105 | 8.18E-14 | 58.65% | 7 | P:cell proliferation; P:transport; P:cellular component organization; P:cell recognition; P:biological_process; C:plasma membrane; P:cell differentiation | G3DSA:2.170.300.10 (GENE3D), PTHR24035 (PANTHER), PTHR24035:SF4 (PANTHER) | |
| 133 | lib_dib_Filter15_IDBA_contig_110473_plus2 | 10 | gonadotropin alpha subunit | 105 | 6.46E-67 | 80.50% | 2 | C:extracellular region; F:receptor binding | IPR000476 (PRINTS); G3DSA:2.10.90.10 (GENE3D), PTHR11509:SF0 (PANTHER), SSF57501 (SUPERFAMILY) | |
| 134 | lib_dib_Filter15_IDBA_contig_110508_minus1 | 6 | von willebrand factor type egf and pentraxin domain-containing protein 1 | 105 | 3.89E-11 | 49.40% | 7 | F:carbohydrate binding; F:hydrolase activity; F:peptidase inhibitor activity; C:extracellular region; P:negative regulation of peptidase activity; P:cell adhesion; C:membrane | IPR000436 (PFAM); G3DSA:2.10.70.10 (GENE3D), PTHR19325 (PANTHER) | |
| 135 | lib_dib_Filter15_IDBA_contig_110513_plus2 | 12 | hhip-like protein 1 | 105 | 1.61E-46 | 76.45% | 3 | F:catalytic activity; P:carbohydrate metabolic process; F:binding | IPR011042 (G3DSA:2.120.10.GENE3D); IPR012938 (PFAM); PTHR19328 (PANTHER), PTHR19328:SF13 (PANTHER) | |
| 136 | lib_dib_Filter15_IDBA_contig_110531_minus1 | 8 | low-density lipoprotein receptor-related protein 2 | 105 | 7.86E-24 | 63.00% | 11 | P:regulation of biological process; P:growth; F:transporter activity; P:biological_process; C:cytoplasmic membrane-bounded vesicle; P:transport; P:anatomical structure morphogenesis; P:multicellular organismal development; F:protein binding; C:plasma membrane; C:Golgi apparatus | IPR002172 (G3DSA:4.10.400.GENE3D); IPR023415 (PROSITE); PR00261 (PRINTS), G3DSA:4.10.1220.10 (GENE3D), PTHR10529 (PANTHER) | |
| 137 | lib_dib_Filter15_IDBA_contig_110540_plus3 | 17 | scavenger receptor cysteine-rich type 1 protein m160 isoform 2 | 104 | 5.50E-13 | 52.90% | 2 | F:receptor activity; C:cell | IPR001190 (PRINTS); IPR017448 (SMART); G3DSA:3.10.250.10 (GENE3D), PTHR19331 (PANTHER) | |
| 138 | lib_dib_Filter15_IDBA_contig_110651_minus1 | 6413 | cubilin- partial | 429 | 1.93E-53 | 52.00% | 4 | F:calcium ion binding; F:transferase activity; F:hydrolase activity; F:transmembrane receptor protein tyrosine kinase activity | IPR000859 (G3DSA:2.60.120.GENE3D); PTHR10127 (PANTHER), SignalP-NN(euk) (SIGNALP), tmhmm (TMHMM) | Yes |
| 139 | lib_dib_Filter15_IDBA_contig_110652_minus1 | 7176 | cubilin- partial | 475 | 6.40E-53 | 51.90% | 4 | F:calcium ion binding; F:transferase activity; F:hydrolase activity; F:transmembrane receptor protein tyrosine kinase activity | IPR000859 (G3DSA:2.60.120.GENE3D); PTHR10127 (PANTHER), SignalP-NN(euk) (SIGNALP), tmhmm (TMHMM) | Yes |

| | | | | | | | | | | |
|---|---|---|---|---|---|---|---|---|---|---|
| 140 | lib_dib_Filter15_IDBA_contig_110653_minus3 | 720 | low quality protein: cubilin | 104 | 6.74E-17 | 55.10% | 10 | P:transport; P:biosynthetic process; P:lipid metabolic process; C:organelle; C:intracellular; C:cell; F:protein binding; F:catalytic activity; C:plasma membrane; C:cytoplasm | IPR000859 (G3DSA:2.60.120.GENE3D); PTHR10127 (PANTHER) | |
| 141 | lib_dib_Filter15_IDBA_contig_110738_minus3 | 172 | ---NA--- | 211 | | | 0 | - | no IPS match | |
| 142 | lib_dib_Filter15_IDBA_contig_110892_minus3 | 37 | leucine-rich repeat neuronal protein 2 | 139 | 5.55E-14 | 55.65% | 2 | P:biological_process; F:protein binding | IPR000372 (SMART); IPR001611 (PROFILE); IPR003591 (SMART); G3DSA:3.80.10.10 (GENE3D), PTHR24367 (PANTHER), PF13855 (PFAM), SSF52058 (SUPERFAMILY) | |
| 143 | lib_dib_Filter15_IDBA_contig_1109_plus1 | 1351 | epididymal secretory protein e1 precursor | 334 | 3.50E-36 | 66.05% | 0 | - | IPR003172 (G3DSA:2.60.40.GENE3D); IPR014756 (SUPERFAMILY); PTHR11306 (PANTHER) | |
| 144 | lib_dib_Filter15_IDBA_contig_11097_minus2 | 2546 | ankyrin repeat protein | 617 | 6.09E-41 | 51.10% | 1 | F:protein binding | IPR002110 (SMART); IPR020683 (G3DSA:1.25.40.GENE3D); PTHR24188 (PANTHER), PF13637 (PFAM), tmhmm (TMHMM) | |
| 145 | lib_dib_Filter15_IDBA_contig_111063_plus1 | 7 | cystatin precursor | 105 | 3.17E-61 | 87.00% | 3 | C:extracellular region; F:enzyme regulator activity; P:biological_process | IPR000010 (PFAM); IPR018073 (PROSITE); IPR027214 (PANTHER); G3DSA:3.10.450.10 (GENE3D), PTHR11413:SF23 (PANTHER), SignalP-NN(euk) (SIGNALP), SSF54403 (SUPERFAMILY) | Yes |
| 146 | lib_dib_Filter15_IDBA_contig_111255_minus1 | 37 | protease-associated domain-containing protein 1 | 169 | 1.72E-34 | 68.35% | 5 | P:proteolysis; F:peptidase activity; C:extracellular region; F:molecular_function; P:biological_process | IPR003137 (PFAM); G3DSA:3.50.30.30 (GENE3D), PTHR22702 (PANTHER) | |
| 147 | lib_dib_Filter15_IDBA_contig_111256_minus2 | 16 | protease-associated domain-containing protein 1 | 104 | 1.18E-34 | 65.00% | 3 | C:extracellular region; F:molecular_function; P:biological_process | IPR003137 (PFAM); G3DSA:3.50.30.30 (GENE3D), PTHR22702 (PANTHER), SSF52025 (SUPERFAMILY) | |
| 148 | lib_dib_Filter15_IDBA_contig_111313_plus3 | 4 | mitogen-activated protein kinase kinase kinase 3 | 104 | 4.42E-28 | 67.85% | 6 | C:cytosol; P:cellular protein modification process; F:protein kinase activity; P:signal transduction; F:protein binding; F:nucleotide binding | IPR000719 (PFAM); IPR002290 (SMART); IPR008271 (PROSITE); IPR011009 (SUPERFAMILY); G3DSA:1.10.510.10 (GENE3D), PTHR24361 (PANTHER) | |
| 149 | lib_dib_Filter15_IDBA_contig_111353_plus2 | 8 | chordin | 104 | 5.61E-16 | 53.25% | 7 | P:regulation of biological process; P:multicellular organismal development; P:signal transduction; P:embryo development; F:protein binding; P:cell differentiation; P:anatomical structure morphogenesis | IPR001007 (PFAM); PTHR11339 (PANTHER), PTHR11339:SF39 (PANTHER), SSF57603 (SUPERFAMILY) | |
| 150 | lib_dib_Filter15_IDBA_contig_11137_minus1 | 350 | alpha- -mannosyl-glycoprotein 4-beta-n-acetylglucosaminyltransferase c | 634 | 1.29E-102 | 59.05% | 3 | F:transferase activity; P:carbohydrate metabolic process; C:cell | IPR006759 (PANTHER); tmhmm (TMHMM) | |
| 151 | lib_dib_Filter15_IDBA_contig_111460_minus2 | 12 | zinc finger protein | 104 | 6.34E-20 | 63.80% | 3 | F:binding; F:nucleic acid binding; C:intracellular | IPR007087 (PROSITE); IPR013087 (G3DSA:3.30.160.GENE3D); IPR015880 (SMART); PTHR11389 (PANTHER), PF13465 (PFAM), SSF57667 (SUPERFAMILY) | |
| 152 | lib_dib_Filter15_IDBA_contig_111536_minus1 | 11 | decapentaplegic | 104 | 6.61E-10 | 53.45% | 1 | F:receptor binding | IPR001839 (PFAM); IPR015615 (PANTHER); G3DSA:2.10.90.10 (GENE3D), PTHR11848:SF50 (PANTHER) | |
| 153 | lib_dib_Filter15_IDBA_contig_111563_plus3 | 17 | thrombospondin type-1 domain-containing protein 7a | 104 | 1.07E-20 | 66.70% | 3 | P:anatomical structure morphogenesis; P:multicellular organismal development; P:biological_process | IPR000884 (PROFILE); PTHR11311 (PANTHER), PTHR11311:SF2 (PANTHER), SignalP-NN(euk) (SIGNALP) | Yes |
| 154 | lib_dib_Filter15_IDBA_contig_111793_plus1 | 7 | microfibril-associated glycoprotein 4-like | 104 | 1.07E-23 | 64.15% | 3 | F:receptor binding; P:signal transduction; C:extracellular space | IPR002181 (PFAM); IPR014716 (G3DSA:3.90.215.GENE3D); PTHR19143 (PANTHER) | |

| | | | | | | | | | |
|---|---|---|---|---|---|---|---|---|---|
| 155 | lib_dib_Filter15_IDBA_contig_111841_plus1 | 8 | zinc finger protein 271 (zinc finger protein 7) (zinc finger protein znfphex133) (epstein-barr virus-induced zinc finger protein) (znf-eb) (ct-zfp48) (zinc finger protein | 104 | 2.76E-21 | 59.40% | 3 | F:binding; F:nucleic acid binding; C:intracellular | IPR007087 (PROSITE); IPR013087 (G3DSA:3.30.160.GENE3D); IPR015880 (SMART); PTHR24375 (PANTHER), PTHR24375:SF0 (PANTHER), PF13465 (PFAM), SSF57667 (SUPERFAMILY) |
| 156 | lib_dib_Filter15_IDBA_contig_111889_minus1 | 10 | zinc finger protein 135-like | 104 | 8.52E-20 | 73.65% | 3 | F:binding; F:nucleic acid binding; C:intracellular | IPR007087 (PROSITE); IPR013087 (G3DSA:3.30.160.GENE3D); IPR015880 (SMART); PTHR24384 (PANTHER), PF13465 (PFAM), PF13913 (PFAM), SSF57667 (SUPERFAMILY) |
| 157 | lib_dib_Filter15_IDBA_contig_111897_minus2 | 8 | cre-ced-1 protein | 104 | 4.97E-09 | 54.35% | 13 | P:cell death; P:transport; P:cellular component organization; C:cytoplasmic membrane-bounded vesicle; P:response to stress; P:response to biotic stimulus; P:cytoskeleton organization; P:biological_process; P:cell recognition; C:plasma membrane; C:cell; F:protein binding; P:organelle organization | PTHR24035 (PANTHER), PTHR24035:SF3 (PANTHER) |
| 158 | lib_dib_Filter15_IDBA_contig_11190_minus2 | 68 | alpha-galactosidase alpha-n-acetylgalactosaminidase | 124 | 2.21E-44 | 71.00% | 2 | F:hydrolase activity; P:carbohydrate metabolic process | IPR002241 (PRINTS); IPR013785 (G3DSA:3.20.20.GENE3D); IPR017853 (SUPERFAMILY); PTHR11452 (PANTHER) |
| 159 | lib_dib_Filter15_IDBA_contig_11191_minus1 | 356 | alpha-galactosidase alpha-n-acetylgalactosaminidase | 180 | 2.64E-41 | 61.90% | 1 | F:hydrolase activity | IPR013780 (G3DSA:2.60.40.GENE3D); IPR013785 (G3DSA:3.20.20.GENE3D); IPR017853 (SUPERFAMILY); PTHR11452 (PANTHER), SignalP-NN(euk) (SIGNALP), SSF51011 (SUPERFAMILY) |
| 160 | lib_dib_Filter15_IDBA_contig_11200_plus1 | 656 | golgi reassembly-stacking protein 2 isoform 2 | 669 | 3.92E-73 | 73.35% | 1 | F:protein binding | IPR001478 (SUPERFAMILY); IPR007583 (PANTHER); IPR024958 (PFAM); tmhmm (TMHMM) |
| 161 | lib_dib_Filter15_IDBA_contig_112156_minus2 | 44 | von willebrand factor a domain-containing protein 8-like | 255 | 4.36E-100 | 82.30% | 8 | F:nucleic acid binding; C:mitochondrion; F:hydrolase activity; P:nucleobase-containing compound metabolic process; P:catabolic process; F:nucleotide binding; C:extracellular region; F:protein binding | IPR002035 (SMART); PTHR21610 (PANTHER), PTHR21610:SF2 (PANTHER), PF13519 (PFAM), SSF53300 (SUPERFAMILY) |
| 162 | lib_dib_Filter15_IDBA_contig_112172_minus1 | 10 | laminin b2 | 104 | 8.22E-36 | 74.35% | 4 | P:anatomical structure morphogenesis; P:embryo development; P:multicellular organismal development; C:proteinaceous extracellular matrix | IPR002049 (PFAM); G3DSA:2.10.25.10 (GENE3D), PTHR10574 (PANTHER), SSF57196 (SUPERFAMILY) |
| 163 | lib_dib_Filter15_IDBA_contig_112178_minus3 | 17 | protein | 103 | 1.24E-31 | 70.20% | 20 | P:cell differentiation; P:multicellular organismal development; P:regulation of biological process; ; P:cell proliferation; P:biological_process; P:anatomical structure morphogenesis; P:embryo development; C:nucleoplasm; P:signal transduction; P:protein metabolic process; P:cellular component organization; C:Golgi apparatus; C:plasma membrane; F:protein binding; F:DNA binding; P:response to stress; P:reproduction; C:endoplasmic reticulum; F:calcium ion binding | IPR000152 (PROSITE); IPR000742 (PFAM); IPR001881 (SMART); IPR013032 (PROSITE); IPR018097 (PROSITE); PR00010 (PRINTS), G3DSA:2.10.25.10 (GENE3D), PTHR24838 (PANTHER), PTHR24838:SF112 (PANTHER), SSF57196 (SUPERFAMILY) |
| 164 | lib_dib_Filter15_IDBA_contig_112205_minus3 | 18 | tripartite motif-containing protein 2 | 161 | 6.46E-93 | 87.10% | 7 | C:cytoplasm; P:cellular protein modification process; F:catalytic activity; F:cytoskeletal protein binding; P:regulation of biological process; P:cell death; F:binding | IPR001258 (PFAM); IPR011042 (G3DSA:2.120.10.GENE3D); IPR013017 (PROFILE); PTHR24103 (PANTHER), PTHR24103:SF2 (PANTHER), SSF63829 (SUPERFAMILY) |
| 165 | lib_dib_Filter15_IDBA_contig_112251_plus2 | 10 | alpha beta hydrolase fold protein | 103 | 1.03E-19 | 66.50% | 1 | F:hydrolase activity | G3DSA:3.40.50.1820 (GENE3D), PTHR10992 (PANTHER), PTHR10992:SF98 (PANTHER), PF12697 (PFAM), SSF53474 (SUPERFAMILY) |

| | | | | | | | | |
|---|---|---|---|---|---|---|---|---|
| 166 | lib_dib_Filter15_IDBA_contig_112301_minus3 | 6 | deleted in malignant brain tumors 1 partial | 103 | 1.17E-11 | 77.25% | 2 | F:receptor activity; C:cell | IPR001190 (PRINTS); IPR017448 (SUPERFAMILY); G3DSA:3.10.250.10 (GENE3D), PTHR19331 (PANTHER), PTHR19331:SF118 (PANTHER) |
| 167 | lib_dib_Filter15_IDBA_contig_112373_minus3 | 13 | neuroserpin | 103 | 7.52E-12 | 60.75% | 3 | P:metabolic process; P:regulation of biological process; F:enzyme regulator activity | IPR000215 (PANTHER); IPR023796 (PFAM); G3DSA:2.30.39.10 (GENE3D) |
| 168 | lib_dib_Filter15_IDBA_contig_112398_minus3 | 19 | intermediate filament protein | 123 | 5.90E-25 | 79.30% | 3 | C:cytoskeleton; C:protein complex; F:structural molecule activity | IPR001664 (PANTHER); IPR016044 (PFAM); IPR018039 (PROSITE); G3DSA:1.20.5.170 (GENE3D) |
| 169 | lib_dib_Filter15_IDBA_contig_11241_plus1 | 1052 | mitogen-activated protein kinase kinase kinase 3 | 312 | 1.54E-56 | 61.80% | 11 | P:signal transduction; P:cellular protein modification process; F:protein binding; P:response to stress; C:cytosol; P:response to external stimulus; P:response to abiotic stimulus; F:protein kinase activity; P:regulation of biological process; ; F:nucleotide binding | IPR000719 (PFAM); IPR002290 (SMART); IPR008271 (PROSITE); IPR011009 (SUPERFAMILY); IPR017441 (PROSITE); IPR020635 (SMART); G3DSA:1.10.510.10 (GENE3D), G3DSA:3.30.200.20 (GENE3D), PTHR24361 (PANTHER) |
| 170 | lib_dib_Filter15_IDBA_contig_112655_minus1 | 9 | von willebrand factor type egf and pentraxin domain-containing protein partial | 103 | 5.13E-22 | 58.85% | 2 | F:binding; F:transferase activity | IPR000436 (PFAM); G3DSA:2.10.70.10 (GENE3D), PTHR19325 (PANTHER) |
| 171 | lib_dib_Filter15_IDBA_contig_112671_minus3 | 15 | variable lymphocyte receptor | 103 | 1.58E-14 | 54.05% | 1 | F:protein binding | IPR000372 (PFAM); IPR001611 (PROFILE); IPR003591 (SMART); G3DSA:3.80.10.10 (GENE3D), PTHR24367 (PANTHER), PF13855 (PFAM), SSF52058 (SUPERFAMILY) |
| 172 | lib_dib_Filter15_IDBA_contig_113021_plus3 | 5 | neutral alkaline nonlysosomal ceramidase family protein | 102 | 4.94E-34 | 69.25% | 2 | F:hydrolase activity; F:ceramidase activity | IPR006823 (PANTHER) |
| 173 | lib_dib_Filter15_IDBA_contig_113066_minus1 | 8 | variable lymphocyte receptor a | 103 | 1.03E-10 | 59.85% | 1 | F:receptor activity | IPR003591 (SMART); PR00019 (PRINTS), G3DSA:3.80.10.10 (GENE3D), PTHR24365 (PANTHER), PTHR24365:SF45 (PANTHER), PF13855 (PFAM), SSF52058 (SUPERFAMILY) |
| 174 | lib_dib_Filter15_IDBA_contig_113238_minus1 | 14 | a disintegrin and metalloproteinase with thrombospondin motifs 7-like | 103 | 1.94E-39 | 74.95% | 5 | F:peptidase activity; C:proteinaceous extracellular matrix; P:protein metabolic process; P:catabolic process; F:binding | IPR013273 (PRINTS); PTHR13723 (PANTHER), PTHR13723:SF30 (PANTHER) |
| 175 | lib_dib_Filter15_IDBA_contig_113239_plus2 | 6 | cell adhesion molecule with homology to l1cam | 102 | 5.20E-13 | 52.45% | 14 | C:nucleus; P:cell cycle; P:biological_process; F:hydrolase activity; P:anatomical structure morphogenesis; P:cellular component organization; P:cell differentiation; P:multicellular organismal development; P:organelle organization; P:signal transduction; C:organelle; C:intracellular; P:response to stress; F:protein binding | IPR003598 (SMART); IPR003599 (SMART); IPR007110 (PROFILE); IPR013783 (G3DSA:2.60.40.GENE3D); PTHR10489 (PANTHER), PF13895 (PFAM), SSF48726 (SUPERFAMILY) |
| 176 | lib_dib_Filter15_IDBA_contig_113291_minus2 | 10 | cadherin egf lag seven-pass g-type receptor 3 | 102 | 2.15E-18 | 62.20% | 8 | P:regulation of biological process; P:growth; P:multicellular organismal development; F:protein binding; P:biological_process; P:signal transduction; F:calcium ion binding; C:cell | IPR002126 (PRINTS); IPR015919 (SUPERFAMILY); PTHR24027 (PANTHER) |
| 177 | lib_dib_Filter15_IDBA_contig_11332_plus1 | 1008 | p21-activated protein kinase-interacting protein 1-like | 739 | 8.21E-92 | 63.45% | 1 | F:protein binding | IPR001680 (PFAM); IPR015943 (G3DSA:2.130.10.GENE3D); IPR017986 (PROFILE); IPR019775 (PROSITE); PTHR22847 (PANTHER), PTHR22847:SF49 (PANTHER), tmhmm (TMHMM) |
| 178 | lib_dib_Filter15_IDBA_contig_113335_minus3 | 4 | zinc finger protein 271-like | 102 | 5.52E-22 | 62.60% | 7 | F:DNA binding; P:regulation of biological process; ; P:biological_process; F:protein binding; C:nucleus; F:binding | IPR007087 (PROSITE); IPR013087 (G3DSA:3.30.160.GENE3D); IPR015880 (SMART); PTHR24402 (PANTHER), PF13465 (PFAM), SSF57667 (SUPERFAMILY) |

| | | | | | | | | | | |
|---|---|---|---|---|---|---|---|---|---|---|
| 179 | lib_dib_Filter15_IDBA_contig_113436_minus2 | 7 | threonine synthase-like 1 | 102 | 2.26E-33 | 69.30% | 1 | F:catalytic activity | IPR001926 (SUPERFAMILY); G3DSA:3.40.50.1100 (GENE3D), PTHR10314 (PANTHER), PTHR10314:SF45 (PANTHER) | |
| 180 | lib_dib_Filter15_IDBA_contig_113449_plus2 | 10 | matrix metalloproteinase 9 | 102 | 1.59E-08 | 44.50% | 19 | F:carbohydrate binding; P:extracellular matrix organization; P:positive regulation of apoptotic process; P:positive regulation of keratinocyte migration; F:metalloendopeptidase activity; P:skeletal system development; F:zinc ion binding; P:embryo implantation; C:proteinaceous extracellular matrix; C:extracellular matrix; P:proteolysis; P:collagen catabolic process; C:extracellular space; P:cell adhesion; F:hydrolase activity; F:metallopeptidase activity; F:peptidase activity; F:metal ion binding; P:metabolic process | IPR000562 (G3DSA:2.10.10.GENE3D); IPR013806 (SUPERFAMILY); PTHR22918 (PANTHER), PTHR22918:SF0 (PANTHER) | |
| 181 | lib_dib_Filter15_IDBA_contig_113464_plus1 | 23 | hypothetical protein EAI_13871 | 103 | 6.51E-06 | 81.00% | 0 | - | no IPS match | |
| 182 | lib_dib_Filter15_IDBA_contig_113464_plus2 | 23 | ---NA--- | 102 | | | 0 | - | no IPS match | |
| 183 | lib_dib_Filter15_IDBA_contig_113591_plus3 | 11 | alkaline phosphatase | 102 | 2.16E-21 | 66.90% | 2 | P:metabolic process; F:hydrolase activity | IPR001952 (PFAM); IPR017849 (G3DSA:3.40.720.GENE3D); IPR017850 (SUPERFAMILY); PTHR11596 (PANTHER), PTHR11596:SF11 (PANTHER) | |
| 184 | lib_dib_Filter15_IDBA_contig_113595_minus1 | 8 | alcohol dehydrogenase | 102 | 2.57E-20 | 72.55% | 2 | F:catalytic activity; P:metabolic process | IPR001395 (PANTHER); IPR018170 (PROSITE); IPR023210 (G3DSA:3.20.20.GENE3D); PTHR11732:SF34 (PANTHER) | |
| 185 | lib_dib_Filter15_IDBA_contig_113621_plus3 | 6 | leucine-rich repeat and immunoglobulin-like domain-containing nogo receptor-interacting protein 2-like | 102 | 3.76E-14 | 58.45% | 4 | F:molecular_function; C:integral to membrane; C:membrane; P:biological_process | IPR003591 (SMART); G3DSA:3.80.10.10 (GENE3D), PTHR24373 (PANTHER), PF13855 (PFAM), SSF52058 (SUPERFAMILY) | |
| 186 | lib_dib_Filter15_IDBA_contig_113627_minus2 | 8 | zinc finger protein 2 | 102 | 1.41E-17 | 70.95% | 3 | F:binding; F:nucleic acid binding; C:intracellular | IPR007087 (PROSITE); IPR013087 (G3DSA:3.30.160.GENE3D); IPR015880 (SMART); PTHR24382 (PANTHER), PTHR24382:SF0 (PANTHER), PF13465 (PFAM), SSF57667 (SUPERFAMILY) | |
| 187 | lib_dib_Filter15_IDBA_contig_11383_minus2 | 315 | alpha-galactosidase a | 171 | 1.90E-81 | 77.80% | 3 | F:hydrolase activity; P:carbohydrate metabolic process; F:binding | IPR000111 (PFAM); IPR002241 (PRINTS); IPR013785 (G3DSA:3.20.20.GENE3D); IPR017853 (SUPERFAMILY); PTHR11452 (PANTHER), SignalP-NN(euk) (SIGNALP), tmhmm (TMHMM) | Yes |
| 188 | lib_dib_Filter15_IDBA_contig_11384_minus2 | 188 | alpha-n-acetylgalactosaminidase | 137 | 1.07E-53 | 72.95% | 2 | F:hydrolase activity; P:carbohydrate metabolic process | IPR002241 (PRINTS); IPR013785 (G3DSA:3.20.20.GENE3D); IPR017853 (SUPERFAMILY); PTHR11452 (PANTHER) | |
| 189 | lib_dib_Filter15_IDBA_contig_113860_plus3 | 7 | egf-like domain-containing protein | 102 | 1.46E-27 | 66.45% | 2 | F:protein binding; F:calcium ion binding | IPR000152 (PROSITE); IPR000742 (SMART); IPR001881 (PFAM); IPR018097 (PROSITE); IPR026823 (PFAM); G3DSA:2.10.25.10 (GENE3D), PTHR24046 (PANTHER), SSF57196 (SUPERFAMILY) | |
| 190 | lib_dib_Filter15_IDBA_contig_113871_plus1 | 21 | paramyosin | 102 | 4.33E-17 | 80.80% | 4 | C:cytoplasm; C:cytoskeleton; C:protein complex; F:motor activity | IPR002928 (PFAM); PTHR13140 (PANTHER), PTHR13140:SF11 (PANTHER) | |
| 191 | lib_dib_Filter15_IDBA_contig_113906_plus1 | 14 | zinc finger protein partial | 102 | 7.89E-27 | 66.45% | 3 | F:binding; F:nucleic acid binding; C:intracellular | IPR007087 (PROSITE); IPR013087 (G3DSA:3.30.160.GENE3D); IPR015880 (SMART); PTHR24402 (PANTHER), PF13465 (PFAM), SSF57667 (SUPERFAMILY) | |

| | | | | | | | | | | |
|---|---|---|---|---|---|---|---|---|---|---|
| 192 | lib_dib_Filter15_IDBA_contig_113924_minus1 | 6 | arylsulfatase b | 102 | 8.08E-31 | 69.10% | 13 | P:multicellular organismal development; C:lysosome; P:transport; C:mitochondrion; P:response to endogenous stimulus; P:response to external stimulus; C:endoplasmic reticulum; P:response to abiotic stimulus; F:hydrolase activity; P:catabolic process; P:biological_process; P:organelle organization; C:Golgi apparatus | IPR000917 (PFAM); IPR017849 (G3DSA:3.40.720.GENE3D); IPR017850 (SUPERFAMILY); PTHR10342 (PANTHER), PTHR10342:SF19 (PANTHER) | |
| 193 | lib_dib_Filter15_IDBA_contig_114002_plus1 | 8 | low quality protein: hemicentin-1 | 102 | 2.06E-20 | 60.90% | 7 | P:reproduction; P:multicellular organismal development; P:anatomical structure morphogenesis; P:behavior; P:biological_process; C:proteinaceous extracellular matrix; F:protein binding | IPR003598 (SMART); IPR007110 (PROFILE); IPR013098 (PFAM); IPR013783 (G3DSA:2.60.40.GENE3D); PTHR10489 (PANTHER), SSF48726 (SUPERFAMILY) | |
| 194 | lib_dib_Filter15_IDBA_contig_114120_plus1 | 107 | deleted in malignant brain tumors 1 | 230 | 2.34E-09 | 42.70% | 2 | F:receptor activity; C:cell | IPR001190 (PFAM); IPR017448 (SUPERFAMILY); G3DSA:3.10.250.10 (GENE3D), PTHR19331 (PANTHER), PTHR19331:SF118 (PANTHER) | |
| 195 | lib_dib_Filter15_IDBA_contig_114259_minus1 | 12 | chloride channel calcium activated 2-like | 102 | 1.92E-19 | 73.05% | 0 | - | IPR013642 (PFAM); PTHR10579 (PANTHER), SignalP-NN(euk) (SIGNALP), tmhmm (TMHMM) | |
| 196 | lib_dib_Filter15_IDBA_contig_114714_plus3 | 6149 | tryptase gamma | 325 | 5.74E-63 | 51.75% | 3 | F:peptidase activity; P:protein metabolic process; P:catabolic process | IPR001254 (PFAM); IPR001314 (PRINTS); IPR009003 (SUPERFAMILY); IPR018114 (PROSITE); G3DSA:2.40.10.10 (GENE3D), PTHR24256 (PANTHER), SignalP-NN(euk) (SIGNALP), tmhmm (TMHMM) | Yes |
| 197 | lib_dib_Filter15_IDBA_contig_114873_minus3 | 15 | retinoid-inducible serine carboxypeptidase | 134 | 2.45E-20 | 64.50% | 3 | F:peptidase activity; P:protein metabolic process; P:catabolic process | IPR001563 (PANTHER); G3DSA:3.40.50.1820 (GENE3D), PTHR11802:SF3 (PANTHER), SSF53474 (SUPERFAMILY) | |
| 198 | lib_dib_Filter15_IDBA_contig_114954_plus3 | 8 | protocadherin- partial | 101 | 4.16E-17 | 59.25% | 5 | P:metabolic process; P:regulation of biological process; F:calcium ion binding; P:biological_process; C:plasma membrane | IPR002126 (G3DSA:2.60.40.GENE3D); IPR015919 (SUPERFAMILY); IPR020894 (PROSITE); PTHR24028 (PANTHER), PTHR24028:SF40 (PANTHER), tmhmm (TMHMM) | |
| 199 | lib_dib_Filter15_IDBA_contig_114971_minus3 | 9 | low-density lipoprotein receptor-related protein 4 | 101 | 4.26E-49 | 91.80% | 24 | P:anatomical structure morphogenesis; P:embryo development; F:peptidase activity; C:cytoplasmic membrane-bounded vesicle; P:multicellular organismal development; P:signal transduction; P:cellular component organization; P:cell differentiation; P:growth; F:receptor binding; P:regulation of biological process; F:calcium ion binding; C:extracellular region; F:receptor activity; C:cell; P:biological_process; P:reproduction; P:cellular protein modification process; P:transport; P:protein metabolic process; P:catabolic process; F:protein binding; C:plasma membrane; P:metabolic process | IPR000033 (PFAM); IPR011042 (G3DSA:2.120.10.GENE3D); PTHR10529 (PANTHER), SSF63825 (SUPERFAMILY) | |

| | | | | | | | | | |
|---|---|---|---|---|---|---|---|---|---|
| 200 | lib_dib_Filter15_IDBA_contig_115004_plus3 | 17 | eph receptor tyrosine | 101 | 2.03E-57 | 90.65% | 30 | P:signal transduction; P:multicellular organismal development; P:regulation of biological process; P:cytoskeleton organization; P:anatomical structure morphogenesis; C:endoplasmic reticulum; P:cell death; P:cellular component organization; P:response to external stimulus; P:response to stress; F:protein kinase activity; F:receptor activity; F:hydrolase activity; P:cell-cell signaling; P:cell differentiation; C:cell; P:cell communication; C:plasma membrane; C:cytoplasm; ; F:protein binding; F:nucleotide binding; P:cellular protein modification process; P:response to abiotic stimulus; C:endosome; P:behavior; P:biological_process; P:cell recognition; P:response to biotic stimulus; C:extracellular | IPR000719 (PROFILE); IPR001245 (PFAM); IPR011009 (SUPERFAMILY); IPR020635 (SMART); G3DSA:1.10.510.10 (GENE3D), G3DSA:3.30.200.20 (GENE3D), PTHR24416 (PANTHER), PTHR24416:SF122 (PANTHER) |
| 201 | lib_dib_Filter15_IDBA_contig_115189_plus2 | 8 | matrix metalloproteinase-9-like | 101 | 9.28E-09 | 53.40% | 35 | C:membrane; C:cytoplasm; C:integral to membrane; P:transport; F:transporter activity; P:bone trabecula formation; P:cellular response to amino acid stimulus; F:metalloendopeptidase activity; P:intramembranous ossification; P:response to hypoxia; P:blood vessel maturation; F:zinc ion binding; P:embryo implantation; C:proteinaceous extracellular matrix; C:sarcomere; P:proteolysis; P:face morphogenesis; P:collagen catabolic process; C:plasma membrane; C:extracellular space; C:nucleus; P:extracellular matrix disassembly; P:extracellular matrix organization; F:serine-type endopeptidase activity; P:angiogenesis; C:extracellular region; P:positive regulation of apoptotic process; P:positive regulation of keratinocyte migration; F:hydrolase activity; P:skeletal system development; F:metallopeptidase activity; C:extracellular matrix; F:peptidase activity; F:metal ion | IPR000562 (G3DSA:2.10.10.GENE3D); IPR013806 (SUPERFAMILY); PTHR22918 (PANTHER), PTHR22918:SF0 (PANTHER) |
| 202 | lib_dib_Filter15_IDBA_contig_115248_plus2 | 14 | riken cdna 3110057o12 gene | 101 | 3.48E-34 | 77.15% | 4 | F:molecular_function; P:biological_process; C:cellular_component; C:extracellular region | IPR019149 (PFAM); G3DSA:3.40.50.1820 (GENE3D), PTHR13617 (PANTHER), PTHR13617:SF5 (PANTHER) |
| 203 | lib_dib_Filter15_IDBA_contig_115398_plus2 | 10 | oocyte zinc finger protein 6-like | 101 | 3.12E-32 | 69.05% | 3 | F:binding; F:nucleic acid binding; C:intracellular | IPR007087 (PROSITE); IPR013087 (G3DSA:3.30.160.GENE3D); IPR015880 (SMART); PTHR24382 (PANTHER), PTHR24382:SF0 (PANTHER), PF13465 (PFAM), SSF57667 (SUPERFAMILY) |
| 204 | lib_dib_Filter15_IDBA_contig_11541_plus2 | 4282 | aminopeptidase puromycin sensitive | 1017 | 0 | 76.70% | 8 | P:response to stress; P:cellular protein modification process; F:peptidase activity; P:biological_process; C:cytosol; P:protein metabolic process; P:catabolic process; F:binding | IPR001930 (PANTHER); IPR014782 (PRINTS); IPR015568 (PTHR11533:PANTHER); IPR024571 (PFAM); G3DSA:1.10.390.10 (GENE3D), SignalP-NN(euk) (SIGNALP), SSF55486 (SUPERFAMILY), SSF63737 (SUPERFAMILY) |

| | | | | | | | | | | |
|---|---|---|---|---|---|---|---|---|---|---|
| 205 | lib_dib_Filter15_IDBA_contig_115471_minus3 | 18 | cysteine-rich motor neuron 1 | 117 | 1.41E-37 | 71.50% | 7 | P:multicellular organismal development; F:enzyme regulator activity; C:cell; P:biological_process; P:embryo development; P:anatomical structure morphogenesis; F:protein binding | IPR001007 (PFAM); G3DSA:2.10.70.10 (GENE3D), PTHR11339 (PANTHER), PTHR11339:SF40 (PANTHER), SSF57603 (SUPERFAMILY) | |
| 206 | lib_dib_Filter15_IDBA_contig_115669_plus2 | 340 | pro-neuregulin- membrane-bound isoform | 250 | 1.62E-08 | 43.60% | 10 | P:regulation of biological process; P:protein metabolic process; P:multicellular organismal development; C:cell; F:receptor binding; P:metabolic process; P:cellular component organization; P:signal transduction; P:biological_process; P:cell differentiation | IPR000742 (PROFILE); IPR003598 (SMART); IPR003599 (SMART); IPR007110 (PROFILE); IPR013032 (PROSITE); IPR013098 (PFAM); IPR013783 (G3DSA:2.60.40.GENE3D); G3DSA:2.10.25.10 (GENE3D), PTHR10489 (PANTHER), SignalP-NN(euk) (SIGNALP), tmhmm (TMHMM), SSF48726 (SUPERFAMILY), SSF57196 (SUPERFAMILY) | |
| 207 | lib_dib_Filter15_IDBA_contig_11572_plus1 | 1887 | a chain elaborate manifold of short hydrogen bond arrays mediating binding of active site-directed serine protease inhibitors | 210 | 4.19E-26 | 74.35% | 4 | F:protein binding; F:peptidase activity; P:protein metabolic process; P:catabolic process | IPR001254 (PFAM); IPR009003 (SUPERFAMILY); IPR018114 (PROSITE); G3DSA:2.40.10.10 (GENE3D), PTHR24264 (PANTHER) | |
| 208 | lib_dib_Filter15_IDBA_contig_116118_plus1 | 29 | glypican-6- partial | 100 | 4.36E-50 | 80.65% | 5 | C:cell; C:extracellular space; C:plasma membrane; C:proteinaceous extracellular matrix; F:protein binding | IPR001863 (PANTHER); IPR019803 (PROSITE); PTHR10822:SF15 (PANTHER) | |
| 209 | lib_dib_Filter15_IDBA_contig_116265_minus2 | 11 | protein wnt-11 | 100 | 1.32E-15 | 59.00% | 25 | P:regulation of biological process; ; F:receptor binding; F:protein binding; P:anatomical structure morphogenesis; P:embryo development; P:signal transduction; P:multicellular organismal development; C:protein complex; C:extracellular space; P:biological_process; F:DNA binding; F:enzyme regulator activity; C:plasma membrane; C:cytoplasm; P:cytoskeleton organization; C:proteinaceous extracellular matrix; P:cell differentiation; P:cell-cell signaling; P:transport; P:cellular component organization; P:response to stress; P:cell death; P:cell growth; P:metabolic process | IPR005817 (PANTHER); IPR026536 (PTHR12027:PANTHER); SignalP-NN(euk) (SIGNALP) | Yes |
| 210 | lib_dib_Filter15_IDBA_contig_116664_plus2 | 41 | protein | 203 | 1.97E-13 | 41.60% | 9 | F:helicase activity; F:ATP-dependent 3'-5' DNA helicase activity; P:DNA recombination; F:hydrolase activity; P:DNA replication; F:nucleic acid binding; F:ATP binding; F:ATP-dependent helicase activity; P:DNA repair | no IPS match | |
| 211 | lib_dib_Filter15_IDBA_contig_116760_plus2 | 51 | echotoxin b2 | 187 | 4.92E-16 | 55.71% | 6 | P:ion transport; F:transporter activity; C:cell; C:protein complex; P:cellular component organization; P:symbiosis, encompassing mutualism through parasitism | IPR009104 (SUPERFAMILY); IPR015926 (G3DSA:2.60.270.GENE3D) | |
| 212 | lib_dib_Filter15_IDBA_contig_116761_plus3 | 77 | echotoxin a | 266 | 9.65E-21 | 52.29% | 6 | P:ion transport; F:transporter activity; C:cell; C:protein complex; P:cellular component organization; P:symbiosis, encompassing mutualism through parasitism | IPR009104 (PFAM); IPR015926 (G3DSA:2.60.270.GENE3D) | |
| 213 | lib_dib_Filter15_IDBA_contig_11681_plus3 | 647 | serine protease 19 | 108 | 4.13E-13 | 55.00% | 3 | F:peptidase activity; P:protein metabolic process; P:catabolic process | IPR001254 (PFAM); IPR009003 (SUPERFAMILY); G3DSA:2.40.10.10 (GENE3D), PTHR24268 (PANTHER) | |

| | | | | | | | | | | |
|---|---|---|---|---|---|---|---|---|---|---|
| 214 | lib_dib_Filter15_IDBA_contig_116873_minus2 | 304 | galactose soluble 8-like | 295 | 2.93E-41 | 57.50% | 1 | F:carbohydrate binding | IPR001079 (PFAM); IPR008985 (SUPERFAMILY); IPR013320 (G3DSA:2.60.120.GENE3D); PTHR11346 (PANTHER) | |
| 215 | lib_dib_Filter15_IDBA_contig_116874_minus2 | 323 | galectin-8 isoform 1 | 295 | 1.01E-41 | 58.10% | 1 | F:carbohydrate binding | IPR001079 (PFAM); IPR008985 (SUPERFAMILY); IPR013320 (G3DSA:2.60.120.GENE3D); PTHR11346 (PANTHER) | |
| 216 | lib_dib_Filter15_IDBA_contig_117539_plus1 | 53 | upf0556 protein c19orf10 homolog | 160 | 3.03E-26 | 53.75% | 2 | F:molecular_function; P:biological_process | IPR018887 (PFAM); PTHR31230 (PANTHER), SignalP-NN(euk) (SIGNALP), tmhmm (TMHMM) | Yes |
| 217 | lib_dib_Filter15_IDBA_contig_117553_plus1 | 19 | polypeptide n-acetylgalactosaminyltransferase 13 | 171 | 2.17E-92 | 71.00% | 6 | C:Golgi apparatus; P:biosynthetic process; P:cellular protein modification process; P:carbohydrate metabolic process; F:binding; F:transferase activity | IPR001173 (PFAM); G3DSA:3.90.550.10 (GENE3D), PTHR11675 (PANTHER), SSF53448 (SUPERFAMILY) | |
| 218 | lib_dib_Filter15_IDBA_contig_117554_plus1 | 18 | polypeptide n-acetylgalactosaminyltransferase 13 | 171 | 2.17E-92 | 71.00% | 6 | C:Golgi apparatus; P:biosynthetic process; P:cellular protein modification process; P:carbohydrate metabolic process; F:binding; F:transferase activity | IPR001173 (PFAM); G3DSA:3.90.550.10 (GENE3D), PTHR11675 (PANTHER), SSF53448 (SUPERFAMILY) | |
| 219 | lib_dib_Filter15_IDBA_contig_117555_plus1 | 350 | acetylcholine receptor subunit alpha | 296 | 2.43E-22 | 46.95% | 4 | P:biological_process; P:ion transport; C:cell; F:ion channel activity | IPR006201 (PANTHER); IPR006202 (G3DSA:2.70.170.GENE3D); PTHR18945:SF153 (PANTHER), SignalP-NN(euk) (SIGNALP) | Yes |
| 220 | lib_dib_Filter15_IDBA_contig_117671_minus1 | 135 | ---NA--- | 207 | | | 1 | F:protein binding | IPR000742 (PROFILE); IPR013032 (PROSITE); G3DSA:2.10.25.10 (GENE3D), SignalP-NN(euk) (SIGNALP), tmhmm (TMHMM), SSF57196 (SUPERFAMILY) | |
| 221 | lib_dib_Filter15_IDBA_contig_117677_plus2 | 12 | fibrinogen c domain-containing protein 1-like | 108 | 4.94E-14 | 59.45% | 4 | P:biological_process; P:lipid metabolic process; F:binding; P:regulation of biological process | IPR002181 (PFAM); IPR014716 (G3DSA:3.90.215.GENE3D); PTHR19143 (PANTHER) | |
| 222 | lib_dib_Filter15_IDBA_contig_117800_minus1 | 91 | low quality protein: prolow-density lipoprotein receptor-related protein 1 | 209 | 4.50E-22 | 51.45% | 5 | P:biological_process; F:protein binding; C:cell; P:regulation of biological process; P:transport | IPR002172 (G3DSA:4.10.400.GENE3D); IPR023415 (PROSITE); PR00261 (PRINTS), PTHR12106 (PANTHER), PTHR12106:SF7 (PANTHER) | |
| 223 | lib_dib_Filter15_IDBA_contig_117801_minus1 | 94 | sortilin-related receptor | 147 | 1.02E-08 | 62.95% | 5 | C:plasma membrane; F:receptor activity; F:protein binding; P:transport; P:cellular component organization | IPR002172 (G3DSA:4.10.400.GENE3D); IPR023415 (PROSITE); PR00261 (PRINTS), PTHR12106 (PANTHER), PTHR12106:SF7 (PANTHER) | |
| 224 | lib_dib_Filter15_IDBA_contig_11783_plus2 | 3292 | pdz and lim domain protein zasp | 157 | 1.75E-23 | 61.55% | 3 | C:cytoplasm; C:plasma membrane; F:protein binding | IPR001478 (PFAM); G3DSA:2.30.42.10 (GENE3D), PTHR24214 (PANTHER), PTHR24214:SF0 (PANTHER) | |
| 225 | lib_dib_Filter15_IDBA_contig_117846_minus3 | 17 | novel protein x-epilectin precursor | 112 | 8.75E-13 | 52.10% | 5 | P:regulation of biological process; P:response to stress; F:carbohydrate binding; C:extracellular region; P:biological_process | IPR000421 (PFAM); IPR006585 (SMART); IPR008979 (SUPERFAMILY); G3DSA:2.60.120.260 (GENE3D), PTHR19277 (PANTHER), PTHR19277:SF6 (PANTHER) | |
| 226 | lib_dib_Filter15_IDBA_contig_117847_minus1 | 1945 | transmembrane protease serine 3 isoform 2 | 326 | 6.59E-47 | 53.00% | 5 | C:endoplasmic reticulum; C:cell; F:peptidase activity; P:protein metabolic process; P:catabolic process | IPR001254 (PFAM); IPR001314 (PRINTS); IPR009003 (SUPERFAMILY); IPR018114 (PROSITE); G3DSA:2.40.10.10 (GENE3D), PTHR24256 (PANTHER) | |
| 227 | lib_dib_Filter15_IDBA_contig_117896_minus1 | 57 | xanthine dehydrogenase oxidase | 177 | 5.71E-38 | 65.30% | 4 | F:electron carrier activity; F:binding; F:catalytic activity; P:metabolic process | IPR001041 (PFAM); IPR002888 (G3DSA:1.10.150.GENE3D); IPR006058 (PROSITE); IPR012675 (G3DSA:3.10.20.GENE3D); PTHR11908 (PANTHER), PTHR11908:SF5 (PANTHER) | |
| 228 | lib_dib_Filter15_IDBA_contig_117996_minus3 | 203 | metallophosphoesterase domain-containing protein 1 | 289 | 7.44E-73 | 61.10% | 1 | F:hydrolase activity | IPR004843 (PFAM); G3DSA:3.60.21.10 (GENE3D), PTHR12905 (PANTHER), SSF56300 (SUPERFAMILY) | |
| 229 | lib_dib_Filter15_IDBA_contig_118039_minus2 | 839 | creg1 precursor | 300 | 3.71E-64 | 59.40% | 3 | F:nucleotide binding; F:catalytic activity; P:metabolic process | IPR012349 (G3DSA:2.30.110.GENE3D); PTHR13343 (PANTHER), PF13883 (PFAM) | |
| 230 | lib_dib_Filter15_IDBA_contig_11806_plus1 | 228 | arylsulfatase j | 107 | 7.57E-24 | 67.05% | 2 | F:hydrolase activity; P:metabolic process | IPR017850 (SUPERFAMILY); G3DSA:3.30.1120.10 (GENE3D), PTHR10342 (PANTHER), PTHR10342:SF19 (PANTHER) | |

| | | | | | | | | | | |
|---|---|---|---|---|---|---|---|---|---|---|
| 231 | lib_dib_Filter15_IDBA_contig_11850_plus1 | 657 | peptidyl-prolyl cis-trans isomerase fkbp14-like | 258 | 3.61E-82 | 79.10% | 6 | P:protein metabolic process; C:extracellular space; F:catalytic activity; F:calcium ion binding; P:behavior; F:binding | IPR001179 (PFAM); IPR002048 (PROFILE); IPR011992 (G3DSA:1.10.238.GENE3D); IPR018247 (PROSITE); IPR023566 (PANTHER); G3DSA:3.10.50.40 (GENE3D), PTHR10516:SF20 (PANTHER), SignalP-NN(euk) (SIGNALP), tmhmm (TMHMM), SSF47473 (SUPERFAMILY), SSF54534 (SUPERFAMILY) | Yes |
| 232 | lib_dib_Filter15_IDBA_contig_11856_plus1 | 1053 | cathepsin l-like | 135 | 4.72E-41 | 85.85% | 3 | F:peptidase activity; P:protein metabolic process; P:catabolic process | IPR000668 (PFAM); IPR013128 (PANTHER); IPR025660 (PROSITE); IPR025661 (PROSITE); G3DSA:3.90.70.10 (GENE3D), PTHR12411:SF149 (PANTHER), SSF54001 (SUPERFAMILY) | |
| 233 | lib_dib_Filter15_IDBA_contig_11860_plus2 | 2307 | suppressor of tumorigenicity 14 protein homolog | 313 | 1.24E-18 | 42.50% | 4 | F:peptidase activity; P:protein metabolic process; P:catabolic process; F:protein binding | IPR001254 (PFAM); IPR002172 (G3DSA:4.10.400.GENE3D); IPR009003 (SUPERFAMILY); IPR018114 (PROSITE); PR00261 (PRINTS), G3DSA:2.40.10.10 (GENE3D), G3DSA:4.10.1220.10 (GENE3D), PTHR24256 (PANTHER), PTHR24256:SF66 (PANTHER), SignalP-NN(euk) (SIGNALP) | |
| 234 | lib_dib_Filter15_IDBA_contig_118652_plus1 | 65 | aminopeptidase n | 189 | 6.20E-24 | 54.90% | 2 | P:protein metabolic process; P:catabolic process | IPR001930 (PANTHER); G3DSA:1.10.390.10 (GENE3D), SSF55486 (SUPERFAMILY) | |
| 235 | lib_dib_Filter15_IDBA_contig_11884_plus1 | 141 | arylsulfatase i | 127 | 2.28E-37 | 71.35% | 2 | F:hydrolase activity; P:metabolic process | IPR017849 (G3DSA:3.40.720.GENE3D); IPR017850 (SUPERFAMILY); G3DSA:3.30.1120.10 (GENE3D), PTHR10342 (PANTHER), PTHR10342:SF68 (PANTHER) | |
| 236 | lib_dib_Filter15_IDBA_contig_11885_plus1 | 120 | arylsulfatase i | 127 | 3.94E-38 | 71.20% | 2 | F:hydrolase activity; P:metabolic process | IPR017849 (G3DSA:3.40.720.GENE3D); IPR017850 (SUPERFAMILY); G3DSA:3.30.1120.10 (GENE3D), PTHR10342 (PANTHER), PTHR10342:SF68 (PANTHER) | |
| 237 | lib_dib_Filter15_IDBA_contig_118978_plus2 | 19 | protein | 143 | 9.02E-31 | 61.45% | 2 | F:protein binding; F:calcium ion binding | IPR000152 (PROSITE); IPR000742 (PFAM); IPR001881 (PFAM); IPR013032 (PROSITE); IPR018097 (PROSITE); PR00010 (PRINTS), G3DSA:2.10.25.10 (GENE3D), PTHR24838 (PANTHER), PTHR24838:SF112 (PANTHER), SSF57196 (SUPERFAMILY) | |
| 238 | lib_dib_Filter15_IDBA_contig_119066_minus2 | 23 | iq motif and sec7 domain-containing protein 2-like | 178 | 2.42E-19 | 46.09% | 0 | - | SignalP-NN(euk) (SIGNALP), tmhmm (TMHMM) | |
| 239 | lib_dib_Filter15_IDBA_contig_119097_minus1 | 59 | egf-like domain-containing protein | 197 | 3.03E-54 | 60.55% | 2 | F:protein binding; F:calcium ion binding | IPR000152 (PROSITE); IPR000742 (SMART); IPR001881 (PFAM); IPR013032 (PROSITE); IPR018097 (PROSITE); G3DSA:2.10.25.10 (GENE3D), PTHR24838 (PANTHER), PTHR24838:SF171 (PANTHER), SSF57196 (SUPERFAMILY) | |
| 240 | lib_dib_Filter15_IDBA_contig_11912_minus1 | 3444 | midline isoform q | 901 | 6.84E-161 | 49.30% | 4 | P:axonogenesis; P:multicellular organism reproduction; C:extracellular space; C:plasma membrane | IPR000782 (G3DSA:2.30.180.GENE3D); PTHR10900 (PANTHER) | |
| 241 | lib_dib_Filter15_IDBA_contig_11936_plus1 | 251 | arylsulfatase i | 151 | 3.29E-50 | 69.80% | 11 | P:multicellular organismal development; P:response to endogenous stimulus; P:response to abiotic stimulus; P:response to external stimulus; C:Golgi apparatus; C:lysosome; C:endoplasmic reticulum; P:biological_process; F:hydrolase activity; C:mitochondrion; P:catabolic process | IPR000917 (PFAM); IPR017849 (G3DSA:3.40.720.GENE3D); IPR017850 (SUPERFAMILY); PTHR10342 (PANTHER), PTHR10342:SF19 (PANTHER) | |
| 242 | lib_dib_Filter15_IDBA_contig_119521_plus3 | 24 | neurotrypsin | 168 | 2.16E-40 | 59.55% | 3 | F:peptidase activity; F:receptor activity; C:cell | IPR001190 (PRINTS); IPR017448 (SMART); G3DSA:3.10.250.10 (GENE3D), PTHR19331 (PANTHER) | |
| 243 | lib_dib_Filter15_IDBA_contig_119535_minus1 | 415 | selenoprotein p precursor | 158 | 2.66E-11 | 56.70% | 1 | F:binding | IPR007671 (PFAM); PTHR10105 (PANTHER), SignalP-NN(euk) (SIGNALP), tmhmm (TMHMM) | |

| | | | | | | | | | | |
|---|---|---|---|---|---|---|---|---|---|---|
| 244 | lib_dib_Filter15_IDBA_contig_119622_plus3 | 24 | fibrinogen-related protein | 160 | 8.47E-37 | 58.00% | 5 | F:receptor binding; C:extracellular space; P:signal transduction; P:response to stilbenoid; C:fibrinogen complex | IPR002181 (PFAM); IPR014715 (G3DSA:4.10.530.GENE3D); IPR014716 (G3DSA:3.90.215.GENE3D); PTHR19143 (PANTHER) | |
| 245 | lib_dib_Filter15_IDBA_contig_119623_plus2 | 78 | fibrinogen-related protein | 321 | 3.91E-53 | 56.20% | 6 | F:receptor binding; C:extracellular space; P:signal transduction; F:carbohydrate binding; C:collagen; P:complement activation, lectin pathway | IPR002181 (PFAM); IPR014715 (G3DSA:4.10.530.GENE3D); IPR014716 (G3DSA:3.90.215.GENE3D); PTHR19143 (PANTHER), SignalP-NN(euk) (SIGNALP), tmhmm (TMHMM) | |
| 246 | lib_dib_Filter15_IDBA_contig_119624_plus1 | 19 | fibrinogen-like protein 1-like | 133 | 7.20E-30 | 57.40% | 5 | F:receptor binding; C:extracellular space; P:signal transduction; P:response to stilbenoid; C:fibrinogen complex | IPR002181 (PFAM); IPR014715 (G3DSA:4.10.530.GENE3D); IPR014716 (G3DSA:3.90.215.GENE3D); PTHR19143 (PANTHER) | |
| 247 | lib_dib_Filter15_IDBA_contig_11985_plus2 | 322 | arylsulfatase j | 230 | 8.16E-66 | 76.70% | 2 | F:hydrolase activity; P:metabolic process | IPR000917 (PFAM); IPR017849 (G3DSA:3.40.720.GENE3D); IPR017850 (SUPERFAMILY); IPR024607 (PROSITE); PTHR10342 (PANTHER), PTHR10342:SF19 (PANTHER) | |
| 248 | lib_dib_Filter15_IDBA_contig_120068_plus3 | 152 | low density lipoprotein- class -containing protein | 241 | 4.68E-17 | 45.25% | 1 | F:protein binding | IPR002172 (G3DSA:4.10.400.GENE3D); IPR023415 (PROSITE); PTHR10529 (PANTHER), SignalP-NN(euk) (SIGNALP), tmhmm (TMHMM) | Yes |
| 249 | lib_dib_Filter15_IDBA_contig_120080_plus3 | 31 | proclotting enzyme | 148 | 1.08E-13 | 62.15% | 7 | C:extracellular region; P:response to external stimulus; P:regulation of biological process; P:response to stress; F:peptidase activity; P:protein metabolic process; P:catabolic process | IPR001254 (PFAM); IPR009003 (SUPERFAMILY); IPR018114 (PROSITE); G3DSA:2.40.10.10 (GENE3D), PTHR24256 (PANTHER) | |
| 250 | lib_dib_Filter15_IDBA_contig_120144_plus2 | 8 | dna methylase n-4 n-6 domain protein | 118 | 1.40E-05 | 64.63% | 0 | - | no IPS match | |
| 251 | lib_dib_Filter15_IDBA_contig_120144_plus3 | 8 | ---NA--- | 117 | | | 0 | - | no IPS match | |
| 252 | lib_dib_Filter15_IDBA_contig_120187_minus2 | 46 | subfamily s1a unassigned peptidase (s01 family) | 181 | 1.54E-32 | 54.75% | 3 | F:peptidase activity; P:protein metabolic process; P:catabolic process | IPR001254 (PFAM); IPR009003 (SUPERFAMILY); IPR018114 (PROSITE); G3DSA:2.40.10.10 (GENE3D), PTHR24272 (PANTHER), tmhmm (TMHMM) | |
| 253 | lib_dib_Filter15_IDBA_contig_12021_minus2 | 25333 | myosin heavy chain | 191 | 2.04E-46 | 84.35% | 7 | C:cytoskeleton; C:protein complex; F:actin binding; F:nucleotide binding; C:cytoplasm; F:motor activity; F:protein binding | IPR002928 (PFAM); PTHR13140 (PANTHER), PTHR13140:SF106 (PANTHER) | |
| 254 | lib_dib_Filter15_IDBA_contig_12022_minus1 | 43 | myosin heavy chain | 224 | 9.59E-28 | 92.45% | 7 | C:cytoskeleton; C:protein complex; F:nucleotide binding; F:actin binding; F:motor activity; F:catalytic activity; P:metabolic process | IPR002928 (PFAM); PTHR13140 (PANTHER), PTHR13140:SF106 (PANTHER), tmhmm (TMHMM) | |
| 255 | lib_dib_Filter15_IDBA_contig_12026_minus2 | 50024 | glyceraldehyde-3-phosphate dehydrogenase | 134 | 2.39E-63 | 90.75% | 17 | P:cell death; C:cytosol; F:nucleotide binding; F:cytoskeletal protein binding; P:metabolic process; C:cytoskeleton; P:regulation of biological process; P:cytoskeleton organization; F:catalytic activity; P:carbohydrate metabolic process; P:generation of precursor metabolites and energy; P:catabolic process; P:biosynthetic process; C:cytoplasm; C:lipid particle; C:cell; C:nucleus | IPR016040 (G3DSA:3.40.50.GENE3D); IPR020828 (PFAM); IPR020829 (PFAM); IPR020830 (PROSITE); IPR020831 (PRINTS); G3DSA:3.30.360.10 (GENE3D), SSF51735 (SUPERFAMILY) | |
| 256 | lib_dib_Filter15_IDBA_contig_120422_plus1 | 22 | lactase-like protein | 124 | 1.82E-28 | 64.20% | 2 | F:hydrolase activity; P:carbohydrate metabolic process | IPR001360 (PANTHER); IPR013781 (G3DSA:3.20.20.GENE3D); IPR017853 (SUPERFAMILY) | |

| | | | | | | | | | |
|---|---|---|---|---|---|---|---|---|---|
| 257 | lib_dib_Filter15_IDBA_contig_120500_plus1 | 222 | thioredoxin-like protein 1-like | 263 | 1.77E-118 | 76.45% | 5 | P:metabolic process; F:electron carrier activity; F:catalytic activity; P:cellular homeostasis; P:regulation of biological process | IPR005746 (PRINTS); IPR008979 (SUPERFAMILY); IPR010400 (G3DSA:2.60.120.GENE3D); IPR012336 (G3DSA:3.40.30.GENE3D); IPR013766 (PFAM); IPR017937 (PROSITE); PTHR10438:SF19 (PANTHER) |
| 258 | lib_dib_Filter15_IDBA_contig_120633_minus1 | 12 | trefoil factor | 116 | 1.29E-20 | 55.45% | 2 | P:biological_process; P:regulation of biological process | IPR000519 (G3DSA:4.10.110.GENE3D); IPR017994 (PRINTS); PTHR13826 (PANTHER), PTHR13826:SF1 (PANTHER) |
| 259 | lib_dib_Filter15_IDBA_contig_12070_plus3 | 374 | protein | 379 | 1.70E-40 | 41.95% | 2 | P:peptide cross-linking; C:cytoplasm | no IPS match |
| 260 | lib_dib_Filter15_IDBA_contig_121381_minus2 | 19 | ankyrin repeat and death domain-containing protein 1a | 117 | 4.33E-29 | 67.15% | 1 | F:protein binding | IPR002110 (PRINTS); IPR020683 (G3DSA:1.25.40.GENE3D); PTHR24125 (PANTHER) |
| 261 | lib_dib_Filter15_IDBA_contig_121703_plus3 | 29 | ---NA--- | 167 | | | 0 | - | IPR008160 (PFAM); PTHR24023 (PANTHER), PTHR24023:SF164 (PANTHER) |
| 262 | lib_dib_Filter15_IDBA_contig_12174_minus2 | 398 | estradiol 17-beta-dehydrogenase 12 | 286 | 2.76E-106 | 72.05% | 7 | F:catalytic activity; F:protein binding; P:biosynthetic process; P:lipid metabolic process; P:biological_process; C:endoplasmic reticulum; F:nucleotide binding | IPR002198 (PRINTS); IPR002347 (PRINTS); IPR016040 (G3DSA:3.40.50.GENE3D); PTHR24316 (PANTHER), PTHR24316:SF68 (PANTHER), SignalP-NN(euk) (SIGNALP), tmhmm (TMHMM), SSF51735 (SUPERFAMILY) |
| 263 | lib_dib_Filter15_IDBA_contig_121890_plus1 | 17 | ---NA--- | 111 | | | 0 | - | no IPS match |
| 264 | lib_dib_Filter15_IDBA_contig_121890_plus2 | 17 | ---NA--- | 110 | | | 0 | - | no IPS match |
| 265 | lib_dib_Filter15_IDBA_contig_122211_plus1 | 14 | slit2 protein | 114 | 2.80E-06 | 48.80% | 79 | P:axon guidance; P:morphogenesis of a branching structure; F:calcium ion binding; P:negative regulation of axon extension; P:endocardial progenitor cell migration to the midline involved in heart field formation; P:axonal defasciculation; C:extracellular region; P:negative regulation of retinal ganglion cell axon guidance; P:negative regulation of protein phosphorylation; P:negative regulation of cell proliferation; P:chemotaxis; P:cell differentiation; P:retinal ganglion cell axon guidance; P:negative regulation of leukocyte chemotaxis; C:plasma membrane; P:negative regulation of mononuclear cell migration; P:negative regulation of catalytic activity; F:chemorepellent activity; P:cell migration involved in sprouting angiogenesis; P:nervous system development; P:negative regulation of smooth muscle cell chemotaxis; P:corticospinal neuron axon guidance through spinal cord; P:telencephalon cell migration; P:mammary duct terminal end bud growth; P:in utero embryonic development; P:negative regulation of cellular response to growth | no IPS match |
| 266 | lib_dib_Filter15_IDBA_contig_12222_plus1 | 250 | myomesin-1 isoform 1 | 216 | 1.45E-13 | 46.75% | 3 | C:organelle; C:intracellular; F:protein binding | IPR003961 (PFAM); IPR013783 (G3DSA:2.60.40.GENE3D); PR00014 (PRINTS), PTHR10489 (PANTHER) |
| 267 | lib_dib_Filter15_IDBA_contig_122221_plus2 | 28 | calbindin-32 | 123 | 8.29E-20 | 77.35% | 1 | F:calcium ion binding | IPR002048 (PROFILE); IPR018247 (PROSITE); PTHR19972 (PANTHER) |

| | | | | | | | | | |
|---|---|---|---|---|---|---|---|---|---|
| 268 | lib_dib_Filter15_IDBA_contig_122330_minus1 | 11 | achain crystal structure of engineered northeast structural genomics consortium target | 126 | 5.64E-16 | 54.20% | 6 | P:primary metabolic process; C:cytoplasm; C:organelle; P:metabolic process; C:nucleus; F:protein binding | IPR002110 (SMART); IPR020683 (G3DSA:1.25.40.GENE3D); PTHR24193 (PANTHER), SignalP-NN(euk) (SIGNALP) |
| 269 | lib_dib_Filter15_IDBA_contig_122418_minus2 | 19 | von willebrand factor type egf and pentraxin domain-containing protein 1 | 121 | 1.28E-11 | 52.10% | 1 | F:binding | IPR000436 (PFAM); G3DSA:2.10.70.10 (GENE3D), PTHR19325 (PANTHER) |
| 270 | lib_dib_Filter15_IDBA_contig_12246_minus3 | 3308 | hypothetical protein CAPTEDRAFT_222921 | 183 | 3.72E-55 | 69.25% | 2 | F:transporter activity; P:transport | IPR001747 (PFAM); IPR011030 (G3DSA:1.25.10.GENE3D); IPR015255 (PFAM); IPR015817 (G3DSA:2.20.50.GENE3D); IPR015819 (SUPERFAMILY); PTHR23345 (PANTHER) |
| 271 | lib_dib_Filter15_IDBA_contig_122599_minus1 | 23 | carboxypeptidase a1-like | 165 | 5.42E-14 | 52.95% | 4 | F:peptidase activity; P:protein metabolic process; P:catabolic process; F:binding | IPR000834 (PFAM); IPR003146 (G3DSA:3.30.70.GENE3D); IPR009020 (SUPERFAMILY); G3DSA:3.40.630.10 (GENE3D), PTHR11705 (PANTHER), SSF53187 (SUPERFAMILY) |
| 272 | lib_dib_Filter15_IDBA_contig_12261_minus1 | 11049 | map kinase-interacting serine threonine-protein kinase 1 | 279 | 2.19E-138 | 78.90% | 3 | P:cellular protein modification process; F:nucleotide binding; F:protein kinase activity | IPR000719 (PFAM); IPR002290 (SMART); IPR008271 (PROSITE); IPR011009 (SUPERFAMILY); IPR017441 (PROSITE); IPR020635 (SMART); G3DSA:1.10.510.10 (GENE3D), G3DSA:3.30.200.20 (GENE3D), PTHR24349 (PANTHER), PTHR24349:SF0 (PANTHER) |
| 273 | lib_dib_Filter15_IDBA_contig_12262_plus1 | 55 | paramyosin | 321 | 4.35E-83 | 78.10% | 4 | C:cytoplasm; C:cytoskeleton; C:protein complex; F:motor activity | IPR002928 (PFAM); PTHR13140 (PANTHER), PTHR13140:SF106 (PANTHER) |
| 274 | lib_dib_Filter15_IDBA_contig_12278_minus1 | 8290 | immunoglobulin i-set domain protein | 219 | 1.18E-32 | 54.85% | 5 | P:regulation of biological process; F:kinase activity; C:cytoplasm; P:growth; F:protein binding | IPR003598 (SMART); IPR003599 (SMART); IPR007110 (PROFILE); IPR013098 (PFAM); IPR013783 (G3DSA:2.60.40.GENE3D); PTHR25963 (PANTHER), SSF48726 (SUPERFAMILY) |
| 275 | lib_dib_Filter15_IDBA_contig_12288_minus1 | 9293 | twitchin-like protein | 235 | 1.27E-125 | 83.20% | 3 | F:nucleotide binding; P:cellular protein modification process; F:protein kinase activity | IPR000719 (PFAM); IPR002290 (SMART); IPR008271 (PROSITE); IPR011009 (SUPERFAMILY); IPR020635 (SMART); IPR020675 (PANTHER); G3DSA:1.10.510.10 (GENE3D), G3DSA:3.30.200.20 (GENE3D), PTHR22964:SF3 (PANTHER) |
| 276 | lib_dib_Filter15_IDBA_contig_12310_minus1 | 5750 | isoform c | 126 | 1.34E-46 | 69.65% | 9 | P:multicellular organismal development; P:cellular protein modification process; C:cytoplasm; F:structural molecule activity; P:anatomical structure morphogenesis; P:cell differentiation; P:cytoskeleton organization; F:protein kinase activity; F:protein binding | IPR003599 (SMART); IPR007110 (PROFILE); IPR013098 (PFAM); IPR013783 (G3DSA:2.60.40.GENE3D); SSF48726 (SUPERFAMILY) |
| 277 | lib_dib_Filter15_IDBA_contig_12311_minus1 | 6731 | isoform h | 301 | 8.39E-130 | 64.30% | 2 | F:protein kinase activity; F:protein binding | IPR003961 (PFAM); IPR013098 (PFAM); IPR013783 (G3DSA:2.60.40.GENE3D); PR00014 (PRINTS), SSF48726 (SUPERFAMILY) |
| 278 | lib_dib_Filter15_IDBA_contig_12312_minus1 | 3164 | camk mlck protein kinase | 119 | 1.03E-29 | 59.60% | 7 | C:cytoplasm; P:regulation of biological process; P:multicellular organismal development; P:biological_process; F:kinase activity; P:growth; F:protein binding | IPR003598 (SMART); IPR003599 (SMART); IPR007110 (PROFILE); IPR013098 (PFAM); IPR013783 (G3DSA:2.60.40.GENE3D); PTHR10489 (PANTHER), SSF48726 (SUPERFAMILY) |
| 279 | lib_dib_Filter15_IDBA_contig_12313_minus1 | 5281 | | 226 | 0 | 100% | 1 | F:protein binding | IPR003961 (PFAM); IPR013098 (PFAM); IPR013783 (G3DSA:2.60.40.GENE3D); PR00014 (PRINTS), SSF48726 (SUPERFAMILY) |
| 280 | lib_dib_Filter15_IDBA_contig_123157_minus3 | 20 | fucl1_angja ame: full=fucolectin-1 flags: precursor | 110 | 2.54E-10 | 55.95% | 5 | C:extracellular space; P:regulation of biological process; P:response to stress; F:carbohydrate binding; P:biological_process | IPR000421 (PFAM); IPR008979 (SUPERFAMILY); G3DSA:2.60.120.260 (GENE3D), PTHR19277 (PANTHER), PTHR19277:SF6 (PANTHER), SignalP-NN(euk) (SIGNALP), tmhmm (TMHMM) |

| | | | | | | | | | | |
|---|---|---|---|---|---|---|---|---|---|---|
| 281 | lib_dib_Filter15_IDBA_contig_12316_minus1 | 3294 | | 148 | 0 | 100% | 1 | F:protein binding | IPR003961 (PFAM); IPR013783 (G3DSA:2.60.40.GENE3D); PR00014 (PRINTS) | |
| 282 | lib_dib_Filter15_IDBA_contig_12317_minus1 | 2915 | | 162 | 0 | 100% | 1 | F:protein binding | IPR003961 (PFAM); IPR013783 (G3DSA:2.60.40.GENE3D); PR00014 (PRINTS) | |
| 283 | lib_dib_Filter15_IDBA_contig_123199_plus1 | 111 | protein | 190 | 9.07E-29 | 47.37% | 9 | F:tubulin binding; C:microtubule; F:structural constituent of cell wall; P:translational initiation; F:translation initiation factor activity; F:metal ion binding; P:biological_process; F:zinc ion binding; C:intracellular | no IPS match | |
| 284 | lib_dib_Filter15_IDBA_contig_12322_minus1 | 9590 | low quality protein: twitchin | 531 | 0 | 60.55% | 2 | F:transferase activity; F:protein binding | IPR003599 (SMART); IPR003961 (PFAM); IPR007110 (PROFILE); IPR013098 (PFAM); IPR013783 (G3DSA:2.60.40.GENE3D); PR00014 (PRINTS), SSF48726 (SUPERFAMILY) | |
| 285 | lib_dib_Filter15_IDBA_contig_123335_minus1 | 11 | ---NA--- | 106 | | | 0 | - | no IPS match | |
| 286 | lib_dib_Filter15_IDBA_contig_12340_minus1 | 2378 | | 194 | 0 | 100% | 1 | F:protein binding | IPR003961 (PFAM); IPR013783 (G3DSA:2.60.40.GENE3D); PR00014 (PRINTS) | |
| 287 | lib_dib_Filter15_IDBA_contig_12350_plus3 | 4438 | uba and wwe domain containing 1 | 5041 | 0 | 75.05% | 10 | C:nucleolus; P:cellular protein modification process; P:response to stress; P:DNA metabolic process; P:organelle organization; F:DNA binding; F:catalytic activity; P:catabolic process; F:protein binding; C:cytoplasm | IPR000569 (PFAM); IPR025527 (PFAM); G3DSA:3.30.2160.10 (GENE3D), PTHR11254 (PANTHER), PTHR11254:SF67 (PANTHER), SignalP-NN(euk) (SIGNALP), tmhmm (TMHMM) | Yes |
| 288 | lib_dib_Filter15_IDBA_contig_12353_minus3 | 24708 | VtaA11 | 4954 | 2.33E-07 | 58.00% | 2 | C:outer membrane; P:pathogenesis | tmhmm (TMHMM) | |
| 289 | lib_dib_Filter15_IDBA_contig_12353_plus1 | 24708 | collagen triple helix repeat protein | 4955 | 7.84E-08 | 60.78% | 5 | C:outer membrane; P:pathogenesis; C:cell wall; C:collagen; F:carboxypeptidase activity | IPR008160 (PFAM); PTHR24023 (PANTHER), tmhmm (TMHMM) | |
| 290 | lib_dib_Filter15_IDBA_contig_12353_plus2 | 24708 | multiple banded antigen | 4954 | 8.25E-11 | 43.33% | 2 | P:proteolysis; F:cysteine-type peptidase activity | tmhmm (TMHMM) | |
| 291 | lib_dib_Filter15_IDBA_contig_12358_plus3 | 1858 | | 3982 | 0 | 100% | 1 | F:protein binding | IPR000048 (SMART); IPR003598 (SMART); IPR003599 (SMART); IPR007110 (PROFILE); IPR013098 (PFAM); IPR013783 (G3DSA:2.60.40.GENE3D); PF13895 (PFAM), SSF48726 (SUPERFAMILY) | |
| 292 | lib_dib_Filter15_IDBA_contig_12361_plus1 | 159 | | 427 | 0 | 100% | 1 | F:protein binding | IPR003598 (SMART); IPR003599 (SMART); IPR007110 (PROFILE); IPR013098 (PFAM); IPR013783 (G3DSA:2.60.40.GENE3D); IPR020675 (PANTHER); IPR020682 (PTHR22964:PANTHER); SSF48726 (SUPERFAMILY) | |
| 293 | lib_dib_Filter15_IDBA_contig_12368_minus1 | 2576 | epididymal sperm-binding protein 1-like | 3222 | 1.58E-109 | 41.25% | 2 | P:single fertilization; C:extracellular region | IPR000001 (PROFILE); IPR000562 (G3DSA:2.10.10.GENE3D); IPR003014 (PFAM); IPR003609 (SMART); IPR013806 (SUPERFAMILY); PTHR22918 (PANTHER), PTHR22918:SF0 (PANTHER), tmhmm (TMHMM), SSF57414 (SUPERFAMILY) | |
| 294 | lib_dib_Filter15_IDBA_contig_12370_plus2 | 4977 | ankyrin repeat domain-containing protein 17 | 4041 | 0 | 82.60% | 6 | F:RNA binding; P:biological_process; C:cytoplasm; C:nucleus; P:multicellular organismal development; F:protein binding | IPR002110 (PRINTS); IPR004087 (SMART); IPR004088 (PFAM); IPR020683 (G3DSA:1.25.40.GENE3D); G3DSA:3.30.1370.10 (GENE3D), PTHR24139 (PANTHER), PTHR24139:SF7 (PANTHER), PF13637 (PFAM), tmhmm (TMHMM), SSF54791 (SUPERFAMILY) | |

| | | | | | | | | | |
|---|---|---|---|---|---|---|---|---|---|
| 295 | lib_dib_Filter15_IDBA_contig_123760_plus2 | 15 | ankyrin unc44 | 102 | 1.42E-09 | 56.20% | 9 | F:RNA binding; F:transmembrane transporter activity; C:integral to membrane; P:RNA-dependent DNA replication; P:transmembrane transport; F:RNA-directed DNA polymerase activity; P:proteolysis; F:serine-type endopeptidase activity; F:catalytic activity | no IPS match |
| 296 | lib_dib_Filter15_IDBA_contig_123804_plus3 | 23 | receptor tyrosine-protein kinase erbb-3 | 154 | 2.11E-16 | 51.95% | 19 | F:signal transducer activity; F:protein kinase activity; P:transport; P:regulation of biological process; P:multicellular organismal development; P:signal transduction; F:receptor activity; P:anatomical structure morphogenesis; P:cell differentiation; C:plasma membrane; P:cell death; P:cell communication; P:biological_process; F:protein binding; C:organelle; C:intracellular; P:cellular protein modification process; P:response to external stimulus; P:response to stress | IPR006212 (SMART); IPR009030 (SUPERFAMILY); G3DSA:2.10.220.10 (GENE3D), G3DSA:3.80.20.20 (GENE3D), PTHR24416 (PANTHER), PTHR24416:SF88 (PANTHER), SSF52058 (SUPERFAMILY) |
| 297 | lib_dib_Filter15_IDBA_contig_123805_plus1 | 30 | epidermal growth factor receptor-like | 185 | 2.47E-24 | 54.40% | 2 | F:kinase activity; C:cell | IPR000494 (PFAM); IPR006212 (SMART); IPR009030 (SUPERFAMILY); G3DSA:2.10.220.10 (GENE3D), G3DSA:3.80.20.20 (GENE3D), PTHR24416 (PANTHER), PTHR24416:SF95 (PANTHER), SSF52058 (SUPERFAMILY) |
| 298 | lib_dib_Filter15_IDBA_contig_123806_plus2 | 30 | epidermal growth factor receptor-like | 185 | 4.55E-25 | 55.40% | 4 | P:biological_process; F:binding; F:protein kinase activity; C:cell | IPR000494 (PFAM); IPR006212 (SMART); IPR009030 (SUPERFAMILY); G3DSA:2.10.220.10 (GENE3D), G3DSA:3.80.20.20 (GENE3D), PTHR24416 (PANTHER), PTHR24416:SF95 (PANTHER), SSF52058 (SUPERFAMILY) |
| 299 | lib_dib_Filter15_IDBA_contig_124071_minus1 | 58 | hypothetical protein NCER_101620 | 136 | 1.86E-04 | 52.00% | 0 | - | no IPS match |
| 300 | lib_dib_Filter15_IDBA_contig_12417_plus1 | 4710 | cytoplasmic dynein 1 heavy chain 1 | 2856 | 0 | 85.15% | 17 | F:motor activity; C:cytosol; P:transport; P:cell death; C:cytoskeleton; C:protein complex; P:cell cycle; F:hydrolase activity; P:nucleobase-containing compound metabolic process; P:catabolic process; P:cytoskeleton organization; P:cellular component organization; C:microtubule organizing center; F:nucleotide binding; P:biological_process; F:protein binding; C:Golgi apparatus | IPR003593 (SMART); IPR004273 (PFAM); IPR024317 (PFAM); IPR024743 (PFAM); IPR026983 (PANTHER); G3DSA:3.40.50.300 (GENE3D), PTHR10676:SF28 (PANTHER), PF12775 (PFAM), PF12781 (PFAM), tmhmm (TMHMM), SSF52540 (SUPERFAMILY) |
| 301 | lib_dib_Filter15_IDBA_contig_12422_plus1 | 1146 | ectonucleotide pyrophosphatase phosphodiesterase family member 3 | 2283 | 0 | 55.70% | 9 | P:ion transport; P:nucleobase-containing compound metabolic process; P:catabolic process; C:cell; F:hydrolase activity; P:metabolic process; C:cytoplasm; F:nucleic acid binding; F:binding | IPR001604 (PFAM); IPR002591 (PFAM); IPR017849 (G3DSA:3.40.720.GENE3D); IPR017850 (SUPERFAMILY); IPR020821 (G3DSA:3.40.570.GENE3D); IPR024873 (PANTHER); PTHR10151:SF56 (PANTHER), tmhmm (TMHMM), SSF54060 (SUPERFAMILY) |

| | | | | | | | | | |
|---|---|---|---|---|---|---|---|---|---|
| 302 | lib_dib_Filter15_IDBA_contig_12423_minus1 | 3849 | serine threonine-protein kinase nlk | 3764 | 0 | 89.50% | 20 | P:cellular protein modification process; P:anatomical structure morphogenesis; P:multicellular organismal development; P:signal transduction; ; P:regulation of biological process; P:behavior; P:response to external stimulus; P:response to abiotic stimulus; P:cellular component organization; P:cell differentiation; P:cell-cell signaling; P:growth; C:cytoplasm; F:protein binding; F:protein kinase activity; F:signal transducer activity; F:binding; C:nucleus; F:nucleotide binding | IPR000719 (PFAM); IPR002290 (SMART); IPR003527 (PROSITE); IPR008271 (PROSITE); IPR011009 (SUPERFAMILY); IPR017441 (PROSITE); IPR020635 (SMART); G3DSA:1.10.510.10 (GENE3D), G3DSA:3.30.200.20 (GENE3D), PTHR24055 (PANTHER), PTHR24055:SF8 (PANTHER), tmhmm (TMHMM) |
| 303 | lib_dib_Filter15_IDBA_contig_12424_minus1 | 1826 | cubilin | 2377 | 0 | 57.25% | 8 | P:transport; P:biosynthetic process; P:lipid metabolic process; C:lysosome; C:cell; C:vacuole; F:protein binding; C:plasma membrane | IPR000859 (G3DSA:2.60.120.GENE3D); PTHR10127 (PANTHER), PTHR10127:SF310 (PANTHER) |
| 304 | lib_dib_Filter15_IDBA_contig_124244_minus2 | 18 | antigen ki-67-like | 106 | 1.56E-11 | 48.17% | 0 | - | SignalP-NN(euk) (SIGNALP) |
| 305 | lib_dib_Filter15_IDBA_contig_124259_plus3 | 20 | antigen -like | 101 | 6.33E-12 | 58.25% | 2 | F:receptor activity; C:cell | IPR001190 (PRINTS); IPR017448 (SMART); G3DSA:3.10.250.10 (GENE3D), PTHR19331 (PANTHER), PTHR19331:SF92 (PANTHER) |
| 306 | lib_dib_Filter15_IDBA_contig_12426_plus2 | 1952 | myosin light chain kinase | 2229 | 0 | 52.25% | 8 | F:protein binding; P:regulation of biological process; P:biological_process; C:cytoplasm; F:protein kinase activity; P:response to stress; F:nucleotide binding; P:cellular protein modification process | IPR000719 (PFAM); IPR002290 (SMART); IPR003598 (SMART); IPR003599 (SMART); IPR003961 (PFAM); IPR007110 (PROFILE); IPR008271 (PROSITE); IPR011009 (SUPERFAMILY); IPR013098 (PFAM); IPR013783 (G3DSA:2.60.40.GENE3D); IPR017441 (PROSITE); IPR020635 (SMART); IPR020675 (PANTHER); G3DSA:1.10.510.10 (GENE3D), G3DSA:3.30.200.20 (GENE3D), PTHR22964:SF3 (PANTHER), tmhmm (TMHMM), SSF48726 (SUPERFAMILY) |
| 307 | lib_dib_Filter15_IDBA_contig_12427_plus3 | 138 | myosin light chain smooth muscle | 114 | 5.63E-18 | 58.25% | 5 | F:protein kinase activity; C:cytoplasm; P:embryo development; P:biological_process; F:protein binding | IPR003599 (SMART); IPR007110 (PROFILE); IPR013098 (PFAM); IPR013783 (G3DSA:2.60.40.GENE3D); PTHR10489 (PANTHER), SSF48726 (SUPERFAMILY) |
| 308 | lib_dib_Filter15_IDBA_contig_12458_minus2 | 1647 | roundabout homolog 2-like | 2210 | 2.65E-123 | 59.00% | 12 | P:anatomical structure morphogenesis; P:cellular component organization; P:cell differentiation; P:multicellular organismal development; P:cell recognition; C:cell; P:behavior; P:response to external stimulus; P:regulation of biological process; P:biological_process; F:protein binding; P:response to endogenous stimulus | IPR003598 (SMART); IPR003599 (SMART); IPR003961 (PFAM); IPR007110 (PROFILE); IPR013098 (PFAM); IPR013783 (G3DSA:2.60.40.GENE3D); PTHR10489 (PANTHER), PTHR10489:SF27 (PANTHER), tmhmm (TMHMM), SSF48726 (SUPERFAMILY) |
| 309 | lib_dib_Filter15_IDBA_contig_12469_minus3 | 2055 | protocadherin fat 4 | 3655 | 0 | 45.30% | 3 | F:calcium ion binding; P:biological_process; C:cell | IPR002126 (PRINTS); IPR015919 (SUPERFAMILY); PTHR24027 (PANTHER), tmhmm (TMHMM) |
| 310 | lib_dib_Filter15_IDBA_contig_12476_minus1 | 9773 | ---NA--- | 2410 | | | 0 | - | no IPS match |
| 311 | lib_dib_Filter15_IDBA_contig_12476_plus1 | 9773 | collagen triple helix repeat protein | 2410 | 1.85E-08 | 60.92% | 4 | C:collagen; C:cell wall; C:outer membrane; P:pathogenesis | IPR008160 (PFAM); PTHR24023 (PANTHER) |
| 312 | lib_dib_Filter15_IDBA_contig_12476_plus2 | 9773 | ---NA--- | 2410 | | | 0 | - | no IPS match |

| | | | | | | | | | |
|---|---|---|---|---|---|---|---|---|---|
| 313 | lib_dib_Filter15_IDBA_contig_12480_minus3 | 1396 | laminin subunit alpha-like | 2308 | 0 | 50.60% | 7 | P:multicellular organismal development; P:anatomical structure morphogenesis; F:receptor binding; C:proteinaceous extracellular matrix; C:protein complex; P:regulation of biological process; P:embryo development | IPR001791 (PFAM); IPR002049 (PFAM); IPR008985 (SUPERFAMILY); IPR009254 (PFAM); IPR010307 (PFAM); IPR013032 (PROSITE); IPR013320 (G3DSA:2.60.120.GENE3D); PR00011 (PRINTS), G3DSA:2.10.25.10 (GENE3D), PTHR10574 (PANTHER), tmhmm (TMHMM), SSF57196 (SUPERFAMILY) |
| 314 | lib_dib_Filter15_IDBA_contig_12481_minus2 | 549 | laminin subunit alpha | 2017 | 0 | 62.75% | 1 | F:protein binding | IPR000034 (PFAM); IPR000742 (SMART); IPR002049 (PFAM); IPR008211 (PFAM); IPR009030 (SUPERFAMILY); IPR018031 (SMART); PR00011 (PRINTS), G3DSA:2.10.25.10 (GENE3D), PTHR10574 (PANTHER), PTHR10574:SF39 (PANTHER), SSF57196 (SUPERFAMILY) |
| 315 | lib_dib_Filter15_IDBA_contig_12482_plus1 | 527 | microtubule-associated serine threonine-protein kinase 2 | 2008 | 0 | 71.65% | 10 | P:cellular protein modification process; P:reproduction; P:cell differentiation; F:protein binding; F:nucleotide binding; F:binding; C:cytoplasm; P:regulation of biological process; P:biosynthetic process; F:protein kinase activity | IPR000719 (PFAM); IPR000961 (PROFILE); IPR001478 (SMART); IPR002290 (SMART); IPR008271 (PROSITE); IPR011009 (SUPERFAMILY); IPR015022 (PFAM); IPR020635 (SMART); IPR023142 (G3DSA:1.20.1480.GENE3D); G3DSA:1.10.510.10 (GENE3D), G3DSA:2.30.42.10 (GENE3D), G3DSA:3.30.200.20 (GENE3D), PTHR24356 (PANTHER), PTHR24356:SF119 (PANTHER), tmhmm (TMHMM) |
| 316 | lib_dib_Filter15_IDBA_contig_12483_minus2 | 7197 | ankyrin isoform u | 2006 | 0 | 82.65% | 16 | F:cytoskeletal protein binding; P:anatomical structure morphogenesis; P:cellular component organization; P:cell differentiation; P:multicellular organismal development; P:cytoskeleton organization; C:cell; F:lipid binding; P:metabolic process; P:transport; C:cellular_component; P:signal transduction; P:cellular homeostasis; F:structural molecule activity; C:extracellular region; C:plasma membrane | IPR000488 (PFAM); IPR000906 (PFAM); IPR002110 (PRINTS); IPR011029 (G3DSA:1.10.533.GENE3D); IPR020683 (G3DSA:1.25.40.GENE3D); PTHR24123 (PANTHER), PTHR24123:SF0 (PANTHER) |
| 317 | lib_dib_Filter15_IDBA_contig_124872_plus1 | 22 | polypeptide n-acetylgalactosaminyltransferase 11 | 104 | 8.07E-34 | 81.75% | 7 | C:Golgi apparatus; F:transferase activity; P:biosynthetic process; P:cellular protein modification process; P:carbohydrate metabolic process; F:carbohydrate binding; C:cell | PTHR11675 (PANTHER), SSF53448 (SUPERFAMILY) |
| 318 | lib_dib_Filter15_IDBA_contig_125042_plus1 | 26 | neuronal cell adhesion | 114 | 1.15E-10 | 43.45% | 1 | F:protein binding | IPR007110 (PROFILE); IPR013783 (G3DSA:2.60.40.GENE3D); PTHR10489 (PANTHER), PF13895 (PFAM), SSF48726 (SUPERFAMILY) |
| 319 | lib_dib_Filter15_IDBA_contig_125167_minus1 | 130 | mtap4 protein | 101 | 1.60E-08 | 54.00% | 6 | F:tubulin binding; C:microtubule; C:cell wall; F:structural constituent of cell wall; P:multicellular organismal development; C:extracellular region | no IPS match |
| 320 | lib_dib_Filter15_IDBA_contig_125189_minus2 | 25 | nidogen and egf-like domain-containing protein 1-like | 106 | 7.64E-20 | 56.75% | 2 | F:protein binding; F:calcium ion binding | IPR000152 (PROSITE); IPR000742 (PFAM); IPR001881 (SMART); IPR013032 (PROSITE); PR00010 (PRINTS), G3DSA:2.10.25.10 (GENE3D), PTHR24838 (PANTHER), PTHR24838:SF112 (PANTHER), SSF57196 (SUPERFAMILY) |
| 321 | lib_dib_Filter15_IDBA_contig_125190_minus2 | 25 | neurogenic locus notch homolog protein 2-like | 104 | 8.25E-21 | 57.35% | 2 | F:protein binding; F:calcium ion binding | IPR000152 (PROSITE); IPR000742 (PFAM); IPR001881 (SMART); IPR013032 (PROSITE); PR00010 (PRINTS), G3DSA:2.10.25.10 (GENE3D), PTHR24044 (PANTHER), PTHR24044:SF0 (PANTHER), SSF57196 (SUPERFAMILY) |

| | | | | | | | | | |
|---|---|---|---|---|---|---|---|---|---|
| 322 | lib_dib_Filter15_IDBA_contig_12520_minus2 | 3661 | cub and sushi domain-containing protein 1- partial | 129 | 8.30E-21 | 54.10% | 7 | F:carbohydrate binding; P:cell adhesion; C:membrane; F:molecular_function; C:integral to membrane; P:biological_process; C:cellular_component | IPR000436 (PFAM); G3DSA:2.10.70.10 (GENE3D), PTHR19325 (PANTHER) |
| 323 | lib_dib_Filter15_IDBA_contig_12522_minus1 | 1550 | von willebrand factor type egf and pentraxin domain-containing protein 1 | 155 | 3.54E-30 | 51.35% | 6 | F:carbohydrate binding; C:cytoplasm; F:calcium ion binding; P:cell adhesion; C:membrane; C:extracellular region | IPR000436 (PFAM); G3DSA:2.10.70.10 (GENE3D), PTHR19325 (PANTHER) |
| 324 | lib_dib_Filter15_IDBA_contig_12546_plus1 | 26427 | 14-3-3 protein epsilon | 2112 | 3.60E-143 | 90.65% | 30 | P:cell differentiation; P:multicellular organismal development; P:cellular protein modification process; P:regulation of biological process; P:signal transduction; P:reproduction; P:cytoskeleton organization; F:protein binding; P:response to stress; P:cell cycle; P:protein transport; C:cytoplasmic membrane-bounded vesicle; P:response to abiotic stimulus; C:cytosol; P:cell death; C:extracellular region; P:organelle organization; F:catalytic activity; P:growth; P:behavior; C:chromosome; F:enzyme regulator activity; P:metabolic process; C:cell; C:cytoskeleton; C:protein complex; C:nucleus; P:anatomical structure morphogenesis; P:biological_process; C:mitochondrion | IPR000308 (PRINTS); IPR023409 (PROSITE); IPR023410 (G3DSA:1.20.190.GENE3D); PTHR18860:SF0 (PANTHER), tmhmm (TMHMM) |
| 325 | lib_dib_Filter15_IDBA_contig_12549_plus1 | 2705 | collagen alpha-3 chain-like | 3023 | 1.59E-154 | 45.10% | 2 | F:protein binding; F:calcium ion binding | IPR000152 (PROSITE); IPR000742 (PFAM); IPR001881 (SMART); IPR002035 (PFAM); IPR013032 (PROSITE); IPR018097 (PROSITE); PR00453 (PRINTS), G3DSA:2.10.25.10 (GENE3D), G3DSA:3.40.50.410 (GENE3D), PTHR22992 (PANTHER), SSF53300 (SUPERFAMILY), SSF57196 (SUPERFAMILY) |
| 326 | lib_dib_Filter15_IDBA_contig_12555_plus2 | 5266 | lipophorin receptor | 2603 | 0 | 61.25% | 3 | F:catalytic activity; F:protein binding; F:calcium ion binding | IPR000033 (PFAM); IPR000152 (PROSITE); IPR000742 (SMART); IPR001881 (SMART); IPR002172 (G3DSA:4.10.400.GENE3D); IPR009030 (SUPERFAMILY); IPR011042 (G3DSA:2.120.10.GENE3D); IPR013032 (PROSITE); IPR018097 (PROSITE); IPR023415 (PROSITE); IPR026823 (PFAM); PR00261 (PRINTS), G3DSA:2.10.25.10 (GENE3D), PTHR10529 (PANTHER), tmhmm (TMHMM), SSF57196 (SUPERFAMILY), SSF63825 (SUPERFAMILY) |
| 327 | lib_dib_Filter15_IDBA_contig_1256_minus1 | 4206 | a disintegrin and metalloproteinase with thrombospondin motifs partial | 421 | 1.41E-15 | 42.80% | 3 | F:peptidase activity; P:protein metabolic process; P:catabolic process | IPR001590 (PROFILE); IPR024079 (G3DSA:3.40.390.GENE3D); PTHR13723 (PANTHER), PTHR13723:SF22 (PANTHER), PF13574 (PFAM), SSF55486 (SUPERFAMILY) |

| | | | | | | | | | |
|---|---|---|---|---|---|---|---|---|---|
| 328 | lib_dib_Filter15_IDBA_contig_12598_plus2 | 1018 | transcription activator brg1-like | 2236 | 0 | 81.40% | 30 | C:nucleus; C:protein complex; P:anatomical structure morphogenesis; P:cellular component organization; P:regulation of gene expression, epigenetic; P:organelle organization; ; P:DNA metabolic process; F:protein binding; P:embryo development; C:cell; P:regulation of biological process; P:cell cycle; P:multicellular organismal development; P:cellular protein modification process; C:chromosome; C:nuclear chromosome; P:reproduction; P:viral reproduction; P:symbiosis, encompassing mutualism through parasitism; P:cell differentiation; F:transcription regulator activity; P:signal transduction; F:receptor binding; F:nucleotide binding; F:chromatin binding; F:hydrolase activity; P:growth; P:cell growth; P:biological_process | IPR000330 (PFAM); IPR001487 (PRINTS); IPR001650 (PFAM); IPR006562 (PFAM); IPR006576 (PFAM); IPR013999 (SMART); IPR014001 (SMART); IPR014012 (PROFILE); IPR014978 (PFAM); IPR018359 (PROSITE); G3DSA:3.40.50.300 (GENE3D), PTHR10799 (PANTHER), PTHR10799:SF76 (PANTHER), SSF52540 (SUPERFAMILY) |
| 329 | lib_dib_Filter15_IDBA_contig_12600_plus2 | 3483 | protein ssq-2 | 2659 | 1.34E-33 | 50.75% | 1 | P:locomotion | PTHR31192 (PANTHER), PTHR31192:SF0 (PANTHER), tmhmm (TMHMM) |
| 330 | lib_dib_Filter15_IDBA_contig_12603_minus1 | 3696 | low-density lipoprotein receptor-related protein 2 | 1965 | 0 | 56.90% | 20 | P:protein transport; P:response to abiotic stimulus; P:biosynthetic process; P:lipid metabolic process; P:transport; P:cellular component organization; C:plasma membrane; P:cell-cell signaling; P:anatomical structure morphogenesis; P:multicellular organismal development; P:response to external stimulus; C:cell; P:metabolic process; P:biological_process; C:extracellular space; F:protein binding; C:endosome; F:receptor activity; F:transporter activity; F:calcium ion binding | IPR000033 (PFAM); IPR000152 (PROSITE); IPR000742 (SMART); IPR001881 (PFAM); IPR002172 (G3DSA:4.10.400.GENE3D); IPR009030 (SUPERFAMILY); IPR011042 (G3DSA:2.120.10.GENE3D); IPR013032 (PROSITE); IPR018097 (PROSITE); IPR023415 (PROSITE); IPR026823 (PFAM); PR00261 (PRINTS), G3DSA:2.10.25.10 (GENE3D), G3DSA:4.10.1220.10 (GENE3D), PTHR10529 (PANTHER), PTHR10529:SF113 (PANTHER), tmhmm (TMHMM), SSF63825 (SUPERFAMILY) |
| 331 | lib_dib_Filter15_IDBA_contig_12605_plus1 | 8756 | collagen alpha-6 chain-like | 2633 | 0 | 52.50% | 2 | F:protein binding; F:calcium ion binding | IPR000152 (PROSITE); IPR000742 (SMART); IPR001881 (SMART); IPR002035 (PFAM); IPR013032 (PROSITE); IPR018097 (PROSITE); PR00453 (PRINTS), G3DSA:2.10.25.10 (GENE3D), G3DSA:3.40.50.410 (GENE3D), PTHR22992 (PANTHER), tmhmm (TMHMM), SSF53300 (SUPERFAMILY) |
| 332 | lib_dib_Filter15_IDBA_contig_12607_plus2 | 168 | collagen alpha-1 chain- partial | 138 | 2.37E-23 | 56.65% | 1 | F:protein binding | IPR002035 (PFAM); PR00453 (PRINTS), G3DSA:3.40.50.410 (GENE3D), PTHR22992 (PANTHER), SSF53300 (SUPERFAMILY) |
| 333 | lib_dib_Filter15_IDBA_contig_12648_minus2 | 2682 | myosin light chain smooth muscle | 1704 | 5.63E-123 | 76.85% | 3 | F:protein kinase activity; P:cellular protein modification process; F:nucleotide binding | IPR000719 (PFAM); IPR002290 (SMART); IPR008271 (PROSITE); IPR011009 (SUPERFAMILY); IPR017441 (PROSITE); IPR020635 (SMART); IPR020675 (PANTHER); G3DSA:1.10.510.10 (GENE3D), G3DSA:3.30.200.20 (GENE3D), PTHR22964:SF3 (PANTHER), SignalP-NN(euk) (SIGNALP) |
| 334 | lib_dib_Filter15_IDBA_contig_12659_plus2 | 1124 | maltase- intestinal-like | 2154 | 0 | 57.70% | 3 | F:hydrolase activity; P:carbohydrate metabolic process; F:carbohydrate binding | IPR000322 (PANTHER); IPR000519 (G3DSA:4.10.110.GENE3D); IPR011013 (SUPERFAMILY); IPR017853 (SUPERFAMILY); PTHR22762:SF22 (PANTHER), tmhmm (TMHMM), SSF51011 (SUPERFAMILY) |

| | | | | | | | | | | |
|---|---|---|---|---|---|---|---|---|---|---|
| 335 | lib_dib_Filter15_IDBA_contig_12688_plus1 | 447 | fibronectin type-iii domain-containing protein 3a isoform 2 | 1627 | 0 | 64.15% | 3 | C:organelle; C:cytoplasm; F:protein binding | IPR003961 (PFAM); IPR013783 (G3DSA:2.60.40.GENE3D); PR00014 (PRINTS) | |
| 336 | lib_dib_Filter15_IDBA_contig_12733_minus3 | 2082 | stonustoxin subunit alpha | 1590 | 0 | 57.85% | 3 | F:nucleotide binding; P:cell cycle; F:protein binding | IPR000038 (PANTHER); IPR003961 (PFAM); IPR013783 (G3DSA:2.60.40.GENE3D); G3DSA:3.40.50.300 (GENE3D), PTHR18884:SF30 (PANTHER), SSF52540 (SUPERFAMILY) | |
| 337 | lib_dib_Filter15_IDBA_contig_12741_minus1 | 1654 | nucleoprotein tpr | 2518 | 1.64E-85 | 49.95% | 8 | C:protein complex; C:chromosome; P:transport; P:regulation of biological process; F:binding; P:metabolic process; C:nuclear envelope; P:protein transport | IPR009053 (SUPERFAMILY); IPR012929 (PFAM); PTHR18898 (PANTHER), tmhmm (TMHMM) | |
| 338 | lib_dib_Filter15_IDBA_contig_12742_minus1 | 425 | tenascin-like protein | 1500 | 0 | 51.55% | 5 | C:integral to plasma membrane; C:integral to membrane; P:signal transduction; P:metabolic process; F:catalytic activity | IPR011042 (G3DSA:2.120.10.GENE3D); PTHR11219 (PANTHER), SSF101898 (SUPERFAMILY), SSF63825 (SUPERFAMILY) | |
| 339 | lib_dib_Filter15_IDBA_contig_12743_plus2 | 6281 | fk506-binding protein | 1876 | 2.87E-51 | 85.40% | 5 | P:protein metabolic process; C:endoplasmic reticulum; F:binding; P:cellular protein modification process; F:catalytic activity | IPR001179 (PFAM); IPR023566 (PANTHER); G3DSA:3.10.50.40 (GENE3D), PTHR10516:SF116 (PANTHER), tmhmm (TMHMM), SSF54534 (SUPERFAMILY) | |
| 340 | lib_dib_Filter15_IDBA_contig_12745_minus3 | 606 | peroxidasin homolog | 1696 | 0 | 66.05% | 12 | P:response to stress; P:catabolic process; P:metabolic process; C:extracellular space; F:antioxidant activity; F:catalytic activity; F:binding; C:extracellular region; F:structural molecule activity; P:cellular component organization; C:endoplasmic reticulum; F:protein binding | IPR000372 (SMART); IPR000483 (SMART); IPR001007 (PFAM); IPR001611 (PROFILE); IPR002007 (G3DSA:1.10.640.GENE3D); IPR003591 (SMART); IPR003598 (SMART); IPR003599 (SMART); IPR007110 (PROFILE); IPR010255 (SUPERFAMILY); IPR013098 (PFAM); IPR013783 (G3DSA:2.60.40.GENE3D); IPR019791 (PRINTS); G3DSA:3.80.10.10 (GENE3D), PTHR11475 (PANTHER), PTHR11475:SF9 (PANTHER), PF13855 (PFAM), SM00365 (SMART), SignalP-NN(euk) (SIGNALP), SSF48726 (SUPERFAMILY), SSF52058 (SUPERFAMILY), SSF57603 (SUPERFAMILY) | Yes |
| 341 | lib_dib_Filter15_IDBA_contig_12746_plus2 | 1979 | basement membrane-specific heparan sulfate proteoglycan core protein | 2432 | 0 | 49.60% | 5 | P:multicellular organismal development; C:extracellular region; P:anatomical structure morphogenesis; F:protein binding; F:calcium ion binding | IPR000742 (SMART); IPR001791 (PFAM); IPR001881 (SMART); IPR002172 (G3DSA:4.10.400.GENE3D); IPR003598 (SMART); IPR003599 (SMART); IPR007110 (PROFILE); IPR008985 (SUPERFAMILY); IPR013032 (PROSITE); IPR013098 (PFAM); IPR013320 (G3DSA:2.60.120.GENE3D); IPR013783 (G3DSA:2.60.40.GENE3D); IPR023415 (PROSITE); PR00261 (PRINTS), G3DSA:2.10.25.10 (GENE3D), G3DSA:4.10.1220.10 (GENE3D), PTHR10574 (PANTHER), PTHR10574:SF25 (PANTHER), PF13895 (PFAM), PF13927 (PFAM), tmhmm (TMHMM), SSF48726 (SUPERFAMILY), SSF57196 (SUPERFAMILY) | |
| 342 | lib_dib_Filter15_IDBA_contig_12747_plus1 | 34 | basement membrane-specific heparan sulfate proteoglycan core | 121 | 6.66E-19 | 51.50% | 5 | P:biological_process; P:multicellular organismal development; C:proteinaceous extracellular matrix; P:anatomical structure morphogenesis; F:protein binding | IPR013098 (PFAM); IPR013783 (G3DSA:2.60.40.GENE3D); PTHR10489 (PANTHER), SSF48726 (SUPERFAMILY) | |
| 343 | lib_dib_Filter15_IDBA_contig_12748_minus3 | 16244 | deleted in malignant brain tumors 1 | 2137 | 7.84E-127 | 56.10% | 5 | P:biological_process; P:response to stress; C:cytoplasmic membrane-bounded vesicle; F:protein binding; F:receptor activity | IPR000519 (G3DSA:4.10.110.GENE3D); IPR001190 (PRINTS); IPR017448 (SMART); G3DSA:3.10.250.10 (GENE3D), PTHR19331 (PANTHER) | |
| 344 | lib_dib_Filter15_IDBA_contig_12749_minus3 | 10384 | deleted in malignant brain tumors 1 | 804 | 8.01E-138 | 56.50% | 5 | P:biological_process; P:response to stress; C:cytoplasmic membrane-bounded vesicle; F:protein binding; F:receptor activity | IPR000519 (G3DSA:4.10.110.GENE3D); IPR001190 (PRINTS); IPR017448 (SMART); G3DSA:3.10.250.10 (GENE3D), PTHR19331 (PANTHER) | |

| | | | | | | | | | | |
|---|---|---|---|---|---|---|---|---|---|---|
| 345 | lib_dib_Filter15_IDBA_contig_12752_minus2 | 1964 | contactin-associated protein 1 | 1643 | 0 | 57.50% | 10 | P:biological_process; P:cell communication; P:cellular homeostasis; P:multicellular organismal development; P:anatomical structure morphogenesis; P:embryo development; P:cellular component organization; C:plasma membrane; P:cell differentiation; F:protein binding | IPR000421 (PFAM); IPR000742 (SMART); IPR001791 (PFAM); IPR008979 (SUPERFAMILY); IPR008985 (SUPERFAMILY); IPR013320 (G3DSA:2.60.120.GENE3D); G3DSA:2.10.25.10 (GENE3D), G3DSA:2.60.120.260 (GENE3D), PTHR10127 (PANTHER), PTHR10127:SF266 (PANTHER), SignalP-NN(euk) (SIGNALP), tmhmm (TMHMM), SSF57196 (SUPERFAMILY) | Yes |
| 346 | lib_dib_Filter15_IDBA_contig_12753_minus2 | 2031 | contactin-associated protein 1 | 1789 | 0 | 57.50% | 10 | P:biological_process; P:cell communication; P:cellular homeostasis; P:multicellular organismal development; P:anatomical structure morphogenesis; P:embryo development; P:cellular component organization; C:plasma membrane; P:cell differentiation; F:protein binding | IPR000421 (PFAM); IPR000742 (SMART); IPR001791 (PFAM); IPR008979 (SUPERFAMILY); IPR008985 (SUPERFAMILY); IPR013320 (G3DSA:2.60.120.GENE3D); G3DSA:2.10.25.10 (GENE3D), G3DSA:2.60.120.260 (GENE3D), PTHR10127 (PANTHER), PTHR10127:SF266 (PANTHER), SignalP-NN(euk) (SIGNALP), tmhmm (TMHMM), SSF57196 (SUPERFAMILY) | Yes |
| 347 | lib_dib_Filter15_IDBA_contig_1276_minus1 | 1062 | stonustoxin subunit alpha | 874 | 1.20E-146 | 55.85% | 1 | F:protein binding | IPR003961 (PFAM); IPR013783 (G3DSA:2.60.40.GENE3D); PTHR10489 (PANTHER) | |
| 348 | lib_dib_Filter15_IDBA_contig_12770_plus2 | 7728 | | 1482 | 0 | 100% | 4 | F:nucleotide binding; P:cellular protein modification process; F:protein binding; F:protein kinase activity | IPR000719 (PFAM); IPR002290 (SMART); IPR003598 (SMART); IPR003599 (SMART); IPR003961 (PFAM); IPR007110 (PROFILE); IPR008271 (PROSITE); IPR011009 (SUPERFAMILY); IPR013098 (PFAM); IPR013783 (G3DSA:2.60.40.GENE3D); IPR017441 (PROSITE); IPR020635 (SMART); IPR020675 (PANTHER); G3DSA:1.10.510.10 (GENE3D), G3DSA:3.30.200.20 (GENE3D), PTHR22964:SF3 (PANTHER), SSF48726 (SUPERFAMILY) | |
| 349 | lib_dib_Filter15_IDBA_contig_12771_plus2 | 3874 | striated muscle-specific serine threonine-protein kinase-like | 163 | 5.42E-42 | 54.60% | 2 | F:transferase activity; F:protein binding | IPR003598 (SMART); IPR003599 (SMART); IPR007110 (PROFILE); IPR013098 (PFAM); IPR013783 (G3DSA:2.60.40.GENE3D); PTHR10489 (PANTHER), SSF48726 (SUPERFAMILY) | |
| 350 | lib_dib_Filter15_IDBA_contig_12772_plus2 | 1181 | | 194 | 0 | 100% | 1 | F:protein binding | IPR003961 (PFAM); IPR013098 (PFAM); IPR013783 (G3DSA:2.60.40.GENE3D); IPR020675 (PANTHER); PR00014 (PRINTS), SSF48726 (SUPERFAMILY) | |
| 351 | lib_dib_Filter15_IDBA_contig_12773_plus3 | 5059 | aael005317- partial | 251 | 3.22E-26 | 44.90% | 1 | F:protein binding | IPR003598 (SMART); IPR003599 (SMART); IPR007110 (PROFILE); IPR013098 (PFAM); IPR013783 (G3DSA:2.60.40.GENE3D); PTHR10489 (PANTHER), SSF48726 (SUPERFAMILY) | |
| 352 | lib_dib_Filter15_IDBA_contig_12775_plus2 | 448 | | 251 | 0 | 100% | 1 | F:protein binding | IPR003961 (PFAM); IPR013783 (G3DSA:2.60.40.GENE3D); PR00014 (PRINTS) | |
| 353 | lib_dib_Filter15_IDBA_contig_12776_plus1 | 3749 | | 775 | 0 | 100% | 1 | F:protein binding | IPR003598 (SMART); IPR003599 (SMART); IPR003961 (PFAM); IPR007110 (PROFILE); IPR013098 (PFAM); IPR013783 (G3DSA:2.60.40.GENE3D); PR00014 (PRINTS), SSF48726 (SUPERFAMILY) | |
| 354 | lib_dib_Filter15_IDBA_contig_12779_plus1 | 851 | | 234 | 0 | 100% | 1 | F:protein binding | IPR003598 (SMART); IPR003599 (SMART); IPR003961 (PFAM); IPR007110 (PROFILE); IPR013098 (PFAM); IPR013783 (G3DSA:2.60.40.GENE3D); PTHR13817 (PANTHER), PTHR13817:SF2 (PANTHER), SSF48726 (SUPERFAMILY) | |
| 355 | lib_dib_Filter15_IDBA_contig_12780_plus1 | 1880 | | 502 | 0 | 100% | 1 | F:protein binding | IPR003599 (SMART); IPR003961 (PFAM); IPR007110 (PROFILE); IPR013098 (PFAM); IPR013783 (G3DSA:2.60.40.GENE3D); PR00014 (PRINTS), SSF48726 (SUPERFAMILY) | |

| | | | | | | | | | |
|---|---|---|---|---|---|---|---|---|---|
| 356 | lib_dib_Filter15_IDBA_contig_12782_plus1 | 839 | | 263 | 0 | 100% | 1 | F:protein binding | IPR003961 (PFAM); IPR013783 (G3DSA:2.60.40.GENE3D); PR00014 (PRINTS) |
| 357 | lib_dib_Filter15_IDBA_contig_12783_plus1 | 479 | | 207 | 0 | 100% | 1 | F:protein binding | IPR003961 (PFAM); IPR013783 (G3DSA:2.60.40.GENE3D); PR00014 (PRINTS) |
| 358 | lib_dib_Filter15_IDBA_contig_12786_plus1 | 346 | | 129 | 0 | 100% | 1 | F:protein binding | IPR003961 (PFAM); IPR013783 (G3DSA:2.60.40.GENE3D); PR00014 (PRINTS) |
| 359 | lib_dib_Filter15_IDBA_contig_12788_plus1 | 3049 | | 449 | 0 | 100% | 1 | F:protein binding | IPR003961 (PFAM); IPR007110 (PROFILE); IPR013098 (PFAM); IPR013783 (G3DSA:2.60.40.GENE3D); PR00014 (PRINTS), SSF48726 (SUPERFAMILY) |
| 360 | lib_dib_Filter15_IDBA_contig_12789_plus1 | 3799 | | 828 | 0 | 100% | 1 | F:protein binding | IPR003598 (SMART); IPR003599 (SMART); IPR003961 (PFAM); IPR007110 (PROFILE); IPR013098 (PFAM); IPR013783 (G3DSA:2.60.40.GENE3D); PR00014 (PRINTS), SSF48726 (SUPERFAMILY) |
| 361 | lib_dib_Filter15_IDBA_contig_12790_plus1 | 1035 | | 368 | 0 | 100% | 1 | F:protein binding | IPR003598 (SMART); IPR003599 (SMART); IPR003961 (PFAM); IPR007110 (PROFILE); IPR013098 (PFAM); IPR013783 (G3DSA:2.60.40.GENE3D); PR00014 (PRINTS), SSF48726 (SUPERFAMILY) |
| 362 | lib_dib_Filter15_IDBA_contig_12791_plus1 | 1820 | | 297 | 0 | 100% | 1 | F:protein binding | IPR003599 (SMART); IPR003961 (PFAM); IPR007110 (PROFILE); IPR013098 (PFAM); IPR013783 (G3DSA:2.60.40.GENE3D); PR00014 (PRINTS), SSF48726 (SUPERFAMILY) |
| 363 | lib_dib_Filter15_IDBA_contig_12808_plus3 | 59301 | | 5903 | 0 | 100% | 1 | F:protein binding | IPR003596 (SMART); IPR003598 (SMART); IPR003599 (SMART); IPR007110 (PROFILE); IPR013098 (PFAM); IPR013783 (G3DSA:2.60.40.GENE3D); PTHR13817 (PANTHER), tmhmm (TMHMM), SSF48726 (SUPERFAMILY) |
| 364 | lib_dib_Filter15_IDBA_contig_12834_minus2 | 766 | serine threonine-protein phosphatase 6 regulatory ankyrin repeat subunit c-like | 1586 | 7.97E-43 | 42.80% | 4 | F:protein binding; C:intracellular; P:regulation of biological process; P:cell death | IPR001315 (PFAM); IPR002110 (PFAM); IPR011029 (G3DSA:1.10.533.GENE3D); IPR020683 (G3DSA:1.25.40.GENE3D); PTHR24149 (PANTHER), tmhmm (TMHMM) |
| 365 | lib_dib_Filter15_IDBA_contig_12838_minus2 | 1517 | von willebrand factor d and egf domain-containing protein | 1458 | 9.06E-171 | 43.30% | 9 | F:polysaccharide binding; F:calcium ion binding; F:scavenger receptor activity; F:carbohydrate binding; C:membrane; P:immune response; F:chitin binding; P:chitin metabolic process; C:extracellular region | IPR001846 (PFAM); tmhmm (TMHMM) |
| 366 | lib_dib_Filter15_IDBA_contig_12843_plus1 | 22618 | ryanodine receptor | 2707 | 0 | 66.15% | 7 | C:cytoskeleton; C:protein complex; C:cell; F:ion channel activity; P:biological_process; P:ion transport; F:protein binding | IPR000699 (PFAM); IPR001870 (PROFILE); IPR003032 (PFAM); IPR003877 (PFAM); IPR008985 (SUPERFAMILY); IPR013333 (PRINTS); IPR015925 (PANTHER); IPR018355 (SMART); G3DSA:1.25.10.30 (GENE3D), PTHR13715:SF13 (PANTHER) |
| 367 | lib_dib_Filter15_IDBA_contig_12884_minus1 | 522 | insulin-like growth factor-binding protein complex acid labile chain | 1473 | 9.07E-84 | 47.60% | 2 | F:catalytic activity; F:protein binding | IPR000483 (SMART); IPR001611 (PFAM); IPR003591 (SMART); IPR026906 (PFAM); G3DSA:3.80.10.10 (GENE3D), PTHR24365 (PANTHER), PF13855 (PFAM), SM00365 (SMART), tmhmm (TMHMM), SSF52058 (SUPERFAMILY) |

| | | | | | | | | | | |
|---|---|---|---|---|---|---|---|---|---|---|
| 368 | lib_dib_Filter15_IDBA_contig_12889_minus1 | 1143 | protein flightless-1 homolog | 1450 | 0 | 76.35% | 10 | P:anatomical structure morphogenesis; P:cell differentiation; P:multicellular organismal development; P:cytoskeleton organization; C:nucleolus; C:microtubule organizing center; ; F:hydrolase activity; P:biological_process; F:actin binding | IPR001611 (PROFILE); IPR003591 (SMART); IPR007122 (PRINTS); IPR007123 (PFAM); IPR025875 (PFAM); G3DSA:3.40.20.10 (GENE3D), G3DSA:3.80.10.10 (GENE3D), PTHR11977:SF6 (PANTHER), PF13855 (PFAM), SM00364 (SMART), SSF52058 (SUPERFAMILY), SSF55753 (SUPERFAMILY) | |
| 369 | lib_dib_Filter15_IDBA_contig_12900_minus3 | 3973 | collagen alpha-6 chain-like | 3318 | 1.47E-112 | 46.45% | 2 | F:protein binding; F:calcium ion binding | IPR000742 (SMART); IPR001881 (SMART); IPR002035 (PFAM); IPR008160 (PFAM); IPR018097 (PROSITE); PR00453 (PRINTS), G3DSA:2.10.25.10 (GENE3D), G3DSA:3.40.50.410 (GENE3D), PTHR24023 (PANTHER), tmhmm (TMHMM), SSF53300 (SUPERFAMILY), SSF57196 (SUPERFAMILY) | |
| 370 | lib_dib_Filter15_IDBA_contig_12900_plus2 | 3973 | collagen-like protein | 3319 | 4.32E-06 | 67.67% | 1 | C:collagen | PTHR24023 (PANTHER), PTHR24023:SF79 (PANTHER), tmhmm (TMHMM) | |
| 371 | lib_dib_Filter15_IDBA_contig_12901_minus1 | 34 | collagen alpha-1 chain-like | 153 | 1.26E-08 | 50.25% | 1 | F:protein binding | IPR002035 (PFAM); G3DSA:3.40.50.410 (GENE3D), PTHR22992 (PANTHER), SSF53300 (SUPERFAMILY) | |
| 372 | lib_dib_Filter15_IDBA_contig_12915_minus1 | 803 | fibroblast growth factor receptor 2 (bacteria-expressed keratinocyte growth factor craniofacial dysostosis crouzon pfeiffer jackson-weiss syndrome) isoform cra_k | 1712 | 0 | 57.90% | 25 | P:biological_process; P:anatomical structure morphogenesis; P:cellular component organization; P:cell differentiation; P:multicellular organismal development; P:embryo development; P:cellular protein modification process; P:reproduction; F:protein binding; P:metabolic process; P:signal transduction; C:cytoplasm; C:cell; F:receptor activity; F:protein kinase activity; P:regulation of biological process; P:lipid metabolic process; C:nucleus; P:growth; P:cell proliferation; P:cell death; C:extracellular region; P:response to endogenous stimulus; C:plasma membrane; F:nucleotide binding | IPR000719 (PROFILE); IPR001245 (PFAM); IPR001824 (PROSITE); IPR002290 (SMART); IPR003598 (SMART); IPR003599 (SMART); IPR007110 (PROFILE); IPR008266 (PROSITE); IPR011009 (SUPERFAMILY); IPR013098 (PFAM); IPR013106 (PFAM); IPR013783 (G3DSA:2.60.40.GENE3D); IPR017441 (PROSITE); IPR020635 (SMART); G3DSA:1.10.510.10 (GENE3D), G3DSA:3.30.200.20 (GENE3D), PTHR24416 (PANTHER), PTHR24416:SF45 (PANTHER), PF13895 (PFAM), PF13927 (PFAM), tmhmm (TMHMM), SSF48726 (SUPERFAMILY) | |
| 373 | lib_dib_Filter15_IDBA_contig_12918_plus2 | 301 | fibrillin-2 | 1397 | 0 | 61.50% | 7 | P:regulation of biological process; P:multicellular organismal development; P:anatomical structure morphogenesis; C:proteinaceous extracellular matrix; F:protein binding; F:calcium ion binding; F:structural molecule activity | IPR000152 (PROSITE); IPR000742 (PFAM); IPR001881 (PFAM); IPR009030 (SUPERFAMILY); IPR011398 (PTHR24039:PANTHER); IPR013032 (PROSITE); IPR017878 (G3DSA:3.90.290.GENE3D); IPR018097 (PROSITE); IPR026823 (PFAM); PD936484 (PRODOM), G3DSA:2.10.25.10 (GENE3D), PTHR24039 (PANTHER), SSF57196 (SUPERFAMILY) | |
| 374 | lib_dib_Filter15_IDBA_contig_12930_plus2 | 2164 | ---NA--- | 2605 | | | 0 | - | tmhmm (TMHMM) | |
| 375 | lib_dib_Filter15_IDBA_contig_12931_plus1 | 1919 | ---NA--- | 2439 | | | 0 | - | tmhmm (TMHMM) | |
| 376 | lib_dib_Filter15_IDBA_contig_12932_plus3 | 929 | extracellular sulfatase sulf-1 | 1607 | 0 | 63.85% | 13 | P:signal transduction; F:hydrolase activity; C:cell; P:regulation of biological process; P:multicellular organismal development; P:anatomical structure morphogenesis; C:extracellular space; C:Golgi apparatus; P:cell proliferation; P:embryo development; P:metabolic process; C:endoplasmic reticulum; P:biological_process | IPR000917 (PFAM); IPR017849 (G3DSA:3.40.720.GENE3D); IPR017850 (SUPERFAMILY); IPR024607 (PROSITE); IPR024609 (PFAM); PTHR10342 (PANTHER), PTHR10342:SF7 (PANTHER) | Yes |
| 377 | lib_dib_Filter15_IDBA_contig_12955_plus1 | 11129 | centromeric protein | 1615 | 9.04E-49 | 54.55% | 2 | P:metabolic process; F:catalytic activity | IPR009053 (SUPERFAMILY); PTHR18937 (PANTHER), PTHR18937:SF52 (PANTHER) | |

| | | | | | | | | | | |
|---|---|---|---|---|---|---|---|---|---|---|
| 378 | lib_dib_Filter15_IDBA_contig_12969_minus3 | 564 | insulin-like growth factor-binding protein complex acid labile chain | 1342 | 3.24E-60 | 44.45% | 1 | F:protein binding | IPR000372 (PFAM); IPR000483 (SMART); IPR001611 (PROFILE); IPR003591 (SMART); G3DSA:3.80.10.10 (GENE3D), PTHR24365 (PANTHER), PF13504 (PFAM), PF13855 (PFAM), SM00365 (SMART), tmhmm (TMHMM), SSF52058 (SUPERFAMILY) | |
| 379 | lib_dib_Filter15_IDBA_contig_12977_minus1 | 427 | -like protein subfamily c member 14 | 1651 | 4.14E-87 | 71.05% | 4 | P:protein metabolic process; F:protein binding; P:response to stress; P:response to abiotic stimulus | IPR001623 (PRINTS); PTHR24078 (PANTHER), PTHR24078:SF23 (PANTHER), tmhmm (TMHMM) | |
| 380 | lib_dib_Filter15_IDBA_contig_12978_plus3 | 3882 | bcl-6 corepressor | 2593 | 4.32E-56 | 58.50% | 8 | P:regulation of biological process; ; F:protein binding; F:transcription regulator activity; P:organelle organization; P:cellular protein modification process; P:multicellular organismal development; C:nucleus | IPR002110 (PRINTS); IPR020683 (G3DSA:1.25.40.GENE3D); PTHR24117 (PANTHER), tmhmm (TMHMM) | |
| 381 | lib_dib_Filter15_IDBA_contig_12994_minus3 | 444 | dystroglycan preproprotein | 1376 | 5.87E-91 | 56.55% | 12 | P:multicellular organismal development; C:organelle; P:signal transduction; C:protein complex; C:plasma membrane; C:cytoplasm; F:cytoskeletal protein binding; C:cell; C:intracellular; C:extracellular region; F:calcium ion binding; P:cytoskeleton organization | IPR006644 (SMART); IPR008465 (PTHR21559:PANTHER); IPR013783 (G3DSA:2.60.40.GENE3D); IPR015919 (SUPERFAMILY); G3DSA:3.30.70.1040 (GENE3D), PTHR21559 (PANTHER), SignalP-NN(euk) (SIGNALP), tmhmm (TMHMM), SSF111006 (SUPERFAMILY) | |
| 382 | lib_dib_Filter15_IDBA_contig_13010_plus3 | 9920 | | 1300 | 0 | 100% | 1 | F:protein binding | IPR003598 (SMART); IPR003599 (SMART); IPR003961 (PFAM); IPR007110 (PROFILE); IPR013098 (PFAM); IPR013783 (G3DSA:2.60.40.GENE3D); PR00014 (PRINTS), SignalP-NN(euk) (SIGNALP), SSF48726 (SUPERFAMILY) | Yes |
| 383 | lib_dib_Filter15_IDBA_contig_13011_plus1 | 1242 | | 673 | 0 | 100% | 1 | F:protein binding | IPR003961 (PFAM); IPR013783 (G3DSA:2.60.40.GENE3D); PR00014 (PRINTS) | |
| 384 | lib_dib_Filter15_IDBA_contig_13012_plus1 | 2822 | | 198 | 0 | 100% | 1 | F:protein binding | IPR003961 (PFAM); IPR013783 (G3DSA:2.60.40.GENE3D); PR00014 (PRINTS) | |
| 385 | lib_dib_Filter15_IDBA_contig_13016_plus1 | 200 | protein unc- isoform b | 833 | 2.99E-07 | 51.35% | 1 | F:protein binding | IPR003598 (SMART); IPR003599 (SMART); IPR007110 (PROFILE); IPR013098 (PFAM); IPR013783 (G3DSA:2.60.40.GENE3D); PTHR10489 (PANTHER), SSF48726 (SUPERFAMILY) | |
| 386 | lib_dib_Filter15_IDBA_contig_13020_plus1 | 758 | immunoglobulin i-set domain containing protein | 312 | 5.62E-10 | 51.55% | 11 | F:kinase activity; F:transferase activity; P:anatomical structure morphogenesis; P:cell differentiation; P:multicellular organismal development; P:cytoskeleton organization; P:biological_process; C:cytoplasm; F:cytoskeletal protein binding; F:protein binding; P:metabolic process | IPR003599 (SMART); IPR007110 (PROFILE); IPR013098 (PFAM); IPR013783 (G3DSA:2.60.40.GENE3D); PTHR25963 (PANTHER), SSF48726 (SUPERFAMILY) | |
| 387 | lib_dib_Filter15_IDBA_contig_13021_plus1 | 3880 | immunoglobulin i-set domain containing protein | 728 | 2.14E-09 | 52.50% | 3 | C:cytoplasm; P:cytoskeleton organization; F:protein binding | IPR003598 (SMART); IPR003599 (SMART); IPR007110 (PROFILE); IPR013098 (PFAM); IPR013783 (G3DSA:2.60.40.GENE3D); PTHR10489 (PANTHER), SSF48726 (SUPERFAMILY) | |
| 388 | lib_dib_Filter15_IDBA_contig_13029_plus3 | 859 | | 1073 | 0 | 100% | 1 | F:protein binding | IPR003598 (SMART); IPR003599 (SMART); IPR007110 (PROFILE); IPR013098 (PFAM); IPR013783 (G3DSA:2.60.40.GENE3D); SSF48726 (SUPERFAMILY) | |
| 389 | lib_dib_Filter15_IDBA_contig_13030_plus1 | 1987 | | 1298 | 0 | 100% | 1 | F:protein binding | IPR003599 (SMART); IPR003961 (PFAM); IPR007110 (PROFILE); IPR013098 (PFAM); IPR013783 (G3DSA:2.60.40.GENE3D); PR00014 (PRINTS), SSF48726 (SUPERFAMILY) | |

| | | | | | | | | | |
|---|---|---|---|---|---|---|---|---|---|
| 390 | lib_dib_Filter15_IDBA_contig_13031_plus1 | 170 | | 101 | 0 | 100% | 1 | F:protein binding | IPR003961 (PFAM); IPR013783 (G3DSA:2.60.40.GENE3D); PR00014 (PRINTS), PTHR10489 (PANTHER) |
| 391 | lib_dib_Filter15_IDBA_contig_13032_plus1 | 417 | low quality protein: titin | 160 | 4.82E-31 | 55.55% | 4 | F:protein binding; F:protein kinase activity; F:nucleotide binding; P:cellular protein modification process | IPR007110 (PROFILE); IPR013098 (PFAM); IPR013783 (G3DSA:2.60.40.GENE3D); IPR015726 (PTHR22964:PANTHER); IPR020675 (PANTHER); SSF48726 (SUPERFAMILY) |
| 392 | lib_dib_Filter15_IDBA_contig_13047_minus1 | 3517 | low-density lipoprotein receptor-related protein 4 | 1714 | 0 | 45.15% | 3 | P:multicellular organismal development; P:biological_process; F:protein binding | IPR000033 (PFAM); IPR000436 (SMART); IPR000742 (SMART); IPR011042 (G3DSA:2.120.10.GENE3D); G3DSA:2.10.25.10 (GENE3D), G3DSA:2.10.70.10 (GENE3D), PTHR10529 (PANTHER), tmhmm (TMHMM), SSF57196 (SUPERFAMILY), SSF63825 (SUPERFAMILY) |
| 393 | lib_dib_Filter15_IDBA_contig_13048_minus1 | 275 | low-density lipoprotein receptor-related protein 1b | 252 | 1.92E-16 | 51.00% | 2 | C:cell; F:binding | IPR000033 (SMART); IPR011042 (G3DSA:2.120.10.GENE3D); PTHR10529 (PANTHER), SSF63825 (SUPERFAMILY) |
| 394 | lib_dib_Filter15_IDBA_contig_13062_plus3 | 3365 | g5 domain family | 805 | 6.27E-09 | 61.06% | 11 | P:chromosome condensation; F:DNA binding; C:cell wall; C:perinuclear region of cytoplasm; P:regulation of transcription, DNA-dependent; C:cytoplasm; P:transcription, DNA-dependent; F:nucleotide binding; P:cellular metabolic process; F:catalytic activity; F:coenzyme binding | no IPS match |
| 395 | lib_dib_Filter15_IDBA_contig_13064_plus2 | 934 | ---NA--- | 291 | | | 0 | - | no IPS match |
| 396 | lib_dib_Filter15_IDBA_contig_13096_minus2 | 1841 | collagen alpha-1 chain | 447 | 4.27E-37 | 52.40% | 1 | F:protein binding | IPR002035 (PFAM); PR00453 (PRINTS), G3DSA:3.40.50.410 (GENE3D), PTHR22992 (PANTHER), SignalP-NN(euk) (SIGNALP), tmhmm (TMHMM), SSF53300 (SUPERFAMILY) |
| 397 | lib_dib_Filter15_IDBA_contig_13131_plus3 | 735 | neurogenic locus notch homolog protein 2-like | 2032 | 3.58E-60 | 44.70% | 2 | F:protein binding; F:calcium ion binding | IPR000152 (PROSITE); IPR000742 (PFAM); IPR001881 (SMART); IPR006212 (SMART); IPR009030 (SUPERFAMILY); IPR011641 (PFAM); IPR013032 (PFAM); G3DSA:2.10.25.10 (GENE3D), PTHR24044 (PANTHER), tmhmm (TMHMM), SSF57196 (SUPERFAMILY) |
| 398 | lib_dib_Filter15_IDBA_contig_13148_plus3 | 51130 | muscle m-line assembly protein unc-89 | 1123 | 0 | 53.70% | 4 | F:nucleotide binding; P:cellular protein modification process; F:protein binding; F:protein kinase activity | IPR000719 (PFAM); IPR002290 (SMART); IPR003598 (SMART); IPR003961 (PFAM); IPR007110 (PROFILE); IPR011009 (SUPERFAMILY); IPR013098 (PFAM); IPR013783 (G3DSA:2.60.40.GENE3D); IPR020635 (SMART); IPR020675 (PANTHER); G3DSA:1.10.510.10 (GENE3D), G3DSA:3.30.200.20 (GENE3D), PTHR22964:SF6 (PANTHER), SignalP-NN(euk) (SIGNALP), tmhmm (TMHMM), SSF48726 (SUPERFAMILY) |

| | | | | | | | | | | |
|---|---|---|---|---|---|---|---|---|---|---|
| 399 | lib_dib_Filter15_IDBA_contig_13149_plus1 | 102577 | | 3283 | 0 | 100% | 8 | F:enzyme regulator activity; C:intracellular; P:signal transduction; F:nucleotide binding; P:cellular protein modification process; F:protein binding; F:lipid binding; F:protein kinase activity | IPR000219 (G3DSA:1.20.900.GENE3D); IPR000719 (PFAM); IPR001452 (SUPERFAMILY); IPR001849 (SMART); IPR002290 (SMART); IPR003598 (SMART); IPR003599 (SMART); IPR003961 (PFAM); IPR007110 (PROFILE); IPR011009 (SUPERFAMILY); IPR011993 (G3DSA:2.30.29.GENE3D); IPR013098 (PFAM); IPR013783 (G3DSA:2.60.40.GENE3D); IPR020635 (SMART); IPR020675 (PANTHER); G3DSA:1.10.510.10 (GENE3D), G3DSA:2.30.30.40 (GENE3D), G3DSA:3.30.200.20 (GENE3D), SSF48726 (SUPERFAMILY), SSF50729 (SUPERFAMILY) | |
| 400 | lib_dib_Filter15_IDBA_contig_13162_plus1 | 31222 | | 2192 | 0 | 100% | 1 | F:protein binding | IPR003598 (SMART); IPR003599 (SMART); IPR003961 (PFAM); IPR007110 (PROFILE); IPR013098 (PFAM); IPR013783 (G3DSA:2.60.40.GENE3D); PR00014 (PRINTS), SSF48726 (SUPERFAMILY) | |
| 401 | lib_dib_Filter15_IDBA_contig_13163_plus2 | 14355 | | 761 | 0 | 100% | 1 | F:protein binding | IPR003598 (SMART); IPR003599 (SMART); IPR003961 (PFAM); IPR007110 (PROFILE); IPR013098 (PFAM); IPR013783 (G3DSA:2.60.40.GENE3D); PR00014 (PRINTS), SSF48726 (SUPERFAMILY) | |
| 402 | lib_dib_Filter15_IDBA_contig_13164_plus2 | 10775 | | 585 | 0 | 100% | 1 | F:protein binding | IPR003599 (SMART); IPR003961 (PFAM); IPR007110 (PROFILE); IPR013098 (PFAM); IPR013783 (G3DSA:2.60.40.GENE3D); PR00014 (PRINTS), SSF48726 (SUPERFAMILY) | |
| 403 | lib_dib_Filter15_IDBA_contig_13169_plus1 | 693 | sidekick 1-like | 1829 | 0 | 43.10% | 1 | F:protein binding | IPR003598 (SMART); IPR003599 (SMART); IPR003961 (PFAM); IPR007110 (PROFILE); IPR013098 (PFAM); IPR013783 (G3DSA:2.60.40.GENE3D); PR00014 (PRINTS), PTHR10489 (PANTHER), PF13895 (PFAM), tmhmm (TMHMM), SSF48726 (SUPERFAMILY) | |
| 404 | lib_dib_Filter15_IDBA_contig_13173_minus3 | 411 | transient receptor potential cation channel subfamily a member 1 homolog | 1239 | 0 | 56.55% | 1 | F:protein binding | IPR002110 (PRINTS); IPR020683 (G3DSA:1.25.40.GENE3D); PTHR24165 (PANTHER), PTHR24165:SF2 (PANTHER), tmhmm (TMHMM) | |
| 405 | lib_dib_Filter15_IDBA_contig_13194_minus1 | 497 | serine threonine-protein phosphatase 6 regulatory ankyrin repeat subunit a-like | 1223 | 3.43E-108 | 45.35% | 1 | F:protein binding | IPR001496 (SMART); IPR002110 (PRINTS); IPR020683 (G3DSA:1.25.40.GENE3D); PTHR24158 (PANTHER), PTHR24158:SF0 (PANTHER), PF13637 (PFAM) | |
| 406 | lib_dib_Filter15_IDBA_contig_13197_plus3 | 393 | modular serine protease | 1216 | 7.14E-11 | 41.20% | 3 | P:biological_process; P:regulation of biological process; F:protein binding | IPR002172 (G3DSA:4.10.400.GENE3D); IPR023415 (PROSITE); PR00261 (PRINTS), PTHR24652 (PANTHER), tmhmm (TMHMM) | |
| 407 | lib_dib_Filter15_IDBA_contig_13212_plus3 | 6189 | granulin- partial | 1844 | 4.85E-59 | 56.05% | 3 | F:protein binding; C:organelle; C:intracellular | IPR000118 (PFAM); IPR009030 (SUPERFAMILY); PTHR12274 (PANTHER), PTHR12274:SF0 (PANTHER), SignalP-NN(euk) (SIGNALP), tmhmm (TMHMM), SSF57277 (SUPERFAMILY) | Yes |
| 408 | lib_dib_Filter15_IDBA_contig_13213_plus1 | 20 | granulin- partial | 114 | 7.35E-29 | 60.90% | 4 | P:anatomical structure morphogenesis; P:cellular component organization; P:cell differentiation; P:multicellular organismal development | IPR000118 (PFAM); PTHR12274 (PANTHER), PTHR12274:SF0 (PANTHER), SSF57277 (SUPERFAMILY) | |
| 409 | lib_dib_Filter15_IDBA_contig_13215_minus1 | 2202 | ---NA--- | 2574 | | | 0 | - | no IPS match | |
| 410 | lib_dib_Filter15_IDBA_contig_13215_plus1 | 2202 | collagen alpha chain cg42342-like | 2574 | 4.43E-16 | 58.35% | 4 | F:molecular_function; P:biological_process; C:cellular_component; C:collagen | IPR008160 (PFAM); PTHR24023 (PANTHER), PTHR24023:SF3 (PANTHER), SignalP-NN(euk) (SIGNALP), tmhmm (TMHMM) | Yes |

| | | | | | | | | | | |
|---|---|---|---|---|---|---|---|---|---|---|
| 411 | lib_dib_Filter15_IDBA_contig_13217_plus3 | 29 | ---NA--- | 207 | | | 0 | - | IPR008160 (PFAM); PTHR24023 (PANTHER) | |
| 412 | lib_dib_Filter15_IDBA_contig_13232_minus1 | 531 | kinase suppressor of ras 2-like | 1197 | 0 | 69.75% | 3 | F:nucleotide binding; P:cellular protein modification process; F:protein kinase activity | IPR000719 (PROFILE); IPR001245 (PFAM); IPR002219 (PFAM); IPR002290 (SMART); IPR008271 (PROSITE); IPR011009 (SUPERFAMILY); IPR017441 (PROSITE); IPR020635 (SMART); IPR025561 (PFAM); G3DSA:1.10.510.10 (GENE3D), G3DSA:3.30.200.20 (GENE3D), G3DSA:3.30.60.20 (GENE3D), PTHR23257 (PANTHER), PTHR23257:SF90 (PANTHER), SSF57889 (SUPERFAMILY) | |
| 413 | lib_dib_Filter15_IDBA_contig_13242_plus3 | 519 | carboxypeptidase b-like | 1711 | 4.44E-62 | 55.10% | 4 | F:peptidase activity; P:protein metabolic process; P:catabolic process; F:binding | IPR000834 (PFAM); IPR003146 (G3DSA:3.30.70.GENE3D); IPR009020 (SUPERFAMILY); G3DSA:3.40.630.10 (GENE3D), PTHR11705 (PANTHER), SSF53187 (SUPERFAMILY) | |
| 414 | lib_dib_Filter15_IDBA_contig_13245_minus3 | 4511 | mitogen-activated protein kinase kinase kinase 13-like | 1191 | 0 | 82.10% | 15 | C:cytosol; C:protein complex; P:cellular protein modification process; P:signal transduction; F:protein binding; C:cell; P:response to stress; F:binding; F:protein kinase activity; F:signal transducer activity; P:organelle organization; P:regulation of biological process; ; C:plasma membrane; F:nucleotide binding | IPR000719 (PROFILE); IPR001245 (PRINTS); IPR002290 (SMART); IPR008271 (PROSITE); IPR011009 (SUPERFAMILY); IPR020635 (SMART); G3DSA:1.10.510.10 (GENE3D), G3DSA:3.30.200.20 (GENE3D), PTHR23257 (PANTHER), PTHR23257:SF85 (PANTHER) | |
| 415 | lib_dib_Filter15_IDBA_contig_13247_minus2 | 825 | adenosine monophosphate-protein transferase ficd | 1503 | 6.15E-158 | 74.00% | 1 | F:protein binding | IPR003812 (PFAM); IPR011990 (G3DSA:1.25.40.GENE3D); IPR013026 (PROFILE); IPR019734 (PROFILE); G3DSA:1.10.3290.10 (GENE3D), PTHR13504 (PANTHER), PTHR13504:SF12 (PANTHER), PF13414 (PFAM), tmhmm (TMHMM), SSF48452 (SUPERFAMILY) | |
| 416 | lib_dib_Filter15_IDBA_contig_13248_minus3 | 1449 | von willebrand factor a domain-containing protein 5a-like | 1189 | 9.11E-118 | 52.80% | 1 | F:protein binding | IPR002035 (SMART); IPR013694 (PFAM); G3DSA:3.40.50.410 (GENE3D), PTHR10338 (PANTHER), PTHR10338:SF39 (PANTHER), PF13768 (PFAM), SignalP-NN(euk) (SIGNALP), SSF53300 (SUPERFAMILY) | Yes |
| 417 | lib_dib_Filter15_IDBA_contig_13253_minus2 | 406 | ankrd12 partial | 1279 | 2.42E-68 | 65.35% | 8 | C:cytoplasm; C:nucleolus; P:anatomical structure morphogenesis; P:growth; P:multicellular organismal development; P:embryo development; P:biological_process; F:protein binding | IPR002110 (PFAM); IPR020683 (G3DSA:1.25.40.GENE3D); PTHR24149 (PANTHER), PTHR24149:SF0 (PANTHER) | |
| 418 | lib_dib_Filter15_IDBA_contig_13261_plus2 | 608 | neuronal cell adhesion | 1186 | 2.27E-96 | 44.55% | 1 | F:protein binding | IPR003598 (SMART); IPR003599 (SMART); IPR007110 (PROFILE); IPR013098 (PFAM); IPR013783 (G3DSA:2.60.40.GENE3D); IPR026966 (PFAM); PTHR10489 (PANTHER), PF13895 (PFAM), tmhmm (TMHMM), SSF48726 (SUPERFAMILY) | |
| 419 | lib_dib_Filter15_IDBA_contig_13273_plus1 | 2200 | neurexin isoform f | 1725 | 0 | 54.45% | 2 | P:biological_process; P:cellular component organization | IPR001791 (PFAM); IPR008985 (SUPERFAMILY); IPR013320 (G3DSA:2.60.120.GENE3D); PTHR10574 (PANTHER), PTHR10574:SF86 (PANTHER), PF13385 (PFAM), tmhmm (TMHMM) | |
| 420 | lib_dib_Filter15_IDBA_contig_13275_plus1 | 452 | neurexin-3-alpha isoform 3 | 240 | 1.90E-70 | 53.20% | 6 | C:cell; F:protein binding; F:receptor activity; P:cell-cell signaling; P:cellular component organization; P:multicellular organismal development | IPR000152 (PROSITE); IPR000742 (PFAM); IPR001791 (PFAM); IPR008985 (SUPERFAMILY); IPR013320 (G3DSA:2.60.120.GENE3D); IPR027149 (PTHR10127:PANTHER); G3DSA:2.10.25.10 (GENE3D), PTHR10127 (PANTHER), SSF57196 (SUPERFAMILY) | |

| | | | | | | | | | | |
|---|---|---|---|---|---|---|---|---|---|---|
| 421 | lib_dib_Filter15_IDBA_contig_13283_plus3 | 330 | protein | 1179 | 6.70E-61 | 56.15% | 3 | C:extracellular region; F:protein binding; F:calcium ion binding | IPR000152 (PROSITE); IPR000742 (PFAM); IPR001881 (SMART); IPR013032 (PFAM); IPR018097 (PROSITE); PR00010 (PRINTS), G3DSA:2.10.25.10 (GENE3D), PTHR24044 (PANTHER), SignalP-NN(euk) (SIGNALP), tmhmm (TMHMM), SSF57196 (SUPERFAMILY) | Yes |
| 422 | lib_dib_Filter15_IDBA_contig_13327_minus2 | 2149 | n protein | 1210 | 3.21E-110 | 68.65% | 16 | C:nucleoplasm; P:regulation of biological process; C:intracellular; C:protein complex; ; P:signal transduction; P:response to stress; P:response to biotic stimulus; C:nucleolus; C:cytosol; P:cell death; F:protein binding; F:transcription regulator activity; P:biological_process; F:sequence-specific DNA binding transcription factor activity; F:DNA binding | IPR000451 (PRINTS); IPR000488 (PFAM); IPR002110 (PRINTS); IPR002909 (SMART); IPR008967 (SUPERFAMILY); IPR011029 (G3DSA:1.10.533.GENE3D); IPR011539 (G3DSA:2.60.40.GENE3D); IPR013783 (G3DSA:2.60.40.GENE3D); IPR014756 (SUPERFAMILY); IPR020683 (G3DSA:1.25.40.GENE3D); PTHR24169 (PANTHER), PTHR24169:SF5 (PANTHER), PF13857 (PFAM), tmhmm (TMHMM) | |
| 423 | lib_dib_Filter15_IDBA_contig_13332_plus1 | 310 | platelet-activating factor acetylhydrolase | 1163 | 5.41E-133 | 57.75% | 3 | F:hydrolase activity; P:catabolic process; P:lipid metabolic process | IPR005065 (PANTHER); G3DSA:3.40.50.1820 (GENE3D), tmhmm (TMHMM), SSF53474 (SUPERFAMILY) | |
| 424 | lib_dib_Filter15_IDBA_contig_13339_minus3 | 297 | mitogen-activated protein kinase kinase kinase kinase 5 | 1161 | 0 | 79.15% | 7 | F:enzyme regulator activity; F:protein binding; P:signal transduction; P:response to stress; P:cellular protein modification process; F:nucleotide binding; F:protein kinase activity | IPR000719 (PFAM); IPR001180 (PFAM); IPR002290 (SMART); IPR011009 (SUPERFAMILY); IPR017441 (PROSITE); IPR020635 (SMART); G3DSA:1.10.510.10 (GENE3D), G3DSA:3.30.200.20 (GENE3D), PTHR24361 (PANTHER), PTHR24361:SF86 (PANTHER) | |
| 425 | lib_dib_Filter15_IDBA_contig_13349_plus2 | 2038 | low density lipoprotein receptor-related protein 6 | 2852 | 5.66E-93 | 46.45% | 15 | P:anatomical structure morphogenesis; P:embryo development; P:cell differentiation; P:multicellular organismal development; P:regulation of biological process; P:cellular protein modification process; P:biological_process; P:cell proliferation; F:protein binding; P:signal transduction; C:organelle; C:intracellular; C:cytoplasm; C:cell; | IPR000033 (SMART); IPR003410 (PROFILE); IPR009030 (SUPERFAMILY); IPR011042 (G3DSA:2.120.10.GENE3D); IPR011641 (PFAM); PR01217 (PRINTS), G3DSA:2.10.25.10 (GENE3D), PTHR10529 (PANTHER), tmhmm (TMHMM), SSF57196 (SUPERFAMILY), SSF63825 (SUPERFAMILY) | |
| 426 | lib_dib_Filter15_IDBA_contig_13352_plus1 | 460 | e3 ubiquitin-protein ligase mib1 | 1156 | 0 | 85.95% | 19 | P:signal transduction; P:anatomical structure morphogenesis; P:embryo development; P:cell differentiation; P:regulation of biological process; P:multicellular organismal development; F:protein binding; F:binding; C:nuclear envelope; C:cytosol; F:catalytic activity; P:cellular protein modification process; C:microtubule organizing center; C:organelle; C:cytoplasm; C:cytoskeleton; P:transport; P:cellular component organization; C:plasma membrane | IPR000433 (PFAM); IPR001841 (SMART); IPR002110 (PRINTS); IPR010606 (PFAM); IPR013083 (G3DSA:3.30.40.GENE3D); IPR020683 (G3DSA:1.25.40.GENE3D); PTHR24202 (PANTHER), PTHR24202:SF0 (PANTHER), PF13920 (PFAM), SignalP-NN(euk) (SIGNALP), SSF57850 (SUPERFAMILY) | Yes |
| 427 | lib_dib_Filter15_IDBA_contig_13354_minus1 | 640 | collagen alpha-5 chain | 1322 | 7.60E-22 | 53.38% | 7 | F:extracellular matrix structural constituent; C:collagen; C:neuromuscular junction; P:neuromuscular junction development; C:basal lamina; P:cell adhesion; C:collagen type IV | IPR008160 (PFAM); PTHR24023 (PANTHER) | |
| 428 | lib_dib_Filter15_IDBA_contig_13354_plus1 | 640 | cell surface | 1322 | 2.90E-05 | 38.00% | 0 | - | no IPS match | |

| | | | | | | | | | | |
|---|---|---|---|---|---|---|---|---|---|---|
| 429 | lib_dib_Filter15_IDBA_contig_13355_minus1 | 1118 | col4a1 protein | 2019 | 1.23E-127 | 78.95% | 9 | P:anatomical structure morphogenesis; P:cellular component organization; P:cell differentiation; P:multicellular organismal development; C:proteinaceous extracellular matrix; F:protein binding; C:cellular_component; P:response to endogenous stimulus; F:structural molecule activity | IPR001442 (G3DSA:2.170.240.GENE3D); IPR008160 (PFAM); IPR016187 (SUPERFAMILY); PTHR24023 (PANTHER) | |
| 430 | lib_dib_Filter15_IDBA_contig_13355_plus3 | 1118 | cell surface | 2018 | 8.58E-07 | 36.17% | 4 | F:structural constituent of cuticle; C:cell wall; C:membrane; C:extracellular region | no IPS match | |
| 431 | lib_dib_Filter15_IDBA_contig_13361_plus2 | 860 | insulin receptor | 2054 | 0 | 59.30% | 24 | P:signal transduction; C:cell; P:biological_process; P:cellular protein modification process; P:metabolic process; P:regulation of biological process; P:response to endogenous stimulus; F:protein binding; C:cytosol; P:multicellular organismal development; C:plasma membrane; F:binding; P:cell differentiation; P:cellular component organization; F:receptor activity; F:protein kinase activity; C:cellular_component; P:biosynthetic process; P:protein metabolic process; P:response to external stimulus; P:transport; C:nucleus; C:endosome; F:nucleotide binding | IPR000494 (PFAM); IPR000719 (PROFILE); IPR001245 (PRINTS); IPR002290 (SMART); IPR003961 (PFAM); IPR006211 (PFAM); IPR006212 (SMART); IPR008266 (PROSITE); IPR009030 (SUPERFAMILY); IPR011009 (SUPERFAMILY); IPR013783 (G3DSA:2.60.40.GENE3D); IPR017441 (PROSITE); IPR020635 (SMART); G3DSA:1.10.510.10 (GENE3D), G3DSA:2.10.220.10 (GENE3D), G3DSA:3.30.200.20 (GENE3D), G3DSA:3.80.20.20 (GENE3D), PTHR24416 (PANTHER), PTHR24416:SF140 (PANTHER), SSF52058 (SUPERFAMILY) | |
| 432 | lib_dib_Filter15_IDBA_contig_13363_minus3 | 898 | accumulation associated protein | 1843 | 4.77E-06 | 41.50% | 3 | C:cell wall; C:membrane; C:extracellular region | PTHR24023 (PANTHER), PTHR24023:SF80 (PANTHER) | |
| 433 | lib_dib_Filter15_IDBA_contig_13363_plus3 | 898 | collagen alpha-1 chain-like | 1843 | 9.37E-59 | 60.20% | 4 | P:anatomical structure morphogenesis; P:multicellular organismal development; P:cell differentiation; C:proteinaceous extracellular matrix | IPR001791 (SMART); IPR008160 (PFAM); IPR008985 (SUPERFAMILY); PTHR24023 (PANTHER), tmhmm (TMHMM) | |
| 434 | lib_dib_Filter15_IDBA_contig_13364_minus2 | 58311 | a chain solution structure of component iv glycera dibranchiata monomeric hemoglobin-co | 405 | 4.94E-51 | 57.00% | 3 | F:binding; P:transport; F:oxygen binding | IPR000971 (PFAM); IPR009050 (SUPERFAMILY); IPR012292 (G3DSA:1.10.490.GENE3D); IPR013316 (PRINTS); PTHR22924 (PANTHER) | |
| 435 | lib_dib_Filter15_IDBA_contig_13365_minus2 | 59392 | a chain solution structure of component iv glycera dibranchiata monomeric hemoglobin-co | 417 | 9.80E-51 | 56.95% | 3 | F:binding; P:transport; F:oxygen binding | IPR000971 (PFAM); IPR009050 (SUPERFAMILY); IPR012292 (G3DSA:1.10.490.GENE3D); IPR013316 (PRINTS); PTHR22924 (PANTHER) | |
| 436 | lib_dib_Filter15_IDBA_contig_13371_plus2 | 672 | partial | 1311 | 1.62E-62 | 53.75% | 5 | F:protein binding; P:signal transduction; F:nucleotide binding; P:cellular protein modification process; F:protein kinase activity | IPR000488 (PFAM); IPR000719 (PFAM); IPR002290 (SMART); IPR008271 (PROSITE); IPR011009 (SUPERFAMILY); IPR011029 (G3DSA:1.10.533.GENE3D); IPR017441 (PROSITE); IPR020635 (SMART); G3DSA:1.10.510.10 (GENE3D), G3DSA:3.30.200.20 (GENE3D), PTHR24419 (PANTHER), SignalP-NN(euk) (SIGNALP) | |
| 437 | lib_dib_Filter15_IDBA_contig_13382_plus3 | 4240 | growth arrest-specific protein 6-like | 1699 | 3.72E-41 | 50.60% | 4 | F:protein binding; F:calcium ion binding; F:structural molecule activity; C:proteinaceous extracellular matrix | IPR000152 (PROSITE); IPR000436 (SUPERFAMILY); IPR000742 (SMART); IPR001881 (PFAM); IPR002889 (PFAM); IPR011398 (PTHR24039:PANTHER); IPR013994 (SMART); IPR018097 (PROSITE); G3DSA:2.10.25.10 (GENE3D), PTHR24039 (PANTHER), SignalP-NN(euk) (SIGNALP), tmhmm (TMHMM), SSF57196 (SUPERFAMILY) | Yes |

| | | | | | | | | | | |
|---|---|---|---|---|---|---|---|---|---|---|
| 438 | lib_dib_Filter15_IDBA_contig_13395_minus3 | 3698 | map microtubule affinity-regulating kinase 3 isoform 1 | 2583 | 0 | 69.45% | 3 | F:nucleotide binding; P:cellular protein modification process; F:protein kinase activity | IPR000719 (PFAM); IPR001772 (PFAM); IPR002290 (SMART); IPR008271 (PROSITE); IPR011009 (SUPERFAMILY); IPR015940 (SMART); IPR017441 (PROSITE); IPR020635 (SMART); G3DSA:1.10.510.10 (GENE3D), G3DSA:1.10.8.10 (GENE3D), G3DSA:3.30.200.20 (GENE3D), G3DSA:3.30.310.80 (GENE3D), PTHR24346 (PANTHER), PTHR24346:SF0 (PANTHER), tmhmm (TMHMM) | |
| 439 | lib_dib_Filter15_IDBA_contig_1341_plus1 | 14095 | 78 kda glucose-regulated protein | 1018 | 0 | 92.50% | 31 | C:cell; C:endoplasmic reticulum; C:protein complex; F:protein binding; P:signal transduction; P:anatomical structure morphogenesis; P:multicellular organismal development; P:regulation of biological process; P:embryo development; P:nucleobase-containing compound metabolic process; P:catabolic process; P:response to external stimulus; P:response to stress; P:cellular protein modification process; F:enzyme regulator activity; P:protein metabolic process; P:cell death; P:response to biotic stimulus; C:nucleus; C:cytosol; P:transport; C:cytoplasm; F:binding; P:cell communication; P:metabolic process; F:nucleotide binding; F:hydrolase activity; C:cytoplasmic membrane-bounded vesicle; F:calcium ion binding; F:catalytic activity; C:extracellular | IPR013126 (PRINTS); IPR018181 (PROSITE); G3DSA:1.20.1270.10 (GENE3D), G3DSA:2.60.34.10 (GENE3D), G3DSA:3.30.30.30 (GENE3D), G3DSA:3.30.420.40 (GENE3D), G3DSA:3.90.640.10 (GENE3D), PTHR19375 (PANTHER), SignalP-NN(euk) (SIGNALP), tmhmm (TMHMM), SSF100920 (SUPERFAMILY), SSF100934 (SUPERFAMILY), SSF53067 (SUPERFAMILY) | Yes |
| 440 | lib_dib_Filter15_IDBA_contig_13410_minus2 | 2660 | aldehyde mitochondrial-like isoform 1 | 1126 | 0 | 81.85% | 26 | P:anatomical structure morphogenesis; P:multicellular organismal development; P:biological_process; P:response to biotic stimulus; P:metabolic process; P:regulation of biological process; F:catalytic activity; P:embryo development; P:signal transduction; C:mitochondrion; P:cell differentiation; P:response to endogenous stimulus; P:lipid metabolic process; P:secondary metabolic process; P:response to stress; C:cytosol; P:cell proliferation; P:catabolic process; P:response to external stimulus; P:cell communication; P:biosynthetic process; F:lipid binding; F:protein binding; P:cell death; C:cytoplasm; C:nucleus | IPR015590 (PFAM); IPR016160 (PROSITE); IPR016161 (SUPERFAMILY); IPR016162 (G3DSA:3.40.605.GENE3D); IPR016163 (G3DSA:3.40.309.GENE3D); PTHR11699 (PANTHER), PTHR11699:SF46 (PANTHER), tmhmm (TMHMM) | |
| 441 | lib_dib_Filter15_IDBA_contig_13411_plus2 | 4313 | splicing factor 3b subunit 1 | 1801 | 0 | 85.50% | 6 | F:chromatin binding; F:protein binding; C:nucleoplasm; C:nucleus; P:multicellular organismal development; P:nucleobase-containing compound metabolic process | IPR011989 (G3DSA:1.25.10.GENE3D); IPR015016 (PFAM); IPR016024 (SUPERFAMILY); PTHR12097 (PANTHER), tmhmm (TMHMM) | |
| 442 | lib_dib_Filter15_IDBA_contig_13412_plus2 | 4547 | splicing factor 3b subunit 1 | 2004 | 0 | 85.55% | 6 | F:chromatin binding; F:protein binding; C:nucleoplasm; C:nucleus; P:multicellular organismal development; P:nucleobase-containing compound metabolic process | IPR011989 (G3DSA:1.25.10.GENE3D); IPR015016 (PFAM); IPR016024 (SUPERFAMILY); PTHR12097 (PANTHER), tmhmm (TMHMM) | |

| | | | | | | | | |
|---|---|---|---|---|---|---|---|---|
| 443 | lib_dib_Filter15_IDBA_contig_13439_minus2 | 2179 | ribosomal protein s6 kinase alpha-5 | 1271 | 0 | 79.20% | 15 | P:anatomical structure morphogenesis; P:cellular component organization; P:cell differentiation; P:multicellular organismal development; C:nucleoplasm; P:signal transduction; F:protein binding; P:organelle organization; P:cellular protein modification process; P:regulation of biological process; F:protein kinase activity; C:ribosome; P:response to stress; ; F:nucleotide binding | IPR000719 (PFAM); IPR000961 (SMART); IPR002290 (SMART); IPR008271 (PROSITE); IPR011009 (SUPERFAMILY); IPR017441 (PROSITE); IPR017892 (PFAM); IPR020635 (SMART); G3DSA:1.10.510.10 (GENE3D), G3DSA:3.30.200.20 (GENE3D), PTHR24351 (PANTHER), PTHR24351:SF42 (PANTHER) |
| 444 | lib_dib_Filter15_IDBA_contig_13456_plus2 | 425 | sema- isoform i | 1347 | 0 | 59.80% | 10 | P:behavior; P:cellular component organization; P:multicellular organismal development; P:response to external stimulus; P:anatomical structure morphogenesis; F:protein binding; P:cell differentiation; P:cell recognition; C:plasma membrane; F:receptor activity | IPR001627 (PFAM); IPR002165 (PFAM); IPR003659 (SMART); IPR015943 (G3DSA:2.130.10.GENE3D); IPR016201 (SUPERFAMILY); IPR027231 (PANTHER); G3DSA:3.30.1680.10 (GENE3D), PTHR11036:SF29 (PANTHER), tmhmm (TMHMM) |
| 445 | lib_dib_Filter15_IDBA_contig_13481_minus1 | 957 | isoform f | 1828 | 8.68E-78 | 84.35% | 12 | P:anatomical structure morphogenesis; P:cellular component organization; P:cell differentiation; P:multicellular organismal development; ; F:nucleotide binding; P:metabolic process; P:regulation of biological process; P:reproduction; P:behavior; F:protein binding; C:nucleus | IPR001478 (PFAM); IPR001660 (SMART); IPR013761 (G3DSA:1.10.150.GENE3D); IPR021129 (PFAM); G3DSA:2.30.42.10 (GENE3D), PTHR16154 (PANTHER), SignalP-NN(euk) (SIGNALP), SSF46966 (SUPERFAMILY) |
| 446 | lib_dib_Filter15_IDBA_contig_13531_plus3 | 226 | mitochondrial 10-formyltetrahydrofolate dehydrogenase | 1108 | 0 | 82.00% | 7 | F:catalytic activity; P:biosynthetic process; F:transferase activity; P:metabolic process; P:catabolic process; F:binding; C:mitochondrion | IPR002376 (G3DSA:3.40.50.GENE3D); IPR005793 (G3DSA:3.10.25.GENE3D); IPR009081 (G3DSA:1.10.1200.GENE3D); IPR011034 (SUPERFAMILY); IPR015590 (PFAM); IPR016160 (PROSITE); IPR016161 (SUPERFAMILY); IPR016162 (G3DSA:3.40.605.GENE3D); IPR016163 (G3DSA:3.40.309.GENE3D); PTHR11699 (PANTHER), PTHR11699:SF46 (PANTHER), tmhmm (TMHMM) |
| 447 | lib_dib_Filter15_IDBA_contig_13534_minus2 | 194 | cadherin- isoform d | 1088 | 0 | 46.45% | 8 | P:anatomical structure morphogenesis; P:cellular component organization; P:cell differentiation; P:multicellular organismal development; P:regulation of biological process; F:calcium ion binding; P:biological_process; C:plasma membrane | IPR002126 (PRINTS); IPR015919 (SUPERFAMILY); IPR020894 (PROSITE); PTHR24027 (PANTHER) |
| 448 | lib_dib_Filter15_IDBA_contig_13536_minus1 | 3223 | serine threonine-protein kinase pim-3 | 1330 | 2.65E-117 | 78.85% | 8 | P:cellular protein modification process; F:protein kinase activity; F:nucleotide binding; F:protein binding; C:cytoplasm; P:regulation of biological process; P:cell death; P:cell cycle | IPR000719 (PFAM); IPR002290 (SMART); IPR008271 (PROSITE); IPR011009 (SUPERFAMILY); IPR017441 (PROSITE); IPR020635 (SMART); G3DSA:1.10.510.10 (GENE3D), G3DSA:3.30.200.20 (GENE3D), PTHR22984 (PANTHER), PTHR22984:SF0 (PANTHER), tmhmm (TMHMM) |
| 449 | lib_dib_Filter15_IDBA_contig_13539_plus1 | 5649 | hypothetical protein CAPTEDRAFT_212422 | 142 | 1.83E-26 | 48.05% | 2 | F:carbohydrate binding; F:calcium ion binding | IPR000436 (PFAM); G3DSA:2.10.70.10 (GENE3D), PTHR19325 (PANTHER) |
| 450 | lib_dib_Filter15_IDBA_contig_13541_plus2 | 2785 | von willebrand factor type egf and pentraxin domain-containing protein 1 | 108 | 2.27E-08 | 47.90% | 5 | F:calcium ion binding; C:cytoplasm; P:cell adhesion; C:membrane; C:extracellular region | IPR009030 (SUPERFAMILY); IPR011641 (PFAM); G3DSA:2.10.50.10 (GENE3D), PTHR24046 (PANTHER) |
| 451 | lib_dib_Filter15_IDBA_contig_13556_minus1 | 895 | striatin-3 isoform 1 | 1541 | 0 | 71.95% | 6 | ; P:regulation of biological process; F:protein binding; F:sequence-specific DNA binding transcription factor activity; C:nucleoplasm; C:cytoplasm | IPR001680 (PFAM); IPR013258 (PFAM); IPR015943 (G3DSA:2.130.10.GENE3D); IPR017986 (PROFILE); IPR019775 (PROSITE); IPR020472 (PRINTS); PTHR15653 (PANTHER), PTHR15653:SF0 (PANTHER) |

| | | | | | | | | | | |
|---|---|---|---|---|---|---|---|---|---|---|
| 452 | lib_dib_Filter15_IDBA_contig_13558_minus3 | 3470 | calmodulin 2-like | 2229 | 1.12E-119 | 52.25% | 1 | F:calcium ion binding | IPR002048 (SMART); IPR011992 (G3DSA:1.10.238.GENE3D); IPR018247 (PROSITE); PTHR10891 (PANTHER), PF13405 (PFAM), PF13833 (PFAM), tmhmm (TMHMM), SSF47473 (SUPERFAMILY) | |
| 453 | lib_dib_Filter15_IDBA_contig_13576_minus3 | 1431 | cd109 antigen | 1802 | 0 | 54.40% | 3 | F:enzyme regulator activity; F:protein binding; C:extracellular space | IPR001599 (PFAM); IPR002172 (G3DSA:4.10.400.GENE3D); IPR002890 (PFAM); IPR008930 (SUPERFAMILY); IPR009048 (G3DSA:2.60.40.GENE3D); IPR011625 (PFAM); IPR011626 (PFAM); G3DSA:1.50.10.20 (GENE3D), PTHR11412 (PANTHER), PTHR11412:SF22 (PANTHER), SignalP-NN(euk) (SIGNALP) | Yes |
| 454 | lib_dib_Filter15_IDBA_contig_13577_minus3 | 1639 | cd109 antigen | 2011 | 0 | 54.40% | 3 | F:enzyme regulator activity; F:protein binding; C:extracellular space | IPR001599 (PFAM); IPR002172 (G3DSA:4.10.400.GENE3D); IPR002890 (PFAM); IPR008930 (SUPERFAMILY); IPR009048 (G3DSA:2.60.40.GENE3D); IPR011625 (PFAM); IPR011626 (PFAM); G3DSA:1.50.10.20 (GENE3D), PTHR11412 (PANTHER), PTHR11412:SF22 (PANTHER), SignalP-NN(euk) (SIGNALP), tmhmm (TMHMM) | Yes |
| 455 | lib_dib_Filter15_IDBA_contig_13583_plus1 | 176 | tyrosine-protein kinase src42a | 1072 | 4.39E-29 | 52.90% | 2 | F:catalytic activity; F:protein binding | IPR000483 (SMART); IPR001611 (PROFILE); IPR003591 (SMART); IPR026906 (PFAM); G3DSA:3.80.10.10 (GENE3D), PTHR24373 (PANTHER), PF13855 (PFAM), SM00365 (SMART), tmhmm (TMHMM), SSF52058 (SUPERFAMILY) | |
| 456 | lib_dib_Filter15_IDBA_contig_13586_plus2 | 1032 | protein fam208a isoform 2 | 2970 | 2.38E-18 | 48.05% | 0 | - | PTHR16207 (PANTHER), PTHR16207:SF1 (PANTHER), tmhmm (TMHMM) | |
| 457 | lib_dib_Filter15_IDBA_contig_13600_plus2 | 245 | rap guanine nucleotide exchange factor 2 | 1064 | 0 | 72.50% | 4 | F:enzyme regulator activity; C:intracellular; P:signal transduction; F:protein binding | IPR000159 (PFAM); IPR000595 (PFAM); IPR000651 (PFAM); IPR001478 (PFAM); IPR001895 (G3DSA:1.10.840.GENE3D); IPR008937 (PANTHER); IPR014710 (G3DSA:2.60.120.GENE3D); IPR018490 (SUPERFAMILY); IPR023578 (SUPERFAMILY); G3DSA:1.20.870.10 (GENE3D), G3DSA:2.30.42.10 (GENE3D), PTHR23113:SF21 (PANTHER), SSF54236 (SUPERFAMILY) | |
| 458 | lib_dib_Filter15_IDBA_contig_13601_plus3 | 520 | proteinase inhibitor i4 serpin | 1063 | 1.47E-17 | 64.00% | 1 | F:enzyme regulator activity | IPR000215 (PANTHER); IPR023796 (PFAM); G3DSA:2.30.39.10 (GENE3D), G3DSA:3.30.497.10 (GENE3D), SignalP-NN(euk) (SIGNALP), tmhmm (TMHMM) | |
| 459 | lib_dib_Filter15_IDBA_contig_13602_minus1 | 386 | pzp protein | 1063 | 2.93E-48 | 44.35% | 1 | F:enzyme regulator activity | IPR002890 (PFAM); IPR011625 (PFAM); PTHR11412 (PANTHER), PTHR11412:SF36 (PANTHER), tmhmm (TMHMM) | |
| 460 | lib_dib_Filter15_IDBA_contig_13603_plus3 | 309 | ankyrin repeat protein | 1078 | 3.49E-64 | 45.75% | 1 | F:protein binding | IPR002110 (PFAM); IPR020683 (G3DSA:1.25.40.GENE3D); PTHR24158 (PANTHER), PTHR24158:SF0 (PANTHER) | |
| 461 | lib_dib_Filter15_IDBA_contig_13627_plus1 | 189 | dnaj homolog subfamily c member 10 | 1055 | 0 | 63.95% | 7 | F:protein binding; P:protein metabolic process; P:metabolic process; F:electron carrier activity; F:catalytic activity; P:regulation of biological process; P:cellular homeostasis | IPR001623 (PRINTS); IPR005746 (PRINTS); IPR012336 (G3DSA:3.40.30.GENE3D); IPR013766 (PFAM); IPR017937 (PROSITE); PTHR24078 (PANTHER), PTHR24078:SF26 (PANTHER), SignalP-NN(euk) (SIGNALP), tmhmm (TMHMM) | Yes |
| 462 | lib_dib_Filter15_IDBA_contig_13631_plus3 | 1490 | uveal autoantigen with coiled-coil domains and ankyrin repeats-like | 2055 | 3.49E-56 | 59.20% | 5 | P:cell death; P:biological_process; P:regulation of biological process; C:mitochondrion; F:protein binding | IPR002110 (PFAM); IPR009053 (SUPERFAMILY); IPR020683 (G3DSA:1.25.40.GENE3D); PTHR24129 (PANTHER), PTHR24129:SF0 (PANTHER) | |

| | | | | | | | | | | |
|---|---|---|---|---|---|---|---|---|---|---|
| 463 | lib_dib_Filter15_IDBA_contig_13672_minus2 | 449 | polyubiquitin | 1194 | 3.58E-85 | 48.20% | 11 | P:cell death; P:signal transduction; P:cellular protein modification process; C:organelle; C:intracellular; C:cytoplasm; P:biological_process; P:cell cycle; P:regulation of biological process; P:response to stress; F:protein binding | IPR000626 (PFAM); IPR019954 (PROSITE); IPR019955 (PROFILE); IPR019956 (PRINTS); G3DSA:3.10.20.90 (GENE3D), PTHR10666 (PANTHER), PTHR10666:SF9 (PANTHER), SignalP-NN(euk) (SIGNALP), SSF54236 (SUPERFAMILY) | Yes |
| 464 | lib_dib_Filter15_IDBA_contig_13673_minus2 | 437 | polyubiquitin | 1206 | 6.39E-85 | 48.20% | 11 | P:cell death; P:signal transduction; P:cellular protein modification process; C:organelle; C:intracellular; C:cytoplasm; P:biological_process; P:cell cycle; P:regulation of biological process; P:response to stress; F:protein binding | IPR000626 (PFAM); IPR019954 (PROSITE); IPR019955 (PROFILE); IPR019956 (PRINTS); G3DSA:3.10.20.90 (GENE3D), PTHR10666 (PANTHER), PTHR10666:SF9 (PANTHER), SignalP-NN(euk) (SIGNALP), SSF54236 (SUPERFAMILY) | Yes |
| 465 | lib_dib_Filter15_IDBA_contig_13716_minus3 | 1592 | tyrosine-protein kinase src42a-like isoform 1 | 1263 | 0 | 86.50% | 16 | P:signal transduction; F:protein kinase activity; P:cellular component organization; P:multicellular organismal development; P:cytoskeleton organization; P:anatomical structure morphogenesis; P:embryo development; P:biological_process; P:generation of precursor metabolites and energy; P:catabolic process; P:response to stress; P:cellular protein modification process; C:plasma membrane; P:transport; F:nucleotide binding; F:protein binding | IPR000719 (PROFILE); IPR000980 (PRINTS); IPR001245 (PRINTS); IPR001452 (PRINTS); IPR002290 (SMART); IPR008266 (PROSITE); IPR011009 (SUPERFAMILY); IPR017441 (PROSITE); IPR020635 (SMART); G3DSA:1.10.510.10 (GENE3D), G3DSA:2.30.30.40 (GENE3D), G3DSA:3.30.200.20 (GENE3D), PTHR24418 (PANTHER), PTHR24418:SF22 (PANTHER), SSF55550 (SUPERFAMILY) | |
| 466 | lib_dib_Filter15_IDBA_contig_13731_plus2 | 616 | kinase d-interacting substrate of 220 kda | 1600 | 0 | 73.85% | 10 | C:cytosol; P:signal transduction; P:cellular protein modification process; F:protein binding; P:embryo development; P:anatomical structure morphogenesis; P:cellular component organization; P:cell differentiation; P:multicellular organismal development; F:enzyme regulator activity | IPR002110 (SMART); IPR011646 (PFAM); IPR020683 (G3DSA:1.25.40.GENE3D); PTHR24116 (PANTHER), PTHR24116:SF0 (PANTHER), tmhmm (TMHMM) | |
| 467 | lib_dib_Filter15_IDBA_contig_13747_minus3 | 1760 | ---NA--- | 2410 | | | 0 | - | tmhmm (TMHMM) | |
| 468 | lib_dib_Filter15_IDBA_contig_13747_plus2 | 1760 | protein let- isoform a | 2411 | 1.56E-120 | 78.05% | 10 | P:embryo development; P:multicellular organismal development; C:proteinaceous extracellular matrix; P:regulation of biological process; P:growth; P:biological_process; P:response to endogenous stimulus; P:reproduction; P:cellular component organization; F:structural molecule activity | IPR001442 (G3DSA:2.170.240.GENE3D); IPR008160 (PFAM); IPR016187 (SUPERFAMILY); PTHR24023 (PANTHER), PTHR24023:SF80 (PANTHER), tmhmm (TMHMM) | |
| 469 | lib_dib_Filter15_IDBA_contig_13760_plus1 | 3295 | tyrosine-protein phosphatase non-receptor type 13 isoform 2 | 1011 | 4.17E-44 | 62.35% | 6 | C:cytoplasm; F:phosphoprotein phosphatase activity; C:cell; C:plasma membrane; F:protein binding; C:nucleus | IPR001478 (PFAM); G3DSA:2.30.42.10 (GENE3D), PTHR19964 (PANTHER), PTHR19964:SF7 (PANTHER) | |
| 470 | lib_dib_Filter15_IDBA_contig_13760_plus3 | 3295 | tyrosine-protein phosphatase non-receptor type 13 | 1010 | 9.45E-29 | 46.50% | 1 | F:protein binding | IPR001478 (PFAM); G3DSA:2.30.42.10 (GENE3D), PTHR19964 (PANTHER) | |
| 471 | lib_dib_Filter15_IDBA_contig_13763_plus2 | 2636 | tyrosine-protein phosphatase non-receptor type 13 | 549 | 1.21E-110 | 65.55% | 6 | C:cytoplasm; F:phosphoprotein phosphatase activity; C:cell; C:plasma membrane; F:protein binding; C:nucleus | IPR000242 (PRINTS); IPR000387 (PROFILE); IPR001478 (PFAM); IPR003595 (SMART); IPR016130 (PROSITE); G3DSA:2.30.42.10 (GENE3D), G3DSA:3.90.190.10 (GENE3D), PTHR19134 (PANTHER), PTHR19134:SF55 (PANTHER), SSF52799 (SUPERFAMILY) | |
| 472 | lib_dib_Filter15_IDBA_contig_13771_minus3 | 4035 | protein lethal giant larvae-like | 1535 | 0 | 67.75% | 2 | P:multicellular organismal development; F:protein binding | IPR000664 (PRINTS); IPR001680 (SMART); IPR013577 (PFAM); IPR013905 (PFAM); IPR015943 (G3DSA:2.130.10.GENE3D); IPR017986 (PROFILE); IPR019775 (PROSITE); PTHR10241 (PANTHER), PTHR10241:SF10 (PANTHER), tmhmm (TMHMM) | |

| | | | | | | | | | |
|---|---|---|---|---|---|---|---|---|---|
| 473 | lib_dib_Filter15_IDBA_contig_13787_minus3 | 361 | cubilin-like | 1192 | 1.68E-33 | 41.40% | 1 | C:cell | IPR000859 (G3DSA:2.60.120.GENE3D); PTHR10127 (PANTHER), PTHR10127:SF310 (PANTHER), tmhmm (TMHMM) |
| 474 | lib_dib_Filter15_IDBA_contig_13790_minus1 | 60378 | protein bicaudal d homolog 1 isoform 2 | 2357 | 0 | 61.05% | 17 | F:protein binding; C:Golgi apparatus; C:cell; P:metabolic process; P:viral reproduction; F:enzyme regulator activity; F:cytoskeletal protein binding; C:cytoskeleton; C:protein complex; C:cytosol; P:nucleobase-containing compound metabolic process; C:organelle; C:cytoplasm; P:transport; P:cellular component organization; P:regulation of biological process; P:biological_process | IPR018477 (PANTHER) |
| 475 | lib_dib_Filter15_IDBA_contig_13790_plus1 | 60378 | 14-3-3 zeta | 2357 | 8.02E-114 | 88.85% | 22 | P:signal transduction; P:anatomical structure morphogenesis; P:cell differentiation; P:multicellular organismal development; P:reproduction; P:cytoskeleton organization; P:protein metabolic process; P:behavior; P:cell proliferation; P:metabolic process; P:regulation of biological process; F:protein binding; C:extracellular region; F:enzyme regulator activity; P:biological_process; C:cytoskeleton; C:protein complex; P:embryo development; P:cell cycle; C:nucleus; P:biosynthetic process; | IPR000308 (PRINTS); IPR023409 (PROSITE); IPR023410 (G3DSA:1.20.190.GENE3D); PTHR18860:SF0 (PANTHER), tmhmm (TMHMM) |
| 476 | lib_dib_Filter15_IDBA_contig_13791_plus2 | 57375 | 14-3-3 zeta | 1461 | 2.90E-115 | 88.85% | 22 | P:signal transduction; P:anatomical structure morphogenesis; P:cell differentiation; P:multicellular organismal development; P:reproduction; P:cytoskeleton organization; P:protein metabolic process; P:behavior; P:cell proliferation; P:metabolic process; P:regulation of biological process; F:protein binding; C:extracellular region; F:enzyme regulator activity; P:biological_process; C:cytoskeleton; C:protein complex; P:embryo development; P:cell cycle; C:nucleus; P:biosynthetic process; | IPR000308 (PRINTS); IPR023409 (PROSITE); IPR023410 (G3DSA:1.20.190.GENE3D); PTHR18860:SF0 (PANTHER), tmhmm (TMHMM) |
| 477 | lib_dib_Filter15_IDBA_contig_13840_plus3 | 1307 | sodium-calcium exchanger | 1700 | 0 | 75.15% | 17 | P:ion transport; P:biological_process; F:cytoskeletal protein binding; C:cell; P:response to stress; P:embryo development; C:plasma membrane; F:transporter activity; C:cytoskeleton; C:protein complex; P:regulation of biological process; P:cellular homeostasis; C:cytoplasm; P:multicellular organismal development; P:cell differentiation; P:response to external stimulus; C:mitochondrion | IPR003644 (PFAM); IPR004836 (PRINTS); IPR004837 (PFAM); PTHR11878 (PANTHER), tmhmm (TMHMM), SSF141072 (SUPERFAMILY) |
| 478 | lib_dib_Filter15_IDBA_contig_13843_plus3 | 930 | sodium calcium exchanger 1-like | 1108 | 0 | 64.20% | 5 | P:cell communication; C:cell; F:transporter activity; P:ion transport; P:transport | IPR003644 (PFAM); IPR004836 (PRINTS); IPR004837 (PFAM); PTHR11878 (PANTHER), PTHR11878:SF4 (PANTHER), tmhmm (TMHMM), SSF141072 (SUPERFAMILY) |

| | | | | | | | | | | |
|---|---|---|---|---|---|---|---|---|---|---|
| 479 | lib_dib_Filter15_IDBA_contig_13844_plus2 | 923 | sodium calcium exchanger 1-like | 1139 | 0 | 64.20% | 5 | P:cell communication; C:cell; F:transporter activity; P:ion transport; P:transport | IPR003644 (PFAM); IPR004836 (PRINTS); IPR004837 (PFAM); PTHR11878 (PANTHER), PTHR11878:SF4 (PANTHER), tmhmm (TMHMM), SSF141072 (SUPERFAMILY) | |
| 480 | lib_dib_Filter15_IDBA_contig_13857_plus2 | 352 | cortactin-binding protein 2 | 1226 | 4.63E-81 | 50.55% | 3 | F:protein binding; F:nucleotide binding; F:hydrolase activity | IPR002110 (SMART); IPR011704 (PFAM); IPR020683 (G3DSA:1.25.40.GENE3D); G3DSA:3.40.50.300 (GENE3D), PTHR24200 (PANTHER), PTHR24200:SF0 (PANTHER), PF13637 (PFAM), SSF52540 (SUPERFAMILY) | |
| 481 | lib_dib_Filter15_IDBA_contig_13862_plus1 | 1532 | heat shock 70 kda protein 4 | 1199 | 0 | 67.85% | 17 | P:metabolic process; P:regulation of biological process; P:biological_process; F:protein binding; F:nucleotide binding; P:cell death; P:multicellular organismal development; P:anatomical structure morphogenesis; P:cellular component organization; C:nucleolus; C:cytosol; P:cellular protein modification process; P:mitochondrion organization; P:protein transport; P:response to stress; P:response to abiotic stimulus; C:lipid particle | IPR013126 (PRINTS); IPR018181 (PROSITE); G3DSA:1.20.1270.10 (GENE3D), G3DSA:2.60.34.10 (GENE3D), G3DSA:3.30.30.30 (GENE3D), G3DSA:3.30.420.40 (GENE3D), G3DSA:3.90.640.10 (GENE3D), PTHR19375 (PANTHER), PTHR19375:SF17 (PANTHER), SSF100920 (SUPERFAMILY), SSF100934 (SUPERFAMILY), SSF53067 (SUPERFAMILY) | |
| 482 | lib_dib_Filter15_IDBA_contig_13866_minus2 | 3088 | ribonuclease e | 985 | 7.44E-26 | 53.15% | 2 | C:cytoplasm; P:cellular protein modification process | IPR003302 (PFAM) | |
| 483 | lib_dib_Filter15_IDBA_contig_13873_plus2 | 235 | angiogenic factor with g patch and fha domains 1 | 983 | 4.33E-85 | 66.40% | 12 | P:anatomical structure morphogenesis; P:cell differentiation; P:multicellular organismal development; P:regulation of biological process; F:protein binding; P:cell proliferation; P:biological_process; F:binding; C:extracellular region; C:cytoplasm; P:nucleobase-containing compound metabolic process; F:nucleic acid binding | IPR000253 (G3DSA:2.60.200.GENE3D); IPR000467 (PFAM); IPR008984 (SUPERFAMILY); PTHR23106 (PANTHER), PTHR23106:SF2 (PANTHER), tmhmm (TMHMM) | |
| 484 | lib_dib_Filter15_IDBA_contig_13881_plus2 | 919 | ankyrin repeat domain-containing protein 50 | 1958 | 0 | 68.95% | 1 | F:protein binding | IPR002110 (PRINTS); IPR020683 (G3DSA:1.25.40.GENE3D); PTHR24151 (PANTHER), PTHR24151:SF0 (PANTHER), PF13637 (PFAM), SignalP-NN(euk) (SIGNALP) | Yes |
| 485 | lib_dib_Filter15_IDBA_contig_13888_plus3 | 7503 | ankyrin repeat domain-containing protein 29-like | 1694 | 2.87E-144 | 75.20% | 2 | F:hydrolase activity; F:protein binding | IPR002110 (PFAM); IPR020683 (G3DSA:1.25.40.GENE3D); PTHR24193 (PANTHER), PTHR24193:SF15 (PANTHER), SignalP-NN(euk) (SIGNALP), tmhmm (TMHMM) | |
| 486 | lib_dib_Filter15_IDBA_contig_13894_minus3 | 646 | cadherin-87a-like | 980 | 5.57E-145 | 51.00% | 3 | F:calcium ion binding; P:biological_process; C:plasma membrane | IPR002126 (PRINTS); IPR015919 (SUPERFAMILY); IPR020894 (PROSITE); PTHR24027 (PANTHER) | |
| 487 | lib_dib_Filter15_IDBA_contig_13895_minus2 | 4275 | myosin heavy non-muscle-like | 2470 | 0 | 79.30% | 22 | P:anatomical structure morphogenesis; P:cellular component organization; P:cell differentiation; P:multicellular organismal development; P:embryo development; C:cytoskeleton; C:protein complex; P:cytoskeleton organization; P:regulation of biological process; P:cell death; P:response to endogenous stimulus; C:cell; P:biological_process; P:reproduction; F:actin binding; F:cytoskeletal protein binding; C:cytoplasm; F:motor activity; P:cell recognition; F:hydrolase activity; C:plasma membrane; F:nucleotide binding | IPR000048 (PROFILE); IPR001609 (PRINTS); IPR002928 (PFAM); IPR004009 (PFAM); IPR009053 (SUPERFAMILY); PD936484 (PRODOM), G3DSA:1.20.5.340 (GENE3D), G3DSA:4.10.270.10 (GENE3D), PTHR13140 (PANTHER), PTHR13140:SF23 (PANTHER), SignalP-NN(euk) (SIGNALP), tmhmm (TMHMM), SSF52540 (SUPERFAMILY) | |

| | | | | | | | | | |
|---|---|---|---|---|---|---|---|---|---|
| 488 | lib_dib_Filter15_IDBA_contig_14078_plus2 | 328 | dual specificity testis-specific protein kinase 2 | 970 | 1.81E-130 | 60.55% | 4 | C:nucleolus; F:nucleotide binding; P:cellular protein modification process; F:protein kinase activity | IPR000719 (PROFILE); IPR001245 (PRINTS); IPR002290 (SMART); IPR008266 (PROSITE); IPR011009 (SUPERFAMILY); IPR017441 (PROSITE); IPR020635 (SMART); G3DSA:1.10.510.10 (GENE3D), G3DSA:3.30.200.20 (GENE3D), PTHR23257 (PANTHER), PTHR23257:SF95 (PANTHER) |
| 489 | lib_dib_Filter15_IDBA_contig_14086_plus1 | 149 | protein disulfide-isomerase a5 | 1135 | 2.01E-168 | 68.75% | 5 | F:catalytic activity; P:metabolic process; F:electron carrier activity; P:cellular homeostasis; P:regulation of biological process | IPR005746 (PRINTS); IPR012336 (G3DSA:3.40.30.GENE3D); IPR013766 (PFAM); IPR017937 (PROSITE); PTHR18929 (PANTHER), PTHR18929:SF32 (PANTHER) |
| 490 | lib_dib_Filter15_IDBA_contig_14087_plus1 | 13 | protein disulfide-isomerase a5-like | 161 | 2.39E-15 | 50.25% | 2 | P:cellular homeostasis; P:regulation of biological process | IPR012336 (G3DSA:3.40.30.GENE3D); IPR013766 (PFAM); PTHR18929 (PANTHER), PTHR18929:SF32 (PANTHER) |
| 491 | lib_dib_Filter15_IDBA_contig_14094_plus1 | 323 | protein tanc2 | 1228 | 0 | 71.95% | 4 | F:peptidase activity; P:protein metabolic process; P:catabolic process; F:protein binding | IPR001995 (PROFILE); IPR002110 (PFAM); IPR011990 (G3DSA:1.25.40.GENE3D); IPR013026 (PROFILE); IPR019734 (SMART); IPR020683 (G3DSA:1.25.40.GENE3D); PTHR24166 (PANTHER), PTHR24166:SF0 (PANTHER), PF13414 (PFAM), SSF48452 (SUPERFAMILY) |
| 492 | lib_dib_Filter15_IDBA_contig_14115_plus3 | 323 | a chain x-ray structure of c-met with triazolopyridazine | 1186 | 7.43E-63 | 62.90% | 12 | P:anatomical structure morphogenesis; P:cellular component organization; P:cell differentiation; P:multicellular organismal development; C:plasma membrane; F:receptor activity; F:protein kinase activity; P:signal transduction; F:protein binding; P:cell proliferation; F:nucleotide binding; P:cellular protein modification process | IPR000719 (PROFILE); IPR001245 (PRINTS); IPR002290 (SMART); IPR008266 (PROSITE); IPR011009 (SUPERFAMILY); IPR013783 (G3DSA:2.60.40.GENE3D); IPR014756 (SUPERFAMILY); IPR017441 (PROSITE); IPR020635 (SMART); G3DSA:1.10.510.10 (GENE3D), G3DSA:3.30.200.20 (GENE3D), PTHR24416 (PANTHER), tmhmm (TMHMM) |
| 493 | lib_dib_Filter15_IDBA_contig_14122_plus2 | 25771 | aminopeptidase n | 1718 | 0 | 55.80% | 4 | P:protein metabolic process; P:catabolic process; F:peptidase activity; F:binding | IPR001930 (PANTHER); IPR014782 (PRINTS); IPR024571 (PFAM); G3DSA:1.10.390.10 (GENE3D), PTHR11533:SF38 (PANTHER), tmhmm (TMHMM), SSF55486 (SUPERFAMILY), SSF63737 (SUPERFAMILY) |
| 494 | lib_dib_Filter15_IDBA_contig_14123_plus2 | 24540 | aminopeptidase n | 1775 | 0 | 55.80% | 4 | P:protein metabolic process; P:catabolic process; F:peptidase activity; F:binding | IPR001930 (PANTHER); IPR014782 (PRINTS); IPR024571 (PFAM); G3DSA:1.10.390.10 (GENE3D), PTHR11533:SF38 (PANTHER), tmhmm (TMHMM), SSF55486 (SUPERFAMILY), SSF63737 (SUPERFAMILY) |
| 495 | lib_dib_Filter15_IDBA_contig_14130_minus1 | 216 | exostosin-like 3 | 964 | 0 | 77.55% | 10 | F:transferase activity; P:anatomical structure morphogenesis; P:embryo development; P:signal transduction; P:cellular component organization; P:cell differentiation; P:multicellular organismal development; P:biosynthetic process; C:endoplasmic reticulum; P:regulation of biological process | IPR004263 (PFAM); IPR015338 (PFAM); G3DSA:3.90.550.10 (GENE3D), PTHR11062 (PANTHER), PTHR11062:SF5 (PANTHER), SSF53448 (SUPERFAMILY) |
| 496 | lib_dib_Filter15_IDBA_contig_14144_minus3 | 272 | early growth response | 962 | 6.66E-79 | 79.45% | 15 | P:multicellular organismal development; P:anatomical structure morphogenesis; P:embryo development; P:cell-cell signaling; P:metabolic process; F:binding; F:nucleic acid binding; F:catalytic activity; P:regulation of biological process; ; P:biosynthetic process; P:transport; P:cellular component organization; F:nucleotide binding; C:nucleus | IPR007087 (PROSITE); IPR013087 (G3DSA:3.30.160.GENE3D); IPR015880 (SMART); PTHR10042 (PANTHER), PTHR10042:SF5 (PANTHER), PF13465 (PFAM), SSF57667 (SUPERFAMILY) |

| | | | | | | | | | | |
|---|---|---|---|---|---|---|---|---|---|---|
| 497 | lib_dib_Filter15_IDBA_contig_14176_plus2 | 419 | cadherin- isoform d | 1295 | 0 | 53.20% | 9 | P:anatomical structure morphogenesis; P:cellular component organization; P:cell differentiation; P:multicellular organismal development; P:regulation of biological process; C:plasma membrane; F:calcium ion binding; P:biological_process; F:protein binding | IPR000233 (PFAM); IPR000742 (PFAM); IPR001791 (PFAM); IPR008985 (SUPERFAMILY); IPR013032 (PROSITE); IPR013320 (G3DSA:2.60.120.GENE3D); G3DSA:2.10.25.10 (GENE3D), G3DSA:2.170.300.10 (GENE3D), G3DSA:4.10.900.10 (GENE3D), PTHR24027 (PANTHER), tmhmm (TMHMM), SSF57196 (SUPERFAMILY) | |
| 498 | lib_dib_Filter15_IDBA_contig_14204_plus2 | 743 | neutral alpha-glucosidase ab isoform 1 | 1087 | 0 | 71.95% | 6 | F:hydrolase activity; C:organelle; C:intracellular; C:cytoplasm; P:carbohydrate metabolic process; F:carbohydrate binding | IPR000322 (PANTHER); IPR011013 (SUPERFAMILY); IPR017853 (SUPERFAMILY); IPR025887 (PFAM); PTHR22762:SF7 (PANTHER), SignalP-NN(euk) (SIGNALP), tmhmm (TMHMM), SSF51011 (SUPERFAMILY) | Yes |
| 499 | lib_dib_Filter15_IDBA_contig_14221_plus3 | 21021 | isoform f | 2004 | 0 | 54.20% | 6 | P:biological_process; F:transferase activity; P:anatomical structure morphogenesis; F:structural molecule activity; C:cytoplasm; F:protein binding | IPR003598 (SMART); IPR003599 (SMART); IPR003961 (PFAM); IPR007110 (PROFILE); IPR013098 (PFAM); IPR013783 (G3DSA:2.60.40.GENE3D); PR00014 (PRINTS), SSF48726 (SUPERFAMILY) | |
| 500 | lib_dib_Filter15_IDBA_contig_14222_plus1 | 13682 | isoform f | 1231 | 0 | 52.65% | 6 | P:biological_process; F:transferase activity; P:anatomical structure morphogenesis; F:structural molecule activity; C:cytoplasm; F:protein binding | IPR003598 (SMART); IPR003599 (SMART); IPR003961 (PFAM); IPR007110 (PROFILE); IPR013098 (PFAM); IPR013783 (G3DSA:2.60.40.GENE3D); PR00014 (PRINTS), SSF48726 (SUPERFAMILY) | |
| 501 | lib_dib_Filter15_IDBA_contig_14223_plus3 | 10500 | isoform i | 777 | 0 | 54.90% | 6 | P:biological_process; F:transferase activity; P:anatomical structure morphogenesis; F:structural molecule activity; C:cytoplasm; F:protein binding | IPR003598 (SMART); IPR003599 (SMART); IPR003961 (PFAM); IPR007110 (PROFILE); IPR013098 (PFAM); IPR013783 (G3DSA:2.60.40.GENE3D); PR00014 (PRINTS), SSF48726 (SUPERFAMILY) | |
| 502 | lib_dib_Filter15_IDBA_contig_14280_plus3 | 340 | leucine-rich repeat protein soc-2 homolog | 1026 | 0 | 81.85% | 5 | F:phosphoprotein phosphatase activity; F:protein binding; C:cytoplasm; C:protein complex; P:signal transduction | IPR001611 (PFAM); IPR003591 (SMART); IPR025875 (PFAM); IPR027036 (PTHR23155:PANTHER); G3DSA:3.80.10.10 (GENE3D), PTHR23155 (PANTHER), PF13855 (PFAM), SM00364 (SMART), SM00365 (SMART), SSF52047 (SUPERFAMILY), SSF52058 (SUPERFAMILY) | |
| 503 | lib_dib_Filter15_IDBA_contig_14281_plus3 | 333 | leucine-rich repeat protein soc-2 homolog | 992 | 0 | 81.85% | 5 | F:phosphoprotein phosphatase activity; F:protein binding; C:cytoplasm; C:protein complex; P:signal transduction | IPR001611 (PFAM); IPR003591 (SMART); IPR025875 (PFAM); IPR027036 (PTHR23155:PANTHER); G3DSA:3.80.10.10 (GENE3D), PTHR23155 (PANTHER), PF13855 (PFAM), SM00364 (SMART), SM00365 (SMART), SignalP-NN(euk) (SIGNALP), SSF52047 (SUPERFAMILY), SSF52058 (SUPERFAMILY) | Yes |
| 504 | lib_dib_Filter15_IDBA_contig_14282_plus1 | 1504 | Uncharacterized protein C7orf31 | 1778 | 2.55E-61 | 51.90% | 0 | - | PTHR31393 (PANTHER) | |
| 505 | lib_dib_Filter15_IDBA_contig_14294_plus1 | 950 | phospholipase a-2-activating protein | 1325 | 0 | 67.15% | 1 | F:protein binding | IPR001680 (PFAM); IPR013535 (PFAM); IPR015155 (PFAM); IPR015943 (G3DSA:2.130.10.GENE3D); IPR017986 (PROFILE); PTHR19849 (PANTHER), PTHR19849:SF0 (PANTHER), tmhmm (TMHMM) | |
| 506 | lib_dib_Filter15_IDBA_contig_14296_plus1 | 2162 | | 1924 | 0 | 100% | 2 | F:protein binding; F:calcium ion binding | IPR000152 (PROSITE); IPR000742 (PFAM); IPR000859 (G3DSA:2.60.120.GENE3D); IPR001881 (PFAM); IPR013032 (PROSITE); IPR018097 (PROSITE); PD936484 (PRODOM), PR00010 (PRINTS), G3DSA:2.10.25.10 (GENE3D), PTHR24044 (PANTHER), tmhmm (TMHMM), SSF57196 (SUPERFAMILY) | |

| | | | | | | | | | | |
|---|---|---|---|---|---|---|---|---|---|---|
| 507 | lib_dib_Filter15_IDBA_contig_14310_minus3 | 916 | cub and sushi domain-containing protein 3-like | 1355 | 9.98E-52 | 45.60% | 1 | F:calcium ion binding | IPR000152 (PROSITE); IPR000436 (PFAM); IPR001881 (PFAM); IPR018097 (PROSITE); G3DSA:2.10.25.10 (GENE3D), G3DSA:2.10.70.10 (GENE3D), PTHR19325 (PANTHER), tmhmm (TMHMM), SSF57196 (SUPERFAMILY) | |
| 508 | lib_dib_Filter15_IDBA_contig_14311_minus3 | 352 | cub and sushi domain-containing protein 3-like | 401 | 1.32E-32 | 45.65% | 1 | F:calcium ion binding | IPR000436 (PFAM); G3DSA:2.10.70.10 (GENE3D), PTHR19325 (PANTHER) | |
| 509 | lib_dib_Filter15_IDBA_contig_14312_minus3 | 945 | cub and sushi domain-containing protein 3-like | 854 | 1.10E-48 | 44.70% | 1 | F:calcium ion binding | IPR000152 (PROSITE); IPR000436 (PFAM); IPR001881 (SMART); IPR018097 (PROSITE); G3DSA:2.10.25.10 (GENE3D), G3DSA:2.10.70.10 (GENE3D), PTHR19325 (PANTHER) | |
| 510 | lib_dib_Filter15_IDBA_contig_14316_minus3 | 112 | cub and sushi domain-containing protein partial | 198 | 2.90E-19 | 50.85% | 7 | P:proteolysis; F:serine-type endopeptidase activity; F:peptidase inhibitor activity; F:carbohydrate binding; F:catalytic activity; C:extracellular region; P:negative regulation of peptidase activity | IPR000436 (PFAM); G3DSA:2.10.70.10 (GENE3D), PTHR19325 (PANTHER) | |
| 511 | lib_dib_Filter15_IDBA_contig_14327_minus1 | 540 | xanthine dehydrogenase oxidase | 1508 | 0 | 56.40% | 4 | F:catalytic activity; P:metabolic process; F:binding; F:nucleotide binding | IPR000674 (G3DSA:3.90.1170.GENE3D); IPR002346 (PFAM); IPR002888 (G3DSA:1.10.150.GENE3D); IPR005107 (PFAM); IPR008274 (G3DSA:3.30.365.GENE3D); IPR016166 (PROFILE); IPR016169 (G3DSA:3.30.465.GENE3D); G3DSA:3.30.390.50 (GENE3D), PTHR11908 (PANTHER), PTHR11908:SF5 (PANTHER) | |
| 512 | lib_dib_Filter15_IDBA_contig_14328_minus1 | 709 | xanthine dehydrogenase oxidase | 1870 | 0 | 56.45% | 4 | F:catalytic activity; P:metabolic process; F:binding; F:nucleotide binding | IPR000674 (G3DSA:3.90.1170.GENE3D); IPR002346 (PFAM); IPR002888 (G3DSA:1.10.150.GENE3D); IPR005107 (PFAM); IPR008274 (G3DSA:3.30.365.GENE3D); IPR016166 (PROFILE); IPR016169 (G3DSA:3.30.465.GENE3D); G3DSA:3.30.390.50 (GENE3D), PTHR11908 (PANTHER), PTHR11908:SF5 (PANTHER) | |
| 513 | lib_dib_Filter15_IDBA_contig_14329_minus1 | 646 | xanthine dehydrogenase oxidase | 1720 | 0 | 56.40% | 4 | F:catalytic activity; P:metabolic process; F:binding; F:nucleotide binding | IPR000674 (G3DSA:3.90.1170.GENE3D); IPR002346 (PFAM); IPR002888 (G3DSA:1.10.150.GENE3D); IPR005107 (PFAM); IPR008274 (G3DSA:3.30.365.GENE3D); IPR016166 (PROFILE); IPR016169 (G3DSA:3.30.465.GENE3D); G3DSA:3.30.390.50 (GENE3D), PTHR11908 (PANTHER), PTHR11908:SF5 (PANTHER) | |
| 514 | lib_dib_Filter15_IDBA_contig_14334_plus2 | 1856 | serine threonine-protein kinase ulk2 | 2482 | 7.02E-137 | 74.10% | 3 | F:nucleotide binding; P:cellular protein modification process; F:protein kinase activity | IPR000719 (PFAM); IPR002290 (SMART); IPR008271 (PROSITE); IPR011009 (SUPERFAMILY); IPR017441 (PROSITE); IPR020635 (SMART); IPR022708 (PFAM); G3DSA:1.10.510.10 (GENE3D), G3DSA:3.30.200.20 (GENE3D), PTHR24348 (PANTHER), PTHR24348:SF2 (PANTHER), SignalP-NN(euk) (SIGNALP), tmhmm (TMHMM) | Yes |
| 515 | lib_dib_Filter15_IDBA_contig_14335_plus1 | 1855 | serine threonine-protein kinase ulk2 | 2485 | 5.96E-137 | 74.10% | 3 | F:nucleotide binding; P:cellular protein modification process; F:protein kinase activity | IPR000719 (PFAM); IPR002290 (SMART); IPR008271 (PROSITE); IPR011009 (SUPERFAMILY); IPR017441 (PROSITE); IPR020635 (SMART); IPR022708 (PFAM); G3DSA:1.10.510.10 (GENE3D), G3DSA:3.30.200.20 (GENE3D), PTHR24348 (PANTHER), PTHR24348:SF2 (PANTHER), SignalP-NN(euk) (SIGNALP), tmhmm (TMHMM) | |

| | | | | | | | | | | |
|---|---|---|---|---|---|---|---|---|---|---|
| 516 | lib_dib_Filter15_IDBA_contig_14366_plus1 | 164 | a chain x-ray structure of c-met with triazolopyridazine | 923 | 1.05E-87 | 69.90% | 12 | P:anatomical structure morphogenesis; P:cellular component organization; P:cell differentiation; P:multicellular organismal development; C:plasma membrane; F:receptor activity; F:protein kinase activity; P:signal transduction; F:protein binding; P:cell proliferation; F:nucleotide binding; P:cellular protein modification process | IPR000719 (PROFILE); IPR001245 (PRINTS); IPR002011 (PROSITE); IPR002290 (SMART); IPR008266 (PROSITE); IPR011009 (SUPERFAMILY); IPR017441 (PROSITE); IPR020635 (SMART); G3DSA:1.10.510.10 (GENE3D), G3DSA:3.30.200.20 (GENE3D), PTHR24416 (PANTHER), tmhmm (TMHMM) | |
| 517 | lib_dib_Filter15_IDBA_contig_14369_minus1 | 595 | stearoyl- desaturase | 921 | 7.26E-139 | 76.20% | 7 | C:endoplasmic reticulum; C:cell; F:catalytic activity; F:binding; P:biosynthetic process; P:lipid metabolic process; P:metabolic process | IPR001522 (PROSITE); IPR005804 (PFAM); IPR015876 (PRINTS); PTHR11351 (PANTHER), tmhmm (TMHMM) | |
| 518 | lib_dib_Filter15_IDBA_contig_14370_plus1 | 311 | kin of irre-like protein 3 | 1230 | 2.49E-124 | 52.00% | 5 | C:cell; F:protein binding; P:multicellular organismal development; P:biological_process; C:plasma membrane | IPR003598 (SMART); IPR003599 (SMART); IPR007110 (PROFILE); IPR013098 (PFAM); IPR013162 (PFAM); IPR013783 (G3DSA:2.60.40.GENE3D); PTHR11640 (PANTHER), PF13895 (PFAM), tmhmm (TMHMM), SSF48726 (SUPERFAMILY) | |
| 519 | lib_dib_Filter15_IDBA_contig_14375_plus1 | 35116 | b chain rhamnose-binding lectin csl3 | 994 | 2.17E-62 | 47.20% | 1 | F:carbohydrate binding | IPR000922 (PFAM); IPR003582 (PFAM); PTHR12011 (PANTHER), PTHR12011:SF4 (PANTHER), SignalP-NN(euk) (SIGNALP) | |
| 520 | lib_dib_Filter15_IDBA_contig_14383_plus1 | 306 | multiple inositol polyphosphate phosphatase 1-like | 1032 | 1.36E-52 | 44.40% | 1 | F:hydrolase activity | IPR000560 (PFAM); G3DSA:3.40.50.1240 (GENE3D), PTHR20963 (PANTHER), PTHR20963:SF8 (PANTHER), SignalP-NN(euk) (SIGNALP), tmhmm (TMHMM), SSF53254 (SUPERFAMILY) | Yes |
| 521 | lib_dib_Filter15_IDBA_contig_14390_minus1 | 662 | sticks and isoform a | 1192 | 5.93E-18 | 39.95% | 3 | P:biological_process; P:multicellular organismal development; F:protein binding | IPR003597 (PFAM); IPR003598 (SMART); IPR003599 (SMART); IPR003961 (PFAM); IPR007110 (PROFILE); IPR013106 (PFAM); IPR013783 (G3DSA:2.60.40.GENE3D); PTHR10489 (PANTHER), PF13895 (PFAM), SignalP-NN(euk) (SIGNALP), tmhmm (TMHMM), SSF48726 (SUPERFAMILY) | Yes |
| 522 | lib_dib_Filter15_IDBA_contig_14436_plus2 | 316 | protein | 906 | 5.95E-142 | 53.15% | 1 | F:protein binding | IPR001012 (PFAM); G3DSA:3.10.20.90 (GENE3D), G3DSA:3.80.10.10 (GENE3D), PTHR23125 (PANTHER), PF13516 (PFAM), SSF52058 (SUPERFAMILY), SSF54236 (SUPERFAMILY) | |
| 523 | lib_dib_Filter15_IDBA_contig_14501_plus2 | 1739 | tolloid-like protein 2-like | 2095 | 0 | 77.60% | 6 | F:peptidase activity; P:protein metabolic process; P:catabolic process; F:calcium ion binding; F:binding; F:protein binding | IPR000152 (PROSITE); IPR000742 (SMART); IPR000859 (G3DSA:2.60.120.GENE3D); IPR001506 (PRINTS); IPR001881 (PFAM); IPR006026 (SMART); IPR013032 (PROSITE); IPR018097 (PROSITE); IPR024079 (G3DSA:3.40.390.GENE3D); G3DSA:2.10.25.10 (GENE3D), PTHR10127 (PANTHER), PTHR10127:SF72 (PANTHER), tmhmm (TMHMM), SSF55486 (SUPERFAMILY), SSF57196 (SUPERFAMILY) | |
| 524 | lib_dib_Filter15_IDBA_contig_14517_minus1 | 242 | tyrosine-protein kinase htk16-like | 891 | 0 | 67.05% | 4 | F:protein kinase activity; P:cellular protein modification process; F:nucleotide binding; F:protein binding | IPR000719 (PROFILE); IPR000980 (PRINTS); IPR001245 (PRINTS); IPR002110 (SMART); IPR002290 (SMART); IPR008266 (PROSITE); IPR011009 (SUPERFAMILY); IPR017441 (PROSITE); IPR020635 (SMART); IPR020683 (G3DSA:1.25.40.GENE3D); G3DSA:1.10.510.10 (GENE3D), G3DSA:3.30.200.20 (GENE3D), PTHR24418 (PANTHER), SSF55550 (SUPERFAMILY) | |

| | | | | | | | | | | |
|---|---|---|---|---|---|---|---|---|---|---|
| 525 | lib_dib_Filter15_IDBA_contig_14535_minus1 | 476 | btb poz domain-containing protein 2-like | 987 | 0 | 87.60% | 2 | C:cytoplasm; F:protein binding | IPR000210 (SMART); IPR011333 (G3DSA:3.30.710.GENE3D); IPR011705 (PFAM); IPR012983 (PFAM); IPR013069 (PFAM); PTHR24413 (PANTHER), PTHR24413:SF19 (PANTHER) | |
| 526 | lib_dib_Filter15_IDBA_contig_14546_plus2 | 589 | toll- isoform a | 943 | 2.64E-47 | 41.35% | 2 | F:protein binding; P:signal transduction | IPR000157 (PFAM); IPR001611 (PFAM); IPR003591 (SMART); G3DSA:3.40.50.10140 (GENE3D), G3DSA:3.80.10.10 (GENE3D), PTHR24365 (PANTHER), PF13855 (PFAM), tmhmm (TMHMM), SSF52047 (SUPERFAMILY), SSF52058 (SUPERFAMILY) | |
| 527 | lib_dib_Filter15_IDBA_contig_14578_plus1 | 1697 | myeloid lymphoid or mixed-lineage leukemia (trithorax drosophila) translocated 4 | 3008 | 0 | 63.05% | 7 | C:plasma membrane; C:nucleolus; P:cellular component organization; P:cell-cell signaling; F:protein binding; C:cytosol; P:signal transduction | IPR000159 (PFAM); IPR000253 (G3DSA:2.60.200.GENE3D); IPR001478 (PFAM); IPR002710 (PROFILE); IPR008984 (SUPERFAMILY); IPR018444 (PFAM); G3DSA:2.30.42.10 (GENE3D), PTHR10398 (PANTHER), SignalP-NN(euk) (SIGNALP), tmhmm (TMHMM), SSF54236 (SUPERFAMILY) | |
| 528 | lib_dib_Filter15_IDBA_contig_14586_plus1 | 4922 | collagen alpha-6 chain-like | 1975 | 0 | 47.40% | 2 | F:protein binding; F:calcium ion binding | IPR000152 (PROSITE); IPR000742 (PFAM); IPR001881 (SMART); IPR002035 (PFAM); IPR008985 (SUPERFAMILY); IPR013032 (PROSITE); IPR018097 (PROSITE); PR00453 (PRINTS), G3DSA:2.10.25.10 (GENE3D), G3DSA:3.40.50.410 (GENE3D), PTHR22992 (PANTHER), PTHR22992:SF2 (PANTHER), SSF53300 (SUPERFAMILY), SSF57196 (SUPERFAMILY) | |
| 529 | lib_dib_Filter15_IDBA_contig_14608_minus2 | 215 | matrix metalloproteinase-9 | 886 | 2.51E-70 | 41.05% | 10 | F:protein binding; P:multicellular organismal development; P:biological_process; C:extracellular region; P:response to abiotic stimulus; P:regulation of biological process; P:response to stress; F:peptidase activity; P:metabolic process; P:response to external stimulus | IPR000562 (G3DSA:2.10.10.GENE3D); IPR013806 (SUPERFAMILY); PTHR22918 (PANTHER), PTHR22918:SF0 (PANTHER) | |
| 530 | lib_dib_Filter15_IDBA_contig_14622_plus3 | 857 | advillin | 876 | 0 | 62.40% | 8 | P:biological_process; P:cytoskeleton organization; P:cellular component organization; P:regulation of biological process; P:anatomical structure morphogenesis; C:intracellular; C:cell; F:actin binding | IPR003128 (G3DSA:1.10.950.GENE3D); IPR007122 (PRINTS); IPR007123 (PFAM); G3DSA:3.40.20.10 (GENE3D), SSF55753 (SUPERFAMILY) | |
| 531 | lib_dib_Filter15_IDBA_contig_14627_plus2 | 415 | latent-transforming growth factor beta-binding protein 2 | 1370 | 1.11E-33 | 52.15% | 2 | F:protein binding; F:calcium ion binding | IPR000152 (PROSITE); IPR000436 (PROFILE); IPR000742 (SMART); IPR001881 (PFAM); IPR002889 (PFAM); IPR018097 (PROSITE); IPR023413 (G3DSA:2.40.155.GENE3D); G3DSA:2.10.25.10 (GENE3D), PTHR24034 (PANTHER), SignalP-NN(euk) (SIGNALP), SSF57196 (SUPERFAMILY) | Yes |

| | | | | | | | | | |
|---|---|---|---|---|---|---|---|---|---|
| 532 | lib_dib_Filter15_IDBA_contig_14638_plus1 | 1286 | tyrosine-protein phosphatase lar | 2404 | 0 | 66.15% | 3 | F:phosphoprotein phosphatase activity; P:cellular protein modification process; F:protein binding | IPR000242 (PRINTS); IPR000387 (PROFILE); IPR003595 (SMART); IPR003598 (SMART); IPR003599 (SMART); IPR003961 (PFAM); IPR007110 (PROFILE); IPR013098 (PFAM); IPR013783 (G3DSA:2.60.40.GENE3D); IPR016130 (PROSITE); PR00014 (PRINTS), G3DSA:3.90.190.10 (GENE3D), PTHR19134 (PANTHER), PTHR19134:SF48 (PANTHER), tmhmm (TMHMM), SSF48726 (SUPERFAMILY), SSF52799 (SUPERFAMILY) |
| 533 | lib_dib_Filter15_IDBA_contig_14642_plus2 | 220 | pro-neuregulin- membrane-bound isoform-like | 917 | 1.53E-11 | 55.60% | 13 | P:regulation of biological process; P:protein metabolic process; P:multicellular organismal development; C:cell; P:transport; P:metabolic process; P:cellular component organization; P:cellular homeostasis; P:cell communication; P:biological_process; P:signal transduction; P:cell differentiation; F:receptor binding | IPR000742 (PROFILE); IPR013032 (PROSITE); G3DSA:2.10.25.10 (GENE3D), PTHR11100 (PANTHER), PTHR11100:SF7 (PANTHER), tmhmm (TMHMM), SSF57196 (SUPERFAMILY) |
| 534 | lib_dib_Filter15_IDBA_contig_14656_minus3 | 227 | von willebrand factor type egf and pentraxin domain-containing protein 1 | 115 | 3.02E-15 | 54.00% | 11 | F:carbohydrate binding; P:binding of sperm to zona pellucida; F:calcium ion binding; C:extracellular region; C:cytoplasm; P:cell adhesion; C:membrane; F:metal ion binding; P:neuropeptide signaling pathway; C:integral to membrane; F:zinc ion binding | IPR000436 (PFAM); G3DSA:2.10.25.10 (GENE3D), G3DSA:2.10.70.10 (GENE3D), PTHR19325 (PANTHER) |
| 535 | lib_dib_Filter15_IDBA_contig_14659_minus3 | 1538 | cub and sushi domain-containing protein 3-like | 115 | 4.31E-19 | 50.30% | 13 | F:carbohydrate binding; P:proteolysis; F:serine-type endopeptidase activity; F:serine-type peptidase activity; F:hydrolase activity; F:catalytic activity; F:peptidase activity; P:complement activation; C:extracellular region; F:calcium ion binding; C:cytoplasm; P:cell adhesion; C:membrane | IPR000436 (PFAM); G3DSA:2.10.70.10 (GENE3D), PTHR19325 (PANTHER) |
| 536 | lib_dib_Filter15_IDBA_contig_14660_minus1 | 3786 | signal cub and egf-like domain-containing protein 2 isoform 1 | 311 | 1.37E-09 | 62.75% | 3 | F:carbohydrate binding; F:calcium ion binding; C:extracellular region | IPR011641 (PFAM); PTHR24046 (PANTHER), PTHR24046:SF0 (PANTHER) |
| 537 | lib_dib_Filter15_IDBA_contig_14664_minus1 | 5913 | von willebrand factor type egf and pentraxin domain-containing protein 1 | 141 | 3.44E-07 | 46.50% | 9 | F:calcium ion binding; F:chromatin binding; F:complement binding; P:negative regulation of complement activation; C:integral to membrane; C:cytoplasm; P:cell adhesion; C:membrane; C:extracellular region | IPR000436 (PFAM); G3DSA:2.10.70.10 (GENE3D), PTHR19325 (PANTHER), PTHR19325:SF43 (PANTHER) |
| 538 | lib_dib_Filter15_IDBA_contig_14676_minus1 | 2155 | short-chain dehydrogenase reductase sdr | 1103 | 2.70E-131 | 57.35% | 3 | P:metabolic process; F:catalytic activity; F:nucleotide binding | IPR002198 (PFAM); IPR002347 (PRINTS); IPR016040 (G3DSA:3.40.50.GENE3D); PTHR24320 (PANTHER), PTHR24320:SF0 (PANTHER), SignalP-NN(euk) (SIGNALP), tmhmm (TMHMM), SSF51735 (SUPERFAMILY) |
| 539 | lib_dib_Filter15_IDBA_contig_14679_plus2 | 20 | agrin-like protein | 123 | 1.94E-24 | 61.75% | 1 | F:protein binding | IPR002350 (PFAM); G3DSA:3.30.60.30 (GENE3D), PTHR10574 (PANTHER), PTHR10574:SF9 (PANTHER), SSF100895 (SUPERFAMILY) |
| 540 | lib_dib_Filter15_IDBA_contig_14683_plus3 | 194 | oocyte zinc finger protein 6-like | 866 | 6.22E-97 | 61.05% | 3 | F:binding; F:nucleic acid binding; C:intracellular | IPR007087 (PFAM); IPR013087 (G3DSA:3.30.160.GENE3D); IPR015880 (SMART); PTHR24375 (PANTHER), PTHR24375:SF0 (PANTHER), PF13465 (PFAM), SSF57667 (SUPERFAMILY) |

| | | | | | | | | | |
|---|---|---|---|---|---|---|---|---|---|
| 541 | lib_dib_Filter15_IDBA_contig_14691_minus1 | 1364 | tyrosine-protein kinase btk29a | 1411 | 9.62E-82 | 52.50% | 7 | F:nucleotide binding; P:cellular protein modification process; F:protein binding; C:intracellular; P:regulation of biological process; P:cell death; F:protein kinase activity | IPR000719 (PROFILE); IPR000980 (G3DSA:3.30.505.GENE3D); IPR001245 (PRINTS); IPR001315 (PFAM); IPR002290 (SMART); IPR008266 (PROSITE); IPR011009 (SUPERFAMILY); IPR011029 (G3DSA:1.10.533.GENE3D); IPR017441 (PROSITE); IPR020635 (SMART); G3DSA:1.10.510.10 (GENE3D), G3DSA:3.30.200.20 (GENE3D), PTHR24418 (PANTHER), tmhmm (TMHMM), SSF55550 (SUPERFAMILY) |
| 542 | lib_dib_Filter15_IDBA_contig_14692_minus1 | 1358 | tyrosine-protein kinase btk29a | 1405 | 9.39E-82 | 52.50% | 7 | F:nucleotide binding; P:cellular protein modification process; F:protein binding; C:intracellular; P:regulation of biological process; P:cell death; F:protein kinase activity | IPR000719 (PROFILE); IPR000980 (G3DSA:3.30.505.GENE3D); IPR001245 (PRINTS); IPR001315 (PFAM); IPR002290 (SMART); IPR008266 (PROSITE); IPR011009 (SUPERFAMILY); IPR011029 (G3DSA:1.10.533.GENE3D); IPR017441 (PROSITE); IPR020635 (SMART); G3DSA:1.10.510.10 (GENE3D), G3DSA:3.30.200.20 (GENE3D), PTHR24418 (PANTHER), SSF55550 (SUPERFAMILY) |
| 543 | lib_dib_Filter15_IDBA_contig_14700_minus3 | 1638 | membrane metallo isoform cra_a | 1274 | 1.81E-150 | 55.00% | 11 | P:metabolic process; F:binding; C:cell; C:cytoplasmic membrane-bounded vesicle; C:extracellular space; P:biological_process; F:peptidase activity; P:protein metabolic process; P:catabolic process; C:Golgi apparatus; C:endoplasmic reticulum | IPR000718 (PANTHER); IPR008753 (PFAM); IPR018497 (PRINTS); IPR024079 (G3DSA:3.40.390.GENE3D); tmhmm (TMHMM), SSF55486 (SUPERFAMILY) |
| 544 | lib_dib_Filter15_IDBA_contig_14701_minus1 | 711 | reverse transcriptase | 861 | 0 | 54.40% | 4 | F:RNA binding; F:transferase activity; P:DNA metabolic process; P:biosynthetic process | IPR000477 (PFAM); PTHR21301 (PANTHER) |
| 545 | lib_dib_Filter15_IDBA_contig_14709_plus1 | 487 | frizzled-1 precursor | 1073 | 0 | 73.40% | 16 | C:cell; P:signal transduction; C:plasma membrane; P:reproduction; P:multicellular organismal development; P:regulation of biological process; ; F:receptor binding; P:embryo development; P:anatomical structure morphogenesis; P:cellular component organization; P:cell differentiation; C:cytoplasm; F:protein binding; F:receptor activity; P:biological_process | IPR000539 (PRINTS); IPR015526 (PANTHER); IPR017981 (PROFILE); IPR020067 (G3DSA:1.10.2000.GENE3D); IPR026550 (PTHR11309:PANTHER); tmhmm (TMHMM) |
| 546 | lib_dib_Filter15_IDBA_contig_14716_minus2 | 707 | adam family mig-17 | 652 | 8.86E-65 | 47.50% | 3 | F:peptidase activity; P:protein metabolic process; P:catabolic process | IPR001590 (PFAM); IPR024079 (G3DSA:3.40.390.GENE3D); PTHR13723 (PANTHER), PTHR13723:SF9 (PANTHER), SSF55486 (SUPERFAMILY) |
| 547 | lib_dib_Filter15_IDBA_contig_14727_plus3 | 1832 | rab effector | 1852 | 8.36E-54 | 72.00% | 10 | F:cytoskeletal protein binding; F:binding; C:cytoplasmic membrane-bounded vesicle; C:cytoplasm; P:transport; P:regulation of biological process; P:cell-cell signaling; P:protein transport; F:protein binding; F:enzyme regulator activity | IPR003598 (SMART); IPR003599 (SMART); IPR007110 (PROFILE); IPR010911 (PROFILE); IPR011011 (SUPERFAMILY); IPR013083 (G3DSA:3.30.40.GENE3D); IPR013098 (PFAM); IPR013783 (G3DSA:2.60.40.GENE3D); PTHR14555 (PANTHER), PTHR14555:SF3 (PANTHER), SSF48726 (SUPERFAMILY) |

| | | | | | | | | | | |
|---|---|---|---|---|---|---|---|---|---|---|
| 548 | lib_dib_Filter15_IDBA_contig_14728_plus1 | 976 | myosin light chain kinase | 793 | 5.20E-56 | 57.35% | 14 | P:cellular component organization; C:cell; F:protein kinase activity; P:metabolic process; C:cytoplasm; P:regulation of biological process; P:ion transport; P:biological_process; P:response to stress; P:response to abiotic stimulus; C:cytoskeleton; P:anatomical structure morphogenesis; P:multicellular organismal development; F:protein binding | IPR003598 (SMART); IPR003599 (SMART); IPR007110 (PROFILE); IPR013098 (PFAM); IPR013783 (G3DSA:2.60.40.GENE3D); PTHR25963 (PANTHER), SSF48726 (SUPERFAMILY) | |
| 549 | lib_dib_Filter15_IDBA_contig_14730_plus2 | 519 | neuralized-like protein 4 | 1561 | 0 | 69.90% | 3 | F:molecular_function; P:biological_process; C:cellular_component | IPR006573 (PFAM); IPR008985 (SUPERFAMILY); PTHR12429 (PANTHER) | |
| 550 | lib_dib_Filter15_IDBA_contig_14731_plus1 | 108 | neuralized-like protein 4-like | 441 | 2.45E-130 | 67.45% | 3 | F:molecular_function; P:biological_process; C:cellular_component | IPR006573 (PROFILE); PTHR12429 (PANTHER) | |
| 551 | lib_dib_Filter15_IDBA_contig_14870_minus3 | 865 | disintegrin and metalloproteinase domain-containing protein 10-like | 1486 | 0 | 60.40% | 16 | P:signal transduction; C:Golgi apparatus; F:protein binding; C:cell; P:regulation of biological process; P:cell death; C:cytoplasm; P:biological_process; P:protein metabolic process; P:catabolic process; F:peptidase activity; P:cellular protein modification process; C:cytoskeleton; C:nucleus; P:embryo development; F:binding | IPR001590 (PROFILE); IPR001762 (PFAM); IPR002870 (PFAM); IPR024079 (G3DSA:3.40.390.GENE3D); IPR027053 (PTHR11905:PANTHER); PTHR11905 (PANTHER), PF13574 (PFAM), tmhmm (TMHMM), SSF55486 (SUPERFAMILY) | |
| 552 | lib_dib_Filter15_IDBA_contig_14871_minus2 | 396 | sn1-specific diacylglycerol lipase beta | 848 | 0 | 54.10% | 3 | C:cell; F:hydrolase activity; P:lipid metabolic process | IPR002921 (PFAM); G3DSA:3.40.50.1820 (GENE3D), PTHR21493 (PANTHER), PTHR21493:SF20 (PANTHER), SignalP-NN(euk) (SIGNALP), tmhmm (TMHMM), SSF53474 (SUPERFAMILY) | |
| 553 | lib_dib_Filter15_IDBA_contig_14872_plus3 | 406 | sucrase- intestinal | 873 | 0 | 66.10% | 8 | P:biological_process; P:carbohydrate metabolic process; P:catabolic process; C:cell; F:hydrolase activity; C:Golgi apparatus; C:plasma membrane; F:carbohydrate binding | IPR000322 (PANTHER); IPR000519 (G3DSA:4.10.110.GENE3D); IPR011013 (SUPERFAMILY); IPR017853 (SUPERFAMILY); IPR025887 (PFAM); PTHR22762:SF13 (PANTHER), SignalP-NN(euk) (SIGNALP), tmhmm (TMHMM), SSF51011 (SUPERFAMILY) | |
| 554 | lib_dib_Filter15_IDBA_contig_14894_plus2 | 159 | latent-transforming growth factor beta-binding protein 1 isoform 2 | 842 | 3.92E-86 | 50.00% | 2 | F:protein binding; F:calcium ion binding | IPR000152 (PROSITE); IPR000742 (SMART); IPR000884 (SMART); IPR001881 (PFAM); IPR002350 (SMART); IPR013032 (PROSITE); IPR018097 (PROSITE); IPR024731 (PFAM); G3DSA:2.10.25.10 (GENE3D), G3DSA:3.30.60.30 (GENE3D), PTHR24034 (PANTHER), PTHR24034:SF0 (PANTHER), SSF100895 (SUPERFAMILY), SSF57196 (SUPERFAMILY) | |
| 555 | lib_dib_Filter15_IDBA_contig_14901_plus1 | 3540 | ---NA--- | 2429 | | | 0 | - | tmhmm (TMHMM) | |
| 556 | lib_dib_Filter15_IDBA_contig_14907_minus1 | 362 | protein kinase c substrate 80k-h | 997 | 4.78E-100 | 65.85% | 10 | F:protein binding; F:RNA binding; P:biosynthetic process; P:cellular protein modification process; P:carbohydrate metabolic process; P:protein metabolic process; P:response to stress; C:endoplasmic reticulum; C:protein complex; P:multicellular organismal development | IPR002172 (G3DSA:4.10.400.GENE3D); IPR009011 (SUPERFAMILY); IPR026874 (PTHR12630:PANTHER); G3DSA:2.70.130.10 (GENE3D), PTHR12630 (PANTHER), PF12999 (PFAM), PF13015 (PFAM), SignalP-NN(euk) (SIGNALP), tmhmm (TMHMM), SSF47473 (SUPERFAMILY) | Yes |

| | | | | | | | | |
|---|---|---|---|---|---|---|---|---|
| 557 | lib_dib_Filter15_IDBA_contig_14918_plus2 | 1988 | contactin | 1655 | 0 | 52.60% | 6 | C:plasma membrane; P:multicellular organismal development; P:biological_process; P:cell communication; F:carbohydrate binding; F:protein binding | IPR001304 (PFAM); IPR003598 (SMART); IPR003599 (SMART); IPR003961 (PFAM); IPR007110 (PROFILE); IPR013098 (PFAM); IPR013783 (G3DSA:2.60.40.GENE3D); IPR016186 (G3DSA:3.10.100.GENE3D); IPR016187 (SUPERFAMILY); PTHR10489 (PANTHER), PF13895 (PFAM), SignalP-NN(euk) (SIGNALP), tmhmm (TMHMM), SSF48726 (SUPERFAMILY) |
| 558 | lib_dib_Filter15_IDBA_contig_14919_plus3 | 246 | leucine-rich repeat serine threonine-protein kinase 1-like | 848 | 5.39E-128 | 52.95% | 4 | F:nucleotide binding; P:cellular protein modification process; F:protein kinase activity; F:protein binding | IPR000719 (PFAM); IPR002290 (SMART); IPR008271 (PROSITE); IPR011009 (SUPERFAMILY); IPR015943 (G3DSA:2.130.10.GENE3D); IPR017441 (PROSITE); IPR017986 (SUPERFAMILY); IPR020635 (SMART); G3DSA:1.10.510.10 (GENE3D), G3DSA:3.30.200.20 (GENE3D), PTHR23257 (PANTHER), PTHR23257:SF88 (PANTHER), tmhmm (TMHMM) |
| 559 | lib_dib_Filter15_IDBA_contig_14927_plus2 | 3953 | isoform b | 1155 | 0 | 59.85% | 5 | P:transport; P:protein metabolic process; P:catabolic process; F:peptidase activity; F:binding | IPR001930 (PANTHER); IPR014782 (PRINTS); IPR024571 (PFAM); G3DSA:1.10.390.10 (GENE3D), PTHR11533:SF59 (PANTHER), tmhmm (TMHMM), SSF55486 (SUPERFAMILY), SSF63737 (SUPERFAMILY) |
| 560 | lib_dib_Filter15_IDBA_contig_14936_minus2 | 986 | glypican-5 | 983 | 1.79E-90 | 54.00% | 3 | C:cell; C:proteinaceous extracellular matrix; F:protein binding | IPR001863 (PANTHER) |
| 561 | lib_dib_Filter15_IDBA_contig_14944_plus1 | 23 | alpha beta hydrolase fold protein | 145 | 4.90E-19 | 55.75% | 4 | F:hydrolase activity; P:metabolic process; F:catalytic activity; F:epoxide hydrolase activity | G3DSA:3.40.50.1820 (GENE3D), PTHR10992 (PANTHER), PTHR10992:SF98 (PANTHER), PF12697 (PFAM), SSF53474 (SUPERFAMILY) |
| 562 | lib_dib_Filter15_IDBA_contig_14975_minus2 | 165 | serine threonine-protein kinase lmtk1-like | 833 | 0 | 60.75% | 5 | F:protein binding; C:mitochondrion; F:nucleotide binding; P:cellular protein modification process; F:protein kinase activity | IPR000719 (PROFILE); IPR001245 (PRINTS); IPR002290 (SMART); IPR011009 (SUPERFAMILY); IPR020635 (SMART); G3DSA:1.10.510.10 (GENE3D), G3DSA:3.30.200.20 (GENE3D), PTHR24416 (PANTHER), PTHR24416:SF69 (PANTHER), SignalP-NN(euk) (SIGNALP), tmhmm (TMHMM) |
| 563 | lib_dib_Filter15_IDBA_contig_14979_minus3 | 178 | multicopper oxidase | 831 | 1.29E-152 | 52.15% | 3 | F:catalytic activity; P:metabolic process; F:binding | IPR001117 (PFAM); IPR002355 (PROSITE); IPR008972 (G3DSA:2.60.40.GENE3D); IPR011706 (PFAM); IPR011707 (PFAM); PTHR11709 (PANTHER), PTHR11709:SF8 (PANTHER), SignalP-NN(euk) (SIGNALP), tmhmm (TMHMM) |
| 564 | lib_dib_Filter15_IDBA_contig_14996_plus3 | 142 | atrial natriuretic peptide receptor 1-like | 830 | 0 | 71.20% | 7 | F:nucleotide binding; P:cellular protein modification process; P:nucleobase-containing compound metabolic process; P:biosynthetic process; F:catalytic activity; C:cell; F:protein kinase activity | IPR000719 (PROFILE); IPR001054 (G3DSA:3.30.70.GENE3D); IPR001170 (PRINTS); IPR001245 (PFAM); IPR001828 (PFAM); IPR002290 (SMART); IPR011009 (SUPERFAMILY); IPR020635 (SMART); G3DSA:1.10.510.10 (GENE3D), G3DSA:3.30.200.20 (GENE3D), G3DSA:3.40.50.2300 (GENE3D), PTHR11920 (PANTHER), PTHR11920:SF50 (PANTHER), tmhmm (TMHMM), SSF53822 (SUPERFAMILY) |
| 565 | lib_dib_Filter15_IDBA_contig_15014_plus3 | 848 | fibrinogen-like 1 | 826 | 2.43E-49 | 57.60% | 5 | F:receptor binding; C:extracellular space; P:signal transduction; P:response to stilbenoid; C:fibrinogen complex | IPR002181 (PFAM); IPR014715 (G3DSA:4.10.530.GENE3D); IPR014716 (G3DSA:3.90.215.GENE3D); PTHR19143 (PANTHER) |

| | | | | | | | | | |
|---|---|---|---|---|---|---|---|---|---|
| 566 | lib_dib_Filter15_IDBA_contig_15040_plus1 | 947 | protein | 1718 | 0 | 66.90% | 5 | C:proteinaceous extracellular matrix; F:protein binding; F:receptor activity; C:cell; F:calcium ion binding | IPR000152 (PROSITE); IPR000742 (PFAM); IPR001190 (PFAM); IPR001881 (SMART); IPR013032 (PFAM); IPR017448 (SMART); IPR018097 (PROSITE); PR00010 (PRINTS), G3DSA:2.10.25.10 (GENE3D), G3DSA:2.170.300.10 (GENE3D), G3DSA:3.10.250.10 (GENE3D), PTHR24048 (PANTHER), PTHR24048:SF15 (PANTHER), SignalP-NN(euk) (SIGNALP), tmhmm (TMHMM), SSF57196 (SUPERFAMILY) |
| 567 | lib_dib_Filter15_IDBA_contig_15042_plus3 | 52 | protein | 132 | 4.35E-50 | 72.30% | 3 | C:proteinaceous extracellular matrix; F:protein binding; F:calcium ion binding | IPR000152 (PROSITE); IPR000742 (PFAM); IPR001881 (SMART); IPR013032 (PROSITE); IPR018097 (PROSITE); PR00010 (PRINTS), G3DSA:2.10.25.10 (GENE3D), PTHR24044 (PANTHER), SSF57196 (SUPERFAMILY) |
| 568 | lib_dib_Filter15_IDBA_contig_15049_plus1 | 235 | spondin-1 | 825 | 0 | 54.05% | 2 | C:extracellular region; F:enzyme regulator activity | IPR000884 (PFAM); IPR002223 (PRINTS); IPR002861 (PFAM); IPR009465 (PFAM); IPR020901 (PROSITE); G3DSA:2.20.100.10 (GENE3D), PTHR11311 (PANTHER) |
| 569 | lib_dib_Filter15_IDBA_contig_15057_plus3 | 410 | glycogen synthase kinase-3 beta | 823 | 0 | 87.20% | 32 | P:multicellular organismal development; C:cell; P:regulation of biological process; P:cellular protein modification process; F:protein kinase activity; P:regulation of gene expression, epigenetic; P:DNA metabolic process; P:generation of precursor metabolites and energy; P:biosynthetic process; P:carbohydrate metabolic process; P:anatomical structure morphogenesis; P:cellular component organization; P:cell differentiation; F:protein binding; P:protein transport; P:biological_process; C:cytosol; P:signal transduction; C:cytoplasm; C:protein complex; P:nucleobase-containing compound metabolic process; P:cell death; ; P:response to stress; P:response to biotic stimulus; P:cell cycle; C:plasma membrane; F:nucleotide binding; C:microtubule organizing center; C:nucleolus; P:protein metabolic process; P:catabolic process | IPR000719 (PFAM); IPR002290 (SMART); IPR008271 (PROSITE); IPR011009 (SUPERFAMILY); IPR017441 (PROSITE); IPR020635 (SMART); G3DSA:1.10.510.10 (GENE3D), G3DSA:3.30.200.20 (GENE3D), PTHR24057 (PANTHER) |
| 570 | lib_dib_Filter15_IDBA_contig_15058_plus2 | 45753 | thioester-containing protein | 1885 | 2.04E-178 | 54.70% | 2 | F:enzyme regulator activity; C:extracellular space | IPR001599 (PFAM); IPR002890 (PFAM); IPR008930 (SUPERFAMILY); IPR009048 (G3DSA:2.60.40.GENE3D); IPR011625 (PFAM); IPR011626 (PFAM); IPR019565 (PFAM); IPR019742 (PROSITE); G3DSA:1.50.10.20 (GENE3D), PTHR11412 (PANTHER) |
| 571 | lib_dib_Filter15_IDBA_contig_15059_plus3 | 27869 | thioester-containing protein | 1442 | 1.60E-165 | 58.00% | 2 | F:enzyme regulator activity; C:extracellular space | IPR001599 (PFAM); IPR008930 (SUPERFAMILY); IPR009048 (G3DSA:2.60.40.GENE3D); IPR011626 (PFAM); IPR019565 (PFAM); IPR019742 (PROSITE); G3DSA:1.50.10.20 (GENE3D), PTHR11412 (PANTHER), SignalP-NN(euk) (SIGNALP), tmhmm (TMHMM) |
| 572 | lib_dib_Filter15_IDBA_contig_15060_plus1 | 7219 | thiolester containing protein isoform e | 343 | 2.78E-05 | 51.80% | 1 | C:extracellular region | IPR009048 (G3DSA:2.60.40.GENE3D); PTHR11412 (PANTHER) |
| 573 | lib_dib_Filter15_IDBA_contig_15075_plus1 | 146 | protocadherin fat 4 | 823 | 0 | 47.70% | 5 | P:signal transduction; P:multicellular organismal development; F:calcium ion binding; P:biological_process; C:cell | IPR002126 (PRINTS); IPR015919 (SUPERFAMILY); PTHR24027 (PANTHER) |

| | | | | | | | | | |
|---|---|---|---|---|---|---|---|---|---|
| 574 | lib_dib_Filter15_IDBA_contig_15083_minus1 | 290 | neurogenic locus notch homolog protein 2-like | 989 | 1.06E-137 | 63.40% | 26 | P:anatomical structure morphogenesis; P:multicellular organismal development; P:cell differentiation; P:regulation of biological process; P:cellular component organization; P:signal transduction; P:cell proliferation; P:transport; P:biological_process; P:embryo development; P:cell death; F:DNA binding; F:chromatin binding; ; P:response to external stimulus; P:response to stress; C:cell; F:sequence-specific DNA binding transcription factor activity; F:protein binding; C:nucleus; P:reproduction; P:growth; F:enzyme regulator activity; P:response to biotic stimulus; C:cytoplasm; C:plasma membrane | IPR002110 (SMART); IPR011656 (PFAM); IPR020683 (G3DSA:1.25.40.GENE3D); IPR024600 (PFAM); PR01983 (PRINTS), PTHR24044 (PANTHER), PTHR24044:SF11 (PANTHER), tmhmm (TMHMM) |
| 575 | lib_dib_Filter15_IDBA_contig_1509_plus2 | 1098 | lysosomal protective protein precursor | 615 | 3.27E-161 | 65.60% | 10 | F:enzyme regulator activity; P:lipid metabolic process; C:nucleolus; C:lysosome; P:protein transport; P:biological_process; C:endoplasmic reticulum; F:peptidase activity; P:protein metabolic process; P:catabolic process | IPR001563 (PRINTS); IPR018202 (PROSITE); G3DSA:3.40.50.1820 (GENE3D), SSF53474 (SUPERFAMILY) |
| 576 | lib_dib_Filter15_IDBA_contig_15104_plus1 | 371 | n-acetylated-alpha-linked acidic dipeptidase 2 | 819 | 1.88E-109 | 49.90% | 3 | F:peptidase activity; P:protein metabolic process; P:catabolic process | IPR003137 (PFAM); IPR007365 (G3DSA:1.20.930.GENE3D); IPR007484 (PFAM); G3DSA:3.40.630.10 (GENE3D), G3DSA:3.50.30.30 (GENE3D), PTHR10404 (PANTHER), tmhmm (TMHMM), SSF52025 (SUPERFAMILY), SSF53187 (SUPERFAMILY) |
| 577 | lib_dib_Filter15_IDBA_contig_15116_minus3 | 194 | fk506-binding protein 15 | 823 | 1.34E-81 | 63.70% | 7 | C:cell; F:catalytic activity; C:cytoskeleton; C:protein complex; P:metabolic process; P:regulation of biological process; P:protein metabolic process | IPR001179 (PFAM); IPR023566 (PANTHER); G3DSA:3.10.50.40 (GENE3D), PTHR10516:SF16 (PANTHER), SSF54534 (SUPERFAMILY) |
| 578 | lib_dib_Filter15_IDBA_contig_15122_plus1 | 569 | krueppel-like factor 1 | 817 | 1.50E-59 | 88.35% | 9 | F:binding; P:cell differentiation; P:multicellular organismal development; C:nuclear chromosome; P:embryo development; F:sequence-specific DNA binding transcription factor activity; P:regulation of biological process; ; P:organelle organization | IPR007087 (PROSITE); IPR013087 (G3DSA:3.30.160.GENE3D); IPR015880 (SMART); PTHR23223 (PANTHER), PTHR23223:SF23 (PANTHER), PF13465 (PFAM), tmhmm (TMHMM), SSF57667 (SUPERFAMILY) |
| 579 | lib_dib_Filter15_IDBA_contig_15133_minus2 | 176 | atp-dependent rna helicase ddx1 | 814 | 0 | 78.00% | 16 | P:response to biotic stimulus; C:cytoplasm; P:biological_process; F:transcription regulator activity; F:protein binding; F:hydrolase activity; P:regulation of biological process; ; P:response to stress; P:DNA metabolic process; F:DNA binding; F:RNA binding; F:nucleotide binding; F:nuclease activity; P:nucleobase-containing compound metabolic process; F:chromatin binding | IPR001650 (PFAM); IPR001870 (PROFILE); IPR003877 (PFAM); IPR008985 (SUPERFAMILY); IPR011545 (PFAM); IPR014001 (SMART); IPR014014 (PROFILE); IPR018355 (SMART); G3DSA:3.40.50.300 (GENE3D), PTHR24031 (PANTHER), PTHR24031:SF60 (PANTHER), SignalP-NN(euk) (SIGNALP), SSF52540 (SUPERFAMILY) |

| | | | | | | | | | |
|---|---|---|---|---|---|---|---|---|---|
| 580 | lib_dib_Filter15_IDBA_contig_15134_plus3 | 170 | f-box wd repeat-containing protein 7 isoform 2 | 813 | 0 | 84.10% | 21 | P:signal transduction; P:metabolic process; P:regulation of biological process; ; C:Golgi apparatus; C:intracellular; C:protein complex; F:DNA binding; P:response to abiotic stimulus; P:protein metabolic process; P:catabolic process; F:sequence-specific DNA binding transcription factor activity; C:cytoplasm; C:endoplasmic reticulum; P:organelle organization; P:cell cycle; P:DNA metabolic process; P:biosynthetic process; C:nucleus; P:response to stress; F:protein binding | IPR001680 (PFAM); IPR001810 (SMART); IPR015943 (G3DSA:2.130.10.GENE3D); IPR017986 (PROFILE); IPR019775 (PROSITE); IPR020472 (PRINTS); G3DSA:1.10.150.320 (GENE3D), G3DSA:1.20.1280.50 (GENE3D), PTHR22844 (PANTHER), PTHR22844:SF52 (PANTHER), PF12836 (PFAM), PF12937 (PFAM) |
| 581 | lib_dib_Filter15_IDBA_contig_15135_plus2 | 126 | discoidin domain-containing receptor 2-like | 814 | 1.74E-132 | 67.95% | 4 | P:biological_process; F:nucleotide binding; P:cellular protein modification process; F:protein kinase activity | IPR000421 (PFAM); IPR000719 (PROFILE); IPR001245 (PFAM); IPR002290 (SMART); IPR008266 (PROSITE); IPR008979 (SUPERFAMILY); IPR011009 (SUPERFAMILY); IPR017441 (PROSITE); IPR020635 (SMART); G3DSA:1.10.510.10 (GENE3D), G3DSA:2.60.120.260 (GENE3D), G3DSA:3.30.200.20 (GENE3D), PTHR24416 (PANTHER), PTHR24416:SF64 (PANTHER), tmhmm (TMHMM) |
| 582 | lib_dib_Filter15_IDBA_contig_15139_minus1 | 2279 | adamts-like protein 5 | 812 | 1.66E-70 | 56.55% | 4 | F:protein binding; F:peptidase activity; C:proteinaceous extracellular matrix; F:binding | IPR000884 (PFAM); IPR001134 (PROFILE); IPR008993 (SUPERFAMILY); IPR010294 (PFAM); IPR013273 (PRINTS); IPR018933 (PFAM); G3DSA:2.20.100.10 (GENE3D), G3DSA:2.40.50.120 (GENE3D), PTHR13723 (PANTHER), PTHR13723:SF19 (PANTHER), tmhmm (TMHMM) |
| 583 | lib_dib_Filter15_IDBA_contig_15149_minus2 | 367 | interleukin-18 receptor 1 | 812 | 1.14E-14 | 42.50% | 2 | F:protein binding; P:signal transduction | IPR000157 (PFAM); IPR003599 (SMART); IPR007110 (PROFILE); IPR013783 (G3DSA:2.60.40.GENE3D); IPR015621 (PANTHER); PR01537 (PRINTS), G3DSA:3.40.50.10140 (GENE3D), SSF48726 (SUPERFAMILY) |
| 584 | lib_dib_Filter15_IDBA_contig_15158_minus1 | 5483 | kn motif and ankyrin repeat domain-containing protein 1 | 216 | 2.48E-93 | 77.45% | 11 | P:regulation of biological process; C:plasma membrane; P:signal transduction; C:cytoplasm; P:response to endogenous stimulus; P:cellular component organization; P:cell differentiation; P:multicellular organismal development; F:protein binding; P:cytoskeleton organization; C:nucleus | IPR002110 (PRINTS); IPR020683 (G3DSA:1.25.40.GENE3D); PTHR24168 (PANTHER) |
| 585 | lib_dib_Filter15_IDBA_contig_1517_minus2 | 8432 | ---NA--- | 492 | | | 0 | - | PTHR22797 (PANTHER), PTHR22797:SF4 (PANTHER) |
| 586 | lib_dib_Filter15_IDBA_contig_1517_minus3 | 8432 | gram positive anchor | 492 | 1.24E-07 | 63.40% | 18 | C:cell wall; C:membrane; C:extracellular region; C:integral to membrane; P:metal ion transport; P:cation transport; F:ATPase activity, coupled to transmembrane movement of ions, phosphorylative mechanism; F:hydrolase activity; P:ATP metabolic process; F:nucleotide binding; F:ATP binding; P:ATP biosynthetic process; F:catalytic activity; F:metal ion transmembrane transporter activity; F:metal ion binding; F:copper-transporting ATPase activity; P:metabolic process; F:hydrolase activity, acting on acid anhydrides, in phosphorus-containing anhydrides | PTHR22797 (PANTHER), SignalP-NN(euk) (SIGNALP), tmhmm (TMHMM) |

| | | | | | | | | | |
|---|---|---|---|---|---|---|---|---|---|
| 587 | lib_dib_Filter15_IDBA_contig_15230_plus2 | 149 | zinc finger protein plag1-like | 805 | 9.26E-72 | 64.70% | 9 | P:biological_process; P:multicellular organismal development; P:metabolic process; P:regulation of biological process; P:growth; F:protein binding; F:binding; F:nucleic acid binding; C:intracellular | IPR001214 (PROFILE); IPR007087 (PROSITE); IPR013087 (G3DSA:3.30.160.GENE3D); IPR015880 (SMART); G3DSA:2.170.270.10 (GENE3D), PTHR10032 (PANTHER), PTHR10032:SF63 (PANTHER), PF13465 (PFAM), PF13894 (PFAM), SSF57667 (SUPERFAMILY) |
| 588 | lib_dib_Filter15_IDBA_contig_15247_plus1 | 453 | ankyrin repeat protein | 1129 | 8.03E-31 | 46.80% | 4 | P:biological_process; C:cell; P:cellular component organization; F:protein binding | IPR001496 (PFAM); IPR002110 (PRINTS); IPR020683 (G3DSA:1.25.40.GENE3D); PTHR24188 (PANTHER), tmhmm (TMHMM) |
| 589 | lib_dib_Filter15_IDBA_contig_15254_minus1 | 1075 | dnaj homolog subfamily a member 1 | 1643 | 3.22E-177 | 78.15% | 12 | F:protein binding; P:biological_process; P:response to stress; C:cell; P:protein metabolic process; F:binding; P:response to abiotic stimulus; P:reproduction; F:nucleotide binding; F:receptor binding; P:response to biotic stimulus; P:signal transduction | IPR001305 (G3DSA:2.10.230.GENE3D); IPR001623 (PRINTS); IPR002939 (PFAM); IPR008971 (SUPERFAMILY); IPR018253 (PROSITE); G3DSA:2.60.260.20 (GENE3D), PTHR24076 (PANTHER), PTHR24076:SF1 (PANTHER), tmhmm (TMHMM) |
| 590 | lib_dib_Filter15_IDBA_contig_15262_minus2 | 4672 | beta-lactamase | 1362 | 3.26E-110 | 49.95% | 3 | F:nucleic acid binding; F:zinc ion binding; C:intracellular | IPR001466 (PFAM); IPR012338 (G3DSA:3.40.710.GENE3D); PTHR22935 (PANTHER), tmhmm (TMHMM) |
| 591 | lib_dib_Filter15_IDBA_contig_15268_plus1 | 370 | kelch-like protein 20 | 1085 | 0 | 92.25% | 17 | P:protein metabolic process; P:catabolic process; C:nucleolus; C:intracellular; C:protein complex; F:protein binding; P:cytoskeleton organization; C:cell; F:catalytic activity; P:cellular protein modification process; C:nucleoplasm; F:actin binding; C:cytoskeleton; C:cytoplasm; P:regulation of biological process; P:cell death; C:Golgi apparatus | IPR000210 (SMART); IPR006652 (PFAM); IPR011333 (G3DSA:3.30.710.GENE3D); IPR011705 (PFAM); IPR013069 (PFAM); IPR015916 (G3DSA:2.130.10.GENE3D); PTHR24412 (PANTHER), PTHR24412:SF27 (PANTHER), SSF117281 (SUPERFAMILY) |
| 592 | lib_dib_Filter15_IDBA_contig_15270_plus1 | 155 | a chain x-ray structure of c-met with triazolopyridazine | 799 | 6.28E-74 | 62.05% | 12 | P:anatomical structure morphogenesis; P:cellular component organization; P:cell differentiation; P:multicellular organismal development; C:plasma membrane; F:receptor activity; F:protein kinase activity; P:signal transduction; F:protein binding; P:cell proliferation; F:nucleotide binding; P:cellular protein modification process | IPR000719 (PROFILE); IPR001245 (PRINTS); IPR002290 (SMART); IPR008266 (PROSITE); IPR011009 (SUPERFAMILY); IPR017441 (PROSITE); IPR020635 (SMART); G3DSA:1.10.510.10 (GENE3D), G3DSA:3.30.200.20 (GENE3D), PTHR24416 (PANTHER) |
| 593 | lib_dib_Filter15_IDBA_contig_15273_plus2 | 1961 | protein camp- beta | 913 | 0 | 93.70% | 27 | F:protein binding; P:reproduction; P:cell differentiation; C:nucleoplasm; P:biological_process; P:multicellular organismal development; P:regulation of biological process; C:cellular_component; P:response to endogenous stimulus; C:cell; C:Golgi apparatus; P:protein transport; C:cytosol; P:cellular protein modification process; P:cell cycle; C:microtubule organizing center; P:generation of precursor metabolites and energy; C:protein complex; C:intracellular; C:cytoplasm; P:anatomical structure morphogenesis; P:embryo development; P:cell-cell signaling; C:plasma membrane; F:nucleotide binding; F:protein kinase activity; C:mitochondrion | IPR000719 (PFAM); IPR000961 (SMART); IPR002290 (SMART); IPR008271 (PROSITE); IPR011009 (SUPERFAMILY); IPR017441 (PROSITE); IPR020635 (SMART); G3DSA:1.10.510.10 (GENE3D), G3DSA:3.30.200.20 (GENE3D), PTHR24353 (PANTHER), PTHR24353:SF25 (PANTHER) |

| | | | | | | | | | |
|---|---|---|---|---|---|---|---|---|---|
| 594 | lib_dib_Filter15_IDBA_contig_15274_plus1 | 1995 | camp-dependent protein kinase catalytic subunit beta | 963 | 0 | 94.60% | 8 | P:multicellular organismal development; P:behavior; P:reproduction; F:protein kinase activity; P:cellular protein modification process; F:nucleotide binding; F:binding; P:anatomical structure morphogenesis | IPR000719 (PFAM); IPR000961 (SMART); IPR002290 (SMART); IPR008271 (PROSITE); IPR011009 (SUPERFAMILY); IPR017441 (PROSITE); IPR020635 (SMART); G3DSA:1.10.510.10 (GENE3D), G3DSA:3.30.200.20 (GENE3D), PTHR24353 (PANTHER), PTHR24353:SF25 (PANTHER) |
| 595 | lib_dib_Filter15_IDBA_contig_15287_minus2 | 207 | abhydrolase domain-containing protein fam108c1-like | 794 | 0 | 91.60% | 2 | C:extracellular region; F:hydrolase activity | G3DSA:3.40.50.1820 (GENE3D), PTHR12277 (PANTHER), PF12695 (PFAM), SignalP-NN(euk) (SIGNALP), tmhmm (TMHMM), SSF53474 (SUPERFAMILY) |
| 596 | lib_dib_Filter15_IDBA_contig_15288_minus2 | 586 | alkaline tissue-nonspecific isozyme | 794 | 4.26E-131 | 60.40% | 2 | P:metabolic process; F:hydrolase activity | IPR001952 (PRINTS); IPR017849 (G3DSA:3.40.720.GENE3D); IPR017850 (SUPERFAMILY); IPR018299 (PROSITE); PTHR11596 (PANTHER), SignalP-NN(euk) (SIGNALP), tmhmm (TMHMM) |
| 597 | lib_dib_Filter15_IDBA_contig_1533_plus3 | 3546 | blastula protease-10 | 542 | 7.22E-44 | 52.00% | 4 | F:peptidase activity; P:protein metabolic process; P:catabolic process; F:binding | IPR001506 (PRINTS); IPR003582 (PFAM); IPR006026 (SMART); IPR024079 (G3DSA:3.40.390.GENE3D); PTHR10127 (PANTHER), PTHR10127:SF334 (PANTHER), tmhmm (TMHMM), SSF55486 (SUPERFAMILY) |
| 598 | lib_dib_Filter15_IDBA_contig_15358_minus3 | 5199 | neuronal cell adhesion molecule-like | 1177 | 9.19E-58 | 43.30% | 7 | P:anatomical structure morphogenesis; P:cellular component organization; P:cell differentiation; P:multicellular organismal development; P:biological_process; C:cell; F:protein binding | IPR003598 (SMART); IPR003599 (SMART); IPR007110 (PROFILE); IPR013098 (PFAM); IPR013783 (G3DSA:2.60.40.GENE3D); IPR026966 (PFAM); PTHR10489 (PANTHER), PF13895 (PFAM), tmhmm (TMHMM), SSF48726 (SUPERFAMILY) |
| 599 | lib_dib_Filter15_IDBA_contig_15360_minus3 | 28 | peroxidasin homolog | 108 | 2.01E-10 | 51.60% | 1 | F:protein binding | IPR007110 (PROFILE); IPR013098 (PFAM); IPR013783 (G3DSA:2.60.40.GENE3D); PTHR10489 (PANTHER), SSF48726 (SUPERFAMILY) |
| 600 | lib_dib_Filter15_IDBA_contig_15382_minus3 | 938 | ns1 binding protein | 1190 | 1.30E-169 | 54.45% | 1 | F:protein binding | IPR000210 (SMART); IPR006652 (PFAM); IPR011043 (SUPERFAMILY); IPR011333 (G3DSA:3.30.710.GENE3D); IPR011705 (PFAM); IPR013069 (PFAM); IPR015915 (G3DSA:2.120.10.GENE3D); PR00501 (PRINTS), PTHR24412 (PANTHER), PTHR24412:SF3 (PANTHER), tmhmm (TMHMM) |
| 601 | lib_dib_Filter15_IDBA_contig_15383_minus3 | 946 | ns1 binding protein | 1211 | 1.68E-169 | 54.45% | 1 | F:protein binding | IPR000210 (SMART); IPR006652 (PFAM); IPR011043 (SUPERFAMILY); IPR011333 (G3DSA:3.30.710.GENE3D); IPR011705 (PFAM); IPR013069 (PFAM); IPR015915 (G3DSA:2.120.10.GENE3D); PR00501 (PRINTS), PTHR24412 (PANTHER), PTHR24412:SF3 (PANTHER) |
| 602 | lib_dib_Filter15_IDBA_contig_15385_plus3 | 1411 | ---NA--- | 914 | | | 0 | - | PTHR23304 (PANTHER), PTHR23304:SF80 (PANTHER), tmhmm (TMHMM) |
| 603 | lib_dib_Filter15_IDBA_contig_15414_minus1 | 189 | dual specificity protein phosphatase 26-like | 785 | 4.68E-36 | 57.55% | 2 | P:cellular protein modification process; F:phosphoprotein phosphatase activity | IPR000340 (PFAM); IPR000387 (PROFILE); IPR003595 (SMART); IPR020422 (SMART); IPR024950 (PANTHER); G3DSA:3.90.190.10 (GENE3D), SSF52799 (SUPERFAMILY) |
| 604 | lib_dib_Filter15_IDBA_contig_15414_minus2 | 189 | calcium-binding protein | 785 | 1.77E-26 | 36.85% | 11 | F:calcium ion binding; C:extracellular region; P:pathogenesis; P:cell adhesion; C:integrin complex; P:proteolysis; F:serine-type endopeptidase activity; F:metallopeptidase activity; C:extracellular space; F:zinc ion binding; F:metalloendopeptidase activity | IPR011049 (SUPERFAMILY); G3DSA:2.150.10.10 (GENE3D) |

| | | | | | | | | | |
|---|---|---|---|---|---|---|---|---|---|
| 605 | lib_dib_Filter15_IDBA_contig_15415_minus1 | 125 | neurotrypsin-like | 784 | 2.53E-72 | 55.70% | 4 | F:catalytic activity; F:receptor activity; C:cell; F:protein binding | IPR000082 (PFAM); IPR001190 (PRINTS); IPR017448 (SMART); IPR020067 (G3DSA:1.10.2000.GENE3D); G3DSA:3.10.250.10 (GENE3D), G3DSA:3.30.70.960 (GENE3D), PTHR19331 (PANTHER), PTHR19331:SF118 (PANTHER), tmhmm (TMHMM), SSF82671 (SUPERFAMILY) |
| 606 | lib_dib_Filter15_IDBA_contig_15416_minus3 | 236 | glycosyl transferase | 783 | 1.92E-83 | 46.75% | 11 | F:carbohydrate binding; F:transferase activity; C:integral to membrane; C:membrane; C:Golgi apparatus; F:transferase activity, transferring glycosyl groups; P:carbohydrate metabolic process; F:molecular_function; C:cellular_component; P:biological_process; F:polypeptide N-acetylgalactosaminyltransferase activity | IPR000772 (PFAM); IPR001173 (PFAM); G3DSA:2.80.10.50 (GENE3D), G3DSA:3.90.550.10 (GENE3D), PTHR11675 (PANTHER), SSF53448 (SUPERFAMILY) |
| 607 | lib_dib_Filter15_IDBA_contig_15422_plus1 | 433 | serine threonine-protein kinase tao1 | 1190 | 0 | 75.00% | 14 | P:signal transduction; P:response to stress; P:cell cycle; P:regulation of biological process; P:cellular protein modification process; P:biological_process; F:enzyme regulator activity; F:protein binding; P:cytoskeleton organization; P:cell death; C:cytoplasm; F:protein kinase activity; F:nucleotide binding; P:DNA metabolic process | IPR000719 (PFAM); IPR002290 (SMART); IPR011009 (SUPERFAMILY); IPR017441 (PROSITE); IPR020635 (SMART); G3DSA:1.10.510.10 (GENE3D), G3DSA:3.30.200.20 (GENE3D), PTHR24361 (PANTHER), PTHR24361:SF33 (PANTHER) |
| 608 | lib_dib_Filter15_IDBA_contig_15423_plus3 | 2735 | aldehyde dehydrogenase 7 member a1 | 782 | 0 | 81.20% | 11 | C:cytosol; F:catalytic activity; P:metabolic process; F:protein binding; F:nucleotide binding; P:catabolic process; ; C:mitochondrion; P:biological_process; P:biosynthetic process; C:nucleus | IPR015590 (PFAM); IPR016160 (PROSITE); IPR016161 (SUPERFAMILY); IPR016162 (G3DSA:3.40.605.GENE3D); IPR016163 (G3DSA:3.40.309.GENE3D); PTHR11699 (PANTHER), PTHR11699:SF26 (PANTHER), tmhmm (TMHMM) |
| 609 | lib_dib_Filter15_IDBA_contig_15426_plus1 | 685 | #NAME? | 934 | 0 | 78.20% | 9 | C:cell; F:hydrolase activity; P:biosynthetic process; P:carbohydrate metabolic process; P:generation of precursor metabolites and energy; F:binding; F:carbohydrate binding; F:transferase activity; C:cytosol | IPR004193 (PFAM); IPR006047 (PFAM); IPR006048 (PFAM); IPR006589 (SMART); IPR013780 (G3DSA:2.60.40.GENE3D); IPR013781 (G3DSA:3.20.20.GENE3D); IPR013783 (G3DSA:2.60.40.GENE3D); IPR014756 (SUPERFAMILY); IPR015902 (PANTHER); IPR017853 (SUPERFAMILY); PTHR10357:SF27 (PANTHER), SSF51011 (SUPERFAMILY) |
| 610 | lib_dib_Filter15_IDBA_contig_15448_plus3 | 113 | low quality protein: cubilin | 780 | 1.12E-48 | 46.20% | 3 | C:organelle; C:intracellular; C:cytoplasm | IPR000859 (G3DSA:2.60.120.GENE3D); PTHR10127 (PANTHER), PTHR10127:SF345 (PANTHER), tmhmm (TMHMM) |
| 611 | lib_dib_Filter15_IDBA_contig_15478_plus1 | 1941 | swi snf matrix actin dependent regulator of subfamily member 1 | 2084 | 6.35E-87 | 72.35% | 13 | F:protein binding; ; P:regulation of biological process; C:nucleoplasm; C:protein complex; C:nuclear chromosome; C:nucleolus; F:transferase activity; C:nucleus; F:transcription regulator activity; F:receptor binding; F:chromatin binding; P:organelle organization | IPR009071 (G3DSA:1.10.30.GENE3D); PTHR13711 (PANTHER), PTHR13711:SF13 (PANTHER), tmhmm (TMHMM) |
| 612 | lib_dib_Filter15_IDBA_contig_15479_plus1 | 1437 | ---NA--- | 1560 | | | 0 | - | tmhmm (TMHMM) |
| 613 | lib_dib_Filter15_IDBA_contig_15501_plus1 | 275 | zinc finger protein 271-like | 927 | 2.07E-89 | 50.65% | 3 | F:binding; F:nucleic acid binding; C:intracellular | IPR007087 (PFAM); IPR013087 (G3DSA:3.30.160.GENE3D); IPR015880 (SMART); PTHR24375 (PANTHER), PTHR24375:SF0 (PANTHER), PF13465 (PFAM), PF13894 (PFAM), PF13912 (PFAM), SSF57667 (SUPERFAMILY) |

| | | | | | | | | | | |
|---|---|---|---|---|---|---|---|---|---|---|
| 614 | lib_dib_Filter15_IDBA_contig_1552_plus3 | 3705 | hypothetical protein CGI_10002658 | 223 | 6.74E-56 | 56.75% | 2 | F:transporter activity; P:transport | IPR015255 (PFAM); IPR015817 (G3DSA:2.20.50.GENE3D); IPR015818 (G3DSA:2.20.80.GENE3D); IPR015819 (SUPERFAMILY); PTHR23345 (PANTHER) | |
| 615 | lib_dib_Filter15_IDBA_contig_15627_minus2 | 662 | dpy-31 protein | 1428 | 1.02E-101 | 54.30% | 7 | P:biological_process; P:multicellular organismal development; F:peptidase activity; P:protein metabolic process; P:catabolic process; F:protein binding; F:binding | IPR000859 (G3DSA:2.60.120.GENE3D); IPR001506 (PRINTS); IPR002035 (PFAM); IPR006026 (SMART); IPR013032 (PROSITE); IPR024079 (G3DSA:3.40.390.GENE3D); PR00453 (PRINTS), G3DSA:3.40.50.410 (GENE3D), PTHR22992 (PANTHER), tmhmm (TMHMM), SSF53300 (SUPERFAMILY), SSF55486 (SUPERFAMILY) | |
| 616 | lib_dib_Filter15_IDBA_contig_15633_minus1 | 234 | class b secretin-like g-protein coupled receptor | 771 | 2.41E-75 | 48.40% | 5 | F:receptor activity; P:signal transduction; C:cell; P:biological_process; F:carbohydrate binding | IPR000832 (PRINTS); IPR001212 (PROFILE); IPR017981 (PROFILE); PTHR12011 (PANTHER), tmhmm (TMHMM) | |
| 617 | lib_dib_Filter15_IDBA_contig_15643_minus3 | 270 | udp- c:polypeptide n- | 768 | 0 | 58.60% | 1 | P:carbohydrate metabolic process | IPR000772 (PROFILE); IPR001173 (PFAM); IPR003859 (PFAM); G3DSA:3.90.550.10 (GENE3D), PTHR11675 (PANTHER), tmhmm (TMHMM), SSF53448 (SUPERFAMILY) | |
| 618 | lib_dib_Filter15_IDBA_contig_15648_plus1 | 255 | protocadherin-11 x-linked | 768 | 8.07E-98 | 54.00% | 3 | F:calcium ion binding; P:biological_process; C:plasma membrane | IPR002126 (PRINTS); IPR015919 (SUPERFAMILY); IPR020894 (PROSITE); PTHR24027 (PANTHER), tmhmm (TMHMM) | |
| 619 | lib_dib_Filter15_IDBA_contig_15661_plus2 | 4563 | thioester-containing protein | 1810 | 0 | 49.40% | 2 | F:enzyme regulator activity; C:extracellular space | IPR001599 (PFAM); IPR002890 (PFAM); IPR008930 (SUPERFAMILY); IPR009048 (G3DSA:2.60.40.GENE3D); IPR011625 (PFAM); IPR011626 (PFAM); IPR019565 (PFAM); IPR019742 (PROSITE); G3DSA:1.50.10.20 (GENE3D), PTHR11412 (PANTHER) | |
| 620 | lib_dib_Filter15_IDBA_contig_15674_plus1 | 118 | low-density lipoprotein receptor-related protein 6 isoform 1 | 766 | 0 | 63.00% | 20 | P:anatomical structure morphogenesis; P:embryo development; C:organelle; C:cytoplasm; C:cell; F:enzyme regulator activity; P:multicellular organismal development; F:receptor binding; F:protein binding; P:regulation of biological process; C:plasma membrane; P:cell differentiation; ; P:cell death; P:biological_process; P:growth; P:cellular component organization; F:transporter activity; P:cell cycle; F:receptor activity | IPR000033 (PFAM); IPR000742 (SMART); IPR002172 (G3DSA:4.10.400.GENE3D); IPR011042 (G3DSA:2.120.10.GENE3D); IPR023415 (PROSITE); PR00261 (PRINTS), G3DSA:2.10.25.10 (GENE3D), PTHR10529 (PANTHER), PTHR10529:SF109 (PANTHER), tmhmm (TMHMM), SSF57196 (SUPERFAMILY), SSF63825 (SUPERFAMILY) | |
| 621 | lib_dib_Filter15_IDBA_contig_15676_minus1 | 497 | integrin beta-ps | 946 | 0 | 63.50% | 4 | P:biological_process; C:cell; F:receptor activity; P:multicellular organismal development | IPR002369 (PFAM); IPR003659 (SMART); IPR012896 (SUPERFAMILY); IPR013111 (PFAM); IPR014836 (PFAM); IPR015812 (PRINTS); IPR016201 (SUPERFAMILY); G3DSA:1.20.5.100 (GENE3D), G3DSA:2.10.25.10 (GENE3D), G3DSA:3.40.50.410 (GENE3D), PTHR10082:SF16 (PANTHER), SignalP-NN(euk) (SIGNALP), tmhmm (TMHMM), SSF53300 (SUPERFAMILY), SSF57196 (SUPERFAMILY) | Yes |
| 622 | lib_dib_Filter15_IDBA_contig_15677_minus1 | 495 | integrin beta-ps | 933 | 0 | 63.45% | 4 | P:biological_process; C:cell; F:receptor activity; P:multicellular organismal development | IPR002369 (PFAM); IPR003659 (SMART); IPR012896 (SUPERFAMILY); IPR013111 (PFAM); IPR014836 (PFAM); IPR015812 (PRINTS); IPR016201 (SUPERFAMILY); G3DSA:1.20.5.100 (GENE3D), G3DSA:2.10.25.10 (GENE3D), G3DSA:3.40.50.410 (GENE3D), PTHR10082:SF16 (PANTHER), SignalP-NN(euk) (SIGNALP), tmhmm (TMHMM), SSF53300 (SUPERFAMILY), SSF57196 (SUPERFAMILY) | Yes |

| | | | | | | | | | | |
|---|---|---|---|---|---|---|---|---|---|---|
| 623 | lib_dib_Filter15_IDBA_contig_15708_minus2 | 251 | multicopper oxidase | 846 | 2.66E-84 | 48.10% | 3 | F:catalytic activity; P:metabolic process; F:binding | IPR001117 (PFAM); IPR002355 (PROSITE); IPR008972 (G3DSA:2.60.40.GENE3D); IPR011706 (PFAM); IPR011707 (PFAM); PTHR11709 (PANTHER), SignalP-NN(euk) (SIGNALP), tmhmm (TMHMM) | Yes |
| 624 | lib_dib_Filter15_IDBA_contig_1572_plus1 | 176 | macrophage migration inhibitory factor | 286 | 8.58E-11 | 50.80% | 19 | P:metabolic process; P:regulation of biological process; P:response to abiotic stimulus; P:cellular protein modification process; P:biological_process; P:cell death; P:response to stress; P:cell proliferation; P:response to external stimulus; P:signal transduction; P:transport; C:intracellular; P:multicellular organismal development; P:response to biotic stimulus; P:response to endogenous stimulus; C:extracellular region; F:catalytic activity; P:cellular component organization; F:receptor binding | IPR001398 (PRODOM); IPR014347 (SUPERFAMILY); G3DSA:3.30.429.10 (GENE3D) | |
| 625 | lib_dib_Filter15_IDBA_contig_15729_minus2 | 368 | isoform b | 868 | 3.95E-153 | 72.70% | 2 | P:anatomical structure morphogenesis; P:multicellular organismal development | IPR009581 (PFAM); IPR024869 (PANTHER); PTHR12450:SF8 (PANTHER), SignalP-NN(euk) (SIGNALP), tmhmm (TMHMM) | |
| 626 | lib_dib_Filter15_IDBA_contig_15732_plus1 | 24812 | calreticulin | 942 | 2.43E-176 | 85.50% | 4 | P:protein metabolic process; C:endoplasmic reticulum; F:protein binding; F:calcium ion binding | IPR001580 (PRINTS); IPR008985 (SUPERFAMILY); IPR013320 (G3DSA:2.60.120.GENE3D); IPR018124 (PROSITE); PTHR11073:SF2 (PANTHER), tmhmm (TMHMM) | |
| 627 | lib_dib_Filter15_IDBA_contig_15740_minus3 | 263 | slit homolog 2 | 646 | 8.10E-28 | 43.30% | 2 | F:protein binding; P:signal transduction | IPR000157 (PFAM); G3DSA:3.40.50.10140 (GENE3D), G3DSA:3.80.10.10 (GENE3D), PTHR24365 (PANTHER), PF13855 (PFAM), tmhmm (TMHMM), SSF52058 (SUPERFAMILY) | |
| 628 | lib_dib_Filter15_IDBA_contig_15741_minus1 | 189 | leucine-rich repeat | 756 | 7.07E-32 | 42.35% | 1 | F:protein binding | IPR001611 (PROFILE); IPR003591 (SMART); G3DSA:3.80.10.10 (GENE3D), PTHR24365 (PANTHER), PF13855 (PFAM), SSF52058 (SUPERFAMILY) | |
| 629 | lib_dib_Filter15_IDBA_contig_15806_minus2 | 158 | protein | 747 | 0 | 57.20% | 1 | P:biological_process | IPR000421 (PFAM); IPR008979 (SUPERFAMILY); G3DSA:2.60.120.260 (GENE3D), PTHR10127 (PANTHER), PTHR10127:SF476 (PANTHER) | |
| 630 | lib_dib_Filter15_IDBA_contig_15812_plus1 | 203 | serine threonine-protein kinase pak 1 isoform 1 | 746 | 0 | 76.75% | 4 | F:protein binding; F:nucleotide binding; P:cellular protein modification process; F:protein kinase activity | IPR000095 (PFAM); IPR000719 (PFAM); IPR002290 (SMART); IPR008271 (PROSITE); IPR011009 (SUPERFAMILY); IPR017441 (PROSITE); IPR020635 (SMART); G3DSA:1.10.510.10 (GENE3D), G3DSA:3.30.200.20 (GENE3D), G3DSA:3.90.810.10 (GENE3D), PTHR24361 (PANTHER), PTHR24361:SF186 (PANTHER) | |
| 631 | lib_dib_Filter15_IDBA_contig_15819_plus1 | 32 | lpxtg-motif cell wall anchor domain partial | 102 | 1.54E-20 | 56.10% | 4 | F:transferase activity; C:cell wall; C:membrane; C:extracellular region | PTHR14054 (PANTHER), PTHR14054:SF2 (PANTHER) | |

| | | | | | | | | | |
|---|---|---|---|---|---|---|---|---|---|
| 632 | lib_dib_Filter15_IDBA_contig_15829_plus3 | 179 | platelet-activating factor acetylhydrolase ib subunit alpha | 744 | 0 | 89.15% | 35 | C:cytoskeleton; C:protein complex; C:cilium; P:multicellular organismal development; F:protein binding; P:signal transduction; P:response to stress; P:transport; P:organelle organization; P:cell cycle; P:biological_process; C:cell; P:anatomical structure morphogenesis; P:cytoskeleton organization; P:catabolic process; P:lipid metabolic process; F:carbohydrate binding; P:cell-cell signaling; P:cell differentiation; P:cell proliferation; P:cellular component organization; P:regulation of biological process; C:cytosol; C:chromosome; F:transferase activity; P:behavior; F:cytoskeletal protein binding; C:cytoplasm; P:reproduction; F:hydrolase activity; ; C:nuclear envelope; C:microtubule organizing center; C:nucleolus; P:protein | IPR001680 (PFAM); IPR006594 (SMART); IPR013720 (PFAM); IPR015943 (G3DSA:2.130.10.GENE3D); IPR017252 (PTHR22847:PANTHER); IPR017986 (PROFILE); IPR019775 (PROSITE); IPR020472 (PRINTS); PTHR22847 (PANTHER), SSF109925 (SUPERFAMILY) |
| 633 | lib_dib_Filter15_IDBA_contig_15840_minus1 | 14583 | serine protease inhibitor 3 | 1255 | 2.30E-15 | 64.70% | 3 | F:molecular_function; P:metabolic process; F:enzyme regulator activity | IPR000716 (G3DSA:4.10.800.GENE3D); IPR002223 (PRINTS); IPR004094 (PFAM); IPR011061 (SUPERFAMILY); IPR018112 (G3DSA:2.10.22.GENE3D); IPR020901 (PROSITE); PTHR10083 (PANTHER), SignalP-NN(euk) (SIGNALP), tmhmm (TMHMM) |
| 634 | lib_dib_Filter15_IDBA_contig_15862_minus3 | 1067 | hydrophilic acylated surface protein b | 233 | 1.38E-08 | 57.46% | 9 | P:proteolysis; F:serine-type endopeptidase activity; C:outer membrane; F:3-phosphoshikimate 1-carboxyvinyltransferase activity; F:transferase activity, transferring alkyl or aryl (other than methyl) groups; F:transferase activity; F:catalytic activity; P:cellular amino acid biosynthetic process; P:aromatic amino acid family biosynthetic process | no IPS match |
| 635 | lib_dib_Filter15_IDBA_contig_15890_minus1 | 86 | uncharacterized aarf domain-containing protein kinase 2 | 743 | 3.11E-164 | 58.00% | 2 | C:mitochondrion; F:transferase activity | IPR004147 (PFAM); G3DSA:3.90.1200.10 (GENE3D), PTHR10566 (PANTHER), PTHR10566:SF23 (PANTHER), SignalP-NN(euk) (SIGNALP), tmhmm (TMHMM) |
| 636 | lib_dib_Filter15_IDBA_contig_15905_minus2 | 218 | cytolysin src-1-like | 742 | 7.80E-19 | 50.10% | 6 | P:ion transport; F:transporter activity; C:cell; C:protein complex; P:cellular component organization; P:symbiosis, encompassing mutualism through parasitism | IPR009104 (PFAM); IPR015926 (G3DSA:2.60.270.GENE3D) |
| 637 | lib_dib_Filter15_IDBA_contig_15969_minus3 | 402 | tyrosine-protein phosphatase 99a-like | 1254 | 0 | 59.45% | 10 | C:cell; P:anatomical structure morphogenesis; P:cellular component organization; P:cell differentiation; P:multicellular organismal development; P:cell recognition; C:plasma membrane; F:phosphoprotein phosphatase activity; P:cellular protein modification process; F:protein binding | IPR000242 (PRINTS); IPR000387 (PROFILE); IPR003595 (SMART); IPR003961 (PFAM); IPR013783 (G3DSA:2.60.40.GENE3D); IPR016130 (PROSITE); G3DSA:3.90.190.10 (GENE3D), PTHR19134 (PANTHER), PTHR19134:SF45 (PANTHER), tmhmm (TMHMM), SSF52799 (SUPERFAMILY) |

| | | | | | | | | | |
|---|---|---|---|---|---|---|---|---|---|
| 638 | lib_dib_Filter15_IDBA_contig_15970_minus3 | 400 | tyrosine-protein phosphatase 99a-like | 1254 | 0 | 59.45% | 10 | C:cell; P:anatomical structure morphogenesis; P:cellular component organization; P:cell differentiation; P:multicellular organismal development; P:cell recognition; C:plasma membrane; F:phosphoprotein phosphatase activity; P:cellular protein modification process; F:protein binding | IPR000242 (PRINTS); IPR000387 (PROFILE); IPR003595 (SMART); IPR003961 (PFAM); IPR013783 (G3DSA:2.60.40.GENE3D); IPR016130 (PROSITE); G3DSA:3.90.190.10 (GENE3D), PTHR19134 (PANTHER), PTHR19134:SF45 (PANTHER), tmhmm (TMHMM), SSF52799 (SUPERFAMILY) |
| 639 | lib_dib_Filter15_IDBA_contig_15992_plus1 | 1047 | acetylcholinesterase | 965 | 1.26E-180 | 62.80% | 3 | F:hydrolase activity; P:cell-cell signaling; P:metabolic process | IPR000997 (PRINTS); IPR002018 (PFAM); IPR019819 (PROSITE); IPR019826 (PROSITE); G3DSA:3.40.50.1820 (GENE3D), PTHR11559 (PANTHER), PTHR11559:SF33 (PANTHER), tmhmm (TMHMM), SSF53474 (SUPERFAMILY) |
| 640 | lib_dib_Filter15_IDBA_contig_15993_plus1 | 1038 | acetylcholinesterase | 920 | 0 | 62.90% | 3 | F:hydrolase activity; P:cell-cell signaling; P:metabolic process | IPR000997 (PRINTS); IPR002018 (PFAM); IPR019819 (PROSITE); IPR019826 (PROSITE); G3DSA:3.40.50.1820 (GENE3D), PTHR11559 (PANTHER), PTHR11559:SF33 (PANTHER), SignalP-NN(euk) (SIGNALP), tmhmm (TMHMM), SSF53474 (SUPERFAMILY) |
| 641 | lib_dib_Filter15_IDBA_contig_15999_minus1 | 309 | basement membrane-specific heparan sulfate proteoglycan core protein | 865 | 0 | 55.70% | 5 | P:biological_process; F:protein binding; P:multicellular organismal development; P:metabolic process; C:extracellular region | IPR000034 (PFAM); IPR002049 (PFAM); IPR002172 (G3DSA:4.10.400.GENE3D); IPR003598 (SMART); IPR003599 (SMART); IPR007110 (PROFILE); IPR013032 (PROSITE); IPR013098 (PFAM); IPR013783 (G3DSA:2.60.40.GENE3D); IPR018031 (SMART); IPR023415 (PROSITE); PR00261 (PRINTS), G3DSA:2.10.25.10 (GENE3D), PTHR10574 (PANTHER), PTHR10574:SF98 (PANTHER), SSF48726 (SUPERFAMILY), SSF57196 (SUPERFAMILY) |
| 642 | lib_dib_Filter15_IDBA_contig_16000_minus1 | 546 | basement membrane-specific heparan sulfate proteoglycan core protein | 1530 | 0 | 56.70% | 9 | P:anatomical structure morphogenesis; P:embryo development; P:cell differentiation; P:multicellular organismal development; C:proteinaceous extracellular matrix; F:protein binding; P:metabolic process; P:biological_process; P:cellular component organization | IPR000034 (PFAM); IPR000742 (SMART); IPR002049 (PFAM); IPR002172 (G3DSA:4.10.400.GENE3D); IPR003598 (SMART); IPR003599 (SMART); IPR007110 (PROFILE); IPR013032 (PROSITE); IPR013098 (PFAM); IPR013783 (G3DSA:2.60.40.GENE3D); IPR018031 (SMART); IPR023415 (PROSITE); PR00261 (PRINTS), G3DSA:2.10.25.10 (GENE3D), PTHR10574 (PANTHER), PTHR10574:SF25 (PANTHER), SSF48726 (SUPERFAMILY), SSF57196 (SUPERFAMILY) |
| 643 | lib_dib_Filter15_IDBA_contig_16001_minus1 | 2301 | isoform b | 1531 | 1.69E-29 | 65.55% | 14 | P:multicellular organismal development; F:cytoskeletal protein binding; P:metabolic process; P:regulation of biological process; C:cell; F:receptor activity; C:plasma membrane; C:cellular_component; P:anatomical structure morphogenesis; P:cellular component organization; P:cell differentiation; P:response to external stimulus; P:response to abiotic stimulus; C:extracellular region | IPR000716 (G3DSA:4.10.800.GENE3D); IPR001050 (PANTHER); IPR003585 (SMART); PTHR10915:SF1 (PANTHER), tmhmm (TMHMM) |
| 644 | lib_dib_Filter15_IDBA_contig_16013_plus3 | 10501 | protein disulfide-isomerase a3-like | 1079 | 0 | 71.60% | 7 | F:transferase activity; F:catalytic activity; P:metabolic process; F:electron carrier activity; P:cellular homeostasis; P:regulation of biological process; C:endoplasmic reticulum | IPR005746 (PRINTS); IPR005788 (TIGRFAMs); IPR005792 (TIGRFAMs); IPR012336 (G3DSA:3.40.30.GENE3D); IPR013766 (PFAM); IPR017937 (PROSITE); PTHR18929 (PANTHER), PTHR18929:SF32 (PANTHER), PF13848 (PFAM), tmhmm (TMHMM) |

| | | | | | | | | | | |
|---|---|---|---|---|---|---|---|---|---|---|
| 645 | lib_dib_Filter15_IDBA_contig_16016_plus1 | 179 | ---NA--- | 912 | | | 0 | - | IPR008160 (PFAM); PTHR24023 (PANTHER) | |
| 646 | lib_dib_Filter15_IDBA_contig_16038_minus3 | 212 | acid sphingomyelinase-like phosphodiesterase 3b | 814 | 2.42E-95 | 55.55% | 1 | F:hydrolase activity | IPR004843 (PFAM); G3DSA:3.60.21.10 (GENE3D), PTHR10340 (PANTHER), tmhmm (TMHMM), SSF56300 (SUPERFAMILY) | |
| 647 | lib_dib_Filter15_IDBA_contig_16039_minus3 | 211 | acid sphingomyelinase-like phosphodiesterase 3b | 794 | 2.06E-95 | 55.50% | 1 | F:hydrolase activity | IPR004843 (PFAM); G3DSA:3.60.21.10 (GENE3D), PTHR10340 (PANTHER), tmhmm (TMHMM), SSF56300 (SUPERFAMILY) | |
| 648 | lib_dib_Filter15_IDBA_contig_16047_minus3 | 12592 | serine threonine-protein kinase prp4 homolog | 907 | 0 | 87.65% | 8 | P:cellular protein modification process; F:protein kinase activity; C:nucleolus; F:nucleotide binding; F:protein binding; P:nucleobase-containing compound metabolic process; C:nucleus; C:chromosome | IPR000719 (PFAM); IPR002290 (SMART); IPR008271 (PROSITE); IPR011009 (SUPERFAMILY); IPR020635 (SMART); G3DSA:1.10.510.10 (GENE3D), G3DSA:3.30.200.20 (GENE3D), PTHR24056 (PANTHER), PTHR24056:SF45 (PANTHER), SignalP-NN(euk) (SIGNALP), tmhmm (TMHMM) | Yes |
| 649 | lib_dib_Filter15_IDBA_contig_16048_minus3 | 12560 | serine threonine-protein kinase prp4 homolog | 756 | 0 | 87.65% | 8 | P:cellular protein modification process; F:protein kinase activity; C:nucleolus; F:nucleotide binding; F:protein binding; P:nucleobase-containing compound metabolic process; C:nucleus; C:chromosome | IPR000719 (PFAM); IPR002290 (SMART); IPR008271 (PROSITE); IPR011009 (SUPERFAMILY); IPR020635 (SMART); G3DSA:1.10.510.10 (GENE3D), G3DSA:3.30.200.20 (GENE3D), PTHR24056 (PANTHER), PTHR24056:SF45 (PANTHER), SignalP-NN(euk) (SIGNALP), tmhmm (TMHMM) | Yes |
| 650 | lib_dib_Filter15_IDBA_contig_16052_plus1 | 6009 | angiotensin i converting enzyme (peptidyl-dipeptidase a) isoform cra_i | 795 | 4.32E-93 | 53.55% | 11 | P:biological_process; F:protein binding; C:extracellular region; P:regulation of biological process; P:multicellular organismal development; F:binding; C:cell; P:catabolic process; C:plasma membrane; P:protein metabolic process; F:peptidase activity | IPR001548 (PRINTS); PTHR10514:SF16 (PANTHER), SignalP-NN(euk) (SIGNALP), tmhmm (TMHMM), SSF55486 (SUPERFAMILY) | |
| 651 | lib_dib_Filter15_IDBA_contig_16054_plus2 | 557 | #NAME? | 969 | 0 | 73.15% | 3 | F:catalytic activity; P:biosynthetic process; P:carbohydrate metabolic process | IPR001830 (PFAM); IPR003337 (PFAM); IPR006379 (TIGRFAMs); IPR023214 (G3DSA:3.40.50.GENE3D); G3DSA:3.40.50.2000 (GENE3D), PTHR10788 (PANTHER), SSF53756 (SUPERFAMILY) | |
| 652 | lib_dib_Filter15_IDBA_contig_16064_minus1 | 109 | zinc finger protein 271-like | 726 | 8.80E-143 | 64.05% | 3 | F:binding; F:nucleic acid binding; C:intracellular | IPR007087 (PFAM); IPR013087 (G3DSA:3.30.160.GENE3D); IPR015880 (SMART); PTHR24377 (PANTHER), PTHR24377:SF0 (PANTHER), PF13465 (PFAM), SSF57667 (SUPERFAMILY) | |
| 653 | lib_dib_Filter15_IDBA_contig_16071_plus3 | 541 | neuropeptide y | 1050 | 7.22E-07 | 79.25% | 2 | F:receptor binding; C:extracellular region | IPR001955 (PFAM); G3DSA:1.20.5.570 (GENE3D), tmhmm (TMHMM) | |
| 654 | lib_dib_Filter15_IDBA_contig_16083_minus3 | 230 | related to glyoxal oxidase precursor | 770 | 4.13E-16 | 44.55% | 1 | F:calcium ion binding | IPR000152 (PROSITE); IPR000436 (PROFILE); IPR001881 (PFAM); IPR002889 (PFAM); IPR013994 (SMART); IPR018097 (PROSITE); G3DSA:2.10.25.10 (GENE3D), PTHR14949 (PANTHER), PTHR14949:SF6 (PANTHER), SSF57196 (SUPERFAMILY) | |
| 655 | lib_dib_Filter15_IDBA_contig_16084_minus3 | 744 | related to glyoxal oxidase precursor | 1360 | 1.78E-20 | 44.10% | 1 | F:calcium ion binding | IPR000152 (PROSITE); IPR000436 (PROFILE); IPR001881 (PFAM); IPR002889 (PFAM); IPR013994 (SMART); IPR018097 (PROSITE); G3DSA:2.10.25.10 (GENE3D), PTHR14949 (PANTHER), PTHR14949:SF6 (PANTHER), SSF57196 (SUPERFAMILY) | |

| | | | | | | | | | |
|---|---|---|---|---|---|---|---|---|---|
| 656 | lib_dib_Filter15_IDBA_contig_16088_minus2 | 265 | receptor-type tyrosine-protein phosphatase r | 724 | 2.44E-152 | 64.55% | 9 | C:cell; F:receptor activity; F:phosphoprotein phosphatase activity; F:protein binding; P:embryo development; C:Golgi apparatus; P:cell differentiation; P:multicellular organismal development; C:nucleus | IPR000242 (PRINTS); IPR000387 (PROFILE); IPR003595 (SMART); IPR008356 (PRINTS); IPR016130 (PROSITE); G3DSA:3.90.190.10 (GENE3D), PTHR19134 (PANTHER), PTHR19134:SF41 (PANTHER), SignalP-NN(euk) (SIGNALP), tmhmm (TMHMM), SSF52799 (SUPERFAMILY) |
| 657 | lib_dib_Filter15_IDBA_contig_16096_plus2 | 746 | laminin subunit beta-1 | 2068 | 0 | 64.70% | 2 | C:cell; F:protein binding | IPR000742 (SMART); IPR002049 (PFAM); IPR008211 (PFAM); IPR013015 (PROFILE); IPR013032 (PROSITE); PR00011 (PRINTS), G3DSA:1.20.1170.10 (GENE3D), G3DSA:2.10.25.10 (GENE3D), PTHR10574 (PANTHER), PTHR10574:SF36 (PANTHER), tmhmm (TMHMM), SSF57196 (SUPERFAMILY) |
| 658 | lib_dib_Filter15_IDBA_contig_16097_plus2 | 105 | laminin subunit beta-1 | 422 | 1.52E-37 | 54.70% | 2 | P:cellular component organization; P:multicellular organismal development | PTHR10574 (PANTHER), PTHR10574:SF36 (PANTHER), tmhmm (TMHMM) |
| 659 | lib_dib_Filter15_IDBA_contig_16100_minus2 | 1537 | apolipoprotein f precursor | 1128 | 2.75E-07 | 51.00% | 0 | - | IPR001064 (SMART); IPR011024 (SUPERFAMILY); IPR026114 (PANTHER); G3DSA:2.60.20.10 (GENE3D), PTHR15011:SF4 (PANTHER) |
| 660 | lib_dib_Filter15_IDBA_contig_16116_plus3 | 2611 | antistasin-like protein | 2367 | 6.60E-150 | 52.40% | 1 | F:enzyme regulator activity | IPR002223 (PRINTS); IPR004094 (PFAM); IPR011061 (SUPERFAMILY); IPR018112 (G3DSA:2.10.22.GENE3D); IPR020901 (PROSITE); PTHR10083 (PANTHER), tmhmm (TMHMM) |
| 661 | lib_dib_Filter15_IDBA_contig_16117_plus1 | 177 | antistasin-like protein | 278 | 3.77E-56 | 55.85% | 1 | F:enzyme regulator activity | IPR004094 (PFAM); IPR011061 (SUPERFAMILY); IPR018112 (G3DSA:2.10.22.GENE3D); PTHR11339 (PANTHER), PTHR11339:SF40 (PANTHER) |
| 662 | lib_dib_Filter15_IDBA_contig_16120_minus3 | 643 | collagen alpha-1 chain-like | 1288 | 3.07E-23 | 50.65% | 1 | F:protein binding | IPR001073 (PFAM); IPR002035 (PFAM); IPR003609 (SMART); IPR006583 (PFAM); IPR008983 (G3DSA:2.60.120.GENE3D); PR00453 (PRINTS), G3DSA:3.40.50.410 (GENE3D), G3DSA:3.50.4.10 (GENE3D), PTHR22992 (PANTHER), PF14295 (PFAM), SSF53300 (SUPERFAMILY), SSF57414 (SUPERFAMILY) |
| 663 | lib_dib_Filter15_IDBA_contig_16122_minus3 | 3465 | 78 kda glucose-regulated protein precursor | 995 | 0 | 82.45% | 29 | C:cell; C:endoplasmic reticulum; C:protein complex; F:protein binding; P:signal transduction; P:anatomical structure morphogenesis; P:multicellular organismal development; P:regulation of biological process; P:embryo development; P:nucleobase-containing compound metabolic process; P:catabolic process; P:cellular protein modification process; F:enzyme regulator activity; P:cell death; P:protein metabolic process; P:response to stress; P:response to biotic stimulus; C:nucleus; C:cytosol; C:cytoplasm; F:binding; P:response to external stimulus; P:cell communication; P:metabolic process; F:nucleotide binding; F:hydrolase activity; C:cytoplasmic membrane-bounded vesicle; | IPR013126 (PRINTS); IPR018181 (PROSITE); G3DSA:1.20.1270.10 (GENE3D), G3DSA:2.60.34.10 (GENE3D), G3DSA:3.30.30.30 (GENE3D), G3DSA:3.30.420.40 (GENE3D), G3DSA:3.90.640.10 (GENE3D), PTHR19375 (PANTHER), tmhmm (TMHMM), SSF100920 (SUPERFAMILY), SSF100934 (SUPERFAMILY), SSF53067 (SUPERFAMILY) |
| 664 | lib_dib_Filter15_IDBA_contig_16126_minus1 | 226 | tumor necrosis factor ligand superfamily member 10-like | 719 | 2.76E-34 | 52.25% | 3 | F:receptor binding; P:biological_process; C:cell | IPR006052 (PFAM); IPR008983 (G3DSA:2.60.120.GENE3D); IPR021184 (PROSITE); PTHR11471 (PANTHER), tmhmm (TMHMM) |

| | | | | | | | | | |
|---|---|---|---|---|---|---|---|---|---|
| 665 | lib_dib_Filter15_IDBA_contig_16130_minus1 | 393 | atlastin-2 isoform 1 | 756 | 0 | 76.90% | 10 | P:organelle organization; C:cell; P:cellular component organization; F:protein binding; P:nucleobase-containing compound metabolic process; P:catabolic process; P:transport; F:hydrolase activity; C:endoplasmic reticulum; F:nucleotide binding | IPR003191 (PFAM); IPR015894 (PFAM); G3DSA:1.20.1000.10 (GENE3D), G3DSA:3.40.50.300 (GENE3D), PTHR10751 (PANTHER), PTHR10751:SF16 (PANTHER), tmhmm (TMHMM), SSF52540 (SUPERFAMILY) |
| 666 | lib_dib_Filter15_IDBA_contig_16142_plus2 | 6129 | egf-like domain-containing protein | 1446 | 1.11E-33 | 48.60% | 5 | P:cellular component organization; C:plasma membrane; C:cell; F:protein binding; F:calcium ion binding | IPR000152 (PROSITE); IPR000436 (PFAM); IPR000742 (SMART); IPR001881 (PFAM); IPR013032 (PROSITE); IPR018097 (PROSITE); G3DSA:2.10.25.10 (GENE3D), G3DSA:2.10.70.10 (GENE3D), PTHR24035 (PANTHER), PTHR24035:SF19 (PANTHER), SignalP-NN(euk) (SIGNALP), tmhmm (TMHMM), SSF57196 (SUPERFAMILY) |
| 667 | lib_dib_Filter15_IDBA_contig_16167_plus3 | 331 | sex-determining protein fem-1 | 739 | 3.43E-158 | 60.20% | 1 | F:protein binding | IPR002110 (PFAM); IPR020683 (G3DSA:1.25.40.GENE3D); PTHR24182 (PANTHER), PTHR24182:SF2 (PANTHER) |
| 668 | lib_dib_Filter15_IDBA_contig_16227_plus3 | 933 | low quality protein: cubilin | 1496 | 1.66E-38 | 43.50% | 6 | C:cell; C:organelle; C:intracellular; F:binding; C:cytoplasm; P:metabolic process | IPR000859 (G3DSA:2.60.120.GENE3D); PTHR10127 (PANTHER), tmhmm (TMHMM) |
| 669 | lib_dib_Filter15_IDBA_contig_16237_plus3 | 148 | polypeptide n-acetylgalactosaminyltransferase 1 | 710 | 5.86E-169 | 60.75% | 4 | P:biosynthetic process; P:cellular protein modification process; P:carbohydrate metabolic process; F:transferase activity | IPR000772 (PFAM); IPR001173 (PFAM); IPR003859 (PFAM); G3DSA:2.80.10.50 (GENE3D), G3DSA:3.90.550.10 (GENE3D), PTHR11675 (PANTHER), tmhmm (TMHMM), SSF53448 (SUPERFAMILY) |
| 670 | lib_dib_Filter15_IDBA_contig_16260_plus1 | 145 | fibrillin-2-like | 709 | 0 | 64.65% | 9 | P:signal transduction; P:regulation of biological process; F:calcium ion binding; C:proteinaceous extracellular matrix; P:multicellular organismal development; C:extracellular space; F:protein binding; P:anatomical structure morphogenesis; F:structural molecule activity | IPR000152 (PROSITE); IPR000742 (SMART); IPR001881 (PFAM); IPR013032 (PROSITE); IPR017878 (G3DSA:3.90.290.GENE3D); IPR018097 (PROSITE); IPR026823 (PFAM); G3DSA:2.10.25.10 (GENE3D), PTHR24039 (PANTHER), SSF57196 (SUPERFAMILY) |
| 671 | lib_dib_Filter15_IDBA_contig_16261_minus2 | 532 | deleted in malignant brain tumors 1 | 892 | 1.02E-43 | 55.40% | 3 | F:catalytic activity; F:receptor activity; C:cell | IPR001190 (PRINTS); IPR017448 (SMART); G3DSA:3.10.250.10 (GENE3D), PTHR19331 (PANTHER), tmhmm (TMHMM) |
| 672 | lib_dib_Filter15_IDBA_contig_16304_plus1 | 641 | multiple pdz domain protein | 1555 | 1.48E-140 | 64.90% | 3 | C:cytoplasm; C:plasma membrane; F:protein binding | IPR001478 (PFAM); G3DSA:2.30.42.10 (GENE3D), PTHR19964 (PANTHER), PTHR19964:SF10 (PANTHER) |
| 673 | lib_dib_Filter15_IDBA_contig_16310_minus1 | 160 | dna-directed rna polymerase ii subunit rpb1 | 261 | 1.47E-28 | 46.25% | 11 | P:transcription from RNA polymerase II promoter; C:DNA-directed RNA polymerase II, core complex; F:DNA binding; F:nucleotidyltransferase activity; F:transferase activity; P:transcription, DNA-dependent; F:DNA-directed RNA polymerase activity; F:structural constituent of cell wall; C:extracellular region; C:cell wall; P:multicellular organismal development | no IPS match |
| 674 | lib_dib_Filter15_IDBA_contig_16340_plus3 | 12348 | glucose-6-phosphate isomerase | 1763 | 0 | 84.05% | 12 | C:cytoplasm; P:anatomical structure morphogenesis; P:multicellular organismal development; P:biosynthetic process; P:carbohydrate metabolic process; C:extracellular space; F:catalytic activity; C:plasma membrane; F:receptor binding; P:generation of precursor metabolites and energy; P:catabolic process; C:nucleus | IPR001672 (PRINTS); IPR018189 (PROSITE); IPR023096 (G3DSA:1.10.1390.GENE3D); G3DSA:3.40.50.10490 (GENE3D), PTHR11469:SF0 (PANTHER), SSF53697 (SUPERFAMILY) |

| | | | | | | | | | | |
|---|---|---|---|---|---|---|---|---|---|---|
| 675 | lib_dib_Filter15_IDBA_contig_16345_minus3 | 347 | collagen alpha-4 chain-like | 744 | 1.09E-60 | 51.25% | 1 | F:protein binding | IPR002035 (PFAM); PR00453 (PRINTS), PR01217 (PRINTS), G3DSA:3.40.50.410 (GENE3D), PTHR22992 (PANTHER), SignalP-NN(euk) (SIGNALP), SSF53300 (SUPERFAMILY) | Yes |
| 676 | lib_dib_Filter15_IDBA_contig_16364_plus1 | 3937 | viral a-type inclusion protein | 236 | 5.86E-06 | 49.25% | 2 | P:vesicle-mediated transport; C:membrane | no IPS match | |
| 677 | lib_dib_Filter15_IDBA_contig_16388_minus2 | 134 | protocadherin fat 4-like | 725 | 1.93E-95 | 48.85% | 3 | F:calcium ion binding; P:biological_process; C:plasma membrane | IPR002126 (PRINTS); IPR015919 (SUPERFAMILY); IPR020894 (PROSITE); PTHR24027 (PANTHER), SignalP-NN(euk) (SIGNALP) | |
| 678 | lib_dib_Filter15_IDBA_contig_16389_minus2 | 226 | protocadherin fat 4-like | 1185 | 5.97E-101 | 48.55% | 3 | F:calcium ion binding; P:biological_process; C:plasma membrane | IPR002126 (PRINTS); IPR015919 (SUPERFAMILY); IPR020894 (PROSITE); PTHR24027 (PANTHER), SignalP-NN(euk) (SIGNALP), tmhmm (TMHMM) | |
| 679 | lib_dib_Filter15_IDBA_contig_16426_minus2 | 170 | cgmp-dependent protein isozyme 1 | 700 | 0 | 78.70% | 3 | F:nucleotide binding; P:cellular protein modification process; F:protein kinase activity | IPR000595 (PFAM); IPR000719 (PFAM); IPR000961 (SMART); IPR002290 (SMART); IPR002374 (PRINTS); IPR008271 (PROSITE); IPR011009 (SUPERFAMILY); IPR014710 (G3DSA:2.60.120.GENE3D); IPR017441 (PROSITE); IPR018488 (PROSITE); IPR018490 (SUPERFAMILY); IPR020635 (SMART); G3DSA:1.10.510.10 (GENE3D), G3DSA:3.30.200.20 (GENE3D), PTHR24353 (PANTHER), PTHR24353:SF1 (PANTHER) | |
| 680 | lib_dib_Filter15_IDBA_contig_16431_plus2 | 100 | kelch-like protein 9 | 699 | 0 | 57.55% | 3 | C:cell; P:biological_process; F:protein binding | IPR006652 (PFAM); IPR011333 (G3DSA:3.30.710.GENE3D); IPR011705 (PFAM); IPR013069 (PFAM); IPR015915 (G3DSA:2.120.10.GENE3D); PTHR24412 (PANTHER), PTHR24412:SF51 (PANTHER), PF13964 (PFAM), SSF117281 (SUPERFAMILY) | |
| 681 | lib_dib_Filter15_IDBA_contig_16432_plus2 | 3684 | von willebrand factor d and egf domain-containing protein | 1465 | 0 | 43.75% | 9 | F:chitin binding; P:chitin metabolic process; C:extracellular region; F:polysaccharide binding; F:calcium ion binding; F:scavenger receptor activity; F:carbohydrate binding; C:membrane; P:immune response | IPR001846 (PFAM); G3DSA:2.10.25.10 (GENE3D), PTHR22962 (PANTHER), SignalP-NN(euk) (SIGNALP), tmhmm (TMHMM) | |
| 682 | lib_dib_Filter15_IDBA_contig_16443_plus3 | 830 | protein isoform a | 1302 | 4.78E-74 | 61.00% | 27 | C:cell; P:response to external stimulus; P:response to stress; P:regulation of biological process; P:cell death; F:protein binding; F:protein kinase activity; F:receptor activity; P:cell proliferation; P:transport; P:cellular component organization; P:reproduction; P:cell communication; P:metabolic process; P:signal transduction; C:extracellular space; P:anatomical structure morphogenesis; P:multicellular organismal development; P:cell differentiation; P:biological_process; P:response to biotic stimulus; C:cytoplasm; C:plasma membrane; F:lipid binding; P:cell-cell signaling; F:cytoskeletal protein binding; F:nucleotide binding | IPR000719 (PROFILE); IPR001245 (PRINTS); IPR002011 (PROSITE); IPR002290 (SMART); IPR008266 (PROSITE); IPR011009 (SUPERFAMILY); IPR017441 (PROSITE); IPR020067 (G3DSA:1.10.2000.GENE3D); IPR020635 (SMART); G3DSA:1.10.510.10 (GENE3D), G3DSA:3.30.200.20 (GENE3D), PTHR24416 (PANTHER), SignalP-NN(euk) (SIGNALP), tmhmm (TMHMM) | |
| 683 | lib_dib_Filter15_IDBA_contig_16454_plus2 | 1376 | multiple epidermal growth factor-like domains protein 11 | 1193 | 0 | 54.55% | 2 | P:biological_process; F:protein binding | IPR000742 (SMART); IPR002049 (PFAM); IPR009030 (SUPERFAMILY); IPR011489 (PROFILE); IPR013032 (PROSITE); PR00011 (PRINTS), G3DSA:2.170.300.10 (GENE3D), PTHR24035 (PANTHER), PTHR24035:SF3 (PANTHER), tmhmm (TMHMM), SSF57196 (SUPERFAMILY) | |

| | | | | | | | | | |
|---|---|---|---|---|---|---|---|---|---|
| 684 | lib_dib_Filter15_IDBA_contig_16455_plus3 | 1357 | multiple epidermal growth factor-like domains protein 11 | 1169 | 0 | 54.45% | 3 | P:biological_process; C:cell; F:protein binding | IPR000742 (SMART); IPR002049 (PFAM); IPR009030 (SUPERFAMILY); IPR011489 (PROFILE); IPR013032 (PROSITE); PR00011 (PRINTS), G3DSA:2.170.300.10 (GENE3D), PTHR24035 (PANTHER), PTHR24035:SF3 (PANTHER), tmhmm (TMHMM), SSF57196 (SUPERFAMILY) |
| 685 | lib_dib_Filter15_IDBA_contig_16456_plus1 | 41 | multiple epidermal growth factor-like domains protein 11-like | 115 | 5.05E-32 | 63.20% | 4 | P:biological_process; P:anatomical structure morphogenesis; P:multicellular organismal development; F:protein binding | IPR000742 (SMART); IPR002049 (PFAM); IPR013032 (PFAM); PR00011 (PRINTS), G3DSA:2.170.300.10 (GENE3D), PTHR24035 (PANTHER), PTHR24035:SF3 (PANTHER), SSF57196 (SUPERFAMILY) |
| 686 | lib_dib_Filter15_IDBA_contig_16457_plus2 | 1029 | low-density lipoprotein receptor-related protein 6 isoform 2 | 1908 | 7.15E-84 | 43.60% | 7 | P:embryo development; P:multicellular organismal development; P:signal transduction; P:anatomical structure morphogenesis; P:cell differentiation; P:regulation of biological process; F:protein binding | IPR000033 (SMART); IPR000742 (SMART); IPR011042 (G3DSA:2.120.10.GENE3D); G3DSA:2.10.25.10 (GENE3D), PTHR10529 (PANTHER), tmhmm (TMHMM), SSF57196 (SUPERFAMILY), SSF63825 (SUPERFAMILY) |
| 687 | lib_dib_Filter15_IDBA_contig_16463_plus2 | 1923 | low quality protein: neuroglian-like | 1603 | 0 | 53.05% | 1 | F:protein binding | IPR003598 (SMART); IPR003599 (SMART); IPR003961 (PFAM); IPR007110 (PROFILE); IPR013098 (PFAM); IPR013783 (G3DSA:2.60.40.GENE3D); PTHR10489 (PANTHER), PF13895 (PFAM), tmhmm (TMHMM), SSF48726 (SUPERFAMILY) |
| 688 | lib_dib_Filter15_IDBA_contig_16485_minus1 | 138 | tyrosine-protein kinase transmembrane receptor ror1 | 695 | 0 | 72.60% | 6 | C:cell; F:nucleotide binding; P:cellular protein modification process; F:receptor activity; F:protein kinase activity; P:signal transduction | IPR000001 (G3DSA:2.40.20.GENE3D); IPR000719 (PROFILE); IPR001245 (PRINTS); IPR002011 (PROSITE); IPR002290 (SMART); IPR008266 (PROSITE); IPR011009 (SUPERFAMILY); IPR013806 (SUPERFAMILY); IPR017441 (PROSITE); IPR018056 (PROSITE); IPR020635 (SMART); PR00018 (PRINTS), G3DSA:1.10.510.10 (GENE3D), G3DSA:3.30.200.20 (GENE3D), PTHR24416 (PANTHER), PTHR24416:SF134 (PANTHER), tmhmm (TMHMM) |
| 689 | lib_dib_Filter15_IDBA_contig_16499_minus2 | 581 | serine threonine-protein kinase wnk3 | 1937 | 2.41E-177 | 78.55% | 19 | F:molecular_function; P:cell differentiation; P:multicellular organismal development; P:signal transduction; P:cellular protein modification process; F:protein binding; P:transport; P:regulation of biological process; C:cytoplasm; P:cell proliferation; F:enzyme regulator activity; P:biological_process; F:binding; F:protein kinase activity; F:signal transducer activity; P:metabolic process; C:cell; C:plasma membrane; F:nucleotide binding | IPR000719 (PFAM); IPR002290 (SMART); IPR008271 (PROSITE); IPR011009 (SUPERFAMILY); IPR020635 (SMART); IPR024678 (PFAM); G3DSA:1.10.510.10 (GENE3D), G3DSA:3.10.20.90 (GENE3D), G3DSA:3.30.200.20 (GENE3D), PTHR13902 (PANTHER), PTHR13902:SF13 (PANTHER) |

| | | | | | | | | |
|---|---|---|---|---|---|---|---|---|
| 690 | lib_dib_Filter15_IDBA_contig_16500_minus2 | 644 | serine threonine-protein kinase wnk3 | 2035 | 1.06E-176 | 78.90% | 22 | C:plasma membrane; P:cellular component organization; P:regulation of biological process; C:cell; P:signal transduction; P:cellular protein modification process; P:ion transport; F:binding; F:molecular_function; P:biological_process; P:cell death; F:protein kinase activity; F:signal transducer activity; C:cytoplasm; F:protein binding; P:transport; P:cell differentiation; P:multicellular organismal development; F:nucleotide binding; P:metabolic process; P:cell proliferation; F:enzyme regulator activity | IPR000719 (PFAM); IPR002290 (SMART); IPR008271 (PROSITE); IPR011009 (SUPERFAMILY); IPR020635 (SMART); IPR024678 (PFAM); G3DSA:1.10.510.10 (GENE3D), G3DSA:3.10.20.90 (GENE3D), G3DSA:3.30.200.20 (GENE3D), PTHR13902 (PANTHER), PTHR13902:SF13 (PANTHER) |
| 691 | lib_dib_Filter15_IDBA_contig_16513_plus1 | 5916 | neurexin-3-alpha isoform 2 | 2089 | 2.99E-119 | 58.25% | 8 | C:cell; P:anatomical structure morphogenesis; C:plasma membrane; P:transport; P:cell-cell signaling; P:cellular component organization; P:multicellular organismal development; P:regulation of biological process | IPR000742 (PROFILE); IPR001680 (PFAM); IPR001791 (PFAM); IPR008985 (SUPERFAMILY); IPR011047 (SUPERFAMILY); IPR013320 (G3DSA:2.60.120.GENE3D); IPR015943 (G3DSA:2.130.10.GENE3D); IPR017986 (PROFILE); IPR019775 (PROSITE); IPR027158 (PTHR10127:PANTHER); PTHR10127 (PANTHER), tmhmm (TMHMM), SSF57196 (SUPERFAMILY) |
| 692 | lib_dib_Filter15_IDBA_contig_16533_plus3 | 1716 | plasma alpha-l-fucosidase | 741 | 0 | 75.65% | 2 | F:hydrolase activity; P:carbohydrate metabolic process | IPR000933 (PANTHER); IPR013780 (G3DSA:2.60.40.GENE3D); IPR013781 (G3DSA:3.20.20.GENE3D); IPR016286 (PRINTS); IPR017853 (SUPERFAMILY); IPR018526 (PROSITE); PTHR10030:SF3 (PANTHER) |
| 693 | lib_dib_Filter15_IDBA_contig_16545_plus2 | 1751 | attractin-like protein 1-like | 1668 | 0 | 58.80% | 4 | F:protein binding; F:receptor activity; P:multicellular organismal development; C:cell | IPR000742 (SMART); IPR000859 (G3DSA:2.60.120.GENE3D); IPR002049 (PFAM); IPR003659 (SMART); IPR011043 (SUPERFAMILY); IPR013032 (PROSITE); IPR015915 (G3DSA:2.120.10.GENE3D); IPR016201 (SUPERFAMILY); G3DSA:2.10.25.10 (GENE3D), G3DSA:2.170.300.10 (GENE3D), PTHR10574 (PANTHER), PTHR10574:SF3 (PANTHER), PF13964 (PFAM), tmhmm (TMHMM), SSF57196 (SUPERFAMILY) |
| 694 | lib_dib_Filter15_IDBA_contig_16546_plus3 | 118 | protein tag-53 | 598 | 1.35E-43 | 66.45% | 2 | C:integral to membrane; C:membrane | PTHR24661 (PANTHER), PTHR24661:SF48 (PANTHER), tmhmm (TMHMM) |
| 695 | lib_dib_Filter15_IDBA_contig_16549_plus1 | 166 | membrane-bound transcription factor site-1 protease | 917 | 0 | 83.35% | 14 | C:Golgi apparatus; F:peptidase activity; P:regulation of biological process; P:protein transport; C:endoplasmic reticulum; P:biosynthetic process; P:lipid metabolic process; C:cell; P:metabolic process; P:signal transduction; P:response to stress; P:response to biotic stimulus; P:protein metabolic process; P:catabolic process | IPR000209 (G3DSA:3.40.50.GENE3D); IPR015500 (PRINTS); IPR022398 (PROSITE); IPR023828 (PROSITE); PTHR10795:SF30 (PANTHER), SignalP-NN(euk) (SIGNALP), tmhmm (TMHMM) |
| 696 | lib_dib_Filter15_IDBA_contig_16555_minus2 | 195 | lanb2 | 1109 | 0 | 60.45% | 1 | F:protein binding | IPR000034 (PFAM); IPR000742 (SMART); IPR002049 (PFAM); IPR013032 (PROSITE); PR00011 (PRINTS), G3DSA:2.10.25.10 (GENE3D), PTHR10574 (PANTHER), PTHR10574:SF50 (PANTHER), SSF57196 (SUPERFAMILY) |
| 697 | lib_dib_Filter15_IDBA_contig_16571_minus2 | 130 | ankyrin repeat domain protein | 687 | 1.01E-27 | 49.30% | 1 | F:protein binding | IPR002110 (SMART); IPR020683 (G3DSA:1.25.40.GENE3D); PTHR24198 (PANTHER) |

| | | | | | | | | | |
|---|---|---|---|---|---|---|---|---|---|
| 698 | lib_dib_Filter15_IDBA_contig_16572_minus1 | 927 | nk-tumor recognition protein | 1330 | 4.09E-14 | 73.40% | 9 | C:cytoplasm; C:nucleolus; P:nucleobase-containing compound metabolic process; C:nucleoplasm; F:binding; C:nucleus; P:cellular protein modification process; F:catalytic activity; P:protein metabolic process | IPR002130 (PFAM); G3DSA:2.40.100.10 (GENE3D), PTHR11071 (PANTHER), PTHR11071:SF30 (PANTHER) |
| 699 | lib_dib_Filter15_IDBA_contig_16588_plus1 | 2731 | deleted in malignant brain tumors 1 | 1724 | 0 | 44.20% | 4 | F:binding; C:intracellular; C:cell; F:receptor activity | IPR001190 (PRINTS); IPR017448 (SMART); G3DSA:3.10.250.10 (GENE3D), PTHR19331 (PANTHER), PTHR19331:SF118 (PANTHER) |
| 700 | lib_dib_Filter15_IDBA_contig_16589_plus1 | 62 | protein | 116 | 4.61E-16 | 54.65% | 2 | F:receptor activity; C:cell | IPR001190 (PFAM); IPR017448 (SMART); G3DSA:3.10.250.10 (GENE3D), PTHR19331 (PANTHER), PTHR19331:SF118 (PANTHER) |
| 701 | lib_dib_Filter15_IDBA_contig_16592_plus1 | 120 | beta- -galactosyl-o-glycosyl-glycoprotein beta- -n-acetylglucosaminyltransferase 3 | 710 | 3.63E-90 | 56.00% | 4 | C:extracellular space; F:transferase activity; P:response to endogenous stimulus; C:cell | IPR003406 (PFAM); PTHR19297 (PANTHER), PTHR19297:SF6 (PANTHER), tmhmm (TMHMM) |
| 702 | lib_dib_Filter15_IDBA_contig_16610_minus3 | 982 | krueppel-like factor 10 | 684 | 1.44E-45 | 73.85% | 10 | P:regulation of biological process; ; P:cell differentiation; P:cell proliferation; F:DNA binding; P:multicellular organismal development; P:cell death; P:cell cycle; C:nucleus; F:binding | IPR007087 (PROSITE); IPR013087 (G3DSA:3.30.160.GENE3D); IPR015880 (SMART); PTHR23223 (PANTHER), PF13465 (PFAM), SSF57667 (SUPERFAMILY) |
| 703 | lib_dib_Filter15_IDBA_contig_16612_minus1 | 2041 | cadherin 87a | 1891 | 1.94E-108 | 49.65% | 3 | F:calcium ion binding; P:biological_process; C:plasma membrane | IPR002126 (PRINTS); IPR015919 (SUPERFAMILY); IPR020894 (PROSITE); PTHR24027 (PANTHER), tmhmm (TMHMM) |
| 704 | lib_dib_Filter15_IDBA_contig_16619_plus1 | 128 | dipeptidyl peptidase 9 | 684 | 0 | 61.25% | 3 | P:protein metabolic process; P:catabolic process; C:cell | IPR002469 (PFAM); G3DSA:2.140.10.30 (GENE3D), G3DSA:3.40.50.1820 (GENE3D), PTHR11731 (PANTHER), PTHR11731:SF10 (PANTHER), SSF53474 (SUPERFAMILY), SSF82171 (SUPERFAMILY) |
| 705 | lib_dib_Filter15_IDBA_contig_16662_minus2 | 825 | zinc finger protein | 1519 | 7.37E-64 | 86.50% | 8 | P:anatomical structure morphogenesis; P:cellular component organization; P:cell differentiation; P:multicellular organismal development; P:regulation of biological process; F:binding; C:intracellular; F:DNA binding | IPR007087 (PROSITE); IPR013087 (G3DSA:3.30.160.GENE3D); IPR015880 (SMART); PTHR23223 (PANTHER), PTHR23223:SF63 (PANTHER), PF13465 (PFAM), SSF57667 (SUPERFAMILY) |
| 706 | lib_dib_Filter15_IDBA_contig_16663_minus2 | 785 | zinc finger protein | 1483 | 1.01E-63 | 86.50% | 8 | P:anatomical structure morphogenesis; P:cellular component organization; P:cell differentiation; P:multicellular organismal development; P:regulation of biological process; F:binding; C:intracellular; F:DNA binding | IPR007087 (PROSITE); IPR013087 (G3DSA:3.30.160.GENE3D); IPR015880 (SMART); PTHR23223 (PANTHER), PTHR23223:SF63 (PANTHER), PF13465 (PFAM), SSF57667 (SUPERFAMILY) |
| 707 | lib_dib_Filter15_IDBA_contig_16667_minus3 | 310 | receptor-type tyrosine-protein phosphatase alpha | 978 | 6.75E-128 | 54.70% | 4 | P:biological_process; P:multicellular organismal development; F:phosphoprotein phosphatase activity; P:cellular protein modification process | IPR000242 (PRINTS); IPR000387 (PROFILE); IPR003595 (SMART); IPR016130 (PROSITE); G3DSA:3.90.190.10 (GENE3D), PTHR19134 (PANTHER), tmhmm (TMHMM), SSF52799 (SUPERFAMILY) |

| | | | | | | | | | |
|---|---|---|---|---|---|---|---|---|---|
| 708 | lib_dib_Filter15_IDBA_contig_16671_minus2 | 26 | matrix metallopeptidase 2 (gelatinase 72kda 72kda type iv collagenase) | 189 | 3.11E-11 | 64.85% | 19 | P:anatomical structure morphogenesis; P:multicellular organismal development; P:transport; C:cytoplasmic membrane-bounded vesicle; C:Golgi apparatus; P:signal transduction; P:biological_process; P:cellular component organization; F:peptidase activity; F:protein binding; C:extracellular space; C:nuclear envelope; C:cell; C:endosome; C:plasma membrane; F:receptor activity; P:metabolic process; F:carbohydrate binding; C:extracellular region | IPR000562 (G3DSA:2.10.10.GENE3D); IPR013806 (SUPERFAMILY); PR00013 (PRINTS), G3DSA:2.60.120.260 (GENE3D), PTHR22918 (PANTHER), PTHR22918:SF0 (PANTHER) |
| 709 | lib_dib_Filter15_IDBA_contig_16690_plus1 | 387 | arylsulfatase b | 981 | 0 | 70.25% | 2 | F:hydrolase activity; P:metabolic process | IPR000917 (PFAM); IPR017849 (G3DSA:3.40.720.GENE3D); IPR017850 (SUPERFAMILY); IPR024607 (PROSITE); G3DSA:3.30.1120.10 (GENE3D), PTHR10342 (PANTHER), PTHR10342:SF19 (PANTHER), tmhmm (TMHMM) |
| 710 | lib_dib_Filter15_IDBA_contig_16699_minus2 | 210 | gamma-glutamyltransferase ywrd-like | 704 | 0 | 66.30% | 2 | F:transferase activity; | IPR000101 (PRINTS); SSF56235 (SUPERFAMILY) |
| 711 | lib_dib_Filter15_IDBA_contig_16712_plus2 | 468 | annexin a7 | 677 | 3.36E-115 | 70.20% | 7 | P:biological_process; P:behavior; C:organelle; F:protein binding; C:intracellular; F:calcium ion binding; F:lipid binding | IPR001464 (PRINTS); IPR018252 (PROSITE); IPR018502 (G3DSA:1.10.220.GENE3D) |
| 712 | lib_dib_Filter15_IDBA_contig_16717_minus3 | 258 | neuroligin- x-linked isoform 2 | 825 | 1.46E-134 | 52.45% | 8 | P:biological_process; C:cell; C:cellular_component; P:multicellular organismal development; F:protein binding; P:cellular component organization; P:behavior; C:plasma membrane | IPR002018 (PFAM); IPR019819 (PROSITE); IPR019826 (PROSITE); G3DSA:3.40.50.1820 (GENE3D), PTHR11559 (PANTHER), tmhmm (TMHMM), SSF53474 (SUPERFAMILY) |
| 713 | lib_dib_Filter15_IDBA_contig_16718_plus3 | 9224 | catalase | 1427 | 0 | 82.90% | 6 | P:response to stress; P:catabolic process; F:binding; F:catalytic activity; F:antioxidant activity; P:metabolic process | IPR002226 (PROSITE); IPR010582 (PFAM); IPR011614 (G3DSA:2.40.180.GENE3D); IPR018028 (PRINTS); IPR020835 (SUPERFAMILY); IPR024708 (PROSITE); PTHR11465:SF0 (PANTHER), tmhmm (TMHMM) |
| 714 | lib_dib_Filter15_IDBA_contig_16737_minus3 | 508 | isoform c | 990 | 9.27E-16 | 47.20% | 3 | F:calcium ion binding; F:actin binding; P:cytoskeleton organization | IPR000152 (PROSITE); IPR000742 (PFAM); IPR001881 (SMART); IPR002035 (PFAM); IPR005455 (PFAM); IPR013032 (PROSITE); IPR018097 (PROSITE); PR00010 (PRINTS), PR00453 (PRINTS), G3DSA:2.10.25.10 (GENE3D), G3DSA:3.30.450.30 (GENE3D), G3DSA:3.40.50.410 (GENE3D), PTHR24044 (PANTHER), tmhmm (TMHMM), SSF53300 (SUPERFAMILY), SSF57196 (SUPERFAMILY) |
| 715 | lib_dib_Filter15_IDBA_contig_16738_minus3 | 517 | isoform b | 1003 | 2.17E-18 | 47.55% | 3 | F:calcium ion binding; F:actin binding; P:cytoskeleton organization | IPR000152 (PROSITE); IPR000742 (PFAM); IPR001881 (SMART); IPR002035 (PFAM); IPR005455 (PFAM); IPR013032 (PROSITE); IPR018097 (PROSITE); PR00010 (PRINTS), PR00453 (PRINTS), G3DSA:2.10.25.10 (GENE3D), G3DSA:3.30.450.30 (GENE3D), G3DSA:3.40.50.410 (GENE3D), PTHR24044 (PANTHER), tmhmm (TMHMM), SSF53300 (SUPERFAMILY), SSF57196 (SUPERFAMILY) |
| 716 | lib_dib_Filter15_IDBA_contig_16743_plus2 | 367 | protein | 989 | 0 | 59.80% | 1 | P:carbohydrate metabolic process | IPR000772 (SUPERFAMILY); IPR001173 (PFAM); IPR003859 (PFAM); G3DSA:3.90.550.10 (GENE3D), PTHR11675 (PANTHER), tmhmm (TMHMM), SSF53448 (SUPERFAMILY) |

| | | | | | | | | | | |
|---|---|---|---|---|---|---|---|---|---|---|
| 717 | lib_dib_Filter15_IDBA_contig_16748_plus1 | 214 | plasma alpha-l-fucosidase | 698 | 0 | 69.20% | 2 | F:hydrolase activity; P:carbohydrate metabolic process | IPR000933 (PANTHER); IPR013780 (G3DSA:2.60.40.GENE3D); IPR013781 (G3DSA:3.20.20.GENE3D); IPR016286 (PRINTS); IPR017853 (SUPERFAMILY); PTHR10030:SF3 (PANTHER), tmhmm (TMHMM) | |
| 718 | lib_dib_Filter15_IDBA_contig_16750_plus1 | 196 | leucine-rich repeat neuronal protein 2 | 672 | 7.04E-64 | 43.25% | 1 | F:protein binding | IPR001611 (PROFILE); IPR003591 (SMART); IPR003599 (SMART); IPR007110 (PROFILE); IPR013783 (G3DSA:2.60.40.GENE3D); G3DSA:3.80.10.10 (GENE3D), PTHR24367 (PANTHER), PF13504 (PFAM), PF13855 (PFAM), SignalP-NN(euk) (SIGNALP), tmhmm (TMHMM), SSF48726 (SUPERFAMILY), SSF52058 (SUPERFAMILY) | Yes |
| 719 | lib_dib_Filter15_IDBA_contig_16756_minus2 | 1293 | tyrosine-protein kinase src42a | 1742 | 0 | 48.95% | 1 | F:protein binding | IPR000483 (SMART); IPR001611 (PFAM); IPR003591 (SMART); IPR026906 (PFAM); G3DSA:3.80.10.10 (GENE3D), PTHR24365 (PANTHER), PF13855 (PFAM), SM00364 (SMART), SM00365 (SMART), tmhmm (TMHMM), SSF52047 (SUPERFAMILY), SSF52058 (SUPERFAMILY) | |
| 720 | lib_dib_Filter15_IDBA_contig_16777_plus3 | 85 | zinc finger protein 135- partial | 669 | 1.97E-120 | 65.30% | 3 | C:nucleus; F:binding; F:nucleic acid binding | IPR007087 (PROSITE); IPR013087 (G3DSA:3.30.160.GENE3D); IPR015880 (SMART); PTHR24395 (PANTHER), PTHR24395:SF0 (PANTHER), PF13465 (PFAM), SSF57667 (SUPERFAMILY) | |
| 721 | lib_dib_Filter15_IDBA_contig_16785_minus1 | 1018 | retinoid-inducible serine carboxypeptidase | 737 | 2.32E-77 | 54.80% | 3 | F:peptidase activity; P:protein metabolic process; P:catabolic process | IPR001563 (PRINTS); G3DSA:3.40.50.1820 (GENE3D), PTHR11802:SF3 (PANTHER), SignalP-NN(euk) (SIGNALP), SSF53474 (SUPERFAMILY) | Yes |
| 722 | lib_dib_Filter15_IDBA_contig_16802_plus1 | 132 | atrial natriuretic peptide-converting enzyme | 476 | 9.96E-75 | 47.10% | 7 | P:biological_process; C:cell; F:receptor activity; F:peptidase activity; P:protein metabolic process; P:catabolic process; F:protein binding | IPR001190 (PROFILE); IPR001254 (PFAM); IPR002172 (G3DSA:4.10.400.GENE3D); IPR009003 (SUPERFAMILY); IPR017448 (SUPERFAMILY); IPR018114 (PROSITE); IPR023415 (PROSITE); PR00261 (PRINTS), G3DSA:2.40.10.10 (GENE3D), PTHR24256 (PANTHER) | |
| 723 | lib_dib_Filter15_IDBA_contig_16803_plus3 | 505 | | 1688 | 0 | 100% | 6 | C:cell; F:receptor activity; F:peptidase activity; P:protein metabolic process; P:catabolic process; F:protein binding | IPR000998 (PFAM); IPR001190 (PROFILE); IPR001254 (PFAM); IPR002172 (G3DSA:4.10.400.GENE3D); IPR008985 (SUPERFAMILY); IPR009003 (SUPERFAMILY); IPR017448 (SUPERFAMILY); IPR018114 (PROSITE); IPR023415 (PROSITE); PR00261 (PRINTS), G3DSA:2.40.10.10 (GENE3D), PTHR24256 (PANTHER) | |
| 724 | lib_dib_Filter15_IDBA_contig_16804_plus1 | 19 | ldl receptor ligand-binding repeat bearing | 112 | 1.29E-22 | 56.65% | 2 | F:catalytic activity; F:protein binding | IPR002172 (G3DSA:4.10.400.GENE3D); IPR023415 (PROSITE); PR00261 (PRINTS), PTHR10529 (PANTHER) | |
| 725 | lib_dib_Filter15_IDBA_contig_16805_minus1 | 6007 | atlastin-2 isoform 2 | 828 | 6.90E-114 | 66.10% | 10 | P:organelle organization; C:cell; P:cellular component organization; F:protein binding; P:transport; C:endoplasmic reticulum; P:regulation of biological process; P:cell death; F:hydrolase activity; F:nucleotide binding | IPR001315 (PROFILE); IPR003191 (SUPERFAMILY); IPR011029 (G3DSA:1.10.533.GENE3D); IPR015894 (PFAM); G3DSA:1.20.1000.10 (GENE3D), G3DSA:3.40.50.300 (GENE3D), PTHR10751 (PANTHER), PTHR10751:SF3 (PANTHER), SignalP-NN(euk) (SIGNALP), SSF52540 (SUPERFAMILY) | |
| 726 | lib_dib_Filter15_IDBA_contig_16812_minus1 | 2425 | protocadherin fat 4-like | 1214 | 2.32E-107 | 45.05% | 3 | F:calcium ion binding; P:biological_process; C:plasma membrane | IPR002126 (PRINTS); IPR015919 (SUPERFAMILY); IPR020894 (PROSITE); PTHR24027 (PANTHER) | |

| | | | | | | | | |
|---|---|---|---|---|---|---|---|---|
| 727 | lib_dib_Filter15_IDBA_contig_16814_minus2 | 1827 | protein tumorous imaginal mitochondrial-like | 1610 | 1.36E-167 | 73.85% | 7 | F:receptor binding; C:cytosol; P:signal transduction; C:mitochondrion; C:cell; F:protein binding; P:protein metabolic process | IPR001305 (G3DSA:2.10.230.GENE3D); IPR001623 (PRINTS); IPR002939 (PFAM); IPR008971 (SUPERFAMILY); IPR018253 (PROSITE); G3DSA:2.60.260.20 (GENE3D), PTHR24076 (PANTHER), PTHR24076:SF0 (PANTHER), tmhmm (TMHMM) |
| 728 | lib_dib_Filter15_IDBA_contig_16824_plus1 | 661 | deleted in malignant brain tumors 1 | 1237 | 2.49E-78 | 57.25% | 3 | F:peptidase activity; F:receptor activity; C:cell | IPR001190 (PRINTS); IPR017448 (SMART); G3DSA:3.10.250.10 (GENE3D), PTHR19331 (PANTHER), PTHR19331:SF118 (PANTHER), SignalP-NN(euk) (SIGNALP) |
| 729 | lib_dib_Filter15_IDBA_contig_16825_plus1 | 319 | deleted in malignant brain tumors 1 | 457 | 7.40E-49 | 55.80% | 3 | F:peptidase activity; F:receptor activity; C:cell | IPR001190 (PRINTS); IPR017448 (SMART); G3DSA:3.10.250.10 (GENE3D), PTHR19331 (PANTHER) |
| 730 | lib_dib_Filter15_IDBA_contig_16826_plus2 | 294 | deleted in malignant brain tumors 1 | 408 | 1.90E-49 | 56.05% | 3 | F:peptidase activity; F:receptor activity; C:cell | IPR001190 (PRINTS); IPR017448 (SMART); G3DSA:3.10.250.10 (GENE3D), PTHR19331 (PANTHER) |
| 731 | lib_dib_Filter15_IDBA_contig_16844_plus2 | 171 | leukocyte elastase inhibitor | 664 | 6.73E-109 | 62.60% | 4 | P:biological_process; P:protein metabolic process; P:catabolic process; F:enzyme regulator activity | IPR000215 (PANTHER); IPR023795 (PROSITE); IPR023796 (PFAM); G3DSA:2.30.39.10 (GENE3D), G3DSA:3.30.497.10 (GENE3D), SignalP-NN(euk) (SIGNALP) |
| 732 | lib_dib_Filter15_IDBA_contig_16848_minus2 | 737 | transforming growth beta receptor 1 | 823 | 0 | 75.85% | 26 | P:signal transduction; P:regulation of biological process; C:plasma membrane; P:metabolic process; P:cell death; P:embryo development; P:cell proliferation; F:protein binding; P:multicellular organismal development; P:biological_process; P:cellular protein modification process; F:receptor binding; P:response to abiotic stimulus; F:receptor activity; F:protein kinase activity; P:cellular component organization; P:anatomical structure morphogenesis; P:cell differentiation; P:response to stress; P:cell growth; P:reproduction; C:protein complex; P:response to endogenous stimulus; F:nucleotide binding; ; P:protein transport | IPR000472 (PFAM); IPR000719 (PFAM); IPR002290 (SMART); IPR003605 (PFAM); IPR008271 (PROSITE); IPR011009 (SUPERFAMILY); IPR017441 (PROSITE); IPR020635 (SMART); G3DSA:1.10.510.10 (GENE3D), G3DSA:2.10.60.10 (GENE3D), G3DSA:3.30.200.20 (GENE3D), PTHR23255 (PANTHER), SignalP-NN(euk) (SIGNALP), tmhmm (TMHMM), SSF57302 (SUPERFAMILY) |
| 733 | lib_dib_Filter15_IDBA_contig_16892_plus1 | 1467 | bifunctional protein ncoat isoform 1 | 1243 | 0 | 57.15% | 4 | P:transport; P:metabolic process; P:regulation of biological process; F:catalytic activity | IPR011496 (PFAM); IPR016181 (G3DSA:3.40.630.GENE3D); IPR017853 (SUPERFAMILY); PTHR13170 (PANTHER), PTHR13170:SF6 (PANTHER) |
| 734 | lib_dib_Filter15_IDBA_contig_16910_plus1 | 140 | adp-dependent glucokinase-like | 660 | 0 | 68.95% | 2 | F:transferase activity; P:carbohydrate metabolic process | IPR007666 (PANTHER); G3DSA:3.40.1190.20 (GENE3D), PTHR21208:SF0 (PANTHER), SSF53613 (SUPERFAMILY) |
| 735 | lib_dib_Filter15_IDBA_contig_16916_plus2 | 164 | delta-1-pyrroline-5-carboxylate mitochondrial | 659 | 0 | 77.75% | 7 | P:metabolic process; F:electron carrier activity; P:catabolic process; ; F:catalytic activity; C:mitochondrion; P:biosynthetic process | IPR005931 (TIGRFAMs); IPR015590 (PFAM); IPR016160 (PROSITE); IPR016161 (SUPERFAMILY); IPR016162 (G3DSA:3.40.605.GENE3D); IPR016163 (G3DSA:3.40.309.GENE3D); PTHR11699 (PANTHER), PTHR11699:SF52 (PANTHER) |
| 736 | lib_dib_Filter15_IDBA_contig_16917_minus2 | 185 | dipeptidyl aminopeptidase acylaminoacyl peptidase | 781 | 0 | 65.65% | 4 | P:protein metabolic process; P:catabolic process; F:peptidase activity; F:protein binding | IPR001375 (PFAM); IPR015943 (G3DSA:2.130.10.GENE3D); G3DSA:3.40.50.1820 (GENE3D), PTHR11731 (PANTHER), PTHR11731:SF7 (PANTHER), SSF53474 (SUPERFAMILY), SSF69322 (SUPERFAMILY) |

| | | | | | | | | | | |
|---|---|---|---|---|---|---|---|---|---|---|
| 737 | lib_dib_Filter15_IDBA_contig_16921_plus2 | 278 | serine threonine-protein kinase osr1 | 1049 | 0 | 76.70% | 7 | P:response to stress; F:protein binding; P:cellular protein modification process; F:nucleotide binding; F:binding; P:signal transduction; F:protein kinase activity | IPR000719 (PFAM); IPR002290 (SMART); IPR011009 (SUPERFAMILY); IPR017441 (PROSITE); IPR020635 (SMART); IPR024678 (PFAM); G3DSA:1.10.510.10 (GENE3D), G3DSA:3.10.20.90 (GENE3D), G3DSA:3.30.200.20 (GENE3D), PTHR24361 (PANTHER), PTHR24361:SF85 (PANTHER) | |
| 738 | lib_dib_Filter15_IDBA_contig_16933_minus3 | 607 | serine threonine-protein kinase pak 7-like | 1294 | 3.45E-173 | 89.30% | 4 | F:nucleotide binding; P:cellular protein modification process; F:protein kinase activity; F:protein binding | IPR000095 (PFAM); IPR000719 (PFAM); IPR002290 (SMART); IPR011009 (SUPERFAMILY); IPR017441 (PROSITE); IPR020635 (SMART); G3DSA:1.10.510.10 (GENE3D), G3DSA:3.30.200.20 (GENE3D), G3DSA:3.90.810.10 (GENE3D), PTHR24361 (PANTHER), PTHR24361:SF184 (PANTHER), tmhmm (TMHMM) | |
| 739 | lib_dib_Filter15_IDBA_contig_16940_minus1 | 142 | ankyrin repeat and fyve domain-containing protein 1 | 663 | 0 | 81.05% | 5 | F:binding; P:transport; P:cellular component organization; C:endosome; F:protein binding | IPR000306 (PFAM); IPR002110 (PFAM); IPR011011 (SUPERFAMILY); IPR013083 (G3DSA:3.30.40.GENE3D); IPR017455 (PROFILE); IPR020683 (G3DSA:1.25.40.GENE3D); PTHR24189 (PANTHER), PTHR24189:SF0 (PANTHER) | |
| 740 | lib_dib_Filter15_IDBA_contig_16953_minus2 | 162 | protein toll | 656 | 1.22E-39 | 43.30% | 2 | F:protein binding; P:signal transduction | IPR000157 (PFAM); IPR001611 (PROFILE); PR01537 (PRINTS), G3DSA:3.40.50.10140 (GENE3D), G3DSA:3.80.10.10 (GENE3D), PTHR24365 (PANTHER), PF13855 (PFAM), tmhmm (TMHMM), SSF52058 (SUPERFAMILY) | |
| 741 | lib_dib_Filter15_IDBA_contig_16964_plus2 | 237 | serine threonine-protein kinase endoribonuclease ire2 | 980 | 0 | 66.05% | 17 | F:protein binding; C:endoplasmic reticulum; C:nuclear envelope; F:nuclease activity; P:nucleobase-containing compound metabolic process; ; P:regulation of biological process; P:cellular protein modification process; P:cell cycle; F:protein kinase activity; P:metabolic process; P:signal transduction; P:response to stress; P:response to biotic stimulus; P:cell death; F:binding; F:nucleotide binding | IPR000719 (PFAM); IPR002290 (SMART); IPR006567 (SMART); IPR008271 (PROSITE); IPR010513 (PFAM); IPR011009 (SUPERFAMILY); IPR011047 (SUPERFAMILY); IPR018391 (SMART); IPR020635 (SMART); G3DSA:1.10.510.10 (GENE3D), G3DSA:2.140.10.10 (GENE3D), G3DSA:3.30.200.20 (GENE3D), PTHR13954 (PANTHER), PTHR13954:SF1 (PANTHER), tmhmm (TMHMM) | |
| 742 | lib_dib_Filter15_IDBA_contig_16969_minus1 | 360 | tandem-repeat galectin | 326 | 4.26E-33 | 58.25% | 1 | F:carbohydrate binding | IPR001079 (PFAM); IPR008985 (SUPERFAMILY); IPR013320 (G3DSA:2.60.120.GENE3D); PTHR11346 (PANTHER) | |
| 743 | lib_dib_Filter15_IDBA_contig_16971_minus2 | 283 | microfibril-associated glycoprotein 4-like | 654 | 8.90E-44 | 55.05% | 2 | P:biological_process; F:binding | IPR002181 (PFAM); IPR014715 (G3DSA:4.10.530.GENE3D); IPR014716 (G3DSA:3.90.215.GENE3D); PTHR19143 (PANTHER), SignalP-NN(euk) (SIGNALP) | Yes |
| 744 | lib_dib_Filter15_IDBA_contig_16990_plus1 | 1791 | protein roadkill | 1310 | 0 | 51.05% | 2 | F:carbohydrate binding; F:protein binding | IPR001304 (PFAM); IPR003598 (SMART); IPR003599 (SMART); IPR003961 (PFAM); IPR007110 (PROFILE); IPR013098 (PFAM); IPR013783 (G3DSA:2.60.40.GENE3D); IPR016186 (G3DSA:3.10.100.GENE3D); IPR016187 (SUPERFAMILY); PTHR10489 (PANTHER), PF13895 (PFAM), SignalP-NN(euk) (SIGNALP), tmhmm (TMHMM), SSF48726 (SUPERFAMILY) | Yes |
| 745 | lib_dib_Filter15_IDBA_contig_16991_plus1 | 733 | contactin | 488 | 2.57E-85 | 53.45% | 4 | P:biological_process; C:plasma membrane; P:multicellular organismal development; F:protein binding | IPR003961 (PFAM); IPR013783 (G3DSA:2.60.40.GENE3D); PR00014 (PRINTS), PTHR10489 (PANTHER), PTHR10489:SF48 (PANTHER), tmhmm (TMHMM) | |

| | | | | | | | | | |
|---|---|---|---|---|---|---|---|---|---|
| 746 | lib_dib_Filter15_IDBA_contig_17016_plus1 | 148 | dnaj homolog subfamily c member 1-like | 671 | 2.69E-40 | 69.10% | 4 | P:protein metabolic process; F:protein binding; F:chromatin binding; F:DNA binding | IPR001005 (PFAM); IPR001623 (PRINTS); IPR009057 (G3DSA:1.10.10.GENE3D); IPR017877 (PROFILE); IPR018253 (PROSITE); PTHR24078 (PANTHER), PTHR24078:SF10 (PANTHER), SignalP-NN(euk) (SIGNALP), tmhmm (TMHMM) | |
| 747 | lib_dib_Filter15_IDBA_contig_17038_minus3 | 654 | integrin alpha-ps1 | 1627 | 0 | 52.85% | 3 | P:biological_process; C:protein complex; C:plasma membrane | IPR000413 (PRINTS); IPR013517 (PFAM); IPR013519 (SMART); IPR013649 (PFAM); IPR018184 (PROSITE); G3DSA:1.20.5.930 (GENE3D), G3DSA:2.130.10.130 (GENE3D), G3DSA:2.60.40.1460 (GENE3D), G3DSA:2.60.40.1510 (GENE3D), G3DSA:2.60.40.1530 (GENE3D), PTHR23220 (PANTHER), PTHR23220:SF40 (PANTHER), SignalP-NN(euk) (SIGNALP), tmhmm (TMHMM), SSF69179 (SUPERFAMILY), SSF69318 (SUPERFAMILY) | Yes |
| 748 | lib_dib_Filter15_IDBA_contig_17065_plus3 | 1374 | epidermal growth factor receptor | 1670 | 0 | 59.00% | 6 | C:cell; F:nucleotide binding; P:cellular protein modification process; F:receptor activity; F:protein kinase activity; P:signal transduction | [illegible] (PFAM); IPR000719 (PROFILE); IPR001245 (PRINTS); IPR002290 (SMART); IPR006211 (PFAM); IPR006212 (SMART); IPR008266 (PROSITE); IPR009030 (SUPERFAMILY); IPR011009 (SUPERFAMILY); IPR017441 (PROSITE); IPR020635 (SMART); G3DSA:1.10.510.10 (GENE3D), G3DSA:2.10.220.10 (GENE3D), G3DSA:3.30.200.20 (GENE3D), G3DSA:3.80.20.20 (GENE3D), G3DSA:4.10.1140.10 (GENE3D), PTHR24416 (PANTHER), PTHR24416:SF91 (PANTHER), tmhmm (TMHMM), [illegible] (SUPERFAMILY) | |
| 749 | lib_dib_Filter15_IDBA_contig_17066_plus3 | 10 | epidermal growth factor partial | 118 | 1.46E-44 | 76.15% | 33 | P:multicellular organismal development; P:signal transduction; P:anatomical structure morphogenesis; P:cellular component organization; P:cell differentiation; P:biosynthetic process; P:regulation of biological process; F:DNA binding; P:response to stress; P:DNA metabolic process; P:cellular protein modification process; P:protein metabolic process; P:catabolic process; P:biological_process; P:metabolic process; C:plasma membrane; C:extracellular space; C:cell; C:protein complex; P:cell proliferation; F:protein kinase activity; F:signal transducer activity; P:cell cycle; P:response to abiotic stimulus; F:receptor activity; F:protein binding; C:cytoplasmic membrane-bounded vesicle; F:actin binding; F:enzyme regulator activity; P:cell death; C:nucleus; C:endosome; F:nucleotide binding | IPR000719 (PROFILE); IPR001245 (PFAM); IPR011009 (SUPERFAMILY); IPR020635 (SMART); G3DSA:1.10.510.10 (GENE3D), G3DSA:3.30.200.20 (GENE3D), PTHR24416 (PANTHER), PTHR24416:SF91 (PANTHER) | |
| 750 | lib_dib_Filter15_IDBA_contig_17079_minus1 | 1060 | a disintegrin and metalloproteinase with thrombospondin motifs 3 | 1587 | 5.35E-161 | 53.50% | 5 | F:peptidase activity; P:protein metabolic process; P:catabolic process; F:binding; C:proteinaceous extracellular matrix | IPR000884 (PFAM); IPR001590 (PFAM); IPR002870 (PFAM); IPR010294 (PFAM); IPR010909 (PROFILE); IPR013273 (PRINTS); IPR024079 (G3DSA:3.40.390.GENE3D); G3DSA:2.20.100.10 (GENE3D), PTHR13723 (PANTHER), SignalP-NN(euk) (SIGNALP), SSF55486 (SUPERFAMILY) | Yes |

| | | | | | | | | | | |
|---|---|---|---|---|---|---|---|---|---|---|
| 751 | lib_dib_Filter15_IDBA_contig_17121_plus3 | 457 | dbh-like 1 | 642 | 2.76E-110 | 51.25% | 7 | F:binding; P:metabolic process; F:catalytic activity; ; P:carbohydrate metabolic process; P:catabolic process; F:carbohydrate binding | IPR000323 (G3DSA:2.60.120.GENE3D); IPR000945 (PRINTS); IPR005018 (PFAM); IPR008960 (SUPERFAMILY); IPR008977 (SUPERFAMILY); IPR014784 (G3DSA:2.60.120.GENE3D); IPR015920 (G3DSA:2.60.40.GENE3D); PF03712 (PFAM), SignalP-NN(euk) (SIGNALP), tmhmm (TMHMM) | |
| 752 | lib_dib_Filter15_IDBA_contig_17143_plus1 | 380 | wap four-disulfide core domain protein 1 | 641 | 4.57E-26 | 62.85% | 7 | C:extracellular space; P:regulation of biological process; P:cell proliferation; P:biological_process; P:response to endogenous stimulus; P:cell growth; F:enzyme regulator activity | IPR008197 (PFAM); PTHR14308 (PANTHER), tmhmm (TMHMM) | |
| 753 | lib_dib_Filter15_IDBA_contig_17197_plus1 | 191 | 5 -amp-activated protein kinase catalytic subunit alpha-2 | 639 | 0 | 77.75% | 3 | F:protein kinase activity; P:cellular protein modification process; F:nucleotide binding | IPR000719 (PFAM); IPR002290 (SMART); IPR008271 (PROSITE); IPR011009 (SUPERFAMILY); IPR020635 (SMART); G3DSA:1.10.510.10 (GENE3D), G3DSA:3.30.200.20 (GENE3D), PTHR24343 (PANTHER), PTHR24343:SF82 (PANTHER) | |
| 754 | lib_dib_Filter15_IDBA_contig_17204_plus2 | 547 | trehalase isoform 1 | 861 | 3.13E-155 | 63.35% | 5 | C:plasma membrane; P:biological_process; P:carbohydrate metabolic process; P:catabolic process; F:hydrolase activity | IPR001661 (PRINTS); IPR008928 (SUPERFAMILY); IPR018232 (PROSITE); PTHR23403:SF1 (PANTHER), SignalP-NN(euk) (SIGNALP), tmhmm (TMHMM) | Yes |
| 755 | lib_dib_Filter15_IDBA_contig_17220_plus2 | 974 | usherin | 1821 | 1.60E-142 | 47.55% | 8 | P:biological_process; P:cell differentiation; F:protein binding; P:multicellular organismal development; C:intracellular; F:nucleotide binding; P:cellular protein modification process; F:protein kinase activity | IPR000719 (PROFILE); IPR000742 (SMART); IPR001245 (PRINTS); IPR002049 (PFAM); IPR002290 (SMART); IPR002909 (PFAM); IPR008211 (PFAM); IPR008266 (PROSITE); IPR008985 (SUPERFAMILY); IPR011009 (SUPERFAMILY); IPR013320 (G3DSA:2.60.120.GENE3D); IPR013783 (G3DSA:2.60.40.GENE3D); IPR014756 (SUPERFAMILY); IPR017441 (PROSITE); IPR020635 (SMART); PR00011 (PRINTS), G3DSA:1.10.510.10 (GENE3D), G3DSA:2.10.25.10 (GENE3D), G3DSA:2.60.120.260 (GENE3D), G3DSA:3.30.200.20 (GENE3D), PTHR24416 (PANTHER), PF13385 (PFAM), SignalP-NN(euk) (SIGNALP), tmhmm (TMHMM), SSF57196 (SUPERFAMILY) | Yes |
| 756 | lib_dib_Filter15_IDBA_contig_17231_minus2 | 601 | sulfhydryl oxidase 1 | 701 | 1.72E-90 | 49.65% | 7 | C:organelle; C:intracellular; C:cell; F:catalytic activity; P:metabolic process; P:cellular homeostasis; P:regulation of biological process | IPR006863 (G3DSA:1.20.120.GENE3D); IPR012336 (G3DSA:3.40.30.GENE3D); IPR013766 (PFAM); IPR017905 (PROFILE); PTHR22897 (PANTHER) | |
| 757 | lib_dib_Filter15_IDBA_contig_17232_minus2 | 863 | sulfhydryl oxidase 1 | 1028 | 3.06E-88 | 49.65% | 7 | C:organelle; C:intracellular; C:cell; F:catalytic activity; P:metabolic process; P:cellular homeostasis; P:regulation of biological process | IPR006863 (G3DSA:1.20.120.GENE3D); IPR012336 (G3DSA:3.40.30.GENE3D); IPR013766 (PFAM); IPR017905 (PROFILE); PTHR22897 (PANTHER), tmhmm (TMHMM) | |
| 758 | lib_dib_Filter15_IDBA_contig_17233_minus2 | 151 | udp- c:polypeptide n- | 637 | 0 | 60.55% | 12 | F:transferase activity; F:transferase activity, transferring glycosyl groups; F:carbohydrate binding; C:integral to membrane; C:membrane; C:Golgi apparatus; F:polypeptide N-acetylgalactosaminyltransferase activity; P:protein O-linked glycosylation via threonine; P:protein O-linked glycosylation via serine; C:perinuclear region of cytoplasm; P:protein O-linked glycosylation; F:manganese ion binding | IPR000772 (PROFILE); IPR001173 (PFAM); G3DSA:3.90.550.10 (GENE3D), PTHR11675 (PANTHER), SSF53448 (SUPERFAMILY) | |

| | | | | | | | | | | |
|---|---|---|---|---|---|---|---|---|---|---|
| 759 | lib_dib_Filter15_IDBA_contig_17234_plus2 | 136 | aminopeptidase n | 637 | 4.92E-87 | 50.95% | 5 | C:cytoplasm; P:protein metabolic process; P:catabolic process; F:peptidase activity; F:binding | IPR001930 (PANTHER); IPR014782 (PRINTS); G3DSA:1.10.390.10 (GENE3D), SignalP-NN(euk) (SIGNALP), tmhmm (TMHMM), SSF55486 (SUPERFAMILY), SSF63737 (SUPERFAMILY) | |
| 760 | lib_dib_Filter15_IDBA_contig_17236_plus1 | 374 | carboxypeptidase b | 725 | 1.33E-91 | 53.45% | 4 | F:peptidase activity; P:protein metabolic process; P:catabolic process; F:binding | IPR000834 (PRINTS); IPR003146 (G3DSA:3.30.70.GENE3D); IPR009020 (SUPERFAMILY); G3DSA:3.40.630.10 (GENE3D), PTHR11705 (PANTHER), tmhmm (TMHMM), SSF53187 (SUPERFAMILY) | |
| 761 | lib_dib_Filter15_IDBA_contig_17238_plus2 | 8734 | glucose dehydrogenase | 842 | 0 | 60.45% | 2 | F:nucleotide binding; P:metabolic process | IPR000172 (PFAM); IPR007867 (PFAM); G3DSA:3.50.50.60 (GENE3D), PTHR11552 (PANTHER), PTHR11552:SF10 (PANTHER), SSF51905 (SUPERFAMILY), SSF54373 (SUPERFAMILY) | |
| 762 | lib_dib_Filter15_IDBA_contig_17247_plus2 | 394 | transcriptional repressor protein yy1 | 684 | 1.68E-163 | 64.40% | 4 | C:plasma membrane; C:nucleus; F:binding; F:nucleic acid binding | IPR007087 (PROSITE); IPR013087 (G3DSA:3.30.160.GENE3D); IPR015880 (SMART); PTHR14003 (PANTHER), PF13465 (PFAM), PF13894 (PFAM), SSF57667 (SUPERFAMILY) | |
| 763 | lib_dib_Filter15_IDBA_contig_17277_plus2 | 1307 | nad mitochondrial | 634 | 0 | 76.95% | 6 | F:catalytic activity; F:nucleotide binding; P:ion transport; C:cell; C:mitochondrion; P:metabolic process | IPR007698 (PFAM); IPR007886 (PFAM); IPR016040 (G3DSA:3.40.50.GENE3D); IPR024605 (PFAM); IPR026255 (PIR); G3DSA:3.40.50.1770 (GENE3D), PTHR10160 (PANTHER), PTHR10160:SF14 (PANTHER), SignalP-NN(euk) (SIGNALP), tmhmm (TMHMM), SSF51735 (SUPERFAMILY), SSF52283 (SUPERFAMILY) | |
| 764 | lib_dib_Filter15_IDBA_contig_17285_plus2 | 484 | heparanase isoform 1 | 1136 | 2.62E-119 | 53.45% | 10 | F:protein binding; P:metabolic process; C:lysosome; F:hydrolase activity; P:regulation of biological process; P:response to external stimulus; P:response to stress; P:multicellular organismal development; C:cell; P:carbohydrate metabolic process | IPR005199 (PANTHER); IPR013781 (G3DSA:3.20.20.GENE3D); IPR017853 (SUPERFAMILY); PTHR14363:SF1 (PANTHER), SignalP-NN(euk) (SIGNALP) | Yes |
| 765 | lib_dib_Filter15_IDBA_contig_17289_minus1 | 244 | btb poz domain-containing protein 9 | 695 | 0 | 78.75% | 2 | P:biological_process; F:protein binding | IPR000210 (SMART); IPR000421 (PFAM); IPR008979 (SUPERFAMILY); IPR011333 (G3DSA:3.30.710.GENE3D); IPR011705 (PFAM); IPR013069 (PFAM); G3DSA:2.60.120.260 (GENE3D), PTHR24413 (PANTHER), PTHR24413:SF6 (PANTHER), tmhmm (TMHMM) | |
| 766 | lib_dib_Filter15_IDBA_contig_17290_minus2 | 268 | a disintegrin and metalloproteinase with thrombospondin motifs 9 | 1148 | 0 | 56.25% | 6 | P:biological_process; P:protein metabolic process; P:catabolic process; C:proteinaceous extracellular matrix; F:peptidase activity; F:binding | IPR000884 (PFAM); IPR012314 (PFAM); G3DSA:2.20.100.10 (GENE3D), PTHR13723 (PANTHER), PTHR13723:SF76 (PANTHER) | |
| 767 | lib_dib_Filter15_IDBA_contig_17321_minus2 | 170 | ankyrin repeat protein | 699 | 1.98E-16 | 51.50% | 1 | F:protein binding | IPR001496 (PFAM); IPR002110 (SMART); IPR020683 (G3DSA:1.25.40.GENE3D); PTHR24188 (PANTHER), PF13857 (PFAM) | |
| 768 | lib_dib_Filter15_IDBA_contig_17328_plus3 | 768 | low-density lipoprotein receptor-related protein 12 isoform 2 | 1164 | 1.45E-142 | 54.35% | 2 | C:cell; F:protein binding | IPR000859 (G3DSA:2.60.120.GENE3D); IPR002172 (G3DSA:4.10.400.GENE3D); IPR023415 (PROSITE); PR00261 (PRINTS), PTHR10529 (PANTHER), PTHR10529:SF97 (PANTHER), tmhmm (TMHMM) | |
| 769 | lib_dib_Filter15_IDBA_contig_17329_plus1 | 213 | hypothetical protein CAPTEDRAFT_220926 | 756 | 1.04E-04 | 63.25% | 0 | - | PTHR14429 (PANTHER), PTHR14429:SF9 (PANTHER) | |

| | | | | | | | | | | |
|---|---|---|---|---|---|---|---|---|---|---|
| 770 | lib_dib_Filter15_IDBA_contig_17339_plus2 | 1447 | ferric-chelate reductase 1 | 727 | 3.26E-18 | 47.60% | 11 | F:molecular_function; P:defense response to protozoan; C:extracellular region; P:innate immune response; P:defense response to bacterium; P:oxidation-reduction process; F:oxidoreductase activity; C:integral to membrane; C:membrane; P:electron transport chain; P:transport | IPR002861 (PFAM); PTHR23130 (PANTHER), PTHR23130:SF6 (PANTHER), tmhmm (TMHMM) | |
| 771 | lib_dib_Filter15_IDBA_contig_17340_plus1 | 970 | ferric-chelate reductase 1-like | 630 | 1.38E-16 | 47.05% | 6 | P:oxidation-reduction process; F:oxidoreductase activity; C:integral to membrane; C:membrane; P:electron transport chain; P:transport | IPR002861 (PFAM); PTHR23130 (PANTHER), PTHR23130:SF6 (PANTHER), SignalP-NN(euk) (SIGNALP), tmhmm (TMHMM) | Yes |
| 772 | lib_dib_Filter15_IDBA_contig_17382_plus3 | 263 | dehydrogenase reductase sdr family member 1 | 638 | 1.93E-131 | 71.40% | 3 | P:metabolic process; F:catalytic activity; F:nucleotide binding | IPR002198 (PFAM); IPR002347 (PRINTS); IPR016040 (G3DSA:3.40.50.GENE3D); PTHR24322 (PANTHER), PTHR24322:SF73 (PANTHER), tmhmm (TMHMM), SSF51735 (SUPERFAMILY) | |
| 773 | lib_dib_Filter15_IDBA_contig_17420_minus3 | 27239 | heat shock protein 70 | 1319 | 0 | 95.90% | 2 | F:nucleotide binding; P:response to stress | IPR013126 (PRINTS); IPR018181 (PROSITE); G3DSA:1.20.1270.10 (GENE3D), G3DSA:2.60.34.10 (GENE3D), G3DSA:3.30.30.30 (GENE3D), G3DSA:3.30.420.40 (GENE3D), G3DSA:3.90.640.10 (GENE3D), PTHR19375 (PANTHER), SSF100920 (SUPERFAMILY), SSF100934 (SUPERFAMILY), SSF53067 (SUPERFAMILY) | |
| 774 | lib_dib_Filter15_IDBA_contig_17425_minus1 | 291 | endothelin converting enzyme 1 | 1070 | 4.30E-155 | 56.20% | 17 | P:catabolic process; P:anatomical structure morphogenesis; P:embryo development; P:multicellular organismal development; C:cell; C:cytoplasmic membrane-bounded vesicle; P:cell death; P:signal transduction; P:metabolic process; P:regulation of biological process; P:protein metabolic process; P:response to stress; C:plasma membrane; C:endosome; C:cytoplasm; F:protein binding; F:peptidase activity | IPR000718 (PANTHER); IPR008753 (PFAM); IPR018497 (PRINTS); IPR024079 (G3DSA:3.40.390.GENE3D); SignalP-NN(euk) (SIGNALP), tmhmm (TMHMM), SSF55486 (SUPERFAMILY) | |
| 775 | lib_dib_Filter15_IDBA_contig_17426_minus1 | 279 | endothelin converting enzyme 1 | 1038 | 2.02E-155 | 56.20% | 17 | P:catabolic process; P:anatomical structure morphogenesis; P:embryo development; P:multicellular organismal development; C:cell; C:cytoplasmic membrane-bounded vesicle; P:cell death; P:signal transduction; P:metabolic process; P:regulation of biological process; P:protein metabolic process; P:response to stress; C:plasma membrane; C:endosome; C:cytoplasm; F:protein binding; F:peptidase activity | IPR000718 (PANTHER); IPR008753 (PFAM); IPR018497 (PRINTS); IPR024079 (G3DSA:3.40.390.GENE3D); SignalP-NN(euk) (SIGNALP), tmhmm (TMHMM), SSF55486 (SUPERFAMILY) | |
| 776 | lib_dib_Filter15_IDBA_contig_17436_minus1 | 445 | arylsulfatase b | 914 | 0 | 71.05% | 2 | F:hydrolase activity; P:metabolic process | IPR000917 (PFAM); IPR017849 (G3DSA:3.40.720.GENE3D); IPR017850 (SUPERFAMILY); IPR024607 (PROSITE); G3DSA:3.30.1120.10 (GENE3D), PTHR10342 (PANTHER), PTHR10342:SF19 (PANTHER), tmhmm (TMHMM) | |
| 777 | lib_dib_Filter15_IDBA_contig_17444_plus1 | 646 | beta-galactosidase-1-like protein 2-like | 1049 | 0 | 60.00% | 2 | F:hydrolase activity; P:carbohydrate metabolic process | IPR001944 (PRINTS); IPR008979 (SUPERFAMILY); IPR013781 (G3DSA:3.20.20.GENE3D); IPR017853 (SUPERFAMILY); IPR019801 (PROSITE); G3DSA:2.60.120.260 (GENE3D), PTHR23421:SF14 (PANTHER) | |

| | | | | | | | | | |
|---|---|---|---|---|---|---|---|---|---|
| 778 | lib_dib_Filter15_IDBA_contig_17455_plus1 | 458 | kelch-like protein 17 | 863 | 0 | 73.50% | 1 | F:protein binding | IPR000210 (SMART); IPR006652 (PFAM); IPR011333 (G3DSA:3.30.710.GENE3D); IPR011705 (PFAM); IPR013069 (PFAM); IPR015916 (G3DSA:2.130.10.GENE3D); PR00501 (PRINTS), PTHR24412 (PANTHER), PTHR24412:SF20 (PANTHER), SSF117281 (SUPERFAMILY) |
| 779 | lib_dib_Filter15_IDBA_contig_17518_plus1 | 424 | protein toll | 1037 | 6.29E-72 | 47.90% | 2 | F:protein binding; P:signal transduction | IPR000157 (PFAM); IPR000483 (SMART); IPR001611 (PFAM); IPR003591 (SMART); IPR026906 (PFAM); PR01537 (PRINTS), G3DSA:3.40.50.10140 (GENE3D), G3DSA:3.80.10.10 (GENE3D), PTHR24365 (PANTHER), PF13855 (PFAM), SM00364 (SMART), tmhmm (TMHMM), SSF52058 (SUPERFAMILY) |
| 780 | lib_dib_Filter15_IDBA_contig_17519_plus1 | 556 | protein toll | 1405 | 1.10E-70 | 48.05% | 2 | F:protein binding; P:signal transduction | IPR000157 (PFAM); IPR000483 (SMART); IPR001611 (PFAM); IPR003591 (SMART); IPR026906 (PFAM); PR01537 (PRINTS), G3DSA:3.40.50.10140 (GENE3D), G3DSA:3.80.10.10 (GENE3D), PTHR24365 (PANTHER), PF13855 (PFAM), SM00364 (SMART), tmhmm (TMHMM), SSF52058 (SUPERFAMILY) |
| 781 | lib_dib_Filter15_IDBA_contig_17520_plus2 | 562 | protein toll | 1414 | 1.41E-70 | 48.00% | 2 | F:protein binding; P:signal transduction | IPR000157 (PFAM); IPR000483 (SMART); IPR001611 (PFAM); IPR003591 (SMART); IPR026906 (PFAM); PR01537 (PRINTS), G3DSA:3.40.50.10140 (GENE3D), G3DSA:3.80.10.10 (GENE3D), PTHR24365 (PANTHER), PF13855 (PFAM), SM00364 (SMART), tmhmm (TMHMM), SSF52058 (SUPERFAMILY) |
| 782 | lib_dib_Filter15_IDBA_contig_17559_minus2 | 696 | phospholipase b-like 1 | 620 | 0 | 70.55% | 6 | F:hydrolase activity; P:lipid catabolic process; C:extracellular region; F:molecular_function; P:biological_process; C:cellular_component | IPR007000 (PANTHER) |
| 783 | lib_dib_Filter15_IDBA_contig_17580_plus3 | 270 | proteasomal atpase-associated factor 1 | 638 | 2.20E-144 | 63.40% | 1 | F:protein binding | IPR001680 (PFAM); IPR015943 (G3DSA:2.130.10.GENE3D); IPR017986 (PROFILE); IPR019775 (PROSITE); PTHR22844 (PANTHER), PTHR22844:SF11 (PANTHER) |
| 784 | lib_dib_Filter15_IDBA_contig_17633_plus1 | 399 | dual specificity mitogen-activated protein kinase kinase 4 | 864 | 5.61E-166 | 87.90% | 16 | P:signal transduction; P:response to stress; P:cellular protein modification process; F:protein kinase activity; C:cytosol; P:response to external stimulus; P:response to abiotic stimulus; C:cytoplasm; C:cell; P:regulation of biological process; P:DNA metabolic process; P:biosynthetic process; P:cell death; C:nucleus; F:nucleotide binding; F:protein binding | IPR000719 (PFAM); IPR002290 (SMART); IPR008271 (PROSITE); IPR011009 (SUPERFAMILY); IPR017441 (PROSITE); IPR020635 (SMART); G3DSA:1.10.510.10 (GENE3D), G3DSA:3.30.200.20 (GENE3D), PTHR24360 (PANTHER), PTHR24360:SF35 (PANTHER), SignalP-NN(euk) (SIGNALP), tmhmm (TMHMM) |
| 785 | lib_dib_Filter15_IDBA_contig_17640_minus1 | 307 | abhydrolase domain-containing protein 2 | 713 | 1.28E-147 | 66.55% | 4 | P:response to external stimulus; P:response to stress; P:regulation of biological process; F:binding | IPR000073 (PFAM); IPR017896 (PROFILE); G3DSA:3.40.50.1820 (GENE3D), PTHR10794 (PANTHER), PTHR10794:SF9 (PANTHER), SignalP-NN(euk) (SIGNALP), tmhmm (TMHMM), SSF53474 (SUPERFAMILY) |
| 786 | lib_dib_Filter15_IDBA_contig_17641_plus3 | 1853 | collagen alpha-4 chain | 1388 | 0 | 52.15% | 2 | F:protein binding; F:calcium ion binding | IPR000152 (PROSITE); IPR000742 (PFAM); IPR001881 (SMART); IPR002035 (PFAM); IPR013032 (PROSITE); PR00453 (PRINTS), G3DSA:2.10.25.10 (GENE3D), G3DSA:3.40.50.410 (GENE3D), PTHR22992 (PANTHER), SSF53300 (SUPERFAMILY), SSF57196 (SUPERFAMILY) |

| | | | | | | | | | | |
|---|---|---|---|---|---|---|---|---|---|---|
| 787 | lib_dib_Filter15_IDBA_contig_17655_plus3 | 115 | fibrinogen c domain-containing protein 1- partial | 615 | 4.76E-48 | 58.05% | 3 | F:receptor binding; C:extracellular space; P:signal transduction | IPR002181 (PFAM); IPR014715 (G3DSA:4.10.530.GENE3D); IPR014716 (G3DSA:3.90.215.GENE3D); PTHR19143 (PANTHER), SignalP-NN(euk) (SIGNALP) | |
| 788 | lib_dib_Filter15_IDBA_contig_17656_minus1 | 449 | deleted in malignant brain tumors 1 | 1141 | 1.00E-137 | 58.10% | 2 | F:receptor activity; C:cell | IPR001190 (PRINTS); IPR017448 (SMART); G3DSA:3.10.250.10 (GENE3D), PTHR19331 (PANTHER), PTHR19331:SF118 (PANTHER), SignalP-NN(euk) (SIGNALP), tmhmm (TMHMM) | Yes |
| 789 | lib_dib_Filter15_IDBA_contig_17657_minus1 | 483 | deleted in malignant brain tumors 1 | 1237 | 4.33E-137 | 58.10% | 2 | F:receptor activity; C:cell | IPR001190 (PRINTS); IPR017448 (SMART); G3DSA:3.10.250.10 (GENE3D), PTHR19331 (PANTHER), PTHR19331:SF118 (PANTHER), SignalP-NN(euk) (SIGNALP), tmhmm (TMHMM) | Yes |
| 790 | lib_dib_Filter15_IDBA_contig_17671_plus1 | 1199 | macrophage mannose receptor 1-like | 725 | 1.82E-33 | 40.45% | 1 | F:carbohydrate binding | IPR001304 (PFAM); IPR016186 (G3DSA:3.10.100.GENE3D); IPR016187 (SUPERFAMILY); IPR018378 (PROSITE); PTHR22803 (PANTHER), SignalP-NN(euk) (SIGNALP), tmhmm (TMHMM) | Yes |
| 791 | lib_dib_Filter15_IDBA_contig_17672_plus1 | 1284 | macrophage mannose receptor 1-like | 727 | 3.81E-37 | 40.55% | 1 | F:carbohydrate binding | IPR001304 (PFAM); IPR016186 (G3DSA:3.10.100.GENE3D); IPR016187 (SUPERFAMILY); IPR018378 (PROSITE); PTHR22803 (PANTHER), SignalP-NN(euk) (SIGNALP), tmhmm (TMHMM) | Yes |
| 792 | lib_dib_Filter15_IDBA_contig_17673_plus2 | 499 | macrophage mannose receptor 1-like | 240 | 1.36E-20 | 46.20% | 2 | C:cell; F:carbohydrate binding | IPR001304 (PFAM); IPR016186 (G3DSA:3.10.100.GENE3D); IPR016187 (SUPERFAMILY); IPR018378 (PROSITE); PTHR22800 (PANTHER) | |
| 793 | lib_dib_Filter15_IDBA_contig_17674_plus3 | 349 | macrophage mannose receptor 1-like | 614 | 6.86E-27 | 41.00% | 1 | F:carbohydrate binding | IPR001304 (PFAM); IPR016186 (G3DSA:3.10.100.GENE3D); IPR016187 (SUPERFAMILY); IPR018378 (PROSITE); PTHR22803 (PANTHER) | |
| 794 | lib_dib_Filter15_IDBA_contig_17675_plus1 | 134 | c-type mannose receptor 2-like | 147 | 4.69E-11 | 46.20% | 1 | F:carbohydrate binding | IPR001304 (PFAM); IPR016186 (G3DSA:3.10.100.GENE3D); IPR016187 (SUPERFAMILY); IPR018378 (PROSITE); PTHR22800 (PANTHER) | |
| 795 | lib_dib_Filter15_IDBA_contig_17676_plus1 | 78 | c-type mannose receptor 2-like | 318 | 5.09E-22 | 39.80% | 1 | F:carbohydrate binding | IPR001304 (PFAM); IPR016186 (G3DSA:3.10.100.GENE3D); IPR016187 (SUPERFAMILY); IPR018378 (PROSITE); PTHR22800 (PANTHER), SignalP-NN(euk) (SIGNALP) | Yes |
| 796 | lib_dib_Filter15_IDBA_contig_17679_plus2 | 202 | macrophage mannose receptor 1-like | 415 | 6.22E-14 | 42.35% | 1 | F:carbohydrate binding | IPR001304 (PFAM); IPR016186 (G3DSA:3.10.100.GENE3D); IPR016187 (SUPERFAMILY); IPR018378 (PROSITE); PTHR22803 (PANTHER) | |
| 797 | lib_dib_Filter15_IDBA_contig_17680_plus2 | 3282 | von willebrand factor type egf and pentraxin domain-containing protein 1-like | 1516 | 0 | 45.20% | 2 | F:protein binding; F:calcium ion binding | IPR000436 (PFAM); IPR000742 (SMART); IPR000884 (SMART); IPR001881 (SMART); IPR009030 (SUPERFAMILY); IPR013032 (PROSITE); IPR018097 (PROSITE); G3DSA:2.10.25.10 (GENE3D), G3DSA:2.10.70.10 (GENE3D), PTHR19325 (PANTHER), tmhmm (TMHMM), SSF57196 (SUPERFAMILY) | |

| | | | | | | | | | |
|---|---|---|---|---|---|---|---|---|---|
| 798 | lib_dib_Filter15_IDBA_contig_17681_plus1 | 3285 | von willebrand factor type egf and pentraxin domain-containing protein 1-like | 1501 | 0 | 45.20% | 2 | F:protein binding; F:calcium ion binding | IPR000436 (PFAM); IPR000742 (SMART); IPR000884 (SMART); IPR001881 (SMART); IPR009030 (SUPERFAMILY); IPR013032 (PROSITE); IPR018097 (PROSITE); G3DSA:2.10.25.10 (GENE3D), G3DSA:2.10.70.10 (GENE3D), PTHR19325 (PANTHER), SignalP-NN(euk) (SIGNALP), tmhmm (TMHMM), SSF57196 (SUPERFAMILY) Yes |
| 799 | lib_dib_Filter15_IDBA_contig_17703_plus3 | 79137 | protein disulfide isomerase | 1665 | 0 | 78.55% | 12 | P:metabolic process; F:catalytic activity; C:cytoplasm; P:cellular homeostasis; P:regulation of biological process; F:electron carrier activity; C:cell; C:cytoplasmic membrane-bounded vesicle; C:endoplasmic reticulum; P:cellular protein modification process; ; C:plasma membrane | IPR005746 (PRINTS); IPR005788 (TIGRFAMs); IPR005792 (TIGRFAMs); IPR012336 (G3DSA:3.40.30.GENE3D); IPR013766 (PFAM); IPR017937 (PROSITE); PTHR18929 (PANTHER), PTHR18929:SF32 (PANTHER), PF13848 (PFAM), SignalP-NN(euk) (SIGNALP), tmhmm (TMHMM) |
| 800 | lib_dib_Filter15_IDBA_contig_17704_plus1 | 61549 | protein disulfide isomerase | 1361 | 5.63E-95 | 83.50% | 15 | P:metabolic process; P:lipid metabolic process; F:catalytic activity; C:cytoplasm; P:cellular homeostasis; P:regulation of biological process; F:electron carrier activity; C:cell; C:cytoplasmic membrane-bounded vesicle; C:endoplasmic reticulum; P:cellular protein modification process; ; F:protein binding; C:extracellular region; C:plasma membrane | IPR005746 (PRINTS); IPR005788 (TIGRFAMs); IPR012336 (G3DSA:3.40.30.GENE3D); IPR013766 (PFAM); IPR017937 (PROSITE); PTHR18929 (PANTHER), PTHR18929:SF32 (PANTHER), PF13848 (PFAM), tmhmm (TMHMM) |
| 801 | lib_dib_Filter15_IDBA_contig_17713_minus3 | 478 | ankyrin repeat protein | 1273 | 6.74E-125 | 47.40% | 1 | F:protein binding | IPR002110 (PRINTS); IPR020683 (G3DSA:1.25.40.GENE3D); PTHR24158 (PANTHER), PTHR24158:SF0 (PANTHER), PF13857 (PFAM) |
| 802 | lib_dib_Filter15_IDBA_contig_17720_plus2 | 4900 | ferric-chelate reductase 1 | 2227 | 1.21E-175 | 49.35% | 8 | P:oxidation-reduction process; F:oxidoreductase activity; C:integral to membrane; P:electron transport chain; C:membrane; F:metal ion binding; F:ferric-chelate reductase activity; P:transport | IPR002861 (PFAM); IPR005018 (PFAM); IPR006593 (SMART); PTHR23130 (PANTHER), PTHR23130:SF6 (PANTHER), SignalP-NN(euk) (SIGNALP), tmhmm (TMHMM) |
| 803 | lib_dib_Filter15_IDBA_contig_17736_minus1 | 739 | aminopeptidase n-like | 1170 | 6.83E-118 | 48.30% | 6 | C:organelle; C:cell; P:protein metabolic process; P:catabolic process; F:peptidase activity; F:binding | IPR001930 (PANTHER); IPR014782 (PRINTS); IPR024571 (PFAM); G3DSA:1.10.390.10 (GENE3D), SignalP-NN(euk) (SIGNALP), tmhmm (TMHMM), SSF55486 (SUPERFAMILY), SSF63737 (SUPERFAMILY) |
| 804 | lib_dib_Filter15_IDBA_contig_17740_plus1 | 1849 | tenascin xb-like | 1003 | 9.64E-85 | 62.10% | 4 | F:receptor binding; C:extracellular space; P:signal transduction; C:collagen | IPR002181 (PFAM); IPR014715 (G3DSA:4.10.530.GENE3D); IPR014716 (G3DSA:3.90.215.GENE3D); IPR020837 (PROSITE); PTHR19143 (PANTHER) |
| 805 | lib_dib_Filter15_IDBA_contig_17741_plus1 | 340 | carcinolectin 5a isoform | 357 | 1.31E-19 | 62.40% | 2 | P:biological_process; F:carbohydrate binding | IPR002181 (PFAM); IPR014715 (G3DSA:4.10.530.GENE3D); IPR020837 (PROSITE); PTHR19143 (PANTHER) |
| 806 | lib_dib_Filter15_IDBA_contig_17742_plus2 | 359 | fibrinogen c domain-containing protein 1 | 234 | 5.36E-24 | 56.75% | 2 | P:signal transduction; F:receptor binding | IPR002181 (PFAM); IPR014716 (G3DSA:3.90.215.GENE3D); PTHR19143 (PANTHER), SignalP-NN(euk) (SIGNALP) |
| 807 | lib_dib_Filter15_IDBA_contig_17749_minus1 | 2057 | thrombospondin-4 | 627 | 1.73E-60 | 51.35% | 2 | F:protein binding; F:calcium ion binding | IPR000742 (SMART); IPR001881 (SMART); G3DSA:2.10.25.10 (GENE3D), PTHR10199 (PANTHER), PTHR10199:SF11 (PANTHER), tmhmm (TMHMM), SSF57196 (SUPERFAMILY) |
| 808 | lib_dib_Filter15_IDBA_contig_17750_minus1 | 2231 | thrombospondin-4 | 688 | 4.79E-67 | 49.55% | 2 | F:protein binding; F:calcium ion binding | IPR000742 (SMART); IPR001881 (SMART); G3DSA:2.10.25.10 (GENE3D), PTHR24838 (PANTHER), tmhmm (TMHMM), SSF57196 (SUPERFAMILY) |

| | | | | | | | | | |
|---|---|---|---|---|---|---|---|---|---|
| 809 | lib_dib_Filter15_IDBA_contig_17751_minus1 | 6793 | aael008062- partial | 1768 | 0 | 66.90% | 5 | P:biological_process; C:extracellular region; P:regulation of biological process; F:protein binding; F:calcium ion binding | IPR000742 (SMART); IPR001881 (PFAM); IPR003367 (PFAM); IPR008859 (PFAM); IPR008985 (SUPERFAMILY); IPR009030 (SUPERFAMILY); IPR013032 (PROSITE); IPR013320 (G3DSA:2.60.120.GENE3D); IPR017897 (PROFILE); IPR018097 (PROSITE); G3DSA:2.10.25.10 (GENE3D), G3DSA:2.170.300.10 (GENE3D), G3DSA:4.10.1080.10 (GENE3D), PTHR10199 (PANTHER), tmhmm (TMHMM), SSF103647 (SUPERFAMILY), SSF57196 (SUPERFAMILY) | |
| 810 | lib_dib_Filter15_IDBA_contig_17751_plus1 | 6793 | ---NA--- | 1768 | | | 0 | - | no IPS match | |
| 811 | lib_dib_Filter15_IDBA_contig_17752_minus1 | 141 | isoform c | 116 | 4.62E-34 | 58.20% | 7 | C:protein complex; F:carbohydrate binding; C:plasma membrane; P:multicellular organismal development; C:extracellular region; P:biological_process; F:protein binding | IPR000742 (SMART); PTHR10199 (PANTHER) | |
| 812 | lib_dib_Filter15_IDBA_contig_17754_minus1 | 156 | cartilage oligomeric matrix protein | 153 | 5.40E-29 | 53.15% | 4 | F:protein binding; P:regulation of biological process; C:extracellular region; F:calcium ion binding | IPR000742 (SMART); IPR001881 (SMART); G3DSA:2.10.25.10 (GENE3D), PTHR24838 (PANTHER), SSF57196 (SUPERFAMILY) | |
| 813 | lib_dib_Filter15_IDBA_contig_17755_minus1 | 501 | kinase suppressor of ras 2 | 696 | 6.24E-101 | 59.65% | 3 | F:nucleotide binding; P:cellular protein modification process; F:protein kinase activity | IPR000719 (PROFILE); IPR001245 (PFAM); IPR002219 (SMART); IPR002290 (SMART); IPR011009 (SUPERFAMILY); IPR020635 (SMART); G3DSA:1.10.510.10 (GENE3D), G3DSA:3.30.200.20 (GENE3D), G3DSA:3.30.60.20 (GENE3D), PTHR23257 (PANTHER), PTHR23257:SF120 (PANTHER) | |
| 814 | lib_dib_Filter15_IDBA_contig_17770_minus3 | 468 | ectonucleoside triphosphate diphosphohydrolase 5 | 842 | 3.85E-104 | 61.35% | 3 | P:protein metabolic process; P:carbohydrate metabolic process; F:hydrolase activity | IPR000407 (PANTHER); PTHR11782:SF7 (PANTHER), tmhmm (TMHMM) | |
| 815 | lib_dib_Filter15_IDBA_contig_17791_minus2 | 1354 | plexin-b-like isoform 2 | 1544 | 0 | 59.40% | 9 | P:signal transduction; F:receptor activity; F:protein binding; P:multicellular organismal development; P:regulation of biological process; P:anatomical structure morphogenesis; P:biological_process; C:plasma membrane; C:intracellular | IPR002909 (PFAM); IPR003659 (SMART); IPR008936 (SUPERFAMILY); IPR013548 (PFAM); IPR013783 (G3DSA:2.60.40.GENE3D); IPR014756 (SUPERFAMILY); G3DSA:3.10.20.90 (GENE3D), G3DSA:3.30.1680.10 (GENE3D), PTHR22625 (PANTHER) | |
| 816 | lib_dib_Filter15_IDBA_contig_17792_minus3 | 19 | plexin-b3 precursor | 101 | 3.30E-18 | 59.70% | 1 | F:protein binding | IPR002909 (PFAM); IPR013783 (G3DSA:2.60.40.GENE3D); IPR014756 (SUPERFAMILY); PTHR22625 (PANTHER) | |
| 817 | lib_dib_Filter15_IDBA_contig_17854_plus2 | 1043 | rna-binding protein 5 | 926 | 2.40E-105 | 63.80% | 14 | F:RNA binding; C:nucleus; C:nucleolus; P:regulation of biological process; P:nucleobase-containing compound metabolic process; P:cellular component organization; P:cell death; C:protein complex; F:DNA binding; P:cell proliferation; F:protein binding; C:nucleoplasm; F:binding; F:nucleotide binding | IPR000467 (PFAM); IPR000504 (SMART); IPR001876 (PFAM); IPR007087 (PROFILE); IPR012677 (G3DSA:3.30.70.GENE3D); G3DSA:4.10.1060.10 (GENE3D), PTHR13948 (PANTHER), PF14259 (PFAM), SignalP-NN(euk) (SIGNALP), tmhmm (TMHMM), SSF54928 (SUPERFAMILY), SSF90209 (SUPERFAMILY) | Yes |
| 818 | lib_dib_Filter15_IDBA_contig_17855_plus3 | 1017 | rna-binding protein 5 | 1006 | 8.02E-105 | 63.80% | 14 | F:RNA binding; C:nucleus; C:nucleolus; P:regulation of biological process; P:nucleobase-containing compound metabolic process; P:cellular component organization; P:cell death; C:protein complex; F:DNA binding; P:cell proliferation; F:protein binding; C:nucleoplasm; F:binding; F:nucleotide binding | IPR000467 (PFAM); IPR000504 (SMART); IPR001876 (PFAM); IPR007087 (PROFILE); IPR012677 (G3DSA:3.30.70.GENE3D); G3DSA:4.10.1060.10 (GENE3D), PTHR13948 (PANTHER), PF13893 (PFAM), PF14259 (PFAM), tmhmm (TMHMM), SSF54928 (SUPERFAMILY), SSF90209 (SUPERFAMILY) | |

| | | | | | | | | | |
|---|---|---|---|---|---|---|---|---|---|
| 819 | lib_dib_Filter15_IDBA_contig_1786_plus3 | 632 | adam family mig-17 | 1082 | 8.80E-174 | 45.25% | 3 | F:peptidase activity; P:protein metabolic process; P:catabolic process | IPR001590 (PFAM); IPR024079 (G3DSA:3.40.390.GENE3D); PTHR13723 (PANTHER), SignalP-NN(euk) (SIGNALP), tmhmm (TMHMM), SSF55486 (SUPERFAMILY) |
| 820 | lib_dib_Filter15_IDBA_contig_17863_plus1 | 489 | 5 -nucleotidase | 606 | 2.36E-159 | 65.00% | 8 | P:nucleobase-containing compound metabolic process; P:catabolic process; P:DNA metabolic process; F:hydrolase activity; C:cytoplasm; C:plasma membrane; F:nucleotide binding; F:binding | IPR004843 (PFAM); IPR006146 (PROSITE); IPR006179 (PRINTS); IPR008334 (G3DSA:3.90.780.GENE3D); G3DSA:3.60.21.10 (GENE3D), PTHR11575:SF7 (PANTHER), SSF56300 (SUPERFAMILY) |
| 821 | lib_dib_Filter15_IDBA_contig_17880_minus2 | 1568 | leucine-rich repeat-containing g-protein coupled receptor 4 | 1838 | 1.31E-44 | 39.70% | 2 | F:protein binding; P:signal transduction | IPR000157 (PFAM); IPR001611 (PFAM); IPR003591 (SMART); G3DSA:3.40.50.10140 (GENE3D), G3DSA:3.80.10.10 (GENE3D), PTHR24365 (PANTHER), PF13504 (PFAM), PF13855 (PFAM), SM00365 (SMART), tmhmm (TMHMM), SSF52058 (SUPERFAMILY) |
| 822 | lib_dib_Filter15_IDBA_contig_17881_minus2 | 638 | slit homolog 2 | 911 | 6.62E-23 | 42.85% | 2 | P:multicellular organismal development; F:protein binding | IPR001611 (PFAM); IPR003591 (SMART); G3DSA:3.80.10.10 (GENE3D), PTHR24365 (PANTHER), PF13504 (PFAM), PF13855 (PFAM), SM00365 (SMART), SSF52058 (SUPERFAMILY) |
| 823 | lib_dib_Filter15_IDBA_contig_17883_minus1 | 53 | variable lymphocyte receptor a diversity region | 267 | 2.10E-10 | 47.85% | 6 | P:biological_process; P:regulation of biological process; P:response to external stimulus; P:response to stress; C:plasma membrane; F:protein binding | IPR001611 (PFAM); IPR003591 (SMART); G3DSA:3.80.10.10 (GENE3D), PTHR24367 (PANTHER), PF13855 (PFAM), SSF52058 (SUPERFAMILY) |
| 824 | lib_dib_Filter15_IDBA_contig_17889_minus2 | 10297 | dnaj homolog subfamily a member 2 | 2466 | 1.39E-139 | 72.15% | 4 | P:regulation of biological process; P:cell proliferation; F:protein binding; P:protein metabolic process | IPR001305 (G3DSA:2.10.230.GENE3D); IPR001623 (PRINTS); IPR002939 (PFAM); IPR008971 (SUPERFAMILY); IPR018253 (PROSITE); G3DSA:2.60.260.20 (GENE3D), PTHR24076 (PANTHER), PTHR24076:SF1 (PANTHER), tmhmm (TMHMM) |
| 825 | lib_dib_Filter15_IDBA_contig_17890_minus2 | 12157 | dnaj homolog subfamily a member 2 | 2469 | 2.67E-137 | 72.15% | 4 | P:regulation of biological process; P:cell proliferation; F:protein binding; P:protein metabolic process | IPR001305 (G3DSA:2.10.230.GENE3D); IPR001623 (PRINTS); IPR002939 (PFAM); IPR008971 (SUPERFAMILY); IPR018253 (PROSITE); G3DSA:2.60.260.20 (GENE3D), PTHR24076 (PANTHER), PTHR24076:SF1 (PANTHER), tmhmm (TMHMM) |
| 826 | lib_dib_Filter15_IDBA_contig_17901_minus2 | 93 | neurogenic locus notch | 445 | 0 | 74.45% | 6 | P:biological_process; F:hydrolase activity; C:cell; P:regulation of biological process; F:protein binding; F:calcium ion binding | IPR000152 (PROSITE); IPR000742 (PFAM); IPR001881 (SMART); IPR013032 (PROSITE); IPR018097 (PROSITE); PD936484 (PRODOM), PR00010 (PRINTS), G3DSA:2.10.25.10 (GENE3D), G3DSA:2.170.300.10 (GENE3D), PTHR24044 (PANTHER), SSF57196 (SUPERFAMILY) |
| 827 | lib_dib_Filter15_IDBA_contig_17902_minus2 | 85 | neurogenic locus notch | 604 | 0 | 65.90% | 22 | P:multicellular organismal development; P:cellular component organization; ; C:nucleoplasm; P:signal transduction; C:Golgi apparatus; F:protein binding; P:biological_process; P:regulation of biological process; C:endoplasmic reticulum; C:extracellular region; C:cytosol; P:cell proliferation; P:cell death; P:cell cycle; C:cell; P:cell differentiation; P:protein metabolic process; P:anatomical structure morphogenesis; P:cell growth; C:plasma membrane; F:calcium ion binding | IPR000152 (PROSITE); IPR000742 (PFAM); IPR001881 (PFAM); IPR013032 (PFAM); IPR018097 (PROSITE); PR00010 (PRINTS), G3DSA:2.10.25.10 (GENE3D), PTHR24044 (PANTHER), PTHR24044:SF11 (PANTHER), SSF57196 (SUPERFAMILY) |

| | | | | | | | | | |
|---|---|---|---|---|---|---|---|---|---|
| 828 | lib_dib_Filter15_IDBA_contig_17903_minus1 | 17 | neurogenic locus notch | 123 | 1.13E-42 | 70.95% | 35 | P:multicellular organismal development; P:biological_process; P:reproduction; P:cell differentiation; P:embryo development; P:anatomical structure morphogenesis; F:catalytic activity; P:cytoskeleton organization; P:cellular component organization; F:nucleotide binding; C:plasma membrane; F:receptor activity; P:signal transduction; P:cell death; P:regulation of biological process; P:cell-cell signaling; P:growth; P:behavior; C:protein complex; P:cell proliferation; ; C:organelle; C:cytoplasm; F:transferase activity; F:hydrolase activity; C:nucleus; P:response to biotic stimulus; P:cell cycle; F:chromatin binding; F:protein binding; P:response to external stimulus; P:response to stress; C:cell; F:DNA binding; F:calcium ion | IPR000152 (PROSITE); IPR000742 (PFAM); IPR001881 (SMART); IPR013032 (PROSITE); IPR018097 (PROSITE); PR00010 (PRINTS), G3DSA:2.10.25.10 (GENE3D), PTHR24044 (PANTHER), PTHR24044:SF0 (PANTHER), SSF57196 (SUPERFAMILY) |
| 829 | lib_dib_Filter15_IDBA_contig_17909_minus1 | 1175 | dnaj homolog subfamily b member 6-like | 920 | 2.74E-43 | 74.85% | 13 | F:protein binding; P:anatomical structure morphogenesis; P:embryo development; P:protein metabolic process; P:regulation of biological process; ; F:enzyme regulator activity; P:cell death; F:DNA binding; P:cytoskeleton organization; P:biological_process; C:cytoplasm; C:nucleus | IPR001623 (PRINTS); IPR018253 (PROSITE); PTHR24077 (PANTHER), PTHR24077:SF15 (PANTHER) |
| 830 | lib_dib_Filter15_IDBA_contig_17910_minus1 | 1175 | dnaj homolog subfamily b member 6-like | 912 | 2.66E-43 | 74.75% | 13 | F:protein binding; P:anatomical structure morphogenesis; P:embryo development; P:protein metabolic process; P:regulation of biological process; ; F:enzyme regulator activity; P:cell death; F:DNA binding; P:cytoskeleton organization; P:biological_process; C:cytoplasm; C:nucleus | IPR001623 (PRINTS); IPR018253 (PROSITE); PTHR24077 (PANTHER), PTHR24077:SF15 (PANTHER) |
| 831 | lib_dib_Filter15_IDBA_contig_17929_minus1 | 113 | deleted in malignant brain tumors 1 | 603 | 9.32E-47 | 47.15% | 3 | F:catalytic activity; F:receptor activity; C:cell | IPR001190 (PRINTS); IPR001846 (PFAM); IPR017448 (SMART); G3DSA:3.10.250.10 (GENE3D), PTHR19331 (PANTHER), PTHR19331:SF118 (PANTHER) |
| 832 | lib_dib_Filter15_IDBA_contig_17930_minus3 | 523 | multiple epidermal growth factor-like domains protein 8 | 1090 | 0 | 52.10% | 5 | F:protein binding; F:calcium ion binding; C:cell; F:receptor activity; P:multicellular organismal development | IPR000152 (PROSITE); IPR000742 (SMART); IPR001881 (SMART); IPR002049 (PFAM); IPR002165 (PFAM); IPR003659 (SMART); IPR013032 (PROSITE); IPR015915 (G3DSA:2.120.10.GENE3D); IPR016201 (SUPERFAMILY); IPR018097 (PROSITE); IPR023413 (G3DSA:2.40.155.GENE3D); IPR024731 (PFAM); PTHR10574 (PANTHER), PTHR10574:SF11 (PANTHER), PF13418 (PFAM), PF13854 (PFAM), SSF117281 (SUPERFAMILY), SSF57196 (SUPERFAMILY) |

| | | | | | | | | | |
|---|---|---|---|---|---|---|---|---|---|
| 833 | lib_dib_Filter15_IDBA_contig_17931_minus3 | 1523 | multiple epidermal growth factor-like domains protein 8-like | 2590 | 0 | 53.35% | 5 | F:protein binding; F:calcium ion binding; C:cell; F:receptor activity; P:multicellular organismal development | IPR000152 (PROSITE); IPR000742 (SMART); IPR000859 (PROFILE); IPR001881 (SMART); IPR002049 (PFAM); IPR002165 (PFAM); IPR003659 (SMART); IPR006652 (PFAM); IPR013032 (PROSITE); IPR015915 (G3DSA:2.120.10.GENE3D); IPR016201 (SUPERFAMILY); IPR018097 (PROSITE); PR00011 (PRINTS), G3DSA:2.10.25.10 (GENE3D), G3DSA:2.170.300.10 (GENE3D), PTHR10574 (PANTHER), PTHR10574:SF4 (PANTHER), PF13418 (PFAM), PF13854 (PFAM), tmhmm (TMHMM), SSF117281 (SUPERFAMILY), SSF57196 (SUPERFAMILY) |
| 834 | lib_dib_Filter15_IDBA_contig_17932_minus3 | 1492 | multiple epidermal growth factor-like domains protein 8-like | 2184 | 0 | 52.60% | 5 | F:protein binding; F:calcium ion binding; C:cell; F:receptor activity; P:multicellular organismal development | IPR000152 (PROSITE); IPR000742 (SMART); IPR000859 (PROFILE); IPR001881 (SMART); IPR002049 (PFAM); IPR002165 (PFAM); IPR003659 (SMART); IPR006652 (PFAM); IPR013032 (PROSITE); IPR015915 (G3DSA:2.120.10.GENE3D); IPR016201 (SUPERFAMILY); IPR018097 (PROSITE); IPR023413 (G3DSA:2.40.155.GENE3D); IPR024731 (PFAM); PR00011 (PRINTS), G3DSA:2.10.25.10 (GENE3D), G3DSA:2.170.300.10 (GENE3D), PTHR10574 (PANTHER), PTHR10574:SF4 (PANTHER), PF13418 (PFAM), PF13854 (PFAM), SSF117281 (SUPERFAMILY), SSF57196 (SUPERFAMILY) |
| 835 | lib_dib_Filter15_IDBA_contig_17956_plus2 | 374 | a chain x-ray structure of c-met with triazolopyridazine | 1408 | 3.47E-65 | 51.55% | 8 | C:plasma membrane; P:signal transduction; P:multicellular organismal development; P:anatomical structure morphogenesis; F:nucleotide binding; P:cellular protein modification process; F:protein binding; F:protein kinase activity | IPR000719 (PROFILE); IPR001245 (PRINTS); IPR002290 (SMART); IPR002909 (PFAM); IPR008266 (PROSITE); IPR011009 (SUPERFAMILY); IPR013783 (G3DSA:2.60.40.GENE3D); IPR014756 (SUPERFAMILY); IPR020635 (SMART); G3DSA:1.10.510.10 (GENE3D), PTHR24418 (PANTHER), tmhmm (TMHMM) |
| 836 | lib_dib_Filter15_IDBA_contig_17957_plus1 | 120 | a chain x-ray structure of c-met with triazolopyridazine | 498 | 4.28E-38 | 53.70% | 16 | C:organelle; C:intracellular; P:anatomical structure morphogenesis; P:cellular component organization; P:biological_process; P:multicellular organismal development; C:plasma membrane; P:signal transduction; P:regulation of biological process; P:primary metabolic process; P:metabolic process; F:protein binding; C:nucleus; P:cellular protein modification process; F:nucleotide binding; F:protein kinase activity | IPR000719 (PROFILE); IPR001245 (PRINTS); IPR002290 (SMART); IPR008266 (PROSITE); IPR011009 (SUPERFAMILY); IPR020635 (SMART); G3DSA:1.10.510.10 (GENE3D), PTHR24418 (PANTHER), SignalP-NN(euk) (SIGNALP), tmhmm (TMHMM) |
| 837 | lib_dib_Filter15_IDBA_contig_17968_plus2 | 3145 | isoform a | 530 | 7.20E-12 | 52.00% | 2 | C:cytoplasm; P:cellular protein modification process | IPR003302 (PFAM); PR01217 (PRINTS) |
| 838 | lib_dib_Filter15_IDBA_contig_17996_minus2 | 225 | leucine-rich repeat-containing protein 47-like | 600 | 6.07E-156 | 63.80% | 3 | F:protein binding; F:RNA binding; F:catalytic activity | IPR001611 (PFAM); IPR003591 (SMART); IPR005146 (PFAM); IPR025875 (PFAM); PR00019 (PRINTS), G3DSA:3.80.10.10 (GENE3D), PTHR10947 (PANTHER), PTHR10947:SF1 (PANTHER), PF13855 (PFAM), SM00364 (SMART), SSF52058 (SUPERFAMILY) |

| | | | | | | | | | | |
|---|---|---|---|---|---|---|---|---|---|---|
| 839 | lib_dib_Filter15_IDBA_contig_18059_plus1 | 335 | hypothetical protein | 688 | 7.53E-04 | 59.00% | 9 | F:transferase activity; F:nucleotide binding; F:zinc ion binding; P:oxidation-reduction process; F:phosphopantetheine binding; F:oxidoreductase activity; F:catalytic activity; F:transferase activity, transferring acyl groups other than amino-acyl groups; P:metabolic process | PTHR24637 (PANTHER), PTHR24637:SF9 (PANTHER), tmhmm (TMHMM) | |
| 840 | lib_dib_Filter15_IDBA_contig_18059_plus2 | 335 | ---NA--- | 688 | | | 0 | - | tmhmm (TMHMM) | |
| 841 | lib_dib_Filter15_IDBA_contig_18060_plus1 | 749 | ---NA--- | 1068 | | | 0 | - | tmhmm (TMHMM) | |
| 842 | lib_dib_Filter15_IDBA_contig_18060_plus3 | 749 | phosphatidic acid phosphatase family protein | 1067 | 2.46E-73 | 48.85% | 2 | F:catalytic activity; C:cell | IPR000326 (PFAM); IPR016118 (G3DSA:1.20.144.GENE3D); PTHR14969 (PANTHER), PTHR14969:SF4 (PANTHER), tmhmm (TMHMM) | |
| 843 | lib_dib_Filter15_IDBA_contig_18066_minus1 | 1338 | ankyrin unc44 | 950 | 9.86E-46 | 42.95% | 1 | F:protein binding | IPR002110 (SMART); IPR020683 (G3DSA:1.25.40.GENE3D); PTHR24198 (PANTHER), tmhmm (TMHMM) | |
| 844 | lib_dib_Filter15_IDBA_contig_18093_plus3 | 349 | slit homolog 3 | 1237 | 5.13E-126 | 45.15% | 5 | P:response to stress; P:biological_process; C:cell; F:protein binding; P:signal transduction | IPR000157 (PFAM); IPR000483 (SMART); IPR001611 (PROFILE); IPR003591 (SMART); PR01537 (PRINTS), G3DSA:3.40.50.10140 (GENE3D), G3DSA:3.80.10.10 (GENE3D), PTHR24365 (PANTHER), PF13855 (PFAM), SM00364 (SMART), SM00365 (SMART), SignalP-NN(euk) (SIGNALP), tmhmm (TMHMM), SSF52058 (SUPERFAMILY) | Yes |
| 845 | lib_dib_Filter15_IDBA_contig_18106_minus1 | 209 | ---NA--- | 849 | | | 0 | - | no IPS match | |
| 846 | lib_dib_Filter15_IDBA_contig_18106_minus2 | 209 | hypothetical protein EAI_09775 | 849 | 5.28E-06 | 71.00% | 0 | - | no IPS match | |
| 847 | lib_dib_Filter15_IDBA_contig_18106_minus3 | 209 | dna methylase n-4 n-6 domain protein | 849 | 2.64E-16 | 56.73% | 0 | - | no IPS match | |
| 848 | lib_dib_Filter15_IDBA_contig_18106_plus1 | 209 | ---NA--- | 849 | | | 0 | - | tmhmm (TMHMM) | |
| 849 | lib_dib_Filter15_IDBA_contig_18106_plus2 | 209 | ---NA--- | 849 | | | 0 | - | tmhmm (TMHMM) | |
| 850 | lib_dib_Filter15_IDBA_contig_18106_plus3 | 209 | ---NA--- | 849 | | | 0 | - | no IPS match | |
| 851 | lib_dib_Filter15_IDBA_contig_18108_plus2 | 590 | aminopeptidase n | 1251 | 9.00E-136 | 48.30% | 4 | P:protein metabolic process; P:catabolic process; F:peptidase activity; F:binding | IPR001930 (PANTHER); IPR014782 (PRINTS); IPR024571 (PFAM); G3DSA:1.10.390.10 (GENE3D), tmhmm (TMHMM), SSF55486 (SUPERFAMILY), SSF63737 (SUPERFAMILY) | |
| 852 | lib_dib_Filter15_IDBA_contig_18117_minus1 | 155 | cleavage stimulation factor subunit 1 | 625 | 0 | 79.15% | 1 | F:protein binding | IPR001680 (PFAM); IPR015943 (G3DSA:2.130.10.GENE3D); IPR017986 (PROFILE); IPR019775 (PROSITE); IPR020472 (PRINTS); PTHR22840 (PANTHER), PTHR22840:SF5 (PANTHER), tmhmm (TMHMM) | |
| 853 | lib_dib_Filter15_IDBA_contig_18186_plus1 | 457 | matrix metalloproteinase | 110 | 1.08E-22 | 62.05% | 5 | F:peptidase activity; P:protein metabolic process; P:catabolic process; F:binding; C:extracellular region | IPR001818 (PFAM); IPR006026 (SMART); IPR021190 (PRINTS); IPR024079 (G3DSA:3.40.390.GENE3D); PTHR10201 (PANTHER), SSF55486 (SUPERFAMILY) | |
| 854 | lib_dib_Filter15_IDBA_contig_18189_plus2 | 402 | matrix metalloproteinase-17 | 119 | 8.59E-14 | 56.40% | 5 | F:peptidase activity; P:protein metabolic process; P:catabolic process; F:binding; C:extracellular region | IPR002477 (G3DSA:1.10.101.GENE3D); IPR021158 (PROSITE); IPR024079 (G3DSA:3.40.390.GENE3D); PTHR10201 (PANTHER) | |

| | | | | | | | | | | |
|---|---|---|---|---|---|---|---|---|---|---|
| 855 | lib_dib_Filter15_IDBA_contig_18200_minus2 | 133 | beta-galactosidase | 591 | 3.87E-80 | 54.30% | 2 | F:catalytic activity; P:carbohydrate metabolic process | IPR001944 (PRINTS); IPR008979 (SUPERFAMILY); IPR013781 (G3DSA:3.20.20.GENE3D); IPR017853 (SUPERFAMILY); G3DSA:2.60.120.260 (GENE3D) | |
| 856 | lib_dib_Filter15_IDBA_contig_18216_plus1 | 164 | toll | 591 | 3.43E-32 | 43.25% | 7 | P:multicellular organismal development; P:response to biotic stimulus; C:cell; P:regulation of biological process; P:response to stress; P:metabolic process; F:protein binding | IPR000372 (SMART); IPR000483 (SMART); IPR001611 (PFAM); IPR003591 (SMART); G3DSA:3.80.10.10 (GENE3D), PTHR24365 (PANTHER), PF13855 (PFAM), tmhmm (TMHMM), SSF52058 (SUPERFAMILY) | |
| 857 | lib_dib_Filter15_IDBA_contig_18224_plus2 | 806 | serine threonine-protein kinase mig-15 | 1233 | 0 | 78.20% | 17 | P:anatomical structure morphogenesis; P:cellular component organization; P:cell differentiation; P:multicellular organismal development; P:biological_process; P:metabolic process; P:signal transduction; P:response to external stimulus; P:response to stress; F:protein kinase activity; F:signal transducer activity; F:enzyme regulator activity; P:embryo development; P:reproduction; P:regulation of biological process; C:cytoplasm; F:nucleotide binding | IPR000719 (PFAM); IPR001180 (PFAM); IPR002290 (SMART); IPR008271 (PROSITE); IPR011009 (SUPERFAMILY); IPR017441 (PROSITE); IPR020635 (SMART); G3DSA:1.10.510.10 (GENE3D), G3DSA:3.30.200.20 (GENE3D), PTHR24361 (PANTHER), PTHR24361:SF60 (PANTHER), SignalP-NN(euk) (SIGNALP) | |
| 858 | lib_dib_Filter15_IDBA_contig_18265_plus3 | 1225 | adhesin-like protein 1 | 894 | 3.10E-12 | 54.20% | 3 | C:extracellular region; P:carbohydrate metabolic process; F:carbohydrate binding | IPR002557 (PFAM); tmhmm (TMHMM) | |
| 859 | lib_dib_Filter15_IDBA_contig_18266_plus2 | 907 | adhesin-like protein 1 | 876 | 1.26E-12 | 52.46% | 3 | C:extracellular region; P:carbohydrate metabolic process; F:carbohydrate binding | IPR002557 (G3DSA:2.170.140.GENE3D) | |
| 860 | lib_dib_Filter15_IDBA_contig_18272_plus2 | 873 | peptidyl-prolyl cis-trans isomerase fkbp2 isoform 2 | 854 | 1.99E-60 | 89.45% | 6 | C:endoplasmic reticulum; P:protein metabolic process; F:binding; F:protein binding; P:cellular protein modification process; F:catalytic activity | IPR001179 (PFAM); IPR023566 (PANTHER); G3DSA:3.10.50.40 (GENE3D), PTHR10516:SF134 (PANTHER), SignalP-NN(euk) (SIGNALP), tmhmm (TMHMM), SSF54534 (SUPERFAMILY) | |
| 861 | lib_dib_Filter15_IDBA_contig_18299_plus1 | 4686 | dimethylaniline monooxygenase | 587 | 1.24E-87 | 54.25% | 7 | P:nucleobase-containing compound metabolic process; P:secondary metabolic process; C:cell; F:catalytic activity; P:metabolic process; F:nucleotide binding; C:endoplasmic reticulum | IPR000960 (PRINTS); IPR012143 (PIR); IPR020946 (PFAM); G3DSA:3.50.50.60 (GENE3D), PTHR23023 (PANTHER), PTHR23023:SF4 (PANTHER), SSF51905 (SUPERFAMILY) | |
| 862 | lib_dib_Filter15_IDBA_contig_18341_minus2 | 298 | fatty aldehyde dehydrogenase | 657 | 0 | 72.25% | 3 | C:mitochondrion; F:catalytic activity; P:metabolic process | IPR012394 (PTHR11699:PANTHER); IPR015590 (PFAM); IPR016160 (PROSITE); IPR016161 (SUPERFAMILY); IPR016162 (G3DSA:3.40.605.GENE3D); IPR016163 (G3DSA:3.40.309.GENE3D); PTHR11699 (PANTHER), SignalP-NN(euk) (SIGNALP), tmhmm (TMHMM) | Yes |
| 863 | lib_dib_Filter15_IDBA_contig_18344_minus2 | 2382 | l-xylulose reductase | 776 | 1.76E-70 | 67.75% | 9 | P:carbohydrate metabolic process; P:metabolic process; P:nucleobase-containing compound metabolic process; P:secondary metabolic process; C:cell; F:catalytic activity; F:protein binding; P:cellular component organization; F:nucleotide binding | IPR002198 (PRINTS); IPR002347 (PRINTS); IPR016040 (G3DSA:3.40.50.GENE3D); PTHR24311 (PANTHER), PTHR24311:SF0 (PANTHER), PF13561 (PFAM), SSF51735 (SUPERFAMILY) | |
| 864 | lib_dib_Filter15_IDBA_contig_18345_minus2 | 1370 | l-xylulose reductase | 349 | 5.12E-51 | 69.30% | 9 | F:catalytic activity; P:carbohydrate metabolic process; P:metabolic process; P:nucleobase-containing compound metabolic process; P:secondary metabolic process; C:cell; F:protein binding; P:cellular component organization; F:nucleotide binding | IPR002198 (PRINTS); IPR002347 (PRINTS); IPR016040 (G3DSA:3.40.50.GENE3D); PTHR24311 (PANTHER), PTHR24311:SF0 (PANTHER), SSF51735 (SUPERFAMILY) | |

| | | | | | | | | | | |
|---|---|---|---|---|---|---|---|---|---|---|
| 865 | lib_dib_Filter15_IDBA_contig_18346_minus2 | 1313 | l-xylulose reductase | 728 | 5.07E-79 | 70.05% | 9 | F:catalytic activity; P:carbohydrate metabolic process; P:metabolic process; P:nucleobase-containing compound metabolic process; P:secondary metabolic process; C:cell; F:protein binding; P:cellular component organization; F:nucleotide binding | IPR002198 (PRINTS); IPR002347 (PRINTS); IPR016040 (G3DSA:3.40.50.GENE3D); PTHR24311 (PANTHER), PTHR24311:SF0 (PANTHER), PF13561 (PFAM), SSF51735 (SUPERFAMILY) | |
| 866 | lib_dib_Filter15_IDBA_contig_18362_minus2 | 128 | upf0378 protein kiaa0100-like | 614 | 8.87E-164 | 61.05% | 0 | - | IPR019443 (PFAM); PTHR15678 (PANTHER) | |
| 867 | lib_dib_Filter15_IDBA_contig_18363_minus3 | 31 | upf0378 protein kiaa0100-like | 249 | 7.34E-60 | 58.65% | 0 | - | PTHR15678 (PANTHER) | |
| 868 | lib_dib_Filter15_IDBA_contig_18364_minus2 | 179 | cub and sushi domain-containing protein 3-like | 584 | 1.00E-39 | 53.00% | 1 | P:biological_process | IPR000421 (PFAM); IPR006585 (SMART); IPR008979 (SUPERFAMILY); G3DSA:2.60.120.260 (GENE3D), PTHR19277 (PANTHER), PTHR19277:SF6 (PANTHER), SignalP-NN(euk) (SIGNALP), tmhmm (TMHMM) | Yes |
| 869 | lib_dib_Filter15_IDBA_contig_18367_plus2 | 2906 | #NAME? | 1357 | 1.44E-18 | 75.25% | 3 | P:protein metabolic process; P:response to stress; F:protein binding | IPR001623 (PRINTS); IPR018253 (PROSITE); PTHR24077 (PANTHER) | |
| 870 | lib_dib_Filter15_IDBA_contig_18391_minus1 | 189 | a chain quaternary ligand binding to aromatic residues in the active-site gorge of acetylcholinesterase | 604 | 3.21E-115 | 55.25% | 2 | P:biological_process; F:hydrolase activity | IPR000997 (PRINTS); IPR002018 (PFAM); IPR019819 (PROSITE); G3DSA:3.40.50.1820 (GENE3D), PTHR11559 (PANTHER), SignalP-NN(euk) (SIGNALP), tmhmm (TMHMM), SSF53474 (SUPERFAMILY) | |
| 871 | lib_dib_Filter15_IDBA_contig_18443_plus2 | 1398 | neoverrucotoxin subunit alpha-like | 1340 | 7.60E-145 | 50.45% | 3 | F:nucleotide binding; P:cell cycle; F:protein binding | IPR000038 (PANTHER); IPR003961 (SMART); IPR013783 (G3DSA:2.60.40.GENE3D); G3DSA:3.40.50.300 (GENE3D), PTHR18884:SF30 (PANTHER), tmhmm (TMHMM) | |
| 872 | lib_dib_Filter15_IDBA_contig_18456_plus2 | 1294 | semaphorin-2a-like isoform 2 | 1498 | 8.25E-100 | 48.85% | 6 | P:behavior; P:cell differentiation; P:multicellular organismal development; F:protein binding; F:receptor activity; C:cell | IPR001627 (PFAM); IPR007110 (PROFILE); IPR013783 (G3DSA:2.60.40.GENE3D); IPR015943 (G3DSA:2.130.10.GENE3D); IPR016201 (SUPERFAMILY); IPR027231 (PANTHER); G3DSA:3.30.1680.10 (GENE3D), SignalP-NN(euk) (SIGNALP), tmhmm (TMHMM), SSF48726 (SUPERFAMILY) | |
| 873 | lib_dib_Filter15_IDBA_contig_18465_plus2 | 779 | e3 ubiquitin-protein ligase hectd1 | 579 | 0 | 86.75% | 10 | F:binding; F:catalytic activity; P:cellular protein modification process; P:catabolic process; F:transferase activity; C:cytoplasm; C:nucleus; P:anatomical structure morphogenesis; P:embryo development; F:protein binding | IPR002110 (SMART); IPR011989 (G3DSA:1.25.10.GENE3D); IPR016024 (SUPERFAMILY); IPR020683 (G3DSA:1.25.40.GENE3D); PTHR11254 (PANTHER), PTHR11254:SF75 (PANTHER), SignalP-NN(euk) (SIGNALP), tmhmm (TMHMM) | |
| 874 | lib_dib_Filter15_IDBA_contig_18501_minus3 | 705 | gelatinase b | 577 | 7.79E-38 | 47.95% | 10 | F:protein binding; P:multicellular organismal development; P:biological_process; C:extracellular region; P:response to abiotic stimulus; P:regulation of biological process; P:response to stress; F:peptidase activity; P:metabolic process; P:response to external stimulus | IPR000562 (G3DSA:2.10.10.GENE3D); IPR013806 (SUPERFAMILY); PTHR22918 (PANTHER), PTHR22918:SF0 (PANTHER), SignalP-NN(euk) (SIGNALP), tmhmm (TMHMM) | |
| 875 | lib_dib_Filter15_IDBA_contig_18509_minus1 | 140 | lactose-binding lectin l-2-like | 577 | 9.43E-12 | 47.10% | 1 | F:carbohydrate binding | IPR000859 (G3DSA:2.60.120.GENE3D); IPR001304 (PFAM); IPR016186 (G3DSA:3.10.100.GENE3D); IPR016187 (SUPERFAMILY); PTHR22801 (PANTHER), SignalP-NN(euk) (SIGNALP), tmhmm (TMHMM) | Yes |

| | | | | | | | | | |
|---|---|---|---|---|---|---|---|---|---|
| 876 | lib_dib_Filter15_IDBA_contig_18520_plus2 | 295 | protein phosphatase 1 regulatory subunit 7 isoform 2 | 633 | 7.08E-114 | 75.95% | 4 | C:cytoplasm; P:biological_process; F:enzyme regulator activity; F:protein binding | IPR001611 (PROFILE); IPR003591 (SMART); G3DSA:3.80.10.10 (GENE3D), PTHR10588 (PANTHER), PTHR10588:SF96 (PANTHER), PF13855 (PFAM), SM00365 (SMART), tmhmm (TMHMM), SSF52058 (SUPERFAMILY) |
| 877 | lib_dib_Filter15_IDBA_contig_18522_plus1 | 5817 | rna-binding protein 4 | 1273 | 6.66E-40 | 53.65% | 13 | P:metabolic process; P:regulation of biological process; C:nucleoplasm; P:translation; P:nucleobase-containing compound metabolic process; C:cytoplasm; P:cell differentiation; C:organelle; C:intracellular; P:response to stress; F:RNA binding; F:binding; F:nucleotide binding | IPR000504 (PFAM); IPR001878 (G3DSA:4.10.60.GENE3D); IPR012677 (G3DSA:3.30.70.GENE3D); PTHR24011 (PANTHER), PTHR24011:SF59 (PANTHER), SignalP-NN(euk) (SIGNALP), tmhmm (TMHMM), SSF54928 (SUPERFAMILY) |
| 878 | lib_dib_Filter15_IDBA_contig_18525_plus2 | 302 | slit protein | 678 | 1.59E-83 | 48.40% | 2 | C:cell; F:protein binding | IPR001611 (PROFILE); IPR003591 (SMART); G3DSA:3.80.10.10 (GENE3D), PTHR24365 (PANTHER), PF13855 (PFAM), SM00365 (SMART), tmhmm (TMHMM), SSF52058 (SUPERFAMILY) |
| 879 | lib_dib_Filter15_IDBA_contig_18537_plus1 | 638 | arylsulfatase b | 649 | 0 | 63.75% | 2 | P:metabolic process; F:hydrolase activity | IPR000917 (PFAM); IPR017849 (G3DSA:3.40.720.GENE3D); IPR017850 (SUPERFAMILY); IPR024607 (PROSITE); G3DSA:3.30.1120.10 (GENE3D), PTHR10342 (PANTHER), PTHR10342:SF19 (PANTHER) |
| 880 | lib_dib_Filter15_IDBA_contig_18538_plus3 | 511 | beta- -galactosyltransferase 7 | 824 | 2.44E-113 | 74.05% | 10 | P:metabolic process; P:biosynthetic process; P:carbohydrate metabolic process; P:cellular component organization; C:cell; P:cellular protein modification process; P:regulation of biological process; P:cell proliferation; C:Golgi apparatus; F:transferase activity | IPR003859 (PRINTS); G3DSA:3.90.550.10 (GENE3D), PTHR19300:SF1 (PANTHER), PF13733 (PFAM), SignalP-NN(euk) (SIGNALP), tmhmm (TMHMM), SSF53448 (SUPERFAMILY) |
| 881 | lib_dib_Filter15_IDBA_contig_18564_plus2 | 599 | dual oxidase 1 | 467 | 0 | 66.90% | 8 | P:signal transduction; P:multicellular organismal development; P:biological_process; F:catalytic activity; F:antioxidant activity; P:response to stress; F:binding; P:metabolic process | IPR002007 (G3DSA:1.10.640.GENE3D); IPR010255 (SUPERFAMILY); IPR019791 (PRINTS); PTHR11972 (PANTHER), PTHR11972:SF6 (PANTHER) |
| 882 | lib_dib_Filter15_IDBA_contig_18567_plus1 | 68 | dual oxidase 1 | 115 | 1.13E-37 | 66.15% | 8 | P:signal transduction; P:multicellular organismal development; P:biological_process; F:catalytic activity; F:antioxidant activity; P:response to stress; F:binding; P:metabolic process | IPR002007 (G3DSA:1.10.640.GENE3D); IPR010255 (SUPERFAMILY); PTHR11972 (PANTHER), PTHR11972:SF6 (PANTHER) |
| 883 | lib_dib_Filter15_IDBA_contig_18593_minus3 | 513 | integrin beta-ps | 849 | 0 | 62.85% | 4 | P:biological_process; C:cell; F:receptor activity; P:multicellular organismal development | IPR002369 (PFAM); IPR012896 (PFAM); IPR014836 (PFAM); IPR015812 (PRINTS); IPR016201 (SUPERFAMILY); G3DSA:1.20.5.100 (GENE3D), G3DSA:2.10.25.10 (GENE3D), G3DSA:3.40.50.410 (GENE3D), PTHR10082:SF16 (PANTHER), SignalP-NN(euk) (SIGNALP), tmhmm (TMHMM), SSF53300 (SUPERFAMILY), SSF57196 (SUPERFAMILY) |
| 884 | lib_dib_Filter15_IDBA_contig_18594_minus3 | 499 | integrin beta-ps | 841 | 0 | 62.90% | 4 | P:biological_process; C:cell; F:receptor activity; P:multicellular organismal development | IPR002369 (PFAM); IPR012896 (PFAM); IPR014836 (PFAM); IPR015812 (PRINTS); IPR016201 (SUPERFAMILY); G3DSA:1.20.5.100 (GENE3D), G3DSA:2.10.25.10 (GENE3D), G3DSA:3.40.50.410 (GENE3D), PTHR10082:SF16 (PANTHER), SignalP-NN(euk) (SIGNALP), tmhmm (TMHMM), SSF53300 (SUPERFAMILY), SSF57196 (SUPERFAMILY) |
| 885 | lib_dib_Filter15_IDBA_contig_18604_plus2 | 465 | h aca ribonucleoprotein complex non-core subunit naf1 | 1272 | 8.41E-40 | 72.05% | 6 | C:plasma membrane; C:intracellular; P:biological_process; C:microtubule organizing center; F:RNA binding; P:nucleobase-containing compound metabolic process | IPR007504 (PFAM); IPR009000 (SUPERFAMILY); G3DSA:2.40.10.230 (GENE3D), PTHR31991 (PANTHER), PTHR31991:SF0 (PANTHER), tmhmm (TMHMM) |

| | | | | | | | | | | |
|---|---|---|---|---|---|---|---|---|---|---|
| 886 | lib_dib_Filter15_IDBA_contig_18613_minus3 | 944 | suppressor of lurcher protein 1-like | 665 | 1.24E-89 | 57.40% | 0 | - | IPR000859 (G3DSA:2.60.120.GENE3D); PTHR10127 (PANTHER), PTHR10127:SF345 (PANTHER), SignalP-NN(euk) (SIGNALP), tmhmm (TMHMM) | Yes |
| 887 | lib_dib_Filter15_IDBA_contig_18614_minus3 | 1479 | suppressor of lurcher protein 1-like | 1214 | 1.54E-86 | 57.60% | 0 | - | IPR000859 (G3DSA:2.60.120.GENE3D); PTHR10127 (PANTHER), PTHR10127:SF345 (PANTHER), SignalP-NN(euk) (SIGNALP), tmhmm (TMHMM) | Yes |
| 888 | lib_dib_Filter15_IDBA_contig_18631_minus1 | 393 | tissue factor pathway inhibitor | 572 | 3.65E-68 | 53.85% | 2 | F:hydrolase activity; F:enzyme regulator activity | IPR002223 (PRINTS); IPR003609 (PROFILE); IPR020901 (PROSITE); PTHR10083 (PANTHER), SignalP-NN(euk) (SIGNALP) | Yes |
| 889 | lib_dib_Filter15_IDBA_contig_18632_minus2 | 578 | dnaj homolog subfamily b member 4 | 722 | 3.67E-136 | 69.75% | 5 | C:cytoplasm; C:nucleolus; C:cell; C:plasma membrane; F:protein binding | IPR001623 (PRINTS); IPR002939 (PFAM); IPR008971 (SUPERFAMILY); IPR018253 (PROSITE); G3DSA:2.60.260.20 (GENE3D), PTHR24077 (PANTHER), PTHR24077:SF6 (PANTHER), SignalP-NN(euk) (SIGNALP), tmhmm (TMHMM) | |
| 890 | lib_dib_Filter15_IDBA_contig_18638_plus2 | 1065 | aldehyde dehydrogenase 2 family | 822 | 0 | 81.70% | 8 | P:carbohydrate metabolic process; F:catalytic activity; P:metabolic process; P:biosynthetic process; P:cell-cell signaling; F:electron carrier activity; C:mitochondrion; P:catabolic process | IPR015590 (PFAM); IPR016160 (PROSITE); IPR016161 (SUPERFAMILY); IPR016162 (G3DSA:3.40.605.GENE3D); IPR016163 (G3DSA:3.40.309.GENE3D); PTHR11699 (PANTHER), PTHR11699:SF46 (PANTHER) | |
| 891 | lib_dib_Filter15_IDBA_contig_18648_plus2 | 481 | matrix metalloproteinase | 807 | 1.70E-98 | 52.00% | 5 | F:peptidase activity; P:protein metabolic process; P:catabolic process; F:binding; C:extracellular region | IPR000585 (G3DSA:2.110.10.GENE3D); IPR001818 (PFAM); IPR002477 (PFAM); IPR006026 (SMART); IPR018486 (PROSITE); IPR018487 (PFAM); IPR021190 (PRINTS); IPR024079 (G3DSA:3.40.390.GENE3D); PTHR10201 (PANTHER), tmhmm (TMHMM), SSF55486 (SUPERFAMILY) | |
| 892 | lib_dib_Filter15_IDBA_contig_18666_plus2 | 276 | 5-hydroxytryptamine receptor 4-like | 570 | 7.39E-90 | 63.00% | 6 | P:signal transduction; F:receptor activity; P:biological_process; P:nucleobase-containing compound metabolic process; P:biosynthetic process; C:cell | IPR000276 (PRINTS); IPR017452 (PROFILE); G3DSA:1.20.1070.10 (GENE3D), PTHR24249 (PANTHER), PTHR24249:SF33 (PANTHER), tmhmm (TMHMM), SSF81321 (SUPERFAMILY) | |
| 893 | lib_dib_Filter15_IDBA_contig_18668_minus2 | 282 | early endosome antigen 1 | 1047 | 4.37E-80 | 72.70% | 4 | F:binding; F:protein binding; C:cytoplasm; P:transport | IPR000306 (PFAM); IPR009053 (SUPERFAMILY); IPR011011 (SUPERFAMILY); IPR013083 (G3DSA:3.30.40.GENE3D); IPR017455 (PROFILE); G3DSA:1.20.5.390 (GENE3D), PTHR23164 (PANTHER) | |
| 894 | lib_dib_Filter15_IDBA_contig_1874_plus1 | 1233 | dappu_318553-like protein | 100 | 4.03E-27 | 69.05% | 5 | F:catalytic activity; F:antioxidant activity; P:response to stress; F:binding; P:metabolic process | IPR002007 (G3DSA:1.10.640.GENE3D); IPR010255 (SUPERFAMILY); PTHR11475 (PANTHER) | |
| 895 | lib_dib_Filter15_IDBA_contig_18740_minus2 | 453 | integrin alpha-ps2-like | 993 | 0 | 53.65% | 3 | P:biological_process; C:protein complex; C:plasma membrane | IPR000413 (PRINTS); IPR013517 (PFAM); IPR013519 (SMART); IPR013649 (PFAM); G3DSA:2.130.10.130 (GENE3D), G3DSA:2.60.40.1460 (GENE3D), G3DSA:2.60.40.1510 (GENE3D), G3DSA:2.60.40.1530 (GENE3D), PTHR23220 (PANTHER), PTHR23220:SF11 (PANTHER), SSF69179 (SUPERFAMILY), SSF69318 (SUPERFAMILY) | |

| | | | | | | | | | | |
|---|---|---|---|---|---|---|---|---|---|---|
| 896 | lib_dib_Filter15_IDBA_contig_18741_minus2 | 600 | integrin alpha-ps2-like | 1359 | 0 | 53.55% | 3 | P:biological_process; C:protein complex; C:plasma membrane | IPR000413 (PRINTS); IPR013517 (PFAM); IPR013519 (SMART); IPR013649 (PFAM); IPR018184 (PFAM); G3DSA:1.20.5.930 (GENE3D), G3DSA:2.130.10.130 (GENE3D), G3DSA:2.60.40.1460 (GENE3D), G3DSA:2.60.40.1510 (GENE3D), G3DSA:2.60.40.1530 (GENE3D), PTHR23220 (PANTHER), PTHR23220:SF11 (PANTHER), tmhmm (TMHMM), SSF69179 (SUPERFAMILY), SSF69318 (SUPERFAMILY) | |
| 897 | lib_dib_Filter15_IDBA_contig_18742_minus2 | 595 | integrin alpha-ps2-like | 1282 | 0 | 53.50% | 3 | P:biological_process; C:protein complex; C:plasma membrane | IPR000413 (PRINTS); IPR013517 (PFAM); IPR013519 (SMART); IPR013649 (PFAM); IPR018184 (PFAM); G3DSA:1.20.5.930 (GENE3D), G3DSA:2.130.10.130 (GENE3D), G3DSA:2.60.40.1460 (GENE3D), G3DSA:2.60.40.1510 (GENE3D), G3DSA:2.60.40.1530 (GENE3D), PTHR23220 (PANTHER), PTHR23220:SF11 (PANTHER), tmhmm (TMHMM), SSF69179 (SUPERFAMILY), SSF69318 (SUPERFAMILY) | |
| 898 | lib_dib_Filter15_IDBA_contig_1875_plus1 | 455 | dappu_318553-like protein | 101 | 2.15E-27 | 68.70% | 5 | F:catalytic activity; F:antioxidant activity; P:response to stress; F:binding; P:metabolic process | IPR002007 (G3DSA:1.10.640.GENE3D); IPR010255 (SUPERFAMILY); IPR019791 (PRINTS); PTHR11475 (PANTHER) | |
| 899 | lib_dib_Filter15_IDBA_contig_18760_minus1 | 1827 | myocilin | 1294 | 4.70E-21 | 46.15% | 1 | F:protein binding | IPR003112 (PFAM); PTHR23192 (PANTHER) | |
| 900 | lib_dib_Filter15_IDBA_contig_18761_minus1 | 1922 | myocilin | 1350 | 5.25E-21 | 46.15% | 1 | F:protein binding | IPR003112 (PFAM); PTHR23192 (PANTHER) | |
| 901 | lib_dib_Filter15_IDBA_contig_18762_minus1 | 1929 | myocilin | 1341 | 5.70E-21 | 46.35% | 1 | F:protein binding | IPR003112 (PFAM); PTHR23192 (PANTHER) | |
| 902 | lib_dib_Filter15_IDBA_contig_18763_minus3 | 121 | oocyte zinc finger protein 6-like | 804 | 6.38E-68 | 62.95% | 3 | F:binding; F:nucleic acid binding; C:intracellular | IPR007087 (PROSITE); IPR013087 (G3DSA:3.30.160.GENE3D); IPR015880 (SMART); PTHR24375 (PANTHER), PF13465 (PFAM), SSF57667 (SUPERFAMILY) | |
| 903 | lib_dib_Filter15_IDBA_contig_18793_minus2 | 556 | follistatin-related protein 5 | 1232 | 0 | 57.80% | 1 | F:protein binding | IPR002350 (PFAM); IPR003598 (SMART); IPR003599 (SMART); IPR007110 (PROFILE); IPR013098 (PFAM); IPR013783 (G3DSA:2.60.40.GENE3D); IPR015943 (G3DSA:2.130.10.GENE3D); IPR018247 (PROSITE); G3DSA:3.30.60.30 (GENE3D), PTHR10913 (PANTHER), PTHR10913:SF14 (PANTHER), SignalP-NN(euk) (SIGNALP), tmhmm (TMHMM), SSF100895 (SUPERFAMILY), SSF48726 (SUPERFAMILY), SSF75011 (SUPERFAMILY) | Yes |
| 904 | lib_dib_Filter15_IDBA_contig_18794_minus3 | 91 | low quality protein: multiple epidermal growth factor-like domains protein 6 | 566 | 6.27E-17 | 40.95% | 2 | F:calcium ion binding; C:extracellular region | IPR000562 (G3DSA:2.10.10.GENE3D); IPR008979 (SUPERFAMILY); IPR013806 (SUPERFAMILY); G3DSA:2.170.300.10 (GENE3D), G3DSA:2.60.120.260 (GENE3D), PTHR24035 (PANTHER), PTHR24035:SF4 (PANTHER) | |
| 905 | lib_dib_Filter15_IDBA_contig_18828_plus3 | 284 | kalirin isoform 3 | 376 | 2.71E-70 | 51.75% | 4 | F:protein binding; P:cellular component organization; P:signal transduction; C:cytoplasm | IPR001452 (PFAM); IPR003598 (SMART); IPR003599 (SMART); IPR003961 (PFAM); IPR007110 (PROFILE); IPR013098 (PFAM); IPR013783 (G3DSA:2.60.40.GENE3D); IPR020636 (PANTHER); PR00014 (PRINTS), G3DSA:2.30.30.40 (GENE3D), PTHR24347:SF47 (PANTHER), SSF48726 (SUPERFAMILY) | |

| | | | | | | | | | |
|---|---|---|---|---|---|---|---|---|---|
| 906 | lib_dib_Filter15_IDBA_contig_18834_minus2 | 631 | lachesin | 566 | 2.85E-109 | 48.75% | 1 | F:protein binding | IPR003598 (SMART); IPR003599 (SMART); IPR007110 (PROFILE); IPR013098 (PFAM); IPR013106 (PFAM); IPR013783 (G3DSA:2.60.40.GENE3D); PTHR19831 (PANTHER), PTHR19831:SF16 (PANTHER), tmhmm (TMHMM), SSF48726 (SUPERFAMILY) |
| 907 | lib_dib_Filter15_IDBA_contig_18846_minus2 | 187 | beta-galactosidase-1-like protein 2 | 769 | 0 | 59.95% | 2 | F:hydrolase activity; P:carbohydrate metabolic process | IPR001944 (PRINTS); IPR008979 (SUPERFAMILY); IPR013781 (G3DSA:3.20.20.GENE3D); IPR017853 (SUPERFAMILY); G3DSA:2.60.120.260 (GENE3D) |
| 908 | lib_dib_Filter15_IDBA_contig_18874_plus1 | 101 | dual specificity mitogen-activated protein kinase kinase 5 isoform 1 | 566 | 5.16E-157 | 66.30% | 18 | P:anatomical structure morphogenesis; P:regulation of biological process; P:embryo development; P:biosynthetic process; F:protein kinase activity; P:multicellular organismal development; ; C:cytosol; P:signal transduction; P:response to stress; P:cell proliferation; P:cell growth; F:protein binding; F:nucleotide binding; P:cell death; P:biological_process; P:cellular protein modification process; C:nucleus | IPR000270 (PFAM); IPR000719 (PFAM); IPR002290 (SMART); IPR008271 (PROSITE); IPR011009 (SUPERFAMILY); IPR020635 (SMART); G3DSA:1.10.510.10 (GENE3D), G3DSA:3.10.20.240 (GENE3D), G3DSA:3.30.200.20 (GENE3D), PTHR24360 (PANTHER), PTHR24360:SF11 (PANTHER), tmhmm (TMHMM), SSF54277 (SUPERFAMILY) |
| 909 | lib_dib_Filter15_IDBA_contig_18889_minus2 | 162 | collagen alpha-1 chain | 565 | 2.02E-58 | 65.85% | 9 | P:metabolic process; P:anatomical structure morphogenesis; P:embryo development; P:multicellular organismal development; C:proteinaceous extracellular matrix; P:cellular component organization; P:cell differentiation; P:biological_process; F:structural molecule activity | IPR001791 (SMART); IPR008160 (PFAM); IPR008985 (SUPERFAMILY); PTHR24023 (PANTHER), PTHR24023:SF40 (PANTHER) |
| 910 | lib_dib_Filter15_IDBA_contig_18889_plus1 | 162 | ---NA--- | 565 | | | 0 | - | no IPS match |
| 911 | lib_dib_Filter15_IDBA_contig_18988_plus3 | 630 | hyou1 protein | 1104 | 0 | 73.35% | 6 | P:metabolic process; P:signal transduction; P:response to stress; P:response to biotic stimulus; C:endoplasmic reticulum; F:nucleotide binding | IPR013126 (PRINTS); IPR018181 (PROSITE); G3DSA:1.20.1270.10 (GENE3D), G3DSA:2.60.34.10 (GENE3D), G3DSA:3.30.420.40 (GENE3D), G3DSA:3.90.640.10 (GENE3D), PTHR19375 (PANTHER), PTHR19375:SF7 (PANTHER), SignalP-NN(euk) (SIGNALP), tmhmm (TMHMM), SSF100934 (SUPERFAMILY), SSF53067 (SUPERFAMILY) |
| 912 | lib_dib_Filter15_IDBA_contig_19064_plus2 | 385 | ---NA--- | 715 | | | 0 | - | SignalP-NN(euk) (SIGNALP), tmhmm (TMHMM) |
| 913 | lib_dib_Filter15_IDBA_contig_19079_minus1 | 343 | pro-cathepsin h | 558 | 2.94E-117 | 65.75% | 14 | P:anatomical structure morphogenesis; F:peptidase activity; P:biological_process; P:catabolic process; F:protein binding; P:regulation of biological process; P:cell death; C:organelle; C:intracellular; P:metabolic process; P:multicellular organismal development; P:signal transduction; P:protein metabolic process; C:cytoplasm | IPR000169 (PROSITE); IPR000668 (PRINTS); IPR013128 (PANTHER); IPR013201 (PFAM); IPR025660 (PROSITE); G3DSA:3.90.70.10 (GENE3D), PTHR12411:SF49 (PANTHER), tmhmm (TMHMM), SSF54001 (SUPERFAMILY) |
| 914 | lib_dib_Filter15_IDBA_contig_19094_minus3 | 524 | disks large homolog 1 isoform 5 | 1069 | 0 | 77.50% | 12 | P:metabolic process; C:cell; P:anatomical structure morphogenesis; P:multicellular organismal development; C:plasma membrane; P:regulation of biological process; P:cell proliferation; P:biological_process; P:cellular component organization; C:cellular_component; P:protein metabolic process; F:protein binding | IPR001452 (SMART); IPR001478 (PFAM); IPR008144 (PFAM); IPR008145 (SMART); IPR011511 (PFAM); IPR020590 (PROSITE); G3DSA:2.30.30.40 (GENE3D), G3DSA:2.30.42.10 (GENE3D), G3DSA:3.30.63.10 (GENE3D), G3DSA:3.40.50.300 (GENE3D), PTHR23119 (PANTHER), SSF52540 (SUPERFAMILY) |

| | | | | | | | | | |
|---|---|---|---|---|---|---|---|---|---|
| 915 | lib_dib_Filter15_IDBA_contig_19117_minus1 | 645 | cyclin b | 642 | 2.07E-137 | 77.10% | 7 | P:organelle organization; P:cell cycle; F:protein binding; C:nucleus; P:metabolic process; P:regulation of biological process; P:biological_process | IPR004367 (PFAM); IPR006671 (PFAM); IPR013763 (G3DSA:1.10.472.GENE3D); PTHR10177 (PANTHER), PTHR10177:SF64 (PANTHER) |
| 916 | lib_dib_Filter15_IDBA_contig_19118_minus1 | 653 | cyclin b | 649 | 2.25E-137 | 77.40% | 7 | P:organelle organization; P:cell cycle; F:protein binding; C:nucleus; P:metabolic process; P:regulation of biological process; P:biological_process | IPR004367 (PFAM); IPR006671 (PFAM); IPR013763 (G3DSA:1.10.472.GENE3D); PTHR10177 (PANTHER), PTHR10177:SF64 (PANTHER) |
| 917 | lib_dib_Filter15_IDBA_contig_19133_minus3 | 623 | gamma-glutamyltranspeptidase 1 | 808 | 1.20E-137 | 60.15% | 10 | P:metabolic process; C:cell; F:transferase activity; C:extracellular space; P:biosynthetic process; P:response to stress; P:biological_process; P:response to endogenous stimulus; P:response to biotic stimulus; | IPR000101 (PRINTS); tmhmm (TMHMM), SSF56235 (SUPERFAMILY) |
| 918 | lib_dib_Filter15_IDBA_contig_19134_minus3 | 209 | gamma-glutamyltranspeptidase 1 | 285 | 4.48E-30 | 65.20% | 11 | P:metabolic process; P:regulation of biological process; P:biosynthetic process; F:transferase activity; C:extracellular space; P:response to stress; ; P:biological_process; P:response to endogenous stimulus; P:response to biotic stimulus; C:plasma membrane | IPR000101 (PRINTS); tmhmm (TMHMM), SSF56235 (SUPERFAMILY) |
| 919 | lib_dib_Filter15_IDBA_contig_19137_minus3 | 2762 | adp-ribosylation factor 4 | 1252 | 3.34E-110 | 94.35% | 8 | F:nucleotide binding; F:signal transducer activity; C:Golgi apparatus; C:plasma membrane; F:hydrolase activity; P:transport; P:signal transduction; P:protein transport | IPR003579 (SMART); IPR005225 (TIGRFAMs); IPR006687 (SMART); IPR006689 (PRINTS); IPR024156 (SMART); G3DSA:3.40.50.300 (GENE3D), PTHR11711 (PANTHER), tmhmm (TMHMM), SSF52540 (SUPERFAMILY) |
| 920 | lib_dib_Filter15_IDBA_contig_19138_minus1 | 66 | variable lymphocyte receptor a | 555 | 7.25E-19 | 52.95% | 1 | F:protein binding | IPR001611 (PFAM); IPR003591 (SMART); G3DSA:3.80.10.10 (GENE3D), PTHR24369 (PANTHER), PF13855 (PFAM), SSF52058 (SUPERFAMILY) |
| 921 | lib_dib_Filter15_IDBA_contig_19148_minus1 | 80 | zinc finger protein | 554 | 3.38E-52 | 50.20% | 3 | F:binding; F:nucleic acid binding; C:intracellular | IPR007087 (PROSITE); IPR013087 (G3DSA:3.30.160.GENE3D); IPR015880 (SMART); PTHR24375 (PANTHER), PTHR24375:SF0 (PANTHER), PF13465 (PFAM), PF13894 (PFAM), SSF57667 (SUPERFAMILY) |
| 922 | lib_dib_Filter15_IDBA_contig_19164_minus2 | 659 | leucine-rich repeat and calponin homology domain-containing protein 3 | 1356 | 1.24E-90 | 65.95% | 1 | F:protein binding | IPR001611 (PROFILE); IPR001715 (G3DSA:1.10.418.GENE3D); IPR003591 (SMART); IPR025875 (PFAM); G3DSA:3.80.10.10 (GENE3D), PTHR23155 (PANTHER), PTHR23155:SF10 (PANTHER), SM00364 (SMART), tmhmm (TMHMM), SSF52058 (SUPERFAMILY) |
| 923 | lib_dib_Filter15_IDBA_contig_19165_minus2 | 816 | leucine-rich repeat and calponin homology domain-containing protein 3 | 1624 | 3.58E-90 | 65.95% | 1 | F:protein binding | IPR001611 (PROFILE); IPR001715 (G3DSA:1.10.418.GENE3D); IPR003591 (SMART); IPR025875 (PFAM); G3DSA:3.80.10.10 (GENE3D), PTHR23155 (PANTHER), PTHR23155:SF10 (PANTHER), SM00364 (SMART), SSF52058 (SUPERFAMILY) |

| | | | | | | | | | | |
|---|---|---|---|---|---|---|---|---|---|---|
| 924 | lib_dib_Filter15_IDBA_contig_19173_minus1 | 88 | neurogenic locus notch homolog protein 2-like | 553 | 2.43E-76 | 51.50% | 26 | P:regulation of biological process; P:growth; ; P:response to stress; P:anatomical structure morphogenesis; P:cell differentiation; P:multicellular organismal development; P:biological_process; P:cell proliferation; P:signal transduction; P:cytoskeleton organization; C:protein complex; P:reproduction; C:plasma membrane; P:cellular component organization; P:behavior; P:cell-cell signaling; P:embryo development; P:cell communication; C:organelle; C:intracellular; P:cell cycle; P:metabolic process; F:protein binding; F:DNA binding; F:calcium ion binding | IPR000152 (PROSITE); IPR000742 (PFAM); IPR000859 (G3DSA:2.60.120.GENE3D); IPR001881 (SMART); IPR013032 (PFAM); IPR018097 (PROSITE); PR00010 (PRINTS), G3DSA:2.10.25.10 (GENE3D), PTHR24044 (PANTHER), tmhmm (TMHMM), SSF57196 (SUPERFAMILY) | |
| 925 | lib_dib_Filter15_IDBA_contig_19175_plus3 | 172 | tyrosine-protein phosphatase non-receptor type 4 | 684 | 0 | 72.40% | 5 | C:cytoplasm; F:phosphoprotein phosphatase activity; F:protein kinase activity; F:protein binding; C:plasma membrane | IPR000242 (PRINTS); IPR000387 (PROFILE); IPR001478 (PFAM); IPR003595 (SMART); IPR016130 (PROSITE); G3DSA:2.30.42.10 (GENE3D), G3DSA:3.90.190.10 (GENE3D), PTHR19134 (PANTHER), PTHR19134:SF58 (PANTHER), tmhmm (TMHMM), SSF52799 (SUPERFAMILY) | |
| 926 | lib_dib_Filter15_IDBA_contig_19190_minus3 | 337 | nicotinic acetylcholine alpha7-2 subunit | 699 | 1.00E-26 | 49.25% | 3 | P:ion transport; C:cell; F:ion channel activity | IPR006201 (PANTHER); IPR006202 (G3DSA:2.70.170.GENE3D); PTHR18945:SF71 (PANTHER), SignalP-NN(euk) (SIGNALP), tmhmm (TMHMM) | Yes |
| 927 | lib_dib_Filter15_IDBA_contig_19199_plus3 | 1010 | fibrinogen c domain-containing protein 1-like | 430 | 1.64E-45 | 58.30% | 4 | F:receptor binding; C:extracellular space; P:signal transduction; P:blood vessel development | IPR002181 (PFAM); IPR014715 (G3DSA:4.10.530.GENE3D); IPR014716 (G3DSA:3.90.215.GENE3D); PTHR19143 (PANTHER), SignalP-NN(euk) (SIGNALP), tmhmm (TMHMM) | Yes |
| 928 | lib_dib_Filter15_IDBA_contig_19203_plus1 | 74 | fibrinogen-like 1 | 200 | 5.81E-14 | 61.95% | 3 | C:extracellular space; P:biological_process; F:carbohydrate binding | IPR002181 (PFAM); IPR014715 (G3DSA:4.10.530.GENE3D); PTHR19143 (PANTHER) | |
| 929 | lib_dib_Filter15_IDBA_contig_19204_plus1 | 112 | fibrinogen c domain-containing protein 1-like | 155 | 5.75E-33 | 59.05% | 4 | F:receptor binding; C:extracellular space; P:signal transduction; F:carbohydrate binding | IPR002181 (PFAM); IPR014715 (G3DSA:4.10.530.GENE3D); IPR014716 (G3DSA:3.90.215.GENE3D); PTHR19143 (PANTHER) | |
| 930 | lib_dib_Filter15_IDBA_contig_19236_plus3 | 56 | protein turtle-like | 549 | 1.92E-96 | 50.10% | 7 | P:anatomical structure morphogenesis; P:cellular component organization; P:cell differentiation; P:multicellular organismal development; P:cell recognition; P:behavior; F:protein binding | IPR003598 (SMART); IPR003599 (SMART); IPR003961 (PFAM); IPR007110 (PROFILE); IPR013098 (PFAM); IPR013783 (G3DSA:2.60.40.GENE3D); PTHR10489 (PANTHER), PF13895 (PFAM), SSF48726 (SUPERFAMILY) | |
| 931 | lib_dib_Filter15_IDBA_contig_19238_minus2 | 393 | downstream of receptor kinase | 549 | 7.86E-91 | 75.80% | 14 | P:signal transduction; P:anatomical structure morphogenesis; P:cellular component organization; P:regulation of biological process; P:behavior; P:metabolic process; P:cell differentiation; P:multicellular organismal development; P:cytoskeleton organization; P:embryo development; F:protein binding; F:signal transducer activity; C:plasma membrane; F:receptor binding | IPR000108 (PRINTS); IPR000980 (PRINTS); IPR001452 (PRINTS); IPR011511 (PFAM); G3DSA:2.30.30.40 (GENE3D), PTHR22820 (PANTHER), PTHR22820:SF13 (PANTHER), tmhmm (TMHMM), SSF55550 (SUPERFAMILY) | |

| | | | | | | | | | |
|---|---|---|---|---|---|---|---|---|---|
| 932 | lib_dib_Filter15_IDBA_contig_19261_plus3 | 2143 | cathepsin l-like cysteine proteinase | 548 | 3.22E-147 | 74.05% | 3 | P:protein metabolic process; P:catabolic process; F:peptidase activity | IPR000169 (PROSITE); IPR000668 (PRINTS); IPR013128 (PANTHER); IPR013201 (PFAM); IPR025660 (PROSITE); IPR025661 (PROSITE); G3DSA:3.90.70.10 (GENE3D), PTHR12411:SF149 (PANTHER), SignalP-NN(euk) (SIGNALP), tmhmm (TMHMM), SSF54001 (SUPERFAMILY) |
| 933 | lib_dib_Filter15_IDBA_contig_19272_plus3 | 116 | plexin a | 548 | 4.27E-19 | 65.70% | 7 | F:receptor activity; F:protein binding; P:anatomical structure morphogenesis; P:cellular component organization; P:cell differentiation; P:multicellular organismal development; P:cell recognition | IPR001627 (PFAM); IPR015943 (G3DSA:2.130.10.GENE3D); PTHR22625 (PANTHER), PTHR22625:SF12 (PANTHER) |
| 934 | lib_dib_Filter15_IDBA_contig_19282_minus2 | 643 | ---NA--- | 977 | | | 0 | - | SignalP-NN(euk) (SIGNALP) |
| 935 | lib_dib_Filter15_IDBA_contig_19282_plus1 | 643 | egf-like domain-containing protein | 977 | 6.87E-19 | 51.25% | 7 | C:cell; C:plasma membrane; F:protein binding; F:calcium ion binding; C:extracellular region; P:carbohydrate metabolic process; F:carbohydrate binding | IPR000152 (PROSITE); IPR000742 (SMART); IPR001881 (PFAM); IPR002557 (G3DSA:2.170.140.GENE3D); IPR008160 (PFAM); IPR013032 (PROSITE); G3DSA:2.10.25.10 (GENE3D), PTHR24023 (PANTHER), PTHR24023:SF164 (PANTHER), SignalP-NN(euk) (SIGNALP), tmhmm (TMHMM), SSF57196 (SUPERFAMILY) |
| 936 | lib_dib_Filter15_IDBA_contig_19290_minus1 | 415 | ankyrin repeat domain-containing protein 6-like | 1032 | 4.52E-61 | 72.80% | 1 | F:protein binding | IPR002110 (PRINTS); IPR020683 (G3DSA:1.25.40.GENE3D); PTHR24203 (PANTHER), PTHR24203:SF0 (PANTHER), tmhmm (TMHMM) |
| 937 | lib_dib_Filter15_IDBA_contig_19295_minus2 | 285 | von willebrand factor type egf and pentraxin domain-containing protein 1 | 762 | 5.29E-12 | 48.40% | 2 | P:regulation of biological process; F:carbohydrate binding | IPR000436 (PFAM); IPR000859 (G3DSA:2.60.120.GENE3D); IPR000922 (PFAM); G3DSA:2.10.70.10 (GENE3D), PTHR19325 (PANTHER), SignalP-NN(euk) (SIGNALP), tmhmm (TMHMM) |
| 938 | lib_dib_Filter15_IDBA_contig_19298_minus1 | 835 | snake venom metalloprotease inhibitor precursor | 1810 | 4.86E-08 | 52.00% | 7 | F:hormone activity; F:metalloendopeptidase inhibitor activity; P:regulation of blood vessel size; P:negative regulation of catalytic activity; F:enzyme inhibitor activity; C:extracellular region; P:regulation of blood pressure | no IPS match |
| 939 | lib_dib_Filter15_IDBA_contig_19344_minus1 | 636 | n-acetylated-alpha-linked acidic dipeptidase-like protein | 1050 | 1.03E-62 | 45.45% | 2 | P:proteolysis; F:peptidase activity | IPR003137 (PFAM); G3DSA:3.40.630.10 (GENE3D), G3DSA:3.50.30.30 (GENE3D), PTHR10404 (PANTHER), tmhmm (TMHMM), SSF52025 (SUPERFAMILY), SSF53187 (SUPERFAMILY) |
| 940 | lib_dib_Filter15_IDBA_contig_19408_plus2 | 452 | protein phosphatase 1 regulatory subunit 12a | 925 | 2.60E-135 | 76.60% | 18 | F:phosphoprotein phosphatase activity; P:transport; P:regulation of biological process; F:signal transducer activity; P:biological_process; P:cytoskeleton organization; F:protein binding; F:catalytic activity; C:protein complex; C:chromosome; P:organelle organization; P:cell cycle; C:microtubule organizing center; P:signal transduction; ; P:cellular protein modification process; F:enzyme regulator activity; C:cytoplasm | IPR002110 (SMART); IPR020683 (G3DSA:1.25.40.GENE3D); PTHR24179 (PANTHER), PTHR24179:SF0 (PANTHER) |
| 941 | lib_dib_Filter15_IDBA_contig_19417_minus2 | 388 | 15-hydroxyprostaglandin dehydrogenase | 542 | 3.53E-44 | 55.90% | 6 | P:lipid metabolic process; P:metabolic process; F:catalytic activity; F:nucleotide binding; P:regulation of biological process; P:reproduction | IPR002198 (PRINTS); IPR002347 (PRINTS); IPR016040 (G3DSA:3.40.50.GENE3D); PTHR24310 (PANTHER), PTHR24310:SF0 (PANTHER), SSF51735 (SUPERFAMILY) |

| | | | | | | | | | |
|---|---|---|---|---|---|---|---|---|---|
| 942 | lib_dib_Filter15_IDBA_contig_19426_plus2 | 117 | tyrosine-protein phosphatase 10d | 542 | 1.55E-31 | 43.40% | 7 | P:anatomical structure morphogenesis; P:response to external stimulus; P:response to stress; P:multicellular organismal development; P:biological_process; C:extracellular region; F:protein binding | IPR003961 (PFAM); IPR013783 (G3DSA:2.60.40.GENE3D); PTHR19143 (PANTHER) |
| 943 | lib_dib_Filter15_IDBA_contig_19432_plus3 | 111 | low-density lipoprotein receptor-related protein 2 | 541 | 5.61E-21 | 47.65% | 5 | F:receptor activity; P:transport; P:cellular component organization; C:cell; F:protein binding | IPR000998 (PFAM); IPR002172 (G3DSA:4.10.400.GENE3D); IPR008985 (SUPERFAMILY); IPR023415 (PROSITE); PR00261 (PRINTS), G3DSA:4.10.1220.10 (GENE3D), PTHR10529 (PANTHER), PTHR10529:SF99 (PANTHER) |
| 944 | lib_dib_Filter15_IDBA_contig_19450_minus2 | 189 | cartilage matrix protein | 742 | 0 | 52.15% | 2 | F:protein binding; F:calcium ion binding | IPR000152 (PROSITE); IPR000742 (PFAM); IPR001881 (SMART); IPR002035 (PFAM); IPR013032 (PROSITE); PR00453 (PRINTS), G3DSA:2.10.25.10 (GENE3D), G3DSA:3.40.50.410 (GENE3D), PTHR22992 (PANTHER), SSF53300 (SUPERFAMILY) |
| 945 | lib_dib_Filter15_IDBA_contig_19452_minus1 | 715 | hydroxysteroid dehydrogenase-like protein 2 | 540 | 0 | 75.10% | 4 | P:metabolic process; F:catalytic activity; F:lipid binding; F:nucleotide binding | IPR002198 (PFAM); IPR002347 (PRINTS); IPR003033 (PFAM); IPR016040 (G3DSA:3.40.50.GENE3D); G3DSA:3.30.1050.10 (GENE3D), PTHR24322 (PANTHER), PTHR24322:SF43 (PANTHER), SSF51735 (SUPERFAMILY) |
| 946 | lib_dib_Filter15_IDBA_contig_19453_plus1 | 109 | iduronate 2-sulfatase-like | 540 | 2.73E-170 | 63.35% | 2 | P:metabolic process; F:hydrolase activity | IPR000917 (PFAM); IPR017849 (G3DSA:3.40.720.GENE3D); IPR017850 (SUPERFAMILY); G3DSA:3.30.1120.10 (GENE3D), PTHR10342 (PANTHER), PTHR10342:SF13 (PANTHER) |
| 947 | lib_dib_Filter15_IDBA_contig_19458_plus3 | 108 | zinc finger protein | 540 | 1.38E-86 | 55.70% | 2 | F:protein binding; F:binding | IPR001660 (SMART); IPR001965 (SMART); IPR011011 (SUPERFAMILY); IPR013083 (G3DSA:3.30.40.GENE3D); IPR013761 (G3DSA:1.10.150.GENE3D); IPR019787 (PFAM); IPR021129 (PFAM); PTHR12247 (PANTHER), PTHR12247:SF19 (PANTHER), tmhmm (TMHMM) |
| 948 | lib_dib_Filter15_IDBA_contig_19484_minus1 | 87 | hemicentin-1 | 539 | 1.50E-123 | 57.60% | 1 | F:protein binding | IPR003598 (SMART); IPR003599 (SMART); IPR007110 (PROFILE); IPR013098 (PFAM); IPR013783 (G3DSA:2.60.40.GENE3D); SSF48726 (SUPERFAMILY) |
| 949 | lib_dib_Filter15_IDBA_contig_19525_plus1 | 14014 | | 537 | 0 | 100% | 1 | F:protein binding | IPR003598 (SMART); IPR003599 (SMART); IPR003961 (PFAM); IPR007110 (PROFILE); IPR013098 (PFAM); IPR013783 (G3DSA:2.60.40.GENE3D); PR00014 (PRINTS), SSF48726 (SUPERFAMILY) |
| 950 | lib_dib_Filter15_IDBA_contig_19526_plus1 | 3508 | | 154 | 0 | 100% | 1 | F:protein binding | IPR003961 (PFAM); IPR013783 (G3DSA:2.60.40.GENE3D); PR00014 (PRINTS) |
| 951 | lib_dib_Filter15_IDBA_contig_19530_plus3 | 9570 | | 349 | 0 | 100% | 1 | F:protein binding | IPR003961 (PFAM); IPR013783 (G3DSA:2.60.40.GENE3D); PR00014 (PRINTS) |

| | | | | | | | | | | |
|---|---|---|---|---|---|---|---|---|---|---|
| 952 | lib_dib_Filter15_IDBA_contig_19531_plus1 | 4085 | low quality protein: titin | 138 | 2.99E-18 | 51.00% | 18 | C:organelle; C:intracellular; F:protein kinase activity; P:multicellular organismal development; P:regulation of biological process; P:primary metabolic process; P:metabolic process; P:cellular component organization; P:biological_process; P:anatomical structure morphogenesis; P:cell differentiation; P:cytoskeleton organization; C:chromosome; F:protein binding; C:cytoplasm; P:organelle organization; P:cellular protein modification process; F:cytoskeletal protein binding | IPR003598 (SMART); IPR003599 (SMART); IPR007110 (PROFILE); IPR013098 (PFAM); IPR013783 (G3DSA:2.60.40.GENE3D); PTHR10489 (PANTHER), PF13895 (PFAM), SSF48726 (SUPERFAMILY) | |
| 953 | lib_dib_Filter15_IDBA_contig_19532_plus2 | 4830 | titin-like | 169 | 1.80E-46 | 60.10% | 2 | F:transferase activity; F:protein binding | IPR003961 (PFAM); IPR013783 (G3DSA:2.60.40.GENE3D); PR00014 (PRINTS) | |
| 954 | lib_dib_Filter15_IDBA_contig_19535_plus1 | 11484 | | 272 | 0 | 100% | 1 | F:protein binding | IPR003598 (SMART); IPR003599 (SMART); IPR003961 (PFAM); IPR007110 (PROFILE); IPR013098 (PFAM); IPR013783 (G3DSA:2.60.40.GENE3D); PR00014 (PRINTS), SSF48726 (SUPERFAMILY) | |
| 955 | lib_dib_Filter15_IDBA_contig_19536_plus3 | 9566 | | 198 | 0 | 100% | 1 | F:protein binding | IPR003961 (PFAM); IPR013783 (G3DSA:2.60.40.GENE3D); PR00014 (PRINTS) | |
| 956 | lib_dib_Filter15_IDBA_contig_19576_plus3 | 205 | retinoid-inducible serine carboxypeptidase | 535 | 7.10E-61 | 52.30% | 3 | F:peptidase activity; P:protein metabolic process; P:catabolic process | IPR001563 (PRINTS); G3DSA:3.40.50.1820 (GENE3D), PTHR11802:SF3 (PANTHER), SSF53474 (SUPERFAMILY) | |
| 957 | lib_dib_Filter15_IDBA_contig_19586_plus1 | 88 | natriuretic peptide receptor 1-like | 535 | 3.52E-85 | 60.00% | 12 | F:binding; F:catalytic activity; F:protein binding; F:receptor activity; P:signal transduction; P:multicellular organismal development; C:cell; P:nucleobase-containing compound metabolic process; P:biosynthetic process; F:nucleotide binding; P:cellular protein modification process; F:protein kinase activity | IPR000719 (PROFILE); IPR001054 (G3DSA:3.30.70.GENE3D); IPR001245 (PFAM); IPR002290 (SMART); IPR011009 (SUPERFAMILY); IPR020635 (SMART); G3DSA:1.10.510.10 (GENE3D), G3DSA:3.30.200.20 (GENE3D), PTHR11920 (PANTHER), PTHR11920:SF50 (PANTHER), tmhmm (TMHMM) | |
| 958 | lib_dib_Filter15_IDBA_contig_19596_minus2 | 98 | zinc finger protein 271-like | 581 | 3.20E-117 | 61.80% | 3 | F:binding; F:nucleic acid binding; C:intracellular | IPR007087 (PFAM); IPR013087 (G3DSA:3.30.160.GENE3D); IPR015880 (SMART); PTHR24402 (PANTHER), PF13465 (PFAM), SSF57667 (SUPERFAMILY) | |
| 959 | lib_dib_Filter15_IDBA_contig_19597_minus2 | 112 | nuak family snf1-like kinase 1 | 534 | 1.09E-128 | 82.10% | 10 | P:regulation of biological process; P:cellular protein modification process; F:protein binding; F:protein kinase activity; F:binding; F:nucleotide binding; P:cell proliferation; C:cytoplasm; C:nucleus; P:response to stress | IPR000719 (PFAM); IPR002290 (SMART); IPR008271 (PROSITE); IPR011009 (SUPERFAMILY); IPR017441 (PROSITE); IPR020635 (SMART); G3DSA:1.10.510.10 (GENE3D), G3DSA:3.30.200.20 (GENE3D), PTHR24343 (PANTHER), PTHR24343:SF27 (PANTHER) | |
| 960 | lib_dib_Filter15_IDBA_contig_19600_minus2 | 633 | integrin alpha fg-gap repeat containing 1 | 737 | 1.04E-107 | 53.00% | 3 | C:integral to membrane; C:extracellular region; P:integrin-mediated signaling pathway | IPR024881 (PANTHER); G3DSA:2.130.10.130 (GENE3D), PTHR13412:SF0 (PANTHER), PF13517 (PFAM), SignalP-NN(euk) (SIGNALP), tmhmm (TMHMM), SSF69318 (SUPERFAMILY) | Yes |

| | | | | | | | | | |
|---|---|---|---|---|---|---|---|---|---|
| 961 | lib_dib_Filter15_IDBA_contig_19601_minus2 | 80 | agrn partial | 534 | 1.60E-50 | 59.20% | 17 | C:cell; P:signal transduction; F:signal transducer activity; P:regulation of biological process; P:cell death; P:cellular component organization; C:extracellular space; P:cell differentiation; P:cell-cell signaling; P:multicellular organismal development; P:growth; P:cellular protein modification process; ; P:metabolic process; F:protein binding; C:cellular_component; C:proteinaceous extracellular matrix | IPR001791 (PFAM); IPR008985 (SUPERFAMILY); IPR013320 (G3DSA:2.60.120.GENE3D); PTHR10574 (PANTHER), PTHR10574:SF10 (PANTHER) |
| 962 | lib_dib_Filter15_IDBA_contig_19611_minus1 | 84 | cub domain-containing | 533 | 4.89E-98 | 50.55% | 1 | F:protein binding | IPR000859 (G3DSA:2.60.120.GENE3D); IPR002172 (G3DSA:4.10.400.GENE3D); PTHR10127 (PANTHER), PTHR10127:SF345 (PANTHER), tmhmm (TMHMM) |
| 963 | lib_dib_Filter15_IDBA_contig_19629_minus3 | 924 | a chain x-ray structure of c-met with triazolopyridazine | 1150 | 3.16E-84 | 66.80% | 17 | P:anatomical structure morphogenesis; P:cellular component organization; P:cell differentiation; P:multicellular organismal development; P:signal transduction; P:reproduction; P:response to biotic stimulus; F:receptor activity; F:protein kinase activity; P:biological_process; C:plasma membrane; P:regulation of biological process; P:cell proliferation; P:metabolic process; F:protein binding; P:response to stress; F:nucleotide binding | IPR000719 (PROFILE); IPR001245 (PRINTS); IPR002011 (PROSITE); IPR002290 (SMART); IPR002909 (PFAM); IPR008211 (PROFILE); IPR008266 (PROSITE); IPR008985 (SUPERFAMILY); IPR011009 (SUPERFAMILY); IPR013783 (G3DSA:2.60.40.GENE3D); IPR014756 (SUPERFAMILY); IPR017441 (PROSITE); IPR020635 (SMART); G3DSA:1.10.510.10 (GENE3D), G3DSA:3.30.200.20 (GENE3D), PTHR24416 (PANTHER), tmhmm (TMHMM) |
| 964 | lib_dib_Filter15_IDBA_contig_19633_plus2 | 89 | peptidyl-prolyl cis-trans isomerase-like 2-like | 532 | 1.20E-179 | 71.80% | 3 | P:cellular protein modification process; F:catalytic activity; P:protein metabolic process | IPR002130 (PRINTS); IPR020892 (PROSITE); IPR026951 (PTHR11071:PANTHER); G3DSA:2.40.100.10 (GENE3D), PTHR11071 (PANTHER) |
| 965 | lib_dib_Filter15_IDBA_contig_19644_minus3 | 223 | alpha- -mannosyl-glycoprotein 4-beta-n-acetylglucosaminyltransferase c-like | 698 | 3.52E-153 | 62.55% | 2 | P:carbohydrate metabolic process; C:cell | IPR006759 (PANTHER) |
| 966 | lib_dib_Filter15_IDBA_contig_19649_plus2 | 1714 | somatomedin-b and thrombospondin type-1 domain-containing | 1301 | 5.48E-84 | 46.25% | 3 | F:receptor activity; P:biological_process; F:carbohydrate binding | IPR000884 (SMART); IPR001212 (PROSITE); G3DSA:2.20.100.10 (GENE3D), PTHR20920 (PANTHER), PTHR20920:SF0 (PANTHER), tmhmm (TMHMM), SSF90188 (SUPERFAMILY) |
| 967 | lib_dib_Filter15_IDBA_contig_19658_plus1 | 250 | wd40 repeat-containing protein smu1 | 597 | 0 | 93.90% | 3 | C:nucleus; C:cytoplasm; F:protein binding | IPR001680 (PFAM); IPR006594 (SMART); IPR006595 (SMART); IPR015943 (G3DSA:2.130.10.GENE3D); IPR017986 (PROFILE); IPR019775 (PROSITE); IPR020472 (PRINTS); PTHR22848 (PANTHER) |
| 968 | lib_dib_Filter15_IDBA_contig_19659_minus3 | 3274 | 14-3-3 protein | 581 | 1.63E-105 | 82.05% | 19 | P:cellular protein modification process; P:regulation of biological process; P:cellular component organization; F:protein binding; F:transcription regulator activity; P:behavior; P:reproduction; P:biological_process; P:growth; P:multicellular organismal development; C:nucleoplasm; C:protein complex; P:protein transport; ; F:catalytic activity; P:embryo development; C:microtubule organizing center; P:metabolic process; C:cytoplasm | IPR000308 (PRINTS); IPR023409 (PROSITE); IPR023410 (G3DSA:1.20.190.GENE3D); PTHR18860:SF0 (PANTHER) |

| | | | | | | | | | | |
|---|---|---|---|---|---|---|---|---|---|---|
| 969 | lib_dib_Filter15_IDBA_contig_19679_plus1 | 171 | retinal dehydrogenase 2 isoform 1 | 530 | 0 | 80.15% | 22 | P:multicellular organismal development; P:metabolic process; P:regulation of biological process; F:catalytic activity; P:embryo development; P:signal transduction; P:anatomical structure morphogenesis; P:cell differentiation; P:lipid metabolic process; P:secondary metabolic process; P:biological_process; C:cytosol; P:cell proliferation; P:catabolic process; P:response to external stimulus; P:cell communication; P:biosynthetic process; F:lipid binding; P:cell death; C:cytoplasm; C:nucleus; P:response to endogenous stimulus | IPR015590 (PFAM); IPR016160 (PROSITE); IPR016161 (SUPERFAMILY); IPR016162 (G3DSA:3.40.605.GENE3D); IPR016163 (G3DSA:3.40.309.GENE3D); PTHR11699 (PANTHER), PTHR11699:SF46 (PANTHER) | |
| 970 | lib_dib_Filter15_IDBA_contig_19685_minus2 | 275 | peptidyl-prolyl cis-trans isomerase cwc27 homolog | 644 | 7.63E-132 | 65.25% | 4 | P:nucleobase-containing compound metabolic process; C:nucleus; F:catalytic activity; P:protein metabolic process | IPR002130 (PRINTS); G3DSA:2.40.100.10 (GENE3D), PTHR11071 (PANTHER), PTHR11071:SF33 (PANTHER) | |
| 971 | lib_dib_Filter15_IDBA_contig_19687_minus2 | 288 | peptidyl-prolyl cis-trans isomerase cwc27 homolog | 654 | 1.78E-131 | 65.25% | 4 | P:nucleobase-containing compound metabolic process; C:nucleus; F:catalytic activity; P:protein metabolic process | IPR002130 (PRINTS); G3DSA:2.40.100.10 (GENE3D), PTHR11071 (PANTHER), PTHR11071:SF33 (PANTHER) | |
| 972 | lib_dib_Filter15_IDBA_contig_19742_plus1 | 814 | defense protein 3-like | 741 | 2.62E-18 | 54.85% | 9 | P:carbohydrate metabolic process; F:cation binding; F:catalytic activity; P:oxidation-reduction process; F:oxidoreductase activity; C:integral to membrane; C:membrane; P:electron transport chain; P:transport | IPR002861 (PFAM); PTHR23130 (PANTHER), SignalP-NN(euk) (SIGNALP), tmhmm (TMHMM) | |
| 973 | lib_dib_Filter15_IDBA_contig_19749_minus1 | 382 | para-nitrobenzyl esterase-like | 806 | 7.37E-116 | 54.10% | 1 | F:carboxylic ester hydrolase activity | IPR002018 (PFAM); G3DSA:3.40.50.1820 (GENE3D), PTHR11559 (PANTHER), SignalP-NN(euk) (SIGNALP), tmhmm (TMHMM), SSF53474 (SUPERFAMILY) | |
| 974 | lib_dib_Filter15_IDBA_contig_19760_plus1 | 9966 | serine-threonine kinase receptor-associated | 723 | 1.53E-157 | 80.00% | 4 | F:receptor activity; F:kinase activity; P:metabolic process; F:protein binding | IPR001680 (PFAM); IPR015943 (G3DSA:2.130.10.GENE3D); IPR017986 (PROFILE); IPR019775 (PROSITE); IPR020472 (PRINTS); PTHR19877 (PANTHER), PTHR19877:SF2 (PANTHER) | |
| 975 | lib_dib_Filter15_IDBA_contig_19771_minus2 | 201 | zinc finger protein zfat | 1052 | 0 | 50.30% | 5 | P:biological_process; P:multicellular organismal development; C:intracellular; F:binding; F:nucleic acid binding | IPR007087 (PROSITE); IPR013087 (G3DSA:3.30.160.GENE3D); IPR015880 (SMART); PTHR24387 (PANTHER), PTHR24387:SF8 (PANTHER), PF13465 (PFAM), PF13894 (PFAM), SSF57667 (SUPERFAMILY) | |
| 976 | lib_dib_Filter15_IDBA_contig_19772_minus2 | 216 | zinc finger protein zfat | 1145 | 0 | 50.30% | 5 | P:biological_process; P:multicellular organismal development; C:intracellular; F:binding; F:nucleic acid binding | IPR007087 (PROSITE); IPR013087 (G3DSA:3.30.160.GENE3D); IPR015880 (SMART); PTHR24387 (PANTHER), PTHR24387:SF8 (PANTHER), PF13465 (PFAM), PF13894 (PFAM), SSF57667 (SUPERFAMILY) | |
| 977 | lib_dib_Filter15_IDBA_contig_19796_plus2 | 3905 | neuronal acetylcholine receptor subunit alpha-7-like | 786 | 4.81E-74 | 55.35% | 4 | C:plasma membrane; C:cell; F:ion channel activity; P:ion transport | IPR002394 (PRINTS); IPR006029 (SUPERFAMILY); IPR006201 (PRINTS); IPR006202 (G3DSA:2.70.170.GENE3D); IPR018000 (PROSITE); G3DSA:1.20.120.370 (GENE3D), SignalP-NN(euk) (SIGNALP), tmhmm (TMHMM) | Yes |
| 978 | lib_dib_Filter15_IDBA_contig_19816_plus2 | 62 | hypothetical protein BRAFLDRAFT_98740 | 524 | 7.85E-84 | 48.65% | 2 | F:protein binding; F:calcium ion binding | IPR000152 (PROSITE); IPR000742 (SMART); IPR001881 (PFAM); IPR013032 (PROSITE); IPR018097 (PROSITE); IPR023413 (G3DSA:2.40.155.GENE3D); IPR024731 (PFAM); G3DSA:2.10.25.10 (GENE3D), PTHR24048 (PANTHER), PTHR24048:SF8 (PANTHER), SSF57196 (SUPERFAMILY) | |

| | | | | | | | | | | |
|---|---|---|---|---|---|---|---|---|---|---|
| 979 | lib_dib_Filter15_IDBA_contig_19889_plus3 | 121 | plexin a cg11081-pa | 523 | 5.56E-129 | 61.20% | 1 | F:protein binding | IPR002909 (PFAM); IPR013548 (PFAM); IPR013783 (G3DSA:2.60.40.GENE3D); IPR014756 (SUPERFAMILY); PTHR22625 (PANTHER), tmhmm (TMHMM) | |
| 980 | lib_dib_Filter15_IDBA_contig_19890_plus1 | 972 | myostatin | 1191 | 7.58E-58 | 52.15% | 2 | F:receptor binding; P:growth | IPR001111 (PFAM); IPR001839 (PFAM); IPR015615 (PANTHER); IPR017948 (PROSITE); G3DSA:2.10.90.10 (GENE3D), PTHR11848:SF15 (PANTHER), tmhmm (TMHMM), SSF57501 (SUPERFAMILY) | |
| 981 | lib_dib_Filter15_IDBA_contig_19891_plus1 | 92 | myostatin | 479 | 7.36E-26 | 54.75% | 1 | F:receptor binding | IPR001839 (PFAM); IPR015615 (PANTHER); IPR017948 (PROSITE); G3DSA:2.10.90.10 (GENE3D), SSF57501 (SUPERFAMILY) | |
| 982 | lib_dib_Filter15_IDBA_contig_19896_plus2 | 4156 | chymotrypsin-like elastase family member 2a | 523 | 8.07E-42 | 51.95% | 4 | C:cell; F:peptidase activity; P:protein metabolic process; P:catabolic process | IPR000998 (PRINTS); IPR001254 (PFAM); IPR001314 (PRINTS); IPR008985 (SUPERFAMILY); IPR009003 (SUPERFAMILY); IPR018114 (PROSITE); G3DSA:2.40.10.10 (GENE3D), PTHR24265 (PANTHER), SignalP-NN(euk) (SIGNALP) | Yes |
| 983 | lib_dib_Filter15_IDBA_contig_19910_minus3 | 357 | fibrinogen c domain-containing protein 1 | 746 | 2.68E-109 | 59.45% | 4 | F:receptor binding; C:extracellular space; P:signal transduction; F:calcium ion binding | IPR002181 (PFAM); IPR014715 (G3DSA:4.10.530.GENE3D); IPR014716 (G3DSA:3.90.215.GENE3D); PTHR19143 (PANTHER) | |
| 984 | lib_dib_Filter15_IDBA_contig_19911_minus3 | 272 | fibrinogen c domain-containing protein 1 | 592 | 8.69E-111 | 58.55% | 2 | P:biological_process; F:carbohydrate binding | IPR002181 (PFAM); IPR014715 (G3DSA:4.10.530.GENE3D); IPR014716 (G3DSA:3.90.215.GENE3D); PTHR19143 (PANTHER) | |
| 985 | lib_dib_Filter15_IDBA_contig_19940_minus2 | 188 | carbohydrate sulfotransferase 11-like | 520 | 5.93E-26 | 45.35% | 8 | P:anatomical structure morphogenesis; P:multicellular organismal development; P:biological_process; F:transferase activity; C:cell; F:binding; P:biosynthetic process; P:carbohydrate metabolic process | IPR005331 (PFAM); IPR007087 (PROSITE); IPR018011 (PANTHER); SignalP-NN(euk) (SIGNALP), tmhmm (TMHMM) | |
| 986 | lib_dib_Filter15_IDBA_contig_19948_minus2 | 791 | c4b-binding protein beta | 595 | 3.84E-31 | 53.95% | 4 | F:peptidase inhibitor activity; F:hydrolase activity; C:extracellular region; P:negative regulation of peptidase activity | IPR000436 (PFAM); G3DSA:2.10.70.10 (GENE3D), PTHR19325 (PANTHER), tmhmm (TMHMM) | |
| 987 | lib_dib_Filter15_IDBA_contig_19958_minus1 | 215 | wd repeat-containing protein 61 | 827 | 1.68E-174 | 86.90% | 12 | C:nucleoplasm; C:protein complex; P:cell differentiation; P:regulation of biological process; P:multicellular organismal development; F:protein binding; P:signal transduction; ; C:cytoplasm; C:nucleolus; P:organelle organization; P:cellular protein modification process | IPR001680 (PFAM); IPR015943 (G3DSA:2.130.10.GENE3D); IPR017986 (PROFILE); IPR019775 (PROSITE); IPR020472 (PRINTS); PTHR22841 (PANTHER), PTHR22841:SF6 (PANTHER), tmhmm (TMHMM) | |
| 988 | lib_dib_Filter15_IDBA_contig_19967_plus2 | 974 | neurogenic locus notch homolog protein 2-like | 2111 | 0 | 45.30% | 2 | F:protein binding; F:calcium ion binding | IPR000152 (PROSITE); IPR000742 (PFAM); IPR000859 (G3DSA:2.60.120.GENE3D); IPR001881 (PFAM); IPR013032 (PROSITE); IPR013111 (PFAM); IPR018097 (PROSITE); PR00010 (PRINTS), PR01983 (PRINTS), G3DSA:2.10.25.10 (GENE3D), G3DSA:2.170.300.10 (GENE3D), PTHR24044 (PANTHER), SignalP-NN(euk) (SIGNALP), tmhmm (TMHMM), SSF57196 (SUPERFAMILY) | |

| | | | | | | | | | |
|---|---|---|---|---|---|---|---|---|---|
| 989 | lib_dib_Filter15_IDBA_contig_19968_plus1 | 704 | neurogenic locus notch homolog protein 2-like | 1308 | 3.20E-160 | 46.85% | 2 | F:protein binding; F:calcium ion binding | IPR000152 (PROSITE); IPR000742 (PFAM); IPR000859 (G3DSA:2.60.120.GENE3D); IPR001881 (PFAM); IPR013032 (PROSITE); IPR013111 (PFAM); IPR018097 (PROSITE); PR00010 (PRINTS), PR01983 (PRINTS), G3DSA:2.10.25.10 (GENE3D), G3DSA:2.170.300.10 (GENE3D), PTHR24044 (PANTHER), tmhmm (TMHMM), SSF57196 (SUPERFAMILY) |
| 990 | lib_dib_Filter15_IDBA_contig_19969_plus1 | 591 | neurogenic locus notch homolog protein 2-like | 1088 | 1.03E-151 | 48.00% | 2 | F:protein binding; F:calcium ion binding | IPR000152 (PROSITE); IPR000742 (PFAM); IPR000859 (G3DSA:2.60.120.GENE3D); IPR001881 (PFAM); IPR013032 (PROSITE); IPR013111 (PFAM); IPR018097 (PROSITE); PR00010 (PRINTS), PR01983 (PRINTS), G3DSA:2.10.25.10 (GENE3D), G3DSA:2.170.300.10 (GENE3D), PTHR24044 (PANTHER), tmhmm (TMHMM), SSF57196 (SUPERFAMILY) |
| 991 | lib_dib_Filter15_IDBA_contig_19971_plus3 | 1059 | transcription initiation factor tfiid subunit 5 | 879 | 1.75E-173 | 79.60% | 12 | P:cellular component organization; ; F:DNA binding; P:regulation of biological process; C:nucleoplasm; C:protein complex; F:protein binding; C:cytoskeleton; F:sequence-specific DNA binding transcription factor activity; P:cellular protein modification process; P:organelle organization; F:transferase activity | IPR001680 (PFAM); IPR015943 (G3DSA:2.130.10.GENE3D); IPR017986 (PROFILE); IPR019775 (PROSITE); IPR020472 (PRINTS); PTHR19879 (PANTHER), PTHR19879:SF0 (PANTHER) |
| 992 | lib_dib_Filter15_IDBA_contig_19978_minus1 | 815 | serum paraoxonase arylesterase 2-like | 547 | 1.42E-55 | 51.50% | 1 | F:hydrolase activity | IPR002640 (PRINTS); IPR011042 (G3DSA:2.120.10.GENE3D); PTHR11799 (PANTHER), PTHR11799:SF0 (PANTHER), SignalP-NN(euk) (SIGNALP), SSF63829 (SUPERFAMILY) |
| 993 | lib_dib_Filter15_IDBA_contig_2_minus1 | 9244 | cathepsin z-like proteinase | 7460 | 4.49E-06 | 45.50% | 2 | P:proteolysis; F:cysteine-type peptidase activity | no IPS match |
| 994 | lib_dib_Filter15_IDBA_contig_2_minus3 | 9244 | collagen alpha-1 chain | 7459 | 4.39E-08 | 46.80% | 1 | F:protein binding | IPR002035 (PFAM); IPR008160 (PFAM); G3DSA:3.40.50.410 (GENE3D), PTHR24023 (PANTHER), SSF53300 (SUPERFAMILY) |
| 995 | lib_dib_Filter15_IDBA_contig_2_plus1 | 9244 | VtaA24 | 7460 | 7.83E-13 | 52.40% | 2 | C:outer membrane; P:pathogenesis | no IPS match |
| 996 | lib_dib_Filter15_IDBA_contig_20002_plus1 | 311 | reelin isoform 1 | 519 | 1.03E-155 | 62.30% | 21 | P:cellular component organization; F:catalytic activity; P:multicellular organismal development; P:response to external stimulus; P:response to abiotic stimulus; F:receptor binding; P:cell differentiation; P:biological_process; P:regulation of biological process; P:behavior; P:signal transduction; C:extracellular space; C:cell; P:response to endogenous stimulus; P:cellular protein modification process; C:cytoplasm; P:ion transport; P:anatomical structure morphogenesis; P:metabolic process; P:cell-cell signaling; P:response to stress | IPR013032 (PROSITE); PTHR11841 (PANTHER), PTHR11841:SF0 (PANTHER) |

| | | | | | | | | | |
|---|---|---|---|---|---|---|---|---|---|
| 997 | lib_dib_Filter15_IDBA_contig_20003_plus3 | 42 | reelin isoform 1 | 101 | 2.78E-26 | 64.40% | 16 | P:multicellular organismal development; F:receptor binding; P:anatomical structure morphogenesis; P:cellular component organization; P:cell differentiation; P:signal transduction; P:behavior; C:extracellular space; P:metabolic process; P:response to stress; C:cytoplasm; C:proteinaceous extracellular matrix; P:regulation of biological process; ; P:cell-cell signaling; C:cell | IPR000742 (PROFILE); IPR013032 (PROSITE); G3DSA:2.10.25.10 (GENE3D), PTHR11841 (PANTHER), PTHR11841:SF0 (PANTHER) |
| 998 | lib_dib_Filter15_IDBA_contig_20004_plus1 | 35 | reelin isoform 1 | 102 | 1.47E-24 | 62.45% | 6 | P:multicellular organismal development; P:signal transduction; P:regulation of biological process; P:cell differentiation; P:protein metabolic process; F:protein binding | IPR000742 (PROFILE); IPR013032 (PROSITE); G3DSA:2.10.25.10 (GENE3D), PTHR11841 (PANTHER), PTHR11841:SF0 (PANTHER) |
| 999 | lib_dib_Filter15_IDBA_contig_20005_plus3 | 76 | reelin isoform 1 | 108 | 3.06E-23 | 56.60% | 11 | P:biological_process; P:regulation of biological process; P:cell communication; P:signal transduction; P:cell-cell signaling; P:multicellular organismal development; P:cellular component organization; P:metabolic process; P:primary metabolic process; P:cellular protein modification process; F:protein binding | IPR000742 (PROFILE); IPR013032 (PROSITE); G3DSA:2.10.25.10 (GENE3D), PTHR11841 (PANTHER), PTHR11841:SF0 (PANTHER) |
| 1000 | lib_dib_Filter15_IDBA_contig_20006_plus1 | 30 | reelin- partial | 112 | 3.14E-17 | 64.10% | 17 | P:multicellular organismal development; F:receptor binding; P:anatomical structure morphogenesis; P:cellular component organization; P:cell differentiation; P:signal transduction; P:behavior; C:extracellular space; P:metabolic process; P:response to stress; C:cytoplasm; C:proteinaceous extracellular matrix; F:hydrolase activity; P:regulation of biological process; ; P:cell-cell signaling; C:cell | PTHR11841 (PANTHER), PTHR11841:SF0 (PANTHER) |
| 1001 | lib_dib_Filter15_IDBA_contig_20007_plus1 | 36 | reelin | 112 | 1.21E-21 | 61.30% | 18 | P:regulation of biological process; P:cell-cell signaling; P:biological_process; P:multicellular organismal development; P:response to external stimulus; P:response to abiotic stimulus; P:cellular protein modification process; P:cellular component organization; P:behavior; P:signal transduction; C:extracellular space; C:cell; P:anatomical structure morphogenesis; P:cell differentiation; P:response to endogenous stimulus; C:cytoplasm; F:binding; P:response to stress | PTHR11841 (PANTHER), PTHR11841:SF0 (PANTHER) |
| 1002 | lib_dib_Filter15_IDBA_contig_20008_plus1 | 24 | isoform cra_b | 112 | 2.94E-20 | 58.35% | 16 | P:multicellular organismal development; F:receptor binding; F:catalytic activity; P:anatomical structure morphogenesis; P:cellular component organization; P:cell differentiation; P:signal transduction; P:behavior; C:extracellular space; P:metabolic process; P:response to stress; C:cytoplasm; ; P:regulation of biological process; P:cell-cell signaling; C:cell | IPR000742 (PROFILE); IPR013032 (PROSITE); PTHR11841 (PANTHER), PTHR11841:SF0 (PANTHER) |

| | | | | | | | | | |
|---|---|---|---|---|---|---|---|---|---|
| 1003 | lib_dib_Filter15_IDBA_contig_20009_plus1 | 45 | reelin | 112 | 1.41E-19 | 55.25% | 16 | P:multicellular organismal development; F:receptor binding; F:catalytic activity; P:anatomical structure morphogenesis; P:cellular component organization; P:cell differentiation; P:signal transduction; P:behavior; C:extracellular space; P:metabolic process; P:response to stress; C:cytoplasm; P:regulation of biological process; ; P:cell-cell signaling; C:cell | IPR000742 (PROFILE); IPR013032 (PROSITE); PTHR11841 (PANTHER), PTHR11841:SF0 (PANTHER) |
| 1004 | lib_dib_Filter15_IDBA_contig_20010_plus3 | 93 | reelin isoform 1 | 201 | 5.05E-53 | 60.50% | 4 | P:multicellular organismal development; P:signal transduction; P:regulation of biological process; P:cell differentiation | PTHR11841 (PANTHER), PTHR11841:SF0 (PANTHER) |
| 1005 | lib_dib_Filter15_IDBA_contig_20011_plus1 | 239 | reelin isoform 1 | 394 | 2.66E-113 | 62.35% | 10 | P:regulation of biological process; P:cell-cell signaling; P:biological_process; P:cellular protein modification process; P:multicellular organismal development; P:cellular component organization; P:signal transduction; P:anatomical structure morphogenesis; P:cell differentiation; F:binding | IPR013032 (PROSITE); G3DSA:2.170.300.10 (GENE3D), PTHR11841 (PANTHER), PTHR11841:SF0 (PANTHER), SSF57196 (SUPERFAMILY) |
| 1006 | lib_dib_Filter15_IDBA_contig_20012_plus1 | 112 | reelin isoform 1 | 204 | 1.41E-50 | 56.15% | 16 | P:multicellular organismal development; F:receptor binding; F:catalytic activity; P:anatomical structure morphogenesis; P:cellular component organization; P:cell differentiation; P:signal transduction; P:behavior; C:extracellular space; P:metabolic process; P:response to stress; C:cytoplasm; P:regulation of biological process; ; P:cell-cell signaling; C:cell | PTHR11841 (PANTHER), PTHR11841:SF0 (PANTHER) |
| 1007 | lib_dib_Filter15_IDBA_contig_20013_plus1 | 33 | reelin isoform 1 | 159 | 1.84E-44 | 68.30% | 18 | P:multicellular organismal development; F:receptor binding; P:anatomical structure morphogenesis; P:cellular component organization; P:cell differentiation; P:signal transduction; P:behavior; C:extracellular space; F:protein kinase activity; P:metabolic process; P:response to stress; C:cytoplasm; C:proteinaceous extracellular matrix; F:hydrolase activity; P:regulation of biological process; ; P:cell-cell signaling; C:cell | PTHR11841 (PANTHER), PTHR11841:SF0 (PANTHER) |
| 1008 | lib_dib_Filter15_IDBA_contig_20014_plus1 | 30 | reelin isoform 1 | 159 | 1.03E-43 | 68.40% | 18 | P:multicellular organismal development; F:receptor binding; P:anatomical structure morphogenesis; P:cellular component organization; P:cell differentiation; P:signal transduction; P:behavior; C:extracellular space; F:protein kinase activity; P:metabolic process; P:response to stress; C:cytoplasm; C:proteinaceous extracellular matrix; F:hydrolase activity; P:regulation of biological process; ; P:cell-cell signaling; C:cell | PTHR11841 (PANTHER), PTHR11841:SF0 (PANTHER) |

| | | | | | | | | | |
|---|---|---|---|---|---|---|---|---|---|
| 1009 | lib_dib_Filter15_IDBA_contig_20016_plus1 | 61 | reelin isoform 1 | 155 | 1.75E-45 | 63.60% | 17 | P:multicellular organismal development; F:receptor binding; F:catalytic activity; P:anatomical structure morphogenesis; P:cellular component organization; P:cell differentiation; P:signal transduction; P:behavior; C:extracellular space; P:metabolic process; P:response to stress; C:cytoplasm; C:proteinaceous extracellular matrix; P:regulation of biological process; ; P:cell-cell signaling; C:cell | IPR013032 (PROSITE); G3DSA:2.10.25.10 (GENE3D), PTHR11841 (PANTHER), PTHR11841:SF0 (PANTHER), SSF57196 (SUPERFAMILY) |
| 1010 | lib_dib_Filter15_IDBA_contig_20017_plus3 | 233 | reelin isoform 2 | 490 | 8.14E-102 | 57.30% | 15 | P:multicellular organismal development; F:receptor binding; P:anatomical structure morphogenesis; P:cellular component organization; P:cell differentiation; P:signal transduction; P:behavior; C:extracellular space; P:metabolic process; P:response to stress; C:cytoplasm; P:regulation of biological process; ; P:cell-cell signaling; C:cell | IPR011040 (G3DSA:2.120.10.GENE3D); PTHR11841 (PANTHER), PTHR11841:SF0 (PANTHER) |
| 1011 | lib_dib_Filter15_IDBA_contig_20037_plus2 | 145 | galactose soluble 8-like | 518 | 1.35E-37 | 44.25% | 1 | F:carbohydrate binding | IPR001079 (PFAM); IPR008985 (SUPERFAMILY); IPR013320 (G3DSA:2.60.120.GENE3D); PTHR11346 (PANTHER) |
| 1012 | lib_dib_Filter15_IDBA_contig_2004_plus1 | 8662 | type alpha partial | 370 | 9.77E-114 | 68.05% | 9 | P:anatomical structure morphogenesis; P:cellular component organization; P:cell differentiation; P:multicellular organismal development; P:biological_process; C:proteinaceous extracellular matrix; F:protein binding; P:embryo development; F:structural molecule activity | IPR000885 (PRODOM); PTHR24637 (PANTHER), PTHR24637:SF84 (PANTHER) |
| 1013 | lib_dib_Filter15_IDBA_contig_20051_plus1 | 82 | ankyrin unc44 | 518 | 7.40E-77 | 56.45% | 1 | F:protein binding | IPR002110 (PRINTS); IPR020683 (G3DSA:1.25.40.GENE3D); PTHR24198 (PANTHER), PF13637 (PFAM), PF13857 (PFAM) |
| 1014 | lib_dib_Filter15_IDBA_contig_20064_plus2 | 151 | threonine synthase-like 2 | 517 | 0 | 71.10% | 7 | F:binding; P:metabolic process; P:biosynthetic process; P:lipid metabolic process; P:catabolic process; ; F:catalytic activity | IPR000634 (PROSITE); IPR001926 (PFAM); IPR004450 (TIGRFAMs); G3DSA:3.40.50.1100 (GENE3D), G3DSA:3.90.1380.10 (GENE3D), PTHR10314 (PANTHER), PTHR10314:SF44 (PANTHER) |
| 1015 | lib_dib_Filter15_IDBA_contig_20097_plus2 | 265 | hyaluronidase- partial | 785 | 1.11E-69 | 49.75% | 5 | P:biological_process; P:regulation of biological process; C:cytoplasm; F:hydrolase activity; P:carbohydrate metabolic process | IPR013785 (G3DSA:3.20.20.GENE3D); IPR017853 (SUPERFAMILY); IPR018155 (PRINTS); G3DSA:2.10.25.10 (GENE3D), SignalP-NN(euk) (SIGNALP), tmhmm (TMHMM) |
| 1016 | lib_dib_Filter15_IDBA_contig_20109_plus1 | 578 | poly -specific endoribonuclease isoform 3 | 772 | 3.40E-54 | 54.85% | 4 | F:polysaccharide binding; F:scavenger receptor activity; F:hydrolase activity, acting on ester bonds; P:immune response | IPR018998 (PFAM); PTHR12439 (PANTHER), PTHR12439:SF6 (PANTHER), tmhmm (TMHMM), SSF142877 (SUPERFAMILY) |
| 1017 | lib_dib_Filter15_IDBA_contig_20118_plus2 | 128 | adamts-like protein 3-like | 541 | 7.26E-70 | 45.70% | 2 | F:protein binding; F:peptidase activity | IPR000884 (PFAM); IPR003598 (SMART); IPR003599 (SMART); IPR007110 (PROFILE); IPR010909 (PFAM); IPR013098 (PFAM); IPR013783 (G3DSA:2.60.40.GENE3D); G3DSA:2.20.100.10 (GENE3D), PTHR13723 (PANTHER), SSF48726 (SUPERFAMILY) |
| 1018 | lib_dib_Filter15_IDBA_contig_20133_plus2 | 65 | wd-40 repeat protein | 520 | 1.21E-117 | 48.65% | 2 | F:catalytic activity; F:protein binding | IPR001680 (PFAM); IPR015943 (G3DSA:2.130.10.GENE3D); IPR017986 (PROFILE); IPR019775 (PROSITE); PTHR22847 (PANTHER), PTHR22847:SF150 (PANTHER) |

| | | | | | | | | | | |
|---|---|---|---|---|---|---|---|---|---|---|
| 1019 | lib_dib_Filter15_IDBA_contig_20138_minus3 | 84 | epidermal growth factor receptor | 515 | 3.64E-73 | 61.60% | 20 | P:regulation of biological process; P:metabolic process; C:organelle; C:intracellular; P:cell proliferation; F:receptor activity; F:protein kinase activity; P:signal transduction; P:biological_process; C:plasma membrane; C:cell; P:primary metabolic process; F:hydrolase activity; F:protein binding; F:nucleotide binding; P:transport; P:multicellular organismal development; C:cytoplasm; C:nucleus; P:cellular protein modification process | IPR000719 (PROFILE); IPR001245 (PRINTS); IPR011009 (SUPERFAMILY); IPR020635 (SMART); G3DSA:1.10.510.10 (GENE3D), PTHR24416 (PANTHER), PTHR24416:SF90 (PANTHER) | |
| 1020 | lib_dib_Filter15_IDBA_contig_20144_minus1 | 4506 | coatomer subunit alpha isoform 1 | 1637 | 0 | 88.05% | 10 | P:transport; C:cytoplasmic membrane-bounded vesicle; C:Golgi apparatus; C:protein complex; C:cytosol; C:extracellular space; P:organelle organization; P:protein transport; F:structural molecule activity; F:receptor binding | IPR001680 (PFAM); IPR006692 (PFAM); IPR010714 (PFAM); IPR015943 (G3DSA:2.130.10.GENE3D); IPR017986 (PROFILE); IPR019775 (PROSITE); IPR020472 (PRINTS); PTHR19876 (PANTHER), PTHR19876:SF1 (PANTHER), SSF82171 (SUPERFAMILY) | |
| 1021 | lib_dib_Filter15_IDBA_contig_20209_plus1 | 81 | cg3402 cg3402-pa | 513 | 2.82E-66 | 72.25% | 1 | F:protein binding | IPR001478 (PFAM); G3DSA:2.30.42.10 (GENE3D), PTHR23136 (PANTHER), PTHR23136:SF1 (PANTHER), tmhmm (TMHMM) | |
| 1022 | lib_dib_Filter15_IDBA_contig_20241_minus1 | 308 | heat shock 70 kda protein 14 | 689 | 3.21E-150 | 63.00% | 1 | C:cytoplasm | IPR013126 (PRINTS); G3DSA:2.60.34.10 (GENE3D), G3DSA:3.30.30.30 (GENE3D), G3DSA:3.30.420.40 (GENE3D), G3DSA:3.90.640.10 (GENE3D), PTHR19375 (PANTHER), PTHR19375:SF87 (PANTHER), SignalP-NN(euk) (SIGNALP), SSF100920 (SUPERFAMILY), SSF53067 (SUPERFAMILY) | Yes |
| 1023 | lib_dib_Filter15_IDBA_contig_20257_plus2 | 123 | protein fam92b | 533 | 1.44E-40 | 43.05% | 3 | F:binding; F:nucleic acid binding; C:intracellular | IPR007087 (PFAM); IPR013087 (G3DSA:3.30.160.GENE3D); IPR015880 (SMART); PTHR11389 (PANTHER), PF13465 (PFAM), PF13894 (PFAM), SSF57667 (SUPERFAMILY) | |
| 1024 | lib_dib_Filter15_IDBA_contig_20300_minus3 | 33 | four-jointed box protein 1 | 153 | 2.85E-45 | 59.10% | 2 | P:signal transduction; C:extracellular space | IPR024868 (PRINTS) | |
| 1025 | lib_dib_Filter15_IDBA_contig_20307_minus3 | 145 | latrophilin 3-like | 541 | 1.33E-108 | 57.75% | 3 | P:signal transduction; F:receptor activity; C:cell | IPR000203 (PFAM); IPR000832 (PRINTS); IPR017981 (PROFILE); IPR017983 (PROSITE); PTHR12011 (PANTHER), tmhmm (TMHMM), SSF81321 (SUPERFAMILY) | |
| 1026 | lib_dib_Filter15_IDBA_contig_20314_plus1 | 381 | thyrotropin-releasing hormone-degrading ectoenzyme | 955 | 8.94E-84 | 45.45% | 4 | P:protein metabolic process; P:catabolic process; F:peptidase activity; F:binding | IPR001930 (PANTHER); IPR014782 (PRINTS); G3DSA:1.10.390.10 (GENE3D), SSF55486 (SUPERFAMILY), SSF63737 (SUPERFAMILY) | Yes |
| 1027 | lib_dib_Filter15_IDBA_contig_20318_minus1 | 405 | transport and golgi organization-like | 538 | 4.64E-150 | 56.25% | 1 | F:protein binding | IPR000210 (SMART); IPR011333 (G3DSA:3.30.710.GENE3D); IPR011705 (PFAM); IPR013069 (PFAM); PTHR24413 (PANTHER) | |
| 1028 | lib_dib_Filter15_IDBA_contig_20386_minus1 | 2663 | dipeptidyl peptidase 4 | 699 | 4.61E-81 | 57.55% | 11 | P:biological_process; F:peptidase activity; C:cell; C:Golgi apparatus; P:regulation of biological process; F:binding; P:protein metabolic process; P:catabolic process; F:protein binding; C:endoplasmic reticulum; C:plasma membrane | IPR001375 (PFAM); IPR002469 (PFAM); G3DSA:2.140.10.30 (GENE3D), G3DSA:3.40.50.1820 (GENE3D), PTHR11731 (PANTHER), PTHR11731:SF24 (PANTHER), SSF53474 (SUPERFAMILY) | |

| | | | | | | | | | |
|---|---|---|---|---|---|---|---|---|---|
| 1029 | lib_dib_Filter15_IDBA_contig_20393_minus2 | 2741 | low density lipoprotein | 1307 | 0 | 51.70% | 3 | F:protein binding; F:calcium ion binding; P:biological_process | IPR000033 (PFAM); IPR000152 (PROSITE); IPR000742 (SMART); IPR001881 (SMART); IPR003886 (PFAM); IPR006605 (PFAM); IPR009017 (SUPERFAMILY); IPR011042 (G3DSA:2.120.10.GENE3D); IPR013032 (PROSITE); IPR018097 (PROSITE); IPR023413 (G3DSA:2.40.155.GENE3D); IPR024731 (PFAM); G3DSA:2.10.25.10 (GENE3D), G3DSA:2.170.300.10 (GENE3D), PTHR10529 (PANTHER), PTHR10529:SF107 (PANTHER), SSF57196 (SUPERFAMILY), SSF63825 (SUPERFAMILY) |
| 1030 | lib_dib_Filter15_IDBA_contig_20434_plus2 | 92 | 2 -5 -oligoadenylate synthase 1 | 544 | 2.00E-175 | 46.15% | 4 | F:RNA binding; F:nucleotide binding; P:biological_process; F:transferase activity | IPR006116 (PROFILE); IPR018952 (G3DSA:1.10.1410.GENE3D); G3DSA:3.30.460.10 (GENE3D), SSF81301 (SUPERFAMILY), SSF81631 (SUPERFAMILY) |
| 1031 | lib_dib_Filter15_IDBA_contig_20457_plus2 | 253 | neuronal cell adhesion | 701 | 3.74E-59 | 47.80% | 1 | F:protein binding | IPR003598 (SMART); IPR003599 (SMART); IPR007110 (PROFILE); IPR013098 (PFAM); IPR013783 (G3DSA:2.60.40.GENE3D); IPR026966 (PFAM); PTHR10489 (PANTHER), PF13895 (PFAM), tmhmm (TMHMM), SSF48726 (SUPERFAMILY) |
| 1032 | lib_dib_Filter15_IDBA_contig_20470_minus2 | 1014 | plasminogen | 1220 | 4.50E-76 | 53.50% | 13 | P:anatomical structure morphogenesis; F:peptidase activity; P:regulation of biological process; P:embryo development; P:multicellular organismal development; P:cell death; C:plasma membrane; P:response to external stimulus; P:response to stress; P:cell differentiation; P:protein metabolic process; P:catabolic process; F:carbohydrate binding | IPR000001 (G3DSA:2.40.20.GENE3D); IPR001304 (PFAM); IPR013806 (SUPERFAMILY); IPR016186 (G3DSA:3.10.100.GENE3D); IPR016187 (SUPERFAMILY); IPR018056 (PROSITE); IPR018378 (PROSITE); PR00018 (PRINTS), PTHR24259 (PANTHER), PTHR24259:SF2 (PANTHER) |
| 1033 | lib_dib_Filter15_IDBA_contig_20471_minus2 | 1104 | macrophage mannose receptor 1-like | 1000 | 2.00E-47 | 40.65% | 1 | F:carbohydrate binding | IPR001304 (PFAM); IPR016186 (G3DSA:3.10.100.GENE3D); IPR016187 (SUPERFAMILY); IPR018378 (PROSITE); PTHR22803 (PANTHER) |
| 1034 | lib_dib_Filter15_IDBA_contig_20484_plus1 | 128 | beta- -galactosyl-o-glycosyl-glycoprotein beta- -n-acetylglucosaminyltransferase 3 | 541 | 2.78E-104 | 58.60% | 2 | F:transferase activity; C:cell | IPR003406 (PFAM); PTHR19297 (PANTHER), PTHR19297:SF6 (PANTHER), tmhmm (TMHMM) |
| 1035 | lib_dib_Filter15_IDBA_contig_20487_plus3 | 282 | sphingomyelin phosphodiesterase | 751 | 0 | 66.30% | 3 | F:hydrolase activity; P:catabolic process; P:lipid metabolic process | IPR004843 (PFAM); IPR008139 (SMART); IPR011001 (G3DSA:1.10.225.GENE3D); IPR011160 (PIR); G3DSA:3.60.21.10 (GENE3D), PTHR10340 (PANTHER), PTHR10340:SF13 (PANTHER), tmhmm (TMHMM), SSF56300 (SUPERFAMILY) |
| 1036 | lib_dib_Filter15_IDBA_contig_20488_plus1 | 297 | sphingomyelin phosphodiesterase | 754 | 0 | 66.35% | 3 | F:hydrolase activity; P:catabolic process; P:lipid metabolic process | IPR004843 (PFAM); IPR008139 (SMART); IPR011001 (G3DSA:1.10.225.GENE3D); IPR011160 (PIR); G3DSA:3.60.21.10 (GENE3D), PTHR10340 (PANTHER), PTHR10340:SF13 (PANTHER), SSF56300 (SUPERFAMILY) |
| 1037 | lib_dib_Filter15_IDBA_contig_20496_plus2 | 293 | vg1 protein | 504 | 3.26E-14 | 54.95% | 6 | P:regulation of biological process; P:anatomical structure morphogenesis; P:embryo development; P:multicellular organismal development; P:signal transduction; F:receptor binding | IPR001839 (PFAM); IPR015615 (PANTHER); G3DSA:2.10.90.10 (GENE3D), tmhmm (TMHMM), SSF57501 (SUPERFAMILY) |

| | | | | | | | | | |
|---|---|---|---|---|---|---|---|---|---|
| 1038 | lib_dib_Filter15_IDBA_contig_20548_minus2 | 400 | leucine-zipper-like transcriptional regulator 1-like | 957 | 0 | 81.40% | 1 | F:protein binding | IPR000210 (SMART); IPR006652 (SMART); IPR011333 (G3DSA:3.30.710.GENE3D); IPR013069 (PFAM); IPR015915 (G3DSA:2.120.10.GENE3D); PTHR23244 (PANTHER), PTHR23244:SF15 (PANTHER), PF13418 (PFAM), PF13854 (PFAM), PF13964 (PFAM), SSF117281 (SUPERFAMILY) |
| 1039 | lib_dib_Filter15_IDBA_contig_20549_minus2 | 204 | leucine-zipper-like transcriptional regulator 1-like | 502 | 0 | 83.60% | 1 | F:protein binding | IPR006652 (SMART); IPR011333 (G3DSA:3.30.710.GENE3D); IPR015915 (G3DSA:2.120.10.GENE3D); PTHR23244 (PANTHER), PTHR23244:SF15 (PANTHER), PF13418 (PFAM), PF13854 (PFAM), PF13964 (PFAM), SSF117281 (SUPERFAMILY) |
| 1040 | lib_dib_Filter15_IDBA_contig_20552_minus2 | 72 | multicopper oxidase | 502 | 2.73E-82 | 51.05% | 3 | F:catalytic activity; P:metabolic process; F:binding | IPR001117 (PFAM); IPR002355 (PROSITE); IPR008972 (G3DSA:2.60.40.GENE3D); IPR011706 (PFAM); PTHR11709 (PANTHER), PTHR11709:SF8 (PANTHER) |
| 1041 | lib_dib_Filter15_IDBA_contig_20580_minus1 | 9906 | transmembrane protease serine 2-like | 623 | 1.34E-11 | 56.95% | 3 | F:peptidase activity; P:protein metabolic process; P:catabolic process | IPR001254 (PFAM); IPR009003 (SUPERFAMILY); IPR018114 (PROSITE); G3DSA:2.40.10.10 (GENE3D), PTHR24265 (PANTHER) |
| 1042 | lib_dib_Filter15_IDBA_contig_20581_minus1 | 16966 | transmembrane protease serine 3 | 520 | 9.11E-45 | 52.85% | 5 | C:endoplasmic reticulum; C:cell; F:peptidase activity; P:protein metabolic process; P:catabolic process | IPR001254 (PFAM); IPR001314 (PRINTS); IPR009003 (SUPERFAMILY); IPR018114 (PROSITE); G3DSA:2.40.10.10 (GENE3D), PTHR24265 (PANTHER) |
| 1043 | lib_dib_Filter15_IDBA_contig_20582_minus1 | 25201 | transmembrane protease serine 3 | 834 | 1.71E-43 | 52.80% | 5 | C:endoplasmic reticulum; C:cell; F:peptidase activity; P:protein metabolic process; P:catabolic process | IPR001254 (PFAM); IPR001314 (PRINTS); IPR009003 (SUPERFAMILY); IPR018114 (PROSITE); G3DSA:2.40.10.10 (GENE3D), PTHR24265 (PANTHER), tmhmm (TMHMM) |
| 1044 | lib_dib_Filter15_IDBA_contig_20584_minus1 | 570 | transmembrane protease serine 3 | 118 | 1.74E-20 | 60.10% | 9 | P:cellular homeostasis; P:multicellular organismal development; F:molecular_function; C:cell; P:biological_process; C:endoplasmic reticulum; F:peptidase activity; P:protein metabolic process; P:catabolic process | IPR001254 (PFAM); IPR009003 (SUPERFAMILY); IPR018114 (PROSITE); G3DSA:2.40.10.10 (GENE3D), PTHR24256 (PANTHER) |
| 1045 | lib_dib_Filter15_IDBA_contig_20585_minus3 | 85 | transmembrane protease serine 9 | 113 | 2.45E-17 | 59.25% | 4 | P:multicellular organismal development; F:peptidase activity; P:protein metabolic process; P:catabolic process | IPR001254 (PFAM); IPR009003 (SUPERFAMILY); IPR018114 (PROSITE); G3DSA:2.40.10.10 (GENE3D), PTHR24256 (PANTHER) |
| 1046 | lib_dib_Filter15_IDBA_contig_20591_plus3 | 98 | transcriptional repressor ctcf | 501 | 3.44E-159 | 76.05% | 3 | F:binding; C:intracellular; F:DNA binding | IPR007087 (PROSITE); IPR013087 (G3DSA:3.30.160.GENE3D); IPR015880 (SMART); PTHR24375 (PANTHER), PTHR24375:SF1 (PANTHER), PF13465 (PFAM), SSF57667 (SUPERFAMILY) |
| 1047 | lib_dib_Filter15_IDBA_contig_20595_minus2 | 319 | cubilin-like | 543 | 2.80E-98 | 52.15% | 5 | F:hydrolase activity; C:extracellular space; F:molecular_function; P:biological_process; C:cellular_component | IPR000859 (G3DSA:2.60.120.GENE3D); PTHR10127 (PANTHER) |
| 1048 | lib_dib_Filter15_IDBA_contig_20596_minus2 | 787 | cubilin-like | 1096 | 3.06E-153 | 50.65% | 1 | F:protein binding | IPR000859 (G3DSA:2.60.120.GENE3D); IPR002172 (G3DSA:4.10.400.GENE3D); PTHR10127 (PANTHER), PTHR10127:SF345 (PANTHER), tmhmm (TMHMM) |
| 1049 | lib_dib_Filter15_IDBA_contig_20603_plus3 | 353 | fibrinogen c domain-containing protein 1-a-like | 681 | 3.12E-87 | 63.20% | 4 | F:receptor binding; C:extracellular space; P:signal transduction; F:calcium ion binding | IPR002181 (PFAM); IPR014715 (G3DSA:4.10.530.GENE3D); IPR014716 (G3DSA:3.90.215.GENE3D); IPR020837 (PROSITE); PTHR19143 (PANTHER) |
| 1050 | lib_dib_Filter15_IDBA_contig_20606_plus1 | 1931 | protein serine threonine | 962 | 1.84E-40 | 45.60% | 7 | F:protein binding; F:peptidase activity; P:protein metabolic process; P:catabolic process; P:biological_process; F:binding; C:cell | IPR000742 (SMART); IPR001577 (PANTHER); IPR006212 (SMART); IPR009030 (SUPERFAMILY); G3DSA:2.10.220.10 (GENE3D), PTHR10942:SF0 (PANTHER) |

| | | | | | | | | | | |
|---|---|---|---|---|---|---|---|---|---|---|
| 1051 | lib_dib_Filter15_IDBA_contig_20640_plus1 | 677 | growth differentiation factor 8-like | 667 | 1.56E-58 | 51.30% | 2 | F:receptor binding; P:growth | IPR001111 (PFAM); IPR001839 (PFAM); IPR015615 (PANTHER); IPR017948 (PROSITE); G3DSA:2.10.90.10 (GENE3D), SignalP-NN(euk) (SIGNALP), SSF57501 (SUPERFAMILY) | |
| 1052 | lib_dib_Filter15_IDBA_contig_20646_minus2 | 1634 | neuropeptide y prepropeptide | 726 | 2.83E-07 | 78.00% | 2 | F:receptor binding; C:extracellular region | IPR001955 (PFAM); IPR020392 (PROSITE); G3DSA:1.20.5.570 (GENE3D), tmhmm (TMHMM) | |
| 1053 | lib_dib_Filter15_IDBA_contig_20664_minus1 | 133 | 85 kda calcium-independent phospholipase a2 | 499 | 3.09E-122 | 53.30% | 1 | F:protein binding | IPR002110 (SMART); IPR020683 (G3DSA:1.25.40.GENE3D); PTHR24139 (PANTHER), PTHR24139:SF0 (PANTHER) | |
| 1054 | lib_dib_Filter15_IDBA_contig_20665_minus2 | 1843 | arylacetamide deacetylase-like 4-like | 831 | 1.46E-49 | 51.00% | 2 | P:metabolic process; F:hydrolase activity | IPR013094 (PFAM); G3DSA:3.40.50.1820 (GENE3D), PTHR23024 (PANTHER), SignalP-NN(euk) (SIGNALP), tmhmm (TMHMM), SSF53474 (SUPERFAMILY) | |
| 1055 | lib_dib_Filter15_IDBA_contig_20666_minus2 | 2605 | arylacetamide deacetylase-like 4-like | 1489 | 6.72E-48 | 50.95% | 2 | P:metabolic process; F:hydrolase activity | IPR013094 (PFAM); G3DSA:3.40.50.1820 (GENE3D), PTHR23024 (PANTHER), SignalP-NN(euk) (SIGNALP), tmhmm (TMHMM), SSF53474 (SUPERFAMILY) | |
| 1056 | lib_dib_Filter15_IDBA_contig_20691_plus2 | 5928 | fibrinogen-like protein 1 | 674 | 5.30E-31 | 51.45% | 5 | F:receptor binding; C:extracellular space; P:signal transduction; P:response to stilbenoid; C:fibrinogen complex | IPR002181 (PFAM); IPR014715 (G3DSA:4.10.530.GENE3D); IPR014716 (G3DSA:3.90.215.GENE3D); PTHR19143 (PANTHER), SignalP-NN(euk) (SIGNALP), tmhmm (TMHMM) | |
| 1057 | lib_dib_Filter15_IDBA_contig_20692_plus3 | 5673 | fibrinogen-like protein 1 | 663 | 8.30E-31 | 51.60% | 5 | F:receptor binding; C:extracellular space; P:signal transduction; P:response to stilbenoid; C:fibrinogen complex | IPR002181 (PFAM); IPR014715 (G3DSA:4.10.530.GENE3D); IPR014716 (G3DSA:3.90.215.GENE3D); PTHR19143 (PANTHER), SignalP-NN(euk) (SIGNALP), tmhmm (TMHMM) | Yes |
| 1058 | lib_dib_Filter15_IDBA_contig_20693_plus1 | 1687 | fibrinogen-like protein 1-like | 498 | 7.05E-20 | 53.10% | 4 | F:receptor binding; P:signal transduction; C:extracellular space; P:response to stilbenoid | IPR002181 (PFAM); IPR014715 (G3DSA:4.10.530.GENE3D); IPR014716 (G3DSA:3.90.215.GENE3D); PTHR19143 (PANTHER), SignalP-NN(euk) (SIGNALP) | |
| 1059 | lib_dib_Filter15_IDBA_contig_20694_plus1 | 351 | fibrinogen and fibronectin | 108 | 1.75E-11 | 56.05% | 4 | F:protein binding; P:biological_process; P:lipid metabolic process; P:regulation of biological process | IPR002181 (PFAM); IPR014716 (G3DSA:3.90.215.GENE3D); PTHR19143 (PANTHER), PTHR19143:SF21 (PANTHER) | |
| 1060 | lib_dib_Filter15_IDBA_contig_2073_plus1 | 350 | protein | 218 | 5.74E-20 | 39.41% | 8 | F:helicase activity; F:nucleic acid binding; F:ATP binding; F:receptor binding; F:hydrolase activity; F:nucleotide binding; P:signal transduction; F:ATP-dependent helicase activity | no IPS match | |
| 1061 | lib_dib_Filter15_IDBA_contig_2073_plus2 | 350 | protein | 218 | 2.70E-22 | 48.46% | 2 | C:cytoplasm; C:nucleus | SignalP-NN(euk) (SIGNALP) | |
| 1062 | lib_dib_Filter15_IDBA_contig_20740_plus1 | 1133 | subfamily member 7 | 890 | 1.09E-173 | 79.40% | 5 | C:cytoplasm; C:nucleolus; C:cytoskeleton; F:protein binding; P:protein metabolic process | IPR001440 (PFAM); IPR001623 (PRINTS); IPR011990 (G3DSA:1.25.40.GENE3D); IPR013026 (PROFILE); IPR013105 (PFAM); IPR019734 (PFAM); PTHR24078 (PANTHER), PTHR24078:SF0 (PANTHER), PF13414 (PFAM), SSF48452 (SUPERFAMILY) | |
| 1063 | lib_dib_Filter15_IDBA_contig_20744_plus1 | 81 | golgin subfamily a member 1 | 498 | 1.97E-50 | 55.12% | 3 | P:protein targeting to Golgi; F:molecular_function; C:intracellular | IPR009053 (SUPERFAMILY); PTHR23157 (PANTHER), PTHR23157:SF14 (PANTHER) | |
| 1064 | lib_dib_Filter15_IDBA_contig_20751_plus3 | 612 | fibrinogen c domain-containing protein 1-like | 840 | 2.99E-52 | 60.55% | 4 | F:receptor binding; C:extracellular space; P:signal transduction; P:response to stilbenoid | IPR002181 (PFAM); IPR014715 (G3DSA:4.10.530.GENE3D); IPR014716 (G3DSA:3.90.215.GENE3D); PTHR19143 (PANTHER) | |
| 1065 | lib_dib_Filter15_IDBA_contig_20753_plus2 | 398 | fibrinogen c domain-containing protein 1-like | 483 | 1.57E-13 | 58.65% | 2 | P:biological_process; F:carbohydrate binding | IPR002181 (PFAM); IPR014715 (G3DSA:4.10.530.GENE3D); PTHR19143 (PANTHER) | |

| | | | | | | | | | | |
|---|---|---|---|---|---|---|---|---|---|---|
| 1066 | lib_dib_Filter15_IDBA_contig_20756_minus1 | 142 | retinoblastoma binding protein 4 | 497 | 0 | 95.05% | 15 | P:cell cycle; C:nucleus; C:protein complex; F:hydrolase activity; F:protein binding; F:transferase activity; ; P:regulation of biological process; C:nucleoplasm; P:cell proliferation; C:nuclear chromosome; P:organelle organization; P:DNA metabolic process; P:biosynthetic process; P:cellular protein modification process | IPR001680 (PFAM); IPR015943 (G3DSA:2.130.10.GENE3D); IPR017986 (PROFILE); IPR019775 (PROSITE); IPR020472 (PRINTS); IPR022052 (PFAM); PTHR22850 (PANTHER), PTHR22850:SF60 (PANTHER) | |
| 1067 | lib_dib_Filter15_IDBA_contig_20774_plus2 | 812 | ectonucleoside triphosphate diphosphohydrolase 1 | 580 | 7.78E-119 | 50.15% | 2 | P:biological_process; F:hydrolase activity | IPR000407 (PANTHER); SignalP-NN(euk) (SIGNALP), tmhmm (TMHMM) | |
| 1068 | lib_dib_Filter15_IDBA_contig_2084_minus2 | 2816 | troponin t | 120 | 6.30E-23 | 66.33% | 2 | P:response to biotic stimulus; P:response to stress | no IPS match | |
| 1069 | lib_dib_Filter15_IDBA_contig_20840_plus3 | 506 | dipeptidyl peptidase 1-like | 495 | 2.32E-142 | 60.15% | 3 | P:protein metabolic process; P:catabolic process; F:peptidase activity | IPR000169 (PROSITE); IPR000668 (PRINTS); IPR013128 (PANTHER); IPR014882 (PFAM); IPR025660 (PROSITE); G3DSA:2.40.128.80 (GENE3D), G3DSA:3.90.70.10 (GENE3D), PTHR12411:SF17 (PANTHER), SignalP-NN(euk) (SIGNALP), SSF54001 (SUPERFAMILY), SSF75001 (SUPERFAMILY) | Yes |
| 1070 | lib_dib_Filter15_IDBA_contig_20853_plus1 | 111 | prolow-density lipoprotein receptor-related protein 1 | 525 | 8.75E-80 | 49.15% | 11 | P:signal transduction; F:protein binding; C:organelle; C:intracellular; P:transport; P:regulation of biological process; C:cell; P:cellular component organization; C:cytoplasm; P:metabolic process; P:biological_process | IPR000033 (PFAM); IPR000742 (PFAM); IPR011042 (G3DSA:2.120.10.GENE3D); IPR013032 (PROSITE); G3DSA:2.10.25.10 (GENE3D), PTHR10529 (PANTHER), PTHR10529:SF116 (PANTHER), SSF57196 (SUPERFAMILY), SSF63825 (SUPERFAMILY) | |
| 1071 | lib_dib_Filter15_IDBA_contig_20855_plus3 | 297 | focal adhesion kinase 1 | 793 | 6.81E-160 | 83.95% | 29 | F:signal transducer activity; P:cellular component organization; P:signal transduction; C:plasma membrane; P:anatomical structure morphogenesis; P:cell differentiation; P:regulation of biological process; P:multicellular organismal development; P:cell proliferation; P:cell-cell signaling; P:cellular protein modification process; P:embryo development; C:cell; C:cytosol; P:cell death; F:protein binding; P:response to external stimulus; P:response to stress; P:lipid metabolic process; P:cytoskeleton organization; P:biological_process; P:growth; F:nucleotide binding; P:response to endogenous stimulus; C:microtubule organizing center; F:protein kinase activity; C:nucleus; C:cytoplasm; P:metabolic process | IPR000719 (PROFILE); IPR001245 (PRINTS); IPR002290 (SMART); IPR005189 (PRODOM); IPR008266 (PROSITE); IPR011009 (SUPERFAMILY); IPR020635 (SMART); G3DSA:1.10.510.10 (GENE3D), G3DSA:1.20.120.330 (GENE3D), G3DSA:1.20.5.540 (GENE3D), G3DSA:3.30.200.20 (GENE3D), PTHR24418 (PANTHER), PTHR24418:SF78 (PANTHER) | |

| | | | | | | | | | | |
|---|---|---|---|---|---|---|---|---|---|---|
| 1072 | lib_dib_Filter15_IDBA_contig_20856_plus3 | 334 | focal adhesion kinase 1 | 912 | 0 | 77.70% | 33 | C:cytoskeleton; F:signal transducer activity; P:cellular component organization; P:signal transduction; C:plasma membrane; P:anatomical structure morphogenesis; P:cell differentiation; P:regulation of biological process; P:multicellular organismal development; F:protein binding; P:cell-cell signaling; P:cellular protein modification process; P:embryo development; C:cell; C:cytosol; F:receptor binding; P:cell death; P:response to external stimulus; P:response to stress; P:lipid metabolic process; C:nucleoplasm; P:cytoskeleton organization; P:biological_process; P:cell growth; P:growth; F:nucleotide binding; P:response to endogenous stimulus; P:cellular homeostasis; P:transport; P:cell proliferation; F:protein kinase activity; C:nucleolus; P:metabolic process | IPR000719 (PROFILE); IPR001245 (PRINTS); IPR002290 (SMART); IPR005189 (PRODOM); IPR008266 (PROSITE); IPR011009 (SUPERFAMILY); IPR017441 (PROSITE); IPR020635 (SMART); G3DSA:1.10.510.10 (GENE3D), G3DSA:1.20.120.330 (GENE3D), G3DSA:1.20.5.540 (GENE3D), G3DSA:3.30.200.20 (GENE3D), PTHR24418 (PANTHER), PTHR24418:SF78 (PANTHER), SSF50729 (SUPERFAMILY) | |
| 1073 | lib_dib_Filter15_IDBA_contig_20868_plus1 | 110 | maltase 1-like | 495 | 4.13E-105 | 57.95% | 3 | F:catalytic activity; P:carbohydrate metabolic process; F:binding | IPR006047 (PFAM); IPR006589 (SMART); IPR013781 (G3DSA:3.20.20.GENE3D); IPR015902 (PANTHER); IPR017853 (SUPERFAMILY); G3DSA:3.90.400.10 (GENE3D), PTHR10357:SF11 (PANTHER), tmhmm (TMHMM) | |
| 1074 | lib_dib_Filter15_IDBA_contig_20942_minus2 | 243 | sortilin-related receptor | 905 | 0 | 55.15% | 1 | F:protein binding | IPR000033 (PFAM); IPR002172 (G3DSA:4.10.400.GENE3D); IPR011042 (G3DSA:2.120.10.GENE3D); IPR023415 (PROSITE); PR00261 (PRINTS), PTHR12106 (PANTHER), PTHR12106:SF7 (PANTHER), SSF110296 (SUPERFAMILY), SSF63825 (SUPERFAMILY) | |
| 1075 | lib_dib_Filter15_IDBA_contig_20943_minus3 | 19 | sortilin-related receptor | 149 | 1.10E-48 | 61.65% | 1 | F:protein binding | IPR002172 (G3DSA:4.10.400.GENE3D); IPR023415 (PROSITE); PR00261 (PRINTS), PTHR10529 (PANTHER) | |
| 1076 | lib_dib_Filter15_IDBA_contig_20952_plus3 | 116 | neuronal acetylcholine receptor subunit beta-3 | 622 | 1.12E-93 | 52.50% | 4 | C:cell; P:cell communication; F:ion channel activity; P:ion transport | IPR002394 (PRINTS); IPR006029 (PFAM); IPR006201 (PRINTS); IPR006202 (G3DSA:2.70.170.GENE3D); IPR018000 (PROSITE); G3DSA:1.20.120.370 (GENE3D), PTHR18945:SF92 (PANTHER), SignalP-NN(euk) (SIGNALP), tmhmm (TMHMM) | Yes |
| 1077 | lib_dib_Filter15_IDBA_contig_20983_plus3 | 98 | tripartite motif-containing protein 3 | 502 | 1.71E-83 | 55.80% | 1 | F:protein binding | IPR001258 (PFAM); IPR011042 (G3DSA:2.120.10.GENE3D); IPR013017 (PROFILE); IPR013783 (G3DSA:2.60.40.GENE3D); IPR014756 (SUPERFAMILY); PTHR24103 (PANTHER), PTHR24103:SF2 (PANTHER), SSF101898 (SUPERFAMILY) | |
| 1078 | lib_dib_Filter15_IDBA_contig_20994_minus3 | 238 | carboxypeptidase n subunit 2 precursor | 515 | 6.76E-39 | 50.10% | 1 | F:protein binding | IPR001611 (PFAM); IPR003591 (SMART); G3DSA:3.80.10.10 (GENE3D), PTHR24365 (PANTHER), PF13855 (PFAM), SM00364 (SMART), SM00365 (SMART), SSF52058 (SUPERFAMILY) | |
| 1079 | lib_dib_Filter15_IDBA_contig_20995_minus3 | 680 | carboxypeptidase n subunit 2 precursor | 1167 | 7.99E-38 | 50.10% | 1 | F:protein binding | IPR001611 (PFAM); IPR003591 (SMART); G3DSA:3.80.10.10 (GENE3D), PTHR24365 (PANTHER), PF13855 (PFAM), SM00364 (SMART), SM00365 (SMART), tmhmm (TMHMM), SSF52058 (SUPERFAMILY) | |

| | | | | | | | | | |
|---|---|---|---|---|---|---|---|---|---|
| 1080 | lib_dib_Filter15_IDBA_contig_20996_minus1 | 153 | tyrosine-protein kinase src42a | 490 | 2.90E-47 | 50.70% | 1 | F:protein binding | IPR001611 (PFAM); IPR003591 (SMART); G3DSA:3.80.10.10 (GENE3D), PTHR24365 (PANTHER), PF13516 (PFAM), PF13855 (PFAM), tmhmm (TMHMM), SSF52047 (SUPERFAMILY) |
| 1081 | lib_dib_Filter15_IDBA_contig_21002_minus1 | 523 | leucine-rich repeat and immunoglobulin-like domain-containing nogo receptor-interacting protein 3-like | 755 | 2.37E-116 | 43.45% | 1 | F:protein binding | IPR000372 (SMART); IPR000483 (SMART); IPR001611 (PFAM); IPR003591 (SMART); IPR003598 (SMART); IPR003599 (SMART); IPR007110 (PROFILE); IPR013783 (G3DSA:2.60.40.GENE3D); IPR026906 (PFAM); G3DSA:3.80.10.10 (GENE3D), PTHR24365 (PANTHER), PF13855 (PFAM), PF13895 (PFAM), tmhmm (TMHMM), SSF48726 (SUPERFAMILY), SSF52058 (SUPERFAMILY) |
| 1082 | lib_dib_Filter15_IDBA_contig_21013_plus2 | 154 | wd repeat-containing protein 37 | 596 | 0 | 70.70% | 1 | F:protein binding | IPR001680 (PFAM); IPR015943 (G3DSA:2.130.10.GENE3D); IPR017986 (PROFILE); IPR019775 (PROSITE); IPR020472 (PRINTS); PTHR19855 (PANTHER), PTHR19855:SF12 (PANTHER) |
| 1083 | lib_dib_Filter15_IDBA_contig_21016_minus1 | 1075 | ---NA--- | 1483 | | | 0 | - | tmhmm (TMHMM) |
| 1084 | lib_dib_Filter15_IDBA_contig_21016_minus2 | 1075 | hypothetical protein BRAFLDRAFT_70623 | 1483 | 8.73E-05 | 44.00% | 0 | - | tmhmm (TMHMM) |
| 1085 | lib_dib_Filter15_IDBA_contig_21027_plus1 | 244 | glycosaminoglycan xylosylkinase | 768 | 1.56E-135 | 53.25% | 3 | F:transferase activity; P:metabolic process; C:Golgi apparatus | IPR009581 (PFAM); IPR024869 (PANTHER); PTHR12450:SF7 (PANTHER) |
| 1086 | lib_dib_Filter15_IDBA_contig_21028_plus1 | 135 | glycosaminoglycan xylosylkinase | 335 | 5.43E-117 | 52.75% | 1 | P:metabolic process | IPR009581 (PFAM); IPR024869 (PANTHER); PTHR12450:SF7 (PANTHER) |
| 1087 | lib_dib_Filter15_IDBA_contig_21029_plus2 | 86 | dentin matrix protein 4-like | 226 | 4.28E-55 | 55.65% | 0 | - | IPR009581 (PFAM); IPR024869 (PANTHER); PTHR12450:SF8 (PANTHER) |
| 1088 | lib_dib_Filter15_IDBA_contig_21037_minus3 | 169 | gamma-glutamyltransferase isoform cra_b | 650 | 3.43E-148 | 64.15% | 11 | P:metabolic process; P:regulation of biological process; P:biosynthetic process; F:transferase activity; C:extracellular space; P:response to stress; ; P:biological_process; P:response to endogenous stimulus; P:response to biotic stimulus; C:plasma membrane | IPR000101 (PRINTS); SSF56235 (SUPERFAMILY) |
| 1089 | lib_dib_Filter15_IDBA_contig_21038_minus3 | 251 | gamma-glutamyltransferase isoform cra_b | 729 | 5.71E-145 | 64.20% | 11 | P:metabolic process; P:regulation of biological process; P:biosynthetic process; F:transferase activity; C:extracellular space; P:response to stress; ; P:biological_process; P:response to endogenous stimulus; P:response to biotic stimulus; C:plasma membrane | IPR000101 (PRINTS); SSF56235 (SUPERFAMILY) |
| 1090 | lib_dib_Filter15_IDBA_contig_21042_plus1 | 11033 | protein-glutamine gamma-glutamyltransferase k-like | 766 | 3.37E-167 | 60.40% | 2 | F:transferase activity; P:cellular protein modification process | IPR002931 (PFAM); IPR008958 (PFAM); IPR013783 (G3DSA:2.60.40.GENE3D); IPR023608 (PANTHER); G3DSA:3.90.260.10 (GENE3D), SSF54001 (SUPERFAMILY) |
| 1091 | lib_dib_Filter15_IDBA_contig_21065_minus2 | 1139 | tyrosine--trna cytoplasmic | 583 | 0 | 80.25% | 8 | C:cytoplasm; P:nucleobase-containing compound metabolic process; P:translation; ; F:catalytic activity; F:nucleotide binding; C:nucleus; F:RNA binding | IPR002305 (PFAM); IPR002307 (PRINTS); IPR002547 (PFAM); IPR012340 (G3DSA:2.40.50.GENE3D); IPR014729 (G3DSA:3.40.50.GENE3D); IPR023617 (PTHR11946:PANTHER); G3DSA:1.10.240.10 (GENE3D), PTHR11946 (PANTHER), SSF52374 (SUPERFAMILY) |

| | | | | | | | | | | |
|---|---|---|---|---|---|---|---|---|---|---|
| 1092 | lib_dib_Filter15_IDBA_contig_21066_minus3 | 1967 | cartilage intermediate layer protein 2 | 1359 | 1.13E-77 | 40.70% | 1 | F:protein binding | IPR000884 (PFAM); IPR003598 (SMART); IPR003599 (SMART); IPR007110 (PROFILE); IPR013783 (G3DSA:2.60.40.GENE3D); G3DSA:2.20.100.10 (GENE3D), PTHR15031 (PANTHER), PF13895 (PFAM), tmhmm (TMHMM), SSF48726 (SUPERFAMILY) | |
| 1093 | lib_dib_Filter15_IDBA_contig_21067_minus3 | 646 | cartilage intermediate layer protein 2 | 844 | 2.14E-78 | 44.50% | 1 | F:protein binding | IPR000884 (PFAM); IPR003598 (SMART); IPR003599 (SMART); IPR007110 (PROFILE); IPR013783 (G3DSA:2.60.40.GENE3D); PR01705 (PRINTS), G3DSA:2.20.100.10 (GENE3D), PTHR15031 (PANTHER), PF13895 (PFAM), tmhmm (TMHMM), SSF48726 (SUPERFAMILY) | |
| 1094 | lib_dib_Filter15_IDBA_contig_21071_plus2 | 276 | ankyrin unc44 | 849 | 1.03E-97 | 48.60% | 1 | F:protein binding | IPR002110 (PRINTS); IPR020683 (G3DSA:1.25.40.GENE3D); PTHR24121 (PANTHER) | |
| 1095 | lib_dib_Filter15_IDBA_contig_21082_minus2 | 7373 | neuregulin 1 variant iv-beta 1a | 1462 | 6.90E-10 | 46.45% | 8 | F:receptor binding; P:multicellular organismal development; P:regulation of biological process; C:plasma membrane; C:cell; P:signal transduction; P:cell differentiation; P:metabolic process | IPR000742 (PROFILE); IPR003599 (SMART); IPR007110 (PROFILE); IPR013032 (PROSITE); IPR013098 (PFAM); IPR013783 (G3DSA:2.60.40.GENE3D); G3DSA:2.10.25.10 (GENE3D), PTHR11100 (PANTHER), PTHR11100:SF7 (PANTHER), tmhmm (TMHMM), SSF48726 (SUPERFAMILY), SSF57196 (SUPERFAMILY) | |
| 1096 | lib_dib_Filter15_IDBA_contig_21083_minus2 | 1801 | neuregulin 1 variant iv-beta 1a | 320 | 3.95E-11 | 46.10% | 9 | F:receptor binding; P:biological_process; P:multicellular organismal development; P:regulation of biological process; C:plasma membrane; C:cell; P:signal transduction; P:cell differentiation; P:metabolic process | IPR000742 (PROFILE); IPR003599 (SMART); IPR007110 (PROFILE); IPR013032 (PROSITE); IPR013098 (PFAM); IPR013783 (G3DSA:2.60.40.GENE3D); G3DSA:2.10.25.10 (GENE3D), PTHR11100 (PANTHER), PTHR11100:SF7 (PANTHER), tmhmm (TMHMM), SSF48726 (SUPERFAMILY), SSF57196 (SUPERFAMILY) | |
| 1097 | lib_dib_Filter15_IDBA_contig_21092_minus1 | 346 | neuregulin 1 variant iv-beta 1a | 184 | 3.72E-12 | 46.20% | 9 | F:receptor binding; P:biological_process; P:multicellular organismal development; P:regulation of biological process; C:plasma membrane; C:cell; P:signal transduction; P:cell differentiation; P:metabolic process | IPR000742 (PROFILE); IPR003598 (SMART); IPR003599 (SMART); IPR007110 (PROFILE); IPR013032 (PROSITE); IPR013098 (PFAM); IPR013783 (G3DSA:2.60.40.GENE3D); G3DSA:2.10.25.10 (GENE3D), PTHR11100 (PANTHER), SignalP-NN(euk) (SIGNALP), SSF48726 (SUPERFAMILY), SSF57196 (SUPERFAMILY) | Yes |
| 1098 | lib_dib_Filter15_IDBA_contig_21099_minus3 | 446 | fibrinogen c domain-containing protein 1-like | 666 | 8.97E-54 | 59.65% | 3 | F:receptor binding; C:extracellular space; P:signal transduction | IPR002181 (PFAM); IPR014715 (G3DSA:4.10.530.GENE3D); IPR014716 (G3DSA:3.90.215.GENE3D); IPR020837 (PROSITE); PTHR19143 (PANTHER), SignalP-NN(euk) (SIGNALP), tmhmm (TMHMM) | |
| 1099 | lib_dib_Filter15_IDBA_contig_21100_minus3 | 431 | fibrinogen c domain-containing protein 1-like | 630 | 9.11E-54 | 59.90% | 3 | F:receptor binding; C:extracellular space; P:signal transduction | IPR002181 (PFAM); IPR014715 (G3DSA:4.10.530.GENE3D); IPR014716 (G3DSA:3.90.215.GENE3D); IPR020837 (PROSITE); PTHR19143 (PANTHER), SignalP-NN(euk) (SIGNALP), tmhmm (TMHMM) | |
| 1100 | lib_dib_Filter15_IDBA_contig_21101_minus3 | 172 | fibrinogen c domain-containing protein 1-like | 362 | 8.21E-62 | 58.70% | 3 | F:receptor binding; C:extracellular space; P:signal transduction | IPR002181 (PFAM); IPR014715 (G3DSA:4.10.530.GENE3D); IPR014716 (G3DSA:3.90.215.GENE3D); IPR020837 (PROSITE); PTHR19143 (PANTHER), SignalP-NN(euk) (SIGNALP), tmhmm (TMHMM) | |

| | | | | | | | | | | |
|---|---|---|---|---|---|---|---|---|---|---|
| 1101 | lib_dib_Filter15_IDBA_contig_21102_minus3 | 188 | fibrinogen c domain-containing protein 1-like | 337 | 2.30E-53 | 60.65% | 3 | F:receptor binding; C:extracellular space; P:signal transduction | IPR002181 (PFAM); IPR014715 (G3DSA:4.10.530.GENE3D); IPR014716 (G3DSA:3.90.215.GENE3D); IPR020837 (PROSITE); PTHR19143 (PANTHER), SignalP-NN(euk) (SIGNALP), tmhmm (TMHMM) | |
| 1102 | lib_dib_Filter15_IDBA_contig_21103_minus3 | 158 | fibrinogen c domain-containing protein 1-like | 403 | 3.66E-52 | 59.65% | 3 | F:receptor binding; C:extracellular space; P:signal transduction | IPR002181 (PFAM); IPR014715 (G3DSA:4.10.530.GENE3D); IPR014716 (G3DSA:3.90.215.GENE3D); IPR020837 (PROSITE); PTHR19143 (PANTHER), SignalP-NN(euk) (SIGNALP), tmhmm (TMHMM) | |
| 1103 | lib_dib_Filter15_IDBA_contig_21104_minus1 | 254 | fibrinogen c domain-containing protein 1-like | 488 | 3.42E-53 | 60.05% | 3 | F:receptor binding; C:extracellular space; P:signal transduction | IPR002181 (PFAM); IPR014715 (G3DSA:4.10.530.GENE3D); IPR014716 (G3DSA:3.90.215.GENE3D); IPR020837 (PROSITE); PTHR19143 (PANTHER), SignalP-NN(euk) (SIGNALP), tmhmm (TMHMM) | Yes |
| 1104 | lib_dib_Filter15_IDBA_contig_21105_minus1 | 14 | fibrinogen-related protein | 121 | 3.81E-21 | 57.80% | 3 | F:receptor binding; C:extracellular space; P:signal transduction | IPR002181 (PFAM); IPR014715 (G3DSA:4.10.530.GENE3D); IPR014716 (G3DSA:3.90.215.GENE3D); PTHR19143 (PANTHER) | |
| 1105 | lib_dib_Filter15_IDBA_contig_21106_minus2 | 50 | fibrinogen c domain-containing protein 1-like | 241 | 6.63E-47 | 64.50% | 3 | F:receptor binding; C:extracellular space; P:signal transduction | IPR002181 (PFAM); IPR014715 (G3DSA:4.10.530.GENE3D); IPR014716 (G3DSA:3.90.215.GENE3D); IPR020837 (PROSITE); PTHR19143 (PANTHER) | |
| 1106 | lib_dib_Filter15_IDBA_contig_21107_minus3 | 84 | angiopoietin-like 4 | 191 | 1.39E-14 | 56.60% | 9 | P:regulation of biological process; P:cell death; C:extracellular space; P:lipid metabolic process; P:response to stress; P:biological_process; P:cellular component organization; F:binding; F:enzyme regulator activity | IPR002181 (PFAM); IPR014716 (G3DSA:3.90.215.GENE3D); PTHR19143 (PANTHER), SignalP-NN(euk) (SIGNALP) | Yes |
| 1107 | lib_dib_Filter15_IDBA_contig_21108_minus3 | 80 | angiopoietin-related protein 3 | 213 | 2.80E-10 | 56.75% | 11 | F:receptor binding; P:anatomical structure morphogenesis; P:multicellular organismal development; P:regulation of biological process; P:biological_process; P:lipid metabolic process; C:extracellular space; C:cytoplasmic membrane-bounded vesicle; F:binding; C:plasma membrane; F:enzyme regulator activity | IPR002181 (PFAM); IPR014716 (G3DSA:3.90.215.GENE3D); PTHR19143 (PANTHER), PTHR19143:SF18 (PANTHER), SignalP-NN(euk) (SIGNALP) | |
| 1108 | lib_dib_Filter15_IDBA_contig_21110_minus1 | 946 | sel-1 suppressor of lin-12-like ( elegans) | 971 | 0 | 80.25% | 5 | C:endoplasmic reticulum; P:signal transduction; P:response to stress; F:protein binding; C:cell | IPR006597 (PFAM); IPR011990 (G3DSA:1.25.40.GENE3D); PTHR11102 (PANTHER), PTHR11102:SF26 (PANTHER), tmhmm (TMHMM), SSF81901 (SUPERFAMILY) | |
| 1109 | lib_dib_Filter15_IDBA_contig_21111_minus1 | 1081 | protein sel-1 homolog 1 | 1220 | 0 | 80.20% | 5 | C:endoplasmic reticulum; P:signal transduction; P:response to stress; C:cell; F:protein binding | IPR006597 (PFAM); IPR011990 (G3DSA:1.25.40.GENE3D); PTHR11102 (PANTHER), PTHR11102:SF26 (PANTHER), tmhmm (TMHMM), SSF81901 (SUPERFAMILY) | |
| 1110 | lib_dib_Filter15_IDBA_contig_21116_plus1 | 115 | protein toll | 487 | 4.43E-35 | 46.45% | 2 | F:protein binding; P:signal transduction | IPR000157 (PFAM); IPR000372 (SMART); IPR000483 (SMART); G3DSA:3.40.50.10140 (GENE3D), G3DSA:3.80.10.10 (GENE3D), PTHR24365 (PANTHER), tmhmm (TMHMM), SSF52058 (SUPERFAMILY) | |
| 1111 | lib_dib_Filter15_IDBA_contig_21130_minus1 | 183 | deoxyribonuclease lysosomal | 487 | 3.16E-103 | 56.85% | 2 | P:DNA metabolic process; F:nuclease activity | IPR004947 (PANTHER); SignalP-NN(euk) (SIGNALP), tmhmm (TMHMM) | Yes |
| 1112 | lib_dib_Filter15_IDBA_contig_21135_plus3 | 169 | tata element modulatory factor | 860 | 5.46E-167 | 70.00% | 4 | C:Golgi apparatus; F:transcription regulator activity; F:protein binding; | IPR022091 (PFAM); IPR022092 (PFAM); PTHR31724 (PANTHER) | |
| 1113 | lib_dib_Filter15_IDBA_contig_21136_plus1 | 201 | tata element modulatory factor | 1049 | 3.33E-166 | 70.00% | 4 | C:Golgi apparatus; F:transcription regulator activity; F:protein binding; | IPR022091 (PFAM); IPR022092 (PFAM); PTHR31724 (PANTHER) | |

| | | | | | | | | | | |
|---|---|---|---|---|---|---|---|---|---|---|
| 1114 | lib_dib_Filter15_IDBA_contig_21177_plus3 | 2623 | alpha-amylase | 854 | 0 | 62.70% | 3 | F:hydrolase activity; P:carbohydrate metabolic process; F:binding | IPR006046 (PRINTS); IPR006047 (PFAM); IPR006048 (SMART); IPR006589 (SMART); IPR013780 (G3DSA:2.60.40.GENE3D); IPR013781 (G3DSA:3.20.20.GENE3D); IPR015902 (PANTHER); IPR017853 (SUPERFAMILY); PTHR10357:SF26 (PANTHER), SignalP-NN(euk) (SIGNALP), SSF51011 (SUPERFAMILY) | |
| 1115 | lib_dib_Filter15_IDBA_contig_21182_plus1 | 993 | nuclease mitochondrial | 569 | 7.46E-103 | 66.00% | 3 | F:hydrolase activity; F:nucleic acid binding; F:binding | IPR001604 (PFAM); IPR020821 (G3DSA:3.40.570.GENE3D); PTHR13966 (PANTHER), SignalP-NN(euk) (SIGNALP), tmhmm (TMHMM), SSF54060 (SUPERFAMILY) | |
| 1116 | lib_dib_Filter15_IDBA_contig_21184_plus3 | 268 | beta-( )-xylosyltransferase | 763 | 7.52E-69 | 47.20% | 8 | F:transferase activity, transferring glycosyl groups; F:transferase activity; P:protein N-linked glycosylation; F:xylosyltransferase activity; C:Golgi medial cisterna; P:posttranslational protein targeting to membrane, translocation; C:mitochondrion; F:glycoprotein 2-beta-D-xylosyltransferase activity | IPR007657 (PFAM); PTHR20961 (PANTHER), PTHR20961:SF0 (PANTHER), tmhmm (TMHMM) | |
| 1117 | lib_dib_Filter15_IDBA_contig_21192_plus3 | 173 | sulfhydryl oxidase 1-like | 638 | 2.73E-99 | 49.50% | 5 | C:cell; F:catalytic activity; P:metabolic process; P:cellular homeostasis; P:regulation of biological process | IPR006863 (G3DSA:1.20.120.GENE3D); IPR012336 (G3DSA:3.40.30.GENE3D); IPR013766 (PFAM); IPR017905 (PROFILE); IPR017937 (PROSITE); PTHR22897 (PANTHER), tmhmm (TMHMM) | |
| 1118 | lib_dib_Filter15_IDBA_contig_21254_minus2 | 308 | deleted in malignant brain tumors 1 | 484 | 4.46E-64 | 50.30% | 3 | F:catalytic activity; F:receptor activity; C:cell | IPR001190 (PRINTS); IPR017448 (SMART); G3DSA:3.10.250.10 (GENE3D), PTHR19331 (PANTHER), PTHR19331:SF118 (PANTHER), SignalP-NN(euk) (SIGNALP) | Yes |
| 1119 | lib_dib_Filter15_IDBA_contig_21264_plus3 | 518 | protein dpy- isoform b | 912 | 3.86E-132 | 56.10% | 7 | P:embryo development; P:multicellular organismal development; C:cell; F:peptidase activity; P:protein metabolic process; P:catabolic process; F:binding | IPR000859 (G3DSA:2.60.120.GENE3D); IPR000998 (PFAM); IPR001506 (PRINTS); IPR006026 (SMART); IPR008985 (SUPERFAMILY); IPR013032 (PROSITE); IPR024079 (G3DSA:3.40.390.GENE3D); PTHR10127 (PANTHER), PTHR10127:SF334 (PANTHER), tmhmm (TMHMM), SSF55486 (SUPERFAMILY) | |
| 1120 | lib_dib_Filter15_IDBA_contig_21267_minus2 | 104 | fucolectin-4 precursor | 483 | 1.19E-22 | 53.70% | 6 | C:extracellular space; P:regulation of biological process; P:response to stress; F:calcium ion binding; F:carbohydrate binding; P:biological  process | IPR000421 (PFAM); IPR006585 (SMART); IPR008979 (SUPERFAMILY); G3DSA:2.60.120.260 (GENE3D), PTHR19277 (PANTHER), PTHR19277:SF6 (PANTHER) | |
| 1121 | lib_dib_Filter15_IDBA_contig_21276_minus1 | 435 | aspartyl-trna synthetase | 647 | 0 | 84.25% | 9 | C:cytoplasm; C:intracellular; C:protein complex; F:nucleic acid binding; P:nucleobase-containing compound metabolic process; P:translation; ; F:nucleotide binding; F:catalytic activity | IPR002312 (PRINTS); IPR004364 (PFAM); IPR004365 (PFAM); IPR004523 (PTHR22594:PANTHER); IPR006195 (PROFILE); IPR012340 (G3DSA:2.40.50.GENE3D); IPR018150 (PANTHER); G3DSA:3.30.930.10 (GENE3D), SSF55681 (SUPERFAMILY) | |

| | | | | | | | | | |
|---|---|---|---|---|---|---|---|---|---|
| 1122 | lib_dib_Filter15_IDBA_contig_2129_plus2 | 221 | chaperone protein | 390 | 6.76E-24 | 81.05% | 34 | P:regulation of biological process; P:cell death; P:DNA metabolic process; P:mitochondrion organization; P:biosynthetic process; C:plasma membrane; F:protein binding; C:cytosol; C:protein complex; C:endoplasmic reticulum; P:cellular component organization; P:signal transduction; P:cell differentiation; P:metabolic process; P:multicellular organismal development; ; P:embryo development; F:receptor binding; C:cellular_component; C:cytoskeleton; P:cellular protein modification process; P:biological_process; C:mitochondrion; P:nucleobase-containing compound metabolic process; P:response to stress; P:response to abiotic stimulus; F:enzyme regulator activity; C:intracellular; F:binding; F:nucleotide binding; C:cell; P:cell proliferation; C:nucleus; P:protein metabolic | IPR001623 (PRINTS); IPR018253 (PROSITE); PTHR24077 (PANTHER), SignalP-NN(euk) (SIGNALP), tmhmm (TMHMM) |
| 1123 | lib_dib_Filter15_IDBA_contig_21356_minus2 | 62 | inversin | 481 | 2.79E-137 | 79.15% | 10 | F:protein binding; C:cytoskeleton; C:protein complex; P:embryo development; P:multicellular organismal development; C:cilium; P:signal transduction; C:cytoplasm; C:cell; C:nucleus | IPR000048 (PROFILE); IPR002110 (PFAM); IPR020683 (G3DSA:1.25.40.GENE3D); PTHR24178 (PANTHER), PTHR24178:SF0 (PANTHER) |
| 1124 | lib_dib_Filter15_IDBA_contig_21368_minus3 | 92 | mannosyl-oligosaccharide -alpha-mannosidase ib | 480 | 0 | 80.95% | 7 | P:multicellular organismal development; C:Golgi apparatus; C:nucleolus; P:biological_process; P:metabolic process; F:hydrolase activity; F:calcium ion binding | IPR001382 (PRINTS); PTHR11742:SF6 (PANTHER) |
| 1125 | lib_dib_Filter15_IDBA_contig_21375_minus3 | 524 | phosphorylase gamma 1 | 894 | 0 | 73.40% | 13 | P:carbohydrate metabolic process; P:catabolic process; C:cytosol; P:cellular protein modification process; P:generation of precursor metabolites and energy; P:anatomical structure morphogenesis; P:embryo development; F:protein kinase activity; C:protein complex; C:cytoplasm; F:nucleotide binding; F:protein binding; P:biosynthetic process | IPR000719 (PFAM); IPR002290 (SMART); IPR002291 (PRINTS); IPR008271 (PROSITE); IPR011009 (SUPERFAMILY); IPR017441 (PROSITE); IPR020635 (SMART); IPR020636 (PANTHER); G3DSA:1.10.510.10 (GENE3D), G3DSA:3.30.200.20 (GENE3D), PTHR24347:SF48 (PANTHER), tmhmm (TMHMM) |
| 1126 | lib_dib_Filter15_IDBA_contig_21376_minus3 | 616 | phosphorylase b kinase gamma catalytic skeletal muscle isoform | 828 | 0 | 73.05% | 13 | P:carbohydrate metabolic process; P:catabolic process; C:cytosol; P:cellular protein modification process; P:generation of precursor metabolites and energy; P:anatomical structure morphogenesis; P:embryo development; F:protein kinase activity; C:protein complex; C:cytoplasm; F:nucleotide binding; F:protein binding; P:biosynthetic process | IPR000719 (PFAM); IPR002290 (SMART); IPR002291 (PRINTS); IPR008271 (PROSITE); IPR011009 (SUPERFAMILY); IPR017441 (PROSITE); IPR020635 (SMART); IPR020636 (PANTHER); G3DSA:1.10.510.10 (GENE3D), G3DSA:3.30.200.20 (GENE3D), PTHR24347:SF48 (PANTHER), tmhmm (TMHMM) |
| 1127 | lib_dib_Filter15_IDBA_contig_21377_minus3 | 644 | phosphorylase gamma 1 | 836 | 0 | 73.05% | 13 | P:carbohydrate metabolic process; P:catabolic process; C:cytosol; P:cellular protein modification process; P:generation of precursor metabolites and energy; P:anatomical structure morphogenesis; P:embryo development; F:protein kinase activity; C:protein complex; C:cytoplasm; F:nucleotide binding; F:protein binding; P:biosynthetic process | IPR000719 (PFAM); IPR002290 (SMART); IPR002291 (PRINTS); IPR008271 (PROSITE); IPR011009 (SUPERFAMILY); IPR017441 (PROSITE); IPR020635 (SMART); IPR020636 (PANTHER); G3DSA:1.10.510.10 (GENE3D), G3DSA:3.30.200.20 (GENE3D), PTHR24347:SF48 (PANTHER), tmhmm (TMHMM) |

| | | | | | | | | | | |
|---|---|---|---|---|---|---|---|---|---|---|
| 1128 | lib_dib_Filter15_IDBA_contig_21401_plus3 | 609 | steryl- partial | 820 | 0 | 64.30% | 12 | P:biological_process; C:lysosome; P:biosynthetic process; P:catabolic process; P:lipid metabolic process; P:multicellular organismal development; C:endosome; C:cell; F:hydrolase activity; C:endoplasmic reticulum; C:Golgi apparatus; C:plasma membrane | IPR000917 (PFAM); IPR017849 (G3DSA:3.40.720.GENE3D); IPR017850 (SUPERFAMILY); IPR024607 (PROSITE); G3DSA:3.30.1120.10 (GENE3D), PTHR10342 (PANTHER), PTHR10342:SF71 (PANTHER), tmhmm (TMHMM) | |
| 1129 | lib_dib_Filter15_IDBA_contig_21407_plus3 | 602 | syntaxin 7 | 547 | 1.07E-80 | 70.15% | 5 | P:transport; C:cell; C:endosome; F:protein binding; P:protein transport | IPR000727 (PFAM); IPR006011 (PFAM); IPR006012 (PROSITE); IPR010989 (SUPERFAMILY); G3DSA:1.20.58.70 (GENE3D), PTHR19957 (PANTHER), PTHR19957:SF17 (PANTHER), tmhmm (TMHMM) | |
| 1130 | lib_dib_Filter15_IDBA_contig_21411_plus3 | 297 | t-snare domain-containing protein 1 | 479 | 1.54E-56 | 61.85% | 4 | C:cell; F:protein binding; P:protein transport; P:transport | IPR000727 (PFAM); IPR006011 (PFAM); IPR006012 (PROSITE); IPR010989 (SUPERFAMILY); G3DSA:1.20.5.110 (GENE3D), G3DSA:1.20.58.70 (GENE3D), PTHR19957 (PANTHER), PTHR19957:SF17 (PANTHER), tmhmm (TMHMM) | |
| 1131 | lib_dib_Filter15_IDBA_contig_21474_minus3 | 278 | wd repeat-containing protein 3 | 854 | 0 | 75.35% | 3 | C:nucleolus; C:nuclear envelope; F:protein binding | IPR001680 (PFAM); IPR015943 (G3DSA:2.130.10.GENE3D); IPR017986 (PROFILE); IPR019775 (PROSITE); IPR020472 (PRINTS); PTHR19853 (PANTHER) | |
| 1132 | lib_dib_Filter15_IDBA_contig_21475_minus3 | 330 | wd repeat-containing protein 3 | 1001 | 0 | 75.35% | 3 | C:nucleolus; C:nuclear envelope; F:protein binding | IPR001680 (PFAM); IPR007148 (PFAM); IPR015943 (G3DSA:2.130.10.GENE3D); IPR017986 (PROFILE); IPR019775 (PROSITE); IPR020472 (PRINTS); PTHR19853 (PANTHER) | |
| 1133 | lib_dib_Filter15_IDBA_contig_21480_minus2 | 155 | epidermal retinol dehydrogenase 2-like | 479 | 7.28E-104 | 65.55% | 3 | P:metabolic process; F:catalytic activity; F:nucleotide binding | IPR002198 (PRINTS); IPR002347 (PRINTS); IPR016040 (G3DSA:3.40.50.GENE3D); IPR020842 (SMART); PTHR24316 (PANTHER), PTHR24316:SF1 (PANTHER), SignalP-NN(euk) (SIGNALP), tmhmm (TMHMM), SSF51735 (SUPERFAMILY) | |
| 1134 | lib_dib_Filter15_IDBA_contig_21543_minus1 | 696 | ribosome-binding protein 1 | 1076 | 1.71E-14 | 54.00% | 2 | P:biological_process; F:cytoskeletal protein binding | IPR024854 (PANTHER) | |
| 1135 | lib_dib_Filter15_IDBA_contig_21549_plus1 | 128 | neurogenic locus notch homolog protein 3 | 477 | 5.65E-08 | 52.20% | 13 | P:multicellular organismal development; P:regulation of biological process; ; P:signal transduction; F:protein binding; P:cell differentiation; P:cell proliferation; P:response to external stimulus; P:anatomical structure morphogenesis; P:response to stress; P:growth; C:plasma membrane; F:calcium ion binding | IPR000152 (PROSITE); IPR000742 (SMART); IPR001881 (PFAM); IPR002889 (PROFILE); IPR018097 (PROSITE); G3DSA:2.10.25.10 (GENE3D), PTHR24039 (PANTHER), SignalP-NN(euk) (SIGNALP), tmhmm (TMHMM), SSF57196 (SUPERFAMILY) | Yes |
| 1136 | lib_dib_Filter15_IDBA_contig_21570_minus2 | 92 | serine threonine-protein kinase h1 | 498 | 0 | 86.30% | 3 | F:nucleotide binding; P:cellular protein modification process; F:protein kinase activity | IPR000719 (PFAM); IPR002290 (SMART); IPR008271 (PROSITE); IPR011009 (SUPERFAMILY); IPR017441 (PROSITE); IPR020635 (SMART); IPR020636 (PANTHER); G3DSA:1.10.510.10 (GENE3D), G3DSA:3.30.200.20 (GENE3D), PTHR24347:SF15 (PANTHER), SignalP-NN(euk) (SIGNALP) | Yes |
| 1137 | lib_dib_Filter15_IDBA_contig_21571_minus2 | 28370 | cysteine-rich secretory protein mr30 | 476 | 6.16E-53 | 47.90% | 6 | C:extracellular region; P:proteolysis; F:hydrolase activity; F:peptidase activity; F:ion channel inhibitor activity; F:calcium channel inhibitor activity | IPR001283 (PRINTS); IPR003582 (SMART); IPR013032 (PROSITE); IPR014044 (G3DSA:3.40.33.GENE3D); SignalP-NN(euk) (SIGNALP), tmhmm (TMHMM), SSF57196 (SUPERFAMILY) | |

| | | | | | | | | | |
|---|---|---|---|---|---|---|---|---|---|
| 1138 | lib_dib_Filter15_IDBA_contig_21581_plus2 | 5051 | cathepsin d | 821 | 0 | 80.10% | 4 | C:lysosome; P:protein metabolic process; P:catabolic process; F:peptidase activity | IPR001461 (PRINTS); IPR001969 (PROSITE); IPR012848 (PFAM); IPR021109 (G3DSA:2.40.70.GENE3D); PTHR13683:SF84 (PANTHER), SignalP-NN(euk) (SIGNALP), tmhmm (TMHMM) |
| 1139 | lib_dib_Filter15_IDBA_contig_21582_plus2 | 567 | cysteine-rich secretory protein mr30 | 1245 | 2.47E-57 | 51.15% | 1 | C:extracellular region | IPR001283 (PRINTS); IPR002413 (PRINTS); IPR014044 (G3DSA:3.40.33.GENE3D); IPR018244 (PROSITE); tmhmm (TMHMM) |
| 1140 | lib_dib_Filter15_IDBA_contig_21591_minus3 | 161 | kelch-like protein 5 | 502 | 0 | 87.30% | 3 | C:cytoskeleton; C:cytoplasm; F:actin binding | IPR006652 (PFAM); IPR011705 (PFAM); IPR015916 (G3DSA:2.130.10.GENE3D); PTHR24412 (PANTHER), PTHR24412:SF73 (PANTHER), SSF117281 (SUPERFAMILY) |
| 1141 | lib_dib_Filter15_IDBA_contig_21592_minus3 | 353 | kelch-like protein 5 | 959 | 0 | 87.30% | 3 | C:cytoskeleton; C:cytoplasm; F:actin binding | IPR006652 (PFAM); IPR011705 (PFAM); IPR015916 (G3DSA:2.130.10.GENE3D); PTHR24412 (PANTHER), PTHR24412:SF73 (PANTHER), SSF117281 (SUPERFAMILY) |
| 1142 | lib_dib_Filter15_IDBA_contig_216_minus3 | 4564 | kelch-like 9 | 706 | 7.39E-73 | 47.10% | 2 | P:biological_process; F:protein binding | IPR000210 (SMART); IPR006652 (PFAM); IPR011333 (G3DSA:3.30.710.GENE3D); IPR011705 (PFAM); IPR013069 (PFAM); IPR015915 (G3DSA:2.120.10.GENE3D); PTHR24412 (PANTHER), SignalP-NN(euk) (SIGNALP), SSF117281 (SUPERFAMILY) |
| 1143 | lib_dib_Filter15_IDBA_contig_21608_plus1 | 136 | epidermal retinol dehydrogenase 2-like | 496 | 7.48E-101 | 69.65% | 3 | F:catalytic activity; P:metabolic process; F:nucleotide binding | IPR002198 (PRINTS); IPR002347 (PRINTS); IPR016040 (G3DSA:3.40.50.GENE3D); IPR020904 (PROSITE); PTHR24316 (PANTHER), PTHR24316:SF1 (PANTHER), tmhmm (TMHMM), SSF51735 (SUPERFAMILY) |
| 1144 | lib_dib_Filter15_IDBA_contig_21650_plus2 | 1627 | ece1 protein | 981 | 3.27E-44 | 42.30% | 3 | F:peptidase activity; P:protein metabolic process; P:catabolic process | IPR000718 (PANTHER); IPR008753 (PFAM); IPR018497 (PFAM); G3DSA:1.10.1380.10 (GENE3D), tmhmm (TMHMM), SSF55486 (SUPERFAMILY) |
| 1145 | lib_dib_Filter15_IDBA_contig_21669_minus2 | 150 | fat-like cadherin-related tumor suppressor homolog | 773 | 0 | 58.30% | 4 | F:protein binding; F:calcium ion binding; P:biological_process; C:plasma membrane | IPR000742 (SMART); IPR001791 (PFAM); IPR002126 (PRINTS); IPR008985 (SUPERFAMILY); IPR013032 (PROSITE); IPR013320 (G3DSA:2.60.120.GENE3D); IPR015919 (SUPERFAMILY); IPR020894 (PROSITE); G3DSA:2.10.25.10 (GENE3D), PTHR24668 (PANTHER), PTHR24668:SF24 (PANTHER), SSF57196 (SUPERFAMILY) |
| 1146 | lib_dib_Filter15_IDBA_contig_21674_plus3 | 383 | von willebrand factor type a domain containing protein | 672 | 4.82E-136 | 49.75% | 2 | F:transferase activity; F:protein binding | IPR002035 (SMART); G3DSA:3.40.50.410 (GENE3D), PTHR10579 (PANTHER), PF13519 (PFAM), SSF53300 (SUPERFAMILY) |
| 1147 | lib_dib_Filter15_IDBA_contig_21677_plus1 | 1297 | serine threonine-protein kinase plk1 | 1453 | 0 | 60.90% | 16 | F:protein kinase activity; C:protein complex; P:regulation of biological process; P:protein metabolic process; P:biological_process; P:cell cycle; C:cytoskeleton; F:protein binding; P:response to stress; C:cytoplasm; C:nucleus; P:organelle organization; P:cellular protein modification process; P:reproduction; P:cell differentiation; F:nucleotide binding | IPR000719 (PFAM); IPR000959 (PFAM); IPR002290 (SMART); IPR008271 (PROSITE); IPR011009 (SUPERFAMILY); IPR017441 (PROSITE); IPR020635 (SMART); G3DSA:1.10.510.10 (GENE3D), G3DSA:3.30.1120.30 (GENE3D), G3DSA:3.30.200.20 (GENE3D), PTHR24345 (PANTHER), PTHR24345:SF46 (PANTHER), SSF82615 (SUPERFAMILY) |

| | | | | | | | | | | |
|---|---|---|---|---|---|---|---|---|---|---|
| 1148 | lib_dib_Filter15_IDBA_contig_21678_plus1 | 1239 | serine threonine-protein kinase plk1 | 1333 | 0 | 60.90% | 16 | F:protein kinase activity; C:protein complex; P:regulation of biological process; P:protein metabolic process; P:biological_process; P:cell cycle; C:cytoskeleton; F:protein binding; P:response to stress; C:cytoplasm; C:nucleus; P:organelle organization; P:cellular protein modification process; P:reproduction; P:cell differentiation; F:nucleotide binding | IPR000719 (PFAM); IPR000959 (PFAM); IPR002290 (SMART); IPR008271 (PROSITE); IPR011009 (SUPERFAMILY); IPR017441 (PROSITE); IPR020635 (SMART); G3DSA:1.10.510.10 (GENE3D), G3DSA:3.30.1120.30 (GENE3D), G3DSA:3.30.200.20 (GENE3D), PTHR24345 (PANTHER), PTHR24345:SF46 (PANTHER), SSF82615 (SUPERFAMILY) | |
| 1149 | lib_dib_Filter15_IDBA_contig_21693_minus3 | 141 | protein | 615 | 7.33E-39 | 49.60% | 2 | F:catalytic activity; F:protein binding | IPR001611 (PROFILE); IPR003591 (SMART); IPR026906 (PFAM); G3DSA:3.80.10.10 (GENE3D), PTHR24367 (PANTHER), PF13855 (PFAM), tmhmm (TMHMM), SSF52058 (SUPERFAMILY) | |
| 1150 | lib_dib_Filter15_IDBA_contig_21701_minus2 | 116 | leukocyte elastase inhibitor | 504 | 6.66E-97 | 61.50% | 4 | P:biological_process; P:protein metabolic process; P:catabolic process; F:enzyme regulator activity | IPR000215 (PANTHER); IPR023796 (PFAM); G3DSA:2.30.39.10 (GENE3D), G3DSA:3.30.497.10 (GENE3D) | |
| 1151 | lib_dib_Filter15_IDBA_contig_21755_minus3 | 271 | membrane-bound trehalase | 774 | 1.32E-168 | 62.85% | 2 | F:hydrolase activity; P:carbohydrate metabolic process | IPR001661 (PRINTS); IPR008928 (SUPERFAMILY); IPR018232 (PROSITE); PTHR23403:SF1 (PANTHER), tmhmm (TMHMM) | |
| 1152 | lib_dib_Filter15_IDBA_contig_21756_minus3 | 280 | membrane-bound trehalase | 805 | 2.74E-168 | 62.80% | 2 | F:hydrolase activity; P:carbohydrate metabolic process | IPR001661 (PRINTS); IPR008928 (SUPERFAMILY); IPR018232 (PROSITE); PTHR23403:SF1 (PANTHER), tmhmm (TMHMM) | |
| 1153 | lib_dib_Filter15_IDBA_contig_21757_minus3 | 273 | membrane-bound trehalase | 799 | 4.04E-168 | 62.85% | 2 | F:hydrolase activity; P:carbohydrate metabolic process | IPR001661 (PRINTS); IPR008928 (SUPERFAMILY); IPR018232 (PROSITE); PTHR23403:SF1 (PANTHER), tmhmm (TMHMM) | |
| 1154 | lib_dib_Filter15_IDBA_contig_21777_minus3 | 88 | taf5-like rna polymerase ii p300 cbp-associated factor-associated factor 65 kda subunit 5l | 472 | 3.57E-149 | 65.85% | 10 | ; P:regulation of biological process; P:organelle organization; P:cellular protein modification process; C:nucleoplasm; C:protein complex; F:sequence-specific DNA binding transcription factor activity; F:transferase activity; F:transcription regulator activity; F:protein binding | IPR001680 (PFAM); IPR007582 (PFAM); IPR015943 (G3DSA:2.130.10.GENE3D); IPR017986 (PROFILE); IPR019775 (PROSITE); IPR020472 (PRINTS); PTHR19879 (PANTHER), PTHR19879:SF0 (PANTHER) | |
| 1155 | lib_dib_Filter15_IDBA_contig_21787_minus1 | 6058 | ---NA--- | 1042 | | | 0 | - | no IPS match | |
| 1156 | lib_dib_Filter15_IDBA_contig_21787_minus3 | 6058 | collagen alpha-6 chain | 1041 | 7.05E-80 | 47.70% | 1 | F:protein binding | IPR002035 (PFAM); IPR008160 (PFAM); PR00453 (PRINTS), G3DSA:3.40.50.410 (GENE3D), PTHR22992 (PANTHER), SignalP-NN(euk) (SIGNALP), tmhmm (TMHMM), SSF53300 (SUPERFAMILY) | Yes |
| 1157 | lib_dib_Filter15_IDBA_contig_21787_plus1 | 6058 | ---NA--- | 1042 | | | 0 | - | PTHR24023 (PANTHER), PTHR24023:SF119 (PANTHER) | |
| 1158 | lib_dib_Filter15_IDBA_contig_21800_plus2 | 2698 | nicotinic acetylcholine receptor alpha isoform e | 400 | 6.60E-33 | 52.50% | 4 | C:plasma membrane; F:ion channel activity; C:cell; P:ion transport | IPR006201 (PANTHER); IPR006202 (G3DSA:2.70.170.GENE3D); PTHR18945:SF127 (PANTHER) | |
| 1159 | lib_dib_Filter15_IDBA_contig_21803_plus3 | 138 | nicotinic acetylcholine receptor a8 subunit | 521 | 9.20E-158 | 66.55% | 3 | F:ion channel activity; P:ion transport; C:cell | IPR002394 (PRINTS); IPR006029 (PFAM); IPR006201 (PRINTS); IPR006202 (G3DSA:2.70.170.GENE3D); IPR018000 (PROSITE); G3DSA:1.20.120.370 (GENE3D), PTHR18945:SF88 (PANTHER), tmhmm (TMHMM) | |

| | | | | | | | | | |
|---|---|---|---|---|---|---|---|---|---|
| 1160 | lib_dib_Filter15_IDBA_contig_21821_minus3 | 242 | pre-mrna-processing factor 19 | 753 | 0 | 83.05% | 22 | P:cellular protein modification process; P:cell differentiation; P:multicellular organismal development; P:regulation of biological process; C:nucleoplasm; P:biosynthetic process; P:lipid metabolic process; F:catalytic activity; P:cell proliferation; P:embryo development; P:growth; C:protein complex; C:intracellular; C:nucleus; C:cytoskeleton; F:protein binding; P:cellular component organization; P:nucleobase-containing compound metabolic process; F:DNA binding; C:lipid particle; P:response to stress; P:DNA metabolic process | IPR001680 (PFAM); IPR003613 (SMART); IPR013083 (G3DSA:3.30.40.GENE3D); IPR013915 (PFAM); IPR015943 (G3DSA:2.130.10.GENE3D); IPR017986 (PROFILE); IPR019775 (PROSITE); IPR020472 (PRINTS); PTHR13889 (PANTHER), PTHR13889:SF8 (PANTHER), SSF57850 (SUPERFAMILY) |
| 1161 | lib_dib_Filter15_IDBA_contig_21907_plus1 | 691 | blastula protease-10 | 541 | 2.21E-58 | 53.15% | 4 | F:peptidase activity; P:protein metabolic process; P:catabolic process; F:binding | IPR001506 (PRINTS); IPR003582 (PFAM); IPR006026 (SMART); IPR024079 (G3DSA:3.40.390.GENE3D); PTHR10127 (PANTHER), PTHR10127:SF334 (PANTHER), SignalP-NN(euk) (SIGNALP), SSF55486 (SUPERFAMILY) |
| 1162 | lib_dib_Filter15_IDBA_contig_21910_plus1 | 2378 | membrane metallo-endopeptidase-like 1 | 918 | 2.58E-48 | 44.75% | 5 | C:organelle; C:intracellular; F:peptidase activity; P:protein metabolic process; P:catabolic process | IPR000519 (G3DSA:4.10.110.GENE3D); IPR000718 (PANTHER); IPR008753 (PFAM); IPR024079 (G3DSA:3.40.390.GENE3D); G3DSA:1.10.1380.10 (GENE3D), tmhmm (TMHMM), SSF55486 (SUPERFAMILY) |
| 1163 | lib_dib_Filter15_IDBA_contig_21912_plus3 | 95 | low-density lipoprotein | 469 | 4.20E-29 | 52.40% | 23 | P:protein transport; P:response to abiotic stimulus; P:growth; P:regulation of biological process; P:transport; P:cellular component organization; C:plasma membrane; P:cell-cell signaling; P:anatomical structure morphogenesis; P:multicellular organismal development; P:response to external stimulus; C:cell; P:metabolic process; P:biological_process; P:cell proliferation; C:extracellular space; C:Golgi apparatus; F:protein binding; C:cytoplasmic membrane-bounded vesicle; C:endosome; F:receptor activity; C:endoplasmic reticulum; F:transporter activity | IPR000998 (PFAM); IPR002172 (G3DSA:4.10.400.GENE3D); IPR008985 (SUPERFAMILY); IPR023415 (PROSITE); PR00261 (PRINTS), PTHR10529 (PANTHER), tmhmm (TMHMM) |
| 1164 | lib_dib_Filter15_IDBA_contig_21915_minus3 | 1668 | von willebrand factor type egf and pentraxin domain-containing protein 1 | 110 | 1.23E-19 | 52.20% | 9 | P:regulation of transcription, DNA-dependent; F:sequence-specific DNA binding; F:sequence-specific DNA binding transcription factor activity; F:DNA binding; F:calcium ion binding; C:cytoplasm; P:cell adhesion; C:membrane; C:extracellular region | IPR000436 (PFAM); IPR011641 (PFAM); IPR013032 (PFAM); G3DSA:2.10.70.10 (GENE3D), PTHR19325 (PANTHER), PTHR19325:SF43 (PANTHER) |
| 1165 | lib_dib_Filter15_IDBA_contig_21917_minus1 | 698 | fibrillin-1- partial | 101 | 9.77E-28 | 58.35% | 1 | F:calcium ion binding | IPR001881 (PFAM); IPR018097 (PROSITE); G3DSA:2.10.25.10 (GENE3D), PTHR24034 (PANTHER), SSF57196 (SUPERFAMILY) |
| 1166 | lib_dib_Filter15_IDBA_contig_21918_minus2 | 1532 | cub and sushi domain-containing protein 1 | 149 | 7.17E-23 | 52.00% | 3 | F:chitin binding; P:chitin metabolic process; C:extracellular region | IPR000436 (PFAM); G3DSA:2.10.70.10 (GENE3D), PTHR19325 (PANTHER) |
| 1167 | lib_dib_Filter15_IDBA_contig_21919_minus2 | 250 | von willebrand factor type egf and pentraxin domain-containing protein 1-like | 177 | 2.12E-30 | 50.85% | 4 | C:cell; P:biological_process; P:regulation of biological process; F:binding | IPR000436 (PFAM); G3DSA:2.10.70.10 (GENE3D), PTHR19325 (PANTHER) |

| | | | | | | | | | |
|---|---|---|---|---|---|---|---|---|---|
| 1168 | lib_dib_Filter15_IDBA_contig_21968_plus3 | 1688 | f-box wd repeat-containing protein 1a isoform 2 | 1310 | 0 | 90.10% | 19 | P:cellular protein modification process; P:cell proliferation; P:multicellular organismal development; P:cell cycle; P:signal transduction; P:viral reproduction; P:biological_process; P:metabolic process; P:regulation of biological process; ; F:protein binding; C:cytosol; C:intracellular; C:protein complex; F:catalytic activity; P:protein metabolic process; P:catabolic process; P:anatomical structure morphogenesis; C:nucleus | IPR001680 (PFAM); IPR001810 (SMART); IPR015943 (G3DSA:2.130.10.GENE3D); IPR017986 (PROFILE); IPR019775 (PROSITE); IPR020472 (PRINTS); IPR021977 (PFAM); G3DSA:1.20.1280.50 (GENE3D), PTHR22844 (PANTHER), PTHR22844:SF10 (PANTHER), PF12937 (PFAM), tmhmm (TMHMM) |
| 1169 | lib_dib_Filter15_IDBA_contig_21977_minus3 | 59 | ribosomal protein s6 kinase alpha-2 | 468 | 0 | 82.20% | 17 | P:metabolic process; P:regulation of biological process; P:signal transduction; C:ribosome; P:cell death; P:organelle organization; P:cell cycle; F:protein kinase activity; P:multicellular organismal development; P:protein metabolic process; P:biological_process; C:cytoskeleton; P:cellular protein modification process; F:binding; P:reproduction; P:cell differentiation; F:nucleotide binding | IPR000719 (PFAM); IPR002290 (SMART); IPR008271 (PROSITE); IPR011009 (SUPERFAMILY); IPR017441 (PROSITE); IPR020635 (SMART); G3DSA:1.10.510.10 (GENE3D), G3DSA:3.30.200.20 (GENE3D), PTHR24355 (PANTHER), PTHR24355:SF0 (PANTHER) |
| 1170 | lib_dib_Filter15_IDBA_contig_21987_plus2 | 631 | phosphatidylinositol-glycan-specific phospholipase d | 1123 | 1.63E-175 | 56.65% | 17 | P:regulation of biological process; P:cell death; P:lipid metabolic process; P:biological_process; C:extracellular space; P:response to abiotic stimulus; C:cell; F:hydrolase activity; P:cell proliferation; P:transport; P:signal transduction; C:cytoplasm; P:anatomical structure morphogenesis; P:multicellular organismal development; P:protein metabolic process; P:catabolic process; P:response to endogenous stimulus | IPR001028 (PRINTS); IPR013517 (PFAM); IPR013519 (SMART); G3DSA:2.130.10.130 (GENE3D), PTHR23221 (PANTHER), PF00882 (PFAM), PF13517 (PFAM), tmhmm (TMHMM), SSF69318 (SUPERFAMILY) |
| 1171 | lib_dib_Filter15_IDBA_contig_21988_plus1 | 608 | phosphatidylinositol-glycan-specific phospholipase d | 1087 | 3.60E-175 | 57.10% | 17 | P:regulation of biological process; P:cell death; P:lipid metabolic process; P:biological_process; C:extracellular space; P:response to abiotic stimulus; C:cell; F:hydrolase activity; P:cell proliferation; P:transport; P:signal transduction; C:cytoplasm; P:anatomical structure morphogenesis; P:multicellular organismal development; P:protein metabolic process; P:catabolic process; P:response to endogenous stimulus | IPR001028 (PRINTS); IPR013517 (PFAM); IPR013519 (SMART); G3DSA:2.130.10.130 (GENE3D), PTHR23221 (PANTHER), PF00882 (PFAM), PF13517 (PFAM), SignalP-NN(euk) (SIGNALP), tmhmm (TMHMM), SSF69318 (SUPERFAMILY) |
| 1172 | lib_dib_Filter15_IDBA_contig_21989_plus1 | 293 | phosphatidylinositol-glycan-specific phospholipase d-like | 555 | 1.73E-43 | 55.25% | 16 | P:regulation of biological process; P:cell death; P:lipid metabolic process; P:biological_process; C:extracellular space; P:response to abiotic stimulus; C:cell; F:hydrolase activity; P:cell proliferation; P:signal transduction; C:cytoplasm; P:anatomical structure morphogenesis; P:multicellular organismal development; P:protein metabolic process; P:catabolic process; P:response to endogenous stimulus | IPR001028 (PTHR23221:PANTHER); IPR013517 (PFAM); IPR013519 (SMART); G3DSA:2.130.10.130 (GENE3D), PTHR23221 (PANTHER), SSF69318 (SUPERFAMILY) |

| | | | | | | | | | | |
|---|---|---|---|---|---|---|---|---|---|---|
| 1173 | lib_dib_Filter15_IDBA_contig_21992_minus1 | 1344 | adp-ribosylation factor-like protein 1 | 1028 | 6.18E-104 | 91.40% | 13 | P:transport; F:enzyme regulator activity; P:biological_process; P:signal transduction; P:nucleobase-containing compound metabolic process; P:catabolic process; P:secondary metabolic process; P:organelle organization; C:Golgi apparatus; F:protein binding; F:hydrolase activity; F:nucleotide binding; P:protein transport | IPR003579 (SMART); IPR005225 (TIGRFAMs); IPR006687 (SMART); IPR006689 (PRINTS); IPR024156 (SMART); G3DSA:3.40.50.300 (GENE3D), PTHR11711 (PANTHER), PTHR11711:SF41 (PANTHER), tmhmm (TMHMM), SSF52540 (SUPERFAMILY) | |
| 1174 | lib_dib_Filter15_IDBA_contig_21993_minus3 | 114 | kelch domain-containing protein 3 | 468 | 1.42E-164 | 71.05% | 10 | P:metabolic process; P:signal transduction; P:response to stress; P:response to biotic stimulus; C:cytosol; C:nuclear chromosome; F:protein binding; P:DNA metabolic process; P:cell cycle; F:chromatin binding | IPR006652 (PFAM); IPR011498 (PFAM); IPR015915 (G3DSA:2.120.10.GENE3D); PTHR23244 (PANTHER), PTHR23244:SF33 (PANTHER), PF13415 (PFAM), SSF117281 (SUPERFAMILY) | |
| 1175 | lib_dib_Filter15_IDBA_contig_21995_plus2 | 52 | hemicentin-1 | 468 | 1.18E-109 | 53.70% | 3 | P:anatomical structure morphogenesis; P:multicellular organismal development; F:protein binding | IPR003596 (SMART); IPR003598 (SMART); IPR003599 (SMART); IPR007110 (PROFILE); IPR013098 (PFAM); IPR013783 (G3DSA:2.60.40.GENE3D); SSF48726 (SUPERFAMILY) | |
| 1176 | lib_dib_Filter15_IDBA_contig_21996_minus1 | 537 | cub and sushi domain-containing protein partial | 1206 | 3.60E-150 | 48.30% | 2 | F:protein binding; F:calcium ion binding | IPR000152 (PROSITE); IPR000436 (PFAM); IPR000742 (SMART); IPR001881 (PFAM); IPR002919 (SUPERFAMILY); IPR018097 (PROSITE); G3DSA:2.10.25.10 (GENE3D), G3DSA:2.10.70.10 (GENE3D), PTHR19325 (PANTHER), PTHR19325:SF43 (PANTHER), SignalP-NN(euk) (SIGNALP), tmhmm (TMHMM), SSF57196 (SUPERFAMILY), SSF57586 (SUPERFAMILY) | Yes |
| 1177 | lib_dib_Filter15_IDBA_contig_21997_minus1 | 305 | cub and sushi domain-containing protein partial | 700 | 1.74E-101 | 47.55% | 1 | F:carbohydrate binding | IPR000436 (PFAM); G3DSA:2.10.70.10 (GENE3D), PTHR19325 (PANTHER), PTHR19325:SF43 (PANTHER), SignalP-NN(euk) (SIGNALP), SSF57586 (SUPERFAMILY) | Yes |
| 1178 | lib_dib_Filter15_IDBA_contig_21998_minus1 | 189 | cub and sushi domain-containing protein 2 | 501 | 7.09E-53 | 51.25% | 6 | F:carbohydrate binding; F:molecular_function; P:biological_process; C:cellular_component; C:integral to membrane; C:plasma membrane | IPR000436 (PFAM); G3DSA:2.10.70.10 (GENE3D), PTHR19325 (PANTHER), SignalP-NN(euk) (SIGNALP) | Yes |
| 1179 | lib_dib_Filter15_IDBA_contig_22002_plus1 | 1777 | kex2-like endoprotease | 907 | 2.25E-158 | 55.55% | 3 | F:peptidase activity; P:protein metabolic process; P:catabolic process | IPR000209 (G3DSA:3.40.50.GENE3D); IPR002884 (PFAM); IPR008979 (SUPERFAMILY); IPR009020 (SUPERFAMILY); IPR015500 (PRINTS); IPR022398 (PROSITE); IPR023827 (PROSITE); IPR023828 (PROSITE); G3DSA:2.60.120.260 (GENE3D), G3DSA:3.30.70.850 (GENE3D), PTHR10795:SF155 (PANTHER) | |
| 1180 | lib_dib_Filter15_IDBA_contig_22003_minus1 | 2225 | protein disulfide-isomerase a6 | 1035 | 2.48E-175 | 75.20% | 5 | P:metabolic process; F:electron carrier activity; F:catalytic activity; P:cellular homeostasis; P:regulation of biological process | IPR005746 (PRINTS); IPR005788 (TIGRFAMs); IPR012336 (G3DSA:3.40.30.GENE3D); IPR013766 (PFAM); IPR017937 (PROSITE); PTHR18929 (PANTHER), PTHR18929:SF32 (PANTHER), SignalP-NN(euk) (SIGNALP), tmhmm (TMHMM) | |
| 1181 | lib_dib_Filter15_IDBA_contig_22013_plus1 | 412 | ankyrin repeat domain-containing protein 50-like | 1019 | 6.80E-39 | 46.80% | 5 | F:peptidase activity; P:protein metabolic process; P:catabolic process; F:protein binding; P:cell death | IPR001309 (PROFILE); IPR002110 (PRINTS); IPR002138 (PROFILE); IPR011600 (PFAM); IPR015917 (PRINTS); IPR020683 (G3DSA:1.25.40.GENE3D); G3DSA:3.40.50.1460 (GENE3D), PTHR24198 (PANTHER), PF13857 (PFAM), SSF52129 (SUPERFAMILY) | |

| | | | | | | | | | | |
|---|---|---|---|---|---|---|---|---|---|---|
| 1182 | lib_dib_Filter15_IDBA_contig_22016_minus1 | 95 | dmbt1 protein | 520 | 3.74E-43 | 43.15% | 4 | F:binding; C:intracellular; C:cell; F:receptor activity | IPR000519 (G3DSA:4.10.110.GENE3D); IPR000859 (G3DSA:2.60.120.GENE3D); IPR001190 (PRINTS); IPR017448 (SMART); G3DSA:3.10.250.10 (GENE3D), PTHR19331 (PANTHER), PTHR19331:SF118 (PANTHER), tmhmm (TMHMM) | |
| 1183 | lib_dib_Filter15_IDBA_contig_22017_minus1 | 80 | deleted in malignant brain tumors 1 | 352 | 2.87E-23 | 62.45% | 2 | F:receptor activity; C:cell | IPR001190 (PRINTS); IPR017448 (SMART); G3DSA:3.10.250.10 (GENE3D), PTHR19331 (PANTHER), PTHR19331:SF118 (PANTHER) | |
| 1184 | lib_dib_Filter15_IDBA_contig_22018_minus1 | 20 | deleted in malignant brain tumors 1 | 116 | 2.63E-22 | 57.90% | 3 | F:hydrolase activity; F:receptor activity; C:cell | IPR001190 (PRINTS); IPR017448 (SUPERFAMILY); G3DSA:3.10.250.10 (GENE3D), PTHR19331 (PANTHER) | |
| 1185 | lib_dib_Filter15_IDBA_contig_22019_plus1 | 67 | collectin-12-like isoform 3 | 467 | 8.38E-11 | 49.85% | 1 | F:carbohydrate binding | IPR001304 (PFAM); IPR016186 (G3DSA:3.10.100.GENE3D); IPR016187 (SUPERFAMILY); PTHR22802 (PANTHER), PTHR22802:SF16 (PANTHER) | |
| 1186 | lib_dib_Filter15_IDBA_contig_22025_minus3 | 69 | er degradation mannosidase alpha-like 1 | 466 | 0 | 82.25% | 13 | P:protein metabolic process; P:catabolic process; C:endoplasmic reticulum; F:hydrolase activity; F:calcium ion binding; P:metabolic process; P:signal transduction; P:response to stress; P:response to biotic stimulus; F:protein binding; P:cellular protein modification process; P:biosynthetic process; P:carbohydrate metabolic process | IPR001382 (PRINTS); PTHR11742:SF11 (PANTHER), SignalP-NN(euk) (SIGNALP), tmhmm (TMHMM) | Yes |
| 1187 | lib_dib_Filter15_IDBA_contig_22026_minus3 | 106 | retinol dehydrogenase 13-like | 466 | 4.16E-104 | 64.85% | 3 | P:metabolic process; F:catalytic activity; F:nucleotide binding | IPR002198 (PRINTS); IPR002347 (PRINTS); IPR016040 (G3DSA:3.40.50.GENE3D); PTHR24320 (PANTHER), PTHR24320:SF0 (PANTHER), SignalP-NN(euk) (SIGNALP), tmhmm (TMHMM), SSF51735 (SUPERFAMILY) | |
| 1188 | lib_dib_Filter15_IDBA_contig_22038_plus3 | 80 | | 466 | 0 | 100% | 1 | F:protein binding | IPR003599 (SMART); IPR007110 (PROFILE); IPR013098 (PFAM); IPR013783 (G3DSA:2.60.40.GENE3D); PF13895 (PFAM), SSF48726 (SUPERFAMILY) | |
| 1189 | lib_dib_Filter15_IDBA_contig_22050_plus2 | 338 | zinc finger lsd1 subclass family protein | 901 | 6.34E-08 | 44.90% | 8 | P:proteolysis; F:serine-type endopeptidase activity; F:serine-type peptidase activity; F:hydrolase activity; F:peptidase activity; F:zinc ion binding; C:intracellular; F:nucleic acid binding | IPR006212 (SMART); IPR009030 (SUPERFAMILY); G3DSA:2.10.220.10 (GENE3D), PTHR23275 (PANTHER), PTHR23275:SF7 (PANTHER), tmhmm (TMHMM) | |
| 1190 | lib_dib_Filter15_IDBA_contig_22051_plus1 | 368 | zinc finger lsd1 subclass family protein | 953 | 5.79E-08 | 44.90% | 8 | P:proteolysis; F:serine-type endopeptidase activity; F:serine-type peptidase activity; F:hydrolase activity; F:peptidase activity; F:zinc ion binding; C:intracellular; F:nucleic acid binding | IPR006212 (SMART); IPR009030 (SUPERFAMILY); G3DSA:2.10.220.10 (GENE3D), PTHR23275 (PANTHER), PTHR23275:SF7 (PANTHER), tmhmm (TMHMM) | |
| 1191 | lib_dib_Filter15_IDBA_contig_22065_minus1 | 289 | peptidyl-prolyl cis-trans isomerase d | 500 | 5.16E-124 | 69.20% | 12 | P:response to stress; F:protein binding; P:cellular protein modification process; C:cell; C:mitochondrion; C:cytoskeleton; P:regulation of biological process; P:cell death; F:catalytic activity; F:binding; P:cellular homeostasis; P:protein metabolic process | IPR002130 (PRINTS); IPR011990 (G3DSA:1.25.40.GENE3D); IPR013026 (PROFILE); IPR019734 (SMART); IPR020892 (PROSITE); G3DSA:2.40.100.10 (GENE3D), PTHR11071 (PANTHER), PTHR11071:SF110 (PANTHER), PF13414 (PFAM), SSF48452 (SUPERFAMILY) | |
| 1192 | lib_dib_Filter15_IDBA_contig_22077_plus2 | 171 | cyclin-dependent kinase 17 | 637 | 0 | 87.40% | 3 | P:cellular protein modification process; F:nucleotide binding; F:protein kinase activity | IPR000719 (PFAM); IPR002290 (SMART); IPR008271 (PROSITE); IPR011009 (SUPERFAMILY); IPR017441 (PROSITE); IPR020635 (SMART); G3DSA:1.10.510.10 (GENE3D), G3DSA:3.30.200.20 (GENE3D), PTHR24056 (PANTHER), PTHR24056:SF128 (PANTHER) | |

| | | | | | | | | | | |
|---|---|---|---|---|---|---|---|---|---|---|
| 1193 | lib_dib_Filter15_IDBA_contig_2209_plus3 | 974 | serine threonine-protein kinase lats1 | 1573 | 0 | 77.00% | 17 | P:organelle organization; P:cell cycle; P:regulation of biological process; P:cellular protein modification process; P:signal transduction; F:protein binding; P:response to endogenous stimulus; P:protein transport; P:cytoskeleton organization; P:metabolic process; C:cytosol; F:protein kinase activity; F:binding; P:cell differentiation; P:multicellular organismal development; F:nucleotide binding; C:cytoskeleton | IPR000719 (PFAM); IPR002290 (SMART); IPR008271 (PROSITE); IPR009060 (SUPERFAMILY); IPR011009 (SUPERFAMILY); IPR017441 (PROSITE); IPR017892 (PFAM); IPR020635 (SMART); G3DSA:1.10.510.10 (GENE3D), G3DSA:3.30.200.20 (GENE3D), PTHR24356 (PANTHER), PTHR24356:SF2 (PANTHER), tmhmm (TMHMM) | |
| 1194 | lib_dib_Filter15_IDBA_contig_22103_plus2 | 1924 | subtilisin-chymotrypsin inhibitor ci-1b | 524 | 1.17E-13 | 63.25% | 3 | F:enzyme regulator activity; P:response to external stimulus; P:response to stress | IPR000864 (PRODOM); G3DSA:3.30.10.10 (GENE3D) | |
| 1195 | lib_dib_Filter15_IDBA_contig_22104_plus3 | 84 | proteinase inhibitor | 122 | 4.05E-15 | 65.05% | 3 | F:enzyme regulator activity; P:response to external stimulus; P:response to stress | IPR000864 (PRODOM); G3DSA:3.30.10.10 (GENE3D) | |
| 1196 | lib_dib_Filter15_IDBA_contig_22113_minus3 | 57 | protein | 463 | 1.53E-79 | 52.25% | 2 | F:protein binding; F:calcium ion binding | IPR000152 (PROSITE); IPR000742 (SMART); IPR001881 (PFAM); IPR013032 (PROSITE); IPR018097 (PROSITE); IPR023413 (G3DSA:2.40.155.GENE3D); IPR024731 (PFAM); IPR026823 (PFAM); PD968187 (PRODOM), G3DSA:2.10.25.10 (GENE3D), PTHR24034 (PANTHER), SSF57196 (SUPERFAMILY) | |
| 1197 | lib_dib_Filter15_IDBA_contig_22168_minus3 | 2061 | calretinin | 892 | 4.68E-26 | 66.05% | 3 | C:plasma membrane; C:cytoplasm; F:calcium ion binding | IPR002048 (SMART); IPR011992 (G3DSA:1.10.238.GENE3D); IPR018247 (PROSITE); PTHR19972 (PANTHER), SSF47473 (SUPERFAMILY) | |
| 1198 | lib_dib_Filter15_IDBA_contig_22169_minus3 | 2053 | calretinin | 890 | 2.28E-26 | 66.05% | 3 | C:plasma membrane; C:cytoplasm; F:calcium ion binding | IPR002048 (SMART); IPR011992 (G3DSA:1.10.238.GENE3D); IPR018247 (PROSITE); PTHR19972 (PANTHER), SSF47473 (SUPERFAMILY) | |
| 1199 | lib_dib_Filter15_IDBA_contig_22176_minus2 | 94 | zinc finger protein glis2 | 462 | 1.84E-74 | 79.25% | 9 | P:signal transduction; F:binding; ; P:regulation of biological process; F:DNA binding; C:nucleoplasm; P:multicellular organismal development; P:cell differentiation; C:cytoplasm | IPR007087 (PROSITE); IPR013087 (G3DSA:3.30.160.GENE3D); IPR015880 (SMART); PTHR19818 (PANTHER), PTHR19818:SF11 (PANTHER), PF13465 (PFAM), PF13894 (PFAM), SSF57667 (SUPERFAMILY) | |
| 1200 | lib_dib_Filter15_IDBA_contig_22177_minus1 | 124 | 4-trimethylaminobutyraldehyde dehydrogenase | 533 | 0 | 73.40% | 9 | C:cytosol; F:binding; P:metabolic process; P:biosynthetic process; ; F:catalytic activity; P:multicellular organismal development; P:cell-cell signaling; C:plasma membrane | IPR015590 (PFAM); IPR016160 (PROSITE); IPR016161 (SUPERFAMILY); IPR016162 (G3DSA:3.40.605.GENE3D); IPR016163 (G3DSA:3.40.309.GENE3D); PTHR11699 (PANTHER), PTHR11699:SF47 (PANTHER) | |
| 1201 | lib_dib_Filter15_IDBA_contig_22180_minus2 | 75 | leucine-rich repeat-containing protein 20 | 462 | 3.35E-20 | 60.30% | 1 | P:biological_process | IPR025875 (PFAM); G3DSA:3.80.10.10 (GENE3D), PTHR26343 (PANTHER), PTHR26343:SF21 (PANTHER), SignalP-NN(euk) (SIGNALP), SSF52058 (SUPERFAMILY) | Yes |
| 1202 | lib_dib_Filter15_IDBA_contig_22183_minus3 | 41 | carbohydrate sulfotransferase 11 | 461 | 3.76E-36 | 49.35% | 8 | P:multicellular organismal development; F:transferase activity; P:regulation of biological process; P:anatomical structure morphogenesis; P:embryo development; C:cell; P:biosynthetic process; P:carbohydrate metabolic process | IPR005331 (PFAM); IPR018011 (PANTHER) | |

| | | | | | | | | | | |
|---|---|---|---|---|---|---|---|---|---|---|
| 1203 | lib_dib_Filter15_IDBA_contig_22189_plus2 | 478 | serine threonine-protein kinase tnni3k | 921 | 0 | 69.40% | 7 | C:cytoplasm; P:cellular protein modification process; F:protein binding; C:nucleus; F:cytoskeletal protein binding; F:nucleotide binding; F:protein kinase activity | IPR000719 (PROFILE); IPR001245 (PFAM); IPR002110 (PRINTS); IPR002290 (SMART); IPR011009 (SUPERFAMILY); IPR020635 (SMART); IPR020683 (G3DSA:1.25.40.GENE3D); G3DSA:1.10.510.10 (GENE3D), G3DSA:3.30.200.20 (GENE3D), PTHR23257 (PANTHER), PTHR23257:SF63 (PANTHER), SignalP-NN(euk) (SIGNALP) | |
| 1204 | lib_dib_Filter15_IDBA_contig_222_plus2 | 2430 | group 3 secretory phospholipase a2 | 166 | 5.88E-13 | 65.10% | 5 | P:biological_process; F:hydrolase activity; F:calcium ion binding; P:catabolic process; P:lipid metabolic process | IPR001211 (PANTHER); IPR016090 (G3DSA:1.20.90.GENE3D); PTHR12253:SF1 (PANTHER) | |
| 1205 | lib_dib_Filter15_IDBA_contig_22204_plus1 | 136 | viral a-type inclusion protein | 639 | 8.39E-04 | 51.00% | 0 | - | IPR009053 (SUPERFAMILY); SSF46966 (SUPERFAMILY) | |
| 1206 | lib_dib_Filter15_IDBA_contig_22208_minus3 | 279 | nacht and ankyrin domain protein | 461 | 8.26E-34 | 53.85% | 1 | F:protein binding | IPR001496 (PROFILE); IPR002110 (SMART); IPR020683 (G3DSA:1.25.40.GENE3D); PTHR24181 (PANTHER) | |
| 1207 | lib_dib_Filter15_IDBA_contig_22211_minus1 | 87 | sh3 and multiple ankyrin repeat domains protein 3 | 461 | 0 | 74.35% | 6 | C:cellular_component; C:plasma membrane; C:cell; C:organelle; C:intracellular; F:protein binding | IPR002110 (SMART); IPR020683 (G3DSA:1.25.40.GENE3D); PTHR24188 (PANTHER), PTHR24188:SF4 (PANTHER), PF13637 (PFAM) | |
| 1208 | lib_dib_Filter15_IDBA_contig_22212_minus3 | 305 | tyrosine-protein kinase src42a | 1061 | 2.95E-60 | 47.20% | 2 | F:catalytic activity; F:protein binding | IPR000483 (SMART); IPR001611 (PROFILE); IPR003591 (SMART); IPR003961 (SUPERFAMILY); IPR013783 (G3DSA:2.60.40.GENE3D); IPR026906 (PFAM); PR00019 (PRINTS), G3DSA:3.80.10.10 (GENE3D), PTHR24365 (PANTHER), PF13855 (PFAM), SM00364 (SMART), SM00365 (SMART), tmhmm (TMHMM), SSF52058 (SUPERFAMILY) | |
| 1209 | lib_dib_Filter15_IDBA_contig_22250_plus1 | 1134 | cathepsin z | 459 | 1.52E-137 | 80.15% | 8 | P:anatomical structure morphogenesis; P:multicellular organismal development; C:extracellular space; C:lysosome; F:peptidase activity; P:protein metabolic process; P:catabolic process; C:endoplasmic reticulum | IPR000668 (PFAM); IPR013128 (PANTHER); IPR025661 (PROSITE); G3DSA:3.90.70.10 (GENE3D), PTHR12411:SF14 (PANTHER), SignalP-NN(euk) (SIGNALP), tmhmm (TMHMM), SSF54001 (SUPERFAMILY) | Yes |
| 1210 | lib_dib_Filter15_IDBA_contig_22252_plus1 | 493 | cathepsin z | 460 | 2.30E-30 | 83.75% | 8 | P:anatomical structure morphogenesis; P:multicellular organismal development; C:extracellular space; C:lysosome; F:peptidase activity; P:protein metabolic process; P:catabolic process; C:endoplasmic reticulum | IPR000668 (PFAM); IPR013128 (PANTHER); IPR025661 (PROSITE); G3DSA:2.40.50.170 (GENE3D), PTHR12411:SF14 (PANTHER), SSF54001 (SUPERFAMILY) | |
| 1211 | lib_dib_Filter15_IDBA_contig_22259_plus1 | 77 | tyrosine-protein kinase | 460 | 8.58E-36 | 81.90% | 16 | P:cellular protein modification process; P:cell growth; C:cell; P:reproduction; P:signal transduction; F:nucleotide binding; P:cell differentiation; P:multicellular organismal development; F:protein binding; P:metabolic process; P:regulation of biological process; F:receptor activity; F:protein kinase activity; P:cell proliferation; P:ion transport; C:plasma membrane | IPR000719 (PROFILE); IPR001245 (PRINTS); IPR011009 (SUPERFAMILY); IPR020635 (SMART); G3DSA:1.10.510.10 (GENE3D), PTHR24416 (PANTHER), PTHR24416:SF4 (PANTHER), SignalP-NN(euk) (SIGNALP) | Yes |
| 1212 | lib_dib_Filter15_IDBA_contig_22260_plus1 | 221 | pre-mrna-processing factor 17 | 659 | 0 | 83.65% | 5 | F:transferase activity; P:cellular protein modification process; P:organelle organization; C:nucleus; F:protein binding | IPR001680 (PFAM); IPR015943 (G3DSA:2.130.10.GENE3D); IPR017986 (PROFILE); IPR019775 (PROSITE); PTHR19852 (PANTHER), tmhmm (TMHMM) | |
| 1213 | lib_dib_Filter15_IDBA_contig_22275_plus1 | 78 | uncharacterized aarf domain-containing protein kinase 1 isoform 1 | 460 | 4.34E-170 | 76.40% | 2 | P:metabolic process; F:protein kinase activity | IPR004147 (PFAM); IPR011009 (SUPERFAMILY); G3DSA:1.10.510.10 (GENE3D), PTHR10566 (PANTHER), PTHR10566:SF24 (PANTHER) | |

| | | | | | | | | | |
|---|---|---|---|---|---|---|---|---|---|
| 1214 | lib_dib_Filter15_IDBA_contig_22276_plus3 | 444 | nicotinic acetylcholine receptor subunit alpha6 variant | 1173 | 0 | 69.15% | 6 | F:ion channel activity; P:biological_process; C:protein complex; C:plasma membrane; P:ion transport; C:cell | IPR002394 (PRINTS); IPR006029 (PFAM); IPR006201 (PRINTS); IPR006202 (G3DSA:2.70.170.GENE3D); IPR018000 (PROSITE); G3DSA:1.20.120.370 (GENE3D), PTHR18945:SF70 (PANTHER), tmhmm (TMHMM) |
| 1215 | lib_dib_Filter15_IDBA_contig_22282_minus1 | 97 | f-box wd repeat-containing protein 2 | 460 | 9.15E-79 | 62.60% | 4 | C:cytoplasm; P:cellular protein modification process; C:nucleus; F:protein binding | IPR001680 (PFAM); IPR001810 (SMART); IPR015943 (G3DSA:2.130.10.GENE3D); IPR017986 (PROFILE); IPR019775 (PROSITE); IPR020472 (PRINTS); G3DSA:1.20.1280.50 (GENE3D), PTHR22837 (PANTHER), PTHR22837:SF0 (PANTHER), PF12937 (PFAM) |
| 1216 | lib_dib_Filter15_IDBA_contig_22293_plus1 | 123 | ficolin (collagen fibrinogen domain containing) 1 | 193 | 4.54E-21 | 57.80% | 2 | F:binding; P:biological_process | IPR002181 (PFAM); IPR014715 (G3DSA:4.10.530.GENE3D); IPR014716 (G3DSA:3.90.215.GENE3D); IPR020837 (PROSITE); PTHR19143 (PANTHER) |
| 1217 | lib_dib_Filter15_IDBA_contig_22293_plus2 | 123 | angiopoietin-related protein 1 | 192 | 2.95E-09 | 62.90% | 4 | F:receptor binding; P:signal transduction; C:extracellular space; P:transmembrane receptor protein tyrosine kinase signaling pathway | IPR002181 (PFAM); IPR014715 (G3DSA:4.10.530.GENE3D); PTHR19143 (PANTHER), PTHR19143:SF47 (PANTHER) |
| 1218 | lib_dib_Filter15_IDBA_contig_22294_plus1 | 231 | carcinolectin 5a isoform | 459 | 6.91E-10 | 60.35% | 3 | F:receptor binding; C:extracellular space; P:signal transduction | IPR002181 (PFAM); IPR014715 (G3DSA:4.10.530.GENE3D); IPR020837 (PROSITE); PTHR19143 (PANTHER), PTHR19143:SF15 (PANTHER) |
| 1219 | lib_dib_Filter15_IDBA_contig_223_plus2 | 3394 | 5 -nucleotidase isoform 1 | 206 | 1.41E-57 | 61.20% | 6 | P:nucleobase-containing compound metabolic process; P:catabolic process; P:DNA metabolic process; F:hydrolase activity; C:cytoplasm; C:plasma membrane | IPR006179 (PRINTS); IPR008334 (G3DSA:3.90.780.GENE3D); PTHR11575:SF7 (PANTHER) |
| 1220 | lib_dib_Filter15_IDBA_contig_22306_minus2 | 114 | membrane frizzled-related protein | 634 | 2.05E-47 | 47.94% | 1 | F:carbohydrate binding | IPR000859 (G3DSA:2.60.120.GENE3D); IPR000922 (PFAM); PTHR10127 (PANTHER), PTHR10127:SF345 (PANTHER), tmhmm (TMHMM) |
| 1221 | lib_dib_Filter15_IDBA_contig_22311_plus3 | 74 | tankyrase-1 isoform 2 | 458 | 0 | 80.25% | 21 | P:cellular protein modification process; P:transport; C:Golgi apparatus; F:protein binding; F:binding; C:nuclear chromosome; P:cytoskeleton organization; P:cell cycle; C:nuclear envelope; P:regulation of biological process; P:DNA metabolic process; P:organelle organization; P:biosynthetic process; C:cell; P:protein transport; P:biological_process; F:transferase activity; C:protein complex; C:chromosome; C:microtubule organizing center; | IPR001660 (SMART); IPR002110 (PRINTS); IPR013761 (G3DSA:1.10.150.GENE3D); IPR020683 (G3DSA:1.25.40.GENE3D); IPR021129 (PFAM); PTHR24180 (PANTHER), PTHR24180:SF3 (PANTHER), PF13637 (PFAM) |
| 1222 | lib_dib_Filter15_IDBA_contig_22319_minus3 | 215 | indian hedgehog | 458 | 3.66E-37 | 56.25% | 16 | P:signal transduction; P:anatomical structure morphogenesis; P:cell differentiation; P:regulation of biological process; P:multicellular organismal development; P:cell proliferation; P:biosynthetic process; P:metabolic process; P:cellular component organization; P:embryo development; P:growth; P:cell communication; F:protein binding; P:protein metabolic process; P:catabolic process; F:peptidase activity | IPR001657 (PRINTS); IPR001767 (PFAM); IPR003586 (SMART); IPR003587 (SMART); IPR006141 (PROFILE); G3DSA:2.170.16.10 (GENE3D), PTHR11889 (PANTHER), SSF51294 (SUPERFAMILY) |

| | | | | | | | | | |
|---|---|---|---|---|---|---|---|---|---|
| 1223 | lib_dib_Filter15_IDBA_contig_22362_plus3 | 297 | adam 17-like protease-like | 898 | 1.17E-146 | 53.50% | 4 | F:peptidase activity; P:protein metabolic process; P:catabolic process; F:binding | IPR001590 (PROFILE); IPR001762 (G3DSA:4.10.70.GENE3D); IPR002870 (PFAM); IPR024079 (G3DSA:3.40.390.GENE3D); PTHR11905 (PANTHER), PTHR11905:SF3 (PANTHER), PF13688 (PFAM), SSF55486 (SUPERFAMILY) |
| 1224 | lib_dib_Filter15_IDBA_contig_22398_minus1 | 77 | polypeptide n-acetylgalactosaminyltransferase 5 | 458 | 2.76E-34 | 56.85% | 6 | F:transporter activity; C:cell; P:biosynthetic process; P:cellular protein modification process; P:carbohydrate metabolic process; F:transferase activity | IPR001173 (PFAM); G3DSA:3.90.550.10 (GENE3D), PTHR11675 (PANTHER), tmhmm (TMHMM), SSF53448 (SUPERFAMILY) |
| 1225 | lib_dib_Filter15_IDBA_contig_22399_minus2 | 55 | polypeptide n-acetylgalactosaminyltransferase-like protein 1 | 207 | 5.63E-54 | 49.50% | 10 | F:carbohydrate binding; F:transferase activity; C:integral to membrane; P:carbohydrate metabolic process; C:membrane; C:Golgi apparatus; F:transferase activity, transferring glycosyl groups; C:Golgi membrane; P:protein glycosylation; F:polypeptide N-acetylgalactosaminyltransferase activity | IPR000772 (PFAM); G3DSA:2.80.10.50 (GENE3D), PTHR11675 (PANTHER) |
| 1226 | lib_dib_Filter15_IDBA_contig_22400_minus3 | 908 | matrix metalloproteinase-21 | 1222 | 1.79E-128 | 54.65% | 5 | F:peptidase activity; P:protein metabolic process; P:catabolic process; F:binding; C:extracellular region | IPR000585 (G3DSA:2.110.10.GENE3D); IPR001818 (PFAM); IPR002477 (SUPERFAMILY); IPR006026 (SMART); IPR018487 (PFAM); IPR021190 (PRINTS); IPR024079 (G3DSA:3.40.390.GENE3D); PTHR10201 (PANTHER), PTHR10201:SF14 (PANTHER), SSF55486 (SUPERFAMILY) |
| 1227 | lib_dib_Filter15_IDBA_contig_22405_minus3 | 223 | beta- -galactosyltransferase | 457 | 4.83E-33 | 59.25% | 11 | P:reproduction; P:anatomical structure morphogenesis; P:cell differentiation; P:multicellular organismal development; P:behavior; P:cellular component organization; P:carbohydrate metabolic process; P:biosynthetic process; P:lipid metabolic process; F:transferase activity; C:Golgi apparatus | IPR003859 (PRINTS); G3DSA:3.90.550.10 (GENE3D), PTHR19300:SF10 (PANTHER), PF13733 (PFAM), SignalP-NN(euk) (SIGNALP), tmhmm (TMHMM), SSF53448 (SUPERFAMILY) |
| 1228 | lib_dib_Filter15_IDBA_contig_22408_plus1 | 627 | tyrosine-protein kinase btk29a | 1369 | 0 | 56.00% | 4 | F:nucleotide binding; P:cellular protein modification process; F:protein binding; F:protein kinase activity | IPR000719 (PROFILE); IPR000980 (G3DSA:3.30.505.GENE3D); IPR001245 (PRINTS); IPR002290 (SMART); IPR008266 (PROSITE); IPR011009 (SUPERFAMILY); IPR020635 (SMART); G3DSA:1.10.510.10 (GENE3D), G3DSA:3.30.200.20 (GENE3D), PTHR24418 (PANTHER), SSF55550 (SUPERFAMILY) |
| 1229 | lib_dib_Filter15_IDBA_contig_22423_minus3 | 211 | succinate-semialdehyde mitochondrial | 539 | 0 | 77.65% | 14 | P:multicellular organismal development; F:nucleotide binding; P:catabolic process; ; P:metabolic process; C:cell; C:mitochondrion; P:lipid metabolic process; F:catalytic activity; P:generation of precursor metabolites and energy; P:cell-cell signaling; F:binding; P:carbohydrate metabolic process; P:cellular component organization | IPR010102 (TIGRFAMs); IPR015590 (PFAM); IPR016160 (PROSITE); IPR016161 (SUPERFAMILY); IPR016162 (G3DSA:3.40.605.GENE3D); IPR016163 (G3DSA:3.40.309.GENE3D); PTHR11699 (PANTHER), PTHR11699:SF49 (PANTHER), SignalP-NN(euk) (SIGNALP) |
| 1230 | lib_dib_Filter15_IDBA_contig_22426_plus3 | 73 | variable lymphocyte receptor c | 457 | 1.26E-05 | 43.25% | 3 | P:proteolysis; F:serine-type endopeptidase activity; F:catalytic activity | IPR000372 (SMART); G3DSA:3.80.10.10 (GENE3D), PTHR24365 (PANTHER), PF13855 (PFAM), SSF52058 (SUPERFAMILY) |
| 1231 | lib_dib_Filter15_IDBA_contig_22440_minus3 | 154 | patj homolog | 573 | 1.37E-168 | 61.00% | 1 | F:protein binding | IPR001478 (PFAM); G3DSA:2.30.42.10 (GENE3D), PTHR19964 (PANTHER), PTHR19964:SF6 (PANTHER) |

| | | | | | | | | | |
|---|---|---|---|---|---|---|---|---|---|
| 1232 | lib_dib_Filter15_IDBA_contig_22441_minus3 | 50 | multiple pdz domain protein | 346 | 1.41E-47 | 53.00% | 7 | C:organelle; C:intracellular; C:plasma membrane; F:protein binding; P:biological_process; P:cell communication; C:cytoplasm | IPR001478 (PFAM); IPR004172 (PROFILE); IPR015132 (PFAM); G3DSA:2.30.42.10 (GENE3D), PTHR19964 (PANTHER), PTHR19964:SF10 (PANTHER), SignalP-NN(euk) (SIGNALP), tmhmm (TMHMM), SSF101288 (SUPERFAMILY) | |
| 1233 | lib_dib_Filter15_IDBA_contig_22443_plus2 | 4147 | cubilin | 1513 | 5.45E-43 | 43.15% | 6 | C:cell; C:organelle; C:intracellular; F:binding; C:cytoplasm; P:metabolic process | IPR000859 (G3DSA:2.60.120.GENE3D); PTHR10127 (PANTHER), PTHR10127:SF345 (PANTHER), SignalP-NN(euk) (SIGNALP), tmhmm (TMHMM) | Yes |
| 1234 | lib_dib_Filter15_IDBA_contig_22446_plus3 | 57 | tolloid-like protein 2 | 269 | 4.89E-27 | 48.20% | 1 | F:peptidase activity | IPR000859 (G3DSA:2.60.120.GENE3D); PTHR10127 (PANTHER), PTHR10127:SF75 (PANTHER), SignalP-NN(euk) (SIGNALP) | Yes |
| 1235 | lib_dib_Filter15_IDBA_contig_2245_plus1 | 12877 | apolipophorins- partial | 287 | 1.29E-71 | 50.05% | 2 | P:lipid transport; F:lipid transporter activity | IPR014853 (PFAM) | |
| 1236 | lib_dib_Filter15_IDBA_contig_22453_minus2 | 532 | deleted in malignant brain tumors 1 | 487 | 5.53E-53 | 47.65% | 2 | F:receptor activity; C:cell | IPR000519 (G3DSA:4.10.110.GENE3D); IPR001190 (PRINTS); IPR017448 (SMART); IPR017957 (PROSITE); G3DSA:3.10.250.10 (GENE3D), PTHR19331 (PANTHER), PTHR19331:SF118 (PANTHER), SignalP-NN(euk) (SIGNALP) | Yes |
| 1237 | lib_dib_Filter15_IDBA_contig_22454_minus2 | 1005 | deleted in malignant brain tumors 1 | 772 | 6.91E-89 | 49.50% | 2 | F:receptor activity; C:cell | IPR000519 (G3DSA:4.10.110.GENE3D); IPR001190 (PRINTS); IPR017448 (SMART); IPR017957 (PROSITE); G3DSA:3.10.250.10 (GENE3D), PTHR19331 (PANTHER), PTHR19331:SF118 (PANTHER), SignalP-NN(euk) (SIGNALP) | Yes |
| 1238 | lib_dib_Filter15_IDBA_contig_2246_plus1 | 1903 | vitellogenin- partial | 124 | 1.82E-27 | 57.25% | 2 | F:transporter activity; P:transport | IPR001747 (PFAM); IPR011030 (G3DSA:1.25.10.GENE3D) | |
| 1239 | lib_dib_Filter15_IDBA_contig_22462_minus2 | 68 | ankyrin repeat-containing protein | 456 | 2.90E-23 | 51.50% | 1 | F:protein binding | IPR002110 (SMART); IPR020683 (G3DSA:1.25.40.GENE3D); PTHR24188 (PANTHER) | |
| 1240 | lib_dib_Filter15_IDBA_contig_22472_minus2 | 365 | proclotting enzyme | 497 | 1.17E-43 | 56.40% | 4 | F:protein binding; F:peptidase activity; P:protein metabolic process; P:catabolic process | IPR001254 (PFAM); IPR001314 (PRINTS); IPR009003 (SUPERFAMILY); IPR018114 (PROSITE); G3DSA:2.40.10.10 (GENE3D), PTHR24265 (PANTHER), SignalP-NN(euk) (SIGNALP), tmhmm (TMHMM) | Yes |
| 1241 | lib_dib_Filter15_IDBA_contig_22486_minus1 | 1437 | deleted in malignant brain tumors 1 | 583 | 2.21E-63 | 47.25% | 2 | F:receptor activity; C:cell | IPR001190 (PRINTS); IPR017448 (SMART); G3DSA:3.10.250.10 (GENE3D), PTHR19331 (PANTHER), PTHR19331:SF118 (PANTHER), SignalP-NN(euk) (SIGNALP), tmhmm (TMHMM) | |
| 1242 | lib_dib_Filter15_IDBA_contig_22487_minus3 | 2081 | neuroendocrine convertase 1 | 873 | 0 | 71.60% | 17 | C:cell; P:metabolic process; F:peptidase activity; C:cytoplasmic membrane-bounded vesicle; P:generation of precursor metabolites and energy; C:extracellular space; P:transport; P:regulation of biological process; P:cell-cell signaling; C:Golgi apparatus; P:multicellular organismal development; P:protein metabolic process; P:biological_process; P:biosynthetic process; P:response to endogenous stimulus; F:binding; P:catabolic process | IPR000209 (G3DSA:3.40.50.GENE3D); IPR002884 (PFAM); IPR008979 (SUPERFAMILY); IPR009020 (SUPERFAMILY); IPR015500 (PRINTS); IPR022398 (PROSITE); IPR023827 (PROSITE); IPR023828 (PROSITE); G3DSA:2.60.120.260 (GENE3D), G3DSA:3.30.70.850 (GENE3D), PTHR10795:SF12 (PANTHER) | |
| 1243 | lib_dib_Filter15_IDBA_contig_22498_plus3 | 507 | tolloid-like protein 1 | 722 | 2.44E-21 | 59.35% | 6 | P:multicellular organismal development; F:peptidase activity; F:binding; F:protein binding; P:protein metabolic process; P:catabolic process | IPR000859 (G3DSA:2.60.120.GENE3D); IPR002172 (PROFILE); G3DSA:1.20.1070.10 (GENE3D), PTHR10127 (PANTHER), tmhmm (TMHMM) | |

| | | | | | | | | | | |
|---|---|---|---|---|---|---|---|---|---|---|
| 1244 | lib_dib_Filter15_IDBA_contig_22516_plus1 | 419 | protein hook homolog 3 | 1055 | 0 | 77.00% | 15 | C:protein complex; C:cytoplasm; F:cytoskeletal protein binding; C:vacuole; P:cytoskeleton organization; P:cell proliferation; P:multicellular organismal development; P:cell differentiation; P:regulation of biological process; P:transport; F:protein binding; C:microtubule organizing center; P:biological_process; C:Golgi apparatus; P:organelle organization | IPR008636 (PFAM); PTHR18947 (PANTHER), PTHR18947:SF9 (PANTHER), SSF116907 (SUPERFAMILY) | |
| 1245 | lib_dib_Filter15_IDBA_contig_22521_plus3 | 138 | protein | 454 | 1.92E-25 | 47.85% | 1 | F:protein binding | IPR001611 (PROFILE); IPR003591 (SMART); G3DSA:3.80.10.10 (GENE3D), PTHR24365 (PANTHER), PF13504 (PFAM), PF13855 (PFAM), SignalP-NN(euk) (SIGNALP), tmhmm (TMHMM), SSF52047 (SUPERFAMILY) | Yes |
| 1246 | lib_dib_Filter15_IDBA_contig_22546_plus3 | 127 | leucine-rich repeats and immunoglobulin-like domains protein 2 | 454 | 3.35E-66 | 50.55% | 1 | F:protein binding | IPR000483 (SMART); IPR001611 (PROFILE); IPR003591 (SMART); IPR003961 (PROFILE); G3DSA:3.80.10.10 (GENE3D), PTHR24373 (PANTHER), PF13516 (PFAM), PF13855 (PFAM), SM00365 (SMART), tmhmm (TMHMM), SSF52058 (SUPERFAMILY) | |
| 1247 | lib_dib_Filter15_IDBA_contig_22547_plus3 | 19 | amphoterin-induced protein 1 | 145 | 2.96E-16 | 51.00% | 3 | P:regulation of biological process; C:cell; C:extracellular region | IPR000372 (SMART); G3DSA:3.80.10.10 (GENE3D), PTHR24369 (PANTHER), PTHR24369:SF12 (PANTHER), SSF52058 (SUPERFAMILY) | |
| 1248 | lib_dib_Filter15_IDBA_contig_22558_minus1 | 142 | interleukin-6 receptor subunit beta precursor | 609 | 4.19E-29 | 39.15% | 5 | P:signal transduction; C:cell; P:biological_process; F:receptor activity; F:protein binding | IPR003961 (PFAM); IPR013783 (G3DSA:2.60.40.GENE3D); PTHR19143 (PANTHER), tmhmm (TMHMM) | |
| 1249 | lib_dib_Filter15_IDBA_contig_22559_minus1 | 95 | cytokine receptor | 486 | 1.30E-29 | 38.95% | 2 | P:regulation of biological process; F:protein binding | IPR003961 (PFAM); IPR013783 (G3DSA:2.60.40.GENE3D); PTHR19143 (PANTHER), tmhmm (TMHMM) | |
| 1250 | lib_dib_Filter15_IDBA_contig_22560_plus1 | 612 | l-rhamnose-binding lectin csl3-like | 1052 | 3.41E-68 | 46.20% | 1 | F:carbohydrate binding | IPR000922 (PFAM); IPR003582 (PFAM); PTHR12011 (PANTHER), PTHR12011:SF4 (PANTHER), tmhmm (TMHMM) | |
| 1251 | lib_dib_Filter15_IDBA_contig_22569_plus2 | 58 | pp- c- | 453 | 0 | 78.75% | 4 | C:cell; F:transferase activity; F:carbohydrate binding; C:Golgi apparatus | IPR000772 (PFAM); IPR001173 (PFAM); G3DSA:2.80.10.50 (GENE3D), G3DSA:3.90.550.10 (GENE3D), PTHR11675 (PANTHER), SSF53448 (SUPERFAMILY) | |
| 1252 | lib_dib_Filter15_IDBA_contig_22615_minus3 | 375 | f-box-like wd repeat-containing protein tbl1xr1 | 714 | 0 | 87.85% | 4 | F:transferase activity; P:organelle organization; P:cellular protein modification process; F:protein binding | IPR001680 (PFAM); IPR006594 (SMART); IPR013720 (PFAM); IPR015943 (G3DSA:2.130.10.GENE3D); IPR017986 (PROFILE); IPR019775 (PROSITE); IPR020472 (PRINTS); PTHR22846 (PANTHER), tmhmm (TMHMM) | |
| 1253 | lib_dib_Filter15_IDBA_contig_22617_minus1 | 232 | alpha-galactosidase alpha-n-acetylgalactosaminidase | 453 | 7.02E-135 | 61.05% | 2 | F:hydrolase activity; P:carbohydrate metabolic process | IPR000111 (PFAM); IPR002241 (PRINTS); IPR013780 (G3DSA:2.60.40.GENE3D); IPR013785 (G3DSA:3.20.20.GENE3D); IPR017853 (SUPERFAMILY); PTHR11452 (PANTHER), SignalP-NN(euk) (SIGNALP), tmhmm (TMHMM), SSF51011 (SUPERFAMILY) | |

| | | | | | | | | | | |
|---|---|---|---|---|---|---|---|---|---|---|
| 1254 | lib_dib_Filter15_IDBA_contig_22621_plus1 | 149 | hepatocyte growth isoform cra_a | 453 | 2.25E-50 | 56.60% | 19 | C:plasma membrane; P:biological_process; P:cell differentiation; P:multicellular organismal development; F:receptor binding; C:cell; P:protein metabolic process; P:catabolic process; P:embryo development; F:peptidase activity; P:response to external stimulus; P:anatomical structure morphogenesis; P:response to stress; P:growth; P:cell proliferation; P:cellular homeostasis; P:regulation of biological process; P:cell death; C:extracellular region | IPR000001 (G3DSA:2.40.20.GENE3D); IPR013806 (SUPERFAMILY); IPR018056 (PROSITE); PR00018 (PRINTS), PTHR24272 (PANTHER), PTHR24272:SF2 (PANTHER), SignalP-NN(euk) (SIGNALP), tmhmm (TMHMM) | Yes |
| 1255 | lib_dib_Filter15_IDBA_contig_22630_minus2 | 726 | alpha beta hydrolase fold-1 domain-containing protein | 249 | 1.28E-05 | 54.00% | 6 | C:nucleolus; C:nucleus; F:nucleotide binding; P:cellular metabolic process; F:catalytic activity; F:coenzyme binding | no IPS match | |
| 1256 | lib_dib_Filter15_IDBA_contig_22632_minus1 | 482 | ---NA--- | 183 | | | 0 | - | no IPS match | |
| 1257 | lib_dib_Filter15_IDBA_contig_22648_plus1 | 11907 | protein-glutamine gamma-glutamyltransferase k-like | 1072 | 0 | 59.70% | 2 | P:cellular protein modification process; F:transferase activity | IPR001102 (PFAM); IPR002931 (PFAM); IPR008958 (PFAM); IPR013783 (G3DSA:2.60.40.GENE3D); IPR014756 (SUPERFAMILY); IPR023608 (PANTHER); G3DSA:3.90.260.10 (GENE3D), SSF54001 (SUPERFAMILY) | |
| 1258 | lib_dib_Filter15_IDBA_contig_22653_minus3 | 120 | tyrosine-protein kinase-like otk-like | 615 | 6.37E-143 | 54.20% | 2 | F:transferase activity; F:protein binding | IPR003598 (SMART); IPR003599 (SMART); IPR007110 (PROFILE); IPR013098 (PFAM); IPR013783 (G3DSA:2.60.40.GENE3D); PTHR10489 (PANTHER), tmhmm (TMHMM), SSF48726 (SUPERFAMILY) | |
| 1259 | lib_dib_Filter15_IDBA_contig_22659_minus3 | 47 | fras1-related extracellular matrix protein 1 | 451 | 2.08E-93 | 51.75% | 2 | P:biological_process; C:proteinaceous extracellular matrix | PTHR11878 (PANTHER), PTHR11878:SF15 (PANTHER) | |
| 1260 | lib_dib_Filter15_IDBA_contig_22672_minus1 | 359 | von willebrand factor type egf and pentraxin domain-containing protein 1-like | 173 | 1.68E-29 | 51.40% | 7 | F:calcium ion binding; C:integral to membrane; F:chitin binding; P:chitin metabolic process; C:extracellular region; F:carbohydrate binding; F:chromatin binding | IPR000436 (PFAM); G3DSA:2.10.70.10 (GENE3D), PTHR19325 (PANTHER) | |
| 1261 | lib_dib_Filter15_IDBA_contig_22673_minus1 | 3190 | von willebrand factor type egf and pentraxin domain-containing protein 1-like | 257 | 2.41E-56 | 47.30% | 5 | F:carbohydrate binding; P:regulation of transcription, DNA-dependent; F:sequence-specific DNA binding; F:sequence-specific DNA binding transcription factor activity; F:DNA binding | IPR000436 (PFAM); IPR011641 (PFAM); G3DSA:2.10.70.10 (GENE3D), PTHR19325 (PANTHER) | |
| 1262 | lib_dib_Filter15_IDBA_contig_22675_minus1 | 1691 | von willebrand factor type egf and pentraxin domain-containing protein 1 | 174 | 1.28E-22 | 50.10% | 3 | F:carbohydrate binding; F:calcium ion binding; F:chromatin binding | IPR000436 (PFAM); G3DSA:2.10.70.10 (GENE3D), PTHR19325 (PANTHER) | |
| 1263 | lib_dib_Filter15_IDBA_contig_22681_minus1 | 3071 | gcc2 and gcc3 | 271 | 1.09E-62 | 50.25% | 1 | F:protein binding | IPR000742 (SMART); IPR009030 (SUPERFAMILY); IPR011641 (PFAM); IPR013032 (PROSITE); G3DSA:2.10.25.10 (GENE3D), PTHR24838 (PANTHER), SSF57196 (SUPERFAMILY) | |
| 1264 | lib_dib_Filter15_IDBA_contig_22697_minus3 | 162 | fibrinogen gamma chain | 464 | 6.71E-44 | 53.25% | 12 | F:receptor binding; C:extracellular space; P:signal transduction; P:platelet activation; P:protein polymerization; C:external side of plasma membrane; C:fibrinogen complex; P:response to calcium ion; F:eukaryotic cell surface binding; C:cell cortex; C:platelet alpha granule; F:protein binding, bridging | IPR002181 (PFAM); IPR014715 (G3DSA:4.10.530.GENE3D); IPR014716 (G3DSA:3.90.215.GENE3D); PTHR19143 (PANTHER), SignalP-NN(euk) (SIGNALP), tmhmm (TMHMM) | |

| | | | | | | | | | |
|---|---|---|---|---|---|---|---|---|---|
| 1265 | lib_dib_Filter15_IDBA_contig_22716_plus3 | 329 | carboxypeptidase d-like | 1007 | 1.16E-119 | 48.65% | 5 | P:biological_process; F:peptidase activity; P:protein metabolic process; P:catabolic process; F:binding | IPR000834 (PRINTS); IPR008969 (SUPERFAMILY); IPR014766 (G3DSA:2.60.40.GENE3D); G3DSA:3.40.630.10 (GENE3D), PTHR11532 (PANTHER), PF13620 (PFAM), tmhmm (TMHMM), SSF53187 (SUPERFAMILY) | |
| 1266 | lib_dib_Filter15_IDBA_contig_22727_minus2 | 1677 | counting factor associated protein d-like | 766 | 0 | 68.40% | 6 | C:cytoskeleton; C:protein complex; C:lipid particle; F:peptidase activity; P:protein metabolic process; P:catabolic process | IPR000169 (PROSITE); IPR000668 (PRINTS); IPR013128 (PANTHER); IPR013201 (PFAM); IPR025660 (PROSITE); IPR025661 (PROSITE); G3DSA:2.40.50.170 (GENE3D), G3DSA:3.90.70.10 (GENE3D), PTHR12411:SF47 (PANTHER), SignalP-NN(euk) (SIGNALP), tmhmm (TMHMM), SSF54001 (SUPERFAMILY) | Yes |
| 1267 | lib_dib_Filter15_IDBA_contig_22728_minus2 | 1605 | counting factor associated protein d-like | 700 | 0 | 68.40% | 6 | C:cytoskeleton; C:protein complex; C:lipid particle; F:peptidase activity; P:protein metabolic process; P:catabolic process | IPR000169 (PROSITE); IPR000668 (PRINTS); IPR013128 (PANTHER); IPR013201 (PFAM); IPR025660 (PROSITE); IPR025661 (PROSITE); G3DSA:2.40.50.170 (GENE3D), G3DSA:3.90.70.10 (GENE3D), PTHR12411:SF47 (PANTHER), SignalP-NN(euk) (SIGNALP), tmhmm (TMHMM), SSF54001 (SUPERFAMILY) | Yes |
| 1268 | lib_dib_Filter15_IDBA_contig_22745_plus3 | 534 | ectonucleoside triphosphate diphosphohydrolase 1 isoform 1 | 537 | 1.12E-104 | 50.85% | 3 | P:biological_process; C:cell; F:hydrolase activity | IPR000407 (PANTHER); SignalP-NN(euk) (SIGNALP), tmhmm (TMHMM) | Yes |
| 1269 | lib_dib_Filter15_IDBA_contig_22751_plus2 | 167 | plasma kallikrein | 449 | 1.15E-55 | 49.95% | 10 | P:regulation of biological process; P:primary metabolic process; P:protein metabolic process; P:metabolic process; C:extracellular region; P:response to external stimulus; P:response to stress; P:biological_process; F:peptidase activity; P:catabolic process | IPR001254 (PFAM); IPR001314 (PRINTS); IPR009003 (SUPERFAMILY); IPR018114 (PROSITE); G3DSA:2.40.10.10 (GENE3D), PTHR24268 (PANTHER) | |
| 1270 | lib_dib_Filter15_IDBA_contig_22781_plus3 | 1665 | calnexin | 448 | 0 | 77.25% | 17 | C:cytoplasm; C:ribosome; P:biological_process; P:protein transport; C:cell; F:calcium ion binding; P:biosynthetic process; P:cellular protein modification process; P:carbohydrate metabolic process; P:protein metabolic process; F:receptor binding; C:cytoplasmic membrane-bounded vesicle; F:protein binding; C:endoplasmic reticulum; P:transport; P:cellular component organization; F:carbohydrate binding | IPR001580 (PRINTS); IPR008985 (SUPERFAMILY); IPR009033 (G3DSA:2.10.250.GENE3D); IPR013320 (G3DSA:2.60.120.GENE3D); IPR018124 (PROSITE); PTHR11073:SF1 (PANTHER), SignalP-NN(euk) (SIGNALP), tmhmm (TMHMM) | Yes |
| 1271 | lib_dib_Filter15_IDBA_contig_22796_plus3 | 1448 | lysosomal pro-x carboxypeptidase | 886 | 0 | 72.45% | 7 | F:peptidase activity; P:response to external stimulus; P:response to stress; C:plasma membrane; F:protein binding; P:protein metabolic process; P:catabolic process | IPR008758 (PANTHER); G3DSA:3.40.50.1820 (GENE3D), PTHR11010:SF9 (PANTHER), SignalP-NN(euk) (SIGNALP), tmhmm (TMHMM), SSF53474 (SUPERFAMILY) | |
| 1272 | lib_dib_Filter15_IDBA_contig_22799_plus1 | 29 | dipeptidyl-peptidase family protein | 105 | 2.93E-31 | 77.65% | 3 | F:peptidase activity; P:protein metabolic process; P:catabolic process | IPR008758 (PANTHER); PTHR11010:SF10 (PANTHER) | |
| 1273 | lib_dib_Filter15_IDBA_contig_22806_minus1 | 298 | ankyrin repeat domain-containing protein 50-like | 855 | 2.70E-39 | 44.75% | 1 | F:protein binding | IPR002110 (SMART); IPR020683 (G3DSA:1.25.40.GENE3D); PTHR24198 (PANTHER) | |
| 1274 | lib_dib_Filter15_IDBA_contig_22807_plus2 | 166 | ubiquitin | 448 | 3.48E-17 | 67.10% | 1 | F:protein binding | IPR000626 (PFAM); IPR019955 (PROFILE); IPR019956 (PRINTS); G3DSA:3.10.20.90 (GENE3D), PTHR10666 (PANTHER), PTHR10666:SF2 (PANTHER), SSF54236 (SUPERFAMILY) | |

| | | | | | | | | | |
|---|---|---|---|---|---|---|---|---|---|
| 1275 | lib_dib_Filter15_IDBA_contig_22858_minus3 | 134 | probable trna threonylcarbamoyladenosine biosynthesis protein osgep | 478 | 0 | 89.55% | 5 | F:binding; F:peptidase activity; P:protein metabolic process; P:catabolic process; P:nucleobase-containing compound metabolic process | IPR000905 (PFAM); IPR017860 (PROSITE); IPR017861 (PRINTS); G3DSA:3.30.420.40 (GENE3D), PTHR11735 (PANTHER), SignalP-NN(euk) (SIGNALP), SSF53067 (SUPERFAMILY) | Yes |
| 1276 | lib_dib_Filter15_IDBA_contig_22867_minus3 | 1776 | protein | 1179 | 6.81E-157 | 56.95% | 3 | F:peptidase activity; P:protein metabolic process; P:catabolic process | IPR000209 (G3DSA:3.40.50.GENE3D); IPR002884 (PFAM); IPR008979 (SUPERFAMILY); IPR009020 (SUPERFAMILY); IPR015500 (PRINTS); IPR022398 (PROSITE); IPR023827 (PROSITE); IPR023828 (PROSITE); G3DSA:2.60.120.260 (GENE3D), G3DSA:3.30.70.850 (GENE3D), PTHR10795:SF16 (PANTHER), SignalP-NN(euk) (SIGNALP), tmhmm (TMHMM) | Yes |
| 1277 | lib_dib_Filter15_IDBA_contig_22868_minus3 | 1819 | protein | 1247 | 1.59E-156 | 56.85% | 3 | F:peptidase activity; P:protein metabolic process; P:catabolic process | IPR000209 (G3DSA:3.40.50.GENE3D); IPR002884 (PFAM); IPR008979 (SUPERFAMILY); IPR009020 (SUPERFAMILY); IPR015500 (PRINTS); IPR022398 (PROSITE); IPR023827 (PROSITE); IPR023828 (PROSITE); G3DSA:2.60.120.260 (GENE3D), G3DSA:3.30.70.850 (GENE3D), PTHR10795:SF16 (PANTHER), SignalP-NN(euk) (SIGNALP), tmhmm (TMHMM) | Yes |
| 1278 | lib_dib_Filter15_IDBA_contig_22888_plus2 | 66 | calcium calmodulin-dependent protein kinase type 1 | 446 | 6.44E-177 | 80.30% | 3 | F:protein kinase activity; P:cellular protein modification process; F:nucleotide binding | IPR000719 (PFAM); IPR002290 (SMART); IPR008271 (PROSITE); IPR011009 (SUPERFAMILY); IPR017441 (PROSITE); IPR020635 (SMART); IPR020636 (PANTHER); G3DSA:1.10.510.10 (GENE3D), G3DSA:3.30.200.20 (GENE3D), PTHR24347:SF17 (PANTHER) | |
| 1279 | lib_dib_Filter15_IDBA_contig_22943_minus1 | 126 | zinc finger protein 229 | 589 | 8.12E-21 | 45.15% | 3 | F:binding; F:nucleic acid binding; C:intracellular | IPR007087 (PROSITE); IPR013087 (G3DSA:3.30.160.GENE3D); IPR015880 (SMART); PTHR11389 (PANTHER), SignalP-NN(euk) (SIGNALP), SSF57667 (SUPERFAMILY) | |
| 1280 | lib_dib_Filter15_IDBA_contig_22946_plus3 | 277 | tyrosine-protein phosphatase 10d | 1120 | 0 | 53.00% | 6 | P:biological_process; P:cellular protein modification process; F:phosphoprotein phosphatase activity; P:multicellular organismal development; C:cell; F:protein binding | IPR000242 (PRINTS); IPR000387 (PROFILE); IPR003595 (SMART); IPR003961 (PFAM); IPR013783 (G3DSA:2.60.40.GENE3D); IPR016130 (PROSITE); G3DSA:3.90.190.10 (GENE3D), PTHR19134 (PANTHER), PTHR19134:SF75 (PANTHER), tmhmm (TMHMM), SSF52799 (SUPERFAMILY) | |
| 1281 | lib_dib_Filter15_IDBA_contig_22948_plus1 | 10983 | methylmalonate-semialdehyde dehydrogenase | 728 | 0 | 83.55% | 6 | F:catalytic activity; F:lipid binding; P:nucleobase-containing compound metabolic process; ; C:mitochondrion; P:metabolic process | IPR010061 (PTHR11699:PANTHER); IPR015590 (PFAM); IPR016160 (PROSITE); IPR016161 (SUPERFAMILY); IPR016162 (G3DSA:3.40.605.GENE3D); IPR016163 (G3DSA:3.40.309.GENE3D); PTHR11699 (PANTHER), tmhmm (TMHMM) | |
| 1282 | lib_dib_Filter15_IDBA_contig_22949_minus3 | 9790 | blastula protease 10 | 1058 | 9.72E-57 | 48.45% | 6 | F:protein binding; C:cell; F:peptidase activity; P:protein metabolic process; P:catabolic process; F:binding | IPR000742 (PFAM); IPR000859 (G3DSA:2.60.120.GENE3D); IPR000884 (PFAM); IPR000998 (PFAM); IPR001506 (PRINTS); IPR006026 (SMART); IPR008985 (SUPERFAMILY); IPR013032 (PROSITE); IPR024079 (G3DSA:3.40.390.GENE3D); G3DSA:2.10.25.10 (GENE3D), G3DSA:2.20.100.10 (GENE3D), PTHR10127 (PANTHER), PTHR10127:SF334 (PANTHER), SSF55486 (SUPERFAMILY), SSF57196 (SUPERFAMILY) | |

| | | | | | | | | | |
|---|---|---|---|---|---|---|---|---|---|
| 1283 | lib_dib_Filter15_IDBA_contig_22951_minus1 | 95 | beta-hexosaminidase | 445 | 3.97E-105 | 56.70% | 2 | P:carbohydrate metabolic process; F:hydrolase activity | IPR013781 (G3DSA:3.20.20.GENE3D); IPR015883 (PFAM); IPR017853 (SUPERFAMILY); IPR025705 (PRINTS); G3DSA:3.30.379.10 (GENE3D), PTHR22600 (PANTHER), SSF55545 (SUPERFAMILY) |
| 1284 | lib_dib_Filter15_IDBA_contig_22955_plus1 | 87 | af397902_1egf-like protein | 445 | 3.78E-127 | 57.75% | 3 | C:extracellular region; F:protein binding; F:calcium ion binding | IPR000152 (PROSITE); IPR000742 (PFAM); IPR001881 (SMART); IPR013032 (PFAM); IPR018097 (PROSITE); PR00010 (PRINTS), PR01983 (PRINTS), G3DSA:2.10.25.10 (GENE3D), G3DSA:2.170.300.10 (GENE3D), PTHR24048 (PANTHER), PTHR24048:SF15 (PANTHER), SSF57196 (SUPERFAMILY) |
| 1285 | lib_dib_Filter15_IDBA_contig_22960_minus2 | 91 | neuropilin and tolloid -like | 445 | 7.91E-12 | 51.15% | 16 | P:anatomical structure morphogenesis; P:cellular component organization; P:cell differentiation; P:multicellular organismal development; P:metabolic process; P:regulation of biological process; C:lysosome; P:protein transport; F:hydrolase activity; C:cytoplasmic membrane-bounded vesicle; F:protein binding; C:organelle; C:cell; C:vacuole; C:plasma membrane; P:protein metabolic process | IPR000859 (G3DSA:2.60.120.GENE3D); PTHR10127 (PANTHER), SignalP-NN(euk) (SIGNALP), tmhmm (TMHMM) |
| 1286 | lib_dib_Filter15_IDBA_contig_22968_minus3 | 259 | achain nmr structure of the mouse mfg-e8 c2 domain | 898 | 2.49E-23 | 58.60% | 5 | P:transport; F:binding; C:extracellular region; C:cell; P:biological_process | IPR000421 (PFAM); IPR008979 (SUPERFAMILY); G3DSA:2.60.120.260 (GENE3D), PTHR24543 (PANTHER), PTHR24543:SF27 (PANTHER), tmhmm (TMHMM) |
| 1287 | lib_dib_Filter15_IDBA_contig_22969_minus3 | 150 | achain nmr structure of the mouse mfg-e8 c2 domain | 505 | 1.51E-25 | 58.40% | 5 | P:transport; F:binding; C:cell; C:extracellular region; P:biological_process | IPR000421 (PFAM); IPR008979 (SUPERFAMILY); G3DSA:2.60.120.260 (GENE3D), PTHR24543 (PANTHER), PTHR24543:SF27 (PANTHER), tmhmm (TMHMM) |
| 1288 | lib_dib_Filter15_IDBA_contig_22984_minus2 | 126 | protocadherin fat 4 | 445 | 2.15E-130 | 48.35% | 3 | F:calcium ion binding; P:biological_process; C:cell | IPR002126 (PRINTS); IPR015919 (SUPERFAMILY); PTHR24027 (PANTHER) |
| 1289 | lib_dib_Filter15_IDBA_contig_23013_plus1 | 6700 | phosphoglycerate kinase 1 | 676 | 0 | 85.90% | 9 | C:cell; P:biosynthetic process; P:carbohydrate metabolic process; F:nucleotide binding; F:kinase activity; P:metabolic process; P:generation of precursor metabolites and energy; P:catabolic process; C:cytosol | IPR001576 (PRINTS); IPR015824 (G3DSA:3.40.50.GENE3D); IPR015901 (G3DSA:3.40.50.GENE3D); IPR015911 (PROSITE); tmhmm (TMHMM) |
| 1290 | lib_dib_Filter15_IDBA_contig_23021_minus1 | 107 | alcohol dehydrogenase | 444 | 1.30E-118 | 71.25% | 2 | F:catalytic activity; P:metabolic process | IPR001395 (PANTHER); IPR018170 (PROSITE); IPR020471 (PRINTS); IPR023210 (G3DSA:3.20.20.GENE3D); PTHR11732:SF34 (PANTHER) |
| 1291 | lib_dib_Filter15_IDBA_contig_23025_minus3 | 76 | alpha-mannosidase 2 | 443 | 1.92E-43 | 54.70% | 3 | F:hydrolase activity; F:carbohydrate binding; P:carbohydrate metabolic process | IPR011013 (SUPERFAMILY); IPR011682 (PFAM); G3DSA:2.70.98.30 (GENE3D), PTHR11607 (PANTHER), PTHR11607:SF4 (PANTHER) |

| | | | | | | | | | |
|---|---|---|---|---|---|---|---|---|---|
| 1292 | lib_dib_Filter15_IDBA_contig_23028_plus1 | 222 | lysine-specific histone demethylase 1a-like | 744 | 0 | 87.35% | 18 | F:nucleotide binding; P:regulation of biological process; ; P:organelle organization; P:cellular protein modification process; F:transferase activity; F:protein binding; P:multicellular organismal development; F:catalytic activity; C:nucleoplasm; C:protein complex; F:DNA binding; P:cell differentiation; P:embryo development; F:sequence-specific DNA binding transcription factor activity; C:nuclear chromosome; P:metabolic process; P:biosynthetic process | IPR002937 (PFAM); IPR007526 (PFAM); IPR009057 (SUPERFAMILY); IPR011991 (G3DSA:1.10.10.GENE3D); IPR017366 (PIR); G3DSA:1.10.287.80 (GENE3D), G3DSA:3.50.50.60 (GENE3D), PTHR10742 (PANTHER), PTHR10742:SF35 (PANTHER), SSF51905 (SUPERFAMILY), SSF54373 (SUPERFAMILY) |
| 1293 | lib_dib_Filter15_IDBA_contig_23060_plus3 | 107 | low-density lipoprotein | 442 | 8.11E-52 | 53.45% | 1 | F:protein binding | IPR002172 (G3DSA:4.10.400.GENE3D); IPR023415 (PROSITE); PTHR20967 (PANTHER), tmhmm (TMHMM) |
| 1294 | lib_dib_Filter15_IDBA_contig_23075_minus1 | 121 | ankyrin repeat | 521 | 2.28E-39 | 51.60% | 1 | F:protein binding | IPR001496 (PFAM); IPR002110 (PRINTS); IPR020683 (G3DSA:1.25.40.GENE3D); PTHR24188 (PANTHER), PF13637 (PFAM) |
| 1295 | lib_dib_Filter15_IDBA_contig_23120_minus2 | 111 | acid phosphatase-like protein 2 | 516 | 9.43E-75 | 49.50% | 1 | F:hydrolase activity | IPR000560 (PFAM); G3DSA:3.40.50.1240 (GENE3D), PTHR11567 (PANTHER), PTHR11567:SF12 (PANTHER), SignalP-NN(euk) (SIGNALP), tmhmm (TMHMM), SSF53254 (SUPERFAMILY) |
| 1296 | lib_dib_Filter15_IDBA_contig_23122_minus2 | 58 | retinoid-inducible serine carboxypeptidase | 442 | 4.49E-55 | 49.10% | 3 | F:peptidase activity; P:protein metabolic process; P:catabolic process | IPR001563 (PRINTS); G3DSA:3.40.50.1820 (GENE3D), PTHR11802:SF3 (PANTHER), SSF53474 (SUPERFAMILY) |
| 1297 | lib_dib_Filter15_IDBA_contig_23129_plus3 | 572 | carboxypeptidase e | 706 | 1.03E-155 | 65.60% | 10 | F:protein binding; P:protein metabolic process; C:cell; F:binding; P:catabolic process; C:extracellular space; C:cytoplasmic membrane-bounded vesicle; C:Golgi apparatus; P:embryo development; F:peptidase activity | IPR000834 (PRINTS); IPR008969 (SUPERFAMILY); IPR014766 (G3DSA:2.60.40.GENE3D); G3DSA:3.40.630.10 (GENE3D), PTHR11532 (PANTHER), PF13620 (PFAM), SignalP-NN(euk) (SIGNALP), tmhmm (TMHMM), SSF53187 (SUPERFAMILY) |
| 1298 | lib_dib_Filter15_IDBA_contig_23153_plus2 | 1289 | cathepsin l-like | 441 | 3.52E-123 | 68.05% | 3 | P:protein metabolic process; P:catabolic process; F:peptidase activity | IPR000169 (PROSITE); IPR000668 (PRINTS); IPR013128 (PANTHER); IPR013201 (PFAM); IPR025660 (PROSITE); IPR025661 (PROSITE); G3DSA:3.90.70.10 (GENE3D), PTHR12411:SF149 (PANTHER), SSF54001 (SUPERFAMILY) |
| 1299 | lib_dib_Filter15_IDBA_contig_23168_plus2 | 119 | protein | 483 | 1.61E-15 | 55.80% | 8 | P:biological_process; P:multicellular organismal development; P:regulation of biological process; P:anatomical structure morphogenesis; C:nucleoplasm; C:cell; F:protein binding; F:calcium ion binding | IPR000152 (PROSITE); IPR000742 (PFAM); IPR001881 (SMART); IPR002172 (G3DSA:4.10.400.GENE3D); IPR013032 (PROSITE); G3DSA:2.10.25.10 (GENE3D), PTHR24838 (PANTHER), PTHR24838:SF112 (PANTHER), SSF57196 (SUPERFAMILY) |
| 1300 | lib_dib_Filter15_IDBA_contig_23171_plus3 | 109 | ankyrin repeat protein | 439 | 1.68E-80 | 54.20% | 1 | F:protein binding | IPR002110 (PRINTS); IPR020683 (G3DSA:1.25.40.GENE3D); PTHR24188 (PANTHER) |
| 1301 | lib_dib_Filter15_IDBA_contig_23173_minus1 | 86 | protein cbfa2t3 | 440 | 5.84E-124 | 69.15% | 9 | C:nucleus; ; P:regulation of biological process; P:generation of precursor metabolites and energy; F:DNA binding; P:biological_process; F:protein binding; P:cell differentiation; F:binding | IPR002893 (PFAM); IPR013289 (PRINTS); IPR014896 (PFAM); PTHR10379 (PANTHER), SSF144232 (SUPERFAMILY) |
| 1302 | lib_dib_Filter15_IDBA_contig_23203_minus3 | 117 | tubby protein homolog | 438 | 1.02E-145 | 86.15% | 8 | C:cytoplasm; P:biological_process; P:transport; P:cellular component organization; P:regulation of biological process; C:plasma membrane; C:extracellular region; C:nucleus | IPR000007 (PRINTS); IPR005398 (PRINTS); IPR025659 (SUPERFAMILY); PTHR16517 (PANTHER) |

| | | | | | | | | | |
|---|---|---|---|---|---|---|---|---|---|
| 1303 | lib_dib_Filter15_IDBA_contig_23259_plus2 | 55 | oocyte zinc finger protein 6-like | 437 | 5.26E-75 | 67.15% | 3 | F:binding; F:nucleic acid binding; C:intracellular | IPR007087 (PROSITE); IPR013087 (G3DSA:3.30.160.GENE3D); IPR015880 (SMART); PTHR24387 (PANTHER), PTHR24387:SF0 (PANTHER), PF13465 (PFAM), PF13912 (PFAM), SSF57667 (SUPERFAMILY) |
| 1304 | lib_dib_Filter15_IDBA_contig_23269_minus2 | 42 | zinc finger and btb domain-containing protein 41 | 437 | 1.74E-55 | 45.80% | 3 | F:binding; F:nucleic acid binding; C:intracellular | IPR007087 (PFAM); IPR013087 (G3DSA:3.30.160.GENE3D); IPR015880 (SMART); PTHR11389 (PANTHER), PF13894 (PFAM), SSF57667 (SUPERFAMILY) |
| 1305 | lib_dib_Filter15_IDBA_contig_23270_plus2 | 840 | leucine rich repeat protein | 1176 | 2.49E-46 | 53.65% | 1 | F:protein binding | IPR001611 (PFAM); IPR003591 (SMART); IPR025875 (PFAM); G3DSA:3.80.10.10 (GENE3D), PTHR23155 (PANTHER), PF13855 (PFAM), SM00364 (SMART), SSF52058 (SUPERFAMILY) |
| 1306 | lib_dib_Filter15_IDBA_contig_2328_plus2 | 327 | notchless protein homolog 1 | 618 | 0 | 86.00% | 6 | C:nucleolus; P:signal transduction; P:anatomical structure morphogenesis; P:cell differentiation; P:embryo development; F:protein binding | IPR001632 (PRINTS); IPR001680 (PFAM); IPR012972 (PFAM); IPR015943 (G3DSA:2.130.10.GENE3D); IPR017986 (PROFILE); IPR019775 (PROSITE); IPR020472 (PRINTS); PTHR19848 (PANTHER), PTHR19848:SF0 (PANTHER) |
| 1307 | lib_dib_Filter15_IDBA_contig_23315_plus2 | 3566 | stress-70 mitochondrial | 793 | 0 | 91.75% | 8 | P:protein metabolic process; C:nucleolus; P:protein transport; C:mitochondrion; C:cell; F:protein binding; F:nucleotide binding; P:response to stress | IPR012725 (TIGRFAMs); IPR013126 (PRINTS); IPR018181 (PROSITE); G3DSA:1.20.1270.10 (GENE3D), G3DSA:2.60.34.10 (GENE3D), G3DSA:3.30.30.30 (GENE3D), G3DSA:3.30.420.40 (GENE3D), G3DSA:3.90.640.10 (GENE3D), PTHR19375 (PANTHER), PTHR19375:SF1 (PANTHER), SSF100920 (SUPERFAMILY), SSF100934 (SUPERFAMILY), SSF53067 (SUPERFAMILY) |
| 1308 | lib_dib_Filter15_IDBA_contig_23318_minus1 | 265 | cadherin egf lag seven-pass g-type receptor 1 | 812 | 2.11E-94 | 63.70% | 8 | P:cell differentiation; P:multicellular organismal development; P:biological_process; P:anatomical structure morphogenesis; P:embryo development; P:signal transduction; F:receptor activity; C:cell | IPR000203 (PFAM); IPR000832 (PRINTS); IPR017981 (PROFILE); PTHR24026 (PANTHER), PTHR24026:SF1 (PANTHER), tmhmm (TMHMM) |
| 1309 | lib_dib_Filter15_IDBA_contig_23319_minus2 | 24 | cadherin egf lag seven-pass g-type receptor 2 | 103 | 6.70E-15 | 59.15% | 5 | P:cell differentiation; P:multicellular organismal development; P:biological_process; C:cell; P:signal transduction | IPR000203 (PFAM); PTHR24026 (PANTHER) |
| 1310 | lib_dib_Filter15_IDBA_contig_23323_minus3 | 71 | protocadherin fat 4 | 435 | 5.67E-128 | 51.00% | 4 | P:multicellular organismal development; F:calcium ion binding; P:biological_process; C:cell | IPR002126 (PRINTS); IPR015919 (SUPERFAMILY); PTHR24027 (PANTHER) |
| 1311 | lib_dib_Filter15_IDBA_contig_23360_plus2 | 70 | protein sma- isoform a | 435 | 1.37E-23 | 48.60% | 3 | P:biological_process; P:regulation of biological process; F:protein binding | IPR001611 (PFAM); IPR003591 (SMART); IPR025875 (PFAM); PR00019 (PRINTS), G3DSA:3.80.10.10 (GENE3D), PTHR24365 (PANTHER), PF13855 (PFAM), SSF52058 (SUPERFAMILY) |
| 1312 | lib_dib_Filter15_IDBA_contig_23403_minus3 | 46 | epidermis-type lipoxygenase 3-like | 113 | 4.06E-32 | 69.10% | 5 | F:binding; P:lipid metabolic process; F:catalytic activity; C:nuclear envelope; P:metabolic process | IPR000907 (PANTHER); IPR013819 (PRINTS); IPR020834 (PROSITE); G3DSA:1.20.245.10 (GENE3D) |
| 1313 | lib_dib_Filter15_IDBA_contig_23407_plus2 | 161 | wd sam and u-box domain-containing protein 1 | 779 | 1.20E-135 | 59.15% | 5 | F:protein binding; C:protein complex; C:intracellular; F:catalytic activity; P:cellular protein modification process | IPR001660 (SMART); IPR001680 (PFAM); IPR003613 (PFAM); IPR013083 (G3DSA:3.30.40.GENE3D); IPR013761 (G3DSA:1.10.150.GENE3D); IPR015943 (G3DSA:2.130.10.GENE3D); IPR017986 (PROFILE); IPR019775 (PROSITE); IPR020472 (PRINTS); IPR021129 (PFAM); PTHR22849 (PANTHER), SSF57850 (SUPERFAMILY) |

| | | | | | | | | | | |
|---|---|---|---|---|---|---|---|---|---|---|
| 1314 | lib_dib_Filter15_IDBA_contig_23413_plus1 | 1505 | beta-hexosaminidase subunit beta-like | 1301 | 0 | 67.95% | 22 | P:multicellular organismal development; C:cytoplasmic membrane-bounded vesicle; P:catabolic process; P:lipid metabolic process; P:biological_process; P:behavior; P:anatomical structure morphogenesis; P:cellular component organization; P:regulation of biological process; C:cell; P:cellular homeostasis; P:organelle organization; F:hydrolase activity; P:protein metabolic process; C:lysosome; P:reproduction; P:carbohydrate metabolic process; ; F:protein binding; P:cell communication; P:biosynthetic process; P:cell differentiation | IPR013781 (G3DSA:3.20.20.GENE3D); IPR015882 (PFAM); IPR015883 (PFAM); IPR017853 (SUPERFAMILY); IPR025705 (PRINTS); G3DSA:3.30.379.10 (GENE3D), PTHR22600 (PANTHER), SignalP-NN(euk) (SIGNALP), tmhmm (TMHMM), SSF55545 (SUPERFAMILY) | Yes |
| 1315 | lib_dib_Filter15_IDBA_contig_23419_minus3 | 1329 | trypsin-1-like isoform 1 | 816 | 2.73E-60 | 59.55% | 3 | F:peptidase activity; P:protein metabolic process; P:catabolic process | IPR001254 (PFAM); IPR001314 (PRINTS); IPR009003 (SUPERFAMILY); IPR018114 (PROSITE); G3DSA:2.40.10.10 (GENE3D), PTHR24264 (PANTHER), SignalP-NN(euk) (SIGNALP) | Yes |
| 1316 | lib_dib_Filter15_IDBA_contig_23446_minus2 | 724 | annulin-like isoform 1 | 512 | 1.11E-140 | 62.55% | 1 | P:cellular protein modification process | IPR001102 (PFAM); IPR002931 (PFAM); IPR013783 (G3DSA:2.60.40.GENE3D); IPR014756 (SUPERFAMILY); IPR023608 (PANTHER); G3DSA:3.90.260.10 (GENE3D), SSF54001 (SUPERFAMILY) | |
| 1317 | lib_dib_Filter15_IDBA_contig_23447_minus2 | 1221 | protein-glutamine gamma-glutamyltransferase k | 719 | 1.18E-144 | 56.25% | 2 | P:cellular protein modification process; F:transferase activity | IPR001102 (PFAM); IPR002931 (PFAM); IPR008958 (PFAM); IPR013783 (G3DSA:2.60.40.GENE3D); IPR014756 (SUPERFAMILY); IPR023608 (PIR); G3DSA:3.90.260.10 (GENE3D), SSF54001 (SUPERFAMILY) | |
| 1318 | lib_dib_Filter15_IDBA_contig_23448_minus2 | 1752 | protein-glutamine gamma-glutamyltransferase k | 998 | 2.03E-154 | 54.30% | 2 | P:cellular protein modification process; F:transferase activity | IPR001102 (PFAM); IPR002931 (PFAM); IPR008958 (PFAM); IPR013783 (G3DSA:2.60.40.GENE3D); IPR014756 (SUPERFAMILY); IPR023608 (PANTHER); G3DSA:3.90.260.10 (GENE3D), tmhmm (TMHMM), SSF54001 (SUPERFAMILY) | |
| 1319 | lib_dib_Filter15_IDBA_contig_2346_minus1 | 306 | peroxiredoxin 4 | 338 | 2.67E-131 | 88.80% | 4 | F:antioxidant activity; F:catalytic activity; C:mitochondrion; P:metabolic process | IPR000866 (PFAM); IPR012336 (G3DSA:3.40.30.GENE3D); IPR019479 (PFAM); PTHR10681 (PANTHER), PTHR10681:SF46 (PANTHER), SignalP-NN(euk) (SIGNALP), tmhmm (TMHMM) | Yes |
| 1320 | lib_dib_Filter15_IDBA_contig_23466_plus1 | 70 | n-(5-amino-5-carboxypentanoyl)-l-cysteinyl-d valine synthase | 432 | 2.66E-127 | 66.05% | 3 | P:metabolic process; F:catalytic activity; F:nucleotide binding | IPR002198 (PRINTS); IPR002347 (PRINTS); IPR016040 (G3DSA:3.40.50.GENE3D); IPR020842 (SMART); IPR020904 (PROSITE); PTHR24322 (PANTHER), PTHR24322:SF7 (PANTHER), SSF51735 (SUPERFAMILY) | |
| 1321 | lib_dib_Filter15_IDBA_contig_23467_minus1 | 1109 | fibrinogen c domain-containing protein 1-like | 809 | 1.64E-39 | 55.90% | 3 | F:receptor binding; P:signal transduction; C:extracellular space | IPR002181 (PFAM); IPR014715 (G3DSA:4.10.530.GENE3D); IPR014716 (G3DSA:3.90.215.GENE3D); PTHR19143 (PANTHER), SignalP-NN(euk) (SIGNALP), tmhmm (TMHMM) | Yes |
| 1322 | lib_dib_Filter15_IDBA_contig_23468_minus1 | 776 | fibrinogen c domain-containing protein 1-like | 306 | 1.09E-45 | 56.45% | 3 | F:receptor binding; P:signal transduction; C:extracellular space | IPR002181 (PFAM); IPR014715 (G3DSA:4.10.530.GENE3D); IPR014716 (G3DSA:3.90.215.GENE3D); PTHR19143 (PANTHER), SignalP-NN(euk) (SIGNALP), tmhmm (TMHMM) | Yes |

| | | | | | | | | | |
|---|---|---|---|---|---|---|---|---|---|
| 1323 | lib_dib_Filter15_IDBA_contig_23469_minus1 | 246 | angiopoietin-related protein 4 | 139 | 2.50E-12 | 62.00% | 4 | P:regulation of biological process; P:anatomical structure morphogenesis; P:multicellular organismal development; C:extracellular region | IPR002181 (PFAM); IPR014716 (G3DSA:3.90.215.GENE3D); PTHR19143 (PANTHER), PTHR19143:SF20 (PANTHER), SignalP-NN(euk) (SIGNALP), tmhmm (TMHMM) | Yes |
| 1324 | lib_dib_Filter15_IDBA_contig_23471_minus3 | 44 | angiopoietin-related protein 7-like | 135 | 3.58E-26 | 58.10% | 3 | F:receptor binding; C:extracellular space; P:signal transduction | IPR002181 (PFAM); IPR014715 (G3DSA:4.10.530.GENE3D); IPR014716 (G3DSA:3.90.215.GENE3D); PTHR19143 (PANTHER) | |
| 1325 | lib_dib_Filter15_IDBA_contig_23472_minus3 | 53 | angiopoietin-related protein 4 | 103 | 5.43E-17 | 69.30% | 8 | P:regulation of biological process; F:enzyme regulator activity; F:receptor binding; P:anatomical structure morphogenesis; P:multicellular organismal development; P:biological_process; P:cell death; C:extracellular region | IPR002181 (PFAM); IPR014716 (G3DSA:3.90.215.GENE3D); PTHR19143 (PANTHER) | |
| 1326 | lib_dib_Filter15_IDBA_contig_23473_minus2 | 502 | serine threonine-protein kinase tbk1 | 874 | 3.12E-156 | 65.35% | 3 | F:nucleotide binding; P:cellular protein modification process; F:protein kinase activity | IPR000719 (PFAM); IPR002290 (SMART); IPR011009 (SUPERFAMILY); IPR017441 (PROSITE); IPR020635 (SMART); G3DSA:1.10.510.10 (GENE3D), G3DSA:3.10.20.90 (GENE3D), G3DSA:3.30.200.20 (GENE3D), PTHR22969 (PANTHER), PTHR22969:SF2 (PANTHER) | |
| 1327 | lib_dib_Filter15_IDBA_contig_23474_minus2 | 594 | serine threonine-protein kinase tbk1 | 1066 | 8.39E-154 | 65.35% | 3 | F:nucleotide binding; P:cellular protein modification process; F:protein kinase activity | IPR000719 (PFAM); IPR002290 (SMART); IPR011009 (SUPERFAMILY); IPR017441 (PROSITE); IPR020635 (SMART); G3DSA:1.10.510.10 (GENE3D), G3DSA:3.10.20.90 (GENE3D), G3DSA:3.30.200.20 (GENE3D), PTHR22969 (PANTHER), PTHR22969:SF2 (PANTHER) | |
| 1328 | lib_dib_Filter15_IDBA_contig_23518_plus1 | 1160 | cyclin a | 1664 | 2.19E-133 | 62.65% | 4 | P:regulation of biological process; P:cell cycle; P:multicellular organismal development; C:nucleus | IPR004367 (PFAM); IPR006671 (PFAM); IPR013763 (G3DSA:1.10.472.GENE3D); PTHR10177 (PANTHER), PTHR10177:SF69 (PANTHER), SignalP-NN(euk) (SIGNALP), tmhmm (TMHMM) | |
| 1329 | lib_dib_Filter15_IDBA_contig_23522_plus2 | 96 | zinc finger protein 709-like | 431 | 1.07E-37 | 43.80% | 3 | F:binding; F:nucleic acid binding; C:intracellular | IPR007087 (PFAM); IPR013087 (G3DSA:3.30.160.GENE3D); IPR015880 (SMART); PTHR24375 (PANTHER), PTHR24375:SF0 (PANTHER), PF12874 (PFAM), PF13465 (PFAM), SSF57667 (SUPERFAMILY) | |
| 1330 | lib_dib_Filter15_IDBA_contig_23551_plus3 | 73 | neurogenic locus notch-like protein | 266 | 3.63E-11 | 47.05% | 2 | F:binding; P:biological_process | IPR000421 (PFAM); IPR006585 (SMART); IPR008979 (SUPERFAMILY); G3DSA:2.60.120.260 (GENE3D), PTHR19277 (PANTHER), PTHR19277:SF6 (PANTHER), tmhmm (TMHMM) | |
| 1331 | lib_dib_Filter15_IDBA_contig_23553_plus1 | 77 | hypothetical protein CAPTEDRAFT_216318 | 320 | 4.71E-12 | 50.00% | 1 | P:biological_process | IPR000421 (PFAM); IPR006585 (SMART); IPR008979 (SUPERFAMILY); G3DSA:2.60.120.260 (GENE3D), SignalP-NN(euk) (SIGNALP), tmhmm (TMHMM) | Yes |
| 1332 | lib_dib_Filter15_IDBA_contig_23554_plus3 | 13 | fucl7_angja ame: full=fucolectin-7 flags: precursor | 133 | 6.52E-11 | 50.45% | 2 | F:binding; P:biological_process | IPR000421 (PFAM); IPR006585 (SMART); IPR008979 (SUPERFAMILY); G3DSA:2.60.120.260 (GENE3D), PTHR19277 (PANTHER), PTHR19277:SF6 (PANTHER), SignalP-NN(euk) (SIGNALP) | Yes |
| 1333 | lib_dib_Filter15_IDBA_contig_23603_plus1 | 239 | chymotrypsin-like protease ctrl-1 | 545 | 8.47E-60 | 57.45% | 3 | F:peptidase activity; P:protein metabolic process; P:catabolic process | IPR001254 (PFAM); IPR001314 (PRINTS); IPR009003 (SUPERFAMILY); IPR018114 (PROSITE); G3DSA:2.40.10.10 (GENE3D), PTHR24256 (PANTHER), SignalP-NN(euk) (SIGNALP) | |

| | | | | | | | | | | |
|---|---|---|---|---|---|---|---|---|---|---|
| 1334 | lib_dib_Filter15_IDBA_contig_23604_plus1 | 53 | serine protease hepsin | 168 | 1.63E-35 | 57.60% | 21 | C:cell; P:regulation of biological process; P:protein metabolic process; P:cell proliferation; P:reproduction; P:viral reproduction; P:symbiosis, encompassing mutualism through parasitism; ; P:catabolic process; P:anatomical structure morphogenesis; P:cellular component organization; P:cell differentiation; P:multicellular organismal development; C:plasma membrane; P:cell growth; F:protein binding; F:peptidase activity; C:nuclear envelope; P:biological_process; P:cell death; C:endoplasmic reticulum | IPR001254 (PFAM); IPR009003 (SUPERFAMILY); IPR018114 (PROSITE); G3DSA:2.40.10.10 (GENE3D), PTHR24256 (PANTHER) | |
| 1335 | lib_dib_Filter15_IDBA_contig_23623_plus1 | 89 | a disintegrin and metalloproteinase with thrombospondin motifs 14 | 429 | 2.84E-17 | 42.45% | 3 | F:peptidase activity; P:protein metabolic process; P:catabolic process | IPR001590 (PROFILE); IPR024079 (G3DSA:3.40.390.GENE3D); PTHR13723 (PANTHER), PTHR13723:SF22 (PANTHER), PF13574 (PFAM), SSF55486 (SUPERFAMILY) | |
| 1336 | lib_dib_Filter15_IDBA_contig_23639_minus1 | 119 | cadherin egf lag seven-pass g-type receptor 1 | 432 | 5.91E-132 | 59.25% | 4 | P:signal transduction; F:protein binding; F:receptor activity; C:cell | IPR000742 (PFAM); IPR001879 (SMART); IPR002049 (PFAM); IPR013032 (PROSITE); IPR022624 (PFAM); G3DSA:2.10.25.10 (GENE3D), PTHR24026 (PANTHER), PTHR24026:SF1 (PANTHER), SSF57196 (SUPERFAMILY) | |
| 1337 | lib_dib_Filter15_IDBA_contig_23652_minus2 | 44 | protein sidekick-like | 428 | 4.69E-141 | 61.60% | 1 | F:protein binding | IPR003961 (PFAM); IPR013783 (G3DSA:2.60.40.GENE3D); PR00014 (PRINTS), PTHR10489 (PANTHER), PTHR10489:SF57 (PANTHER) | |
| 1338 | lib_dib_Filter15_IDBA_contig_23656_plus1 | 1079 | fibrinogen-like 1 | 595 | 2.01E-42 | 54.75% | 1 | C:extracellular region | IPR002181 (PFAM); IPR014715 (G3DSA:4.10.530.GENE3D); IPR014716 (G3DSA:3.90.215.GENE3D); PTHR19143 (PANTHER), SignalP-NN(euk) (SIGNALP), tmhmm (TMHMM) | Yes |
| 1339 | lib_dib_Filter15_IDBA_contig_23657_plus2 | 935 | fibrinogen-like 1 | 613 | 1.80E-42 | 54.60% | 1 | C:extracellular region | IPR002181 (PFAM); IPR014715 (G3DSA:4.10.530.GENE3D); IPR014716 (G3DSA:3.90.215.GENE3D); PTHR19143 (PANTHER), SignalP-NN(euk) (SIGNALP) | Yes |
| 1340 | lib_dib_Filter15_IDBA_contig_23658_plus1 | 984 | fibrinogen-like 1 | 603 | 2.66E-43 | 54.50% | 1 | C:extracellular region | IPR002181 (PFAM); IPR014715 (G3DSA:4.10.530.GENE3D); IPR014716 (G3DSA:3.90.215.GENE3D); PTHR19143 (PANTHER), SignalP-NN(euk) (SIGNALP), tmhmm (TMHMM) | Yes |
| 1341 | lib_dib_Filter15_IDBA_contig_23659_plus1 | 302 | fibrinogen-like protein 1 | 428 | 9.76E-49 | 56.55% | 1 | C:extracellular region | IPR002181 (PFAM); IPR014715 (G3DSA:4.10.530.GENE3D); IPR014716 (G3DSA:3.90.215.GENE3D); IPR020837 (PROSITE); PTHR19143 (PANTHER), tmhmm (TMHMM) | |
| 1342 | lib_dib_Filter15_IDBA_contig_23661_plus1 | 21 | ficolin (collagen fibrinogen domain containing) 1 | 104 | 1.03E-17 | 56.10% | 1 | F:binding | IPR002181 (PFAM); IPR014715 (G3DSA:4.10.530.GENE3D); IPR014716 (G3DSA:3.90.215.GENE3D); IPR020837 (PROSITE); PTHR19143 (PANTHER) | |
| 1343 | lib_dib_Filter15_IDBA_contig_23685_plus2 | 62 | thioredoxin domain-containing protein 15 | 427 | 2.20E-64 | 60.55% | 2 | P:cellular homeostasis; P:regulation of biological process | IPR012336 (G3DSA:3.40.30.GENE3D); IPR013766 (PFAM); PTHR14684 (PANTHER), PTHR14684:SF0 (PANTHER), SignalP-NN(euk) (SIGNALP) | Yes |

| | | | | | | | | | |
|---|---|---|---|---|---|---|---|---|---|
| 1344 | lib_dib_Filter15_IDBA_contig_23699_minus1 | 169 | low-density lipoprotein receptor-related protein 8-like | 588 | 9.38E-82 | 53.90% | 2 | F:protein binding; F:calcium ion binding | IPR000033 (PFAM); IPR000152 (PROSITE); IPR000742 (SMART); IPR001881 (PFAM); IPR009030 (SUPERFAMILY); IPR011042 (G3DSA:2.120.10.GENE3D); IPR018097 (PROSITE); G3DSA:2.10.25.10 (GENE3D), PTHR10529 (PANTHER), SSF63825 (SUPERFAMILY) | |
| 1345 | lib_dib_Filter15_IDBA_contig_23701_plus2 | 112 | low quality protein: basement membrane-specific heparan sulfate proteoglycan core | 552 | 1.62E-14 | 39.15% | 1 | F:protein binding | IPR002172 (G3DSA:4.10.400.GENE3D); PR00261 (PRINTS), PTHR12106 (PANTHER), PTHR12106:SF7 (PANTHER), tmhmm (TMHMM) | |
| 1346 | lib_dib_Filter15_IDBA_contig_23709_plus3 | 361 | hydroxysteroid (17-beta) dehydrogenase 4 | 427 | 1.52E-166 | 76.35% | 8 | F:catalytic activity; P:biosynthetic process; P:lipid metabolic process; P:catabolic process; C:peroxisome; F:receptor binding; F:protein binding; F:nucleotide binding | IPR002198 (PRINTS); IPR002347 (PRINTS); IPR016040 (G3DSA:3.40.50.GENE3D); IPR020842 (SMART); G3DSA:3.10.129.10 (GENE3D), PTHR24316 (PANTHER), PTHR24316:SF2 (PANTHER), SSF51735 (SUPERFAMILY), SSF54637 (SUPERFAMILY) | |
| 1347 | lib_dib_Filter15_IDBA_contig_23723_minus2 | 104 | transmembrane protease serine 3 | 570 | 1.31E-121 | 57.95% | 8 | C:endoplasmic reticulum; F:molecular_function; P:biological_process; P:cellular homeostasis; C:cell; F:peptidase activity; P:protein metabolic process; P:catabolic process | IPR001254 (PFAM); IPR001314 (PRINTS); IPR009003 (SUPERFAMILY); IPR018114 (PROSITE); G3DSA:2.40.10.10 (GENE3D), PTHR24265 (PANTHER) | |
| 1348 | lib_dib_Filter15_IDBA_contig_23759_plus2 | 1768 | ankyrin repeat-containing protein | 1504 | 5.91E-42 | 49.80% | 1 | F:protein binding | IPR001496 (PFAM); IPR002110 (PRINTS); IPR020683 (G3DSA:1.25.40.GENE3D); PTHR24198 (PANTHER) | |
| 1349 | lib_dib_Filter15_IDBA_contig_23762_plus1 | 248 | osteoclast-stimulating factor 1 | 472 | 6.98E-91 | 76.75% | 1 | F:protein binding | IPR001452 (PRINTS); IPR002110 (SMART); IPR020683 (G3DSA:1.25.40.GENE3D); G3DSA:2.30.30.40 (GENE3D), PTHR24155 (PANTHER), PTHR24155:SF0 (PANTHER) | |
| 1350 | lib_dib_Filter15_IDBA_contig_23764_plus3 | 65 | mitogen-activated protein kinase kinase kinase 2 | 426 | 2.16E-127 | 78.65% | 13 | F:protein binding; P:signal transduction; P:response to stress; P:cellular protein modification process; C:cytosol; P:response to external stimulus; P:response to abiotic stimulus; F:protein kinase activity; F:signal transducer activity; P:regulation of biological process; ; F:nucleotide binding; C:nucleus | IPR000719 (PFAM); IPR002290 (SMART); IPR011009 (SUPERFAMILY); IPR017441 (PROSITE); IPR020635 (SMART); G3DSA:1.10.510.10 (GENE3D), G3DSA:3.30.200.20 (GENE3D), PTHR24360 (PANTHER), PTHR24360:SF37 (PANTHER) | |
| 1351 | lib_dib_Filter15_IDBA_contig_23765_minus1 | 168 | leucine-rich repeats and immunoglobulin-like domains protein 2 | 480 | 1.78E-12 | 47.05% | 1 | F:protein binding | IPR001611 (PROFILE); IPR003961 (PFAM); IPR013783 (G3DSA:2.60.40.GENE3D); G3DSA:3.80.10.10 (GENE3D), PTHR24365 (PANTHER), PF13855 (PFAM), SignalP-NN(euk) (SIGNALP), tmhmm (TMHMM), SSF52058 (SUPERFAMILY) | |
| 1352 | lib_dib_Filter15_IDBA_contig_23775_minus1 | 682 | phospholipid-hydroperoxide glutathione peroxidase | 252 | 2.36E-80 | 75.25% | 4 | F:antioxidant activity; F:catalytic activity; P:response to stress; P:metabolic process | IPR000889 (PRINTS); IPR012336 (G3DSA:3.40.30.GENE3D); PTHR11592:SF0 (PANTHER) | |
| 1353 | lib_dib_Filter15_IDBA_contig_23783_minus1 | 150 | stromal cell derived factor 4 | 633 | 1.95E-114 | 64.85% | 5 | P:biological_process; F:calcium ion binding; P:transport; F:protein binding; C:Golgi apparatus | IPR002048 (PFAM); IPR011992 (G3DSA:1.10.238.GENE3D); IPR018247 (PROSITE); IPR027240 (PTHR10827:PANTHER); PTHR10827 (PANTHER), SignalP-NN(euk) (SIGNALP), tmhmm (TMHMM), SSF47473 (SUPERFAMILY) | Yes |
| 1354 | lib_dib_Filter15_IDBA_contig_23800_minus3 | 91 | acid sphingomyelinase-like phosphodiesterase 3a | 425 | 8.44E-63 | 51.10% | 1 | F:hydrolase activity | IPR004843 (PFAM); G3DSA:3.60.21.10 (GENE3D), PTHR10340 (PANTHER), SignalP-NN(euk) (SIGNALP), tmhmm (TMHMM), SSF56300 (SUPERFAMILY) | |

| | | | | | | | | | |
|---|---|---|---|---|---|---|---|---|---|
| 1355 | lib_dib_Filter15_IDBA_contig_23806_plus3 | 4831 | achain crystal structure of engineered northeast structural genomics consortium target | 937 | 1.69E-20 | 54.90% | 1 | F:protein binding | IPR002110 (SMART); IPR020683 (G3DSA:1.25.40.GENE3D); PTHR24193 (PANTHER), tmhmm (TMHMM) |
| 1356 | lib_dib_Filter15_IDBA_contig_23824_plus3 | 253 | cysteine-rich with egf-like domain protein 2-like | 708 | 8.37E-74 | 56.20% | 2 | F:protein binding; F:calcium ion binding | IPR000152 (PROSITE); IPR000742 (SMART); IPR001881 (PFAM); IPR002049 (PROSITE); IPR006212 (SMART); IPR009030 (SUPERFAMILY); IPR013032 (PROSITE); IPR018097 (PROSITE); IPR021852 (PFAM); G3DSA:2.10.220.10 (GENE3D), G3DSA:2.10.25.10 (GENE3D), PTHR24838 (PANTHER), PTHR24838:SF13 (PANTHER) |
| 1357 | lib_dib_Filter15_IDBA_contig_23832_minus3 | 76 | cre-wrt-6 protein | 455 | 5.82E-14 | 52.70% | 6 | P:biological_process; P:multicellular organismal development; P:cell communication; P:protein metabolic process; P:catabolic process; F:peptidase activity | IPR001657 (PRINTS); IPR001767 (PFAM); IPR003587 (SMART); IPR006141 (PROFILE); G3DSA:2.170.16.10 (GENE3D), PTHR11889 (PANTHER), SSF51294 (SUPERFAMILY) |
| 1358 | lib_dib_Filter15_IDBA_contig_23865_plus2 | 196 | frizzled receptor 5 8 | 577 | 5.14E-72 | 89.15% | 16 | P:anatomical structure morphogenesis; P:multicellular organismal development; P:cell differentiation; P:signal transduction; P:cell-cell signaling; P:response to stress; P:cellular protein modification process; P:response to biotic stimulus; P:cell death; F:protein binding; F:receptor activity; C:cell; P:embryo development; P:regulation of biological process; P:cell proliferation; | IPR015526 (PANTHER); IPR020067 (G3DSA:1.10.2000.GENE3D); IPR026547 (PTHR11309:PANTHER) |
| 1359 | lib_dib_Filter15_IDBA_contig_23866_plus1 | 52 | frizzled receptor 5 8 | 147 | 1.10E-76 | 88.80% | 16 | P:anatomical structure morphogenesis; P:multicellular organismal development; P:cell differentiation; P:signal transduction; P:cell-cell signaling; P:response to stress; P:cellular protein modification process; P:response to biotic stimulus; P:cell death; F:protein binding; F:receptor activity; C:cell; P:embryo development; P:regulation of biological process; P:cell proliferation; | IPR015526 (PANTHER); IPR020067 (G3DSA:1.10.2000.GENE3D); IPR026547 (PTHR11309:PANTHER) |
| 1360 | lib_dib_Filter15_IDBA_contig_23867_plus1 | 132 | frizzled homolog 8a | 328 | 7.27E-168 | 77.40% | 14 | P:signal transduction; P:cell differentiation; P:multicellular organismal development; F:protein binding; P:anatomical structure morphogenesis; P:cellular component organization; F:receptor activity; P:regulation of biological process; ; P:embryo development; C:cell; P:reproduction; C:cytoplasm; C:plasma membrane | IPR000539 (PRINTS); IPR015526 (PANTHER); IPR017981 (PROFILE); IPR020067 (G3DSA:1.10.2000.GENE3D); IPR026545 (PTHR11309:PANTHER); tmhmm (TMHMM) |

| | | | | | | | | | |
|---|---|---|---|---|---|---|---|---|---|
| 1361 | lib_dib_Filter15_IDBA_contig_23872_minus3 | 51 | achain structural basis of the 70-kilodalton heat shock cognate protein atp hydrolytic structure of the active site with adp or atp bound to wild type and mutant atpase fragment | 162 | 8.24E-73 | 93.90% | 33 | C:cell; F:receptor binding; F:lipid binding; P:cellular component organization; P:transport; P:cell-cell signaling; P:organelle organization; P:cell cycle; C:cytoplasmic membrane-bounded vesicle; F:nucleotide binding; P:metabolic process; P:regulation of biological process; ; C:nucleolus; C:cytosol; C:nuclear chromosome; P:reproduction; P:cell differentiation; F:protein binding; C:organelle; C:extracellular region; P:protein metabolic process; C:nucleus; C:protein complex; P:response to stress; P:response to abiotic stimulus; F:hydrolase activity; P:nucleobase-containing compound metabolic process; C:plasma membrane; C:intracellular; P:response to biotic stimulus; P:biological_process; C:mitochondrion | IPR013126 (PRINTS); IPR018181 (PROSITE); G3DSA:3.30.30.30 (GENE3D), G3DSA:3.30.420.40 (GENE3D), PTHR19375 (PANTHER), SSF53067 (SUPERFAMILY) |
| 1362 | lib_dib_Filter15_IDBA_contig_23873_minus1 | 136 | heat shock protein 70 | 246 | 1.11E-111 | 87.20% | 25 | C:cell; F:receptor binding; P:cellular component organization; P:transport; P:cell-cell signaling; C:cytoplasmic membrane-bounded vesicle; F:nucleotide binding; P:regulation of biological process; ; C:nucleolus; C:cytosol; P:cell cycle; F:protein binding; C:organelle; C:extracellular region; P:protein metabolic process; C:protein complex; F:hydrolase activity; P:nucleobase-containing compound metabolic process; P:catabolic process; C:plasma membrane; C:intracellular; P:response to stress; P:response to biotic stimulus; P:biological_process | IPR013126 (PFAM); G3DSA:1.20.1270.10 (GENE3D), G3DSA:2.60.34.10 (GENE3D), PTHR19375 (PANTHER), SSF100920 (SUPERFAMILY), SSF100934 (SUPERFAMILY) |
| 1363 | lib_dib_Filter15_IDBA_contig_23874_minus1 | 257 | heat shock protein 70 | 421 | 1.27E-108 | 90.60% | 25 | C:cell; F:receptor binding; P:cellular component organization; P:transport; P:cell-cell signaling; C:cytoplasmic membrane-bounded vesicle; F:nucleotide binding; P:regulation of biological process; ; C:nucleolus; C:cytosol; P:cell cycle; F:protein binding; C:organelle; C:extracellular region; P:protein metabolic process; C:protein complex; F:hydrolase activity; P:nucleobase-containing compound metabolic process; P:catabolic process; C:plasma membrane; C:intracellular; P:response to stress; P:response to biotic stimulus; P:biological_process | IPR013126 (PFAM); G3DSA:1.20.1270.10 (GENE3D), G3DSA:2.60.34.10 (GENE3D), PTHR19375 (PANTHER), SSF100920 (SUPERFAMILY), SSF100934 (SUPERFAMILY) |

| | | | | | | | | | | |
|---|---|---|---|---|---|---|---|---|---|---|
| 1364 | lib_dib_Filter15_IDBA_contig_23875_minus1 | 155 | heat shock protein 70 | 424 | 0 | 94.10% | 18 | C:cell; F:receptor binding; F:nucleotide binding; P:regulation of biological process; ; C:cytosol; P:cell cycle; C:protein complex; F:protein binding; P:protein metabolic process; C:organelle; C:extracellular region; C:cytoplasmic membrane-bounded vesicle; P:response to stress; F:hydrolase activity; P:nucleobase-containing compound metabolic process; P:catabolic process; C:intracellular | IPR013126 (PRINTS); IPR018181 (PROSITE); G3DSA:2.60.34.10 (GENE3D), G3DSA:3.30.30.30 (GENE3D), G3DSA:3.30.420.40 (GENE3D), G3DSA:3.90.640.10 (GENE3D), PTHR19375 (PANTHER), SSF100920 (SUPERFAMILY), SSF53067 (SUPERFAMILY) | |
| 1365 | lib_dib_Filter15_IDBA_contig_23876_minus2 | 51 | ensangg00000017398 protein | 166 | 6.66E-79 | 95.15% | 25 | C:cell; F:receptor binding; P:cellular component organization; P:transport; P:cell-cell signaling; C:cytoplasmic membrane-bounded vesicle; F:nucleotide binding; P:regulation of biological process; ; C:nucleolus; C:cytosol; P:cell cycle; F:protein binding; C:organelle; C:extracellular region; P:protein metabolic process; C:protein complex; F:hydrolase activity; P:nucleobase-containing compound metabolic process; P:catabolic process; C:plasma membrane; C:intracellular; P:response to stress; P:response to biotic stimulus; P:biological_process | IPR013126 (PRINTS); IPR018181 (PROSITE); G3DSA:2.60.34.10 (GENE3D), G3DSA:3.30.420.40 (GENE3D), PTHR19375 (PANTHER), SSF100920 (SUPERFAMILY), SSF53067 (SUPERFAMILY) | |
| 1366 | lib_dib_Filter15_IDBA_contig_23885_plus3 | 178 | low quality protein: obscurin | 760 | 3.27E-26 | 41.05% | 3 | F:protein binding; C:intracellular; P:regulation of biological process | IPR000436 (SMART); IPR003598 (SMART); IPR003599 (SMART); IPR007110 (PROFILE); IPR013098 (PFAM); IPR013783 (G3DSA:2.60.40.GENE3D); G3DSA:2.10.70.10 (GENE3D), PTHR10489 (PANTHER), PF13895 (PFAM), PF13927 (PFAM), tmhmm (TMHMM), SSF48726 (SUPERFAMILY) | |
| 1367 | lib_dib_Filter15_IDBA_contig_23896_plus2 | 344 | leukotriene a-4 hydrolase | 866 | 0 | 73.00% | 12 | C:cytosol; F:binding; P:response to external stimulus; P:response to stress; P:biosynthetic process; P:lipid metabolic process; C:nucleolus; P:catabolic process; F:peptidase activity; P:biological_process; F:hydrolase activity; P:protein metabolic process | IPR001930 (PANTHER); IPR012777 (TIGRFAMs); IPR014782 (PRINTS); IPR015211 (PFAM); IPR016024 (SUPERFAMILY); G3DSA:1.10.390.10 (GENE3D), PTHR11533:SF4 (PANTHER), SSF55486 (SUPERFAMILY), SSF63737 (SUPERFAMILY) | |
| 1368 | lib_dib_Filter15_IDBA_contig_23910_plus1 | 145 | glypican-6 | 423 | 3.94E-74 | 71.10% | 3 | C:plasma membrane; C:proteinaceous extracellular matrix; F:protein binding | IPR001863 (PANTHER); PTHR10822:SF7 (PANTHER), tmhmm (TMHMM) | |
| 1369 | lib_dib_Filter15_IDBA_contig_23911_minus1 | 490 | aldehyde dehydrogenase 16 member a1 | 717 | 0 | 79.95% | 2 | F:catalytic activity; P:metabolic process | IPR015590 (PFAM); IPR016160 (PROSITE); IPR016161 (SUPERFAMILY); IPR016162 (G3DSA:3.40.605.GENE3D); IPR016163 (G3DSA:3.40.309.GENE3D); PTHR11699 (PANTHER), PTHR11699:SF81 (PANTHER), SignalP-NN(euk) (SIGNALP) | Yes |
| 1370 | lib_dib_Filter15_IDBA_contig_23912_minus1 | 513 | aldehyde dehydrogenase 16 member a1 | 761 | 0 | 79.95% | 2 | F:catalytic activity; P:metabolic process | IPR015590 (PFAM); IPR016160 (PROSITE); IPR016161 (SUPERFAMILY); IPR016162 (G3DSA:3.40.605.GENE3D); IPR016163 (G3DSA:3.40.309.GENE3D); PTHR11699 (PANTHER), PTHR11699:SF81 (PANTHER), SignalP-NN(euk) (SIGNALP) | Yes |
| 1371 | lib_dib_Filter15_IDBA_contig_23915_plus2 | 109 | ankyrin repeat protein | 423 | 1.25E-23 | 52.35% | 1 | F:protein binding | IPR002110 (PRINTS); IPR020683 (G3DSA:1.25.40.GENE3D); PTHR24198 (PANTHER) | |

| | | | | | | | | | | |
|---|---|---|---|---|---|---|---|---|---|---|
| 1372 | lib_dib_Filter15_IDBA_contig_23916_plus2 | 1661 | coatomer subunit beta | 709 | 0 | 93.80% | 11 | C:cytoplasmic membrane-bounded vesicle; C:Golgi apparatus; C:protein complex; F:protein binding; C:cytosol; P:transport; C:cytoskeleton; P:organelle organization; P:embryo development; P:protein transport; F:structural molecule activity | IPR001680 (PFAM); IPR006692 (PFAM); IPR011048 (SUPERFAMILY); IPR015943 (G3DSA:2.130.10.GENE3D); IPR017986 (PROFILE); IPR020472 (PRINTS); PTHR19876 (PANTHER), PTHR19876:SF2 (PANTHER) | |
| 1373 | lib_dib_Filter15_IDBA_contig_23917_minus3 | 438 | cd22 antigen | 879 | 1.29E-19 | 39.85% | 2 | C:cell; F:protein binding | IPR003598 (SMART); IPR003599 (SMART); IPR007110 (PROFILE); IPR013106 (PFAM); IPR013783 (G3DSA:2.60.40.GENE3D); PTHR10489 (PANTHER), PF13895 (PFAM), SSF48726 (SUPERFAMILY) | |
| 1374 | lib_dib_Filter15_IDBA_contig_23939_minus3 | 270 | von willebrand factor type egf and pentraxin domain-containing protein 1-like | 869 | 6.37E-45 | 42.15% | 6 | P:response to stress; F:binding; P:response to external stimulus; P:regulation of biological process; P:protein metabolic process; P:metabolic process | IPR000436 (PFAM); G3DSA:2.10.70.10 (GENE3D), PTHR19325 (PANTHER), SignalP-NN(euk) (SIGNALP), tmhmm (TMHMM) | |
| 1375 | lib_dib_Filter15_IDBA_contig_23943_plus3 | 125 | neogenin isoform 3 | 565 | 2.80E-167 | 63.35% | 10 | P:anatomical structure morphogenesis; P:cellular component organization; P:cell differentiation; P:multicellular organismal development; F:protein binding; P:regulation of biological process; ; P:biological_process; F:receptor activity; C:plasma membrane | IPR003598 (SMART); IPR003599 (SMART); IPR003961 (PFAM); IPR007110 (PROFILE); IPR013098 (PFAM); IPR013783 (G3DSA:2.60.40.GENE3D); PR00014 (PRINTS), PTHR10489 (PANTHER), PTHR10489:SF55 (PANTHER), SSF48726 (SUPERFAMILY) | |
| 1376 | lib_dib_Filter15_IDBA_contig_23968_minus1 | 1742 | deleted in malignant brain tumors 1 | 734 | 2.63E-77 | 56.65% | 8 | C:extracellular region; F:peptidase activity; C:cell; P:protein metabolic process; P:catabolic process; C:organelle; C:cytoplasm; F:receptor activity | IPR001190 (PRINTS); IPR017448 (SMART); G3DSA:3.10.250.10 (GENE3D), PTHR19331 (PANTHER), SignalP-NN(euk) (SIGNALP), tmhmm (TMHMM) | Yes |
| 1377 | lib_dib_Filter15_IDBA_contig_23969_minus1 | 1490 | deleted in malignant brain tumors 1 | 669 | 9.41E-78 | 56.80% | 3 | F:peptidase activity; F:receptor activity; C:cell | IPR001190 (PRINTS); IPR017448 (SMART); G3DSA:3.10.250.10 (GENE3D), PTHR19331 (PANTHER), SignalP-NN(euk) (SIGNALP), tmhmm (TMHMM) | Yes |
| 1378 | lib_dib_Filter15_IDBA_contig_23970_minus2 | 168 | galectin-3-binding protein precursor | 146 | 2.58E-06 | 64.14% | 2 | F:receptor activity; C:cell | IPR001190 (PFAM); IPR017448 (SUPERFAMILY); G3DSA:3.10.250.10 (GENE3D), PTHR19331 (PANTHER), PTHR19331:SF118 (PANTHER) | |
| 1379 | lib_dib_Filter15_IDBA_contig_23980_minus3 | 128 | | 421 | 0 | 100% | 1 | F:protein binding | IPR003598 (SMART); IPR003599 (SMART); IPR007110 (PROFILE); IPR013098 (PFAM); IPR013783 (G3DSA:2.60.40.GENE3D); SSF48726 (SUPERFAMILY) | |
| 1380 | lib_dib_Filter15_IDBA_contig_23996_minus1 | 599 | transmembrane protease serine 9-like | 638 | 2.91E-46 | 52.25% | 4 | C:cell; F:peptidase activity; P:protein metabolic process; P:catabolic process | IPR000859 (G3DSA:2.60.120.GENE3D); IPR000998 (PFAM); IPR001254 (PFAM); IPR001314 (PRINTS); IPR008985 (SUPERFAMILY); IPR009003 (SUPERFAMILY); IPR018114 (PROSITE); G3DSA:2.40.10.10 (GENE3D), PTHR24265 (PANTHER), SignalP-NN(euk) (SIGNALP) | |
| 1381 | lib_dib_Filter15_IDBA_contig_23997_minus1 | 103 | tolloid-like protein 2 | 177 | 5.15E-18 | 60.30% | 2 | F:binding; F:peptidase activity | IPR000859 (G3DSA:2.60.120.GENE3D); PTHR10127 (PANTHER), SignalP-NN(euk) (SIGNALP), tmhmm (TMHMM) | |
| 1382 | lib_dib_Filter15_IDBA_contig_24011_minus1 | 222 | dehydrogenase reductase sdr family member 4 | 421 | 2.85E-103 | 75.40% | 3 | P:metabolic process; F:catalytic activity; F:nucleotide binding | IPR002198 (PRINTS); IPR002347 (PRINTS); IPR016040 (G3DSA:3.40.50.GENE3D); IPR020904 (PROSITE); IPR027052 (PTHR24322:PANTHER); PTHR24322 (PANTHER), PF13561 (PFAM), SSF51735 (SUPERFAMILY) | |
| 1383 | lib_dib_Filter15_IDBA_contig_24032_minus1 | 815 | 60 kda lysophospholipase-like | 808 | 0 | 69.60% | 3 | F:protein binding; F:hydrolase activity; | IPR002110 (SMART); IPR006033 (TIGRFAMs); IPR006034 (PRINTS); IPR020683 (G3DSA:1.25.40.GENE3D); IPR020827 (PROSITE); G3DSA:3.40.50.1170 (GENE3D), G3DSA:3.40.50.40 (GENE3D), PTHR11707 (PANTHER), PTHR11707:SF3 (PANTHER), PF13857 (PFAM) | |

| | | | | | | | | | | |
|---|---|---|---|---|---|---|---|---|---|---|
| 1384 | lib_dib_Filter15_IDBA_contig_24067_minus3 | 74 | netrin-1-like | 420 | 0 | 87.05% | 11 | P:anatomical structure morphogenesis; P:multicellular organismal development; P:cellular component organization; P:cell differentiation; P:regulation of biological process; F:receptor binding; C:proteinaceous extracellular matrix; P:biological_process; P:cell death; C:Golgi apparatus; P:cell proliferation | IPR002049 (PFAM); IPR008211 (PFAM); G3DSA:2.10.25.10 (GENE3D), G3DSA:2.60.120.260 (GENE3D), PTHR10574 (PANTHER), PTHR10574:SF44 (PANTHER), SSF57196 (SUPERFAMILY) | |
| 1385 | lib_dib_Filter15_IDBA_contig_24072_plus3 | 108 | retinoid-inducible serine carboxypeptidase | 511 | 3.19E-70 | 54.60% | 3 | F:peptidase activity; P:protein metabolic process; P:catabolic process | IPR001563 (PRINTS); G3DSA:3.40.50.1820 (GENE3D), PTHR11802:SF3 (PANTHER), SignalP-NN(euk) (SIGNALP), tmhmm (TMHMM), SSF53474 (SUPERFAMILY) | Yes |
| 1386 | lib_dib_Filter15_IDBA_contig_24073_minus1 | 52 | ankyrin repeat protein | 420 | 2.43E-73 | 50.90% | 1 | F:protein binding | IPR002110 (SMART); IPR020683 (G3DSA:1.25.40.GENE3D); PTHR24166 (PANTHER), PTHR24166:SF6 (PANTHER), PF13637 (PFAM), tmhmm (TMHMM) | |
| 1387 | lib_dib_Filter15_IDBA_contig_24081_plus1 | 152 | kelch-like protein 18 | 442 | 2.57E-130 | 59.85% | 6 | P:protein metabolic process; C:nucleus; C:organelle; C:intracellular; F:protein binding; C:cytoplasm | IPR006652 (PFAM); IPR015916 (G3DSA:2.130.10.GENE3D); PTHR24412 (PANTHER), PTHR24412:SF48 (PANTHER), SSF117281 (SUPERFAMILY) | |
| 1388 | lib_dib_Filter15_IDBA_contig_24082_plus2 | 273 | ring canal kelch homolog | 720 | 0 | 60.20% | 2 | C:intracellular; F:protein binding | IPR000210 (SMART); IPR006652 (PFAM); IPR011333 (G3DSA:3.30.710.GENE3D); IPR011705 (PFAM); IPR013069 (PFAM); IPR015916 (G3DSA:2.130.10.GENE3D); PTHR24412 (PANTHER), PTHR24412:SF48 (PANTHER), SSF117281 (SUPERFAMILY) | |
| 1389 | lib_dib_Filter15_IDBA_contig_24083_minus2 | 145 | kelch-like protein 18 | 457 | 0 | 77.10% | 1 | F:protein binding | IPR000210 (SMART); IPR006652 (PFAM); IPR011333 (G3DSA:3.30.710.GENE3D); IPR011705 (PFAM); IPR013069 (PFAM); IPR015916 (G3DSA:2.130.10.GENE3D); PTHR24414 (PANTHER), PTHR24414:SF0 (PANTHER), SSF117281 (SUPERFAMILY) | |
| 1390 | lib_dib_Filter15_IDBA_contig_24084_minus2 | 204 | kelch-like protein 18 | 627 | 0 | 76.45% | 1 | F:protein binding | IPR000210 (SMART); IPR006652 (PFAM); IPR011333 (G3DSA:3.30.710.GENE3D); IPR011705 (PFAM); IPR013069 (PFAM); IPR015916 (G3DSA:2.130.10.GENE3D); IPR017096 (PIR); PTHR24414 (PANTHER), PTHR24414:SF0 (PANTHER), SSF117281 (SUPERFAMILY) | |
| 1391 | lib_dib_Filter15_IDBA_contig_24118_plus1 | 61 | protein-glutamine gamma-glutamyltransferase k-like | 420 | 3.20E-141 | 61.85% | 1 | P:cellular protein modification process | IPR001102 (PFAM); IPR002931 (PFAM); IPR013783 (G3DSA:2.60.40.GENE3D); IPR013808 (PROSITE); IPR014756 (SUPERFAMILY); IPR023608 (PANTHER); G3DSA:3.90.260.10 (GENE3D), SSF54001 (SUPERFAMILY) | |
| 1392 | lib_dib_Filter15_IDBA_contig_24170_minus1 | 696 | acid sphingomyelinase-like phosphodiesterase 3a | 547 | 1.30E-80 | 52.00% | 1 | F:hydrolase activity | IPR004843 (PFAM); G3DSA:3.60.21.10 (GENE3D), PTHR10340 (PANTHER), SignalP-NN(euk) (SIGNALP), SSF56300 (SUPERFAMILY) | |
| 1393 | lib_dib_Filter15_IDBA_contig_24185_plus2 | 34 | arylsulfatase b | 140 | 3.49E-45 | 80.20% | 2 | F:hydrolase activity; P:metabolic process | IPR000917 (PFAM); IPR017849 (G3DSA:3.40.720.GENE3D); IPR017850 (SUPERFAMILY); IPR024607 (PROSITE); PTHR10342 (PANTHER), PTHR10342:SF19 (PANTHER), SignalP-NN(euk) (SIGNALP) | |
| 1394 | lib_dib_Filter15_IDBA_contig_24186_plus1 | 119 | arylsulfatase b | 419 | 4.43E-63 | 50.70% | 2 | P:metabolic process; F:hydrolase activity | IPR000917 (PFAM); IPR017849 (G3DSA:3.40.720.GENE3D); IPR017850 (SUPERFAMILY); G3DSA:3.30.1120.10 (GENE3D), PTHR10342 (PANTHER), PTHR10342:SF19 (PANTHER), tmhmm (TMHMM) | |

| | | | | | | | | | | |
|---|---|---|---|---|---|---|---|---|---|---|
| 1395 | lib_dib_Filter15_IDBA_contig_24187_plus1 | 17 | arylsulfatase j-like | 118 | 3.64E-46 | 75.45% | 2 | P:metabolic process; F:hydrolase activity | IPR000917 (PFAM); IPR017849 (G3DSA:3.40.720.GENE3D); IPR017850 (SUPERFAMILY); IPR024607 (PROSITE); PTHR10342 (PANTHER), PTHR10342:SF19 (PANTHER) | |
| 1396 | lib_dib_Filter15_IDBA_contig_24188_plus1 | 10 | arylsulfatase j-like | 114 | 1.42E-41 | 75.70% | 2 | F:hydrolase activity; P:metabolic process | IPR000917 (PFAM); IPR017849 (G3DSA:3.40.720.GENE3D); IPR017850 (SUPERFAMILY); IPR024607 (PROSITE); PTHR10342 (PANTHER), PTHR10342:SF19 (PANTHER) | |
| 1397 | lib_dib_Filter15_IDBA_contig_24190_plus1 | 59 | collagen triple helix repeat protein | 419 | 2.40E-15 | 59.00% | 1 | C:collagen | IPR008160 (PFAM); PTHR24023 (PANTHER) | |
| 1398 | lib_dib_Filter15_IDBA_contig_24281_plus2 | 207 | uncharacterized family 31 glucosidase kiaa1161-like | 798 | 0 | 60.80% | 2 | P:carbohydrate metabolic process; F:catalytic activity | IPR000322 (PANTHER); IPR013785 (G3DSA:3.20.20.GENE3D); IPR017853 (SUPERFAMILY); PTHR22762:SF2 (PANTHER), SSF51011 (SUPERFAMILY) | |
| 1399 | lib_dib_Filter15_IDBA_contig_24289_plus1 | 282 | galectin-9 isoform 2 | 196 | 4.23E-32 | 57.10% | 3 | F:signal transducer activity; F:carbohydrate binding; P:signal transduction | IPR001079 (PFAM); IPR008985 (SUPERFAMILY); IPR013320 (G3DSA:2.60.120.GENE3D); PTHR11346 (PANTHER) | |
| 1400 | lib_dib_Filter15_IDBA_contig_24304_minus3 | 109 | inhibitor of nuclear factor kappa-b kinase subunit alpha | 416 | 5.91E-98 | 64.10% | 20 | C:cytosol; C:protein complex; P:reproduction; P:multicellular organismal development; P:response to biotic stimulus; P:cell differentiation; P:cell proliferation; P:signal transduction; F:protein kinase activity; F:signal transducer activity; C:cell; P:biological_process; P:response to stress; P:anatomical structure morphogenesis; F:protein binding; P:response to endogenous stimulus; P:cellular protein modification process; C:nucleus; C:plasma membrane; F:nucleotide binding | IPR000719 (PFAM); IPR002290 (SMART); IPR008271 (PROSITE); IPR011009 (SUPERFAMILY); IPR017441 (PROSITE); IPR020635 (SMART); G3DSA:1.10.510.10 (GENE3D), G3DSA:3.30.200.20 (GENE3D), PTHR22969 (PANTHER) | |
| 1401 | lib_dib_Filter15_IDBA_contig_24336_plus1 | 166 | zinc finger protein 271-like | 688 | 2.99E-43 | 56.15% | 3 | F:binding; F:nucleic acid binding; C:intracellular | IPR007087 (PROSITE); IPR013087 (G3DSA:3.30.160.GENE3D); IPR015880 (SMART); PTHR11389 (PANTHER), PF13465 (PFAM), PF13894 (PFAM), PF13912 (PFAM), SignalP-NN(euk) (SIGNALP), tmhmm (TMHMM), SSF57667 (SUPERFAMILY) | Yes |
| 1402 | lib_dib_Filter15_IDBA_contig_24362_plus1 | 266 | 3-phosphoinositide-dependent protein kinase 1-like | 677 | 0 | 71.75% | 20 | P:metabolic process; P:regulation of biological process; P:signal transduction; P:cellular component organization; C:cytosol; ; F:protein kinase activity; C:plasma membrane; P:cellular protein modification process; C:cytoplasmic membrane-bounded vesicle; P:response to endogenous stimulus; F:receptor binding; F:protein binding; P:response to stress; P:response to abiotic stimulus; P:cell differentiation; P:multicellular organismal development; P:transport; P:cell death; F:nucleotide binding | IPR000719 (PFAM); IPR002290 (SMART); IPR008271 (PROSITE); IPR011009 (SUPERFAMILY); IPR011993 (G3DSA:2.30.29.GENE3D); IPR017441 (PROSITE); IPR020635 (SMART); G3DSA:1.10.510.10 (GENE3D), G3DSA:3.30.200.20 (GENE3D), PTHR24352 (PANTHER), PTHR24352:SF27 (PANTHER), SSF50729 (SUPERFAMILY) | |

| | | | | | | | | | | |
|---|---|---|---|---|---|---|---|---|---|---|
| 1403 | lib_dib_Filter15_IDBA_contig_24387_plus3 | 39 | tyrosine-protein kinase btk29a | 224 | 3.07E-26 | 63.35% | 19 | P:multicellular organismal development; P:behavior; P:anatomical structure morphogenesis; F:protein kinase activity; P:reproduction; P:regulation of biological process; P:cytoskeleton organization; C:cytoplasm; C:extracellular region; P:embryo development; P:DNA metabolic process; P:biosynthetic process; P:cell cycle; P:transport; P:cellular component organization; P:organelle organization; C:plasma membrane; F:nucleotide binding; P:cellular protein modification process | IPR000719 (PROFILE); IPR001245 (PRINTS); IPR002290 (SMART); IPR008266 (PROSITE); IPR011009 (SUPERFAMILY); IPR020635 (SMART); G3DSA:1.10.510.10 (GENE3D), PTHR24418 (PANTHER), PTHR24418:SF0 (PANTHER) | |
| 1404 | lib_dib_Filter15_IDBA_contig_24399_plus1 | 160 | protein kinase c iota type | 693 | 0 | 81.65% | 27 | P:regulation of biological process; P:cell death; P:anatomical structure morphogenesis; P:cellular component organization; P:multicellular organismal development; P:cytoskeleton organization; C:plasma membrane; P:biological_process; P:cell cycle; C:cell; P:embryo development; P:cell differentiation; P:cell proliferation; C:protein complex; F:lipid binding; P:transport; C:cytosol; P:organelle organization; F:protein kinase activity; P:response to endogenous stimulus; F:protein binding; F:binding; ; F:nucleotide binding; C:nucleus; P:cellular protein modification process; C:endosome | IPR000270 (PFAM); IPR000719 (PFAM); IPR000961 (SMART); IPR002219 (PFAM); IPR002290 (SMART); IPR008271 (PROSITE); IPR011009 (SUPERFAMILY); IPR017441 (PROSITE); IPR017892 (PFAM); IPR020454 (PRINTS); IPR020635 (SMART); G3DSA:1.10.510.10 (GENE3D), G3DSA:3.10.20.240 (GENE3D), G3DSA:3.30.200.20 (GENE3D), G3DSA:3.30.60.20 (GENE3D), PTHR24357 (PANTHER), PTHR24357:SF58 (PANTHER), SignalP-NN(euk) (SIGNALP), SSF54277 (SUPERFAMILY), SSF57889 (SUPERFAMILY) | Yes |
| 1405 | lib_dib_Filter15_IDBA_contig_24413_plus3 | 86 | estradiol 17-beta-dehydrogenase 2 | 414 | 1.53E-126 | 60.55% | 3 | P:metabolic process; F:catalytic activity; F:nucleotide binding | IPR002198 (PRINTS); IPR002347 (PRINTS); IPR016040 (G3DSA:3.40.50.GENE3D); IPR020904 (PROSITE); PTHR24316 (PANTHER), PTHR24316:SF35 (PANTHER), SSF51735 (SUPERFAMILY) | |
| 1406 | lib_dib_Filter15_IDBA_contig_24414_plus2 | 234 | neuronal cell adhesion | 697 | 5.39E-42 | 42.15% | 2 | P:biological_process; F:protein binding | IPR003598 (SMART); IPR003599 (SMART); IPR007110 (PROFILE); IPR013098 (PFAM); IPR013783 (G3DSA:2.60.40.GENE3D); PTHR10489 (PANTHER), PF13895 (PFAM), PF13927 (PFAM), SignalP-NN(euk) (SIGNALP), tmhmm (TMHMM), SSF48726 (SUPERFAMILY) | Yes |
| 1407 | lib_dib_Filter15_IDBA_contig_24455_plus1 | 1895 | serine threonine-protein kinase pak 2 | 1005 | 2.90E-157 | 65.00% | 9 | C:nucleoplasm; F:protein binding; P:cellular protein modification process; C:cytosol; P:regulation of biological process; P:cell death; P:metabolic process; F:nucleotide binding; F:protein kinase activity | IPR000095 (PFAM); IPR000719 (PFAM); IPR002290 (SMART); IPR008271 (PROSITE); IPR011009 (SUPERFAMILY); IPR017441 (PROSITE); IPR020635 (SMART); G3DSA:1.10.510.10 (GENE3D), G3DSA:3.30.200.20 (GENE3D), G3DSA:3.90.810.10 (GENE3D), PTHR24361 (PANTHER), PTHR24361:SF186 (PANTHER) | |
| 1408 | lib_dib_Filter15_IDBA_contig_24501_minus3 | 143 | glycoprotein-n-acetylgalactosamine 3-beta-galactosyltransferase 1-like | 485 | 4.98E-68 | 72.80% | 5 | C:cell; F:transferase activity; P:biosynthetic process; P:cellular protein modification process; P:carbohydrate metabolic process | IPR003378 (PFAM); IPR026842 (PTHR23033:PANTHER); PTHR23033 (PANTHER), tmhmm (TMHMM) | |
| 1409 | lib_dib_Filter15_IDBA_contig_24507_minus1 | 500 | lamin dm0 | 926 | 9.17E-153 | 58.35% | 1 | F:structural molecule activity | IPR001664 (PANTHER); IPR016044 (PFAM); G3DSA:1.20.5.170 (GENE3D), G3DSA:2.60.40.1260 (GENE3D), PTHR23239:SF12 (PANTHER), SignalP-NN(euk) (SIGNALP), tmhmm (TMHMM), SSF74853 (SUPERFAMILY) | |

| | | | | | | | | | | |
|---|---|---|---|---|---|---|---|---|---|---|
| 1410 | lib_dib_Filter15_IDBA_contig_24531_plus2 | 167 | mitogen-activated protein kinase 1-like | 412 | 0 | 90.05% | 5 | F:protein kinase activity; F:signal transducer activity; P:signal transduction; P:cellular protein modification process; F:nucleotide binding | IPR000719 (PFAM); IPR002290 (SMART); IPR003527 (PROSITE); IPR008271 (PROSITE); IPR008349 (PRINTS); IPR011009 (SUPERFAMILY); IPR017441 (PROSITE); IPR020635 (SMART); G3DSA:1.10.510.10 (GENE3D), G3DSA:3.30.200.20 (GENE3D), PTHR24055 (PANTHER), PTHR24055:SF23 (PANTHER) | |
| 1411 | lib_dib_Filter15_IDBA_contig_24565_plus1 | 915 | notch gene homolog 1 | 1067 | 1.35E-21 | 47.60% | 21 | P:cell differentiation; P:multicellular organismal development; P:regulation of biological process; P:cell proliferation; P:anatomical structure morphogenesis; P:signal transduction; P:embryo development; P:cellular component organization; C:organelle; C:intracellular; ; P:cell death; C:cytoplasm; P:cell communication; F:DNA binding; P:biological_process; P:reproduction; C:cell; F:calcium ion binding; F:actin binding; P:cytoskeleton organization | IPR000152 (PROSITE); IPR000742 (SMART); IPR001881 (SMART); IPR002035 (PFAM); IPR005455 (PFAM); IPR011641 (PFAM); IPR013032 (PROSITE); IPR018097 (PROSITE); G3DSA:2.10.25.10 (GENE3D), G3DSA:3.30.450.30 (GENE3D), G3DSA:3.40.50.410 (GENE3D), PTHR24838 (PANTHER), SignalP-NN(euk) (SIGNALP), tmhmm (TMHMM), SSF53300 (SUPERFAMILY), SSF57196 (SUPERFAMILY) | |
| 1412 | lib_dib_Filter15_IDBA_contig_24619_plus3 | 91 | tumor necrosis factor-inducible gene 6 | 410 | 6.30E-18 | 52.75% | 2 | P:biological_process; C:cell | IPR000859 (G3DSA:2.60.120.GENE3D); IPR000998 (PFAM); IPR008985 (SUPERFAMILY); PTHR10127 (PANTHER), SignalP-NN(euk) (SIGNALP), tmhmm (TMHMM) | Yes |
| 1413 | lib_dib_Filter15_IDBA_contig_24636_minus2 | 664 | lysosomal alpha-mannosidase | 743 | 0 | 66.60% | 6 | C:cell; F:hydrolase activity; C:lysosome; F:carbohydrate binding; P:carbohydrate metabolic process; F:binding | IPR000602 (PFAM); IPR011013 (SUPERFAMILY); IPR011330 (SUPERFAMILY); IPR011682 (PFAM); IPR013780 (G3DSA:2.60.40.GENE3D); IPR015341 (PFAM); IPR027291 (G3DSA:3.20.110.GENE3D); G3DSA:1.20.1270.50 (GENE3D), G3DSA:2.70.98.30 (GENE3D), PTHR11607 (PANTHER), PTHR11607:SF3 (PANTHER), SignalP-NN(euk) (SIGNALP), tmhmm (TMHMM), SSF88688 (SUPERFAMILY) | |
| 1414 | lib_dib_Filter15_IDBA_contig_24641_plus3 | 274 | protein nas-13 | 409 | 8.30E-55 | 50.20% | 4 | F:peptidase activity; P:protein metabolic process; P:catabolic process; F:binding | IPR001506 (PRINTS); IPR003582 (PFAM); IPR006026 (SMART); IPR024079 (G3DSA:3.40.390.GENE3D); PTHR10127 (PANTHER), SignalP-NN(euk) (SIGNALP), tmhmm (TMHMM), SSF55486 (SUPERFAMILY) | Yes |
| 1415 | lib_dib_Filter15_IDBA_contig_24650_minus2 | 44 | ecto-nox disulfide-thiol exchanger 1-like | 208 | 1.20E-05 | 75.60% | 2 | F:nucleic acid binding; F:nucleotide binding | PTHR16001 (PANTHER) | |
| 1416 | lib_dib_Filter15_IDBA_contig_24651_minus2 | 115 | ecto-nox disulfide-thiol exchanger 1-like | 511 | 9.91E-25 | 83.10% | 10 | P:biological_process; F:nucleic acid binding; C:plasma membrane; P:transport; C:extracellular region; F:catalytic activity; P:regulation of biological process; P:growth; F:nucleotide binding; P:generation of precursor metabolites and energy | PTHR16001 (PANTHER), PTHR16001:SF1 (PANTHER) | |
| 1417 | lib_dib_Filter15_IDBA_contig_24652_minus2 | 70 | ecto-nox disulfide-thiol exchanger 2 isoform 1 | 409 | 2.08E-111 | 79.05% | 11 | C:extracellular space; P:biological_process; F:nucleic acid binding; C:plasma membrane; P:cell growth; C:cytosol; F:catalytic activity; P:regulation of biological process; P:growth; F:nucleotide binding; P:generation of precursor metabolites and energy | IPR000504 (PFAM); IPR012677 (G3DSA:3.30.70.GENE3D); PTHR16001 (PANTHER), SSF54928 (SUPERFAMILY) | |

| | | | | | | | | | |
|---|---|---|---|---|---|---|---|---|---|
| 1418 | lib_dib_Filter15_IDBA_contig_24656_minus2 | 1389 | arylsulfatase b | 590 | 1.53E-130 | 64.90% | 2 | P:metabolic process; F:hydrolase activity | IPR000917 (PFAM); IPR017849 (G3DSA:3.40.720.GENE3D); IPR017850 (SUPERFAMILY); IPR024607 (PROSITE); G3DSA:3.30.1120.10 (GENE3D), PTHR10342 (PANTHER), PTHR10342:SF19 (PANTHER), tmhmm (TMHMM) |
| 1419 | lib_dib_Filter15_IDBA_contig_24657_minus2 | 2406 | arylsulfatase b | 1320 | 9.24E-139 | 58.90% | 2 | P:metabolic process; F:hydrolase activity | IPR000917 (PFAM); IPR017849 (G3DSA:3.40.720.GENE3D); IPR017850 (SUPERFAMILY); IPR024607 (PROSITE); G3DSA:3.30.1120.10 (GENE3D), PTHR10342 (PANTHER), PTHR10342:SF19 (PANTHER), tmhmm (TMHMM) |
| 1420 | lib_dib_Filter15_IDBA_contig_24658_minus2 | 2362 | arylsulfatase b | 1294 | 9.88E-139 | 58.95% | 2 | P:metabolic process; F:hydrolase activity | IPR000917 (PFAM); IPR017849 (G3DSA:3.40.720.GENE3D); IPR017850 (SUPERFAMILY); IPR024607 (PROSITE); G3DSA:3.30.1120.10 (GENE3D), PTHR10342 (PANTHER), PTHR10342:SF19 (PANTHER), tmhmm (TMHMM) |
| 1421 | lib_dib_Filter15_IDBA_contig_24659_minus2 | 2348 | arylsulfatase b | 1315 | 1.15E-138 | 58.95% | 2 | P:metabolic process; F:hydrolase activity | IPR000917 (PFAM); IPR017849 (G3DSA:3.40.720.GENE3D); IPR017850 (SUPERFAMILY); IPR024607 (PROSITE); G3DSA:3.30.1120.10 (GENE3D), PTHR10342 (PANTHER), PTHR10342:SF19 (PANTHER), tmhmm (TMHMM) |
| 1422 | lib_dib_Filter15_IDBA_contig_24664_minus3 | 213 | arylsulfatase j | 151 | 3.45E-40 | 67.35% | 2 | P:metabolic process; F:hydrolase activity | IPR000917 (PFAM); IPR017849 (G3DSA:3.40.720.GENE3D); IPR017850 (SUPERFAMILY); PTHR10342 (PANTHER), PTHR10342:SF19 (PANTHER) |
| 1423 | lib_dib_Filter15_IDBA_contig_24699_minus2 | 217 | multiple inositol polyphosphate phosphatase 1 | 484 | 3.30E-50 | 46.40% | 1 | F:hydrolase activity | IPR000560 (PFAM); G3DSA:3.40.50.1240 (GENE3D), PTHR20963 (PANTHER), PTHR20963:SF8 (PANTHER), SSF53254 (SUPERFAMILY) |
| 1424 | lib_dib_Filter15_IDBA_contig_24701_minus2 | 315 | multiple inositol polyphosphate phosphatase 1 | 847 | 4.04E-48 | 46.25% | 1 | F:hydrolase activity | IPR000560 (PFAM); G3DSA:3.40.50.1240 (GENE3D), PTHR20963 (PANTHER), PTHR20963:SF8 (PANTHER), tmhmm (TMHMM), SSF53254 (SUPERFAMILY) |
| 1425 | lib_dib_Filter15_IDBA_contig_24765_plus3 | 99 | low quality protein: hemicentin-2 | 406 | 3.37E-103 | 56.65% | 3 | C:extracellular region; F:protein binding; F:calcium ion binding | IPR000152 (PROSITE); IPR000742 (SMART); IPR001881 (PFAM); IPR002919 (SUPERFAMILY); IPR013032 (PROSITE); IPR018097 (PROSITE); IPR026823 (PFAM); G3DSA:2.10.25.10 (GENE3D), PTHR24034 (PANTHER), SSF57196 (SUPERFAMILY) |
| 1426 | lib_dib_Filter15_IDBA_contig_24895_plus1 | 895 | very low-density lipoprotein receptor precursor | 885 | 1.36E-128 | 50.90% | 2 | F:protein binding; F:calcium ion binding | IPR000033 (PFAM); IPR000152 (PROSITE); IPR000742 (SMART); IPR001881 (PFAM); IPR002172 (G3DSA:4.10.400.GENE3D); IPR009030 (SUPERFAMILY); IPR011042 (G3DSA:2.120.10.GENE3D); IPR018097 (PROSITE); IPR023415 (PROSITE); PR00261 (PRINTS), G3DSA:2.10.25.10 (GENE3D), PTHR10529 (PANTHER), SignalP-NN(euk) (SIGNALP), SSF57196 (SUPERFAMILY), SSF63825 (SUPERFAMILY) |
| 1427 | lib_dib_Filter15_IDBA_contig_24896_minus2 | 66 | zinc finger protein 135- partial | 405 | 9.43E-36 | 50.70% | 3 | F:binding; F:nucleic acid binding; C:intracellular | IPR007087 (PROSITE); IPR013087 (G3DSA:3.30.160.GENE3D); IPR015880 (SMART); PTHR24395 (PANTHER), PTHR24395:SF0 (PANTHER), PF13465 (PFAM), PF13894 (PFAM), tmhmm (TMHMM), SSF57667 (SUPERFAMILY) |

| | | | | | | | | | | |
|---|---|---|---|---|---|---|---|---|---|---|
| 1428 | lib_dib_Filter15_IDBA_contig_24897_minus1 | 1972 | serine threonine-protein kinase d3 | 744 | 9.10E-57 | 57.10% | 1 | F:protein binding | IPR000008 (PFAM); IPR008973 (SUPERFAMILY); IPR018029 (PROFILE); G3DSA:2.60.40.150 (GENE3D), PTHR21119 (PANTHER), SignalP-NN(euk) (SIGNALP), tmhmm (TMHMM) | |
| 1429 | lib_dib_Filter15_IDBA_contig_24907_plus3 | 187 | epidermal growth factor receptor | 435 | 1.00E-82 | 52.20% | 27 | P:regulation of biological process; P:multicellular organismal development; C:nucleus; P:cell proliferation; C:organelle; C:intracellular; P:cellular component organization; ; P:signal transduction; C:cell; P:metabolic process; P:transport; P:anatomical structure morphogenesis; C:plasma membrane; C:cellular_component; P:cell death; P:cellular protein modification process; C:mitochondrion; P:cell differentiation; P:biological_process; F:protein binding; P:response to stress; P:response to endogenous stimulus; C:extracellular region; F:receptor activity; F:protein kinase activity; F:nucleotide binding | IPR000494 (PFAM); IPR006211 (PFAM); IPR006212 (SMART); IPR009030 (SUPERFAMILY); G3DSA:2.10.220.10 (GENE3D), G3DSA:3.80.20.20 (GENE3D), PTHR24416 (PANTHER), PTHR24416:SF88 (PANTHER), SSF52058 (SUPERFAMILY) | |
| 1430 | lib_dib_Filter15_IDBA_contig_24908_plus1 | 96 | epidermal growth factor receptor | 199 | 3.73E-20 | 53.10% | 6 | F:receptor activity; F:protein kinase activity; F:nucleotide binding; P:cellular protein modification process; P:signal transduction; C:cell | IPR006211 (PFAM); IPR006212 (SMART); IPR009030 (SUPERFAMILY); G3DSA:2.10.220.10 (GENE3D), PTHR24416 (PANTHER), PTHR24416:SF88 (PANTHER) | |
| 1431 | lib_dib_Filter15_IDBA_contig_24920_minus2 | 868 | nicotinic acetylcholine receptor subunit a2 | 553 | 1.31E-62 | 57.45% | 3 | P:ion transport; C:cell; F:ion channel activity | IPR006029 (SUPERFAMILY); IPR006201 (PRINTS); IPR006202 (G3DSA:2.70.170.GENE3D); IPR018000 (PROSITE); G3DSA:1.20.120.370 (GENE3D), PTHR18945:SF127 (PANTHER), SignalP-NN(euk) (SIGNALP), tmhmm (TMHMM) | Yes |
| 1432 | lib_dib_Filter15_IDBA_contig_24938_plus1 | 33640 | proactivator polypeptide | 1639 | 2.94E-158 | 51.50% | 2 | C:lysosome; P:lipid metabolic process | IPR003119 (PFAM); IPR007856 (PFAM); IPR008138 (PFAM); IPR008139 (SMART); IPR008373 (PRINTS); IPR011001 (G3DSA:1.10.225.GENE3D); PTHR11480 (PANTHER), PTHR11480:SF3 (PANTHER), SignalP-NN(euk) (SIGNALP) | |
| 1433 | lib_dib_Filter15_IDBA_contig_24945_plus2 | 216 | protein flp | 741 | 1.81E-104 | 51.70% | 5 | P:oxidation-reduction process; F:oxidoreductase activity; F:riboflavin reductase (NADPH) activity; F:FMN binding; F:protein domain specific binding | IPR001466 (PFAM); IPR012338 (G3DSA:3.40.710.GENE3D); PTHR22935 (PANTHER), SignalP-NN(euk) (SIGNALP), tmhmm (TMHMM) | |
| 1434 | lib_dib_Filter15_IDBA_contig_24951_minus3 | 312 | dual specificity mitogen-activated protein kinase kinase 6-like | 680 | 5.69E-151 | 78.50% | 3 | F:nucleotide binding; P:cellular protein modification process; F:protein kinase activity | IPR000719 (PFAM); IPR002290 (SMART); IPR008271 (PROSITE); IPR011009 (SUPERFAMILY); IPR017441 (PROSITE); IPR020635 (SMART); G3DSA:1.10.510.10 (GENE3D), G3DSA:3.30.200.20 (GENE3D), PTHR24361 (PANTHER), PTHR24361:SF40 (PANTHER) | |
| 1435 | lib_dib_Filter15_IDBA_contig_24989_minus2 | 61 | maltose phosphorylase | 403 | 3.89E-127 | 56.65% | 2 | F:catalytic activity; P:carbohydrate metabolic process | IPR005195 (PFAM); IPR008928 (SUPERFAMILY); IPR012341 (G3DSA:1.50.10.GENE3D); PTHR11051 (PANTHER), PTHR11051:SF4 (PANTHER) | |
| 1436 | lib_dib_Filter15_IDBA_contig_25006_minus3 | 574 | family s60 non-peptidase homologue (s60 family) | 910 | 0 | 54.05% | 4 | C:extracellular region; P:ion transport; P:cellular homeostasis; F:binding | IPR001156 (PRINTS); G3DSA:3.40.190.10 (GENE3D), SignalP-NN(euk) (SIGNALP), tmhmm (TMHMM), SSF53850 (SUPERFAMILY) | Yes |
| 1437 | lib_dib_Filter15_IDBA_contig_25055_plus2 | 56 | ankyrin repeat and fyve domain-containing protein 1 | 402 | 9.10E-154 | 71.60% | 5 | F:hydrolase activity; C:endosome; P:transport; P:cellular component organization; F:protein binding | IPR000210 (SMART); IPR002110 (SMART); IPR011333 (G3DSA:3.30.710.GENE3D); IPR013069 (PFAM); IPR020683 (G3DSA:1.25.40.GENE3D); PTHR24189 (PANTHER), PTHR24189:SF0 (PANTHER) | |

| | | | | | | | | | | |
|---|---|---|---|---|---|---|---|---|---|---|
| 1438 | lib_dib_Filter15_IDBA_contig_25079_minus2 | 2318 | n-acetylated-alpha-linked acidic dipeptidase 2 | 781 | 0 | 62.90% | 3 | F:peptidase activity; P:protein metabolic process; P:catabolic process | IPR003137 (PFAM); IPR007365 (G3DSA:1.20.930.GENE3D); IPR007484 (PFAM); G3DSA:3.40.630.10 (GENE3D), G3DSA:3.50.30.30 (GENE3D), PTHR10404 (PANTHER), PTHR10404:SF15 (PANTHER), SignalP-NN(euk) (SIGNALP), tmhmm (TMHMM), SSF52025 (SUPERFAMILY), SSF53187 (SUPERFAMILY) | |
| 1439 | lib_dib_Filter15_IDBA_contig_25099_plus3 | 272 | kruppel-like factor 9 | 754 | 8.06E-58 | 58.85% | 8 | P:regulation of biological process; ; P:reproduction; P:multicellular organismal development; P:signal transduction; C:nucleus; F:binding; F:nucleic acid binding | IPR007087 (PROSITE); IPR013087 (G3DSA:3.30.160.GENE3D); IPR015880 (SMART); PTHR23223 (PANTHER), PTHR23223:SF31 (PANTHER), PF13465 (PFAM), SSF57667 (SUPERFAMILY) | |
| 1440 | lib_dib_Filter15_IDBA_contig_25150_plus2 | 125 | transcriptional repressor ctcf | 400 | 5.34E-83 | 84.60% | 3 | F:binding; F:nucleic acid binding; C:intracellular | IPR007087 (PFAM); IPR013087 (G3DSA:3.30.160.GENE3D); IPR015880 (SMART); PTHR24375 (PANTHER), PF13465 (PFAM), SSF57667 (SUPERFAMILY) | |
| 1441 | lib_dib_Filter15_IDBA_contig_25168_minus3 | 246 | upf0510 protein inm02-like | 225 | 9.20E-57 | 60.05% | 2 | C:integral to membrane; C:ER membrane protein complex | SignalP-NN(euk) (SIGNALP), tmhmm (TMHMM) | Yes |
| 1442 | lib_dib_Filter15_IDBA_contig_25171_plus1 | 48 | lactase-phlorizin hydrolase | 400 | 2.57E-40 | 70.10% | 2 | F:hydrolase activity; P:carbohydrate metabolic process | IPR001360 (PANTHER); IPR013781 (G3DSA:3.20.20.GENE3D); IPR017853 (SUPERFAMILY); IPR018120 (PROSITE) | |
| 1443 | lib_dib_Filter15_IDBA_contig_25171_plus2 | 48 | ---NA--- | 400 | | | 0 | - | no IPS match | |
| 1444 | lib_dib_Filter15_IDBA_contig_25171_plus3 | 48 | ---NA--- | 399 | | | 0 | - | tmhmm (TMHMM) | |
| 1445 | lib_dib_Filter15_IDBA_contig_25213_plus1 | 88 | glyoxal reductase-like | 399 | 1.45E-73 | 71.40% | 2 | F:catalytic activity; P:metabolic process | IPR001395 (PANTHER); IPR020471 (PRINTS); IPR023210 (G3DSA:3.20.20.GENE3D); PTHR11732:SF34 (PANTHER) | |
| 1446 | lib_dib_Filter15_IDBA_contig_25215_plus3 | 189 | small gtp-binding protein | 531 | 4.72E-17 | 49.00% | 1 | F:protein binding | IPR001611 (PFAM); IPR003591 (SMART); G3DSA:3.80.10.10 (GENE3D), PTHR24367 (PANTHER), PF13855 (PFAM), SM00364 (SMART), SSF52058 (SUPERFAMILY) | |
| 1447 | lib_dib_Filter15_IDBA_contig_25216_plus2 | 288 | small gtp-binding protein | 658 | 7.77E-17 | 48.95% | 1 | F:protein binding | IPR001611 (PFAM); IPR003591 (SMART); G3DSA:3.80.10.10 (GENE3D), PTHR24367 (PANTHER), PF13855 (PFAM), SM00364 (SMART), SSF52058 (SUPERFAMILY) | |
| 1448 | lib_dib_Filter15_IDBA_contig_25217_plus2 | 15 | leucine-rich repeat-containing g-protein coupled receptor 4 | 156 | 1.81E-11 | 51.35% | 1 | F:protein binding | IPR001611 (PROFILE); IPR003591 (SMART); G3DSA:3.80.10.10 (GENE3D), PTHR24365 (PANTHER), PF13855 (PFAM), SSF52058 (SUPERFAMILY) | |
| 1449 | lib_dib_Filter15_IDBA_contig_25266_plus1 | 49669 | guanine nucleotide-binding protein subunit beta-2-like 1 | 377 | 0 | 93.55% | 25 | P:regulation of biological process; P:cell death; C:cytoskeleton; F:protein binding; C:cell; C:ribosome; F:receptor activity; F:receptor binding; P:biological_process; P:cell cycle; P:cellular component organization; C:plasma membrane; P:translation; P:cellular protein modification process; P:transport; P:signal transduction; C:cytoplasm; F:molecular_function; P:protein metabolic process; P:catabolic process; F:enzyme regulator activity; F:kinase activity; C:nucleus; P:cell growth; P:metabolic process | IPR001680 (PFAM); IPR015943 (G3DSA:2.130.10.GENE3D); IPR017986 (PROFILE); IPR019775 (PROSITE); IPR020472 (PRINTS); PTHR19868 (PANTHER), PTHR19868:SF0 (PANTHER), SignalP-NN(euk) (SIGNALP) | |

| | | | | | | | | | | |
|---|---|---|---|---|---|---|---|---|---|---|
| 1450 | lib_dib_Filter15_IDBA_contig_25291_minus3 | 92 | serine threonine-protein kinase sbk1 | 537 | 3.90E-146 | 71.05% | 4 | P:cellular protein modification process; P:multicellular organismal development; F:protein kinase activity; F:nucleotide binding | IPR000719 (PFAM); IPR002290 (SMART); IPR008271 (PROSITE); IPR011009 (SUPERFAMILY); IPR017441 (PROSITE); IPR020635 (SMART); G3DSA:1.10.510.10 (GENE3D), G3DSA:3.30.200.20 (GENE3D), PTHR24359 (PANTHER), PTHR24359:SF0 (PANTHER) | |
| 1451 | lib_dib_Filter15_IDBA_contig_25333_plus1 | 15103 | cathepsin b | 885 | 6.41E-132 | 74.35% | 7 | C:organelle; C:intracellular; C:cytoplasm; P:protein metabolic process; P:catabolic process; F:peptidase activity; P:biological_process | IPR000169 (PROSITE); IPR000668 (PRINTS); IPR012599 (PFAM); IPR013128 (PANTHER); IPR015643 (PTHR12411:PANTHER); IPR025660 (PROSITE); IPR025661 (PROSITE); G3DSA:3.90.70.10 (GENE3D), tmhmm (TMHMM), SSF54001 (SUPERFAMILY) | |
| 1452 | lib_dib_Filter15_IDBA_contig_25355_plus3 | 71 | probable carboxypeptidase pm20d1 precursor | 409 | 1.63E-142 | 64.95% | 2 | P:metabolic process; F:hydrolase activity | IPR002933 (PFAM); IPR011650 (PFAM); G3DSA:3.40.630.10 (GENE3D), PTHR11014 (PANTHER), PTHR11014:SF9 (PANTHER), SignalP-NN(euk) (SIGNALP), tmhmm (TMHMM), SSF53187 (SUPERFAMILY) | |
| 1453 | lib_dib_Filter15_IDBA_contig_25358_minus2 | 168 | epidermal growth factor receptor | 711 | 0 | 63.95% | 4 | C:cell; F:nucleotide binding; P:cellular protein modification process; F:protein kinase activity | IPR000494 (PFAM); IPR000719 (PROFILE); IPR001245 (PRINTS); IPR002290 (SMART); IPR006212 (SMART); IPR008266 (PROSITE); IPR009030 (SUPERFAMILY); IPR011009 (SUPERFAMILY); IPR017441 (PROSITE); IPR020635 (SMART); G3DSA:1.10.510.10 (GENE3D), G3DSA:2.10.220.10 (GENE3D), G3DSA:3.30.200.20 (GENE3D), G3DSA:3.80.20.20 (GENE3D), G3DSA:4.10.1140.10 (GENE3D), PTHR24416 (PANTHER), PTHR24416:SF95 (PANTHER), tmhmm (TMHMM), SSF52058 (SUPERFAMILY) | |
| 1454 | lib_dib_Filter15_IDBA_contig_25359_minus3 | 63 | receptor protein-tyrosine phosphatase 10d | 397 | 4.14E-16 | 40.45% | 1 | F:protein binding | IPR003961 (PFAM); IPR013783 (G3DSA:2.60.40.GENE3D); PTHR19143 (PANTHER) | |
| 1455 | lib_dib_Filter15_IDBA_contig_25383_plus3 | 513 | collagen alpha-6 chain | 899 | 1.69E-70 | 42.45% | 1 | F:protein binding | IPR002035 (PFAM); PR00453 (PRINTS), PR01217 (PRINTS), G3DSA:3.40.50.410 (GENE3D), PTHR22992 (PANTHER), SSF53300 (SUPERFAMILY) | |
| 1456 | lib_dib_Filter15_IDBA_contig_25391_minus3 | 1202 | lysyl oxidase-like 2 | 920 | 0 | 57.55% | 4 | F:receptor activity; C:cell; F:binding; P:metabolic process | IPR001190 (PRINTS); IPR001695 (PRINTS); IPR017448 (SMART); G3DSA:3.10.250.10 (GENE3D), PTHR19331 (PANTHER), PTHR19331:SF25 (PANTHER), tmhmm (TMHMM) | |
| 1457 | lib_dib_Filter15_IDBA_contig_25392_minus3 | 48 | scavenger partial | 100 | 2.10E-27 | 60.75% | 2 | F:receptor activity; C:cell | IPR001190 (PFAM); IPR017448 (SMART); G3DSA:3.10.250.10 (GENE3D), PTHR19331 (PANTHER), PTHR19331:SF10 (PANTHER) | |
| 1458 | lib_dib_Filter15_IDBA_contig_25467_minus1 | 57 | cg32791 cg32791-pa | 396 | 1.88E-113 | 53.20% | 1 | F:protein binding | IPR003598 (SMART); IPR003599 (SMART); IPR007110 (PROFILE); IPR013098 (PFAM); IPR013783 (G3DSA:2.60.40.GENE3D); PTHR19831 (PANTHER), SignalP-NN(euk) (SIGNALP), SSF48726 (SUPERFAMILY) | Yes |
| 1459 | lib_dib_Filter15_IDBA_contig_25475_minus1 | 142 | 3-ketodihydrosphingosine reductase | 396 | 1.94E-142 | 77.70% | 8 | C:endoplasmic reticulum; C:extracellular space; F:catalytic activity; P:biosynthetic process; P:lipid metabolic process; F:binding; F:nucleotide binding; P:metabolic process | IPR002198 (PRINTS); IPR002347 (PRINTS); IPR016040 (G3DSA:3.40.50.GENE3D); IPR020842 (SMART); IPR020904 (PROSITE); PTHR24320 (PANTHER), PTHR24320:SF1 (PANTHER), SignalP-NN(euk) (SIGNALP), tmhmm (TMHMM), SSF51735 (SUPERFAMILY) | |
| 1460 | lib_dib_Filter15_IDBA_contig_25509_plus2 | 266 | predicted protein | 587 | 1.22E-04 | 70.00% | 0 | - | tmhmm (TMHMM) | |

| | | | | | | | | | | |
|---|---|---|---|---|---|---|---|---|---|---|
| 1461 | lib_dib_Filter15_IDBA_contig_25527_minus1 | 75 | ---NA--- | 395 | | | 0 | - | no IPS match | |
| 1462 | lib_dib_Filter15_IDBA_contig_25544_minus2 | 89 | syntaxin-16-like isoform 4 | 394 | 2.98E-133 | 76.35% | 4 | C:cell; F:protein binding; P:protein transport; P:transport | IPR000727 (PFAM); IPR006011 (PFAM); IPR006012 (PROSITE); IPR010989 (SUPERFAMILY); G3DSA:1.20.58.70 (GENE3D), PTHR19957 (PANTHER), PTHR19957:SF5 (PANTHER), tmhmm (TMHMM) | |
| 1463 | lib_dib_Filter15_IDBA_contig_25562_minus1 | 259 | cd109 antigen | 773 | 0 | 52.50% | 1 | C:extracellular space | IPR008930 (SUPERFAMILY); IPR009048 (G3DSA:2.60.40.GENE3D); IPR011626 (PFAM); G3DSA:1.50.10.20 (GENE3D), PTHR11412 (PANTHER), PTHR11412:SF22 (PANTHER) | |
| 1464 | lib_dib_Filter15_IDBA_contig_25563_plus3 | 14440 | calmodulin-like protein 12 | 1064 | 3.85E-61 | 55.00% | 4 | P:biological_process; P:response to abiotic stimulus; C:cell; F:calcium ion binding | IPR002048 (SMART); IPR011992 (G3DSA:1.10.238.GENE3D); IPR018247 (PROSITE); PTHR10891 (PANTHER), SSF47473 (SUPERFAMILY) | |
| 1465 | lib_dib_Filter15_IDBA_contig_25633_minus3 | 396 | plasma kallikrein | 461 | 3.17E-101 | 50.80% | 6 | P:response to external stimulus; P:regulation of biological process; P:protein metabolic process; P:response to stress; F:peptidase activity; P:catabolic process | IPR001254 (PFAM); IPR001314 (PRINTS); IPR009003 (SUPERFAMILY); IPR018114 (PROSITE); G3DSA:2.40.10.10 (GENE3D), PTHR24260 (PANTHER) | |
| 1466 | lib_dib_Filter15_IDBA_contig_25640_plus2 | 1921 | adam family mig-17 | 553 | 2.19E-22 | 44.05% | 3 | F:peptidase activity; P:protein metabolic process; P:catabolic process | IPR001590 (PROFILE); IPR024079 (G3DSA:3.40.390.GENE3D); PTHR13723 (PANTHER), PF13574 (PFAM), SSF55486 (SUPERFAMILY) | |
| 1467 | lib_dib_Filter15_IDBA_contig_25665_plus3 | 54 | protein sidekick-1 | 392 | 4.89E-84 | 56.10% | 1 | F:protein binding | IPR003961 (PFAM); IPR013783 (G3DSA:2.60.40.GENE3D); PR00014 (PRINTS), PTHR10489 (PANTHER), PTHR10489:SF36 (PANTHER) | |
| 1468 | lib_dib_Filter15_IDBA_contig_25691_minus1 | 1226 | fibropellin ib | 392 | 9.18E-83 | 57.80% | 3 | C:extracellular region; F:protein binding; F:calcium ion binding | IPR000152 (PROSITE); IPR000742 (PFAM); IPR001881 (SMART); IPR013032 (PFAM); IPR018097 (PROSITE); PD936484 (PRODOM), G3DSA:2.10.25.10 (GENE3D), PTHR24044 (PANTHER), SignalP-NN(euk) (SIGNALP), tmhmm (TMHMM), SSF57196 (SUPERFAMILY) | Yes |
| 1469 | lib_dib_Filter15_IDBA_contig_25805_minus1 | 1451 | deleted in malignant brain tumors 1 | 1141 | 3.79E-78 | 44.45% | 2 | F:receptor activity; C:cell | IPR000519 (G3DSA:4.10.110.GENE3D); IPR000859 (G3DSA:2.60.120.GENE3D); IPR001190 (PRINTS); IPR001846 (PFAM); IPR017448 (SMART); G3DSA:3.10.250.10 (GENE3D), PTHR19331 (PANTHER), PTHR19331:SF118 (PANTHER), SignalP-NN(euk) (SIGNALP), tmhmm (TMHMM) | |
| 1470 | lib_dib_Filter15_IDBA_contig_25806_minus1 | 1510 | deleted in malignant brain tumors 1 | 1095 | 2.57E-78 | 44.15% | 2 | F:receptor activity; C:cell | IPR000436 (PFAM); IPR000519 (G3DSA:4.10.110.GENE3D); IPR000859 (G3DSA:2.60.120.GENE3D); IPR001190 (PRINTS); IPR017448 (SMART); G3DSA:2.10.70.10 (GENE3D), G3DSA:3.10.250.10 (GENE3D), PTHR19331 (PANTHER), PTHR19331:SF118 (PANTHER), SignalP-NN(euk) (SIGNALP), tmhmm (TMHMM) | |
| 1471 | lib_dib_Filter15_IDBA_contig_25807_minus1 | 676 | deleted in malignant brain tumors 1 | 567 | 5.50E-37 | 50.50% | 2 | F:receptor activity; C:cell | IPR000519 (G3DSA:4.10.110.GENE3D); IPR000859 (G3DSA:2.60.120.GENE3D); IPR001190 (PRINTS); IPR017448 (SMART); G3DSA:3.10.250.10 (GENE3D), PTHR19331 (PANTHER), PTHR19331:SF118 (PANTHER), SignalP-NN(euk) (SIGNALP), tmhmm (TMHMM) | |
| 1472 | lib_dib_Filter15_IDBA_contig_25808_minus2 | 57 | lysyl oxidase homolog 4 | 104 | 3.54E-24 | 60.40% | 2 | F:receptor activity; C:cell | IPR001190 (PRINTS); IPR017448 (SMART); G3DSA:3.10.250.10 (GENE3D), PTHR19331 (PANTHER), PTHR19331:SF118 (PANTHER) | |

| | | | | | | | | | | |
|---|---|---|---|---|---|---|---|---|---|---|
| 1473 | lib_dib_Filter15_IDBA_contig_25809_minus3 | 29 | deleted in malignant brain tumors 1 protein | 140 | 1.06E-20 | 55.30% | 2 | F:receptor activity; C:cell | IPR001190 (PRINTS); IPR017448 (SMART); G3DSA:3.10.250.10 (GENE3D), PTHR19331 (PANTHER), PTHR19331:SF118 (PANTHER) | |
| 1474 | lib_dib_Filter15_IDBA_contig_2581_plus2 | 730 | cub and sushi domain-containing protein partial | 671 | 6.97E-57 | 45.50% | 1 | F:calcium ion binding | IPR000436 (PFAM); IPR001881 (SMART); IPR018097 (PROSITE); G3DSA:2.10.25.10 (GENE3D), G3DSA:2.10.70.10 (GENE3D), PTHR19325 (PANTHER) | |
| 1475 | lib_dib_Filter15_IDBA_contig_25826_plus1 | 51 | zinc finger | 391 | 2.67E-61 | 71.45% | 12 | P:regulation of biological process; ; P:reproduction; F:binding; P:multicellular organismal development; P:cell differentiation; P:anatomical structure morphogenesis; P:cellular component organization; F:sequence-specific DNA binding transcription factor activity; P:biological_process; P:response to biotic stimulus; C:nucleus | IPR007087 (PFAM); IPR013087 (G3DSA:3.30.160.GENE3D); IPR015880 (SMART); PTHR24391 (PANTHER), PTHR24391:SF0 (PANTHER), PF13465 (PFAM), PF13894 (PFAM), SignalP-NN(euk) (SIGNALP), SSF57667 (SUPERFAMILY) | Yes |
| 1476 | lib_dib_Filter15_IDBA_contig_25856_plus3 | 3308 | nicotinic acetylcholine receptor subunit alpha 6 transcript variant partial | 684 | 4.14E-68 | 57.05% | 5 | F:ion channel activity; P:biological_process; C:protein complex; C:plasma membrane; P:ion transport | IPR006029 (SUPERFAMILY); IPR006201 (PRINTS); IPR006202 (G3DSA:2.70.170.GENE3D); G3DSA:1.20.120.370 (GENE3D), PTHR18945:SF92 (PANTHER), tmhmm (TMHMM) | |
| 1477 | lib_dib_Filter15_IDBA_contig_25885_minus1 | 106 | slit protein | 390 | 5.79E-92 | 55.65% | 1 | F:protein binding | IPR000742 (SMART); IPR001791 (PFAM); IPR006207 (SMART); IPR006208 (PFAM); IPR008985 (SUPERFAMILY); IPR013032 (PROSITE); IPR013320 (G3DSA:2.60.120.GENE3D); G3DSA:2.10.25.10 (GENE3D), PTHR24373 (PANTHER), PTHR24373:SF8 (PANTHER), SSF57196 (SUPERFAMILY) | |
| 1478 | lib_dib_Filter15_IDBA_contig_25886_minus2 | 142 | f-type lectin | 465 | 1.11E-24 | 45.25% | 1 | P:biological_process | IPR000421 (PFAM); IPR000562 (G3DSA:2.10.10.GENE3D); IPR006585 (SMART); IPR008979 (SUPERFAMILY); IPR013806 (SUPERFAMILY); G3DSA:2.60.120.260 (GENE3D), PTHR19277 (PANTHER), PTHR19277:SF6 (PANTHER), SignalP-NN(euk) (SIGNALP), tmhmm (TMHMM) | Yes |
| 1479 | lib_dib_Filter15_IDBA_contig_25887_minus2 | 126 | fucose binding lectin | 442 | 9.63E-25 | 45.15% | 1 | P:biological_process | IPR000421 (PFAM); IPR000562 (G3DSA:2.10.10.GENE3D); IPR006585 (SMART); IPR008979 (SUPERFAMILY); IPR013806 (SUPERFAMILY); G3DSA:2.60.120.260 (GENE3D), PTHR19277 (PANTHER), PTHR19277:SF6 (PANTHER), SignalP-NN(euk) (SIGNALP), tmhmm (TMHMM) | Yes |
| 1480 | lib_dib_Filter15_IDBA_contig_25917_plus1 | 63 | sticks and isoform a | 389 | 1.06E-75 | 52.35% | 6 | P:biological_process; P:multicellular organismal development; P:cell differentiation; P:anatomical structure morphogenesis; C:cell; F:protein binding | IPR003598 (SMART); IPR003599 (SMART); IPR007110 (PROFILE); IPR013098 (PFAM); IPR013783 (G3DSA:2.60.40.GENE3D); PTHR11640 (PANTHER), PTHR11640:SF7 (PANTHER), PF13895 (PFAM), SSF48726 (SUPERFAMILY) | |
| 1481 | lib_dib_Filter15_IDBA_contig_25923_minus1 | 271 | glutaminyl-peptide cyclotransferase | 632 | 3.80E-76 | 58.90% | 6 | P:cellular protein modification process; F:transferase activity; F:binding; P:protein metabolic process; P:catabolic process; F:peptidase activity | IPR007484 (PFAM); G3DSA:3.40.630.10 (GENE3D), PTHR12283 (PANTHER), PTHR12283:SF0 (PANTHER), SignalP-NN(euk) (SIGNALP), tmhmm (TMHMM), SSF53187 (SUPERFAMILY) | |
| 1482 | lib_dib_Filter15_IDBA_contig_25924_minus1 | 279 | glutaminyl-peptide cyclotransferase | 633 | 3.22E-76 | 58.75% | 6 | P:cellular protein modification process; F:transferase activity; F:binding; P:protein metabolic process; P:catabolic process; F:peptidase activity | IPR007484 (PFAM); G3DSA:3.40.630.10 (GENE3D), PTHR12283 (PANTHER), PTHR12283:SF0 (PANTHER), SignalP-NN(euk) (SIGNALP), tmhmm (TMHMM), SSF53187 (SUPERFAMILY) | |

| | | | | | | | | | |
|---|---|---|---|---|---|---|---|---|---|
| 1483 | lib_dib_Filter15_IDBA_contig_25939_plus2 | 60 | alpha- -mannosyl-glycoprotein 4-beta-n-acetylglucosaminyltransferase b | 389 | 6.63E-134 | 76.30% | 5 | C:Golgi apparatus; P:biosynthetic process; P:cellular protein modification process; P:carbohydrate metabolic process; F:transferase activity | IPR006759 (PANTHER) |
| 1484 | lib_dib_Filter15_IDBA_contig_25954_minus1 | 5646 | nf-kappa-b inhibitor epsilon | 929 | 1.11E-39 | 54.20% | 1 | F:protein binding | IPR002110 (SMART); IPR020683 (G3DSA:1.25.40.GENE3D); PTHR24128 (PANTHER), PTHR24128:SF1 (PANTHER) |
| 1485 | lib_dib_Filter15_IDBA_contig_25955_minus1 | 7949 | nf-kappa-b inhibitor epsilon | 1426 | 6.44E-39 | 54.80% | 1 | F:protein binding | IPR002110 (SMART); IPR020683 (G3DSA:1.25.40.GENE3D); PTHR24128 (PANTHER), PTHR24128:SF1 (PANTHER), tmhmm (TMHMM) |
| 1486 | lib_dib_Filter15_IDBA_contig_25956_minus1 | 7081 | nf-kappa-b inhibitor epsilon | 1454 | 8.72E-39 | 54.45% | 1 | F:protein binding | IPR002110 (SMART); IPR020683 (G3DSA:1.25.40.GENE3D); PTHR24128 (PANTHER), PTHR24128:SF1 (PANTHER), tmhmm (TMHMM) |
| 1487 | lib_dib_Filter15_IDBA_contig_25957_minus1 | 7034 | nf-kappa-b inhibitor epsilon | 1442 | 6.23E-39 | 54.20% | 1 | F:protein binding | IPR002110 (SMART); IPR020683 (G3DSA:1.25.40.GENE3D); PTHR24128 (PANTHER), PTHR24128:SF1 (PANTHER) |
| 1488 | lib_dib_Filter15_IDBA_contig_25960_minus3 | 54 | small gtp-binding protein | 400 | 1.06E-16 | 58.45% | 1 | F:protein binding | IPR001611 (PROFILE); IPR003591 (SMART); G3DSA:3.80.10.10 (GENE3D), PTHR23155 (PANTHER), PF13855 (PFAM), SSF52058 (SUPERFAMILY) |
| 1489 | lib_dib_Filter15_IDBA_contig_25965_plus2 | 635 | delta protein | 951 | 1.55E-66 | 42.80% | 14 | P:cell differentiation; P:anatomical structure morphogenesis; P:multicellular organismal development; P:biological_process; P:reproduction; P:embryo development; P:regulation of biological process; F:protein binding; F:DNA binding; C:intracellular; P:cell communication; C:cell; ; F:calcium ion binding | IPR000152 (PROSITE); IPR000742 (PFAM); IPR001774 (PFAM); IPR001881 (SMART); IPR013032 (PROSITE); IPR018097 (PROSITE); G3DSA:2.10.25.10 (GENE3D), PTHR24838 (PANTHER), tmhmm (TMHMM), SSF57196 (SUPERFAMILY) |
| 1490 | lib_dib_Filter15_IDBA_contig_25967_plus2 | 129 | neurogenic locus notch homolog protein 2-like | 382 | 5.93E-66 | 45.45% | 5 | F:protein binding; F:calcium ion binding; P:signal transduction; P:multicellular organismal development; C:cell | IPR000152 (PROSITE); IPR000742 (PFAM); IPR001774 (PFAM); IPR001881 (PFAM); IPR011651 (PFAM); IPR013032 (PROSITE); IPR018097 (PROSITE); G3DSA:2.10.25.10 (GENE3D), PTHR24044 (PANTHER), SSF57196 (SUPERFAMILY) |
| 1491 | lib_dib_Filter15_IDBA_contig_26007_plus3 | 678 | fibrinogen c domain-containing protein 1-like | 387 | 3.29E-51 | 60.15% | 3 | F:receptor binding; C:extracellular space; P:signal transduction | IPR002181 (PFAM); IPR014715 (G3DSA:4.10.530.GENE3D); IPR014716 (G3DSA:3.90.215.GENE3D); PTHR19143 (PANTHER), tmhmm (TMHMM) |
| 1492 | lib_dib_Filter15_IDBA_contig_26015_plus1 | 16925 | ---NA--- | 1668 | | | 0 | - | tmhmm (TMHMM) |
| 1493 | lib_dib_Filter15_IDBA_contig_26031_plus3 | 1979 | dnaj homolog subfamily c member 4-like | 1083 | 4.87E-29 | 51.75% | 8 | F:unfolded protein binding; P:protein folding; F:heat shock protein binding; C:mitochondrion; P:response to unfolded protein; C:integral to membrane; C:membrane; C:membrane fraction | IPR001623 (PRINTS); PTHR24078 (PANTHER), PTHR24078:SF22 (PANTHER), SignalP-NN(euk) (SIGNALP), tmhmm (TMHMM) |
| 1494 | lib_dib_Filter15_IDBA_contig_26038_plus3 | 84 | u5 small nuclear ribonucleoprotein 40 kda protein | 387 | 0 | 83.85% | 8 | C:cytoplasm; C:nucleolus; C:nucleus; C:nucleoplasm; F:nucleic acid binding; C:intracellular; F:protein binding; P:nucleobase-containing compound metabolic process | IPR001680 (PFAM); IPR015943 (G3DSA:2.130.10.GENE3D); IPR017986 (PROFILE); IPR019775 (PROSITE); IPR020472 (PRINTS); PTHR22847 (PANTHER), PTHR22847:SF47 (PANTHER) |
| 1495 | lib_dib_Filter15_IDBA_contig_26039_plus2 | 845 | crumbs homolog 1 | 387 | 3.65E-13 | 59.20% | 2 | F:protein binding; F:calcium ion binding | IPR000152 (PROSITE); IPR000742 (PFAM); IPR001881 (PFAM); IPR013032 (PFAM); IPR018097 (PROSITE); PR00010 (PRINTS), G3DSA:2.10.25.10 (GENE3D), PTHR24044 (PANTHER), SSF57196 (SUPERFAMILY) |

| | | | | | | | | | |
|---|---|---|---|---|---|---|---|---|---|
| 1496 | lib_dib_Filter15_IDBA_contig_26041_plus1 | 70 | protein | 283 | 7.05E-25 | 63.60% | 2 | F:protein binding; F:calcium ion binding | IPR000152 (PROSITE); IPR000742 (PFAM); IPR001881 (PFAM); IPR013032 (PROSITE); IPR018097 (PROSITE); PR00010 (PRINTS), G3DSA:2.10.25.10 (GENE3D), PTHR24048 (PANTHER), PTHR24048:SF15 (PANTHER), SSF57196 (SUPERFAMILY) |
| 1497 | lib_dib_Filter15_IDBA_contig_26046_minus3 | 73 | dehydrogenase reductase sdr family protein 7-like | 387 | 3.43E-97 | 72.15% | 5 | C:cell; C:peroxisome; P:metabolic process; F:catalytic activity; F:nucleotide binding | IPR002198 (PRINTS); IPR002347 (PRINTS); IPR016040 (G3DSA:3.40.50.GENE3D); PTHR24322 (PANTHER), PTHR24322:SF27 (PANTHER), tmhmm (TMHMM), SSF51735 (SUPERFAMILY) |
| 1498 | lib_dib_Filter15_IDBA_contig_26057_minus2 | 828 | carbonyl reductase | 945 | 1.14E-113 | 71.70% | 3 | P:metabolic process; F:catalytic activity; F:nucleotide binding | IPR002198 (PFAM); IPR002347 (PRINTS); IPR016040 (G3DSA:3.40.50.GENE3D); PTHR24322 (PANTHER), PTHR24322:SF58 (PANTHER), SSF51735 (SUPERFAMILY) |
| 1499 | lib_dib_Filter15_IDBA_contig_26058_minus2 | 544 | carbonyl reductase | 640 | 8.09E-117 | 71.85% | 3 | P:metabolic process; F:catalytic activity; F:nucleotide binding | IPR002198 (PFAM); IPR002347 (PRINTS); IPR016040 (G3DSA:3.40.50.GENE3D); PTHR24322 (PANTHER), PTHR24322:SF58 (PANTHER), SSF51735 (SUPERFAMILY) |
| 1500 | lib_dib_Filter15_IDBA_contig_26061_plus3 | 34872 | soma ferritin-like | 614 | 2.95E-35 | 62.15% | 5 | P:ion transport; P:cellular homeostasis; F:binding; F:catalytic activity; P:metabolic process | IPR001519 (PANTHER); IPR008331 (PFAM); IPR009040 (PROFILE); IPR009078 (SUPERFAMILY); IPR012347 (G3DSA:1.20.1260.GENE3D) |
| 1501 | lib_dib_Filter15_IDBA_contig_26062_plus1 | 22687 | soma ferritin-like | 423 | 2.43E-28 | 63.80% | 5 | P:ion transport; P:cellular homeostasis; F:binding; F:catalytic activity; P:metabolic process | IPR001519 (PANTHER); IPR008331 (PFAM); IPR009040 (PROFILE); IPR009078 (SUPERFAMILY); IPR012347 (G3DSA:1.20.1260.GENE3D); PTHR11431:SF8 (PANTHER) |
| 1502 | lib_dib_Filter15_IDBA_contig_26064_plus3 | 279 | ferritin heavy chain 1 | 386 | 7.76E-28 | 56.65% | 5 | P:ion transport; P:cellular homeostasis; F:binding; F:catalytic activity; P:metabolic process | IPR001519 (PANTHER); IPR008331 (PFAM); IPR009040 (PROFILE); IPR009078 (SUPERFAMILY); IPR012347 (G3DSA:1.20.1260.GENE3D) |
| 1503 | lib_dib_Filter15_IDBA_contig_2612_minus2 | 358 | fibrinogen alpha chain | 392 | 7.83E-28 | 59.60% | 6 | P:biological_process; C:plasma membrane; F:binding; C:extracellular space; C:cytoplasmic membrane-bounded vesicle; F:protein binding | IPR002181 (PFAM); IPR014715 (G3DSA:4.10.530.GENE3D); IPR014716 (G3DSA:3.90.215.GENE3D); PTHR19143 (PANTHER) |
| 1504 | lib_dib_Filter15_IDBA_contig_26146_plus2 | 449 | map kinase-activated protein kinase 2 | 633 | 0 | 82.05% | 16 | P:signal transduction; P:response to stress; P:cell cycle; P:response to biotic stimulus; P:regulation of biological process; P:nucleobase-containing compound metabolic process; F:protein binding; F:protein kinase activity; C:cytoplasm; P:cellular protein modification process; P:multicellular organismal development; P:anatomical structure morphogenesis; P:embryo development; P:response to external stimulus; C:nucleus; F:nucleotide binding | IPR000719 (PFAM); IPR002290 (SMART); IPR008271 (PROSITE); IPR011009 (SUPERFAMILY); IPR017441 (PROSITE); IPR020635 (SMART); G3DSA:1.10.510.10 (GENE3D), G3DSA:3.30.200.20 (GENE3D), G3DSA:4.10.1170.10 (GENE3D), PTHR24349 (PANTHER), PTHR24349:SF63 (PANTHER) |
| 1505 | lib_dib_Filter15_IDBA_contig_26155_minus3 | 228 | arylsulfatase b | 697 | 8.10E-105 | 55.90% | 2 | P:metabolic process; F:hydrolase activity | IPR000917 (PFAM); IPR017849 (G3DSA:3.40.720.GENE3D); IPR017850 (SUPERFAMILY); G3DSA:3.30.1120.10 (GENE3D), PTHR10342 (PANTHER), PTHR10342:SF19 (PANTHER), SignalP-NN(euk) (SIGNALP), tmhmm (TMHMM) |

| | | | | | | | | | | |
|---|---|---|---|---|---|---|---|---|---|---|
| 1506 | lib_dib_Filter15_IDBA_contig_26156_minus3 | 75 | arylsulfatase b | 246 | 1.67E-43 | 68.05% | 2 | F:hydrolase activity; P:metabolic process | IPR000917 (PFAM); IPR017849 (G3DSA:3.40.720.GENE3D); IPR017850 (SUPERFAMILY); PTHR10342 (PANTHER), PTHR10342:SF19 (PANTHER), SignalP-NN(euk) (SIGNALP), tmhmm (TMHMM) | |
| 1507 | lib_dib_Filter15_IDBA_contig_26171_plus1 | 313 | fibroblast growth factor 1 | 821 | 1.25E-09 | 54.45% | 5 | P:anatomical structure morphogenesis; P:multicellular organismal development; C:cytoplasm; P:regulation of biological process; F:receptor binding | IPR002348 (PANTHER); IPR008996 (SUPERFAMILY); G3DSA:2.80.10.50 (GENE3D) | |
| 1508 | lib_dib_Filter15_IDBA_contig_26217_plus2 | 255 | carbonic anhydrase | 586 | 3.16E-55 | 58.05% | 8 | P:transport; P:response to stress; P:response to abiotic stimulus; F:catalytic activity; P:biological_process; P:ion transport; P:metabolic process; F:binding | IPR001148 (G3DSA:3.10.200.GENE3D); IPR018338 (PROSITE); IPR023561 (PANTHER); tmhmm (TMHMM) | |
| 1509 | lib_dib_Filter15_IDBA_contig_26226_minus3 | 95 | acid sphingomyelinase-like phosphodiesterase 3a-like | 384 | 9.63E-58 | 51.55% | 1 | F:hydrolase activity | IPR004843 (PFAM); G3DSA:3.60.21.10 (GENE3D), PTHR10340 (PANTHER), SignalP-NN(euk) (SIGNALP), SSF56300 (SUPERFAMILY) | |
| 1510 | lib_dib_Filter15_IDBA_contig_26230_minus2 | 149 | sulfhydryl oxidase 1 | 467 | 7.48E-88 | 51.95% | 7 | C:Golgi apparatus; F:catalytic activity; C:cell; C:extracellular region; P:metabolic process; P:cellular homeostasis; P:regulation of biological process | IPR006863 (G3DSA:1.20.120.GENE3D); IPR012336 (G3DSA:3.40.30.GENE3D); IPR013766 (PFAM); IPR017905 (PROFILE); PTHR22897 (PANTHER), SignalP-NN(euk) (SIGNALP), tmhmm (TMHMM) | Yes |
| 1511 | lib_dib_Filter15_IDBA_contig_26232_minus3 | 31 | sulfhydryl oxidase 1 | 204 | 5.63E-45 | 63.10% | 5 | P:cellular protein modification process; C:extracellular space; F:catalytic activity; C:Golgi apparatus; P:metabolic process | IPR006863 (G3DSA:1.20.120.GENE3D); IPR017905 (PROFILE); PTHR22897 (PANTHER), SignalP-NN(euk) (SIGNALP) | Yes |
| 1512 | lib_dib_Filter15_IDBA_contig_26256_minus1 | 49 | protein | 384 | 1.02E-11 | 51.00% | 1 | F:protein binding | IPR000742 (PFAM); IPR013032 (PROSITE); G3DSA:2.10.25.10 (GENE3D), PTHR24033 (PANTHER), tmhmm (TMHMM), SSF57196 (SUPERFAMILY) | |
| 1513 | lib_dib_Filter15_IDBA_contig_26258_minus2 | 667 | mitogen-activated protein kinase kinase kinase 7 | 915 | 0 | 62.00% | 14 | P:signal transduction; P:anatomical structure morphogenesis; P:embryo development; C:cell; F:protein kinase activity; P:cellular protein modification process; P:regulation of biological process; P:cell death; F:signal transducer activity; F:protein binding; F:nucleotide binding; C:intracellular; P:response to stress; P:multicellular organismal development | IPR000719 (PROFILE); IPR001245 (PRINTS); IPR002290 (SMART); IPR008271 (PROSITE); IPR011009 (SUPERFAMILY); IPR017441 (PROSITE); IPR020635 (SMART); G3DSA:1.10.510.10 (GENE3D), G3DSA:3.30.200.20 (GENE3D), PTHR26392 (PANTHER), PTHR26392:SF68 (PANTHER), SignalP-NN(euk) (SIGNALP), tmhmm (TMHMM) | |
| 1514 | lib_dib_Filter15_IDBA_contig_26260_plus2 | 66 | fibrinogen and fibronectin | 384 | 3.63E-10 | 44.60% | 1 | F:protein binding | IPR000742 (PROFILE); IPR002181 (PFAM); IPR013032 (PROSITE); IPR014716 (G3DSA:3.90.215.GENE3D); G3DSA:2.170.300.10 (GENE3D), PTHR19143 (PANTHER), PTHR19143:SF87 (PANTHER) | |
| 1515 | lib_dib_Filter15_IDBA_contig_26300_plus3 | 3509 | fibropellin ib | 479 | 2.26E-69 | 58.95% | 3 | C:extracellular region; F:protein binding; F:calcium ion binding | IPR000152 (PROSITE); IPR000742 (PFAM); IPR001881 (SMART); IPR002035 (PFAM); IPR013032 (PFAM); IPR018097 (PROSITE); PD936484 (PRODOM), G3DSA:2.10.25.10 (GENE3D), G3DSA:2.170.300.10 (GENE3D), G3DSA:3.40.50.410 (GENE3D), PTHR24048 (PANTHER), PTHR24048:SF15 (PANTHER), SSF53300 (SUPERFAMILY), SSF57196 (SUPERFAMILY) | |
| 1516 | lib_dib_Filter15_IDBA_contig_26310_plus3 | 354 | protein | 689 | 9.22E-173 | 54.25% | 4 | F:peptidase activity; P:protein metabolic process; P:catabolic process; F:binding | IPR000834 (PFAM); G3DSA:3.40.630.10 (GENE3D), PTHR11705 (PANTHER), tmhmm (TMHMM), SSF53187 (SUPERFAMILY) | |
| 1517 | lib_dib_Filter15_IDBA_contig_2632_plus2 | 4045 | chorion peroxidase | 219 | 8.58E-59 | 56.50% | 5 | F:catalytic activity; F:antioxidant activity; P:response to stress; F:binding; P:metabolic process | IPR002007 (G3DSA:1.10.640.GENE3D); IPR010255 (SUPERFAMILY); PTHR11475 (PANTHER) | |

| | | | | | | | | | | |
|---|---|---|---|---|---|---|---|---|---|---|
| 1518 | lib_dib_Filter15_IDBA_contig_26343_minus3 | 98 | neuroendocrine protein 7b2 | 388 | 4.62E-67 | 51.70% | 3 | P:biological_process; P:signal transduction; C:cytoplasmic membrane-bounded vesicle | IPR007945 (PANTHER); PTHR12738:SF0 (PANTHER), SignalP-NN(euk) (SIGNALP), tmhmm (TMHMM) | |
| 1519 | lib_dib_Filter15_IDBA_contig_26346_plus3 | 234 | cubilin-like | 910 | 0 | 62.60% | 2 | F:protein binding; F:calcium ion binding | IPR000742 (PFAM); IPR000859 (G3DSA:2.60.120.GENE3D); IPR001881 (PFAM); IPR013032 (PROSITE); IPR018097 (PROSITE); G3DSA:2.10.25.10 (GENE3D), PTHR10127 (PANTHER), PTHR10127:SF310 (PANTHER), SSF57196 (SUPERFAMILY) | |
| 1520 | lib_dib_Filter15_IDBA_contig_26347_plus1 | 254 | cubilin | 1008 | 0 | 62.50% | 2 | F:protein binding; F:calcium ion binding | IPR000152 (PROSITE); IPR000742 (PFAM); IPR000859 (G3DSA:2.60.120.GENE3D); IPR001881 (PFAM); IPR013032 (PROSITE); IPR018097 (PROSITE); G3DSA:2.10.25.10 (GENE3D), PTHR10127 (PANTHER), PTHR10127:SF310 (PANTHER), SSF57196 (SUPERFAMILY) | |
| 1521 | lib_dib_Filter15_IDBA_contig_26348_plus3 | 281 | cubilin | 1155 | 0 | 60.10% | 2 | F:protein binding; F:calcium ion binding | IPR000152 (PROSITE); IPR000742 (PFAM); IPR000859 (G3DSA:2.60.120.GENE3D); IPR001881 (PFAM); IPR013032 (PROSITE); IPR018097 (PROSITE); G3DSA:2.10.25.10 (GENE3D), PTHR10127 (PANTHER), PTHR10127:SF310 (PANTHER), SSF57196 (SUPERFAMILY) | |
| 1522 | lib_dib_Filter15_IDBA_contig_26349_plus2 | 199 | tolloid-like protein 2 | 467 | 3.32E-46 | 46.55% | 1 | F:hydrolase activity | IPR000859 (G3DSA:2.60.120.GENE3D); PTHR10127 (PANTHER) | |
| 1523 | lib_dib_Filter15_IDBA_contig_26350_plus2 | 241 | tolloid-like protein 2 | 567 | 2.09E-52 | 47.05% | 5 | F:calcium ion binding; F:molecular_function; P:extracellular matrix organization; P:chitin localization; C:cellular_component | IPR000859 (G3DSA:2.60.120.GENE3D); PTHR10127 (PANTHER) | |
| 1524 | lib_dib_Filter15_IDBA_contig_26351_plus1 | 66 | dorsal-ventral patterning protein tolloid | 171 | 3.90E-25 | 51.10% | 2 | C:intracellular; F:catalytic activity | IPR000859 (G3DSA:2.60.120.GENE3D); PTHR10127 (PANTHER) | |
| 1525 | lib_dib_Filter15_IDBA_contig_26352_plus1 | 56 | extracellular serine proteinase-like | 189 | 2.72E-27 | 51.85% | 1 | F:catalytic activity | IPR000859 (G3DSA:2.60.120.GENE3D); PTHR10127 (PANTHER) | |
| 1526 | lib_dib_Filter15_IDBA_contig_26387_plus2 | 92 | ---NA--- | 242 | | | 0 | - | PTHR23304 (PANTHER) | |
| 1527 | lib_dib_Filter15_IDBA_contig_26437_plus1 | 53 | sticks and isoform a | 382 | 3.47E-59 | 56.20% | 8 | P:biological_process; P:anatomical structure morphogenesis; P:multicellular organismal development; P:reproduction; P:cell differentiation; C:plasma membrane; P:cytoskeleton organization; F:protein binding | IPR003598 (SMART); IPR003599 (SMART); IPR007110 (PROFILE); IPR013098 (PFAM); IPR013162 (PFAM); IPR013783 (G3DSA:2.60.40.GENE3D); PTHR11640 (PANTHER), PF13895 (PFAM), SSF48726 (SUPERFAMILY) | |
| 1528 | lib_dib_Filter15_IDBA_contig_26474_plus1 | 523 | neurotrypsin-like | 513 | 5.89E-48 | 47.70% | 3 | F:catalytic activity; F:receptor activity; C:cell | IPR000519 (G3DSA:4.10.110.GENE3D); IPR001190 (PRINTS); IPR017448 (SMART); G3DSA:3.10.250.10 (GENE3D), PTHR19331 (PANTHER), PTHR19331:SF118 (PANTHER), SignalP-NN(euk) (SIGNALP), tmhmm (TMHMM) | |
| 1529 | lib_dib_Filter15_IDBA_contig_26475_plus1 | 487 | neurotrypsin-like | 493 | 4.48E-47 | 47.60% | 3 | F:catalytic activity; F:receptor activity; C:cell | IPR000519 (G3DSA:4.10.110.GENE3D); IPR001190 (PRINTS); IPR017448 (SMART); G3DSA:3.10.250.10 (GENE3D), PTHR19331 (PANTHER), PTHR19331:SF118 (PANTHER), SignalP-NN(euk) (SIGNALP), tmhmm (TMHMM) | Yes |
| 1530 | lib_dib_Filter15_IDBA_contig_26478_minus3 | 87 | plexin a | 381 | 1.39E-127 | 87.45% | 5 | C:cell; P:signal transduction; P:multicellular organismal development; F:receptor activity; C:intracellular | IPR008936 (SUPERFAMILY); IPR013548 (PFAM); PTHR22625 (PANTHER), PTHR22625:SF12 (PANTHER) | |

| | | | | | | | | | | |
|---|---|---|---|---|---|---|---|---|---|---|
| 1531 | lib_dib_Filter15_IDBA_contig_26533_minus3 | 652 | protein mom-5 | 695 | 3.09E-33 | 54.35% | 15 | P:multicellular organismal development; P:signal transduction; P:behavior; P:reproduction; F:protein binding; P:transport; P:cellular component organization; P:regulation of biological process; P:growth; P:cytoskeleton organization; P:cell cycle; P:biological_process; P:anatomical structure morphogenesis; P:cell differentiation; P:embryo development | IPR015526 (PANTHER); IPR020067 (G3DSA:1.10.2000.GENE3D); PTHR11309:SF43 (PANTHER) | |
| 1532 | lib_dib_Filter15_IDBA_contig_26548_plus2 | 198 | golgin subfamily a member 4 | 804 | 1.49E-35 | 55.15% | 3 | P:protein transport; C:Golgi apparatus; F:protein binding | IPR009053 (SUPERFAMILY); PTHR19327 (PANTHER) | |
| 1533 | lib_dib_Filter15_IDBA_contig_26549_plus1 | 198 | golgin subfamily a member 4 | 772 | 4.82E-33 | 56.20% | 3 | P:protein transport; C:Golgi apparatus; F:protein binding | PTHR19327 (PANTHER) | |
| 1534 | lib_dib_Filter15_IDBA_contig_26592_minus3 | 1715 | cathepsin l | 428 | 2.39E-155 | 74.30% | 3 | P:protein metabolic process; P:catabolic process; F:peptidase activity | IPR000169 (PROSITE); IPR000668 (PRINTS); IPR013128 (PANTHER); IPR013201 (PFAM); IPR025660 (PROSITE); IPR025661 (PROSITE); G3DSA:3.90.70.10 (GENE3D), PTHR12411:SF149 (PANTHER), SignalP-NN(euk) (SIGNALP), tmhmm (TMHMM), SSF54001 (SUPERFAMILY) | |
| 1535 | lib_dib_Filter15_IDBA_contig_26611_plus2 | 537 | cathepsin b | 623 | 2.21E-149 | 75.85% | 4 | P:protein metabolic process; P:catabolic process; F:peptidase activity; P:biological_process | IPR000169 (PROSITE); IPR000668 (PRINTS); IPR012599 (PFAM); IPR013128 (PANTHER); IPR015643 (PTHR12411:PANTHER); IPR025660 (PROSITE); IPR025661 (PROSITE); G3DSA:3.90.70.10 (GENE3D), tmhmm (TMHMM), SSF54001 (SUPERFAMILY) | |
| 1536 | lib_dib_Filter15_IDBA_contig_26617_plus2 | 405 | endothelin-converting enzyme 1 | 1009 | 0 | 57.40% | 8 | P:metabolic process; F:binding; C:cell; C:cytoplasmic membrane-bounded vesicle; P:biological_process; P:protein metabolic process; P:catabolic process; F:peptidase activity | IPR000718 (PANTHER); IPR008753 (PFAM); IPR018497 (PRINTS); IPR024079 (G3DSA:3.40.390.GENE3D); tmhmm (TMHMM), SSF55486 (SUPERFAMILY) | |
| 1537 | lib_dib_Filter15_IDBA_contig_26618_plus1 | 389 | endothelin-converting enzyme 1 | 956 | 0 | 57.35% | 8 | P:metabolic process; F:binding; C:cell; C:cytoplasmic membrane-bounded vesicle; P:biological_process; P:protein metabolic process; P:catabolic process; F:peptidase activity | IPR000718 (PANTHER); IPR008753 (PFAM); IPR018497 (PRINTS); IPR024079 (G3DSA:3.40.390.GENE3D); tmhmm (TMHMM), SSF55486 (SUPERFAMILY) | |
| 1538 | lib_dib_Filter15_IDBA_contig_26625_minus2 | 55 | beta- -galactosyl-o-glycosyl-glycoprotein beta- -n-acetylglucosaminyltransferase | 379 | 1.19E-124 | 56.70% | 2 | F:transferase activity; C:cell | IPR003406 (PFAM); PTHR19297 (PANTHER) | |
| 1539 | lib_dib_Filter15_IDBA_contig_26642_plus3 | 183 | canopy 4 homolog | 404 | 1.19E-81 | 70.50% | 1 | F:receptor binding | IPR021852 (PFAM); PTHR15382 (PANTHER), SignalP-NN(euk) (SIGNALP) | |
| 1540 | lib_dib_Filter15_IDBA_contig_26652_minus3 | 3039 | procollagen c-endopeptidase enhancer 2 precursor | 647 | 3.78E-09 | 49.40% | 20 | F:metal ion binding; P:proteolysis; F:metallopeptidase activity; F:calcium ion binding; F:hydrolase activity; F:zinc ion binding; F:peptidase activity; F:metalloendopeptidase activity; C:collagen; C:extracellular region; C:membrane; P:positive regulation of cartilage development; C:integral to membrane; F:extracellular ligand-gated ion channel activity; P:ion transport; P:transport; F:peptidase activator activity; P:positive regulation of peptidase activity; F:heparin binding; F:collagen binding | IPR000859 (G3DSA:2.60.120.GENE3D); PTHR10127 (PANTHER), tmhmm (TMHMM) | Yes |
| 1541 | lib_dib_Filter15_IDBA_contig_26660_plus3 | 28 | ankyrin motif protein | 378 | 8.54E-26 | 48.90% | 1 | F:protein binding | IPR002110 (PRINTS); IPR020683 (G3DSA:1.25.40.GENE3D); PTHR24198 (PANTHER) | |

| | | | | | | | | | |
|---|---|---|---|---|---|---|---|---|---|
| 1542 | lib_dib_Filter15_IDBA_contig_26669_plus1 | 1063 | delta protein | 1011 | 9.59E-116 | 45.70% | 14 | P:cell differentiation; P:anatomical structure morphogenesis; P:multicellular organismal development; P:biological_process; P:reproduction; P:embryo development; P:regulation of biological process; F:protein binding; F:DNA binding; C:intracellular; P:cell communication; C:cell; ; F:calcium ion binding | IPR000152 (PROSITE); IPR000742 (PFAM); IPR001774 (PFAM); IPR001881 (SMART); IPR013032 (PROSITE); IPR018097 (PROSITE); PD936484 (PRODOM), PR00010 (PRINTS), G3DSA:2.10.25.10 (GENE3D), PTHR24044 (PANTHER), tmhmm (TMHMM), SSF57196 (SUPERFAMILY) |
| 1543 | lib_dib_Filter15_IDBA_contig_26670_plus1 | 410 | versican core | 460 | 9.27E-12 | 76.60% | 8 | C:extracellular space; F:carbohydrate binding; P:multicellular organismal development; P:biological_process; F:calcium ion binding; C:proteinaceous extracellular matrix; P:cell differentiation; F:protein binding | IPR000152 (PROSITE); IPR000742 (PFAM); IPR001881 (SMART); IPR013032 (PROSITE); IPR018097 (PROSITE); G3DSA:2.10.25.10 (GENE3D), PTHR24838 (PANTHER), tmhmm (TMHMM), SSF57196 (SUPERFAMILY) |
| 1544 | lib_dib_Filter15_IDBA_contig_26737_plus3 | 180 | isoform a | 996 | 3.88E-48 | 38.15% | 2 | F:protein binding; F:calcium ion binding | IPR000152 (PROSITE); IPR000436 (PFAM); IPR000742 (SMART); IPR001881 (SMART); IPR003410 (PFAM); IPR009030 (SUPERFAMILY); IPR011641 (PFAM); G3DSA:2.10.25.10 (GENE3D), G3DSA:2.10.70.10 (GENE3D), PTHR24046 (PANTHER), SSF57196 (SUPERFAMILY) |
| 1545 | lib_dib_Filter15_IDBA_contig_26738_plus1 | 76 | gcc2 and gcc3 | 343 | 4.96E-13 | 42.75% | 3 | F:calcium ion binding; F:carbohydrate binding; P:cell adhesion | IPR009030 (SUPERFAMILY); IPR011641 (PFAM); PTHR24046 (PANTHER), PTHR24046:SF0 (PANTHER) |
| 1546 | lib_dib_Filter15_IDBA_contig_26749_plus1 | 129 | tyrosine kinase | 554 | 1.51E-152 | 73.85% | 12 | C:protein complex; C:plasma membrane; P:cell differentiation; P:multicellular organismal development; F:protein kinase activity; P:signal transduction; C:cytosol; P:cellular protein modification process; F:protein binding; F:nucleotide binding; P:regulation of biological process; P:cell proliferation | IPR000719 (PROFILE); IPR001245 (PRINTS); IPR002290 (SMART); IPR008266 (PROSITE); IPR011009 (SUPERFAMILY); IPR017441 (PROSITE); IPR020635 (SMART); G3DSA:1.10.510.10 (GENE3D), G3DSA:3.30.200.20 (GENE3D), PTHR24418 (PANTHER), PTHR24418:SF79 (PANTHER) |
| 1547 | lib_dib_Filter15_IDBA_contig_26753_plus2 | 70 | fibrillin- partial | 377 | 7.28E-122 | 59.10% | 4 | F:protein binding; F:calcium ion binding; F:structural molecule activity; C:proteinaceous extracellular matrix | IPR000152 (PROSITE); IPR000742 (SMART); IPR001881 (PFAM); IPR011398 (PTHR24039:PANTHER); IPR013032 (PROSITE); IPR017878 (G3DSA:3.90.290.GENE3D); IPR018097 (PROSITE); G3DSA:2.10.25.10 (GENE3D), PTHR24039 (PANTHER), SSF57196 (SUPERFAMILY) |
| 1548 | lib_dib_Filter15_IDBA_contig_26788_minus2 | 79 | apoptosis stimulating of | 376 | 9.80E-124 | 85.85% | 2 | F:hydrolase activity; F:protein binding | IPR001452 (PFAM); IPR002110 (SMART); IPR020683 (G3DSA:1.25.40.GENE3D); G3DSA:2.30.30.40 (GENE3D), PTHR24164 (PANTHER), PTHR24164:SF0 (PANTHER) |
| 1549 | lib_dib_Filter15_IDBA_contig_26819_plus2 | 1256 | lysosomal alpha-glucosidase-like | 1262 | 0 | 64.50% | 14 | P:regulation of biological process; F:hydrolase activity; C:lysosome; P:multicellular organismal development; P:generation of precursor metabolites and energy; P:carbohydrate metabolic process; P:catabolic process; P:biological_process; C:cell; P:behavior; P:anatomical structure morphogenesis; P:cellular homeostasis; F:carbohydrate binding; P:organelle organization | IPR000322 (PANTHER); IPR000519 (G3DSA:4.10.110.GENE3D); IPR011013 (SUPERFAMILY); IPR013785 (G3DSA:3.20.20.GENE3D); IPR017853 (SUPERFAMILY); IPR017957 (PROSITE); IPR025887 (PFAM); PTHR22762:SF11 (PANTHER), tmhmm (TMHMM), SSF51011 (SUPERFAMILY) |

| | | | | | | | | | | |
|---|---|---|---|---|---|---|---|---|---|---|
| 1550 | lib_dib_Filter15_IDBA_contig_26820_plus1 | 30 | lysosomal alpha-glucosidase | 101 | 1.75E-43 | 78.15% | 7 | P:biological_process; P:multicellular organismal development; F:hydrolase activity; P:anatomical structure morphogenesis; P:generation of precursor metabolites and energy; P:carbohydrate metabolic process; F:carbohydrate binding | IPR000322 (PANTHER); IPR011013 (SUPERFAMILY); IPR017853 (SUPERFAMILY); PTHR22762:SF11 (PANTHER) | |
| 1551 | lib_dib_Filter15_IDBA_contig_26833_plus2 | 92 | ankyrin repeat family a protein 2 | 376 | 5.35E-46 | 58.00% | 3 | C:cell; C:cytosol; F:protein binding | IPR002110 (PFAM); IPR020683 (G3DSA:1.25.40.GENE3D); PTHR24124 (PANTHER), PTHR24124:SF0 (PANTHER) | |
| 1552 | lib_dib_Filter15_IDBA_contig_26859_plus1 | 2885 | peptidyl-prolyl isomerase fkbp12 | 922 | 1.12E-47 | 72.40% | 12 | C:cytosol; P:biosynthetic process; ; P:cellular protein modification process; P:protein metabolic process; F:catalytic activity; F:binding; F:protein binding; P:nucleobase-containing compound metabolic process; C:cell; C:plastid; C:thylakoid | IPR001179 (PFAM); IPR023566 (PANTHER); G3DSA:3.10.50.40 (GENE3D), PTHR10516:SF145 (PANTHER), SSF54534 (SUPERFAMILY) | |
| 1553 | lib_dib_Filter15_IDBA_contig_26877_minus1 | 123 | carbonic anhydrase 4-like | 426 | 3.21E-48 | 54.20% | 8 | F:metal ion binding; F:zinc ion binding; P:one-carbon metabolic process; F:carbonate dehydratase activity; F:lyase activity; C:extracellular region; C:membrane fraction; C:anchored to plasma membrane | IPR001148 (G3DSA:3.10.200.GENE3D); IPR023561 (PANTHER); SignalP-NN(euk) (SIGNALP), tmhmm (TMHMM) | |
| 1554 | lib_dib_Filter15_IDBA_contig_26882_plus2 | 51 | probable beta-d-xylosidase 2-like | 375 | 2.15E-153 | 70.10% | 2 | F:hydrolase activity; P:carbohydrate metabolic process | IPR001764 (PRINTS); IPR002772 (G3DSA:3.40.50.GENE3D); IPR017853 (SUPERFAMILY); IPR026892 (PANTHER); PTHR30620:SF0 (PANTHER) | |
| 1555 | lib_dib_Filter15_IDBA_contig_26927_minus2 | 2666 | zinc metalloproteinase toh-2 | 1227 | 5.64E-40 | 43.55% | 6 | P:multicellular organismal development; F:protein binding; F:peptidase activity; P:protein metabolic process; P:catabolic process; F:binding | IPR000562 (G3DSA:2.10.10.GENE3D); IPR000742 (SMART); IPR000859 (SMART); IPR001506 (PRINTS); IPR006026 (SMART); IPR013032 (PROSITE); IPR013806 (SUPERFAMILY); IPR024079 (G3DSA:3.40.390.GENE3D); PR00013 (PRINTS), G3DSA:2.10.25.10 (GENE3D), PTHR10127 (PANTHER), PTHR10127:SF334 (PANTHER), SSF55486 (SUPERFAMILY), SSF57196 (SUPERFAMILY) | |
| 1556 | lib_dib_Filter15_IDBA_contig_26933_minus1 | 1183 | cre-acr-25 protein | 375 | 3.97E-25 | 50.30% | 3 | C:cell; P:ion transport; F:ion channel activity | IPR006201 (PANTHER); IPR006202 (G3DSA:2.70.170.GENE3D); SignalP-NN(euk) (SIGNALP) | |
| 1557 | lib_dib_Filter15_IDBA_contig_26946_plus3 | 425 | subfamily member 11 | 977 | 1.49E-162 | 78.95% | 9 | P:metabolic process; P:signal transduction; P:response to stress; P:response to biotic stimulus; P:nucleobase-containing compound metabolic process; F:protein binding; P:protein metabolic process; C:endoplasmic reticulum; C:nucleus | IPR001623 (PRINTS); IPR002939 (PFAM); IPR008971 (SUPERFAMILY); IPR018253 (PROSITE); G3DSA:2.60.260.20 (GENE3D), PTHR24077 (PANTHER), PTHR24077:SF14 (PANTHER), SignalP-NN(euk) (SIGNALP), tmhmm (TMHMM) | Yes |
| 1558 | lib_dib_Filter15_IDBA_contig_26948_plus2 | 78 | glucosidase 2 subunit beta | 508 | 3.78E-81 | 63.15% | 1 | F:protein binding | IPR002172 (G3DSA:4.10.400.GENE3D); IPR009011 (SUPERFAMILY); G3DSA:2.70.130.10 (GENE3D), PTHR12630 (PANTHER), PF12999 (PFAM), PF13015 (PFAM), SignalP-NN(euk) (SIGNALP), tmhmm (TMHMM) | |
| 1559 | lib_dib_Filter15_IDBA_contig_26952_minus2 | 248 | tyrosine-protein phosphatase 10d | 890 | 4.16E-63 | 42.70% | 5 | P:biological_process; C:extracellular region; C:cell; F:catalytic activity; F:protein binding | IPR003961 (PFAM); IPR013783 (G3DSA:2.60.40.GENE3D); PTHR19143 (PANTHER) | |
| 1560 | lib_dib_Filter15_IDBA_contig_26953_minus2 | 252 | tyrosine-protein phosphatase 10d | 886 | 3.45E-66 | 42.50% | 7 | C:cell; C:extracellular region; P:regulation of biological process; P:cellular component organization; P:biological_process; F:catalytic activity; F:protein binding | IPR003961 (PFAM); IPR013783 (G3DSA:2.60.40.GENE3D); PTHR19143 (PANTHER) | |

| | | | | | | | | | | |
|---|---|---|---|---|---|---|---|---|---|---|
| 1561 | lib_dib_Filter15_IDBA_contig_26954_minus2 | 99 | tenascin r | 337 | 7.23E-28 | 43.20% | 9 | P:regulation of biological process; P:cell-cell signaling; P:cellular component organization; P:anatomical structure morphogenesis; P:cell differentiation; F:protein binding; C:intracellular; P:biological_process; C:extracellular region | IPR003961 (PFAM); IPR013783 (G3DSA:2.60.40.GENE3D); PTHR19143 (PANTHER) | |
| 1562 | lib_dib_Filter15_IDBA_contig_26971_minus2 | 307 | grip and coiled-coil domain-containing protein 2 | 789 | 1.09E-121 | 58.45% | 6 | P:transport; P:organelle organization; C:organelle; C:intracellular; P:protein transport; F:protein binding | IPR000237 (G3DSA:1.10.220.GENE3D); IPR009053 (SUPERFAMILY); PTHR18902 (PANTHER), PTHR18902:SF9 (PANTHER) | |
| 1563 | lib_dib_Filter15_IDBA_contig_26993_plus3 | 262 | deleted in malignant brain tumors 1 | 698 | 7.20E-49 | 54.35% | 3 | F:receptor activity; C:cell; F:protein binding | IPR001190 (PRINTS); IPR017448 (SMART); IPR020067 (G3DSA:1.10.2000.GENE3D); G3DSA:3.10.250.10 (GENE3D), PTHR19331 (PANTHER), PTHR19331:SF118 (PANTHER) | |
| 1564 | lib_dib_Filter15_IDBA_contig_27009_plus2 | 143 | protein o-mannosyl-transferase 2-like | 432 | 6.63E-161 | 69.95% | 2 | F:transferase activity; C:cell | IPR016093 (PFAM); IPR027005 (PANTHER); G3DSA:2.80.10.50 (GENE3D), PTHR10050:SF11 (PANTHER), tmhmm (TMHMM) | |
| 1565 | lib_dib_Filter15_IDBA_contig_27011_minus2 | 72 | tyrosine-protein kinase jak2 | 373 | 4.73E-33 | 49.20% | 12 | P:metabolic process; P:regulation of biological process; P:multicellular organismal development; C:cell; P:cellular protein modification process; P:signal transduction; P:primary metabolic process; F:protein binding; P:cell differentiation; P:biological_process; F:nucleotide binding; F:protein kinase activity | IPR000719 (PROFILE); IPR001245 (PFAM); IPR011009 (SUPERFAMILY); IPR016251 (PRINTS); IPR020635 (SMART); G3DSA:1.10.510.10 (GENE3D), G3DSA:3.30.200.20 (GENE3D), PTHR24418 (PANTHER), PTHR24418:SF69 (PANTHER) | |
| 1566 | lib_dib_Filter15_IDBA_contig_27020_plus2 | 74 | ras suppressor protein 1-like | 373 | 1.67E-144 | 81.30% | 2 | P:signal transduction; F:protein binding | IPR001611 (PFAM); IPR003591 (SMART); G3DSA:3.80.10.10 (GENE3D), PTHR23155 (PANTHER), PTHR23155:SF21 (PANTHER), PF13504 (PFAM), PF13855 (PFAM), SM00364 (SMART), SSF52058 (SUPERFAMILY) | |
| 1567 | lib_dib_Filter15_IDBA_contig_27027_plus3 | 617 | collagen alpha-5 chain | 861 | 2.60E-61 | 49.75% | 1 | F:protein binding | IPR002035 (PFAM); PR00453 (PRINTS), G3DSA:3.40.50.410 (GENE3D), PTHR22992 (PANTHER), tmhmm (TMHMM), SSF53300 (SUPERFAMILY) | |
| 1568 | lib_dib_Filter15_IDBA_contig_27041_minus1 | 46 | protocadherin fat 4 | 373 | 3.33E-56 | 52.65% | 1 | F:protein binding | IPR000742 (PFAM); IPR001791 (PFAM); IPR008985 (SUPERFAMILY); IPR013032 (PFAM); IPR013320 (G3DSA:2.60.120.GENE3D); G3DSA:2.10.25.10 (GENE3D), PTHR24026 (PANTHER), SSF57196 (SUPERFAMILY) | |
| 1569 | lib_dib_Filter15_IDBA_contig_27042_plus1 | 180 | kelch-like protein 20-like | 495 | 1.74E-106 | 61.25% | 13 | P:protein metabolic process; P:catabolic process; C:nucleolus; C:intracellular; C:protein complex; F:protein binding; C:cell; F:catalytic activity; P:cellular protein modification process; C:nucleoplasm; P:regulation of biological process; P:cell death; C:Golgi apparatus | IPR000210 (SMART); IPR006652 (PFAM); IPR011333 (G3DSA:3.30.710.GENE3D); IPR011705 (PFAM); IPR013069 (PFAM); IPR015916 (G3DSA:2.130.10.GENE3D); PTHR24412 (PANTHER), SignalP-NN(euk) (SIGNALP), SSF117281 (SUPERFAMILY) | |
| 1570 | lib_dib_Filter15_IDBA_contig_27082_plus2 | 56 | pro-neuregulin- membrane-bound isoform-like | 372 | 4.45E-10 | 44.95% | 8 | F:receptor binding; P:multicellular organismal development; P:regulation of biological process; C:plasma membrane; C:cell; P:signal transduction; P:cell differentiation; P:metabolic process | IPR000742 (PROFILE); IPR003598 (SMART); IPR003599 (SMART); IPR007110 (PROFILE); IPR013032 (PROSITE); IPR013098 (PFAM); IPR013783 (G3DSA:2.60.40.GENE3D); G3DSA:2.10.25.10 (GENE3D), PTHR10489 (PANTHER), SignalP-NN(euk) (SIGNALP), tmhmm (TMHMM), SSF48726 (SUPERFAMILY), SSF57196 (SUPERFAMILY) | Yes |

| | | | | | | | | | | |
|---|---|---|---|---|---|---|---|---|---|---|
| 1571 | lib_dib_Filter15_IDBA_contig_27083_minus1 | 509 | nacht and ankyrin domain protein | 578 | 5.03E-17 | 43.75% | 9 | C:cell; F:protein binding; P:biological_process; P:regulation of biological process; P:transport; P:cellular component organization; C:organelle; C:intracellular; C:plasma membrane | IPR001496 (PFAM); IPR002110 (SMART); IPR020683 (G3DSA:1.25.40.GENE3D); PTHR24188 (PANTHER), SignalP-NN(euk) (SIGNALP), tmhmm (TMHMM) | Yes |
| 1572 | lib_dib_Filter15_IDBA_contig_27126_minus1 | 107 | n-acetylglucosamine-1-phosphotransferase subunit gamma precursor | 372 | 1.02E-92 | 63.25% | 1 | F:transferase activity | IPR009011 (SUPERFAMILY); G3DSA:2.70.130.10 (GENE3D), PTHR12630 (PANTHER), PTHR12630:SF2 (PANTHER), PF13015 (PFAM), SignalP-NN(euk) (SIGNALP), tmhmm (TMHMM) | Yes |
| 1573 | lib_dib_Filter15_IDBA_contig_27133_minus2 | 69 | beta-galactosidase-1-like protein 2 | 372 | 1.58E-76 | 69.15% | 2 | F:hydrolase activity; P:carbohydrate metabolic process | IPR001944 (PRINTS); IPR013781 (G3DSA:3.20.20.GENE3D); IPR017853 (SUPERFAMILY); tmhmm (TMHMM) | |
| 1574 | lib_dib_Filter15_IDBA_contig_27157_plus2 | 1320 | low quality protein: cubilin-like | 531 | 9.98E-37 | 45.90% | 4 | F:calcium ion binding; F:transferase activity; F:hydrolase activity; F:transmembrane receptor protein tyrosine kinase activity | IPR000859 (G3DSA:2.60.120.GENE3D); PTHR10127 (PANTHER) | |
| 1575 | lib_dib_Filter15_IDBA_contig_27158_plus1 | 1200 | low quality protein: cubilin-like | 493 | 3.45E-37 | 45.95% | 4 | F:calcium ion binding; F:transferase activity; F:hydrolase activity; F:transmembrane receptor protein tyrosine kinase activity | IPR000859 (G3DSA:2.60.120.GENE3D); PTHR10127 (PANTHER) | |
| 1576 | lib_dib_Filter15_IDBA_contig_27160_minus3 | 79 | chloride channel calcium activated 2-like | 398 | 5.12E-69 | 55.25% | 1 | P:biological_process | IPR013642 (PFAM); PTHR10579 (PANTHER), PTHR10579:SF8 (PANTHER), tmhmm (TMHMM) | |
| 1577 | lib_dib_Filter15_IDBA_contig_27161_minus3 | 351 | chloride channel calcium activated 2-like | 1211 | 5.32E-136 | 50.70% | 2 | P:biological_process; F:protein binding | IPR002035 (SMART); IPR013642 (PFAM); IPR015394 (PFAM); G3DSA:3.40.50.410 (GENE3D), PTHR10579 (PANTHER), PTHR10579:SF7 (PANTHER), PF13519 (PFAM), tmhmm (TMHMM), SSF53300 (SUPERFAMILY) | |
| 1578 | lib_dib_Filter15_IDBA_contig_27174_minus2 | 230 | btb poz domain-containing protein 17 | 646 | 3.40E-60 | 49.20% | 1 | F:protein binding | IPR000210 (SMART); IPR011333 (G3DSA:3.30.710.GENE3D); IPR011705 (PFAM); IPR013069 (PFAM); PTHR24413 (PANTHER) | |
| 1579 | lib_dib_Filter15_IDBA_contig_27181_minus2 | 149 | low quality protein: basement membrane-specific heparan sulfate proteoglycan core | 680 | 3.98E-16 | 45.65% | 2 | C:cell; F:protein binding | IPR000998 (PFAM); IPR002172 (G3DSA:4.10.400.GENE3D); IPR008985 (SUPERFAMILY); IPR023415 (PROSITE); PR00261 (PRINTS), PTHR23282 (PANTHER), PTHR23282:SF9 (PANTHER) | |
| 1580 | lib_dib_Filter15_IDBA_contig_27208_minus1 | 36 | kelch-like protein 26 | 371 | 3.17E-84 | 53.45% | 1 | F:protein binding | IPR000210 (SMART); IPR011333 (G3DSA:3.30.710.GENE3D); IPR011705 (PFAM); IPR013069 (PFAM); PTHR24412 (PANTHER) | |
| 1581 | lib_dib_Filter15_IDBA_contig_27211_minus2 | 1218 | c3 and pzp-like alpha-2-macroglobulin domain-containing protein 8 | 846 | 1.21E-133 | 57.30% | 2 | P:cell differentiation; P:cell-cell signaling | IPR022041 (PFAM) | |
| 1582 | lib_dib_Filter15_IDBA_contig_27212_minus2 | 2733 | isoform d | 1191 | 0 | 60.40% | 2 | P:cell differentiation; P:cell-cell signaling | IPR018017 (PANTHER); IPR022041 (PFAM); G3DSA:3.40.50.1580 (GENE3D), PTHR21234:SF9 (PANTHER), tmhmm (TMHMM), SSF53167 (SUPERFAMILY) | |
| 1583 | lib_dib_Filter15_IDBA_contig_27241_plus3 | 177 | hypothetical protein CAPTEDRAFT_206360 | 625 | 6.47E-45 | 53.75% | 0 | - | PF13383 (PFAM), tmhmm (TMHMM) | |
| 1584 | lib_dib_Filter15_IDBA_contig_27244_minus3 | 616 | tyrosine-protein phosphatase 10d | 1147 | 4.55E-45 | 61.45% | 11 | C:plasma membrane; P:biological_process; F:phosphoprotein phosphatase activity; F:protein binding; C:cell; P:cellular component organization; C:cytoplasm; P:signal transduction; P:regulation of biological process; F:receptor activity; P:anatomical structure morphogenesis | IPR000242 (PRINTS); IPR000387 (PROFILE); IPR000562 (G3DSA:2.10.10.GENE3D); IPR003595 (SMART); IPR003961 (PROFILE); IPR008979 (SUPERFAMILY); IPR013806 (SUPERFAMILY); IPR016130 (PROSITE); G3DSA:2.170.300.10 (GENE3D), G3DSA:2.60.120.260 (GENE3D), G3DSA:3.90.190.10 (GENE3D), PTHR19134 (PANTHER), tmhmm (TMHMM), SSF52799 (SUPERFAMILY), SSF57196 (SUPERFAMILY) | |

| | | | | | | | | | |
|---|---|---|---|---|---|---|---|---|---|
| 1585 | lib_dib_Filter15_IDBA_contig_27247_minus3 | 68 | multiple epidermal growth factor-like domains 6 | 302 | 5.52E-11 | 50.35% | 1 | F:protein binding | IPR003961 (PROFILE); IPR013783 (G3DSA:2.60.40.GENE3D); G3DSA:2.170.300.10 (GENE3D), PTHR24035 (PANTHER), PTHR24035:SF4 (PANTHER), tmhmm (TMHMM) |
| 1586 | lib_dib_Filter15_IDBA_contig_27255_plus1 | 272 | low quality protein: neogenin-like | 900 | 1.03E-100 | 55.50% | 4 | P:anatomical structure morphogenesis; P:embryo development; P:multicellular organismal development; F:protein binding | IPR003961 (PFAM); IPR013783 (G3DSA:2.60.40.GENE3D); PR00014 (PRINTS), PTHR10489 (PANTHER), PTHR10489:SF55 (PANTHER), tmhmm (TMHMM) |
| 1587 | lib_dib_Filter15_IDBA_contig_27328_plus1 | 4637 | cytosolic non-specific dipeptidase isoform 2 | 668 | 2.26E-161 | 84.20% | 8 | F:binding; P:metabolic process; F:peptidase activity; P:protein metabolic process; P:catabolic process; C:cytosol; P:biosynthetic process; | IPR002933 (PFAM); IPR011650 (PFAM); G3DSA:3.30.70.360 (GENE3D), G3DSA:3.40.630.10 (GENE3D), PTHR11014 (PANTHER), PTHR11014:SF15 (PANTHER), tmhmm (TMHMM), SSF53187 (SUPERFAMILY) |
| 1588 | lib_dib_Filter15_IDBA_contig_27348_minus1 | 263 | collagen alpha-1 chain | 965 | 1.12E-70 | 61.20% | 9 | P:anatomical structure morphogenesis; P:multicellular organismal development; F:binding; P:response to external stimulus; P:response to abiotic stimulus; C:proteinaceous extracellular matrix; P:biological_process; P:cellular component organization; F:structural molecule activity | IPR000885 (PRODOM); IPR008160 (PFAM); PTHR24023 (PANTHER) |
| 1589 | lib_dib_Filter15_IDBA_contig_27348_plus2 | 263 | ---NA--- | 965 | | | 0 | - | PTHR24023 (PANTHER), PTHR24023:SF196 (PANTHER) |
| 1590 | lib_dib_Filter15_IDBA_contig_27372_plus2 | 1389 | mucosa-associated lymphoid tissue lymphoma translocation protein 1 | 1164 | 7.67E-63 | 42.85% | 8 | P:biological_process; P:metabolic process; P:signal transduction; P:regulation of biological process; F:peptidase activity; P:protein metabolic process; P:catabolic process; F:protein binding | IPR001309 (PROFILE); IPR003598 (SMART); IPR003599 (SMART); IPR007110 (PROFILE); IPR011029 (G3DSA:1.10.533.GENE3D); IPR011600 (PFAM); IPR013098 (PFAM); IPR013783 (G3DSA:2.60.40.GENE3D); G3DSA:3.40.50.1460 (GENE3D), PTHR22576 (PANTHER), PF13895 (PFAM), SSF48726 (SUPERFAMILY), SSF52129 (SUPERFAMILY) |
| 1591 | lib_dib_Filter15_IDBA_contig_27432_minus2 | 71 | zinc finger protein 271-like | 442 | 4.59E-68 | 66.20% | 3 | F:binding; F:nucleic acid binding; C:intracellular | IPR007087 (PROSITE); IPR013087 (G3DSA:3.30.160.GENE3D); IPR015880 (SMART); PTHR24387 (PANTHER), PTHR24387:SF0 (PANTHER), PF13465 (PFAM), SSF57667 (SUPERFAMILY) |
| 1592 | lib_dib_Filter15_IDBA_contig_27459_minus1 | 226 | serine threonine-protein kinase pelle-like | 608 | 5.15E-47 | 52.35% | 3 | F:nucleotide binding; P:cellular protein modification process; F:protein kinase activity | IPR000719 (PROFILE); IPR001245 (PFAM); IPR002290 (SMART); IPR008266 (PROSITE); IPR011009 (SUPERFAMILY); IPR011029 (G3DSA:1.10.533.GENE3D); IPR017441 (PROSITE); IPR020635 (SMART); G3DSA:1.10.510.10 (GENE3D), G3DSA:3.30.200.20 (GENE3D), PTHR24419 (PANTHER) |
| 1593 | lib_dib_Filter15_IDBA_contig_27460_minus1 | 224 | serine threonine-protein kinase pelle-like | 605 | 5.50E-47 | 52.30% | 3 | F:nucleotide binding; P:cellular protein modification process; F:protein kinase activity | IPR000719 (PROFILE); IPR001245 (PFAM); IPR002290 (SMART); IPR008266 (PROSITE); IPR011009 (SUPERFAMILY); IPR011029 (G3DSA:1.10.533.GENE3D); IPR017441 (PROSITE); IPR020635 (SMART); G3DSA:1.10.510.10 (GENE3D), G3DSA:3.30.200.20 (GENE3D), PTHR24419 (PANTHER) |
| 1594 | lib_dib_Filter15_IDBA_contig_27461_minus1 | 359 | gigaxonin | 708 | 0 | 58.25% | 3 | F:protein binding; P:biological_process; C:intracellular | IPR000210 (SMART); IPR006652 (PFAM); IPR011333 (G3DSA:3.30.710.GENE3D); IPR011705 (PFAM); IPR013069 (PFAM); IPR015915 (G3DSA:2.120.10.GENE3D); PTHR24412 (PANTHER), PTHR24412:SF29 (PANTHER), PF13964 (PFAM), SSF117281 (SUPERFAMILY) |

| | | | | | | | | | |
|---|---|---|---|---|---|---|---|---|---|
| 1595 | lib_dib_Filter15_IDBA_contig_27462_minus1 | 357 | gigaxonin | 709 | 0 | 58.25% | 3 | F:protein binding; P:biological_process; C:intracellular | IPR000210 (SMART); IPR006652 (PFAM); IPR011333 (G3DSA:3.30.710.GENE3D); IPR011705 (PFAM); IPR013069 (PFAM); IPR015915 (G3DSA:2.120.10.GENE3D); PTHR24412 (PANTHER), PTHR24412:SF29 (PANTHER), PF13964 (PFAM), SSF117281 (SUPERFAMILY) |
| 1596 | lib_dib_Filter15_IDBA_contig_27516_plus1 | 70 | zinc finger protein 271-like | 550 | 1.32E-114 | 54.75% | 3 | F:binding; F:nucleic acid binding; C:intracellular | IPR007087 (PROSITE); IPR013087 (G3DSA:3.30.160.GENE3D); IPR015880 (SMART); PTHR24377 (PANTHER), PTHR24377:SF0 (PANTHER), PF13465 (PFAM), PF13894 (PFAM), SSF57667 (SUPERFAMILY) |
| 1597 | lib_dib_Filter15_IDBA_contig_27517_minus1 | 320 | zinc finger protein 271-like | 800 | 6.12E-90 | 59.05% | 3 | F:binding; F:nucleic acid binding; C:intracellular | IPR007087 (PFAM); IPR013087 (G3DSA:3.30.160.GENE3D); IPR015880 (SMART); PTHR24384 (PANTHER), PTHR24384:SF0 (PANTHER), PF13465 (PFAM), SSF57667 (SUPERFAMILY) |
| 1598 | lib_dib_Filter15_IDBA_contig_27518_minus1 | 149 | oocyte zinc finger protein 6-like | 334 | 2.46E-68 | 56.60% | 3 | F:binding; F:nucleic acid binding; C:intracellular | IPR007087 (PFAM); IPR013087 (G3DSA:3.30.160.GENE3D); IPR015880 (SMART); PTHR24375 (PANTHER), PTHR24375:SF0 (PANTHER), PF13465 (PFAM), SSF57667 (SUPERFAMILY) |
| 1599 | lib_dib_Filter15_IDBA_contig_27523_plus1 | 34 | fibrillin 1-like | 367 | 4.75E-62 | 53.15% | 3 | F:catalytic activity; F:protein binding; F:calcium ion binding | IPR000152 (PROSITE); IPR000742 (SMART); IPR001846 (PFAM); IPR001881 (PFAM); IPR013032 (PROSITE); IPR018097 (PROSITE); IPR023413 (G3DSA:2.40.155.GENE3D); G3DSA:2.10.25.10 (GENE3D), PTHR24838 (PANTHER), PTHR24838:SF171 (PANTHER), SSF57196 (SUPERFAMILY) |
| 1600 | lib_dib_Filter15_IDBA_contig_27526_minus3 | 72 | receptor-type tyrosine-protein phosphatase kappa | 378 | 8.90E-17 | 51.75% | 1 | F:protein binding | IPR003961 (PROFILE); IPR013783 (G3DSA:2.60.40.GENE3D); G3DSA:2.170.300.10 (GENE3D), G3DSA:2.60.120.260 (GENE3D), PTHR24035 (PANTHER), PTHR24035:SF4 (PANTHER) |
| 1601 | lib_dib_Filter15_IDBA_contig_27535_minus3 | 232 | deformed epidermal autoregulatory factor 1 homolog | 672 | 3.60E-98 | 59.75% | 13 | F:protein binding; P:regulation of biological process; ; P:cell proliferation; P:multicellular organismal development; P:embryo development; C:cytoplasm; C:nucleoplasm; C:protein complex; P:anatomical structure morphogenesis; P:reproduction; P:cell differentiation; F:DNA binding | IPR000770 (PFAM); IPR010919 (G3DSA:3.10.390.GENE3D); IPR024119 (PANTHER); PTHR10237:SF0 (PANTHER), tmhmm (TMHMM) |
| 1602 | lib_dib_Filter15_IDBA_contig_27556_plus3 | 586 | plasminogen precursor | 668 | 8.34E-57 | 55.15% | 19 | P:anatomical structure morphogenesis; P:protein metabolic process; P:catabolic process; P:cellular component organization; F:peptidase activity; P:embryo development; C:cell; P:multicellular organismal development; C:extracellular space; P:cell death; P:biological_process; P:response to external stimulus; P:regulation of biological process; P:response to stress; F:protein binding; F:binding; P:cell differentiation; C:plasma membrane; F:receptor activity | IPR000001 (G3DSA:2.40.20.GENE3D); IPR001190 (PRINTS); IPR013806 (SUPERFAMILY); IPR017448 (SMART); IPR018056 (PROSITE); PR00018 (PRINTS), G3DSA:3.10.250.10 (GENE3D), PTHR24259 (PANTHER), PTHR24259:SF2 (PANTHER), tmhmm (TMHMM) |
| 1603 | lib_dib_Filter15_IDBA_contig_27585_plus1 | 11777 | granulins precursor | 868 | 0 | 55.15% | 4 | P:anatomical structure morphogenesis; P:cellular component organization; P:cell differentiation; P:multicellular organismal development | IPR000118 (PFAM); PD968187 (PRODOM), PTHR12274 (PANTHER), PTHR12274:SF0 (PANTHER), SSF57277 (SUPERFAMILY) |

| | | | | | | | | | | |
|---|---|---|---|---|---|---|---|---|---|---|
| 1604 | lib_dib_Filter15_IDBA_contig_27586_plus1 | 13947 | grn protein | 1102 | 0 | 55.50% | 4 | P:anatomical structure morphogenesis; P:cellular component organization; P:cell differentiation; P:multicellular organismal development | IPR000118 (PFAM); PD968187 (PRODOM), PTHR12274 (PANTHER), PTHR12274:SF0 (PANTHER), SSF57277 (SUPERFAMILY) | |
| 1605 | lib_dib_Filter15_IDBA_contig_27627_plus1 | 52 | protein let- isoform a | 365 | 1.52E-38 | 43.95% | 2 | P:multicellular organismal development; F:protein binding | IPR003961 (PFAM); IPR013783 (G3DSA:2.60.40.GENE3D); PR00014 (PRINTS), PTHR10489 (PANTHER) | |
| 1606 | lib_dib_Filter15_IDBA_contig_27662_minus3 | 1536 | serine protease -like | 762 | 0 | 71.00% | 3 | P:protein metabolic process; P:catabolic process; F:peptidase activity | IPR008758 (PANTHER); G3DSA:3.40.50.1820 (GENE3D), PTHR11010:SF4 (PANTHER), SignalP-NN(euk) (SIGNALP), SSF53474 (SUPERFAMILY) | Yes |
| 1607 | lib_dib_Filter15_IDBA_contig_27663_minus3 | 1447 | serine protease -like | 609 | 0 | 71.00% | 3 | P:protein metabolic process; P:catabolic process; F:peptidase activity | IPR008758 (PANTHER); G3DSA:3.40.50.1820 (GENE3D), PTHR11010:SF4 (PANTHER), SignalP-NN(euk) (SIGNALP), SSF53474 (SUPERFAMILY) | Yes |
| 1608 | lib_dib_Filter15_IDBA_contig_27664_minus3 | 1737 | serine protease -like | 819 | 0 | 71.00% | 3 | P:protein metabolic process; P:catabolic process; F:peptidase activity | IPR008758 (PANTHER); G3DSA:3.40.50.1820 (GENE3D), PTHR11010:SF4 (PANTHER), SignalP-NN(euk) (SIGNALP), SSF53474 (SUPERFAMILY) | Yes |
| 1609 | lib_dib_Filter15_IDBA_contig_27668_minus3 | 308 | sortilin-related receptor-like | 844 | 2.15E-50 | 41.25% | 1 | F:protein binding | IPR002172 (G3DSA:4.10.400.GENE3D); IPR003961 (PFAM); IPR013783 (G3DSA:2.60.40.GENE3D); IPR023415 (PROSITE); PR00261 (PRINTS), G3DSA:4.10.1220.10 (GENE3D), PTHR10529 (PANTHER), PTHR10529:SF113 (PANTHER), tmhmm (TMHMM) | |
| 1610 | lib_dib_Filter15_IDBA_contig_27669_minus3 | 367 | sortilin-related receptor-like | 951 | 5.30E-62 | 42.10% | 1 | F:protein binding | IPR002172 (G3DSA:4.10.400.GENE3D); IPR003961 (PFAM); IPR013783 (G3DSA:2.60.40.GENE3D); IPR023415 (PROSITE); PR00261 (PRINTS), G3DSA:4.10.1220.10 (GENE3D), PTHR10529 (PANTHER), tmhmm (TMHMM) | |
| 1611 | lib_dib_Filter15_IDBA_contig_27670_minus1 | 14 | ---NA--- | 108 | | | 1 | F:protein binding | IPR002172 (G3DSA:4.10.400.GENE3D); IPR023415 (PROSITE); PR00261 (PRINTS), PTHR10529 (PANTHER) | |
| 1612 | lib_dib_Filter15_IDBA_contig_27681_minus2 | 126 | protein kinase c and casein kinase substrate in neurons protein 2-like isoform 2 | 462 | 1.04E-22 | 74.10% | 3 | P:metabolic process; F:kinase activity; F:protein binding | IPR001452 (PRINTS); G3DSA:2.30.30.40 (GENE3D), PTHR10959 (PANTHER) | |
| 1613 | lib_dib_Filter15_IDBA_contig_27695_minus1 | 59 | ptk7-like protein | 364 | 7.29E-157 | 72.40% | 19 | P:signal transduction; P:anatomical structure morphogenesis; P:growth; C:plasma membrane; F:protein binding; P:multicellular organismal development; P:cellular component organization; P:cell differentiation; P:response to external stimulus; P:response to stress; P:cellular protein modification process; P:embryo development; P:cell communication; F:receptor activity; F:protein kinase activity; P:regulation of biological process; P:cytoskeleton organization; F:nucleotide binding; P:biological_process | IPR000719 (PROFILE); IPR001245 (PRINTS); IPR002290 (SMART); IPR008266 (PROSITE); IPR011009 (SUPERFAMILY); IPR020635 (SMART); G3DSA:1.10.510.10 (GENE3D), G3DSA:3.30.200.20 (GENE3D), PTHR24418 (PANTHER), PTHR24418:SF95 (PANTHER) | |
| 1614 | lib_dib_Filter15_IDBA_contig_277_minus3 | 695 | von willebrand factor type egf and pentraxin domain-containing protein 1 | 271 | 3.51E-21 | 54.65% | 3 | P:biological_process; C:cell; F:carbohydrate binding | IPR000436 (PFAM); G3DSA:2.10.70.10 (GENE3D), PTHR19325 (PANTHER) | |
| 1615 | lib_dib_Filter15_IDBA_contig_27731_plus3 | 34 | metal regulatory transcription factor 1 | 363 | 6.80E-120 | 83.20% | 9 | P:response to stress; F:binding; F:protein binding; P:regulation of biological process; ; F:sequence-specific DNA binding transcription factor activity; P:biological_process; C:cytoplasm; C:nucleus | IPR007087 (PFAM); IPR013087 (G3DSA:3.30.160.GENE3D); IPR015880 (SMART); PTHR11389 (PANTHER), PTHR11389:SF347 (PANTHER), PF13465 (PFAM), SSF57667 (SUPERFAMILY) | |

| | | | | | | | | |
|---|---|---|---|---|---|---|---|---|
| 1616 | lib_dib_Filter15_IDBA_contig_27733_plus1 | 40 | chordin-like protein 2 isoform 2 | 364 | 7.21E-23 | 43.80% | 2 | P:biological_process; F:protein binding | IPR001007 (PFAM); PTHR11339 (PANTHER), PTHR11339:SF39 (PANTHER), SSF57603 (SUPERFAMILY) |
| 1617 | lib_dib_Filter15_IDBA_contig_27773_minus3 | 536 | fibrinogen-like protein 1 | 406 | 4.42E-49 | 56.30% | 4 | F:receptor binding; P:signal transduction; C:extracellular space; P:response to stilbenoid | IPR002181 (PFAM); IPR014715 (G3DSA:4.10.530.GENE3D); IPR014716 (G3DSA:3.90.215.GENE3D); PTHR19143 (PANTHER), tmhmm (TMHMM) |
| 1618 | lib_dib_Filter15_IDBA_contig_27777_minus3 | 6142 | cub and sushi domain-containing protein 3-like | 498 | 6.21E-44 | 48.75% | 3 | F:carbohydrate binding; F:calcium ion binding; F:chromatin binding | IPR000436 (PFAM); IPR000884 (SMART); G3DSA:2.10.70.10 (GENE3D), PTHR19325 (PANTHER), tmhmm (TMHMM) |
| 1619 | lib_dib_Filter15_IDBA_contig_27778_minus3 | 11375 | von willebrand factor type egf and pentraxin domain-containing protein 1-like | 682 | 1.32E-95 | 45.20% | 5 | F:chitin binding; P:chitin metabolic process; C:extracellular region; F:carbohydrate binding; F:calcium ion binding | IPR000436 (PFAM); IPR000884 (SMART); G3DSA:2.10.70.10 (GENE3D), PTHR19325 (PANTHER), tmhmm (TMHMM) |
| 1620 | lib_dib_Filter15_IDBA_contig_27779_minus3 | 20351 | von willebrand factor type egf and pentraxin domain-containing protein 1-like | 1177 | 0 | 46.40% | 2 | F:protein binding; F:calcium ion binding | IPR000152 (PROSITE); IPR000436 (PFAM); IPR000742 (PROFILE); IPR000884 (SMART); IPR001881 (PFAM); IPR018097 (PROSITE); G3DSA:2.10.25.10 (GENE3D), G3DSA:2.10.70.10 (GENE3D), PTHR19325 (PANTHER), SSF57196 (SUPERFAMILY) |
| 1621 | lib_dib_Filter15_IDBA_contig_27780_minus3 | 26549 | von willebrand factor type egf and pentraxin domain-containing protein 1-like | 1337 | 0 | 46.05% | 2 | F:protein binding; F:calcium ion binding | IPR000152 (PROSITE); IPR000436 (PFAM); IPR000742 (SMART); IPR000884 (SMART); IPR001881 (PFAM); IPR009030 (SUPERFAMILY); IPR011641 (PFAM); IPR013032 (PROSITE); IPR018097 (PROSITE); G3DSA:2.10.25.10 (GENE3D), G3DSA:2.10.70.10 (GENE3D), PTHR19325 (PANTHER), SSF57196 (SUPERFAMILY) |
| 1622 | lib_dib_Filter15_IDBA_contig_27787_minus2 | 454 | lactase-phlorizin hydrolase-like | 773 | 1.76E-19 | 72.40% | 2 | F:hydrolase activity; P:carbohydrate metabolic process | IPR001360 (PRINTS); IPR013781 (G3DSA:3.20.20.GENE3D); IPR017853 (SUPERFAMILY); tmhmm (TMHMM) |
| 1623 | lib_dib_Filter15_IDBA_contig_27788_minus2 | 205 | lactase-phlorizin hydrolase-like | 216 | 4.90E-22 | 72.50% | 2 | F:hydrolase activity; P:carbohydrate metabolic process | IPR001360 (PRINTS); IPR013781 (G3DSA:3.20.20.GENE3D); IPR017853 (SUPERFAMILY) |
| 1624 | lib_dib_Filter15_IDBA_contig_27790_minus3 | 15 | lactase-phlorizin hydrolase-like | 105 | 7.76E-19 | 81.70% | 3 | P:carbohydrate metabolic process; F:hydrolase activity; F:binding | IPR001360 (PANTHER); IPR013781 (G3DSA:3.20.20.GENE3D); IPR017853 (SUPERFAMILY) |
| 1625 | lib_dib_Filter15_IDBA_contig_27794_plus2 | 174 | chondroitin sulfate synthase 1 | 615 | 1.51E-105 | 63.30% | 7 | P:regulation of biological process; P:multicellular organismal development; F:transferase activity; C:extracellular region; P:biosynthetic process; P:carbohydrate metabolic process; C:Golgi apparatus | IPR008428 (PANTHER); G3DSA:3.90.550.10 (GENE3D), PTHR12369:SF5 (PANTHER), SSF53448 (SUPERFAMILY) |
| 1626 | lib_dib_Filter15_IDBA_contig_27855_minus3 | 1056 | macrophage-stimulating protein receptor-like | 1205 | 6.99E-107 | 67.35% | 17 | P:anatomical structure morphogenesis; P:cellular component organization; P:cell differentiation; P:multicellular organismal development; P:signal transduction; P:reproduction; P:response to biotic stimulus; F:receptor activity; F:protein kinase activity; P:biological_process; C:plasma membrane; P:regulation of biological process; P:cell proliferation; P:metabolic process; F:protein binding; P:response to stress; F:nucleotide binding | IPR000719 (PROFILE); IPR001245 (PRINTS); IPR002290 (SMART); IPR003598 (SMART); IPR003599 (SMART); IPR007110 (PROFILE); IPR008266 (PROSITE); IPR011009 (SUPERFAMILY); IPR013098 (PFAM); IPR013783 (G3DSA:2.60.40.GENE3D); IPR017441 (PROSITE); IPR020635 (SMART); G3DSA:1.10.510.10 (GENE3D), G3DSA:3.30.200.20 (GENE3D), PTHR24416 (PANTHER), PF13895 (PFAM), tmhmm (TMHMM), SSF48726 (SUPERFAMILY) |

| | | | | | | | | | | |
|---|---|---|---|---|---|---|---|---|---|---|
| 1627 | lib_dib_Filter15_IDBA_contig_27856_minus3 | 1062 | macrophage-stimulating protein receptor-like | 1206 | 7.37E-107 | 67.40% | 17 | P:anatomical structure morphogenesis; P:cellular component organization; P:cell differentiation; P:multicellular organismal development; P:signal transduction; P:reproduction; P:response to biotic stimulus; F:receptor activity; F:protein kinase activity; P:biological_process; C:plasma membrane; P:regulation of biological process; P:cell proliferation; P:metabolic process; F:protein binding; P:response to stress; F:nucleotide binding | IPR000719 (PROFILE); IPR001245 (PRINTS); IPR002290 (SMART); IPR003598 (SMART); IPR003599 (SMART); IPR007110 (PROFILE); IPR008266 (PROSITE); IPR011009 (SUPERFAMILY); IPR013098 (PFAM); IPR013783 (G3DSA:2.60.40.GENE3D); IPR017441 (PROSITE); IPR020635 (SMART); G3DSA:1.10.510.10 (GENE3D), G3DSA:3.30.200.20 (GENE3D), PTHR24416 (PANTHER), PF13895 (PFAM), tmhmm (TMHMM), SSF48726 (SUPERFAMILY) | |
| 1628 | lib_dib_Filter15_IDBA_contig_27977_minus1 | 204 | isoform cra_a | 536 | 0 | 74.85% | 23 | C:cell; C:Golgi apparatus; P:regulation of biological process; P:protein metabolic process; P:catabolic process; F:peptidase activity; P:biological_process; P:multicellular organismal development; P:cell proliferation; C:extracellular space; F:enzyme regulator activity; C:endoplasmic reticulum; P:cell death; P:reproduction; P:viral reproduction; C:cytoplasmic membrane-bounded vesicle; F:protein binding; C:endosome; F:binding; P:signal transduction; P:biosynthetic process; C:plasma membrane; P:transport | IPR000209 (G3DSA:3.40.50.GENE3D); IPR009020 (SUPERFAMILY); IPR015500 (PRINTS); IPR022398 (PROSITE); IPR023827 (PROSITE); IPR023828 (PROSITE); G3DSA:3.30.70.850 (GENE3D), PTHR10795:SF16 (PANTHER) | |
| 1629 | lib_dib_Filter15_IDBA_contig_27978_minus1 | 261 | furin | 617 | 0 | 72.05% | 23 | C:cell; C:Golgi apparatus; P:regulation of biological process; P:protein metabolic process; P:catabolic process; F:peptidase activity; P:biological_process; P:multicellular organismal development; P:cell proliferation; C:extracellular space; F:enzyme regulator activity; C:endoplasmic reticulum; P:cell death; P:reproduction; P:viral reproduction; C:cytoplasmic membrane-bounded vesicle; F:protein binding; C:endosome; F:binding; P:signal transduction; P:biosynthetic process; C:plasma membrane; P:transport | IPR000209 (G3DSA:3.40.50.GENE3D); IPR002884 (PFAM); IPR008979 (SUPERFAMILY); IPR009020 (SUPERFAMILY); IPR015500 (PRINTS); IPR022398 (PROSITE); IPR023827 (PROSITE); IPR023828 (PROSITE); G3DSA:2.60.120.260 (GENE3D), G3DSA:3.30.70.850 (GENE3D), PTHR10795:SF153 (PANTHER) | |
| 1630 | lib_dib_Filter15_IDBA_contig_27983_minus2 | 66 | zinc finger protein 271-like | 433 | 1.65E-100 | 62.85% | 3 | F:binding; F:nucleic acid binding; C:intracellular | IPR007087 (PFAM); IPR013087 (G3DSA:3.30.160.GENE3D); IPR015880 (SMART); PTHR24384 (PANTHER), PF13465 (PFAM), SSF57667 (SUPERFAMILY) | |
| 1631 | lib_dib_Filter15_IDBA_contig_27999_minus1 | 375 | chymotrypsinogen a-like | 463 | 4.40E-69 | 60.30% | 3 | F:peptidase activity; P:protein metabolic process; P:catabolic process | IPR000519 (G3DSA:4.10.110.GENE3D); IPR001254 (PFAM); IPR001314 (PRINTS); IPR009003 (SUPERFAMILY); IPR017957 (PROSITE); IPR018114 (PROSITE); G3DSA:2.40.10.10 (GENE3D), PTHR24256 (PANTHER), SignalP-NN(euk) (SIGNALP) | Yes |
| 1632 | lib_dib_Filter15_IDBA_contig_28014_plus2 | 463 | serine protease 30-like | 614 | 1.39E-52 | 55.40% | 10 | F:protein binding; P:regulation of biological process; P:cellular component organization; P:response to external stimulus; P:response to stress; C:extracellular region; C:plasma membrane; F:peptidase activity; P:protein metabolic process; P:catabolic process | IPR001254 (PFAM); IPR001314 (PRINTS); IPR009003 (SUPERFAMILY); IPR018114 (PROSITE); G3DSA:2.40.10.10 (GENE3D), PTHR24265 (PANTHER) | |
| 1633 | lib_dib_Filter15_IDBA_contig_28049_minus3 | 72 | beta- -galactosyl-o-glycosyl-glycoprotein beta- -n-acetylglucosaminyltransferase | 360 | 1.07E-123 | 60.10% | 2 | F:transferase activity; C:cell | IPR003406 (PFAM); PTHR19297 (PANTHER), PTHR19297:SF6 (PANTHER) | |

| | | | | | | | | | |
|---|---|---|---|---|---|---|---|---|---|
| 1634 | lib_dib_Filter15_IDBA_contig_28065_minus2 | 53 | toll-like receptor 3 | 360 | 2.61E-13 | 49.90% | 7 | P:regulation of biological process; P:primary metabolic process; C:cell; P:biosynthetic process; P:signal transduction; P:protein metabolic process; F:protein binding | IPR000157 (PFAM); PR01537 (PRINTS), G3DSA:3.40.50.10140 (GENE3D), PTHR24365 (PANTHER), tmhmm (TMHMM) |
| 1635 | lib_dib_Filter15_IDBA_contig_28069_plus1 | 59 | wd repeat-containing protein 48 | 360 | 0 | 85.05% | 5 | P:cellular protein modification process; C:lysosome; F:protein binding; C:nucleus; P:biological_process | IPR001680 (PFAM); IPR015943 (G3DSA:2.130.10.GENE3D); IPR017986 (PROFILE); IPR019775 (PROSITE); IPR020472 (PRINTS); PTHR19862 (PANTHER), PTHR19862:SF6 (PANTHER) |
| 1636 | lib_dib_Filter15_IDBA_contig_28082_minus2 | 51 | transducin beta-like protein 3 | 360 | 2.57E-120 | 66.95% | 4 | C:nucleolus; F:signal transducer activity; P:signal transduction; F:protein binding | IPR001680 (PFAM); IPR015943 (G3DSA:2.130.10.GENE3D); IPR017986 (PROFILE); IPR020472 (PRINTS); PTHR19854 (PANTHER), PTHR19854:SF14 (PANTHER) |
| 1637 | lib_dib_Filter15_IDBA_contig_28097_minus1 | 158 | wd repeat-containing protein 5 | 582 | 0 | 92.75% | 9 | C:nucleoplasm; C:protein complex; F:transferase activity; F:protein binding; ; P:regulation of biological process; P:organelle organization; P:cellular protein modification process; P:multicellular organismal development | IPR001680 (PFAM); IPR015943 (G3DSA:2.130.10.GENE3D); IPR017986 (PROFILE); IPR019775 (PROSITE); IPR020472 (PRINTS); PTHR22847 (PANTHER), PTHR22847:SF48 (PANTHER) |
| 1638 | lib_dib_Filter15_IDBA_contig_28111_plus3 | 70 | #NAME? | 359 | 4.65E-13 | 54.65% | 1 | F:protein binding | IPR002110 (SMART); IPR020683 (G3DSA:1.25.40.GENE3D); PTHR24193 (PANTHER) |
| 1639 | lib_dib_Filter15_IDBA_contig_28114_plus1 | 56 | slk partial | 360 | 2.40E-136 | 79.60% | 9 | P:cellular protein modification process; P:cell cycle; P:cell death; F:nucleotide binding; F:protein binding; C:cytoplasm; C:plasma membrane; F:protein kinase activity; F:signal transducer activity | IPR000719 (PFAM); IPR002290 (SMART); IPR008271 (PROSITE); IPR011009 (SUPERFAMILY); IPR017441 (PROSITE); IPR020635 (SMART); G3DSA:1.10.510.10 (GENE3D), G3DSA:3.30.200.20 (GENE3D), PTHR24361 (PANTHER), PTHR24361:SF195 (PANTHER), SignalP-NN(euk) (SIGNALP) |
| 1640 | lib_dib_Filter15_IDBA_contig_28115_minus3 | 108 | dipeptidyl peptidase 9 | 606 | 9.83E-27 | 87.85% | 4 | F:peptidase activity; C:cell; P:protein metabolic process; P:catabolic process | IPR001375 (PFAM); G3DSA:3.40.50.1820 (GENE3D), PTHR11731 (PANTHER), PTHR11731:SF10 (PANTHER), tmhmm (TMHMM), SSF53474 (SUPERFAMILY) |
| 1641 | lib_dib_Filter15_IDBA_contig_2817_plus1 | 5587 | ---NA--- | 227 | | | 0 | - | IPR008160 (PFAM); PTHR24023 (PANTHER) |
| 1642 | lib_dib_Filter15_IDBA_contig_28222_minus3 | 61 | nischarin isoform 2 | 358 | 3.74E-146 | 65.55% | 6 | C:cytosol; P:regulation of biological process; P:cytoskeleton organization; F:receptor binding; P:signal transduction; C:plasma membrane | IPR001611 (PROFILE); IPR025875 (PFAM); G3DSA:3.80.10.10 (GENE3D), PTHR15454 (PANTHER), PTHR15454:SF8 (PANTHER), PF13855 (PFAM), SSF52058 (SUPERFAMILY) |
| 1643 | lib_dib_Filter15_IDBA_contig_28239_minus1 | 423 | leucine-rich repeats and immunoglobulin-like domains protein 3 | 1035 | 0 | 67.95% | 1 | F:protein binding | IPR000372 (PFAM); IPR000483 (SMART); IPR001611 (PROFILE); IPR003591 (SMART); IPR003598 (SMART); IPR003599 (SMART); IPR007110 (PROFILE); IPR013098 (PFAM); IPR013783 (G3DSA:2.60.40.GENE3D); PR00019 (PRINTS), G3DSA:3.80.10.10 (GENE3D), PTHR24367 (PANTHER), PTHR24367:SF106 (PANTHER), PF13516 (PFAM), PF13855 (PFAM), PF13895 (PFAM), SM00365 (SMART), tmhmm (TMHMM), SSF48726 (SUPERFAMILY), SSF52047 (SUPERFAMILY), SSF52058 (SUPERFAMILY) |

| | | | | | | | | | | |
|---|---|---|---|---|---|---|---|---|---|---|
| 1644 | lib_dib_Filter15_IDBA_contig_28240_minus1 | 623 | leucine-rich repeats and immunoglobulin-like domains protein 3 | 1295 | 0 | 67.95% | 1 | F:protein binding | IPR000372 (PFAM); IPR000483 (SMART); IPR001611 (PROFILE); IPR003591 (SMART); IPR003598 (SMART); IPR003599 (SMART); IPR007110 (PROFILE); IPR013098 (PFAM); IPR013783 (G3DSA:2.60.40.GENE3D); PR00019 (PRINTS), G3DSA:3.80.10.10 (GENE3D), PTHR24367 (PANTHER), PTHR24367:SF106 (PANTHER), PF13516 (PFAM), PF13855 (PFAM), PF13895 (PFAM), SM00365 (SMART), tmhmm (TMHMM), SSF48726 (SUPERFAMILY), SSF52047 (SUPERFAMILY), SSF52058 (SUPERFAMILY) | |
| 1645 | lib_dib_Filter15_IDBA_contig_28251_plus3 | 499 | prolyl 3-hydroxylase 2-like | 955 | 0 | 53.35% | 9 | C:cytoplasm; F:catalytic activity; P:cell growth; P:cellular component organization; P:multicellular organismal development; P:regulation of biological process; C:nucleus; P:metabolic process; F:binding | IPR005123 (PFAM); IPR006620 (SMART); PTHR14049 (PANTHER), SignalP-NN(euk) (SIGNALP), tmhmm (TMHMM) | Yes |
| 1646 | lib_dib_Filter15_IDBA_contig_28252_plus1 | 45 | prolyl 3-hydroxylase 2 | 358 | 1.01E-81 | 66.40% | 7 | C:organelle; C:intracellular; C:proteinaceous extracellular matrix; F:catalytic activity; P:metabolic process; C:cytoplasm; F:binding | IPR005123 (PFAM); IPR006620 (SMART); PTHR14049 (PANTHER), tmhmm (TMHMM) | |
| 1647 | lib_dib_Filter15_IDBA_contig_28266_plus1 | 168 | trans- -dihydrobenzene- -diol dehydrogenase-like | 673 | 1.67E-106 | 64.90% | 2 | F:catalytic activity; F:nucleotide binding | IPR000683 (PFAM); IPR016040 (G3DSA:3.40.50.GENE3D); G3DSA:3.30.360.10 (GENE3D), PTHR22604 (PANTHER), PTHR22604:SF15 (PANTHER), SSF51735 (SUPERFAMILY), SSF55347 (SUPERFAMILY) | |
| 1648 | lib_dib_Filter15_IDBA_contig_28275_minus3 | 1086 | delta-like protein 1 | 926 | 0 | 65.95% | 13 | P:multicellular organismal development; P:cell differentiation; P:regulation of biological process; P:cell-cell signaling; P:embryo development; P:signal transduction; C:organelle; C:cytoplasm; P:anatomical structure morphogenesis; C:plasma membrane; F:receptor binding; F:calcium ion binding; C:cell | IPR000152 (PROSITE); IPR000742 (PFAM); IPR001774 (PFAM); IPR001881 (SMART); IPR011651 (PFAM); IPR013032 (PROSITE); IPR018097 (PROSITE); PD936484 (PRODOM), PR00010 (PRINTS), G3DSA:2.10.25.10 (GENE3D), G3DSA:2.170.300.10 (GENE3D), PTHR24044 (PANTHER), PTHR24044:SF49 (PANTHER), SignalP-NN(euk) (SIGNALP), tmhmm (TMHMM), SSF57196 (SUPERFAMILY) | |
| 1649 | lib_dib_Filter15_IDBA_contig_28278_minus1 | 59 | lim domain kinase 1-like isoform 2 | 358 | 2.05E-126 | 74.35% | 5 | P:cellular protein modification process; F:binding; F:protein kinase activity; P:regulation of biological process; F:nucleotide binding | IPR000719 (PROFILE); IPR001245 (PFAM); IPR002290 (SMART); IPR011009 (SUPERFAMILY); IPR020635 (SMART); G3DSA:1.10.510.10 (GENE3D), G3DSA:3.30.200.20 (GENE3D), PTHR23257 (PANTHER), PTHR23257:SF94 (PANTHER), SignalP-NN(euk) (SIGNALP) | |
| 1650 | lib_dib_Filter15_IDBA_contig_28299_minus3 | 989 | lachesin | 499 | 5.73E-142 | 51.60% | 1 | F:protein binding | IPR003596 (SMART); IPR003598 (SMART); IPR003599 (SMART); IPR007110 (PROFILE); IPR013098 (PFAM); IPR013106 (PFAM); IPR013783 (G3DSA:2.60.40.GENE3D); PTHR19831 (PANTHER), PTHR19831:SF16 (PANTHER), PF13895 (PFAM), tmhmm (TMHMM), SSF48726 (SUPERFAMILY) | |
| 1651 | lib_dib_Filter15_IDBA_contig_28338_minus1 | 200 | fibrinogen c domain-containing protein 1-like | 523 | 9.47E-54 | 57.65% | 3 | F:receptor binding; C:extracellular space; P:signal transduction | IPR002181 (PFAM); IPR014715 (G3DSA:4.10.530.GENE3D); IPR014716 (G3DSA:3.90.215.GENE3D); IPR020837 (PROSITE); PTHR19143 (PANTHER) | |
| 1652 | lib_dib_Filter15_IDBA_contig_28340_minus1 | 195 | fibrinogen c domain-containing protein 1-like | 535 | 1.27E-53 | 57.65% | 3 | F:receptor binding; C:extracellular space; P:signal transduction | IPR002181 (PFAM); IPR014715 (G3DSA:4.10.530.GENE3D); IPR014716 (G3DSA:3.90.215.GENE3D); IPR020837 (PROSITE); PTHR19143 (PANTHER) | |

| | | | | | | | | | | |
|---|---|---|---|---|---|---|---|---|---|---|
| 1653 | lib_dib_Filter15_IDBA_contig_28368_plus2 | 134 | peptidyl-prolyl cis-trans isomerase e | 523 | 4.68E-163 | 79.90% | 7 | P:protein metabolic process; P:nucleobase-containing compound metabolic process; C:nucleus; F:RNA binding; P:cellular protein modification process; F:catalytic activity; F:nucleotide binding | IPR000504 (PFAM); IPR002130 (PRINTS); IPR012677 (G3DSA:3.30.70.GENE3D); IPR020892 (PROSITE); G3DSA:2.40.100.10 (GENE3D), PTHR11071 (PANTHER), PTHR11071:SF72 (PANTHER), SSF54928 (SUPERFAMILY) | |
| 1654 | lib_dib_Filter15_IDBA_contig_28371_plus2 | 360 | cub and sushi domain-containing protein 2 | 356 | 7.52E-50 | 54.35% | 5 | F:carbohydrate binding; F:molecular_function; P:biological_process; C:cellular_component; F:calcium ion binding | IPR000436 (PFAM); IPR000884 (PROFILE); G3DSA:2.10.70.10 (GENE3D), PTHR19325 (PANTHER) | |
| 1655 | lib_dib_Filter15_IDBA_contig_28411_plus2 | 44 | loc733382 protein | 356 | 3.11E-53 | 54.90% | 1 | F:carbohydrate binding | IPR000922 (PFAM); PTHR25100 (PANTHER), PTHR25100:SF16 (PANTHER) | |
| 1656 | lib_dib_Filter15_IDBA_contig_28421_plus2 | 1002 | tartrate-resistant acid phosphatase type 5 precursor | 528 | 4.14E-132 | 69.80% | 7 | P:regulation of biological process; C:cell; P:biological_process; C:cytosol; P:metabolic process; F:binding; F:hydrolase activity | IPR004843 (PFAM); G3DSA:3.60.21.10 (GENE3D), PTHR10161 (PANTHER), PTHR10161:SF8 (PANTHER), SignalP-NN(euk) (SIGNALP), SSF56300 (SUPERFAMILY) | Yes |
| 1657 | lib_dib_Filter15_IDBA_contig_28458_minus1 | 89 | ankyrin repeat-containing protein | 355 | 1.39E-18 | 48.35% | 1 | F:protein binding | IPR002110 (SMART); IPR020683 (G3DSA:1.25.40.GENE3D); PTHR24193 (PANTHER), PF13857 (PFAM) | |
| 1658 | lib_dib_Filter15_IDBA_contig_28465_plus2 | 990 | alpha macroglobulin | 149 | 1.20E-10 | 64.20% | 1 | F:enzyme regulator activity | IPR011625 (PFAM); PTHR11412 (PANTHER) | |
| 1659 | lib_dib_Filter15_IDBA_contig_28466_plus2 | 4286 | alpha macroglobulin | 666 | 9.91E-104 | 51.50% | 1 | F:enzyme regulator activity | IPR002890 (PFAM); IPR011625 (PFAM); PTHR11412 (PANTHER), PTHR11412:SF36 (PANTHER), SignalP-NN(euk) (SIGNALP) | |
| 1660 | lib_dib_Filter15_IDBA_contig_28467_plus1 | 397 | alpha-1-macroglobulin- partial | 116 | 2.15E-29 | 61.60% | 5 | P:negative regulation of endopeptidase activity; C:extracellular space; C:extracellular region; F:endopeptidase inhibitor activity; F:hydrolase activity | PTHR11412 (PANTHER) | |
| 1661 | lib_dib_Filter15_IDBA_contig_28483_minus2 | 83 | zinc finger protein 668 | 396 | 1.30E-40 | 53.30% | 3 | F:binding; F:nucleic acid binding; C:intracellular | IPR007087 (PFAM); IPR013087 (G3DSA:3.30.160.GENE3D); IPR015880 (SMART); PTHR11389 (PANTHER), PF13465 (PFAM), PF13894 (PFAM), SSF57667 (SUPERFAMILY) | |
| 1662 | lib_dib_Filter15_IDBA_contig_28484_minus2 | 78 | zinc finger protein 668 | 377 | 9.25E-41 | 53.30% | 3 | F:binding; F:nucleic acid binding; C:intracellular | IPR007087 (PFAM); IPR013087 (G3DSA:3.30.160.GENE3D); IPR015880 (SMART); PTHR11389 (PANTHER), PF13465 (PFAM), PF13894 (PFAM), SSF57667 (SUPERFAMILY) | |
| 1663 | lib_dib_Filter15_IDBA_contig_28526_minus1 | 40 | von willebrand factor d and egf domain-containing protein | 354 | 6.80E-67 | 47.95% | 1 | F:calcium ion binding | IPR001846 (PFAM); PTHR11339 (PANTHER) | |
| 1664 | lib_dib_Filter15_IDBA_contig_28603_plus3 | 31 | a disintegrin and metalloproteinase with thrombospondin motifs 20 | 250 | 4.14E-76 | 72.90% | 11 | P:signal transduction; F:binding; C:extracellular space; F:peptidase activity; P:protein metabolic process; P:catabolic process; P:cell differentiation; P:regulation of biological process; C:extracellular region; P:cell death; P:cellular component organization | IPR001590 (PFAM); IPR024079 (G3DSA:3.40.390.GENE3D); PTHR13723 (PANTHER), SSF55486 (SUPERFAMILY) | |
| 1665 | lib_dib_Filter15_IDBA_contig_28605_plus2 | 41 | cadherin-related tumor suppressor | 353 | 8.17E-70 | 55.95% | 4 | F:transferase activity; F:calcium ion binding; P:biological_process; C:plasma membrane | IPR002126 (PRINTS); IPR015919 (SUPERFAMILY); IPR020894 (PROSITE); PTHR24027 (PANTHER) | |
| 1666 | lib_dib_Filter15_IDBA_contig_28669_plus2 | 106 | zag-1 | 537 | 9.66E-50 | 87.05% | 10 | P:anatomical structure morphogenesis; P:cellular component organization; P:cell differentiation; P:multicellular organismal development; F:binding; P:regulation of biological process; ; F:DNA binding; F:sequence-specific DNA binding transcription factor activity; C:nucleus | IPR007087 (PROSITE); IPR013087 (G3DSA:3.30.160.GENE3D); IPR015880 (SMART); PTHR24391 (PANTHER), PTHR24391:SF0 (PANTHER), PF13465 (PFAM), SignalP-NN(euk) (SIGNALP), tmhmm (TMHMM), SSF57667 (SUPERFAMILY) | |

| | | | | | | | | | |
|---|---|---|---|---|---|---|---|---|---|
| 1667 | lib_dib_Filter15_IDBA_contig_28670_plus2 | 153 | zag-1 | 756 | 4.45E-49 | 86.60% | 10 | P:anatomical structure morphogenesis; P:cellular component organization; P:cell differentiation; P:multicellular organismal development; F:binding; P:regulation of biological process; ; F:DNA binding; F:sequence-specific DNA binding transcription factor activity; C:nucleus | IPR007087 (PROSITE); IPR013087 (G3DSA:3.30.160.GENE3D); IPR015880 (SMART); PTHR24391 (PANTHER), PTHR24391:SF0 (PANTHER), PF13465 (PFAM), tmhmm (TMHMM), SSF57667 (SUPERFAMILY) |
| 1668 | lib_dib_Filter15_IDBA_contig_28697_plus2 | 150 | neuferricin precursor | 559 | 4.03E-85 | 62.65% | 1 | F:binding | IPR001199 (G3DSA:3.10.120.GENE3D); PTHR10281 (PANTHER), PTHR10281:SF4 (PANTHER), SignalP-NN(euk) (SIGNALP), tmhmm (TMHMM) |
| 1669 | lib_dib_Filter15_IDBA_contig_28698_plus1 | 164 | neuferricin precursor | 579 | 1.10E-84 | 62.75% | 1 | F:binding | IPR001199 (G3DSA:3.10.120.GENE3D); PTHR10281 (PANTHER), PTHR10281:SF4 (PANTHER), SignalP-NN(euk) (SIGNALP), tmhmm (TMHMM) |
| 1670 | lib_dib_Filter15_IDBA_contig_28731_minus2 | 53 | acetylcholinesterase | 400 | 4.20E-163 | 64.30% | 3 | F:hydrolase activity; P:cell-cell signaling; P:metabolic process | IPR002018 (PFAM); IPR019826 (PROSITE); G3DSA:3.40.50.1820 (GENE3D), PTHR11559 (PANTHER), PTHR11559:SF33 (PANTHER), SSF53474 (SUPERFAMILY) |
| 1671 | lib_dib_Filter15_IDBA_contig_28732_minus3 | 47 | acetylcholinesterase | 296 | 5.83E-134 | 76.70% | 4 | P:cell-cell signaling; F:hydrolase activity; ; C:cell | IPR000997 (PRINTS); IPR002018 (PFAM); IPR019819 (PROSITE); IPR019826 (PROSITE); G3DSA:3.40.50.1820 (GENE3D), PTHR11559 (PANTHER), PTHR11559:SF33 (PANTHER), SignalP-NN(euk) (SIGNALP), tmhmm (TMHMM), SSF53474 (SUPERFAMILY) |
| 1672 | lib_dib_Filter15_IDBA_contig_28738_minus1 | 180 | bactericidal permeability increasing protein | 529 | 5.80E-65 | 48.30% | 1 | F:lipid binding | IPR001124 (PFAM); IPR010562 (PFAM); IPR017942 (PFAM); IPR017943 (SUPERFAMILY); G3DSA:3.15.10.10 (GENE3D), G3DSA:3.15.20.10 (GENE3D), PTHR10504 (PANTHER), PTHR10504:SF18 (PANTHER), SignalP-NN(euk) (SIGNALP) |
| 1673 | lib_dib_Filter15_IDBA_contig_28739_minus1 | 188 | bactericidal permeability increasing protein | 610 | 5.05E-64 | 48.25% | 1 | F:lipid binding | IPR001124 (PFAM); IPR010562 (PFAM); IPR017942 (PFAM); IPR017943 (SUPERFAMILY); G3DSA:3.15.10.10 (GENE3D), G3DSA:3.15.20.10 (GENE3D), PTHR10504 (PANTHER), PTHR10504:SF18 (PANTHER), SignalP-NN(euk) (SIGNALP) |
| 1674 | lib_dib_Filter15_IDBA_contig_28750_minus1 | 473 | glutathione peroxidase 7 precursor | 680 | 3.00E-52 | 66.65% | 4 | F:catalytic activity; F:antioxidant activity; P:response to stress; P:metabolic process | IPR000889 (PRINTS); IPR012336 (G3DSA:3.40.30.GENE3D); PTHR11592:SF0 (PANTHER) |
| 1675 | lib_dib_Filter15_IDBA_contig_28754_plus1 | 1034 | carbonic anhydrase | 451 | 2.30E-103 | 63.10% | 3 | F:catalytic activity; P:metabolic process; F:binding | IPR001148 (G3DSA:3.10.200.GENE3D); IPR018338 (PROSITE); IPR023561 (PANTHER); SignalP-NN(euk) (SIGNALP) |
| 1676 | lib_dib_Filter15_IDBA_contig_28764_minus1 | 37 | collagen alpha-1 chain | 352 | 9.30E-04 | 40.00% | 1 | F:protein binding | IPR003961 (PFAM); IPR013783 (G3DSA:2.60.40.GENE3D); PTHR19143 (PANTHER) |
| 1677 | lib_dib_Filter15_IDBA_contig_28785_plus2 | 66 | multiple epidermal growth factor-like domains protein 9-like | 351 | 5.29E-23 | 49.55% | 5 | P:anatomical structure morphogenesis; P:multicellular organismal development; P:cell differentiation; C:proteinaceous extracellular matrix; P:cellular component organization | IPR002049 (PFAM); G3DSA:2.10.25.10 (GENE3D), PTHR10574 (PANTHER), PTHR10574:SF36 (PANTHER), tmhmm (TMHMM), SSF57196 (SUPERFAMILY) |
| 1678 | lib_dib_Filter15_IDBA_contig_28816_plus1 | 600 | mam domain-containing glycosylphosphatidylinositol anchor protein 1 | 504 | 1.51E-24 | 51.20% | 2 | C:cell; F:protein binding | IPR000998 (PFAM); IPR002035 (PFAM); IPR008985 (SUPERFAMILY); G3DSA:3.40.50.410 (GENE3D), PTHR23282 (PANTHER), SSF53300 (SUPERFAMILY) |

| | | | | | | | | | |
|---|---|---|---|---|---|---|---|---|---|
| 1679 | lib_dib_Filter15_IDBA_contig_28817_plus1 | 880 | collagen alpha-3 chain | 844 | 9.46E-54 | 49.65% | 2 | C:cell; F:protein binding | IPR000998 (PFAM); IPR002035 (PFAM); IPR008985 (SUPERFAMILY); PR00453 (PRINTS), G3DSA:3.40.50.410 (GENE3D), PTHR22992 (PANTHER), SignalP-NN(euk) (SIGNALP), tmhmm (TMHMM), SSF53300 (SUPERFAMILY) |
| 1680 | lib_dib_Filter15_IDBA_contig_28818_plus2 | 532 | bactericidal permeability increasing protein | 758 | 8.66E-54 | 43.45% | 1 | F:lipid binding | IPR001124 (PFAM); IPR017942 (PFAM); IPR017943 (SUPERFAMILY); G3DSA:3.15.10.10 (GENE3D), G3DSA:3.15.20.10 (GENE3D), PTHR10504 (PANTHER), SignalP-NN(euk) (SIGNALP) |
| 1681 | lib_dib_Filter15_IDBA_contig_28833_minus3 | 44 | integrin-linked protein kinase-like | 350 | 1.09E-81 | 86.05% | 28 | P:regulation of biological process; P:cell proliferation; F:signal transducer activity; C:cell; P:cell death; P:cytoskeleton organization; C:plasma membrane; F:protein kinase activity; F:protein binding; C:cytoplasm; P:cell differentiation; P:multicellular organismal development; P:anatomical structure morphogenesis; P:cellular component organization; C:protein complex; P:signal transduction; P:metabolic process; C:cytosol; F:receptor binding; P:biological_process; F:hydrolase activity; P:cell communication; P:cellular homeostasis; P:cell cycle; F:nucleotide binding; C:cytoskeleton; P:cellular protein modification process; | IPR000719 (PROFILE); IPR001245 (PFAM); IPR011009 (SUPERFAMILY); G3DSA:1.10.510.10 (GENE3D), PTHR23257 (PANTHER), PTHR23257:SF30 (PANTHER), tmhmm (TMHMM) |
| 1682 | lib_dib_Filter15_IDBA_contig_28842_minus2 | 11693 | ---NA--- | 992 | | | 0 | - | no IPS match |
| 1683 | lib_dib_Filter15_IDBA_contig_28842_plus1 | 11693 | cartilage matrix | 992 | 5.10E-53 | 50.85% | 4 | P:multicellular organismal development; P:cell differentiation; F:protein binding; F:calcium ion binding | IPR000152 (PROSITE); IPR000742 (SMART); IPR001881 (SMART); IPR002035 (PFAM); IPR008160 (PFAM); IPR018097 (PROSITE); PR00453 (PRINTS), G3DSA:2.10.25.10 (GENE3D), G3DSA:3.40.50.410 (GENE3D), PTHR24023 (PANTHER), tmhmm (TMHMM), SSF53300 (SUPERFAMILY), SSF57196 (SUPERFAMILY) |
| 1684 | lib_dib_Filter15_IDBA_contig_28842_plus2 | 11693 | ---NA--- | 992 | | | 0 | - | SignalP-NN(euk) (SIGNALP) |
| 1685 | lib_dib_Filter15_IDBA_contig_28857_minus3 | 704 | dehydrogenase reductase sdr family member 11-like | 404 | 1.42E-75 | 63.70% | 3 | P:metabolic process; F:catalytic activity; F:nucleotide binding | IPR002198 (PFAM); IPR002347 (PRINTS); IPR016040 (G3DSA:3.40.50.GENE3D); PTHR24322 (PANTHER), PTHR24322:SF7 (PANTHER), SSF51735 (SUPERFAMILY) |
| 1686 | lib_dib_Filter15_IDBA_contig_28866_minus3 | 431 | gamma-glutamyltransferase 1 | 788 | 7.56E-111 | 53.20% | 4 | P:biosynthetic process; C:cell; F:transferase activity; | IPR000101 (PRINTS); tmhmm (TMHMM), SSF56235 (SUPERFAMILY) |
| 1687 | lib_dib_Filter15_IDBA_contig_28868_plus3 | 1058 | enzymatic glycosylation-regulating-like | 755 | 0 | 56.60% | 2 | F:transferase activity; C:cell | IPR003406 (PFAM); PTHR19297 (PANTHER), PTHR19297:SF6 (PANTHER), tmhmm (TMHMM) |
| 1688 | lib_dib_Filter15_IDBA_contig_28929_plus1 | 46 | oocyte zinc finger protein 6-like | 350 | 2.20E-82 | 65.35% | 3 | F:binding; F:nucleic acid binding; C:intracellular | IPR007087 (PROSITE); IPR013087 (G3DSA:3.30.160.GENE3D); IPR015880 (SMART); PTHR24387 (PANTHER), PF13465 (PFAM), SSF57667 (SUPERFAMILY) |
| 1689 | lib_dib_Filter15_IDBA_contig_28934_minus2 | 118 | indian hedgehog b | 527 | 7.63E-33 | 55.70% | 5 | P:cell communication; P:multicellular organismal development; P:protein metabolic process; P:catabolic process; F:peptidase activity | IPR001657 (PRINTS); IPR001767 (PFAM); IPR003587 (SMART); IPR006141 (PROFILE); G3DSA:2.170.16.10 (GENE3D), PTHR11889 (PANTHER), SignalP-NN(euk) (SIGNALP), tmhmm (TMHMM), SSF51294 (SUPERFAMILY) |

| | | | | | | | | | |
|---|---|---|---|---|---|---|---|---|---|
| 1690 | lib_dib_Filter15_IDBA_contig_28935_minus2 | 44 | indian hedgehog protein | 248 | 1.09E-17 | 54.40% | 11 | P:embryo development; P:cell differentiation; P:regulation of biological process; P:multicellular organismal development; P:cell proliferation; P:cell communication; P:biological_process; P:anatomical structure morphogenesis; P:protein metabolic process; P:catabolic process; F:peptidase activity | IPR001657 (PRINTS); IPR001767 (PFAM); IPR003587 (SMART); IPR006141 (PROFILE); G3DSA:2.170.16.10 (GENE3D), PTHR11889 (PANTHER), SignalP-NN(euk) (SIGNALP), tmhmm (TMHMM), SSF51294 (SUPERFAMILY) |
| 1691 | lib_dib_Filter15_IDBA_contig_28936_minus1 | 15 | sonic hedgehog | 131 | 1.36E-06 | 66.05% | 3 | P:protein metabolic process; P:catabolic process; F:peptidase activity | IPR001767 (PFAM); G3DSA:2.170.16.10 (GENE3D), PTHR11889 (PANTHER), PTHR11889:SF16 (PANTHER), SSF51294 (SUPERFAMILY) Yes |
| 1692 | lib_dib_Filter15_IDBA_contig_28969_plus2 | 4068 | cysteine-rich secretory protein mr30 | 109 | 8.54E-10 | 53.50% | 7 | F:ion channel inhibitor activity; C:extracellular region; F:calcium channel inhibitor activity; P:cellular response to drug; F:molecular_function; C:cell surface; P:pathogenesis | IPR001283 (PANTHER); IPR014044 (G3DSA:3.40.33.GENE3D); SignalP-NN(euk) (SIGNALP) Yes |
| 1693 | lib_dib_Filter15_IDBA_contig_28972_plus3 | 3488 | cysteine-rich secretory protein 2-like | 120 | 2.25E-14 | 54.60% | 3 | F:ion channel inhibitor activity; C:extracellular region; F:calcium channel inhibitor activity | IPR001283 (PANTHER); IPR014044 (G3DSA:3.40.33.GENE3D) |
| 1694 | lib_dib_Filter15_IDBA_contig_29015_plus2 | 2044 | angiotensin-converting enzyme | 625 | 6.82E-163 | 61.10% | 15 | F:peptidase activity; P:biological_process; P:catabolic process; F:binding; P:multicellular organismal development; C:cell; F:receptor binding; P:regulation of biological process; P:transport; C:extracellular space; F:actin binding; C:plasma membrane; P:cell differentiation; C:endosome; P:protein metabolic process | IPR001548 (PRINTS); SignalP-NN(euk) (SIGNALP), tmhmm (TMHMM), SSF55486 (SUPERFAMILY) |
| 1695 | lib_dib_Filter15_IDBA_contig_29020_minus3 | 51 | pappalysin-2 | 348 | 5.30E-22 | 65.30% | 6 | C:cell; P:cell differentiation; P:protein metabolic process; P:catabolic process; F:peptidase activity; F:binding | IPR000800 (PFAM); IPR026918 (PTHR19325:PANTHER); PTHR19325 (PANTHER), tmhmm (TMHMM) |
| 1696 | lib_dib_Filter15_IDBA_contig_29025_minus3 | 729 | von willebrand factor type egf and pentraxin domain-containing protein 1 | 1045 | 1.68E-70 | 46.40% | 4 | F:calcium ion binding; F:chromatin binding; C:integral to membrane; C:plasma membrane | IPR000436 (PFAM); G3DSA:2.10.70.10 (GENE3D), PTHR19325 (PANTHER), tmhmm (TMHMM) |
| 1697 | lib_dib_Filter15_IDBA_contig_29026_minus3 | 580 | von willebrand factor type egf and pentraxin domain-containing protein 1 | 752 | 1.84E-70 | 46.25% | 2 | F:calcium ion binding; F:chromatin binding | IPR000436 (PFAM); G3DSA:2.10.70.10 (GENE3D), PTHR19325 (PANTHER), tmhmm (TMHMM) |
| 1698 | lib_dib_Filter15_IDBA_contig_29041_plus3 | 90 | microfibrillar-associated protein 1 | 348 | 2.08E-79 | 79.45% | 1 | C:proteinaceous extracellular matrix | IPR009730 (PANTHER); PTHR15327:SF0 (PANTHER) |
| 1699 | lib_dib_Filter15_IDBA_contig_29069_minus3 | 237 | zinc metalloproteinase nas-15-like | 487 | 2.13E-31 | 45.95% | 4 | F:peptidase activity; P:protein metabolic process; P:catabolic process; F:binding | IPR001506 (PRINTS); IPR003582 (PFAM); IPR006026 (SMART); IPR024079 (G3DSA:3.40.390.GENE3D); PTHR10127 (PANTHER), PTHR10127:SF334 (PANTHER), SignalP-NN(euk) (SIGNALP), tmhmm (TMHMM), SSF55486 (SUPERFAMILY) Yes |
| 1700 | lib_dib_Filter15_IDBA_contig_29100_minus2 | 131 | a-kinase anchor protein 9 | 606 | 5.73E-21 | 53.50% | 3 | C:pericentriolar material; F:kinase activity; P:phosphorylation | IPR009053 (SUPERFAMILY); PF13514 (PFAM) |
| 1701 | lib_dib_Filter15_IDBA_contig_29122_minus3 | 81 | protein flp | 448 | 5.87E-87 | 50.35% | 0 | - | IPR001466 (PFAM); IPR012338 (G3DSA:3.40.710.GENE3D); IPR021860 (PFAM); PTHR22935 (PANTHER) |
| 1702 | lib_dib_Filter15_IDBA_contig_29123_minus3 | 115 | protein flp | 524 | 1.85E-90 | 49.70% | 0 | - | IPR001466 (PFAM); IPR012338 (G3DSA:3.40.710.GENE3D); IPR021860 (PFAM); PTHR22935 (PANTHER), tmhmm (TMHMM) |
| 1703 | lib_dib_Filter15_IDBA_contig_29124_minus1 | 39 | xylosyltransferase 2 | 348 | 4.06E-62 | 47.75% | 2 | F:transferase activity; C:cell | IPR003406 (PFAM); PTHR19297 (PANTHER) |
| 1704 | lib_dib_Filter15_IDBA_contig_29169_minus1 | 159 | low-density lipoprotein receptor-related protein 1b | 551 | 9.89E-77 | 46.60% | 1 | F:protein binding | IPR002172 (G3DSA:4.10.400.GENE3D); IPR023415 (PROSITE); PR00261 (PRINTS), PTHR10529 (PANTHER) |

| | | | | | | | | | |
|---|---|---|---|---|---|---|---|---|---|
| 1705 | lib_dib_Filter15_IDBA_contig_29187_minus1 | 141 | polypeptide n-acetylgalactosaminyltransferase 1-like | 531 | 1.01E-177 | 62.70% | 4 | F:transferase activity; P:biosynthetic process; P:cellular protein modification process; P:carbohydrate metabolic process | IPR000772 (PFAM); IPR001173 (PFAM); IPR003859 (PFAM); G3DSA:2.80.10.50 (GENE3D), G3DSA:3.90.550.10 (GENE3D), PTHR11675 (PANTHER), SSF53448 (SUPERFAMILY) |
| 1706 | lib_dib_Filter15_IDBA_contig_29188_minus1 | 127 | polypeptide n-acetylgalactosaminyltransferase 13-like | 481 | 2.03E-175 | 63.85% | 2 | P:carbohydrate metabolic process; F:transferase activity | IPR000772 (PFAM); IPR001173 (PFAM); IPR003859 (PFAM); G3DSA:2.80.10.50 (GENE3D), G3DSA:3.90.550.10 (GENE3D), PTHR11675 (PANTHER), SSF53448 (SUPERFAMILY) |
| 1707 | lib_dib_Filter15_IDBA_contig_29229_minus1 | 47 | hypothetical protein CAPTEDRAFT_223351 | 130 | 5.01E-04 | 53.00% | 0 | - | IPR002861 (PFAM); PTHR23130 (PANTHER) |
| 1708 | lib_dib_Filter15_IDBA_contig_29275_plus3 | 53 | zinc finger protein 135-like | 345 | 4.46E-71 | 55.95% | 3 | F:binding; F:nucleic acid binding; C:intracellular | IPR007087 (PROSITE); IPR013087 (G3DSA:3.30.160.GENE3D); IPR015880 (SMART); PTHR24387 (PANTHER), PF13465 (PFAM), SSF57667 (SUPERFAMILY) |
| 1709 | lib_dib_Filter15_IDBA_contig_29299_minus3 | 137 | transcription factor sp4 | 636 | 1.21E-74 | 81.65% | 8 | P:regulation of biological process; ; F:binding; F:DNA binding; F:sequence-specific DNA binding transcription factor activity; F:transcription regulator activity; F:protein binding; C:nucleus | IPR007087 (PROSITE); IPR013087 (G3DSA:3.30.160.GENE3D); IPR015880 (SMART); PTHR23235 (PANTHER), PTHR23235:SF5 (PANTHER), PF13465 (PFAM), SSF57667 (SUPERFAMILY) |
| 1710 | lib_dib_Filter15_IDBA_contig_29309_minus2 | 44 | follistatin | 345 | 1.34E-68 | 59.00% | 3 | P:multicellular organismal development; P:regulation of biological process; F:protein binding | IPR002350 (PFAM); IPR003645 (SMART); G3DSA:3.30.60.30 (GENE3D), PTHR10913 (PANTHER), PTHR10913:SF6 (PANTHER), SignalP-NN(euk) (SIGNALP), SSF100895 (SUPERFAMILY) |
| 1711 | lib_dib_Filter15_IDBA_contig_29342_plus1 | 1778 | protein | 1266 | 1.51E-110 | 57.15% | 5 | C:extracellular region; F:protein binding; F:calcium ion binding; P:carbohydrate metabolic process; F:carbohydrate binding | IPR000152 (PROSITE); IPR000742 (PFAM); IPR001881 (SMART); IPR002557 (SMART); IPR013032 (PFAM); IPR018097 (PROSITE); PD936484 (PRODOM), PR00010 (PRINTS), G3DSA:2.10.25.10 (GENE3D), G3DSA:2.170.300.10 (GENE3D), PTHR24044 (PANTHER), SignalP-NN(euk) (SIGNALP), SSF57196 (SUPERFAMILY) |
| 1712 | lib_dib_Filter15_IDBA_contig_29343_plus3 | 53 | protein | 100 | 1.59E-27 | 63.10% | 8 | P:cell differentiation; P:embryo development; P:multicellular organismal development; C:cytoplasm; F:protein binding; C:proteinaceous extracellular matrix; C:plasma membrane; F:calcium ion binding | IPR000152 (PROSITE); IPR000742 (PFAM); IPR001881 (SMART); IPR013032 (PFAM); IPR018097 (PROSITE); PR00010 (PRINTS), G3DSA:2.10.25.10 (GENE3D), PTHR24044 (PANTHER), SignalP-NN(euk) (SIGNALP), SSF57196 (SUPERFAMILY) |
| 1713 | lib_dib_Filter15_IDBA_contig_29357_plus2 | 36 | zinc finger protein 271-like | 345 | 6.30E-62 | 52.00% | 3 | F:binding; F:nucleic acid binding; C:intracellular | IPR007087 (PROSITE); IPR013087 (G3DSA:3.30.160.GENE3D); IPR015880 (SMART); PTHR24375 (PANTHER), PTHR24375:SF0 (PANTHER), PF13465 (PFAM), PF13912 (PFAM), SSF57667 (SUPERFAMILY) |

| | | | | | | | | | | |
|---|---|---|---|---|---|---|---|---|---|---|
| 1714 | lib_dib_Filter15_IDBA_contig_29362_plus1 | 1336 | neurogenic locus notch homolog protein 2-like | 494 | 5.00E-104 | 55.65% | 30 | P:multicellular organismal development; P:biological_process; P:cytoskeleton organization; P:anatomical structure morphogenesis; P:cell differentiation; P:cellular component organization; F:nucleotide binding; C:plasma membrane; P:signal transduction; P:reproduction; P:regulation of biological process; P:cell-cell signaling; P:growth; C:protein complex; P:cell proliferation; P:behavior; ; C:organelle; C:cytoplasm; P:embryo development; C:nucleus; P:response to biotic stimulus; P:metabolic process; P:cell cycle; F:chromatin binding; F:protein binding; P:response to external stimulus; P:response to stress; F:DNA binding; F:calcium ion binding | IPR000152 (PROSITE); IPR000742 (PFAM); IPR001881 (SMART); IPR013032 (PFAM); IPR018097 (PROSITE); PD936484 (PRODOM), PR01983 (PRINTS), G3DSA:2.10.25.10 (GENE3D), G3DSA:2.170.300.10 (GENE3D), PTHR24838 (PANTHER), PTHR24838:SF112 (PANTHER), SignalP-NN(euk) (SIGNALP), tmhmm (TMHMM), SSF57196 (SUPERFAMILY) | Yes |
| 1715 | lib_dib_Filter15_IDBA_contig_29379_minus3 | 682 | sarcosine oxidase | 513 | 1.42E-144 | 60.10% | 2 | F:catalytic activity; P:metabolic process | IPR006076 (PFAM); G3DSA:3.30.9.10 (GENE3D), G3DSA:3.50.50.60 (GENE3D), PTHR10961 (PANTHER), PTHR10961:SF7 (PANTHER), tmhmm (TMHMM), SSF51905 (SUPERFAMILY), SSF54373 (SUPERFAMILY) | Yes |
| 1716 | lib_dib_Filter15_IDBA_contig_29380_minus3 | 620 | sarcosine oxidase | 493 | 3.52E-145 | 60.05% | 2 | F:catalytic activity; P:metabolic process | IPR006076 (PFAM); G3DSA:3.30.9.10 (GENE3D), G3DSA:3.50.50.60 (GENE3D), PTHR10961 (PANTHER), PTHR10961:SF7 (PANTHER), tmhmm (TMHMM), SSF51905 (SUPERFAMILY), SSF54373 (SUPERFAMILY) | Yes |
| 1717 | lib_dib_Filter15_IDBA_contig_29389_plus3 | 55 | voltage-dependent calcium channel subunit alpha-2 delta-3 | 344 | 5.45E-172 | 69.65% | 1 | F:protein binding | IPR002035 (SMART); IPR013608 (PFAM); G3DSA:3.30.450.20 (GENE3D), G3DSA:3.40.50.410 (GENE3D), PTHR10166 (PANTHER), PF13519 (PFAM), SSF53300 (SUPERFAMILY) | |
| 1718 | lib_dib_Filter15_IDBA_contig_29476_minus2 | 100 | cytokine receptor-like factor 1 | 473 | 3.96E-15 | 42.60% | 1 | F:protein binding | IPR003961 (SUPERFAMILY); IPR013783 (G3DSA:2.60.40.GENE3D) | |
| 1719 | lib_dib_Filter15_IDBA_contig_29504_minus1 | 53 | extracellular domains-containing protein cg31004-like | 344 | 5.94E-49 | 48.70% | 3 | P:cell-matrix adhesion; F:molecular_function; C:cellular_component | IPR001846 (PFAM); IPR005533 (PFAM); PTHR13802 (PANTHER), PTHR13802:SF1 (PANTHER) | |
| 1720 | lib_dib_Filter15_IDBA_contig_29510_minus1 | 358 | serine threonine-protein phosphatase 6 regulatory ankyrin repeat subunit c-like | 650 | 7.32E-37 | 48.45% | 1 | F:protein binding | IPR001496 (PFAM); IPR002110 (PFAM); IPR020683 (G3DSA:1.25.40.GENE3D); PTHR24198 (PANTHER), tmhmm (TMHMM) | |
| 1721 | lib_dib_Filter15_IDBA_contig_29531_minus2 | 106 | arylsulfatase b | 647 | 3.33E-172 | 60.05% | 2 | P:metabolic process; F:hydrolase activity | IPR000917 (PFAM); IPR017849 (G3DSA:3.40.720.GENE3D); IPR017850 (SUPERFAMILY); IPR024607 (PROSITE); G3DSA:3.30.1120.10 (GENE3D), PTHR10342 (PANTHER), PTHR10342:SF19 (PANTHER) | |
| 1722 | lib_dib_Filter15_IDBA_contig_29532_minus2 | 10 | arylsulfatase j-like | 103 | 5.84E-38 | 76.40% | 2 | F:hydrolase activity; P:metabolic process | IPR000917 (PFAM); IPR017849 (G3DSA:3.40.720.GENE3D); IPR017850 (SUPERFAMILY); IPR024607 (PROSITE); PTHR10342 (PANTHER), PTHR10342:SF19 (PANTHER) | |
| 1723 | lib_dib_Filter15_IDBA_contig_29533_minus2 | 508 | leucine-rich repeat-containing protein 58 | 771 | 2.25E-125 | 69.20% | 1 | F:protein binding | IPR001611 (PFAM); IPR003591 (SMART); IPR025875 (PFAM); G3DSA:3.80.10.10 (GENE3D), PTHR23155 (PANTHER), PTHR23155:SF25 (PANTHER), PF13855 (PFAM), SSF52058 (SUPERFAMILY) | |

| | | | | | | | | | | |
|---|---|---|---|---|---|---|---|---|---|---|
| 1724 | lib_dib_Filter15_IDBA_contig_29534_minus2 | 547 | leucine-rich repeat-containing protein 58 | 820 | 1.07E-124 | 69.45% | 1 | F:protein binding | IPR001611 (PFAM); IPR003591 (SMART); IPR025875 (PFAM); G3DSA:3.80.10.10 (GENE3D), PTHR23155 (PANTHER), PTHR23155:SF25 (PANTHER), SSF52058 (SUPERFAMILY) | |
| 1725 | lib_dib_Filter15_IDBA_contig_29556_plus1 | 202 | neutral ceramidase | 727 | 0 | 66.05% | 6 | P:lipid metabolic process; P:cell proliferation; C:plasma membrane; P:signal transduction; C:mitochondrion; F:hydrolase activity | IPR006823 (PANTHER); tmhmm (TMHMM) | |
| 1726 | lib_dib_Filter15_IDBA_contig_29557_plus1 | 63 | neutral ceramidase | 215 | 2.07E-34 | 56.85% | 1 | C:plasma membrane | IPR006823 (PANTHER); tmhmm (TMHMM) | |
| 1727 | lib_dib_Filter15_IDBA_contig_29558_plus3 | 86 | neutral ceramidase | 271 | 1.03E-60 | 60.20% | 1 | C:plasma membrane | IPR006823 (PANTHER); tmhmm (TMHMM) | |
| 1728 | lib_dib_Filter15_IDBA_contig_29604_plus3 | 33 | vanin 1 | 342 | 4.14E-89 | 61.95% | 12 | P:biological_process; F:hydrolase activity; P:response to external stimulus; P:response to stress; C:cell; C:extracellular space; P:regulation of biological process; P:cell death; F:lipid binding; P:cell differentiation; P:multicellular organismal development; P:metabolic process | IPR003010 (G3DSA:3.60.110.GENE3D); IPR012101 (PANTHER); SignalP-NN(euk) (SIGNALP), tmhmm (TMHMM) | |
| 1729 | lib_dib_Filter15_IDBA_contig_29617_plus1 | 49 | brother of cdo | 342 | 2.02E-63 | 49.80% | 6 | P:biological_process; P:multicellular organismal development; P:cellular component organization; P:cell differentiation; P:regulation of biological process; F:protein binding | IPR003598 (SMART); IPR003599 (SMART); IPR007110 (PROFILE); IPR013098 (PFAM); IPR013106 (PFAM); IPR013783 (G3DSA:2.60.40.GENE3D); PTHR10489 (PANTHER), SSF48726 (SUPERFAMILY) | |
| 1730 | lib_dib_Filter15_IDBA_contig_29626_minus3 | 110 | glycosaminoglycan xylosylkinase | 389 | 7.33E-51 | 75.30% | 3 | C:Golgi apparatus; F:transferase activity; P:metabolic process | IPR024869 (PANTHER); tmhmm (TMHMM) | |
| 1731 | lib_dib_Filter15_IDBA_contig_29636_plus1 | 1559 | carbonic anhydrase | 519 | 1.41E-55 | 54.70% | 3 | F:catalytic activity; P:metabolic process; F:binding | IPR001148 (G3DSA:3.10.200.GENE3D); IPR018338 (PROSITE); IPR023561 (PANTHER); tmhmm (TMHMM) | |
| 1732 | lib_dib_Filter15_IDBA_contig_29652_plus2 | 87 | eukaryotic translation initiation factor 2-alpha kinase 3 | 541 | 4.21E-56 | 60.45% | 22 | P:biological_process; P:multicellular organismal development; P:regulation of biological process; P:response to stress; P:translation; P:cell differentiation; P:organelle organization; P:signal transduction; F:protein kinase activity; P:response to biotic stimulus; P:transport; P:cell-cell signaling; P:cellular component organization; P:cell death; F:protein binding; P:lipid metabolic process; P:cellular protein modification process; P:cell communication; P:cellular homeostasis; P:reproduction; C:endoplasmic reticulum; F:nucleotide binding | IPR000719 (PFAM); IPR002290 (SMART); IPR008271 (PROSITE); IPR011009 (SUPERFAMILY); IPR020635 (SMART); G3DSA:1.10.510.10 (GENE3D), PTHR11042 (PANTHER), PTHR11042:SF15 (PANTHER), tmhmm (TMHMM) | |
| 1733 | lib_dib_Filter15_IDBA_contig_29653_plus2 | 418 | dimethylaniline monooxygenase | 912 | 5.72E-100 | 54.90% | 4 | P:nucleobase-containing compound metabolic process; P:secondary metabolic process; F:catalytic activity; F:nucleotide binding | IPR000960 (PRINTS); IPR020946 (PFAM); G3DSA:3.50.50.60 (GENE3D), PTHR23023 (PANTHER), PTHR23023:SF4 (PANTHER), tmhmm (TMHMM), SSF51905 (SUPERFAMILY) | |
| 1734 | lib_dib_Filter15_IDBA_contig_29660_plus1 | 714 | cub and sushi domain-containing protein 3-like | 991 | 1.21E-161 | 48.55% | 1 | F:calcium ion binding | IPR000152 (PROSITE); IPR000436 (PFAM); IPR001881 (PFAM); IPR009030 (SUPERFAMILY); IPR018097 (PROSITE); G3DSA:2.10.25.10 (GENE3D), G3DSA:2.10.70.10 (GENE3D), PTHR19325 (PANTHER), SignalP-NN(euk) (SIGNALP), tmhmm (TMHMM), SSF57196 (SUPERFAMILY) | Yes |

| | | | | | | | | | | |
|---|---|---|---|---|---|---|---|---|---|---|
| 1735 | lib_dib_Filter15_IDBA_contig_29666_plus2 | 57 | cubitus interruptus | 341 | 3.50E-84 | 95.55% | 17 | P:regulation of biological process; P:cell cycle; ; P:multicellular organismal development; F:protein binding; F:binding; C:cell; P:anatomical structure morphogenesis; P:cellular component organization; P:cell differentiation; C:cytoplasm; P:reproduction; P:signal transduction; P:embryo development; C:nucleus; C:intracellular; C:protein complex | IPR007087 (PFAM); IPR013087 (G3DSA:3.30.160.GENE3D); IPR015880 (SMART); PTHR19818 (PANTHER), PTHR19818:SF7 (PANTHER), PF13465 (PFAM), SSF57667 (SUPERFAMILY) | |
| 1736 | lib_dib_Filter15_IDBA_contig_29671_minus3 | 59 | ankyrin unc44 | 341 | 4.72E-71 | 53.05% | 15 | F:RNA binding; P:RNA-dependent DNA replication; F:RNA-directed DNA polymerase activity; P:proteolysis; F:serine-type endopeptidase activity; F:catalytic activity; F:receptor activity; C:integral to membrane; P:G-protein coupled receptor signaling pathway; F:G-protein coupled receptor activity; P:signal transduction; F:signal transducer activity; F:endonuclease activity; F:transmembrane transporter activity; P:transmembrane transport | PTHR21301 (PANTHER) | |
| 1737 | lib_dib_Filter15_IDBA_contig_29698_minus1 | 144 | peroxisomal trans-2-enoyl-reductase | 376 | 1.11E-121 | 75.95% | 8 | F:catalytic activity; P:metabolic process; C:peroxisome; F:receptor binding; P:biosynthetic process; P:lipid metabolic process; C:mitochondrion; F:nucleotide binding | IPR002198 (PRINTS); IPR002347 (PRINTS); IPR016040 (G3DSA:3.40.50.GENE3D); PTHR24317 (PANTHER), PTHR24317:SF0 (PANTHER), PF13561 (PFAM), SSF51735 (SUPERFAMILY) | |
| 1738 | lib_dib_Filter15_IDBA_contig_29700_plus2 | 225 | epidermal retinol dehydrogenase 2-like | 412 | 3.20E-117 | 69.35% | 3 | P:metabolic process; F:catalytic activity; F:nucleotide binding | IPR002198 (PRINTS); IPR002347 (PRINTS); IPR016040 (G3DSA:3.40.50.GENE3D); PTHR24316 (PANTHER), PTHR24316:SF1 (PANTHER), SSF51735 (SUPERFAMILY) | |
| 1739 | lib_dib_Filter15_IDBA_contig_29786_minus1 | 118 | nicotinic acetylcholine receptor alpha 4-1 subunit | 578 | 2.84E-65 | 53.90% | 3 | P:ion transport; C:cell; F:ion channel activity | IPR006029 (SUPERFAMILY); IPR006201 (PRINTS); IPR006202 (G3DSA:2.70.170.GENE3D); IPR018000 (PROSITE); G3DSA:1.20.120.370 (GENE3D), PTHR18945:SF127 (PANTHER), SignalP-NN(euk) (SIGNALP), tmhmm (TMHMM) | Yes |
| 1740 | lib_dib_Filter15_IDBA_contig_29824_plus1 | 184 | ankyrin repeat protein | 691 | 6.12E-44 | 49.25% | 1 | F:protein binding | IPR001496 (PFAM); IPR002110 (SMART); IPR020683 (G3DSA:1.25.40.GENE3D); PTHR24188 (PANTHER) | |
| 1741 | lib_dib_Filter15_IDBA_contig_29827_minus2 | 79 | otopetrin-2-like isoform 2 | 396 | 2.94E-48 | 59.55% | 0 | - | IPR004878 (PANTHER); PTHR21522:SF20 (PANTHER), SignalP-NN(euk) (SIGNALP), tmhmm (TMHMM) | |
| 1742 | lib_dib_Filter15_IDBA_contig_29828_plus2 | 30 | zinc finger | 339 | 3.28E-13 | 57.30% | 3 | F:binding; F:nucleic acid binding; C:intracellular | IPR007087 (PROSITE); IPR013087 (G3DSA:3.30.160.GENE3D); IPR015880 (SMART); PTHR11389 (PANTHER), PF13465 (PFAM), SSF57667 (SUPERFAMILY) | |
| 1743 | lib_dib_Filter15_IDBA_contig_29843_minus2 | 52 | protein | 339 | 2.25E-11 | 53.35% | 1 | F:carbohydrate binding | IPR000922 (PFAM); PTHR12011 (PANTHER), PTHR12011:SF106 (PANTHER) | |
| 1744 | lib_dib_Filter15_IDBA_contig_29848_minus1 | 1761 | nucleolar protein 58 | 1033 | 0 | 86.85% | 5 | C:nucleolus; C:cytoplasm; C:intracellular; F:RNA binding; C:nucleoplasm | IPR002687 (PFAM); IPR012974 (PFAM); IPR012976 (PFAM); PTHR10894 (PANTHER), SSF89124 (SUPERFAMILY) | |
| 1745 | lib_dib_Filter15_IDBA_contig_29849_minus1 | 1849 | nucleolar protein 58 | 1164 | 0 | 86.80% | 5 | C:nucleolus; C:cytoplasm; C:intracellular; F:RNA binding; C:nucleoplasm | IPR002687 (PFAM); IPR012974 (PFAM); IPR012976 (PFAM); PTHR10894 (PANTHER), SSF89124 (SUPERFAMILY) | |

| | | | | | | | | | | |
|---|---|---|---|---|---|---|---|---|---|---|
| 1746 | lib_dib_Filter15_IDBA_contig_29894_minus2 | 841 | isoform cra_a | 541 | 0 | 74.20% | 6 | C:extracellular space; F:binding; F:hydrolase activity; P:carbohydrate metabolic process; P:catabolic process; F:calcium ion binding | IPR006046 (PRINTS); IPR006047 (PFAM); IPR006048 (PFAM); IPR006589 (SMART); IPR013780 (G3DSA:2.60.40.GENE3D); IPR013781 (G3DSA:3.20.20.GENE3D); IPR015902 (PANTHER); IPR017853 (SUPERFAMILY); PTHR10357:SF26 (PANTHER), SignalP-NN(euk) (SIGNALP), SSF51011 (SUPERFAMILY) | Yes |
| 1747 | lib_dib_Filter15_IDBA_contig_29919_plus3 | 265 | u4 u6 small nuclear ribonucleoprotein prp31 | 711 | 0 | 83.05% | 7 | C:nucleus; F:binding; C:nucleoplasm; C:protein complex; F:RNA binding; P:cellular component organization; P:nucleobase-containing compound metabolic process | IPR002687 (PFAM); IPR012976 (PFAM); IPR019175 (PFAM); IPR027105 (PANTHER); SSF89124 (SUPERFAMILY) | |
| 1748 | lib_dib_Filter15_IDBA_contig_29921_plus2 | 455 | acidic mammalian chitinase-like | 338 | 2.05E-21 | 86.15% | 6 | F:hydrolase activity; P:carbohydrate metabolic process; P:catabolic process; F:binding; F:carbohydrate binding; C:extracellular region | IPR001223 (PFAM); IPR013781 (G3DSA:3.20.20.GENE3D); IPR017853 (SUPERFAMILY); PTHR11177 (PANTHER), PTHR11177:SF34 (PANTHER) | |
| 1749 | lib_dib_Filter15_IDBA_contig_29923_plus3 | 1142 | brain chitinase and chia | 106 | 3.80E-31 | 67.35% | 6 | C:extracellular space; F:hydrolase activity; P:carbohydrate metabolic process; P:catabolic process; P:response to biotic stimulus; F:carbohydrate binding | IPR001223 (PFAM); IPR013781 (G3DSA:3.20.20.GENE3D); IPR017853 (SUPERFAMILY); G3DSA:3.10.50.10 (GENE3D), PTHR11177 (PANTHER), PTHR11177:SF34 (PANTHER), SSF54556 (SUPERFAMILY) | |
| 1750 | lib_dib_Filter15_IDBA_contig_29924_plus1 | 1286 | chitotriosidase-1 isoform 2 | 304 | 5.05E-121 | 74.05% | 7 | C:extracellular space; F:hydrolase activity; P:carbohydrate metabolic process; P:catabolic process; P:response to biotic stimulus; F:carbohydrate binding; P:biological_process | IPR001223 (PFAM); IPR001579 (PROSITE); IPR011583 (SMART); IPR013781 (G3DSA:3.20.20.GENE3D); IPR017853 (SUPERFAMILY); G3DSA:3.10.50.10 (GENE3D), PTHR11177 (PANTHER), PTHR11177:SF34 (PANTHER), SignalP-NN(euk) (SIGNALP), SSF54556 (SUPERFAMILY) | Yes |
| 1751 | lib_dib_Filter15_IDBA_contig_29952_minus2 | 263 | pleiotropic regulator 1 | 606 | 0 | 74.75% | 6 | F:signal transducer activity; F:protein binding; F:transcription regulator activity; P:nucleobase-containing compound metabolic process; P:signal transduction; C:nucleus | IPR001680 (PFAM); IPR015943 (G3DSA:2.130.10.GENE3D); IPR017986 (PROFILE); IPR019775 (PROSITE); IPR020472 (PRINTS); PTHR19923 (PANTHER) | |
| 1752 | lib_dib_Filter15_IDBA_contig_29965_minus3 | 80 | dual specificity mitogen-activated protein kinase kinase 7 isoform 1 | 337 | 2.81E-157 | 74.30% | 11 | F:protein binding; P:signal transduction; P:response to stress; P:cellular protein modification process; C:cytosol; F:binding; F:protein kinase activity; P:regulation of biological process; P:cell death; C:nucleus; F:nucleotide binding | IPR000719 (PFAM); IPR002290 (SMART); IPR008271 (PROSITE); IPR011009 (SUPERFAMILY); IPR020635 (SMART); G3DSA:1.10.510.10 (GENE3D), G3DSA:3.30.200.20 (GENE3D), PTHR24360 (PANTHER), PTHR24360:SF1 (PANTHER) | |
| 1753 | lib_dib_Filter15_IDBA_contig_29992_plus3 | 472 | bone morphogenetic protein 5 | 1169 | 1.42E-21 | 41.50% | 5 | P:multicellular organismal development; P:biological_process; P:regulation of biological process; F:receptor binding; P:growth | IPR001111 (PFAM); IPR001839 (PFAM); IPR015615 (PANTHER); IPR017948 (PROSITE); G3DSA:2.10.90.10 (GENE3D), SSF57501 (SUPERFAMILY) | |
| 1754 | lib_dib_Filter15_IDBA_contig_29996_minus1 | 199 | coagulation factor x | 641 | 6.38E-105 | 55.05% | 3 | F:peptidase activity; P:protein metabolic process; P:catabolic process | IPR000884 (SMART); IPR001254 (PFAM); IPR001314 (PRINTS); IPR009003 (SUPERFAMILY); IPR018114 (PROSITE); G3DSA:2.20.100.10 (GENE3D), G3DSA:2.40.10.10 (GENE3D), PTHR24265 (PANTHER), tmhmm (TMHMM) | |
| 1755 | lib_dib_Filter15_IDBA_contig_29999_plus1 | 618 | n-acetylgalactosamine-6-sulfatase precursor | 824 | 0 | 78.70% | 4 | C:lysosome; F:binding; P:metabolic process; F:hydrolase activity | IPR000917 (PFAM); IPR017849 (G3DSA:3.40.720.GENE3D); IPR017850 (SUPERFAMILY); IPR024607 (PROSITE); G3DSA:3.30.1120.10 (GENE3D), PTHR10342 (PANTHER), PTHR10342:SF27 (PANTHER), SignalP-NN(euk) (SIGNALP) | Yes |
| 1756 | lib_dib_Filter15_IDBA_contig_3_minus2 | 81 | collagen alpha-1 chain-like | 407 | 6.08E-32 | 51.15% | 1 | F:protein binding | IPR002035 (PFAM); PR00453 (PRINTS), G3DSA:3.40.50.410 (GENE3D), PTHR22992 (PANTHER), SSF53300 (SUPERFAMILY) | |

| | | | | | | | | | | |
|---|---|---|---|---|---|---|---|---|---|---|
| 1757 | lib_dib_Filter15_IDBA_contig_30013_minus3 | 40 | endoplasmic reticulum mannosyl-oligosaccharide - alpha-mannosidase-like | 336 | 4.88E-162 | 79.00% | 8 | P:protein metabolic process; P:catabolic process; F:calcium ion binding; P:biosynthetic process; P:cellular protein modification process; P:carbohydrate metabolic process; C:cell; F:hydrolase activity | IPR001382 (PRINTS); PTHR11742:SF7 (PANTHER) | |
| 1758 | lib_dib_Filter15_IDBA_contig_30015_minus1 | 53 | zinc finger protein 2 homolog | 337 | 3.08E-66 | 51.25% | 3 | F:binding; F:nucleic acid binding; C:intracellular | IPR007087 (PFAM); IPR013087 (G3DSA:3.30.160.GENE3D); IPR015880 (SMART); PTHR24402 (PANTHER), PF13465 (PFAM), PF13894 (PFAM), SSF57667 (SUPERFAMILY) | |
| 1759 | lib_dib_Filter15_IDBA_contig_30040_plus1 | 212 | peptidylglycine alpha-hydroxylating monooxygenase | 522 | 3.05E-135 | 62.50% | 4 | F:binding; P:metabolic process; F:catalytic activity; C:cell | IPR000323 (G3DSA:2.60.120.GENE3D); IPR000720 (PRINTS); IPR008977 (SUPERFAMILY); IPR014784 (G3DSA:2.60.120.GENE3D); PTHR10680 (PANTHER), PTHR10680:SF11 (PANTHER), PF03712 (PFAM), SignalP-NN(euk) (SIGNALP), tmhmm (TMHMM) | |
| 1760 | lib_dib_Filter15_IDBA_contig_30045_minus1 | 135 | cgmp-dependent protein kinase 1 isoform 2 | 673 | 0 | 82.15% | 14 | P:cellular component organization; P:cell differentiation; P:multicellular organismal development; P:biological_process; F:protein binding; C:Golgi apparatus; C:cytosol; P:cytoskeleton organization; P:cellular protein modification process; F:protein kinase activity; P:signal transduction; C:plasma membrane; F:nucleotide binding; F:molecular_function | IPR000595 (PFAM); IPR000719 (PFAM); IPR000961 (SMART); IPR002290 (SMART); IPR002374 (PRINTS); IPR008271 (PROSITE); IPR011009 (SUPERFAMILY); IPR014710 (G3DSA:2.60.120.GENE3D); IPR017441 (PROSITE); IPR018488 (PROSITE); IPR018490 (SUPERFAMILY); IPR020635 (SMART); G3DSA:1.10.510.10 (GENE3D), G3DSA:3.30.200.20 (GENE3D), PTHR24353 (PANTHER), PTHR24353:SF2 (PANTHER) | |
| 1761 | lib_dib_Filter15_IDBA_contig_30075_plus3 | 65 | ankyrin repeat protein | 336 | 3.12E-18 | 52.75% | 1 | F:protein binding | IPR002110 (PFAM); IPR020683 (G3DSA:1.25.40.GENE3D); PTHR24188 (PANTHER) | |
| 1762 | lib_dib_Filter15_IDBA_contig_30078_minus2 | 105 | gcc2 and gcc3 domain containing protein | 440 | 2.22E-55 | 45.45% | 3 | F:zinc ion binding; C:intracellular; F:carbohydrate binding | IPR009030 (SUPERFAMILY); G3DSA:2.10.50.10 (GENE3D), PTHR22727 (PANTHER), PTHR22727:SF4 (PANTHER), tmhmm (TMHMM) | |
| 1763 | lib_dib_Filter15_IDBA_contig_30114_plus2 | 34 | zinc finger protein 341 | 336 | 4.40E-35 | 72.60% | 3 | C:nucleolus; F:nucleic acid binding; F:binding | IPR007087 (PFAM); IPR013087 (G3DSA:3.30.160.GENE3D); IPR015880 (SMART); PTHR24387 (PANTHER), PTHR24387:SF10 (PANTHER), PF13894 (PFAM), SSF57667 (SUPERFAMILY) | |
| 1764 | lib_dib_Filter15_IDBA_contig_30152_plus1 | 75 | a disintegrin and metalloproteinase with thrombospondin motifs 9 | 336 | 2.47E-138 | 76.15% | 7 | P:catabolic process; P:regulation of biological process; P:protein metabolic process; P:multicellular organismal development; C:proteinaceous extracellular matrix; F:peptidase activity; F:binding | IPR000884 (PFAM); IPR010294 (PFAM); IPR013273 (PRINTS); G3DSA:2.20.100.10 (GENE3D), PTHR13723 (PANTHER), PTHR13723:SF33 (PANTHER) | |
| 1765 | lib_dib_Filter15_IDBA_contig_30158_plus2 | 173 | zinc finger protein 143 isoform 2 | 817 | 5.09E-157 | 65.60% | 6 | ; P:regulation of biological process; C:nucleoplasm; F:nucleic acid binding; F:protein binding; F:binding | IPR007087 (PFAM); IPR013087 (G3DSA:3.30.160.GENE3D); IPR015880 (SMART); PTHR11389 (PANTHER), PTHR11389:SF362 (PANTHER), PF13465 (PFAM), SSF57667 (SUPERFAMILY) | |
| 1766 | lib_dib_Filter15_IDBA_contig_30191_minus1 | 48 | chymotrypsinogen a-like | 336 | 1.70E-85 | 61.95% | 3 | P:protein metabolic process; P:catabolic process; F:peptidase activity | IPR001254 (PFAM); IPR001314 (PRINTS); IPR009003 (SUPERFAMILY); IPR018114 (PROSITE); G3DSA:2.40.10.10 (GENE3D), PTHR24256 (PANTHER), tmhmm (TMHMM) | |
| 1767 | lib_dib_Filter15_IDBA_contig_30224_plus2 | 966 | angiotensin-converting enzyme-like | 863 | 0 | 71.45% | 8 | P:biological_process; P:catabolic process; F:binding; F:peptidase activity; C:cell; F:protein binding; C:extracellular region; P:protein metabolic process | IPR001548 (PRINTS); SignalP-NN(euk) (SIGNALP), tmhmm (TMHMM), SSF55486 (SUPERFAMILY) | Yes |

| | | | | | | | | | |
|---|---|---|---|---|---|---|---|---|---|
| 1768 | lib_dib_Filter15_IDBA_contig_30237_plus2 | 1104 | transmembrane protease serine 9 | 730 | 1.55E-47 | 56.55% | 3 | F:peptidase activity; P:protein metabolic process; P:catabolic process | IPR000562 (G3DSA:2.10.10.GENE3D); IPR001254 (PFAM); IPR001314 (PRINTS); IPR009003 (SUPERFAMILY); IPR013806 (SUPERFAMILY); IPR018114 (PROSITE); PR00013 (PRINTS), G3DSA:2.40.10.10 (GENE3D), PTHR24256 (PANTHER), SignalP-NN(euk) (SIGNALP), tmhmm (TMHMM) |
| 1769 | lib_dib_Filter15_IDBA_contig_30238_plus3 | 538 | transmembrane protease serine 9 | 505 | 1.04E-48 | 56.65% | 3 | F:peptidase activity; P:protein metabolic process; P:catabolic process | IPR000562 (G3DSA:2.10.10.GENE3D); IPR001254 (PFAM); IPR001314 (PRINTS); IPR009003 (SUPERFAMILY); IPR013806 (SUPERFAMILY); IPR018114 (PROSITE); PR00013 (PRINTS), G3DSA:2.40.10.10 (GENE3D), PTHR24256 (PANTHER) |
| 1770 | lib_dib_Filter15_IDBA_contig_30253_plus1 | 49 | sh3 domain-containing kinase-binding protein 1-like | 335 | 2.23E-51 | 54.45% | 10 | P:biological_process; C:organelle; C:intracellular; F:protein binding; P:regulation of biological process; P:cell communication; P:cellular component organization; C:cytoplasm; C:cell; C:nucleus | IPR000108 (PRINTS); IPR001452 (PRINTS); IPR011511 (PFAM); G3DSA:2.30.30.40 (GENE3D), PTHR14167 (PANTHER), PTHR14167:SF7 (PANTHER) |
| 1771 | lib_dib_Filter15_IDBA_contig_30258_plus3 | 177 | c3po protein | 717 | 2.59E-32 | 44.05% | 8 | F:RNA binding; F:calcium ion binding; F:double-stranded RNA binding; C:intracellular; F:molecular_function; P:extracellular matrix organization; P:chitin localization; C:cellular_component | IPR000859 (G3DSA:2.60.120.GENE3D); PTHR10127 (PANTHER), SignalP-NN(euk) (SIGNALP), tmhmm (TMHMM) |
| 1772 | lib_dib_Filter15_IDBA_contig_30280_minus3 | 100 | dipeptidyl peptidase 4 | 362 | 5.50E-113 | 69.30% | 6 | F:protein binding; P:regulation of biological process; C:cell; P:protein metabolic process; P:catabolic process; F:peptidase activity | IPR001375 (PFAM); G3DSA:3.40.50.1820 (GENE3D), PTHR11731 (PANTHER), PTHR11731:SF24 (PANTHER), SSF53474 (SUPERFAMILY) |
| 1773 | lib_dib_Filter15_IDBA_contig_30284_minus1 | 162 | deleted in malignant brain tumors 1 | 540 | 1.20E-68 | 45.30% | 3 | F:catalytic activity; F:receptor activity; C:cell | IPR001190 (PRINTS); IPR017448 (SMART); G3DSA:3.10.250.10 (GENE3D), PTHR19331 (PANTHER), PTHR19331:SF118 (PANTHER) |
| 1774 | lib_dib_Filter15_IDBA_contig_30328_minus1 | 95 | mitogen-activated protein kinase kinase kinase 3 | 334 | 3.58E-36 | 59.70% | 5 | P:cellular protein modification process; P:signal transduction; F:protein binding; F:nucleotide binding; F:protein kinase activity | IPR000719 (PFAM); IPR002290 (SMART); IPR008271 (PROSITE); IPR011009 (SUPERFAMILY); IPR020635 (SMART); G3DSA:1.10.510.10 (GENE3D), PTHR24361 (PANTHER) |
| 1775 | lib_dib_Filter15_IDBA_contig_30395_minus1 | 430 | ankyrin repeat protein | 531 | 5.08E-30 | 48.85% | 1 | F:protein binding | IPR001496 (PFAM); IPR002110 (PRINTS); IPR020683 (G3DSA:1.25.40.GENE3D); PTHR24125 (PANTHER), PTHR24125:SF0 (PANTHER) |
| 1776 | lib_dib_Filter15_IDBA_contig_30401_plus1 | 98 | estradiol 17-beta-dehydrogenase 11-like | 383 | 8.45E-67 | 57.80% | 5 | P:embryo development; P:response to stress; P:metabolic process; F:catalytic activity; F:nucleotide binding | IPR002198 (PRINTS); IPR002347 (PRINTS); IPR016040 (G3DSA:3.40.50.GENE3D); IPR020904 (PROSITE); PTHR24316 (PANTHER), PTHR24316:SF1 (PANTHER), SignalP-NN(euk) (SIGNALP), tmhmm (TMHMM), SSF51735 (SUPERFAMILY) |
| 1777 | lib_dib_Filter15_IDBA_contig_30404_plus2 | 107 | latrophilin isoform cra_h | 340 | 4.01E-42 | 54.60% | 5 | C:cell; P:multicellular organismal development; F:protein binding; P:signal transduction; F:receptor activity | IPR000203 (PFAM); IPR000832 (PRINTS); IPR017452 (PROFILE); IPR017981 (PROFILE); G3DSA:1.20.1070.10 (GENE3D), PTHR12011 (PANTHER), tmhmm (TMHMM) |
| 1778 | lib_dib_Filter15_IDBA_contig_30405_plus1 | 196 | latrophilin- partial | 637 | 3.15E-55 | 45.20% | 6 | P:signal transduction; F:receptor activity; C:cell; P:anatomical structure morphogenesis; P:regulation of biological process; P:multicellular organismal development | IPR000203 (PFAM); IPR000832 (PRINTS); IPR001879 (PFAM); IPR008077 (PRINTS); IPR017452 (PROFILE); IPR017981 (PROFILE); IPR022624 (PFAM); G3DSA:1.20.1070.10 (GENE3D), PTHR12011 (PANTHER), SignalP-NN(euk) (SIGNALP), tmhmm (TMHMM) |

| | | | | | | | | | |
|---|---|---|---|---|---|---|---|---|---|
| 1779 | lib_dib_Filter15_IDBA_contig_30410_plus1 | 575 | protein sidekick-2-like | 333 | 8.72E-21 | 45.95% | 1 | F:protein binding | IPR003598 (SMART); IPR003599 (SMART); IPR007110 (PROFILE); IPR013098 (PFAM); IPR013783 (G3DSA:2.60.40.GENE3D); PTHR10489 (PANTHER), tmhmm (TMHMM), SSF48726 (SUPERFAMILY) | |
| 1780 | lib_dib_Filter15_IDBA_contig_30422_minus2 | 48 | proto-oncogene tyrosine-protein kinase lck | 333 | 1.92E-17 | 47.60% | 2 | F:catalytic activity; F:protein binding | IPR001611 (PROFILE); IPR003591 (SMART); G3DSA:3.80.10.10 (GENE3D), PTHR24367 (PANTHER), PTHR24367:SF68 (PANTHER), PF13855 (PFAM), SSF52058 (SUPERFAMILY) | |
| 1781 | lib_dib_Filter15_IDBA_contig_30424_minus3 | 86 | 1-phosphatidylinositol phosphodiesterase-like | 342 | 8.63E-44 | 51.80% | 2 | P:lipid metabolic process; F:hydrolase activity | IPR000909 (PFAM); IPR017946 (G3DSA:3.20.20.GENE3D); PTHR13593 (PANTHER), SignalP-NN(euk) (SIGNALP) | Yes |
| 1782 | lib_dib_Filter15_IDBA_contig_30426_minus2 | 71 | fat-like cadherin-related tumor suppressor-like protein | 545 | 1.55E-167 | 64.10% | 3 | F:calcium ion binding; P:biological_process; C:plasma membrane | IPR002126 (PRINTS); IPR015919 (SUPERFAMILY); IPR020894 (PROSITE); PTHR24027 (PANTHER), PTHR24027:SF110 (PANTHER) | |
| 1783 | lib_dib_Filter15_IDBA_contig_30462_minus3 | 4819 | a disintegrin and metalloproteinase with thrombospondin motifs 3 | 624 | 2.38E-18 | 44.05% | 3 | F:peptidase activity; P:protein metabolic process; P:catabolic process | IPR001590 (PROFILE); IPR024079 (G3DSA:3.40.390.GENE3D); PTHR13723 (PANTHER), PF13574 (PFAM), SignalP-NN(euk) (SIGNALP), SSF55486 (SUPERFAMILY) | Yes |
| 1784 | lib_dib_Filter15_IDBA_contig_30464_minus3 | 10926 | a disintegrin and metalloproteinase with thrombospondin motifs 13 | 843 | 1.29E-17 | 43.25% | 3 | F:peptidase activity; P:protein metabolic process; P:catabolic process | IPR001590 (PFAM); IPR024079 (G3DSA:3.40.390.GENE3D); PTHR13723 (PANTHER), SignalP-NN(euk) (SIGNALP), SSF55486 (SUPERFAMILY) | Yes |
| 1785 | lib_dib_Filter15_IDBA_contig_30465_minus3 | 788 | a disintegrin and metalloproteinase with thrombospondin motifs 13 | 332 | 3.15E-12 | 49.25% | 3 | F:peptidase activity; P:protein metabolic process; P:catabolic process | IPR001590 (PROFILE); IPR024079 (G3DSA:3.40.390.GENE3D); PTHR11905 (PANTHER), PF13688 (PFAM), SSF55486 (SUPERFAMILY) | |
| 1786 | lib_dib_Filter15_IDBA_contig_30468_minus1 | 295 | a disintegrin and metalloproteinase with thrombospondin motifs 3 | 135 | 5.39E-07 | 47.95% | 15 | P:proteolysis; F:hydrolase activity; F:peptidase activity; F:metalloendopeptidase activity; F:metallopeptidase activity; C:extracellular matrix; F:zinc ion binding; C:proteinaceous extracellular matrix; C:extracellular region; F:metal ion binding; P:protein processing; P:collagen catabolic process; F:heparin binding; P:collagen fibril organization; P:integrin-mediated signaling pathway | PTHR13723 (PANTHER) | |
| 1787 | lib_dib_Filter15_IDBA_contig_30482_plus3 | 76 | ---NA--- | 332 | | | 0 | - | no IPS match | |
| 1788 | lib_dib_Filter15_IDBA_contig_30487_plus1 | 79 | latrophilin 3-like | 511 | 6.77E-97 | 57.00% | 3 | P:signal transduction; F:receptor activity; C:cell | IPR000203 (PFAM); IPR000832 (PRINTS); IPR017981 (PROFILE); PTHR12011 (PANTHER), PTHR12011:SF92 (PANTHER), tmhmm (TMHMM), SSF81321 (SUPERFAMILY) | |
| 1789 | lib_dib_Filter15_IDBA_contig_3052_plus2 | 262 | fad-linked sulfhydryl oxidase alr-like | 370 | 2.31E-57 | 66.15% | 7 | C:extracellular region; F:catalytic activity; P:multicellular organismal development; C:nucleus; C:mitochondrion; F:protein binding; P:metabolic process | IPR006863 (G3DSA:1.20.120.GENE3D); IPR017905 (PROFILE); PTHR12645 (PANTHER), PTHR12645:SF0 (PANTHER) | |
| 1790 | lib_dib_Filter15_IDBA_contig_3054_plus3 | 6586 | staphylococcal nuclease domain-containing protein 1 | 1499 | 0 | 73.65% | 8 | C:organelle; C:intracellular; F:hydrolase activity; P:metabolic process; P:regulation of biological process; C:cytoplasm; ; F:nucleic acid binding | IPR002999 (PFAM); IPR006021 (G3DSA:2.40.50.GENE3D); IPR016071 (SUPERFAMILY); G3DSA:2.30.30.140 (GENE3D), PTHR12302 (PANTHER), PTHR12302:SF0 (PANTHER), tmhmm (TMHMM), SSF63748 (SUPERFAMILY) | |
| 1791 | lib_dib_Filter15_IDBA_contig_30546_minus1 | 1083 | cubilin | 854 | 4.97E-42 | 45.00% | 8 | P:transport; F:protein binding; C:organelle; C:intracellular; P:lipid metabolic process; C:cell; C:plasma membrane; C:cytoplasm | IPR000859 (G3DSA:2.60.120.GENE3D); PTHR10127 (PANTHER), PTHR10127:SF345 (PANTHER), tmhmm (TMHMM) | |

| | | | | | | | | |
|---|---|---|---|---|---|---|---|---|
| 1792 | lib_dib_Filter15_IDBA_contig_30547_minus1 | 1057 | cubilin | 844 | 2.83E-42 | 44.85% | 8 | P:transport; F:protein binding; C:organelle; C:intracellular; P:lipid metabolic process; C:cell; C:plasma membrane; C:cytoplasm | IPR000859 (G3DSA:2.60.120.GENE3D); PTHR10127 (PANTHER), PTHR10127:SF345 (PANTHER), tmhmm (TMHMM) |
| 1793 | lib_dib_Filter15_IDBA_contig_30549_minus3 | 472 | collagen alpha-5 chain | 331 | 3.45E-30 | 50.00% | 1 | F:protein binding | IPR002035 (PFAM); PR00453 (PRINTS), G3DSA:3.40.50.410 (GENE3D), PTHR22992 (PANTHER), tmhmm (TMHMM), SSF53300 (SUPERFAMILY) |
| 1794 | lib_dib_Filter15_IDBA_contig_30556_minus2 | 112 | egf-like domain-containing protein | 556 | 3.65E-16 | 59.15% | 3 | C:extracellular region; F:protein binding; F:calcium ion binding | IPR000152 (PROSITE); IPR000742 (SMART); IPR001881 (PFAM); IPR008160 (PFAM); IPR013032 (PROSITE); IPR018097 (PROSITE); G3DSA:2.10.25.10 (GENE3D), PTHR24048 (PANTHER), tmhmm (TMHMM), SSF57196 (SUPERFAMILY) |
| 1795 | lib_dib_Filter15_IDBA_contig_30557_minus2 | 19 | egf-like domain-containing protein | 166 | 3.95E-17 | 60.05% | 3 | C:extracellular region; F:protein binding; F:calcium ion binding | IPR000152 (PROSITE); IPR000742 (SMART); IPR001881 (PFAM); IPR008160 (PFAM); IPR013032 (PROSITE); IPR018097 (PROSITE); G3DSA:2.10.25.10 (GENE3D), PTHR24048 (PANTHER), SSF57196 (SUPERFAMILY) |
| 1796 | lib_dib_Filter15_IDBA_contig_30584_plus3 | 620 | protein jagged-2-like | 385 | 7.97E-28 | 41.15% | 4 | F:protein binding; P:signal transduction; P:multicellular organismal development; C:cell | IPR000742 (PFAM); IPR001774 (PFAM); IPR011651 (PFAM); IPR013032 (PROSITE); G3DSA:2.10.25.10 (GENE3D), PTHR24044 (PANTHER), PTHR24044:SF49 (PANTHER), tmhmm (TMHMM), SSF57196 (SUPERFAMILY) |
| 1797 | lib_dib_Filter15_IDBA_contig_30588_plus2 | 109 | protein jagged-2 | 139 | 5.96E-11 | 56.30% | 5 | P:biological_process; P:regulation of biological process; P:multicellular organismal development; F:protein binding; F:calcium ion binding | IPR000152 (PROSITE); IPR000742 (PFAM); IPR001881 (SMART); IPR013032 (PROSITE); G3DSA:2.10.25.10 (GENE3D), PTHR24044 (PANTHER), SSF57196 (SUPERFAMILY) |
| 1798 | lib_dib_Filter15_IDBA_contig_30589_plus2 | 180 | neurogenic locus notch-like protein 2 | 139 | 1.44E-11 | 53.55% | 3 | P:biological_process; P:multicellular organismal development; F:protein binding | IPR000152 (PROSITE); IPR000742 (PFAM); IPR013032 (PROSITE); G3DSA:2.10.25.10 (GENE3D), PTHR24044 (PANTHER), SSF57196 (SUPERFAMILY) |
| 1799 | lib_dib_Filter15_IDBA_contig_30590_plus2 | 333 | protein jagged-2-like | 241 | 9.99E-25 | 44.60% | 6 | P:cell differentiation; P:multicellular organismal development; F:protein binding; P:cell communication; C:cell; F:calcium ion binding | IPR000152 (PROSITE); IPR000742 (PFAM); IPR001774 (PFAM); IPR001881 (PFAM); IPR013032 (PROSITE); IPR018097 (PROSITE); PR00011 (PRINTS), G3DSA:2.10.25.10 (GENE3D), PTHR24033 (PANTHER), SSF57196 (SUPERFAMILY) |
| 1800 | lib_dib_Filter15_IDBA_contig_30601_plus1 | 251 | von willebrand factor type egf and pentraxin domain-containing protein 1-like | 692 | 1.15E-11 | 42.55% | 1 | F:protein binding | IPR002035 (PFAM); G3DSA:3.40.50.410 (GENE3D), PTHR22992 (PANTHER), tmhmm (TMHMM), SSF53300 (SUPERFAMILY) |
| 1801 | lib_dib_Filter15_IDBA_contig_30630_plus2 | 523 | aggrecan core protein | 331 | 4.31E-13 | 43.15% | 1 | F:carbohydrate binding | IPR001304 (PFAM); IPR016186 (G3DSA:3.10.100.GENE3D); IPR016187 (SUPERFAMILY); IPR018378 (PROSITE); PTHR22802 (PANTHER), tmhmm (TMHMM) |
| 1802 | lib_dib_Filter15_IDBA_contig_30688_plus1 | 580 | retinoid-inducible serine carboxypeptidase | 521 | 3.50E-71 | 49.90% | 3 | F:peptidase activity; P:protein metabolic process; P:catabolic process | IPR001563 (PRINTS); G3DSA:3.40.50.1820 (GENE3D), PTHR11802:SF3 (PANTHER), tmhmm (TMHMM), SSF53474 (SUPERFAMILY) |
| 1803 | lib_dib_Filter15_IDBA_contig_30689_plus1 | 58 | serine carboxypeptidase-like 51 | 165 | 5.92E-14 | 57.40% | 3 | F:peptidase activity; P:protein metabolic process; P:catabolic process | IPR001563 (PANTHER); G3DSA:3.40.50.1820 (GENE3D), PTHR11802:SF3 (PANTHER), tmhmm (TMHMM), SSF53474 (SUPERFAMILY) |
| 1804 | lib_dib_Filter15_IDBA_contig_30709_minus2 | 234 | periodic tryptophan protein 2 homolog | 579 | 0 | 79.30% | 3 | C:nucleolus; C:cytoplasm; F:protein binding | IPR001680 (PFAM); IPR007148 (PFAM); IPR015943 (G3DSA:2.130.10.GENE3D); IPR017986 (PROFILE); IPR019775 (PROSITE); IPR020472 (PRINTS); IPR027145 (PANTHER) |

| | | | | | | | | | | |
|---|---|---|---|---|---|---|---|---|---|---|
| 1805 | lib_dib_Filter15_IDBA_contig_30710_minus2 | 29 | periodic tryptophan protein 2 homolog | 218 | 6.26E-85 | 82.50% | 5 | C:nucleolus; F:signal transducer activity; C:cytoplasm; P:signal transduction; F:protein binding | IPR001680 (PFAM); IPR011047 (SUPERFAMILY); IPR015943 (G3DSA:2.130.10.GENE3D); IPR017986 (PROFILE); IPR019775 (PROSITE); IPR027145 (PANTHER) | |
| 1806 | lib_dib_Filter15_IDBA_contig_30775_minus3 | 103 | growth hormone i | 329 | 1.34E-148 | 97.80% | 3 | C:extracellular region; F:binding; F:receptor binding | IPR001400 (PRINTS); IPR009079 (SUPERFAMILY); IPR012351 (G3DSA:1.20.1250.GENE3D); IPR018116 (PROSITE); PTHR11417:SF2 (PANTHER), SignalP-NN(euk) (SIGNALP), tmhmm (TMHMM) | Yes |
| 1807 | lib_dib_Filter15_IDBA_contig_30781_plus3 | 557 | insulin-degrading enzyme | 851 | 0 | 74.70% | 19 | P:protein metabolic process; P:catabolic process; P:multicellular organismal development; F:receptor binding; F:binding; P:signal transduction; P:response to endogenous stimulus; C:extracellular space; F:protein binding; C:peroxisome; C:cytosol; F:peptidase activity; P:metabolic process; P:cellular component organization; P:cellular homeostasis; P:regulation of biological process; C:nucleus; F:nucleotide binding; C:mitochondrion | IPR007863 (PFAM); IPR011237 (G3DSA:3.30.830.GENE3D); IPR011249 (SUPERFAMILY); PTHR11851 (PANTHER), PTHR11851:SF64 (PANTHER) | |
| 1808 | lib_dib_Filter15_IDBA_contig_30782_plus3 | 712 | insulin-degrading enzyme | 1076 | 0 | 75.85% | 23 | P:protein metabolic process; P:catabolic process; P:multicellular organismal development; P:cellular component organization; F:binding; F:receptor binding; P:signal transduction; P:response to endogenous stimulus; C:cytosol; C:protein complex; C:extracellular space; F:protein binding; C:cell; P:regulation of biological process; F:peptidase activity; P:metabolic process; F:hydrolase activity; P:nucleobase-containing compound metabolic process; P:cellular homeostasis; C:nucleus; C:peroxisome; F:nucleotide binding; C:mitochondrion | IPR001431 (PROSITE); IPR007863 (PFAM); IPR011237 (G3DSA:3.30.830.GENE3D); IPR011249 (SUPERFAMILY); IPR011765 (PFAM); PTHR11851 (PANTHER), PTHR11851:SF64 (PANTHER), SignalP-NN(euk) (SIGNALP), tmhmm (TMHMM) | |
| 1809 | lib_dib_Filter15_IDBA_contig_30783_plus2 | 33 | insulin-degrading enzyme | 172 | 2.67E-58 | 77.35% | 19 | P:protein metabolic process; P:catabolic process; P:multicellular organismal development; F:receptor binding; F:binding; P:signal transduction; P:response to endogenous stimulus; C:extracellular space; F:protein binding; C:peroxisome; C:cytosol; F:peptidase activity; P:metabolic process; P:cellular component organization; P:cellular homeostasis; P:regulation of biological process; C:nucleus; F:nucleotide binding; C:mitochondrion | IPR011237 (G3DSA:3.30.830.GENE3D); IPR011249 (SUPERFAMILY); PTHR11851 (PANTHER), PTHR11851:SF64 (PANTHER) | |
| 1810 | lib_dib_Filter15_IDBA_contig_30800_minus1 | 49 | katanin p80 wd40-containing subunit b1 | 329 | 8.76E-102 | 66.15% | 10 | P:regulation of biological process; P:cytoskeleton organization; F:hydrolase activity; C:microtubule organizing center; C:protein complex; F:protein binding; C:plasma membrane; C:cytoskeleton; F:cytoskeletal protein binding; P:biological_process | IPR001680 (PFAM); IPR015943 (G3DSA:2.130.10.GENE3D); IPR017986 (PROFILE); IPR019775 (PROSITE); IPR026962 (PANTHER) | |

| | | | | | | | | | |
|---|---|---|---|---|---|---|---|---|---|
| 1811 | lib_dib_Filter15_IDBA_contig_3081_minus2 | 66 | angiopoietin-like 4 | 195 | 2.91E-23 | 61.70% | 9 | P:response to stress; P:regulation of biological process; P:lipid metabolic process; P:cellular component organization; P:biological_process; C:extracellular space; F:enzyme regulator activity; P:cell communication; P:cell death | IPR002181 (PFAM); IPR014716 (G3DSA:3.90.215.GENE3D); PTHR19143 (PANTHER), tmhmm (TMHMM) |
| 1812 | lib_dib_Filter15_IDBA_contig_30816_plus3 | 1013 | leukocyte elastase inhibitor | 455 | 1.60E-112 | 61.90% | 4 | P:biological_process; P:protein metabolic process; P:catabolic process; F:enzyme regulator activity | IPR000215 (PANTHER); IPR023795 (PROSITE); IPR023796 (PFAM); G3DSA:2.30.39.10 (GENE3D), G3DSA:3.30.497.10 (GENE3D) |
| 1813 | lib_dib_Filter15_IDBA_contig_30819_minus2 | 47 | zinc finger protein | 328 | 1.85E-92 | 51.85% | 3 | F:binding; F:nucleic acid binding; C:intracellular | IPR007087 (PFAM); IPR013087 (G3DSA:3.30.160.GENE3D); IPR015880 (SMART); PTHR24375 (PANTHER), PTHR24375:SF0 (PANTHER), PF13465 (PFAM), PF13894 (PFAM), PF13912 (PFAM), SSF57667 (SUPERFAMILY) |
| 1814 | lib_dib_Filter15_IDBA_contig_30828_plus2 | 442 | lysyl-trna synthetase | 648 | 0 | 82.50% | 15 | C:cytosol; C:cytoskeleton; C:cell; F:catalytic activity; F:binding; P:nucleobase-containing compound metabolic process; F:nucleotide binding; C:mitochondrion; P:biological_process; P:translation; ; F:RNA binding; C:extracellular region; C:nucleus; C:plasma membrane | IPR002313 (PTHR22594:PANTHER); IPR004364 (PFAM); IPR004365 (PFAM); IPR006195 (PROFILE); IPR012340 (G3DSA:2.40.50.GENE3D); IPR018149 (PRINTS); IPR018150 (PANTHER); G3DSA:3.30.930.10 (GENE3D), SSF55681 (SUPERFAMILY) |
| 1815 | lib_dib_Filter15_IDBA_contig_30909_plus3 | 78 | inter-alpha-trypsin inhibitor heavy chain h2 | 327 | 1.24E-32 | 55.75% | 3 | C:extracellular region; P:metabolic process; F:enzyme regulator activity | PTHR10338 (PANTHER), PTHR10338:SF24 (PANTHER), tmhmm (TMHMM) |
| 1816 | lib_dib_Filter15_IDBA_contig_30980_minus1 | 121 | transmembrane protease serine 9-like | 467 | 1.37E-66 | 58.35% | 3 | F:peptidase activity; P:protein metabolic process; P:catabolic process | IPR001254 (PFAM); IPR001314 (PRINTS); IPR009003 (SUPERFAMILY); IPR018114 (PROSITE); G3DSA:2.40.10.10 (GENE3D), PTHR24259 (PANTHER) |
| 1817 | lib_dib_Filter15_IDBA_contig_30987_minus1 | 24 | slit like protein 2 partial | 193 | 6.91E-72 | 53.35% | 9 | P:behavior; P:response to external stimulus; P:regulation of biological process; P:anatomical structure morphogenesis; P:cellular component organization; P:cell differentiation; P:multicellular organismal development; P:biological_process; F:binding | IPR000372 (PFAM); IPR000483 (SMART); IPR025875 (PFAM); G3DSA:3.80.10.10 (GENE3D), PTHR26342 (PANTHER), PTHR26342:SF82 (PANTHER), SSF52058 (SUPERFAMILY) |
| 1818 | lib_dib_Filter15_IDBA_contig_30988_minus3 | 44 | slit protein | 185 | 1.69E-71 | 72.30% | 16 | P:anatomical structure morphogenesis; P:cellular component organization; P:cell differentiation; P:multicellular organismal development; P:cell recognition; F:receptor binding; P:cell-cell signaling; F:transferase activity; P:reproduction; P:embryo development; F:carbohydrate binding; C:proteinaceous extracellular matrix; P:regulation of biological process; P:behavior; P:response to external stimulus; F:calcium ion binding | IPR000152 (PROSITE); IPR000742 (PFAM); IPR001881 (SMART); IPR008985 (SUPERFAMILY); IPR013032 (PFAM); IPR013320 (G3DSA:2.60.120.GENE3D); IPR018097 (PROSITE); PR00010 (PRINTS), G3DSA:2.10.25.10 (GENE3D), PTHR24044 (PANTHER), SSF57196 (SUPERFAMILY) |
| 1819 | lib_dib_Filter15_IDBA_contig_30989_minus1 | 69 | protein slit | 327 | 2.63E-149 | 72.85% | 15 | P:anatomical structure morphogenesis; P:cellular component organization; P:cell differentiation; P:multicellular organismal development; P:cell recognition; F:receptor binding; P:cell-cell signaling; P:reproduction; P:embryo development; F:carbohydrate binding; C:proteinaceous extracellular matrix; P:regulation of biological process; P:behavior; P:response to external stimulus; F:calcium ion binding | IPR000152 (PROSITE); IPR000483 (SMART); IPR000742 (PFAM); IPR001611 (PFAM); IPR001881 (SMART); IPR003591 (SMART); IPR013032 (PROSITE); IPR018097 (PROSITE); PR00010 (PRINTS), G3DSA:2.10.25.10 (GENE3D), G3DSA:3.80.10.10 (GENE3D), PTHR24373 (PANTHER), PTHR24373:SF51 (PANTHER), PF13855 (PFAM), SSF52058 (SUPERFAMILY), SSF57196 (SUPERFAMILY) |

| | | | | | | | | | | |
|---|---|---|---|---|---|---|---|---|---|---|
| 1820 | lib_dib_Filter15_IDBA_contig_31003_minus2 | 168 | tyrosine-protein kinase fps85d-like isoform 2 | 595 | 4.40E-98 | 59.00% | 14 | P:anatomical structure morphogenesis; P:cellular component organization; P:cell differentiation; P:multicellular organismal development; P:cellular protein modification process; C:plasma membrane; P:cytoskeleton organization; P:embryo development; F:protein kinase activity; P:regulation of biological process; P:cell growth; C:cytoplasm; F:nucleotide binding; F:protein binding | IPR000719 (PROFILE); IPR000980 (G3DSA:3.30.505.GENE3D); IPR001245 (PFAM); IPR011009 (SUPERFAMILY); IPR017441 (PROSITE); G3DSA:3.30.200.20 (GENE3D), PTHR24418 (PANTHER), PTHR24418:SF0 (PANTHER), SSF55550 (SUPERFAMILY) | |
| 1821 | lib_dib_Filter15_IDBA_contig_31027_minus2 | 74 | toll-like receptor | 326 | 4.19E-45 | 66.20% | 4 | F:receptor activity; C:cell; F:protein binding; P:signal transduction | IPR000157 (PFAM); PR01537 (PRINTS), G3DSA:3.40.50.10140 (GENE3D), PTHR24365 (PANTHER), SignalP-NN(euk) (SIGNALP), tmhmm (TMHMM) | |
| 1822 | lib_dib_Filter15_IDBA_contig_31028_plus3 | 108 | ectonucleoside triphosphate diphosphohydrolase 2 | 356 | 1.43E-53 | 62.10% | 8 | P:response to external stimulus; P:response to stress; F:hydrolase activity; P:nucleobase-containing compound metabolic process; P:catabolic process; C:proteinaceous extracellular matrix; F:protein binding; P:signal transduction | IPR000407 (PANTHER); PTHR11782:SF4 (PANTHER), tmhmm (TMHMM) | |
| 1823 | lib_dib_Filter15_IDBA_contig_31039_plus3 | 221 | matrix metalloproteinase 2 | 326 | 7.30E-13 | 49.05% | 20 | F:metalloendopeptidase activity; F:hydrolase activity; F:zinc ion binding; C:collagen; F:metallopeptidase activity; C:extracellular matrix; F:peptidase activity; P:proteolysis; F:metal ion binding; P:metabolic process; F:carbohydrate binding; P:positive regulation of apoptotic process; P:skeletal system development; P:collagen catabolic process; P:positive regulation of keratinocyte migration; C:extracellular space; P:extracellular matrix organization; C:proteinaceous extracellular matrix; P:embryo implantation; C:extracellular region | IPR000562 (G3DSA:2.10.10.GENE3D); IPR013806 (SUPERFAMILY); PTHR22803 (PANTHER) | |
| 1824 | lib_dib_Filter15_IDBA_contig_31050_minus3 | 54 | carboxypeptidase | 325 | 6.66E-45 | 56.80% | 4 | F:peptidase activity; P:protein metabolic process; P:catabolic process; F:binding | IPR000834 (PRINTS); IPR003146 (G3DSA:3.30.70.GENE3D); IPR009020 (SUPERFAMILY); G3DSA:3.40.630.10 (GENE3D), PTHR11705 (PANTHER), SSF53187 (SUPERFAMILY) | |
| 1825 | lib_dib_Filter15_IDBA_contig_31069_plus1 | 47 | signal cub and egf-like domain-containing protein 2-like | 326 | 6.51E-122 | 65.55% | 9 | F:protein binding; P:biological_process; C:extracellular space; P:response to external stimulus; P:response to stress; P:multicellular organismal development; P:cellular component organization; C:plasma membrane; F:calcium ion binding | IPR000152 (PROSITE); IPR000742 (SMART); IPR001881 (PFAM); IPR013032 (PROSITE); IPR018097 (PROSITE); IPR023413 (G3DSA:2.40.155.GENE3D); IPR024731 (PFAM); G3DSA:2.10.25.10 (GENE3D), PTHR24046 (PANTHER), PTHR24046:SF0 (PANTHER), SSF57196 (SUPERFAMILY) | |
| 1826 | lib_dib_Filter15_IDBA_contig_31094_minus1 | 824 | ankyrin repeat domain-containing protein 17 | 705 | 2.98E-87 | 53.00% | 1 | F:protein binding | IPR001496 (PFAM); IPR002110 (PRINTS); IPR020683 (G3DSA:1.25.40.GENE3D); PTHR24133 (PANTHER), PTHR24133:SF5 (PANTHER), PF13857 (PFAM) | |
| 1827 | lib_dib_Filter15_IDBA_contig_31095_minus1 | 837 | ankyrin repeat domain-containing protein 17 | 666 | 1.63E-87 | 53.10% | 1 | F:protein binding | IPR001496 (PFAM); IPR002110 (PRINTS); IPR020683 (G3DSA:1.25.40.GENE3D); PTHR24133 (PANTHER), PTHR24133:SF5 (PANTHER), PF13857 (PFAM) | |
| 1828 | lib_dib_Filter15_IDBA_contig_31155_plus2 | 10055 | glutathione peroxidase 3 precursor | 402 | 9.53E-53 | 69.25% | 5 | C:extracellular region; F:catalytic activity; F:antioxidant activity; P:response to stress; P:metabolic process | IPR000889 (PRINTS); IPR012336 (G3DSA:3.40.30.GENE3D); PTHR11592:SF0 (PANTHER), SignalP-NN(euk) (SIGNALP) | Yes |

| | | | | | | | | | |
|---|---|---|---|---|---|---|---|---|---|
| 1829 | lib_dib_Filter15_IDBA_contig_31159_minus2 | 50 | polypeptide n-acetylgalactosaminyltransferase 1 | 325 | 0 | 85.05% | 10 | P:biosynthetic process; P:cellular protein modification process; P:carbohydrate metabolic process; C:Golgi apparatus; C:cell; F:binding; F:transferase activity; C:cytoplasm; C:extracellular region; F:carbohydrate binding | IPR001173 (PFAM); G3DSA:3.90.550.10 (GENE3D), PTHR11675 (PANTHER), SSF53448 (SUPERFAMILY) |
| 1830 | lib_dib_Filter15_IDBA_contig_31166_plus2 | 59 | bone morphogenetic protein receptor type-2 | 325 | 5.00E-125 | 72.25% | 21 | P:multicellular organismal development; C:cell; P:cell differentiation; P:regulation of biological process; C:plasma membrane; P:signal transduction; F:receptor activity; F:protein kinase activity; P:cellular protein modification process; P:anatomical structure morphogenesis; P:embryo development; C:cytoplasm; P:biological_process; P:response to external stimulus; P:cell communication; P:response to stress; F:protein binding; ; P:cell growth; P:cell proliferation; F:nucleotide binding | IPR000719 (PFAM); IPR002290 (SMART); IPR011009 (SUPERFAMILY); IPR015770 (PTHR23255:PANTHER); IPR020635 (SMART); G3DSA:1.10.510.10 (GENE3D), G3DSA:3.30.200.20 (GENE3D), PTHR23255 (PANTHER), SignalP-NN(euk) (SIGNALP), tmhmm (TMHMM) |
| 1831 | lib_dib_Filter15_IDBA_contig_31168_minus2 | 180 | carbonyl reductase | 463 | 1.47E-111 | 70.05% | 3 | P:metabolic process; F:catalytic activity; F:nucleotide binding | IPR002198 (PRINTS); IPR002347 (PRINTS); IPR016040 (G3DSA:3.40.50.GENE3D); IPR020904 (PROSITE); PTHR24322 (PANTHER), PTHR24322:SF58 (PANTHER), SSF51735 (SUPERFAMILY) |
| 1832 | lib_dib_Filter15_IDBA_contig_31174_plus1 | 59 | tyrosine-protein kinase fer | 325 | 3.01E-69 | 63.75% | 28 | P:regulation of biological process; P:signal transduction; C:nuclear chromosome; P:cytoskeleton organization; P:response to stress; P:cellular protein modification process; F:receptor binding; P:cell proliferation; F:lipid binding; C:cell; C:cytosol; C:cytoskeleton; C:plasma membrane; P:anatomical structure morphogenesis; P:cellular component organization; P:cell differentiation; P:behavior; P:response to external stimulus; C:intracellular; F:protein binding; F:enzyme regulator activity; ; P:cell cycle; P:biological_process; F:protein kinase activity; P:response to biotic stimulus; F:actin binding; F:nucleotide binding | IPR000719 (PROFILE); IPR001245 (PRINTS); IPR002290 (SMART); IPR011009 (SUPERFAMILY); IPR020635 (SMART); G3DSA:1.10.510.10 (GENE3D), G3DSA:3.30.200.20 (GENE3D), PTHR24418 (PANTHER) |
| 1833 | lib_dib_Filter15_IDBA_contig_31183_plus3 | 109 | fibrinogen-related protein | 612 | 3.21E-107 | 61.90% | 4 | F:receptor binding; C:extracellular space; P:signal transduction; F:carbohydrate binding | IPR002181 (PFAM); IPR014715 (G3DSA:4.10.530.GENE3D); IPR014716 (G3DSA:3.90.215.GENE3D); IPR020837 (PROSITE); PTHR19143 (PANTHER), tmhmm (TMHMM) |
| 1834 | lib_dib_Filter15_IDBA_contig_31241_plus3 | 272 | ubiquitin thioesterase partial | 324 | 3.67E-17 | 50.25% | 0 | - | PTHR23304 (PANTHER), PTHR23304:SF83 (PANTHER) |
| 1835 | lib_dib_Filter15_IDBA_contig_31259_minus1 | 71 | adam 19 | 324 | 9.67E-122 | 66.80% | 3 | F:peptidase activity; P:protein metabolic process; P:catabolic process | IPR001762 (G3DSA:4.10.70.GENE3D); IPR006586 (PFAM); IPR013032 (PROSITE); IPR024079 (G3DSA:3.40.390.GENE3D); PTHR11905 (PANTHER), PTHR11905:SF15 (PANTHER), SSF55486 (SUPERFAMILY) |
| 1836 | lib_dib_Filter15_IDBA_contig_31264_plus1 | 60 | retinoid-inducible serine carboxypeptidase | 324 | 1.30E-42 | 50.45% | 3 | F:peptidase activity; P:protein metabolic process; P:catabolic process | IPR001563 (PANTHER); G3DSA:3.40.50.1820 (GENE3D), PTHR11802:SF3 (PANTHER), SSF53474 (SUPERFAMILY) |
| 1837 | lib_dib_Filter15_IDBA_contig_31272_minus1 | 60 | galectin-4 | 324 | 9.73E-35 | 48.15% | 1 | F:carbohydrate binding | IPR001079 (PFAM); IPR008985 (SUPERFAMILY); IPR013320 (G3DSA:2.60.120.GENE3D); PTHR11346 (PANTHER) |

| | | | | | | | | | | |
|---|---|---|---|---|---|---|---|---|---|---|
| 1838 | lib_dib_Filter15_IDBA_contig_31295_minus1 | 103 | tyrosine-protein kinase fps85d-like isoform 1 | 488 | 7.02E-143 | 68.85% | 4 | F:nucleotide binding; P:cellular protein modification process; F:protein binding; F:protein kinase activity | IPR000719 (PROFILE); IPR000980 (G3DSA:3.30.505.GENE3D); IPR001245 (PRINTS); IPR002290 (SMART); IPR008266 (PROSITE); IPR011009 (SUPERFAMILY); IPR017441 (PROSITE); IPR020635 (SMART); G3DSA:1.10.510.10 (GENE3D), G3DSA:3.30.200.20 (GENE3D), PTHR24418 (PANTHER), PTHR24418:SF0 (PANTHER), SSF55550 (SUPERFAMILY) | |
| 1839 | lib_dib_Filter15_IDBA_contig_3132_minus1 | 285 | cartilage intermediate layer protein 2 | 349 | 3.46E-11 | 54.90% | 1 | F:hydrolase activity | IPR025155 (PFAM); PTHR15031 (PANTHER), SignalP-NN(euk) (SIGNALP), tmhmm (TMHMM) | |
| 1840 | lib_dib_Filter15_IDBA_contig_31349_minus3 | 60 | serine protease 27-like | 323 | 7.05E-47 | 54.45% | 4 | C:cell; F:peptidase activity; P:protein metabolic process; P:catabolic process | IPR001254 (PFAM); IPR001314 (PRINTS); IPR009003 (SUPERFAMILY); IPR018114 (PROSITE); G3DSA:2.40.10.10 (GENE3D), PTHR24259 (PANTHER), SignalP-NN(euk) (SIGNALP) | Yes |
| 1841 | lib_dib_Filter15_IDBA_contig_31350_plus2 | 518 | poly -specific endoribonuclease-b-like | 296 | 1.13E-117 | 67.55% | 4 | F:nuclease activity; F:binding; P:nucleobase-containing compound metabolic process; C:nucleus | IPR018998 (PFAM); PTHR12439 (PANTHER), SignalP-NN(euk) (SIGNALP), SSF142877 (SUPERFAMILY) | Yes |
| 1842 | lib_dib_Filter15_IDBA_contig_31353_plus2 | 129 | protein kinase related to protein kinase isoform e | 559 | 0 | 87.90% | 10 | P:cellular protein modification process; P:multicellular organismal development; P:biological_process; P:anatomical structure morphogenesis; P:embryo development; C:intracellular; F:nucleotide binding; F:protein kinase activity; P:signal transduction; F:signal transducer activity | IPR000719 (PFAM); IPR000961 (SMART); IPR002290 (SMART); IPR008271 (PROSITE); IPR011009 (SUPERFAMILY); IPR017441 (PROSITE); IPR017892 (PFAM); IPR020635 (SMART); G3DSA:1.10.510.10 (GENE3D), G3DSA:3.30.200.20 (GENE3D), PTHR24357 (PANTHER), PTHR24357:SF1 (PANTHER) | |
| 1843 | lib_dib_Filter15_IDBA_contig_31354_plus1 | 50 | na(+) h(+) exchange regulatory cofactor nhe-rf1 | 323 | 7.36E-45 | 54.40% | 19 | P:regulation of biological process; P:signal transduction; P:cellular homeostasis; P:biological_process; F:protein binding; C:plasma membrane; P:metabolic process; P:ion transport; P:transport; F:receptor binding; P:cell death; P:cell proliferation; P:cytoskeleton organization; C:microtubule organizing center; P:cellular component organization; F:molecular_function; P:nucleobase-containing compound metabolic process; P:biosynthetic process; C:nucleus | IPR001478 (PFAM); G3DSA:2.30.42.10 (GENE3D), PTHR14191 (PANTHER), PTHR14191:SF0 (PANTHER) | |
| 1844 | lib_dib_Filter15_IDBA_contig_31365_plus1 | 71 | cubilin precursor | 323 | 8.38E-117 | 62.75% | 15 | P:protein transport; P:cellular component organization; C:protein complex; P:response to external stimulus; C:plasma membrane; C:lysosome; F:receptor activity; F:protein binding; C:endosome; C:cell; C:cytoplasmic membrane-bounded vesicle; P:transport; P:embryo development; C:Golgi apparatus; C:endoplasmic reticulum | IPR000859 (G3DSA:2.60.120.GENE3D); PTHR10127 (PANTHER), PTHR10127:SF310 (PANTHER) | |
| 1845 | lib_dib_Filter15_IDBA_contig_31382_plus1 | 337 | plexin isoform f | 651 | 6.09E-87 | 53.30% | 8 | P:anatomical structure morphogenesis; P:cellular component organization; P:cell differentiation; P:multicellular organismal development; F:receptor activity; P:signal transduction; F:protein binding; C:cell | IPR001627 (PFAM); IPR002165 (PFAM); IPR003659 (SMART); IPR015943 (G3DSA:2.130.10.GENE3D); IPR016201 (SUPERFAMILY); G3DSA:3.30.1680.10 (GENE3D), PTHR22625 (PANTHER), SignalP-NN(euk) (SIGNALP), tmhmm (TMHMM) | |

| | | | | | | | | |
|---|---|---|---|---|---|---|---|---|
| 1846 | lib_dib_Filter15_IDBA_contig_31383_plus1 | 133 | plexin a | 238 | 4.87E-32 | 52.65% | 9 | P:anatomical structure morphogenesis; P:cellular component organization; P:cell differentiation; P:multicellular organismal development; P:signal transduction; P:regulation of biological process; F:receptor activity; F:protein binding; C:cell | IPR001627 (PFAM); IPR002165 (PFAM); IPR003659 (SMART); IPR015943 (G3DSA:2.130.10.GENE3D); IPR016201 (SUPERFAMILY); G3DSA:3.30.1680.10 (GENE3D), PTHR22625 (PANTHER) |
| 1847 | lib_dib_Filter15_IDBA_contig_31420_minus2 | 93 | beta- -galactosyltransferase 6-like | 322 | 3.07E-13 | 62.35% | 2 | F:transferase activity; P:carbohydrate metabolic process | IPR003859 (PANTHER); G3DSA:3.90.550.10 (GENE3D), PF13733 (PFAM), SignalP-NN(euk) (SIGNALP), tmhmm (TMHMM), SSF53448 (SUPERFAMILY) |
| 1848 | lib_dib_Filter15_IDBA_contig_31426_minus2 | 689 | collagen alpha-6 chain | 549 | 6.00E-46 | 58.40% | 1 | F:protein binding | IPR002035 (PFAM); PR00453 (PRINTS), G3DSA:3.40.50.410 (GENE3D), PTHR22992 (PANTHER), tmhmm (TMHMM), SSF53300 (SUPERFAMILY) |
| 1849 | lib_dib_Filter15_IDBA_contig_31427_minus2 | 1614 | collagen alpha-6 chain | 856 | 9.20E-45 | 58.55% | 1 | F:protein binding | IPR002035 (PFAM); PR00453 (PRINTS), G3DSA:3.40.50.410 (GENE3D), PTHR22992 (PANTHER), tmhmm (TMHMM), SSF53300 (SUPERFAMILY) |
| 1850 | lib_dib_Filter15_IDBA_contig_31428_minus2 | 1589 | collagen alpha-6 chain | 846 | 8.46E-45 | 58.55% | 1 | F:protein binding | IPR002035 (PFAM); PR00453 (PRINTS), G3DSA:3.40.50.410 (GENE3D), PTHR22992 (PANTHER), tmhmm (TMHMM), SSF53300 (SUPERFAMILY) |
| 1851 | lib_dib_Filter15_IDBA_contig_31431_minus1 | 90 | alpha- -mannosylglycoprotein 6-beta-n-acetylglucosaminyltransferase a | 322 | 5.66E-62 | 57.15% | 4 | C:cell; P:biosynthetic process; P:cellular protein modification process; P:carbohydrate metabolic process | IPR026116 (PANTHER); PTHR15075:SF0 (PANTHER), SignalP-NN(euk) (SIGNALP), tmhmm (TMHMM) |
| 1852 | lib_dib_Filter15_IDBA_contig_31433_minus1 | 68 | isoform f | 307 | 8.00E-11 | 40.20% | 6 | P:anatomical structure morphogenesis; P:biological_process; P:multicellular organismal development; P:regulation of biological process; P:cell differentiation; F:protein binding | IPR001611 (PFAM); G3DSA:3.80.10.10 (GENE3D), PTHR24365 (PANTHER), SSF52047 (SUPERFAMILY), SSF52058 (SUPERFAMILY) |
| 1853 | lib_dib_Filter15_IDBA_contig_31435_minus1 | 82 | variable lymphocyte receptor c | 402 | 8.05E-17 | 45.35% | 1 | F:protein binding | IPR001611 (PROFILE); IPR003591 (SMART); G3DSA:3.80.10.10 (GENE3D), PTHR24365 (PANTHER), PF13504 (PFAM), PF13855 (PFAM), SSF52058 (SUPERFAMILY) |
| 1854 | lib_dib_Filter15_IDBA_contig_31436_minus2 | 36 | slit protein | 322 | 1.88E-12 | 46.85% | 1 | F:protein binding | IPR001611 (PFAM); G3DSA:3.80.10.10 (GENE3D), PTHR24365 (PANTHER), SSF52058 (SUPERFAMILY) |
| 1855 | lib_dib_Filter15_IDBA_contig_31452_minus2 | 48 | ankyrin repeat protein | 322 | 3.52E-29 | 52.50% | 1 | F:protein binding | IPR002110 (SMART); IPR020683 (G3DSA:1.25.40.GENE3D); PTHR24198 (PANTHER), SignalP-NN(euk) (SIGNALP) |
| 1856 | lib_dib_Filter15_IDBA_contig_31480_plus1 | 93 | peptidyl-prolyl cis-trans isomerase fkbp4-like | 433 | 3.25E-147 | 72.40% | 21 | P:biological_process; F:binding; P:cellular protein modification process; C:cell; F:protein binding; F:nucleotide binding; P:signal transduction; P:reproduction; P:multicellular organismal development; F:cytoskeletal protein binding; C:cytosol; P:cellular component organization; C:protein complex; P:regulation of biological process; P:cytoskeleton organization; F:catalytic activity; C:cytoplasm; P:ion transport; F:receptor binding; C:nucleus; P:protein metabolic process | IPR001179 (PFAM); IPR011990 (G3DSA:1.25.40.GENE3D); IPR013026 (PROFILE); IPR019734 (PFAM); IPR023114 (G3DSA:1.10.150.GENE3D); IPR023566 (PANTHER); G3DSA:3.10.50.40 (GENE3D), PTHR10516:SF46 (PANTHER), PF13414 (PFAM), SSF48452 (SUPERFAMILY), SSF54534 (SUPERFAMILY) |

| | | | | | | | | | |
|---|---|---|---|---|---|---|---|---|---|
| 1857 | lib_dib_Filter15_IDBA_contig_31481_plus2 | 109 | protein dachsous-like | 380 | 5.87E-17 | 55.85% | 7 | P:multicellular organismal development; P:embryo development; F:calcium ion binding; P:biological_process; C:cell; P:metabolic process; F:catalytic activity | IPR002126 (G3DSA:2.60.40.GENE3D); IPR002227 (PROSITE); IPR015919 (SUPERFAMILY); PTHR24028 (PANTHER), tmhmm (TMHMM) |
| 1858 | lib_dib_Filter15_IDBA_contig_31482_plus1 | 174 | protocadherin fat 1-like | 528 | 5.61E-48 | 56.15% | 5 | F:calcium ion binding; P:biological_process; P:metabolic process; F:catalytic activity; C:plasma membrane | IPR002126 (PRINTS); IPR002227 (PROSITE); IPR015919 (SUPERFAMILY); IPR020894 (PROSITE); PTHR24027 (PANTHER), tmhmm (TMHMM) |
| 1859 | lib_dib_Filter15_IDBA_contig_31483_plus3 | 346 | protocadherin-11 x-linked | 982 | 4.58E-98 | 51.20% | 5 | F:calcium ion binding; P:biological_process; P:metabolic process; F:catalytic activity; C:plasma membrane | IPR002126 (PRINTS); IPR002227 (PROSITE); IPR015919 (SUPERFAMILY); IPR020894 (PROSITE); PTHR24026 (PANTHER), SignalP-NN(euk) (SIGNALP), tmhmm (TMHMM) |
| 1860 | lib_dib_Filter15_IDBA_contig_31484_plus2 | 377 | protocadherin-11 x-linked | 996 | 7.61E-98 | 51.15% | 5 | F:calcium ion binding; P:biological_process; P:metabolic process; F:catalytic activity; C:plasma membrane | IPR002126 (PRINTS); IPR002227 (PROSITE); IPR015919 (SUPERFAMILY); IPR020894 (PROSITE); PTHR24027 (PANTHER), tmhmm (TMHMM) |
| 1861 | lib_dib_Filter15_IDBA_contig_31499_minus2 | 1098 | isoform a | 987 | 0 | 46.45% | 1 | F:protein binding | IPR001496 (PFAM); IPR002110 (SMART); IPR020683 (G3DSA:1.25.40.GENE3D); PTHR24188 (PANTHER), SignalP-NN(euk) (SIGNALP) |
| 1862 | lib_dib_Filter15_IDBA_contig_31506_plus2 | 696 | transmembrane protease serine partial | 686 | 1.42E-49 | 54.45% | 4 | C:plasma membrane; F:peptidase activity; P:protein metabolic process; P:catabolic process | IPR001254 (PFAM); IPR001314 (PRINTS); IPR009003 (SUPERFAMILY); IPR018114 (PROSITE); G3DSA:2.40.10.10 (GENE3D), PTHR24265 (PANTHER), SignalP-NN(euk) (SIGNALP), tmhmm (TMHMM) |
| 1863 | lib_dib_Filter15_IDBA_contig_31541_minus3 | 394 | cub domain protein | 321 | 7.45E-33 | 49.35% | 1 | F:peptidase activity | IPR000859 (G3DSA:2.60.120.GENE3D); PTHR10127 (PANTHER) |
| 1864 | lib_dib_Filter15_IDBA_contig_31550_minus1 | 41 | pappalysin- partial | 322 | 1.15E-79 | 59.15% | 2 | C:extracellular region; F:peptidase activity | IPR011936 (TIGRFAMs) |
| 1865 | lib_dib_Filter15_IDBA_contig_31611_plus1 | 3127 | bactericidal permeability increasing protein | 832 | 9.89E-62 | 46.20% | 1 | F:lipid binding | IPR001124 (PFAM); IPR017942 (PFAM); IPR017943 (SUPERFAMILY); G3DSA:3.15.10.10 (GENE3D), G3DSA:3.15.20.10 (GENE3D), PTHR10504 (PANTHER) |
| 1866 | lib_dib_Filter15_IDBA_contig_31648_minus3 | 49 | deleted in malignant brain tumors 1 | 320 | 5.42E-40 | 50.80% | 2 | F:receptor activity; C:cell | IPR001190 (PFAM); IPR017448 (SMART); G3DSA:3.10.250.10 (GENE3D), PTHR19331 (PANTHER), PTHR19331:SF118 (PANTHER), SignalP-NN(euk) (SIGNALP), tmhmm (TMHMM) |
| 1867 | lib_dib_Filter15_IDBA_contig_31671_plus2 | 72 | leucine-rich repeat-containing protein 40 | 342 | 2.73E-108 | 67.60% | 1 | F:protein binding | IPR001611 (PROFILE); IPR003591 (SMART); IPR015766 (PTHR23155:PANTHER); IPR025875 (PFAM); G3DSA:3.80.10.10 (GENE3D), PTHR23155 (PANTHER), PF13855 (PFAM), SM00364 (SMART), SSF52058 (SUPERFAMILY) |
| 1868 | lib_dib_Filter15_IDBA_contig_31693_plus1 | 37 | btb poz domain-containing protein 17 | 320 | 2.57E-23 | 49.05% | 7 | F:scavenger receptor activity; P:cell adhesion; C:membrane; C:proteinaceous extracellular matrix; C:extracellular region; P:B cell receptor signaling pathway; P:germinal center formation | IPR011705 (PFAM); PTHR24412 (PANTHER), SignalP-NN(euk) (SIGNALP) |
| 1869 | lib_dib_Filter15_IDBA_contig_31699_minus3 | 126 | roundabout homolog 3 | 405 | 9.27E-103 | 67.05% | 18 | P:anatomical structure morphogenesis; P:cellular component organization; P:cell differentiation; P:multicellular organismal development; P:cell recognition; F:protein binding; P:regulation of biological process; P:cell proliferation; P:signal transduction; P:cell death; P:growth; P:behavior; P:response to external stimulus; C:cytoplasm; C:cell; P:biological_process; F:receptor activity; C:plasma membrane | IPR003596 (SMART); IPR003598 (SMART); IPR003599 (SMART); IPR007110 (PROFILE); IPR013098 (PFAM); IPR013783 (G3DSA:2.60.40.GENE3D); PTHR10489 (PANTHER), PTHR10489:SF92 (PANTHER), PF13895 (PFAM), tmhmm (TMHMM), SSF48726 (SUPERFAMILY) |

| | | | | | | | | | | |
|---|---|---|---|---|---|---|---|---|---|---|
| 1870 | lib_dib_Filter15_IDBA_contig_31764_plus3 | 392 | tolloid-like protein 1-like | 319 | 2.70E-35 | 49.20% | 6 | F:peptidase activity; F:binding; C:organelle; C:intracellular; C:cytoplasm; C:cell | IPR000859 (G3DSA:2.60.120.GENE3D); PTHR10127 (PANTHER), SignalP-NN(euk) (SIGNALP) | Yes |
| 1871 | lib_dib_Filter15_IDBA_contig_31775_minus3 | 70 | adenosine deaminase cecr1 | 319 | 9.34E-121 | 68.05% | 8 | F:hydrolase activity; F:binding; C:extracellular space; F:protein binding; P:nucleobase-containing compound metabolic process; P:catabolic process; F:receptor binding; P:biosynthetic process | IPR001365 (PFAM); IPR006331 (TIGRFAMs); G3DSA:3.20.20.140 (GENE3D), PTHR11409 (PANTHER), PTHR11409:SF5 (PANTHER), SSF51556 (SUPERFAMILY) | |
| 1872 | lib_dib_Filter15_IDBA_contig_31782_plus2 | 145 | fk506-binding protein 1a | 517 | 2.09E-48 | 79.35% | 5 | P:protein metabolic process; C:endoplasmic reticulum; F:binding; P:cellular protein modification process; F:catalytic activity | IPR001179 (PFAM); IPR023566 (PANTHER); G3DSA:3.10.50.40 (GENE3D), PTHR10516:SF116 (PANTHER), tmhmm (TMHMM), SSF54534 (SUPERFAMILY) | |
| 1873 | lib_dib_Filter15_IDBA_contig_31836_minus1 | 138 | class b secretin-like g-protein coupled receptor | 838 | 4.09E-31 | 47.95% | 5 | F:receptor activity; P:signal transduction; C:cell; P:biological_process; F:carbohydrate binding | IPR000832 (PFAM); IPR001212 (PFAM); IPR017981 (PROFILE); PTHR12011 (PANTHER), tmhmm (TMHMM), SSF90188 (SUPERFAMILY) | |
| 1874 | lib_dib_Filter15_IDBA_contig_31853_minus3 | 75 | zinc finger protein 568 | 458 | 1.90E-77 | 67.65% | 3 | F:binding; F:nucleic acid binding; C:intracellular | IPR007087 (PROSITE); IPR013087 (G3DSA:3.30.160.GENE3D); IPR015880 (SMART); PTHR24402 (PANTHER), PF13465 (PFAM), SSF57667 (SUPERFAMILY) | |
| 1875 | lib_dib_Filter15_IDBA_contig_31858_plus1 | 713 | ankyrin repeat protein | 949 | 1.53E-107 | 50.60% | 1 | F:protein binding | IPR002110 (PRINTS); IPR020683 (G3DSA:1.25.40.GENE3D); PTHR24123 (PANTHER) | |
| 1876 | lib_dib_Filter15_IDBA_contig_31860_minus3 | 1878 | fcn3-a protein | 345 | 5.55E-32 | 53.30% | 3 | F:receptor binding; C:extracellular space; P:signal transduction | IPR002181 (PFAM); IPR014715 (G3DSA:4.10.530.GENE3D); IPR014716 (G3DSA:3.90.215.GENE3D); PTHR19143 (PANTHER), SignalP-NN(euk) (SIGNALP) | Yes |
| 1877 | lib_dib_Filter15_IDBA_contig_31861_minus3 | 2075 | fcn3-a protein | 447 | 8.12E-32 | 53.10% | 3 | F:receptor binding; C:extracellular space; P:signal transduction | IPR002181 (PFAM); IPR014715 (G3DSA:4.10.530.GENE3D); IPR014716 (G3DSA:3.90.215.GENE3D); PTHR19143 (PANTHER), SignalP-NN(euk) (SIGNALP) | Yes |
| 1878 | lib_dib_Filter15_IDBA_contig_31862_minus3 | 2828 | fcn3-a protein | 620 | 4.25E-31 | 53.35% | 3 | F:receptor binding; C:extracellular space; P:signal transduction | IPR002181 (PFAM); IPR014715 (G3DSA:4.10.530.GENE3D); IPR014716 (G3DSA:3.90.215.GENE3D); PTHR19143 (PANTHER), SignalP-NN(euk) (SIGNALP), tmhmm (TMHMM) | |
| 1879 | lib_dib_Filter15_IDBA_contig_31939_minus2 | 58 | dipeptidyl peptidase 2 | 318 | 7.69E-122 | 64.95% | 3 | P:protein metabolic process; P:catabolic process; F:peptidase activity | IPR008758 (PANTHER); G3DSA:3.40.50.1820 (GENE3D), PTHR11010:SF10 (PANTHER), SSF53474 (SUPERFAMILY) | |
| 1880 | lib_dib_Filter15_IDBA_contig_31977_minus3 | 51 | xdh protein | 316 | 2.19E-97 | 68.60% | 3 | F:catalytic activity; F:binding; P:metabolic process | IPR008274 (G3DSA:3.30.365.GENE3D); PTHR11908 (PANTHER), PTHR11908:SF5 (PANTHER) | |
| 1881 | lib_dib_Filter15_IDBA_contig_32014_plus3 | 51488 | peroxiredoxin 6 | 493 | 3.34E-107 | 79.95% | 8 | C:cytoplasm; F:hydrolase activity; P:response to stress; P:catabolic process; P:lipid metabolic process; F:antioxidant activity; F:catalytic activity; P:metabolic process | IPR000866 (PFAM); IPR012336 (G3DSA:3.40.30.GENE3D); IPR019479 (PFAM); G3DSA:3.30.1020.10 (GENE3D), PTHR10681 (PANTHER) | |

| | | | | | | | | | | |
|---|---|---|---|---|---|---|---|---|---|---|
| 1882 | lib_dib_Filter15_IDBA_contig_32024_plus1 | 101 | ribosomal protein s6 kinase beta-1-like | 555 | 0 | 83.35% | 33 | C:cell; P:response to abiotic stimulus; P:regulation of biological process; P:translation; P:response to endogenous stimulus; P:biological_process; P:response to external stimulus; P:cell cycle; F:hydrolase activity; P:signal transduction; C:cytosol; F:protein kinase activity; P:lipid metabolic process; C:plasma membrane; P:response to stress; C:cytoplasm; F:binding; P:transport; F:protein binding; F:nucleotide binding; P:multicellular organismal development; P:growth; P:reproduction; P:cell differentiation; C:cellular_component; C:ribosome; C:mitochondrion; P:cell death; P:cell proliferation; C:nucleus; P:cellular protein modification process; P:behavior; P:response | IPR000719 (PFAM); IPR000961 (SMART); IPR002290 (SMART); IPR008271 (PROSITE); IPR011009 (SUPERFAMILY); IPR016238 (PIR); IPR017441 (PROSITE); IPR017892 (PFAM); IPR020635 (SMART); G3DSA:1.10.510.10 (GENE3D), G3DSA:3.30.200.20 (GENE3D), PTHR24351 (PANTHER), PTHR24351:SF0 (PANTHER) | |
| 1883 | lib_dib_Filter15_IDBA_contig_32047_minus3 | 131 | ferritin | 316 | 2.10E-94 | 86.75% | 6 | C:cytoplasm; P:cellular homeostasis; F:binding; F:catalytic activity; P:ion transport; P:metabolic process | IPR001519 (PANTHER); IPR008331 (PFAM); IPR009040 (PROFILE); IPR009078 (SUPERFAMILY); IPR012347 (G3DSA:1.20.1260.GENE3D); IPR014034 (PROSITE) | |
| 1884 | lib_dib_Filter15_IDBA_contig_32050_plus1 | 525 | deleted in malignant brain tumors 1 | 391 | 3.73E-75 | 55.30% | 2 | F:receptor activity; C:cell | IPR001190 (PRINTS); IPR017448 (SMART); G3DSA:3.10.250.10 (GENE3D), PTHR19331 (PANTHER), PTHR19331:SF118 (PANTHER), SignalP-NN(euk) (SIGNALP), tmhmm (TMHMM) | Yes |
| 1885 | lib_dib_Filter15_IDBA_contig_32051_plus1 | 611 | deleted in malignant brain tumors 1 | 277 | 1.09E-54 | 59.35% | 2 | F:receptor activity; C:cell | IPR001190 (PRINTS); IPR017448 (SMART); G3DSA:3.10.250.10 (GENE3D), PTHR19331 (PANTHER), PTHR19331:SF118 (PANTHER), SignalP-NN(euk) (SIGNALP), tmhmm (TMHMM) | |
| 1886 | lib_dib_Filter15_IDBA_contig_32052_plus3 | 79 | lysyl oxidase homolog 2a-like | 130 | 3.21E-24 | 62.35% | 2 | F:receptor activity; C:cell | IPR001190 (PRINTS); IPR017448 (SMART); G3DSA:3.10.250.10 (GENE3D), PTHR19331 (PANTHER), PTHR19331:SF118 (PANTHER) | |
| 1887 | lib_dib_Filter15_IDBA_contig_32053_plus1 | 57 | lysyl oxidase homolog 4 | 104 | 1.50E-10 | 71.25% | 3 | F:binding; F:receptor activity; C:cell | IPR001190 (PFAM); IPR017448 (SUPERFAMILY); G3DSA:3.10.250.10 (GENE3D), PTHR19331 (PANTHER), PTHR19331:SF118 (PANTHER) | |
| 1888 | lib_dib_Filter15_IDBA_contig_32074_plus2 | 1338 | macrophage mannose receptor 1 | 779 | 4.04E-38 | 48.90% | 1 | F:carbohydrate binding | IPR001304 (PFAM); IPR016186 (G3DSA:3.10.100.GENE3D); IPR016187 (SUPERFAMILY); PTHR22802 (PANTHER), SignalP-NN(euk) (SIGNALP), tmhmm (TMHMM) | Yes |
| 1889 | lib_dib_Filter15_IDBA_contig_32150_plus2 | 13223 | pancreatic lipase-related protein 2 | 401 | 3.41E-57 | 50.55% | 2 | F:catalytic activity; P:lipid metabolic process | IPR000734 (PRINTS); IPR013818 (PFAM); G3DSA:3.40.50.1820 (GENE3D), SignalP-NN(euk) (SIGNALP), tmhmm (TMHMM), SSF53474 (SUPERFAMILY) | Yes |
| 1890 | lib_dib_Filter15_IDBA_contig_32161_minus2 | 27 | beta-glucuronidase | 162 | 1.70E-70 | 73.55% | 3 | F:hydrolase activity; C:cell; P:carbohydrate metabolic process | IPR006101 (PRINTS); IPR006102 (SUPERFAMILY); IPR006103 (PFAM); IPR013781 (G3DSA:3.20.20.GENE3D); IPR013812 (G3DSA:2.60.40.GENE3D); IPR017853 (SUPERFAMILY); PTHR10066 (PANTHER), PTHR10066:SF40 (PANTHER) | |
| 1891 | lib_dib_Filter15_IDBA_contig_32164_minus2 | 478 | chloride channel calcium activated 2-like | 659 | 9.02E-130 | 53.35% | 1 | F:protein binding | IPR002035 (SMART); IPR013642 (PFAM); IPR015394 (PFAM); G3DSA:3.40.50.410 (GENE3D), PTHR10579 (PANTHER), PF13519 (PFAM), tmhmm (TMHMM), SSF53300 (SUPERFAMILY) | |

| | | | | | | | | | |
|---|---|---|---|---|---|---|---|---|---|
| 1892 | lib_dib_Filter15_IDBA_contig_32165_minus2 | 917 | chloride channel calcium activated 2-like | 976 | 1.07E-157 | 50.80% | 1 | F:protein binding | IPR002035 (SMART); IPR013642 (PFAM); IPR015394 (PFAM); G3DSA:3.40.50.410 (GENE3D), PTHR10579 (PANTHER), PF13519 (PFAM), tmhmm (TMHMM), SSF53300 (SUPERFAMILY) |
| 1893 | lib_dib_Filter15_IDBA_contig_32166_minus2 | 1051 | chloride channel calcium activated 2-like | 1058 | 6.50E-156 | 50.65% | 1 | F:protein binding | IPR002035 (SMART); IPR013642 (PFAM); IPR015394 (PFAM); G3DSA:3.40.50.410 (GENE3D), PTHR10579 (PANTHER), PF13519 (PFAM), tmhmm (TMHMM), SSF53300 (SUPERFAMILY) |
| 1894 | lib_dib_Filter15_IDBA_contig_32167_minus2 | 977 | chloride channel calcium activated 2-like | 976 | 6.01E-157 | 50.65% | 1 | F:protein binding | IPR002035 (SMART); IPR013642 (PFAM); IPR015394 (PFAM); G3DSA:3.40.50.410 (GENE3D), PTHR10579 (PANTHER), PF13519 (PFAM), tmhmm (TMHMM), SSF53300 (SUPERFAMILY) |
| 1895 | lib_dib_Filter15_IDBA_contig_32168_minus2 | 1039 | chloride channel calcium activated 2-like | 1178 | 1.19E-154 | 50.65% | 1 | F:protein binding | IPR002035 (SMART); IPR013642 (PFAM); IPR015394 (PFAM); G3DSA:3.40.50.410 (GENE3D), PTHR10579 (PANTHER), PF13519 (PFAM), tmhmm (TMHMM), SSF53300 (SUPERFAMILY) |
| 1896 | lib_dib_Filter15_IDBA_contig_32169_minus3 | 88 | chloride channel calcium activated 2-like | 166 | 7.80E-22 | 71.35% | 2 | C:secretory granule; C:integral to membrane | IPR013642 (PFAM); PTHR10579 (PANTHER), SignalP-NN(euk) (SIGNALP), tmhmm (TMHMM) |
| 1897 | lib_dib_Filter15_IDBA_contig_32170_minus2 | 21 | chloride channel calcium activated 2-like | 109 | 7.32E-16 | 71.25% | 2 | P:response to stress; C:cytoplasmic membrane-bounded vesicle | IPR013642 (PFAM); PTHR10579 (PANTHER), SignalP-NN(euk) (SIGNALP), tmhmm (TMHMM) |
| 1898 | lib_dib_Filter15_IDBA_contig_32171_minus2 | 48 | chloride channel calcium activated 2-like | 206 | 3.57E-45 | 58.65% | 1 | F:protein binding | IPR002035 (PROFILE); G3DSA:3.40.50.410 (GENE3D), PTHR10579 (PANTHER), PTHR10579:SF7 (PANTHER), PF13519 (PFAM), SSF53300 (SUPERFAMILY) |
| 1899 | lib_dib_Filter15_IDBA_contig_32172_minus2 | 74 | calcium-activated chloride channel regulator 4 | 263 | 3.22E-31 | 49.95% | 0 | - | IPR015394 (PFAM); PTHR10579 (PANTHER), SignalP-NN(euk) (SIGNALP) |
| 1900 | lib_dib_Filter15_IDBA_contig_32176_plus2 | 210 | aldo-keto reductase | 428 | 8.86E-20 | 44.40% | 4 | P:oxidation-reduction process; F:oxidoreductase activity; F:alditol:NADP+ 1-oxidoreductase activity; F:17-alpha,20-alpha-dihydroxypregn-4-en-3-one dehydrogenase activity | IPR001395 (PANTHER); IPR023210 (G3DSA:3.20.20.GENE3D); PTHR11732:SF34 (PANTHER) |
| 1901 | lib_dib_Filter15_IDBA_contig_32193_minus1 | 106 | cdc2l2 protein | 346 | 8.92E-93 | 93.95% | 17 | P:signal transduction; P:cellular protein modification process; P:cell death; P:regulation of biological process; P:nucleobase-containing compound metabolic process; ; P:cell growth; P:cell cycle; P:organelle organization; P:biological_process; F:nucleotide binding; F:protein binding; P:embryo development; F:protein kinase activity; P:cell proliferation; C:cytoplasm; C:nucleus | IPR000719 (PFAM); IPR002290 (SMART); IPR008271 (PROSITE); IPR011009 (SUPERFAMILY); IPR020635 (SMART); G3DSA:1.10.510.10 (GENE3D), G3DSA:3.30.200.20 (GENE3D), PTHR24056 (PANTHER), PTHR24056:SF96 (PANTHER) |
| 1902 | lib_dib_Filter15_IDBA_contig_32234_plus2 | 64 | cysteine-rich secretory protein mr30 | 340 | 1.75E-17 | 46.80% | 1 | C:extracellular region | IPR001283 (PRINTS); IPR002413 (PRINTS); IPR014044 (G3DSA:3.40.33.GENE3D) |
| 1903 | lib_dib_Filter15_IDBA_contig_32263_minus3 | 62 | calcium binding egf domain containing protein | 314 | 1.25E-22 | 54.00% | 2 | F:protein binding; F:calcium ion binding | IPR000742 (SMART); IPR001881 (SMART); IPR013032 (PROSITE); IPR026823 (PFAM); G3DSA:2.10.25.10 (GENE3D), PTHR10529 (PANTHER), SSF57196 (SUPERFAMILY) |
| 1904 | lib_dib_Filter15_IDBA_contig_32291_minus2 | 142 | death-associated protein kinase 1 | 615 | 1.39E-133 | 55.45% | 8 | P:cellular protein modification process; P:regulation of biological process; P:cell death; F:protein kinase activity; F:protein binding; F:nucleotide binding; P:signal transduction; C:intracellular | IPR002110 (SMART); IPR020675 (PANTHER); IPR020676 (PTHR22964:PANTHER); IPR020683 (G3DSA:1.25.40.GENE3D); G3DSA:3.40.50.300 (GENE3D), SSF52540 (SUPERFAMILY) |
| 1905 | lib_dib_Filter15_IDBA_contig_32336_plus1 | 167 | beta- -n-acetylgalactosaminyltransferase bre-4-like | 509 | 1.37E-74 | 61.30% | 3 | F:transferase activity; C:cell; P:carbohydrate metabolic process | IPR003859 (PRINTS); G3DSA:3.90.550.10 (GENE3D), PF13733 (PFAM), tmhmm (TMHMM), SSF53448 (SUPERFAMILY) |

| | | | | | | | | | |
|---|---|---|---|---|---|---|---|---|---|
| 1906 | lib_dib_Filter15_IDBA_contig_32339_plus2 | 689 | retinoid-inducible serine carboxypeptidase | 444 | 1.27E-128 | 69.40% | 3 | F:peptidase activity; P:protein metabolic process; P:catabolic process | IPR001563 (PRINTS); IPR018202 (PROSITE); G3DSA:3.40.50.1820 (GENE3D), PTHR11802:SF3 (PANTHER), SignalP-NN(euk) (SIGNALP), tmhmm (TMHMM), SSF53474 (SUPERFAMILY) | |
| 1907 | lib_dib_Filter15_IDBA_contig_32342_plus1 | 346 | serine carboxypeptidase 1 | 313 | 4.36E-68 | 64.75% | 3 | F:peptidase activity; P:protein metabolic process; P:catabolic process | IPR001563 (PANTHER); G3DSA:3.40.50.1820 (GENE3D), PTHR11802:SF3 (PANTHER), SSF53474 (SUPERFAMILY) | |
| 1908 | lib_dib_Filter15_IDBA_contig_32355_plus3 | 118 | tyrosine-protein kinase fps85d-like isoform 2 | 313 | 2.09E-76 | 83.10% | 13 | P:anatomical structure morphogenesis; P:cellular component organization; P:cell differentiation; P:multicellular organismal development; P:cellular protein modification process; C:plasma membrane; P:cytoskeleton organization; P:embryo development; F:protein kinase activity; F:nucleotide binding; P:regulation of biological process; P:cell growth; C:cytoplasm | IPR000719 (PROFILE); IPR001245 (PRINTS); IPR002290 (SMART); IPR008266 (PROSITE); IPR011009 (SUPERFAMILY); IPR020635 (SMART); G3DSA:1.10.510.10 (GENE3D), PTHR24418 (PANTHER) | |
| 1909 | lib_dib_Filter15_IDBA_contig_32408_plus2 | 37 | isoamyl acetate-hydrolyzing esterase 1 homolog | 313 | 3.25E-76 | 64.90% | 1 | F:hydrolase activity | IPR013831 (G3DSA:3.40.50.GENE3D); PTHR14209 (PANTHER), PF13472 (PFAM), SignalP-NN(euk) (SIGNALP), tmhmm (TMHMM), SSF52266 (SUPERFAMILY) | Yes |
| 1910 | lib_dib_Filter15_IDBA_contig_32413_plus2 | 40 | von willebrand factor a domain-containing protein 8-like | 313 | 2.48E-65 | 54.85% | 9 | F:ATPase activity; C:mitochondrion; F:ATP binding; F:nucleotide binding; F:nucleoside-triphosphatase activity; P:ATP catabolic process; F:molecular_function; P:biological_process; C:extracellular region | PTHR21610 (PANTHER), PTHR21610:SF2 (PANTHER) | |
| 1911 | lib_dib_Filter15_IDBA_contig_32438_plus1 | 29 | fat-like cadherin-related tumor suppressor-like protein | 313 | 6.30E-91 | 62.40% | 3 | F:calcium ion binding; P:biological_process; C:plasma membrane | IPR002126 (PRINTS); IPR015919 (SUPERFAMILY); IPR020894 (PROSITE); PTHR24027 (PANTHER) | |
| 1912 | lib_dib_Filter15_IDBA_contig_32442_minus2 | 10193 | neutral and basic amino acid transport protein rbat | 736 | 0 | 59.15% | 3 | F:hydrolase activity; P:carbohydrate metabolic process; F:binding | IPR006047 (PFAM); IPR006589 (SMART); IPR013781 (G3DSA:3.20.20.GENE3D); IPR015902 (PANTHER); IPR017853 (SUPERFAMILY); G3DSA:3.90.400.10 (GENE3D), PTHR10357:SF76 (PANTHER), tmhmm (TMHMM) | |
| 1913 | lib_dib_Filter15_IDBA_contig_32448_minus1 | 51 | acid trehalase-like protein 1-like | 313 | 4.82E-57 | 53.80% | 1 | F:catalytic activity | IPR005194 (PFAM); IPR008928 (SUPERFAMILY); G3DSA:2.60.420.10 (GENE3D), PTHR11051 (PANTHER), PTHR11051:SF4 (PANTHER), tmhmm (TMHMM) | |
| 1914 | lib_dib_Filter15_IDBA_contig_32457_plus2 | 1389 | neurogenic locus notch homolog protein 2-like | 866 | 3.66E-21 | 48.15% | 3 | P:biological_process; F:protein binding; F:calcium ion binding | IPR000152 (PROSITE); IPR000742 (PFAM); IPR001881 (SMART); IPR013032 (PROSITE); IPR018097 (PROSITE); G3DSA:2.10.25.10 (GENE3D), PTHR24838 (PANTHER), PTHR24838:SF112 (PANTHER), SignalP-NN(euk) (SIGNALP), tmhmm (TMHMM), SSF57196 (SUPERFAMILY) | Yes |
| 1915 | lib_dib_Filter15_IDBA_contig_32458_plus1 | 1140 | neurogenic locus notch homolog protein 2-like | 919 | 1.15E-12 | 45.05% | 2 | F:protein binding; F:calcium ion binding | IPR000742 (PFAM); IPR001881 (SMART); IPR013032 (PROSITE); G3DSA:2.10.25.10 (GENE3D), PTHR24044 (PANTHER), PTHR24044:SF49 (PANTHER), tmhmm (TMHMM), SSF57196 (SUPERFAMILY) | |
| 1916 | lib_dib_Filter15_IDBA_contig_32462_minus3 | 115 | carbohydrate sulfotransferase 9 | 444 | 2.56E-19 | 54.45% | 4 | F:transferase activity; C:cell; P:biosynthetic process; P:carbohydrate metabolic process | IPR005331 (PFAM); IPR018011 (PANTHER); tmhmm (TMHMM) | |

| | | | | | | | | | | |
|---|---|---|---|---|---|---|---|---|---|---|
| 1917 | lib_dib_Filter15_IDBA_contig_32478_minus1 | 80 | a chain x-ray structure of c-met with triazolopyridazine | 312 | 1.46E-110 | 75.90% | 23 | P:metabolic process; P:regulation of biological process; P:multicellular organismal development; P:anatomical structure morphogenesis; P:cellular component organization; P:cell differentiation; C:plasma membrane; P:cellular protein modification process; P:nucleobase-containing compound metabolic process; ; P:signal transduction; P:biological_process; C:cellular_component; P:reproduction; C:cell; F:receptor activity; F:protein kinase activity; P:transport; P:cell proliferation; F:protein binding; C:nucleolus; C:extracellular region; F:nucleotide binding | IPR000719 (PROFILE); IPR001245 (PRINTS); IPR002290 (SMART); IPR008266 (PROSITE); IPR011009 (SUPERFAMILY); IPR020635 (SMART); G3DSA:1.10.510.10 (GENE3D), G3DSA:3.30.200.20 (GENE3D), PTHR24416 (PANTHER) | |
| 1918 | lib_dib_Filter15_IDBA_contig_32480_plus3 | 1035 | elastase 2a | 638 | 4.63E-42 | 53.65% | 3 | F:peptidase activity; P:protein metabolic process; P:catabolic process | IPR001254 (PFAM); IPR001314 (PRINTS); IPR009003 (SUPERFAMILY); IPR018114 (PROSITE); G3DSA:2.40.10.10 (GENE3D), PTHR24256 (PANTHER), SignalP-NN(euk) (SIGNALP) | Yes |
| 1919 | lib_dib_Filter15_IDBA_contig_32488_minus3 | 44 | beta- -galactosyltransferase 2 | 311 | 6.49E-37 | 63.40% | 9 | C:Golgi apparatus; P:multicellular organismal development; F:transferase activity; P:carbohydrate metabolic process; P:biological_process; C:cell; P:regulation of biological process; F:binding; P:reproduction | IPR003859 (PRINTS); G3DSA:3.90.550.10 (GENE3D), PTHR19300:SF7 (PANTHER), SSF53448 (SUPERFAMILY) | |
| 1920 | lib_dib_Filter15_IDBA_contig_32494_minus2 | 53 | endoplasmic reticulum mannosyl-oligosaccharide -alpha-mannosidase | 312 | 3.03E-68 | 71.80% | 6 | C:cell; F:hydrolase activity; F:calcium ion binding; P:protein metabolic process; P:catabolic process; C:endoplasmic reticulum | IPR001382 (PRINTS); PTHR11742:SF7 (PANTHER), tmhmm (TMHMM) | |
| 1921 | lib_dib_Filter15_IDBA_contig_32519_plus1 | 74 | neuroglobin | 312 | 1.54E-34 | 57.65% | 3 | F:binding; P:transport; F:oxygen binding | IPR000971 (PFAM); IPR009050 (SUPERFAMILY); IPR012292 (G3DSA:1.10.490.GENE3D); IPR013316 (PRINTS); PTHR22924 (PANTHER) | |
| 1922 | lib_dib_Filter15_IDBA_contig_32529_minus1 | 99 | upf0669 protein c6orf120 homolog | 312 | 6.17E-41 | 64.40% | 2 | C:extracellular region; P:apoptotic process | PTHR31703 (PANTHER), PTHR31703:SF0 (PANTHER), tmhmm (TMHMM) | |
| 1923 | lib_dib_Filter15_IDBA_contig_32533_plus3 | 204 | amine oxidase | 606 | 3.08E-82 | 58.45% | 2 | F:catalytic activity; P:metabolic process | IPR001613 (PRINTS); IPR002937 (PFAM); G3DSA:3.90.660.10 (GENE3D), PTHR10742 (PANTHER), PTHR10742:SF108 (PANTHER), tmhmm (TMHMM), SSF51905 (SUPERFAMILY), SSF54373 (SUPERFAMILY) | |
| 1924 | lib_dib_Filter15_IDBA_contig_32534_plus1 | 359 | amine oxidase | 881 | 1.17E-129 | 56.50% | 3 | F:catalytic activity; P:metabolic process; F:nucleotide binding | IPR001613 (PRINTS); IPR002937 (PFAM); IPR016040 (G3DSA:3.40.50.GENE3D); G3DSA:1.10.405.10 (GENE3D), G3DSA:3.90.660.10 (GENE3D), PTHR10742 (PANTHER), PTHR10742:SF108 (PANTHER), tmhmm (TMHMM), SSF51905 (SUPERFAMILY), SSF54373 (SUPERFAMILY) | |
| 1925 | lib_dib_Filter15_IDBA_contig_32545_plus2 | 626 | a disintegrin and metalloproteinase with thrombospondin motifs 13 | 557 | 4.27E-19 | 45.90% | 3 | F:peptidase activity; P:protein metabolic process; P:catabolic process | IPR001590 (PROFILE); IPR024079 (G3DSA:3.40.390.GENE3D); PTHR13723 (PANTHER), PF13688 (PFAM), tmhmm (TMHMM), SSF55486 (SUPERFAMILY) | |
| 1926 | lib_dib_Filter15_IDBA_contig_32547_plus1 | 2871 | a disintegrin and metalloproteinase with thrombospondin motifs 18 | 594 | 2.20E-20 | 43.25% | 3 | F:peptidase activity; P:protein metabolic process; P:catabolic process | IPR001590 (PROFILE); IPR024079 (G3DSA:3.40.390.GENE3D); PTHR13723 (PANTHER), PF13688 (PFAM), SignalP-NN(euk) (SIGNALP), SSF55486 (SUPERFAMILY) | Yes |

| | | | | | | | | |
|---|---|---|---|---|---|---|---|---|
| 1927 | lib_dib_Filter15_IDBA_contig_32555_plus3 | 223 | protocadherin-11 x-linked | 667 | 4.39E-140 | 53.45% | 3 | F:calcium ion binding; P:biological_process; C:plasma membrane | IPR002126 (PRINTS); IPR015919 (SUPERFAMILY); IPR020894 (PROSITE); PTHR24028 (PANTHER), PTHR24028:SF12 (PANTHER) |
| 1928 | lib_dib_Filter15_IDBA_contig_32562_minus2 | 135 | neuronal cell adhesion | 446 | 2.25E-74 | 45.90% | 1 | F:protein binding | IPR003598 (SMART); IPR003599 (SMART); IPR007110 (PROFILE); IPR013098 (PFAM); IPR013783 (G3DSA:2.60.40.GENE3D); PTHR10489 (PANTHER), PF13895 (PFAM), SSF48726 (SUPERFAMILY) |
| 1929 | lib_dib_Filter15_IDBA_contig_32563_minus2 | 187 | neuronal cell adhesion | 521 | 2.42E-73 | 45.90% | 1 | F:protein binding | IPR003598 (SMART); IPR003599 (SMART); IPR007110 (PROFILE); IPR013098 (PFAM); IPR013783 (G3DSA:2.60.40.GENE3D); PTHR10489 (PANTHER), PF13895 (PFAM), SSF48726 (SUPERFAMILY) |
| 1930 | lib_dib_Filter15_IDBA_contig_32626_plus1 | 134 | peptidylglycine alpha-amidating monooxygenase | 478 | 5.95E-139 | 57.85% | 27 | P:reproduction; P:regulation of biological process; P:protein transport; C:cell; P:multicellular organismal development; C:Golgi apparatus; F:binding; F:catalytic activity; ; F:protein binding; P:cellular protein modification process; P:lipid metabolic process; P:response to abiotic stimulus; P:anatomical structure morphogenesis; C:extracellular space; F:calcium ion binding; P:response to endogenous stimulus; P:metabolic process; P:cytoskeleton organization; P:cellular component organization; P:cell differentiation; C:cytoplasmic membrane-bounded vesicle; P:biological_process; P:response to stress; P:secondary metabolic process; C:cytoplasm; C:plasma membrane | IPR000720 (PRINTS); IPR001258 (PFAM); IPR008977 (SUPERFAMILY); IPR011042 (G3DSA:2.120.10.GENE3D); IPR013017 (PROFILE); IPR014784 (G3DSA:2.60.120.GENE3D); PTHR10680 (PANTHER), PTHR10680:SF11 (PANTHER), PF03712 (PFAM), tmhmm (TMHMM), SSF101898 (SUPERFAMILY) |
| 1931 | lib_dib_Filter15_IDBA_contig_32684_plus1 | 25 | zinc finger protein 271-like | 310 | 3.24E-40 | 65.50% | 3 | C:intracellular; F:binding; F:nucleic acid binding | IPR007087 (PROSITE); IPR013087 (G3DSA:3.30.160.GENE3D); IPR015880 (SMART); PTHR11389 (PANTHER), PF13465 (PFAM), SSF57667 (SUPERFAMILY) |
| 1932 | lib_dib_Filter15_IDBA_contig_32689_plus1 | 226 | fch and double sh3 domains protein 2-like | 574 | 3.04E-38 | 57.35% | 9 | P:regulation of biological process; P:cytoskeleton organization; P:cell-cell signaling; C:endosome; F:protein binding; P:cellular component organization; P:cell differentiation; P:multicellular organismal development; P:growth | IPR001452 (PRINTS); G3DSA:2.30.30.40 (GENE3D), PTHR15735 (PANTHER) |
| 1933 | lib_dib_Filter15_IDBA_contig_32690_plus3 | 330 | nervous isoform c | 971 | 7.32E-138 | 62.30% | 9 | P:regulation of biological process; P:cytoskeleton organization; P:cell-cell signaling; C:endosome; F:protein binding; P:cellular component organization; P:cell differentiation; P:multicellular organismal development; P:growth | IPR001060 (PFAM); IPR001452 (PRINTS); G3DSA:2.30.30.40 (GENE3D), PTHR15735 (PANTHER) |
| 1934 | lib_dib_Filter15_IDBA_contig_32721_plus3 | 66 | bursicon | 319 | 2.30E-24 | 59.90% | 2 | F:receptor binding; P:multicellular organismal development | IPR004133 (PFAM); IPR006207 (SMART) |
| 1935 | lib_dib_Filter15_IDBA_contig_32725_minus3 | 163 | myeloid zinc finger 1 | 462 | 3.46E-28 | 59.45% | 3 | F:binding; F:nucleic acid binding; C:intracellular | IPR007087 (PFAM); IPR013087 (G3DSA:3.30.160.GENE3D); IPR015880 (SMART); PTHR11389 (PANTHER), PF13465 (PFAM), PF13912 (PFAM), SSF57667 (SUPERFAMILY) |

| | | | | | | | | | | |
|---|---|---|---|---|---|---|---|---|---|---|
| 1936 | lib_dib_Filter15_IDBA_contig_32815_minus1 | 40 | ryk partial | 309 | 1.10E-71 | 85.00% | 18 | F:receptor binding; P:signal transduction; C:cell; C:cytoplasm; P:cellular protein modification process; F:receptor activity; P:behavior; P:response to external stimulus; F:protein kinase activity; P:anatomical structure morphogenesis; P:multicellular organismal development; C:plasma membrane; F:protein binding; C:nucleus; F:nucleotide binding; P:cellular component organization; P:cell differentiation; P:regulation of biological process | IPR000719 (PROFILE); IPR001245 (PRINTS); IPR011009 (SUPERFAMILY); IPR020635 (SMART); G3DSA:1.10.510.10 (GENE3D), PTHR24416 (PANTHER), PTHR24416:SF85 (PANTHER) | |
| 1937 | lib_dib_Filter15_IDBA_contig_32820_plus3 | 1597 | carbonic anhydrase | 492 | 3.43E-68 | 58.90% | 3 | F:catalytic activity; P:metabolic process; F:binding | IPR001148 (G3DSA:3.10.200.GENE3D); IPR018338 (PROSITE); IPR023561 (PANTHER); SignalP-NN(euk) (SIGNALP), tmhmm (TMHMM) | |
| 1938 | lib_dib_Filter15_IDBA_contig_32844_minus3 | 124 | protein | 308 | 7.01E-43 | 48.45% | 2 | F:protein binding; F:carbohydrate binding | IPR000742 (PROFILE); IPR000922 (PFAM); IPR013032 (PROSITE); PTHR12011 (PANTHER), PTHR12011:SF4 (PANTHER) | |
| 1939 | lib_dib_Filter15_IDBA_contig_32849_minus3 | 61 | tyrosine protein | 340 | 1.68E-53 | 58.05% | 3 | F:nucleotide binding; P:cellular protein modification process; F:protein kinase activity | IPR000719 (PROFILE); IPR001245 (PRINTS); IPR002290 (SMART); IPR008266 (PROSITE); IPR011009 (SUPERFAMILY); IPR017441 (PROSITE); IPR020635 (SMART); G3DSA:1.10.510.10 (GENE3D), G3DSA:3.30.200.20 (GENE3D), PTHR24418 (PANTHER) | |
| 1940 | lib_dib_Filter15_IDBA_contig_32852_plus2 | 38 | e3 ubiquitin-protein ligase lrsam1 | 308 | 3.68E-75 | 70.75% | 12 | F:catalytic activity; C:cell; P:cellular protein modification process; P:transport; P:cellular component organization; P:regulation of biological process; P:cell differentiation; P:multicellular organismal development; P:reproduction; P:viral reproduction; C:cytoplasm; F:protein binding | IPR001611 (PFAM); IPR003591 (SMART); G3DSA:3.80.10.10 (GENE3D), PTHR23155 (PANTHER), PTHR23155:SF13 (PANTHER), PF13855 (PFAM), SM00364 (SMART), SSF52058 (SUPERFAMILY) | |
| 1941 | lib_dib_Filter15_IDBA_contig_32864_minus3 | 78 | fibrinogen-like protein 1 | 495 | 4.29E-49 | 54.75% | 4 | F:receptor binding; C:extracellular space; P:response to stilbenoid; P:signal transduction | IPR002181 (PFAM); IPR014715 (G3DSA:4.10.530.GENE3D); IPR014716 (G3DSA:3.90.215.GENE3D); PTHR19143 (PANTHER), SignalP-NN(euk) (SIGNALP), tmhmm (TMHMM) | Yes |
| 1942 | lib_dib_Filter15_IDBA_contig_32865_minus3 | 71 | fibrinogen-like protein 1 | 297 | 6.06E-49 | 51.70% | 5 | F:receptor binding; C:extracellular space; P:response to stilbenoid; P:signal transduction; C:fibrinogen complex | IPR002181 (PFAM); IPR014715 (G3DSA:4.10.530.GENE3D); IPR014716 (G3DSA:3.90.215.GENE3D); PTHR19143 (PANTHER), SignalP-NN(euk) (SIGNALP), tmhmm (TMHMM) | Yes |
| 1943 | lib_dib_Filter15_IDBA_contig_32889_minus3 | 159 | cysteine-rich secretory protein isoform cra_a | 334 | 4.01E-51 | 48.45% | 5 | C:extracellular region; P:proteolysis; F:hydrolase activity; F:peptidase activity; C:extracellular space | IPR001283 (PRINTS); IPR014044 (G3DSA:3.40.33.GENE3D); SignalP-NN(euk) (SIGNALP), tmhmm (TMHMM) | Yes |
| 1944 | lib_dib_Filter15_IDBA_contig_32891_minus1 | 35 | four-jointed box protein 1-like | 308 | 4.80E-104 | 64.05% | 1 | C:extracellular region | IPR009581 (PFAM); IPR024868 (PRINTS) | |
| 1945 | lib_dib_Filter15_IDBA_contig_32905_plus1 | 35 | pr domain zinc finger protein 4 | 308 | 1.34E-67 | 57.20% | 7 | P:regulation of biological process; P:signal transduction; ; C:intracellular; F:protein binding; F:binding; F:nucleic acid binding | IPR001214 (PROFILE); IPR007087 (PFAM); IPR013087 (G3DSA:3.30.160.GENE3D); IPR015880 (SMART); G3DSA:2.170.270.10 (GENE3D), PTHR24402 (PANTHER), PTHR24402:SF112 (PANTHER), PF13465 (PFAM), PF13894 (PFAM), SSF57667 (SUPERFAMILY) | |
| 1946 | lib_dib_Filter15_IDBA_contig_32940_minus3 | 48 | isoform b | 307 | 6.88E-46 | 53.55% | 3 | C:proteinaceous extracellular matrix; F:peptidase activity; F:binding | IPR000884 (PFAM); IPR013273 (PRINTS); G3DSA:2.20.100.10 (GENE3D), PTHR13723 (PANTHER), tmhmm (TMHMM) | |

| | | | | | | | | | | |
|---|---|---|---|---|---|---|---|---|---|---|
| 1947 | lib_dib_Filter15_IDBA_contig_32951_plus2 | 107 | perlucin 1 | 383 | 5.71E-15 | 50.60% | 1 | F:carbohydrate binding | IPR001304 (PFAM); IPR016186 (G3DSA:3.10.100.GENE3D); IPR016187 (SUPERFAMILY); PTHR22802 (PANTHER) | |
| 1948 | lib_dib_Filter15_IDBA_contig_33006_plus2 | 271 | carboxypeptidase d | 894 | 0 | 64.25% | 5 | F:peptidase activity; C:cell; P:protein metabolic process; P:catabolic process; F:binding | IPR000834 (PRINTS); IPR008969 (SUPERFAMILY); IPR014766 (G3DSA:2.60.40.GENE3D); IPR015567 (PTHR11532:PANTHER); G3DSA:3.40.630.10 (GENE3D), PTHR11532 (PANTHER), PF13620 (PFAM), SSF53187 (SUPERFAMILY) | |
| 1949 | lib_dib_Filter15_IDBA_contig_33020_plus1 | 49 | chondroitin sulfate synthase 2-like | 307 | 7.15E-100 | 65.90% | 1 | C:Golgi apparatus | IPR008428 (PANTHER); PTHR12369:SF5 (PANTHER) | |
| 1950 | lib_dib_Filter15_IDBA_contig_331_plus1 | 1767 | group iii secreted phospholipase | 117 | 5.13E-28 | 69.00% | 4 | F:hydrolase activity; P:catabolic process; P:lipid metabolic process; F:calcium ion binding | IPR001211 (PANTHER); IPR013090 (PROSITE); IPR016090 (G3DSA:1.20.90.GENE3D) | |
| 1951 | lib_dib_Filter15_IDBA_contig_33102_minus1 | 412 | serine threonine-protein kinase 3 | 631 | 0 | 78.50% | 4 | P:cellular protein modification process; F:nucleotide binding; P:signal transduction; F:protein kinase activity | IPR000719 (PFAM); IPR002290 (SMART); IPR011009 (SUPERFAMILY); IPR011524 (PROFILE); IPR017441 (PROSITE); IPR020635 (SMART); IPR024205 (PFAM); G3DSA:1.10.510.10 (GENE3D), G3DSA:3.30.200.20 (GENE3D), PTHR24361 (PANTHER), PTHR24361:SF193 (PANTHER), SignalP-NN(euk) (SIGNALP), tmhmm (TMHMM) | |
| 1952 | lib_dib_Filter15_IDBA_contig_33125_plus2 | 7251 | protein | 344 | 2.96E-27 | 47.45% | 1 | F:protein binding | IPR002350 (PFAM); G3DSA:1.10.1890.10 (GENE3D), G3DSA:3.30.60.30 (GENE3D), PTHR10913 (PANTHER), PTHR10913:SF21 (PANTHER), SignalP-NN(euk) (SIGNALP), tmhmm (TMHMM), SSF100895 (SUPERFAMILY) | Yes |
| 1953 | lib_dib_Filter15_IDBA_contig_33130_minus1 | 1249 | coagulation factor x | 551 | 1.15E-38 | 55.10% | 3 | F:peptidase activity; P:protein metabolic process; P:catabolic process | IPR001254 (PFAM); IPR001314 (PRINTS); IPR009003 (SUPERFAMILY); IPR018114 (PROSITE); G3DSA:2.40.10.10 (GENE3D), PTHR24265 (PANTHER), SignalP-NN(euk) (SIGNALP), tmhmm (TMHMM) | Yes |
| 1954 | lib_dib_Filter15_IDBA_contig_33131_minus1 | 1206 | coagulation factor x | 534 | 1.61E-38 | 55.15% | 3 | F:peptidase activity; P:protein metabolic process; P:catabolic process | IPR001254 (PFAM); IPR001314 (PRINTS); IPR009003 (SUPERFAMILY); IPR018114 (PROSITE); G3DSA:2.40.10.10 (GENE3D), PTHR24265 (PANTHER), SignalP-NN(euk) (SIGNALP), tmhmm (TMHMM) | Yes |
| 1955 | lib_dib_Filter15_IDBA_contig_33136_minus3 | 83 | polypeptide n-acetylgalactosaminyltransferase 5 | 305 | 6.84E-125 | 75.35% | 5 | P:biosynthetic process; P:cellular protein modification process; P:carbohydrate metabolic process; F:transferase activity; C:cell | IPR000772 (PFAM); G3DSA:2.80.10.50 (GENE3D), PTHR11675 (PANTHER), SSF53448 (SUPERFAMILY) | |
| 1956 | lib_dib_Filter15_IDBA_contig_33141_plus3 | 59 | zinc finger protein 236 | 373 | 1.91E-73 | 58.35% | 3 | F:binding; F:nucleic acid binding; C:intracellular | IPR007087 (PROSITE); IPR013087 (G3DSA:3.30.160.GENE3D); IPR015880 (SMART); PTHR24396 (PANTHER), PTHR24396:SF0 (PANTHER), PF13465 (PFAM), SSF57667 (SUPERFAMILY) | |
| 1957 | lib_dib_Filter15_IDBA_contig_33160_plus1 | 1375 | epididymal sperm-binding protein 1-like | 742 | 4.50E-42 | 41.30% | 3 | C:extracellular region; P:carbohydrate metabolic process; F:carbohydrate binding | IPR000562 (G3DSA:2.10.10.GENE3D); IPR002557 (PROFILE); IPR003609 (PROFILE); IPR013806 (SUPERFAMILY); PTHR22918 (PANTHER), PTHR22918:SF0 (PANTHER), SignalP-NN(euk) (SIGNALP), tmhmm (TMHMM) | |
| 1958 | lib_dib_Filter15_IDBA_contig_33199_plus1 | 58 | neuroligin- y-linked-like | 305 | 4.00E-126 | 49.25% | 6 | P:anatomical structure morphogenesis; P:multicellular organismal development; C:cell; P:cell differentiation; P:regulation of biological process; F:binding | IPR002018 (PFAM); G3DSA:3.40.50.1820 (GENE3D), PTHR11559 (PANTHER), tmhmm (TMHMM), SSF53474 (SUPERFAMILY) | |
| 1959 | lib_dib_Filter15_IDBA_contig_33231_minus3 | 80 | sh3 domain-containing kinase-binding protein 1-like | 410 | 6.32E-19 | 75.45% | 1 | F:protein binding | IPR001452 (PRINTS); IPR011511 (PFAM); G3DSA:2.30.30.40 (GENE3D), PTHR14167 (PANTHER) | |

| | | | | | | | | | | |
|---|---|---|---|---|---|---|---|---|---|---|
| 1960 | lib_dib_Filter15_IDBA_contig_33254_plus1 | 58 | class member 1 | 436 | 1.78E-154 | 66.00% | 8 | P:protein metabolic process; P:catabolic process; F:calcium ion binding; P:biosynthetic process; P:cellular protein modification process; P:carbohydrate metabolic process; C:cell; F:hydrolase activity | IPR001382 (PRINTS); PTHR11742:SF7 (PANTHER) | |
| 1961 | lib_dib_Filter15_IDBA_contig_33274_plus1 | 43 | adam family mig-17 | 304 | 9.28E-71 | 47.60% | 3 | F:peptidase activity; P:protein metabolic process; P:catabolic process | IPR001590 (PROFILE); IPR024079 (G3DSA:3.40.390.GENE3D); PTHR13723 (PANTHER), PTHR13723:SF22 (PANTHER), PF13574 (PFAM), SSF55486 (SUPERFAMILY) | |
| 1962 | lib_dib_Filter15_IDBA_contig_33281_plus1 | 42 | af355375_1 reverse transcriptase | 199 | 1.23E-34 | 49.90% | 1 | F:binding | PTHR21301 (PANTHER) | |
| 1963 | lib_dib_Filter15_IDBA_contig_33321_plus1 | 372 | dnaj homolog subfamily c member 3 | 680 | 1.88E-102 | 59.35% | 7 | F:protein binding; P:metabolic process; P:regulation of biological process; C:endoplasmic reticulum; P:signal transduction; P:response to stress; P:response to biotic stimulus | IPR001623 (PRINTS); IPR011990 (G3DSA:1.25.40.GENE3D); IPR013026 (PROFILE); IPR013105 (PFAM); IPR019734 (SMART); IPR026901 (PTHR24078:PANTHER); PTHR24078 (PANTHER), PF13371 (PFAM), PF13414 (PFAM), SignalP-NN(euk) (SIGNALP), tmhmm (TMHMM), SSF48452 (SUPERFAMILY) | Yes |
| 1964 | lib_dib_Filter15_IDBA_contig_33322_plus2 | 412 | subfamily member 3 | 697 | 2.84E-102 | 59.35% | 7 | F:protein binding; P:metabolic process; P:regulation of biological process; C:endoplasmic reticulum; P:signal transduction; P:response to stress; P:response to biotic stimulus | IPR001623 (PRINTS); IPR011990 (G3DSA:1.25.40.GENE3D); IPR013026 (PROFILE); IPR013105 (PFAM); IPR019734 (SMART); IPR026901 (PTHR24078:PANTHER); PTHR24078 (PANTHER), PF13371 (PFAM), PF13414 (PFAM), SignalP-NN(euk) (SIGNALP), tmhmm (TMHMM), SSF48452 (SUPERFAMILY) | |
| 1965 | lib_dib_Filter15_IDBA_contig_33323_plus1 | 211 | dnaj homolog subfamily c member 3-like | 402 | 2.35E-26 | 59.25% | 7 | F:protein binding; P:metabolic process; P:regulation of biological process; C:endoplasmic reticulum; P:signal transduction; P:response to stress; P:response to biotic stimulus | IPR001623 (PRINTS); IPR011990 (G3DSA:1.25.40.GENE3D); IPR026901 (PTHR24078:PANTHER); PTHR24078 (PANTHER) | |
| 1966 | lib_dib_Filter15_IDBA_contig_33324_minus3 | 599 | a chain crystal structure of porcine beta trypsin with % polydocanol | 541 | 3.37E-53 | 57.85% | 3 | F:peptidase activity; P:protein metabolic process; P:catabolic process | IPR001254 (PFAM); IPR001314 (PRINTS); IPR009003 (SUPERFAMILY); IPR018114 (PROSITE); G3DSA:2.40.10.10 (GENE3D), PTHR24265 (PANTHER) | |
| 1967 | lib_dib_Filter15_IDBA_contig_33337_plus2 | 39 | ankyrin repeat domain-containing protein 50- partial | 303 | 1.88E-68 | 59.25% | 1 | F:protein binding | IPR002110 (PRINTS); IPR020683 (G3DSA:1.25.40.GENE3D); PTHR24198 (PANTHER), PF13637 (PFAM) | |
| 1968 | lib_dib_Filter15_IDBA_contig_33405_minus3 | 1902 | annexin a7 | 633 | 2.54E-142 | 75.45% | 16 | C:cytosol; P:cellular homeostasis; P:anatomical structure morphogenesis; P:cellular component organization; P:regulation of biological process; P:response to stress; P:response to abiotic stimulus; F:lipid binding; C:organelle; P:biological_process; F:protein binding; C:cell; C:nuclear envelope; P:cell proliferation; C:plasma membrane; F:calcium ion binding | IPR001464 (PRINTS); IPR018252 (PROSITE); IPR018502 (G3DSA:1.10.220.GENE3D); SignalP-NN(euk) (SIGNALP) | Yes |
| 1969 | lib_dib_Filter15_IDBA_contig_33457_plus3 | 208 | glutaredoxin-3 | 465 | 7.02E-148 | 77.70% | 5 | F:electron carrier activity; P:regulation of biological process; P:cellular homeostasis; C:cytoplasm; F:catalytic activity | IPR002109 (PFAM); IPR004480 (PANTHER); IPR012336 (G3DSA:3.40.30.GENE3D); IPR013766 (PFAM); PTHR10293:SF17 (PANTHER) | |
| 1970 | lib_dib_Filter15_IDBA_contig_3346_minus1 | 603 | ---NA--- | 282 | | | 0 | - | no IPS match | |
| 1971 | lib_dib_Filter15_IDBA_contig_3346_plus3 | 603 | ---NA--- | 281 | | | 0 | - | IPR008160 (PFAM); PTHR24023 (PANTHER), tmhmm (TMHMM) | |

| | | | | | | | | | | |
|---|---|---|---|---|---|---|---|---|---|---|
| 1972 | lib_dib_Filter15_IDBA_contig_33499_plus2 | 555 | serine threonine-protein kinase sgk3 | 778 | 0 | 75.80% | 10 | P:cellular protein modification process; F:protein kinase activity; F:lipid binding; C:endosome; P:regulation of biological process; P:cell death; C:cytoplasmic membrane-bounded vesicle; F:nucleotide binding; P:cell communication; F:protein binding | IPR000719 (PFAM); IPR000961 (SMART); IPR001683 (G3DSA:3.30.1520.GENE3D); IPR002290 (SMART); IPR011009 (SUPERFAMILY); IPR017441 (PROSITE); IPR020635 (SMART); G3DSA:1.10.510.10 (GENE3D), G3DSA:3.30.200.20 (GENE3D), PTHR24356 (PANTHER), PTHR24356:SF121 (PANTHER) | |
| 1973 | lib_dib_Filter15_IDBA_contig_33500_plus1 | 344 | serine threonine-protein kinase sgk1 isoform 1 | 520 | 0 | 85.95% | 11 | P:cellular protein modification process; F:protein kinase activity; C:mitochondrion; P:cell death; F:lipid binding; F:nucleotide binding; P:cell communication; C:endoplasmic reticulum; C:nucleus; C:plasma membrane; P:response to stress | IPR000719 (PFAM); IPR000961 (SMART); IPR002290 (SMART); IPR011009 (SUPERFAMILY); IPR017441 (PROSITE); IPR020635 (SMART); G3DSA:1.10.510.10 (GENE3D), G3DSA:3.30.200.20 (GENE3D), PTHR24356 (PANTHER), PTHR24356:SF120 (PANTHER) | |
| 1974 | lib_dib_Filter15_IDBA_contig_33510_minus1 | 101 | proline racemase | 302 | 8.03E-87 | 71.80% | 1 | F:catalytic activity | IPR008794 (PFAM); G3DSA:3.10.310.10 (GENE3D), PTHR32298 (PANTHER), PTHR32298:SF0 (PANTHER), SSF54506 (SUPERFAMILY) | |
| 1975 | lib_dib_Filter15_IDBA_contig_33534_plus2 | 1015 | neuronal cell adhesion | 620 | 1.16E-72 | 45.75% | 3 | P:biological_process; C:cell; F:protein binding | IPR003598 (SMART); IPR003599 (SMART); IPR007110 (PROFILE); IPR013098 (PFAM); IPR013783 (G3DSA:2.60.40.GENE3D); PTHR10489 (PANTHER), PF13895 (PFAM), SignalP-NN(euk) (SIGNALP), SSF48726 (SUPERFAMILY) | Yes |
| 1976 | lib_dib_Filter15_IDBA_contig_33535_plus3 | 1017 | neuronal cell adhesion | 623 | 1.42E-72 | 45.75% | 3 | P:biological_process; C:cell; F:protein binding | IPR003598 (SMART); IPR003599 (SMART); IPR007110 (PROFILE); IPR013098 (PFAM); IPR013783 (G3DSA:2.60.40.GENE3D); PTHR10489 (PANTHER), PF13895 (PFAM), SignalP-NN(euk) (SIGNALP), SSF48726 (SUPERFAMILY) | Yes |
| 1977 | lib_dib_Filter15_IDBA_contig_33554_minus3 | 35 | nicotinic acetylcholine receptor subunit type e | 301 | 2.27E-120 | 77.40% | 3 | F:ion channel activity; P:ion transport; C:cell | IPR002394 (PRINTS); IPR006029 (PFAM); IPR006201 (PRINTS); IPR006202 (G3DSA:2.70.170.GENE3D); IPR018000 (PROSITE); G3DSA:1.20.120.370 (GENE3D), PTHR18945:SF88 (PANTHER), tmhmm (TMHMM) | |
| 1978 | lib_dib_Filter15_IDBA_contig_33561_plus2 | 55 | reelin isoform 1 | 301 | 1.80E-36 | 54.30% | 15 | P:multicellular organismal development; F:receptor binding; P:anatomical structure morphogenesis; P:cellular component organization; P:cell differentiation; P:signal transduction; P:behavior; C:extracellular space; P:metabolic process; P:response to stress; C:cytoplasm; P:regulation of biological process; ; P:cell-cell signaling; C:cell | IPR002861 (PFAM); PTHR11841 (PANTHER), PTHR11841:SF0 (PANTHER) | |
| 1979 | lib_dib_Filter15_IDBA_contig_33579_minus2 | 37 | beta-lactamase | 301 | 4.17E-46 | 59.15% | 3 | F:hydrolase activity, acting on carbon-nitrogen (but not peptide) bonds, in linear amides; F:hydrolase activity, acting on carbon-nitrogen (but not peptide) bonds; F:zinc ion binding | IPR001466 (PFAM); IPR012338 (G3DSA:3.40.710.GENE3D); PTHR22935 (PANTHER), PTHR22935:SF37 (PANTHER) | |
| 1980 | lib_dib_Filter15_IDBA_contig_33592_plus1 | 32 | ankyrin repeat protein | 301 | 6.63E-29 | 52.85% | 1 | F:protein binding | IPR002110 (PFAM); IPR020683 (G3DSA:1.25.40.GENE3D); PTHR24188 (PANTHER) | |
| 1981 | lib_dib_Filter15_IDBA_contig_33602_plus2 | 100 | type-2 ice-structuring | 393 | 7.53E-39 | 51.45% | 1 | F:carbohydrate binding | IPR001304 (PFAM); IPR016186 (G3DSA:3.10.100.GENE3D); IPR016187 (SUPERFAMILY); IPR018378 (PROSITE); PTHR22801 (PANTHER) | |

| | | | | | | | | | | |
|---|---|---|---|---|---|---|---|---|---|---|
| 1982 | lib_dib_Filter15_IDBA_contig_33620_minus1 | 201 | dehydrogenase reductase (sdr family) member 7 | 301 | 8.71E-72 | 68.50% | 3 | P:metabolic process; F:catalytic activity; F:nucleotide binding | IPR002198 (PRINTS); IPR002347 (PRINTS); IPR016040 (G3DSA:3.40.50.GENE3D); IPR020904 (PROSITE); PTHR24322 (PANTHER), PTHR24322:SF27 (PANTHER), SignalP-NN(euk) (SIGNALP), tmhmm (TMHMM), SSF51735 (SUPERFAMILY) | |
| 1983 | lib_dib_Filter15_IDBA_contig_33699_plus2 | 86 | proopiomelanocortin | 300 | 1.05E-112 | 71.40% | 3 | C:extracellular region; P:signal transduction; F:receptor binding | IPR001941 (PRINTS); IPR013531 (PFAM); IPR013532 (PFAM); IPR013593 (PFAM); PTHR11416 (PANTHER), PTHR11416:SF6 (PANTHER), SignalP-NN(euk) (SIGNALP) | |
| 1984 | lib_dib_Filter15_IDBA_contig_33720_plus2 | 49 | abhydrolase domain-containing protein 3 | 300 | 2.76E-88 | 67.55% | 1 | F:hydrolase activity | IPR012020 (PIR); G3DSA:3.40.50.1820 (GENE3D), PTHR10794 (PANTHER), PTHR10794:SF23 (PANTHER), PF12697 (PFAM), SSF53474 (SUPERFAMILY) | |
| 1985 | lib_dib_Filter15_IDBA_contig_33723_minus2 | 123 | endothelin-converting enzyme | 576 | 1.95E-158 | 56.95% | 17 | P:catabolic process; P:anatomical structure morphogenesis; P:embryo development; P:multicellular organismal development; C:cell; C:cytoplasmic membrane-bounded vesicle; P:cell death; P:signal transduction; P:metabolic process; P:regulation of biological process; P:protein metabolic process; P:response to stress; C:plasma membrane; C:endosome; C:cytoplasm; F:protein binding; F:peptidase activity | IPR000718 (PANTHER); IPR008753 (PFAM); IPR018497 (PRINTS); IPR024079 (G3DSA:3.40.390.GENE3D); SSF55486 (SUPERFAMILY) | |
| 1986 | lib_dib_Filter15_IDBA_contig_33732_minus2 | 66 | von willebrand factor d and egf domain-containing protein | 324 | 1.49E-25 | 45.20% | 10 | F:calcium ion binding; F:carbohydrate binding; P:homophilic cell adhesion; C:membrane; F:chitin binding; P:chitin metabolic process; C:extracellular region; F:polysaccharide binding; F:scavenger receptor activity; P:immune response | G3DSA:2.10.25.10 (GENE3D), tmhmm (TMHMM) | |
| 1987 | lib_dib_Filter15_IDBA_contig_33746_plus2 | 73 | plexin a cg11081-pa | 388 | 2.30E-131 | 57.60% | 3 | C:cell; F:receptor activity; P:multicellular organismal development | IPR002165 (PFAM); IPR003659 (SMART); IPR013783 (G3DSA:2.60.40.GENE3D); IPR014756 (SUPERFAMILY); IPR016201 (SUPERFAMILY); G3DSA:3.30.1680.10 (GENE3D), PTHR22625 (PANTHER) | |
| 1988 | lib_dib_Filter15_IDBA_contig_33751_minus1 | 537 | protein gly- isoform b | 899 | 2.11E-150 | 56.60% | 4 | P:cellular protein modification process; P:biosynthetic process; P:carbohydrate metabolic process; F:transferase activity | IPR000772 (PFAM); IPR001173 (PFAM); IPR003859 (PFAM); G3DSA:2.80.10.50 (GENE3D), G3DSA:3.90.550.10 (GENE3D), PTHR11675 (PANTHER), tmhmm (TMHMM), SSF53448 (SUPERFAMILY) | |
| 1989 | lib_dib_Filter15_IDBA_contig_33752_minus1 | 268 | n-acetylgalactosaminyltransferase 7 | 436 | 1.90E-19 | 66.25% | 4 | F:transferase activity; C:Golgi apparatus; P:biosynthetic process; P:carbohydrate metabolic process | IPR001173 (PFAM); G3DSA:3.90.550.10 (GENE3D), PTHR11675 (PANTHER), tmhmm (TMHMM), SSF53448 (SUPERFAMILY) | |
| 1990 | lib_dib_Filter15_IDBA_contig_33798_plus2 | 72 | multiple inositol polyphosphate phosphatase 1-like | 300 | 5.80E-36 | 50.00% | 1 | F:hydrolase activity | IPR000560 (PFAM); G3DSA:3.40.50.1240 (GENE3D), PTHR20963 (PANTHER), PTHR20963:SF8 (PANTHER), SignalP-NN(euk) (SIGNALP), SSF53254 (SUPERFAMILY) | Yes |

| | | | | | | | | | | |
|---|---|---|---|---|---|---|---|---|---|---|
| 1991 | lib_dib_Filter15_IDBA_contig_33816_minus2 | 508 | indian hedgehog | 478 | 7.72E-29 | 55.70% | 16 | P:signal transduction; P:metabolic process; P:cell differentiation; P:anatomical structure morphogenesis; P:regulation of biological process; P:multicellular organismal development; P:cell proliferation; P:biosynthetic process; P:embryo development; P:cellular component organization; P:growth; P:cell communication; F:protein binding; P:protein metabolic process; P:catabolic process; F:peptidase activity | IPR001657 (PRINTS); IPR001767 (PFAM); IPR003586 (SMART); IPR003587 (SMART); G3DSA:2.170.16.10 (GENE3D), PTHR11889 (PANTHER), SignalP-NN(euk) (SIGNALP), tmhmm (TMHMM), SSF51294 (SUPERFAMILY) | |
| 1992 | lib_dib_Filter15_IDBA_contig_33906_plus2 | 31 | kelch-like protein 12 | 299 | 4.83E-140 | 80.90% | 8 | P:signal transduction; C:intracellular; C:protein complex; P:cellular protein modification process; C:cytoplasmic membrane-bounded vesicle; P:transport; P:organelle organization; F:protein binding | IPR000210 (SMART); IPR006652 (PFAM); IPR011333 (G3DSA:3.30.710.GENE3D); IPR011705 (PFAM); IPR013069 (PFAM); IPR015915 (G3DSA:2.120.10.GENE3D); PTHR24412 (PANTHER), PTHR24412:SF45 (PANTHER), SSF117281 (SUPERFAMILY) | |
| 1993 | lib_dib_Filter15_IDBA_contig_33909_minus1 | 81 | protein | 405 | 2.83E-12 | 53.29% | 5 | C:outer membrane; P:pathogenesis; F:molecular_function; C:apicoplast; P:biological_process | SignalP-NN(euk) (SIGNALP) | Yes |
| 1994 | lib_dib_Filter15_IDBA_contig_33909_minus2 | 81 | cell surface protein | 404 | 3.33E-10 | 65.44% | 3 | F:molecular_function; P:biological_process; C:cellular_component | PTHR11064 (PANTHER), PTHR11064:SF11 (PANTHER), tmhmm (TMHMM) | |
| 1995 | lib_dib_Filter15_IDBA_contig_33909_plus2 | 81 | hypothetical protein BRAFLDRAFT_218575 | 404 | 2.84E-10 | 57.00% | 0 | - | no IPS match | |
| 1996 | lib_dib_Filter15_IDBA_contig_33910_minus1 | 107 | protein | 424 | 3.16E-16 | 49.76% | 5 | C:outer membrane; P:pathogenesis; F:molecular_function; C:apicoplast; P:biological_process | SignalP-NN(euk) (SIGNALP), tmhmm (TMHMM) | Yes |
| 1997 | lib_dib_Filter15_IDBA_contig_33910_minus2 | 107 | cell surface protein | 424 | 2.93E-10 | 65.44% | 3 | F:molecular_function; P:biological_process; C:cellular_component | PTHR11064 (PANTHER), PTHR11064:SF11 (PANTHER), tmhmm (TMHMM) | |
| 1998 | lib_dib_Filter15_IDBA_contig_33910_plus3 | 107 | hypothetical protein BRAFLDRAFT_218575 | 423 | 3.03E-10 | 57.33% | 0 | - | no IPS match | |
| 1999 | lib_dib_Filter15_IDBA_contig_33920_plus3 | 10495 | serine threonine-protein kinase pim-1 | 1424 | 5.24E-119 | 81.35% | 14 | F:binding; P:regulation of biological process; P:cell death; P:cell proliferation; C:cytoplasm; P:cellular protein modification process; ; F:protein kinase activity; P:cell cycle; P:metabolic process; C:plasma membrane; F:protein binding; C:nucleus; F:nucleotide binding | IPR000719 (PFAM); IPR002290 (SMART); IPR008271 (PROSITE); IPR011009 (SUPERFAMILY); IPR017441 (PROSITE); IPR020635 (SMART); G3DSA:1.10.510.10 (GENE3D), G3DSA:3.30.200.20 (GENE3D), PTHR22984 (PANTHER), PTHR22984:SF0 (PANTHER), tmhmm (TMHMM) | |
| 2000 | lib_dib_Filter15_IDBA_contig_33945_minus2 | 52 | multiple epidermal growth factor-like domains protein 6-like | 298 | 6.97E-95 | 63.25% | 2 | F:protein binding; F:binding | IPR000742 (SMART); IPR002049 (PFAM); IPR007527 (PROFILE); IPR013032 (PFAM); PR00011 (PRINTS), G3DSA:2.170.300.10 (GENE3D), PTHR24035 (PANTHER), PTHR24035:SF4 (PANTHER), SSF57196 (SUPERFAMILY) | |
| 2001 | lib_dib_Filter15_IDBA_contig_33947_minus3 | 96 | ankyrin unc44 | 433 | 9.74E-52 | 50.70% | 10 | F:RNA binding; P:RNA-dependent DNA replication; F:RNA-directed DNA polymerase activity; F:endonuclease activity; C:integral to membrane; F:G-protein coupled receptor activity; P:signal transduction; F:receptor activity; F:signal transducer activity; P:G-protein coupled receptor signaling pathway | PTHR21301 (PANTHER) | |

| | | | | | | | | | |
|---|---|---|---|---|---|---|---|---|---|
| 2002 | lib_dib_Filter15_IDBA_contig_33948_minus3 | 110 | ankyrin unc44 | 401 | 2.04E-51 | 50.70% | 13 | F:RNA binding; P:RNA-dependent DNA replication; F:RNA-directed DNA polymerase activity; F:endonuclease activity; C:integral to membrane; F:G-protein coupled receptor activity; P:signal transduction; F:receptor activity; F:signal transducer activity; P:G-protein coupled receptor signaling pathway; P:proteolysis; F:serine-type endopeptidase activity; F:catalytic activity | PTHR21301 (PANTHER) |
| 2003 | lib_dib_Filter15_IDBA_contig_33949_minus3 | 349 | protein twisted gastrulation | 653 | 7.96E-80 | 62.20% | 0 | - | IPR006761 (PANTHER); SignalP-NN(euk) (SIGNALP), tmhmm (TMHMM) |
| 2004 | lib_dib_Filter15_IDBA_contig_34027_plus3 | 228 | neuronal acetylcholine receptor subunit alpha-7-like | 297 | 1.21E-58 | 59.75% | 3 | F:ion channel activity; P:ion transport; C:cell | IPR002394 (PRINTS); IPR006201 (PRINTS); IPR006202 (G3DSA:2.70.170.GENE3D); IPR018000 (PROSITE); PTHR18945:SF66 (PANTHER), tmhmm (TMHMM) |
| 2005 | lib_dib_Filter15_IDBA_contig_34034_plus3 | 18 | neurotransmitter-gated ion-channel ligand binding domain protein | 123 | 1.98E-27 | 62.75% | 3 | P:ion transport; C:cell; F:ion channel activity | IPR006201 (PANTHER); IPR006202 (G3DSA:2.70.170.GENE3D); IPR018000 (PROSITE); PTHR18945:SF92 (PANTHER) |
| 2006 | lib_dib_Filter15_IDBA_contig_34035_plus3 | 46 | neuronal acetylcholine receptor subunit alpha-7-like | 160 | 3.16E-34 | 60.80% | 3 | P:ion transport; C:cell; F:ion channel activity | IPR006201 (PANTHER); IPR006202 (G3DSA:2.70.170.GENE3D); IPR018000 (PROSITE); PTHR18945:SF71 (PANTHER), tmhmm (TMHMM) |
| 2007 | lib_dib_Filter15_IDBA_contig_34060_minus3 | 8398 | protein | 182 | 6.58E-15 | 46.50% | 1 | F:protein binding | IPR002350 (PFAM); G3DSA:3.30.60.30 (GENE3D), PTHR10913 (PANTHER), PTHR10913:SF21 (PANTHER), SSF100895 (SUPERFAMILY) |
| 2008 | lib_dib_Filter15_IDBA_contig_34065_minus3 | 45 | variable lymphocyte receptor | 297 | 7.06E-05 | 51.10% | 1 | F:protein binding | IPR000372 (PFAM); IPR001611 (PROFILE); IPR003591 (SMART); G3DSA:3.80.10.10 (GENE3D), PTHR24365 (PANTHER), PF13855 (PFAM), SSF52058 (SUPERFAMILY) |
| 2009 | lib_dib_Filter15_IDBA_contig_34073_minus2 | 51 | receptor-type tyrosine-protein phosphatase o isoform 2 | 297 | 3.56E-49 | 69.35% | 14 | P:regulation of biological process; P:signal transduction; C:plasma membrane; P:cellular component organization; C:cell; F:receptor activity; F:phosphoprotein phosphatase activity; P:biological_process; P:cell proliferation; P:cell differentiation; P:multicellular organismal development; P:behavior; P:response to external stimulus; F:protein binding | IPR000242 (PRINTS); IPR000387 (PROFILE); IPR003595 (SMART); IPR016130 (PROSITE); G3DSA:3.90.190.10 (GENE3D), PTHR19134 (PANTHER), SSF52799 (SUPERFAMILY) |
| 2010 | lib_dib_Filter15_IDBA_contig_34093_plus3 | 53 | para-nitrobenzyl esterase-like | 297 | 5.33E-62 | 58.30% | 1 | F:carboxylic ester hydrolase activity | IPR002018 (PFAM); IPR019819 (PROSITE); G3DSA:3.40.50.1820 (GENE3D), PTHR11559 (PANTHER), SignalP-NN(euk) (SIGNALP), tmhmm (TMHMM), SSF53474 (SUPERFAMILY) |
| 2011 | lib_dib_Filter15_IDBA_contig_34169_plus2 | 1831 | pro-neuregulin- membrane-bound isoform-like | 615 | 3.04E-15 | 49.00% | 3 | P:multicellular organismal development; P:biological_process; F:protein binding | IPR000742 (SMART); IPR003598 (SMART); IPR003599 (SMART); IPR007110 (PROFILE); IPR013032 (PROSITE); IPR013098 (PFAM); IPR013783 (G3DSA:2.60.40.GENE3D); G3DSA:2.10.25.10 (GENE3D), PTHR11100 (PANTHER), PTHR11100:SF10 (PANTHER), tmhmm (TMHMM), SSF48726 (SUPERFAMILY), SSF57196 (SUPERFAMILY) |
| 2012 | lib_dib_Filter15_IDBA_contig_34188_plus1 | 1172 | glutathione peroxidase | 297 | 3.48E-54 | 64.40% | 4 | F:catalytic activity; F:antioxidant activity; P:response to stress; P:metabolic process | IPR000889 (PANTHER); IPR012336 (G3DSA:3.40.30.GENE3D); PTHR11592:SF0 (PANTHER), SignalP-NN(euk) (SIGNALP), tmhmm (TMHMM) |
| 2013 | lib_dib_Filter15_IDBA_contig_34216_minus1 | 133 | bola-like protein 1-like | 376 | 9.54E-39 | 76.85% | 1 | F:catalytic activity | IPR002634 (G3DSA:3.30.300.GENE3D); PTHR12735 (PANTHER) |

| | | | | | | | | | |
|---|---|---|---|---|---|---|---|---|---|
| 2014 | lib_dib_Filter15_IDBA_contig_34270_plus1 | 50 | dimethylaniline monooxygenase | 296 | 2.50E-65 | 56.00% | 7 | P:nucleobase-containing compound metabolic process; P:secondary metabolic process; C:cell; C:endoplasmic reticulum; F:catalytic activity; P:metabolic process; F:nucleotide binding | IPR000960 (PRINTS); IPR020946 (PFAM); G3DSA:3.50.50.60 (GENE3D), PTHR23023 (PANTHER), PTHR23023:SF4 (PANTHER), SSF51905 (SUPERFAMILY) |
| 2015 | lib_dib_Filter15_IDBA_contig_34402_minus1 | 42 | matrix metallopeptidase 9 (gelatinase 92kda 92kda type iv collagenase) | 295 | 7.29E-22 | 41.15% | 7 | P:biological_process; F:protein binding; P:multicellular organismal development; P:regulation of biological process; F:hydrolase activity; P:response to external stimulus; P:response to stress | IPR000562 (G3DSA:2.10.10.GENE3D); IPR013806 (SUPERFAMILY); PTHR22918 (PANTHER), PTHR22918:SF0 (PANTHER) |
| 2016 | lib_dib_Filter15_IDBA_contig_34416_plus3 | 11497 | angiopoietin-related protein 1 | 709 | 1.38E-35 | 57.15% | 1 | C:extracellular space | IPR002181 (PFAM); IPR014715 (G3DSA:4.10.530.GENE3D); IPR014716 (G3DSA:3.90.215.GENE3D); PTHR19143 (PANTHER), tmhmm (TMHMM) |
| 2017 | lib_dib_Filter15_IDBA_contig_34423_minus3 | 202 | epoxide hydrolase 4 | 416 | 1.83E-105 | 60.20% | 1 | F:catalytic activity | IPR000073 (PRINTS); IPR000639 (PRINTS); G3DSA:3.40.50.1820 (GENE3D), PTHR10992 (PANTHER), PTHR10992:SF98 (PANTHER), PF12697 (PFAM), SignalP-NN(euk) (SIGNALP), tmhmm (TMHMM), SSF53474 (SUPERFAMILY) |
| 2018 | lib_dib_Filter15_IDBA_contig_34424_minus2 | 30 | epoxide hydrolase 4-like | 128 | 2.43E-30 | 73.55% | 2 | P:metabolic process; F:catalytic activity | G3DSA:3.40.50.1820 (GENE3D), PTHR10992 (PANTHER), PTHR10992:SF98 (PANTHER), SignalP-NN(euk) (SIGNALP), tmhmm (TMHMM), SSF53474 (SUPERFAMILY) |
| 2019 | lib_dib_Filter15_IDBA_contig_34431_minus2 | 254 | ---NA--- | 295 | | | 0 | - | no IPS match |
| 2020 | lib_dib_Filter15_IDBA_contig_34440_minus2 | 187 | neurofilament heavy polypeptide | 612 | 1.19E-19 | 50.25% | 18 | F:chitin binding; P:chitin metabolic process; C:extracellular region; C:intermediate filament; C:nucleosome; P:nucleosome assembly; F:DNA binding; F:hydrolase activity, acting on carbon-nitrogen (but not peptide) bonds; P:carbohydrate metabolic process; F:catalytic activity; F:polysaccharide binding; F:scavenger receptor activity; P:immune response; F:beta-N-acetylhexosaminidase activity; F:hydrolase activity; P:metabolic process; F:hydrolase activity, acting on glycosyl bonds; C:membrane | SignalP-NN(euk) (SIGNALP) |
| 2021 | lib_dib_Filter15_IDBA_contig_34441_minus1 | 785 | deleted in malignant brain tumors 1 | 331 | 2.31E-58 | 55.65% | 2 | F:receptor activity; C:cell | IPR001190 (PRINTS); IPR017448 (SMART); G3DSA:3.10.250.10 (GENE3D), PTHR19331 (PANTHER), SignalP-NN(euk) (SIGNALP), tmhmm (TMHMM) |
| 2022 | lib_dib_Filter15_IDBA_contig_34480_minus2 | 31 | zinc finger protein | 294 | 1.17E-33 | 45.30% | 3 | F:binding; F:nucleic acid binding; C:intracellular | IPR007087 (PFAM); IPR013087 (G3DSA:3.30.160.GENE3D); IPR015880 (SMART); PTHR24375 (PANTHER), PTHR24375:SF0 (PANTHER), PF12874 (PFAM), PF13465 (PFAM), PF13894 (PFAM), PF13912 (PFAM), SSF57667 (SUPERFAMILY) |

| | | | | | | | | | | |
|---|---|---|---|---|---|---|---|---|---|---|
| 2023 | lib_dib_Filter15_IDBA_contig_34498_minus3 | 50 | transforming growth factor beta receptor type 3 | 294 | 6.79E-25 | 41.30% | 13 | P:signal transduction; F:receptor activity; P:embryo development; P:biological_process; P:anatomical structure morphogenesis; P:cellular component organization; P:cell differentiation; P:multicellular organismal development; C:plasma membrane; C:cell; P:metabolic process; F:protein binding; P:regulation of biological process | IPR001507 (PFAM); PTHR14002 (PANTHER) | |
| 2024 | lib_dib_Filter15_IDBA_contig_34521_plus2 | 1009 | limbic system-associated membrane protein | 774 | 4.64E-73 | 45.50% | 3 | P:biological_process; P:behavior; F:protein binding | IPR003598 (SMART); IPR003599 (SMART); IPR003961 (SMART); IPR007110 (PROFILE); IPR013098 (PFAM); IPR013783 (G3DSA:2.60.40.GENE3D); PTHR19831 (PANTHER), PTHR19831:SF16 (PANTHER), PF13895 (PFAM), SignalP-NN(euk) (SIGNALP), tmhmm (TMHMM), SSF48726 (SUPERFAMILY) | |
| 2025 | lib_dib_Filter15_IDBA_contig_34527_minus1 | 1712 | isoform k | 634 | 0 | 54.90% | 11 | P:cell cycle; P:anatomical structure morphogenesis; P:organelle organization; P:biological_process; P:cell differentiation; P:multicellular organismal development; C:organelle; C:intracellular; F:structural molecule activity; C:cytoplasm; F:protein binding | IPR003598 (SMART); IPR003599 (SMART); IPR007110 (PROFILE); IPR013098 (PFAM); IPR013783 (G3DSA:2.60.40.GENE3D); SSF48726 (SUPERFAMILY) | |
| 2026 | lib_dib_Filter15_IDBA_contig_34541_minus1 | 82 | pregnancy zone protein | 157 | 5.86E-21 | 57.00% | 1 | F:enzyme regulator activity | IPR001599 (PFAM); PTHR11412 (PANTHER) | |
| 2027 | lib_dib_Filter15_IDBA_contig_34542_minus1 | 548 | alpha-2-macroglobulin-like protein 1- partial | 872 | 1.01E-117 | 52.30% | 3 | P:biological_process; F:enzyme regulator activity; C:extracellular space | IPR001599 (PFAM); IPR008930 (SUPERFAMILY); IPR009048 (G3DSA:2.60.40.GENE3D); IPR011626 (PFAM); IPR019565 (PFAM); IPR019742 (PROSITE); G3DSA:1.50.10.20 (GENE3D), PTHR11412 (PANTHER) | |
| 2028 | lib_dib_Filter15_IDBA_contig_34543_minus2 | 1895 | wd repeat-containing protein 53 | 653 | 1.63E-93 | 56.40% | 1 | F:protein binding | IPR001680 (PFAM); IPR015943 (G3DSA:2.130.10.GENE3D); IPR017986 (PROFILE); IPR019775 (PROSITE); PTHR22847 (PANTHER), PTHR22847:SF44 (PANTHER), SignalP-NN(euk) (SIGNALP), tmhmm (TMHMM) | |
| 2029 | lib_dib_Filter15_IDBA_contig_34550_minus1 | 496 | iduronate 2-sulfatase-like | 822 | 0 | 64.35% | 2 | P:metabolic process; F:hydrolase activity | IPR000917 (PFAM); IPR017849 (G3DSA:3.40.720.GENE3D); IPR017850 (SUPERFAMILY); G3DSA:3.30.1120.10 (GENE3D), PTHR10342 (PANTHER), PTHR10342:SF13 (PANTHER) | |
| 2030 | lib_dib_Filter15_IDBA_contig_34565_minus2 | 24 | abhydrolase domain-containing protein 13 | 293 | 2.71E-110 | 71.35% | 6 | F:hydrolase activity; F:molecular_function; C:integral to membrane; C:membrane; P:biological_process; C:cellular_component | G3DSA:3.40.50.1820 (GENE3D), PTHR12277 (PANTHER), PF12695 (PFAM), SignalP-NN(euk) (SIGNALP), SSF53474 (SUPERFAMILY) | Yes |
| 2031 | lib_dib_Filter15_IDBA_contig_34574_plus2 | 65 | neurogenic locus notch | 293 | 6.81E-101 | 67.95% | 3 | P:biological_process; F:protein binding; F:calcium ion binding | IPR000152 (PROSITE); IPR000742 (PFAM); IPR001881 (SMART); IPR013032 (PROSITE); IPR018097 (PROSITE); PR00010 (PRINTS), G3DSA:2.10.25.10 (GENE3D), G3DSA:2.170.300.10 (GENE3D), PTHR24044 (PANTHER), PTHR24044:SF0 (PANTHER), SSF57196 (SUPERFAMILY) | |
| 2032 | lib_dib_Filter15_IDBA_contig_34611_plus3 | 40 | low quality protein: zinc finger protein 434 | 293 | 6.79E-21 | 64.75% | 4 | F:nucleic acid binding; C:intracellular; F:protein binding; F:binding | IPR007087 (PROSITE); IPR013087 (G3DSA:3.30.160.GENE3D); IPR015880 (SMART); PTHR11389 (PANTHER), PF13465 (PFAM), SSF57667 (SUPERFAMILY) | |
| 2033 | lib_dib_Filter15_IDBA_contig_34633_minus1 | 105 | upf0378 protein kiaa0100-like | 552 | 1.27E-50 | 62.65% | 0 | - | IPR019443 (PFAM); PTHR15678 (PANTHER) | |

| | | | | | | | | | | |
|---|---|---|---|---|---|---|---|---|---|---|
| 2034 | lib_dib_Filter15_IDBA_contig_34666_minus3 | 102 | adp-ribosylation factor 6 | 292 | 9.08E-123 | 97.70% | 22 | F:nucleotide binding; P:regulation of biological process; P:cytoskeleton organization; P:cell death; P:transport; P:cellular component organization; C:endosome; P:multicellular organismal development; P:anatomical structure morphogenesis; P:embryo development; P:biological_process; C:plasma membrane; P:signal transduction; P:response to stress; C:Golgi apparatus; F:protein binding; F:hydrolase activity; P:nucleobase-containing compound metabolic process; P:catabolic process; C:cell; C:cytoplasm; P:protein transport | IPR003579 (SMART); IPR005225 (TIGRFAMs); IPR006687 (SMART); IPR006689 (PRINTS); IPR024156 (SMART); G3DSA:3.40.50.300 (GENE3D), PTHR11711 (PANTHER), SSF52540 (SUPERFAMILY) | |
| 2035 | lib_dib_Filter15_IDBA_contig_34678_plus2 | 81 | tyrosine-protein phosphatase 10d | 389 | 1.05E-41 | 44.60% | 3 | P:biological_process; F:phosphoprotein phosphatase activity; F:protein binding | IPR003961 (PFAM); IPR013783 (G3DSA:2.60.40.GENE3D); PTHR19143 (PANTHER) | |
| 2036 | lib_dib_Filter15_IDBA_contig_34701_minus3 | 143 | cathepsin c | 500 | 2.37E-162 | 68.50% | 6 | C:lysosome; F:binding; F:peptidase activity; P:biological_process; P:protein metabolic process; P:catabolic process | IPR000169 (PROSITE); IPR000668 (PRINTS); IPR013128 (PANTHER); IPR014882 (PFAM); IPR025661 (PROSITE); G3DSA:2.40.128.80 (GENE3D), G3DSA:3.90.70.10 (GENE3D), PTHR12411:SF17 (PANTHER), SignalP-NN(euk) (SIGNALP), tmhmm (TMHMM), SSF54001 (SUPERFAMILY), SSF75001 (SUPERFAMILY) | Yes |
| 2037 | lib_dib_Filter15_IDBA_contig_34756_minus1 | 232 | adam 17-like protease-like | 654 | 0 | 64.90% | 4 | F:binding; F:peptidase activity; P:protein metabolic process; P:catabolic process | IPR001590 (PROFILE); IPR001762 (G3DSA:4.10.70.GENE3D); IPR024079 (G3DSA:3.40.390.GENE3D); PTHR11905 (PANTHER), PTHR11905:SF3 (PANTHER), PF13688 (PFAM), SSF55486 (SUPERFAMILY) | |
| 2038 | lib_dib_Filter15_IDBA_contig_34757_minus1 | 54 | adam 17-like protease-like | 241 | 4.70E-67 | 64.55% | 4 | F:binding; F:peptidase activity; P:protein metabolic process; P:catabolic process | IPR001590 (PROFILE); IPR024079 (G3DSA:3.40.390.GENE3D); PTHR11905 (PANTHER), PTHR11905:SF3 (PANTHER), PF13688 (PFAM), SSF55486 (SUPERFAMILY) | |
| 2039 | lib_dib_Filter15_IDBA_contig_34758_minus3 | 53 | adam 17-like protease | 178 | 5.96E-08 | 49.65% | 16 | P:regulation of biological process; F:receptor binding; P:protein metabolic process; P:catabolic process; C:plasma membrane; P:biological_process; P:behavior; P:response to external stimulus; F:protein binding; C:intracellular; P:metabolic process; P:signal transduction; P:response to stress; P:response to endogenous stimulus; F:peptidase activity; F:binding | IPR002870 (PFAM); PTHR11905 (PANTHER), PTHR11905:SF3 (PANTHER) | |
| 2040 | lib_dib_Filter15_IDBA_contig_34783_plus3 | 27 | ankyrin repeat domain-containing protein 50-like | 291 | 1.36E-48 | 54.90% | 1 | F:protein binding | IPR002110 (PRINTS); IPR020683 (G3DSA:1.25.40.GENE3D); PTHR24198 (PANTHER) | |
| 2041 | lib_dib_Filter15_IDBA_contig_34804_minus1 | 202 | b-cell lymphoma 3 protein | 606 | 4.04E-57 | 63.30% | 18 | P:regulation of biological process; ; P:biological_process; P:biosynthetic process; P:translation; P:multicellular organismal development; C:intracellular; C:protein complex; F:protein binding; P:signal transduction; P:response to stress; P:cell death; P:response to abiotic stimulus; P:protein transport; C:cytoplasm; P:cell differentiation; P:response to biotic stimulus; C:nucleus | IPR002110 (PRINTS); IPR020683 (G3DSA:1.25.40.GENE3D); PTHR24118 (PANTHER), PTHR24118:SF8 (PANTHER) | |

| | | | | | | | | | | |
|---|---|---|---|---|---|---|---|---|---|---|
| 2042 | lib_dib_Filter15_IDBA_contig_34846_plus1 | 119 | epidermis-type lipoxygenase 3-like | 291 | 7.75E-60 | 62.15% | 4 | F:binding; F:catalytic activity; P:lipid metabolic process; P:metabolic process | IPR000907 (PANTHER); IPR013819 (PFAM); G3DSA:1.20.245.10 (GENE3D) | |
| 2043 | lib_dib_Filter15_IDBA_contig_34887_plus1 | 37 | matrix metalloproteinase 9 | 291 | 4.61E-16 | 52.30% | 15 | P:anatomical structure morphogenesis; P:multicellular organismal development; P:response to endogenous stimulus; F:protein binding; P:biological_process; P:response to external stimulus; P:response to biotic stimulus; P:regulation of biological process; P:cell communication; P:response to stress; P:response to abiotic stimulus; F:peptidase activity; P:metabolic process; C:extracellular region; P:reproduction | IPR000562 (G3DSA:2.10.10.GENE3D); IPR003014 (PFAM); IPR003609 (PROFILE); IPR013806 (SUPERFAMILY); PR00013 (PRINTS), PTHR22918 (PANTHER), PTHR22918:SF0 (PANTHER) | |
| 2044 | lib_dib_Filter15_IDBA_contig_34982_plus1 | 57 | leucine-rich repeats and immunoglobulin-like domains protein 2 | 290 | 1.06E-13 | 46.90% | 1 | F:protein binding | IPR000483 (SMART); IPR001611 (PFAM); IPR003591 (SMART); PR00019 (PRINTS), G3DSA:3.80.10.10 (GENE3D), PTHR24373 (PANTHER), PF13855 (PFAM), SSF52058 (SUPERFAMILY) | |
| 2045 | lib_dib_Filter15_IDBA_contig_35003_plus1 | 28 | nicotinic acetylcholine receptor subunit alpha 6 transcript variant partial | 290 | 5.22E-81 | 67.35% | 3 | F:ion channel activity; P:ion transport; C:cell | IPR002394 (PRINTS); IPR006029 (SUPERFAMILY); IPR006201 (PRINTS); IPR006202 (G3DSA:2.70.170.GENE3D); IPR018000 (PROSITE); G3DSA:1.20.120.370 (GENE3D), PTHR18945:SF92 (PANTHER), tmhmm (TMHMM) | |
| 2046 | lib_dib_Filter15_IDBA_contig_35010_plus2 | 290 | aminoacylase-1 isoform 1 | 725 | 0 | 75.30% | 6 | P:protein metabolic process; P:catabolic process; F:peptidase activity; F:hydrolase activity; C:cytoplasm; | IPR001261 (PROSITE); IPR002933 (PFAM); IPR010159 (TIGRFAMs); IPR011650 (PFAM); G3DSA:3.30.70.360 (GENE3D), G3DSA:3.40.630.10 (GENE3D), PTHR11014 (PANTHER), PTHR11014:SF8 (PANTHER), SSF53187 (SUPERFAMILY) | |
| 2047 | lib_dib_Filter15_IDBA_contig_35011_plus1 | 314 | aminoacylase-1 isoform 1 | 737 | 0 | 75.30% | 6 | P:protein metabolic process; P:catabolic process; F:peptidase activity; F:hydrolase activity; C:cytoplasm; | IPR001261 (PROSITE); IPR002933 (PFAM); IPR010159 (TIGRFAMs); IPR011650 (PFAM); G3DSA:3.30.70.360 (GENE3D), G3DSA:3.40.630.10 (GENE3D), PTHR11014 (PANTHER), PTHR11014:SF8 (PANTHER), SignalP-NN(euk) (SIGNALP), SSF53187 (SUPERFAMILY) | |
| 2048 | lib_dib_Filter15_IDBA_contig_35014_plus2 | 525 | transforming growth factor-beta-induced protein ig-h3 | 313 | 1.00E-40 | 58.15% | 1 | P:biological_process | IPR000782 (G3DSA:2.30.180.GENE3D); PTHR10900 (PANTHER), SignalP-NN(euk) (SIGNALP) | |
| 2049 | lib_dib_Filter15_IDBA_contig_35015_plus1 | 175 | fasciclin-like protein | 126 | 1.49E-11 | 51.80% | 6 | P:signal transduction; F:carbohydrate binding; P:multicellular organismal development; P:biological_process; C:proteinaceous extracellular matrix; P:cellular component organization | IPR000782 (G3DSA:2.30.180.GENE3D); PTHR10900 (PANTHER) | |
| 2050 | lib_dib_Filter15_IDBA_contig_35016_plus3 | 283 | transforming growth factor-beta-induced protein ig-h3 | 151 | 1.69E-19 | 55.00% | 7 | C:extracellular space; F:binding; P:biological_process; P:cell differentiation; P:multicellular organismal development; P:cellular component organization; C:proteinaceous extracellular matrix | IPR000782 (G3DSA:2.30.180.GENE3D); PTHR10900 (PANTHER) | |
| 2051 | lib_dib_Filter15_IDBA_contig_35054_plus2 | 139 | membrane-associated progesterone receptor component 1 | 557 | 7.41E-66 | 78.70% | 6 | F:binding; C:cell; F:catalytic activity; F:receptor activity; F:protein binding; F:lipid binding | IPR001199 (G3DSA:3.10.120.GENE3D); PTHR10281 (PANTHER), PTHR10281:SF23 (PANTHER), tmhmm (TMHMM) | |
| 2052 | lib_dib_Filter15_IDBA_contig_35151_plus1 | 268 | a chain crystal structure of porcine beta trypsin with % polydocanol | 406 | 5.73E-67 | 59.45% | 3 | F:peptidase activity; P:protein metabolic process; P:catabolic process | IPR000519 (G3DSA:4.10.110.GENE3D); IPR001254 (PFAM); IPR001314 (PRINTS); IPR009003 (SUPERFAMILY); IPR018114 (PROSITE); G3DSA:2.40.10.10 (GENE3D), PTHR24259 (PANTHER), SignalP-NN(euk) (SIGNALP), tmhmm (TMHMM) | Yes |

| | | | | | | | | | | |
|---|---|---|---|---|---|---|---|---|---|---|
| 2053 | lib_dib_Filter15_IDBA_contig_35152_plus1 | 31 | plasma kallikrein | 113 | 1.65E-21 | 76.15% | 10 | F:receptor activity; C:extracellular space; F:peptidase activity; F:carbohydrate binding; P:protein metabolic process; P:catabolic process; C:cell; P:response to external stimulus; P:regulation of biological process; P:response to stress | IPR001254 (PFAM); IPR009003 (SUPERFAMILY); IPR018114 (PROSITE); G3DSA:2.40.10.10 (GENE3D), PTHR24265 (PANTHER) | |
| 2054 | lib_dib_Filter15_IDBA_contig_35179_plus3 | 47 | upf0378 protein kiaa0100-like | 288 | 2.90E-142 | 74.10% | 0 | - | IPR019441 (PFAM); PTHR15678 (PANTHER) | |
| 2055 | lib_dib_Filter15_IDBA_contig_35188_minus2 | 37 | dn cadherin-like protein | 288 | 5.15E-43 | 51.65% | 3 | F:calcium ion binding; P:biological_process; C:cell | IPR002126 (PRINTS); IPR015919 (SUPERFAMILY); PTHR24027 (PANTHER) | |
| 2056 | lib_dib_Filter15_IDBA_contig_35195_minus1 | 101 | ankyrin repeat protein | 427 | 2.20E-27 | 47.75% | 1 | F:protein binding | IPR002110 (SMART); IPR020683 (G3DSA:1.25.40.GENE3D); PTHR24198 (PANTHER), PF13857 (PFAM) | |
| 2057 | lib_dib_Filter15_IDBA_contig_35199_plus3 | 162 | zinc finger protein 37 | 678 | 3.56E-69 | 53.75% | 4 | P:biological_process; F:binding; F:nucleic acid binding; C:intracellular | IPR007087 (PFAM); IPR013087 (G3DSA:3.30.160.GENE3D); IPR015880 (SMART); PTHR24402 (PANTHER), PF13465 (PFAM), PF13894 (PFAM), PF13912 (PFAM), SSF57667 (SUPERFAMILY) | |
| 2058 | lib_dib_Filter15_IDBA_contig_35233_minus1 | 38 | fibroblast growth factor receptor-like 1 | 289 | 1.21E-57 | 58.75% | 4 | P:multicellular organismal development; P:anatomical structure morphogenesis; F:protein binding; P:cell differentiation | IPR003598 (SMART); IPR003599 (SMART); IPR007110 (PROFILE); IPR013098 (PFAM); IPR013783 (G3DSA:2.60.40.GENE3D); PTHR19890 (PANTHER), PTHR19890:SF4 (PANTHER), SignalP-NN(euk) (SIGNALP), SSF48726 (SUPERFAMILY) | Yes |
| 2059 | lib_dib_Filter15_IDBA_contig_35270_minus1 | 141 | aldehyde dehydrogenase 2 family | 502 | 0 | 80.45% | 9 | P:carbohydrate metabolic process; F:catalytic activity; P:metabolic process; P:biosynthetic process; P:cell-cell signaling; F:electron carrier activity; C:mitochondrion; P:catabolic process; C:nucleus | IPR015590 (PFAM); IPR016160 (PROSITE); IPR016161 (SUPERFAMILY); IPR016162 (G3DSA:3.40.605.GENE3D); IPR016163 (G3DSA:3.40.309.GENE3D); PTHR11699 (PANTHER), PTHR11699:SF46 (PANTHER) | |
| 2060 | lib_dib_Filter15_IDBA_contig_35305_plus2 | 116 | 26s proteasome non-atpase regulatory subunit 10-like | 313 | 8.37E-81 | 66.80% | 7 | P:cellular component organization; P:regulation of biological process; P:protein metabolic process; P:signal transduction; C:organelle; C:intracellular; F:protein binding | IPR002110 (PRINTS); IPR020683 (G3DSA:1.25.40.GENE3D); PTHR24199 (PANTHER), PTHR24199:SF0 (PANTHER) | |
| 2061 | lib_dib_Filter15_IDBA_contig_35350_plus1 | 84 | ankyrin unc44 | 315 | 1.40E-59 | 51.95% | 8 | F:RNA binding; P:RNA-dependent DNA replication; F:RNA-directed DNA polymerase activity; F:zinc ion binding; C:intracellular; P:proteolysis; F:serine-type endopeptidase activity; F:catalytic activity | PTHR21301 (PANTHER) | |
| 2062 | lib_dib_Filter15_IDBA_contig_35400_plus3 | 413 | tyrosine-protein kinase yes | 746 | 0 | 70.80% | 9 | P:cellular protein modification process; P:metabolic process; P:regulation of biological process; F:protein kinase activity; C:cytoplasm; P:anatomical structure morphogenesis; P:embryo development; F:nucleotide binding; F:protein binding | IPR000719 (PROFILE); IPR000980 (PRINTS); IPR001245 (PRINTS); IPR001452 (PRINTS); IPR002290 (SMART); IPR008266 (PROSITE); IPR011009 (SUPERFAMILY); IPR017441 (PROSITE); IPR020635 (SMART); G3DSA:1.10.510.10 (GENE3D), G3DSA:2.30.30.40 (GENE3D), G3DSA:3.30.200.20 (GENE3D), PTHR24418 (PANTHER), SSF55550 (SUPERFAMILY) | |
| 2063 | lib_dib_Filter15_IDBA_contig_35408_plus2 | 280 | er degradation-enhancing alpha-mannosidase-like 3 | 868 | 0 | 75.50% | 6 | F:hydrolase activity; C:endoplasmic reticulum; P:catabolic process; P:protein metabolic process; F:calcium ion binding; C:cell | IPR001382 (PRINTS); IPR003137 (PFAM); G3DSA:3.50.30.30 (GENE3D), PTHR11742:SF8 (PANTHER), tmhmm (TMHMM), SSF52025 (SUPERFAMILY) | |
| 2064 | lib_dib_Filter15_IDBA_contig_35409_plus3 | 46 | leucine-rich repeat serine threonine-protein kinase 1-like | 286 | 6.92E-25 | 53.30% | 3 | P:cellular component organization; F:protein kinase activity; F:protein binding | IPR002110 (SMART); IPR020683 (G3DSA:1.25.40.GENE3D); PTHR24198 (PANTHER) | |

| | | | | | | | | | | |
|---|---|---|---|---|---|---|---|---|---|---|
| 2065 | lib_dib_Filter15_IDBA_contig_35413_plus3 | 66 | cdk5 regulatory subunit associated protein 2 | 374 | 1.09E-28 | 52.05% | 6 | P:cell cycle; P:cytoskeleton organization; P:regulation of biological process; C:microtubule organizing center; F:cytoskeletal protein binding; P:multicellular organismal development | PTHR13895 (PANTHER), PTHR13895:SF3 (PANTHER) | |
| 2066 | lib_dib_Filter15_IDBA_contig_35470_plus2 | 120 | low-density lipoprotein receptor-related protein 5 | 286 | 1.78E-15 | 65.55% | 17 | P:anatomical structure morphogenesis; P:multicellular organismal development; P:regulation of biological process; P:response to endogenous stimulus; P:cell-cell signaling; P:cell differentiation; ; P:biosynthetic process; P:lipid metabolic process; P:embryo development; F:protein binding; F:receptor activity; C:mitochondrion; P:signal transduction; C:cell; P:cell death; P:cell proliferation | IPR000033 (PFAM); IPR003582 (PFAM); IPR011042 (G3DSA:2.120.10.GENE3D); PTHR10529 (PANTHER), SSF63825 (SUPERFAMILY) | |
| 2067 | lib_dib_Filter15_IDBA_contig_35471_plus1 | 63 | low-density lipoprotein receptor-related protein 2 | 159 | 4.08E-31 | 49.85% | 16 | P:cell differentiation; P:multicellular organismal development; P:embryo development; F:receptor activity; P:biological_process; P:signal transduction; P:regulation of biological process; P:cellular protein modification process; P:anatomical structure morphogenesis; C:plasma membrane; P:cell proliferation; F:protein binding; C:organelle; C:intracellular; C:cytoplasm; | IPR000033 (PFAM); IPR011042 (G3DSA:2.120.10.GENE3D); PTHR10529 (PANTHER), tmhmm (TMHMM), SSF63825 (SUPERFAMILY) | |
| 2068 | lib_dib_Filter15_IDBA_contig_3552_minus2 | 644 | fibrinogen alpha chain | 356 | 1.71E-26 | 58.55% | 6 | P:biological_process; C:plasma membrane; F:binding; C:extracellular space; C:cytoplasmic membrane-bounded vesicle; F:protein binding | IPR002181 (PFAM); IPR014715 (G3DSA:4.10.530.GENE3D); IPR014716 (G3DSA:3.90.215.GENE3D); PTHR19143 (PANTHER) | |
| 2069 | lib_dib_Filter15_IDBA_contig_35568_minus1 | 81 | scavenger receptor cysteine-rich protein | 286 | 1.86E-46 | 57.75% | 3 | C:cell; F:hydrolase activity; F:receptor activity | IPR000519 (G3DSA:4.10.110.GENE3D); IPR001190 (PRINTS); IPR017448 (SMART); G3DSA:3.10.250.10 (GENE3D), PTHR19331 (PANTHER), PTHR19331:SF118 (PANTHER), SignalP-NN(euk) (SIGNALP) | Yes |
| 2070 | lib_dib_Filter15_IDBA_contig_3558_plus3 | 3211 | hypothetical protein CGI_10002658 | 255 | 6.88E-29 | 56.60% | 2 | F:transporter activity; P:transport | IPR001747 (PFAM); IPR015816 (G3DSA:2.30.230.GENE3D); IPR015819 (SUPERFAMILY); PTHR23345 (PANTHER), tmhmm (TMHMM) | |
| 2071 | lib_dib_Filter15_IDBA_contig_35582_plus1 | 92 | globin x | 323 | 7.71E-26 | 54.55% | 5 | P:response to stress; C:cell; F:oxygen binding; F:binding; P:transport | IPR000971 (PFAM); IPR009050 (SUPERFAMILY); IPR012292 (G3DSA:1.10.490.GENE3D); IPR013316 (PRINTS); PTHR22924 (PANTHER) | |
| 2072 | lib_dib_Filter15_IDBA_contig_35621_minus3 | 110 | ph domain leucine-rich repeat-containing protein phosphatase 1 | 487 | 0 | 57.75% | 4 | F:catalytic activity; P:signal transduction; C:intracellular; F:protein binding | IPR001611 (PFAM); IPR003591 (SMART); PR00019 (PRINTS), G3DSA:3.80.10.10 (GENE3D), PTHR23155 (PANTHER), PTHR23155:SF16 (PANTHER), PF13504 (PFAM), PF13516 (PFAM), PF13855 (PFAM), SM00364 (SMART), SignalP-NN(euk) (SIGNALP), tmhmm (TMHMM), SSF52058 (SUPERFAMILY), SSF52075 (SUPERFAMILY) | |
| 2073 | lib_dib_Filter15_IDBA_contig_35632_minus3 | 134 | ankyrin repeat protein | 400 | 5.43E-24 | 49.45% | 1 | F:protein binding | IPR002110 (SMART); IPR020683 (G3DSA:1.25.40.GENE3D); PTHR24198 (PANTHER), PF13857 (PFAM) | |
| 2074 | lib_dib_Filter15_IDBA_contig_35646_plus1 | 38 | paramyosin | 285 | 1.62E-28 | 75.95% | 4 | C:cytoplasm; C:cytoskeleton; C:protein complex; F:motor activity | IPR002928 (PFAM); PTHR25370 (PANTHER), PTHR25370:SF62 (PANTHER), tmhmm (TMHMM) | |
| 2075 | lib_dib_Filter15_IDBA_contig_35649_minus2 | 73 | proopiomelanocortin b precursor | 285 | 5.30E-149 | 72.15% | 3 | C:extracellular region; P:signal transduction; F:receptor binding | IPR001941 (PRINTS); IPR013531 (PFAM); IPR013532 (PFAM); IPR013593 (PFAM); PTHR11416 (PANTHER), tmhmm (TMHMM) | |

| | | | | | | | | | |
|---|---|---|---|---|---|---|---|---|---|
| 2076 | lib_dib_Filter15_IDBA_contig_35716_plus2 | 222 | isoform a | 650 | 6.47E-71 | 55.70% | 14 | P:response to biotic stimulus; P:multicellular organismal development; P:anatomical structure morphogenesis; P:embryo development; P:signal transduction; P:response to stress; C:extracellular space; P:cellular component organization; P:cell differentiation; P:reproduction; P:behavior; F:binding; F:nucleic acid binding; C:intracellular | IPR007087 (PROSITE); IPR013087 (G3DSA:3.30.160.GENE3D); IPR015880 (SMART); PTHR23233 (PANTHER), PTHR23233:SF22 (PANTHER), PF13465 (PFAM), SSF57667 (SUPERFAMILY) |
| 2077 | lib_dib_Filter15_IDBA_contig_35717_plus3 | 230 | isoform a | 704 | 2.30E-70 | 55.90% | 14 | P:response to biotic stimulus; P:multicellular organismal development; P:anatomical structure morphogenesis; P:embryo development; P:signal transduction; P:response to stress; C:extracellular space; P:cellular component organization; P:cell differentiation; P:reproduction; P:behavior; F:binding; F:nucleic acid binding; C:intracellular | IPR007087 (PROSITE); IPR013087 (G3DSA:3.30.160.GENE3D); IPR015880 (SMART); PTHR23233 (PANTHER), PTHR23233:SF22 (PANTHER), PF13465 (PFAM), SSF57667 (SUPERFAMILY) |
| 2078 | lib_dib_Filter15_IDBA_contig_35721_plus3 | 48 | polyamine-modulated factor 1-binding protein 1 | 284 | 5.03E-78 | 58.29% | 0 | - | PTHR18937 (PANTHER), PTHR18937:SF83 (PANTHER) |
| 2079 | lib_dib_Filter15_IDBA_contig_35735_minus1 | 197 | plexin a | 713 | 1.70E-77 | 48.70% | 7 | F:protein kinase activity; P:cellular protein modification process; F:protein binding; F:receptor activity; P:multicellular organismal development; C:cell; F:nucleotide binding | IPR001245 (PFAM); IPR002909 (PFAM); IPR011009 (SUPERFAMILY); IPR013783 (G3DSA:2.60.40.GENE3D); IPR014756 (SUPERFAMILY); IPR016201 (SUPERFAMILY); IPR017441 (PROSITE); G3DSA:3.30.1680.10 (GENE3D), G3DSA:3.30.200.20 (GENE3D), PTHR22625 (PANTHER), PTHR22625:SF16 (PANTHER), tmhmm (TMHMM) |
| 2080 | lib_dib_Filter15_IDBA_contig_35745_plus3 | 134 | hypothetical protein | 586 | 2.94E-04 | 47.00% | 0 | - | IPR009053 (SUPERFAMILY); SSF46966 (SUPERFAMILY) |
| 2081 | lib_dib_Filter15_IDBA_contig_35754_minus1 | 34 | polypeptide n-acetylgalactosaminyltransferase 11 | 284 | 2.05E-127 | 78.90% | 7 | C:Golgi apparatus; F:transferase activity; P:biosynthetic process; P:cellular protein modification process; P:carbohydrate metabolic process; F:carbohydrate binding; C:cell | IPR001173 (PFAM); G3DSA:3.90.550.10 (GENE3D), PTHR11675 (PANTHER), SSF53448 (SUPERFAMILY) |
| 2082 | lib_dib_Filter15_IDBA_contig_35790_minus1 | 42 | achain crystal structure of the n-terminal kinase domain of human rsk-1 bound to amp-pcp | 284 | 3.61E-162 | 91.80% | 24 | P:anatomical structure morphogenesis; P:cellular component organization; P:cell differentiation; P:multicellular organismal development; P:regulation of biological process; P:cell growth; P:response to stress; P:translation; C:nucleoplasm; P:response to biotic stimulus; P:signal transduction; C:ribosome; P:cell death; F:protein binding; F:enzyme regulator activity; C:cytosol; C:cytoskeleton; P:cellular protein modification process; F:protein kinase activity; F:binding; ; P:cell-cell signaling; F:nucleotide binding; P:cell cycle | IPR000719 (PFAM); IPR000961 (PROFILE); IPR002290 (SMART); IPR008271 (PROSITE); IPR011009 (SUPERFAMILY); IPR017441 (PROSITE); IPR020635 (SMART); G3DSA:1.10.510.10 (GENE3D), G3DSA:3.30.200.20 (GENE3D), PTHR24351 (PANTHER), PTHR24351:SF25 (PANTHER) |
| 2083 | lib_dib_Filter15_IDBA_contig_35812_plus1 | 26 | gastrula zinc finger | 284 | 1.98E-53 | 65.20% | 3 | F:binding; F:nucleic acid binding; C:intracellular | IPR007087 (PFAM); IPR013087 (G3DSA:3.30.160.GENE3D); IPR015880 (SMART); PTHR24402 (PANTHER), PF13465 (PFAM), SSF57667 (SUPERFAMILY) |
| 2084 | lib_dib_Filter15_IDBA_contig_35838_plus1 | 67 | leucine-rich repeat-containing protein 57 | 309 | 5.25E-57 | 73.25% | 1 | F:protein binding | IPR001611 (PROFILE); IPR003591 (SMART); IPR025875 (PFAM); G3DSA:3.80.10.10 (GENE3D), PTHR23155 (PANTHER), PTHR23155:SF26 (PANTHER), tmhmm (TMHMM), SSF52058 (SUPERFAMILY) |

| | | | | | | | | | | |
|---|---|---|---|---|---|---|---|---|---|---|
| 2085 | lib_dib_Filter15_IDBA_contig_35881_plus3 | 157 | bile salt-activated lipase | 649 | 9.33E-79 | 51.00% | 8 | F:binding; C:organelle; C:intracellular; F:hydrolase activity; P:catabolic process; P:lipid metabolic process; C:cytoplasm; C:extracellular region | IPR002018 (PFAM); IPR019819 (PROSITE); IPR019826 (PROSITE); G3DSA:3.40.50.1820 (GENE3D), PTHR11559 (PANTHER), tmhmm (TMHMM), SSF53474 (SUPERFAMILY) | |
| 2086 | lib_dib_Filter15_IDBA_contig_35882_plus1 | 119 | carboxylesterase precursor | 447 | 2.15E-29 | 49.10% | 6 | F:hydrolase activity; F:carboxylesterase activity; C:cellular_component; P:response to acid; F:acylcarnitine hydrolase activity; C:microsome | IPR002018 (PFAM); G3DSA:3.40.50.1820 (GENE3D), PTHR11559 (PANTHER), tmhmm (TMHMM), SSF53474 (SUPERFAMILY) | |
| 2087 | lib_dib_Filter15_IDBA_contig_35939_minus2 | 319 | complement component c6 | 282 | 1.52E-09 | 59.00% | 8 | C:membrane attack complex; P:immune response; P:DNA-dependent transcription, termination; P:carboxylic acid metabolic process; F:catalytic activity; F:carboxy-lyase activity; F:pyridoxal phosphate binding; F:lyase activity | PTHR19376 (PANTHER), PTHR19376:SF30 (PANTHER) | |
| 2088 | lib_dib_Filter15_IDBA_contig_3594_minus2 | 566 | ankyrin repeat protein | 786 | 5.25E-51 | 49.90% | 1 | F:protein binding | IPR001496 (PFAM); IPR002110 (PRINTS); IPR020683 (G3DSA:1.25.40.GENE3D); PTHR24198 (PANTHER), tmhmm (TMHMM) | |
| 2089 | lib_dib_Filter15_IDBA_contig_36025_minus3 | 28 | low quality protein: neuroglian-like | 281 | 5.53E-27 | 44.65% | 1 | F:protein binding | IPR003598 (SMART); IPR007110 (PROFILE); IPR013098 (PFAM); IPR013783 (G3DSA:2.60.40.GENE3D); PTHR10489 (PANTHER), SignalP-NN(euk) (SIGNALP), SSF48726 (SUPERFAMILY) | Yes |
| 2090 | lib_dib_Filter15_IDBA_contig_36038_minus3 | 1234 | peroxisomal -trans-enoyl-isomerase | 729 | 5.32E-150 | 69.65% | 3 | P:metabolic process; F:catalytic activity; F:lipid binding | IPR000582 (PRINTS); IPR001753 (PFAM); IPR014352 (G3DSA:1.20.80.GENE3D); G3DSA:3.90.226.10 (GENE3D), PTHR11941 (PANTHER), SSF52096 (SUPERFAMILY) | |
| 2091 | lib_dib_Filter15_IDBA_contig_36101_plus2 | 50 | low quality protein: espin | 281 | 4.17E-40 | 54.70% | 2 | C:cell; F:protein binding | IPR002110 (SMART); IPR020683 (G3DSA:1.25.40.GENE3D); PTHR24188 (PANTHER) | |
| 2092 | lib_dib_Filter15_IDBA_contig_36122_minus2 | 53 | stress-activated protein kinase jnk | 281 | 1.56E-166 | 93.05% | 5 | F:protein kinase activity; F:signal transducer activity; P:signal transduction; P:cellular protein modification process; F:nucleotide binding | IPR000719 (PFAM); IPR002290 (SMART); IPR008271 (PROSITE); IPR008351 (PRINTS); IPR011009 (SUPERFAMILY); G3DSA:1.10.510.10 (GENE3D), PTHR24055 (PANTHER), PTHR24055:SF105 (PANTHER) | |
| 2093 | lib_dib_Filter15_IDBA_contig_36124_minus1 | 42 | probable g-protein coupled receptor 125-like | 281 | 2.08E-74 | 61.60% | 3 | C:cell; P:signal transduction; F:protein binding | IPR000483 (SMART); IPR001611 (PROFILE); IPR003591 (SMART); IPR003599 (SMART); IPR007110 (PROFILE); IPR013783 (G3DSA:2.60.40.GENE3D); PR00019 (PRINTS), G3DSA:3.80.10.10 (GENE3D), PTHR12011 (PANTHER), PTHR12011:SF35 (PANTHER), PF13855 (PFAM), PF13895 (PFAM), SSF48726 (SUPERFAMILY), SSF52058 (SUPERFAMILY) | |
| 2094 | lib_dib_Filter15_IDBA_contig_36127_plus2 | 222 | serine proteinase stubble-like | 526 | 5.07E-07 | 43.85% | 3 | F:peptidase activity; P:protein metabolic process; P:catabolic process | IPR001254 (PFAM); IPR009003 (SUPERFAMILY); G3DSA:2.40.10.10 (GENE3D), PTHR24278 (PANTHER) | |
| 2095 | lib_dib_Filter15_IDBA_contig_36128_plus2 | 456 | von willebrand factor type egf and pentraxin domain-containing protein 1 | 896 | 3.97E-29 | 44.95% | 5 | F:protein binding; F:peptidase activity; P:protein metabolic process; P:catabolic process; F:calcium ion binding | IPR000152 (PROSITE); IPR000436 (PFAM); IPR000742 (SMART); IPR001254 (PFAM); IPR001881 (SMART); IPR009003 (SUPERFAMILY); IPR013032 (PROSITE); IPR023413 (G3DSA:2.40.155.GENE3D); IPR024731 (PFAM); G3DSA:2.10.70.10 (GENE3D), G3DSA:2.40.10.10 (GENE3D), PTHR19325 (PANTHER), tmhmm (TMHMM), SSF57196 (SUPERFAMILY) | |

| | | | | | | | | | |
|---|---|---|---|---|---|---|---|---|---|
| 2096 | lib_dib_Filter15_IDBA_contig_36169_minus1 | 5055 | von willebrand factor type egf and pentraxin domain-containing protein 1-like | 1005 | 2.59E-114 | 47.65% | 1 | F:calcium ion binding | IPR000152 (PROSITE); IPR000436 (PFAM); IPR000884 (SMART); IPR001881 (PFAM); IPR009030 (SUPERFAMILY); IPR011641 (PFAM); IPR018097 (PROSITE); G3DSA:2.10.25.10 (GENE3D), G3DSA:2.10.70.10 (GENE3D), PTHR19325 (PANTHER), SSF57196 (SUPERFAMILY) |
| 2097 | lib_dib_Filter15_IDBA_contig_36170_minus1 | 5013 | von willebrand factor type egf and pentraxin domain-containing protein 1-like | 1066 | 4.63E-114 | 47.70% | 1 | F:calcium ion binding | IPR000152 (PROSITE); IPR000436 (PFAM); IPR000884 (SMART); IPR001881 (PFAM); IPR009030 (SUPERFAMILY); IPR011641 (PFAM); IPR018097 (PROSITE); G3DSA:2.10.25.10 (GENE3D), G3DSA:2.10.70.10 (GENE3D), PTHR19325 (PANTHER), SSF57196 (SUPERFAMILY) |
| 2098 | lib_dib_Filter15_IDBA_contig_36171_minus1 | 171 | cub and sushi domain-containing protein 1- partial | 128 | 6.40E-20 | 55.85% | 10 | F:carbohydrate binding; F:calcium ion binding; P:homophilic cell adhesion; C:integral to membrane; P:cell adhesion; C:membrane; C:plasma membrane; F:molecular_function; P:biological_process; C:cellular_component | IPR000436 (PFAM); G3DSA:2.10.70.10 (GENE3D), PTHR19325 (PANTHER) |
| 2099 | lib_dib_Filter15_IDBA_contig_36173_minus1 | 480 | cub and sushi domain-containing protein 3 isoform 1 | 278 | 6.46E-31 | 48.85% | 5 | F:carbohydrate binding; P:regulation of transcription, DNA-dependent; F:sequence-specific DNA binding; F:sequence-specific DNA binding transcription factor activity; F:DNA binding | IPR000436 (PFAM); IPR000884 (SMART); IPR011641 (PFAM); G3DSA:2.10.70.10 (GENE3D), G3DSA:2.20.100.10 (GENE3D), PTHR19325 (PANTHER) |
| 2100 | lib_dib_Filter15_IDBA_contig_36194_plus3 | 17 | protein crumbs-like | 116 | 3.69E-17 | 65.70% | 8 | P:cell differentiation; P:regulation of biological process; P:multicellular organismal development; P:anatomical structure morphogenesis; C:proteinaceous extracellular matrix; P:cellular component organization; F:protein binding; F:calcium ion binding | IPR000742 (PFAM); IPR000859 (G3DSA:2.60.120.GENE3D); IPR001881 (SMART); IPR013032 (PROSITE); G3DSA:2.10.25.10 (GENE3D), PTHR24033 (PANTHER), SSF57196 (SUPERFAMILY) |
| 2101 | lib_dib_Filter15_IDBA_contig_36195_plus2 | 34 | protein | 281 | 1.41E-82 | 61.85% | 3 | C:extracellular region; F:protein binding; F:calcium ion binding | IPR000152 (PROSITE); IPR000742 (PFAM); IPR001881 (PFAM); IPR013032 (PFAM); IPR018097 (PROSITE); PR00010 (PRINTS), G3DSA:2.10.25.10 (GENE3D), G3DSA:2.170.300.10 (GENE3D), PTHR24044 (PANTHER), SSF57196 (SUPERFAMILY) |
| 2102 | lib_dib_Filter15_IDBA_contig_36199_minus1 | 32 | zinc finger protein 271-like | 281 | 2.90E-65 | 64.05% | 3 | F:binding; F:nucleic acid binding; C:intracellular | IPR007087 (PROSITE); IPR013087 (G3DSA:3.30.160.GENE3D); IPR015880 (SMART); PTHR24402 (PANTHER), PF12756 (PFAM), PF13465 (PFAM), SSF57667 (SUPERFAMILY) |

| | | | | | | | | | | |
|---|---|---|---|---|---|---|---|---|---|---|
| 2103 | lib_dib_Filter15_IDBA_contig_36222_plus2 | 30 | isoform b | 280 | 1.50E-26 | 54.50% | 25 | P:cell differentiation; P:multicellular organismal development; P:regulation of biological process; F:protein kinase activity; P:anatomical structure morphogenesis; P:cellular component organization; P:embryo development; P:response to external stimulus; P:signal transduction; C:plasma membrane; P:reproduction; P:cell growth; F:receptor activity; P:response to stress; P:transport; P:growth; P:cellular protein modification process; P:biological_process; F:protein binding; P:behavior; P:response to abiotic stimulus; P:cell death; P:cell communication; P:catabolic process; F:nucleotide binding | IPR000719 (PROFILE); IPR001245 (PFAM); IPR002290 (SMART); IPR011009 (SUPERFAMILY); IPR020635 (SMART); G3DSA:1.10.510.10 (GENE3D), PTHR24418 (PANTHER), PTHR24418:SF14 (PANTHER) | |
| 2104 | lib_dib_Filter15_IDBA_contig_36227_minus2 | 1076 | collagen alpha-4 chain-like | 865 | 0 | 51.25% | 2 | F:protein binding; F:calcium ion binding | IPR000152 (PROSITE); IPR000742 (PFAM); IPR001881 (SMART); IPR002035 (PFAM); IPR013032 (PROSITE); PR00010 (PRINTS), PR00453 (PRINTS), G3DSA:2.10.25.10 (GENE3D), G3DSA:3.40.50.410 (GENE3D), PTHR22992 (PANTHER), SignalP-NN(euk) (SIGNALP), tmhmm (TMHMM), SSF53300 (SUPERFAMILY), SSF57196 (SUPERFAMILY) | Yes |
| 2105 | lib_dib_Filter15_IDBA_contig_36248_plus3 | 26 | semaphorin- isoform b | 280 | 4.83E-81 | 57.85% | 7 | P:multicellular organismal development; P:behavior; P:response to external stimulus; P:anatomical structure morphogenesis; P:embryo development; C:cell; F:receptor activity | IPR000884 (PFAM); IPR002165 (PFAM); IPR016201 (SUPERFAMILY); IPR027231 (PANTHER); PR01705 (PRINTS), G3DSA:2.20.100.10 (GENE3D), G3DSA:3.30.1680.10 (GENE3D), PTHR11036:SF3 (PANTHER) | |
| 2106 | lib_dib_Filter15_IDBA_contig_36291_minus1 | 34 | e3 ubiquitin-protein ligase traf7 | 280 | 8.98E-138 | 82.10% | 15 | P:metabolic process; P:signal transduction; C:organelle; C:intracellular; F:binding; P:regulation of biological process; ; F:receptor activity; C:cytoplasm; F:catalytic activity; P:cellular protein modification process; P:cell death; C:protein complex; C:plasma membrane; F:protein binding | IPR001680 (PFAM); IPR015943 (G3DSA:2.130.10.GENE3D); IPR017986 (PROFILE); IPR019775 (PROSITE); IPR020472 (PRINTS); PTHR22844 (PANTHER), PTHR22844:SF1 (PANTHER) | |
| 2107 | lib_dib_Filter15_IDBA_contig_36292_plus2 | 865 | histone -like | 400 | 1.43E-64 | 99.20% | 5 | C:chromosome; F:DNA binding; P:organelle organization; C:nucleus; F:protein binding | IPR002119 (PRINTS); IPR007125 (PFAM); IPR009072 (G3DSA:1.10.20.GENE3D); PTHR23430 (PANTHER) | |
| 2108 | lib_dib_Filter15_IDBA_contig_36359_minus2 | 1649 | protein | 353 | 1.95E-27 | 47.75% | 1 | F:protein binding | IPR002350 (PFAM); G3DSA:3.30.60.30 (GENE3D), PTHR10913 (PANTHER), PTHR10913:SF21 (PANTHER), SignalP-NN(euk) (SIGNALP), tmhmm (TMHMM), SSF100895 (SUPERFAMILY) | Yes |
| 2109 | lib_dib_Filter15_IDBA_contig_36360_plus1 | 482 | phospholipid scramblase 2-like | 811 | 2.85E-108 | 70.25% | 0 | - | IPR005552 (PANTHER); PTHR23248:SF7 (PANTHER), tmhmm (TMHMM) | |
| 2110 | lib_dib_Filter15_IDBA_contig_36376_minus1 | 410 | sparc-related modular calcium-binding protein 1 | 656 | 3.33E-60 | 54.55% | 5 | P:biological_process; P:multicellular organismal development; C:proteinaceous extracellular matrix; F:calcium ion binding; P:signal transduction | IPR000716 (G3DSA:4.10.800.GENE3D); IPR011992 (G3DSA:1.10.238.GENE3D); IPR018247 (PROSITE); IPR019577 (PFAM); PTHR12352 (PANTHER), PTHR12352:SF3 (PANTHER), SignalP-NN(euk) (SIGNALP), SSF47473 (SUPERFAMILY) | |
| 2111 | lib_dib_Filter15_IDBA_contig_36377_minus1 | 206 | thyroglobulin type-1 repeat-containing domain protein | 339 | 1.91E-49 | 58.90% | 3 | F:calcium ion binding; C:proteinaceous extracellular matrix; P:signal transduction | IPR011992 (G3DSA:1.10.238.GENE3D); IPR018247 (PROSITE); IPR019577 (PFAM); PTHR12352 (PANTHER), PTHR12352:SF3 (PANTHER), SignalP-NN(euk) (SIGNALP), SSF47473 (SUPERFAMILY) | |

| | | | | | | | | | |
|---|---|---|---|---|---|---|---|---|---|
| 2112 | lib_dib_Filter15_IDBA_contig_36378_minus3 | 51 | sparc-related modular calcium-binding protein 1 | 270 | 3.36E-26 | 57.45% | 1 | F:protein binding | IPR000716 (G3DSA:4.10.800.GENE3D); IPR002350 (PFAM); G3DSA:3.30.60.30 (GENE3D), PTHR12352 (PANTHER), PTHR12352:SF3 (PANTHER), SSF100895 (SUPERFAMILY) | |
| 2113 | lib_dib_Filter15_IDBA_contig_36389_minus3 | 195 | dipeptidyl peptidase 1 | 573 | 3.50E-129 | 60.70% | 7 | P:protein metabolic process; C:organelle; C:intracellular; F:peptidase activity; P:biological_process; P:catabolic process; C:cytoplasm | IPR000169 (PROSITE); IPR000668 (PRINTS); IPR013128 (PANTHER); IPR014882 (PFAM); IPR025660 (PROSITE); G3DSA:2.40.128.80 (GENE3D), G3DSA:3.90.70.10 (GENE3D), PTHR12411:SF17 (PANTHER), SignalP-NN(euk) (SIGNALP), SSF54001 (SUPERFAMILY), SSF75001 (SUPERFAMILY) | Yes |
| 2114 | lib_dib_Filter15_IDBA_contig_36390_minus3 | 207 | dipeptidyl peptidase 1 | 600 | 7.76E-129 | 60.80% | 7 | P:protein metabolic process; C:organelle; C:intracellular; F:peptidase activity; P:biological_process; P:catabolic process; C:cytoplasm | IPR000169 (PROSITE); IPR000668 (PRINTS); IPR013128 (PANTHER); IPR014882 (PFAM); IPR025660 (PROSITE); G3DSA:2.40.128.80 (GENE3D), G3DSA:3.90.70.10 (GENE3D), PTHR12411:SF17 (PANTHER), SignalP-NN(euk) (SIGNALP), SSF54001 (SUPERFAMILY), SSF75001 (SUPERFAMILY) | Yes |
| 2115 | lib_dib_Filter15_IDBA_contig_36415_plus1 | 72 | stretchin- isoform s | 279 | 1.77E-20 | 62.90% | 4 | F:protein kinase activity; C:cytoskeleton; C:protein complex; F:protein binding | IPR003598 (SMART); IPR003599 (SMART); IPR007110 (PROFILE); IPR013098 (PFAM); IPR013783 (G3DSA:2.60.40.GENE3D); IPR020675 (PANTHER); PTHR22964:SF3 (PANTHER), SSF48726 (SUPERFAMILY) | |
| 2116 | lib_dib_Filter15_IDBA_contig_36417_minus1 | 1996 | matrix metalloproteinase-9-like | 615 | 2.34E-07 | 61.10% | 26 | P:collagen catabolic process; P:face morphogenesis; C:nucleus; C:sarcomere; P:extracellular matrix disassembly; C:extracellular space; P:extracellular matrix organization; F:metalloendopeptidase activity; F:serine-type endopeptidase activity; P:response to hypoxia; P:proteolysis; P:angiogenesis; P:bone trabecula formation; P:cellular response to amino acid stimulus; F:zinc ion binding; P:intramembranous ossification; C:proteinaceous extracellular matrix; P:embryo implantation; P:blood vessel maturation; C:plasma membrane; C:extracellular region; P:single fertilization; P:sperm capacitation; F:metallopeptidase activity; P:metabolic process; C:extracellular | IPR000562 (G3DSA:2.10.10.GENE3D); IPR013806 (SUPERFAMILY); PTHR22918 (PANTHER), PTHR22918:SF0 (PANTHER), SignalP-NN(euk) (SIGNALP) | Yes |
| 2117 | lib_dib_Filter15_IDBA_contig_36508_plus1 | 39 | fibrillin- partial | 278 | 1.63E-93 | 71.85% | 9 | P:anatomical structure morphogenesis; P:cell differentiation; P:multicellular organismal development; F:receptor activity; C:proteinaceous extracellular matrix; P:embryo development; F:protein binding; F:calcium ion binding; F:structural molecule activity | IPR000152 (PROSITE); IPR000742 (SMART); IPR001881 (PFAM); IPR011398 (PTHR24039:PANTHER); IPR013032 (PROSITE); IPR017878 (G3DSA:3.90.290.GENE3D); IPR018097 (PROSITE); IPR023413 (G3DSA:2.40.155.GENE3D); IPR024731 (PFAM); G3DSA:2.10.25.10 (GENE3D), PTHR24039 (PANTHER), SSF57196 (SUPERFAMILY) | |

| | | | | | | | | | |
|---|---|---|---|---|---|---|---|---|---|
| 2118 | lib_dib_Filter15_IDBA_contig_36510_plus1 | 230 | mitogen-activated protein kinase 14 isoform 1 | 698 | 0 | 84.75% | 28 | P:carbohydrate metabolic process; P:anatomical structure morphogenesis; P:cellular component organization; P:lipid metabolic process; P:cell differentiation; P:regulation of biological process; P:cell death; P:signal transduction; P:response to stress; P:response to abiotic stimulus; F:protein binding; C:cytosol; C:cell; P:biological_process; F:protein kinase activity; P:multicellular organismal development; C:nucleoplasm; P:response to external stimulus; P:nucleobase-containing compound metabolic process; F:signal transducer activity; P:response to biotic stimulus; ; P:behavior; C:cytoskeleton; P:cell cycle; C:mitochondrion; F:nucleotide binding; P:cellular protein modification | IPR000719 (PFAM); IPR002290 (SMART); IPR003527 (PROSITE); IPR008352 (PRINTS); IPR011009 (SUPERFAMILY); IPR017441 (PROSITE); IPR020635 (SMART); G3DSA:1.10.510.10 (GENE3D), G3DSA:3.30.200.20 (GENE3D), PTHR24055 (PANTHER), PTHR24055:SF109 (PANTHER), SignalP-NN(euk) (SIGNALP), tmhmm (TMHMM) |
| 2119 | lib_dib_Filter15_IDBA_contig_36522_minus2 | 54 | kelch-like ech-associated protein 1 | 278 | 8.89E-125 | 74.15% | 14 | C:plasma membrane; P:biological_process; F:protein binding; P:regulation of biological process; P:protein transport; P:metabolic process; ; C:nucleolus; C:microtubule organizing center; P:cell differentiation; P:multicellular organismal development; C:cell; C:endoplasmic reticulum; P:embryo development | IPR006652 (PFAM); IPR015916 (G3DSA:2.130.10.GENE3D); PTHR24412 (PANTHER), PTHR24412:SF1 (PANTHER), SSF117281 (SUPERFAMILY) |
| 2120 | lib_dib_Filter15_IDBA_contig_36551_plus1 | 38 | protocadherin-11 x-linked | 278 | 8.28E-65 | 57.75% | 3 | F:calcium ion binding; P:biological_process; C:plasma membrane | IPR002126 (PRINTS); IPR015919 (SUPERFAMILY); IPR020894 (PROSITE); PTHR24027 (PANTHER) |
| 2121 | lib_dib_Filter15_IDBA_contig_36580_minus3 | 106 | golgi-associated pdz and coiled-coil motif-containing protein | 486 | 8.55E-119 | 83.65% | 14 | C:plasma membrane; F:enzyme regulator activity; F:receptor binding; P:cellular component organization; F:protein binding; C:cell; C:cytoplasmic membrane-bounded vesicle; C:Golgi apparatus; P:reproduction; P:cell differentiation; P:organelle organization; P:biological_process; P:catabolic process; P:protein transport | IPR001478 (PFAM); G3DSA:2.30.42.10 (GENE3D), PTHR16528 (PANTHER) |
| 2122 | lib_dib_Filter15_IDBA_contig_36604_plus1 | 52 | zinc metalloproteinase dpy-31 | 297 | 3.21E-25 | 45.90% | 11 | F:metal ion binding; P:proteolysis; F:metallopeptidase activity; F:hydrolase activity; F:zinc ion binding; F:peptidase activity; F:metalloendopeptidase activity; P:multicellular organismal development; C:extracellular region; P:cuticle development involved in collagen and cuticulin-based cuticle molting cycle; P:embryo development ending in birth or egg hatching | IPR000859 (G3DSA:2.60.120.GENE3D); IPR000884 (PROFILE) |
| 2123 | lib_dib_Filter15_IDBA_contig_36624_minus2 | 36 | zinc finger protein 271-like | 295 | 4.26E-70 | 55.20% | 3 | F:binding; F:nucleic acid binding; C:intracellular | IPR007087 (PROSITE); IPR013087 (G3DSA:3.30.160.GENE3D); IPR015880 (SMART); PTHR24375 (PANTHER), PTHR24375:SF0 (PANTHER), PF13465 (PFAM), PF13912 (PFAM), SSF57667 (SUPERFAMILY) |
| 2124 | lib_dib_Filter15_IDBA_contig_36635_plus1 | 49 | hemocyte protein-glutamine gamma-glutamyltransferase-like | 277 | 9.32E-47 | 51.55% | 2 | F:transferase activity; P:cellular protein modification process | IPR008958 (PFAM); IPR013783 (G3DSA:2.60.40.GENE3D); IPR023608 (PANTHER); G3DSA:3.90.260.10 (GENE3D), PTHR11590:SF17 (PANTHER), SSF54001 (SUPERFAMILY) |

| | | | | | | | | | | |
|---|---|---|---|---|---|---|---|---|---|---|
| 2125 | lib_dib_Filter15_IDBA_contig_36655_plus1 | 23 | beta- -galactosyltransferase 6 | 277 | 1.28E-40 | 59.15% | 2 | F:transferase activity; P:carbohydrate metabolic process | IPR003859 (PRINTS); G3DSA:3.90.550.10 (GENE3D), PF13733 (PFAM), SSF53448 (SUPERFAMILY) | |
| 2126 | lib_dib_Filter15_IDBA_contig_36683_plus3 | 57 | gamma-glutamyltranspeptidase 1 | 276 | 1.96E-58 | 55.65% | 2 | F:transferase activity; | IPR000101 (PRINTS); SignalP-NN(euk) (SIGNALP), tmhmm (TMHMM), SSF56235 (SUPERFAMILY) | Yes |
| 2127 | lib_dib_Filter15_IDBA_contig_36687_plus1 | 38 | transforming growth factor beta receptor type 3 | 277 | 1.25E-29 | 49.90% | 21 | C:cell; P:cellular component organization; P:anatomical structure morphogenesis; P:multicellular organismal development; F:receptor activity; F:protein kinase activity; P:embryo development; F:carbohydrate binding; P:cell differentiation; F:receptor binding; C:plasma membrane; P:growth; P:biological_process; C:cytoplasm; P:regulation of biological process; P:cell communication; F:protein binding; P:cell proliferation; P:signal transduction; P:cellular protein modification process; C:extracellular region | PTHR14002 (PANTHER), tmhmm (TMHMM) | |
| 2128 | lib_dib_Filter15_IDBA_contig_36696_minus3 | 64 | protein | 420 | 2.28E-124 | 60.20% | 3 | C:extracellular region; F:protein binding; F:calcium ion binding | IPR000152 (PROSITE); IPR000742 (PFAM); IPR001881 (SMART); IPR013032 (PROSITE); IPR018097 (PROSITE); IPR026823 (PFAM); PR00010 (PRINTS), G3DSA:2.10.25.10 (GENE3D), PTHR24838 (PANTHER), PTHR24838:SF112 (PANTHER), SSF57196 (SUPERFAMILY) | |
| 2129 | lib_dib_Filter15_IDBA_contig_36709_minus1 | 54 | polypeptide n-acetylgalactosaminyltransferase 1 | 277 | 4.76E-10 | 57.50% | 7 | P:biosynthetic process; P:cellular protein modification process; P:carbohydrate metabolic process; F:binding; C:Golgi apparatus; F:transferase activity; C:cytoplasm | tmhmm (TMHMM) | |
| 2130 | lib_dib_Filter15_IDBA_contig_36719_plus2 | 120 | adam family mig-17 | 495 | 1.08E-62 | 46.75% | 3 | F:peptidase activity; P:protein metabolic process; P:catabolic process | IPR001590 (PFAM); IPR024079 (G3DSA:3.40.390.GENE3D); PTHR13723 (PANTHER), SSF55486 (SUPERFAMILY) | |
| 2131 | lib_dib_Filter15_IDBA_contig_36766_plus1 | 32 | ankyrin repeat domain-containing protein 50- partial | 276 | 2.20E-38 | 54.85% | 1 | F:protein binding | IPR002110 (PRINTS); IPR020683 (G3DSA:1.25.40.GENE3D); PTHR24198 (PANTHER) | |
| 2132 | lib_dib_Filter15_IDBA_contig_36777_minus2 | 1005 | ankyrin repeat protein | 572 | 1.86E-38 | 46.25% | 1 | F:protein binding | IPR001496 (PFAM); IPR002110 (PRINTS); IPR020683 (G3DSA:1.25.40.GENE3D); PTHR24198 (PANTHER) | |
| 2133 | lib_dib_Filter15_IDBA_contig_36866_minus1 | 32 | low-density lipoprotein receptor-related protein 4 | 276 | 2.92E-52 | 63.20% | 19 | P:transport; P:anatomical structure morphogenesis; P:embryo development; F:peptidase activity; P:multicellular organismal development; P:cellular component organization; P:cell differentiation; P:growth; F:receptor binding; C:cell; P:regulation of biological process; P:biological_process; P:signal transduction; P:reproduction; P:cellular protein modification process; P:protein metabolic process; F:protein binding; C:plasma membrane; P:metabolic process | IPR002172 (G3DSA:4.10.400.GENE3D); IPR023415 (PROSITE); PR00261 (PRINTS), PTHR10529 (PANTHER), SignalP-NN(euk) (SIGNALP), tmhmm (TMHMM) | |
| 2134 | lib_dib_Filter15_IDBA_contig_36899_minus2 | 32 | krueppel-like factor 12 | 275 | 7.16E-45 | 87.80% | 7 | F:binding; P:regulation of biological process; ; F:DNA binding; F:protein binding; F:sequence-specific DNA binding transcription factor activity; C:nucleus | IPR007087 (PROSITE); IPR013087 (G3DSA:3.30.160.GENE3D); IPR015880 (SMART); PTHR23223 (PANTHER), PF13465 (PFAM), SSF57667 (SUPERFAMILY) | |

| | | | | | | | | | | |
|---|---|---|---|---|---|---|---|---|---|---|
| 2135 | lib_dib_Filter15_IDBA_contig_36906_plus2 | 26 | nacht and ankyrin domain protein | 275 | 1.78E-41 | 54.40% | 1 | F:protein binding | IPR002110 (PRINTS); IPR020683 (G3DSA:1.25.40.GENE3D); PTHR24198 (PANTHER) | |
| 2136 | lib_dib_Filter15_IDBA_contig_36984_minus3 | 80 | parkinson disease 7 domain-containing protein 1 isoform 1 | 357 | 2.29E-90 | 73.80% | 4 | C:extracellular region; F:molecular_function; P:biological_process; C:cellular_component | IPR002818 (PFAM); G3DSA:3.40.50.880 (GENE3D), PTHR31141 (PANTHER), SSF52317 (SUPERFAMILY) | Yes |
| 2137 | lib_dib_Filter15_IDBA_contig_37023_minus3 | 4448 | endo-beta- -glucanase | 855 | 6.06E-42 | 49.15% | 2 | F:catalytic activity; P:carbohydrate metabolic process | IPR001701 (PFAM); IPR008928 (SUPERFAMILY); IPR012341 (G3DSA:1.50.10.GENE3D); PTHR22298 (PANTHER), SignalP-NN(euk) (SIGNALP), tmhmm (TMHMM) | |
| 2138 | lib_dib_Filter15_IDBA_contig_37024_minus3 | 4558 | endo-b- -glucanase | 785 | 1.17E-44 | 49.30% | 2 | F:catalytic activity; P:carbohydrate metabolic process | IPR001701 (PFAM); IPR008928 (SUPERFAMILY); IPR012341 (G3DSA:1.50.10.GENE3D); PTHR22298 (PANTHER), SignalP-NN(euk) (SIGNALP), tmhmm (TMHMM) | |
| 2139 | lib_dib_Filter15_IDBA_contig_37028_minus2 | 598 | endo-beta- -glucanase | 183 | 4.77E-10 | 52.90% | 2 | F:catalytic activity; P:carbohydrate metabolic process | IPR001701 (PFAM); IPR008928 (SUPERFAMILY); IPR012341 (G3DSA:1.50.10.GENE3D); PTHR22298 (PANTHER) | |
| 2140 | lib_dib_Filter15_IDBA_contig_37052_minus3 | 1669 | matrix metalloproteinase-9 | 472 | 5.52E-20 | 56.65% | 10 | P:biological_process; F:binding; P:reproduction; P:multicellular organismal development; P:regulation of biological process; P:cell death; F:peptidase activity; C:extracellular region; P:metabolic process; P:cellular component organization | IPR000001 (PROFILE); IPR000562 (G3DSA:2.10.10.GENE3D); IPR013806 (SUPERFAMILY); PTHR22918 (PANTHER), PTHR22918:SF0 (PANTHER), SignalP-NN(euk) (SIGNALP) | Yes |
| 2141 | lib_dib_Filter15_IDBA_contig_37053_minus3 | 3107 | matrix metalloproteinase-9 | 920 | 2.67E-21 | 56.05% | 10 | P:biological_process; F:binding; P:reproduction; P:multicellular organismal development; P:regulation of biological process; P:cell death; F:peptidase activity; C:extracellular region; P:metabolic process; P:cellular component organization | IPR000001 (PROFILE); IPR000562 (G3DSA:2.10.10.GENE3D); IPR013806 (SUPERFAMILY); PTHR22918 (PANTHER), PTHR22918:SF0 (PANTHER), SignalP-NN(euk) (SIGNALP), tmhmm (TMHMM) | Yes |
| 2142 | lib_dib_Filter15_IDBA_contig_37118_plus1 | 106 | ---NA--- | 513 | | | 0 | - | SignalP-NN(euk) (SIGNALP), tmhmm (TMHMM) | Yes |
| 2143 | lib_dib_Filter15_IDBA_contig_37154_minus3 | 519 | a chain quaternary ligand binding to aromatic residues in the active-site gorge of acetylcholinesterase | 454 | 8.65E-92 | 60.90% | 1 | F:hydrolase activity | IPR000997 (PRINTS); IPR002018 (PFAM); IPR019819 (PROSITE); IPR019826 (PROSITE); G3DSA:3.40.50.1820 (GENE3D), PTHR11559 (PANTHER), PTHR11559:SF33 (PANTHER), tmhmm (TMHMM), SSF53474 (SUPERFAMILY) | |
| 2144 | lib_dib_Filter15_IDBA_contig_37164_plus1 | 32 | neurogenic locus notch homolog protein 2-like | 273 | 6.34E-33 | 47.10% | 3 | C:extracellular region; F:protein binding; F:calcium ion binding | IPR000152 (PROSITE); IPR000742 (PFAM); IPR000859 (G3DSA:2.60.120.GENE3D); IPR001881 (SMART); IPR013032 (PROSITE); IPR018097 (PROSITE); PR00010 (PRINTS), G3DSA:2.10.25.10 (GENE3D), PTHR24838 (PANTHER), PTHR24838:SF112 (PANTHER), SSF57196 (SUPERFAMILY) | |
| 2145 | lib_dib_Filter15_IDBA_contig_37184_minus2 | 53 | low-density lipoprotein receptor-related protein 4 | 273 | 2.64E-22 | 56.45% | 12 | P:multicellular organismal development; F:peptidase activity; F:protein binding; P:signal transduction; P:regulation of biological process; P:anatomical structure morphogenesis; P:embryo development; P:protein metabolic process; P:biological_process; P:cellular component organization; P:cell differentiation; C:plasma membrane | IPR002172 (G3DSA:4.10.400.GENE3D); IPR023415 (PROSITE); PR00261 (PRINTS), G3DSA:4.10.1220.10 (GENE3D), PTHR10529 (PANTHER) | |
| 2146 | lib_dib_Filter15_IDBA_contig_37207_plus2 | 153 | inter-alpha-trypsin inhibitor heavy chain h5 | 687 | 3.75E-163 | 52.00% | 3 | F:protein binding; F:enzyme regulator activity; P:carbohydrate metabolic process | IPR002035 (PFAM); IPR010600 (PFAM); G3DSA:3.40.50.410 (GENE3D), PTHR10338 (PANTHER), PTHR10338:SF24 (PANTHER), tmhmm (TMHMM), SSF53300 (SUPERFAMILY) | |

| | | | | | | | | | |
|---|---|---|---|---|---|---|---|---|---|
| 2147 | lib_dib_Filter15_IDBA_contig_37208_plus3 | 194 | inter-alpha-trypsin inhibitor heavy chain h5 | 867 | 0 | 51.10% | 3 | F:protein binding; F:enzyme regulator activity; P:carbohydrate metabolic process | IPR002035 (PFAM); IPR010600 (PFAM); IPR013694 (PFAM); G3DSA:3.40.50.410 (GENE3D), PTHR10338 (PANTHER), PTHR10338:SF24 (PANTHER), SSF53300 (SUPERFAMILY) |
| 2148 | lib_dib_Filter15_IDBA_contig_37310_plus1 | 47 | plexin isoform a | 272 | 1.14E-82 | 58.50% | 7 | F:receptor activity; F:protein binding; P:anatomical structure morphogenesis; P:cellular component organization; P:cell differentiation; P:multicellular organismal development; P:cell recognition | IPR001627 (PFAM); IPR015943 (G3DSA:2.130.10.GENE3D); PTHR22625 (PANTHER), PTHR22625:SF12 (PANTHER) |
| 2149 | lib_dib_Filter15_IDBA_contig_37312_minus3 | 48 | zinc finger protein 135-like | 272 | 5.40E-88 | 64.50% | 3 | F:binding; F:nucleic acid binding; C:intracellular | IPR007087 (PROSITE); IPR013087 (G3DSA:3.30.160.GENE3D); IPR015880 (SMART); PTHR24402 (PANTHER), PF13465 (PFAM), SSF57667 (SUPERFAMILY) |
| 2150 | lib_dib_Filter15_IDBA_contig_37500_minus1 | 26 | zinc finger protein 397 | 271 | 2.34E-53 | 56.20% | 5 | P:biological_process; F:protein binding; C:nucleus; F:binding; F:nucleic acid binding | IPR007087 (PFAM); IPR013087 (G3DSA:3.30.160.GENE3D); IPR015880 (SMART); PTHR24402 (PANTHER), PF13465 (PFAM), SSF57667 (SUPERFAMILY) |
| 2151 | lib_dib_Filter15_IDBA_contig_37569_minus3 | 26 | deoxyribonuclease-1-like 2 | 270 | 4.64E-79 | 62.65% | 3 | F:nuclease activity; P:DNA metabolic process; P:catabolic process | IPR005135 (PFAM); IPR008185 (PRINTS); IPR016202 (PANTHER); G3DSA:3.60.10.10 (GENE3D) |
| 2152 | lib_dib_Filter15_IDBA_contig_37703_plus2 | 32 | protocadherin-like wing polarity protein stan-like | 311 | 2.85E-122 | 66.80% | 16 | P:anatomical structure morphogenesis; P:cellular component organization; P:cell differentiation; P:multicellular organismal development; C:plasma membrane; P:biological_process; F:signal transducer activity; P:cell recognition; C:cell; P:signal transduction; P:embryo development; P:regulation of biological process; P:cell-cell signaling; F:receptor activity; F:protein binding; F:calcium ion binding | IPR000742 (PFAM); IPR001791 (PFAM); IPR001881 (SMART); IPR008985 (SUPERFAMILY); IPR013032 (PROSITE); IPR013320 (G3DSA:2.60.120.GENE3D); G3DSA:2.10.25.10 (GENE3D), PTHR24026 (PANTHER), PTHR24026:SF3 (PANTHER), SSF57196 (SUPERFAMILY) |
| 2153 | lib_dib_Filter15_IDBA_contig_37765_plus3 | 55 | beta- -galactosyltransferase | 269 | 1.01E-15 | 63.35% | 11 | P:reproduction; P:anatomical structure morphogenesis; P:cell differentiation; P:multicellular organismal development; P:behavior; P:cellular component organization; P:carbohydrate metabolic process; P:biosynthetic process; P:lipid metabolic process; F:transferase activity; C:Golgi apparatus | IPR003859 (PANTHER); G3DSA:3.90.550.10 (GENE3D), PTHR19300:SF7 (PANTHER), SSF53448 (SUPERFAMILY) |
| 2154 | lib_dib_Filter15_IDBA_contig_37795_plus1 | 408 | isoform a | 569 | 1.19E-169 | 71.85% | 3 | F:catalytic activity; P:regulation of biological process; P:cellular homeostasis | IPR012336 (G3DSA:3.40.30.GENE3D); IPR013766 (PFAM); PTHR18929 (PANTHER), PTHR18929:SF32 (PANTHER), PF13848 (PFAM), tmhmm (TMHMM) |
| 2155 | lib_dib_Filter15_IDBA_contig_37798_minus1 | 43 | ankyrin unc44 | 137 | 2.83E-11 | 57.80% | 5 | F:zinc ion binding; C:intracellular; F:RNA binding; P:RNA-dependent DNA replication; F:RNA-directed DNA polymerase activity | no IPS match |
| 2156 | lib_dib_Filter15_IDBA_contig_37799_minus1 | 48 | ankyrin unc44 | 167 | 5.13E-11 | 57.75% | 6 | F:zinc ion binding; C:intracellular; F:RNA binding; P:RNA-dependent DNA replication; F:RNA-directed DNA polymerase activity; F:endonuclease activity | no IPS match |
| 2157 | lib_dib_Filter15_IDBA_contig_37801_minus2 | 17 | ankyrin unc44 | 102 | 3.59E-12 | 60.45% | 6 | F:RNA binding; P:RNA-dependent DNA replication; F:RNA-directed DNA polymerase activity; F:transmembrane transporter activity; C:integral to membrane; P:transmembrane transport | no IPS match |

| | | | | | | | | | | |
|---|---|---|---|---|---|---|---|---|---|---|
| 2158 | lib_dib_Filter15_IDBA_contig_37826_plus1 | 246 | fibrinogen-like 1 | 308 | 3.98E-51 | 56.80% | 1 | C:extracellular space | IPR002181 (PFAM); IPR014715 (G3DSA:4.10.530.GENE3D); IPR014716 (G3DSA:3.90.215.GENE3D); PTHR19143 (PANTHER), SignalP-NN(euk) (SIGNALP), tmhmm (TMHMM) | Yes |
| 2159 | lib_dib_Filter15_IDBA_contig_37827_plus1 | 121 | fibrinogen-like protein 1-like | 210 | 1.51E-06 | 61.20% | 5 | F:receptor binding; C:extracellular space; P:response to stilbenoid; P:signal transduction; C:fibrinogen complex | IPR002181 (PFAM); IPR014715 (G3DSA:4.10.530.GENE3D); PTHR19143 (PANTHER) | |
| 2160 | lib_dib_Filter15_IDBA_contig_3787_plus1 | 5022 | ubiquitin c | 122 | 1.38E-79 | 100.00% | 3 | C:nucleus; C:cytoplasm; F:protein binding | IPR000626 (PFAM); IPR019954 (PROSITE); IPR019955 (PROFILE); IPR019956 (PRINTS); G3DSA:3.10.20.90 (GENE3D), PTHR10666 (PANTHER), SSF54236 (SUPERFAMILY) | |
| 2161 | lib_dib_Filter15_IDBA_contig_37894_minus1 | 31 | amine oxidase | 268 | 1.09E-108 | 70.80% | 9 | F:catalytic activity; P:catabolic process; ; P:metabolic process; C:cell; C:mitochondrion; P:cell-cell signaling; F:nucleotide binding; F:binding | IPR001613 (PRINTS); IPR002937 (PFAM); G3DSA:1.10.405.10 (GENE3D), G3DSA:3.50.50.60 (GENE3D), PTHR10742 (PANTHER), PTHR10742:SF5 (PANTHER), SSF51905 (SUPERFAMILY) | |
| 2162 | lib_dib_Filter15_IDBA_contig_37933_minus2 | 30 | egf-like domain-containing protein | 268 | 1.25E-70 | 58.95% | 2 | F:protein binding; F:calcium ion binding | IPR000152 (PROSITE); IPR000742 (SMART); IPR001881 (PFAM); IPR013032 (PROSITE); IPR018097 (PROSITE); IPR026823 (PFAM); G3DSA:2.10.25.10 (GENE3D), PTHR24034 (PANTHER), PTHR24034:SF0 (PANTHER), SSF57196 (SUPERFAMILY) | |
| 2163 | lib_dib_Filter15_IDBA_contig_3794_plus1 | 211 | deleted in malignant brain tumors 1 | 101 | 2.20E-14 | 64.45% | 2 | F:receptor activity; C:cell | IPR001190 (PFAM); IPR017448 (SMART); G3DSA:3.10.250.10 (GENE3D), PTHR19331 (PANTHER) | |
| 2164 | lib_dib_Filter15_IDBA_contig_3798_plus1 | 3621 | ---NA--- | 177 | | | 0 | - | IPR008160 (PFAM); PTHR24023 (PANTHER) | |
| 2165 | lib_dib_Filter15_IDBA_contig_38_minus3 | 376 | acylamino-acid-releasing enzyme | 703 | 0 | 64.70% | 4 | C:intracellular; P:protein metabolic process; P:catabolic process; F:peptidase activity | IPR001375 (PFAM); IPR002471 (PROSITE); IPR011042 (G3DSA:2.120.10.GENE3D); G3DSA:3.40.50.1820 (GENE3D), PTHR11731 (PANTHER), PTHR11731:SF7 (PANTHER), SSF53474 (SUPERFAMILY), SSF82171 (SUPERFAMILY) | |
| 2166 | lib_dib_Filter15_IDBA_contig_38000_minus3 | 42 | carboxypeptidase polypeptide 2 | 280 | 1.09E-10 | 51.80% | 1 | F:protein binding | IPR001611 (PFAM); IPR003591 (SMART); G3DSA:3.80.10.10 (GENE3D), PTHR24367 (PANTHER), PF13504 (PFAM), PF13855 (PFAM), SignalP-NN(euk) (SIGNALP), SSF52058 (SUPERFAMILY) | |
| 2167 | lib_dib_Filter15_IDBA_contig_38040_plus3 | 7931 | thioredoxin-dependent peroxide mitochondrial | 652 | 2.44E-119 | 84.55% | 12 | P:biological_process; P:response to stress; F:antioxidant activity; F:catalytic activity; C:organelle; C:intracellular; P:metabolic process; P:regulation of biological process; P:cell death; P:multicellular organismal development; C:cytoplasm; F:protein binding | IPR000866 (PFAM); IPR012336 (G3DSA:3.40.30.GENE3D); IPR019479 (PFAM); PTHR10681 (PANTHER), PTHR10681:SF45 (PANTHER), SignalP-NN(euk) (SIGNALP) | |
| 2168 | lib_dib_Filter15_IDBA_contig_38083_minus2 | 415 | peroxidasin homolog | 478 | 1.03E-104 | 43.95% | 1 | F:protein binding | IPR000483 (SMART); IPR001611 (PROFILE); IPR003591 (SMART); G3DSA:3.80.10.10 (GENE3D), PTHR24373 (PANTHER), PF13855 (PFAM), SignalP-NN(euk) (SIGNALP), tmhmm (TMHMM), SSF52058 (SUPERFAMILY) | Yes |
| 2169 | lib_dib_Filter15_IDBA_contig_38084_minus2 | 407 | peroxidasin homolog | 480 | 3.82E-105 | 43.95% | 1 | F:protein binding | IPR000483 (SMART); IPR001611 (PROFILE); IPR003591 (SMART); G3DSA:3.80.10.10 (GENE3D), PTHR24373 (PANTHER), PF13855 (PFAM), SignalP-NN(euk) (SIGNALP), tmhmm (TMHMM), SSF52058 (SUPERFAMILY) | Yes |

| | | | | | | | | | | |
|---|---|---|---|---|---|---|---|---|---|---|
| 2170 | lib_dib_Filter15_IDBA_contig_38085_minus2 | 387 | leucine-rich repeat-containing protein 4b-like | 458 | 2.63E-105 | 43.85% | 1 | F:protein binding | IPR000483 (SMART); IPR001611 (PROFILE); IPR003591 (SMART); G3DSA:3.80.10.10 (GENE3D), PTHR24373 (PANTHER), PF13855 (PFAM), SignalP-NN(euk) (SIGNALP), tmhmm (TMHMM), SSF52058 (SUPERFAMILY) | Yes |
| 2171 | lib_dib_Filter15_IDBA_contig_38096_plus1 | 37 | ---NA--- | 267 | | | 0 | - | IPR008160 (PFAM); PTHR24023 (PANTHER) | |
| 2172 | lib_dib_Filter15_IDBA_contig_38106_minus1 | 84 | slit like protein 2 partial | 337 | 5.16E-125 | 70.45% | 10 | P:multicellular organismal development; P:anatomical structure morphogenesis; P:cellular component organization; P:cell differentiation; P:regulation of biological process; P:growth; F:protein binding; P:biological_process; P:behavior; P:response to external stimulus | IPR000372 (PFAM); IPR000483 (SMART); IPR001611 (PROFILE); IPR003591 (SMART); G3DSA:3.80.10.10 (GENE3D), PTHR24373 (PANTHER), PTHR24373:SF39 (PANTHER), PF13855 (PFAM), SSF52058 (SUPERFAMILY) | |
| 2173 | lib_dib_Filter15_IDBA_contig_38174_minus1 | 48 | neuroligin 2b | 267 | 1.25E-119 | 57.70% | 3 | P:cell adhesion; C:membrane; F:hydrolase activity | IPR002018 (PFAM); IPR019819 (PROSITE); G3DSA:3.40.50.1820 (GENE3D), PTHR11559 (PANTHER), SSF53474 (SUPERFAMILY) | |
| 2174 | lib_dib_Filter15_IDBA_contig_38203_plus3 | 41 | ankyrin | 266 | 9.42E-31 | 51.70% | 1 | F:protein binding | IPR002110 (SMART); IPR020683 (G3DSA:1.25.40.GENE3D); PTHR24188 (PANTHER), PF13857 (PFAM) | |
| 2175 | lib_dib_Filter15_IDBA_contig_38204_minus2 | 159 | gli pathogenesis-related 1 -like | 415 | 4.39E-42 | 50.10% | 2 | F:metal ion binding; C:extracellular region | IPR001283 (PRINTS); IPR014044 (G3DSA:3.40.33.GENE3D); PTHR10334:SF12 (PANTHER), SignalP-NN(euk) (SIGNALP), tmhmm (TMHMM) | |
| 2176 | lib_dib_Filter15_IDBA_contig_38230_plus2 | 39 | polypeptide n-acetylgalactosaminyltransferase 5 | 266 | 3.44E-65 | 51.45% | 8 | F:carbohydrate binding; F:transferase activity; C:integral to membrane; C:membrane; C:Golgi apparatus; F:transferase activity, transferring glycosyl groups; F:polypeptide N-acetylgalactosaminyltransferase activity; P:carbohydrate metabolic process | PTHR11675 (PANTHER) | |
| 2177 | lib_dib_Filter15_IDBA_contig_38251_plus2 | 32 | low-density lipoprotein receptor-related protein 2 | 266 | 3.01E-62 | 59.85% | 2 | F:protein binding; F:calcium ion binding | IPR000033 (SMART); IPR000152 (PROSITE); IPR000742 (SMART); IPR001881 (PFAM); IPR002172 (G3DSA:4.10.400.GENE3D); IPR011042 (G3DSA:2.120.10.GENE3D); IPR018097 (PROSITE); IPR023415 (PROSITE); G3DSA:2.10.25.10 (GENE3D), PTHR10529 (PANTHER), SSF57196 (SUPERFAMILY), SSF63825 (SUPERFAMILY) | |
| 2178 | lib_dib_Filter15_IDBA_contig_38265_minus1 | 845 | procollagen c-endopeptidase enhancer 2 | 807 | 1.83E-107 | 51.70% | 6 | C:collagen; C:extracellular region; F:peptidase activator activity; F:heparin binding; F:collagen binding; P:positive regulation of peptidase activity | IPR000859 (G3DSA:2.60.120.GENE3D); IPR003014 (PFAM); IPR003609 (SMART); G3DSA:3.50.4.10 (GENE3D), PTHR10127 (PANTHER), tmhmm (TMHMM), SSF57414 (SUPERFAMILY) | |
| 2179 | lib_dib_Filter15_IDBA_contig_38280_plus1 | 48 | protein fam172a isoform 1 | 266 | 1.52E-74 | 65.00% | 1 | C:endoplasmic reticulum | IPR019154 (PFAM); PTHR21357 (PANTHER), PTHR21357:SF0 (PANTHER) | |
| 2180 | lib_dib_Filter15_IDBA_contig_38283_plus3 | 192 | nicotinic acetylcholine receptor subunit alpha 6 transcript variant partial | 591 | 1.22E-56 | 55.60% | 3 | F:ion channel activity; P:ion transport; C:cell | IPR002394 (PRINTS); IPR006029 (SUPERFAMILY); IPR006201 (PRINTS); IPR006202 (G3DSA:2.70.170.GENE3D); IPR018000 (PROSITE); G3DSA:1.20.120.370 (GENE3D), PTHR18945:SF92 (PANTHER), SignalP-NN(euk) (SIGNALP), tmhmm (TMHMM) | Yes |
| 2181 | lib_dib_Filter15_IDBA_contig_38284_plus1 | 189 | nicotinic acetylcholine receptor subunit alpha 6 transcript variant partial | 599 | 4.70E-57 | 55.45% | 3 | F:ion channel activity; P:ion transport; C:cell | IPR002394 (PRINTS); IPR006029 (SUPERFAMILY); IPR006201 (PRINTS); IPR006202 (G3DSA:2.70.170.GENE3D); IPR018000 (PROSITE); G3DSA:1.20.120.370 (GENE3D), PTHR18945:SF92 (PANTHER), tmhmm (TMHMM) | |

| | | | | | | | | | |
|---|---|---|---|---|---|---|---|---|---|
| 2182 | lib_dib_Filter15_IDBA_contig_38353_plus1 | 415 | leucine-rich immunoglobulin-like domain and transmembrane domain-containing protein 1 | 245 | 1.44E-10 | 43.25% | 1 | F:protein binding | IPR000483 (SMART); IPR001611 (PFAM); PR00019 (PRINTS), G3DSA:3.80.10.10 (GENE3D), PTHR24373 (PANTHER), SSF52058 (SUPERFAMILY) |
| 2183 | lib_dib_Filter15_IDBA_contig_38354_plus1 | 1265 | carboxypeptidase polypeptide 2 | 580 | 1.96E-20 | 43.75% | 1 | F:protein binding | IPR000483 (SMART); IPR001611 (PFAM); IPR003591 (SMART); G3DSA:3.80.10.10 (GENE3D), PTHR24373 (PANTHER), PF13855 (PFAM), SSF52058 (SUPERFAMILY) |
| 2184 | lib_dib_Filter15_IDBA_contig_38360_minus2 | 33 | signal cub and egf-like domain-containing protein 1 | 265 | 2.06E-89 | 61.80% | 2 | F:protein binding; F:calcium ion binding | IPR000152 (PROSITE); IPR000742 (SMART); IPR001881 (PFAM); IPR013032 (PROSITE); IPR018097 (PROSITE); G3DSA:2.10.25.10 (GENE3D), PTHR24046 (PANTHER), SSF57196 (SUPERFAMILY) |
| 2185 | lib_dib_Filter15_IDBA_contig_38384_plus2 | 158 | histone-lysine n-methyltransferase prdm9 | 383 | 4.98E-50 | 58.30% | 3 | F:binding; F:nucleic acid binding; C:intracellular | IPR007087 (PFAM); IPR013087 (G3DSA:3.30.160.GENE3D); IPR015880 (SMART); PTHR24375 (PANTHER), PTHR24375:SF0 (PANTHER), PF13465 (PFAM), tmhmm (TMHMM), SSF57667 (SUPERFAMILY) |
| 2186 | lib_dib_Filter15_IDBA_contig_38404_plus1 | 65 | dual specificity mitogen-activated protein kinase kinase 1 | 359 | 4.49E-81 | 73.35% | 37 | C:plasma membrane; F:protein kinase activity; P:regulation of biological process; P:transport; C:cell; F:protein binding; F:structural molecule activity; P:signal transduction; P:response to stress; P:organelle organization; P:anatomical structure morphogenesis; P:cellular component organization; P:cell differentiation; P:multicellular organismal development; P:embryo development; F:phosphoprotein phosphatase activity; F:signal transducer activity; C:endoplasmic reticulum; P:cellular protein modification process; P:response to endogenous stimulus; C:cytosol; F:enzyme regulator activity; C:cytoplasm; C:peroxisome; ; C:endosome; C:Golgi apparatus; C:mitochondrion; P:cell cycle; F:nucleotide binding; C:microtubule organizing center; P:cell proliferation; C:cytoskeleton; C:protein complex; C:nucleus; P:response to external stimulus; | IPR000719 (PFAM); IPR002290 (SMART); IPR008271 (PROSITE); IPR011009 (SUPERFAMILY); G3DSA:1.10.510.10 (GENE3D), PTHR24360 (PANTHER), PTHR24360:SF36 (PANTHER), tmhmm (TMHMM) |
| 2187 | lib_dib_Filter15_IDBA_contig_38472_minus1 | 36 | bone morphogenetic protein 4 | 265 | 9.52E-26 | 56.00% | 14 | P:regulation of biological process; P:multicellular organismal development; P:biosynthetic process; P:anatomical structure morphogenesis; P:cell differentiation; P:metabolic process; P:cell proliferation; P:signal transduction; P:reproduction; P:embryo development; P:growth; P:cellular protein modification process; P:cell death; F:receptor binding | IPR001111 (PFAM); IPR015615 (PANTHER); PTHR11848:SF50 (PANTHER), SignalP-NN(euk) (SIGNALP), tmhmm (TMHMM) |
| 2188 | lib_dib_Filter15_IDBA_contig_38580_plus2 | 103 | endoplasmic reticulum resident protein 29-like | 264 | 5.48E-82 | 65.15% | 2 | C:endoplasmic reticulum; P:protein transport | IPR011679 (G3DSA:1.20.1150.GENE3D); IPR012336 (G3DSA:3.40.30.GENE3D); IPR012883 (PFAM); PTHR12211 (PANTHER), PTHR12211:SF0 (PANTHER) |
| 2189 | lib_dib_Filter15_IDBA_contig_38645_minus1 | 38 | upf0378 protein kiaa0100-like | 264 | 5.63E-56 | 61.60% | 0 | - | IPR019409 (PFAM); PTHR15678 (PANTHER), PTHR15678:SF6 (PANTHER) |

| | | | | | | | | | |
|---|---|---|---|---|---|---|---|---|---|
| 2190 | lib_dib_Filter15_IDBA_contig_38666_plus1 | 79 | achain structural determinants for improved thermal stability of designed ankyrin repeat proteins with a redesigned c-capping | 284 | 7.47E-76 | 58.30% | 1 | F:protein binding | IPR001496 (PFAM); IPR002110 (PRINTS); IPR020683 (G3DSA:1.25.40.GENE3D); PTHR24188 (PANTHER) | |
| 2191 | lib_dib_Filter15_IDBA_contig_38671_plus1 | 73 | serine threonine-protein kinase tricorner-like | 264 | 6.10E-145 | 92.30% | 3 | F:protein kinase activity; P:cellular protein modification process; F:nucleotide binding | IPR000719 (PFAM); IPR002290 (SMART); IPR008271 (PROSITE); IPR011009 (SUPERFAMILY); IPR017441 (PROSITE); IPR020635 (SMART); G3DSA:1.10.510.10 (GENE3D), G3DSA:3.30.200.20 (GENE3D), PTHR24358 (PANTHER) | |
| 2192 | lib_dib_Filter15_IDBA_contig_38676_minus1 | 294 | nedd4 family-interacting protein 1 | 362 | 6.53E-41 | 56.50% | 4 | C:integral to membrane; C:membrane; C:Golgi apparatus; C:Golgi membrane | IPR019325 (PFAM); PTHR13396 (PANTHER), tmhmm (TMHMM) | |
| 2193 | lib_dib_Filter15_IDBA_contig_38683_minus3 | 2775 | ferric-chelate reductase 1 | 1174 | 1.00E-66 | 41.30% | 1 | C:cell | IPR002861 (PFAM); IPR004877 (PFAM); IPR005018 (PFAM); IPR006593 (SMART); PTHR23130 (PANTHER), SignalP-NN(euk) (SIGNALP), tmhmm (TMHMM) | Yes |
| 2194 | lib_dib_Filter15_IDBA_contig_38740_plus1 | 1378 | pdz and lim domain protein 5 isoform 1 | 374 | 9.77E-20 | 53.35% | 3 | F:cytoskeletal protein binding; C:cytoplasm; C:cytoskeleton | IPR001478 (PFAM); G3DSA:2.30.42.10 (GENE3D), PTHR24214 (PANTHER) | |
| 2195 | lib_dib_Filter15_IDBA_contig_38774_minus1 | 50 | protein crumbs | 263 | 9.97E-56 | 58.50% | 2 | F:protein binding; F:calcium ion binding | IPR000152 (PROSITE); IPR000742 (PFAM); IPR001881 (SMART); IPR008985 (SUPERFAMILY); IPR013032 (PROSITE); IPR013320 (G3DSA:2.60.120.GENE3D); IPR018097 (PROSITE); PR00010 (PRINTS), G3DSA:2.10.25.10 (GENE3D), PTHR24838 (PANTHER), PTHR24838:SF112 (PANTHER), SSF57196 (SUPERFAMILY) | |
| 2196 | lib_dib_Filter15_IDBA_contig_38778_minus2 | 23 | toll-like receptor 5 | 263 | 1.78E-14 | 53.65% | 3 | P:signal transduction; P:response to stress; C:cell | IPR000483 (SMART); G3DSA:3.80.10.10 (GENE3D), PTHR24365 (PANTHER), PF13504 (PFAM), PF13516 (PFAM), PF13855 (PFAM), tmhmm (TMHMM), SSF52058 (SUPERFAMILY) | |
| 2197 | lib_dib_Filter15_IDBA_contig_38816_plus2 | 70 | collagen alpha-3 chain | 316 | 2.99E-41 | 50.15% | 1 | F:protein binding | IPR002035 (PFAM); PR00453 (PRINTS), G3DSA:3.40.50.410 (GENE3D), PTHR22992 (PANTHER), SSF53300 (SUPERFAMILY) | |
| 2198 | lib_dib_Filter15_IDBA_contig_38850_plus2 | 7733 | antimicrobial-like peptide pp-1 | 890 | 5.91E-13 | 79.20% | 3 | P:response to stress; P:response to biotic stimulus; P:biological_process | tmhmm (TMHMM) | |
| 2199 | lib_dib_Filter15_IDBA_contig_38856_minus1 | 38 | fad-dependent amine oxidase | 262 | 1.50E-88 | 65.55% | 3 | C:cellular_component; P:oxidation-reduction process; F:oxidoreductase activity | G3DSA:3.50.50.60 (GENE3D), G3DSA:3.90.660.10 (GENE3D), PTHR23357 (PANTHER) | |
| 2200 | lib_dib_Filter15_IDBA_contig_38861_minus3 | 306 | fibrinogen c domain-containing protein 1-like | 421 | 4.45E-56 | 57.30% | 7 | F:receptor binding; C:extracellular space; P:signal transduction; P:cell-matrix adhesion; F:G-protein coupled receptor activity; C:membrane; P:G-protein coupled receptor signaling pathway | IPR002181 (PFAM); IPR014715 (G3DSA:4.10.530.GENE3D); IPR014716 (G3DSA:3.90.215.GENE3D); IPR020837 (PROSITE); PTHR19143 (PANTHER), SignalP-NN(euk) (SIGNALP), tmhmm (TMHMM) | Yes |
| 2201 | lib_dib_Filter15_IDBA_contig_38862_minus3 | 221 | ficolin (collagen fibrinogen domain containing lectin) 2 | 405 | 1.86E-56 | 59.85% | 2 | P:biological_process; F:protein binding | IPR002181 (PFAM); IPR014715 (G3DSA:4.10.530.GENE3D); IPR014716 (G3DSA:3.90.215.GENE3D); IPR020837 (PROSITE); PTHR19143 (PANTHER), SignalP-NN(euk) (SIGNALP), tmhmm (TMHMM) | Yes |
| 2202 | lib_dib_Filter15_IDBA_contig_38863_minus1 | 61 | angiopoietin-related protein 4-like | 167 | 4.27E-12 | 62.20% | 1 | P:biological_process | IPR002181 (PFAM); IPR014716 (G3DSA:3.90.215.GENE3D); PTHR19143 (PANTHER), SignalP-NN(euk) (SIGNALP) | |
| 2203 | lib_dib_Filter15_IDBA_contig_38901_plus2 | 23 | n-acetylglucosamine-1-phosphodiester alpha-n-acetylglucosaminidase-like | 262 | 3.95E-70 | 59.90% | 1 | F:protein binding | IPR000742 (SMART); IPR013032 (PROSITE); IPR018711 (PFAM); G3DSA:2.10.25.10 (GENE3D), G3DSA:2.170.300.10 (GENE3D), PTHR11219 (PANTHER), PTHR11219:SF2 (PANTHER), tmhmm (TMHMM), SSF57196 (SUPERFAMILY) | |

| | | | | | | | | | |
|---|---|---|---|---|---|---|---|---|---|
| 2204 | lib_dib_Filter15_IDBA_contig_38936_plus2 | 36 | kelch-like ech-associated protein 1-like | 261 | 1.00E-121 | 73.45% | 2 | F:actin binding; P:response to stress | IPR000210 (SMART); IPR011333 (G3DSA:3.30.710.GENE3D); IPR011705 (PFAM); IPR013069 (PFAM); PTHR24412 (PANTHER), PTHR24412:SF1 (PANTHER) |
| 2205 | lib_dib_Filter15_IDBA_contig_38941_plus2 | 642 | pentapeptide repeat protein | 451 | 9.05E-28 | 56.25% | 0 | - | PTHR14136 (PANTHER), PTHR14136:SF0 (PANTHER), PF13599 (PFAM), tmhmm (TMHMM), SSF141571 (SUPERFAMILY) |
| 2206 | lib_dib_Filter15_IDBA_contig_38942_plus2 | 677 | pentapeptide repeat-containing protein | 586 | 1.83E-26 | 60.00% | 0 | - | PTHR14136 (PANTHER), PTHR14136:SF0 (PANTHER), PF13599 (PFAM), SSF141571 (SUPERFAMILY) |
| 2207 | lib_dib_Filter15_IDBA_contig_38942_plus3 | 677 | ---NA--- | 585 | | | 0 | - | IPR006954 (PANTHER); PTHR21523:SF0 (PANTHER), tmhmm (TMHMM) |
| 2208 | lib_dib_Filter15_IDBA_contig_39010_minus1 | 3920 | interleukin-6 receptor subunit beta | 603 | 9.45E-13 | 42.10% | 1 | F:protein binding | IPR003961 (PFAM); IPR013783 (G3DSA:2.60.40.GENE3D); IPR015321 (PFAM); PTHR23036 (PANTHER), tmhmm (TMHMM) |
| 2209 | lib_dib_Filter15_IDBA_contig_39037_plus1 | 30 | c-type mannose receptor 2 | 261 | 1.52E-20 | 41.60% | 1 | F:carbohydrate binding | IPR001304 (PFAM); IPR016186 (G3DSA:3.10.100.GENE3D); IPR016187 (SUPERFAMILY); IPR018378 (PROSITE); PTHR22802 (PANTHER), PTHR22802:SF16 (PANTHER) |
| 2210 | lib_dib_Filter15_IDBA_contig_39048_plus3 | 33 | secreted frizzled-related protein 3 | 260 | 1.94E-06 | 59.70% | 1 | F:protein binding | IPR001134 (PROFILE); IPR008993 (SUPERFAMILY); IPR015526 (PANTHER); IPR018933 (PFAM); IPR026556 (PTHR11309:PANTHER); G3DSA:2.40.50.120 (GENE3D), tmhmm (TMHMM) |
| 2211 | lib_dib_Filter15_IDBA_contig_39056_plus1 | 34 | zinc finger protein 160-like | 261 | 3.18E-19 | 47.30% | 3 | F:binding; F:nucleic acid binding; C:intracellular | IPR007087 (PROSITE); IPR013087 (G3DSA:3.30.160.GENE3D); IPR015880 (SMART); PTHR11389 (PANTHER), PTHR11389:SF382 (PANTHER), PF13465 (PFAM), PF13894 (PFAM), SSF57667 (SUPERFAMILY) |
| 2212 | lib_dib_Filter15_IDBA_contig_39064_minus2 | 37 | ankyrin repeat protein | 261 | 5.34E-27 | 55.25% | 1 | F:protein binding | IPR001496 (SMART); IPR002110 (SMART); IPR020683 (G3DSA:1.25.40.GENE3D); PTHR24188 (PANTHER) |
| 2213 | lib_dib_Filter15_IDBA_contig_39080_minus1 | 36 | protein sidekick-like | 261 | 1.98E-48 | 58.75% | 1 | F:protein binding | IPR003598 (SMART); IPR003961 (PFAM); IPR007110 (PROFILE); IPR013098 (PFAM); IPR013783 (G3DSA:2.60.40.GENE3D); PR00014 (PRINTS), PTHR10489 (PANTHER), SSF48726 (SUPERFAMILY) |
| 2214 | lib_dib_Filter15_IDBA_contig_39088_plus3 | 22 | glucose dehydrogenase | 260 | 5.57E-75 | 60.25% | 2 | F:nucleotide binding; P:metabolic process | IPR000172 (PFAM); IPR007867 (PFAM); G3DSA:3.30.560.10 (GENE3D), G3DSA:3.50.50.60 (GENE3D), PTHR11552 (PANTHER), PTHR11552:SF10 (PANTHER), SSF51905 (SUPERFAMILY), SSF54373 (SUPERFAMILY) |
| 2215 | lib_dib_Filter15_IDBA_contig_39089_minus2 | 36 | a disintegrin and metalloproteinase with thrombospondin motifs 7-like | 260 | 3.24E-52 | 52.65% | 1 | F:peptidase activity | IPR000884 (PFAM); G3DSA:2.20.100.10 (GENE3D), PTHR13723 (PANTHER), PTHR13723:SF30 (PANTHER) |
| 2216 | lib_dib_Filter15_IDBA_contig_39091_plus3 | 140 | thrombospondin type-1 domain-containing protein 7a | 540 | 1.56E-66 | 48.15% | 0 | - | IPR000884 (PFAM); G3DSA:2.20.100.10 (GENE3D), PTHR11311 (PANTHER), tmhmm (TMHMM) |
| 2217 | lib_dib_Filter15_IDBA_contig_39092_plus3 | 93 | thrombospondin type-1 domain-containing protein 7a | 331 | 1.97E-22 | 42.20% | 0 | - | PTHR11311 (PANTHER), tmhmm (TMHMM) |
| 2218 | lib_dib_Filter15_IDBA_contig_39145_minus2 | 70 | zinc finger protein 271-like | 498 | 3.32E-85 | 51.20% | 3 | F:binding; F:nucleic acid binding; C:intracellular | IPR007087 (PFAM); IPR013087 (G3DSA:3.30.160.GENE3D); IPR015880 (SMART); IPR022755 (PFAM); PTHR24375 (PANTHER), PTHR24375:SF0 (PANTHER), PF12756 (PFAM), PF13465 (PFAM), PF13894 (PFAM), PF13912 (PFAM), tmhmm (TMHMM), SSF57667 (SUPERFAMILY) |

| | | | | | | | | | | |
|---|---|---|---|---|---|---|---|---|---|---|
| 2219 | lib_dib_Filter15_IDBA_contig_39146_minus1 | 23 | zinc finger protein 2 homolog | 178 | 2.09E-28 | 54.50% | 3 | F:binding; F:nucleic acid binding; C:intracellular | IPR007087 (PROSITE); IPR013087 (G3DSA:3.30.160.GENE3D); IPR015880 (SMART); PTHR11389 (PANTHER), PF13465 (PFAM), PF13894 (PFAM), PF13912 (PFAM), SSF57667 (SUPERFAMILY) | |
| 2220 | lib_dib_Filter15_IDBA_contig_39160_plus2 | 607 | cyclin-dependent kinase 9-like | 451 | 0 | 89.45% | 4 | F:protein kinase activity; P:cellular protein modification process; F:nucleotide binding; P:biological_process | IPR000719 (PFAM); IPR002290 (SMART); IPR008271 (PROSITE); IPR011009 (SUPERFAMILY); IPR017441 (PROSITE); IPR020635 (SMART); G3DSA:1.10.510.10 (GENE3D), G3DSA:3.30.200.20 (GENE3D), PTHR24056 (PANTHER), PTHR24056:SF34 (PANTHER) | |
| 2221 | lib_dib_Filter15_IDBA_contig_39169_plus1 | 36 | arylacetamide deacetylase-like 4-like | 260 | 7.29E-21 | 48.15% | 2 | P:metabolic process; F:hydrolase activity | IPR013094 (PFAM); G3DSA:3.40.50.1820 (GENE3D), PTHR23024 (PANTHER), SSF53474 (SUPERFAMILY) | |
| 2222 | lib_dib_Filter15_IDBA_contig_39271_plus1 | 57 | 15-hydroxyprostaglandin dehydrogenase | 260 | 1.81E-59 | 62.70% | 4 | P:biological_process; F:nucleotide binding; P:metabolic process; F:catalytic activity | IPR002198 (PRINTS); IPR002347 (PRINTS); IPR016040 (G3DSA:3.40.50.GENE3D); IPR020904 (PROSITE); PTHR24310 (PANTHER), PTHR24310:SF0 (PANTHER), SSF51735 (SUPERFAMILY) | |
| 2223 | lib_dib_Filter15_IDBA_contig_39282_minus2 | 95 | partial | 405 | 5.62E-61 | 50.50% | 3 | F:transferase activity, transferring glycosyl groups; F:transferase activity; C:extracellular region | IPR007657 (PFAM); PTHR20961 (PANTHER), PTHR20961:SF0 (PANTHER) | |
| 2224 | lib_dib_Filter15_IDBA_contig_39330_minus1 | 43 | neuronal acetylcholine receptor subunit alpha-7-like | 281 | 1.30E-121 | 66.50% | 5 | F:ion channel activity; F:receptor activity; C:cell; C:plasma membrane; P:ion transport | IPR002394 (PRINTS); IPR006029 (PFAM); IPR006201 (PRINTS); IPR006202 (G3DSA:2.70.170.GENE3D); IPR018000 (PROSITE); G3DSA:1.20.120.370 (GENE3D), SignalP-NN(euk) (SIGNALP), tmhmm (TMHMM) | Yes |
| 2225 | lib_dib_Filter15_IDBA_contig_39384_plus3 | 10 | low-density lipoprotein receptor-related protein 1b | 123 | 1.56E-20 | 60.05% | 3 | C:cell; F:receptor activity; F:protein binding | IPR000033 (SMART); IPR011042 (G3DSA:2.120.10.GENE3D); PTHR10529 (PANTHER), PTHR10529:SF161 (PANTHER), SSF63825 (SUPERFAMILY) | |
| 2226 | lib_dib_Filter15_IDBA_contig_39400_plus1 | 107 | retinol dehydrogenase 13-like | 371 | 9.01E-105 | 71.90% | 3 | P:metabolic process; F:catalytic activity; F:nucleotide binding | IPR002198 (PRINTS); IPR002347 (PRINTS); IPR016040 (G3DSA:3.40.50.GENE3D); PTHR24320 (PANTHER), PTHR24320:SF0 (PANTHER), SSF51735 (SUPERFAMILY) | |
| 2227 | lib_dib_Filter15_IDBA_contig_39424_minus1 | 60 | heparanase-2 | 335 | 3.17E-15 | 57.35% | 4 | F:protein binding; C:proteinaceous extracellular matrix; F:hydrolase activity; C:cell | IPR005199 (PANTHER) | |
| 2228 | lib_dib_Filter15_IDBA_contig_39425_minus2 | 23 | heparanase isoform 1 | 189 | 2.26E-49 | 64.65% | 13 | P:regulation of biological process; P:cell proliferation; C:cell; P:biological_process; F:hydrolase activity; F:protein binding; P:catabolic process; C:lysosome; P:response to external stimulus; P:response to stress; P:multicellular organismal development; C:nucleus; P:signal transduction | IPR005199 (PANTHER); IPR017853 (SUPERFAMILY); PTHR14363:SF1 (PANTHER) | |
| 2229 | lib_dib_Filter15_IDBA_contig_39533_minus1 | 240 | histone h2a | 287 | 1.34E-65 | 92.25% | 8 | C:chromosome; P:DNA metabolic process; P:cell cycle; P:response to stress; F:DNA binding; P:organelle organization; C:nucleus; F:protein binding | IPR002119 (PRINTS); IPR007125 (PFAM); IPR009072 (G3DSA:1.10.20.GENE3D); PTHR23430 (PANTHER) | |
| 2230 | lib_dib_Filter15_IDBA_contig_39539_minus3 | 1265 | aminopeptidase n | 1262 | 7.08E-119 | 48.05% | 4 | P:protein metabolic process; P:catabolic process; F:peptidase activity; F:binding | IPR001930 (PANTHER); IPR014782 (PRINTS); IPR024571 (PFAM); G3DSA:1.10.390.10 (GENE3D), tmhmm (TMHMM), SSF55486 (SUPERFAMILY), SSF63737 (SUPERFAMILY) | |
| 2231 | lib_dib_Filter15_IDBA_contig_39540_minus1 | 81 | aminopeptidase n-like | 258 | 5.26E-39 | 50.70% | 2 | P:protein metabolic process; P:catabolic process | IPR001930 (PANTHER); IPR024571 (PFAM) | |

| | | | | | | | | | |
|---|---|---|---|---|---|---|---|---|---|
| 2232 | lib_dib_Filter15_IDBA_contig_39542_minus1 | 57 | glutamyl aminopeptidase | 161 | 1.17E-33 | 59.55% | 10 | P:biological_process; C:organelle; F:binding; C:cell; C:plasma membrane; P:protein metabolic process; P:catabolic process; F:peptidase activity; P:metabolic process; P:cell proliferation | IPR001930 (PANTHER); IPR014782 (PRINTS); SSF55486 (SUPERFAMILY) |
| 2233 | lib_dib_Filter15_IDBA_contig_39543_minus1 | 45 | aminopeptidase n-like | 150 | 2.03E-16 | 52.40% | 4 | P:protein metabolic process; P:catabolic process; F:peptidase activity; F:binding | IPR001930 (PANTHER); IPR014782 (PFAM); SSF63737 (SUPERFAMILY) |
| 2234 | lib_dib_Filter15_IDBA_contig_39580_plus1 | 763 | alpha-galactosidase alpha-n-acetylgalactosaminidase | 703 | 1.54E-171 | 68.15% | 2 | F:hydrolase activity; P:carbohydrate metabolic process | IPR000111 (PFAM); IPR002241 (PRINTS); IPR013780 (G3DSA:2.60.40.GENE3D); IPR013785 (G3DSA:3.20.20.GENE3D); IPR017853 (SUPERFAMILY); PTHR11452 (PANTHER), SSF51011 (SUPERFAMILY) |
| 2235 | lib_dib_Filter15_IDBA_contig_39596_plus1 | 441 | cellular retinoic acid-binding protein 2 | 262 | 9.22E-21 | 53.90% | 9 | F:lipid binding; C:cytosol; P:biological_process; P:lipid metabolic process; P:secondary metabolic process; P:multicellular organismal development; C:nucleus; F:transporter activity; P:transport | IPR000463 (PRINTS); IPR000566 (PFAM); IPR011038 (SUPERFAMILY); IPR012674 (G3DSA:2.40.128.GENE3D); PTHR11955 (PANTHER), PTHR11955:SF47 (PANTHER) |
| 2236 | lib_dib_Filter15_IDBA_contig_39599_plus1 | 81 | cellular retinoic acid-binding protein 1 | 207 | 4.54E-31 | 54.30% | 7 | F:lipid binding; P:biological_process; P:multicellular organismal development; C:cytoplasm; C:nucleus; F:transporter activity; P:transport | IPR000463 (PRINTS); IPR000566 (PFAM); IPR011038 (SUPERFAMILY); IPR012674 (G3DSA:2.40.128.GENE3D); PTHR11955 (PANTHER), PTHR11955:SF47 (PANTHER), tmhmm (TMHMM) |
| 2237 | lib_dib_Filter15_IDBA_contig_39606_minus3 | 40 | scabrous protein | 257 | 4.43E-57 | 59.25% | 7 | P:cell cycle; P:anatomical structure morphogenesis; P:multicellular organismal development; P:cell differentiation; P:biological_process; P:cellular component organization; C:extracellular region | IPR002181 (PFAM); IPR014716 (G3DSA:3.90.215.GENE3D); PTHR19143 (PANTHER) |
| 2238 | lib_dib_Filter15_IDBA_contig_39644_minus1 | 1366 | neuroglobin | 623 | 7.31E-48 | 55.55% | 3 | F:binding; P:transport; F:oxygen binding | IPR000971 (PFAM); IPR009050 (SUPERFAMILY); IPR012292 (G3DSA:1.10.490.GENE3D); IPR013316 (PRINTS); PTHR22924 (PANTHER), tmhmm (TMHMM) |
| 2239 | lib_dib_Filter15_IDBA_contig_39692_minus1 | 74 | prolactin | 257 | 1.47E-121 | 91.50% | 2 | C:extracellular region; F:receptor binding | IPR001400 (PRINTS); IPR009079 (SUPERFAMILY); IPR012351 (G3DSA:1.20.1250.GENE3D); IPR018116 (PROSITE); PTHR11417:SF28 (PANTHER), SignalP-NN(euk) (SIGNALP), tmhmm (TMHMM) |
| 2240 | lib_dib_Filter15_IDBA_contig_39706_plus3 | 46 | mitogen-activated protein kinase kinase kinase 1 | 256 | 3.21E-127 | 83.85% | 25 | P:regulation of biological process; P:cell death; C:cytoskeleton; P:signal transduction; P:response to stress; P:cellular protein modification process; P:cytoskeleton organization; F:lipid binding; F:protein kinase activity; P:response to abiotic stimulus; F:signal transducer activity; ; P:response to external stimulus; F:catalytic activity; C:cytoplasm; P:reproduction; P:viral reproduction; F:binding; P:multicellular organismal development; F:protein binding; F:nucleotide binding; P:mitochondrion organization; P:catabolic process; P:cellular component organization; C:cell | IPR000719 (PFAM); IPR002290 (SMART); IPR008271 (PROSITE); IPR011009 (SUPERFAMILY); IPR020635 (SMART); G3DSA:1.10.510.10 (GENE3D), G3DSA:3.30.200.20 (GENE3D), PTHR24361 (PANTHER), PTHR24361:SF96 (PANTHER) |

| | | | | | | | | | |
|---|---|---|---|---|---|---|---|---|---|
| 2241 | lib_dib_Filter15_IDBA_contig_39779_minus1 | 26 | fbn22 protein | 102 | 5.42E-20 | 61.50% | 8 | F:receptor binding; P:signal transduction; F:receptor activity; C:integral to membrane; F:G-protein coupled receptor activity; F:signal transducer activity; P:G-protein coupled receptor signaling pathway; C:extracellular space | IPR002181 (PFAM); IPR014716 (G3DSA:3.90.215.GENE3D); PTHR19143 (PANTHER) |
| 2242 | lib_dib_Filter15_IDBA_contig_39780_minus2 | 71 | angiopoietin-related protein 7 | 256 | 4.69E-33 | 58.45% | 3 | F:receptor binding; C:extracellular space; P:signal transduction | IPR002181 (PFAM); IPR014715 (G3DSA:4.10.530.GENE3D); IPR014716 (G3DSA:3.90.215.GENE3D); PTHR19143 (PANTHER) |
| 2243 | lib_dib_Filter15_IDBA_contig_39814_plus3 | 1591 | plasminogen activator spa | 256 | 1.81E-53 | 58.15% | 3 | F:peptidase activity; P:protein metabolic process; P:catabolic process | IPR001254 (PFAM); IPR001314 (PRINTS); IPR009003 (SUPERFAMILY); IPR018114 (PROSITE); G3DSA:2.40.10.10 (GENE3D), PTHR24265 (PANTHER) |
| 2244 | lib_dib_Filter15_IDBA_contig_39815_plus1 | 302 | transmembrane protease serine 5 | 103 | 4.93E-10 | 82.80% | 8 | F:receptor activity; C:extracellular space; F:binding; F:peptidase activity; C:plasma membrane; P:protein metabolic process; P:catabolic process; P:biological_process | IPR001254 (PFAM); IPR009003 (SUPERFAMILY); G3DSA:2.40.10.10 (GENE3D), PTHR24264 (PANTHER) |
| 2245 | lib_dib_Filter15_IDBA_contig_39816_plus1 | 159 | transmembrane protease serine 5 | 103 | 5.34E-10 | 82.65% | 5 | C:cell; F:receptor activity; P:protein metabolic process; P:catabolic process; F:peptidase activity | IPR001254 (PFAM); IPR009003 (SUPERFAMILY); G3DSA:2.40.10.10 (GENE3D), PTHR24264 (PANTHER) |
| 2246 | lib_dib_Filter15_IDBA_contig_39818_minus3 | 16633 | enolase | 256 | 1.51E-117 | 86.50% | 17 | P:biosynthetic process; P:carbohydrate metabolic process; C:cell; C:cytosol; C:protein complex; P:response to external stimulus; P:anatomical structure morphogenesis; P:multicellular organismal development; P:response to stress; P:growth; P:biological_process; F:catalytic activity; F:hydrolase activity; F:binding; P:generation of precursor metabolites and energy; P:catabolic process; F:protein binding | IPR000941 (PRINTS); IPR020810 (PFAM); IPR020811 (PFAM); G3DSA:3.20.20.120 (GENE3D), G3DSA:3.30.390.10 (GENE3D), SSF51604 (SUPERFAMILY), SSF54826 (SUPERFAMILY) |
| 2247 | lib_dib_Filter15_IDBA_contig_39819_minus1 | 7130 | enolase | 146 | 5.18E-86 | 86.20% | 13 | P:reproduction; P:multicellular organismal development; P:embryo development; C:cytosol; C:protein complex; P:regulation of biological process; P:growth; F:catalytic activity; F:hydrolase activity; F:binding; P:carbohydrate metabolic process; P:generation of precursor metabolites and energy; P:catabolic process | IPR000941 (PRINTS); IPR020810 (PFAM); G3DSA:3.20.20.120 (GENE3D), SSF51604 (SUPERFAMILY) |
| 2248 | lib_dib_Filter15_IDBA_contig_39821_minus1 | 6752 | enolase | 172 | 2.07E-74 | 84.55% | 23 | P:response to biotic stimulus; C:cytosol; C:protein complex; P:biosynthetic process; P:carbohydrate metabolic process; P:regulation of biological process; ; P:biological_process; F:protein binding; F:transcription regulator activity; C:cell; F:hydrolase activity; C:plasma membrane; C:cytoplasm; F:catalytic activity; P:generation of precursor metabolites and energy; P:catabolic process; F:DNA binding; P:cell growth; F:binding; F:sequence-specific DNA binding transcription factor activity; C:nucleus; P:embryo development | IPR000941 (PRINTS); IPR020811 (PFAM); G3DSA:3.20.20.120 (GENE3D), G3DSA:3.30.390.10 (GENE3D), SSF54826 (SUPERFAMILY) |

| | | | | | | | | | | |
|---|---|---|---|---|---|---|---|---|---|---|
| 2249 | lib_dib_Filter15_IDBA_contig_39823_minus1 | 6766 | eno1 partial | 186 | 6.02E-100 | 87.15% | 21 | P:response to biotic stimulus; C:cytosol; C:protein complex; P:biosynthetic process; P:carbohydrate metabolic process; P:regulation of biological process; ; F:protein binding; F:transcription regulator activity; C:cell; C:cytoplasm; F:catalytic activity; P:generation of precursor metabolites and energy; P:catabolic process; F:DNA binding; P:cell growth; F:binding; F:sequence-specific DNA binding transcription factor activity; C:plasma membrane; C:nucleus; F:structural molecule activity | IPR000941 (PRINTS); IPR020809 (PROSITE); IPR020810 (PFAM); G3DSA:3.20.20.120 (GENE3D), SSF51604 (SUPERFAMILY) | |
| 2250 | lib_dib_Filter15_IDBA_contig_39841_minus2 | 184 | egf-like domain-containing protein | 576 | 1.74E-64 | 63.30% | 2 | F:protein binding; F:calcium ion binding | IPR000152 (PROSITE); IPR000742 (SMART); IPR001881 (PFAM); IPR008160 (PFAM); IPR013032 (PROSITE); IPR018097 (PROSITE); G3DSA:2.10.25.10 (GENE3D), PTHR24048 (PANTHER), SignalP-NN(euk) (SIGNALP), tmhmm (TMHMM), SSF57196 (SUPERFAMILY) | Yes |
| 2251 | lib_dib_Filter15_IDBA_contig_39869_plus3 | 32 | heparanase precursor | 256 | 3.83E-49 | 47.65% | 8 | P:multicellular organismal development; P:regulation of biological process; P:response to external stimulus; P:response to stress; P:biological_process; F:catalytic activity; F:binding; C:cell | IPR005199 (PANTHER); IPR017853 (SUPERFAMILY); PTHR14363:SF1 (PANTHER) | |
| 2252 | lib_dib_Filter15_IDBA_contig_39890_minus2 | 906 | peptidylprolyl isomerase b | 223 | 3.44E-83 | 83.85% | 3 | P:protein metabolic process; P:cellular protein modification process; F:catalytic activity | IPR002130 (PRINTS); IPR020892 (PROSITE); G3DSA:2.40.100.10 (GENE3D), PTHR11071 (PANTHER), PTHR11071:SF63 (PANTHER), SignalP-NN(euk) (SIGNALP), tmhmm (TMHMM) | |
| 2253 | lib_dib_Filter15_IDBA_contig_39898_minus2 | 37 | metalloproteinase | 256 | 1.67E-22 | 46.25% | 3 | F:peptidase activity; P:protein metabolic process; P:catabolic process | IPR001590 (PFAM); IPR001762 (G3DSA:4.10.70.GENE3D); IPR024079 (G3DSA:3.40.390.GENE3D); PTHR11905 (PANTHER), SSF55486 (SUPERFAMILY) | |
| 2254 | lib_dib_Filter15_IDBA_contig_39929_plus1 | 197 | segment polarity protein dishevelled homolog dvl-3-like | 715 | 0 | 74.95% | 7 | P:anatomical structure morphogenesis; P:cellular component organization; F:protein binding; C:intracellular; P:cytoskeleton organization; F:signal transducer activity; P:multicellular organismal development | IPR000591 (PFAM); IPR001158 (PFAM); IPR001478 (PFAM); IPR003351 (PFAM); IPR008339 (PRINTS); IPR008342 (PTHR10878:PANTHER); IPR011991 (G3DSA:1.10.10.GENE3D); IPR015506 (PANTHER); G3DSA:2.30.42.10 (GENE3D), SSF46785 (SUPERFAMILY) | |
| 2255 | lib_dib_Filter15_IDBA_contig_39938_minus2 | 774 | ---NA--- | 530 | | | 0 | - | no IPS match | |
| 2256 | lib_dib_Filter15_IDBA_contig_39939_minus2 | 786 | ---NA--- | 586 | | | 0 | - | no IPS match | |
| 2257 | lib_dib_Filter15_IDBA_contig_39992_plus3 | 19 | gamma-glutamyltranspeptidase 1 | 102 | 7.45E-27 | 66.10% | 2 | F:transferase activity; | IPR000101 (PANTHER); SSF56235 (SUPERFAMILY) | |
| 2258 | lib_dib_Filter15_IDBA_contig_39996_plus2 | 49 | alpha- -mannosyl-glycoprotein 4-beta-n-acetylglucosaminyltransferase b-like | 255 | 1.26E-51 | 61.15% | 3 | F:transferase activity; C:cell; P:carbohydrate metabolic process | IPR006759 (PANTHER); SignalP-NN(euk) (SIGNALP), tmhmm (TMHMM) | |
| 2259 | lib_dib_Filter15_IDBA_contig_40049_plus3 | 43 | discoidin domain-containing receptor 2 | 255 | 2.81E-121 | 73.10% | 13 | P:multicellular organismal development; P:biological_process; P:regulation of biological process; ; P:cellular component organization; P:metabolic process; P:anatomical structure morphogenesis; P:growth; P:cell differentiation; F:protein binding; P:cell proliferation; C:plasma membrane; F:nucleotide binding | IPR000719 (PROFILE); IPR001245 (PRINTS); IPR002290 (SMART); IPR008266 (PROSITE); IPR011009 (SUPERFAMILY); IPR020635 (SMART); G3DSA:1.10.510.10 (GENE3D), G3DSA:3.30.200.20 (GENE3D), PTHR24416 (PANTHER), PTHR24416:SF64 (PANTHER), SignalP-NN(euk) (SIGNALP) | |

| | | | | | | | | | | |
|---|---|---|---|---|---|---|---|---|---|---|
| 2260 | lib_dib_Filter15_IDBA_contig_40080_plus3 | 89 | plexin isoform a | 444 | 2.91E-48 | 47.15% | 7 | P:anatomical structure morphogenesis; P:cellular component organization; P:cell differentiation; P:multicellular organismal development; F:protein binding; C:cell; F:receptor activity | IPR001627 (PFAM); IPR002165 (PFAM); IPR003659 (SMART); IPR015943 (G3DSA:2.130.10.GENE3D); IPR016201 (SUPERFAMILY); G3DSA:3.30.1680.10 (GENE3D), PTHR22625 (PANTHER) | |
| 2261 | lib_dib_Filter15_IDBA_contig_40133_plus3 | 47 | neurotrypsin | 254 | 1.48E-30 | 51.45% | 3 | F:peptidase activity; F:receptor activity; C:cell | IPR001190 (PRINTS); IPR017448 (SMART); G3DSA:3.10.250.10 (GENE3D), PTHR19331 (PANTHER), PTHR19331:SF118 (PANTHER) | |
| 2262 | lib_dib_Filter15_IDBA_contig_40203_minus2 | 26 | zinc finger | 254 | 5.96E-22 | 66.50% | 3 | F:binding; F:nucleic acid binding; C:intracellular | IPR007087 (PROSITE); IPR013087 (G3DSA:3.30.160.GENE3D); IPR015880 (SMART); PTHR24402 (PANTHER), PTHR24402:SF42 (PANTHER), PF13465 (PFAM), SSF57667 (SUPERFAMILY) | |
| 2263 | lib_dib_Filter15_IDBA_contig_40271_plus1 | 11 | protein phosphatase 1 regulatory subunit 16a-like | 113 | 3.20E-52 | 83.30% | 2 | F:hydrolase activity; F:protein binding | IPR002110 (SMART); IPR020683 (G3DSA:1.25.40.GENE3D); PTHR24186 (PANTHER), PTHR24186:SF0 (PANTHER) | |
| 2264 | lib_dib_Filter15_IDBA_contig_40275_plus3 | 76 | fibrinogen c domain-containing protein 1-like | 300 | 1.79E-47 | 56.70% | 4 | F:receptor binding; C:extracellular space; P:signal transduction; F:carbohydrate binding | IPR002181 (PFAM); IPR014715 (G3DSA:4.10.530.GENE3D); IPR014716 (G3DSA:3.90.215.GENE3D); IPR020837 (PROSITE); PTHR19143 (PANTHER), SignalP-NN(euk) (SIGNALP) | Yes |
| 2265 | lib_dib_Filter15_IDBA_contig_40276_plus1 | 18 | fibrinogen c domain-containing protein 1- partial | 175 | 1.81E-40 | 62.55% | 4 | F:receptor binding; P:signal transduction; C:extracellular space; F:carbohydrate binding | IPR002181 (PFAM); IPR014716 (G3DSA:3.90.215.GENE3D); PTHR19143 (PANTHER) | |
| 2266 | lib_dib_Filter15_IDBA_contig_40280_plus3 | 237 | fibrinogen c domain-containing protein 1-like | 276 | 1.77E-57 | 62.25% | 1 | C:extracellular region | IPR002181 (PFAM); IPR014715 (G3DSA:4.10.530.GENE3D); IPR014716 (G3DSA:3.90.215.GENE3D); IPR020837 (PROSITE); PTHR19143 (PANTHER) | |
| 2267 | lib_dib_Filter15_IDBA_contig_40281_plus3 | 239 | fibrinogen c domain-containing protein 1-like | 320 | 1.31E-56 | 61.20% | 3 | F:receptor binding; C:extracellular space; P:signal transduction | IPR002181 (PFAM); IPR014715 (G3DSA:4.10.530.GENE3D); IPR014716 (G3DSA:3.90.215.GENE3D); IPR020837 (PROSITE); PTHR19143 (PANTHER) | |
| 2268 | lib_dib_Filter15_IDBA_contig_40282_plus1 | 77 | fibrinogen c domain-containing protein 1- partial | 223 | 1.28E-09 | 58.45% | 4 | P:biological_process; F:binding; P:regulation of biological process; P:primary metabolic process | IPR002181 (PFAM); IPR014716 (G3DSA:3.90.215.GENE3D); PTHR19143 (PANTHER) | |
| 2269 | lib_dib_Filter15_IDBA_contig_40285_plus1 | 46 | fibrinogen c domain-containing protein 1-like | 121 | 3.86E-32 | 66.40% | 3 | F:receptor binding; C:extracellular space; P:signal transduction | IPR002181 (PFAM); IPR014715 (G3DSA:4.10.530.GENE3D); IPR014716 (G3DSA:3.90.215.GENE3D); IPR020837 (PROSITE); PTHR19143 (PANTHER) | |
| 2270 | lib_dib_Filter15_IDBA_contig_40286_plus3 | 26 | fibrinogen-related protein | 106 | 3.81E-24 | 64.20% | 3 | F:receptor binding; C:extracellular space; P:signal transduction | IPR002181 (PFAM); IPR014715 (G3DSA:4.10.530.GENE3D); IPR014716 (G3DSA:3.90.215.GENE3D); IPR020837 (PROSITE); PTHR19143 (PANTHER) | |
| 2271 | lib_dib_Filter15_IDBA_contig_40287_plus1 | 38 | fibrinogen-related protein | 116 | 1.84E-27 | 64.35% | 3 | F:receptor binding; C:extracellular space; P:signal transduction | IPR002181 (PFAM); IPR014715 (G3DSA:4.10.530.GENE3D); IPR014716 (G3DSA:3.90.215.GENE3D); IPR020837 (PROSITE); PTHR19143 (PANTHER) | |
| 2272 | lib_dib_Filter15_IDBA_contig_40288_plus3 | 46 | microfibril-associated glycoprotein 4-like | 135 | 3.73E-20 | 64.80% | 2 | P:biological_process; F:carbohydrate binding | IPR002181 (PFAM); IPR014715 (G3DSA:4.10.530.GENE3D); IPR020837 (PROSITE); PTHR19143 (PANTHER) | |
| 2273 | lib_dib_Filter15_IDBA_contig_40323_plus1 | 48 | gaba-b receptor | 254 | 2.04E-114 | 70.35% | 3 | C:cell; F:receptor activity; P:signal transduction | IPR002455 (PRINTS); IPR002456 (PRINTS); IPR017978 (PFAM); PTHR10519 (PANTHER), PTHR10519:SF3 (PANTHER), tmhmm (TMHMM), SSF53822 (SUPERFAMILY) | |
| 2274 | lib_dib_Filter15_IDBA_contig_40328_minus3 | 147 | retinoschisin precursor | 199 | 1.72E-10 | 52.50% | 5 | F:lipid binding; C:intracellular; P:signal transduction; C:plasma membrane; P:biological_process | IPR000421 (PFAM); IPR008979 (SUPERFAMILY); G3DSA:2.60.120.260 (GENE3D), PTHR24543 (PANTHER), PTHR24543:SF27 (PANTHER) | |

| | | | | | | | | | |
|---|---|---|---|---|---|---|---|---|---|
| 2275 | lib_dib_Filter15_IDBA_contig_40329_minus3 | 768 | transmembrane protease serine 3 | 690 | 2.09E-71 | 53.50% | 6 | C:endoplasmic reticulum; C:cell; P:biological_process; F:peptidase activity; P:protein metabolic process; P:catabolic process | IPR000421 (PFAM); IPR001254 (PFAM); IPR001314 (PRINTS); IPR008979 (SUPERFAMILY); IPR009003 (SUPERFAMILY); IPR018114 (PROSITE); G3DSA:2.40.10.10 (GENE3D), G3DSA:2.60.120.260 (GENE3D), PTHR24265 (PANTHER) |
| 2276 | lib_dib_Filter15_IDBA_contig_40340_minus3 | 33 | kelch-like protein 17 | 253 | 1.96E-32 | 55.65% | 8 | C:cytoplasm; F:protein binding; F:structural molecule activity; P:multicellular organismal development; F:actin binding; P:cytoskeleton organization; C:cytoskeleton; C:cell | IPR000210 (SMART); IPR011333 (G3DSA:3.30.710.GENE3D); IPR011705 (PFAM); IPR013069 (PFAM); PTHR24412 (PANTHER) |
| 2277 | lib_dib_Filter15_IDBA_contig_40401_plus1 | 41 | zinc finger protein 850-like | 254 | 1.02E-16 | 42.00% | 3 | F:binding; F:nucleic acid binding; C:intracellular | IPR007087 (PFAM); IPR013087 (G3DSA:3.30.160.GENE3D); IPR015880 (SMART); PTHR11389 (PANTHER), PTHR11389:SF357 (PANTHER), PF12874 (PFAM), PF13465 (PFAM), PF13894 (PFAM), SSF57667 (SUPERFAMILY) |
| 2278 | lib_dib_Filter15_IDBA_contig_40405_minus1 | 255 | si:ch211- protein | 612 | 7.10E-11 | 43.20% | 1 | F:carbohydrate binding | IPR001304 (PFAM); IPR002889 (PFAM); IPR016186 (G3DSA:3.10.100.GENE3D); IPR016187 (SUPERFAMILY); PTHR22802 (PANTHER) |
| 2279 | lib_dib_Filter15_IDBA_contig_40423_minus2 | 109 | neutral cholesterol ester hydrolase 1 | 398 | 2.23E-69 | 54.35% | 3 | P:metabolic process; F:hydrolase activity; C:cell | IPR013094 (PFAM); IPR017157 (PIR); G3DSA:3.40.50.1820 (GENE3D), PTHR23024 (PANTHER), SignalP-NN(euk) (SIGNALP), tmhmm (TMHMM), SSF53474 (SUPERFAMILY) |
| 2280 | lib_dib_Filter15_IDBA_contig_40424_minus2 | 158 | neutral cholesterol ester hydrolase 1 | 545 | 1.52E-67 | 53.70% | 2 | P:metabolic process; F:hydrolase activity | IPR013094 (PFAM); G3DSA:3.40.50.1820 (GENE3D), PTHR23024 (PANTHER), SignalP-NN(euk) (SIGNALP), tmhmm (TMHMM), SSF53474 (SUPERFAMILY) |
| 2281 | lib_dib_Filter15_IDBA_contig_40425_minus1 | 50 | alpha beta hydrolase domain-containing protein | 288 | 2.15E-21 | 55.85% | 2 | P:metabolic process; F:hydrolase activity | IPR013094 (PFAM); G3DSA:3.40.50.1820 (GENE3D), PTHR23024 (PANTHER), tmhmm (TMHMM), SSF53474 (SUPERFAMILY) |
| 2282 | lib_dib_Filter15_IDBA_contig_40440_minus1 | 49 | egf-like domain-containing protein | 208 | 1.75E-54 | 61.15% | 2 | F:protein binding; F:calcium ion binding | IPR000152 (PROSITE); IPR000742 (SMART); IPR001881 (PFAM); IPR013032 (PROSITE); IPR018097 (PROSITE); IPR026823 (PFAM); PD968187 (PRODOM), G3DSA:2.10.25.10 (GENE3D), PTHR24034 (PANTHER), PTHR24034:SF0 (PANTHER), SSF57196 (SUPERFAMILY) |
| 2283 | lib_dib_Filter15_IDBA_contig_40441_minus1 | 42 | egf-like domain-containing protein | 253 | 1.32E-69 | 61.05% | 2 | F:protein binding; F:calcium ion binding | IPR000152 (PROSITE); IPR000742 (SMART); IPR001881 (PFAM); IPR013032 (PROSITE); IPR018097 (PROSITE); IPR026823 (PFAM); PD968187 (PRODOM), G3DSA:2.10.25.10 (GENE3D), PTHR24034 (PANTHER), SSF57196 (SUPERFAMILY) |
| 2284 | lib_dib_Filter15_IDBA_contig_40442_minus3 | 18 | egf-like domain-containing protein | 125 | 1.78E-33 | 65.60% | 2 | F:protein binding; F:calcium ion binding | IPR000152 (PROSITE); IPR000742 (SMART); IPR001881 (PFAM); IPR013032 (PROSITE); IPR018097 (PROSITE); G3DSA:2.10.25.10 (GENE3D), PTHR24838 (PANTHER), PTHR24838:SF171 (PANTHER), SSF57196 (SUPERFAMILY) |
| 2285 | lib_dib_Filter15_IDBA_contig_40449_minus1 | 51 | probable proline racemase-like | 253 | 1.23E-43 | 70.45% | 1 | F:catalytic activity | IPR008794 (PFAM); G3DSA:3.10.310.10 (GENE3D), PTHR32298 (PANTHER), PTHR32298:SF0 (PANTHER), SSF54506 (SUPERFAMILY) |
| 2286 | lib_dib_Filter15_IDBA_contig_40484_minus1 | 450 | protein | 418 | 1.11E-49 | 66.20% | 4 | C:proteinaceous extracellular matrix; F:protein binding; F:calcium ion binding; P:biological_process | IPR000152 (PROSITE); IPR000742 (PFAM); IPR001881 (SMART); IPR003886 (PFAM); IPR013032 (PROSITE); IPR018097 (PROSITE); PR00010 (PRINTS), G3DSA:2.10.25.10 (GENE3D), PTHR24048 (PANTHER), PTHR24048:SF15 (PANTHER), SSF57196 (SUPERFAMILY) |

| | | | | | | | | | |
|---|---|---|---|---|---|---|---|---|---|
| 2287 | lib_dib_Filter15_IDBA_contig_40485_minus3 | 27 | protein | 101 | 2.76E-33 | 74.25% | 5 | C:proteinaceous extracellular matrix; C:organelle; C:cytoplasm; F:protein binding; F:calcium ion binding | IPR000152 (PROSITE); IPR000742 (PFAM); IPR001881 (SMART); IPR013032 (PROSITE); IPR018097 (PROSITE); PR00010 (PRINTS), G3DSA:2.10.25.10 (GENE3D), PTHR24048 (PANTHER), PTHR24048:SF15 (PANTHER), SSF57196 (SUPERFAMILY) | |
| 2288 | lib_dib_Filter15_IDBA_contig_40487_minus1 | 239 | ubiquitin | 328 | 2.53E-20 | 69.80% | 1 | F:protein binding | IPR000626 (PFAM); IPR019955 (PROFILE); IPR019956 (PRINTS); G3DSA:3.10.20.90 (GENE3D), PTHR10666 (PANTHER), SSF54236 (SUPERFAMILY) | |
| 2289 | lib_dib_Filter15_IDBA_contig_40507_minus2 | 627 | kazal-type proteinase inhibitor | 253 | 3.61E-07 | 61.95% | 1 | F:protein binding | IPR002350 (PFAM); G3DSA:3.30.60.30 (GENE3D), PTHR10913 (PANTHER), PTHR10913:SF21 (PANTHER), tmhmm (TMHMM), SSF100895 (SUPERFAMILY) | |
| 2290 | lib_dib_Filter15_IDBA_contig_40587_plus2 | 1080 | acidic mammalian chitinase-like | 678 | 3.78E-134 | 63.50% | 3 | F:hydrolase activity; P:carbohydrate metabolic process; P:catabolic process | IPR001223 (PFAM); IPR011583 (SMART); IPR013781 (G3DSA:3.20.20.GENE3D); IPR017853 (SUPERFAMILY); G3DSA:3.10.50.10 (GENE3D), PTHR11177 (PANTHER), PTHR11177:SF34 (PANTHER), SignalP-NN(euk) (SIGNALP), tmhmm (TMHMM), SSF54556 (SUPERFAMILY) | Yes |
| 2291 | lib_dib_Filter15_IDBA_contig_40588_plus3 | 63 | brain chitinase and chia | 114 | 3.13E-31 | 61.40% | 2 | F:hydrolase activity; P:carbohydrate metabolic process | IPR001223 (PFAM); IPR013781 (G3DSA:3.20.20.GENE3D); IPR017853 (SUPERFAMILY); G3DSA:3.10.50.10 (GENE3D), PTHR11177 (PANTHER), SSF54556 (SUPERFAMILY) | |
| 2292 | lib_dib_Filter15_IDBA_contig_40628_minus3 | 73 | fkbp-type peptidyl-prolyl cis-trans isomerase | 252 | 3.07E-40 | 73.95% | 2 | F:catalytic activity; P:protein metabolic process | IPR001179 (PFAM); IPR023566 (PANTHER); G3DSA:3.10.50.40 (GENE3D), PTHR10516:SF125 (PANTHER), SSF54534 (SUPERFAMILY) | |
| 2293 | lib_dib_Filter15_IDBA_contig_40631_minus2 | 100 | e3 ubiquitin-protein ligase mib2-like | 500 | 7.09E-102 | 61.05% | 13 | P:transport; P:cellular component organization; P:metabolic process; P:regulation of biological process; P:cell differentiation; F:signal transducer activity; C:organelle; C:cytoplasm; P:signal transduction; F:catalytic activity; P:cellular protein modification process; F:protein binding; F:binding | IPR001841 (SMART); IPR002110 (PRINTS); IPR013083 (G3DSA:3.30.40.GENE3D); IPR020683 (G3DSA:1.25.40.GENE3D); PTHR24202 (PANTHER), PTHR24202:SF1 (PANTHER), PF13920 (PFAM), SSF57850 (SUPERFAMILY) | |
| 2294 | lib_dib_Filter15_IDBA_contig_40639_plus1 | 34 | carboxypeptidase a2 | 252 | 3.89E-29 | 53.75% | 5 | F:peptidase activity; C:extracellular region; F:binding; P:protein metabolic process; P:catabolic process | IPR000834 (PRINTS); IPR003146 (G3DSA:3.30.70.GENE3D); IPR009020 (SUPERFAMILY); G3DSA:3.40.630.10 (GENE3D), PTHR11705 (PANTHER), SignalP-NN(euk) (SIGNALP), SSF53187 (SUPERFAMILY) | Yes |
| 2295 | lib_dib_Filter15_IDBA_contig_40645_plus3 | 211 | corticosteroid 11-beta-dehydrogenase isozyme 1-like | 373 | 4.91E-54 | 58.10% | 9 | P:multicellular organismal development; C:cell; P:biosynthetic process; P:lipid metabolic process; P:metabolic process; P:biological_process; F:catalytic activity; C:endoplasmic reticulum; F:nucleotide binding | IPR002198 (PFAM); IPR002347 (PRINTS); IPR016040 (G3DSA:3.40.50.GENE3D); IPR020904 (PROSITE); PTHR24322 (PANTHER), PTHR24322:SF32 (PANTHER), SignalP-NN(euk) (SIGNALP), tmhmm (TMHMM), SSF51735 (SUPERFAMILY) | |
| 2296 | lib_dib_Filter15_IDBA_contig_40706_minus3 | 33 | hypothetical protein TRIADDRAFT_33213 | 251 | 9.96E-45 | 52.90% | 2 | F:protein binding; F:calcium ion binding | IPR000152 (PROSITE); IPR000742 (SMART); IPR001881 (PFAM); IPR013032 (PROSITE); IPR018097 (PROSITE); IPR023413 (G3DSA:2.40.155.GENE3D); IPR024731 (PFAM); G3DSA:2.10.25.10 (GENE3D), PTHR24838 (PANTHER), PTHR24838:SF171 (PANTHER), SSF57196 (SUPERFAMILY) | |

| | | | | | | | | | |
|---|---|---|---|---|---|---|---|---|---|
| 2297 | lib_dib_Filter15_IDBA_contig_40710_minus1 | 25 | disintegrin and metalloproteinase domain-containing protein 12-like | 252 | 3.85E-46 | 54.80% | 6 | P:biological_process; P:regulation of biological process; F:peptidase activity; P:protein metabolic process; P:catabolic process; F:binding | IPR001590 (PFAM); IPR002870 (PFAM); IPR024079 (G3DSA:3.40.390.GENE3D); PTHR11905 (PANTHER), SSF55486 (SUPERFAMILY) |
| 2298 | lib_dib_Filter15_IDBA_contig_40718_minus1 | 66 | cysteine-rich motor neuron 1 protein | 429 | 5.07E-61 | 47.00% | 5 | P:biological_process; P:regulation of biological process; P:growth; F:protein binding; F:enzyme regulator activity | IPR001007 (PFAM); IPR004094 (PFAM); IPR011061 (SUPERFAMILY); IPR018112 (G3DSA:2.10.22.GENE3D); G3DSA:2.10.70.10 (GENE3D), PTHR11339 (PANTHER), PTHR11339:SF40 (PANTHER), SSF57603 (SUPERFAMILY) |
| 2299 | lib_dib_Filter15_IDBA_contig_40738_minus2 | 821 | isoform c | 251 | 1.29E-09 | 43.30% | 1 | F:protein binding | IPR002172 (G3DSA:4.10.400.GENE3D); PTHR10529 (PANTHER), SignalP-NN(euk) (SIGNALP), tmhmm (TMHMM) |
| 2300 | lib_dib_Filter15_IDBA_contig_40760_plus2 | 33 | oocyte zinc finger protein 6-partial | 251 | 6.50E-44 | 53.65% | 3 | F:binding; F:nucleic acid binding; C:intracellular | IPR007087 (PFAM); IPR013087 (G3DSA:3.30.160.GENE3D); IPR015880 (SMART); PTHR24375 (PANTHER), PTHR24375:SF0 (PANTHER), PF13465 (PFAM), PF13894 (PFAM), SSF57667 (SUPERFAMILY) |
| 2301 | lib_dib_Filter15_IDBA_contig_40782_minus2 | 31 | zinc finger protein 26 | 251 | 4.34E-35 | 51.85% | 3 | F:binding; F:nucleic acid binding; C:intracellular | IPR007087 (PFAM); IPR013087 (G3DSA:3.30.160.GENE3D); IPR015880 (SMART); PTHR11389 (PANTHER), PF13465 (PFAM), PF13894 (PFAM), SSF57667 (SUPERFAMILY) |
| 2302 | lib_dib_Filter15_IDBA_contig_40797_plus2 | 29 | low-density lipoprotein receptor-related protein 4 | 251 | 5.74E-116 | 78.30% | 22 | P:anatomical structure morphogenesis; P:embryo development; F:peptidase activity; C:cytoplasmic membrane-bounded vesicle; P:multicellular organismal development; P:signal transduction; P:cellular component organization; P:cell differentiation; P:growth; F:receptor binding; P:regulation of biological process; F:calcium ion binding; C:cell; P:biological_process; P:reproduction; P:cellular protein modification process; P:protein metabolic process; P:catabolic process; F:receptor activity; F:protein binding; C:plasma membrane; P:metabolic process | IPR000033 (PFAM); IPR011042 (G3DSA:2.120.10.GENE3D); PTHR10529 (PANTHER), SSF63825 (SUPERFAMILY) |
| 2303 | lib_dib_Filter15_IDBA_contig_40830_plus1 | 10897 | deleted in malignant brain tumors 1 | 690 | 1.38E-126 | 51.95% | 2 | F:receptor activity; C:cell | IPR001190 (PRINTS); IPR017448 (SMART); G3DSA:3.10.250.10 (GENE3D), PTHR19331 (PANTHER), PTHR19331:SF118 (PANTHER), SignalP-NN(euk) (SIGNALP) |
| 2304 | lib_dib_Filter15_IDBA_contig_40831_plus1 | 765 | deleted in malignant brain tumors 1 | 136 | 1.04E-28 | 61.60% | 2 | F:receptor activity; C:cell | IPR001190 (PRINTS); IPR017448 (SMART); G3DSA:3.10.250.10 (GENE3D), PTHR19331 (PANTHER), PTHR19331:SF118 (PANTHER) |
| 2305 | lib_dib_Filter15_IDBA_contig_4087_plus1 | 36874 | vitellogenin | 1145 | 1.77E-42 | 46.70% | 2 | F:transporter activity; P:transport | IPR001747 (PFAM); IPR001846 (PFAM); IPR011030 (G3DSA:1.25.10.GENE3D); IPR015255 (PFAM); IPR015817 (G3DSA:2.20.50.GENE3D); IPR015818 (G3DSA:2.20.80.GENE3D); IPR015819 (SUPERFAMILY); PTHR23345 (PANTHER) |
| 2306 | lib_dib_Filter15_IDBA_contig_40966_minus1 | 1499 | cadherin-23-like isoform 1 | 531 | 1.99E-64 | 48.20% | 3 | P:biological_process; F:calcium ion binding; C:plasma membrane | IPR002126 (PRINTS); IPR015919 (SUPERFAMILY); IPR020894 (PROSITE); PTHR24027 (PANTHER), PTHR24027:SF110 (PANTHER) |

| | | | | | | | | | | |
|---|---|---|---|---|---|---|---|---|---|---|
| 2307 | lib_dib_Filter15_IDBA_contig_40982_plus1 | 39 | neuronal cell adhesion molecule | 250 | 6.43E-26 | 49.00% | 9 | C:cell; P:anatomical structure morphogenesis; P:cellular component organization; P:cell differentiation; P:multicellular organismal development; P:biological_process; P:regulation of biological process; C:plasma membrane; F:protein binding | IPR003598 (SMART); IPR003599 (SMART); IPR007110 (PROFILE); IPR013783 (G3DSA:2.60.40.GENE3D); PTHR10489 (PANTHER), PF13895 (PFAM), SSF48726 (SUPERFAMILY) | |
| 2308 | lib_dib_Filter15_IDBA_contig_40993_plus2 | 22 | serine threonine-protein phosphatase 6 regulatory ankyrin repeat subunit b | 250 | 4.42E-93 | 77.85% | 2 | C:nucleoplasm; F:protein binding | IPR002110 (PRINTS); IPR020683 (G3DSA:1.25.40.GENE3D); PTHR24158 (PANTHER), PTHR24158:SF0 (PANTHER), PF13857 (PFAM) | |
| 2309 | lib_dib_Filter15_IDBA_contig_41007_plus3 | 334 | nucleobindin 2 precursor | 436 | 1.31E-91 | 72.40% | 6 | C:Golgi apparatus; C:nuclear envelope; C:cytoplasm; F:calcium ion binding; C:endoplasmic reticulum; F:receptor binding | IPR002048 (PROFILE); IPR011992 (G3DSA:1.10.238.GENE3D); IPR018247 (PROSITE); PTHR19237 (PANTHER), SignalP-NN(euk) (SIGNALP), SSF47473 (SUPERFAMILY) | |
| 2310 | lib_dib_Filter15_IDBA_contig_41062_minus1 | 103 | proto-oncogene tyrosine-protein kinase ros | 581 | 4.53E-112 | 53.80% | 4 | F:nucleotide binding; P:cellular protein modification process; F:protein binding; F:protein kinase activity | IPR000719 (PROFILE); IPR001245 (PRINTS); IPR002290 (SMART); IPR003961 (PFAM); IPR008266 (PROSITE); IPR011009 (SUPERFAMILY); IPR013783 (G3DSA:2.60.40.GENE3D); IPR017441 (PROSITE); IPR020635 (SMART); G3DSA:1.10.510.10 (GENE3D), G3DSA:3.30.200.20 (GENE3D), PTHR24416 (PANTHER), PTHR24416:SF4 (PANTHER), tmhmm (TMHMM) | |
| 2311 | lib_dib_Filter15_IDBA_contig_4107_minus1 | 503 | latrophilin cirl-like isoform 1 | 584 | 2.35E-32 | 77.85% | 4 | P:signal transduction; C:cell; C:plasma membrane; F:receptor activity | IPR000832 (PFAM); IPR017983 (PROSITE); PTHR12011 (PANTHER), PTHR12011:SF61 (PANTHER), SignalP-NN(euk) (SIGNALP), tmhmm (TMHMM) | |
| 2312 | lib_dib_Filter15_IDBA_contig_41128_minus2 | 33 | egf-like domain-containing protein | 274 | 2.79E-51 | 54.25% | 2 | F:protein binding; F:calcium ion binding | IPR000152 (PROSITE); IPR000742 (SMART); IPR000884 (PFAM); IPR001881 (PFAM); IPR013032 (PROSITE); IPR018097 (PROSITE); PD968187 (PRODOM), G3DSA:2.10.25.10 (GENE3D), G3DSA:2.20.100.10 (GENE3D), PTHR24048 (PANTHER), SSF57196 (SUPERFAMILY) | |
| 2313 | lib_dib_Filter15_IDBA_contig_41131_plus2 | 55 | leucine-rich repeat-containing protein 15-like | 249 | 3.33E-20 | 48.75% | 1 | F:protein binding | IPR001611 (PFAM); IPR003591 (SMART); PR00019 (PRINTS), G3DSA:3.80.10.10 (GENE3D), PTHR24365 (PANTHER), PF13855 (PFAM), SignalP-NN(euk) (SIGNALP), SSF52058 (SUPERFAMILY) | Yes |
| 2314 | lib_dib_Filter15_IDBA_contig_41218_plus1 | 20 | tectonic-like protein | 249 | 8.28E-71 | 54.90% | 0 | - | PTHR14611 (PANTHER), PTHR14611:SF0 (PANTHER) | |
| 2315 | lib_dib_Filter15_IDBA_contig_41249_plus2 | 17 | zinc finger x-linked protein zxdb | 249 | 1.79E-60 | 61.55% | 3 | F:binding; F:nucleic acid binding; C:intracellular | IPR007087 (PFAM); IPR013087 (G3DSA:3.30.160.GENE3D); IPR015880 (SMART); PTHR19818 (PANTHER), PTHR19818:SF18 (PANTHER), PF13465 (PFAM), PF13894 (PFAM), SSF57667 (SUPERFAMILY) | |
| 2316 | lib_dib_Filter15_IDBA_contig_41308_minus3 | 189 | cubilin precursor | 644 | 7.06E-19 | 45.00% | 7 | C:lysosome; C:organelle; C:intracellular; C:cell; F:protein binding; P:metabolic process; P:protein transport | IPR000859 (G3DSA:2.60.120.GENE3D); PTHR10127 (PANTHER), PTHR10127:SF75 (PANTHER), tmhmm (TMHMM) | |
| 2317 | lib_dib_Filter15_IDBA_contig_41309_minus3 | 52 | cubilin | 204 | 4.96E-12 | 57.80% | 15 | P:biosynthetic process; P:lipid metabolic process; C:cytoplasmic membrane-bounded vesicle; P:transport; P:cellular component organization; C:plasma membrane; C:cell; P:metabolic process; P:protein transport; F:protein binding; C:protein complex; F:receptor activity; C:lysosome; P:response to external stimulus; P:embryo development | IPR000859 (G3DSA:2.60.120.GENE3D); PTHR10127 (PANTHER), PTHR10127:SF310 (PANTHER) | |

| | | | | | | | | | | |
|---|---|---|---|---|---|---|---|---|---|---|
| 2318 | lib_dib_Filter15_IDBA_contig_41347_minus2 | 32 | serine threonine-protein kinase mrck alpha- partial | 248 | 2.32E-156 | 89.85% | 16 | P:biological_process; P:cytoskeleton organization; F:enzyme regulator activity; P:cellular protein modification process; F:protein kinase activity; P:anatomical structure morphogenesis; P:cellular component organization; F:lipid binding; F:protein binding; C:cell; F:nucleotide binding; C:plasma membrane; F:binding; P:signal transduction; C:cytoskeleton; C:cytoplasm | IPR000719 (PFAM); IPR002290 (SMART); IPR008271 (PROSITE); IPR011009 (SUPERFAMILY); IPR020635 (SMART); IPR026611 (PTHR22988:PANTHER); G3DSA:1.10.510.10 (GENE3D), G3DSA:3.30.200.20 (GENE3D), PTHR22988 (PANTHER) | |
| 2319 | lib_dib_Filter15_IDBA_contig_41376_plus3 | 3340 | neurogenic locus notch homolog protein 2-like | 515 | 2.43E-51 | 57.70% | 4 | C:extracellular region; F:protein binding; F:carbohydrate binding; F:calcium ion binding | IPR000152 (PROSITE); IPR000742 (PFAM); IPR001304 (PFAM); IPR001881 (SMART); IPR013032 (PFAM); IPR016186 (G3DSA:3.10.100.GENE3D); IPR016187 (SUPERFAMILY); IPR018097 (PROSITE); G3DSA:2.10.25.10 (GENE3D), PTHR24048 (PANTHER), PTHR24048:SF15 (PANTHER), SSF57196 (SUPERFAMILY) | |
| 2320 | lib_dib_Filter15_IDBA_contig_41395_minus1 | 38 | inactive dipeptidyl peptidase 10-like | 248 | 1.19E-90 | 59.20% | 4 | C:cell; F:peptidase activity; P:protein metabolic process; P:catabolic process | IPR002469 (PFAM); G3DSA:2.140.10.30 (GENE3D), PTHR11731 (PANTHER), SSF82171 (SUPERFAMILY) | |
| 2321 | lib_dib_Filter15_IDBA_contig_41414_plus2 | 127 | nicotinic acetylcholine receptor subunit alpha 6 transcript variant partial | 379 | 5.73E-48 | 55.05% | 3 | P:ion transport; C:cell; F:ion channel activity | IPR006201 (PRINTS); IPR006202 (G3DSA:2.70.170.GENE3D); IPR018000 (PROSITE); PTHR18945:SF127 (PANTHER), tmhmm (TMHMM) | |
| 2322 | lib_dib_Filter15_IDBA_contig_41415_plus1 | 51 | protein | 169 | 5.31E-26 | 57.45% | 3 | P:ion transport; C:cell; F:ion channel activity | IPR006201 (PANTHER); IPR006202 (G3DSA:2.70.170.GENE3D); IPR018000 (PROSITE); PTHR18945:SF127 (PANTHER), tmhmm (TMHMM) | |
| 2323 | lib_dib_Filter15_IDBA_contig_41417_plus2 | 357 | aminoacyl trna synthase complex-interacting multifunctional protein 1 isoform 1 | 377 | 1.65E-99 | 67.15% | 2 | P:biological_process; F:RNA binding | IPR002547 (PFAM); IPR012340 (G3DSA:2.40.50.GENE3D); PTHR11946 (PANTHER), PTHR11946:SF3 (PANTHER) | |
| 2324 | lib_dib_Filter15_IDBA_contig_41476_plus2 | 40 | fasciclin ii transmembrane isoform | 247 | 2.89E-42 | 47.80% | 1 | F:protein binding | IPR003598 (SMART); IPR003599 (SMART); IPR007110 (PROFILE); IPR013098 (PFAM); IPR013783 (G3DSA:2.60.40.GENE3D); PTHR10489 (PANTHER), SignalP-NN(euk) (SIGNALP), tmhmm (TMHMM), SSF48726 (SUPERFAMILY) | |
| 2325 | lib_dib_Filter15_IDBA_contig_41491_plus3 | 34 | er degradation-enhancing alpha-mannosidase-like 2-like | 247 | 2.53E-113 | 78.90% | 7 | F:calcium ion binding; P:biosynthetic process; P:cellular protein modification process; P:carbohydrate metabolic process; P:protein metabolic process; F:hydrolase activity; C:endoplasmic reticulum | IPR001382 (PRINTS); PTHR11742:SF10 (PANTHER) | |
| 2326 | lib_dib_Filter15_IDBA_contig_41573_plus1 | 1099 | exostosin-2 | 848 | 0 | 74.15% | 15 | C:Golgi apparatus; P:cell differentiation; P:multicellular organismal development; P:biosynthetic process; P:carbohydrate metabolic process; P:anatomical structure morphogenesis; P:embryo development; P:cellular component organization; C:extracellular space; P:signal transduction; C:cell; F:protein binding; F:transferase activity; P:regulation of biological process; C:endoplasmic reticulum | IPR004263 (PFAM); IPR015338 (PFAM); G3DSA:3.90.550.10 (GENE3D), PTHR11062 (PANTHER), PTHR11062:SF6 (PANTHER), SignalP-NN(euk) (SIGNALP), tmhmm (TMHMM), SSF53448 (SUPERFAMILY) | Yes |

| | | | | | | | | | | |
|---|---|---|---|---|---|---|---|---|---|---|
| 2327 | lib_dib_Filter15_IDBA_contig_41586_minus1 | 1130 | von willebrand factor type egf and pentraxin domain-containing protein 1-like | 914 | 3.60E-107 | 45.65% | 2 | F:protein binding; F:calcium ion binding | IPR000152 (PROSITE); IPR000436 (PFAM); IPR000742 (PROFILE); IPR000884 (PROFILE); IPR001881 (PFAM); IPR011641 (PFAM); IPR013032 (PROSITE); IPR018097 (PROSITE); G3DSA:2.10.25.10 (GENE3D), G3DSA:2.10.70.10 (GENE3D), PTHR19325 (PANTHER), SSF57196 (SUPERFAMILY), SSF57586 (SUPERFAMILY) | |
| 2328 | lib_dib_Filter15_IDBA_contig_41603_plus3 | 228 | 3-oxoacyl- | 360 | 3.99E-40 | 55.70% | 3 | P:metabolic process; F:catalytic activity; F:nucleotide binding | IPR002198 (PRINTS); IPR002347 (PRINTS); IPR016040 (G3DSA:3.40.50.GENE3D); IPR020904 (PROSITE); PTHR24322 (PANTHER), PTHR24322:SF0 (PANTHER), PF13561 (PFAM), SSF51735 (SUPERFAMILY) | |
| 2329 | lib_dib_Filter15_IDBA_contig_41624_plus2 | 61 | protocadherin fat 4 | 246 | 1.34E-88 | 53.25% | 3 | F:calcium ion binding; P:biological_process; C:cell | IPR002126 (G3DSA:2.60.40.GENE3D); IPR015919 (SUPERFAMILY); PTHR24026 (PANTHER) | |
| 2330 | lib_dib_Filter15_IDBA_contig_41649_plus1 | 51 | probable s-acyltransferase at2g14255-like | 247 | 8.69E-108 | 68.40% | 3 | F:binding; C:Golgi apparatus; F:protein binding | IPR002110 (PFAM); IPR020683 (G3DSA:1.25.40.GENE3D); PTHR24188 (PANTHER) | |
| 2331 | lib_dib_Filter15_IDBA_contig_41663_minus1 | 30 | penicillin-binding protein | 247 | 3.43E-38 | 56.05% | 1 | F:protein domain specific binding | IPR001466 (PFAM); IPR012338 (G3DSA:3.40.710.GENE3D); PTHR22935 (PANTHER), SignalP-NN(euk) (SIGNALP), tmhmm (TMHMM) | Yes |
| 2332 | lib_dib_Filter15_IDBA_contig_41665_minus3 | 28 | protocadherin-11 x-linked | 246 | 3.97E-26 | 58.90% | 3 | F:calcium ion binding; P:biological_process; C:cell | IPR002126 (PRINTS); IPR013164 (PFAM); PTHR24028 (PANTHER), PTHR24028:SF46 (PANTHER) | |
| 2333 | lib_dib_Filter15_IDBA_contig_41667_minus1 | 3851 | manganese superoxide dismutase | 762 | 1.09E-114 | 81.80% | 4 | P:biological_process; F:binding; P:metabolic process; F:catalytic activity | IPR001189 (PRINTS); IPR019831 (PFAM); IPR019832 (PFAM); IPR019833 (PROSITE); G3DSA:1.10.287.990 (GENE3D), PTHR11404:SF5 (PANTHER) | |
| 2334 | lib_dib_Filter15_IDBA_contig_41709_plus1 | 30 | fat-like cadherin-related tumor suppressor homolog | 246 | 5.21E-66 | 66.40% | 3 | F:calcium ion binding; P:biological_process; C:plasma membrane | IPR002126 (PRINTS); IPR015919 (SUPERFAMILY); IPR020894 (PROSITE); PTHR24027 (PANTHER), PTHR24027:SF110 (PANTHER) | |
| 2335 | lib_dib_Filter15_IDBA_contig_41781_minus2 | 46 | low-density lipoprotein receptor-related protein 4 | 246 | 3.89E-49 | 49.45% | 10 | P:anatomical structure morphogenesis; F:peptidase activity; F:protein binding; C:cell; P:regulation of biological process; P:signal transduction; P:biological_process; P:multicellular organismal development; P:cell differentiation; P:protein metabolic process | IPR002172 (G3DSA:4.10.400.GENE3D); IPR023415 (PROSITE); PR00261 (PRINTS), PTHR10529 (PANTHER) | |
| 2336 | lib_dib_Filter15_IDBA_contig_41825_plus1 | 30 | membrane-associated guanylate ww and pdz domain-containing protein 1-like | 246 | 2.26E-79 | 73.05% | 1 | F:protein binding | IPR001478 (PFAM); G3DSA:2.30.42.10 (GENE3D), PTHR10316 (PANTHER), PTHR10316:SF30 (PANTHER) | |
| 2337 | lib_dib_Filter15_IDBA_contig_41838_plus2 | 104 | transmembrane protease serine 9-like | 396 | 1.20E-59 | 56.70% | 4 | C:cell; F:peptidase activity; P:protein metabolic process; P:catabolic process | IPR001254 (PFAM); IPR001314 (PRINTS); IPR009003 (SUPERFAMILY); IPR018114 (PROSITE); G3DSA:2.40.10.10 (GENE3D), PTHR24256 (PANTHER), SignalP-NN(euk) (SIGNALP), tmhmm (TMHMM) | |
| 2338 | lib_dib_Filter15_IDBA_contig_41885_plus2 | 3115 | protein | 409 | 2.83E-27 | 54.45% | 4 | F:receptor binding; P:signal transduction; C:extracellular space; C:cellular_component | IPR002181 (PFAM); IPR014715 (G3DSA:4.10.530.GENE3D); IPR014716 (G3DSA:3.90.215.GENE3D); PTHR19143 (PANTHER), SignalP-NN(euk) (SIGNALP) | |
| 2339 | lib_dib_Filter15_IDBA_contig_41897_minus2 | 392 | translocase of inner mitochondrial membrane 8 homolog b | 604 | 5.88E-20 | 80.40% | 5 | F:binding; P:mitochondrion organization; P:protein transport; C:mitochondrion; C:protein complex | IPR000909 (PROFILE); IPR004217 (G3DSA:1.10.287.GENE3D); PTHR21535 (PANTHER), tmhmm (TMHMM) | |

| | | | | | | | | | | |
|---|---|---|---|---|---|---|---|---|---|---|
| 2340 | lib_dib_Filter15_IDBA_contig_41938_plus3 | 1068 | secreted frizzled-related protein 5 precursor | 915 | 1.15E-81 | 64.95% | 11 | P:signal transduction; F:protein binding; ; P:regulation of biological process; C:cell; P:anatomical structure morphogenesis; P:multicellular organismal development; P:growth; P:embryo development; P:response to stress; P:cellular protein modification process | IPR001134 (PROFILE); IPR008993 (SUPERFAMILY); IPR015526 (PANTHER); IPR018933 (PFAM); IPR020067 (G3DSA:1.10.2000.GENE3D); IPR026561 (PTHR11309:PANTHER); G3DSA:2.40.50.120 (GENE3D) | |
| 2341 | lib_dib_Filter15_IDBA_contig_42012_plus3 | 202 | lachesin | 776 | 5.13E-143 | 42.80% | 2 | P:multicellular organismal development; F:protein binding | IPR003598 (SMART); IPR003599 (SMART); IPR007110 (PROFILE); IPR013098 (PFAM); IPR013106 (PFAM); IPR013783 (G3DSA:2.60.40.GENE3D); PTHR10489 (PANTHER), PF13895 (PFAM), SSF48726 (SUPERFAMILY) | |
| 2342 | lib_dib_Filter15_IDBA_contig_42052_plus1 | 74 | ankyrin repeat | 285 | 1.12E-30 | 50.00% | 1 | F:protein binding | IPR002110 (SMART); IPR020683 (G3DSA:1.25.40.GENE3D); PTHR24188 (PANTHER), PF13637 (PFAM) | |
| 2343 | lib_dib_Filter15_IDBA_contig_42074_plus3 | 21 | protein | 244 | 1.08E-35 | 55.35% | 3 | C:extracellular region; F:protein binding; F:calcium ion binding | IPR000152 (PROSITE); IPR000436 (SMART); IPR000742 (PFAM); IPR001881 (PFAM); IPR009030 (SUPERFAMILY); IPR013032 (PROSITE); IPR018097 (PROSITE); PR00010 (PRINTS), G3DSA:2.10.25.10 (GENE3D), G3DSA:2.10.70.10 (GENE3D), PTHR24048 (PANTHER), PTHR24048:SF15 (PANTHER), SSF57196 (SUPERFAMILY) | |
| 2344 | lib_dib_Filter15_IDBA_contig_42075_minus2 | 34 | ankyrin repeat protein | 244 | 5.68E-32 | 52.45% | 1 | F:protein binding | IPR002110 (PRINTS); IPR020683 (G3DSA:1.25.40.GENE3D); PTHR24188 (PANTHER) | |
| 2345 | lib_dib_Filter15_IDBA_contig_42080_plus3 | 215 | mucin core | 316 | 2.18E-10 | 66.05% | 18 | C:lysosome; P:heterophilic cell-cell adhesion; C:endosome; C:plasma membrane; C:endosome membrane; C:integral to membrane; P:muscle organ development; C:lysosomal membrane; P:cell adhesion; P:multicellular organismal development; P:signal transduction; C:extracellular region; P:negative regulation of cell adhesion; F:protein binding; P:hemopoiesis; P:negative regulation of cell proliferation; C:integral to plasma membrane; P:immune response | IPR007947 (PTHR11337:PANTHER); PTHR11337 (PANTHER), SignalP-NN(euk) (SIGNALP), tmhmm (TMHMM) | Yes |
| 2346 | lib_dib_Filter15_IDBA_contig_42113_plus3 | 399 | epididymal secretory protein e1 precursor | 689 | 1.71E-33 | 61.65% | 6 | P:biological_process; C:lysosome; P:transport; F:protein binding; P:response to biotic stimulus; F:lipid binding | IPR003172 (G3DSA:2.60.40.GENE3D); IPR014756 (SUPERFAMILY); PTHR11306 (PANTHER), SignalP-NN(euk) (SIGNALP), tmhmm (TMHMM) | |
| 2347 | lib_dib_Filter15_IDBA_contig_42180_plus2 | 30 | leucine-rich repeat and fibronectin type iii domain-containing protein 1-like | 244 | 4.18E-27 | 44.50% | 1 | F:protein binding | IPR000483 (SMART); IPR003598 (SMART); IPR003599 (SMART); IPR007110 (PROFILE); IPR013098 (PFAM); IPR013783 (G3DSA:2.60.40.GENE3D); G3DSA:3.80.10.10 (GENE3D), PTHR24373 (PANTHER), SSF48726 (SUPERFAMILY), SSF52058 (SUPERFAMILY) | |

| | | | | | | | | | |
|---|---|---|---|---|---|---|---|---|---|
| 2348 | lib_dib_Filter15_IDBA_contig_4220_minus2 | 584 | calcium calmodulin-dependent protein kinase type ii alpha chain-like isoform 1 | 1424 | 0 | 90.35% | 16 | P:regulation of biological process; F:protein kinase activity; P:reproduction; C:cellular_component; P:behavior; C:cell; P:cellular component organization; P:cell differentiation; P:multicellular organismal development; P:cellular protein modification process; P:cell-cell signaling; C:plasma membrane; F:protein binding; F:nucleotide binding; C:intracellular; C:protein complex | IPR000719 (PFAM); IPR002290 (SMART); IPR008271 (PROSITE); IPR011009 (SUPERFAMILY); IPR013543 (PFAM); IPR017441 (PROSITE); IPR020635 (SMART); IPR020636 (PANTHER); G3DSA:1.10.510.10 (GENE3D), G3DSA:3.10.450.50 (GENE3D), G3DSA:3.30.200.20 (GENE3D), PTHR24347:SF13 (PANTHER), tmhmm (TMHMM), SSF54427 (SUPERFAMILY) |
| 2349 | lib_dib_Filter15_IDBA_contig_42314_plus2 | 23 | corticosteroid 11-beta-dehydrogenase isozyme 1 | 243 | 2.33E-27 | 57.95% | 3 | F:catalytic activity; P:metabolic process; F:nucleotide binding | IPR002198 (PFAM); IPR016040 (G3DSA:3.40.50.GENE3D); IPR020904 (PROSITE); PTHR24322 (PANTHER), PTHR24322:SF32 (PANTHER), SSF51735 (SUPERFAMILY) |
| 2350 | lib_dib_Filter15_IDBA_contig_42377_plus1 | 331 | lysosomal alpha-mannosidase | 341 | 2.40E-99 | 64.95% | 3 | F:carbohydrate binding; P:carbohydrate metabolic process; F:hydrolase activity | IPR011013 (SUPERFAMILY); IPR011682 (PFAM); G3DSA:2.60.40.1360 (GENE3D), G3DSA:2.70.98.30 (GENE3D), PTHR11607 (PANTHER), PTHR11607:SF3 (PANTHER) |
| 2351 | lib_dib_Filter15_IDBA_contig_42417_plus3 | 34 | dual serine threonine and tyrosine protein kinase-like | 281 | 1.60E-168 | 86.70% | 4 | C:cytoplasm; F:protein kinase activity; P:cellular protein modification process; F:nucleotide binding | IPR000719 (PFAM); IPR002290 (SMART); IPR008271 (PROSITE); IPR011009 (SUPERFAMILY); IPR017441 (PROSITE); IPR020635 (SMART); G3DSA:1.10.510.10 (GENE3D), G3DSA:3.30.200.20 (GENE3D), PTHR13902 (PANTHER), PTHR13902:SF2 (PANTHER) |
| 2352 | lib_dib_Filter15_IDBA_contig_42441_minus1 | 22 | inactive dipeptidyl peptidase 10 | 243 | 3.62E-35 | 52.35% | 4 | F:peptidase activity; C:cell; P:protein metabolic process; P:catabolic process | IPR002469 (PFAM); G3DSA:2.140.10.30 (GENE3D), PTHR11731 (PANTHER), PTHR11731:SF24 (PANTHER), SSF82171 (SUPERFAMILY) |
| 2353 | lib_dib_Filter15_IDBA_contig_42485_minus2 | 736 | tumor necrosis factor receptor superfamily member 27 | 347 | 1.68E-11 | 41.00% | 1 | F:protein binding | IPR001368 (PROSITE); G3DSA:2.10.50.10 (GENE3D), PTHR12120 (PANTHER), PTHR12120:SF3 (PANTHER), tmhmm (TMHMM) |
| 2354 | lib_dib_Filter15_IDBA_contig_42499_minus2 | 276 | loc432087 protein | 269 | 9.11E-109 | 81.65% | 6 | C:cell; F:binding; P:carbohydrate metabolic process; P:catabolic process; C:lysosome; F:hydrolase activity | IPR000933 (PANTHER); IPR013781 (G3DSA:3.20.20.GENE3D); IPR016286 (PRINTS); IPR017853 (SUPERFAMILY); PTHR10030:SF2 (PANTHER), SignalP-NN(euk) (SIGNALP), tmhmm (TMHMM) |
| 2355 | lib_dib_Filter15_IDBA_contig_42504_minus1 | 26 | inactive hydroxysteroid dehydrogenase-like protein 1 | 242 | 6.84E-70 | 69.45% | 3 | P:metabolic process; F:catalytic activity; F:nucleotide binding | IPR002198 (PRINTS); IPR002347 (PRINTS); IPR016040 (G3DSA:3.40.50.GENE3D); PTHR24316 (PANTHER), PTHR24316:SF68 (PANTHER), SSF51735 (SUPERFAMILY) |
| 2356 | lib_dib_Filter15_IDBA_contig_42545_minus2 | 30 | notch | 242 | 2.15E-14 | 62.25% | 2 | F:protein binding; F:calcium ion binding | IPR000152 (PROSITE); IPR000742 (PFAM); IPR001881 (SMART); IPR013032 (PROSITE); G3DSA:2.10.25.10 (GENE3D), PTHR24044 (PANTHER), PTHR24044:SF49 (PANTHER), tmhmm (TMHMM), SSF57196 (SUPERFAMILY) |
| 2357 | lib_dib_Filter15_IDBA_contig_42621_minus1 | 37 | nidogen and egf-like domain-containing protein 1 | 242 | 3.90E-07 | 50.25% | 1 | F:protein binding | IPR000436 (PFAM); IPR000742 (PROFILE); IPR013032 (PROSITE); G3DSA:2.10.25.10 (GENE3D), G3DSA:2.10.70.10 (GENE3D), PTHR24033 (PANTHER), SSF57196 (SUPERFAMILY) |
| 2358 | lib_dib_Filter15_IDBA_contig_42689_plus2 | 43 | procollagen c-endopeptidase enhancer | 241 | 7.66E-27 | 48.55% | 2 | F:binding; F:hydrolase activity | IPR000859 (G3DSA:2.60.120.GENE3D); PTHR10127 (PANTHER), SignalP-NN(euk) (SIGNALP), tmhmm (TMHMM) |

| | | | | | | | | | | |
|---|---|---|---|---|---|---|---|---|---|---|
| 2359 | lib_dib_Filter15_IDBA_contig_42692_plus2 | 91 | stabilin-2 | 398 | 4.49E-60 | 48.30% | 3 | C:cell; F:protein binding; F:calcium ion binding | IPR000742 (SMART); IPR000782 (G3DSA:2.30.180.GENE3D); IPR001881 (SMART); IPR013032 (PROSITE); IPR023413 (G3DSA:2.40.155.GENE3D); IPR024731 (PFAM); G3DSA:2.10.25.10 (GENE3D), PTHR24038 (PANTHER), PTHR24038:SF0 (PANTHER), SSF57196 (SUPERFAMILY) | |
| 2360 | lib_dib_Filter15_IDBA_contig_42718_plus1 | 98 | tissue inhibitor of metalloproteinase 3 (sorsby fundus pseudoinflammatory) isoform cra_a | 241 | 2.62E-04 | 46.80% | 2 | F:protein binding; F:enzyme regulator activity | IPR001134 (PROFILE); IPR001820 (PANTHER); IPR008993 (SUPERFAMILY); G3DSA:2.40.50.120 (GENE3D), PTHR11844:SF9 (PANTHER), SignalP-NN(euk) (SIGNALP), tmhmm (TMHMM) | Yes |
| 2361 | lib_dib_Filter15_IDBA_contig_42719_plus1 | 65 | gli pathogenesis-related 1 -like | 430 | 2.98E-65 | 56.00% | 1 | C:extracellular region | IPR001283 (PRINTS); IPR002413 (PRINTS); IPR014044 (G3DSA:3.40.33.GENE3D); IPR018244 (PROSITE); PTHR10334:SF12 (PANTHER) | |
| 2362 | lib_dib_Filter15_IDBA_contig_42788_minus1 | 42 | 72 kda type iv collagenase | 241 | 8.00E-11 | 50.25% | 14 | F:peptidase activity; P:response to endogenous stimulus; P:response to external stimulus; P:regulation of biological process; P:multicellular organismal development; P:reproduction; C:cytoplasm; P:biological_process; P:response to abiotic stimulus; P:anatomical structure morphogenesis; P:metabolic process; C:extracellular region; F:binding; P:response to stress | IPR000562 (G3DSA:2.10.10.GENE3D); IPR013806 (SUPERFAMILY); PR00013 (PRINTS), PTHR22918 (PANTHER), PTHR22918:SF0 (PANTHER), SignalP-NN(euk) (SIGNALP) | |
| 2363 | lib_dib_Filter15_IDBA_contig_42801_plus2 | 25 | protocadherin-16-like | 241 | 3.30E-74 | 61.50% | 3 | F:calcium ion binding; P:biological_process; C:plasma membrane | IPR002126 (PRINTS); IPR015919 (SUPERFAMILY); IPR020894 (PROSITE); PTHR24027 (PANTHER) | |
| 2364 | lib_dib_Filter15_IDBA_contig_42811_plus1 | 41 | acyl- -binding domain-containing protein 6 | 305 | 8.01E-71 | 65.65% | 3 | C:intracellular; F:lipid binding; F:protein binding | IPR000582 (PRINTS); IPR002110 (SMART); IPR014352 (G3DSA:1.20.80.GENE3D); IPR020683 (G3DSA:1.25.40.GENE3D); PTHR24119 (PANTHER), PTHR24119:SF0 (PANTHER) | |
| 2365 | lib_dib_Filter15_IDBA_contig_42812_plus1 | 42 | low-density lipoprotein receptor-related protein 6 isoform 2 | 241 | 1.08E-123 | 84.95% | 37 | P:reproduction; P:anatomical structure morphogenesis; P:multicellular organismal development; C:cell; C:endosome; P:biological_process; P:cell differentiation; P:regulation of biological process; P:cell-cell signaling; C:protein complex; F:protein binding; P:embryo development; P:response to external stimulus; P:cell proliferation; F:receptor activity; P:response to endogenous stimulus; C:cellular_component; F:receptor binding; P:cell death; P:transport; P:cellular component organization; ; P:carbohydrate metabolic process; P:catabolic process; C:endoplasmic reticulum; P:signal transduction; C:organelle; C:cytoplasm; C:mitochondrion; F:transporter activity; P:cellular homeostasis; P:biosynthetic process; P:lipid metabolic process; F:enzyme regulator activity; P:organelle organization; P:cell cycle; C:plasma | IPR000033 (PFAM); IPR011042 (G3DSA:2.120.10.GENE3D); G3DSA:2.10.25.10 (GENE3D), PTHR10529 (PANTHER), SSF57196 (SUPERFAMILY), SSF63825 (SUPERFAMILY) | |
| 2366 | lib_dib_Filter15_IDBA_contig_42870_minus2 | 20 | gcc2 and gcc3 family protein | 240 | 2.78E-13 | 46.30% | 2 | F:protein binding; F:binding | IPR000742 (PROFILE); IPR007087 (PROSITE); IPR009030 (SUPERFAMILY); IPR011641 (PFAM); IPR013032 (PROSITE); G3DSA:2.10.25.10 (GENE3D), PTHR24046 (PANTHER), tmhmm (TMHMM), SSF57196 (SUPERFAMILY) | |

| | | | | | | | | | |
|---|---|---|---|---|---|---|---|---|---|
| 2367 | lib_dib_Filter15_IDBA_contig_42900_plus1 | 26 | gamma-aminobutyric acid type b receptor subunit 1 isoform 2 | 240 | 5.95E-45 | 73.95% | 17 | C:cell; P:regulation of biological process; P:nucleobase-containing compound metabolic process; P:biosynthetic process; P:cell differentiation; P:multicellular organismal development; P:transport; F:receptor activity; P:biological_process; C:endoplasmic reticulum; C:mitochondrion; P:cell proliferation; P:signal transduction; P:cell-cell signaling; C:cytoplasmic membrane-bounded vesicle; C:plasma membrane; F:protein binding | IPR001828 (PFAM); IPR002455 (PRINTS); IPR002456 (PRINTS); G3DSA:3.40.50.2300 (GENE3D), PTHR10519 (PANTHER), PTHR10519:SF3 (PANTHER), SignalP-NN(euk) (SIGNALP), tmhmm (TMHMM), SSF53822 (SUPERFAMILY) |
| 2368 | lib_dib_Filter15_IDBA_contig_42948_plus1 | 31 | signaling partial | 240 | 1.37E-48 | 76.30% | 4 | P:multicellular organismal development; C:proteinaceous extracellular matrix; F:receptor binding; P:signal transduction | IPR005817 (PANTHER) |
| 2369 | lib_dib_Filter15_IDBA_contig_43004_plus2 | 268 | matrix metalloproteinase-16-like | 371 | 1.29E-57 | 61.00% | 5 | F:peptidase activity; P:protein metabolic process; P:catabolic process; F:binding; C:extracellular region | IPR001818 (PFAM); IPR002477 (PFAM); IPR006026 (SMART); IPR021190 (PRINTS); IPR024079 (G3DSA:3.40.390.GENE3D); PTHR10201 (PANTHER), SSF55486 (SUPERFAMILY) |
| 2370 | lib_dib_Filter15_IDBA_contig_43027_plus2 | 38 | polycomb protein eed-like | 239 | 4.59E-126 | 81.95% | 19 | P:nucleobase-containing compound metabolic process; F:transferase activity; P:cellular protein modification process; P:organelle organization; C:nucleoplasm; C:protein complex; C:cell; P:regulation of biological process; ; C:nuclear chromosome; F:protein binding; C:nucleus; F:chromatin binding; F:hydrolase activity; F:transporter activity; P:regulation of gene expression, epigenetic; P:ion transport; F:binding; F:nucleotide binding | IPR001680 (PFAM); IPR015943 (G3DSA:2.130.10.GENE3D); IPR017986 (PROFILE); IPR019775 (PROSITE); PTHR10253 (PANTHER), PTHR10253:SF4 (PANTHER) |
| 2371 | lib_dib_Filter15_IDBA_contig_43037_minus1 | 68 | pdz domain containing 11 | 240 | 1.06E-48 | 67.00% | 3 | F:protein binding; C:cytosol; C:plasma membrane | IPR001478 (PFAM); G3DSA:2.30.42.10 (GENE3D), PTHR14063 (PANTHER), PTHR14063:SF1 (PANTHER) |
| 2372 | lib_dib_Filter15_IDBA_contig_43069_plus3 | 41 | ephrin type-a receptor 4-a-like | 239 | 3.10E-114 | 81.25% | 23 | P:anatomical structure morphogenesis; P:multicellular organismal development; P:biological_process; P:cellular protein modification process; P:regulation of biological process; C:cellular_component; P:signal transduction; F:protein kinase activity; F:receptor activity; F:hydrolase activity; F:receptor binding; P:cell death; C:cell; P:embryo development; F:molecular_function; P:behavior; P:response to external stimulus; P:cellular component organization; P:cell differentiation; P:cell recognition; P:cell-cell signaling; C:plasma membrane; F:nucleotide binding | IPR000719 (PROFILE); IPR001245 (PRINTS); IPR001660 (SMART); IPR011009 (SUPERFAMILY); IPR013761 (G3DSA:1.10.150.GENE3D); IPR020635 (SMART); IPR021129 (PFAM); G3DSA:1.10.510.10 (GENE3D), PTHR24416 (PANTHER), PTHR24416:SF122 (PANTHER) |
| 2373 | lib_dib_Filter15_IDBA_contig_43277_plus1 | 390 | interleukin-18 receptor 1 | 620 | 3.18E-11 | 45.00% | 3 | C:cell; F:protein binding; P:signal transduction | IPR000157 (PFAM); IPR007110 (PROFILE); IPR015621 (PANTHER); PR01537 (PRINTS), G3DSA:3.40.50.10140 (GENE3D), tmhmm (TMHMM), SSF48726 (SUPERFAMILY) |
| 2374 | lib_dib_Filter15_IDBA_contig_43286_minus3 | 2030 | apolipoprotein o-like | 647 | 1.60E-34 | 48.30% | 5 | C:mitochondrion; F:molecular_function; P:biological_process; C:mitochondrial inner membrane; C:extracellular region | IPR019166 (PFAM); PTHR14564 (PANTHER), PTHR14564:SF0 (PANTHER), tmhmm (TMHMM) |

| | | | | | | | | | | |
|---|---|---|---|---|---|---|---|---|---|---|
| 2375 | lib_dib_Filter15_IDBA_contig_43312_minus2 | 20 | serum paraoxonase arylesterase 2-like | 238 | 8.37E-36 | 54.20% | 1 | F:hydrolase activity | IPR002640 (PRINTS); IPR011042 (G3DSA:2.120.10.GENE3D); PTHR11799 (PANTHER), PTHR11799:SF0 (PANTHER), SSF63829 (SUPERFAMILY) | |
| 2376 | lib_dib_Filter15_IDBA_contig_43349_minus1 | 148 | low-density lipoprotein receptor-related protein 1b | 636 | 2.65E-66 | 51.75% | 2 | C:cell; F:protein binding | IPR000082 (PFAM); IPR002172 (G3DSA:4.10.400.GENE3D); IPR023415 (PROSITE); PR00261 (PRINTS), PTHR10529 (PANTHER) | |
| 2377 | lib_dib_Filter15_IDBA_contig_43358_minus2 | 738 | probable serine carboxypeptidase cpvl-like | 594 | 1.97E-177 | 73.35% | 3 | F:peptidase activity; P:protein metabolic process; P:catabolic process | IPR001563 (PRINTS); IPR018202 (PROSITE); G3DSA:3.40.50.1820 (GENE3D), tmhmm (TMHMM), SSF53474 (SUPERFAMILY) | |
| 2378 | lib_dib_Filter15_IDBA_contig_43377_plus1 | 62 | l-xylulose reductase | 238 | 1.53E-61 | 65.05% | 9 | P:carbohydrate metabolic process; P:metabolic process; P:nucleobase-containing compound metabolic process; P:secondary metabolic process; C:cell; F:catalytic activity; F:protein binding; P:cellular component organization; F:nucleotide binding | IPR002198 (PRINTS); IPR002347 (PRINTS); IPR016040 (G3DSA:3.40.50.GENE3D); PTHR24311 (PANTHER), PTHR24311:SF0 (PANTHER), SSF51735 (SUPERFAMILY) | |
| 2379 | lib_dib_Filter15_IDBA_contig_43419_minus1 | 32 | zinc finger protein 271-like | 238 | 2.20E-75 | 65.55% | 3 | F:binding; F:nucleic acid binding; C:intracellular | IPR007087 (PROSITE); IPR013087 (G3DSA:3.30.160.GENE3D); IPR015880 (SMART); PTHR24387 (PANTHER), PF13465 (PFAM), PF13912 (PFAM), SSF57667 (SUPERFAMILY) | |
| 2380 | lib_dib_Filter15_IDBA_contig_43434_minus2 | 24 | dbh-like 1 | 237 | 1.27E-59 | 58.70% | 3 | F:catalytic activity; ; P:metabolic process | IPR000945 (PANTHER); IPR008977 (SUPERFAMILY); IPR014784 (G3DSA:2.60.120.GENE3D); PF03712 (PFAM) | |
| 2381 | lib_dib_Filter15_IDBA_contig_4347_minus2 | 8428 | ---NA--- | 2622 | | | 0 | - | PTHR24023 (PANTHER), PTHR24023:SF106 (PANTHER), SignalP-NN(euk) (SIGNALP) | Yes |
| 2382 | lib_dib_Filter15_IDBA_contig_4347_plus1 | 8428 | ---NA--- | 2622 | | | 0 | - | no IPS match | |
| 2383 | lib_dib_Filter15_IDBA_contig_4347_plus3 | 8428 | ---NA--- | 2621 | | | 0 | - | IPR008160 (PFAM); PTHR24023 (PANTHER), tmhmm (TMHMM) | |
| 2384 | lib_dib_Filter15_IDBA_contig_43520_minus1 | 130 | dipeptidyl peptidase 1 | 444 | 5.57E-163 | 66.55% | 6 | C:lysosome; P:protein metabolic process; F:binding; F:peptidase activity; P:biological_process; P:catabolic process | IPR000169 (PROSITE); IPR000668 (PRINTS); IPR013128 (PANTHER); IPR014882 (PFAM); IPR025660 (PROSITE); IPR025661 (PROSITE); G3DSA:2.40.128.80 (GENE3D), G3DSA:3.90.70.10 (GENE3D), PTHR12411:SF17 (PANTHER), SignalP-NN(euk) (SIGNALP), SSF54001 (SUPERFAMILY), SSF75001 (SUPERFAMILY) | Yes |
| 2385 | lib_dib_Filter15_IDBA_contig_43563_plus1 | 33 | prolow-density lipoprotein receptor-related protein 1 | 237 | 5.51E-32 | 45.85% | 6 | P:biological_process; F:protein binding; C:cell; P:regulation of biological process; P:transport; C:cytoplasm | IPR000742 (SMART); IPR013032 (PROSITE); PD936484 (PRODOM), G3DSA:2.10.25.10 (GENE3D), PTHR24044 (PANTHER), tmhmm (TMHMM), SSF57196 (SUPERFAMILY) | |
| 2386 | lib_dib_Filter15_IDBA_contig_43564_plus1 | 23 | zinc finger protein 484 | 237 | 2.15E-26 | 50.00% | 3 | F:binding; F:nucleic acid binding; C:intracellular | IPR007087 (PFAM); IPR013087 (G3DSA:3.30.160.GENE3D); IPR015880 (SMART); PTHR24402 (PANTHER), PTHR24402:SF3 (PANTHER), PF13465 (PFAM), PF13894 (PFAM), SSF57667 (SUPERFAMILY) | |
| 2387 | lib_dib_Filter15_IDBA_contig_43568_minus3 | 4510 | retinoid-inducible serine carboxypeptidase | 609 | 5.51E-118 | 60.80% | 6 | P:lipid metabolic process; P:secondary metabolic process; C:cytosol; F:peptidase activity; P:protein metabolic process; P:catabolic process | IPR001563 (PRINTS); G3DSA:3.40.50.1820 (GENE3D), PTHR11802:SF3 (PANTHER), SSF53474 (SUPERFAMILY) | |
| 2388 | lib_dib_Filter15_IDBA_contig_43577_plus2 | 2068 | na subunit | 614 | 3.20E-162 | 65.15% | 3 | F:ion channel activity; P:ion transport; C:cell | IPR002394 (PRINTS); IPR006029 (PFAM); IPR006201 (PRINTS); IPR006202 (G3DSA:2.70.170.GENE3D); IPR018000 (PROSITE); G3DSA:1.20.120.370 (GENE3D), PTHR18945:SF92 (PANTHER), SignalP-NN(euk) (SIGNALP), tmhmm (TMHMM) | |

| | | | | | | | | | | |
|---|---|---|---|---|---|---|---|---|---|---|
| 2389 | lib_dib_Filter15_IDBA_contig_43592_plus2 | 23 | slit homolog 2 protein | 237 | 6.39E-21 | 56.10% | 21 | P:regulation of biological process; P:cell death; C:cell; P:behavior; P:response to external stimulus; P:cell differentiation; P:multicellular organismal development; P:signal transduction; F:protein binding; P:biological_process; F:carbohydrate binding; P:cellular component organization; P:anatomical structure morphogenesis; C:extracellular space; P:response to endogenous stimulus; P:cellular protein modification process; F:receptor binding; P:cytoskeleton organization; C:cytoplasm; F:enzyme regulator activity; P:cell growth | IPR003591 (SMART); G3DSA:3.80.10.10 (GENE3D), PTHR24365 (PANTHER), PF13855 (PFAM), SignalP-NN(euk) (SIGNALP), tmhmm (TMHMM), SSF52058 (SUPERFAMILY) | Yes |
| 2390 | lib_dib_Filter15_IDBA_contig_43669_plus1 | 219 | dysferlin-interacting protein 1 | 412 | 1.54E-63 | 70.70% | 3 | F:protein binding; P:metabolic process; P:regulation of biological process | IPR002110 (SMART); IPR020683 (G3DSA:1.25.40.GENE3D); PTHR24140 (PANTHER) | |
| 2391 | lib_dib_Filter15_IDBA_contig_4371_minus1 | 3466 | ubiquitin c | 204 | 8.81E-40 | 100.00% | 22 | P:cell cycle; P:signal transduction; P:biological_process; P:response to stress; P:DNA metabolic process; P:reproduction; P:viral reproduction; C:cytosol; C:nucleoplasm; P:protein metabolic process; P:catabolic process; P:regulation of biological process; P:cell death; F:protein binding; C:plasma membrane; ; C:endosome; P:cellular component organization; C:cytoplasmic membrane-bounded vesicle; P:nucleobase-containing compound metabolic process; P:cellular protein modification process; P:transport | IPR000626 (PFAM); IPR019954 (PROSITE); IPR019955 (PROFILE); IPR019956 (PRINTS); G3DSA:3.10.20.90 (GENE3D), PTHR10666 (PANTHER), SSF54236 (SUPERFAMILY) | |
| 2392 | lib_dib_Filter15_IDBA_contig_43711_minus3 | 34 | gastric triacylglycerol lipase | 236 | 2.26E-36 | 66.70% | 3 | P:lipid metabolic process; P:metabolic process; F:hydrolase activity | G3DSA:3.40.50.1820 (GENE3D), PTHR11005 (PANTHER), PTHR11005:SF0 (PANTHER), SSF53474 (SUPERFAMILY) | |
| 2393 | lib_dib_Filter15_IDBA_contig_43719_minus3 | 303 | fibropellin-1- partial | 355 | 5.51E-61 | 66.65% | 3 | C:proteinaceous extracellular matrix; F:protein binding; F:calcium ion binding | IPR000152 (PROSITE); IPR000742 (PFAM); IPR001881 (SMART); IPR013032 (PROSITE); IPR018097 (PROSITE); PR00010 (PRINTS), G3DSA:2.10.25.10 (GENE3D), PTHR24838 (PANTHER), PTHR24838:SF112 (PANTHER), tmhmm (TMHMM), SSF57196 (SUPERFAMILY) | |
| 2394 | lib_dib_Filter15_IDBA_contig_43720_minus2 | 1141 | epidermal growth factor | 105 | 3.05E-28 | 71.25% | 6 | C:proteinaceous extracellular matrix; C:cell; C:organelle; C:cytoplasm; F:protein binding; F:calcium ion binding | IPR000152 (PROSITE); IPR000742 (PFAM); IPR001881 (SMART); IPR013032 (PFAM); IPR018097 (PROSITE); PR00010 (PRINTS), G3DSA:2.10.25.10 (GENE3D), PTHR24044 (PANTHER), SSF57196 (SUPERFAMILY) | |
| 2395 | lib_dib_Filter15_IDBA_contig_43721_minus3 | 88 | fibropellin partial | 124 | 1.01E-30 | 69.20% | 5 | C:proteinaceous extracellular matrix; C:organelle; C:cytoplasm; F:protein binding; F:calcium ion binding | IPR000152 (PROSITE); IPR000742 (PFAM); IPR001881 (SMART); IPR013032 (PFAM); IPR018097 (PROSITE); PR00010 (PRINTS), G3DSA:2.10.25.10 (GENE3D), PTHR24044 (PANTHER), SSF57196 (SUPERFAMILY) | |
| 2396 | lib_dib_Filter15_IDBA_contig_43725_plus3 | 527 | cubilin- partial | 882 | 2.94E-15 | 58.10% | 9 | P:transport; F:protein binding; P:biosynthetic process; P:lipid metabolic process; C:organelle; C:intracellular; C:cell; C:plasma membrane; C:cytoplasm | IPR000859 (G3DSA:2.60.120.GENE3D); PTHR10127 (PANTHER), SignalP-NN(euk) (SIGNALP), tmhmm (TMHMM) | Yes |
| 2397 | lib_dib_Filter15_IDBA_contig_43726_plus1 | 531 | cubilin- partial | 891 | 2.82E-15 | 58.15% | 9 | P:transport; F:protein binding; P:biosynthetic process; P:lipid metabolic process; C:organelle; C:intracellular; C:cell; C:plasma membrane; C:cytoplasm | IPR000859 (G3DSA:2.60.120.GENE3D); PTHR10127 (PANTHER), SignalP-NN(euk) (SIGNALP), tmhmm (TMHMM) | Yes |

| | | | | | | | | | | |
|---|---|---|---|---|---|---|---|---|---|---|
| 2398 | lib_dib_Filter15_IDBA_contig_43741_plus1 | 24 | tolloid-like protein 2 | 236 | 7.00E-21 | 57.30% | 1 | F:hydrolase activity | IPR000859 (G3DSA:2.60.120.GENE3D); PTHR10127 (PANTHER), PTHR10127:SF310 (PANTHER) | |
| 2399 | lib_dib_Filter15_IDBA_contig_43814_plus3 | 227 | group xv phospholipase a2 | 452 | 8.21E-155 | 68.20% | 8 | C:organelle; C:intracellular; F:lipid binding; P:catabolic process; P:lipid metabolic process; C:cytoplasm; F:hydrolase activity; F:transferase activity | IPR003386 (PANTHER); G3DSA:3.40.50.1820 (GENE3D), PTHR11440:SF14 (PANTHER), SignalP-NN(euk) (SIGNALP), SSF53474 (SUPERFAMILY) | Yes |
| 2400 | lib_dib_Filter15_IDBA_contig_43847_plus3 | 2478 | fibulin 1 | 1071 | 0 | 49.00% | 4 | P:regulation of biological process; C:extracellular region; F:protein binding; F:calcium ion binding | IPR000020 (PROSITE); IPR000152 (PROSITE); IPR000742 (SMART); IPR001881 (PFAM); IPR013032 (PROSITE); IPR018097 (PROSITE); IPR026823 (PFAM); G3DSA:2.10.25.10 (GENE3D), PTHR24048 (PANTHER), PTHR24048:SF10 (PANTHER), SignalP-NN(euk) (SIGNALP), SSF57196 (SUPERFAMILY) | Yes |
| 2401 | lib_dib_Filter15_IDBA_contig_43849_plus1 | 112 | protein fbl- isoform a | 112 | 1.22E-22 | 53.50% | 5 | P:biological_process; P:regulation of biological process; P:metabolic process; C:proteinaceous extracellular matrix; F:calcium ion binding | IPR001881 (SMART); IPR013032 (PROSITE); IPR018097 (PROSITE); IPR026823 (PFAM); G3DSA:2.10.25.10 (GENE3D), PTHR24048 (PANTHER), PTHR24048:SF0 (PANTHER), SSF57196 (SUPERFAMILY) | |
| 2402 | lib_dib_Filter15_IDBA_contig_43862_plus1 | 3799 | endothelin-converting enzyme 1 | 805 | 4.53E-24 | 40.50% | 3 | F:peptidase activity; P:protein metabolic process; P:catabolic process | IPR000718 (PANTHER); IPR008753 (PFAM); IPR024079 (G3DSA:3.40.390.GENE3D); SignalP-NN(euk) (SIGNALP), tmhmm (TMHMM), SSF55486 (SUPERFAMILY) | |
| 2403 | lib_dib_Filter15_IDBA_contig_43866_plus2 | 25 | zinc finger protein 271 (zinc finger protein 7) (zinc finger protein znfphex133) (epstein-barr virus-induced zinc finger protein) (znf-eb) (ct-zfp48) (zinc finger protein | 235 | 4.60E-11 | 54.80% | 3 | F:binding; F:nucleic acid binding; C:intracellular | IPR007087 (PROSITE); IPR013087 (G3DSA:3.30.160.GENE3D); IPR015880 (SMART); PTHR24375 (PANTHER), PTHR24375:SF0 (PANTHER), PF13465 (PFAM), SSF57667 (SUPERFAMILY) | |
| 2404 | lib_dib_Filter15_IDBA_contig_43898_plus2 | 53 | homolog subfamily c member 12 | 235 | 1.65E-44 | 62.35% | 3 | F:unfolded protein binding; P:protein folding; F:heat shock protein binding | IPR001623 (PRINTS); PTHR24078 (PANTHER), PTHR24078:SF20 (PANTHER) | |
| 2405 | lib_dib_Filter15_IDBA_contig_43934_plus2 | 117 | fibrinogen c domain-containing protein 1-like | 296 | 1.44E-47 | 55.95% | 3 | F:receptor binding; C:extracellular space; P:signal transduction | IPR002181 (PFAM); IPR014715 (G3DSA:4.10.530.GENE3D); IPR014716 (G3DSA:3.90.215.GENE3D); IPR020837 (PROSITE); PTHR19143 (PANTHER) | |
| 2406 | lib_dib_Filter15_IDBA_contig_43935_plus1 | 365 | fibrinogen c domain-containing protein 1-like | 603 | 1.56E-46 | 57.00% | 3 | F:receptor binding; C:extracellular space; P:signal transduction | IPR002181 (PFAM); IPR014715 (G3DSA:4.10.530.GENE3D); IPR014716 (G3DSA:3.90.215.GENE3D); IPR020837 (PROSITE); PTHR19143 (PANTHER), SignalP-NN(euk) (SIGNALP) | |
| 2407 | lib_dib_Filter15_IDBA_contig_43949_minus3 | 366 | epididymal sperm-binding protein 1-like | 763 | 1.52E-56 | 58.20% | 1 | F:hydrolase activity | IPR000562 (G3DSA:2.10.10.GENE3D); IPR013806 (SUPERFAMILY); PR00013 (PRINTS), PTHR22918 (PANTHER), PTHR22918:SF0 (PANTHER), SignalP-NN(euk) (SIGNALP), tmhmm (TMHMM) | Yes |
| 2408 | lib_dib_Filter15_IDBA_contig_43952_minus1 | 171 | dnaj homolog subfamily c member 3-like | 292 | 2.42E-79 | 72.60% | 3 | C:endoplasmic reticulum; F:protein binding; P:protein metabolic process | IPR001623 (PRINTS); IPR011990 (G3DSA:1.25.40.GENE3D); IPR013026 (PROFILE); IPR019734 (SMART); IPR026901 (PTHR24078:PANTHER); PTHR24078 (PANTHER), SSF48452 (SUPERFAMILY) | |
| 2409 | lib_dib_Filter15_IDBA_contig_43953_minus2 | 179 | dnaj homolog subfamily c member 3-like | 235 | 2.37E-58 | 66.40% | 1 | F:protein binding | IPR011990 (G3DSA:1.25.40.GENE3D); IPR013026 (PROFILE); IPR013105 (PFAM); IPR019734 (SMART); IPR026901 (PTHR24078:PANTHER); PTHR24078 (PANTHER), PF13414 (PFAM), SignalP-NN(euk) (SIGNALP), SSF48452 (SUPERFAMILY) | |

| | | | | | | | | | |
|---|---|---|---|---|---|---|---|---|---|
| 2410 | lib_dib_Filter15_IDBA_contig_43954_minus1 | 41 | dnaj homolog subfamily c member 3-like | 126 | 1.35E-43 | 71.70% | 1 | F:protein binding | IPR011990 (G3DSA:1.25.40.GENE3D); IPR013026 (PROFILE); IPR019734 (PROFILE); IPR026901 (PTHR24078:PANTHER); PTHR24078 (PANTHER), PF13414 (PFAM), SSF48452 (SUPERFAMILY) |
| 2411 | lib_dib_Filter15_IDBA_contig_43991_plus2 | 105 | tyrosine-protein kinase fps85d-like isoform 1 | 337 | 9.67E-68 | 74.15% | 13 | P:anatomical structure morphogenesis; P:cellular component organization; P:cell differentiation; P:multicellular organismal development; P:cellular protein modification process; C:plasma membrane; P:cytoskeleton organization; P:embryo development; F:protein kinase activity; P:regulation of biological process; P:cell growth; C:cytoplasm; F:nucleotide binding | IPR000719 (PROFILE); IPR001245 (PRINTS); IPR002290 (SMART); IPR008266 (PROSITE); IPR011009 (SUPERFAMILY); IPR020635 (SMART); G3DSA:1.10.510.10 (GENE3D), PTHR24418 (PANTHER), PTHR24418:SF0 (PANTHER), tmhmm (TMHMM) |
| 2412 | lib_dib_Filter15_IDBA_contig_43998_minus3 | 28 | flavin-containing monooxygenase fmo gs-ox-like 2-like | 234 | 4.67E-76 | 64.00% | 3 | F:catalytic activity; F:nucleotide binding; P:metabolic process | IPR000960 (PRINTS); IPR020946 (PFAM); G3DSA:3.50.50.60 (GENE3D), PTHR23023 (PANTHER), SSF51905 (SUPERFAMILY) |
| 2413 | lib_dib_Filter15_IDBA_contig_44007_minus3 | 31 | probable beta-d-xylosidase 2-like | 234 | 7.66E-85 | 65.40% | 2 | F:hydrolase activity; P:carbohydrate metabolic process | IPR001764 (PRINTS); IPR017853 (SUPERFAMILY); IPR026892 (PANTHER); PTHR30620:SF0 (PANTHER) |
| 2414 | lib_dib_Filter15_IDBA_contig_44045_minus1 | 18 | signal cub and egf-like domain-containing protein 2 | 235 | 6.90E-33 | 55.25% | 3 | F:protein binding; F:carbohydrate binding; F:calcium ion binding | IPR000742 (SMART); IPR001304 (PFAM); IPR001881 (PFAM); IPR016186 (G3DSA:3.10.100.GENE3D); IPR016187 (SUPERFAMILY); G3DSA:2.10.25.10 (GENE3D), PTHR24039 (PANTHER), SSF57196 (SUPERFAMILY) |
| 2415 | lib_dib_Filter15_IDBA_contig_44091_plus1 | 610 | protein | 297 | 8.48E-30 | 45.75% | 3 | P:proteolysis; F:serine-type endopeptidase activity; C:outer membrane | no IPS match |
| 2416 | lib_dib_Filter15_IDBA_contig_44105_plus3 | 46 | thrombospondin type-1 domain-containing protein 7a | 234 | 1.35E-53 | 55.00% | 0 | - | IPR000884 (PFAM); G3DSA:2.20.100.10 (GENE3D), PTHR11311 (PANTHER), PTHR11311:SF2 (PANTHER) |
| 2417 | lib_dib_Filter15_IDBA_contig_44143_minus3 | 26 | activin receptor type-1 | 234 | 2.51E-123 | 84.50% | 27 | P:signal transduction; P:regulation of biological process; P:multicellular organismal development; P:cell differentiation; F:protein binding; P:embryo development; P:cell cycle; F:receptor activity; F:protein kinase activity; C:protein complex; C:plasma membrane; P:anatomical structure morphogenesis; F:binding; P:cellular protein modification process; P:cellular component organization; ; P:response to external stimulus; P:response to stress; F:signal transducer activity; F:nucleotide binding; P:response to endogenous stimulus; P:reproduction; P:biological_process; C:cell; P:cell death; P:metabolic process; C:cytoplasm | IPR000719 (PFAM); IPR002290 (SMART); IPR008271 (PROSITE); IPR011009 (SUPERFAMILY); IPR020635 (SMART); G3DSA:1.10.510.10 (GENE3D), PTHR23255 (PANTHER), PTHR23255:SF14 (PANTHER) |
| 2418 | lib_dib_Filter15_IDBA_contig_44215_minus2 | 33 | zinc finger protein 135-like | 259 | 9.89E-44 | 51.15% | 3 | F:binding; F:nucleic acid binding; C:intracellular | IPR007087 (PFAM); IPR013087 (G3DSA:3.30.160.GENE3D); IPR015880 (SMART); PTHR24375 (PANTHER), PTHR24375:SF0 (PANTHER), PF13465 (PFAM), PF13912 (PFAM), SSF57667 (SUPERFAMILY) |
| 2419 | lib_dib_Filter15_IDBA_contig_44244_minus2 | 2788 | nadh dehydrogenase isoform 1 | 304 | 8.51E-38 | 78.90% | 6 | P:generation of precursor metabolites and energy; P:biosynthetic process; P:lipid metabolic process; F:transporter activity; F:binding; C:mitochondrion | IPR003231 (PRODOM); IPR009081 (G3DSA:1.10.1200.GENE3D); PTHR20863 (PANTHER), PTHR20863:SF5 (PANTHER), tmhmm (TMHMM) |

| | | | | | | | | |
|---|---|---|---|---|---|---|---|---|
| 2420 | lib_dib_Filter15_IDBA_contig_44245_minus3 | 26 | l-xylulose reductase | 233 | 2.90E-49 | 66.50% | 5 | P:carbohydrate metabolic process; F:catalytic activity; P:cellular component organization; C:cell; F:nucleotide binding | IPR002198 (PRINTS); IPR002347 (PRINTS); IPR016040 (G3DSA:3.40.50.GENE3D); PTHR24311 (PANTHER), PTHR24311:SF0 (PANTHER), PF13561 (PFAM), SSF51735 (SUPERFAMILY) |
| 2421 | lib_dib_Filter15_IDBA_contig_44374_plus1 | 37 | fibroblast growth factor receptor 3 | 233 | 9.45E-41 | 65.90% | 26 | P:signal transduction; P:regulation of biological process; P:cell death; C:plasma membrane; P:cell differentiation; P:multicellular organismal development; C:lysosome; P:cell proliferation; P:cell communication; P:cellular homeostasis; P:biological_process; P:anatomical structure morphogenesis; F:protein binding; P:organelle organization; P:cell cycle; P:lipid metabolic process; P:cellular protein modification process; F:receptor activity; F:protein kinase activity; P:growth; C:cytoplasm; ; P:cellular component organization; P:response to endogenous stimulus; P:response to external stimulus; P:response to stress | IPR003598 (SMART); IPR003599 (SMART); IPR007110 (PROFILE); IPR013098 (PFAM); IPR013783 (G3DSA:2.60.40.GENE3D); PTHR24416 (PANTHER), PTHR24416:SF128 (PANTHER), PF13895 (PFAM), tmhmm (TMHMM), SSF48726 (SUPERFAMILY) |
| 2422 | lib_dib_Filter15_IDBA_contig_44399_plus2 | 23 | matrix metalloproteinase 19 isoform rasi-1 prepro | 233 | 2.66E-78 | 53.55% | 5 | F:peptidase activity; P:protein metabolic process; P:catabolic process; F:binding; C:extracellular region | IPR000585 (G3DSA:2.110.10.GENE3D); IPR001818 (PFAM); IPR006026 (SMART); IPR021190 (PRINTS); IPR024079 (G3DSA:3.40.390.GENE3D); PTHR10201 (PANTHER), SSF55486 (SUPERFAMILY) |
| 2423 | lib_dib_Filter15_IDBA_contig_44411_plus3 | 142 | monoacylglycerol lipase abhd12 | 511 | 7.08E-75 | 63.05% | 1 | F:hydrolase activity | IPR026605 (PTHR12277:PANTHER); G3DSA:3.40.50.1820 (GENE3D), PTHR12277 (PANTHER), PF12695 (PFAM), tmhmm (TMHMM), SSF53474 (SUPERFAMILY) |
| 2424 | lib_dib_Filter15_IDBA_contig_44476_plus3 | 31 | cadherin 23 | 232 | 2.15E-22 | 50.65% | 5 | P:biological_process; P:cellular component organization; P:cell differentiation; P:multicellular organismal development; C:cell | tmhmm (TMHMM) |
| 2425 | lib_dib_Filter15_IDBA_contig_44480_plus3 | 184 | odd oz protein | 639 | 0 | 63.60% | 1 | F:protein binding | IPR000742 (SMART); IPR008969 (SUPERFAMILY); IPR013032 (PFAM); IPR013111 (PFAM); G3DSA:2.10.25.10 (GENE3D), G3DSA:2.170.300.10 (GENE3D), PTHR11219 (PANTHER), SSF57196 (SUPERFAMILY) |
| 2426 | lib_dib_Filter15_IDBA_contig_44483_plus3 | 168 | ddb1- and cul4-associated factor 13 | 459 | 0 | 82.75% | 6 | C:nucleolus; P:cellular protein modification process; P:nucleobase-containing compound metabolic process; C:intracellular; C:protein complex; F:protein binding | IPR001680 (PFAM); IPR007287 (PFAM); IPR015943 (G3DSA:2.130.10.GENE3D); IPR017986 (PROFILE); IPR019775 (PROSITE); PTHR22851 (PANTHER), PTHR22851:SF0 (PANTHER) |
| 2427 | lib_dib_Filter15_IDBA_contig_44497_plus1 | 3930 | nicotinic acetylcholine receptor alpha6 subunit | 887 | 6.63E-51 | 57.70% | 3 | P:ion transport; C:cell; F:ion channel activity | IPR006029 (SUPERFAMILY); IPR006201 (PRINTS); IPR006202 (G3DSA:2.70.170.GENE3D); IPR018000 (PROSITE); G3DSA:1.20.120.370 (GENE3D), PTHR18945:SF92 (PANTHER), tmhmm (TMHMM) |
| 2428 | lib_dib_Filter15_IDBA_contig_44628_minus1 | 1072 | epididymal sperm-binding protein 1-like | 528 | 1.04E-39 | 45.95% | 1 | F:carbohydrate binding | IPR000562 (G3DSA:2.10.10.GENE3D); IPR013806 (SUPERFAMILY); PTHR22918 (PANTHER), PTHR22918:SF0 (PANTHER), SignalP-NN(euk) (SIGNALP) |
| 2429 | lib_dib_Filter15_IDBA_contig_44641_minus3 | 26 | f-box lrr-repeat protein 12-like | 231 | 3.60E-26 | 48.95% | 4 | C:cytoplasm; C:ubiquitin ligase complex; F:protein binding; P:ubiquitin-dependent protein catabolic process | IPR006553 (SMART); G3DSA:3.80.10.10 (GENE3D), PTHR23125 (PANTHER), PF13516 (PFAM), SSF52047 (SUPERFAMILY) |

| | | | | | | | | | | |
|---|---|---|---|---|---|---|---|---|---|---|
| 2430 | lib_dib_Filter15_IDBA_contig_44647_minus1 | 48 | transducin -like 3 | 232 | 4.53E-75 | 73.90% | 4 | C:nucleolus; F:signal transducer activity; P:signal transduction; F:protein binding | IPR001680 (PFAM); IPR015943 (G3DSA:2.130.10.GENE3D); IPR017986 (PROFILE); IPR019775 (PROSITE); PTHR19854 (PANTHER), PTHR19854:SF14 (PANTHER), SignalP-NN(euk) (SIGNALP) | Yes |
| 2431 | lib_dib_Filter15_IDBA_contig_44672_plus1 | 38 | protein scribble homolog | 232 | 1.77E-51 | 62.85% | 14 | F:protein binding; P:behavior; P:response to external stimulus; C:plasma membrane; P:transport; P:cell-cell signaling; P:anatomical structure morphogenesis; P:multicellular organismal development; P:response to stress; P:biological_process; P:embryo development; P:cell differentiation; P:regulation of biological process; P:cellular component organization | IPR001478 (PFAM); G3DSA:2.30.42.10 (GENE3D), PTHR23119 (PANTHER), PTHR23119:SF8 (PANTHER) | |
| 2432 | lib_dib_Filter15_IDBA_contig_44700_plus2 | 102 | ankyrin repeat protein | 365 | 1.44E-32 | 52.35% | 1 | F:protein binding | IPR002110 (PRINTS); IPR020683 (G3DSA:1.25.40.GENE3D); PTHR24198 (PANTHER), tmhmm (TMHMM) | |
| 2433 | lib_dib_Filter15_IDBA_contig_44730_plus3 | 104 | protein | 298 | 5.87E-17 | 51.65% | 1 | F:protein binding | IPR002035 (PFAM); G3DSA:3.40.50.410 (GENE3D), PTHR22992 (PANTHER), tmhmm (TMHMM), SSF53300 (SUPERFAMILY) | |
| 2434 | lib_dib_Filter15_IDBA_contig_44731_plus3 | 102 | protein | 298 | 4.60E-17 | 51.60% | 1 | F:protein binding | IPR002035 (PFAM); G3DSA:3.40.50.410 (GENE3D), PTHR22992 (PANTHER), tmhmm (TMHMM), SSF53300 (SUPERFAMILY) | |
| 2435 | lib_dib_Filter15_IDBA_contig_44733_plus1 | 66 | collagen alpha-3 chain | 164 | 9.24E-23 | 56.00% | 1 | F:protein binding | IPR002035 (PFAM); G3DSA:3.40.50.410 (GENE3D), PTHR22992 (PANTHER), SSF53300 (SUPERFAMILY) | |
| 2436 | lib_dib_Filter15_IDBA_contig_44758_plus3 | 20 | ectonucleotide pyrophosphatase phosphodiesterase family member 5 isoform 1 | 231 | 1.21E-65 | 62.95% | 3 | C:cell; F:catalytic activity; P:metabolic process | IPR002591 (PFAM); IPR017849 (G3DSA:3.40.720.GENE3D); IPR017850 (SUPERFAMILY); IPR024873 (PANTHER) | |
| 2437 | lib_dib_Filter15_IDBA_contig_44804_minus3 | 116 | protein dpy-11 | 270 | 8.71E-74 | 72.05% | 11 | C:cytoplasm; P:anatomical structure morphogenesis; P:regulation of biological process; P:growth; P:behavior; P:reproduction; P:cellular protein modification process; P:biological_process; F:catalytic activity; C:cell; P:cellular homeostasis | IPR012336 (G3DSA:3.40.30.GENE3D); IPR013766 (PFAM); IPR017937 (PROSITE); PTHR18929 (PANTHER), SignalP-NN(euk) (SIGNALP), tmhmm (TMHMM) | yes |
| 2438 | lib_dib_Filter15_IDBA_contig_44805_plus2 | 97 | kelch-like protein 9 | 389 | 5.14E-71 | 49.50% | 2 | P:biological_process; F:protein binding | IPR006652 (PFAM); IPR011705 (PFAM); IPR015915 (G3DSA:2.120.10.GENE3D); PTHR24412 (PANTHER), SSF117281 (SUPERFAMILY) | |
| 2439 | lib_dib_Filter15_IDBA_contig_44912_plus3 | 542 | carboxypeptidase | 674 | 2.17E-101 | 61.10% | 4 | F:peptidase activity; P:protein metabolic process; P:catabolic process; F:binding | IPR000834 (PRINTS); IPR003146 (G3DSA:3.30.70.GENE3D); IPR006970 (PFAM); IPR009020 (SUPERFAMILY); G3DSA:3.40.630.10 (GENE3D), PTHR11705 (PANTHER), SSF53187 (SUPERFAMILY) | |
| 2440 | lib_dib_Filter15_IDBA_contig_44918_minus2 | 15 | truncated furin endoprotease | 230 | 6.06E-125 | 88.10% | 18 | C:cell; C:cytoplasmic membrane-bounded vesicle; C:Golgi apparatus; P:regulation of biological process; P:cell death; P:protein metabolic process; P:catabolic process; F:peptidase activity; P:cell proliferation; F:enzyme regulator activity; P:reproduction; P:viral reproduction; F:protein binding; P:signal transduction; F:binding; P:biological_process; P:biosynthetic process; P:transport | IPR000209 (G3DSA:3.40.50.GENE3D); IPR015500 (PRINTS); IPR022398 (PROSITE); IPR023827 (PROSITE); IPR023828 (PROSITE); PTHR10795:SF16 (PANTHER) | |

| | | | | | | | | | | |
|---|---|---|---|---|---|---|---|---|---|---|
| 2441 | lib_dib_Filter15_IDBA_contig_44933_plus1 | 53 | lactase-phlorizin hydrolase | 231 | 7.99E-31 | 67.20% | 9 | P:response to stress; F:binding; P:biological_process; P:response to external stimulus; P:response to endogenous stimulus; C:cell; F:hydrolase activity; F:transferase activity; P:carbohydrate metabolic process | IPR001360 (PRINTS); IPR013781 (G3DSA:3.20.20.GENE3D); IPR017853 (SUPERFAMILY) | |
| 2442 | lib_dib_Filter15_IDBA_contig_44955_plus1 | 20 | inactive dipeptidyl peptidase 10-like | 231 | 6.60E-76 | 55.00% | 4 | F:peptidase activity; P:protein metabolic process; P:catabolic process; C:cell | IPR002469 (PFAM); G3DSA:2.140.10.30 (GENE3D), PTHR11731 (PANTHER), SSF82171 (SUPERFAMILY) | |
| 2443 | lib_dib_Filter15_IDBA_contig_44969_plus1 | 24 | wd repeat-containing protein 33 | 159 | 1.73E-93 | 88.25% | 4 | C:nucleolus; P:nucleobase-containing compound metabolic process; C:proteinaceous extracellular matrix; F:protein binding | IPR001680 (PFAM); IPR015943 (G3DSA:2.130.10.GENE3D); IPR017986 (PROFILE); PTHR22836 (PANTHER) | |
| 2444 | lib_dib_Filter15_IDBA_contig_44994_plus2 | 359 | leucine-rich repeat-containing protein 59 | 354 | 3.72E-50 | 78.70% | 4 | C:endoplasmic reticulum; C:cell; C:mitochondrion; F:protein binding | IPR001611 (PFAM); IPR003591 (SMART); G3DSA:3.80.10.10 (GENE3D), PTHR23155 (PANTHER), PTHR23155:SF22 (PANTHER), PF13855 (PFAM), SignalP-NN(euk) (SIGNALP), tmhmm (TMHMM), SSF52058 (SUPERFAMILY) | |
| 2445 | lib_dib_Filter15_IDBA_contig_45_plus3 | 35916 | hypothetical protein | 969 | 1.15E-25 | 64.45% | 0 | - | tmhmm (TMHMM) | |
| 2446 | lib_dib_Filter15_IDBA_contig_45055_minus3 | 51 | zinc finger protein | 279 | 3.95E-48 | 65.20% | 3 | F:binding; F:nucleic acid binding; C:intracellular | IPR007087 (PROSITE); IPR013087 (G3DSA:3.30.160.GENE3D); IPR015880 (SMART); PTHR11389 (PANTHER), PF13465 (PFAM), SSF57667 (SUPERFAMILY) | |
| 2447 | lib_dib_Filter15_IDBA_contig_45056_plus1 | 498 | protein | 806 | 1.33E-15 | 55.00% | 2 | F:protein binding; F:calcium ion binding | IPR000152 (PROSITE); IPR000742 (PFAM); IPR001881 (SMART); IPR002035 (PFAM); IPR013032 (PROSITE); IPR018097 (PROSITE); G3DSA:2.10.25.10 (GENE3D), G3DSA:3.40.50.410 (GENE3D), PTHR24048 (PANTHER), PTHR24048:SF15 (PANTHER), tmhmm (TMHMM), SSF53300 (SUPERFAMILY), SSF57196 (SUPERFAMILY) | |
| 2448 | lib_dib_Filter15_IDBA_contig_45089_plus1 | 106 | d-beta-hydroxybutyrate mitochondrial | 415 | 5.57E-78 | 59.75% | 3 | P:metabolic process; F:catalytic activity; F:nucleotide binding | IPR002198 (PFAM); IPR002347 (PRINTS); IPR016040 (G3DSA:3.40.50.GENE3D); IPR020904 (PROSITE); PTHR24316 (PANTHER), PTHR24316:SF34 (PANTHER), SignalP-NN(euk) (SIGNALP), tmhmm (TMHMM), SSF51735 (SUPERFAMILY) | |
| 2449 | lib_dib_Filter15_IDBA_contig_45139_plus3 | 112 | zinc c2h2 type family protein | 181 | 4.87E-16 | 59.70% | 3 | F:binding; F:nucleic acid binding; C:intracellular | IPR007087 (PROSITE); IPR013087 (G3DSA:3.30.160.GENE3D); IPR015880 (SMART); PTHR24382 (PANTHER), PTHR24382:SF0 (PANTHER), PF13465 (PFAM), SSF57667 (SUPERFAMILY) | |
| 2450 | lib_dib_Filter15_IDBA_contig_45140_plus3 | 62 | oocyte zinc finger protein 6-like | 145 | 2.35E-28 | 65.25% | 3 | F:binding; F:nucleic acid binding; C:intracellular | IPR007087 (PROSITE); IPR013087 (G3DSA:3.30.160.GENE3D); IPR015880 (SMART); PTHR24382 (PANTHER), PTHR24382:SF0 (PANTHER), PF13465 (PFAM), SSF57667 (SUPERFAMILY) | |
| 2451 | lib_dib_Filter15_IDBA_contig_45209_plus2 | 73 | di-n-acetylchitobiase precursor | 255 | 3.23E-97 | 66.25% | 3 | F:hydrolase activity; P:carbohydrate metabolic process; P:catabolic process | IPR001223 (PFAM); IPR011583 (SMART); IPR013781 (G3DSA:3.20.20.GENE3D); IPR017853 (SUPERFAMILY); PTHR11177 (PANTHER), PTHR11177:SF8 (PANTHER) | |
| 2452 | lib_dib_Filter15_IDBA_contig_45210_plus2 | 103 | di-n-acetylchitobiase precursor | 323 | 1.07E-117 | 65.80% | 3 | F:hydrolase activity; P:carbohydrate metabolic process; P:catabolic process | IPR001223 (PFAM); IPR011583 (SMART); IPR013781 (G3DSA:3.20.20.GENE3D); IPR017853 (SUPERFAMILY); PTHR11177 (PANTHER), PTHR11177:SF8 (PANTHER), SignalP-NN(euk) (SIGNALP), tmhmm (TMHMM) | yes |

| | | | | | | | | | |
|---|---|---|---|---|---|---|---|---|---|
| 2453 | lib_dib_Filter15_IDBA_contig_45295_plus1 | 10714 | a disintegrin and metalloproteinase with thrombospondin motifs 17 | 293 | 3.37E-22 | 47.80% | 3 | F:peptidase activity; P:protein metabolic process; P:catabolic process | IPR001590 (PROFILE); IPR024079 (G3DSA:3.40.390.GENE3D); PTHR13723 (PANTHER), PF13574 (PFAM), SSF55486 (SUPERFAMILY) |
| 2454 | lib_dib_Filter15_IDBA_contig_45305_minus2 | 78 | serine threonine-protein kinase sik2 | 229 | 2.53E-101 | 88.15% | 9 | P:cellular protein modification process; P:signal transduction; F:protein kinase activity; F:nucleotide binding; F:protein binding; F:binding; P:response to endogenous stimulus; C:Golgi apparatus; C:nucleus | IPR000719 (PFAM); IPR002290 (SMART); IPR008271 (PROSITE); IPR011009 (SUPERFAMILY); IPR017441 (PROSITE); IPR020635 (SMART); G3DSA:1.10.510.10 (GENE3D), G3DSA:3.30.200.20 (GENE3D), PTHR24343 (PANTHER), PTHR24343:SF80 (PANTHER) |
| 2455 | lib_dib_Filter15_IDBA_contig_45307_minus1 | 25 | zinc finger protein 271-like | 229 | 1.63E-54 | 70.05% | 3 | F:binding; F:nucleic acid binding; C:intracellular | IPR007087 (PROSITE); IPR013087 (G3DSA:3.30.160.GENE3D); IPR015880 (SMART); PTHR24387 (PANTHER), PTHR24387:SF0 (PANTHER), PF13465 (PFAM), SignalP-NN(euk) (SIGNALP), SSF57667 (SUPERFAMILY) |
| 2456 | lib_dib_Filter15_IDBA_contig_45327_minus3 | 347 | beta- -galactosyl-o-glycosyl-glycoprotein beta- -n-acetylglucosaminyltransferase | 698 | 7.68E-97 | 57.55% | 2 | F:transferase activity; C:cell | IPR003406 (PFAM); PTHR19297 (PANTHER), PTHR19297:SF6 (PANTHER), tmhmm (TMHMM) |
| 2457 | lib_dib_Filter15_IDBA_contig_45328_minus3 | 361 | beta- -galactosyl-o-glycosyl-glycoprotein beta- -n-acetylglucosaminyltransferase | 750 | 2.00E-96 | 57.55% | 2 | F:transferase activity; C:cell | IPR003406 (PFAM); PTHR19297 (PANTHER), PTHR19297:SF6 (PANTHER), tmhmm (TMHMM) |
| 2458 | lib_dib_Filter15_IDBA_contig_45329_minus3 | 367 | beta- -galactosyl-o-glycosyl-glycoprotein beta- -n-acetylglucosaminyltransferase | 752 | 1.69E-96 | 57.55% | 2 | F:transferase activity; C:cell | IPR003406 (PFAM); PTHR19297 (PANTHER), PTHR19297:SF6 (PANTHER), tmhmm (TMHMM) |
| 2459 | lib_dib_Filter15_IDBA_contig_45352_minus1 | 96 | toll protein | 229 | 1.19E-07 | 53.15% | 8 | F:transmembrane signaling receptor activity; C:integral to membrane; C:membrane; P:signal transduction; P:innate immune response; C:intrinsic to membrane; C:intracellular; F:receptor activity | IPR003591 (SMART); G3DSA:3.80.10.10 (GENE3D), PTHR24365 (PANTHER), PF13855 (PFAM), SignalP-NN(euk) (SIGNALP), SSF52058 (SUPERFAMILY) |
| 2460 | lib_dib_Filter15_IDBA_contig_45353_minus1 | 55 | insulin-like growth factor-binding protein complex acid labile subunit-like | 205 | 9.79E-18 | 50.75% | 6 | P:transport; P:cell differentiation; P:multicellular organismal development; P:biological_process; P:anatomical structure morphogenesis; P:cellular component organization | IPR000483 (SMART); IPR003591 (SMART); G3DSA:3.80.10.10 (GENE3D), PTHR24373 (PANTHER), PF13855 (PFAM), tmhmm (TMHMM), SSF52058 (SUPERFAMILY) |
| 2461 | lib_dib_Filter15_IDBA_contig_45453_plus3 | 26 | zinc finger and scan domain-containing protein 10 | 228 | 3.06E-17 | 58.75% | 3 | C:intracellular; F:binding; F:nucleic acid binding | IPR007087 (PROSITE); IPR013087 (G3DSA:3.30.160.GENE3D); IPR015880 (SMART); PTHR23229 (PANTHER), PTHR23229:SF13 (PANTHER), PF13465 (PFAM), SSF57667 (SUPERFAMILY) |
| 2462 | lib_dib_Filter15_IDBA_contig_45461_minus1 | 35 | cre-sul-2 protein | 228 | 3.34E-56 | 62.55% | 2 | P:metabolic process; F:hydrolase activity | IPR000917 (PFAM); IPR017849 (G3DSA:3.40.720.GENE3D); IPR017850 (SUPERFAMILY); PTHR10342 (PANTHER) |
| 2463 | lib_dib_Filter15_IDBA_contig_45495_plus1 | 30 | von willebrand factor type egf and pentraxin domain-containing protein 1 | 228 | 7.71E-36 | 50.95% | 6 | F:calcium ion binding; C:cytoplasm; P:cell adhesion; C:membrane; C:extracellular region; P:cell redox homeostasis | IPR000436 (PFAM); G3DSA:2.10.70.10 (GENE3D), PTHR19325 (PANTHER) |
| 2464 | lib_dib_Filter15_IDBA_contig_45552_plus1 | 715 | ankyrin repeat protein | 644 | 1.79E-10 | 46.70% | 1 | F:protein binding | IPR000742 (PFAM); IPR002110 (SMART); IPR013032 (PROSITE); IPR020683 (G3DSA:1.25.40.GENE3D); G3DSA:2.10.25.10 (GENE3D), PTHR24193 (PANTHER), tmhmm (TMHMM), SSF57196 (SUPERFAMILY) |
| 2465 | lib_dib_Filter15_IDBA_contig_45563_minus2 | 117 | coiled-coil domain-containing protein 18 | 532 | 5.08E-83 | 57.80% | 2 | F:zinc ion binding; F:metalloendopeptidase activity | IPR009053 (SUPERFAMILY); PTHR18875 (PANTHER), PTHR18875:SF3 (PANTHER) |

| | | | | | | | | | |
|---|---|---|---|---|---|---|---|---|---|
| 2466 | lib_dib_Filter15_IDBA_contig_45604_plus3 | 711 | ferric-chelate reductase 1-like | 486 | 2.33E-12 | 45.75% | 11 | F:molecular_function; P:defense response to protozoan; C:extracellular region; P:innate immune response; P:defense response to bacterium; P:oxidation-reduction process; F:oxidoreductase activity; C:integral to membrane; C:membrane; P:electron transport chain; P:transport | IPR002861 (PFAM); PTHR23130 (PANTHER), PTHR23130:SF6 (PANTHER), tmhmm (TMHMM) | |
| 2467 | lib_dib_Filter15_IDBA_contig_45639_plus2 | 64 | bola-like protein 2-like | 227 | 3.95E-31 | 77.25% | 0 | - | IPR002634 (G3DSA:3.30.300.GENE3D); PTHR12735 (PANTHER) | |
| 2468 | lib_dib_Filter15_IDBA_contig_4564_plus3 | 1831 | phospholipase b-like 2 isoform 1 | 743 | 0 | 66.15% | 4 | C:lysosome; F:hydrolase activity; P:lipid catabolic process; C:lysosomal lumen | IPR007000 (PANTHER); SignalP-NN(euk) (SIGNALP), tmhmm (TMHMM) | yes |
| 2469 | lib_dib_Filter15_IDBA_contig_45652_minus2 | 28 | deleted in malignant brain tumors 1 | 227 | 2.30E-21 | 52.45% | 2 | F:receptor activity; C:cell | IPR001190 (PRINTS); IPR017448 (SMART); G3DSA:3.10.250.10 (GENE3D), PTHR19331 (PANTHER), PTHR19331:SF118 (PANTHER), SignalP-NN(euk) (SIGNALP), tmhmm (TMHMM) | |
| 2470 | lib_dib_Filter15_IDBA_contig_45669_plus3 | 60 | endothelin-converting enzyme 2 isoform 3 | 227 | 1.14E-22 | 48.55% | 3 | F:peptidase activity; P:protein metabolic process; P:catabolic process | IPR000718 (PANTHER); IPR008753 (PFAM); G3DSA:1.10.1380.10 (GENE3D), SSF55486 (SUPERFAMILY) | |
| 2471 | lib_dib_Filter15_IDBA_contig_45704_minus3 | 102 | hypothetical protein BRAFLDRAFT_219292 | 376 | 8.74E-26 | 46.14% | 1 | C:membrane | SignalP-NN(euk) (SIGNALP), tmhmm (TMHMM) | |
| 2472 | lib_dib_Filter15_IDBA_contig_45704_plus3 | 102 | protein | 376 | 3.77E-15 | 56.25% | 0 | - | SignalP-NN(euk) (SIGNALP), tmhmm (TMHMM) | |
| 2473 | lib_dib_Filter15_IDBA_contig_45806_plus1 | 39 | low quality protein: protocadherin-16 | 247 | 5.33E-69 | 58.75% | 3 | F:calcium ion binding; P:biological_process; C:plasma membrane | IPR002126 (PRINTS); IPR015919 (SUPERFAMILY); IPR020894 (PROSITE); PTHR24027 (PANTHER) | |
| 2474 | lib_dib_Filter15_IDBA_contig_45819_minus3 | 24 | fibroblast growth factor receptor | 226 | 1.50E-67 | 70.55% | 6 | C:cell; P:signal transduction; F:receptor activity; F:protein kinase activity; F:nucleotide binding; P:cellular protein modification process | IPR000719 (PROFILE); IPR001245 (PFAM); IPR011009 (SUPERFAMILY); IPR020635 (SMART); G3DSA:3.30.200.20 (GENE3D), PTHR24416 (PANTHER), PTHR24416:SF127 (PANTHER), SignalP-NN(euk) (SIGNALP) | |
| 2475 | lib_dib_Filter15_IDBA_contig_45941_plus2 | 20 | n-acetylated-alpha-linked acidic dipeptidase-like protein | 226 | 2.60E-51 | 59.55% | 4 | C:cell; F:peptidase activity; P:protein metabolic process; P:catabolic process | IPR007484 (PFAM); G3DSA:3.40.630.10 (GENE3D), PTHR10404 (PANTHER), SSF53187 (SUPERFAMILY) | |
| 2476 | lib_dib_Filter15_IDBA_contig_45996_minus3 | 49 | extracellular serine proteinase-like | 224 | 1.19E-09 | 57.70% | 2 | F:binding; F:peptidase activity | IPR000859 (G3DSA:2.60.120.GENE3D); PTHR10127 (PANTHER) | |
| 2477 | lib_dib_Filter15_IDBA_contig_45997_minus3 | 52 | extracellular serine proteinase-like | 229 | 2.82E-11 | 57.75% | 2 | F:binding; F:peptidase activity | IPR000859 (G3DSA:2.60.120.GENE3D); PTHR10127 (PANTHER) | |
| 2478 | lib_dib_Filter15_IDBA_contig_45998_minus3 | 28 | extracellular serine proteinase-like | 177 | 5.69E-10 | 61.30% | 2 | F:binding; F:peptidase activity | IPR000859 (G3DSA:2.60.120.GENE3D); PTHR10127 (PANTHER) | |
| 2479 | lib_dib_Filter15_IDBA_contig_45999_minus1 | 39 | bacillolysin (neutral protease) | 226 | 2.78E-13 | 60.95% | 2 | F:binding; F:peptidase activity | IPR000859 (G3DSA:2.60.120.GENE3D); PTHR10127 (PANTHER), SignalP-NN(euk) (SIGNALP) | |
| 2480 | lib_dib_Filter15_IDBA_contig_46031_minus1 | 341 | rhamnose-binding lectin-like | 417 | 3.45E-36 | 49.60% | 3 | C:extracellular region; P:carbohydrate metabolic process; F:carbohydrate binding | IPR000922 (PFAM); IPR002557 (PROFILE); IPR003582 (PFAM); PTHR12011 (PANTHER), PTHR12011:SF4 (PANTHER) | |
| 2481 | lib_dib_Filter15_IDBA_contig_46040_minus3 | 26 | epidermal growth factor receptor | 225 | 9.95E-46 | 51.20% | 12 | P:regulation of biological process; P:cell communication; P:transport; F:protein binding; P:cell death; P:multicellular organismal development; P:cellular protein modification process; P:signal transduction; C:plasma membrane; F:receptor activity; F:protein kinase activity; F:nucleotide binding | IPR006211 (PFAM); IPR006212 (SMART); IPR009030 (SUPERFAMILY); G3DSA:2.10.220.10 (GENE3D), G3DSA:3.80.20.20 (GENE3D), PTHR24416 (PANTHER), PTHR24416:SF137 (PANTHER), SSF52058 (SUPERFAMILY) | |
| 2482 | lib_dib_Filter15_IDBA_contig_46048_plus1 | 5544 | nuclease-sensitive element-binding protein 1-like protein | 153 | 6.14E-27 | 86.50% | 5 | F:DNA binding; F:RNA binding; C:nucleus; ; P:regulation of biological process | IPR002059 (PRINTS); IPR012340 (G3DSA:2.40.50.GENE3D); IPR019844 (PROSITE); PTHR11544 (PANTHER), PTHR11544:SF8 (PANTHER) | |

| | | | | | | | | | | |
|---|---|---|---|---|---|---|---|---|---|---|
| 2483 | lib_dib_Filter15_IDBA_contig_46080_plus3 | 28 | lysophosphatidic acid phosphatase type 6 | 225 | 3.15E-30 | 59.15% | 2 | C:mitochondrion; F:hydrolase activity | IPR000560 (PFAM); G3DSA:3.40.50.1240 (GENE3D), PTHR11567 (PANTHER), PTHR11567:SF16 (PANTHER), tmhmm (TMHMM), SSF53254 (SUPERFAMILY) | |
| 2484 | lib_dib_Filter15_IDBA_contig_46156_plus1 | 23 | neoverrucotoxin subunit beta-like | 225 | 5.53E-31 | 68.75% | 1 | F:molecular_function | no IPS match | |
| 2485 | lib_dib_Filter15_IDBA_contig_46183_minus1 | 48 | bone morphogenetic protein 5 | 225 | 1.17E-55 | 80.10% | 28 | P:biological_process; F:receptor binding; P:anatomical structure morphogenesis; P:cellular component organization; P:cell differentiation; P:multicellular organismal development; P:response to endogenous stimulus; P:regulation of biological process; P:organelle organization; P:cell cycle; P:embryo development; P:reproduction; F:protein binding; P:metabolic process; P:signal transduction; C:cytoplasm; P:cell death; P:response to stress; P:biosynthetic process; P:lipid metabolic process; P:cellular protein modification process; P:response to external stimulus; ; P:cell proliferation; P:protein transport; C:extracellular space; P:growth; C:organelle | IPR001839 (PFAM); IPR002405 (PRINTS); IPR015615 (PANTHER); IPR017948 (PROSITE); G3DSA:2.10.90.10 (GENE3D), PTHR11848:SF54 (PANTHER), tmhmm (TMHMM), SSF57501 (SUPERFAMILY) | |
| 2486 | lib_dib_Filter15_IDBA_contig_46186_plus2 | 28 | kelch domain-containing protein 8a | 225 | 3.99E-86 | 64.90% | 1 | F:protein binding | IPR006652 (PFAM); IPR015915 (G3DSA:2.120.10.GENE3D); PTHR24412 (PANTHER), PTHR24412:SF15 (PANTHER), PF13418 (PFAM), SSF117281 (SUPERFAMILY) | |
| 2487 | lib_dib_Filter15_IDBA_contig_46241_minus3 | 289 | furin-like protease 2-like | 576 | 7.15E-62 | 45.45% | 1 | F:peptidase activity | IPR006212 (SMART); IPR009030 (SUPERFAMILY); G3DSA:2.10.220.10 (GENE3D), PTHR23275 (PANTHER), PTHR23275:SF7 (PANTHER), tmhmm (TMHMM) | |
| 2488 | lib_dib_Filter15_IDBA_contig_46242_minus1 | 56 | proprotein convertase subtilisin kexin type 6-like | 196 | 1.27E-29 | 45.60% | 7 | F:binding; P:biological_process; P:regulation of biological process; P:protein metabolic process; C:cell; F:peptidase activity; P:catabolic process | IPR002884 (PFAM); IPR006212 (SMART); IPR008979 (SUPERFAMILY); IPR009030 (SUPERFAMILY); IPR015500 (PANTHER); G3DSA:2.10.220.10 (GENE3D), G3DSA:2.60.120.260 (GENE3D), PTHR10795:SF153 (PANTHER) | |
| 2489 | lib_dib_Filter15_IDBA_contig_46244_minus2 | 45 | f-type lectin | 225 | 1.28E-16 | 49.70% | 2 | F:binding; P:biological_process | IPR000421 (PFAM); IPR006585 (SMART); IPR008979 (SUPERFAMILY); G3DSA:2.60.120.260 (GENE3D), PTHR19277 (PANTHER), PTHR19277:SF6 (PANTHER), SignalP-NN(euk) (SIGNALP), tmhmm (TMHMM) | yes |
| 2490 | lib_dib_Filter15_IDBA_contig_46245_plus2 | 30 | zinc finger protein 26 | 225 | 4.69E-39 | 55.80% | 3 | F:binding; F:nucleic acid binding; C:intracellular | IPR007087 (PROSITE); IPR013087 (G3DSA:3.30.160.GENE3D); IPR015880 (SMART); PTHR11389 (PANTHER), PF13465 (PFAM), PF13894 (PFAM), SSF57667 (SUPERFAMILY) | |
| 2491 | lib_dib_Filter15_IDBA_contig_46272_minus2 | 30 | deleted in malignant brain tumors 1 | 224 | 4.21E-30 | 52.90% | 2 | F:receptor activity; C:cell | IPR001190 (PRINTS); IPR017448 (SMART); G3DSA:3.10.250.10 (GENE3D), PTHR19331 (PANTHER), PTHR19331:SF118 (PANTHER) | |
| 2492 | lib_dib_Filter15_IDBA_contig_46295_minus2 | 23 | kelch-like protein 26 | 224 | 1.30E-63 | 66.90% | 3 | C:cell; P:biological_process; F:protein binding | IPR000210 (SMART); IPR011333 (G3DSA:3.30.710.GENE3D); IPR011705 (PFAM); IPR013069 (PFAM); PTHR24412 (PANTHER) | |
| 2493 | lib_dib_Filter15_IDBA_contig_46408_plus3 | 702 | nucleolar protein 56 | 732 | 0 | 87.15% | 1 | P:multicellular organismal development | IPR002687 (PFAM); IPR012974 (PFAM); IPR012976 (PFAM); PTHR10894 (PANTHER), SSF89124 (SUPERFAMILY) | |

| | | | | | | | | | |
|---|---|---|---|---|---|---|---|---|---|
| 2494 | lib_dib_Filter15_IDBA_contig_46410_plus1 | 22 | deleted in malignant brain tumors 1 | 224 | 6.87E-40 | 52.40% | 3 | F:catalytic activity; F:receptor activity; C:cell | IPR001190 (PRINTS); IPR017448 (SMART); G3DSA:3.10.250.10 (GENE3D), PTHR19331 (PANTHER), PTHR19331:SF118 (PANTHER) | |
| 2495 | lib_dib_Filter15_IDBA_contig_46419_minus3 | 510 | pancreatic lipase-related protein 2 | 403 | 1.18E-61 | 51.45% | 2 | F:catalytic activity; P:lipid metabolic process | IPR000734 (PRINTS); IPR013818 (PFAM); G3DSA:3.40.50.1820 (GENE3D), SignalP-NN(euk) (SIGNALP), SSF53474 (SUPERFAMILY) | yes |
| 2496 | lib_dib_Filter15_IDBA_contig_46420_plus3 | 532 | deleted in malignant brain tumors 1 | 223 | 2.99E-29 | 65.45% | 3 | F:peptidase activity; F:receptor activity; C:cell | IPR001190 (PRINTS); IPR017448 (SMART); G3DSA:3.10.250.10 (GENE3D), PTHR19331 (PANTHER), SignalP-NN(euk) (SIGNALP), tmhmm (TMHMM) | |
| 2497 | lib_dib_Filter15_IDBA_contig_46426_plus1 | 5637 | pzp protein | 604 | 4.54E-172 | 60.60% | 2 | F:enzyme regulator activity; C:extracellular space | IPR001599 (PFAM); IPR008930 (SUPERFAMILY); IPR011626 (PFAM); IPR019565 (PFAM); IPR019742 (PROSITE); G3DSA:1.50.10.20 (GENE3D), PTHR11412 (PANTHER), PTHR11412:SF36 (PANTHER) | |
| 2498 | lib_dib_Filter15_IDBA_contig_46427_plus1 | 3978 | mgc82112 protein | 353 | 7.92E-111 | 63.30% | 1 | C:extracellular space | IPR008930 (SUPERFAMILY); IPR011626 (PFAM); IPR019565 (PFAM); IPR019742 (PROSITE); G3DSA:1.50.10.20 (GENE3D), PTHR11412 (PANTHER), PTHR11412:SF36 (PANTHER) | |
| 2499 | lib_dib_Filter15_IDBA_contig_46428_plus2 | 5160 | pzp protein | 704 | 9.89E-162 | 61.40% | 2 | F:enzyme regulator activity; C:extracellular space | IPR001599 (PFAM); IPR008930 (SUPERFAMILY); IPR011626 (PFAM); IPR019565 (PFAM); IPR019742 (PROSITE); G3DSA:1.50.10.20 (GENE3D), PTHR11412 (PANTHER), PTHR11412:SF36 (PANTHER), tmhmm (TMHMM) | |
| 2500 | lib_dib_Filter15_IDBA_contig_46430_plus3 | 180 | pro-neuregulin- membrane-bound isoform-like | 536 | 3.34E-11 | 45.40% | 1 | F:protein binding | IPR000742 (SMART); IPR003598 (SMART); IPR003599 (SMART); IPR007110 (PROFILE); IPR013032 (PROSITE); IPR013098 (PFAM); IPR013783 (G3DSA:2.60.40.GENE3D); G3DSA:2.10.25.10 (GENE3D), PTHR11100 (PANTHER), SignalP-NN(euk) (SIGNALP), tmhmm (TMHMM), SSF48726 (SUPERFAMILY), SSF57196 (SUPERFAMILY) | yes |
| 2501 | lib_dib_Filter15_IDBA_contig_46458_plus1 | 26 | protein | 251 | 8.97E-37 | 64.80% | 3 | C:extracellular region; F:protein binding; F:calcium ion binding | IPR000152 (PROSITE); IPR000742 (PFAM); IPR000859 (G3DSA:2.60.120.GENE3D); IPR001881 (SMART); IPR013032 (PROSITE); IPR018097 (PROSITE); PR00010 (PRINTS), G3DSA:2.10.25.10 (GENE3D), PTHR24044 (PANTHER), SSF57196 (SUPERFAMILY) | |
| 2502 | lib_dib_Filter15_IDBA_contig_46554_minus1 | 30 | protocadherin fat 3 | 223 | 1.58E-34 | 56.55% | 3 | F:calcium ion binding; P:biological_process; C:plasma membrane | IPR002126 (PRINTS); IPR015919 (SUPERFAMILY); IPR020894 (PROSITE); PTHR24027 (PANTHER) | |
| 2503 | lib_dib_Filter15_IDBA_contig_46700_minus2 | 36 | immunoglobulin superfamily member 10 | 222 | 7.19E-96 | 52.65% | 2 | P:biological_process; F:protein binding | IPR000372 (PFAM); IPR001611 (PFAM); IPR003591 (SMART); G3DSA:3.80.10.10 (GENE3D), PTHR24367 (PANTHER), PF13855 (PFAM), SSF52058 (SUPERFAMILY) | |
| 2504 | lib_dib_Filter15_IDBA_contig_46737_plus1 | 22 | sushi domain-containing protein 2-like | 223 | 5.38E-58 | 57.65% | 4 | F:polysaccharide binding; F:scavenger receptor activity; P:immune response; C:integral to membrane | IPR001846 (PFAM); IPR005533 (PFAM); PTHR13802 (PANTHER), PTHR13802:SF1 (PANTHER) | |
| 2505 | lib_dib_Filter15_IDBA_contig_46797_plus2 | 4631 | cysteine-rich secretory protein mr30 | 321 | 6.12E-59 | 52.70% | 6 | P:proteolysis; F:hydrolase activity; F:peptidase activity; C:extracellular region; F:ion channel inhibitor activity; F:calcium channel inhibitor activity | IPR001283 (PANTHER); IPR014044 (G3DSA:3.40.33.GENE3D); SignalP-NN(euk) (SIGNALP), tmhmm (TMHMM) | yes |
| 2506 | lib_dib_Filter15_IDBA_contig_46798_plus1 | 3072 | cysteine-rich secretory protein mr30 | 325 | 1.35E-57 | 53.85% | 6 | P:proteolysis; F:hydrolase activity; F:peptidase activity; C:extracellular region; F:ion channel inhibitor activity; F:calcium channel inhibitor activity | IPR001283 (PANTHER); IPR014044 (G3DSA:3.40.33.GENE3D); SignalP-NN(euk) (SIGNALP), tmhmm (TMHMM) | yes |

| | | | | | | | | | | |
|---|---|---|---|---|---|---|---|---|---|---|
| 2507 | lib_dib_Filter15_IDBA_contig_46799_plus1 | 2742 | cysteine-rich secretory protein mr30 | 244 | 1.58E-42 | 52.80% | 6 | P:proteolysis; F:hydrolase activity; F:peptidase activity; C:extracellular region; F:ion channel inhibitor activity; F:calcium channel inhibitor activity | IPR001283 (PANTHER); IPR014044 (G3DSA:3.40.33.GENE3D); PTHR10334:SF13 (PANTHER), tmhmm (TMHMM) | |
| 2508 | lib_dib_Filter15_IDBA_contig_46822_minus1 | 225 | neurogenic locus notch homolog protein 2-like | 576 | 2.46E-127 | 55.45% | 3 | C:extracellular region; F:protein binding; F:calcium ion binding | IPR000152 (PROSITE); IPR000742 (PFAM); IPR001881 (PFAM); IPR013032 (PROSITE); IPR018097 (PROSITE); PD936484 (PRODOM), PR00010 (PRINTS), G3DSA:2.10.25.10 (GENE3D), PTHR24044 (PANTHER), SSF57196 (SUPERFAMILY) | |
| 2509 | lib_dib_Filter15_IDBA_contig_46830_minus2 | 80 | beta 1 | 354 | 1.73E-137 | 73.40% | 8 | P:carbohydrate metabolic process; P:catabolic process; C:cell; P:lipid metabolic process; F:protein binding; C:lysosome; F:carbohydrate binding; F:hydrolase activity | IPR001944 (PRINTS); IPR013781 (G3DSA:3.20.20.GENE3D); IPR017853 (SUPERFAMILY); IPR019801 (PROSITE); PTHR23421:SF26 (PANTHER), SignalP-NN(euk) (SIGNALP) | yes |
| 2510 | lib_dib_Filter15_IDBA_contig_46832_minus2 | 31 | zinc finger protein 271 (zinc finger protein 7) (zinc finger protein znfphex133) (epstein-barr virus-induced zinc finger protein) (znf-eb) (ct-zfp48) (zinc finger protein | 222 | 1.42E-16 | 45.55% | 2 | F:binding; C:intracellular | IPR007087 (PFAM); IPR015880 (SMART); G3DSA:3.90.10.10 (GENE3D), PTHR24375 (PANTHER), PTHR24375:SF0 (PANTHER), PF13465 (PFAM), PF13912 (PFAM), SSF57667 (SUPERFAMILY) | |
| 2511 | lib_dib_Filter15_IDBA_contig_46873_minus2 | 1233 | pancreatic triacylglycerol lipase-like | 626 | 4.05E-104 | 53.00% | 3 | P:lipid metabolic process; F:protein binding; F:hydrolase activity | IPR000734 (PRINTS); IPR001024 (G3DSA:2.60.60.GENE3D); IPR002331 (PRINTS); IPR008976 (SUPERFAMILY); IPR013818 (PFAM); G3DSA:3.40.50.1820 (GENE3D), tmhmm (TMHMM), SSF53474 (SUPERFAMILY) | |
| 2512 | lib_dib_Filter15_IDBA_contig_46874_minus2 | 354 | pancreatic triacylglycerol lipase-like | 249 | 1.44E-34 | 57.25% | 2 | P:lipid metabolic process; F:hydrolase activity | IPR000734 (PRINTS); IPR002331 (PRINTS); IPR013818 (PFAM); G3DSA:3.40.50.1820 (GENE3D), tmhmm (TMHMM), SSF53474 (SUPERFAMILY) | |
| 2513 | lib_dib_Filter15_IDBA_contig_46875_minus3 | 42 | triacylglycerol lipase | 111 | 3.57E-16 | 56.80% | 8 | P:biological_process; P:catabolic process; P:lipid metabolic process; C:extracellular space; P:response to endogenous stimulus; P:transport; P:multicellular organismal development; F:hydrolase activity | IPR000734 (PANTHER); IPR013818 (PFAM); G3DSA:3.40.50.1820 (GENE3D), SSF53474 (SUPERFAMILY) | |
| 2514 | lib_dib_Filter15_IDBA_contig_4692_plus3 | 12191 | vitellogenin | 461 | 2.26E-45 | 48.25% | 2 | F:transporter activity; P:transport | IPR001747 (PFAM); IPR015816 (G3DSA:2.30.230.GENE3D); IPR015819 (SUPERFAMILY); PTHR23345 (PANTHER), SignalP-NN(euk) (SIGNALP), tmhmm (TMHMM) | yes |
| 2515 | lib_dib_Filter15_IDBA_contig_46932_minus2 | 257 | upf0369 protein c6orf57-like | 210 | 3.93E-20 | 65.25% | 2 | C:intracellular; C:mitochondrion | IPR012875 (PFAM) | |
| 2516 | lib_dib_Filter15_IDBA_contig_46933_minus2 | 733 | upf0369 protein c6orf57-like | 643 | 6.14E-21 | 64.30% | 1 | C:mitochondrion | IPR012875 (PFAM) | |
| 2517 | lib_dib_Filter15_IDBA_contig_46934_minus2 | 678 | upf0369 protein c6orf57-like | 633 | 2.71E-21 | 65.00% | 1 | C:mitochondrion | IPR012875 (PFAM) | |
| 2518 | lib_dib_Filter15_IDBA_contig_46946_plus2 | 31 | epidermal growth factor receptor | 221 | 6.25E-40 | 62.40% | 9 | P:multicellular organismal development; P:biological_process; P:signal transduction; P:cell differentiation; P:regulation of biological process; F:kinase activity; F:binding; P:cellular protein modification process; C:cell | IPR000494 (PFAM); G3DSA:3.80.20.20 (GENE3D), PTHR24416 (PANTHER), PTHR24416:SF88 (PANTHER), SignalP-NN(euk) (SIGNALP), tmhmm (TMHMM), SSF52058 (SUPERFAMILY) | |
| 2519 | lib_dib_Filter15_IDBA_contig_46980_plus3 | 302 | neuronal acetylcholine receptor subunit alpha-7 | 551 | 3.86E-61 | 55.65% | 3 | F:ion channel activity; P:ion transport; C:cell | IPR002394 (PRINTS); IPR006029 (PFAM); IPR006201 (PRINTS); IPR006202 (G3DSA:2.70.170.GENE3D); IPR018000 (PROSITE); G3DSA:1.20.120.370 (GENE3D), PTHR18945:SF153 (PANTHER), tmhmm (TMHMM) | |

| | | | | | | | | |
|---|---|---|---|---|---|---|---|---|
| 2520 | lib_dib_Filter15_IDBA_contig_47035_minus3 | 30 | thrombospondin type-1 domain-containing protein 4 | 220 | 8.94E-72 | 56.25% | 5 | C:extracellular matrix; F:peptidase activity; F:metalloendopeptidase activity; P:elastic fiber assembly; C:microfibril | IPR000884 (PFAM); G3DSA:2.20.100.10 (GENE3D), PTHR13723 (PANTHER) |
| 2521 | lib_dib_Filter15_IDBA_contig_47179_plus3 | 27 | cubilin- partial | 132 | 4.30E-17 | 59.80% | 13 | F:peptidase activity; P:primary metabolic process; P:protein transport; F:binding; C:cell; C:cytoplasmic membrane-bounded vesicle; C:endosome; F:protein binding; C:plasma membrane; P:transport; P:cellular component organization; C:Golgi apparatus; C:endoplasmic reticulum | IPR000859 (G3DSA:2.60.120.GENE3D); PTHR10127 (PANTHER) |
| 2522 | lib_dib_Filter15_IDBA_contig_47180_plus3 | 45 | cubilin- partial | 155 | 1.72E-17 | 59.15% | 1 | F:hydrolase activity | IPR000859 (G3DSA:2.60.120.GENE3D); PTHR10127 (PANTHER) |
| 2523 | lib_dib_Filter15_IDBA_contig_47181_plus2 | 23 | bone morphogenic protein 1 | 220 | 1.05E-29 | 48.05% | 2 | F:binding; F:catalytic activity | IPR000859 (G3DSA:2.60.120.GENE3D); PTHR10127 (PANTHER) |
| 2524 | lib_dib_Filter15_IDBA_contig_47205_plus3 | 36 | protein o-mannosyltransferase 1-like | 220 | 9.47E-122 | 86.05% | 5 | P:biosynthetic process; P:cellular protein modification process; P:carbohydrate metabolic process; C:cell; F:transferase activity | IPR016093 (PFAM); IPR027005 (PANTHER); G3DSA:2.80.10.50 (GENE3D), PTHR10050:SF18 (PANTHER) |
| 2525 | lib_dib_Filter15_IDBA_contig_47231_minus3 | 21 | low-density lipoprotein receptor-related protein 2-like | 220 | 5.23E-49 | 61.95% | 1 | F:protein binding | IPR002172 (G3DSA:4.10.400.GENE3D); IPR011042 (G3DSA:2.120.10.GENE3D); IPR023415 (PROSITE); PR00261 (PRINTS), G3DSA:2.10.25.10 (GENE3D), PTHR10529 (PANTHER), PTHR10529:SF113 (PANTHER), SSF57196 (SUPERFAMILY) |
| 2526 | lib_dib_Filter15_IDBA_contig_4725_minus1 | 558 | latrophilin-3-like isoform 5 | 696 | 2.32E-136 | 51.50% | 3 | P:signal transduction; F:receptor activity; C:cell | IPR000203 (PFAM); IPR000832 (PRINTS); IPR001879 (SMART); IPR017981 (PROFILE); IPR022624 (PFAM); PTHR12011 (PANTHER), PTHR12011:SF62 (PANTHER), tmhmm (TMHMM) |
| 2527 | lib_dib_Filter15_IDBA_contig_47273_plus1 | 63 | low-density lipoprotein receptor-related protein 2 | 228 | 3.15E-23 | 58.30% | 1 | F:protein binding | IPR002172 (G3DSA:4.10.400.GENE3D); IPR023415 (PROSITE); PR00261 (PRINTS), G3DSA:4.10.1220.10 (GENE3D), PTHR10529 (PANTHER) |
| 2528 | lib_dib_Filter15_IDBA_contig_47274_plus3 | 33 | low-density lipoprotein receptor-related protein 1b-like | 116 | 1.29E-23 | 61.00% | 1 | F:protein binding | IPR002172 (G3DSA:4.10.400.GENE3D); IPR023415 (PROSITE); PR00261 (PRINTS), PTHR10529 (PANTHER) |
| 2529 | lib_dib_Filter15_IDBA_contig_47275_plus3 | 42 | low-density lipoprotein receptor-related protein 4 | 219 | 5.34E-34 | 54.35% | 11 | P:multicellular organismal development; F:peptidase activity; F:protein binding; P:anatomical structure morphogenesis; P:embryo development; P:signal transduction; P:protein metabolic process; P:biological_process; P:cellular component organization; P:cell differentiation; C:plasma membrane | IPR002172 (G3DSA:4.10.400.GENE3D); IPR023415 (PROSITE); PR00261 (PRINTS), PTHR10529 (PANTHER) |
| 2530 | lib_dib_Filter15_IDBA_contig_47295_plus2 | 177 | chondroitin sulfate synthase 1 | 532 | 2.45E-130 | 71.90% | 10 | P:anatomical structure morphogenesis; P:multicellular organismal development; P:regulation of biological process; C:extracellular region; P:embryo development; F:transferase activity; P:biosynthetic process; P:carbohydrate metabolic process; P:signal transduction; C:Golgi apparatus | IPR008428 (PANTHER); PTHR12369:SF5 (PANTHER), tmhmm (TMHMM) |

| | | | | | | | | | |
|---|---|---|---|---|---|---|---|---|---|
| 2531 | lib_dib_Filter15_IDBA_contig_4734_minus1 | 3970 | sparc | 118 | 2.61E-12 | 75.05% | 12 | P:multicellular organismal development; P:regulation of biological process; P:growth; F:calcium ion binding; P:anatomical structure morphogenesis; P:embryo development; C:proteinaceous extracellular matrix; C:extracellular space; P:biological_process; P:transport; P:cellular component organization; P:signal transduction | IPR011992 (G3DSA:1.10.238.GENE3D); IPR018247 (PROSITE); IPR019577 (PFAM); PTHR13866 (PANTHER), PTHR13866:SF5 (PANTHER), SSF47473 (SUPERFAMILY) |
| 2532 | lib_dib_Filter15_IDBA_contig_47348_plus3 | 68 | phosphatidylserine phosphatidylglycerophosphate cardiolipin synthase-like protein | 219 | 3.17E-39 | 73.90% | 13 | P:reproduction; P:cell differentiation; P:cytoplasm organization; P:embryo development; P:organelle organization; P:nucleobase-containing compound metabolic process; C:mitochondrion; P:cell cycle; P:anatomical structure morphogenesis; P:mitochondrion organization; P:regulation of gene expression, epigenetic; P:DNA metabolic process; F:protein binding | IPR001736 (SMART); IPR025202 (PFAM); G3DSA:3.30.870.10 (GENE3D), PTHR21248 (PANTHER), PTHR21248:SF0 (PANTHER), SSF56024 (SUPERFAMILY) |
| 2533 | lib_dib_Filter15_IDBA_contig_47404_minus1 | 43 | frizzled homolog 9 | 220 | 2.96E-63 | 59.20% | 11 | F:protein binding; P:behavior; P:signal transduction; P:cell differentiation; P:multicellular organismal development; P:cell proliferation; P:regulation of biological process; P:nucleobase-containing compound metabolic process; F:receptor activity; C:plasma membrane; C:cytoplasm | IPR000539 (PRINTS); IPR015526 (PANTHER); IPR020067 (G3DSA:1.10.2000.GENE3D); IPR026546 (PTHR11309:PANTHER); tmhmm (TMHMM) |
| 2534 | lib_dib_Filter15_IDBA_contig_47409_minus2 | 33 | anti-dorsalizing morphogenic protein precursor | 219 | 3.81E-48 | 55.55% | 4 | P:biological_process; C:cell; F:receptor binding; P:growth | IPR001111 (PFAM); IPR015615 (PANTHER); PTHR11848:SF45 (PANTHER), SignalP-NN(euk) (SIGNALP), tmhmm (TMHMM) |
| 2535 | lib_dib_Filter15_IDBA_contig_47427_plus2 | 18 | dipeptidyl peptidase 1 | 219 | 1.04E-70 | 64.80% | 5 | C:lysosome; F:peptidase activity; P:biological_process; P:protein metabolic process; P:catabolic process | IPR000169 (PROSITE); IPR000668 (PRINTS); IPR013128 (PANTHER); IPR025660 (PROSITE); G3DSA:3.90.70.10 (GENE3D), PTHR12411:SF17 (PANTHER), SSF54001 (SUPERFAMILY) |
| 2536 | lib_dib_Filter15_IDBA_contig_47435_plus3 | 1789 | nicotinic acetylcholine receptor alpha6 subunit | 716 | 4.59E-58 | 57.45% | 23 | C:cell; P:response to external stimulus; P:regulation of biological process; P:response to stress; C:plasma membrane; P:behavior; C:cytoskeleton; P:biological_process; F:protein binding; P:biosynthetic process; ; F:ion channel activity; P:cell-cell signaling; F:binding; P:reproduction; C:cytoplasm; P:ion transport; P:transport; P:cellular component organization; C:protein complex; P:cell communication; P:cellular homeostasis; C:cellular_component | IPR002394 (PRINTS); IPR006029 (PFAM); IPR006201 (PRINTS); IPR006202 (G3DSA:2.70.170.GENE3D); IPR018000 (PROSITE); G3DSA:1.20.120.370 (GENE3D), PTHR18945:SF92 (PANTHER), tmhmm (TMHMM) |
| 2537 | lib_dib_Filter15_IDBA_contig_47495_plus1 | 913 | cathepsin l-like | 407 | 4.10E-114 | 68.25% | 3 | P:protein metabolic process; P:catabolic process; F:peptidase activity | IPR000169 (PROSITE); IPR000668 (PRINTS); IPR013128 (PANTHER); IPR013201 (PFAM); IPR025660 (PROSITE); IPR025661 (PROSITE); G3DSA:3.90.70.10 (GENE3D), PTHR12411:SF149 (PANTHER), tmhmm (TMHMM), SSF54001 (SUPERFAMILY) |
| 2538 | lib_dib_Filter15_IDBA_contig_47539_plus1 | 39 | chymotrypsinogen b | 212 | 5.62E-30 | 56.85% | 3 | P:protein metabolic process; P:catabolic process; F:peptidase activity | IPR001254 (PFAM); IPR001314 (PRINTS); IPR009003 (SUPERFAMILY); IPR018114 (PROSITE); G3DSA:2.40.10.10 (GENE3D), PTHR24272 (PANTHER) |

| | | | | | | | | | | |
|---|---|---|---|---|---|---|---|---|---|---|
| 2539 | lib_dib_Filter15_IDBA_contig_47540_plus1 | 150 | plasma kallikrein | 583 | 2.14E-52 | 55.45% | 10 | P:protein metabolic process; P:response to external stimulus; P:response to stress; C:extracellular space; P:regulation of biological process; F:protein binding; P:cellular component organization; C:cell; F:peptidase activity; P:catabolic process | IPR000859 (G3DSA:2.60.120.GENE3D); IPR000998 (PFAM); IPR001254 (PFAM); IPR001314 (PRINTS); IPR008985 (SUPERFAMILY); IPR009003 (SUPERFAMILY); IPR018114 (PROSITE); G3DSA:2.40.10.10 (GENE3D), PTHR24265 (PANTHER) | |
| 2540 | lib_dib_Filter15_IDBA_contig_47546_minus1 | 20 | n-acetylgalactosaminyltransferase 7-like | 219 | 2.84E-33 | 64.35% | 2 | F:transferase activity; C:cell | IPR001173 (PFAM); G3DSA:3.90.550.10 (GENE3D), PTHR11675 (PANTHER), SSF53448 (SUPERFAMILY) | |
| 2541 | lib_dib_Filter15_IDBA_contig_47568_minus3 | 130 | nicotinic acetylcholine receptor subunit type g | 516 | 2.12E-50 | 54.45% | 3 | P:ion transport; C:cell; F:ion channel activity | IPR006029 (PFAM); IPR006201 (PRINTS); IPR006202 (G3DSA:2.70.170.GENE3D); IPR018000 (PROSITE); G3DSA:1.20.120.370 (GENE3D), PTHR18945:SF153 (PANTHER), SignalP-NN(euk) (SIGNALP), tmhmm (TMHMM) | yes |
| 2542 | lib_dib_Filter15_IDBA_contig_47640_minus3 | 26 | -cyclic-nucleotide 2-phosphodiesterase | 218 | 2.42E-66 | 67.00% | 3 | F:hydrolase activity; P:nucleobase-containing compound metabolic process; P:catabolic process | IPR006179 (PANTHER); IPR008334 (G3DSA:3.90.780.GENE3D); PTHR11575:SF8 (PANTHER) | |
| 2543 | lib_dib_Filter15_IDBA_contig_47648_plus2 | 24 | matrix metalloproteinase 9 | 218 | 6.90E-15 | 45.15% | 1 | F:hydrolase activity | IPR000001 (PROFILE); IPR000562 (G3DSA:2.10.10.GENE3D); IPR013806 (SUPERFAMILY); PTHR22918 (PANTHER), PTHR22918:SF0 (PANTHER) | |
| 2544 | lib_dib_Filter15_IDBA_contig_477_plus2 | 3304 | 5 ecto-like | 305 | 8.68E-77 | 68.05% | 7 | P:biological_process; F:hydrolase activity; C:cell; F:nucleotide binding; F:binding; P:nucleobase-containing compound metabolic process; P:catabolic process | IPR004843 (PFAM); IPR006146 (PROSITE); IPR006179 (PRINTS); G3DSA:3.60.21.10 (GENE3D), PTHR11575:SF7 (PANTHER), SignalP-NN(euk) (SIGNALP), tmhmm (TMHMM), SSF56300 (SUPERFAMILY) | |
| 2545 | lib_dib_Filter15_IDBA_contig_47801_plus3 | 826 | zinc finger protein 16-like | 940 | 6.78E-41 | 50.85% | 4 | F:protein binding; F:binding; F:nucleic acid binding; C:intracellular | IPR000210 (SMART); IPR007087 (PFAM); IPR011333 (G3DSA:3.30.710.GENE3D); IPR013069 (PFAM); IPR013087 (G3DSA:3.30.160.GENE3D); IPR015880 (SMART); PTHR11389 (PANTHER), PF13465 (PFAM), PF13894 (PFAM), SSF57667 (SUPERFAMILY) | |
| 2546 | lib_dib_Filter15_IDBA_contig_47837_plus2 | 51 | thioredoxin domain-containing protein 16 | 238 | 1.00E-31 | 49.90% | 2 | P:cell redox homeostasis; C:extracellular region | IPR012336 (G3DSA:3.40.30.GENE3D); PTHR22699 (PANTHER) | |
| 2547 | lib_dib_Filter15_IDBA_contig_47848_minus1 | 67 | zinc finger | 402 | 8.56E-58 | 78.70% | 3 | F:binding; F:nucleic acid binding; C:intracellular | IPR007087 (PROSITE); IPR013087 (G3DSA:3.30.160.GENE3D); IPR015880 (SMART); PTHR23229 (PANTHER), PTHR23229:SF46 (PANTHER), PF13465 (PFAM), SSF57667 (SUPERFAMILY) | |
| 2548 | lib_dib_Filter15_IDBA_contig_47881_minus3 | 28 | pregnancy-associated plasma protein a | 217 | 1.88E-37 | 53.20% | 3 | F:peptidase activity; C:extracellular region; P:metabolic process | IPR000436 (PFAM); G3DSA:2.10.70.10 (GENE3D), PTHR19325 (PANTHER), PTHR19325:SF119 (PANTHER) | |
| 2549 | lib_dib_Filter15_IDBA_contig_47902_plus3 | 24 | ---NA--- | 217 | | | 0 | - | IPR011677 (PFAM); PTHR14611 (PANTHER), PTHR14611:SF0 (PANTHER) | |
| 2550 | lib_dib_Filter15_IDBA_contig_47938_plus3 | 1184 | cytosolic non-specific dipeptidase | 217 | 6.65E-79 | 80.95% | 3 | F:peptidase activity; P:protein metabolic process; P:catabolic process | IPR001261 (PROSITE); IPR002933 (PFAM); G3DSA:3.40.630.10 (GENE3D), PTHR11014 (PANTHER), PTHR11014:SF15 (PANTHER), SSF53187 (SUPERFAMILY) | |
| 2551 | lib_dib_Filter15_IDBA_contig_48020_plus2 | 28 | 3-oxoacyl- | 217 | 9.99E-43 | 64.35% | 3 | P:metabolic process; F:catalytic activity; F:nucleotide binding | IPR002198 (PRINTS); IPR002347 (PRINTS); IPR016040 (G3DSA:3.40.50.GENE3D); PTHR24322 (PANTHER), PTHR24322:SF0 (PANTHER), SSF51735 (SUPERFAMILY) | |
| 2552 | lib_dib_Filter15_IDBA_contig_48046_plus1 | 453 | cre-pat-3 protein | 261 | 4.54E-09 | 57.75% | 6 | P:biological_process; P:regulation of biological process; C:cytoplasm; C:cell; F:protein binding; P:multicellular organismal development | IPR015812 (PANTHER); G3DSA:2.10.25.10 (GENE3D), PTHR10082:SF16 (PANTHER), SignalP-NN(euk) (SIGNALP), tmhmm (TMHMM), SSF57196 (SUPERFAMILY) | |

| | | | | | | | | | |
|---|---|---|---|---|---|---|---|---|---|
| 2553 | lib_dib_Filter15_IDBA_contig_48047_plus1 | 638 | cre-pat-3 protein | 261 | 2.38E-09 | 57.70% | 6 | P:biological_process; P:regulation of biological process; C:cytoplasm; C:cell; F:protein binding; P:multicellular organismal development | IPR015812 (PANTHER); G3DSA:2.10.25.10 (GENE3D), PTHR10082:SF16 (PANTHER), SignalP-NN(euk) (SIGNALP), tmhmm (TMHMM), SSF57196 (SUPERFAMILY) | |
| 2554 | lib_dib_Filter15_IDBA_contig_48049_plus1 | 295 | integrin beta-1-like | 153 | 2.04E-18 | 59.35% | 8 | P:biological_process; P:anatomical structure morphogenesis; P:embryo development; F:protein binding; P:cell differentiation; P:multicellular organismal development; P:regulation of biological process; C:plasma membrane | IPR013111 (PFAM); IPR015812 (PANTHER); G3DSA:2.10.25.10 (GENE3D), PTHR10082:SF16 (PANTHER), SignalP-NN(euk) (SIGNALP), SSF57196 (SUPERFAMILY) | yes |
| 2555 | lib_dib_Filter15_IDBA_contig_48170_minus2 | 31 | loc100137684 protein | 216 | 2.91E-24 | 58.55% | 2 | F:receptor activity; C:cell | IPR000859 (G3DSA:2.60.120.GENE3D); IPR001190 (PRINTS); IPR017448 (SMART); G3DSA:3.10.250.10 (GENE3D), PTHR19331 (PANTHER) | |
| 2556 | lib_dib_Filter15_IDBA_contig_48221_minus2 | 141 | matrix metalloproteinase-14-like | 216 | 6.16E-12 | 61.95% | 2 | F:binding; F:peptidase activity | IPR002477 (G3DSA:1.10.101.GENE3D); PTHR10201 (PANTHER) | |
| 2557 | lib_dib_Filter15_IDBA_contig_48336_minus3 | 641 | chymotrypsin-like elastase family member 2a-like | 447 | 1.01E-78 | 59.25% | 3 | F:peptidase activity; P:protein metabolic process; P:catabolic process | IPR000519 (G3DSA:4.10.110.GENE3D); IPR001254 (PFAM); IPR001314 (PRINTS); IPR009003 (SUPERFAMILY); IPR017957 (PROSITE); IPR018114 (PROSITE); G3DSA:2.40.10.10 (GENE3D), PTHR24256 (PANTHER), SignalP-NN(euk) (SIGNALP) | yes |
| 2558 | lib_dib_Filter15_IDBA_contig_48347_minus1 | 58 | ankyrin repeat and sam domain-containing protein 3 | 216 | 6.73E-17 | 52.90% | 1 | F:protein binding | IPR002110 (PFAM); IPR020683 (G3DSA:1.25.40.GENE3D); PTHR24128 (PANTHER) | |
| 2559 | lib_dib_Filter15_IDBA_contig_48382_minus2 | 35 | zinc finger protein gfi-1b | 216 | 2.59E-42 | 83.70% | 13 | P:regulation of biological process; ; F:binding; P:cell proliferation; P:cell differentiation; P:multicellular organismal development; C:nucleoplasm; C:protein complex; F:DNA binding; C:nucleus; P:cell cycle; P:organelle organization; P:cellular protein modification process | IPR007087 (PFAM); IPR013087 (G3DSA:3.30.160.GENE3D); IPR015880 (SMART); PTHR23228 (PANTHER), PTHR23228:SF42 (PANTHER), PF13465 (PFAM), SSF57667 (SUPERFAMILY) | |
| 2560 | lib_dib_Filter15_IDBA_contig_48391_plus1 | 131 | protocadherin fat 3-like | 359 | 1.48E-16 | 56.10% | 3 | F:calcium ion binding; P:biological_process; C:cell | IPR002126 (G3DSA:2.60.40.GENE3D); IPR015919 (SUPERFAMILY); PTHR24026 (PANTHER), PTHR24026:SF0 (PANTHER), tmhmm (TMHMM) | |
| 2561 | lib_dib_Filter15_IDBA_contig_48410_minus3 | 25 | microneme protein 4 | 215 | 3.05E-27 | 56.70% | 2 | F:protein binding; F:calcium ion binding | IPR000152 (PROSITE); IPR000742 (SMART); IPR001881 (PFAM); IPR002919 (SUPERFAMILY); IPR013032 (PROSITE); IPR018097 (PROSITE); IPR025615 (PFAM); G3DSA:2.10.25.10 (GENE3D), PTHR24034 (PANTHER), PTHR24034:SF0 (PANTHER), SSF57196 (SUPERFAMILY) | |
| 2562 | lib_dib_Filter15_IDBA_contig_4844_minus1 | 60 | sparc cwcv and kazal-like domains proteoglycan 2 precursor | 393 | 6.49E-39 | 54.05% | 3 | F:calcium ion binding; C:proteinaceous extracellular matrix; P:signal transduction | IPR000716 (G3DSA:4.10.800.GENE3D); IPR011992 (G3DSA:1.10.238.GENE3D); IPR018247 (PROSITE); IPR019577 (PFAM); PTHR12036 (PANTHER), SSF47473 (SUPERFAMILY) | |
| 2563 | lib_dib_Filter15_IDBA_contig_48462_minus2 | 21 | trophoblast glyco | 215 | 6.18E-19 | 48.15% | 1 | F:protein binding | IPR001611 (PFAM); IPR003591 (SMART); G3DSA:3.80.10.10 (GENE3D), PTHR24365 (PANTHER), PF13855 (PFAM), SSF52058 (SUPERFAMILY) | |
| 2564 | lib_dib_Filter15_IDBA_contig_48586_plus3 | 42 | beta- -galactosyl-o-glycosyl-glycoprotein beta- -n-acetylglucosaminyltransferase 3 | 215 | 1.58E-50 | 65.00% | 5 | C:extracellular space; P:anatomical structure morphogenesis; F:transferase activity; P:response to endogenous stimulus; C:cell | IPR003406 (PFAM); PTHR19297 (PANTHER), PTHR19297:SF6 (PANTHER) | |
| 2565 | lib_dib_Filter15_IDBA_contig_48587_minus2 | 18 | ds_drome ame: full=protein dachsous ame: full=adherin flags: precursor | 215 | 8.76E-49 | 62.85% | 3 | F:calcium ion binding; P:biological_process; C:plasma membrane | IPR002126 (PRINTS); IPR015919 (SUPERFAMILY); IPR020894 (PROSITE); PTHR24027 (PANTHER), PTHR24027:SF53 (PANTHER) | |

| | | | | | | | | | | |
|---|---|---|---|---|---|---|---|---|---|---|
| 2566 | lib_dib_Filter15_IDBA_contig_48714_minus1 | 25 | dimethylaniline monooxygenase | 215 | 2.22E-72 | 65.45% | 5 | F:catalytic activity; C:endoplasmic reticulum; C:cell; F:nucleotide binding; P:metabolic process | IPR000960 (PRINTS); IPR020946 (PFAM); G3DSA:3.50.50.60 (GENE3D), PTHR23023 (PANTHER), PTHR23023:SF4 (PANTHER), SSF51905 (SUPERFAMILY) | |
| 2567 | lib_dib_Filter15_IDBA_contig_48725_plus3 | 113 | arylsulfatase b | 521 | 2.19E-106 | 56.75% | 2 | P:metabolic process; F:hydrolase activity | IPR000917 (PFAM); IPR017849 (G3DSA:3.40.720.GENE3D); IPR017850 (SUPERFAMILY); G3DSA:3.30.1120.10 (GENE3D), PTHR10342 (PANTHER), PTHR10342:SF19 (PANTHER), tmhmm (TMHMM) | |
| 2568 | lib_dib_Filter15_IDBA_contig_48727_plus1 | 24 | laminin subunit gamma-1 | 215 | 1.43E-63 | 78.05% | 13 | P:biological_process; P:anatomical structure morphogenesis; P:cellular component organization; P:cell differentiation; P:multicellular organismal development; C:proteinaceous extracellular matrix; C:protein complex; P:regulation of biological process; P:cell proliferation; F:lipid binding; C:extracellular space; F:protein binding; F:structural molecule activity | IPR008211 (PFAM); G3DSA:2.60.120.260 (GENE3D), PTHR10574 (PANTHER), PTHR10574:SF50 (PANTHER), SignalP-NN(euk) (SIGNALP) | yes |
| 2569 | lib_dib_Filter15_IDBA_contig_48732_minus3 | 42 | leucine rich repeat protein | 214 | 1.43E-49 | 61.15% | 1 | F:protein binding | IPR001611 (PROFILE); IPR003591 (SMART); IPR025875 (PFAM); PR00019 (PRINTS), G3DSA:3.80.10.10 (GENE3D), PTHR23155 (PANTHER), PF13855 (PFAM), SM00364 (SMART), SSF52058 (SUPERFAMILY) | |
| 2570 | lib_dib_Filter15_IDBA_contig_48769_minus1 | 344 | ---NA--- | 763 | | | 0 | - | SignalP-NN(euk) (SIGNALP), tmhmm (TMHMM) | yes |
| 2571 | lib_dib_Filter15_IDBA_contig_48852_plus1 | 21 | gelatinase b | 214 | 7.54E-08 | 75.35% | 26 | P:multicellular organismal development; P:reproduction; P:viral reproduction; P:symbiosis, encompassing mutualism through parasitism; P:biological_process; P:response to biotic stimulus; P:anatomical structure morphogenesis; P:cellular component organization; P:response to external stimulus; P:response to abiotic stimulus; P:regulation of biological process; P:cell-cell signaling; P:response to endogenous stimulus; C:extracellular space; P:cell communication; P:response to stress; F:binding; F:peptidase activity; P:cell death; C:protein complex; P:metabolic process; P:protein metabolic process; P:catabolic process; F:protein binding; P:cell growth; C:extracellular region | IPR000562 (G3DSA:2.10.10.GENE3D); IPR013806 (SUPERFAMILY); PR00013 (PRINTS), PTHR22918 (PANTHER), PTHR22918:SF0 (PANTHER) | |
| 2572 | lib_dib_Filter15_IDBA_contig_48925_minus3 | 26 | tyrosine-protein kinase pr2 | 213 | 3.01E-86 | 71.65% | 6 | F:protein kinase activity; P:cellular protein modification process; P:anatomical structure morphogenesis; P:cell death; P:multicellular organismal development; F:nucleotide binding | IPR000719 (PROFILE); IPR001245 (PFAM); IPR002290 (SMART); IPR008266 (PROSITE); IPR011009 (SUPERFAMILY); IPR017441 (PROSITE); IPR020635 (SMART); G3DSA:1.10.510.10 (GENE3D), G3DSA:3.30.200.20 (GENE3D), PTHR24418 (PANTHER), PTHR24418:SF59 (PANTHER) | |
| 2573 | lib_dib_Filter15_IDBA_contig_49023_plus3 | 39 | variable lymphocyte receptor a diversity region | 213 | 5.86E-15 | 55.45% | 1 | F:receptor activity | IPR003591 (SMART); G3DSA:3.80.10.10 (GENE3D), PTHR24365 (PANTHER), PF13855 (PFAM), tmhmm (TMHMM), SSF52058 (SUPERFAMILY) | |
| 2574 | lib_dib_Filter15_IDBA_contig_49042_minus3 | 21 | cadherin egf lag seven-pass g-type receptor 2 | 213 | 1.63E-29 | 51.75% | 3 | F:calcium ion binding; P:biological_process; C:plasma membrane | IPR002126 (PRINTS); IPR015919 (SUPERFAMILY); IPR020894 (PROSITE); PTHR24027 (PANTHER) | |

| | | | | | | | | | | |
|---|---|---|---|---|---|---|---|---|---|---|
| 2575 | lib_dib_Filter15_IDBA_contig_49063_minus2 | 15 | d-glucuronyl c5-epimerase | 213 | 3.88E-67 | 76.85% | 5 | F:catalytic activity; C:Golgi apparatus; P:biosynthetic process; C:cell; P:carbohydrate metabolic process | PTHR13174 (PANTHER), SignalP-NN(euk) (SIGNALP) | yes |
| 2576 | lib_dib_Filter15_IDBA_contig_49065_minus2 | 39 | fat-like cadherin-related tumor suppressor-like protein | 245 | 2.31E-57 | 62.90% | 8 | P:anatomical structure morphogenesis; P:cellular component organization; P:cytoskeleton organization; C:cell; C:plasma membrane; F:protein binding; F:calcium ion binding; P:biological_process | IPR002126 (PRINTS); IPR015919 (SUPERFAMILY); PTHR24027 (PANTHER), PTHR24027:SF110 (PANTHER) | |
| 2577 | lib_dib_Filter15_IDBA_contig_49093_minus1 | 24 | beta-mannosidase | 213 | 7.73E-46 | 64.55% | 2 | F:hydrolase activity; P:carbohydrate metabolic process | IPR006104 (PFAM); IPR008979 (SUPERFAMILY); G3DSA:2.60.120.260 (GENE3D), PTHR10066 (PANTHER), PTHR10066:SF12 (PANTHER), tmhmm (TMHMM) | |
| 2578 | lib_dib_Filter15_IDBA_contig_49144_minus1 | 633 | dipeptidyl peptidase | 213 | 2.50E-36 | 50.90% | 4 | F:peptidase activity; P:protein metabolic process; P:catabolic process; C:cell | IPR002469 (PFAM); G3DSA:2.140.10.30 (GENE3D), PTHR11731 (PANTHER), PTHR11731:SF23 (PANTHER), SSF82171 (SUPERFAMILY) | |
| 2579 | lib_dib_Filter15_IDBA_contig_49174_plus2 | 46 | isoform b | 256 | 2.11E-10 | 54.55% | 8 | P:cell-cell signaling; P:transport; C:cellular_component; P:anatomical structure morphogenesis; P:multicellular organismal development; P:cell differentiation; C:cell; P:cellular component organization | IPR000483 (SMART); G3DSA:3.80.10.10 (GENE3D), PTHR24373 (PANTHER), tmhmm (TMHMM), SSF52058 (SUPERFAMILY) | |
| 2580 | lib_dib_Filter15_IDBA_contig_49206_plus2 | 24 | glycosaminoglycan xylosylkinase-like | 212 | 5.17E-52 | 64.25% | 6 | F:transferase activity; P:biosynthetic process; P:anatomical structure morphogenesis; P:embryo development; C:cell; C:Golgi apparatus | IPR009581 (PFAM); IPR024869 (PANTHER) | |
| 2581 | lib_dib_Filter15_IDBA_contig_49262_plus1 | 101 | cytokine receptor-like factor 1 | 409 | 2.62E-17 | 39.10% | 1 | F:protein binding | IPR003961 (PFAM); IPR007110 (PROFILE); IPR013783 (G3DSA:2.60.40.GENE3D) | |
| 2582 | lib_dib_Filter15_IDBA_contig_49313_minus1 | 15 | adenosine deaminase | 212 | 5.63E-80 | 66.85% | 15 | P:nucleobase-containing compound metabolic process; P:biosynthetic process; P:signal transduction; P:regulation of biological process; P:catabolic process; P:cell differentiation; P:multicellular organismal development; C:lysosome; C:cytosol; P:biological_process; P:response to stress; F:hydrolase activity; F:binding; F:protein binding; C:plasma membrane | IPR001365 (PFAM); IPR006330 (TIGRFAMs); G3DSA:3.20.20.140 (GENE3D), PTHR11409 (PANTHER), PTHR11409:SF35 (PANTHER), SSF51556 (SUPERFAMILY) | |
| 2583 | lib_dib_Filter15_IDBA_contig_49314_plus2 | 63 | mam domain-containing glycosylphosphatidylinositol anchor protein 1 | 384 | 1.13E-60 | 56.40% | 1 | C:cell | IPR000998 (PRINTS); IPR008985 (SUPERFAMILY); PTHR23282 (PANTHER) | |
| 2584 | lib_dib_Filter15_IDBA_contig_49315_plus3 | 41 | mam domain-containing glycosylphosphatidylinositol anchor protein 1 | 215 | 3.47E-35 | 57.10% | 1 | C:plasma membrane | IPR000998 (PRINTS); IPR008985 (SUPERFAMILY); PTHR23282 (PANTHER) | |
| 2585 | lib_dib_Filter15_IDBA_contig_49358_minus2 | 1317 | fibrinogen-like protein 1 | 727 | 3.82E-67 | 58.00% | 4 | F:protein binding; P:signal transduction; P:multicellular organismal development; C:cell | IPR000742 (SMART); IPR001774 (PFAM); IPR002181 (PFAM); IPR011651 (PFAM); IPR013032 (PROSITE); IPR014715 (G3DSA:4.10.530.GENE3D); IPR014716 (G3DSA:3.90.215.GENE3D); IPR020837 (PROSITE); G3DSA:2.170.300.10 (GENE3D), PTHR19143 (PANTHER), SignalP-NN(euk) (SIGNALP), SSF57196 (SUPERFAMILY) | yes |

| | | | | | | | | | | |
|---|---|---|---|---|---|---|---|---|---|---|
| 2586 | lib_dib_Filter15_IDBA_contig_49359_minus2 | 1408 | fibrinogen-like protein 1 | 705 | 1.58E-67 | 57.80% | 4 | F:protein binding; P:signal transduction; P:multicellular organismal development; C:cell | IPR000742 (SMART); IPR001774 (PFAM); IPR002181 (PFAM); IPR011651 (PFAM); IPR013032 (PROSITE); IPR014715 (G3DSA:4.10.530.GENE3D); IPR014716 (G3DSA:3.90.215.GENE3D); IPR020837 (PROSITE); G3DSA:2.170.300.10 (GENE3D), PTHR19143 (PANTHER), SignalP-NN(euk) (SIGNALP), SSF57196 (SUPERFAMILY) | yes |
| 2587 | lib_dib_Filter15_IDBA_contig_49387_plus3 | 34 | serine proteinase | 211 | 3.33E-45 | 50.45% | 1 | F:enzyme regulator activity | IPR002223 (PRINTS); IPR020901 (PROSITE); PTHR10083 (PANTHER) | |
| 2588 | lib_dib_Filter15_IDBA_contig_49391_plus1 | 77 | multiple epidermal growth factor-like domains protein 8-like | 212 | 4.10E-43 | 64.95% | 3 | F:calcium ion binding; C:integral to membrane; C:membrane | IPR000859 (G3DSA:2.60.120.GENE3D); PTHR22991 (PANTHER), PTHR22991:SF0 (PANTHER), SignalP-NN(euk) (SIGNALP) | yes |
| 2589 | lib_dib_Filter15_IDBA_contig_49409_plus1 | 97 | membrane metallo isoform cra_a | 316 | 9.48E-25 | 60.55% | 8 | P:metabolic process; F:binding; C:cell; C:cytoplasmic membrane-bounded vesicle; P:biological_process; P:protein metabolic process; P:catabolic process; F:peptidase activity | IPR000718 (PANTHER); IPR008753 (PFAM); G3DSA:1.10.1380.10 (GENE3D), SignalP-NN(euk) (SIGNALP), tmhmm (TMHMM), SSF55486 (SUPERFAMILY) | yes |
| 2590 | lib_dib_Filter15_IDBA_contig_49471_plus1 | 774 | angiopoietin- partial | 382 | 1.38E-36 | 55.20% | 3 | F:receptor binding; C:extracellular space; P:signal transduction | IPR002181 (PFAM); IPR014715 (G3DSA:4.10.530.GENE3D); IPR014716 (G3DSA:3.90.215.GENE3D); IPR020837 (PROSITE); PTHR19143 (PANTHER), SignalP-NN(euk) (SIGNALP) | |
| 2591 | lib_dib_Filter15_IDBA_contig_49497_plus1 | 18 | probable beta-d-xylosidase 2-like | 212 | 2.53E-62 | 63.20% | 2 | F:hydrolase activity; P:carbohydrate metabolic process | IPR002772 (G3DSA:3.40.50.GENE3D); IPR026892 (PANTHER); PTHR30620:SF0 (PANTHER) | |
| 2592 | lib_dib_Filter15_IDBA_contig_49527_minus2 | 25 | inactive serine protease pamr1 | 211 | 3.01E-07 | 55.75% | 24 | F:hydrolase activity; C:integral to membrane; C:membrane; F:extracellular ligand-gated ion channel activity; P:ion transport; P:transport; C:endocytic vesicle; C:brush border; C:endosome membrane; F:cobalamin binding; P:receptor-mediated endocytosis; F:protein homodimerization activity; C:endosome; C:Golgi apparatus; C:lysosomal membrane; C:apical plasma membrane; P:lipoprotein transport; P:cholesterol metabolic process; C:endoplasmic reticulum; F:calcium ion binding; C:coated pit; P:proteolysis; F:serine-type endopeptidase activity; C:extracellular region | IPR000859 (G3DSA:2.60.120.GENE3D); PTHR10127 (PANTHER), SignalP-NN(euk) (SIGNALP), tmhmm (TMHMM) | |
| 2593 | lib_dib_Filter15_IDBA_contig_49531_plus2 | 33 | fibrillin-1- partial | 211 | 3.38E-21 | 50.30% | 2 | F:protein binding; F:calcium ion binding | IPR000152 (PROSITE); IPR000742 (PFAM); IPR001881 (SMART); IPR013032 (PROSITE); IPR018097 (PROSITE); G3DSA:2.10.25.10 (GENE3D), PTHR24838 (PANTHER), SSF57196 (SUPERFAMILY) | |
| 2594 | lib_dib_Filter15_IDBA_contig_49550_minus1 | 21 | allene oxide synthase-lipoxygenase | 211 | 1.14E-80 | 73.45% | 3 | F:catalytic activity; F:binding; P:metabolic process | IPR000907 (PANTHER); IPR013819 (PRINTS); G3DSA:1.20.245.10 (GENE3D), PTHR11771:SF16 (PANTHER) | |
| 2595 | lib_dib_Filter15_IDBA_contig_49576_plus1 | 26 | protein - partial | 211 | 4.19E-05 | 55.78% | 1 | F:calcium ion binding | IPR000859 (G3DSA:2.60.120.GENE3D); PTHR10127 (PANTHER), PTHR10127:SF75 (PANTHER), tmhmm (TMHMM) | |
| 2596 | lib_dib_Filter15_IDBA_contig_49647_minus2 | 727 | ---NA--- | 814 | | | 0 | - | G3DSA:2.10.60.10 (GENE3D), tmhmm (TMHMM), SSF57302 (SUPERFAMILY) | |
| 2597 | lib_dib_Filter15_IDBA_contig_49648_minus2 | 703 | ---NA--- | 808 | | | 0 | - | G3DSA:2.10.60.10 (GENE3D), tmhmm (TMHMM), SSF57302 (SUPERFAMILY) | |

| | | | | | | | | | |
|---|---|---|---|---|---|---|---|---|---|
| 2598 | lib_dib_Filter15_IDBA_contig_49737_plus1 | 24 | zinc finger protein 415 | 211 | 5.87E-29 | 55.45% | 5 | C:cytoplasm; C:nucleolus; C:cytoskeleton; F:binding; F:nucleic acid binding | IPR007087 (PROSITE); IPR013087 (G3DSA:3.30.160.GENE3D); IPR015880 (SMART); PTHR11389 (PANTHER), PF13465 (PFAM), SSF57667 (SUPERFAMILY) |
| 2599 | lib_dib_Filter15_IDBA_contig_49774_minus3 | 76 | beta- -galactosyl-o-glycosyl-glycoprotein beta- -n-acetylglucosaminyltransferase 4 | 337 | 4.20E-94 | 67.00% | 2 | F:transferase activity; C:cell | IPR003406 (PFAM); PTHR19297 (PANTHER), PTHR19297:SF6 (PANTHER) |
| 2600 | lib_dib_Filter15_IDBA_contig_49817_plus1 | 22 | beta- -n-acetylgalactosaminyltransferase bre-4-like | 210 | 9.04E-114 | 72.50% | 2 | F:transferase activity; P:carbohydrate metabolic process | IPR003859 (PRINTS); G3DSA:3.90.550.10 (GENE3D), PF13733 (PFAM), SSF53448 (SUPERFAMILY) |
| 2601 | lib_dib_Filter15_IDBA_contig_49869_minus1 | 84 | group xiia secretory phospholipase a2 precursor | 210 | 1.44E-26 | 53.15% | 5 | F:hydrolase activity; F:calcium ion binding; C:extracellular region; P:catabolic process; P:lipid metabolic process | IPR010711 (PANTHER); IPR013090 (PROSITE); IPR016090 (G3DSA:1.20.90.GENE3D); PTHR12824:SF3 (PANTHER), SignalP-NN(euk) (SIGNALP), tmhmm (TMHMM) |
| 2602 | lib_dib_Filter15_IDBA_contig_49973_plus1 | 44 | protein fem-1 homolog b | 210 | 2.04E-94 | 82.05% | 11 | P:reproduction; P:cell differentiation; P:multicellular organismal development; P:regulation of biological process; P:cell death; P:biological_process; C:nucleolus; F:receptor binding; F:catalytic activity; P:anatomical structure morphogenesis; C:cytoplasm | IPR002110 (SMART); IPR020683 (G3DSA:1.25.40.GENE3D); PTHR24173 (PANTHER), PTHR24173:SF6 (PANTHER), PF13637 (PFAM) |
| 2603 | lib_dib_Filter15_IDBA_contig_5_minus1 | 6614 | ---NA--- | 5279 | | | 0 | - | PTHR24023 (PANTHER), PTHR24023:SF79 (PANTHER) |
| 2604 | lib_dib_Filter15_IDBA_contig_5_plus1 | 6614 | collagen alpha-6 chain | 5279 | 5.33E-28 | 51.70% | 1 | F:protein binding | IPR002035 (PFAM); IPR008160 (PFAM); G3DSA:3.40.50.410 (GENE3D), PTHR24023 (PANTHER), tmhmm (TMHMM), SSF53300 (SUPERFAMILY) |
| 2605 | lib_dib_Filter15_IDBA_contig_5_plus2 | 6614 | cathepsin z-like proteinase | 5279 | 4.42E-04 | 42.00% | 2 | P:proteolysis; F:cysteine-type peptidase activity | no IPS match |
| 2606 | lib_dib_Filter15_IDBA_contig_5017_plus1 | 236 | deleted in malignant brain tumors 1 | 131 | 4.49E-22 | 53.60% | 2 | F:receptor activity; C:cell | IPR001190 (PRINTS); IPR017448 (SMART); G3DSA:3.10.250.10 (GENE3D), PTHR19331 (PANTHER), PTHR19331:SF118 (PANTHER) |
| 2607 | lib_dib_Filter15_IDBA_contig_50201_plus1 | 21 | protein | 209 | 1.01E-40 | 55.65% | 2 | F:protein binding; F:calcium ion binding | IPR000152 (PROSITE); IPR000742 (PFAM); IPR001881 (SMART); IPR013032 (PROSITE); PD936484 (PRODOM), PR00010 (PRINTS), G3DSA:2.10.25.10 (GENE3D), PTHR24044 (PANTHER), SSF57196 (SUPERFAMILY) |
| 2608 | lib_dib_Filter15_IDBA_contig_50208_plus1 | 176 | l-xylulose reductase | 382 | 2.74E-67 | 67.65% | 3 | P:carbohydrate metabolic process; F:catalytic activity; F:nucleotide binding | IPR002198 (PRINTS); IPR002347 (PRINTS); IPR016040 (G3DSA:3.40.50.GENE3D); IPR020842 (SMART); IPR020904 (PROSITE); PTHR24311 (PANTHER), PTHR24311:SF0 (PANTHER), SSF51735 (SUPERFAMILY) |
| 2609 | lib_dib_Filter15_IDBA_contig_50231_plus1 | 28 | protocadherin-11 x-linked | 209 | 5.88E-36 | 55.00% | 3 | F:calcium ion binding; P:biological_process; C:cell | IPR002126 (PRINTS); IPR015919 (SUPERFAMILY); PTHR24027 (PANTHER), PTHR24027:SF13 (PANTHER) |
| 2610 | lib_dib_Filter15_IDBA_contig_50244_minus3 | 192 | serine partial | 284 | 7.36E-28 | 61.80% | 3 | F:hydrolase activity; F:receptor activity; C:cell | IPR001190 (PRINTS); IPR017448 (SMART); G3DSA:3.10.250.10 (GENE3D), PTHR19331 (PANTHER), PTHR19331:SF118 (PANTHER) |
| 2611 | lib_dib_Filter15_IDBA_contig_50245_minus3 | 20 | lysyl oxidase homolog 4 | 130 | 7.92E-27 | 61.50% | 10 | ; P:cell differentiation; P:regulation of biological process; P:multicellular organismal development; F:catalytic activity; C:extracellular region; P:response to stress; F:binding; F:receptor activity; C:cell | IPR001190 (PRINTS); IPR017448 (SMART); G3DSA:3.10.250.10 (GENE3D), PTHR19331 (PANTHER) |

| | | | | | | | | | |
|---|---|---|---|---|---|---|---|---|---|
| 2612 | lib_dib_Filter15_IDBA_contig_50249_plus1 | 31 | procollagen c-endopeptidase enhancer 1 precursor | 209 | 2.06E-14 | 47.95% | 2 | F:peptidase activity; F:protein binding | IPR000859 (G3DSA:2.60.120.GENE3D); IPR002172 (G3DSA:4.10.400.GENE3D); IPR023415 (PROSITE); PTHR10127 (PANTHER) |
| 2613 | lib_dib_Filter15_IDBA_contig_50257_plus2 | 31 | leucine-rich repeat-containing g-protein coupled receptor 5 | 209 | 4.11E-17 | 46.85% | 6 | P:inner ear development; F:protein-hormone receptor activity; C:integral to membrane; P:G-protein coupled receptor signaling pathway; F:G-protein coupled receptor activity; C:integral to plasma membrane | IPR003591 (SMART); G3DSA:3.80.10.10 (GENE3D), PTHR24365 (PANTHER), PTHR24365:SF55 (PANTHER), PF13855 (PFAM), SSF52058 (SUPERFAMILY) |
| 2614 | lib_dib_Filter15_IDBA_contig_50265_plus1 | 21 | arylsulfatase j | 239 | 2.87E-66 | 66.40% | 2 | P:metabolic process; F:hydrolase activity | IPR000917 (PFAM); IPR017849 (G3DSA:3.40.720.GENE3D); IPR017850 (SUPERFAMILY); G3DSA:3.30.1120.10 (GENE3D), PTHR10342 (PANTHER), PTHR10342:SF68 (PANTHER) |
| 2615 | lib_dib_Filter15_IDBA_contig_50281_plus2 | 17 | zinc finger protein 271-like | 208 | 1.20E-47 | 63.45% | 3 | F:binding; F:nucleic acid binding; C:intracellular | IPR007087 (PROSITE); IPR013087 (G3DSA:3.30.160.GENE3D); IPR015880 (SMART); PTHR24377 (PANTHER), PTHR24377:SF0 (PANTHER), PF13465 (PFAM), SSF57667 (SUPERFAMILY) |
| 2616 | lib_dib_Filter15_IDBA_contig_50282_minus1 | 259 | kelch-like 11-like | 549 | 3.28E-15 | 46.95% | 1 | F:protein binding | IPR011333 (G3DSA:3.30.710.GENE3D); IPR011705 (PFAM); IPR013069 (PFAM); PTHR24412 (PANTHER) |
| 2617 | lib_dib_Filter15_IDBA_contig_50283_minus1 | 153 | kelch-like protein 11 | 340 | 3.03E-13 | 45.60% | 1 | F:protein binding | IPR011333 (G3DSA:3.30.710.GENE3D); IPR011705 (PFAM); IPR013069 (PFAM); PTHR24412 (PANTHER) |
| 2618 | lib_dib_Filter15_IDBA_contig_50331_minus1 | 14 | neurogenic locus notch | 209 | 3.72E-62 | 67.25% | 3 | P:biological_process; F:protein binding; F:calcium ion binding | IPR000152 (PROSITE); IPR000742 (PFAM); IPR001881 (SMART); IPR013032 (PROSITE); IPR018097 (PROSITE); PR00010 (PRINTS), G3DSA:2.10.25.10 (GENE3D), PTHR24048 (PANTHER), PTHR24048:SF15 (PANTHER), SSF57196 (SUPERFAMILY) |
| 2619 | lib_dib_Filter15_IDBA_contig_50358_plus1 | 20 | n-acetyl galactosaminyl transferase 6 | 208 | 8.37E-74 | 66.05% | 2 | C:cell; F:transferase activity | IPR001173 (PFAM); G3DSA:3.90.550.10 (GENE3D), PTHR11675 (PANTHER), SignalP-NN(euk) (SIGNALP), tmhmm (TMHMM), SSF53448 (SUPERFAMILY) |
| 2620 | lib_dib_Filter15_IDBA_contig_50394_minus2 | 52 | ephrin type-a receptor 4-a-like | 410 | 9.76E-77 | 52.50% | 2 | F:kinase activity; F:protein binding | IPR003961 (PFAM); IPR009030 (SUPERFAMILY); IPR011641 (PFAM); IPR013783 (G3DSA:2.60.40.GENE3D); PTHR24416 (PANTHER), PTHR24416:SF16 (PANTHER), tmhmm (TMHMM) |
| 2621 | lib_dib_Filter15_IDBA_contig_50407_plus1 | 66 | egf-like domain-containing protein | 327 | 2.10E-90 | 61.00% | 6 | F:protein binding; F:receptor activity; P:response to external stimulus; P:response to stress; C:cell; F:calcium ion binding | IPR000152 (PROSITE); IPR000742 (SMART); IPR001491 (PRINTS); IPR001881 (PFAM); IPR013032 (PROSITE); IPR018097 (PROSITE); PD968187 (PRODOM), G3DSA:2.10.25.10 (GENE3D), PTHR24034 (PANTHER), PTHR24034:SF0 (PANTHER), SSF57196 (SUPERFAMILY) |
| 2622 | lib_dib_Filter15_IDBA_contig_50408_plus1 | 24 | egf-like domain-containing protein | 124 | 3.26E-32 | 63.00% | 2 | F:protein binding; F:calcium ion binding | IPR000152 (PROSITE); IPR000742 (SMART); IPR001881 (PFAM); IPR013032 (PROSITE); IPR018097 (PROSITE); IPR026823 (PFAM); G3DSA:2.10.25.10 (GENE3D), PTHR24048 (PANTHER), SSF57196 (SUPERFAMILY) |
| 2623 | lib_dib_Filter15_IDBA_contig_50409_plus3 | 49 | egf-like domain-containing protein | 174 | 7.26E-50 | 63.40% | 2 | F:protein binding; F:calcium ion binding | IPR000152 (PROSITE); IPR000742 (SMART); IPR001881 (PFAM); IPR013032 (PROSITE); IPR018097 (PROSITE); G3DSA:2.10.25.10 (GENE3D), PTHR24034 (PANTHER), SSF57196 (SUPERFAMILY) |

| | | | | | | | | | |
|---|---|---|---|---|---|---|---|---|---|
| 2624 | lib_dib_Filter15_IDBA_contig_50422_minus3 | 27 | macrophage migration inhibitory factor | 208 | 7.24E-05 | 49.00% | 0 | - | IPR001398 (PANTHER); IPR014347 (SUPERFAMILY); G3DSA:3.30.429.10 (GENE3D) | |
| 2625 | lib_dib_Filter15_IDBA_contig_50431_minus2 | 26 | peroxidasin homolog | 208 | 3.90E-28 | 46.75% | 4 | P:biological_process; C:extracellular region; F:catalytic activity; F:protein binding | IPR003598 (SMART); IPR003599 (SMART); IPR007110 (PROFILE); IPR013098 (PFAM); IPR013783 (G3DSA:2.60.40.GENE3D); PTHR10489 (PANTHER), PF13895 (PFAM), SSF48726 (SUPERFAMILY) | |
| 2626 | lib_dib_Filter15_IDBA_contig_5054_minus1 | 343 | ---NA--- | 1262 | | | 0 | - | SignalP-NN(euk) (SIGNALP), tmhmm (TMHMM) | |
| 2627 | lib_dib_Filter15_IDBA_contig_5054_plus1 | 343 | fibrillin-1- partial | 1262 | 1.46E-43 | 48.95% | 2 | F:protein binding; F:calcium ion binding | IPR000152 (PROSITE); IPR000742 (SMART); IPR001881 (SMART); IPR008160 (PFAM); IPR013032 (PROSITE); IPR018097 (PROSITE); G3DSA:2.10.25.10 (GENE3D), PTHR24023 (PANTHER), tmhmm (TMHMM), SSF57196 (SUPERFAMILY) | |
| 2628 | lib_dib_Filter15_IDBA_contig_50552_minus1 | 37 | dbh-like monooxygenase protein 1 | 208 | 7.50E-39 | 57.10% | 6 | F:catalytic activity; ; P:metabolic process; P:carbohydrate metabolic process; P:catabolic process; F:carbohydrate binding | IPR000945 (PANTHER); IPR005018 (PFAM); IPR008960 (SUPERFAMILY); IPR015920 (G3DSA:2.60.40.GENE3D); PTHR10157:SF17 (PANTHER), SignalP-NN(euk) (SIGNALP) | yes |
| 2629 | lib_dib_Filter15_IDBA_contig_50615_minus1 | 30 | low-density lipoprotein receptor-related protein 4 | 208 | 9.65E-22 | 57.75% | 7 | C:organelle; C:intracellular; P:multicellular organismal development; C:cell; P:biological_process; C:cytoplasm; F:protein binding | IPR002172 (G3DSA:4.10.400.GENE3D); IPR023415 (PROSITE); PR00261 (PRINTS), G3DSA:4.10.1220.10 (GENE3D), PTHR10529 (PANTHER), PTHR10529:SF113 (PANTHER), SignalP-NN(euk) (SIGNALP) | yes |
| 2630 | lib_dib_Filter15_IDBA_contig_50693_plus3 | 75 | 15-hydroxyprostaglandin dehydrogenase | 371 | 4.17E-41 | 55.95% | 3 | P:metabolic process; F:catalytic activity; F:nucleotide binding | IPR002198 (PRINTS); IPR002347 (PRINTS); IPR016040 (G3DSA:3.40.50.GENE3D); PTHR24310 (PANTHER), PTHR24310:SF0 (PANTHER), SSF51735 (SUPERFAMILY) | |
| 2631 | lib_dib_Filter15_IDBA_contig_50814_minus2 | 27 | stonustoxin subunit alpha | 207 | 1.43E-41 | 55.35% | 3 | F:molecular_function; P:biological_process; C:cellular_component | no IPS match | |
| 2632 | lib_dib_Filter15_IDBA_contig_50829_plus2 | 46 | fibrinogen c domain-containing protein 1-like | 221 | 2.08E-37 | 57.60% | 3 | F:receptor binding; P:signal transduction; C:extracellular space | IPR002181 (PFAM); IPR014715 (G3DSA:4.10.530.GENE3D); IPR014716 (G3DSA:3.90.215.GENE3D); IPR020837 (PROSITE); PTHR19143 (PANTHER) | |
| 2633 | lib_dib_Filter15_IDBA_contig_50866_plus3 | 17 | uv radiation resistance-associated gene protein | 206 | 1.89E-46 | 70.50% | 8 | C:endosome; C:protein complex; C:lysosome; F:protein binding; P:regulation of biological process; P:catabolic process; P:response to stress; P:DNA metabolic process | IPR018791 (PFAM); PTHR15157 (PANTHER), PTHR15157:SF5 (PANTHER) | |
| 2634 | lib_dib_Filter15_IDBA_contig_50867_plus2 | 20 | carbonic anhydrase-related protein 10-like | 206 | 2.09E-39 | 66.95% | 3 | F:catalytic activity; C:extracellular region; F:binding | IPR001148 (G3DSA:3.10.200.GENE3D); IPR018347 (PTHR18952:PANTHER); IPR023561 (PANTHER) | |
| 2635 | lib_dib_Filter15_IDBA_contig_50869_minus1 | 23 | | 207 | 0 | 100% | 1 | F:protein binding | IPR003598 (SMART); IPR003599 (SMART); IPR007110 (PROFILE); IPR013098 (PFAM); IPR013783 (G3DSA:2.60.40.GENE3D); PTHR10489 (PANTHER), PF13895 (PFAM), SSF48726 (SUPERFAMILY) | |
| 2636 | lib_dib_Filter15_IDBA_contig_50883_minus2 | 32 | isoform c | 206 | 1.56E-83 | 74.85% | 4 | F:binding; F:protein kinase activity; F:signal transducer activity; P:signal transduction | PTHR26391 (PANTHER), PTHR26391:SF101 (PANTHER) | |
| 2637 | lib_dib_Filter15_IDBA_contig_50947_plus1 | 29 | enterokinase light chain | 206 | 1.77E-36 | 59.65% | 3 | F:peptidase activity; P:protein metabolic process; P:catabolic process | IPR001254 (PFAM); IPR001314 (PRINTS); IPR009003 (SUPERFAMILY); IPR018114 (PROSITE); G3DSA:2.40.10.10 (GENE3D), PTHR24256 (PANTHER) | |

| | | | | | | | | | | |
|---|---|---|---|---|---|---|---|---|---|---|
| 2638 | lib_dib_Filter15_IDBA_contig_50970_minus1 | 40 | iglon family member 5 | 206 | 5.95E-05 | 48.20% | 1 | F:protein binding | IPR003599 (SMART); IPR007110 (PROFILE); IPR013783 (G3DSA:2.60.40.GENE3D); PTHR10489 (PANTHER), PF13895 (PFAM), tmhmm (TMHMM), SSF48726 (SUPERFAMILY) | |
| 2639 | lib_dib_Filter15_IDBA_contig_51005_plus2 | 32 | syntaxin 1a | 206 | 7.36E-142 | 92.35% | 5 | C:cell; F:protein binding; C:intracellular; P:transport; P:protein transport | IPR000727 (PFAM); IPR006011 (PFAM); IPR006012 (PROSITE); IPR010989 (SUPERFAMILY); G3DSA:1.20.58.70 (GENE3D), PTHR19957 (PANTHER), PTHR19957:SF33 (PANTHER) | |
| 2640 | lib_dib_Filter15_IDBA_contig_51092_minus2 | 20 | chaperone activity of bc1 complex- mitochondrial-like | 205 | 1.26E-84 | 82.30% | 2 | C:mitochondrion; F:transferase activity | IPR004147 (PFAM); PTHR10566 (PANTHER), PTHR10566:SF22 (PANTHER) | |
| 2641 | lib_dib_Filter15_IDBA_contig_51231_minus1 | 2035 | thioredoxin peroxidase | 193 | 3.59E-105 | 89.85% | 3 | P:metabolic process; F:antioxidant activity; F:catalytic activity | IPR000866 (PFAM); IPR012336 (G3DSA:3.40.30.GENE3D); IPR019479 (PFAM); IPR024706 (PIR); PTHR10681 (PANTHER), PTHR10681:SF44 (PANTHER) | |
| 2642 | lib_dib_Filter15_IDBA_contig_51298_minus3 | 24 | low quality protein: cubilin-like | 204 | 4.64E-15 | 50.60% | 5 | F:calcium ion binding; P:proteolysis; F:serine-type endopeptidase activity; F:catalytic activity; F:carbohydrate binding | IPR000859 (G3DSA:2.60.120.GENE3D); PTHR10127 (PANTHER) | |
| 2643 | lib_dib_Filter15_IDBA_contig_51386_minus3 | 27 | glycoside hydrolase family 3 domain protein | 204 | 3.92E-69 | 71.00% | 2 | P:carbohydrate metabolic process; F:hydrolase activity | IPR001764 (G3DSA:3.20.20.GENE3D); IPR017853 (SUPERFAMILY); IPR026892 (PANTHER); PTHR30620:SF0 (PANTHER), SignalP-NN(euk) (SIGNALP), tmhmm (TMHMM) | yes |
| 2644 | lib_dib_Filter15_IDBA_contig_51482_minus1 | 46 | ---NA--- | 204 | | | 0 | - | no IPS match | |
| 2645 | lib_dib_Filter15_IDBA_contig_51482_plus1 | 46 | ---NA--- | 204 | | | 0 | - | PTHR23304 (PANTHER) | |
| 2646 | lib_dib_Filter15_IDBA_contig_51558_minus1 | 30 | l-xylulose reductase | 204 | 5.50E-43 | 62.20% | 3 | P:metabolic process; F:catalytic activity; F:nucleotide binding | IPR002198 (PRINTS); IPR002347 (PRINTS); IPR016040 (G3DSA:3.40.50.GENE3D); IPR020904 (PROSITE); PTHR24311 (PANTHER), PTHR24311:SF0 (PANTHER), PF13561 (PFAM), SSF51735 (SUPERFAMILY) | |
| 2647 | lib_dib_Filter15_IDBA_contig_51581_plus3 | 17 | protein creg2-like | 203 | 5.39E-39 | 58.15% | 3 | F:nucleotide binding; F:catalytic activity; P:metabolic process | IPR012349 (G3DSA:2.30.110.GENE3D); PTHR13343 (PANTHER), PTHR13343:SF4 (PANTHER), PF13883 (PFAM) | |
| 2648 | lib_dib_Filter15_IDBA_contig_51582_plus2 | 73 | 72 kda type iv collagenase | 372 | 1.85E-26 | 45.25% | 10 | F:peptidase activity; P:biological_process; C:intracellular; P:anatomical structure morphogenesis; P:multicellular organismal development; P:response to endogenous stimulus; P:response to stress; C:extracellular region; P:reproduction; P:metabolic process | IPR000562 (G3DSA:2.10.10.GENE3D); IPR013806 (SUPERFAMILY); PTHR22918 (PANTHER), PTHR22918:SF0 (PANTHER) | |
| 2649 | lib_dib_Filter15_IDBA_contig_51583_plus2 | 35 | 72 kda type iv collagenase | 247 | 1.78E-23 | 45.00% | 10 | F:peptidase activity; P:biological_process; C:intracellular; P:anatomical structure morphogenesis; P:multicellular organismal development; P:response to endogenous stimulus; P:response to stress; C:extracellular region; P:reproduction; P:metabolic process | IPR000562 (G3DSA:2.10.10.GENE3D); IPR013806 (SUPERFAMILY); PTHR22918 (PANTHER), PTHR22918:SF0 (PANTHER) | |
| 2650 | lib_dib_Filter15_IDBA_contig_51610_plus3 | 23 | zinc finger protein 675 | 203 | 2.69E-36 | 54.70% | 12 | P:regulation of biological process; ; P:signal transduction; P:response to stress; P:cell differentiation; P:multicellular organismal development; F:protein binding; P:cellular protein modification process; C:cytoplasm; C:nucleus; F:binding; F:nucleic acid binding | IPR007087 (PFAM); IPR013087 (G3DSA:3.30.160.GENE3D); IPR015880 (SMART); PTHR24375 (PANTHER), PTHR24375:SF0 (PANTHER), PF13465 (PFAM), PF13894 (PFAM), PF13913 (PFAM), SSF57667 (SUPERFAMILY) | |

| | | | | | | | | | |
|---|---|---|---|---|---|---|---|---|---|
| 2651 | lib_dib_Filter15_IDBA_contig_51617_minus1 | 427 | frizzled-2 precursor | 497 | 2.94E-64 | 60.85% | 12 | P:anatomical structure morphogenesis; P:cellular component organization; P:multicellular organismal development; P:embryo development; C:plasma membrane; C:cell; F:receptor activity; F:protein binding; P:signal transduction; P:cell differentiation; P:regulation of biological process; | IPR015526 (PANTHER); IPR020067 (G3DSA:1.10.2000.GENE3D); SignalP-NN(euk) (SIGNALP) |
| 2652 | lib_dib_Filter15_IDBA_contig_51618_minus1 | 424 | frizzled-2 precursor | 497 | 2.75E-64 | 60.85% | 12 | P:anatomical structure morphogenesis; P:cellular component organization; P:multicellular organismal development; P:embryo development; C:plasma membrane; C:cell; F:receptor activity; F:protein binding; P:signal transduction; P:cell differentiation; P:regulation of biological process; | IPR015526 (PANTHER); IPR020067 (G3DSA:1.10.2000.GENE3D); SignalP-NN(euk) (SIGNALP) |
| 2653 | lib_dib_Filter15_IDBA_contig_51702_plus2 | 35 | von willebrand factor d and egf domain-containing protein | 203 | 4.08E-24 | 45.70% | 1 | F:protein binding | IPR000742 (PROFILE); IPR013032 (PROSITE); IPR013111 (PFAM); G3DSA:2.10.25.10 (GENE3D), PTHR11219 (PANTHER) |
| 2654 | lib_dib_Filter15_IDBA_contig_51715_plus1 | 36 | upf0670 protein cg4666-like | 204 | 2.09E-43 | 59.80% | 1 | C:extracellular region | G3DSA:3.10.129.10 (GENE3D), PTHR12475 (PANTHER), PF13279 (PFAM), SignalP-NN(euk) (SIGNALP), tmhmm (TMHMM), SSF54637 (SUPERFAMILY) |
| 2655 | lib_dib_Filter15_IDBA_contig_51737_minus3 | 33 | deoxyribonuclease-1-like 2 | 214 | 1.67E-30 | 64.25% | 3 | F:nuclease activity; P:DNA metabolic process; P:catabolic process | IPR005135 (SUPERFAMILY); IPR008185 (PRINTS); IPR016202 (PANTHER); G3DSA:3.60.10.10 (GENE3D), tmhmm (TMHMM) |
| 2656 | lib_dib_Filter15_IDBA_contig_51816_plus2 | 406 | thyroglobulin precursor | 282 | 1.52E-10 | 49.90% | 17 | P:regulation of myelination; P:thyroid hormone metabolic process; P:iodide transport; C:extracellular space; P:thyroid gland development; P:cell-matrix adhesion; F:calcium ion binding; C:cell surface; F:neurexin family protein binding; C:synapse; F:carboxylesterase activity; F:hormone activity; F:receptor activity; C:integral to plasma membrane; P:hormone biosynthetic process; P:thyroid hormone generation; P:synapse assembly | IPR000716 (G3DSA:4.10.800.GENE3D); PTHR12352 (PANTHER), SignalP-NN(euk) (SIGNALP), tmhmm (TMHMM) |
| 2657 | lib_dib_Filter15_IDBA_contig_51859_plus2 | 22 | activated cdc42 kinase 1 | 203 | 1.88E-124 | 85.20% | 15 | F:protein binding; F:enzyme regulator activity; F:receptor binding; P:signal transduction; C:protein complex; C:plasma membrane; C:cytoplasmic membrane-bounded vesicle; F:protein kinase activity; C:cell; F:binding; P:regulation of biological process; P:cellular protein modification process; C:endosome; C:nucleus; F:nucleotide binding | IPR000719 (PROFILE); IPR001245 (PRINTS); IPR002290 (SMART); IPR008266 (PROSITE); IPR011009 (SUPERFAMILY); IPR020635 (SMART); G3DSA:1.10.510.10 (GENE3D), G3DSA:3.30.200.20 (GENE3D), PTHR24418 (PANTHER), PTHR24418:SF66 (PANTHER) |
| 2658 | lib_dib_Filter15_IDBA_contig_51936_plus3 | 1180 | angiotensin-converting enzyme-like isoform 1 | 750 | 7.40E-176 | 63.30% | 17 | P:regulation of biological process; P:protein metabolic process; P:catabolic process; F:peptidase activity; F:binding; P:multicellular organismal development; C:cell; F:receptor binding; P:transport; C:extracellular space; P:biological_process; F:actin binding; C:plasma membrane; C:endosome; P:cell differentiation; P:anatomical structure morphogenesis; P:cell proliferation | IPR001548 (PRINTS); SignalP-NN(euk) (SIGNALP), SSF55486 (SUPERFAMILY) |

| | | | | | | | | | | |
|---|---|---|---|---|---|---|---|---|---|---|
| 2659 | lib_dib_Filter15_IDBA_contig_51937_plus2 | 1187 | angiotensin-converting enzyme-like isoform 1 | 733 | 5.06E-176 | 63.35% | 17 | P:regulation of biological process; P:protein metabolic process; P:catabolic process; F:peptidase activity; F:binding; P:multicellular organismal development; C:cell; F:receptor binding; P:transport; C:extracellular space; P:biological_process; F:actin binding; C:plasma membrane; C:endosome; P:cell differentiation; P:anatomical structure morphogenesis; P:cell proliferation | IPR001548 (PRINTS); SignalP-NN(euk) (SIGNALP), tmhmm (TMHMM), SSF55486 (SUPERFAMILY) | yes |
| 2660 | lib_dib_Filter15_IDBA_contig_51938_plus1 | 73 | angiotensin-converting enzyme-like | 115 | 5.19E-41 | 73.20% | 7 | P:biological_process; P:catabolic process; F:binding; F:peptidase activity; C:cell; F:protein binding; P:protein metabolic process | IPR001548 (PRINTS); SSF55486 (SUPERFAMILY) | |
| 2661 | lib_dib_Filter15_IDBA_contig_51954_plus3 | 42 | dipeptidyl peptidase 2-like | 202 | 1.28E-19 | 78.00% | 3 | F:peptidase activity; P:protein metabolic process; P:catabolic process | IPR008758 (PANTHER); PTHR11010:SF10 (PANTHER) | |
| 2662 | lib_dib_Filter15_IDBA_contig_52085_plus3 | 35 | matrix metalloproteinase | 202 | 3.44E-46 | 59.85% | 2 | F:binding; F:peptidase activity | IPR000585 (G3DSA:2.110.10.GENE3D); IPR018487 (PFAM); PTHR10201 (PANTHER), tmhmm (TMHMM) | |
| 2663 | lib_dib_Filter15_IDBA_contig_52118_plus1 | 25 | cg4553 cg4553-pa | 202 | 4.29E-39 | 58.65% | 0 | - | IPR010487 (PFAM); PTHR13475 (PANTHER) | |
| 2664 | lib_dib_Filter15_IDBA_contig_52120_minus1 | 20 | ankyrin repeat domain protein | 202 | 4.78E-25 | 53.65% | 1 | F:protein binding | IPR002110 (PRINTS); IPR020683 (G3DSA:1.25.40.GENE3D); PTHR24188 (PANTHER) | |
| 2665 | lib_dib_Filter15_IDBA_contig_52141_plus3 | 35 | deleted in malignant brain tumors 1 | 202 | 2.04E-33 | 57.25% | 2 | F:receptor activity; C:cell | IPR001190 (PRINTS); IPR017448 (SMART); G3DSA:3.10.250.10 (GENE3D), PTHR19331 (PANTHER), PTHR19331:SF118 (PANTHER), SignalP-NN(euk) (SIGNALP), tmhmm (TMHMM) | yes |
| 2666 | lib_dib_Filter15_IDBA_contig_52211_plus3 | 152 | uncharacterized family 31 glucosidase kiaa1161-like | 476 | 8.44E-179 | 64.95% | 2 | P:carbohydrate metabolic process; F:catalytic activity | IPR000322 (PANTHER); IPR013785 (G3DSA:3.20.20.GENE3D); IPR017853 (SUPERFAMILY); PTHR22762:SF2 (PANTHER) | |
| 2667 | lib_dib_Filter15_IDBA_contig_52215_plus1 | 16 | zinc finger protein 135-like | 202 | 2.16E-47 | 67.55% | 3 | F:nucleic acid binding; F:binding; C:intracellular | IPR007087 (PROSITE); IPR013087 (G3DSA:3.30.160.GENE3D); IPR015880 (SMART); PTHR24402 (PANTHER), PF13465 (PFAM), PF13912 (PFAM), SSF57667 (SUPERFAMILY) | |
| 2668 | lib_dib_Filter15_IDBA_contig_52247_plus1 | 17444 | glioma pathogenesis-related protein 1-like | 335 | 3.63E-18 | 48.10% | 1 | F:protein binding | IPR001283 (PRINTS); IPR002172 (G3DSA:4.10.400.GENE3D); IPR002413 (PRINTS); IPR014044 (G3DSA:3.40.33.GENE3D); SignalP-NN(euk) (SIGNALP), tmhmm (TMHMM) | yes |
| 2669 | lib_dib_Filter15_IDBA_contig_52248_plus3 | 16869 | glioma pathogenesis-related protein 1-like | 326 | 3.02E-18 | 48.30% | 1 | F:protein binding | IPR001283 (PRINTS); IPR002172 (G3DSA:4.10.400.GENE3D); IPR002413 (PRINTS); IPR014044 (G3DSA:3.40.33.GENE3D); SignalP-NN(euk) (SIGNALP) | yes |
| 2670 | lib_dib_Filter15_IDBA_contig_52249_plus3 | 10148 | glioma pathogenesis-related protein 1-like | 213 | 2.00E-13 | 49.55% | 5 | C:extracellular region; P:small molecule metabolic process; P:cellular lipid metabolic process; C:integral to membrane; C:plasma membrane | IPR001283 (PRINTS); IPR002413 (PRINTS); IPR014044 (G3DSA:3.40.33.GENE3D) | |
| 2671 | lib_dib_Filter15_IDBA_contig_52285_minus1 | 22 | upf0565 protein c2orf69 homolog | 202 | 2.50E-51 | 54.00% | 4 | F:molecular_function; P:biological_process; C:extracellular region; C:cellular_component | IPR018881 (PFAM); PTHR31296 (PANTHER), PTHR31296:SF0 (PANTHER) | |
| 2672 | lib_dib_Filter15_IDBA_contig_52300_plus2 | 3836 | dipeptidyl peptidase 1-like | 494 | 0 | 69.50% | 3 | P:protein metabolic process; P:catabolic process; F:peptidase activity | IPR000169 (PROSITE); IPR000668 (PRINTS); IPR013128 (PANTHER); IPR014882 (PFAM); IPR025660 (PROSITE); IPR025661 (PROSITE); G3DSA:2.40.128.80 (GENE3D), G3DSA:3.90.70.10 (GENE3D), PTHR12411:SF17 (PANTHER), SignalP-NN(euk) (SIGNALP), tmhmm (TMHMM), SSF54001 (SUPERFAMILY), SSF75001 (SUPERFAMILY) | yes |

| | | | | | | | | | | |
|---|---|---|---|---|---|---|---|---|---|---|
| 2673 | lib_dib_Filter15_IDBA_contig_52301_plus1 | 348 | dipeptidyl peptidase 1 | 128 | 3.64E-57 | 80.40% | 11 | C:lysosome; F:peptidase activity; F:protein binding; F:binding; P:protein metabolic process; P:catabolic process; P:regulation of biological process; P:cell death; P:biological_process; C:endoplasmic reticulum; C:Golgi apparatus | IPR000668 (PFAM); IPR013128 (PANTHER); IPR025661 (PROSITE); G3DSA:3.90.70.10 (GENE3D), PTHR12411:SF17 (PANTHER), SSF54001 (SUPERFAMILY) | |
| 2674 | lib_dib_Filter15_IDBA_contig_52371_plus1 | 30 | cathepsin l-like | 201 | 1.84E-36 | 60.00% | 3 | P:protein metabolic process; P:catabolic process; F:peptidase activity | IPR000169 (PROSITE); IPR000668 (PFAM); IPR013128 (PANTHER); IPR013201 (PFAM); G3DSA:3.90.70.10 (GENE3D), SignalP-NN(euk) (SIGNALP), tmhmm (TMHMM), SSF54001 (SUPERFAMILY) | |
| 2675 | lib_dib_Filter15_IDBA_contig_52373_minus2 | 878 | fibrinogen c domain-containing protein 1-like | 593 | 3.35E-49 | 60.05% | 3 | F:receptor binding; C:extracellular space; P:signal transduction | IPR002181 (PFAM); IPR014715 (G3DSA:4.10.530.GENE3D); IPR014716 (G3DSA:3.90.215.GENE3D); PTHR19143 (PANTHER) | |
| 2676 | lib_dib_Filter15_IDBA_contig_52374_minus2 | 789 | fibrinogen c domain-containing protein 1-like | 456 | 1.18E-49 | 59.55% | 3 | F:receptor binding; C:extracellular space; P:signal transduction | IPR002181 (PFAM); IPR014715 (G3DSA:4.10.530.GENE3D); IPR014716 (G3DSA:3.90.215.GENE3D); PTHR19143 (PANTHER) | |
| 2677 | lib_dib_Filter15_IDBA_contig_52402_minus1 | 35 | serine threonine-protein kinase 17a | 201 | 1.82E-86 | 76.65% | 6 | P:cellular protein modification process; P:regulation of biological process; P:cell death; P:signal transduction; F:protein kinase activity; F:nucleotide binding | IPR000719 (PFAM); IPR002290 (SMART); IPR008271 (PROSITE); IPR011009 (SUPERFAMILY); IPR020635 (SMART); G3DSA:1.10.510.10 (GENE3D), G3DSA:3.30.200.20 (GENE3D), PTHR24342 (PANTHER), PTHR24342:SF6 (PANTHER) | |
| 2678 | lib_dib_Filter15_IDBA_contig_52483_plus2 | 39 | von willebrand factor a domain containing 8 | 274 | 3.79E-128 | 80.55% | 6 | C:mitochondrion; P:nucleobase-containing compound metabolic process; P:catabolic process; F:nucleotide binding; F:hydrolase activity; C:extracellular region | IPR011704 (PFAM); G3DSA:3.40.50.300 (GENE3D), PTHR21610 (PANTHER), PTHR21610:SF2 (PANTHER), SSF52540 (SUPERFAMILY) | |
| 2679 | lib_dib_Filter15_IDBA_contig_52520_minus1 | 216 | polehole | 418 | 7.29E-42 | 52.80% | 6 | P:cell death; P:signal transduction; P:regulation of biological process; F:nucleotide binding; P:cellular protein modification process; F:protein kinase activity | IPR000719 (PROFILE); IPR001245 (PRINTS); IPR002290 (SMART); IPR008271 (PROSITE); IPR011009 (SUPERFAMILY); IPR017441 (PROSITE); IPR020635 (SMART); G3DSA:1.10.510.10 (GENE3D), G3DSA:3.30.200.20 (GENE3D), PTHR23257 (PANTHER), SignalP-NN(euk) (SIGNALP) | yes |
| 2680 | lib_dib_Filter15_IDBA_contig_52521_minus1 | 343 | polehole | 712 | 5.54E-40 | 53.20% | 5 | F:protein binding; P:signal transduction; F:nucleotide binding; P:cellular protein modification process; F:protein kinase activity | IPR000488 (PFAM); IPR000719 (PROFILE); IPR001245 (PRINTS); IPR002290 (SMART); IPR008271 (PROSITE); IPR011009 (SUPERFAMILY); IPR011029 (G3DSA:1.10.533.GENE3D); IPR017441 (PROSITE); IPR020635 (SMART); G3DSA:1.10.510.10 (GENE3D), G3DSA:3.30.200.20 (GENE3D), PTHR23257 (PANTHER), SignalP-NN(euk) (SIGNALP) | yes |
| 2681 | lib_dib_Filter15_IDBA_contig_52522_minus1 | 310 | polehole | 699 | 5.99E-40 | 53.15% | 5 | F:protein binding; P:signal transduction; F:nucleotide binding; P:cellular protein modification process; F:protein kinase activity | IPR000488 (PFAM); IPR000719 (PROFILE); IPR001245 (PRINTS); IPR002290 (SMART); IPR008271 (PROSITE); IPR011009 (SUPERFAMILY); IPR011029 (G3DSA:1.10.533.GENE3D); IPR017441 (PROSITE); IPR020635 (SMART); G3DSA:1.10.510.10 (GENE3D), G3DSA:3.30.200.20 (GENE3D), PTHR23257 (PANTHER), SignalP-NN(euk) (SIGNALP) | yes |

| | | | | | | | | | | |
|---|---|---|---|---|---|---|---|---|---|---|
| 2682 | lib_dib_Filter15_IDBA_contig_52543_plus3 | 56 | protocadherin fat 1 | 200 | 1.94E-30 | 64.40% | 4 | P:biological_process; C:cell; F:protein binding; F:calcium ion binding | IPR000152 (PROSITE); IPR000742 (SMART); IPR001881 (PFAM); IPR013032 (PROSITE); IPR018097 (PROSITE); PR00010 (PRINTS), G3DSA:2.10.25.10 (GENE3D), PTHR24668 (PANTHER), PTHR24668:SF24 (PANTHER), tmhmm (TMHMM), SSF57196 (SUPERFAMILY) | |
| 2683 | lib_dib_Filter15_IDBA_contig_52651_minus3 | 35 | iron zinc purple acid phosphatase-like protein precursor | 257 | 5.04E-83 | 74.60% | 2 | P:metabolic process; F:hydrolase activity | IPR004843 (PFAM); IPR025733 (PFAM); G3DSA:3.60.21.10 (GENE3D), PTHR22953 (PANTHER), PTHR22953:SF0 (PANTHER), SSF56300 (SUPERFAMILY) | |
| 2684 | lib_dib_Filter15_IDBA_contig_52655_plus1 | 154 | fibrinogen c domain-containing protein 1-like | 333 | 1.38E-26 | 64.15% | 3 | F:receptor binding; C:extracellular space; P:signal transduction | IPR002181 (PFAM); IPR014715 (G3DSA:4.10.530.GENE3D); IPR014716 (G3DSA:3.90.215.GENE3D); IPR020837 (PROSITE); PTHR19143 (PANTHER) | |
| 2685 | lib_dib_Filter15_IDBA_contig_52723_plus3 | 19 | collagen alpha-1 chain-like | 200 | 2.60E-07 | 58.60% | 5 | F:extracellular matrix structural constituent; C:basement membrane; C:collagen; C:proteinaceous extracellular matrix; C:extracellular region | IPR008160 (PFAM); PTHR24023 (PANTHER) | |
| 2686 | lib_dib_Filter15_IDBA_contig_52816_minus1 | 42 | integrin-linked protein kinase-like | 228 | 5.30E-124 | 77.20% | 6 | F:protein kinase activity; P:cellular protein modification process; F:hydrolase activity; F:nucleotide binding; P:signal transduction; F:protein binding | IPR001245 (PFAM); IPR002110 (SMART); IPR011009 (SUPERFAMILY); IPR020683 (G3DSA:1.25.40.GENE3D); G3DSA:3.30.200.20 (GENE3D), PTHR23257 (PANTHER), PTHR23257:SF30 (PANTHER) | |
| 2687 | lib_dib_Filter15_IDBA_contig_52830_plus2 | 2377 | thioredoxin domain-containing protein 5-like | 1165 | 2.58E-109 | 62.40% | 5 | P:metabolic process; F:electron carrier activity; F:catalytic activity; P:cellular homeostasis; P:regulation of biological process | IPR005746 (PRINTS); IPR005788 (TIGRFAMs); IPR012336 (G3DSA:3.40.30.GENE3D); IPR013766 (PFAM); IPR017937 (PROSITE); PTHR18929 (PANTHER), PTHR18929:SF32 (PANTHER), SignalP-NN(euk) (SIGNALP), tmhmm (TMHMM) | |
| 2688 | lib_dib_Filter15_IDBA_contig_52839_minus1 | 622 | kelch-like protein 26 | 767 | 0 | 48.10% | 1 | F:protein binding | IPR000210 (SMART); IPR006652 (SMART); IPR011333 (G3DSA:3.30.710.GENE3D); IPR011705 (PFAM); IPR013069 (PFAM); IPR015915 (G3DSA:2.120.10.GENE3D); PTHR24412 (PANTHER), PF13964 (PFAM), SSF117281 (SUPERFAMILY) | |
| 2689 | lib_dib_Filter15_IDBA_contig_52873_minus3 | 878 | ---NA--- | 811 | | | 0 | - | tmhmm (TMHMM) | |
| 2690 | lib_dib_Filter15_IDBA_contig_52873_plus3 | 878 | collagen alpha-5 chain | 811 | 1.07E-11 | 59.67% | 1 | C:collagen | IPR008160 (PFAM); PTHR24023 (PANTHER), PTHR24023:SF230 (PANTHER), tmhmm (TMHMM) | |
| 2691 | lib_dib_Filter15_IDBA_contig_52917_minus3 | 41 | core histone macro- -like | 199 | 3.94E-63 | 85.35% | 8 | P:organelle organization; C:chromosome; F:DNA binding; C:microtubule organizing center; C:nuclear chromosome; P:regulation of gene expression, epigenetic; F:chromatin binding; F:protein binding | IPR002119 (PRINTS); IPR007125 (PFAM); IPR009072 (G3DSA:1.10.20.GENE3D); PTHR23430 (PANTHER) | |
| 2692 | lib_dib_Filter15_IDBA_contig_52990_minus3 | 29 | fibrinogen and fibronectin | 199 | 3.84E-11 | 56.00% | 6 | P:biological_process; C:plasma membrane; F:protein binding; F:binding; C:extracellular space; C:cytoplasmic membrane-bounded vesicle | IPR002181 (PFAM); IPR014716 (G3DSA:3.90.215.GENE3D); PTHR19143 (PANTHER), PTHR19143:SF47 (PANTHER), tmhmm (TMHMM) | |
| 2693 | lib_dib_Filter15_IDBA_contig_53012_plus1 | 1986 | fibril-forming collagen alpha chain-like | 412 | 2.97E-37 | 47.33% | 0 | - | tmhmm (TMHMM) | |
| 2694 | lib_dib_Filter15_IDBA_contig_53017_minus2 | 885 | hypothetical protein IEC_02821, partial | 199 | 1.40E-04 | 52.00% | 0 | - | PTHR24023 (PANTHER), PTHR24023:SF167 (PANTHER), SignalP-NN(euk) (SIGNALP), tmhmm (TMHMM) | yes |
| 2695 | lib_dib_Filter15_IDBA_contig_53100_minus2 | 36 | zinc finger protein 236 | 199 | 5.99E-51 | 71.55% | 4 | P:biological_process; F:binding; F:nucleic acid binding; C:intracellular | IPR007087 (PFAM); IPR013087 (G3DSA:3.30.160.GENE3D); IPR015880 (SMART); PTHR24396 (PANTHER), PTHR24396:SF0 (PANTHER), PF13465 (PFAM), PF13912 (PFAM), SSF57667 (SUPERFAMILY) | |

| | | | | | | | | | | |
|---|---|---|---|---|---|---|---|---|---|---|
| 2696 | lib_dib_Filter15_IDBA_contig_53203_plus1 | 125 | endothelin-converting enzyme 1-like | 276 | 1.15E-70 | 63.55% | 15 | P:catabolic process; C:cell; C:cytoplasmic membrane-bounded vesicle; P:cell death; P:signal transduction; P:multicellular organismal development; P:response to stress; C:plasma membrane; P:embryo development; P:protein metabolic process; C:endosome; F:binding; P:metabolic process; P:regulation of biological process; F:peptidase activity | IPR000718 (PANTHER); IPR018497 (PRINTS); IPR024079 (G3DSA:3.40.390.GENE3D); SSF55486 (SUPERFAMILY) | |
| 2697 | lib_dib_Filter15_IDBA_contig_53204_plus2 | 37 | endothelin-converting enzyme 1 | 125 | 4.35E-21 | 62.70% | 15 | P:catabolic process; C:cell; C:cytoplasmic membrane-bounded vesicle; P:cell death; P:signal transduction; P:multicellular organismal development; P:response to stress; C:plasma membrane; P:embryo development; P:protein metabolic process; C:endosome; F:binding; P:metabolic process; P:regulation of biological process; F:peptidase activity | IPR000718 (PANTHER); IPR018497 (PFAM); IPR024079 (G3DSA:3.40.390.GENE3D); SSF55486 (SUPERFAMILY) | |
| 2698 | lib_dib_Filter15_IDBA_contig_53228_plus1 | 28 | variable lymphocyte receptor a | 199 | 1.36E-08 | 51.45% | 1 | F:protein binding | IPR001611 (PROFILE); G3DSA:3.80.10.10 (GENE3D), PTHR24369 (PANTHER), tmhmm (TMHMM), SSF52058 (SUPERFAMILY) | |
| 2699 | lib_dib_Filter15_IDBA_contig_53249_minus2 | 17 | angiopoietin-related protein 1-like | 198 | 4.22E-35 | 57.00% | 3 | F:receptor binding; C:extracellular space; P:signal transduction | IPR002181 (PFAM); IPR014715 (G3DSA:4.10.530.GENE3D); IPR014716 (G3DSA:3.90.215.GENE3D); PTHR19143 (PANTHER) | |
| 2700 | lib_dib_Filter15_IDBA_contig_53299_minus2 | 26 | zinc finger protein 271 (zinc finger protein 7) (zinc finger protein znfphex133) (epstein-barr virus-induced zinc finger protein) (znf-eb) (ct-zfp48) (zinc finger protein | 202 | 2.37E-09 | 58.45% | 3 | F:binding; F:nucleic acid binding; C:intracellular | IPR007087 (PROSITE); IPR013087 (G3DSA:3.30.160.GENE3D); IPR015880 (SMART); PTHR24402 (PANTHER), PTHR24402:SF54 (PANTHER), PF13465 (PFAM), SSF57667 (SUPERFAMILY) | |
| 2701 | lib_dib_Filter15_IDBA_contig_5331_minus2 | 269 | serine threonine-protein kinase prkx-like | 741 | 3.51E-155 | 73.45% | 7 | P:cellular protein modification process; P:anatomical structure morphogenesis; P:multicellular organismal development; F:protein kinase activity; C:protein complex; C:intracellular; F:nucleotide binding | IPR000719 (PFAM); IPR000961 (SMART); IPR002290 (SMART); IPR008271 (PROSITE); IPR011009 (SUPERFAMILY); IPR017441 (PROSITE); IPR020635 (SMART); G3DSA:1.10.510.10 (GENE3D), G3DSA:3.30.200.20 (GENE3D), PTHR24353 (PANTHER), PTHR24353:SF4 (PANTHER), tmhmm (TMHMM) | |
| 2702 | lib_dib_Filter15_IDBA_contig_53346_minus3 | 134 | trafficking protein particle complex subunit 1 | 421 | 2.88E-71 | 82.85% | 5 | P:transport; C:endoplasmic reticulum; C:Golgi apparatus; C:protein complex; F:protein binding | IPR007233 (PANTHER); IPR011012 (SUPERFAMILY); G3DSA:3.30.450.70 (GENE3D), PTHR23249:SF9 (PANTHER) | |
| 2703 | lib_dib_Filter15_IDBA_contig_53357_minus3 | 605 | neurotransmitter-gated ion-channel ligand binding domain protein | 349 | 3.01E-17 | 47.35% | 3 | P:ion transport; C:cell; F:ion channel activity | IPR006201 (PANTHER); IPR006202 (G3DSA:2.70.170.GENE3D); PTHR18945:SF92 (PANTHER), SignalP-NN(euk) (SIGNALP) | yes |
| 2704 | lib_dib_Filter15_IDBA_contig_53358_minus3 | 554 | protein acr- isoform b | 354 | 3.14E-17 | 47.50% | 3 | P:ion transport; C:cell; F:ion channel activity | IPR006201 (PANTHER); IPR006202 (G3DSA:2.70.170.GENE3D); PTHR18945:SF92 (PANTHER), SignalP-NN(euk) (SIGNALP) | yes |
| 2705 | lib_dib_Filter15_IDBA_contig_53359_minus1 | 158 | neurotransmitter-gated ion-channel ligand binding domain protein | 170 | 8.63E-18 | 50.00% | 3 | C:cell; P:ion transport; F:ion channel activity | IPR006201 (PANTHER); IPR006202 (G3DSA:2.70.170.GENE3D); PTHR18945:SF127 (PANTHER) | |
| 2706 | lib_dib_Filter15_IDBA_contig_53389_plus2 | 23 | protein disulfide-isomerase tmx3 | 198 | 5.29E-62 | 62.10% | 5 | P:metabolic process; F:electron carrier activity; F:catalytic activity; P:cellular homeostasis; P:regulation of biological process | IPR005746 (PRINTS); IPR012336 (G3DSA:3.40.30.GENE3D); IPR013766 (PFAM); IPR017937 (PROSITE); PTHR18929 (PANTHER) | |
| 2707 | lib_dib_Filter15_IDBA_contig_53407_plus1 | 322 | hypothetical protein CAPTEDRAFT_227535 | 379 | 9.32E-49 | 56.05% | 0 | - | PF13383 (PFAM), SignalP-NN(euk) (SIGNALP) | yes |

| | | | | | | | | | |
|---|---|---|---|---|---|---|---|---|---|
| 2708 | lib_dib_Filter15_IDBA_contig_53408_plus3 | 559 | hypothetical protein CAPTEDRAFT_206360 | 384 | 4.74E-50 | 54.65% | 0 | - | PF13383 (PFAM), tmhmm (TMHMM) | |
| 2709 | lib_dib_Filter15_IDBA_contig_53432_minus2 | 57 | zinc finger protein 891-like | 305 | 1.31E-38 | 57.25% | 3 | F:binding; F:nucleic acid binding; C:intracellular | IPR007087 (PROSITE); IPR013087 (G3DSA:3.30.160.GENE3D); IPR015880 (SMART); PTHR11389 (PANTHER), PF13465 (PFAM), SSF57667 (SUPERFAMILY) | |
| 2710 | lib_dib_Filter15_IDBA_contig_53433_minus2 | 61 | zinc finger protein 891-like | 310 | 1.23E-38 | 57.25% | 3 | F:binding; F:nucleic acid binding; C:intracellular | IPR007087 (PROSITE); IPR013087 (G3DSA:3.30.160.GENE3D); IPR015880 (SMART); PTHR11389 (PANTHER), PF13465 (PFAM), SSF57667 (SUPERFAMILY) | |
| 2711 | lib_dib_Filter15_IDBA_contig_53463_plus2 | 3914 | neuroplastin precursor | 587 | 4.98E-45 | 51.75% | 1 | F:protein binding | IPR003598 (SMART); IPR003599 (SMART); IPR007110 (PROFILE); IPR013098 (PFAM); IPR013783 (G3DSA:2.60.40.GENE3D); PTHR10075 (PANTHER), tmhmm (TMHMM), SSF48726 (SUPERFAMILY) | |
| 2712 | lib_dib_Filter15_IDBA_contig_53541_plus3 | 255 | dehydrogenase reductase sdr family member 12 | 585 | 1.61E-145 | 77.85% | 3 | F:catalytic activity; P:metabolic process; F:nucleotide binding | IPR002198 (PFAM); IPR002347 (PRINTS); IPR016040 (G3DSA:3.40.50.GENE3D); PTHR24316 (PANTHER), PTHR24316:SF19 (PANTHER), SignalP-NN(euk) (SIGNALP), SSF51735 (SUPERFAMILY) | yes |
| 2713 | lib_dib_Filter15_IDBA_contig_53575_plus3 | 74 | collagen alpha-1 chain | 252 | 7.42E-11 | 66.50% | 9 | P:metabolic process; P:anatomical structure morphogenesis; P:embryo development; P:multicellular organismal development; C:proteinaceous extracellular matrix; P:cellular component organization; P:cell differentiation; P:biological_process; F:structural molecule activity | IPR008160 (PFAM); PTHR24023 (PANTHER) | |
| 2714 | lib_dib_Filter15_IDBA_contig_53576_plus3 | 90 | collagen alpha-1 chain | 324 | 1.80E-10 | 66.45% | 9 | P:metabolic process; P:anatomical structure morphogenesis; P:embryo development; P:multicellular organismal development; C:proteinaceous extracellular matrix; P:cellular component organization; P:cell differentiation; P:biological_process; F:structural molecule activity | IPR008160 (PFAM); PTHR24023 (PANTHER), PTHR24023:SF42 (PANTHER) | |
| 2715 | lib_dib_Filter15_IDBA_contig_53594_minus3 | 137 | 39s ribosomal protein mitochondrial | 444 | 1.01E-120 | 66.70% | 3 | C:cytoplasm; C:organelle; C:intracellular | IPR008914 (G3DSA:3.90.280.GENE3D); PTHR11362 (PANTHER), PTHR11362:SF1 (PANTHER) | |
| 2716 | lib_dib_Filter15_IDBA_contig_53605_plus2 | 33 | l-xylulose reductase | 197 | 1.76E-77 | 73.55% | 11 | F:catalytic activity; P:carbohydrate metabolic process; P:metabolic process; P:nucleobase-containing compound metabolic process; P:secondary metabolic process; C:cell; C:organelle; C:cytoplasm; F:protein binding; P:cellular component organization; F:nucleotide binding | IPR002198 (PRINTS); IPR002347 (PRINTS); IPR016040 (G3DSA:3.40.50.GENE3D); IPR020904 (PROSITE); PTHR24311 (PANTHER), PTHR24311:SF0 (PANTHER), PF13561 (PFAM), SSF51735 (SUPERFAMILY) | |
| 2717 | lib_dib_Filter15_IDBA_contig_53630_minus3 | 24 | neurogenic locus notch homolog protein 2-like | 197 | 9.10E-31 | 62.20% | 2 | F:protein binding; F:calcium ion binding | IPR000152 (PROSITE); IPR000436 (SMART); IPR000742 (PFAM); IPR001881 (SMART); IPR013032 (PROSITE); IPR018097 (PROSITE); PR00010 (PRINTS), G3DSA:2.10.25.10 (GENE3D), G3DSA:2.10.70.10 (GENE3D), PTHR24044 (PANTHER), SSF57196 (SUPERFAMILY) | |
| 2718 | lib_dib_Filter15_IDBA_contig_53639_plus3 | 18 | protocadherin fat 4 | 197 | 4.54E-55 | 55.55% | 10 | P:cellular component organization; P:cell differentiation; P:multicellular organismal development; P:anatomical structure morphogenesis; F:signal transducer activity; C:cell; P:signal transduction; P:biological_process; P:regulation of biological process; F:calcium ion binding | IPR002126 (PRINTS); IPR015919 (SUPERFAMILY); PTHR24027 (PANTHER) | |

| | | | | | | | | | | |
|---|---|---|---|---|---|---|---|---|---|---|
| 2719 | lib_dib_Filter15_IDBA_contig_53647_minus3 | 609 | cub and sushi domain-containing protein 1-like | 395 | 6.74E-108 | 51.45% | 1 | F:calcium ion binding | IPR000152 (PROSITE); IPR000436 (PFAM); IPR001881 (PFAM); IPR011641 (PFAM); IPR018097 (PROSITE); G3DSA:2.10.25.10 (GENE3D), G3DSA:2.10.70.10 (GENE3D), PTHR19325 (PANTHER), SSF57196 (SUPERFAMILY) | |
| 2720 | lib_dib_Filter15_IDBA_contig_53697_minus2 | 27 | ankyrin repeat and socs box protein 13 | 197 | 4.46E-37 | 61.35% | 1 | F:protein binding | IPR001496 (PFAM); IPR002110 (SMART); IPR020683 (G3DSA:1.25.40.GENE3D); PTHR24196 (PANTHER), PTHR24196:SF3 (PANTHER) | |
| 2721 | lib_dib_Filter15_IDBA_contig_53702_minus3 | 68 | discoidin domain-containing receptor 2-like | 266 | 1.92E-60 | 73.20% | 7 | P:biological_process; P:anatomical structure morphogenesis; P:multicellular organismal development; F:receptor activity; F:protein kinase activity; P:cellular component organization; P:cell differentiation | IPR000421 (PFAM); IPR008979 (SUPERFAMILY); IPR027143 (PTHR10127:PANTHER); G3DSA:2.60.120.260 (GENE3D), PTHR10127 (PANTHER), SignalP-NN(euk) (SIGNALP) | yes |
| 2722 | lib_dib_Filter15_IDBA_contig_53756_minus1 | 91 | protein o-linked-mannose beta- -n-acetylglucosaminyltransferase 1 | 361 | 3.31E-156 | 58.25% | 9 | C:integral to membrane; P:protein glycosylation; C:Golgi membrane; F:acetylglucosaminyltransferase activity; F:molecular_function; P:biological_process; C:cellular_component; F:transferase activity; F:transferase activity, transferring glycosyl groups | IPR026101 (PANTHER); G3DSA:3.90.550.10 (GENE3D), PTHR14592:SF1 (PANTHER) | |
| 2723 | lib_dib_Filter15_IDBA_contig_53757_minus1 | 38 | oncoprotein induced transcript 1 | 167 | 9.31E-31 | 57.80% | 7 | F:molecular_function; C:cytoplasmic vesicle; P:multicellular organismal development; P:biological_process; C:extracellular region; C:cellular_component; P:negative regulation of insulin secretion | IPR026101 (PANTHER) | |
| 2724 | lib_dib_Filter15_IDBA_contig_53758_minus3 | 23 | l-ascorbate oxidase-like | 197 | 4.22E-47 | 62.85% | 2 | F:catalytic activity; F:binding | IPR008972 (G3DSA:2.60.40.GENE3D); IPR011707 (PFAM); PTHR11709 (PANTHER) | |
| 2725 | lib_dib_Filter15_IDBA_contig_53818_plus2 | 33 | inactive dipeptidyl peptidase 10-like | 197 | 1.03E-24 | 49.70% | 3 | P:protein metabolic process; P:catabolic process; C:cell | IPR002469 (PFAM); G3DSA:2.140.10.30 (GENE3D), PTHR11731 (PANTHER), PTHR11731:SF23 (PANTHER), SSF82171 (SUPERFAMILY) | |
| 2726 | lib_dib_Filter15_IDBA_contig_53848_plus3 | 51 | arylacetamide deacetylase-like | 371 | 1.11E-68 | 53.40% | 2 | P:metabolic process; F:hydrolase activity | IPR013094 (PFAM); G3DSA:3.40.50.1820 (GENE3D), PTHR23024 (PANTHER), SignalP-NN(euk) (SIGNALP), tmhmm (TMHMM), SSF53474 (SUPERFAMILY) | |
| 2727 | lib_dib_Filter15_IDBA_contig_53850_minus1 | 29 | chondroitin beta- -n-acetyl galactosaminyl transferase 1 | 197 | 8.26E-103 | 79.20% | 6 | P:response to external stimulus; P:response to stress; C:Golgi apparatus; F:transferase activity; P:biosynthetic process; P:carbohydrate metabolic process | IPR008428 (PANTHER); G3DSA:3.90.550.10 (GENE3D), SSF53448 (SUPERFAMILY) | |
| 2728 | lib_dib_Filter15_IDBA_contig_53878_plus1 | 17 | slit homolog 3 protein | 197 | 3.13E-65 | 54.90% | 13 | P:anatomical structure morphogenesis; P:cellular component organization; P:cell differentiation; P:multicellular organismal development; P:signal transduction; F:receptor binding; P:biological_process; C:extracellular region; P:behavior; P:response to external stimulus; P:cell growth; P:regulation of biological process; P:response to endogenous stimulus | IPR001611 (PROFILE); IPR003591 (SMART); G3DSA:3.80.10.10 (GENE3D), PTHR24367 (PANTHER), PF13855 (PFAM), SSF52058 (SUPERFAMILY) | |
| 2729 | lib_dib_Filter15_IDBA_contig_53915_plus3 | 125 | e3 ubiquitin-protein ligase traf7 | 415 | 4.48E-102 | 58.70% | 2 | P:biological_process; F:protein binding | IPR001680 (PFAM); IPR015943 (G3DSA:2.130.10.GENE3D); IPR017986 (PROFILE); IPR019775 (PROSITE); IPR020472 (PRINTS); PTHR22844 (PANTHER), tmhmm (TMHMM) | |

| | | | | | | | | | |
|---|---|---|---|---|---|---|---|---|---|
| 2730 | lib_dib_Filter15_IDBA_contig_53919_plus2 | 23 | ankyrin repeat domain-containing protein 54 | 196 | 1.19E-54 | 69.20% | 12 | P:metabolic process; P:regulation of biological process; P:cell cycle; C:cell; F:protein binding; P:transport; P:cell differentiation; P:multicellular organismal development; P:signal transduction; C:cytoplasm; C:nucleus; F:enzyme regulator activity | IPR002110 (SMART); IPR020683 (G3DSA:1.25.40.GENE3D); PTHR24197 (PANTHER), PTHR24197:SF1 (PANTHER) |
| 2731 | lib_dib_Filter15_IDBA_contig_53970_plus1 | 96 | protein crumbs-like | 250 | 5.53E-12 | 79.45% | 18 | F:protein binding; F:cytoskeletal protein binding; P:signal transduction; P:multicellular organismal development; C:plasma membrane; P:anatomical structure morphogenesis; P:embryo development; P:regulation of biological process; P:growth; P:cellular component organization; P:cell differentiation; P:organelle organization; C:cell; P:metabolic process; F:calcium ion binding; P:cell proliferation; P:biological_process; P:reproduction | PTHR24838 (PANTHER), PTHR24838:SF54 (PANTHER) |
| 2732 | lib_dib_Filter15_IDBA_contig_54064_plus2 | 27 | matrix metalloproteinase 9 | 196 | 9.12E-16 | 45.10% | 9 | P:response to external stimulus; F:protein binding; P:multicellular organismal development; P:regulation of biological process; P:biological_process; P:response to stress; F:peptidase activity; C:extracellular region; P:metabolic process | IPR000562 (G3DSA:2.10.10.GENE3D); IPR013806 (SUPERFAMILY); PTHR22918 (PANTHER), PTHR22918:SF0 (PANTHER) |
| 2733 | lib_dib_Filter15_IDBA_contig_54080_plus3 | 19 | low quality protein: hemicentin-1 | 196 | 1.09E-61 | 63.35% | 1 | C:extracellular region | IPR000884 (PFAM); IPR027231 (PANTHER); PR01705 (PRINTS), G3DSA:2.20.100.10 (GENE3D), PTHR11036:SF3 (PANTHER) |
| 2734 | lib_dib_Filter15_IDBA_contig_54097_minus3 | 63 | discoidin domain-containing receptor 2 | 344 | 2.55E-62 | 66.35% | 13 | P:regulation of biological process; ; P:cellular protein modification process; P:multicellular organismal development; P:cellular component organization; P:metabolic process; P:anatomical structure morphogenesis; P:growth; P:cell differentiation; F:protein binding; P:cell proliferation; C:plasma membrane; F:nucleotide binding | IPR000719 (PROFILE); IPR001245 (PFAM); IPR011009 (SUPERFAMILY); G3DSA:3.30.200.20 (GENE3D), PTHR24416 (PANTHER), PTHR24416:SF67 (PANTHER), tmhmm (TMHMM) |
| 2735 | lib_dib_Filter15_IDBA_contig_54133_plus1 | 85 | slit homolog 2 | 259 | 2.44E-44 | 65.60% | 9 | C:cell; F:protein binding; F:receptor activity; P:regulation of biological process; P:response to stress; P:biosynthetic process; P:response to biotic stimulus; P:embryo development; P:signal transduction | IPR000157 (PFAM); IPR027202 (PTHR24371:PANTHER); PR01537 (PRINTS), G3DSA:3.40.50.10140 (GENE3D), PTHR24371 (PANTHER), SignalP-NN(euk) (SIGNALP), tmhmm (TMHMM) |
| 2736 | lib_dib_Filter15_IDBA_contig_54148_minus3 | 22 | furin-like protease isoforms 1 1-x 2-like | 195 | 7.03E-77 | 71.80% | 3 | F:peptidase activity; P:protein metabolic process; P:catabolic process | IPR000209 (G3DSA:3.40.50.GENE3D); IPR002884 (PFAM); IPR008979 (SUPERFAMILY); IPR015500 (PANTHER); G3DSA:2.60.120.260 (GENE3D), PTHR10795:SF16 (PANTHER) |
| 2737 | lib_dib_Filter15_IDBA_contig_54186_minus3 | 48 | peptidylglycine alpha-amidating monooxygenase | 195 | 5.61E-10 | 56.20% | 15 | ; C:cytoplasmic membrane-bounded vesicle; C:cytoskeleton; P:regulation of biological process; P:cytoskeleton organization; P:metabolic process; C:cell; P:anatomical structure morphogenesis; P:cellular component organization; P:cell differentiation; P:multicellular organismal development; F:protein binding; C:plasma membrane; F:binding; F:catalytic activity | IPR000323 (G3DSA:2.60.120.GENE3D); IPR000720 (PRINTS); IPR008977 (SUPERFAMILY); PTHR10680 (PANTHER), PTHR10680:SF11 (PANTHER), SignalP-NN(euk) (SIGNALP) |

| | | | | | | | | | | |
|---|---|---|---|---|---|---|---|---|---|---|
| 2738 | lib_dib_Filter15_IDBA_contig_5419_minus3 | 1874 | ankyrin repeat-containing protein | 555 | 1.90E-27 | 46.35% | 1 | F:protein binding | IPR001496 (PFAM); IPR002110 (PRINTS); IPR020683 (G3DSA:1.25.40.GENE3D); PTHR24128 (PANTHER) | |
| 2739 | lib_dib_Filter15_IDBA_contig_5424_minus2 | 371 | l-xylulose reductase | 149 | 5.15E-36 | 69.20% | 5 | P:carbohydrate metabolic process; F:catalytic activity; P:cellular component organization; C:cell; F:nucleotide binding | IPR002198 (PFAM); IPR002347 (PRINTS); IPR016040 (G3DSA:3.40.50.GENE3D); PTHR24311 (PANTHER), PTHR24311:SF0 (PANTHER), SSF51735 (SUPERFAMILY) | |
| 2740 | lib_dib_Filter15_IDBA_contig_54354_minus3 | 26 | low quality protein: multiple epidermal growth factor-like domains protein 6 | 195 | 1.97E-08 | 65.85% | 2 | F:protein binding; F:calcium ion binding | IPR000742 (SMART); IPR001881 (SMART); G3DSA:2.10.25.10 (GENE3D), PTHR14949 (PANTHER), PTHR14949:SF13 (PANTHER), SignalP-NN(euk) (SIGNALP), SSF57196 (SUPERFAMILY) | yes |
| 2741 | lib_dib_Filter15_IDBA_contig_54404_plus1 | 25 | isoform a | 211 | 1.36E-18 | 49.50% | 1 | P:biological_process | IPR009030 (SUPERFAMILY); IPR011641 (PFAM); PTHR24046 (PANTHER), PTHR24046:SF0 (PANTHER), tmhmm (TMHMM) | |
| 2742 | lib_dib_Filter15_IDBA_contig_54453_minus1 | 4489 | hhip-like protein 1 | 762 | 2.87E-14 | 52.25% | 2 | F:receptor activity; C:cell | IPR001190 (PRINTS); IPR017448 (SMART); PR01217 (PRINTS), G3DSA:3.10.250.10 (GENE3D), PTHR19331 (PANTHER), PTHR19331:SF118 (PANTHER) | |
| 2743 | lib_dib_Filter15_IDBA_contig_5448_minus1 | 893 | fibrinogen-like protein 1-like | 111 | 6.90E-13 | 58.00% | 8 | P:regulation of biological process; P:cell death; P:response to biotic stimulus; C:extracellular space; P:lipid metabolic process; P:biological_process; F:binding; F:enzyme regulator activity | IPR002181 (PFAM); IPR014716 (G3DSA:3.90.215.GENE3D); PTHR19143 (PANTHER), PTHR19143:SF47 (PANTHER) | |
| 2744 | lib_dib_Filter15_IDBA_contig_5453_minus2 | 364 | delta-like protein 2 | 1471 | 1.52E-140 | 53.15% | 2 | F:protein binding; F:calcium ion binding | IPR000152 (PROSITE); IPR000742 (SMART); IPR001368 (PROFILE); IPR001881 (SMART); IPR013032 (PROSITE); IPR018097 (PROSITE); IPR024731 (PFAM); G3DSA:2.10.25.10 (GENE3D), G3DSA:2.10.50.10 (GENE3D), PTHR24039 (PANTHER), SignalP-NN(euk) (SIGNALP), SSF57196 (SUPERFAMILY) | ye |
| 2745 | lib_dib_Filter15_IDBA_contig_54563_minus3 | 41 | tubby protein homolog | 194 | 4.92E-34 | 93.30% | 0 | - | IPR000007 (PRINTS); IPR025659 (SUPERFAMILY); PTHR16517 (PANTHER), tmhmm (TMHMM) | |
| 2746 | lib_dib_Filter15_IDBA_contig_54579_plus1 | 277 | dicarbonyl l-xylulose reductase | 336 | 2.19E-69 | 68.55% | 5 | F:catalytic activity; P:carbohydrate metabolic process; P:metabolic process; P:cellular component organization; F:nucleotide binding | IPR002198 (PRINTS); IPR002347 (PRINTS); IPR016040 (G3DSA:3.40.50.GENE3D); IPR020904 (PROSITE); PTHR24311 (PANTHER), PTHR24311:SF0 (PANTHER), PF13561 (PFAM), SSF51735 (SUPERFAMILY) | |
| 2747 | lib_dib_Filter15_IDBA_contig_5458_minus3 | 75 | sea star regeneration-associated protease srap-like | 271 | 6.62E-56 | 58.75% | 3 | F:peptidase activity; P:protein metabolic process; P:catabolic process | IPR001254 (PFAM); IPR001314 (PRINTS); IPR009003 (SUPERFAMILY); IPR018114 (PROSITE); G3DSA:2.40.10.10 (GENE3D), PTHR24256 (PANTHER), SignalP-NN(euk) (SIGNALP) | |
| 2748 | lib_dib_Filter15_IDBA_contig_5459_minus2 | 1862 | sea star regeneration-associated protease srap-like | 306 | 7.07E-64 | 57.65% | 12 | P:cellular component organization; F:peptidase activity; C:extracellular space; P:biological_process; P:response to external stimulus; P:regulation of biological process; P:response to stress; F:protein binding; F:binding; C:plasma membrane; P:protein metabolic process; P:catabolic process | IPR001254 (PFAM); IPR001314 (PRINTS); IPR009003 (SUPERFAMILY); IPR018114 (PROSITE); G3DSA:2.40.10.10 (GENE3D), PTHR24265 (PANTHER) | |
| 2749 | lib_dib_Filter15_IDBA_contig_54644_minus3 | 80 | fibrillin 1-like | 367 | 8.84E-10 | 66.15% | 2 | F:protein binding; F:calcium ion binding | IPR000152 (PROSITE); IPR000742 (SMART); IPR001881 (PFAM); IPR013032 (PROSITE); IPR018097 (PROSITE); G3DSA:2.10.25.10 (GENE3D), PTHR24048 (PANTHER), tmhmm (TMHMM), SSF57196 (SUPERFAMILY) | |

| | | | | | | | | | | |
|---|---|---|---|---|---|---|---|---|---|---|
| 2750 | lib_dib_Filter15_IDBA_contig_54648_plus3 | 24 | thrombospondin type-1 domain-containing protein 7b | 194 | 4.64E-44 | 51.10% | 0 | - | IPR000884 (PFAM); G3DSA:2.20.100.10 (GENE3D), PTHR11311 (PANTHER), PTHR11311:SF2 (PANTHER) | |
| 2751 | lib_dib_Filter15_IDBA_contig_54653_plus3 | 16 | zfp64 protein | 194 | 8.74E-67 | 49.80% | 3 | F:binding; F:nucleic acid binding; C:intracellular | IPR007087 (PROSITE); IPR013087 (G3DSA:3.30.160.GENE3D); IPR015880 (SMART); PTHR11389 (PANTHER), PF13465 (PFAM), PF13894 (PFAM), SSF57667 (SUPERFAMILY) | |
| 2752 | lib_dib_Filter15_IDBA_contig_54672_plus1 | 33 | collagen triple helix repeat protein | 195 | 8.42E-07 | 74.75% | 1 | C:collagen | IPR008160 (PFAM); PTHR24023 (PANTHER) | |
| 2753 | lib_dib_Filter15_IDBA_contig_54733_minus3 | 135 | galectin-8 isoform 1 | 378 | 2.32E-59 | 53.15% | 1 | F:carbohydrate binding | IPR001079 (PFAM); IPR008985 (SUPERFAMILY); IPR013320 (G3DSA:2.60.120.GENE3D); PTHR11346 (PANTHER) | |
| 2754 | lib_dib_Filter15_IDBA_contig_54734_minus3 | 138 | galectin-8 isoform 1 | 385 | 3.13E-59 | 52.70% | 1 | F:carbohydrate binding | IPR001079 (PFAM); IPR008985 (SUPERFAMILY); IPR013320 (G3DSA:2.60.120.GENE3D); PTHR11346 (PANTHER) | |
| 2755 | lib_dib_Filter15_IDBA_contig_54735_plus1 | 22 | low density lipo protein 1b (deleted in tumors) | 194 | 4.42E-32 | 58.20% | 14 | P:transport; P:cellular component organization; P:biological_process; P:cell proliferation; P:regulation of biological process; P:metabolic process; P:signal transduction; P:cell death; C:cell; P:protein transport; C:endosome; F:protein binding; F:receptor activity; C:cytoplasmic membrane-bounded vesicle | IPR002172 (G3DSA:4.10.400.GENE3D); IPR023415 (PROSITE); PR00261 (PRINTS), G3DSA:4.10.1220.10 (GENE3D), PTHR10529 (PANTHER) | |
| 2756 | lib_dib_Filter15_IDBA_contig_54772_minus1 | 545 | nicotinic acetylcholine receptor alpha6 subunit | 444 | 7.72E-34 | 54.90% | 3 | P:ion transport; C:cell; F:ion channel activity | IPR006201 (PANTHER); IPR006202 (G3DSA:2.70.170.GENE3D); PTHR18945:SF92 (PANTHER) | |
| 2757 | lib_dib_Filter15_IDBA_contig_54773_minus1 | 588 | nicotinic acetylcholine receptor alpha6 subunit | 495 | 8.31E-34 | 54.85% | 3 | P:ion transport; C:cell; F:ion channel activity | IPR006201 (PANTHER); IPR006202 (G3DSA:2.70.170.GENE3D); PTHR18945:SF92 (PANTHER) | |
| 2758 | lib_dib_Filter15_IDBA_contig_54801_plus3 | 15 | zinc finger protein 705d | 193 | 1.23E-12 | 63.25% | 3 | F:binding; F:nucleic acid binding; C:intracellular | IPR007087 (PROSITE); IPR013087 (G3DSA:3.30.160.GENE3D); IPR015880 (SMART); PTHR24382 (PANTHER), PTHR24382:SF0 (PANTHER), PF13465 (PFAM), SSF57667 (SUPERFAMILY) | |
| 2759 | lib_dib_Filter15_IDBA_contig_54849_plus1 | 30 | discoidin domain-containing receptor 2-like | 194 | 5.37E-25 | 60.55% | 2 | F:catalytic activity; P:biological_process | IPR000421 (PFAM); IPR008979 (SUPERFAMILY); G3DSA:2.60.120.260 (GENE3D), PTHR24543 (PANTHER), SignalP-NN(euk) (SIGNALP) | yes |
| 2760 | lib_dib_Filter15_IDBA_contig_54876_minus2 | 566 | kelch-like protein 24 | 753 | 0 | 51.40% | 1 | F:protein binding | IPR000210 (SMART); IPR006652 (PFAM); IPR011333 (G3DSA:3.30.710.GENE3D); IPR011705 (PFAM); IPR013069 (PFAM); IPR015915 (G3DSA:2.120.10.GENE3D); PTHR24412 (PANTHER), PF13964 (PFAM), SSF117281 (SUPERFAMILY) | |
| 2761 | lib_dib_Filter15_IDBA_contig_54891_plus1 | 30 | partial | 194 | 6.56E-59 | 52.20% | 2 | F:hydrolase activity; F:cholinesterase activity | IPR002018 (PFAM); G3DSA:3.40.50.1820 (GENE3D), PTHR11559 (PANTHER), SSF53474 (SUPERFAMILY) | |
| 2762 | lib_dib_Filter15_IDBA_contig_54948_minus1 | 19 | retinol retinaldehyde reductase | 194 | 9.35E-57 | 64.80% | 7 | P:anatomical structure morphogenesis; P:cell differentiation; P:multicellular organismal development; P:biosynthetic process; P:lipid metabolic process; F:catalytic activity; F:nucleotide binding | IPR002198 (PFAM); IPR002347 (PRINTS); IPR016040 (G3DSA:3.40.50.GENE3D); IPR020904 (PROSITE); PTHR24316 (PANTHER), PTHR24316:SF39 (PANTHER), tmhmm (TMHMM), SSF51735 (SUPERFAMILY) | |
| 2763 | lib_dib_Filter15_IDBA_contig_5495_minus2 | 23 | carboxypeptidase b-like | 275 | 2.11E-50 | 56.60% | 4 | F:peptidase activity; P:protein metabolic process; P:catabolic process; F:binding | IPR000834 (PFAM); G3DSA:3.40.630.10 (GENE3D), PTHR11705 (PANTHER), SSF53187 (SUPERFAMILY) | |
| 2764 | lib_dib_Filter15_IDBA_contig_55076_minus1 | 74 | ankyrin unc44 | 349 | 2.79E-51 | 58.15% | 1 | F:protein binding | IPR002110 (PRINTS); IPR020683 (G3DSA:1.25.40.GENE3D); PTHR24188 (PANTHER) | |

| | | | | | | | | | |
|---|---|---|---|---|---|---|---|---|---|
| 2765 | lib_dib_Filter15_IDBA_contig_55118_minus2 | 171 | dnaj homolog subfamily c member 17-like | 437 | 4.63E-81 | 62.30% | 1 | F:binding | IPR001623 (PRINTS); PTHR24078 (PANTHER), PTHR24078:SF5 (PANTHER) |
| 2766 | lib_dib_Filter15_IDBA_contig_55168_plus2 | 33 | low-density lipoprotein receptor-related protein 6 isoform 2 | 193 | 8.61E-79 | 77.80% | 25 | P:anatomical structure morphogenesis; P:embryo development; C:organelle; C:cytoplasm; C:cell; F:enzyme regulator activity; P:multicellular organismal development; P:regulation of biological process; F:receptor binding; F:protein binding; P:transport; P:cellular component organization; C:plasma membrane; P:cell-cell signaling; ; P:cell differentiation; P:cell death; P:biological_process; C:intracellular; C:protein complex; F:receptor activity; F:transporter activity; C:cellular_component; P:cell cycle; P:reproduction | IPR000033 (PFAM); IPR000742 (SMART); IPR011042 (G3DSA:2.120.10.GENE3D); G3DSA:2.10.25.10 (GENE3D), PTHR10529 (PANTHER), SSF57196 (SUPERFAMILY), SSF63825 (SUPERFAMILY) |
| 2767 | lib_dib_Filter15_IDBA_contig_55182_minus1 | 884 | | 280 | 0 | 100% | 1 | F:protein binding | IPR003961 (PFAM); IPR013783 (G3DSA:2.60.40.GENE3D); PR00014 (PRINTS) |
| 2768 | lib_dib_Filter15_IDBA_contig_55194_minus2 | 26 | matrix metalloproteinase 2 | 193 | 3.64E-13 | 40.35% | 11 | F:metalloendopeptidase activity; F:hydrolase activity; F:zinc ion binding; F:metallopeptidase activity; C:extracellular matrix; F:peptidase activity; P:proteolysis; F:metal ion binding; P:metabolic process; F:carbohydrate binding; C:collagen | IPR000562 (G3DSA:2.10.10.GENE3D); IPR013806 (SUPERFAMILY); PTHR22918 (PANTHER), PTHR22918:SF0 (PANTHER) |
| 2769 | lib_dib_Filter15_IDBA_contig_55232_plus2 | 28 | leucine-rich repeat-containing protein 4c | 193 | 1.08E-07 | 47.55% | 5 | C:integral to membrane; C:membrane; P:signal transduction; C:intracellular; P:regulation of axonogenesis | IPR000483 (SMART); IPR003591 (SMART); G3DSA:3.80.10.10 (GENE3D), PTHR24373 (PANTHER), PF13855 (PFAM), tmhmm (TMHMM), SSF52058 (SUPERFAMILY) |
| 2770 | lib_dib_Filter15_IDBA_contig_55253_minus1 | 1230 | serine protease partial | 1020 | 1.49E-44 | 50.85% | 4 | C:cell; F:peptidase activity; P:protein metabolic process; P:catabolic process | IPR000998 (PRINTS); IPR001254 (PFAM); IPR001314 (PRINTS); IPR008985 (SUPERFAMILY); IPR009003 (SUPERFAMILY); IPR018114 (PROSITE); G3DSA:2.40.10.10 (GENE3D), PTHR24268 (PANTHER) |
| 2771 | lib_dib_Filter15_IDBA_contig_55264_minus3 | 46 | probable cytosolic iron-sulfur protein assembly protein ciao1 | 192 | 1.44E-87 | 81.25% | 6 | ; P:regulation of biological process; P:biosynthetic process; P:biological_process; P:cell proliferation; F:protein binding | IPR001680 (PFAM); IPR015943 (G3DSA:2.130.10.GENE3D); IPR017986 (PROFILE); IPR019775 (PROSITE); IPR020472 (PRINTS); PTHR19920 (PANTHER), PTHR19920:SF0 (PANTHER) |
| 2772 | lib_dib_Filter15_IDBA_contig_55319_minus1 | 59 | kelch-like protein 5 | 265 | 3.87E-86 | 85.60% | 3 | C:cytoskeleton; C:cytoplasm; F:actin binding | IPR000210 (SMART); IPR011333 (G3DSA:3.30.710.GENE3D); IPR011705 (PFAM); IPR013069 (PFAM); PTHR24412 (PANTHER), PTHR24412:SF73 (PANTHER) |
| 2773 | lib_dib_Filter15_IDBA_contig_55384_minus1 | 287 | ribonuclease oy-like | 316 | 1.43E-64 | 52.30% | 2 | F:RNA binding; F:nuclease activity | IPR001568 (G3DSA:3.90.730.GENE3D); IPR018188 (PROSITE); tmhmm (TMHMM) |
| 2774 | lib_dib_Filter15_IDBA_contig_55610_plus3 | 19 | unconventional myosin-xvi | 191 | 3.59E-51 | 63.35% | 6 | C:cell; P:regulation of biological process; C:nucleolus; F:actin binding; C:cytoplasm; C:nucleoplasm | IPR002110 (PRINTS); IPR020683 (G3DSA:1.25.40.GENE3D); PTHR24186 (PANTHER), PTHR24186:SF0 (PANTHER) |

| | | | | | | | | | | |
|---|---|---|---|---|---|---|---|---|---|---|
| 2775 | lib_dib_Filter15_IDBA_contig_55624_plus1 | 4086 | adp-ribosylation factor 1 | 177 | 8.82E-92 | 99.40% | 25 | F:signal transducer activity; C:Golgi apparatus; P:anatomical structure morphogenesis; P:cellular component organization; P:regulation of biological process; F:protein binding; P:biological_process; P:viral reproduction; P:transport; P:cell-cell signaling; F:nucleotide binding; P:cellular homeostasis; P:nucleobase-containing compound metabolic process; P:catabolic process; C:cytosol; P:response to stress; P:response to biotic stimulus; P:signal transduction; P:multicellular organismal development; C:cytoplasm; F:hydrolase activity; C:plasma membrane; P:organelle organization; P:cellular protein modification process; P:protein transport | IPR005225 (TIGRFAMs); IPR006687 (SMART); IPR006689 (PRINTS); IPR024156 (SMART); G3DSA:3.40.50.300 (GENE3D), PTHR11711 (PANTHER), SSF52540 (SUPERFAMILY) | |
| 2776 | lib_dib_Filter15_IDBA_contig_55630_plus1 | 608 | chymotrypsinogen b2 | 550 | 4.97E-56 | 57.95% | 3 | F:peptidase activity; P:protein metabolic process; P:catabolic process | IPR000519 (G3DSA:4.10.110.GENE3D); IPR001254 (PFAM); IPR001314 (PRINTS); IPR009003 (SUPERFAMILY); IPR017957 (PROSITE); IPR018114 (PROSITE); G3DSA:2.40.10.10 (GENE3D), PTHR24259 (PANTHER), SignalP-NN(euk) (SIGNALP) | yes |
| 2777 | lib_dib_Filter15_IDBA_contig_55631_plus3 | 728 | chymotrypsinogen b2 | 565 | 6.77E-56 | 57.95% | 3 | F:peptidase activity; P:protein metabolic process; P:catabolic process | IPR000519 (G3DSA:4.10.110.GENE3D); IPR001254 (PFAM); IPR001314 (PRINTS); IPR009003 (SUPERFAMILY); IPR017957 (PROSITE); IPR018114 (PROSITE); G3DSA:2.40.10.10 (GENE3D), PTHR24259 (PANTHER), SignalP-NN(euk) (SIGNALP) | yes |
| 2778 | lib_dib_Filter15_IDBA_contig_55658_plus1 | 1622 | bactericidal permeability increasing protein | 737 | 1.03E-49 | 50.20% | 1 | F:lipid binding | IPR001124 (PFAM); IPR017942 (PFAM); IPR017943 (SUPERFAMILY); G3DSA:3.15.10.10 (GENE3D), G3DSA:3.15.20.10 (GENE3D), PTHR10504 (PANTHER) | |
| 2779 | lib_dib_Filter15_IDBA_contig_55659_plus3 | 1951 | bactericidal permeability increasing protein | 753 | 9.48E-77 | 51.95% | 1 | F:lipid binding | IPR001124 (PFAM); IPR010562 (PFAM); IPR017942 (PFAM); IPR017943 (SUPERFAMILY); G3DSA:3.15.10.10 (GENE3D), G3DSA:3.15.20.10 (GENE3D), PTHR10504 (PANTHER), SignalP-NN(euk) (SIGNALP) | yes |
| 2780 | lib_dib_Filter15_IDBA_contig_55661_plus1 | 217 | bactericidal permeability increasing protein | 130 | 1.32E-20 | 55.05% | 1 | F:lipid binding | IPR001124 (PFAM); IPR017943 (SUPERFAMILY); G3DSA:3.15.20.10 (GENE3D), PTHR10504 (PANTHER) | |
| 2781 | lib_dib_Filter15_IDBA_contig_55664_plus1 | 17 | polypeptide n-acetylgalactosaminyltransferase 6-like | 192 | 3.85E-14 | 49.50% | 2 | F:transferase activity; P:protein metabolic process | IPR000772 (PFAM); G3DSA:2.80.10.50 (GENE3D), PTHR11675 (PANTHER), tmhmm (TMHMM) | |
| 2782 | lib_dib_Filter15_IDBA_contig_55694_minus2 | 36 | isoform f | 225 | 2.11E-58 | 53.75% | 3 | F:molecular_function; P:biological_process; C:cellular_component | IPR000859 (G3DSA:2.60.120.GENE3D); PTHR10127 (PANTHER), PTHR10127:SF345 (PANTHER) | |
| 2783 | lib_dib_Filter15_IDBA_contig_55741_minus3 | 17 | polypeptide n-acetylgalactosaminyltransferase 5 | 191 | 4.85E-39 | 62.85% | 5 | C:Golgi apparatus; P:biosynthetic process; P:cellular protein modification process; P:carbohydrate metabolic process; F:transferase activity | IPR001173 (PFAM); G3DSA:3.90.550.10 (GENE3D), PTHR11675 (PANTHER), SSF53448 (SUPERFAMILY) | |
| 2784 | lib_dib_Filter15_IDBA_contig_55776_plus3 | 16 | histone-lysine n-methyltransferase ehmt1 | 191 | 1.55E-35 | 65.85% | 7 | P:organelle organization; P:cellular protein modification process; F:protein binding; F:transferase activity; P:regulation of biological process; ; C:nucleus | IPR002110 (PRINTS); IPR020683 (G3DSA:1.25.40.GENE3D); PTHR22884 (PANTHER), PTHR22884:SF22 (PANTHER) | |
| 2785 | lib_dib_Filter15_IDBA_contig_55809_plus3 | 21 | gamma-interferon-inducible lysosomal thiol reductase | 191 | 4.20E-62 | 69.90% | 0 | - | IPR004911 (PANTHER); PTHR13234:SF7 (PANTHER) | |

| | | | | | | | | | | |
|---|---|---|---|---|---|---|---|---|---|---|
| 2786 | lib_dib_Filter15_IDBA_contig_55827_minus2 | 25 | gastrula zinc finger | 191 | 1.13E-22 | 67.40% | 4 | F:protein binding; F:binding; F:nucleic acid binding; C:intracellular | IPR001841 (PROFILE); IPR007087 (PFAM); IPR013087 (G3DSA:3.30.160.GENE3D); IPR015880 (SMART); PTHR11389 (PANTHER), PF13465 (PFAM), tmhmm (TMHMM), SSF57667 (SUPERFAMILY) | |
| 2787 | lib_dib_Filter15_IDBA_contig_55949_plus2 | 61 | microfibrillar-associated protein 4 type i | 232 | 2.13E-48 | 58.05% | 3 | F:receptor binding; P:signal transduction; C:extracellular space | IPR002181 (PFAM); IPR014715 (G3DSA:4.10.530.GENE3D); IPR014716 (G3DSA:3.90.215.GENE3D); PTHR19143 (PANTHER) | |
| 2788 | lib_dib_Filter15_IDBA_contig_55963_plus1 | 33 | hypothetical protein EAI_07730 | 191 | 8.96E-05 | 74.00% | 0 | - | PR01217 (PRINTS) | |
| 2789 | lib_dib_Filter15_IDBA_contig_56062_plus3 | 16 | protein o-linked-mannose beta-n-acetylglucosaminyltransferase 1-like | 190 | 6.90E-80 | 78.40% | 5 | C:Golgi apparatus; F:transferase activity; P:biosynthetic process; P:cellular protein modification process; P:carbohydrate metabolic process | IPR004139 (PANTHER); G3DSA:3.90.550.10 (GENE3D), PTHR10468:SF1 (PANTHER) | |
| 2790 | lib_dib_Filter15_IDBA_contig_56087_minus2 | 29 | deleted in malignant brain tumors 1 | 224 | 1.36E-29 | 52.00% | 2 | F:receptor activity; C:cell | IPR001190 (PRINTS); IPR017448 (SMART); G3DSA:3.10.250.10 (GENE3D), PTHR19331 (PANTHER), PTHR19331:SF118 (PANTHER) | |
| 2791 | lib_dib_Filter15_IDBA_contig_56108_minus2 | 690 | iron zinc purple acid phosphatase-like | 868 | 4.10E-147 | 65.50% | 2 | F:hydrolase activity; F:binding | IPR004843 (PFAM); IPR008963 (SUPERFAMILY); IPR015914 (G3DSA:2.60.40.GENE3D); IPR025733 (PFAM); G3DSA:3.60.21.10 (GENE3D), PTHR22953 (PANTHER), PTHR22953:SF0 (PANTHER), tmhmm (TMHMM), SSF56300 (SUPERFAMILY) | |
| 2792 | lib_dib_Filter15_IDBA_contig_56112_plus1 | 15 | syntaxin-binding protein 5 isoform 1 | 190 | 1.15E-93 | 86.20% | 11 | C:plasma membrane; C:cytoplasmic membrane-bounded vesicle; C:protein complex; P:transport; P:regulation of biological process; F:protein binding; P:anatomical structure morphogenesis; P:cellular component organization; P:cell differentiation; P:multicellular organismal development; P:protein transport | IPR015943 (G3DSA:2.130.10.GENE3D); IPR017986 (SUPERFAMILY); PTHR10241 (PANTHER), PTHR10241:SF19 (PANTHER) | |
| 2793 | lib_dib_Filter15_IDBA_contig_56123_plus2 | 25 | protocadherin-11 x-linked | 190 | 2.98E-43 | 57.40% | 3 | F:calcium ion binding; P:biological_process; C:plasma membrane | IPR002126 (PRINTS); IPR015919 (SUPERFAMILY); IPR020894 (PROSITE); PTHR24028 (PANTHER), PTHR24028:SF12 (PANTHER) | |
| 2794 | lib_dib_Filter15_IDBA_contig_56188_minus2 | 50 | glypican-6- partial | 190 | 1.03E-26 | 66.75% | 3 | C:plasma membrane; C:proteinaceous extracellular matrix; F:protein binding | IPR001863 (PANTHER); PTHR10822:SF15 (PANTHER), SignalP-NN(euk) (SIGNALP) | |
| 2795 | lib_dib_Filter15_IDBA_contig_56237_plus1 | 16 | cysteine-rich motor neuron 1 | 190 | 2.20E-20 | 66.10% | 5 | P:multicellular organismal development; P:embryo development; P:regulation of biological process; P:anatomical structure morphogenesis; F:protein binding | IPR001007 (PFAM); G3DSA:2.10.70.10 (GENE3D), PTHR11339 (PANTHER), PTHR11339:SF40 (PANTHER), tmhmm (TMHMM), SSF57603 (SUPERFAMILY) | |
| 2796 | lib_dib_Filter15_IDBA_contig_56256_minus2 | 16 | protein wnt-5b-like | 189 | 6.09E-101 | 82.65% | 7 | P:cell differentiation; P:regulation of biological process; P:multicellular organismal development; C:cell; F:receptor binding; P:signal transduction; C:proteinaceous extracellular matrix | IPR005817 (PRINTS); IPR018161 (PROSITE); IPR026537 (PTHR12027:PANTHER) | |
| 2797 | lib_dib_Filter15_IDBA_contig_56371_minus3 | 7594 | serine protease | 373 | 2.37E-61 | 54.20% | 3 | F:peptidase activity; P:protein metabolic process; P:catabolic process | IPR001254 (PFAM); IPR001314 (PRINTS); IPR009003 (SUPERFAMILY); IPR018114 (PROSITE); G3DSA:2.40.10.10 (GENE3D), PTHR24259 (PANTHER), SignalP-NN(euk) (SIGNALP) | yes |
| 2798 | lib_dib_Filter15_IDBA_contig_56377_plus2 | 11 | xylosyltransferase 1 | 189 | 1.91E-33 | 59.15% | 1 | F:transferase activity | PTHR19297 (PANTHER), PTHR19297:SF1 (PANTHER), SignalP-NN(euk) (SIGNALP) | yes |
| 2799 | lib_dib_Filter15_IDBA_contig_56382_plus1 | 21 | thrombospondin type-1 domain-containing protein 7b | 189 | 1.49E-45 | 47.90% | 1 | C:integral to membrane | IPR000884 (SUPERFAMILY); PTHR11311 (PANTHER), PTHR11311:SF2 (PANTHER) | |
| 2800 | lib_dib_Filter15_IDBA_contig_56409_minus3 | 23 | btb poz domain-containing protein 17-like | 189 | 6.62E-40 | 56.10% | 1 | F:protein binding | IPR000210 (SMART); IPR011333 (G3DSA:3.30.710.GENE3D); IPR013069 (PFAM); PTHR24412 (PANTHER) | |

| | | | | | | | | | | |
|---|---|---|---|---|---|---|---|---|---|---|
| 2801 | lib_dib_Filter15_IDBA_contig_56417_plus2 | 625 | defense protein 3-like | 435 | 3.38E-27 | 57.10% | 3 | P:carbohydrate metabolic process; F:cation binding; F:catalytic activity | IPR002861 (PFAM); PTHR23130 (PANTHER) | |
| 2802 | lib_dib_Filter15_IDBA_contig_56434_minus3 | 7326 | cell surface | 280 | 1.12E-19 | 48.60% | 16 | C:collagen; F:extracellular matrix structural constituent; F:extracellular matrix structural constituent conferring tensile strength; P:cellular component organization; C:collagen type IV; C:basement membrane; P:reproduction; P:positive regulation of multicellular organism growth; P:nematode larval development; P:embryo development ending in birth or egg hatching; P:locomotion; P:growth; F:structural molecule activity; P:heterophilic cell-cell adhesion; C:integral to membrane; P:pathogenesis | PTHR24023 (PANTHER), PTHR24023:SF80 (PANTHER), tmhmm (TMHMM) | |
| 2803 | lib_dib_Filter15_IDBA_contig_56456_minus1 | 162 | protein llp homolog | 322 | 1.28E-14 | 64.10% | 0 | - | IPR018784 (PFAM); SignalP-NN(euk) (SIGNALP), tmhmm (TMHMM) | yes |
| 2804 | lib_dib_Filter15_IDBA_contig_56457_minus1 | 124 | protein llp homolog | 243 | 2.90E-15 | 64.05% | 0 | - | IPR018784 (PFAM); SignalP-NN(euk) (SIGNALP) | yes |
| 2805 | lib_dib_Filter15_IDBA_contig_56470_plus3 | 11764 | l-xylulose reductase | 305 | 1.81E-62 | 67.95% | 3 | P:carbohydrate metabolic process; F:catalytic activity; F:nucleotide binding | IPR002198 (PRINTS); IPR002347 (PRINTS); IPR016040 (G3DSA:3.40.50.GENE3D); PTHR24311 (PANTHER), PTHR24311:SF0 (PANTHER), SSF51735 (SUPERFAMILY) | |
| 2806 | lib_dib_Filter15_IDBA_contig_56528_plus2 | 35 | hyaluronoglucosaminidase 4 | 209 | 2.31E-23 | 48.05% | 2 | F:hydrolase activity; P:carbohydrate metabolic process | IPR013785 (G3DSA:3.20.20.GENE3D); IPR017853 (SUPERFAMILY); IPR018155 (PANTHER) | |
| 2807 | lib_dib_Filter15_IDBA_contig_56670_plus1 | 17 | trefoil factor 2 | 189 | 6.14E-22 | 59.95% | 6 | P:regulation of biological process; F:receptor binding; C:extracellular region; F:peptidase activity; P:protein metabolic process; P:catabolic process | IPR000519 (G3DSA:4.10.110.GENE3D); IPR001254 (PFAM); IPR009003 (SUPERFAMILY); IPR017957 (PROSITE); IPR018114 (PROSITE); G3DSA:2.40.10.10 (GENE3D), PTHR24257 (PANTHER), PTHR24257:SF5 (PANTHER) | |
| 2808 | lib_dib_Filter15_IDBA_contig_56700_plus3 | 33 | retinol retinaldehyde reductase | 188 | 2.14E-56 | 66.95% | 6 | F:catalytic activity; P:biosynthetic process; P:lipid metabolic process; P:secondary metabolic process; P:metabolic process; F:nucleotide binding | IPR002198 (PRINTS); IPR002347 (PRINTS); IPR016040 (G3DSA:3.40.50.GENE3D); IPR020904 (PROSITE); PTHR24316 (PANTHER), PTHR24316:SF39 (PANTHER), SSF51735 (SUPERFAMILY) | |
| 2809 | lib_dib_Filter15_IDBA_contig_56704_plus1 | 50 | zinc finger protein 234-like | 247 | 4.54E-34 | 58.60% | 3 | F:binding; F:nucleic acid binding; C:intracellular | IPR007087 (PFAM); IPR013087 (G3DSA:3.30.160.GENE3D); IPR015880 (SMART); PTHR11389 (PANTHER), PF13465 (PFAM), SSF57667 (SUPERFAMILY) | |
| 2810 | lib_dib_Filter15_IDBA_contig_56705_minus3 | 430 | matrix metalloproteinase-9 | 684 | 4.70E-21 | 53.60% | 1 | F:hydrolase activity | IPR000562 (G3DSA:2.10.10.GENE3D); IPR013806 (SUPERFAMILY); PR00013 (PRINTS), PTHR22918 (PANTHER), PTHR22918:SF0 (PANTHER) | |
| 2811 | lib_dib_Filter15_IDBA_contig_56706_minus3 | 440 | matrix metalloproteinase-9 | 640 | 1.91E-21 | 53.55% | 1 | F:hydrolase activity | IPR000562 (G3DSA:2.10.10.GENE3D); IPR013806 (SUPERFAMILY); PR00013 (PRINTS), PTHR22918 (PANTHER), PTHR22918:SF0 (PANTHER), tmhmm (TMHMM) | |
| 2812 | lib_dib_Filter15_IDBA_contig_56707_minus1 | 17 | matrix metalloproteinase-9 | 123 | 8.36E-13 | 48.30% | 10 | F:protein binding; P:multicellular organismal development; P:biological_process; C:extracellular region; P:response to abiotic stimulus; P:regulation of biological process; P:response to stress; F:peptidase activity; P:metabolic process; P:response to external stimulus | IPR000562 (G3DSA:2.10.10.GENE3D); IPR013806 (SUPERFAMILY); PTHR22918 (PANTHER), PTHR22918:SF0 (PANTHER) | |
| 2813 | lib_dib_Filter15_IDBA_contig_56764_minus2 | 25 | angiopoietin-related protein 3 | 215 | 1.96E-22 | 59.85% | 4 | P:biological_process; F:binding; P:regulation of biological process; P:primary metabolic process | IPR002181 (PFAM); IPR014716 (G3DSA:3.90.215.GENE3D); PTHR19143 (PANTHER) | |

| | | | | | | | | | |
|---|---|---|---|---|---|---|---|---|---|
| 2814 | lib_dib_Filter15_IDBA_contig_56793_plus3 | 30 | btb poz domain-containing protein 17-like | 188 | 2.58E-32 | 63.00% | 1 | F:protein binding | IPR000210 (SMART); IPR011333 (G3DSA:3.30.710.GENE3D); IPR013069 (PFAM); PTHR24412 (PANTHER), PTHR24412:SF96 (PANTHER), tmhmm (TMHMM) |
| 2815 | lib_dib_Filter15_IDBA_contig_56811_minus1 | 140 | mesencephalic astrocyte-derived neurotrophic factor | 376 | 4.93E-07 | 77.10% | 1 | C:cytoplasm | IPR019345 (PFAM); PTHR12990 (PANTHER), PTHR12990:SF4 (PANTHER), SSF68906 (SUPERFAMILY) |
| 2816 | lib_dib_Filter15_IDBA_contig_56812_minus1 | 163 | mesencephalic astrocyte-derived neurotrophic factor | 344 | 4.00E-07 | 77.10% | 1 | C:cytoplasm | IPR019345 (PFAM); PTHR12990 (PANTHER), PTHR12990:SF4 (PANTHER), tmhmm (TMHMM), SSF68906 (SUPERFAMILY) |
| 2817 | lib_dib_Filter15_IDBA_contig_56813_minus1 | 121 | mesencephalic astrocyte-derived neurotrophic factor | 272 | 1.95E-07 | 77.10% | 1 | C:cytoplasm | IPR019345 (PFAM); PTHR12990 (PANTHER), PTHR12990:SF4 (PANTHER), SSF68906 (SUPERFAMILY) |
| 2818 | lib_dib_Filter15_IDBA_contig_56814_minus2 | 157 | mesencephalic astrocyte-derived neurotrophic factor | 188 | 2.61E-33 | 71.70% | 3 | P:cellular component organization; C:extracellular region; P:cellular homeostasis | IPR011001 (G3DSA:1.10.225.GENE3D); IPR019345 (PFAM); PTHR12990 (PANTHER), PTHR12990:SF6 (PANTHER), tmhmm (TMHMM), SSF68906 (SUPERFAMILY) |
| 2819 | lib_dib_Filter15_IDBA_contig_56829_minus1 | 21 | soma ferritin | 188 | 3.83E-77 | 81.55% | 5 | P:cellular homeostasis; F:catalytic activity; F:binding; P:ion transport; P:metabolic process | IPR001519 (PANTHER); IPR008331 (PFAM); IPR009040 (PROFILE); IPR009078 (SUPERFAMILY); IPR012347 (G3DSA:1.20.1260.GENE3D); IPR014034 (PROSITE) |
| 2820 | lib_dib_Filter15_IDBA_contig_56916_minus3 | 41 | ankyrin repeat protein | 260 | 1.98E-15 | 47.90% | 1 | F:protein binding | IPR002110 (PRINTS); IPR020683 (G3DSA:1.25.40.GENE3D); PTHR24197 (PANTHER) |
| 2821 | lib_dib_Filter15_IDBA_contig_56917_minus1 | 31 | protein | 188 | 9.27E-30 | 58.95% | 3 | C:extracellular region; F:protein binding; F:calcium ion binding | IPR000152 (PROSITE); IPR000742 (PFAM); IPR001881 (SMART); IPR013032 (PROSITE); IPR018097 (PROSITE); PR00010 (PRINTS), G3DSA:2.10.25.10 (GENE3D), PTHR24044 (PANTHER), SSF57196 (SUPERFAMILY) |
| 2822 | lib_dib_Filter15_IDBA_contig_56922_minus2 | 12 | collagen alpha-1 chain-like | 188 | 3.40E-23 | 54.85% | 7 | F:RNA binding; P:RNA-dependent DNA replication; F:RNA-directed DNA polymerase activity; P:cell adhesion; C:collagen; F:structural molecule activity; C:extracellular matrix | IPR008985 (SUPERFAMILY); IPR013320 (G3DSA:2.60.120.GENE3D); PTHR24023 (PANTHER), PTHR24023:SF68 (PANTHER), PF13385 (PFAM), SignalP-NN(euk) (SIGNALP) |
| 2823 | lib_dib_Filter15_IDBA_contig_56988_plus2 | 15 | ---NA--- | 187 | | | 0 | - | no IPS match |
| 2824 | lib_dib_Filter15_IDBA_contig_56988_plus3 | 15 | ---NA--- | 187 | | | 0 | - | no IPS match |
| 2825 | lib_dib_Filter15_IDBA_contig_57003_minus3 | 33 | ankyrin repeat protein | 187 | 1.57E-30 | 53.40% | 1 | F:protein binding | IPR002110 (PFAM); IPR020683 (G3DSA:1.25.40.GENE3D); PTHR24188 (PANTHER) |
| 2826 | lib_dib_Filter15_IDBA_contig_57097_minus1 | 21 | serine protease 56-like | 187 | 1.06E-24 | 51.75% | 3 | F:peptidase activity; P:protein metabolic process; P:catabolic process | IPR001254 (PFAM); IPR001314 (PRINTS); IPR009003 (SUPERFAMILY); IPR018114 (PROSITE); G3DSA:2.40.10.10 (GENE3D), PTHR24272 (PANTHER), SignalP-NN(euk) (SIGNALP) |
| 2827 | lib_dib_Filter15_IDBA_contig_57149_plus2 | 20 | thrombospondin type-1 domain-containing protein 7b | 187 | 4.02E-32 | 47.35% | 1 | C:integral to membrane | IPR000884 (PFAM); G3DSA:2.20.100.10 (GENE3D), PTHR11311 (PANTHER), PTHR11311:SF2 (PANTHER) |
| 2828 | lib_dib_Filter15_IDBA_contig_57204_plus3 | 26 | beta-d-xylosidase 5 | 187 | 9.04E-52 | 61.30% | 1 | P:carbohydrate metabolic process | IPR002772 (G3DSA:3.40.50.GENE3D); IPR026892 (PANTHER); PTHR30620:SF0 (PANTHER) |
| 2829 | lib_dib_Filter15_IDBA_contig_57217_plus3 | 17 | protein | 186 | 8.84E-17 | 58.15% | 3 | F:binding; F:nucleic acid binding; C:intracellular | IPR007087 (PROSITE); IPR013087 (G3DSA:3.30.160.GENE3D); IPR015880 (SMART); PTHR11389 (PANTHER), PF13465 (PFAM), SSF57667 (SUPERFAMILY) |

| | | | | | | | | | | |
|---|---|---|---|---|---|---|---|---|---|---|
| 2830 | lib_dib_Filter15_IDBA_contig_57250_minus2 | 43 | protein | 248 | 9.54E-41 | 65.85% | 8 | P:regulation of biological process; P:biological_process; P:signal transduction; P:multicellular organismal development; P:anatomical structure morphogenesis; C:cell; F:protein binding; F:calcium ion binding | IPR000152 (PROSITE); IPR000742 (PFAM); IPR000859 (G3DSA:2.60.120.GENE3D); IPR001881 (SMART); IPR013032 (PROSITE); PR00010 (PRINTS), G3DSA:2.10.25.10 (GENE3D), PTHR24044 (PANTHER), SSF57196 (SUPERFAMILY) | |
| 2831 | lib_dib_Filter15_IDBA_contig_57251_minus2 | 27 | protein | 113 | 1.59E-19 | 70.35% | 6 | C:cell; P:signal transduction; P:multicellular organismal development; P:anatomical structure morphogenesis; F:protein binding; F:calcium ion binding | IPR000152 (PROSITE); IPR000742 (PFAM); IPR000859 (G3DSA:2.60.120.GENE3D); IPR001881 (SMART); IPR013032 (PROSITE); PR00010 (PRINTS), G3DSA:2.10.25.10 (GENE3D), PTHR24044 (PANTHER), SSF57196 (SUPERFAMILY) | |
| 2832 | lib_dib_Filter15_IDBA_contig_57253_plus3 | 26 | fibrinogen gamma chain | 186 | 1.48E-29 | 58.15% | 12 | F:receptor binding; C:extracellular space; P:signal transduction; P:platelet activation; P:protein polymerization; C:external side of plasma membrane; C:fibrinogen complex; P:response to calcium ion; F:eukaryotic cell surface binding; C:cell cortex; C:platelet alpha granule; F:protein binding, bridging | IPR002181 (PFAM); IPR014716 (G3DSA:3.90.215.GENE3D); PTHR19143 (PANTHER), SignalP-NN(euk) (SIGNALP) | yes |
| 2833 | lib_dib_Filter15_IDBA_contig_57267_minus2 | 22 | btb poz domain-containing protein 6 | 187 | 2.73E-33 | 53.00% | 1 | F:protein binding | IPR000210 (SMART); IPR011333 (G3DSA:3.30.710.GENE3D); IPR011705 (PFAM); IPR013069 (PFAM); PTHR24413 (PANTHER) | |
| 2834 | lib_dib_Filter15_IDBA_contig_57310_minus2 | 30 | thrombospondin type-1 domain-containing protein 7a | 187 | 4.58E-50 | 48.15% | 5 | F:molecular_function; C:integral to membrane; C:membrane; P:biological_process; C:cellular_component | IPR000884 (SMART); G3DSA:2.20.100.10 (GENE3D), PTHR11311 (PANTHER), PTHR11311:SF2 (PANTHER) | |
| 2835 | lib_dib_Filter15_IDBA_contig_57366_minus2 | 617 | neurotrypsin | 733 | 4.60E-101 | 59.30% | 8 | C:extracellular region; F:peptidase activity; C:cell; P:protein metabolic process; P:catabolic process; C:organelle; C:cytoplasm; F:receptor activity | IPR000519 (G3DSA:4.10.110.GENE3D); IPR001190 (PRINTS); IPR017448 (SMART); G3DSA:3.10.250.10 (GENE3D), PTHR19331 (PANTHER), SignalP-NN(euk) (SIGNALP) | yes |
| 2836 | lib_dib_Filter15_IDBA_contig_57390_plus2 | 42 | cathepsin l | 186 | 1.43E-54 | 71.00% | 3 | P:protein metabolic process; P:catabolic process; F:peptidase activity | IPR000668 (PFAM); IPR013128 (PANTHER); IPR025660 (PROSITE); IPR025661 (PROSITE); G3DSA:3.90.70.10 (GENE3D), PTHR12411:SF57 (PANTHER), SSF54001 (SUPERFAMILY) | |
| 2837 | lib_dib_Filter15_IDBA_contig_57477_plus3 | 23 | tissue factor pathway inhibitor | 186 | 1.19E-59 | 54.55% | 1 | F:enzyme regulator activity | IPR002223 (PRINTS); IPR004094 (PFAM); IPR018112 (G3DSA:2.10.22.GENE3D); IPR020901 (PROSITE); PTHR10083 (PANTHER) | |
| 2838 | lib_dib_Filter15_IDBA_contig_57512_plus1 | 17 | ankyrin repeat protein | 186 | 9.63E-20 | 59.40% | 1 | F:protein binding | IPR002110 (PRINTS); IPR020683 (G3DSA:1.25.40.GENE3D); PTHR24198 (PANTHER) | |
| 2839 | lib_dib_Filter15_IDBA_contig_57532_minus1 | 33 | zinc metalloproteinase toh-2 | 237 | 4.93E-73 | 55.25% | 6 | P:biological_process; P:multicellular organismal development; F:peptidase activity; P:protein metabolic process; P:catabolic process; F:binding | IPR000859 (G3DSA:2.60.120.GENE3D); IPR001506 (PRINTS); IPR006026 (SMART); IPR013032 (PROSITE); IPR024079 (G3DSA:3.40.390.GENE3D); PTHR10127 (PANTHER), SSF55486 (SUPERFAMILY) | |
| 2840 | lib_dib_Filter15_IDBA_contig_57537_minus1 | 18 | neurogenic locus notch homolog protein 2-like | 186 | 5.85E-44 | 66.75% | 3 | C:proteinaceous extracellular matrix; F:protein binding; F:calcium ion binding | IPR000152 (PROSITE); IPR000742 (PFAM); IPR000859 (G3DSA:2.60.120.GENE3D); IPR001881 (SMART); IPR013032 (PROSITE); IPR018097 (PROSITE); PR00010 (PRINTS), G3DSA:2.10.25.10 (GENE3D), PTHR24048 (PANTHER), PTHR24048:SF15 (PANTHER), SSF57196 (SUPERFAMILY) | |

| | | | | | | | | | | |
|---|---|---|---|---|---|---|---|---|---|---|
| 2841 | lib_dib_Filter15_IDBA_contig_57545_plus3 | 34 | a chain structural consequences of accommodation of four non-cognate amino-acid residues in the s1 pocket of bovine trypsin and chymotrypsin | 186 | 1.11E-40 | 62.55% | 3 | F:peptidase activity; P:protein metabolic process; P:catabolic process | IPR001254 (PFAM); IPR001314 (PRINTS); IPR009003 (SUPERFAMILY); IPR018114 (PROSITE); G3DSA:2.40.10.10 (GENE3D), PTHR24256 (PANTHER) | |
| 2842 | lib_dib_Filter15_IDBA_contig_57598_minus1 | 61 | leucine-rich repeat-containing protein 40 | 325 | 3.19E-100 | 70.70% | 1 | F:protein binding | IPR001611 (PROFILE); IPR003591 (SMART); IPR015766 (PTHR23155:PANTHER); PR00019 (PRINTS), G3DSA:3.80.10.10 (GENE3D), PTHR23155 (PANTHER), PF13855 (PFAM), SM00364 (SMART), SSF52047 (SUPERFAMILY) | |
| 2843 | lib_dib_Filter15_IDBA_contig_57653_minus2 | 129 | low-density lipoprotein | 296 | 1.51E-13 | 57.85% | 11 | F:protein binding; P:biosynthetic process; P:lipid metabolic process; F:receptor activity; P:metabolic process; P:regulation of biological process; C:cell; P:transport; C:endosome; P:catabolic process; P:biological_process | IPR002172 (G3DSA:4.10.400.GENE3D); IPR023415 (PROSITE); PR00261 (PRINTS), PTHR10529 (PANTHER), PTHR10529:SF113 (PANTHER), SignalP-NN(euk) (SIGNALP), tmhmm (TMHMM) | yes |
| 2844 | lib_dib_Filter15_IDBA_contig_57719_plus3 | 19 | ankyrin repeat protein | 185 | 2.75E-24 | 57.05% | 1 | F:protein binding | IPR002110 (PRINTS); IPR020683 (G3DSA:1.25.40.GENE3D); PTHR24188 (PANTHER), PF13637 (PFAM) | |
| 2845 | lib_dib_Filter15_IDBA_contig_57778_plus2 | 24 | hepatocyte growth factor | 185 | 7.98E-40 | 57.90% | 13 | P:signal transduction; F:protein binding; P:anatomical structure morphogenesis; P:regulation of biological process; P:cell-cell signaling; P:multicellular organismal development; P:cellular component organization; C:extracellular region; P:response to external stimulus; P:response to stress; P:behavior; P:cellular protein modification process; F:peptidase activity | IPR000001 (G3DSA:2.40.20.GENE3D); IPR013806 (SUPERFAMILY); PR00018 (PRINTS), PTHR24272 (PANTHER), PTHR24272:SF3 (PANTHER) | |
| 2846 | lib_dib_Filter15_IDBA_contig_57829_plus2 | 16 | zinc finger protein 271- partial | 185 | 8.78E-31 | 55.85% | 3 | F:binding; F:nucleic acid binding; C:intracellular | IPR007087 (PROSITE); IPR013087 (G3DSA:3.30.160.GENE3D); IPR015880 (SMART); PTHR24402 (PANTHER), PTHR24402:SF3 (PANTHER), PF13465 (PFAM), PF13894 (PFAM), SSF57667 (SUPERFAMILY) | |
| 2847 | lib_dib_Filter15_IDBA_contig_57859_plus2 | 43 | netrin- partial | 266 | 5.87E-44 | 71.70% | 8 | P:anatomical structure morphogenesis; P:multicellular organismal development; P:cellular component organization; P:cell differentiation; P:regulation of biological process; F:protein binding; C:extracellular region; P:biological_process | IPR001134 (PROFILE); IPR008993 (SUPERFAMILY); IPR018933 (PFAM); G3DSA:2.40.50.120 (GENE3D), PTHR10574 (PANTHER), PTHR10574:SF44 (PANTHER) | |
| 2848 | lib_dib_Filter15_IDBA_contig_57883_minus3 | 27 | upf0454 protein c12orf49 homolog | 185 | 1.96E-59 | 78.55% | 1 | C:extracellular region | IPR019352 (PANTHER) | |
| 2849 | lib_dib_Filter15_IDBA_contig_57942_plus1 | 34 | subfamily member 9 | 329 | 5.42E-49 | 65.15% | 6 | F:unfolded protein binding; P:protein folding; F:heat shock protein binding; P:biological_process; C:cellular_component; P:social behavior | IPR001623 (PRINTS); IPR018253 (PROSITE); PTHR24078 (PANTHER), PTHR24078:SF16 (PANTHER) | |
| 2850 | lib_dib_Filter15_IDBA_contig_57998_plus3 | 19 | cyclin-dependent kinase 10-like | 184 | 2.33E-86 | 83.45% | 4 | F:protein kinase activity; P:cellular protein modification process; F:nucleotide binding; P:biological_process | IPR000719 (PFAM); IPR002290 (SMART); IPR008271 (PROSITE); IPR011009 (SUPERFAMILY); IPR017441 (PROSITE); IPR020635 (SMART); G3DSA:1.10.510.10 (GENE3D), G3DSA:3.30.200.20 (GENE3D), PTHR24056 (PANTHER), PTHR24056:SF133 (PANTHER) | |

| | | | | | | | | | |
|---|---|---|---|---|---|---|---|---|---|
| 2851 | lib_dib_Filter15_IDBA_contig_58018_plus2 | 2201 | acetylcholinesterase | 639 | 6.25E-134 | 56.80% | 1 | F:hydrolase activity | IPR000997 (PRINTS); IPR002018 (PFAM); IPR019819 (PROSITE); IPR019826 (PROSITE); G3DSA:3.40.50.1820 (GENE3D), PTHR11559 (PANTHER), PTHR11559:SF33 (PANTHER), SignalP-NN(euk) (SIGNALP), tmhmm (TMHMM), SSF53474 (SUPERFAMILY) |
| 2852 | lib_dib_Filter15_IDBA_contig_58019_plus3 | 20 | carbonic anhydrase-related protein 10-like | 184 | 1.66E-82 | 73.55% | 3 | F:catalytic activity; C:extracellular region; F:binding | IPR001148 (G3DSA:3.10.200.GENE3D); IPR018347 (PTHR18952:PANTHER); IPR023561 (PANTHER) |
| 2853 | lib_dib_Filter15_IDBA_contig_58024_plus1 | 38 | transmembrane protease serine 3 | 238 | 2.35E-45 | 57.05% | 3 | F:peptidase activity; P:protein metabolic process; P:catabolic process | IPR001254 (PFAM); IPR001314 (PRINTS); IPR009003 (SUPERFAMILY); IPR018114 (PROSITE); G3DSA:2.40.10.10 (GENE3D), PTHR24256 (PANTHER) |
| 2854 | lib_dib_Filter15_IDBA_contig_58052_minus2 | 17 | isoform a | 184 | 4.41E-34 | 55.15% | 2 | P:transport; F:binding | IPR002157 (PFAM); G3DSA:1.50.10.20 (GENE3D) |
| 2855 | lib_dib_Filter15_IDBA_contig_58166_minus1 | 91 | ---NA--- | 184 | | | 0 | - | PTHR23304 (PANTHER) |
| 2856 | lib_dib_Filter15_IDBA_contig_58166_minus3 | 91 | ---NA--- | 184 | | | 0 | - | no IPS match |
| 2857 | lib_dib_Filter15_IDBA_contig_58166_plus1 | 91 | ---NA--- | 184 | | | 0 | - | no IPS match |
| 2858 | lib_dib_Filter15_IDBA_contig_58252_plus2 | 21 | oocyte zinc finger protein 6-like | 184 | 4.24E-60 | 69.75% | 3 | F:binding; F:nucleic acid binding; C:intracellular | IPR007087 (PROSITE); IPR013087 (G3DSA:3.30.160.GENE3D); IPR015880 (SMART); PTHR24377 (PANTHER), PF13465 (PFAM), SSF57667 (SUPERFAMILY) |
| 2859 | lib_dib_Filter15_IDBA_contig_58264_plus3 | 49 | carboxypeptidase b-like | 184 | 5.95E-63 | 70.20% | 4 | F:peptidase activity; P:protein metabolic process; P:catabolic process; F:binding | IPR000834 (PFAM); G3DSA:3.40.630.10 (GENE3D), PTHR11705 (PANTHER), SSF53187 (SUPERFAMILY) |
| 2860 | lib_dib_Filter15_IDBA_contig_58298_minus2 | 24 | signal cub and egf-like domain-containing protein 1 | 184 | 3.05E-17 | 65.30% | 13 | P:signal transduction; C:cytoplasmic membrane-bounded vesicle; C:plasma membrane; F:calcium ion binding; P:response to external stimulus; P:response to stress; P:multicellular organismal development; P:cellular component organization; F:protein binding; C:extracellular space; P:cell differentiation; P:embryo development; P:biological_process | PTHR24046 (PANTHER), PTHR24046:SF0 (PANTHER) |
| 2861 | lib_dib_Filter15_IDBA_contig_58342_plus2 | 24 | | 184 | 0 | 100% | 1 | F:protein binding | IPR003598 (SMART); IPR003599 (SMART); IPR007110 (PROFILE); IPR013098 (PFAM); IPR013783 (G3DSA:2.60.40.GENE3D); SSF48726 (SUPERFAMILY) |
| 2862 | lib_dib_Filter15_IDBA_contig_58410_plus3 | 29 | discoidin domain-containing receptor 2 | 183 | 2.76E-52 | 77.90% | 5 | F:receptor activity; F:protein kinase activity; P:biological_process; P:cellular protein modification process; F:nucleotide binding | IPR000719 (PROFILE); IPR001245 (PFAM); IPR011009 (SUPERFAMILY); IPR020635 (SMART); G3DSA:1.10.510.10 (GENE3D), PTHR24416 (PANTHER), PTHR24416:SF64 (PANTHER), SignalP-NN(euk) (SIGNALP) |
| 2863 | lib_dib_Filter15_IDBA_contig_58420_plus3 | 17 | zinc finger protein 271-like | 183 | 2.69E-54 | 61.90% | 3 | F:binding; F:nucleic acid binding; C:intracellular | IPR007087 (PROSITE); IPR013087 (G3DSA:3.30.160.GENE3D); IPR015880 (SMART); PTHR24387 (PANTHER), PF13465 (PFAM), SSF57667 (SUPERFAMILY) |
| 2864 | lib_dib_Filter15_IDBA_contig_58434_minus3 | 34 | galectin-3-binding protein | 183 | 2.57E-26 | 61.25% | 4 | F:hydrolase activity; C:extracellular region; F:receptor activity; C:cell | IPR001190 (PRINTS); IPR017448 (SMART); G3DSA:3.10.250.10 (GENE3D), PTHR19331 (PANTHER), tmhmm (TMHMM) |
| 2865 | lib_dib_Filter15_IDBA_contig_58456_plus2 | 32 | allene oxide synthase-lipoxygenase | 183 | 4.13E-64 | 62.05% | 3 | F:catalytic activity; F:binding; P:metabolic process | IPR000907 (PANTHER); IPR013819 (PFAM); G3DSA:1.20.245.10 (GENE3D), PTHR11771:SF16 (PANTHER) |

| | | | | | | | | | | |
|---|---|---|---|---|---|---|---|---|---|---|
| 2866 | lib_dib_Filter15_IDBA_contig_58534_plus3 | 18 | agrin-like | 183 | 5.33E-24 | 57.40% | 5 | P:metabolic process; P:regulation of biological process; P:primary metabolic process; F:protein binding; P:cellular component organization | IPR001791 (PFAM); IPR008985 (SUPERFAMILY); IPR013320 (G3DSA:2.60.120.GENE3D); PTHR10574 (PANTHER), PTHR10574:SF79 (PANTHER), tmhmm (TMHMM) | |
| 2867 | lib_dib_Filter15_IDBA_contig_58598_minus3 | 45 | fibroblast growth factor receptor 3- partial | 212 | 5.36E-39 | 74.40% | 26 | C:cell; P:anatomical structure morphogenesis; P:cell proliferation; P:multicellular organismal development; F:protein binding; P:regulation of biological process; P:cell differentiation; P:signal transduction; P:cellular protein modification process; P:embryo development; P:biological_process; P:growth; P:reproduction; P:cellular component organization; F:receptor activity; F:protein kinase activity; ; C:plasma membrane; C:cellular_component; F:nucleotide binding; P:cell-cell signaling; P:cell cycle; C:nucleus; C:cytoplasm; P:response to endogenous stimulus; C:extracellular region | IPR000719 (PROFILE); IPR001245 (PRINTS); IPR011009 (SUPERFAMILY); IPR020635 (SMART); G3DSA:1.10.510.10 (GENE3D), PTHR24416 (PANTHER), tmhmm (TMHMM) | |
| 2868 | lib_dib_Filter15_IDBA_contig_58615_plus1 | 126 | thioredoxin 2 | 287 | 1.56E-43 | 69.25% | 14 | P:response to stress; F:catalytic activity; P:biological_process; P:response to endogenous stimulus; C:mitochondrion; C:nucleolus; P:response to external stimulus; P:cell communication; C:cell; F:protein binding; P:metabolic process; F:electron carrier activity; P:cellular homeostasis; P:regulation of biological process | IPR005746 (PRINTS); IPR012336 (G3DSA:3.40.30.GENE3D); IPR013766 (PFAM); IPR017937 (PROSITE); PTHR10438:SF13 (PANTHER) | |
| 2869 | lib_dib_Filter15_IDBA_contig_58625_minus2 | 1083 | clotting factor b-like | 611 | 4.11E-48 | 55.05% | 3 | F:peptidase activity; P:protein metabolic process; P:catabolic process | IPR001254 (PFAM); IPR001314 (PRINTS); IPR009003 (SUPERFAMILY); IPR018114 (PROSITE); G3DSA:2.40.10.10 (GENE3D), PTHR24265 (PANTHER), SignalP-NN(euk) (SIGNALP), tmhmm (TMHMM) | yes |
| 2870 | lib_dib_Filter15_IDBA_contig_58626_minus2 | 1170 | clotting factor b-like | 664 | 7.96E-48 | 55.10% | 3 | F:peptidase activity; P:protein metabolic process; P:catabolic process | IPR001254 (PFAM); IPR001314 (PRINTS); IPR009003 (SUPERFAMILY); IPR018114 (PROSITE); G3DSA:2.40.10.10 (GENE3D), PTHR24265 (PANTHER), SignalP-NN(euk) (SIGNALP), tmhmm (TMHMM) | yes |
| 2871 | lib_dib_Filter15_IDBA_contig_58635_minus3 | 32 | a disintegrin and metalloproteinase with thrombospondin motifs 3 | 204 | 2.21E-15 | 51.95% | 1 | F:hydrolase activity | IPR000884 (PFAM); G3DSA:2.20.100.10 (GENE3D), PTHR13723 (PANTHER) | |
| 2872 | lib_dib_Filter15_IDBA_contig_58636_minus3 | 99 | a disintegrin and metalloproteinase with thrombospondin motifs 3 | 468 | 1.48E-23 | 52.70% | 1 | F:hydrolase activity | IPR000884 (PFAM); G3DSA:2.20.100.10 (GENE3D), PTHR13723 (PANTHER) | |
| 2873 | lib_dib_Filter15_IDBA_contig_58714_plus1 | 33 | thrombospondin type-1 domain-containing protein 7a-like | 226 | 4.34E-36 | 48.45% | 2 | P:blood vessel endothelial cell migration; P:angiogenesis | IPR000884 (PFAM); G3DSA:2.20.100.10 (GENE3D), PTHR11311 (PANTHER), PTHR11311:SF2 (PANTHER) | |
| 2874 | lib_dib_Filter15_IDBA_contig_58746_plus2 | 18 | fibrinogen c domain-containing protein 1-like | 183 | 1.76E-07 | 62.55% | 4 | P:biological_process; P:lipid metabolic process; F:binding; P:regulation of biological process | IPR002181 (PFAM); IPR014716 (G3DSA:3.90.215.GENE3D); PTHR19143 (PANTHER) | |
| 2875 | lib_dib_Filter15_IDBA_contig_58767_plus1 | 18 | variable lymphocyte receptor a | 183 | 1.70E-10 | 48.50% | 1 | F:protein binding | IPR001611 (PFAM); IPR003591 (SMART); G3DSA:3.80.10.10 (GENE3D), PTHR24367 (PANTHER), PF13855 (PFAM), SSF52058 (SUPERFAMILY) | |

| | | | | | | | | | | |
|---|---|---|---|---|---|---|---|---|---|---|
| 2876 | lib_dib_Filter15_IDBA_contig_58777_minus1 | 20 | agrin | 183 | 1.20E-48 | 58.00% | 2 | P:multicellular organismal development; F:protein binding | IPR000742 (PFAM); IPR001791 (PFAM); IPR008985 (SUPERFAMILY); IPR013032 (PROSITE); IPR013320 (G3DSA:2.60.120.GENE3D); G3DSA:2.10.25.10 (GENE3D), PTHR10574 (PANTHER), SSF57196 (SUPERFAMILY) | |
| 2877 | lib_dib_Filter15_IDBA_contig_58805_minus3 | 42 | beta-hexosaminidase | 245 | 7.54E-40 | 57.90% | 3 | F:hydrolase activity; F:binding; P:carbohydrate metabolic process | IPR013781 (G3DSA:3.20.20.GENE3D); IPR013812 (G3DSA:2.60.40.GENE3D); IPR014756 (SUPERFAMILY); IPR015883 (PFAM); IPR017853 (SUPERFAMILY); PTHR22600 (PANTHER) | |
| 2878 | lib_dib_Filter15_IDBA_contig_58836_plus2 | 22 | von willebrand factor type egf and pentraxin domain-containing protein 1 | 182 | 4.55E-14 | 40.55% | 7 | F:calcium ion binding; P:cell adhesion; F:carbohydrate binding; C:apical plasma membrane; F:Notch binding; P:negative regulation of Notch signaling pathway; P:regulation of tube architecture, open tracheal system | IPR000436 (PFAM); G3DSA:2.10.70.10 (GENE3D), PTHR19325 (PANTHER) | |
| 2879 | lib_dib_Filter15_IDBA_contig_58893_plus2 | 46 | 3-oxoacyl-acyl-carrier-protein reductase | 182 | 3.09E-74 | 79.45% | 3 | F:catalytic activity; P:metabolic process; F:nucleotide binding | IPR002198 (PRINTS); IPR002347 (PRINTS); IPR016040 (G3DSA:3.40.50.GENE3D); IPR020904 (PROSITE); PTHR24322 (PANTHER), PTHR24322:SF0 (PANTHER), SignalP-NN(euk) (SIGNALP), SSF51735 (SUPERFAMILY) | yes |
| 2880 | lib_dib_Filter15_IDBA_contig_58896_plus3 | 39 | discoidin domain-containing receptor 2 | 182 | 4.87E-92 | 80.30% | 14 | P:multicellular organismal development; P:biological_process; P:regulation of biological process; ; P:metabolic process; P:anatomical structure morphogenesis; P:growth; P:cellular component organization; P:cell differentiation; F:protein binding; P:cell proliferation; C:plasma membrane; P:reproduction; F:nucleotide binding | IPR000719 (PROFILE); IPR001245 (PRINTS); IPR002290 (SMART); IPR008266 (PROSITE); IPR011009 (SUPERFAMILY); IPR020635 (SMART); G3DSA:1.10.510.10 (GENE3D), G3DSA:3.30.200.20 (GENE3D), PTHR24416 (PANTHER), PTHR24416:SF65 (PANTHER) | |
| 2881 | lib_dib_Filter15_IDBA_contig_58915_minus2 | 20 | exostosin-1 | 182 | 2.16E-72 | 79.85% | 11 | P:anatomical structure morphogenesis; P:cellular component organization; P:cell differentiation; P:multicellular organismal development; P:biosynthetic process; P:carbohydrate metabolic process; P:embryo development; C:endoplasmic reticulum; F:protein binding; F:transferase activity; C:Golgi apparatus | IPR015338 (PFAM); G3DSA:3.90.550.10 (GENE3D), PTHR11062 (PANTHER), PTHR11062:SF7 (PANTHER), SSF53448 (SUPERFAMILY) | |
| 2882 | lib_dib_Filter15_IDBA_contig_58946_plus2 | 12 | aldo-keto reductase | 182 | 1.78E-50 | 65.90% | 3 | P:biological_process; F:catalytic activity; P:metabolic process | IPR001395 (PANTHER); IPR018170 (PROSITE); IPR020471 (PRINTS); IPR023210 (G3DSA:3.20.20.GENE3D); PTHR11732:SF34 (PANTHER) | |
| 2883 | lib_dib_Filter15_IDBA_contig_59079_minus3 | 171 | peroxisomal sarcosine oxidase | 416 | 4.93E-105 | 63.05% | 3 | F:catalytic activity; C:peroxisome; P:metabolic process | IPR006076 (PFAM); G3DSA:3.30.9.10 (GENE3D), G3DSA:3.50.50.60 (GENE3D), PTHR10961 (PANTHER), PTHR10961:SF7 (PANTHER), SignalP-NN(euk) (SIGNALP), SSF51905 (SUPERFAMILY), SSF54373 (SUPERFAMILY) | |
| 2884 | lib_dib_Filter15_IDBA_contig_59163_plus3 | 40 | oocyte zinc finger protein 6-like | 181 | 2.47E-63 | 70.00% | 3 | F:binding; F:nucleic acid binding; C:intracellular | IPR007087 (PROSITE); IPR013087 (G3DSA:3.30.160.GENE3D); IPR015880 (SMART); PTHR24395 (PANTHER), PTHR24395:SF0 (PANTHER), PF13465 (PFAM), SSF57667 (SUPERFAMILY) | |
| 2885 | lib_dib_Filter15_IDBA_contig_59173_plus2 | 46 | gamma-glutamyltranspeptidase 1 | 181 | 2.69E-40 | 61.90% | 2 | F:transferase activity; | IPR000101 (PRINTS); SSF56235 (SUPERFAMILY) | |

| | | | | | | | | | | |
|---|---|---|---|---|---|---|---|---|---|---|
| 2886 | lib_dib_Filter15_IDBA_contig_5919_minus1 | 1251 | calumenin isoform 2 | 1168 | 1.44E-106 | 71.30% | 14 | P:cellular component organization; P:anatomical structure morphogenesis; P:response to external stimulus; P:cell differentiation; P:response to stress; P:multicellular organismal development; F:calcium ion binding; F:protein binding; F:enzyme regulator activity; P:biological_process; C:cell; C:endoplasmic reticulum; P:transport; C:Golgi apparatus | IPR002048 (SMART); IPR011992 (G3DSA:1.10.238.GENE3D); IPR018247 (PROSITE); IPR027241 (PTHR10827:PANTHER); PTHR10827 (PANTHER), SignalP-NN(euk) (SIGNALP), tmhmm (TMHMM), SSF47473 (SUPERFAMILY) | |
| 2887 | lib_dib_Filter15_IDBA_contig_59277_minus2 | 123 | sulfhydryl oxidase 1 | 329 | 3.59E-35 | 48.00% | 5 | P:metabolic process; F:electron carrier activity; F:catalytic activity; P:cellular homeostasis; P:regulation of biological process | IPR005746 (PRINTS); IPR012336 (G3DSA:3.40.30.GENE3D); IPR013766 (PFAM); IPR017937 (PROSITE); PTHR22897 (PANTHER), SignalP-NN(euk) (SIGNALP) | yes |
| 2888 | lib_dib_Filter15_IDBA_contig_59292_plus2 | 21 | multiple epidermal growth factor-like domains protein 6-like | 181 | 3.48E-66 | 66.20% | 2 | F:protein binding; F:calcium ion binding | IPR000152 (PROSITE); IPR000742 (SMART); IPR001881 (PFAM); IPR013032 (PROSITE); IPR018097 (PROSITE); IPR026823 (PFAM); G3DSA:2.10.25.10 (GENE3D), PTHR24035 (PANTHER), PTHR24035:SF11 (PANTHER), SSF57196 (SUPERFAMILY) | |
| 2889 | lib_dib_Filter15_IDBA_contig_59331_plus2 | 51 | deoxyribonuclease i | 226 | 1.80E-39 | 66.95% | 3 | F:nuclease activity; P:DNA metabolic process; P:catabolic process | IPR005135 (PFAM); IPR008185 (PRINTS); IPR016202 (PANTHER); IPR018057 (PROSITE); G3DSA:3.60.10.10 (GENE3D) | |
| 2890 | lib_dib_Filter15_IDBA_contig_59344_plus3 | 19 | glutathione peroxidase | 181 | 3.47E-35 | 63.60% | 4 | F:catalytic activity; F:antioxidant activity; P:response to stress; P:metabolic process | IPR000889 (PIR); IPR012336 (G3DSA:3.40.30.GENE3D); PTHR11592:SF0 (PANTHER), SignalP-NN(euk) (SIGNALP) | |
| 2891 | lib_dib_Filter15_IDBA_contig_59409_plus1 | 13 | cadherin egf lag seven-pass g-type receptor 1 | 181 | 1.46E-40 | 56.90% | 4 | P:signal transduction; F:calcium ion binding; P:biological_process; C:cell | IPR002126 (PRINTS); IPR015919 (SUPERFAMILY); PTHR24027 (PANTHER) | |
| 2892 | lib_dib_Filter15_IDBA_contig_59425_minus1 | 26 | fibropellin- partial | 181 | 3.35E-41 | 64.05% | 2 | F:protein binding; F:calcium ion binding | IPR000152 (PROSITE); IPR000742 (PFAM); IPR001881 (SMART); IPR013032 (PROSITE); IPR013320 (G3DSA:2.60.120.GENE3D); IPR018097 (PROSITE); PR00010 (PRINTS), G3DSA:2.10.25.10 (GENE3D), PTHR24048 (PANTHER), PTHR24048:SF15 (PANTHER), SSF57196 (SUPERFAMILY) | |
| 2893 | lib_dib_Filter15_IDBA_contig_59439_minus3 | 29 | von willebrand factor a domain-containing protein 8 | 180 | 2.27E-79 | 78.70% | 4 | F:hydrolase activity; P:nucleobase-containing compound metabolic process; P:catabolic process; F:nucleotide binding | PTHR21610 (PANTHER), PTHR21610:SF2 (PANTHER) | |
| 2894 | lib_dib_Filter15_IDBA_contig_59524_minus3 | 51 | fibroblast growth factor receptor-like 1 | 263 | 5.37E-34 | 75.85% | 16 | P:multicellular organismal development; P:anatomical structure morphogenesis; C:organelle; C:intracellular; P:regulation of biological process; P:cell growth; F:receptor activity; P:cell proliferation; F:carbohydrate binding; P:cellular protein modification process; C:cell; P:signal transduction; F:protein kinase activity; F:protein binding; C:plasma membrane; C:cytoplasm | IPR003598 (SMART); IPR003599 (SMART); IPR007110 (PROFILE); IPR013098 (PFAM); IPR013783 (G3DSA:2.60.40.GENE3D); PTHR19890 (PANTHER), PTHR19890:SF4 (PANTHER), tmhmm (TMHMM), SSF48726 (SUPERFAMILY) | |
| 2895 | lib_dib_Filter15_IDBA_contig_59566_minus3 | 32 | ---NA--- | 207 | | | 0 | - | no IPS match | |
| 2896 | lib_dib_Filter15_IDBA_contig_59566_plus1 | 32 | acetylcholinesterase collagenic tail peptide | 208 | 5.70E-61 | 57.15% | 3 | F:carbohydrate binding; C:extracellular region; C:proteinaceous extracellular matrix | IPR008160 (PFAM); IPR011936 (TIGRFAMs); PTHR24023 (PANTHER), PTHR24023:SF96 (PANTHER) | |
| 2897 | lib_dib_Filter15_IDBA_contig_59567_minus3 | 68 | ---NA--- | 331 | | | 0 | - | no IPS match | |
| 2898 | lib_dib_Filter15_IDBA_contig_59567_plus2 | 68 | acetylcholinesterase collagenic tail peptide-like | 332 | 3.11E-54 | 56.80% | 3 | F:carbohydrate binding; C:extracellular region; C:proteinaceous extracellular matrix | IPR008160 (PFAM); IPR011936 (TIGRFAMs); PTHR24023 (PANTHER) | |

| | | | | | | | | | | |
|---|---|---|---|---|---|---|---|---|---|---|
| 2899 | lib_dib_Filter15_IDBA_contig_59625_plus3 | 20 | retinol dehydrogenase 11 | 180 | 5.45E-32 | 64.15% | 3 | P:metabolic process; F:catalytic activity; F:nucleotide binding | IPR002198 (PFAM); IPR002347 (PRINTS); IPR016040 (G3DSA:3.40.50.GENE3D); PTHR24320 (PANTHER), PTHR24320:SF0 (PANTHER), SignalP-NN(euk) (SIGNALP), tmhmm (TMHMM), SSF51735 (SUPERFAMILY) | |
| 2900 | lib_dib_Filter15_IDBA_contig_59698_minus3 | 25 | basement membrane-specific heparan sulfate proteoglycan core | 180 | 5.19E-16 | 44.25% | 1 | F:protein binding | IPR000742 (PFAM); IPR001791 (PROFILE); IPR008985 (SUPERFAMILY); IPR013032 (PROSITE); IPR013320 (G3DSA:2.60.120.GENE3D); G3DSA:2.10.25.10 (GENE3D), PTHR10574 (PANTHER), SSF57196 (SUPERFAMILY) | |
| 2901 | lib_dib_Filter15_IDBA_contig_59714_minus1 | 7 | heat shock 70 kda protein 13 | 103 | 1.19E-28 | 72.10% | 1 | F:nucleotide binding | IPR013126 (PRINTS); IPR018181 (PROSITE); G3DSA:3.30.420.40 (GENE3D), PTHR19375 (PANTHER), PTHR19375:SF11 (PANTHER), SSF53067 (SUPERFAMILY) | |
| 2902 | lib_dib_Filter15_IDBA_contig_59720_minus2 | 13 | deleted in autism protein 1 | 180 | 2.55E-24 | 49.85% | 1 | C:Golgi apparatus | IPR020519 (PRODOM); PTHR32073 (PANTHER), SignalP-NN(euk) (SIGNALP) | yes |
| 2903 | lib_dib_Filter15_IDBA_contig_59722_minus3 | 974 | bone morphogenetic protein 1 | 248 | 2.25E-16 | 53.30% | 5 | P:anatomical structure morphogenesis; P:cell differentiation; P:multicellular organismal development; P:signal transduction; F:protein binding | IPR000859 (G3DSA:2.60.120.GENE3D); PTHR10127 (PANTHER), PTHR10127:SF436 (PANTHER), tmhmm (TMHMM) | |
| 2904 | lib_dib_Filter15_IDBA_contig_59748_minus3 | 126 | dnaj homolog subfamily c member 5-like | 366 | 3.08E-53 | 80.90% | 4 | P:protein metabolic process; C:plasma membrane; C:cytoplasmic membrane-bounded vesicle; F:protein binding | IPR001623 (PRINTS); IPR018253 (PROSITE); PTHR24078 (PANTHER), PTHR24078:SF7 (PANTHER), tmhmm (TMHMM) | |
| 2905 | lib_dib_Filter15_IDBA_contig_59774_minus3 | 91 | zinc finger protein 680 | 320 | 1.20E-54 | 52.60% | 3 | C:nucleus; F:binding; F:nucleic acid binding | IPR007087 (PFAM); IPR013087 (G3DSA:3.30.160.GENE3D); IPR015880 (SMART); PTHR24375 (PANTHER), PTHR24375:SF0 (PANTHER), PF12874 (PFAM), PF13465 (PFAM), PF13894 (PFAM), PF13912 (PFAM), SSF57667 (SUPERFAMILY) | |
| 2906 | lib_dib_Filter15_IDBA_contig_59784_minus2 | 24 | gelsolin-like protein 2-like | 180 | 2.58E-33 | 57.25% | 1 | F:actin binding | IPR007122 (PRINTS); IPR007123 (PFAM); G3DSA:3.40.20.10 (GENE3D), PTHR11977:SF19 (PANTHER), SSF55753 (SUPERFAMILY) | |
| 2907 | lib_dib_Filter15_IDBA_contig_59918_minus3 | 9291 | serine protease inhibitor dipetalogastin | 680 | 2.33E-40 | 47.50% | 1 | F:protein binding | IPR002350 (PFAM); G3DSA:3.30.60.30 (GENE3D), PTHR10913 (PANTHER), SignalP-NN(euk) (SIGNALP), tmhmm (TMHMM), SSF100895 (SUPERFAMILY) | |
| 2908 | lib_dib_Filter15_IDBA_contig_59952_minus1 | 11275 | prostasin precursor | 355 | 9.32E-58 | 53.00% | 4 | P:response to endogenous stimulus; F:peptidase activity; P:protein metabolic process; P:catabolic process | IPR001254 (PFAM); IPR001314 (PRINTS); IPR009003 (SUPERFAMILY); IPR018114 (PROSITE); G3DSA:2.40.10.10 (GENE3D), PTHR24268 (PANTHER), SignalP-NN(euk) (SIGNALP) | yes |
| 2909 | lib_dib_Filter15_IDBA_contig_59953_minus1 | 10747 | prostasin precursor | 350 | 1.38E-57 | 53.20% | 4 | P:response to endogenous stimulus; F:peptidase activity; P:protein metabolic process; P:catabolic process | IPR001254 (PFAM); IPR001314 (PRINTS); IPR009003 (SUPERFAMILY); IPR018114 (PROSITE); G3DSA:2.40.10.10 (GENE3D), PTHR24268 (PANTHER), SignalP-NN(euk) (SIGNALP) | yes |
| 2910 | lib_dib_Filter15_IDBA_contig_59954_minus3 | 1182 | 36 precursor | 179 | 6.14E-17 | 53.05% | 3 | F:peptidase activity; P:protein metabolic process; P:catabolic process | IPR001254 (PFAM); IPR001314 (PRINTS); IPR009003 (SUPERFAMILY); IPR018114 (PROSITE); G3DSA:2.40.10.10 (GENE3D), PTHR24265 (PANTHER), SignalP-NN(euk) (SIGNALP) | yes |

| | | | | | | | | | | |
|---|---|---|---|---|---|---|---|---|---|---|
| 2911 | lib_dib_Filter15_IDBA_contig_60030_minus3 | 23 | xtld protein | 179 | 5.56E-17 | 53.75% | 2 | F:protein binding; F:calcium ion binding | IPR000152 (PROSITE); IPR000742 (PFAM); IPR000859 (G3DSA:2.60.120.GENE3D); IPR001881 (SMART); IPR013032 (PROSITE); IPR018097 (PROSITE); PR00010 (PRINTS), G3DSA:2.10.25.10 (GENE3D), PTHR24044 (PANTHER), SSF57196 (SUPERFAMILY) | |
| 2912 | lib_dib_Filter15_IDBA_contig_60109_minus2 | 32 | adenosine deaminase-like protein | 205 | 6.75E-67 | 69.80% | 4 | F:hydrolase activity; P:nucleobase-containing compound metabolic process; P:catabolic process; P:biosynthetic process | IPR001365 (PFAM); G3DSA:3.20.20.140 (GENE3D), PTHR11409 (PANTHER), PTHR11409:SF21 (PANTHER), SSF51556 (SUPERFAMILY) | |
| 2913 | lib_dib_Filter15_IDBA_contig_60132_plus1 | 17 | low quality protein: prolow-density lipoprotein receptor-related protein 1 | 179 | 4.02E-25 | 59.15% | 6 | P:signal transduction; P:transport; P:regulation of biological process; C:intracellular; C:cell; F:protein binding | IPR002172 (G3DSA:4.10.400.GENE3D); IPR023415 (PROSITE); PR00261 (PRINTS), PTHR10529 (PANTHER) | |
| 2914 | lib_dib_Filter15_IDBA_contig_60172_plus3 | 6065 | kazal-type serine protease inhibitor domain-containing protein 1-like | 490 | 4.01E-89 | 59.90% | 5 | C:proteinaceous extracellular matrix; P:cellular component organization; P:regulation of biological process; P:cell growth; F:protein binding | IPR000867 (PFAM); IPR002350 (PFAM); IPR003598 (SMART); IPR003599 (SMART); IPR007110 (PROFILE); IPR011390 (PANTHER); IPR013098 (PFAM); IPR013783 (G3DSA:2.60.40.GENE3D); G3DSA:3.30.60.30 (GENE3D), PTHR14186:SF7 (PANTHER), SSF100895 (SUPERFAMILY), SSF48726 (SUPERFAMILY) | |
| 2915 | lib_dib_Filter15_IDBA_contig_60241_plus2 | 189 | upf0764 protein c16orf89 homolog | 350 | 5.30E-50 | 47.35% | 1 | C:extracellular region | SignalP-NN(euk) (SIGNALP) | yes |
| 2916 | lib_dib_Filter15_IDBA_contig_60264_minus1 | 92 | endothelin-converting enzyme 1-like | 296 | 1.41E-34 | 50.95% | 4 | C:cell; F:peptidase activity; P:protein metabolic process; P:catabolic process | IPR000718 (PANTHER); IPR008753 (PFAM); G3DSA:1.10.1380.10 (GENE3D), SignalP-NN(euk) (SIGNALP), tmhmm (TMHMM), SSF55486 (SUPERFAMILY) | |
| 2917 | lib_dib_Filter15_IDBA_contig_60278_plus1 | 22 | galactose-specific c-type | 179 | 3.92E-06 | 48.45% | 1 | F:carbohydrate binding | IPR001304 (PFAM); IPR016186 (G3DSA:3.10.100.GENE3D); IPR016187 (SUPERFAMILY); PTHR22802 (PANTHER) | |
| 2918 | lib_dib_Filter15_IDBA_contig_60336_plus3 | 16 | von willebrand factor type egf and pentraxin domain-containing protein 1 | 178 | 2.90E-14 | 43.25% | 12 | F:calcium ion binding; F:chromatin binding; F:transferase activity; F:carbohydrate binding; P:phosphorylation; F:protein kinase activity; P:protein phosphorylation; F:nucleotide binding; F:ATP binding; F:kinase activity; F:protein tyrosine kinase activity; F:transferase activity, transferring phosphorus-containing groups | IPR000436 (PFAM); G3DSA:2.10.70.10 (GENE3D), PTHR19325 (PANTHER) | |
| 2919 | lib_dib_Filter15_IDBA_contig_60344_plus3 | 16 | slit homolog 2 protein isoform 2 | 178 | 4.23E-19 | 49.75% | 14 | P:anatomical structure morphogenesis; P:cellular component organization; P:cell differentiation; P:multicellular organismal development; P:behavior; P:response to external stimulus; C:cell; P:regulation of biological process; P:metabolic process; P:signal transduction; F:protein binding; P:biological_process; P:cell growth; C:extracellular region | IPR000483 (SMART); IPR001611 (PROFILE); G3DSA:3.80.10.10 (GENE3D), PTHR24373 (PANTHER), PTHR24373:SF8 (PANTHER), PF13855 (PFAM), SSF52058 (SUPERFAMILY) | |
| 2920 | lib_dib_Filter15_IDBA_contig_60447_plus2 | 17 | protocadherin fat 4 | 178 | 2.74E-29 | 57.15% | 3 | F:calcium ion binding; P:biological_process; C:cell | IPR002126 (PRINTS); IPR015919 (SUPERFAMILY); PTHR24027 (PANTHER) | |
| 2921 | lib_dib_Filter15_IDBA_contig_60455_minus1 | 2118 | pancreatic lipase-related protein 1 | 410 | 2.87E-64 | 51.45% | 2 | F:catalytic activity; P:lipid metabolic process | IPR000734 (PRINTS); IPR013818 (PFAM); G3DSA:3.40.50.1820 (GENE3D), SignalP-NN(euk) (SIGNALP), SSF53474 (SUPERFAMILY) | yes |
| 2922 | lib_dib_Filter15_IDBA_contig_60503_plus3 | 20 | plasma alpha-l-fucosidase | 178 | 2.37E-79 | 74.95% | 2 | F:hydrolase activity; P:carbohydrate metabolic process | IPR000933 (PANTHER); IPR013781 (G3DSA:3.20.20.GENE3D); IPR016286 (PRINTS); IPR017853 (SUPERFAMILY); PTHR10030:SF3 (PANTHER) | |
| 2923 | lib_dib_Filter15_IDBA_contig_60540_plus3 | 17 | fmrfamide partial | 177 | 1.56E-12 | 65.10% | 1 | P:signal transduction | IPR002544 (PFAM); PTHR20986 (PANTHER) | |

| | | | | | | | | | |
|---|---|---|---|---|---|---|---|---|---|
| 2924 | lib_dib_Filter15_IDBA_contig_60575_minus2 | 28 | carboxypeptidase d | 178 | 1.75E-43 | 68.45% | 5 | C:cell; F:peptidase activity; P:protein metabolic process; P:catabolic process; F:binding | IPR000834 (PRINTS); G3DSA:3.40.630.10 (GENE3D), PTHR11532 (PANTHER), SignalP-NN(euk) (SIGNALP), SSF53187 (SUPERFAMILY) | yes |
| 2925 | lib_dib_Filter15_IDBA_contig_60582_plus3 | 19 | carboxypeptidase d | 177 | 3.80E-49 | 66.60% | 4 | F:peptidase activity; P:protein metabolic process; P:catabolic process; F:binding | IPR000834 (PRINTS); G3DSA:3.40.630.10 (GENE3D), PTHR11532 (PANTHER), SSF53187 (SUPERFAMILY) | |
| 2926 | lib_dib_Filter15_IDBA_contig_60622_plus1 | 17 | polypeptide n-acetylgalactosaminyltransferase 10 | 178 | 1.35E-43 | 70.50% | 6 | C:Golgi apparatus; P:biosynthetic process; P:cellular protein modification process; P:carbohydrate metabolic process; F:binding; F:transferase activity | IPR001173 (PFAM); G3DSA:3.90.550.10 (GENE3D), PTHR11675 (PANTHER), SSF53448 (SUPERFAMILY) | |
| 2927 | lib_dib_Filter15_IDBA_contig_60659_minus3 | 14 | protocadherin-19 isoform 1 | 177 | 1.37E-49 | 65.35% | 3 | F:calcium ion binding; P:biological_process; C:plasma membrane | IPR002126 (PRINTS); IPR015919 (SUPERFAMILY); IPR020894 (PROSITE); PTHR24028 (PANTHER) | |
| 2928 | lib_dib_Filter15_IDBA_contig_60669_plus1 | 41 | thrombospondin type-1 domain-containing protein 4-like | 199 | 4.58E-35 | 59.85% | 4 | F:protein binding; P:regulation of biological process; P:cell death; F:peptidase activity | IPR000884 (PFAM); IPR010909 (PFAM); G3DSA:2.20.100.10 (GENE3D), PTHR13723 (PANTHER), PTHR13723:SF16 (PANTHER) | |
| 2929 | lib_dib_Filter15_IDBA_contig_60703_minus2 | 28 | zinc finger transcription factor e2s-vp64 | 233 | 1.23E-42 | 62.25% | 3 | F:binding; F:nucleic acid binding; C:intracellular | IPR007087 (PROSITE); IPR013087 (G3DSA:3.30.160.GENE3D); IPR015880 (SMART); PTHR24377 (PANTHER), PTHR24377:SF0 (PANTHER), PF13465 (PFAM), PF13894 (PFAM), SSF57667 (SUPERFAMILY) | |
| 2930 | lib_dib_Filter15_IDBA_contig_60753_minus1 | 47 | tryptase alpha beta-1 | 178 | 6.26E-18 | 73.90% | 5 | C:extracellular region; P:protein metabolic process; P:catabolic process; F:carbohydrate binding; F:peptidase activity | IPR001254 (PFAM); IPR009003 (SUPERFAMILY); IPR018114 (PROSITE); G3DSA:2.40.10.10 (GENE3D), PTHR24256 (PANTHER), SignalP-NN(euk) (SIGNALP), tmhmm (TMHMM) | |
| 2931 | lib_dib_Filter15_IDBA_contig_60767_plus1 | 15 | neural-cadherin-like | 178 | 4.23E-75 | 59.75% | 3 | F:calcium ion binding; P:biological_process; C:plasma membrane | IPR002126 (PRINTS); IPR015919 (SUPERFAMILY); IPR020894 (PROSITE); PTHR24027 (PANTHER), PTHR24027:SF66 (PANTHER) | |
| 2932 | lib_dib_Filter15_IDBA_contig_60809_plus1 | 89 | von willebrand factor type egf and pentraxin domain-containing protein 1 | 283 | 2.92E-08 | 48.35% | 1 | F:binding | IPR006585 (SMART); IPR008979 (SUPERFAMILY); G3DSA:2.60.120.260 (GENE3D), PTHR19277 (PANTHER), PTHR19277:SF6 (PANTHER) | |
| 2933 | lib_dib_Filter15_IDBA_contig_60830_plus2 | 18 | serine protease mitochondrial-like | 177 | 3.40E-32 | 69.65% | 2 | F:catalytic activity; F:protein binding | IPR001478 (PFAM); G3DSA:2.30.42.10 (GENE3D), PTHR22939 (PANTHER), PTHR22939:SF12 (PANTHER) | |
| 2934 | lib_dib_Filter15_IDBA_contig_60845_minus3 | 29 | neural cell adhesion molecule | 177 | 1.50E-32 | 57.35% | 7 | P:regulation of biological process; ; F:receptor activity; F:protein binding; P:anatomical structure morphogenesis; P:cell differentiation; P:multicellular organismal development | IPR003598 (SMART); IPR003599 (SMART); IPR007110 (PROFILE); IPR013098 (PFAM); IPR013783 (G3DSA:2.60.40.GENE3D); PTHR10489 (PANTHER), PF13895 (PFAM), SSF48726 (SUPERFAMILY) | |
| 2935 | lib_dib_Filter15_IDBA_contig_60911_plus3 | 18 | cub and sushi domain-containing protein 2 | 176 | 1.26E-17 | 53.40% | 3 | C:integral to membrane; C:plasma membrane; F:calcium ion binding | IPR000436 (PFAM); IPR000859 (G3DSA:2.60.120.GENE3D); G3DSA:2.10.70.10 (GENE3D), PTHR19325 (PANTHER) | |
| 2936 | lib_dib_Filter15_IDBA_contig_61007_plus3 | 1522 | subfamily s1a unassigned peptidase (s01 family) | 362 | 3.83E-39 | 54.60% | 4 | P:response to endogenous stimulus; F:peptidase activity; P:protein metabolic process; P:catabolic process | IPR001254 (PFAM); IPR001314 (PRINTS); IPR009003 (SUPERFAMILY); G3DSA:2.40.10.10 (GENE3D), PTHR24265 (PANTHER) | |
| 2937 | lib_dib_Filter15_IDBA_contig_61008_plus3 | 2194 | brain-specific serine protease 4 | 451 | 4.75E-51 | 54.25% | 3 | F:peptidase activity; P:protein metabolic process; P:catabolic process | IPR001254 (PFAM); IPR001314 (PRINTS); IPR009003 (SUPERFAMILY); IPR018114 (PROSITE); G3DSA:2.40.10.10 (GENE3D), PTHR24256 (PANTHER), SignalP-NN(euk) (SIGNALP), tmhmm (TMHMM) | yes |
| 2938 | lib_dib_Filter15_IDBA_contig_61009_plus2 | 5706 | serine protease 30-like | 426 | 2.40E-52 | 54.50% | 3 | F:peptidase activity; P:protein metabolic process; P:catabolic process | IPR001254 (PFAM); IPR001314 (PRINTS); IPR009003 (SUPERFAMILY); G3DSA:2.40.10.10 (GENE3D), PTHR24265 (PANTHER), SignalP-NN(euk) (SIGNALP), tmhmm (TMHMM) | yes |
| 2939 | lib_dib_Filter15_IDBA_contig_61129_minus2 | 21 | von willebrand factor a domain-containing protein 8 | 205 | 2.69E-45 | 72.35% | 2 | F:hydrolase activity; F:nucleotide binding | PTHR21610 (PANTHER), PTHR21610:SF2 (PANTHER) | |
| 2940 | lib_dib_Filter15_IDBA_contig_6113_minus1 | 1797 | low quality protein: cubilin-like | 368 | 9.43E-31 | 46.95% | 1 | F:catalytic activity | IPR000859 (G3DSA:2.60.120.GENE3D); PTHR10127 (PANTHER) | |

| | | | | | | | | | | |
|---|---|---|---|---|---|---|---|---|---|---|
| 2941 | lib_dib_Filter15_IDBA_contig_61138_minus2 | 21 | zinc finger protein 569 | 176 | 5.10E-24 | 59.10% | 3 | F:binding; F:nucleic acid binding; C:intracellular | IPR007087 (PFAM); IPR013087 (G3DSA:3.30.160.GENE3D); IPR015880 (SMART); PTHR24402 (PANTHER), PTHR24402:SF54 (PANTHER), PF13465 (PFAM), SSF57667 (SUPERFAMILY) | |
| 2942 | lib_dib_Filter15_IDBA_contig_61145_plus2 | 50 | zinc finger protein 271 (zinc finger protein 7) (zinc finger protein znfphex133) (epstein-barr virus-induced zinc finger protein) (znf-eb) (ct-zfp48) (zinc finger protein | 230 | 2.08E-25 | 53.60% | 3 | F:binding; F:nucleic acid binding; C:intracellular | IPR007087 (PROSITE); IPR013087 (G3DSA:3.30.160.GENE3D); IPR015880 (SMART); PTHR24375 (PANTHER), PTHR24375:SF0 (PANTHER), PF13465 (PFAM), SignalP-NN(euk) (SIGNALP), SSF57667 (SUPERFAMILY) | yes |
| 2943 | lib_dib_Filter15_IDBA_contig_6117_plus1 | 456 | ankyrin unc44 | 723 | 2.81E-12 | 61.05% | 1 | P:biological_process | no IPS match | |
| 2944 | lib_dib_Filter15_IDBA_contig_61249_minus2 | 71 | epididymal sperm-binding protein 1-like | 309 | 2.97E-32 | 46.10% | 2 | F:carbohydrate binding; C:collagen | IPR000562 (G3DSA:2.10.10.GENE3D); IPR013806 (SUPERFAMILY); PTHR22918 (PANTHER), PTHR22918:SF0 (PANTHER), SignalP-NN(euk) (SIGNALP), tmhmm (TMHMM) | yes |
| 2945 | lib_dib_Filter15_IDBA_contig_61261_minus1 | 16 | hypothetical protein BRAFLDRAFT_80519 | 176 | 4.34E-21 | 66.85% | 3 | F:binding; F:nucleic acid binding; C:intracellular | IPR007087 (PROSITE); IPR013087 (G3DSA:3.30.160.GENE3D); IPR015880 (SMART); PTHR24381 (PANTHER), PTHR24381:SF76 (PANTHER), PF13465 (PFAM), SSF57667 (SUPERFAMILY) | |
| 2946 | lib_dib_Filter15_IDBA_contig_61310_plus2 | 114 | nidogen and egf-like domain-containing protein partial | 364 | 7.77E-16 | 55.30% | 2 | F:protein binding; F:calcium ion binding | IPR000742 (PFAM); IPR001881 (SMART); IPR013032 (PROSITE); G3DSA:2.10.25.10 (GENE3D), PTHR24044 (PANTHER), SSF57196 (SUPERFAMILY) | |
| 2947 | lib_dib_Filter15_IDBA_contig_61322_minus2 | 23 | tyrosine-protein kinase btk29a | 176 | 9.24E-61 | 71.25% | 21 | P:multicellular organismal development; P:behavior; P:anatomical structure morphogenesis; F:protein kinase activity; P:reproduction; P:regulation of biological process; P:cytoskeleton organization; C:cytoplasm; C:extracellular region; P:embryo development; P:signal transduction; P:response to stress; P:cellular protein modification process; P:DNA metabolic process; P:biosynthetic process; P:cell cycle; P:transport; P:cellular component organization; P:organelle organization; C:plasma membrane; F:nucleotide binding | IPR000719 (PROFILE); IPR001245 (PRINTS); IPR002290 (SMART); IPR008266 (PROSITE); IPR011009 (SUPERFAMILY); IPR020635 (SMART); G3DSA:1.10.510.10 (GENE3D), G3DSA:3.30.200.20 (GENE3D), PTHR24418 (PANTHER) | |
| 2948 | lib_dib_Filter15_IDBA_contig_61343_minus2 | 21 | protein | 176 | 3.61E-21 | 53.35% | 3 | F:receptor activity; P:signal transduction; C:cell | IPR000832 (PFAM); IPR017981 (PROFILE); IPR022343 (PRINTS); PTHR12011 (PANTHER), SignalP-NN(euk) (SIGNALP), tmhmm (TMHMM) | |
| 2949 | lib_dib_Filter15_IDBA_contig_61357_plus3 | 36 | carbohydrate sulfotransferase 9 | 299 | 5.69E-12 | 51.45% | 5 | F:transferase activity; P:metabolic process; C:cell; P:biosynthetic process; P:carbohydrate metabolic process | IPR005331 (PFAM); IPR018011 (PANTHER) | |
| 2950 | lib_dib_Filter15_IDBA_contig_61459_plus3 | 18 | zinc finger protein 484 | 175 | 8.08E-21 | 55.00% | 3 | F:binding; F:nucleic acid binding; C:intracellular | IPR007087 (PFAM); IPR013087 (G3DSA:3.30.160.GENE3D); IPR015880 (SMART); PTHR11389 (PANTHER), PTHR11389:SF381 (PANTHER), PF13465 (PFAM), SSF57667 (SUPERFAMILY) | |
| 2951 | lib_dib_Filter15_IDBA_contig_6153_minus2 | 176 | kelch-like protein 8 | 660 | 0 | 74.50% | 1 | F:protein binding | IPR000210 (SMART); IPR006652 (PFAM); IPR011333 (G3DSA:3.30.710.GENE3D); IPR011705 (PFAM); IPR013069 (PFAM); IPR015916 (G3DSA:2.130.10.GENE3D); IPR017096 (PIR); PTHR24412 (PANTHER), PTHR24412:SF33 (PANTHER), SSF117281 (SUPERFAMILY) | |

| | | | | | | | | | | |
|---|---|---|---|---|---|---|---|---|---|---|
| 2952 | lib_dib_Filter15_IDBA_contig_61601_minus3 | 21 | kazal-type serine protease inhibitor domain protein | 175 | 3.23E-44 | 60.15% | 1 | F:protein binding | IPR002350 (PFAM); G3DSA:3.30.60.30 (GENE3D), PTHR10913 (PANTHER), PTHR10913:SF21 (PANTHER), SSF100895 (SUPERFAMILY) | |
| 2953 | lib_dib_Filter15_IDBA_contig_61633_plus2 | 15 | protein o-glucosyltransferase 1-like | 175 | 2.83E-31 | 74.80% | 6 | P:biosynthetic process; P:cellular protein modification process; P:carbohydrate metabolic process; C:endoplasmic reticulum; P:signal transduction; F:transferase activity | IPR006598 (PFAM); PTHR12203 (PANTHER), PTHR12203:SF6 (PANTHER) | |
| 2954 | lib_dib_Filter15_IDBA_contig_61728_plus1 | 882 | fibrinogen gamma polypeptide | 556 | 2.22E-46 | 56.90% | 3 | F:receptor binding; C:extracellular space; P:signal transduction | IPR002181 (PFAM); IPR014715 (G3DSA:4.10.530.GENE3D); IPR014716 (G3DSA:3.90.215.GENE3D); IPR020837 (PROSITE); PTHR19143 (PANTHER), SignalP-NN(euk) (SIGNALP) | |
| 2955 | lib_dib_Filter15_IDBA_contig_61729_plus2 | 1052 | fibrinogen gamma polypeptide | 608 | 4.10E-46 | 57.05% | 3 | F:receptor binding; C:extracellular space; P:signal transduction | IPR002181 (PFAM); IPR014715 (G3DSA:4.10.530.GENE3D); IPR014716 (G3DSA:3.90.215.GENE3D); IPR020837 (PROSITE); PTHR19143 (PANTHER), SignalP-NN(euk) (SIGNALP) | yes |
| 2956 | lib_dib_Filter15_IDBA_contig_61813_minus1 | 17 | serine threonine-protein phosphatase 6 regulatory ankyrin repeat subunit a-like | 215 | 2.88E-12 | 48.70% | 1 | F:protein binding | IPR002110 (PRINTS); IPR020683 (G3DSA:1.25.40.GENE3D); PTHR24188 (PANTHER) | |
| 2957 | lib_dib_Filter15_IDBA_contig_61831_plus1 | 65 | von willebrand factor a domain containing 8 | 345 | 1.47E-130 | 72.80% | 2 | F:nucleotide binding; F:hydrolase activity | IPR011704 (PFAM); G3DSA:3.40.50.300 (GENE3D), PTHR21610 (PANTHER), PTHR21610:SF2 (PANTHER), SSF52540 (SUPERFAMILY) | |
| 2958 | lib_dib_Filter15_IDBA_contig_61840_minus1 | 20 | myom_lymst ame: full=myomodulin neuropeptides contains: ame: full=glqmlrl-amide contains: ame: full=qipmlrl-amide contains: ame: full=smsmlrl-amide contains: ame: full=slsmlrl-amide contains: ame: full=myomodulin-a ame: full=pmsmlrl-amide flags: precursor | 175 | 1.17E-15 | 70.71% | 2 | P:neuropeptide signaling pathway; C:extracellular region | no IPS match | |
| 2959 | lib_dib_Filter15_IDBA_contig_61868_plus1 | 17 | zinc finger protein 84- partial | 175 | 3.87E-49 | 67.55% | 3 | F:binding; F:nucleic acid binding; C:intracellular | IPR007087 (PROSITE); IPR013087 (G3DSA:3.30.160.GENE3D); IPR015880 (SMART); PTHR24402 (PANTHER), PF13465 (PFAM), SSF57667 (SUPERFAMILY) | |
| 2960 | lib_dib_Filter15_IDBA_contig_61878_plus1 | 572 | #NAME? | 369 | 1.68E-129 | 75.55% | 7 | C:mitochondrion; C:nucleolus; F:catalytic activity; P:catabolic process; P:lipid metabolic process; F:nucleotide binding; P:cellular component organization | IPR002347 (PRINTS); IPR016040 (G3DSA:3.40.50.GENE3D); PTHR24315 (PANTHER), PTHR24315:SF2 (PANTHER), PF13561 (PFAM), SignalP-NN(euk) (SIGNALP), tmhmm (TMHMM), SSF51735 (SUPERFAMILY) | |
| 2961 | lib_dib_Filter15_IDBA_contig_61959_plus1 | 214 | thrombospondin type-1 domain-containing protein 7a | 204 | 2.25E-15 | 57.40% | 9 | F:hydrolase activity; F:arylesterase activity; F:metal ion binding; P:cell adhesion; C:proteinaceous extracellular matrix; C:extracellular region; P:innate immune response; P:cell differentiation; C:membrane | IPR000884 (PFAM); G3DSA:2.20.100.10 (GENE3D), PTHR11311 (PANTHER) | |
| 2962 | lib_dib_Filter15_IDBA_contig_61960_plus3 | 633 | spondin-2 precursor | 480 | 1.77E-83 | 56.40% | 3 | P:biological_process; C:extracellular region; F:receptor binding | IPR000884 (PFAM); IPR009465 (PFAM); G3DSA:2.20.100.10 (GENE3D), PTHR11311 (PANTHER), PTHR11311:SF3 (PANTHER) | |
| 2963 | lib_dib_Filter15_IDBA_contig_62057_plus2 | 31 | hypothetical protein EAI_09601 | 174 | 3.90E-05 | 65.00% | 0 | - | no IPS match | |

| | | | | | | | | | |
|---|---|---|---|---|---|---|---|---|---|
| 2964 | lib_dib_Filter15_IDBA_contig_62087_minus1 | 112 | cyclin-dependent kinase 5 | 374 | 0 | 92.75% | 39 | P:anatomical structure morphogenesis; P:cellular component organization; P:cell differentiation; P:multicellular organismal development; P:regulation of biological process; ; P:behavior; P:cell cycle; P:transport; P:biological_process; C:cell; C:cytoskeleton; P:cell death; P:signal transduction; P:cellular protein modification process; P:embryo development; C:cellular_component; C:cytosol; P:cytoskeleton organization; P:response to external stimulus; P:response to stress; P:organelle organization; P:cell-cell signaling; P:cellular homeostasis; F:protein binding; C:plasma membrane; F:protein kinase activity; P:response to abiotic stimulus; P:ion transport; F:signal transducer activity; F:nucleotide binding; P:protein transport; P:cell proliferation; C:nucleus; C:protein complex; P:metabolic process; F:receptor binding; P:catabolic process; | IPR000719 (PFAM); IPR002290 (SMART); IPR008271 (PROSITE); IPR011009 (SUPERFAMILY); IPR017441 (PROSITE); IPR020635 (SMART); G3DSA:1.10.510.10 (GENE3D), G3DSA:3.30.200.20 (GENE3D), PTHR24056 (PANTHER), PTHR24056:SF134 (PANTHER) |
| 2965 | lib_dib_Filter15_IDBA_contig_62088_minus1 | 119 | cyclin-dependent kinase 5 | 397 | 0 | 92.85% | 39 | P:anatomical structure morphogenesis; P:cellular component organization; P:cell differentiation; P:multicellular organismal development; P:regulation of biological process; ; P:behavior; P:cell cycle; P:transport; P:biological_process; C:cell; C:cytoskeleton; P:cell death; P:signal transduction; P:cellular protein modification process; P:embryo development; C:cellular_component; C:cytosol; P:cytoskeleton organization; P:response to external stimulus; P:response to stress; P:organelle organization; P:cell-cell signaling; P:cellular homeostasis; F:protein binding; C:plasma membrane; F:protein kinase activity; P:response to abiotic stimulus; P:ion transport; F:signal transducer activity; F:nucleotide binding; P:protein transport; P:cell proliferation; C:nucleus; C:protein complex; P:metabolic process; F:receptor binding; P:catabolic process; | IPR000719 (PFAM); IPR002290 (SMART); IPR008271 (PROSITE); IPR011009 (SUPERFAMILY); IPR017441 (PROSITE); IPR020635 (SMART); G3DSA:1.10.510.10 (GENE3D), G3DSA:3.30.200.20 (GENE3D), PTHR24056 (PANTHER), PTHR24056:SF134 (PANTHER) |
| 2966 | lib_dib_Filter15_IDBA_contig_62102_plus2 | 13 | zinc finger | 174 | 3.23E-52 | 70.30% | 3 | F:binding; F:nucleic acid binding; C:intracellular | IPR007087 (PROSITE); IPR013087 (G3DSA:3.30.160.GENE3D); IPR015880 (SMART); PTHR11389 (PANTHER), PF13465 (PFAM), PF13909 (PFAM), SSF57667 (SUPERFAMILY) |
| 2967 | lib_dib_Filter15_IDBA_contig_62115_plus3 | 21 | glycosaminoglycan xylosylkinase | 174 | 9.92E-65 | 70.20% | 2 | F:transferase activity; C:Golgi apparatus | IPR009581 (PFAM); IPR024869 (PANTHER); PTHR12450:SF7 (PANTHER) |
| 2968 | lib_dib_Filter15_IDBA_contig_62134_plus1 | 17 | ---NA--- | 174 | | | 0 | - | no IPS match |
| 2969 | lib_dib_Filter15_IDBA_contig_62134_plus2 | 17 | ---NA--- | 174 | | | 0 | - | no IPS match |

| | | | | | | | | | |
|---|---|---|---|---|---|---|---|---|---|
| 2970 | lib_dib_Filter15_IDBA_contig_62200_minus1 | 77 | wnt1-inducible-signaling pathway protein 1 | 174 | 5.67E-11 | 52.05% | 5 | P:signal transduction; P:metabolic process; P:multicellular organismal development; F:protein binding; P:regulation of biological process | IPR000884 (PFAM); IPR006207 (PROFILE); PTHR11348 (PANTHER), PTHR11348:SF6 (PANTHER) |
| 2971 | lib_dib_Filter15_IDBA_contig_62215_minus2 | 22 | collagen alpha-3 chain | 174 | 9.64E-29 | 57.10% | 1 | F:protein binding | IPR002035 (PFAM); PR00453 (PRINTS), G3DSA:3.40.50.410 (GENE3D), PTHR22992 (PANTHER), SSF53300 (SUPERFAMILY) |
| 2972 | lib_dib_Filter15_IDBA_contig_62243_plus1 | 32 | arylacetamide deacetylase-like 2-like | 174 | 5.37E-20 | 54.75% | 2 | P:metabolic process; F:hydrolase activity | IPR002168 (PROSITE); IPR013094 (PFAM); G3DSA:3.40.50.1820 (GENE3D), PTHR23024 (PANTHER), SSF53474 (SUPERFAMILY) |
| 2973 | lib_dib_Filter15_IDBA_contig_62380_plus2 | 14 | protein | 173 | 9.15E-29 | 55.55% | 4 | F:receptor binding; C:extracellular space; P:signal transduction; F:calcium ion binding | IPR002181 (PFAM); IPR014715 (G3DSA:4.10.530.GENE3D); IPR014716 (G3DSA:3.90.215.GENE3D); PTHR19143 (PANTHER) |
| 2974 | lib_dib_Filter15_IDBA_contig_62411_minus2 | 14 | zinc finger protein 271-like | 173 | 1.43E-40 | 58.50% | 3 | F:binding; F:nucleic acid binding; C:intracellular | IPR007087 (PROSITE); IPR013087 (G3DSA:3.30.160.GENE3D); IPR015880 (SMART); PTHR24375 (PANTHER), PTHR24375:SF0 (PANTHER), PF12874 (PFAM), PF13465 (PFAM), PF13912 (PFAM), SSF57667 (SUPERFAMILY) |
| 2975 | lib_dib_Filter15_IDBA_contig_62462_minus2 | 21 | acyl- synthetase family member 4 isoform 2 | 173 | 6.38E-58 | 70.70% | 2 | P:lipid metabolic process; F:catalytic activity | IPR011047 (SUPERFAMILY); IPR018391 (SMART); IPR026897 (PTHR24095:PANTHER); G3DSA:2.140.10.10 (GENE3D), PTHR24095 (PANTHER), PF13360 (PFAM), SignalP-NN(euk) (SIGNALP) |
| 2976 | lib_dib_Filter15_IDBA_contig_62470_minus3 | 9 | tumor necrosis factor receptor superfamily member 19 precursor | 173 | 2.55E-11 | 45.55% | 3 | P:multicellular organismal development; P:signal transduction; F:protein binding | IPR001368 (PFAM); G3DSA:2.10.50.10 (GENE3D), PTHR14415 (PANTHER), SignalP-NN(euk) (SIGNALP), tmhmm (TMHMM), SSF57586 (SUPERFAMILY) |
| 2977 | lib_dib_Filter15_IDBA_contig_62539_minus3 | 24 | neural ectodermal development factor imp-l2 | 173 | 1.56E-40 | 51.95% | 1 | F:protein binding | IPR003598 (SMART); IPR003599 (SMART); IPR007110 (PROFILE); IPR013783 (G3DSA:2.60.40.GENE3D); PTHR10489 (PANTHER), PF13895 (PFAM), SSF48726 (SUPERFAMILY) |
| 2978 | lib_dib_Filter15_IDBA_contig_62547_plus3 | 687 | peptidyl-prolyl cis-trans isomerase fkbp3 | 355 | 2.06E-71 | 69.65% | 6 | F:binding; P:cellular protein modification process; C:cell; C:nucleus; F:catalytic activity; P:protein metabolic process | IPR001179 (PFAM); IPR023566 (PANTHER); G3DSA:3.10.50.40 (GENE3D), PTHR10516:SF115 (PANTHER), SSF54534 (SUPERFAMILY) |
| 2979 | lib_dib_Filter15_IDBA_contig_62568_minus3 | 17 | zinc finger protein 271-like | 173 | 5.45E-34 | 53.05% | 3 | F:binding; F:nucleic acid binding; C:intracellular | IPR007087 (PFAM); IPR013087 (G3DSA:3.30.160.GENE3D); IPR015880 (SMART); PTHR24402 (PANTHER), PTHR24402:SF3 (PANTHER), PF13465 (PFAM), PF13894 (PFAM), SSF57667 (SUPERFAMILY) |
| 2980 | lib_dib_Filter15_IDBA_contig_62584_plus2 | 62 | beta- -n-acetylgalactosaminyltransferase bre-4-like | 173 | 1.82E-26 | 63.95% | 2 | F:transferase activity; P:carbohydrate metabolic process | IPR003859 (PRINTS); G3DSA:3.90.550.10 (GENE3D), SignalP-NN(euk) (SIGNALP), SSF53448 (SUPERFAMILY) |
| 2981 | lib_dib_Filter15_IDBA_contig_62604_plus3 | 109 | calcium binding egf domain containing protein | 172 | 1.34E-12 | 54.00% | 8 | F:metal ion binding; P:proteolysis; F:metallopeptidase activity; F:calcium ion binding; F:hydrolase activity; F:zinc ion binding; F:peptidase activity; F:metalloendopeptidase activity | IPR000859 (G3DSA:2.60.120.GENE3D); PTHR10127 (PANTHER) |

| | | | | | | | | | | |
|---|---|---|---|---|---|---|---|---|---|---|
| 2982 | lib_dib_Filter15_IDBA_contig_62605_plus2 | 287 | neurogenic locus notch homolog protein 4 | 161 | 1.41E-21 | 55.35% | 15 | P:anatomical structure morphogenesis; ; P:multicellular organismal development; F:protein binding; P:protein metabolic process; C:cell; C:extracellular region; C:organelle; P:cell differentiation; P:regulation of biological process; C:intracellular; C:plasma membrane; C:cytoplasm; C:nucleus; F:calcium ion binding | IPR000742 (PFAM); IPR000859 (G3DSA:2.60.120.GENE3D); IPR001881 (SMART); IPR013032 (PROSITE); PR00010 (PRINTS), G3DSA:2.10.25.10 (GENE3D), PTHR24044 (PANTHER), SSF57196 (SUPERFAMILY) | yes |
| 2983 | lib_dib_Filter15_IDBA_contig_62663_plus2 | 18 | epidermal growth factor receptor | 173 | 1.93E-72 | 77.10% | 33 | P:multicellular organismal development; P:signal transduction; P:anatomical structure morphogenesis; P:cellular component organization; P:cell differentiation; P:biosynthetic process; P:regulation of biological process; F:DNA binding; P:response to stress; P:DNA metabolic process; P:cellular protein modification process; P:protein metabolic process; P:catabolic process; P:biological_process; P:metabolic process; C:plasma membrane; C:extracellular space; C:cell; C:protein complex; P:cell proliferation; F:protein kinase activity; F:signal transducer activity; P:cell cycle; P:response to abiotic stimulus; F:receptor activity; F:protein binding; C:cytoplasmic membrane-bounded vesicle; F:nucleotide binding; F:actin binding; F:enzyme regulator activity; P:cell death; C:nucleus; C:endosome | IPR000719 (PROFILE); IPR001245 (PRINTS); IPR002290 (SMART); IPR008266 (PROSITE); IPR011009 (SUPERFAMILY); IPR020635 (SMART); G3DSA:1.10.510.10 (GENE3D), G3DSA:3.30.200.20 (GENE3D), PTHR24416 (PANTHER), PTHR24416:SF95 (PANTHER), SignalP-NN(euk) (SIGNALP) | |
| 2984 | lib_dib_Filter15_IDBA_contig_62694_minus3 | 17 | low quality protein: semaphorin-5b | 172 | 8.04E-33 | 50.40% | 4 | F:receptor activity; C:integral to membrane; P:multicellular organismal development; C:membrane | IPR000884 (PFAM); IPR027231 (PANTHER); PR01705 (PRINTS), G3DSA:2.20.100.10 (GENE3D), PTHR11036:SF39 (PANTHER) | |
| 2985 | lib_dib_Filter15_IDBA_contig_62717_minus2 | 29 | gram-negative bacteria binding protein | 173 | 9.04E-34 | 62.15% | 2 | F:catalytic activity; P:carbohydrate metabolic process | IPR000757 (PFAM); IPR008985 (SUPERFAMILY); IPR013320 (G3DSA:2.60.120.GENE3D); PTHR10963 (PANTHER), PTHR10963:SF4 (PANTHER) | |
| 2986 | lib_dib_Filter15_IDBA_contig_62741_plus2 | 29 | von willebrand factor a domain-containing protein 8 | 173 | 1.90E-30 | 69.80% | 1 | C:mitochondrion | PTHR21610 (PANTHER), PTHR21610:SF2 (PANTHER) | |
| 2987 | lib_dib_Filter15_IDBA_contig_62927_plus1 | 25 | dipeptidyl peptidase 1-like | 172 | 1.14E-70 | 72.10% | 6 | C:lysosome; F:binding; F:peptidase activity; P:biological_process; P:protein metabolic process; P:catabolic process | IPR000668 (PFAM); IPR013128 (PANTHER); IPR025660 (PROSITE); IPR025661 (PROSITE); G3DSA:3.90.70.10 (GENE3D), PTHR12411:SF17 (PANTHER), SSF54001 (SUPERFAMILY) | |
| 2988 | lib_dib_Filter15_IDBA_contig_62978_plus2 | 15 | neuroligin- x-linked | 173 | 1.22E-53 | 59.60% | 1 | F:hydrolase activity | IPR002018 (PFAM); G3DSA:3.40.50.1820 (GENE3D), PTHR11559 (PANTHER), SignalP-NN(euk) (SIGNALP), SSF53474 (SUPERFAMILY) | yes |
| 2989 | lib_dib_Filter15_IDBA_contig_63008_plus3 | 2516 | cysteine-rich secretory protein mr30 | 384 | 8.32E-53 | 53.45% | 6 | P:proteolysis; F:hydrolase activity; F:peptidase activity; C:extracellular region; F:ion channel inhibitor activity; F:calcium channel inhibitor activity | IPR001283 (PANTHER); IPR014044 (G3DSA:3.40.33.GENE3D); SignalP-NN(euk) (SIGNALP) | |
| 2990 | lib_dib_Filter15_IDBA_contig_63073_plus1 | 48 | c-type lectin domain family 4 member m | 327 | 7.34E-11 | 54.00% | 1 | F:carbohydrate binding | IPR001304 (PFAM); IPR016186 (G3DSA:3.10.100.GENE3D); IPR016187 (SUPERFAMILY); PTHR22802 (PANTHER), tmhmm (TMHMM) | |

| | | | | | | | | | |
|---|---|---|---|---|---|---|---|---|---|
| 2991 | lib_dib_Filter15_IDBA_contig_63074_plus1 | 26 | c-type lectin domain family 4 member m | 200 | 1.65E-11 | 53.55% | 1 | F:carbohydrate binding | IPR001304 (PFAM); IPR016186 (G3DSA:3.10.100.GENE3D); IPR016187 (SUPERFAMILY); PTHR22802 (PANTHER), SignalP-NN(euk) (SIGNALP) yes |
| 2992 | lib_dib_Filter15_IDBA_contig_63075_plus1 | 14 | galactose-specific c-type | 127 | 1.65E-18 | 50.20% | 1 | F:carbohydrate binding | IPR001304 (PFAM); IPR016186 (G3DSA:3.10.100.GENE3D); IPR016187 (SUPERFAMILY); IPR018378 (PROSITE); PTHR22802 (PANTHER), tmhmm (TMHMM) |
| 2993 | lib_dib_Filter15_IDBA_contig_63130_minus1 | 12 | cub domain protein | 172 | 2.30E-18 | 55.20% | 1 | F:hydrolase activity | IPR000859 (G3DSA:2.60.120.GENE3D); PTHR10127 (PANTHER) |
| 2994 | lib_dib_Filter15_IDBA_contig_63163_plus3 | 11 | fat-like cadherin-related tumor suppressor homolog | 171 | 6.35E-42 | 61.65% | 3 | C:cell; F:calcium ion binding; P:biological_process | IPR002126 (PRINTS); IPR015919 (SUPERFAMILY); PTHR24027 (PANTHER) |
| 2995 | lib_dib_Filter15_IDBA_contig_63182_plus1 | 39 | laminin b2 | 172 | 3.17E-55 | 65.15% | 2 | F:transferase activity; F:transmembrane receptor protein tyrosine kinase activity | IPR000034 (PFAM); IPR002049 (PFAM); G3DSA:2.10.25.10 (GENE3D), PTHR10574 (PANTHER), PTHR10574:SF48 (PANTHER), SSF57196 (SUPERFAMILY) |
| 2996 | lib_dib_Filter15_IDBA_contig_63190_minus3 | 400 | phosphatidylethanolamine binding protein isoform 2 | 272 | 1.67E-72 | 70.05% | 1 | F:lipid binding | IPR008914 (G3DSA:3.90.280.GENE3D); PTHR11362 (PANTHER) |
| 2997 | lib_dib_Filter15_IDBA_contig_6332_plus1 | 1836 | deleted in malignant brain tumors 1 | 1089 | 0 | 44.25% | 2 | F:receptor activity; C:cell | IPR001190 (PRINTS); IPR017448 (SMART); G3DSA:3.10.250.10 (GENE3D), PTHR19331 (PANTHER), PTHR19331:SF118 (PANTHER) |
| 2998 | lib_dib_Filter15_IDBA_contig_63409_plus2 | 23 | zinc finger protein 271 (zinc finger protein 7) (zinc finger protein znfphex133) (epstein-barr virus-induced zinc finger protein) (znf-eb) (ct-zfp48) (zinc finger protein | 171 | 3.40E-45 | 59.90% | 3 | F:binding; F:nucleic acid binding; C:intracellular | IPR007087 (PFAM); IPR013087 (G3DSA:3.30.160.GENE3D); IPR015880 (SMART); PTHR24377 (PANTHER), PTHR24377:SF0 (PANTHER), PF13465 (PFAM), SSF57667 (SUPERFAMILY) |
| 2999 | lib_dib_Filter15_IDBA_contig_6354_minus3 | 680 | xanthine dehydrogenase oxidase | 1501 | 0 | 56.90% | 5 | F:electron carrier activity; F:binding; F:catalytic activity; P:metabolic process; F:nucleotide binding | IPR000674 (G3DSA:3.90.1170.GENE3D); IPR001041 (SUPERFAMILY); IPR002346 (PFAM); IPR002888 (G3DSA:1.10.150.GENE3D); IPR005107 (PFAM); IPR006058 (PROSITE); IPR008274 (G3DSA:3.30.365.GENE3D); IPR012675 (G3DSA:3.10.20.GENE3D); IPR016166 (PROFILE); IPR016169 (G3DSA:3.30.465.GENE3D); G3DSA:3.30.390.50 (GENE3D), PTHR11908 (PANTHER), PTHR11908:SF5 (PANTHER), SignalP-NN(euk) (SIGNALP) |
| 3000 | lib_dib_Filter15_IDBA_contig_63568_plus1 | 60 | cd109 antigen | 274 | 1.06E-45 | 55.65% | 4 | P:negative regulation of endopeptidase activity; C:extracellular space; C:extracellular region; F:endopeptidase inhibitor activity | PTHR11412 (PANTHER), SignalP-NN(euk) (SIGNALP), tmhmm (TMHMM) |
| 3001 | lib_dib_Filter15_IDBA_contig_63587_plus1 | 2608 | c-type lectin domain family 4 member a isoform 4 | 708 | 6.30E-07 | 45.95% | 1 | F:carbohydrate binding | IPR001304 (PFAM); IPR016186 (G3DSA:3.10.100.GENE3D); IPR016187 (SUPERFAMILY); PTHR22802 (PANTHER), SignalP-NN(euk) (SIGNALP), tmhmm (TMHMM) yes |
| 3002 | lib_dib_Filter15_IDBA_contig_63588_plus3 | 2552 | c-type lectin domain family member a | 707 | 6.23E-07 | 45.75% | 1 | F:carbohydrate binding | IPR001304 (PFAM); IPR016186 (G3DSA:3.10.100.GENE3D); IPR016187 (SUPERFAMILY); PTHR22802 (PANTHER), SignalP-NN(euk) (SIGNALP), tmhmm (TMHMM) yes |

| | | | | | | | | | |
|---|---|---|---|---|---|---|---|---|---|
| 3003 | lib_dib_Filter15_IDBA_contig_63614_plus3 | 54 | procollagen c-endopeptidase enhancer 2 | 302 | 8.45E-22 | 44.70% | 18 | P:proteolysis; F:serine-type endopeptidase activity; F:catalytic activity; C:collagen; C:extracellular region; P:single fertilization; F:scavenger receptor activity; F:carbohydrate binding; P:cell adhesion; C:membrane; F:serine-type peptidase activity; F:hydrolase activity; F:peptidase activity; F:peptidase activator activity; P:positive regulation of peptidase activity; F:heparin binding; F:collagen binding; F:calcium ion binding | IPR000859 (G3DSA:2.60.120.GENE3D); PTHR10127 (PANTHER) |
| 3004 | lib_dib_Filter15_IDBA_contig_63667_minus2 | 19 | inversin | 170 | 1.60E-89 | 78.60% | 4 | C:cytoskeleton; P:signal transduction; F:protein binding; P:multicellular organismal development | IPR002110 (PFAM); IPR020683 (G3DSA:1.25.40.GENE3D); PTHR24178 (PANTHER), PTHR24178:SF0 (PANTHER) |
| 3005 | lib_dib_Filter15_IDBA_contig_63713_plus3 | 852 | hypothetical protein BRAFLDRAFT_125152 | 170 | 2.02E-11 | 49.10% | 9 | F:calcium ion binding; C:integral to membrane; C:membrane; F:extracellular ligand-gated ion channel activity; P:ion transport; P:transport; F:transferase activity; F:hydrolase activity; F:transmembrane receptor protein tyrosine kinase activity | IPR000859 (G3DSA:2.60.120.GENE3D); PTHR10127 (PANTHER) |
| 3006 | lib_dib_Filter15_IDBA_contig_63826_plus2 | 105 | adp-ribosylation factor 1 | 381 | 2.75E-63 | 75.15% | 13 | C:cytosol; C:plasma membrane; F:enzyme regulator activity; P:biosynthetic process; P:cellular protein modification process; F:transporter activity; P:protein transport; C:vacuole; C:cytoplasmic membrane-bounded vesicle; C:Golgi apparatus; C:cell; F:nucleotide binding; P:signal transduction | IPR005225 (TIGRFAMs); IPR006687 (SMART); IPR006689 (PRINTS); IPR024156 (SMART); G3DSA:3.40.50.300 (GENE3D), PTHR11711 (PANTHER), tmhmm (TMHMM), SSF52540 (SUPERFAMILY) |
| 3007 | lib_dib_Filter15_IDBA_contig_63837_plus1 | 28 | protein gly- isoform a | 170 | 1.74E-57 | 69.00% | 5 | F:transferase activity; C:Golgi apparatus; P:biosynthetic process; P:carbohydrate metabolic process; C:cell | IPR001173 (PFAM); G3DSA:3.90.550.10 (GENE3D), PTHR11675 (PANTHER), SSF53448 (SUPERFAMILY) |
| 3008 | lib_dib_Filter15_IDBA_contig_63869_plus3 | 117 | superoxide dismutase | 422 | 5.04E-38 | 59.20% | 3 | P:metabolic process; F:binding; F:catalytic activity | IPR001424 (PRINTS); IPR018152 (PROSITE); IPR024134 (PANTHER); SignalP-NN(euk) (SIGNALP), tmhmm (TMHMM) |
| 3009 | lib_dib_Filter15_IDBA_contig_63881_minus1 | 70 | heat shock 70 kda protein 13-like | 305 | 2.96E-127 | 69.05% | 1 | F:nucleotide binding | IPR013126 (PRINTS); IPR018181 (PROSITE); G3DSA:3.30.420.40 (GENE3D), G3DSA:3.90.640.10 (GENE3D), PTHR19375 (PANTHER), PTHR19375:SF11 (PANTHER), SSF53067 (SUPERFAMILY) |
| 3010 | lib_dib_Filter15_IDBA_contig_63930_minus1 | 53 | prolow-density lipoprotein receptor-related protein 1 | 301 | 9.13E-92 | 65.95% | 15 | P:transport; P:regulation of biological process; P:cellular component organization; P:biological_process; P:signal transduction; P:cell proliferation; C:cell; P:metabolic process; P:cell death; F:receptor binding; P:protein transport; C:endosome; F:protein binding; F:receptor activity; C:cytoplasmic membrane-bounded vesicle | IPR002172 (G3DSA:4.10.400.GENE3D); IPR023415 (PROSITE); PR00261 (PRINTS), PTHR10529 (PANTHER), PTHR10529:SF100 (PANTHER) |
| 3011 | lib_dib_Filter15_IDBA_contig_64080_plus1 | 38 | matrix metalloproteinase 2 | 216 | 8.23E-14 | 41.55% | 16 | F:metalloendopeptidase activity; F:hydrolase activity; F:zinc ion binding; F:metallopeptidase activity; C:extracellular matrix; F:peptidase activity; P:proteolysis; F:metal ion binding; P:metabolic process; F:carbohydrate binding; P:collagen catabolic process; C:extracellular space; C:proteinaceous extracellular matrix; P:single fertilization; C:extracellular region; C:collagen | IPR000562 (G3DSA:2.10.10.GENE3D); IPR013806 (SUPERFAMILY); PTHR22918 (PANTHER), PTHR22918:SF0 (PANTHER), SignalP-NN(euk) (SIGNALP) |

| | | | | | | | | | | |
|---|---|---|---|---|---|---|---|---|---|---|
| 3012 | lib_dib_Filter15_IDBA_contig_64081_plus1 | 43 | matrix metalloproteinase 2 | 257 | 1.50E-13 | 41.35% | 16 | F:carbohydrate binding; F:metalloendopeptidase activity; F:hydrolase activity; F:zinc ion binding; F:metallopeptidase activity; C:extracellular matrix; F:peptidase activity; P:proteolysis; F:metal ion binding; P:metabolic process; P:collagen catabolic process; C:extracellular space; C:proteinaceous extracellular matrix; P:single fertilization; C:extracellular region; C:collagen | IPR000562 (G3DSA:2.10.10.GENE3D); IPR013806 (SUPERFAMILY); PTHR22918 (PANTHER), PTHR22918:SF0 (PANTHER) | |
| 3013 | lib_dib_Filter15_IDBA_contig_64091_minus3 | 16 | transport and golgi organization-like | 169 | 2.30E-29 | 61.30% | 7 | P:anatomical structure morphogenesis; P:cellular component organization; P:cell differentiation; P:multicellular organismal development; P:protein transport; C:cytosol; F:protein binding | IPR000210 (SMART); IPR011333 (G3DSA:3.30.710.GENE3D); IPR013069 (PFAM); PTHR24413 (PANTHER) | |
| 3014 | lib_dib_Filter15_IDBA_contig_64115_minus2 | 18 | neurogenic locus notch homolog protein 2-like | 169 | 1.44E-23 | 52.10% | 10 | P:metabolic process; P:regulation of biological process; P:multicellular organismal development; P:embryo development; P:anatomical structure morphogenesis; P:cell differentiation; P:cell proliferation; P:biological_process; F:protein binding; F:calcium ion binding | IPR000152 (PROSITE); IPR000742 (PFAM); IPR001881 (SMART); IPR013032 (PFAM); PR00010 (PRINTS), G3DSA:2.10.25.10 (GENE3D), PTHR24044 (PANTHER), SSF57196 (SUPERFAMILY) | |
| 3015 | lib_dib_Filter15_IDBA_contig_64130_minus2 | 20 | cg3603-pa | 169 | 3.57E-61 | 69.95% | 3 | P:metabolic process; F:catalytic activity; F:nucleotide binding | IPR002198 (PRINTS); IPR002347 (PRINTS); IPR016040 (G3DSA:3.40.50.GENE3D); PTHR24316 (PANTHER), PTHR24316:SF3 (PANTHER), SignalP-NN(euk) (SIGNALP), SSF51735 (SUPERFAMILY) | |
| 3016 | lib_dib_Filter15_IDBA_contig_64434_plus1 | 37 | prolow-density lipoprotein receptor-related protein 1 | 169 | 1.16E-11 | 59.85% | 15 | C:organelle; C:intracellular; P:transport; P:cellular component organization; P:biosynthetic process; P:lipid metabolic process; P:regulation of biological process; F:calcium ion binding; P:signal transduction; P:cytoskeleton organization; C:cell; P:biological_process; F:protein binding; C:plasma membrane; C:cytoplasm | IPR002172 (G3DSA:4.10.400.GENE3D); IPR023415 (PROSITE); PR00261 (PRINTS), PTHR10529 (PANTHER), PTHR10529:SF113 (PANTHER), SignalP-NN(euk) (SIGNALP), tmhmm (TMHMM) | yes |
| 3017 | lib_dib_Filter15_IDBA_contig_64445_minus2 | 22 | bola-like protein 3 | 169 | 2.95E-33 | 72.65% | 4 | F:molecular_function; P:biological_process; C:cellular_component; C:extracellular region | IPR002634 (PFAM); G3DSA:3.30.70.1500 (GENE3D), PTHR12735 (PANTHER), PTHR12735:SF4 (PANTHER), SignalP-NN(euk) (SIGNALP) | |
| 3018 | lib_dib_Filter15_IDBA_contig_64466_plus1 | 67 | von willebrand factor d and egf domain-containing protein | 288 | 1.58E-38 | 54.05% | 1 | F:protein binding | IPR000742 (PFAM); IPR013032 (PROSITE); G3DSA:2.10.25.10 (GENE3D), PTHR24838 (PANTHER), SSF57196 (SUPERFAMILY) | |
| 3019 | lib_dib_Filter15_IDBA_contig_64467_plus3 | 242 | von willebrand factor d and egf domain-containing protein | 738 | 2.23E-137 | 56.10% | 1 | F:protein binding | IPR000742 (SMART); IPR003598 (SMART); IPR003599 (SMART); IPR007110 (PROFILE); IPR013032 (PROSITE); IPR013098 (PFAM); IPR013783 (G3DSA:2.60.40.GENE3D); PTHR10489 (PANTHER), tmhmm (TMHMM), SSF48726 (SUPERFAMILY), SSF57196 (SUPERFAMILY) | |
| 3020 | lib_dib_Filter15_IDBA_contig_64536_minus2 | 18 | carboxypeptidase m | 168 | 6.90E-11 | 59.00% | 1 | F:hydrolase activity | IPR008969 (SUPERFAMILY); IPR014766 (G3DSA:2.60.40.GENE3D); PTHR11532 (PANTHER), PF13620 (PFAM) | |
| 3021 | lib_dib_Filter15_IDBA_contig_64564_plus3 | 28 | discoidin domain | 198 | 3.73E-25 | 75.30% | 5 | F:receptor activity; F:protein kinase activity; P:biological_process; P:cellular protein modification process; F:nucleotide binding | IPR001245 (PFAM); IPR011009 (SUPERFAMILY); G3DSA:1.10.510.10 (GENE3D), PTHR24416 (PANTHER), PTHR24416:SF64 (PANTHER) | |

| | | | | | | | | | | |
|---|---|---|---|---|---|---|---|---|---|---|
| 3022 | lib_dib_Filter15_IDBA_contig_64602_plus3 | 18 | glass protein | 240 | 2.98E-17 | 65.80% | 4 | ; F:nucleic acid binding; C:intracellular; F:binding | IPR007087 (PFAM); IPR013087 (G3DSA:3.30.160.GENE3D); IPR015880 (SMART); PTHR11389 (PANTHER), PF13465 (PFAM), SSF57667 (SUPERFAMILY) | |
| 3023 | lib_dib_Filter15_IDBA_contig_64605_minus3 | 573 | galectin-3-binding protein precursor | 367 | 3.17E-26 | 64.40% | 3 | F:catalytic activity; F:receptor activity; C:cell | IPR001190 (PRINTS); IPR017448 (SMART); G3DSA:3.10.250.10 (GENE3D), PTHR19331 (PANTHER), SignalP-NN(euk) (SIGNALP), tmhmm (TMHMM) | yes |
| 3024 | lib_dib_Filter15_IDBA_contig_64620_minus3 | 36 | e3 ubiquitin-protein ligase rnf123 | 168 | 4.81E-43 | 75.15% | 4 | F:binding; F:catalytic activity; C:cytoplasm; F:protein binding | IPR003877 (PFAM); IPR008985 (SUPERFAMILY); PTHR13363 (PANTHER), PTHR13363:SF0 (PANTHER) | |
| 3025 | lib_dib_Filter15_IDBA_contig_64629_plus3 | 17 | gcc2 and gcc3 | 168 | 4.59E-12 | 44.35% | 2 | F:calcium ion binding; P:cell adhesion | IPR009030 (SUPERFAMILY); IPR011641 (PFAM) | |
| 3026 | lib_dib_Filter15_IDBA_contig_64684_minus3 | 14 | oocyte zinc finger protein 6-like | 168 | 2.12E-29 | 56.40% | 3 | F:binding; F:nucleic acid binding; C:intracellular | IPR007087 (PROSITE); IPR013087 (G3DSA:3.30.160.GENE3D); IPR015880 (SMART); PTHR11389 (PANTHER), PF13465 (PFAM), SSF57667 (SUPERFAMILY) | |
| 3027 | lib_dib_Filter15_IDBA_contig_64823_plus1 | 20 | slit homolog 1 protein | 168 | 2.10E-06 | 73.74% | 34 | P:brain development; P:telencephalon cell migration; P:tangential migration from the subventricular zone to the olfactory bulb; P:dorsal/ventral axon guidance; P:axonogenesis; P:establishment of nucleus localization; P:negative regulation of synapse assembly; P:retinal ganglion cell axon guidance; P:negative chemotaxis; C:extracellular space; P:axon extension involved in axon guidance; F:calcium ion binding; P:negative regulation of axon extension involved in axon guidance; P:neuron projection morphogenesis; P:motor axon guidance; P:spinal cord development; C:cytoplasm; F:heparan sulfate proteoglycan binding; P:axon guidance; P:olfactory bulb development; P:chemorepulsion involved in embryonic olfactory bulb interneuron precursor migration; F:protein binding; F:Roundabout binding; C:extracellular region; P:morphogenesis of a branching structure; P:negative regulation of axon extension; P:endocardial progenitor cell migration to the midline involved in heart field formation; P:axonal defasciculation; F:metal ion binding; F:zinc ion binding; | IPR000372 (PFAM); PTHR24369 (PANTHER), tmhmm (TMHMM) | |
| 3028 | lib_dib_Filter15_IDBA_contig_64846_minus3 | 25 | receptor tyrosine-protein kinase erbb-3-like | 167 | 5.13E-41 | 61.50% | 13 | F:enzyme regulator activity; F:protein binding; P:cell proliferation; P:regulation of biological process; P:signal transduction; P:transport; C:plasma membrane; C:extracellular space; P:cell death; P:multicellular organismal development; F:receptor activity; F:protein kinase activity; F:nucleotide binding | IPR006211 (PFAM); IPR006212 (SMART); IPR009030 (SUPERFAMILY); G3DSA:2.10.220.10 (GENE3D), PTHR26391 (PANTHER), PTHR26391:SF0 (PANTHER) | |

| | | | | | | | | | | |
|---|---|---|---|---|---|---|---|---|---|---|
| 3029 | lib_dib_Filter15_IDBA_contig_64915_minus1 | 104 | lipophorin receptor | 382 | 1.88E-85 | 58.30% | 4 | F:receptor activity; C:plasma membrane; F:protein binding; F:calcium ion binding | IPR000033 (PFAM); IPR000152 (PROSITE); IPR000742 (SMART); IPR001881 (PFAM); IPR002172 (G3DSA:4.10.400.GENE3D); IPR011042 (G3DSA:2.120.10.GENE3D); IPR013032 (PROSITE); IPR018097 (PROSITE); IPR023415 (PROSITE); PR00261 (PRINTS), G3DSA:2.10.25.10 (GENE3D), PTHR10529 (PANTHER), SSF57196 (SUPERFAMILY), SSF63825 (SUPERFAMILY) | |
| 3030 | lib_dib_Filter15_IDBA_contig_64916_minus1 | 75 | lipophorin receptor | 249 | 7.21E-40 | 56.05% | 2 | F:protein binding; F:calcium ion binding | IPR000152 (PROSITE); IPR000742 (SMART); IPR001881 (PFAM); IPR002172 (G3DSA:4.10.400.GENE3D); IPR013032 (PROSITE); IPR018097 (PROSITE); IPR023415 (PROSITE); PR00261 (PRINTS), G3DSA:2.10.25.10 (GENE3D), PTHR10529 (PANTHER), SSF57196 (SUPERFAMILY) | |
| 3031 | lib_dib_Filter15_IDBA_contig_64917_minus1 | 19 | vitellogenin receptor | 119 | 8.19E-09 | 58.90% | 2 | C:cell; F:protein binding | IPR002172 (G3DSA:4.10.400.GENE3D); IPR023415 (PROSITE); PR00261 (PRINTS), G3DSA:4.10.1220.10 (GENE3D), PTHR10529 (PANTHER) | |
| 3032 | lib_dib_Filter15_IDBA_contig_64946_plus1 | 17 | glass protein | 168 | 1.11E-21 | 67.95% | 3 | F:binding; F:nucleic acid binding; C:intracellular | IPR007087 (PROSITE); IPR013087 (G3DSA:3.30.160.GENE3D); IPR015880 (SMART); PTHR11389 (PANTHER), PF13465 (PFAM), SSF57667 (SUPERFAMILY) | |
| 3033 | lib_dib_Filter15_IDBA_contig_64949_minus1 | 24 | apical endosomal glycoprotein | 168 | 3.16E-12 | 51.65% | 2 | F:catalytic activity; C:cell | IPR000998 (PRINTS); IPR008985 (SUPERFAMILY); PTHR23282 (PANTHER) | |
| 3034 | lib_dib_Filter15_IDBA_contig_64969_minus2 | 23 | transport and golgi organization-like | 167 | 8.21E-36 | 50.95% | 1 | F:protein binding | IPR011333 (G3DSA:3.30.710.GENE3D); IPR011705 (PFAM); IPR013069 (PFAM); PTHR24413 (PANTHER) | |
| 3035 | lib_dib_Filter15_IDBA_contig_64998_plus1 | 39157 | gelsolin-like protein 2-like | 848 | 7.11E-118 | 68.30% | 5 | F:calcium ion binding; P:biological_process; P:cytoskeleton organization; F:actin binding; P:regulation of biological process | IPR007122 (PRINTS); IPR007123 (PFAM); G3DSA:3.40.20.10 (GENE3D), PTHR11977:SF19 (PANTHER), tmhmm (TMHMM), SSF55753 (SUPERFAMILY) | |
| 3036 | lib_dib_Filter15_IDBA_contig_64999_plus3 | 45088 | gelsolin-like protein 2-like | 1009 | 1.16E-170 | 71.90% | 6 | F:calcium ion binding; P:biological_process; P:cytoskeleton organization; F:actin binding; C:intracellular; P:regulation of biological process | IPR007122 (PRINTS); IPR007123 (PFAM); G3DSA:3.40.20.10 (GENE3D), PTHR11977:SF19 (PANTHER), SignalP-NN(euk) (SIGNALP), tmhmm (TMHMM), SSF55753 (SUPERFAMILY) | yes |
| 3037 | lib_dib_Filter15_IDBA_contig_65010_plus3 | 19 | cysteine-rich secretory protein mr30 | 167 | 2.89E-13 | 53.36% | 4 | P:proteolysis; F:hydrolase activity; F:peptidase activity; C:extracellular region | no IPS match | |
| 3038 | lib_dib_Filter15_IDBA_contig_65135_plus2 | 102 | cd209l1 protein | 290 | 1.37E-06 | 43.84% | 1 | F:carbohydrate binding | IPR001304 (PFAM); IPR016186 (G3DSA:3.10.100.GENE3D); IPR016187 (SUPERFAMILY); PTHR22801 (PANTHER), SignalP-NN(euk) (SIGNALP), tmhmm (TMHMM) | yes |
| 3039 | lib_dib_Filter15_IDBA_contig_65307_minus3 | 52 | leucine-rich repeat neuronal protein 1 | 166 | 1.44E-14 | 49.95% | 5 | C:membrane; F:protein binding; P:nervous system development; C:integral to membrane; C:PML body | IPR003591 (SMART); G3DSA:3.80.10.10 (GENE3D), PTHR24373 (PANTHER), PF13855 (PFAM), SSF52058 (SUPERFAMILY) | |
| 3040 | lib_dib_Filter15_IDBA_contig_65311_plus2 | 1803 | l-rhamnose-binding lectin csl3-like | 597 | 1.12E-11 | 51.35% | 1 | F:carbohydrate binding | IPR000922 (PFAM); PTHR12011 (PANTHER), PTHR12011:SF4 (PANTHER), SignalP-NN(euk) (SIGNALP), tmhmm (TMHMM) | |
| 3041 | lib_dib_Filter15_IDBA_contig_65388_plus3 | 21 | retinol dehydrogenase 13-like | 166 | 4.54E-55 | 71.35% | 3 | F:catalytic activity; F:nucleotide binding; P:metabolic process | IPR002198 (PFAM); IPR002347 (PRINTS); IPR016040 (G3DSA:3.40.50.GENE3D); PTHR24316 (PANTHER), PTHR24316:SF44 (PANTHER), SSF51735 (SUPERFAMILY) | |
| 3042 | lib_dib_Filter15_IDBA_contig_65397_plus1 | 56 | ---NA--- | 112 | | | 0 | - | no IPS match | |
| 3043 | lib_dib_Filter15_IDBA_contig_65397_plus2 | 56 | ---NA--- | 112 | | | 0 | - | no IPS match | |

| | | | | | | | | | | |
|---|---|---|---|---|---|---|---|---|---|---|
| 3044 | lib_dib_Filter15_IDBA_contig_65399_minus1 | 63 | ---NA--- | 120 | | | 0 | - | no IPS match | |
| 3045 | lib_dib_Filter15_IDBA_contig_65401_minus2 | 3110 | ---NA--- | 139 | | | 0 | - | PR01217 (PRINTS) | |
| 3046 | lib_dib_Filter15_IDBA_contig_65412_minus1 | 297 | 3-oxoacyl-acyl-carrier-protein reductase | 448 | 8.86E-59 | 60.40% | 3 | P:metabolic process; F:catalytic activity; F:nucleotide binding | IPR002198 (PRINTS); IPR002347 (PRINTS); IPR016040 (G3DSA:3.40.50.GENE3D); PTHR24322 (PANTHER), PTHR24322:SF0 (PANTHER), SSF51735 (SUPERFAMILY) | |
| 3047 | lib_dib_Filter15_IDBA_contig_65429_minus3 | 22 | myom_lymst ame: full=myomodulin neuropeptides contains: ame: full=glqmlrl-amide contains: ame: full=qipmlrl-amide contains: ame: full=smsmlrl-amide contains: ame: full=slsmlrl-amide contains: ame: full=myomodulin-a ame: full=pmsmlrl-amide flags: precursor | 166 | 8.32E-30 | 54.09% | 2 | P:neuropeptide signaling pathway; C:extracellular region | no IPS match | |
| 3048 | lib_dib_Filter15_IDBA_contig_65501_plus3 | 46 | receptor for egg jelly-like partial | 247 | 5.75E-11 | 47.80% | 1 | P:biological_process | IPR000421 (PFAM); IPR006585 (SMART); IPR008979 (SUPERFAMILY); G3DSA:2.60.120.260 (GENE3D), PTHR19277 (PANTHER), PTHR19277:SF6 (PANTHER) | |
| 3049 | lib_dib_Filter15_IDBA_contig_65592_minus3 | 48 | pro-neuregulin- membrane-bound isoform-like | 166 | 5.77E-07 | 54.83% | 1 | F:protein binding | IPR000742 (PFAM); IPR013032 (PROSITE); G3DSA:2.10.25.10 (GENE3D), PTHR11100 (PANTHER), PTHR11100:SF7 (PANTHER), SignalP-NN(euk) (SIGNALP), tmhmm (TMHMM), SSF57196 (SUPERFAMILY) | yes |
| 3050 | lib_dib_Filter15_IDBA_contig_65858_minus3 | 176 | cubilin | 641 | 8.14E-38 | 45.80% | 6 | C:cell; C:organelle; C:intracellular; F:binding; C:cytoplasm; P:metabolic process | IPR000859 (G3DSA:2.60.120.GENE3D); PTHR10127 (PANTHER), tmhmm (TMHMM) | |
| 3051 | lib_dib_Filter15_IDBA_contig_65859_minus3 | 100 | low quality protein: cubilin | 343 | 2.86E-31 | 45.40% | 1 | F:calcium ion binding | IPR000859 (G3DSA:2.60.120.GENE3D); PTHR10127 (PANTHER) | |
| 3052 | lib_dib_Filter15_IDBA_contig_65879_minus3 | 35 | ---NA--- | 165 | | | 0 | - | SignalP-NN(euk) (SIGNALP), tmhmm (TMHMM) | yes |
| 3053 | lib_dib_Filter15_IDBA_contig_65898_plus2 | 107 | ---NA--- | 199 | | | 0 | - | IPR008160 (PFAM); PTHR24023 (PANTHER), PTHR24023:SF164 (PANTHER), SignalP-NN(euk) (SIGNALP) | |
| 3054 | lib_dib_Filter15_IDBA_contig_65913_plus2 | 26 | a disintegrin and metalloproteinase with thrombospondin motifs partial | 166 | 1.63E-82 | 84.40% | 7 | C:cell; F:peptidase activity; P:signal transduction; P:protein metabolic process; P:catabolic process; F:binding; C:proteinaceous extracellular matrix | IPR001590 (PFAM); IPR024079 (G3DSA:3.40.390.GENE3D); PTHR13723 (PANTHER), PTHR13723:SF30 (PANTHER), SSF55486 (SUPERFAMILY) | |
| 3055 | lib_dib_Filter15_IDBA_contig_65934_plus2 | 15 | u4 u6 small nuclear ribonucleoprotein prp4 | 165 | 1.61E-96 | 86.40% | 6 | C:nucleus; F:protein kinase activity; C:nucleoplasm; F:nucleic acid binding; P:nucleobase-containing compound metabolic process; F:protein binding | IPR001680 (PFAM); IPR015943 (G3DSA:2.130.10.GENE3D); IPR017986 (PROFILE); IPR019775 (PROSITE); IPR020472 (PRINTS); IPR027106 (PANTHER) | |
| 3056 | lib_dib_Filter15_IDBA_contig_66016_minus1 | 40 | pdz domain-containing protein 2 | 233 | 1.48E-29 | 66.60% | 6 | P:biological_process; C:extracellular region; C:cytoplasm; C:nucleus; C:plasma membrane; F:protein binding | IPR001478 (PFAM); G3DSA:2.30.42.10 (GENE3D), PTHR11324 (PANTHER) | |
| 3057 | lib_dib_Filter15_IDBA_contig_66049_plus2 | 15 | serine threonine-protein phosphatase 6 regulatory ankyrin repeat subunit a | 165 | 4.79E-49 | 66.65% | 1 | F:protein binding | IPR002110 (PFAM); IPR020683 (G3DSA:1.25.40.GENE3D); PTHR24158 (PANTHER) | |
| 3058 | lib_dib_Filter15_IDBA_contig_66053_plus3 | 73 | c-factor | 260 | 5.90E-35 | 61.60% | 1 | F:nucleotide binding | IPR016040 (G3DSA:3.40.50.GENE3D); PTHR24322 (PANTHER), PTHR24322:SF57 (PANTHER), SSF51735 (SUPERFAMILY) | |

| | | | | | | | | | |
|---|---|---|---|---|---|---|---|---|---|
| 3059 | lib_dib_Filter15_IDBA_contig_66121_minus1 | 27 | zinc finger protein 271-like | 165 | 6.52E-50 | 67.10% | 3 | F:binding; F:nucleic acid binding; C:intracellular | IPR007087 (PROSITE); IPR013087 (G3DSA:3.30.160.GENE3D); IPR015880 (SMART); PTHR24402 (PANTHER), PF13465 (PFAM), PF13894 (PFAM), SSF57667 (SUPERFAMILY) |
| 3060 | lib_dib_Filter15_IDBA_contig_66180_plus2 | 24 | serine threonine-protein kinase nek1 | 165 | 2.63E-106 | 93.15% | 15 | P:cellular protein modification process; F:protein kinase activity; P:response to abiotic stimulus; C:nuclear envelope; C:nucleolus; F:binding; P:biological_process; F:nucleotide binding; F:protein binding; P:organelle organization; P:cell cycle; P:anatomical structure morphogenesis; P:cellular component organization; C:microtubule organizing center; P:response to stress | IPR000719 (PFAM); IPR002290 (SMART); IPR008271 (PROSITE); IPR011009 (SUPERFAMILY); IPR020635 (SMART); G3DSA:1.10.510.10 (GENE3D), G3DSA:3.30.200.20 (GENE3D), PTHR24362 (PANTHER), PTHR24362:SF106 (PANTHER) |
| 3061 | lib_dib_Filter15_IDBA_contig_66198_plus1 | 29 | immunoglobulin superfamily member 10-like | 165 | 6.46E-42 | 50.70% | 1 | F:protein binding | IPR003598 (SMART); IPR003599 (SMART); IPR003961 (PFAM); IPR007110 (PROFILE); IPR013098 (PFAM); IPR013783 (G3DSA:2.60.40.GENE3D); PTHR10489 (PANTHER), SSF48726 (SUPERFAMILY) |
| 3062 | lib_dib_Filter15_IDBA_contig_66248_plus2 | 12 | protocadherin fat 4 | 165 | 2.35E-42 | 56.60% | 10 | P:cell differentiation; P:multicellular organismal development; P:anatomical structure morphogenesis; P:cellular component organization; P:cell recognition; F:protein binding; P:signal transduction; F:calcium ion binding; P:biological_process; C:cell | IPR002126 (PRINTS); IPR015919 (SUPERFAMILY); PTHR24027 (PANTHER) |
| 3063 | lib_dib_Filter15_IDBA_contig_66291_minus1 | 11 | protocadherin- partial | 165 | 6.04E-38 | 61.65% | 3 | F:calcium ion binding; P:biological_process; C:plasma membrane | IPR002126 (PRINTS); IPR015919 (SUPERFAMILY); IPR020894 (PROSITE); PTHR24028 (PANTHER), PTHR24028:SF12 (PANTHER) |
| 3064 | lib_dib_Filter15_IDBA_contig_66328_plus2 | 29 | beta 1 | 193 | 1.53E-40 | 57.25% | 5 | F:hydrolase activity; P:lipid metabolic process; C:lysosome; F:protein binding; P:carbohydrate metabolic process | IPR001944 (PANTHER); G3DSA:2.60.120.260 (GENE3D), PTHR23421:SF14 (PANTHER), SignalP-NN(euk) (SIGNALP), tmhmm (TMHMM) |
| 3065 | lib_dib_Filter15_IDBA_contig_66378_plus2 | 14 | ---NA--- | 164 | | | 0 | - | tmhmm (TMHMM) |
| 3066 | lib_dib_Filter15_IDBA_contig_66421_plus2 | 29 | zinc finger protein 420-like | 164 | 2.93E-34 | 59.10% | 3 | F:binding; F:nucleic acid binding; C:intracellular | IPR007087 (PROSITE); IPR013087 (G3DSA:3.30.160.GENE3D); IPR015880 (SMART); PTHR24382 (PANTHER), PTHR24382:SF0 (PANTHER), PF13465 (PFAM), SSF57667 (SUPERFAMILY) |
| 3067 | lib_dib_Filter15_IDBA_contig_66562_plus3 | 85 | polypeptide n-acetylgalactosaminyltransferase 13 | 359 | 1.05E-90 | 79.70% | 7 | C:Golgi apparatus; F:transferase activity; P:biosynthetic process; P:cellular protein modification process; P:carbohydrate metabolic process; F:carbohydrate binding; C:cell | IPR000772 (PFAM); G3DSA:2.80.10.50 (GENE3D), PTHR11675 (PANTHER), SSF53448 (SUPERFAMILY) |
| 3068 | lib_dib_Filter15_IDBA_contig_66588_plus3 | 18 | zinc finger protein 271 (zinc finger protein 7) (zinc finger protein znfphex133) (epstein-barr virus-induced zinc finger protein) (znf-eb) (ct-zfp48) (zinc finger protein | 164 | 2.43E-48 | 61.55% | 3 | F:binding; F:nucleic acid binding; C:intracellular | IPR007087 (PROSITE); IPR013087 (G3DSA:3.30.160.GENE3D); IPR015880 (SMART); PTHR24382 (PANTHER), PF13465 (PFAM), SSF57667 (SUPERFAMILY) |
| 3069 | lib_dib_Filter15_IDBA_contig_66590_plus2 | 13 | aldehyde dehydrogenase 8 member a1 | 164 | 2.01E-69 | 76.05% | 6 | P:lipid metabolic process; P:secondary metabolic process; C:intracellular; P:biosynthetic process; P:metabolic process; F:catalytic activity | IPR015590 (PFAM); IPR016161 (SUPERFAMILY); IPR016163 (G3DSA:3.40.309.GENE3D); PTHR11699 (PANTHER), PTHR11699:SF42 (PANTHER) |

| | | | | | | | | | | |
|---|---|---|---|---|---|---|---|---|---|---|
| 3070 | lib_dib_Filter15_IDBA_contig_66600_plus3 | 20 | #NAME? | 164 | 6.52E-18 | 59.15% | 1 | F:protein binding | IPR002110 (SMART); IPR020683 (G3DSA:1.25.40.GENE3D); PTHR24161 (PANTHER), SignalP-NN(euk) (SIGNALP), tmhmm (TMHMM) | yes |
| 3071 | lib_dib_Filter15_IDBA_contig_66609_plus3 | 18 | ---NA--- | 164 | | | 0 | - | IPR008160 (PFAM); PTHR24023 (PANTHER) | |
| 3072 | lib_dib_Filter15_IDBA_contig_66681_plus3 | 18 | ankyrin unc44 | 163 | 2.67E-48 | 58.95% | 1 | F:protein binding | IPR002110 (SMART); IPR020683 (G3DSA:1.25.40.GENE3D); PTHR24193 (PANTHER) | |
| 3073 | lib_dib_Filter15_IDBA_contig_66764_minus1 | 16 | zinc finger protein 236 | 164 | 2.30E-72 | 72.75% | 3 | F:binding; F:nucleic acid binding; C:intracellular | IPR007087 (PFAM); IPR013087 (G3DSA:3.30.160.GENE3D); IPR015880 (SMART); PTHR24396 (PANTHER), PTHR24396:SF0 (PANTHER), PF13465 (PFAM), PF13912 (PFAM), SSF57667 (SUPERFAMILY) | |
| 3074 | lib_dib_Filter15_IDBA_contig_66811_plus1 | 26 | inter-alpha-trypsin inhibitor heavy chain h4 isoform 1 | 164 | 4.32E-30 | 59.00% | 3 | P:metabolic process; F:enzyme regulator activity; F:protein binding | IPR002035 (PROFILE); G3DSA:3.40.50.410 (GENE3D), PTHR10338 (PANTHER), PTHR10338:SF13 (PANTHER), PF13519 (PFAM), SSF53300 (SUPERFAMILY) | |
| 3075 | lib_dib_Filter15_IDBA_contig_66840_plus3 | 52 | receptor tyrosine-protein kinase erbb-4 isoform 1 | 196 | 2.73E-41 | 60.10% | 19 | F:protein binding; F:receptor binding; P:multicellular organismal development; P:cell proliferation; P:regulation of biological process; P:signal transduction; F:receptor activity; F:protein kinase activity; C:plasma membrane; P:lipid metabolic process; P:biological_process; F:DNA binding; C:mitochondrion; P:cellular protein modification process; P:cell differentiation; ; P:cell death; C:nucleolus; P:reproduction | IPR000494 (PFAM); G3DSA:3.80.20.20 (GENE3D), PTHR24416 (PANTHER), PTHR24416:SF88 (PANTHER), SignalP-NN(euk) (SIGNALP), tmhmm (TMHMM), SSF52058 (SUPERFAMILY) | yes |
| 3076 | lib_dib_Filter15_IDBA_contig_66847_plus2 | 11 | proprotein convertase subtilisin kexin type 7 | 163 | 6.82E-49 | 60.20% | 3 | F:peptidase activity; P:protein metabolic process; P:catabolic process | IPR002884 (PFAM); IPR008979 (SUPERFAMILY); IPR015500 (PANTHER); G3DSA:2.60.120.260 (GENE3D), PTHR10795:SF9 (PANTHER) | |
| 3077 | lib_dib_Filter15_IDBA_contig_6685_plus3 | 965 | retinoid-inducible serine carboxypeptidase isoform 2 | 390 | 1.26E-49 | 51.00% | 3 | F:peptidase activity; P:protein metabolic process; P:catabolic process | IPR001563 (PRINTS); G3DSA:3.40.50.1820 (GENE3D), PTHR11802:SF3 (PANTHER), SSF53474 (SUPERFAMILY) | |
| 3078 | lib_dib_Filter15_IDBA_contig_66998_minus1 | 19 | l-xylulose reductase | 163 | 4.70E-33 | 64.90% | 6 | F:catalytic activity; P:carbohydrate metabolic process; P:metabolic process; C:cell; P:cellular component organization; F:nucleotide binding | IPR002198 (PRINTS); IPR002347 (PRINTS); IPR016040 (G3DSA:3.40.50.GENE3D); PTHR24311 (PANTHER), PTHR24311:SF0 (PANTHER), SSF51735 (SUPERFAMILY) | |
| 3079 | lib_dib_Filter15_IDBA_contig_67001_plus2 | 29 | ankyrin repeat-containing protein | 163 | 5.18E-14 | 58.20% | 1 | F:protein binding | IPR002110 (PRINTS); IPR020683 (G3DSA:1.25.40.GENE3D); PTHR24198 (PANTHER) | |
| 3080 | lib_dib_Filter15_IDBA_contig_67042_minus3 | 16 | zinc finger protein 606 | 163 | 2.00E-37 | 63.15% | 5 | C:cytoplasm; C:nucleolus; F:protein binding; F:binding; F:nucleic acid binding | IPR007087 (PROSITE); IPR013087 (G3DSA:3.30.160.GENE3D); IPR015880 (SMART); PTHR24402 (PANTHER), PTHR24402:SF3 (PANTHER), PF13465 (PFAM), SSF57667 (SUPERFAMILY) | |
| 3081 | lib_dib_Filter15_IDBA_contig_67076_minus1 | 27 | protein | 163 | 1.22E-14 | 49.05% | 3 | F:catalytic activity; F:protein binding; F:calcium ion binding | IPR000152 (PROSITE); IPR000742 (SMART); IPR001881 (PFAM); IPR013032 (PROSITE); IPR018097 (PROSITE); G3DSA:2.10.25.10 (GENE3D), PTHR24838 (PANTHER), PTHR24838:SF171 (PANTHER), SSF57196 (SUPERFAMILY) | |
| 3082 | lib_dib_Filter15_IDBA_contig_67102_plus1 | 16 | variable lymphocyte receptor a diversity region | 163 | 3.63E-08 | 53.35% | 1 | F:protein binding | IPR001611 (PROFILE); IPR003591 (SMART); G3DSA:3.80.10.10 (GENE3D), PTHR24367 (PANTHER), PF13855 (PFAM), SSF52058 (SUPERFAMILY) | |

| | | | | | | | | | |
|---|---|---|---|---|---|---|---|---|---|
| 3083 | lib_dib_Filter15_IDBA_contig_67114_minus2 | 27 | peptidylprolyl isomerase domain and wd repeat-containing protein 1 | 163 | 2.57E-80 | 92.85% | 5 | P:protein metabolic process; P:cellular protein modification process; P:nucleobase-containing compound metabolic process; F:catalytic activity; C:nucleus | IPR002130 (PRINTS); IPR024936 (PIR); G3DSA:2.40.100.10 (GENE3D), PTHR11071 (PANTHER), PTHR11071:SF40 (PANTHER) | |
| 3084 | lib_dib_Filter15_IDBA_contig_67148_plus3 | 17 | vitamin k-dependent protein s-like | 162 | 1.47E-07 | 55.65% | 1 | F:calcium ion binding | IPR000152 (PROSITE); IPR000436 (PROFILE); IPR001881 (PFAM); IPR018097 (PROSITE); G3DSA:2.10.25.10 (GENE3D), G3DSA:2.10.70.10 (GENE3D), PTHR24040 (PANTHER), SSF57196 (SUPERFAMILY) | |
| 3085 | lib_dib_Filter15_IDBA_contig_67171_plus2 | 26 | crumbs homolog 1 | 163 | 3.28E-22 | 49.65% | 1 | F:protein binding | IPR000152 (PROSITE); IPR000742 (PFAM); IPR001791 (PFAM); IPR008985 (SUPERFAMILY); IPR013032 (PROSITE); IPR013320 (G3DSA:2.60.120.GENE3D); G3DSA:2.10.25.10 (GENE3D), PTHR24026 (PANTHER), SSF57196 (SUPERFAMILY) | |
| 3086 | lib_dib_Filter15_IDBA_contig_67239_minus1 | 20 | matrix metallopeptidase 9 (gelatinase 92kda 92kda type iv collagenase) | 201 | 1.15E-16 | 45.65% | 7 | F:protein binding; P:multicellular organismal development; P:regulation of biological process; P:biological_process; F:peptidase activity; C:extracellular region; P:metabolic process | IPR000562 (G3DSA:2.10.10.GENE3D); IPR013806 (SUPERFAMILY); PR00013 (PRINTS), PTHR22918 (PANTHER), PTHR22918:SF0 (PANTHER) | |
| 3087 | lib_dib_Filter15_IDBA_contig_67254_plus2 | 19 | pathogenesis-related protein 1a | 163 | 1.92E-11 | 53.10% | 4 | P:response to biotic stimulus; P:response to stress; P:biological_process; P:regulation of biological process | IPR001283 (PANTHER); IPR014044 (G3DSA:3.40.33.GENE3D); SignalP-NN(euk) (SIGNALP), tmhmm (TMHMM) | yes |
| 3088 | lib_dib_Filter15_IDBA_contig_67280_plus3 | 27 | heat shock protein partial | 172 | 1.60E-106 | 96.30% | 2 | F:nucleotide binding; P:response to stress | IPR013126 (PRINTS); IPR018181 (PROSITE); G3DSA:3.30.420.40 (GENE3D), G3DSA:3.90.640.10 (GENE3D), PTHR19375 (PANTHER), SSF53067 (SUPERFAMILY) | |
| 3089 | lib_dib_Filter15_IDBA_contig_67470_minus2 | 12 | protocadherin gamma-a3 | 162 | 5.14E-36 | 62.75% | 3 | F:calcium ion binding; P:biological_process; C:plasma membrane | IPR002126 (PRINTS); IPR015919 (SUPERFAMILY); IPR020894 (PROSITE); PTHR24028 (PANTHER), PTHR24028:SF12 (PANTHER) | |
| 3090 | lib_dib_Filter15_IDBA_contig_67500_minus1 | 24 | atrial natriuretic peptide-converting enzyme | 162 | 1.44E-06 | 50.35% | 2 | C:cell; F:protein binding | IPR000998 (PROFILE); IPR002172 (SMART); IPR008985 (SUPERFAMILY); IPR020067 (G3DSA:1.10.2000.GENE3D) | |
| 3091 | lib_dib_Filter15_IDBA_contig_67513_plus1 | 18 | zinc finger protein 236 | 162 | 2.04E-54 | 80.15% | 7 | F:binding; ; P:regulation of biological process; F:DNA binding; P:biological_process; F:sequence-specific DNA binding transcription factor activity; C:nucleus | IPR007087 (PROSITE); IPR013087 (G3DSA:3.30.160.GENE3D); IPR015880 (SMART); PTHR24396 (PANTHER), PTHR24396:SF6 (PANTHER), PF13465 (PFAM), SSF57667 (SUPERFAMILY) | |
| 3092 | lib_dib_Filter15_IDBA_contig_67516_plus2 | 25 | acid sphingomyelinase-like phosphodiesterase 3a-like | 162 | 1.07E-08 | 47.35% | 3 | F:sphingomyelin phosphodiesterase activity; F:hydrolase activity; P:sphingomyelin catabolic process | PTHR10340 (PANTHER), tmhmm (TMHMM) | |
| 3093 | lib_dib_Filter15_IDBA_contig_67626_plus1 | 10 | uncharacterized oxidoreductase tm_0325-like | 162 | 3.07E-13 | 64.15% | 3 | P:metabolic process; F:catalytic activity; F:nucleotide binding | IPR002198 (PFAM); IPR016040 (G3DSA:3.40.50.GENE3D); PTHR24322 (PANTHER), PTHR24322:SF0 (PANTHER), SSF51735 (SUPERFAMILY) | |
| 3094 | lib_dib_Filter15_IDBA_contig_67717_plus1 | 4625 | blastula protease-10 | 435 | 4.81E-54 | 49.35% | 4 | F:peptidase activity; P:protein metabolic process; P:catabolic process; F:binding | IPR001506 (PRINTS); IPR003582 (PFAM); IPR006026 (SMART); IPR024079 (G3DSA:3.40.390.GENE3D); PTHR10127 (PANTHER), SignalP-NN(euk) (SIGNALP), tmhmm (TMHMM), SSF55486 (SUPERFAMILY) | yes |
| 3095 | lib_dib_Filter15_IDBA_contig_67721_plus2 | 14 | anti-dorsalizing morphogenic protein precursor | 162 | 2.85E-25 | 89.10% | 12 | C:cell; P:growth; F:transferase activity; C:endoplasmic reticulum; C:protein complex; P:multicellular organismal development; P:lipid metabolic process; C:extracellular region; F:receptor binding; P:anatomical structure morphogenesis; P:cell differentiation; P:cell-cell signaling | IPR001839 (PFAM); IPR015615 (PANTHER); G3DSA:2.10.90.10 (GENE3D), PTHR11848:SF45 (PANTHER), SSF57501 (SUPERFAMILY) | |

| | | | | | | | | | | |
|---|---|---|---|---|---|---|---|---|---|---|
| 3096 | lib_dib_Filter15_IDBA_contig_67787_minus1 | 10 | neurogenic locus protein delta | 162 | 1.97E-22 | 58.15% | 2 | F:protein binding; F:calcium ion binding | IPR000152 (PROSITE); IPR000742 (SMART); IPR001881 (SMART); IPR013032 (PROSITE); IPR018097 (PROSITE); G3DSA:2.10.25.10 (GENE3D), PTHR24044 (PANTHER), SSF57196 (SUPERFAMILY) | |
| 3097 | lib_dib_Filter15_IDBA_contig_67824_plus1 | 25 | enolase-like protein eno4 | 162 | 2.39E-22 | 57.60% | 7 | C:cytosol; C:protein complex; F:binding; F:catalytic activity; P:carbohydrate metabolic process; P:generation of precursor metabolites and energy; P:catabolic process | IPR000941 (PANTHER); IPR020810 (PFAM); G3DSA:3.20.20.120 (GENE3D), SSF51604 (SUPERFAMILY) | |
| 3098 | lib_dib_Filter15_IDBA_contig_67826_minus2 | 17 | protocadherin alpha-8-like | 161 | 1.16E-23 | 56.55% | 3 | F:calcium ion binding; P:biological_process; C:plasma membrane | IPR002126 (PRINTS); IPR015919 (SUPERFAMILY); IPR020894 (PROSITE); PTHR24028 (PANTHER), PTHR24028:SF12 (PANTHER) | |
| 3099 | lib_dib_Filter15_IDBA_contig_67833_minus1 | 14 | starry isoform a | 162 | 3.84E-58 | 72.80% | 15 | P:anatomical structure morphogenesis; P:cellular component organization; P:cell differentiation; P:multicellular organismal development; F:protein binding; C:plasma membrane; P:signal transduction; P:biological_process; F:signal transducer activity; P:cell recognition; F:receptor activity; P:embryo development; P:regulation of biological process; P:cell-cell signaling; F:calcium ion binding | IPR002126 (PRINTS); IPR015919 (SUPERFAMILY); IPR020894 (PROSITE); PTHR24027 (PANTHER), PTHR24027:SF46 (PANTHER) | |
| 3100 | lib_dib_Filter15_IDBA_contig_67850_minus3 | 66 | ankyrin repeat protein | 314 | 4.65E-57 | 55.30% | 1 | F:protein binding | IPR002110 (PRINTS); IPR020683 (G3DSA:1.25.40.GENE3D); PTHR24198 (PANTHER), PF13637 (PFAM) | |
| 3101 | lib_dib_Filter15_IDBA_contig_67902_plus3 | 9 | isoform d | 161 | 1.87E-25 | 50.75% | 1 | F:hydrolase activity | IPR000560 (PFAM); G3DSA:3.40.50.1240 (GENE3D), PTHR11567 (PANTHER), PTHR11567:SF26 (PANTHER), SSF53254 (SUPERFAMILY) | |
| 3102 | lib_dib_Filter15_IDBA_contig_67927_minus1 | 14 | lysosomal acid lipase | 162 | 9.49E-66 | 74.90% | 5 | F:hydrolase activity; P:cell cycle; P:biological_process; P:catabolic process; P:lipid metabolic process | IPR000073 (PFAM); IPR006693 (PFAM); G3DSA:3.40.50.1820 (GENE3D), PTHR11005 (PANTHER), PTHR11005:SF0 (PANTHER), SSF53474 (SUPERFAMILY) | |
| 3103 | lib_dib_Filter15_IDBA_contig_67981_plus1 | 14 | heparanase isoform 1 | 161 | 5.43E-32 | 52.00% | 10 | F:protein binding; P:metabolic process; C:lysosome; F:hydrolase activity; P:regulation of biological process; P:response to external stimulus; P:response to stress; P:multicellular organismal development; C:cell; P:carbohydrate metabolic process | IPR005199 (PANTHER); IPR013781 (G3DSA:3.20.20.GENE3D); IPR017853 (SUPERFAMILY) | |
| 3104 | lib_dib_Filter15_IDBA_contig_68000_plus3 | 64 | ankyrin repeat and socs box protein 3 | 333 | 6.98E-57 | 56.25% | 1 | F:protein binding | IPR002110 (PRINTS); IPR020683 (G3DSA:1.25.40.GENE3D); PTHR24188 (PANTHER), PTHR24188:SF1 (PANTHER), PF13637 (PFAM) | |
| 3105 | lib_dib_Filter15_IDBA_contig_68001_plus2 | 33 | ankyrin repeat and socs box-containing 3 | 213 | 1.63E-31 | 54.40% | 1 | F:protein binding | IPR002110 (PRINTS); IPR020683 (G3DSA:1.25.40.GENE3D); PTHR24188 (PANTHER), PTHR24188:SF1 (PANTHER) | |
| 3106 | lib_dib_Filter15_IDBA_contig_68034_minus2 | 28 | retinoid-inducible serine carboxypeptidase-like | 161 | 4.65E-30 | 64.20% | 3 | F:peptidase activity; P:protein metabolic process; P:catabolic process | IPR001563 (PANTHER); G3DSA:3.40.50.1820 (GENE3D), PTHR11802:SF3 (PANTHER), SignalP-NN(euk) (SIGNALP), SSF53474 (SUPERFAMILY) | yes |
| 3107 | lib_dib_Filter15_IDBA_contig_68118_minus3 | 27 | arylsulfatase j-like | 172 | 2.64E-68 | 71.55% | 2 | P:metabolic process; F:hydrolase activity | IPR000917 (PFAM); IPR017849 (G3DSA:3.40.720.GENE3D); IPR017850 (SUPERFAMILY); IPR024607 (PROSITE); PTHR10342 (PANTHER), PTHR10342:SF19 (PANTHER) | |

| | | | | | | | | | | |
|---|---|---|---|---|---|---|---|---|---|---|
| 3108 | lib_dib_Filter15_IDBA_contig_68119_minus2 | 22 | von willebrand factor a domain containing 8 | 161 | 1.11E-81 | 91.10% | 6 | C:mitochondrion; P:nucleobase-containing compound metabolic process; P:catabolic process; F:nucleotide binding; F:hydrolase activity; C:extracellular region | IPR011704 (PFAM); G3DSA:3.40.50.300 (GENE3D), PTHR26086 (PANTHER), PTHR26086:SF0 (PANTHER), SSF52540 (SUPERFAMILY) | |
| 3109 | lib_dib_Filter15_IDBA_contig_6812_minus1 | 12190 | aplp_locmi ame: full=apolipophorins contains: ame: full=apolipophorin-2 ame: full=apolipophorin ii ame: full=apolp-2 contains: ame: full=apolipophorin-1 ame: full=apolipophorin i ame: full=apolp-1 flags: precursor | 257 | 8.47E-36 | 52.15% | 6 | P:lipid transport; F:lipid transporter activity; F:lipid binding; P:Wnt receptor signaling pathway; C:extracellular region; P:transport | IPR001846 (PFAM); PTHR11339 (PANTHER) | |
| 3110 | lib_dib_Filter15_IDBA_contig_68217_minus3 | 22 | dbh-like 1 | 160 | 4.07E-30 | 60.65% | 5 | P:primary metabolic process; F:binding; P:metabolic process; F:catalytic activity; | IPR000323 (G3DSA:2.60.120.GENE3D); IPR000945 (PRINTS); IPR008977 (SUPERFAMILY) | |
| 3111 | lib_dib_Filter15_IDBA_contig_68275_minus1 | 20 | epidermal growth factor receptor | 161 | 3.53E-27 | 65.95% | 39 | C:cell; C:plasma membrane; P:signal transduction; P:anatomical structure morphogenesis; P:cellular component organization; P:cell differentiation; P:multicellular organismal development; P:biosynthetic process; P:regulation of biological process; P:response to stress; P:DNA metabolic process; P:cellular protein modification process; P:protein metabolic process; P:catabolic process; P:biological_process; P:metabolic process; F:protein binding; F:receptor binding; P:cell cycle; P:response to abiotic stimulus; P:cell proliferation; P:reproduction; F:receptor activity; F:protein kinase activity; C:cytoplasmic membrane-bounded vesicle; C:protein complex; P:response to external stimulus; P:cell-cell signaling; F:actin binding; F:enzyme regulator activity; P:response to endogenous stimulus; P:translation; P:cell death; C:nucleus; P:carbohydrate metabolic process; F:signal transducer activity; C:endosome; P:lipid metabolic process; P:secondary metabolic | IPR001245 (PFAM); IPR011009 (SUPERFAMILY); G3DSA:1.10.510.10 (GENE3D), PTHR24416 (PANTHER), PTHR24416:SF90 (PANTHER) | |
| 3112 | lib_dib_Filter15_IDBA_contig_68361_minus3 | 22 | macrophage mannose receptor 1-like | 160 | 2.41E-13 | 47.00% | 1 | F:carbohydrate binding | IPR001304 (PFAM); IPR016186 (G3DSA:3.10.100.GENE3D); IPR016187 (SUPERFAMILY); PTHR22802 (PANTHER), PTHR22802:SF16 (PANTHER) | |
| 3113 | lib_dib_Filter15_IDBA_contig_68384_plus2 | 14 | protein kinase c-binding protein nell1-like | 160 | 3.73E-25 | 50.75% | 4 | F:protein binding; C:cell; C:extracellular region; F:calcium ion binding | IPR001007 (PFAM); IPR001881 (PFAM); G3DSA:2.10.70.10 (GENE3D), PTHR24042 (PANTHER), PTHR24042:SF0 (PANTHER), SSF57603 (SUPERFAMILY) | |
| 3114 | lib_dib_Filter15_IDBA_contig_68398_plus2 | 25 | bone morphogenetic protein 7-like | 160 | 3.04E-21 | 52.75% | 7 | P:anatomical structure morphogenesis; P:embryo development; P:multicellular organismal development; P:signal transduction; F:protein binding; F:receptor binding; P:growth | IPR001111 (PFAM); IPR015615 (PANTHER); PTHR11848:SF54 (PANTHER), SignalP-NN(euk) (SIGNALP) | yes |
| 3115 | lib_dib_Filter15_IDBA_contig_68416_plus1 | 13 | kallmann syndrome partial | 161 | 4.10E-38 | 47.10% | 1 | F:protein binding | IPR003961 (SUPERFAMILY); IPR013783 (G3DSA:2.60.40.GENE3D); PTHR14131 (PANTHER), PTHR14131:SF0 (PANTHER) | |
| 3116 | lib_dib_Filter15_IDBA_contig_68426_minus2 | 29 | protein | 160 | 2.50E-41 | 64.70% | 2 | F:nucleotide binding; P:protein metabolic process | IPR010448 (PANTHER); SignalP-NN(euk) (SIGNALP) | yes |

| | | | | | | | | | | |
|---|---|---|---|---|---|---|---|---|---|---|
| 3117 | lib_dib_Filter15_IDBA_contig_68549_minus3 | 48 | leucine rich repeat protein | 205 | 1.99E-13 | 58.70% | 1 | F:protein binding | IPR001611 (PROFILE); IPR003591 (SMART); G3DSA:3.80.10.10 (GENE3D), PTHR24367 (PANTHER), PF13855 (PFAM), SignalP-NN(euk) (SIGNALP), SSF52058 (SUPERFAMILY) | yes |
| 3118 | lib_dib_Filter15_IDBA_contig_68555_plus3 | 28 | zinc finger protein 467 | 160 | 5.09E-28 | 52.00% | 3 | F:binding; F:nucleic acid binding; C:intracellular | IPR007087 (PROSITE); IPR013087 (G3DSA:3.30.160.GENE3D); IPR015880 (SMART); PTHR11389 (PANTHER), PF13465 (PFAM), SSF57667 (SUPERFAMILY) | |
| 3119 | lib_dib_Filter15_IDBA_contig_68597_plus2 | 25 | short-chain dehydrogenase reductase sdr | 160 | 1.62E-44 | 65.10% | 3 | P:metabolic process; F:catalytic activity; F:nucleotide binding | IPR002198 (PFAM); IPR002347 (PRINTS); IPR016040 (G3DSA:3.40.50.GENE3D); PTHR24320 (PANTHER), PTHR24320:SF0 (PANTHER), SignalP-NN(euk) (SIGNALP), SSF51735 (SUPERFAMILY) | |
| 3120 | lib_dib_Filter15_IDBA_contig_68633_plus2 | 11 | partitioning defective 3 homolog | 160 | 3.37E-49 | 69.70% | 3 | C:cytoplasm; C:plasma membrane; F:protein binding | IPR001478 (PFAM); G3DSA:2.30.42.10 (GENE3D), PTHR16484 (PANTHER) | |
| 3121 | lib_dib_Filter15_IDBA_contig_68706_minus2 | 24 | molting fluid carboxypeptidase a | 186 | 1.53E-44 | 59.00% | 4 | F:peptidase activity; P:protein metabolic process; P:catabolic process; F:binding | IPR000834 (PFAM); G3DSA:3.40.630.10 (GENE3D), PTHR11705 (PANTHER), SSF53187 (SUPERFAMILY) | |
| 3122 | lib_dib_Filter15_IDBA_contig_68743_plus2 | 1164 | pancreatic triacylglycerol lipase-like | 466 | 1.17E-54 | 50.70% | 2 | F:catalytic activity; P:lipid metabolic process | IPR000734 (PRINTS); IPR013818 (PFAM); G3DSA:3.40.50.1820 (GENE3D), SignalP-NN(euk) (SIGNALP), tmhmm (TMHMM), SSF53474 (SUPERFAMILY) | |
| 3123 | lib_dib_Filter15_IDBA_contig_68744_plus1 | 1177 | pancreatic triacylglycerol lipase-like | 406 | 7.27E-55 | 50.85% | 2 | F:catalytic activity; P:lipid metabolic process | IPR000734 (PRINTS); IPR013818 (PFAM); G3DSA:3.40.50.1820 (GENE3D), SignalP-NN(euk) (SIGNALP), tmhmm (TMHMM), SSF53474 (SUPERFAMILY) | yes |
| 3124 | lib_dib_Filter15_IDBA_contig_68772_minus2 | 11 | ubiquitin | 159 | 9.32E-23 | 74.55% | 4 | C:cytoplasm; C:organelle; C:intracellular; F:protein binding | IPR000626 (PFAM); IPR019955 (PROFILE); IPR019956 (PRINTS); G3DSA:3.10.20.90 (GENE3D), PTHR10666 (PANTHER), SSF54236 (SUPERFAMILY) | |
| 3125 | lib_dib_Filter15_IDBA_contig_68798_plus3 | 263 | protein | 362 | 4.93E-65 | 63.00% | 3 | P:metabolic process; F:catalytic activity; F:nucleotide binding | IPR002198 (PFAM); IPR002347 (PRINTS); IPR016040 (G3DSA:3.40.50.GENE3D); PTHR24322 (PANTHER), PTHR24322:SF57 (PANTHER), SignalP-NN(euk) (SIGNALP), tmhmm (TMHMM), SSF51735 (SUPERFAMILY) | yes |
| 3126 | lib_dib_Filter15_IDBA_contig_68799_plus2 | 62 | short-chain dehydrogenase reductase sdr | 135 | 9.84E-15 | 73.50% | 2 | P:metabolic process; F:nucleotide binding | IPR016040 (G3DSA:3.40.50.GENE3D); PTHR24322 (PANTHER), PTHR24322:SF57 (PANTHER), SSF51735 (SUPERFAMILY) | |
| 3127 | lib_dib_Filter15_IDBA_contig_68800_plus2 | 87 | short-chain dehydrogenase reductase sdr | 210 | 8.02E-16 | 69.75% | 2 | P:metabolic process; F:nucleotide binding | IPR016040 (G3DSA:3.40.50.GENE3D); PTHR24322 (PANTHER), PTHR24322:SF57 (PANTHER), SSF51735 (SUPERFAMILY) | |
| 3128 | lib_dib_Filter15_IDBA_contig_68814_plus2 | 17 | protein o-glucosyltransferase 1 | 159 | 4.95E-65 | 75.90% | 6 | F:transferase activity; P:signal transduction; C:endoplasmic reticulum; P:biosynthetic process; P:cellular protein modification process; P:carbohydrate metabolic process | IPR006598 (PFAM); PTHR12203 (PANTHER), PTHR12203:SF6 (PANTHER) | |
| 3129 | lib_dib_Filter15_IDBA_contig_68837_minus1 | 22 | tyrosine-protein kinase src42a | 160 | 5.36E-17 | 51.65% | 1 | F:protein binding | IPR001611 (PFAM); IPR003591 (SMART); G3DSA:3.80.10.10 (GENE3D), PTHR24367 (PANTHER), PF13855 (PFAM), SSF52058 (SUPERFAMILY) | |
| 3130 | lib_dib_Filter15_IDBA_contig_68894_plus3 | 16 | ankyrin repeat and death domain-containing protein 1a | 159 | 8.81E-41 | 62.90% | 1 | F:protein binding | IPR002110 (PRINTS); IPR020683 (G3DSA:1.25.40.GENE3D); PTHR24125 (PANTHER) | |
| 3131 | lib_dib_Filter15_IDBA_contig_68915_minus3 | 11 | xanthine dehydrogenase oxidase | 159 | 1.30E-49 | 67.90% | 3 | F:binding; F:catalytic activity; P:metabolic process | IPR008274 (G3DSA:3.30.365.GENE3D); PTHR11908 (PANTHER), PTHR11908:SF5 (PANTHER) | |
| 3132 | lib_dib_Filter15_IDBA_contig_68946_minus3 | 13 | serine threonine-protein phosphatase 6 regulatory ankyrin repeat subunit c | 159 | 2.26E-37 | 61.95% | 1 | F:protein binding | IPR002110 (PFAM); IPR020683 (G3DSA:1.25.40.GENE3D); PTHR24158 (PANTHER), PTHR24158:SF0 (PANTHER) | |

| | | | | | | | | | | |
|---|---|---|---|---|---|---|---|---|---|---|
| 3133 | lib_dib_Filter15_IDBA_contig_69127_minus3 | 20 | protein fem-1 homolog c | 158 | 2.86E-84 | 89.05% | 3 | C:cytoplasm; P:cellular protein modification process; F:protein binding | IPR002110 (PRINTS); IPR020683 (G3DSA:1.25.40.GENE3D); PTHR24173 (PANTHER), PTHR24173:SF0 (PANTHER) | |
| 3134 | lib_dib_Filter15_IDBA_contig_69183_plus2 | 18 | dimethylaniline monooxygenase | 159 | 2.85E-18 | 62.90% | 6 | P:metabolic process; F:catalytic activity; C:cell; P:primary metabolic process; C:endoplasmic reticulum; F:nucleotide binding | IPR020946 (PFAM); G3DSA:3.50.50.60 (GENE3D), PTHR23023 (PANTHER), PTHR23023:SF4 (PANTHER), SSF51905 (SUPERFAMILY) | |
| 3135 | lib_dib_Filter15_IDBA_contig_69186_plus2 | 19 | variable lymphocyte receptor diversity region | 159 | 9.00E-13 | 50.70% | 1 | P:biological_process | IPR003591 (SMART); G3DSA:3.80.10.10 (GENE3D), PTHR24365 (PANTHER), PF13855 (PFAM), SignalP-NN(euk) (SIGNALP), tmhmm (TMHMM), SSF52058 (SUPERFAMILY) | |
| 3136 | lib_dib_Filter15_IDBA_contig_69287_minus1 | 14 | zinc finger protein 271 (zinc finger protein 7) (zinc finger protein znfphex133) (epstein-barr virus-induced zinc finger protein) (znf-eb) (ct-zfp48) (zinc finger protein | 159 | 1.51E-17 | 60.05% | 3 | F:binding; F:nucleic acid binding; C:intracellular | IPR007087 (PFAM); IPR013087 (G3DSA:3.30.160.GENE3D); IPR015880 (SMART); PTHR24375 (PANTHER), PTHR24375:SF0 (PANTHER), PF13465 (PFAM), SSF57667 (SUPERFAMILY) | |
| 3137 | lib_dib_Filter15_IDBA_contig_69368_minus3 | 40 | tyrosine-protein kinase ryk | 158 | 1.17E-37 | 64.75% | 17 | F:receptor binding; P:signal transduction; F:protein kinase activity; C:cytoplasm; F:receptor activity; P:behavior; P:response to external stimulus; P:anatomical structure morphogenesis; P:multicellular organismal development; C:plasma membrane; F:protein binding; C:nucleus; P:cellular component organization; P:cell differentiation; P:regulation of biological process; F:nucleotide binding; P:cellular protein modification process | IPR000719 (PROFILE); IPR001245 (PFAM); IPR011009 (SUPERFAMILY); G3DSA:3.30.200.20 (GENE3D), PTHR24416 (PANTHER), PTHR24416:SF85 (PANTHER) | |
| 3138 | lib_dib_Filter15_IDBA_contig_69396_minus1 | 201 | neuronal acetylcholine receptor subunit alpha-7-like | 513 | 1.16E-62 | 56.35% | 6 | P:regulation of biological process; P:biological_process; C:cell; P:behavior; P:ion transport; F:ion channel activity | IPR006029 (SUPERFAMILY); IPR006201 (PRINTS); IPR006202 (G3DSA:2.70.170.GENE3D); IPR018000 (PROSITE); G3DSA:1.20.120.370 (GENE3D), SignalP-NN(euk) (SIGNALP), tmhmm (TMHMM) | |
| 3139 | lib_dib_Filter15_IDBA_contig_69412_plus2 | 21 | glycoprotein-n-acetylgalactosamine 3-beta-galactosyltransferase 1-like | 158 | 1.63E-79 | 77.30% | 10 | P:anatomical structure morphogenesis; P:multicellular organismal development; F:transferase activity; C:cell; C:Golgi apparatus; F:binding; P:cellular protein modification process; P:cell differentiation; P:biosynthetic process; P:carbohydrate metabolic process | IPR003378 (PFAM); IPR026842 (PTHR23033:PANTHER); PTHR23033 (PANTHER) | |
| 3140 | lib_dib_Filter15_IDBA_contig_69419_minus3 | 3037 | protein | 222 | 9.34E-27 | 50.00% | 9 | F:transferase activity; C:cell surface; F:hormone activity; F:metalloendopeptidase inhibitor activity; P:regulation of blood vessel size; P:negative regulation of catalytic activity; F:enzyme inhibitor activity; C:extracellular region; P:regulation of blood pressure | SignalP-NN(euk) (SIGNALP) | yes |
| 3141 | lib_dib_Filter15_IDBA_contig_69420_minus3 | 1883 | protein | 222 | 1.66E-27 | 49.85% | 9 | F:transferase activity; C:cell surface; F:hormone activity; F:metalloendopeptidase inhibitor activity; P:regulation of blood vessel size; P:negative regulation of catalytic activity; F:enzyme inhibitor activity; C:extracellular region; P:regulation of blood pressure | SignalP-NN(euk) (SIGNALP) | yes |

| | | | | | | | | |
|---|---|---|---|---|---|---|---|---|
| 3142 | lib_dib_Filter15_IDBA_contig_69470_plus2 | 31 | immunoglobulin kappa light chain | 158 | 6.62E-49 | 90.10% | 3 | P:cell differentiation; P:multicellular organismal development; F:protein binding | IPR003006 (PROSITE); IPR003597 (PFAM); IPR007110 (PROFILE); IPR013783 (G3DSA:2.60.40.GENE3D); PTHR23266 (PANTHER), PTHR23266:SF1 (PANTHER), SSF48726 (SUPERFAMILY) |
| 3143 | lib_dib_Filter15_IDBA_contig_69529_plus2 | 39 | alpha-galactosidase a | 270 | 1.54E-123 | 70.75% | 2 | F:hydrolase activity; P:carbohydrate metabolic process | IPR000111 (PFAM); IPR002241 (PRINTS); IPR013785 (G3DSA:3.20.20.GENE3D); IPR017853 (SUPERFAMILY); PTHR11452 (PANTHER), SignalP-NN(euk) (SIGNALP), tmhmm (TMHMM) |
| 3144 | lib_dib_Filter15_IDBA_contig_69566_plus1 | 13 | zinc finger protein 846 | 158 | 1.39E-31 | 55.55% | 3 | F:binding; F:nucleic acid binding; C:intracellular | IPR007087 (PFAM); IPR013087 (G3DSA:3.30.160.GENE3D); IPR015880 (SMART); PTHR11389 (PANTHER), PF13894 (PFAM), SSF57667 (SUPERFAMILY) |
| 3145 | lib_dib_Filter15_IDBA_contig_69612_plus3 | 15 | cre-lrp-1 protein | 158 | 1.31E-13 | 49.10% | 8 | P:biological_process; C:organelle; C:intracellular; P:regulation of biological process; P:growth; C:cell; C:cytoplasm; F:protein binding | IPR002172 (G3DSA:4.10.400.GENE3D); PR00261 (PRINTS), PTHR10529 (PANTHER), PTHR10529:SF113 (PANTHER) |
| 3146 | lib_dib_Filter15_IDBA_contig_69676_plus3 | 643 | cub and sushi domain-containing protein 3- partial | 120 | 4.07E-19 | 60.15% | 1 | F:hydrolase activity | IPR000859 (G3DSA:2.60.120.GENE3D); PTHR10127 (PANTHER) |
| 3147 | lib_dib_Filter15_IDBA_contig_69677_plus3 | 837 | procollagen c-endopeptidase | 122 | 4.59E-11 | 60.15% | 1 | F:hydrolase activity | IPR000859 (G3DSA:2.60.120.GENE3D); PTHR10127 (PANTHER) |
| 3148 | lib_dib_Filter15_IDBA_contig_69678_plus2 | 124 | extracellular serine proteinase-like | 105 | 1.25E-16 | 61.95% | 1 | F:peptidase activity | IPR000859 (G3DSA:2.60.120.GENE3D); PTHR10127 (PANTHER) |
| 3149 | lib_dib_Filter15_IDBA_contig_69679_plus3 | 1020 | tolloid-like protein 2-like | 108 | 2.88E-18 | 60.00% | 2 | F:binding; F:peptidase activity | IPR000859 (G3DSA:2.60.120.GENE3D); PTHR10127 (PANTHER) |
| 3150 | lib_dib_Filter15_IDBA_contig_69698_minus1 | 18 | neurogenic locus protein delta | 158 | 3.62E-17 | 56.10% | 2 | F:protein binding; F:calcium ion binding | IPR000742 (PFAM); IPR001881 (SMART); IPR013032 (PROSITE); G3DSA:2.10.25.10 (GENE3D), PTHR24033 (PANTHER), tmhmm (TMHMM), SSF57196 (SUPERFAMILY) |
| 3151 | lib_dib_Filter15_IDBA_contig_69792_minus3 | 20 | upf0364 protein c6orf211 homolog | 157 | 3.67E-48 | 68.05% | 3 | F:molecular_function; P:biological_process; C:cellular_component | IPR002791 (PFAM); PTHR12260 (PANTHER) |
| 3152 | lib_dib_Filter15_IDBA_contig_69837_minus3 | 21 | protein | 157 | 1.31E-27 | 73.95% | 2 | F:calcium ion binding; F:protein binding | IPR000152 (PROSITE); IPR000742 (PFAM); IPR001881 (SMART); IPR013032 (PROSITE); IPR018097 (PROSITE); PR00010 (PRINTS), G3DSA:2.10.25.10 (GENE3D), G3DSA:3.10.250.10 (GENE3D), PTHR24048 (PANTHER), PTHR24048:SF15 (PANTHER), SSF57196 (SUPERFAMILY) |
| 3153 | lib_dib_Filter15_IDBA_contig_69981_minus2 | 22 | mgc79752 protein | 157 | 2.09E-26 | 58.10% | 3 | P:metabolic process; F:catalytic activity; F:nucleotide binding | IPR002198 (PRINTS); IPR002347 (PRINTS); IPR016040 (G3DSA:3.40.50.GENE3D); IPR020904 (PROSITE); PTHR24322 (PANTHER), SSF51735 (SUPERFAMILY) |
| 3154 | lib_dib_Filter15_IDBA_contig_7000_plus2 | 1242 | deleted in malignant brain tumors 1 | 1180 | 0 | 44.90% | 3 | F:binding; C:cell; F:receptor activity | IPR001190 (PRINTS); IPR017448 (SMART); G3DSA:3.10.250.10 (GENE3D), PTHR19331 (PANTHER), PTHR19331:SF118 (PANTHER) |

| | | | | | | | | | | |
|---|---|---|---|---|---|---|---|---|---|---|
| 3155 | lib_dib_Filter15_IDBA_contig_70236_plus1 | 22 | loc100036993 protein | 157 | 5.06E-80 | 91.25% | 23 | P:cellular protein modification process; P:transport; C:Golgi apparatus; F:binding; C:nuclear chromosome; P:cytoskeleton organization; P:cell cycle; C:nuclear envelope; P:regulation of biological process; P:DNA metabolic process; P:organelle organization; P:biosynthetic process; C:cell; P:protein transport; P:biological_process; F:transferase activity; C:protein complex; C:chromosome; C:microtubule organizing center; ; C:cytoplasm; F:protein binding; P:growth | IPR002110 (PRINTS); IPR020683 (G3DSA:1.25.40.GENE3D); PTHR24180 (PANTHER), PTHR24180:SF3 (PANTHER) | |
| 3156 | lib_dib_Filter15_IDBA_contig_70239_minus1 | 30 | protein wnt-4 | 157 | 3.77E-71 | 66.15% | 9 | P:signal transduction; P:response to external stimulus; P:anatomical structure morphogenesis; P:response to stress; P:multicellular organismal development; P:growth; P:embryo development; F:receptor binding; C:proteinaceous extracellular matrix | IPR005817 (PRINTS); SignalP-NN(euk) (SIGNALP), tmhmm (TMHMM) | yes |
| 3157 | lib_dib_Filter15_IDBA_contig_70349_plus2 | 84 | leucine-rich repeat neuronal protein 3 | 280 | 9.01E-11 | 51.50% | 1 | F:protein binding | IPR001611 (PFAM); IPR003591 (SMART); IPR025875 (PFAM); G3DSA:3.80.10.10 (GENE3D), PTHR24365 (PANTHER), SSF52058 (SUPERFAMILY) | |
| 3158 | lib_dib_Filter15_IDBA_contig_70350_plus2 | 37 | leucine-rich repeat neuronal protein 3 | 178 | 1.20E-11 | 51.55% | 1 | F:protein binding | IPR001611 (PFAM); IPR003591 (SMART); IPR025875 (PFAM); PR00019 (PRINTS), G3DSA:3.80.10.10 (GENE3D), PTHR24367 (PANTHER), SignalP-NN(euk) (SIGNALP), tmhmm (TMHMM), SSF52058 (SUPERFAMILY) | yes |
| 3159 | lib_dib_Filter15_IDBA_contig_70368_plus3 | 51 | peptidyl-prolyl cis-trans isomerase-like 4 | 156 | 7.28E-69 | 86.35% | 6 | P:protein metabolic process; F:RNA binding; P:cellular protein modification process; C:nucleus; F:catalytic activity; F:nucleotide binding | IPR002130 (PRINTS); G3DSA:2.40.100.10 (GENE3D), PTHR11071 (PANTHER), PTHR11071:SF27 (PANTHER), SignalP-NN(euk) (SIGNALP) | |
| 3160 | lib_dib_Filter15_IDBA_contig_70402_plus1 | 19 | semaphorin 5a-like protein | 157 | 8.40E-49 | 61.85% | 8 | P:anatomical structure morphogenesis; P:cellular component organization; P:cell differentiation; P:multicellular organismal development; F:receptor activity; C:cell; P:signal transduction; F:protein binding | IPR001627 (PFAM); IPR015943 (G3DSA:2.130.10.GENE3D); IPR027231 (PANTHER); PTHR11036:SF3 (PANTHER) | |
| 3161 | lib_dib_Filter15_IDBA_contig_70478_minus2 | 39 | peptidylglycine alpha-amidating monooxygenase | 156 | 1.01E-49 | 60.75% | 3 | F:catalytic activity; F:binding; P:metabolic process | IPR000323 (G3DSA:2.60.120.GENE3D); IPR008977 (SUPERFAMILY); IPR014784 (G3DSA:2.60.120.GENE3D); PTHR10680 (PANTHER), PTHR10680:SF11 (PANTHER), PF03712 (PFAM) | |
| 3162 | lib_dib_Filter15_IDBA_contig_70615_plus2 | 14 | oxysterol-binding protein 1 | 156 | 9.01E-38 | 75.70% | 5 | C:cytoplasm; P:transport; C:organelle; C:intracellular; F:protein binding | IPR002110 (SMART); IPR020683 (G3DSA:1.25.40.GENE3D); PTHR24193 (PANTHER), PTHR24193:SF3 (PANTHER), SignalP-NN(euk) (SIGNALP), tmhmm (TMHMM) | |
| 3163 | lib_dib_Filter15_IDBA_contig_70821_minus2 | 14 | tight junction protein zo-1-like | 161 | 1.06E-37 | 69.15% | 1 | F:protein binding | IPR001478 (PFAM); G3DSA:2.30.42.10 (GENE3D), PTHR13865 (PANTHER) | |
| 3164 | lib_dib_Filter15_IDBA_contig_70883_plus3 | 46 | beta- -n-acetylgalactosaminyltransferase bre-4 | 177 | 7.24E-64 | 72.90% | 8 | P:signal transduction; P:anatomical structure morphogenesis; P:symbiosis, encompassing mutualism through parasitism; P:biological_process; P:biosynthetic process; P:cellular protein modification process; P:carbohydrate metabolic process; F:transferase activity | IPR003859 (PRINTS); G3DSA:3.90.550.10 (GENE3D), PF13733 (PFAM), SSF53448 (SUPERFAMILY) | |

| | | | | | | | | | | |
|---|---|---|---|---|---|---|---|---|---|---|
| 3165 | lib_dib_Filter15_IDBA_contig_70923_plus3 | 18 | angiopoietin-related protein 4-like | 155 | 7.81E-15 | 61.05% | 10 | P:regulation of biological process; P:cell death; C:extracellular space; P:biological_process; P:cellular component organization; F:enzyme regulator activity; P:response to external stimulus; P:cell communication; P:response to stress; P:lipid metabolic process | IPR002181 (PFAM); IPR014716 (G3DSA:3.90.215.GENE3D); PTHR19143 (PANTHER), SignalP-NN(euk) (SIGNALP), tmhmm (TMHMM) | yes |
| 3166 | lib_dib_Filter15_IDBA_contig_7096_minus3 | 1427 | ---NA--- | 630 | | | 0 | - | IPR008160 (PFAM); PTHR24023 (PANTHER), PTHR24023:SF164 (PANTHER), tmhmm (TMHMM) | |
| 3167 | lib_dib_Filter15_IDBA_contig_71000_minus2 | 14 | deleted in malignant brain tumors 1 protein | 155 | 2.69E-29 | 56.30% | 2 | F:receptor activity; C:cell | IPR001190 (PRINTS); IPR017448 (SMART); G3DSA:3.10.250.10 (GENE3D), PTHR19331 (PANTHER) | |
| 3168 | lib_dib_Filter15_IDBA_contig_71034_minus2 | 277 | protein | 155 | 3.25E-27 | 49.88% | 3 | F:transferase activity; C:integral to membrane; C:membrane | no IPS match | |
| 3169 | lib_dib_Filter15_IDBA_contig_71048_plus2 | 17 | macrophage migration inhibitory factor | 155 | 5.17E-08 | 47.45% | 50 | P:negative regulation of DNA damage response, signal transduction by p53 class mediator; F:phenylpyruvate tautomerase activity; F:cytokine receptor binding; F:cytokine activity; C:cell surface; P:positive regulation of peptidyl-tyrosine phosphorylation; P:cell proliferation; F:cell surface binding; F:dopachrome isomerase activity; P:prostaglandin biosynthetic process; P:positive regulation of cytokine secretion; P:positive regulation of ERK1 and ERK2 cascade; P:protein homotrimerization; C:extracellular space; P:positive regulation of protein kinase A signaling cascade; F:chemoattractant activity; P:positive regulation of B cell proliferation; P:negative regulation of gene expression; P:positive regulation of fibroblast proliferation; P:negative regulation of cell aging; C:cytoplasm; P:inflammatory response; P:cell surface receptor signaling pathway; P:negative regulation of cell cycle arrest; P:positive regulation of peptidyl-serine phosphorylation; P:innate immune response; P:negative regulation of apoptotic process; P:cell differentiation; P:preantral ovarian | IPR001398 (PRODOM); IPR014347 (SUPERFAMILY); G3DSA:3.30.429.10 (GENE3D), SignalP-NN(euk) (SIGNALP) | |
| 3170 | lib_dib_Filter15_IDBA_contig_71097_minus3 | 11 | zinc finger protein 410 | 155 | 3.60E-29 | 77.40% | 4 | ; F:nucleic acid binding; C:intracellular; F:binding | IPR007087 (PROSITE); IPR013087 (G3DSA:3.30.160.GENE3D); IPR015880 (SMART); PTHR11389 (PANTHER), PTHR11389:SF363 (PANTHER), PF13465 (PFAM), SSF57667 (SUPERFAMILY) | |
| 3171 | lib_dib_Filter15_IDBA_contig_71222_plus3 | 14 | aldehyde oxidase | 154 | 7.93E-46 | 66.05% | 3 | F:binding; F:catalytic activity; P:metabolic process | IPR008274 (G3DSA:3.30.365.GENE3D); PTHR11908 (PANTHER), PTHR11908:SF5 (PANTHER) | |
| 3172 | lib_dib_Filter15_IDBA_contig_71226_plus2 | 112 | metalloproteinase inhibitor 3 | 236 | 2.66E-06 | 40.60% | 1 | F:enzyme regulator activity | IPR001820 (PANTHER); IPR008993 (SUPERFAMILY); G3DSA:2.40.50.120 (GENE3D), PTHR11844:SF9 (PANTHER), SignalP-NN(euk) (SIGNALP), tmhmm (TMHMM) | yes |

| | | | | | | | | | | |
|---|---|---|---|---|---|---|---|---|---|---|
| 3173 | lib_dib_Filter15_IDBA_contig_71240_minus2 | 27 | frizzled-10 | 155 | 1.37E-48 | 68.75% | 13 | P:signal transduction; P:regulation of biological process; P:primary metabolic process; F:protein binding; P:multicellular organismal development; P:metabolic process; P:reproduction; C:cytoplasm; F:receptor activity; P:embryo development; P:biological_process; C:cell; C:plasma membrane | IPR015526 (PANTHER); IPR020067 (G3DSA:1.10.2000.GENE3D); SignalP-NN(euk) (SIGNALP) | yes |
| 3174 | lib_dib_Filter15_IDBA_contig_71244_plus1 | 915 | low-density lipoprotein receptor-related protein 2-like | 282 | 4.78E-08 | 50.50% | 2 | C:cell; F:protein binding | IPR002172 (G3DSA:4.10.400.GENE3D); PR00261 (PRINTS), PTHR10529 (PANTHER), PTHR10529:SF161 (PANTHER), SignalP-NN(euk) (SIGNALP) | yes |
| 3175 | lib_dib_Filter15_IDBA_contig_71304_minus3 | 7862 | aldehyde dehydrogenase 2 family | 368 | 1.57E-146 | 81.55% | 11 | P:metabolic process; P:response to endogenous stimulus; P:multicellular organismal development; P:biological_process; F:protein binding; P:catabolic process; C:mitochondrion; P:response to biotic stimulus; F:catalytic activity; P:response to stress; C:nucleus | IPR015590 (PFAM); IPR016160 (PROSITE); IPR016161 (SUPERFAMILY); IPR016162 (G3DSA:3.40.605.GENE3D); PTHR11699 (PANTHER), PTHR11699:SF46 (PANTHER) | |
| 3176 | lib_dib_Filter15_IDBA_contig_71305_minus3 | 14574 | retinal dehydrogenase 1-like | 720 | 0 | 81.45% | 5 | C:cytoplasm; F:catalytic activity; P:lipid metabolic process; P:secondary metabolic process; P:metabolic process | IPR015590 (PFAM); IPR016160 (PROSITE); IPR016161 (SUPERFAMILY); IPR016162 (G3DSA:3.40.605.GENE3D); IPR016163 (G3DSA:3.40.309.GENE3D); PTHR11699 (PANTHER), PTHR11699:SF46 (PANTHER) | |
| 3177 | lib_dib_Filter15_IDBA_contig_71309_plus1 | 211 | hypothetical protein CAPTEDRAFT_186176 | 422 | 3.22E-80 | 57.00% | 1 | F:calcium ion binding | IPR002048 (SMART); IPR011992 (G3DSA:1.10.238.GENE3D); IPR018247 (PROSITE); IPR027241 (PTHR10827:PANTHER); PTHR10827 (PANTHER), SignalP-NN(euk) (SIGNALP), SSF47473 (SUPERFAMILY) | |
| 3178 | lib_dib_Filter15_IDBA_contig_71310_plus1 | 226 | calumenin isoform 1 | 443 | 5.90E-80 | 56.70% | 1 | F:calcium ion binding | IPR002048 (SMART); IPR011992 (G3DSA:1.10.238.GENE3D); IPR018247 (PROSITE); IPR027241 (PTHR10827:PANTHER); PTHR10827 (PANTHER), SSF47473 (SUPERFAMILY) | |
| 3179 | lib_dib_Filter15_IDBA_contig_71311_plus2 | 1062 | galectin-3-binding protein | 386 | 6.24E-27 | 64.05% | 3 | F:hydrolase activity; F:receptor activity; C:cell | IPR001190 (PRINTS); IPR017448 (SMART); G3DSA:3.10.250.10 (GENE3D), PTHR19331 (PANTHER), SignalP-NN(euk) (SIGNALP), tmhmm (TMHMM) | yes |
| 3180 | lib_dib_Filter15_IDBA_contig_71312_plus2 | 72 | galectin-3-binding protein precursor | 102 | 9.56E-16 | 66.85% | 6 | P:transport; F:binding; P:regulation of biological process; P:biological_process; F:receptor activity; C:cell | IPR001190 (PRINTS); IPR017448 (SMART); G3DSA:3.10.250.10 (GENE3D), PTHR19331 (PANTHER), PTHR19331:SF118 (PANTHER), SignalP-NN(euk) (SIGNALP), tmhmm (TMHMM) | yes |
| 3181 | lib_dib_Filter15_IDBA_contig_71322_minus3 | 15 | chitinase domain-containing protein 1 | 154 | 4.42E-43 | 77.65% | 6 | F:hydrolase activity; P:carbohydrate metabolic process; P:catabolic process; F:binding; C:lysosome; C:extracellular region | PTHR11177 (PANTHER) | |
| 3182 | lib_dib_Filter15_IDBA_contig_71358_minus3 | 13 | zinc finger protein 271-like | 154 | 1.80E-33 | 74.25% | 3 | F:binding; F:nucleic acid binding; C:intracellular | IPR007087 (PROSITE); IPR013087 (G3DSA:3.30.160.GENE3D); IPR015880 (SMART); PTHR24402 (PANTHER), PF13465 (PFAM), SSF57667 (SUPERFAMILY) | |

| | | | | | | | | | |
|---|---|---|---|---|---|---|---|---|---|
| 3183 | lib_dib_Filter15_IDBA_contig_71367_plus1 | 26 | bcl-6 corepressor isoform 1 | 155 | 8.05E-34 | 66.30% | 13 | P:biological_process; P:regulation of biological process; ; F:protein binding; F:transcription regulator activity; P:multicellular organismal development; P:organelle organization; P:cellular protein modification process; P:anatomical structure morphogenesis; F:DNA binding; F:catalytic activity; C:nucleus; C:protein complex | IPR002110 (PRINTS); IPR020683 (G3DSA:1.25.40.GENE3D); PTHR24117 (PANTHER), PTHR24117:SF0 (PANTHER) |
| 3184 | lib_dib_Filter15_IDBA_contig_7141_minus1 | 776 | latrophilin 3-like | 340 | 1.80E-40 | 54.80% | 3 | F:receptor activity; P:signal transduction; C:cell | IPR000832 (PFAM); IPR017981 (PROFILE); G3DSA:1.20.1070.10 (GENE3D), PTHR12011 (PANTHER), PTHR12011:SF92 (PANTHER), SignalP-NN(euk) (SIGNALP), tmhmm (TMHMM), SSF81321 (SUPERFAMILY) |
| 3185 | lib_dib_Filter15_IDBA_contig_71449_minus1 | 48 | zinc finger protein 271-like | 175 | 5.05E-37 | 62.35% | 3 | F:binding; F:nucleic acid binding; C:intracellular | IPR007087 (PROSITE); IPR013087 (G3DSA:3.30.160.GENE3D); IPR015880 (SMART); PTHR24382 (PANTHER), PTHR24382:SF0 (PANTHER), PF13465 (PFAM), SSF57667 (SUPERFAMILY) |
| 3186 | lib_dib_Filter15_IDBA_contig_71450_minus1 | 163 | zinc finger protein 271-like | 363 | 1.52E-102 | 63.85% | 3 | F:binding; F:nucleic acid binding; C:intracellular | IPR007087 (PROSITE); IPR013087 (G3DSA:3.30.160.GENE3D); IPR015880 (SMART); PTHR24377 (PANTHER), PTHR24377:SF0 (PANTHER), PF13465 (PFAM), SSF57667 (SUPERFAMILY) |
| 3187 | lib_dib_Filter15_IDBA_contig_71519_plus1 | 12 | bone morphogenetic protein 10 | 155 | 1.45E-08 | 46.70% | 3 | P:biological_process; P:multicellular organismal development; F:receptor binding | IPR001839 (PFAM); IPR015615 (PANTHER); G3DSA:2.10.90.10 (GENE3D), PTHR11848:SF71 (PANTHER), SSF57501 (SUPERFAMILY) |
| 3188 | lib_dib_Filter15_IDBA_contig_71542_minus1 | 17 | chondroitin sulfate synthase 2-like | 155 | 1.74E-24 | 58.20% | 2 | F:transferase activity; C:Golgi apparatus | IPR008428 (PANTHER); PTHR12369:SF5 (PANTHER) |
| 3189 | lib_dib_Filter15_IDBA_contig_71579_plus2 | 12 | chorion peroxidase-like | 154 | 2.96E-31 | 54.60% | 5 | F:catalytic activity; F:antioxidant activity; P:response to stress; F:binding; P:metabolic process | IPR002007 (G3DSA:1.10.640.GENE3D); IPR010255 (SUPERFAMILY); PTHR11475 (PANTHER) |
| 3190 | lib_dib_Filter15_IDBA_contig_71583_minus2 | 13 | peptidyl-prolyl cis-trans isomerase h | 154 | 3.27E-87 | 90.00% | 9 | C:cytoplasm; P:anatomical structure morphogenesis; P:multicellular organismal development; P:protein metabolic process; C:nucleus; C:nucleoplasm; F:binding; P:cellular protein modification process; F:catalytic activity | IPR002130 (PRINTS); IPR020892 (PROSITE); IPR024936 (PIR); G3DSA:2.40.100.10 (GENE3D), PTHR11071 (PANTHER), PTHR11071:SF58 (PANTHER) |
| 3191 | lib_dib_Filter15_IDBA_contig_71588_plus1 | 877 | lysyl oxidase homolog 2 precursor | 414 | 5.63E-24 | 61.90% | 7 | P:multicellular organismal development; ; C:extracellular region; P:cellular component organization; P:regulation of biological process; F:receptor activity; C:cell | IPR001190 (PRINTS); IPR017448 (SMART); G3DSA:3.10.250.10 (GENE3D), PTHR19331 (PANTHER), PTHR19331:SF118 (PANTHER), tmhmm (TMHMM) |
| 3192 | lib_dib_Filter15_IDBA_contig_71589_plus1 | 28 | macrophage scavenger receptor types i and ii isoform 1 | 137 | 1.84E-12 | 69.35% | 10 | P:transport; F:binding; P:regulation of biological process; P:metabolic process; F:catalytic activity; F:peptidase activity; P:biological_process; C:extracellular region; C:cell; F:receptor activity | IPR001190 (PFAM); IPR017448 (SUPERFAMILY); G3DSA:3.10.250.10 (GENE3D), PTHR19331 (PANTHER), PTHR19331:SF118 (PANTHER) |
| 3193 | lib_dib_Filter15_IDBA_contig_71590_plus2 | 785 | lysyl oxidase homolog 2 precursor | 369 | 9.75E-26 | 62.20% | 9 | P:anatomical structure morphogenesis; P:multicellular organismal development; P:regulation of biological process; ; P:biological_process; C:proteinaceous extracellular matrix; P:cell differentiation; F:receptor activity; C:cell | IPR001190 (PRINTS); IPR017448 (SMART); G3DSA:3.10.250.10 (GENE3D), PTHR19331 (PANTHER), PTHR19331:SF118 (PANTHER), tmhmm (TMHMM) |
| 3194 | lib_dib_Filter15_IDBA_contig_71591_plus2 | 24 | deleted in malignant brain tumors 1 | 154 | 4.17E-25 | 52.80% | 2 | F:receptor activity; C:cell | IPR001190 (PRINTS); IPR017448 (SMART); G3DSA:3.10.250.10 (GENE3D), PTHR19331 (PANTHER) |

| | | | | | | | | | | |
|---|---|---|---|---|---|---|---|---|---|---|
| 3195 | lib_dib_Filter15_IDBA_contig_71612_plus1 | 20 | blastula protease-10 | 154 | 2.35E-57 | 63.40% | 4 | F:peptidase activity; P:protein metabolic process; P:catabolic process; F:binding | IPR001506 (PRINTS); IPR006026 (SMART); IPR024079 (G3DSA:3.40.390.GENE3D); PTHR10127 (PANTHER), SSF55486 (SUPERFAMILY) | |
| 3196 | lib_dib_Filter15_IDBA_contig_71739_plus3 | 19 | probable proline racemase-like | 154 | 6.27E-42 | 72.85% | 1 | F:catalytic activity | IPR008794 (PFAM); G3DSA:3.10.310.10 (GENE3D), PTHR18835 (PANTHER), SSF54506 (SUPERFAMILY) | |
| 3197 | lib_dib_Filter15_IDBA_contig_71747_plus2 | 23 | uncharacterized family 31 glucosidase kiaa1161-like | 154 | 1.93E-40 | 68.95% | 2 | F:catalytic activity; P:carbohydrate metabolic process | IPR000322 (PANTHER); IPR017853 (SUPERFAMILY); PTHR22762:SF2 (PANTHER), SSF51011 (SUPERFAMILY) | |
| 3198 | lib_dib_Filter15_IDBA_contig_71751_plus3 | 30 | megakaryocyte-associated tyrosine-protein kinase | 187 | 4.89E-40 | 50.05% | 1 | F:calcium ion binding | IPR000436 (PFAM); IPR001881 (SMART); IPR018097 (PROSITE); G3DSA:2.10.25.10 (GENE3D), G3DSA:2.10.70.10 (GENE3D), PTHR24032 (PANTHER), PTHR24032:SF0 (PANTHER), SSF57196 (SUPERFAMILY) | |
| 3199 | lib_dib_Filter15_IDBA_contig_71773_minus3 | 306 | protease inhibitor epi11 | 146 | 5.64E-06 | 59.40% | 1 | F:protein binding | IPR002350 (PFAM); G3DSA:3.30.60.30 (GENE3D), PTHR21312 (PANTHER), PTHR21312:SF13 (PANTHER), SignalP-NN(euk) (SIGNALP), tmhmm (TMHMM), SSF100895 (SUPERFAMILY) | yes |
| 3200 | lib_dib_Filter15_IDBA_contig_71798_plus1 | 25 | protein phosphatase 1 regulatory subunit | 154 | 2.39E-27 | 59.15% | 1 | F:protein binding | IPR001611 (PFAM); IPR025875 (PFAM); G3DSA:3.80.10.10 (GENE3D), PTHR10588 (PANTHER), PTHR10588:SF161 (PANTHER), SSF52058 (SUPERFAMILY) | |
| 3201 | lib_dib_Filter15_IDBA_contig_71815_minus3 | 10 | lrrc3-like protein ensp00000367157 | 153 | 2.56E-07 | 59.45% | 1 | F:protein binding | IPR001611 (PFAM); IPR003591 (SMART); G3DSA:3.80.10.10 (GENE3D), PTHR24369 (PANTHER), PF13516 (PFAM), PF13855 (PFAM), SignalP-NN(euk) (SIGNALP), SSF52058 (SUPERFAMILY) | |
| 3202 | lib_dib_Filter15_IDBA_contig_7203_minus3 | 2419 | elastase 2a | 138 | 5.78E-22 | 60.10% | 3 | F:peptidase activity; P:protein metabolic process; P:catabolic process | IPR001254 (PFAM); IPR001314 (PRINTS); IPR009003 (SUPERFAMILY); IPR018114 (PROSITE); G3DSA:2.40.10.10 (GENE3D), PTHR24268 (PANTHER) | |
| 3203 | lib_dib_Filter15_IDBA_contig_72108_plus2 | 553 | arylacetamide deacetylase-like | 608 | 5.59E-72 | 54.75% | 4 | P:metabolic process; F:hydrolase activity; C:cell; P:primary metabolic process | IPR013094 (PFAM); G3DSA:3.40.50.1820 (GENE3D), PTHR23024 (PANTHER), SignalP-NN(euk) (SIGNALP), tmhmm (TMHMM), SSF53474 (SUPERFAMILY) | |
| 3204 | lib_dib_Filter15_IDBA_contig_72110_plus1 | 73 | neutral cholesterol ester hydrolase 1 | 153 | 1.58E-30 | 57.75% | 5 | F:hydrolase activity; P:metabolic process; F:binding; P:cellular protein modification process; C:cell | IPR013094 (PFAM); G3DSA:3.40.50.1820 (GENE3D), PTHR23024 (PANTHER), SSF53474 (SUPERFAMILY) | |
| 3205 | lib_dib_Filter15_IDBA_contig_72111_plus1 | 59 | arylacetamide deacetylase-like | 124 | 7.68E-30 | 63.05% | 2 | P:metabolic process; F:hydrolase activity | IPR013094 (PFAM); G3DSA:3.40.50.1820 (GENE3D), PTHR23024 (PANTHER), SSF53474 (SUPERFAMILY) | |
| 3206 | lib_dib_Filter15_IDBA_contig_72166_plus3 | 14 | zinc finger | 153 | 5.06E-45 | 59.20% | 3 | F:binding; F:nucleic acid binding; C:intracellular | IPR007087 (PFAM); IPR013087 (G3DSA:3.30.160.GENE3D); IPR015880 (SMART); PTHR11389 (PANTHER), PF13894 (PFAM), PF13912 (PFAM), SSF57667 (SUPERFAMILY) | |
| 3207 | lib_dib_Filter15_IDBA_contig_72252_minus2 | 475 | achain crystal structure of engineered northeast structural genomics consortium target | 335 | 3.12E-21 | 55.40% | 1 | F:protein binding | IPR002110 (PFAM); IPR020683 (G3DSA:1.25.40.GENE3D); PTHR24198 (PANTHER) | |
| 3208 | lib_dib_Filter15_IDBA_contig_72253_minus2 | 1169 | ankyrin repeat protein e4_2 | 680 | 1.72E-20 | 55.50% | 1 | F:protein binding | IPR002110 (PFAM); IPR020683 (G3DSA:1.25.40.GENE3D); PTHR24198 (PANTHER) | |
| 3209 | lib_dib_Filter15_IDBA_contig_72299_minus1 | 94 | copper chaperone for superoxide dismutase | 225 | 1.12E-47 | 61.70% | 6 | F:binding; P:ion transport; P:metabolic process; F:protein binding; C:cytoplasm; C:nucleus | IPR001424 (G3DSA:2.60.40.GENE3D); IPR024134 (PANTHER); IPR024142 (PTHR10003:PANTHER) | |

| | | | | | | | | | | |
|---|---|---|---|---|---|---|---|---|---|---|
| 3210 | lib_dib_Filter15_IDBA_contig_72300_minus1 | 93 | copper chaperone for superoxide dismutase | 256 | 6.56E-47 | 61.45% | 6 | F:binding; P:ion transport; P:metabolic process; F:protein binding; C:cytoplasm; C:nucleus | IPR001424 (G3DSA:2.60.40.GENE3D); IPR024134 (PANTHER); IPR024142 (PTHR10003:PANTHER) | |
| 3211 | lib_dib_Filter15_IDBA_contig_72327_plus1 | 16 | isoform a | 153 | 7.99E-19 | 64.10% | 32 | P:multicellular organismal development; P:biological_process; P:reproduction; P:cell differentiation; P:embryo development; P:anatomical structure morphogenesis; P:cytoskeleton organization; P:cellular component organization; F:nucleotide binding; C:plasma membrane; F:receptor activity; P:signal transduction; P:cell death; P:regulation of biological process; P:cell-cell signaling; P:growth; P:behavior; C:protein complex; P:cell proliferation; ; C:organelle; C:cytoplasm; C:nucleus; P:response to biotic stimulus; P:cell cycle; F:chromatin binding; F:protein binding; P:response to external stimulus; P:response to stress; C:cell; F:DNA binding; F:calcium ion binding | IPR000152 (PROSITE); IPR000742 (PFAM); IPR000859 (G3DSA:2.60.120.GENE3D); IPR001881 (SMART); IPR013032 (PROSITE); IPR018097 (PROSITE); PR00010 (PRINTS), G3DSA:2.10.25.10 (GENE3D), PTHR24048 (PANTHER), PTHR24048:SF15 (PANTHER), SSF57196 (SUPERFAMILY) | |
| 3212 | lib_dib_Filter15_IDBA_contig_72373_minus3 | 238 | cathepsin l | 427 | 3.76E-106 | 64.85% | 3 | P:protein metabolic process; P:catabolic process; F:peptidase activity | IPR000169 (PROSITE); IPR000668 (PRINTS); IPR013128 (PANTHER); IPR013201 (PFAM); IPR025661 (PROSITE); G3DSA:3.90.70.10 (GENE3D), PTHR12411:SF149 (PANTHER), tmhmm (TMHMM), SSF54001 (SUPERFAMILY) | |
| 3213 | lib_dib_Filter15_IDBA_contig_72374_minus3 | 254 | cathepsin l | 425 | 2.90E-106 | 64.85% | 3 | P:protein metabolic process; P:catabolic process; F:peptidase activity | IPR000169 (PROSITE); IPR000668 (PRINTS); IPR013128 (PANTHER); IPR013201 (PFAM); IPR025661 (PROSITE); G3DSA:3.90.70.10 (GENE3D), PTHR12411:SF149 (PANTHER), tmhmm (TMHMM), SSF54001 (SUPERFAMILY) | |
| 3214 | lib_dib_Filter15_IDBA_contig_72375_minus3 | 92 | cathepsin s | 136 | 2.92E-13 | 60.55% | 7 | C:lysosome; F:peptidase activity; C:endosome; P:biological_process; P:protein metabolic process; P:catabolic process; C:cell | IPR013128 (PANTHER); IPR013201 (PFAM); G3DSA:3.90.70.10 (GENE3D), PTHR12411:SF149 (PANTHER), tmhmm (TMHMM), SSF54001 (SUPERFAMILY) | |
| 3215 | lib_dib_Filter15_IDBA_contig_72392_plus2 | 13 | esterase lipase | 153 | 1.70E-21 | 54.30% | 2 | P:metabolic process; F:hydrolase activity | IPR002168 (PROSITE); IPR013094 (PFAM); G3DSA:3.40.50.1820 (GENE3D), PTHR23024 (PANTHER), SSF53474 (SUPERFAMILY) | |
| 3216 | lib_dib_Filter15_IDBA_contig_72487_plus1 | 21 | neurogenic locus notch homolog protein 2-like | 174 | 7.04E-20 | 47.75% | 4 | P:biological_process; P:multicellular organismal development; F:protein binding; F:calcium ion binding | IPR000152 (PROSITE); IPR000742 (PFAM); IPR001881 (PFAM); IPR013032 (PROSITE); IPR018097 (PROSITE); PR00010 (PRINTS), G3DSA:2.10.25.10 (GENE3D), PTHR24044 (PANTHER), SSF57196 (SUPERFAMILY) | |
| 3217 | lib_dib_Filter15_IDBA_contig_72506_plus2 | 11 | proprotein convertase subtilisin kexin type 7 | 152 | 1.01E-42 | 72.70% | 4 | F:peptidase activity; P:protein metabolic process; C:Golgi apparatus; P:catabolic process | IPR000209 (G3DSA:3.40.50.GENE3D); IPR015500 (PRINTS); IPR022398 (PROSITE); IPR023827 (PROSITE); PTHR10795:SF9 (PANTHER) | |
| 3218 | lib_dib_Filter15_IDBA_contig_72516_minus3 | 13 | dbh-like monooxygenase protein 1 homolog | 152 | 6.41E-32 | 65.30% | 7 | F:transferase activity; F:catalytic activity; ; P:metabolic process; P:carbohydrate metabolic process; P:catabolic process; F:carbohydrate binding | IPR000945 (PANTHER); IPR005018 (PFAM); IPR008960 (SUPERFAMILY); IPR015920 (G3DSA:2.60.40.GENE3D); SignalP-NN(euk) (SIGNALP) | yes |
| 3219 | lib_dib_Filter15_IDBA_contig_72595_minus1 | 4795 | low density lipoprotein- class - containing protein | 250 | 3.34E-08 | 46.89% | 1 | F:protein binding | IPR002172 (G3DSA:4.10.400.GENE3D); PTHR20967 (PANTHER), SignalP-NN(euk) (SIGNALP), tmhmm (TMHMM) | yes |
| 3220 | lib_dib_Filter15_IDBA_contig_72612_plus3 | 14 | pancreatic lipase-related protein 1 | 152 | 8.53E-18 | 47.80% | 2 | F:catalytic activity; P:lipid metabolic process | IPR000734 (PANTHER); IPR013818 (PFAM); G3DSA:3.40.50.1820 (GENE3D), SSF53474 (SUPERFAMILY) | |

| | | | | | | | | | | |
|---|---|---|---|---|---|---|---|---|---|---|
| 3221 | lib_dib_Filter15_IDBA_contig_72785_minus3 | 8 | delta-like protein 4 | 151 | 1.99E-29 | 55.10% | 8 | P:regulation of biological process; P:anatomical structure morphogenesis; P:multicellular organismal development; F:receptor binding; P:cell communication; P:cell differentiation; C:cell; F:calcium ion binding | IPR000742 (PFAM); IPR001881 (SMART); IPR013032 (PROSITE); G3DSA:2.10.25.10 (GENE3D), PTHR24838 (PANTHER), PTHR24838:SF112 (PANTHER), SSF57196 (SUPERFAMILY) | |
| 3222 | lib_dib_Filter15_IDBA_contig_72810_plus1 | 13 | oocyte zinc finger protein 6-partial | 152 | 1.95E-33 | 57.75% | 3 | F:binding; F:nucleic acid binding; C:intracellular | IPR007087 (PROSITE); IPR013087 (G3DSA:3.30.160.GENE3D); IPR015880 (SMART); PTHR24382 (PANTHER), PF13465 (PFAM), SSF57667 (SUPERFAMILY) | |
| 3223 | lib_dib_Filter15_IDBA_contig_72821_minus2 | 16 | matrilin cartilage matrix protein precursor | 152 | 2.25E-40 | 56.55% | 2 | F:protein binding; F:calcium ion binding | IPR000742 (PFAM); IPR001881 (SMART); IPR002035 (PFAM); IPR013032 (PROSITE); PR00453 (PRINTS), G3DSA:2.10.25.10 (GENE3D), G3DSA:3.40.50.410 (GENE3D), PTHR22992 (PANTHER), SSF53300 (SUPERFAMILY) | |
| 3224 | lib_dib_Filter15_IDBA_contig_72928_plus3 | 26 | low quality protein: hemicentin-1 | 151 | 9.62E-36 | 58.95% | 1 | C:extracellular region | IPR000884 (PFAM); PR01705 (PRINTS), G3DSA:2.20.100.10 (GENE3D), PTHR10199 (PANTHER), PTHR10199:SF37 (PANTHER) | |
| 3225 | lib_dib_Filter15_IDBA_contig_72989_minus1 | 18 | protocadherin-12 | 152 | 4.93E-45 | 64.10% | 3 | F:calcium ion binding; P:biological_process; C:plasma membrane | IPR002126 (PRINTS); IPR015919 (SUPERFAMILY); IPR020894 (PROSITE); PTHR24028 (PANTHER), PTHR24028:SF42 (PANTHER) | |
| 3226 | lib_dib_Filter15_IDBA_contig_73046_minus2 | 3619 | superoxide dismutase | 377 | 1.50E-56 | 77.95% | 3 | F:binding; P:metabolic process; F:catalytic activity | IPR001424 (PRINTS); IPR018152 (PROSITE); IPR024134 (PANTHER); SignalP-NN(euk) (SIGNALP), tmhmm (TMHMM) | yes |
| 3227 | lib_dib_Filter15_IDBA_contig_73047_minus2 | 3603 | superoxide dismutase | 361 | 8.69E-57 | 77.95% | 3 | F:binding; P:metabolic process; F:catalytic activity | IPR001424 (PRINTS); IPR018152 (PROSITE); IPR024134 (PANTHER); SignalP-NN(euk) (SIGNALP), tmhmm (TMHMM) | yes |
| 3228 | lib_dib_Filter15_IDBA_contig_73079_minus1 | 16 | cub and sushi domain-containing protein 1 | 152 | 3.29E-31 | 55.10% | 7 | F:carbohydrate binding; F:calcium ion binding; C:integral to membrane; C:plasma membrane; F:chitin binding; P:chitin metabolic process; C:extracellular region | IPR000436 (PFAM); G3DSA:2.10.70.10 (GENE3D), PTHR19325 (PANTHER) | |
| 3229 | lib_dib_Filter15_IDBA_contig_73179_minus3 | 13 | af355375_1 reverse transcriptase | 151 | 3.64E-16 | 50.90% | 5 | F:RNA binding; P:RNA-dependent DNA replication; F:RNA-directed DNA polymerase activity; F:zinc ion binding; C:intracellular | no IPS match | |
| 3230 | lib_dib_Filter15_IDBA_contig_73212_plus2 | 7461 | fibrinogen c domain-containing protein 1-like | 421 | 1.57E-40 | 59.35% | 3 | F:receptor binding; C:extracellular space; P:signal transduction | IPR002181 (PFAM); IPR014715 (G3DSA:4.10.530.GENE3D); IPR014716 (G3DSA:3.90.215.GENE3D); IPR020837 (PROSITE); PTHR19143 (PANTHER), tmhmm (TMHMM) | |
| 3231 | lib_dib_Filter15_IDBA_contig_73213_plus1 | 2286 | fibrinogen c domain-containing protein 1-like | 146 | 1.67E-16 | 59.95% | 2 | P:biological_process; F:carbohydrate binding | IPR002181 (PFAM); IPR014715 (G3DSA:4.10.530.GENE3D); IPR020837 (PROSITE); PTHR19143 (PANTHER), PTHR19143:SF70 (PANTHER), tmhmm (TMHMM) | |
| 3232 | lib_dib_Filter15_IDBA_contig_73288_minus3 | 12 | von willebrand factor type egf and pentraxin domain-containing protein 1 | 150 | 4.10E-15 | 43.00% | 7 | F:calcium ion binding; F:carbohydrate binding; P:cell adhesion; F:chromatin binding; C:cytoplasm; C:membrane; C:extracellular region | IPR009030 (SUPERFAMILY); IPR011641 (PFAM); SignalP-NN(euk) (SIGNALP) | yes |
| 3233 | lib_dib_Filter15_IDBA_contig_73512_minus2 | 81 | metalloproteinase inhibitor 2-like | 283 | 1.32E-04 | 39.00% | 2 | F:protein binding; F:enzyme regulator activity | IPR001134 (PROFILE); IPR001820 (PFAM); IPR008993 (SUPERFAMILY); G3DSA:2.40.50.120 (GENE3D), SignalP-NN(euk) (SIGNALP), tmhmm (TMHMM) | |
| 3234 | lib_dib_Filter15_IDBA_contig_73529_plus3 | 19 | protein sidekick-2-like | 150 | 2.55E-28 | 55.95% | 1 | F:protein binding | IPR003598 (SMART); IPR003599 (SMART); IPR007110 (PROFILE); IPR013098 (PFAM); IPR013783 (G3DSA:2.60.40.GENE3D); PTHR10489 (PANTHER), SSF48726 (SUPERFAMILY) | |

| | | | | | | | | | | |
|---|---|---|---|---|---|---|---|---|---|---|
| 3235 | lib_dib_Filter15_IDBA_contig_73630_plus3 | 10 | dipeptidyl peptidase 4 | 150 | 6.04E-70 | 73.70% | 12 | F:peptidase activity; P:regulation of biological process; C:plasma membrane; P:biological_process; P:cellular component organization; C:cytoplasmic membrane-bounded vesicle; C:cell; F:receptor binding; F:protein binding; P:cell proliferation; P:protein metabolic process; P:catabolic process | IPR001375 (PFAM); G3DSA:3.40.50.1820 (GENE3D), PTHR11731 (PANTHER), PTHR11731:SF23 (PANTHER), SSF53474 (SUPERFAMILY) | |
| 3236 | lib_dib_Filter15_IDBA_contig_73776_minus2 | 613 | retinoid-inducible serine carboxypeptidase | 532 | 2.49E-63 | 52.40% | 3 | F:peptidase activity; P:protein metabolic process; P:catabolic process | IPR001563 (PRINTS); G3DSA:3.40.50.1820 (GENE3D), PTHR11802:SF3 (PANTHER), SignalP-NN(euk) (SIGNALP), SSF53474 (SUPERFAMILY) | |
| 3237 | lib_dib_Filter15_IDBA_contig_73777_minus2 | 580 | retinoid-inducible serine carboxypeptidase | 534 | 2.92E-63 | 52.40% | 3 | F:peptidase activity; P:protein metabolic process; P:catabolic process | IPR001563 (PRINTS); G3DSA:3.40.50.1820 (GENE3D), PTHR11802:SF3 (PANTHER), SignalP-NN(euk) (SIGNALP), SSF53474 (SUPERFAMILY) | |
| 3238 | lib_dib_Filter15_IDBA_contig_73806_minus2 | 12 | isoform b | 150 | 3.40E-47 | 65.15% | 2 | F:peptidase activity; F:enzyme regulator activity | IPR002223 (PRINTS); IPR020901 (PROSITE); PTHR10083 (PANTHER) | |
| 3239 | lib_dib_Filter15_IDBA_contig_73807_plus2 | 55 | protein phosphatase 1 regulatory subunit 16a-like | 230 | 6.01E-58 | 62.20% | 1 | F:protein binding | IPR002110 (PRINTS); IPR020683 (G3DSA:1.25.40.GENE3D); PTHR24186 (PANTHER), PTHR24186:SF0 (PANTHER) | |
| 3240 | lib_dib_Filter15_IDBA_contig_73835_plus3 | 12 | dbh-like monooxygenase protein 2-like | 149 | 1.41E-12 | 66.60% | 5 | F:binding; P:primary metabolic process; F:catalytic activity; ; P:metabolic process | IPR000945 (PANTHER); IPR008977 (SUPERFAMILY); IPR014784 (G3DSA:2.60.120.GENE3D); PF03712 (PFAM) | |
| 3241 | lib_dib_Filter15_IDBA_contig_73856_plus2 | 15 | receptor tyrosine-protein kinase erbb-3-like | 150 | 4.00E-20 | 60.20% | 23 | F:protein kinase activity; P:anatomical structure morphogenesis; P:cellular component organization; P:cell differentiation; P:cell communication; P:cellular homeostasis; P:multicellular organismal development; P:response to external stimulus; P:regulation of biological process; C:plasma membrane; C:extracellular space; P:response to stress; P:growth; P:cell death; P:cell proliferation; C:cell; F:receptor activity; P:signal transduction; F:protein binding; P:cell recognition; P:transport; F:enzyme regulator activity; P:biological_process | IPR000494 (PFAM); G3DSA:3.80.20.20 (GENE3D), PTHR24416 (PANTHER), PTHR24416:SF88 (PANTHER), SignalP-NN(euk) (SIGNALP), tmhmm (TMHMM), SSF52058 (SUPERFAMILY) | |
| 3242 | lib_dib_Filter15_IDBA_contig_73879_plus1 | 10 | aminopeptidase n | 150 | 1.99E-09 | 43.00% | 2 | P:protein metabolic process; P:catabolic process | IPR001930 (PANTHER); G3DSA:1.10.390.10 (GENE3D), SignalP-NN(euk) (SIGNALP), SSF55486 (SUPERFAMILY) | |
| 3243 | lib_dib_Filter15_IDBA_contig_73920_minus1 | 36 | matrix metalloproteinase 9 | 150 | 1.88E-12 | 55.90% | 7 | F:peptidase activity; P:response to biotic stimulus; P:response to external stimulus; P:regulation of biological process; P:response to stress; P:biological_process; C:extracellular region | IPR000562 (G3DSA:2.10.10.GENE3D); IPR013806 (SUPERFAMILY); PR00013 (PRINTS), PTHR22918 (PANTHER), PTHR22918:SF0 (PANTHER), tmhmm (TMHMM) | |
| 3244 | lib_dib_Filter15_IDBA_contig_73923_plus2 | 16 | neuropilin and tolloid -like | 150 | 1.40E-17 | 57.85% | 14 | P:protein transport; P:cellular component organization; C:protein complex; P:response to external stimulus; C:plasma membrane; C:lysosome; F:receptor activity; F:protein binding; C:cell; C:cytoplasmic membrane-bounded vesicle; P:metabolic process; P:transport; F:hydrolase activity; P:embryo development | IPR000859 (G3DSA:2.60.120.GENE3D); PTHR10127 (PANTHER), SignalP-NN(euk) (SIGNALP) | yes |

| | | | | | | | | |
|---|---|---|---|---|---|---|---|---|
| 3245 | lib_dib_Filter15_IDBA_contig_74005_minus3 | 11 | zinc finger protein 271 (zinc finger protein 7) (zinc finger protein znfphex133) (epstein-barr virus-induced zinc finger protein) (znf-eb) (ct-zfp48) (zinc finger protein | 149 | 4.02E-46 | 54.55% | 3 | F:binding; F:nucleic acid binding; C:intracellular | IPR007087 (PROSITE); IPR013087 (G3DSA:3.30.160.GENE3D); IPR015880 (SMART); PTHR11389 (PANTHER), PF13465 (PFAM), PF13912 (PFAM), SSF57667 (SUPERFAMILY) |
| 3246 | lib_dib_Filter15_IDBA_contig_74017_minus2 | 29 | slit homolog 1 | 149 | 4.13E-14 | 51.95% | 6 | P:anatomical structure morphogenesis; P:cellular component organization; P:cell differentiation; P:multicellular organismal development; F:protein binding; P:regulation of biological process | IPR000483 (SMART); IPR001611 (PROFILE); G3DSA:3.80.10.10 (GENE3D), PTHR24369 (PANTHER), PF13855 (PFAM), tmhmm (TMHMM), SSF52058 (SUPERFAMILY) |
| 3247 | lib_dib_Filter15_IDBA_contig_74044_plus2 | 48 | low quality protein: protein scribble homolog | 211 | 2.48E-24 | 78.45% | 20 | F:protein binding; P:anatomical structure morphogenesis; P:cell differentiation; P:cell death; P:signal transduction; P:regulation of biological process; P:cell cycle; P:behavior; P:response to external stimulus; C:cell; C:plasma membrane; C:cytoplasm; C:protein complex; P:cell proliferation; P:biological_process; P:metabolic process; P:transport; P:multicellular organismal development; P:cellular component organization; P:embryo development | IPR001478 (PFAM); G3DSA:2.30.42.10 (GENE3D), PTHR19964 (PANTHER) |
| 3248 | lib_dib_Filter15_IDBA_contig_74144_plus3 | 47 | toll-like receptor 13 | 237 | 2.62E-40 | 64.55% | 2 | F:protein binding; P:signal transduction | IPR000157 (PFAM); PR01537 (PRINTS), G3DSA:3.40.50.10140 (GENE3D), PTHR24365 (PANTHER), SignalP-NN(euk) (SIGNALP), tmhmm (TMHMM) |
| 3249 | lib_dib_Filter15_IDBA_contig_74154_minus3 | 8 | discoidin domain-containing receptor 2 | 149 | 3.16E-65 | 75.15% | 13 | P:regulation of biological process; ; P:cellular protein modification process; P:multicellular organismal development; P:cellular component organization; P:metabolic process; P:anatomical structure morphogenesis; P:growth; P:cell differentiation; F:protein binding; P:cell proliferation; C:plasma membrane; F:nucleotide binding | IPR000719 (PROFILE); IPR001245 (PRINTS); IPR002290 (SMART); IPR008266 (PROSITE); IPR011009 (SUPERFAMILY); IPR020635 (SMART); G3DSA:1.10.510.10 (GENE3D), G3DSA:3.30.200.20 (GENE3D), PTHR24416 (PANTHER), PTHR24416:SF64 (PANTHER) |
| 3250 | lib_dib_Filter15_IDBA_contig_74225_minus1 | 16 | raf homolog serine threonine-protein kinase phl-like | 149 | 9.45E-91 | 92.95% | 20 | P:biological_process; P:cellular component organization; P:metabolic process; P:regulation of biological process; P:signal transduction; F:protein binding; P:cellular protein modification process; C:cell; F:calcium ion binding; C:cytosol; P:cell death; P:response to endogenous stimulus; P:cell-cell signaling; P:anatomical structure morphogenesis; P:multicellular organismal development; F:protein kinase activity; F:signal transducer activity; C:plasma membrane; C:nucleus; F:nucleotide binding | IPR000719 (PROFILE); IPR001245 (PFAM); IPR002290 (SMART); IPR008271 (PROSITE); IPR011009 (SUPERFAMILY); IPR020635 (SMART); G3DSA:1.10.510.10 (GENE3D), G3DSA:3.30.200.20 (GENE3D), PTHR23257 (PANTHER), PTHR23257:SF41 (PANTHER), SignalP-NN(euk) (SIGNALP) |
| 3251 | lib_dib_Filter15_IDBA_contig_74321_plus1 | 11 | arylsulfatase j | 149 | 3.85E-25 | 59.45% | 2 | F:catalytic activity; P:metabolic process | IPR017850 (SUPERFAMILY); G3DSA:3.30.1120.10 (GENE3D), PTHR10342 (PANTHER), PTHR10342:SF19 (PANTHER) |
| 3252 | lib_dib_Filter15_IDBA_contig_74356_plus2 | 21 | signal cub and egf-like domain-containing protein 3-like | 149 | 3.51E-59 | 81.05% | 5 | F:protein binding; C:extracellular space; P:cellular component organization; C:plasma membrane; F:calcium ion binding | IPR000859 (G3DSA:2.60.120.GENE3D); PTHR24046 (PANTHER), PTHR24046:SF0 (PANTHER) |

| | | | | | | | | | |
|---|---|---|---|---|---|---|---|---|---|
| 3253 | lib_dib_Filter15_IDBA_contig_74383_plus2 | 14 | low-density lipoprotein receptor-related protein 4 | 149 | 6.42E-68 | 77.20% | 22 | P:anatomical structure morphogenesis; P:embryo development; F:peptidase activity; C:cytoplasmic membrane-bounded vesicle; P:multicellular organismal development; P:signal transduction; P:cellular component organization; P:cell differentiation; P:growth; F:receptor binding; P:regulation of biological process; F:calcium ion binding; C:cell; P:biological_process; P:reproduction; P:cellular protein modification process; P:protein metabolic process; P:catabolic process; F:receptor activity; F:protein binding; C:plasma membrane; P:metabolic process | IPR000033 (PFAM); IPR000742 (SMART); IPR011042 (G3DSA:2.120.10.GENE3D); G3DSA:2.10.25.10 (GENE3D), PTHR10529 (PANTHER), SSF57196 (SUPERFAMILY), SSF63825 (SUPERFAMILY) |
| 3254 | lib_dib_Filter15_IDBA_contig_74462_plus3 | 10 | egf-like domain-containing protein | 148 | 9.60E-24 | 56.85% | 2 | F:protein binding; F:calcium ion binding | IPR000152 (PROSITE); IPR000742 (SMART); IPR001881 (PFAM); IPR013032 (PROSITE); IPR018097 (PROSITE); G3DSA:2.10.25.10 (GENE3D), PTHR24048 (PANTHER), SSF57196 (SUPERFAMILY) |
| 3255 | lib_dib_Filter15_IDBA_contig_74531_minus3 | 2384 | tumor necrosis factor receptor superfamily member 27 | 344 | 5.62E-06 | 41.56% | 1 | F:protein binding | IPR001368 (PROFILE); G3DSA:2.10.50.10 (GENE3D), PTHR14415 (PANTHER), PTHR14415:SF0 (PANTHER), tmhmm (TMHMM) |
| 3256 | lib_dib_Filter15_IDBA_contig_74536_plus1 | 82 | low quality protein: cadherin egf lag seven-pass g-type receptor 3 | 291 | 1.54E-79 | 53.30% | 3 | P:signal transduction; F:protein binding; F:calcium ion binding | IPR000742 (PFAM); IPR001791 (PFAM); IPR001881 (SMART); IPR008985 (SUPERFAMILY); IPR013032 (PROSITE); IPR013320 (G3DSA:2.60.120.GENE3D); G3DSA:2.10.25.10 (GENE3D), PTHR24026 (PANTHER), PTHR24026:SF1 (PANTHER), SSF57196 (SUPERFAMILY) |
| 3257 | lib_dib_Filter15_IDBA_contig_74716_plus3 | 17 | cre-mig-6 protein | 148 | 4.79E-23 | 48.35% | 1 | F:enzyme regulator activity | IPR002223 (PRINTS); IPR020901 (PROSITE); PTHR10083 (PANTHER) |
| 3258 | lib_dib_Filter15_IDBA_contig_74777_minus2 | 13 | low quality protein: protein scribble homolog | 148 | 3.93E-35 | 80.95% | 21 | F:protein binding; P:anatomical structure morphogenesis; P:cell differentiation; P:cell death; P:signal transduction; P:regulation of biological process; P:cell cycle; P:behavior; P:response to external stimulus; C:cell; C:plasma membrane; C:cytoplasm; C:protein complex; P:cell proliferation; P:biological_process; P:transport; P:cell-cell signaling; P:metabolic process; P:multicellular organismal development; P:cellular component organization; P:embryo development | IPR001611 (PFAM); IPR003591 (SMART); IPR025875 (PFAM); G3DSA:3.80.10.10 (GENE3D), PTHR23155 (PANTHER), PTHR23155:SF30 (PANTHER), SM00364 (SMART), SSF52058 (SUPERFAMILY) |
| 3259 | lib_dib_Filter15_IDBA_contig_74937_minus3 | 21 | polypeptide n-acetylgalactosaminyltransferase 9-like isoform 1 | 148 | 1.23E-49 | 75.65% | 5 | P:biosynthetic process; P:cellular protein modification process; P:carbohydrate metabolic process; F:transferase activity; C:cell | IPR001173 (PFAM); PTHR11675 (PANTHER), SSF53448 (SUPERFAMILY) |
| 3260 | lib_dib_Filter15_IDBA_contig_75023_minus1 | 137 | reelin isoform 1 | 318 | 2.71E-57 | 52.35% | 4 | P:multicellular organismal development; P:biological_process; P:regulation of biological process; P:cell differentiation | PTHR11841 (PANTHER), PTHR11841:SF0 (PANTHER) |
| 3261 | lib_dib_Filter15_IDBA_contig_75061_minus2 | 17 | fibrinogen c domain-containing protein 1-like | 148 | 6.94E-14 | 53.90% | 7 | F:receptor binding; C:extracellular space; P:signal transduction; F:metal ion binding; F:chitin binding; C:integral to membrane; C:membrane | IPR002181 (PFAM); IPR014715 (G3DSA:4.10.530.GENE3D); PTHR19143 (PANTHER), PTHR19143:SF42 (PANTHER) |

| | | | | | | | | | | |
|---|---|---|---|---|---|---|---|---|---|---|
| 3262 | lib_dib_Filter15_IDBA_contig_75073_minus1 | 15 | ankyrin repeat protein | 148 | 1.92E-22 | 62.60% | 1 | F:protein binding | IPR002110 (PRINTS); IPR020683 (G3DSA:1.25.40.GENE3D); PTHR24193 (PANTHER) | |
| 3263 | lib_dib_Filter15_IDBA_contig_75171_minus2 | 12 | zinc finger protein 236 | 148 | 1.64E-40 | 69.00% | 3 | F:nucleic acid binding; C:intracellular; F:binding | IPR007087 (PFAM); IPR013087 (G3DSA:3.30.160.GENE3D); IPR015880 (SMART); PTHR24396 (PANTHER), PTHR24396:SF0 (PANTHER), PF13465 (PFAM), SSF57667 (SUPERFAMILY) | |
| 3264 | lib_dib_Filter15_IDBA_contig_75236_plus1 | 1471 | transmembrane protease serine 13 | 369 | 1.30E-57 | 52.90% | 3 | F:peptidase activity; P:protein metabolic process; P:catabolic process | IPR001254 (PFAM); IPR001314 (PRINTS); IPR009003 (SUPERFAMILY); G3DSA:2.40.10.10 (GENE3D), PTHR24256 (PANTHER), SignalP-NN(euk) (SIGNALP), tmhmm (TMHMM) | yes |
| 3265 | lib_dib_Filter15_IDBA_contig_75237_plus3 | 142 | transmembrane protease serine 3 | 127 | 1.75E-22 | 53.05% | 3 | F:peptidase activity; P:protein metabolic process; P:catabolic process | IPR001254 (PFAM); IPR009003 (SUPERFAMILY); G3DSA:2.40.10.10 (GENE3D), PTHR24260 (PANTHER) | |
| 3266 | lib_dib_Filter15_IDBA_contig_75250_minus3 | 14 | protein dachsous | 147 | 3.27E-44 | 62.20% | 3 | F:calcium ion binding; P:biological_process; C:plasma membrane | IPR002126 (PRINTS); IPR015919 (SUPERFAMILY); IPR020894 (PROSITE); PTHR24027 (PANTHER) | |
| 3267 | lib_dib_Filter15_IDBA_contig_75259_minus3 | 10 | dbh-like 1 | 147 | 9.16E-31 | 61.10% | 4 | F:binding; P:metabolic process; F:catalytic activity; | IPR000323 (G3DSA:2.60.120.GENE3D); IPR000945 (PRINTS); IPR008977 (SUPERFAMILY); PTHR10157:SF17 (PANTHER) | |
| 3268 | lib_dib_Filter15_IDBA_contig_75299_plus2 | 20 | calbindin-32 | 147 | 1.03E-70 | 81.00% | 2 | F:calcium ion binding; P:cellular homeostasis | IPR002048 (PFAM); IPR011992 (G3DSA:1.10.238.GENE3D); IPR018247 (PROSITE); PTHR19972 (PANTHER), PTHR19972:SF2 (PANTHER), SSF47473 (SUPERFAMILY) | |
| 3269 | lib_dib_Filter15_IDBA_contig_75387_plus3 | 8 | wnt-receptor frizzled-like protein variant 2 | 147 | 1.66E-41 | 76.45% | 20 | P:signal transduction; ; P:regulation of biological process; P:biological_process; P:anatomical structure morphogenesis; P:embryo development; C:plasma membrane; P:response to external stimulus; P:cell differentiation; P:response to stress; P:multicellular organismal development; P:growth; P:reproduction; P:cellular component organization; F:receptor activity; C:cytoplasm; F:protein binding; C:cell; P:cell proliferation; P:metabolic process | IPR015526 (PANTHER); IPR020067 (G3DSA:1.10.2000.GENE3D) | |
| 3270 | lib_dib_Filter15_IDBA_contig_75416_plus2 | 19 | collagen alpha-3 chain | 147 | 1.61E-48 | 60.65% | 1 | F:protein binding | IPR002035 (PFAM); G3DSA:3.40.50.410 (GENE3D), PTHR22992 (PANTHER), SSF53300 (SUPERFAMILY) | |
| 3271 | lib_dib_Filter15_IDBA_contig_75573_plus1 | 19 | carboxypeptidase b-like | 160 | 4.22E-13 | 77.10% | 4 | P:protein metabolic process; P:catabolic process; F:peptidase activity; F:binding | G3DSA:3.40.630.10 (GENE3D), PTHR11705 (PANTHER), PTHR11705:SF31 (PANTHER), tmhmm (TMHMM), SSF53187 (SUPERFAMILY) | |
| 3272 | lib_dib_Filter15_IDBA_contig_75654_plus1 | 16 | tyrosine-protein kinase src42a | 147 | 3.56E-27 | 60.80% | 2 | F:transferase activity; F:protein binding | IPR001611 (PFAM); IPR003591 (SMART); G3DSA:3.80.10.10 (GENE3D), PTHR24369 (PANTHER), PF13504 (PFAM), PF13855 (PFAM), SSF52058 (SUPERFAMILY) | |
| 3273 | lib_dib_Filter15_IDBA_contig_75824_plus1 | 13 | zinc finger protein 570-like | 147 | 1.13E-35 | 59.95% | 3 | F:binding; F:nucleic acid binding; C:intracellular | IPR007087 (PROSITE); IPR013087 (G3DSA:3.30.160.GENE3D); IPR015880 (SMART); PTHR24375 (PANTHER), PTHR24375:SF0 (PANTHER), PF13465 (PFAM), PF13912 (PFAM), SSF57667 (SUPERFAMILY) | |
| 3274 | lib_dib_Filter15_IDBA_contig_75860_minus3 | 38 | ---NA--- | 172 | | | 0 | - | tmhmm (TMHMM) | |

| | | | | | | | | | | |
|---|---|---|---|---|---|---|---|---|---|---|
| 3275 | lib_dib_Filter15_IDBA_contig_75969_plus2 | 12 | carbohydrate sulfotransferase 12-like | 146 | 6.59E-21 | 55.25% | 7 | P:anatomical structure morphogenesis; P:multicellular organismal development; P:biological_process; F:transferase activity; C:cell; P:biosynthetic process; P:carbohydrate metabolic process | IPR005331 (PFAM); IPR018011 (PANTHER) | |
| 3276 | lib_dib_Filter15_IDBA_contig_76096_plus3 | 17 | retinol dehydrogenase 13 | 146 | 2.68E-50 | 73.15% | 3 | P:metabolic process; F:catalytic activity; F:nucleotide binding | IPR002198 (PRINTS); IPR002347 (PRINTS); IPR016040 (G3DSA:3.40.50.GENE3D); PTHR24320 (PANTHER), PTHR24320:SF0 (PANTHER), SSF51735 (SUPERFAMILY) | |
| 3277 | lib_dib_Filter15_IDBA_contig_76191_plus3 | 12 | lysosomal acid phosphatase precursor | 146 | 3.89E-34 | 60.35% | 2 | C:lysosome; F:hydrolase activity | IPR000560 (PFAM); G3DSA:3.40.50.1240 (GENE3D), PTHR11567 (PANTHER), PTHR11567:SF26 (PANTHER), SSF53254 (SUPERFAMILY) | |
| 3278 | lib_dib_Filter15_IDBA_contig_76247_plus3 | 953 | superoxide dismutase | 640 | 4.77E-62 | 61.60% | 15 | P:biological_process; P:cell death; P:regulation of biological process; F:binding; P:metabolic process; P:reproduction; P:primary metabolic process; P:cellular homeostasis; P:response to stress; P:cell communication; C:organelle; C:intracellular; C:cytoplasm; P:multicellular organismal development; F:catalytic activity | IPR001424 (PRINTS); IPR018152 (PROSITE); IPR024134 (PANTHER) | |
| 3279 | lib_dib_Filter15_IDBA_contig_7625_plus2 | 1397 | protein disulfide-isomerase a4 | 1580 | 0 | 67.45% | 7 | P:protein transport; P:metabolic process; F:electron carrier activity; F:catalytic activity; P:cellular homeostasis; P:regulation of biological process; C:endoplasmic reticulum | IPR005746 (PRINTS); IPR005788 (TIGRFAMs); IPR005792 (TIGRFAMs); IPR012336 (G3DSA:3.40.30.GENE3D); IPR013766 (PFAM); IPR017937 (PROSITE); PTHR18929 (PANTHER), PTHR18929:SF32 (PANTHER), PF13848 (PFAM), SignalP-NN(euk) (SIGNALP), tmhmm (TMHMM) | yes |
| 3280 | lib_dib_Filter15_IDBA_contig_76285_plus1 | 11 | | 146 | 0 | 100% | 3 | F:calcium ion binding; P:biological_process; C:plasma membrane | IPR002126 (PRINTS); IPR015919 (SUPERFAMILY); IPR020894 (PROSITE); PTHR24027 (PANTHER), PTHR24027:SF49 (PANTHER) | |
| 3281 | lib_dib_Filter15_IDBA_contig_76373_plus1 | 3501 | soma ferritin-like | 410 | 1.77E-35 | 62.40% | 5 | P:ion transport; P:cellular homeostasis; F:binding; F:catalytic activity; P:metabolic process | IPR001519 (PANTHER); IPR008331 (PFAM); IPR009040 (PROFILE); IPR009078 (SUPERFAMILY); IPR012347 (G3DSA:1.20.1260.GENE3D) | |
| 3282 | lib_dib_Filter15_IDBA_contig_76447_minus1 | 18 | collagen alpha-1 chain | 146 | 3.81E-44 | 68.20% | 3 | C:extracellular region; F:structural molecule activity; P:biological_process | IPR010515 (PFAM); IPR016186 (G3DSA:3.10.100.GENE3D); IPR016187 (SUPERFAMILY); PTHR24637 (PANTHER), PTHR24637:SF88 (PANTHER) | |
| 3283 | lib_dib_Filter15_IDBA_contig_76497_plus2 | 18 | discoidin domain-containing receptor 2-like | 145 | 5.68E-22 | 68.55% | 7 | P:biological_process; P:anatomical structure morphogenesis; P:multicellular organismal development; F:receptor activity; F:protein kinase activity; P:cellular component organization; P:cell differentiation | IPR000421 (PFAM); IPR008979 (SUPERFAMILY); IPR027143 (PTHR10127:PANTHER); G3DSA:2.60.120.260 (GENE3D), PTHR10127 (PANTHER), SignalP-NN(euk) (SIGNALP), tmhmm (TMHMM) | |
| 3284 | lib_dib_Filter15_IDBA_contig_76519_plus3 | 759 | chymotrypsin-like elastase family member 2a-like | 405 | 5.14E-84 | 49.60% | 3 | F:peptidase activity; P:protein metabolic process; P:catabolic process | IPR001254 (PFAM); IPR001314 (PRINTS); IPR009003 (SUPERFAMILY); IPR018114 (PROSITE); G3DSA:2.40.10.10 (GENE3D), PTHR24265 (PANTHER) | |

| | | | | | | | | | | |
|---|---|---|---|---|---|---|---|---|---|---|
| 3285 | lib_dib_Filter15_IDBA_contig_76546_plus2 | 290 | macrophage migration inhibitory factor | 226 | 9.15E-33 | 65.50% | 29 | P:metabolic process; P:regulation of biological process; P:cell proliferation; P:behavior; P:response to endogenous stimulus; P:generation of precursor metabolites and energy; P:carbohydrate metabolic process; P:catabolic process; F:protein binding; P:cellular protein modification process; P:signal transduction; P:response to stress; P:response to external stimulus; P:response to abiotic stimulus; C:cytoplasm; P:multicellular organismal development; P:biological_process; P:protein metabolic process; P:ion transport; P:reproduction; P:anatomical structure morphogenesis; P:cellular component organization; P:cell differentiation; F:catalytic activity; C:nucleus; C:extracellular space; P:cell death; | IPR001398 (PRODOM); IPR014347 (SUPERFAMILY); IPR019829 (PROSITE); G3DSA:3.30.429.10 (GENE3D), PTHR11954:SF4 (PANTHER), SignalP-NN(euk) (SIGNALP), tmhmm (TMHMM) | yes |
| 3286 | lib_dib_Filter15_IDBA_contig_76594_plus3 | 15 | collagen alpha-1 chain | 145 | 4.64E-23 | 55.55% | 1 | F:protein binding | IPR002035 (PFAM); G3DSA:3.40.50.410 (GENE3D), PTHR22992 (PANTHER), SSF53300 (SUPERFAMILY) | |
| 3287 | lib_dib_Filter15_IDBA_contig_76596_minus2 | 14 | oocyte zinc finger protein 6-like | 145 | 1.02E-26 | 63.55% | 3 | F:binding; F:nucleic acid binding; C:intracellular | IPR007087 (PROSITE); IPR013087 (G3DSA:3.30.160.GENE3D); IPR015880 (SMART); PTHR11389 (PANTHER), PF13465 (PFAM), SSF57667 (SUPERFAMILY) | |
| 3288 | lib_dib_Filter15_IDBA_contig_76675_plus1 | 20 | c-type lectin domain family member a | 146 | 1.06E-14 | 47.65% | 1 | F:carbohydrate binding | IPR001304 (PFAM); IPR016186 (G3DSA:3.10.100.GENE3D); IPR016187 (SUPERFAMILY); IPR018378 (PROSITE); PTHR22802 (PANTHER) | |
| 3289 | lib_dib_Filter15_IDBA_contig_76676_minus2 | 30 | frazzled protein | 145 | 3.21E-40 | 64.60% | 1 | F:protein binding | IPR003598 (SMART); IPR003599 (SMART); IPR007110 (PROFILE); IPR013098 (PFAM); IPR013783 (G3DSA:2.60.40.GENE3D); PTHR10489 (PANTHER), PTHR10489:SF75 (PANTHER), SSF48726 (SUPERFAMILY) | |
| 3290 | lib_dib_Filter15_IDBA_contig_76774_plus1 | 17 | protocadherin fat 1 | 145 | 3.65E-44 | 71.80% | 5 | C:plasma membrane; C:cell; C:intracellular; F:calcium ion binding; P:biological_process | IPR002126 (PRINTS); IPR015919 (SUPERFAMILY); PTHR24027 (PANTHER), PTHR24027:SF110 (PANTHER) | |
| 3291 | lib_dib_Filter15_IDBA_contig_76804_plus2 | 22 | tolloid-like 1 | 145 | 2.17E-05 | 45.05% | 17 | P:proteolysis; F:serine-type endopeptidase activity; P:metabolic process; F:catalytic activity; C:extracellular region; F:metalloendopeptidase activity; F:hydrolase activity; F:zinc ion binding; F:metallopeptidase activity; F:collagen binding; F:peptidase activity; F:metal ion binding; F:calcium ion binding; P:multicellular organismal development; P:cell differentiation; F:protein binding; P:skeletal system development | IPR000859 (G3DSA:2.60.120.GENE3D); PTHR24254 (PANTHER) | |
| 3292 | lib_dib_Filter15_IDBA_contig_76899_plus1 | 17 | isoform f | 145 | 5.58E-83 | 84.25% | 8 | F:binding; F:nucleic acid binding; P:anatomical structure morphogenesis; P:multicellular organismal development; F:sequence-specific DNA binding transcription factor activity; P:regulation of biological process; ; C:nucleus | IPR007087 (PROSITE); IPR013087 (G3DSA:3.30.160.GENE3D); IPR015880 (SMART); PTHR11389 (PANTHER), PTHR11389:SF336 (PANTHER), PF13465 (PFAM), PF13894 (PFAM), SSF57667 (SUPERFAMILY) | |

| | | | | | | | | | | |
|---|---|---|---|---|---|---|---|---|---|---|
| 3293 | lib_dib_Filter15_IDBA_contig_76918_plus2 | 1783 | cysteine-rich protease inhibitor | 453 | 1.13E-39 | 56.80% | 1 | C:extracellular region | IPR001283 (PRINTS); IPR002413 (PRINTS); IPR014044 (G3DSA:3.40.33.GENE3D); IPR018244 (PROSITE); PTHR10334:SF12 (PANTHER) | |
| 3294 | lib_dib_Filter15_IDBA_contig_76958_plus1 | 22 | leucine-rich alpha-2-glycoprotein 1 | 145 | 9.44E-12 | 53.75% | 1 | F:protein binding | IPR001611 (PROFILE); IPR003591 (SMART); IPR025875 (PFAM); G3DSA:3.80.10.10 (GENE3D), PTHR24364 (PANTHER), SSF52058 (SUPERFAMILY) | |
| 3295 | lib_dib_Filter15_IDBA_contig_77058_minus3 | 20 | leucine-rich repeat and immunoglobulin-like domain-containing nogo receptor-interacting protein 2-like | 144 | 5.55E-18 | 47.95% | 1 | F:protein binding | IPR003598 (SMART); IPR003599 (SMART); IPR007110 (PROFILE); IPR013098 (PFAM); IPR013783 (G3DSA:2.60.40.GENE3D); PTHR24373 (PANTHER), SSF48726 (SUPERFAMILY) | |
| 3296 | lib_dib_Filter15_IDBA_contig_77116_plus2 | 13 | polypeptide n-acetylgalactosaminyltransferase 1 | 144 | 3.92E-53 | 68.80% | 1 | P:carbohydrate metabolic process | IPR003859 (PFAM); G3DSA:3.90.550.10 (GENE3D), PTHR11675 (PANTHER), SSF53448 (SUPERFAMILY) | |
| 3297 | lib_dib_Filter15_IDBA_contig_77124_minus3 | 495 | ---NA--- | 229 | | | 0 | - | PTHR11905 (PANTHER), PTHR11905:SF48 (PANTHER), SignalP-NN(euk) (SIGNALP) | yes |
| 3298 | lib_dib_Filter15_IDBA_contig_77124_plus1 | 495 | ---NA--- | 230 | | | 0 | - | PTHR24023 (PANTHER), PTHR24023:SF164 (PANTHER) | |
| 3299 | lib_dib_Filter15_IDBA_contig_77170_minus3 | 53 | zinc finger protein 271 (zinc finger protein 7) (zinc finger protein znfphex133) (epstein-barr virus-induced zinc finger protein) (znf-eb) (ct-zfp48) (zinc finger protein | 251 | 4.02E-23 | 61.40% | 3 | F:binding; F:nucleic acid binding; C:intracellular | IPR007087 (PFAM); IPR013087 (G3DSA:3.30.160.GENE3D); IPR015880 (SMART); PTHR11389 (PANTHER), PF13465 (PFAM), SSF57667 (SUPERFAMILY) | |
| 3300 | lib_dib_Filter15_IDBA_contig_77204_plus1 | 14 | endoplasmic reticulum mannosyl-oligosaccharide -alpha-mannosidase-like | 145 | 2.45E-57 | 80.05% | 9 | P:protein metabolic process; P:catabolic process; F:calcium ion binding; P:biosynthetic process; P:cellular protein modification process; P:carbohydrate metabolic process; C:cell; F:hydrolase activity; C:endoplasmic reticulum | IPR001382 (PRINTS); PTHR11742:SF7 (PANTHER) | |
| 3301 | lib_dib_Filter15_IDBA_contig_77222_plus1 | 9 | uncharacterized aarf domain-containing protein kinase 5-like | 145 | 9.54E-40 | 63.90% | 2 | C:mitochondrion; F:transferase activity | PTHR10566 (PANTHER), PTHR10566:SF25 (PANTHER), tmhmm (TMHMM) | |
| 3302 | lib_dib_Filter15_IDBA_contig_775_minus2 | 2728 | neuroglian | 1355 | 0 | 51.15% | 1 | F:protein binding | IPR003598 (SMART); IPR003599 (SMART); IPR003961 (PFAM); IPR007110 (PROFILE); IPR013098 (PFAM); IPR013783 (G3DSA:2.60.40.GENE3D); IPR026966 (PFAM); PTHR10489 (PANTHER), tmhmm (TMHMM), SSF48726 (SUPERFAMILY) | |
| 3303 | lib_dib_Filter15_IDBA_contig_77536_minus3 | 24 | neogenin isoform 2 | 143 | 4.91E-52 | 74.00% | 8 | C:plasma membrane; P:biological_process; P:anatomical structure morphogenesis; P:cellular component organization; P:cell differentiation; P:multicellular organismal development; P:regulation of biological process; F:protein binding | IPR003961 (PFAM); IPR013783 (G3DSA:2.60.40.GENE3D); PR00014 (PRINTS), PTHR10489 (PANTHER), PTHR10489:SF55 (PANTHER) | |
| 3304 | lib_dib_Filter15_IDBA_contig_7762_plus1 | 1511 | ankyrin repeat protein | 583 | 2.96E-36 | 42.80% | 1 | F:protein binding | IPR002110 (PFAM); IPR020683 (G3DSA:1.25.40.GENE3D); PTHR24128 (PANTHER) | |
| 3305 | lib_dib_Filter15_IDBA_contig_777_minus2 | 38410 | ankyrin repeat and fyve domain-containing protein 1 | 249 | 2.40E-56 | 71.45% | 4 | P:transport; P:cellular component organization; C:endosome; F:protein binding | IPR002110 (PRINTS); IPR020683 (G3DSA:1.25.40.GENE3D); PTHR24189 (PANTHER), PTHR24189:SF0 (PANTHER) | |
| 3306 | lib_dib_Filter15_IDBA_contig_77716_plus2 | 171 | fibrinogen c domain-containing protein 1-like | 369 | 5.01E-22 | 53.25% | 3 | F:receptor binding; C:extracellular space; P:signal transduction | IPR002181 (PFAM); IPR014715 (G3DSA:4.10.530.GENE3D); IPR014716 (G3DSA:3.90.215.GENE3D); PTHR19143 (PANTHER) | |

| | | | | | | | | | |
|---|---|---|---|---|---|---|---|---|---|
| 3307 | lib_dib_Filter15_IDBA_contig_7773_minus3 | 155 | ankyrin repeat protein | 281 | 1.53E-14 | 53.75% | 1 | F:protein binding | IPR002110 (SMART); IPR020683 (G3DSA:1.25.40.GENE3D); PTHR24193 (PANTHER), SignalP-NN(euk) (SIGNALP) |
| 3308 | lib_dib_Filter15_IDBA_contig_77940_plus1 | 77 | tyrosine-protein kinase fps85d-like isoform 2 | 230 | 2.33E-103 | 75.50% | 14 | P:anatomical structure morphogenesis; P:cellular component organization; P:cell differentiation; P:multicellular organismal development; P:cellular protein modification process; C:plasma membrane; P:cytoskeleton organization; P:embryo development; F:protein kinase activity; P:regulation of biological process; P:cell growth; C:cytoplasm; F:nucleotide binding; F:protein binding | IPR000719 (PROFILE); IPR000980 (PRINTS); IPR001245 (PFAM); IPR011009 (SUPERFAMILY); IPR017441 (PROSITE); IPR020635 (SMART); G3DSA:3.30.200.20 (GENE3D), PTHR24418 (PANTHER), PTHR24418:SF0 (PANTHER), SSF55550 (SUPERFAMILY) |
| 3309 | lib_dib_Filter15_IDBA_contig_77941_plus3 | 106 | tyrosine-protein kinase fps85d-like isoform 2 | 328 | 1.57E-102 | 74.90% | 14 | P:anatomical structure morphogenesis; P:cellular component organization; P:cell differentiation; P:multicellular organismal development; P:cellular protein modification process; C:plasma membrane; P:cytoskeleton organization; P:embryo development; F:protein kinase activity; P:regulation of biological process; P:cell growth; C:cytoplasm; F:nucleotide binding; F:protein binding | IPR000719 (PROFILE); IPR000980 (PRINTS); IPR001245 (PFAM); IPR011009 (SUPERFAMILY); IPR017441 (PROSITE); IPR020635 (SMART); G3DSA:3.30.200.20 (GENE3D), PTHR24418 (PANTHER), PTHR24418:SF0 (PANTHER), SSF55550 (SUPERFAMILY) |
| 3310 | lib_dib_Filter15_IDBA_contig_77949_plus3 | 13 | low-density lipoprotein receptor-related protein 6 isoform 1 | 143 | 7.40E-66 | 85.20% | 30 | P:regulation of biological process; P:cell death; C:organelle; C:cytoplasm; C:cell; P:anatomical structure morphogenesis; P:embryo development; F:enzyme regulator activity; P:cellular homeostasis; F:receptor binding; F:protein binding; P:cell proliferation; P:transport; P:cellular component organization; C:plasma membrane; P:response to external stimulus; P:cell-cell signaling; P:cell differentiation; P:multicellular organismal development; C:endoplasmic reticulum; ; P:biological_process; P:signal transduction; C:protein complex; F:receptor activity; F:transporter activity; C:endosome; C:cellular_component; P:cell cycle; P:reproduction | IPR000033 (PFAM); IPR011042 (G3DSA:2.120.10.GENE3D); PTHR10529 (PANTHER), SSF63825 (SUPERFAMILY) |
| 3311 | lib_dib_Filter15_IDBA_contig_77975_minus3 | 8 | low quality protein: cub domain-containing protein 2-like | 143 | 3.35E-10 | 50.60% | 3 | F:binding; F:hydrolase activity; C:cell | IPR000859 (G3DSA:2.60.120.GENE3D); PTHR10127 (PANTHER), PTHR10127:SF345 (PANTHER) |
| 3312 | lib_dib_Filter15_IDBA_contig_78158_minus2 | 163 | ankyrin repeat protein | 283 | 8.79E-19 | 53.20% | 1 | F:protein binding | IPR002110 (SMART); IPR020683 (G3DSA:1.25.40.GENE3D); PTHR24198 (PANTHER) |
| 3313 | lib_dib_Filter15_IDBA_contig_78159_minus2 | 212 | ankyrin repeat protein | 397 | 3.84E-18 | 53.25% | 1 | F:protein binding | IPR001496 (PFAM); IPR002110 (SMART); IPR020683 (G3DSA:1.25.40.GENE3D); PTHR24198 (PANTHER) |
| 3314 | lib_dib_Filter15_IDBA_contig_78160_minus2 | 213 | ankyrin repeat protein | 401 | 3.08E-18 | 53.25% | 1 | F:protein binding | IPR001496 (PFAM); IPR002110 (SMART); IPR020683 (G3DSA:1.25.40.GENE3D); PTHR24198 (PANTHER) |
| 3315 | lib_dib_Filter15_IDBA_contig_78161_plus2 | 14 | variable lymphocyte receptor b | 143 | 2.29E-14 | 53.00% | 1 | F:protein binding | IPR001611 (PROFILE); IPR003591 (SMART); PR00019 (PRINTS), G3DSA:3.80.10.10 (GENE3D), PTHR24367 (PANTHER), PF13855 (PFAM), SSF52058 (SUPERFAMILY) |

| | | | | | | | | | | |
|---|---|---|---|---|---|---|---|---|---|---|
| 3316 | lib_dib_Filter15_IDBA_contig_78207_minus1 | 125 | neurogenic locus notch homolog protein 2-like | 241 | 1.16E-58 | 64.05% | 3 | C:extracellular region; F:protein binding; F:calcium ion binding | IPR000152 (PROSITE); IPR000742 (PFAM); IPR001881 (SMART); IPR013032 (PROSITE); IPR018097 (PROSITE); PR00010 (PRINTS), G3DSA:2.10.25.10 (GENE3D), PTHR24048 (PANTHER), PTHR24048:SF15 (PANTHER), SignalP-NN(euk) (SIGNALP), SSF57196 (SUPERFAMILY) | yes |
| 3317 | lib_dib_Filter15_IDBA_contig_78208_minus1 | 136 | neurogenic locus notch homolog protein 2-like | 208 | 1.83E-47 | 65.65% | 3 | C:proteinaceous extracellular matrix; F:protein binding; F:calcium ion binding | IPR000152 (PROSITE); IPR000742 (PFAM); IPR001881 (SMART); IPR013032 (PROSITE); IPR018097 (PROSITE); PR00010 (PRINTS), G3DSA:2.10.25.10 (GENE3D), PTHR24048 (PANTHER), PTHR24048:SF15 (PANTHER), SignalP-NN(euk) (SIGNALP), SSF57196 (SUPERFAMILY) | yes |
| 3318 | lib_dib_Filter15_IDBA_contig_78209_minus1 | 175 | protein | 308 | 1.08E-88 | 66.35% | 3 | C:proteinaceous extracellular matrix; F:protein binding; F:calcium ion binding | IPR000152 (PROSITE); IPR000742 (PFAM); IPR001881 (SMART); IPR013032 (PROSITE); IPR018097 (PROSITE); PR00010 (PRINTS), G3DSA:2.10.25.10 (GENE3D), PTHR24048 (PANTHER), PTHR24048:SF15 (PANTHER), SignalP-NN(euk) (SIGNALP), SSF57196 (SUPERFAMILY) | yes |
| 3319 | lib_dib_Filter15_IDBA_contig_78210_minus3 | 41 | protein | 142 | 3.56E-38 | 60.05% | 3 | C:extracellular region; F:protein binding; F:calcium ion binding | IPR000152 (PROSITE); IPR000742 (PFAM); IPR001881 (SMART); IPR013032 (PROSITE); IPR018097 (PROSITE); PR00010 (PRINTS), G3DSA:2.10.25.10 (GENE3D), PTHR24048 (PANTHER), PTHR24048:SF15 (PANTHER), SSF57196 (SUPERFAMILY) | |
| 3320 | lib_dib_Filter15_IDBA_contig_78211_minus3 | 63 | protein | 130 | 7.40E-46 | 70.15% | 3 | C:proteinaceous extracellular matrix; F:protein binding; F:calcium ion binding | IPR000152 (PROSITE); IPR000742 (PFAM); IPR001881 (SMART); IPR013032 (PROSITE); IPR018097 (PROSITE); PR00010 (PRINTS), G3DSA:2.10.25.10 (GENE3D), PTHR24048 (PANTHER), PTHR24048:SF15 (PANTHER), SSF57196 (SUPERFAMILY) | |
| 3321 | lib_dib_Filter15_IDBA_contig_78212_minus1 | 35 | protein | 111 | 9.94E-40 | 73.00% | 5 | C:proteinaceous extracellular matrix; C:organelle; C:cytoplasm; F:protein binding; F:calcium ion binding | IPR000152 (PROSITE); IPR000742 (PFAM); IPR001881 (SMART); IPR013032 (PROSITE); IPR018097 (PROSITE); PR00010 (PRINTS), G3DSA:2.10.25.10 (GENE3D), PTHR24048 (PANTHER), PTHR24048:SF15 (PANTHER), SSF57196 (SUPERFAMILY) | |
| 3322 | lib_dib_Filter15_IDBA_contig_78242_minus2 | 19 | ring canal kelch homolog | 143 | 3.84E-74 | 82.40% | 5 | C:cytosol; C:cytoskeleton; P:ion transport; P:cellular protein modification process; F:actin binding | IPR006652 (PFAM); IPR015916 (G3DSA:2.130.10.GENE3D); PTHR24412 (PANTHER), PTHR24412:SF4 (PANTHER), SSF117281 (SUPERFAMILY) | |
| 3323 | lib_dib_Filter15_IDBA_contig_78390_plus1 | 21 | low-density lipoprotein receptor-related protein 4 | 143 | 3.98E-31 | 61.45% | 5 | C:cell; P:multicellular organismal development; P:regulation of biological process; P:biological_process; F:protein binding | IPR002172 (G3DSA:4.10.400.GENE3D); IPR023415 (PROSITE); PR00261 (PRINTS), PTHR10529 (PANTHER), SignalP-NN(euk) (SIGNALP) | yes |
| 3324 | lib_dib_Filter15_IDBA_contig_7853_minus3 | 5086 | chymotrypsinogen b precursor | 212 | 1.09E-25 | 54.50% | 3 | F:peptidase activity; P:protein metabolic process; P:catabolic process | IPR001254 (PFAM); IPR009003 (SUPERFAMILY); IPR018114 (PROSITE); G3DSA:2.40.10.10 (GENE3D), PTHR24272 (PANTHER) | |

| | | | | | | | | | | |
|---|---|---|---|---|---|---|---|---|---|---|
| 3325 | lib_dib_Filter15_IDBA_contig_78536_plus2 | 17 | low-density lipoprotein receptor-related protein 2 | 142 | 1.03E-17 | 49.95% | 14 | F:protein binding; C:organelle; C:intracellular; P:biological_process; P:protein transport; P:multicellular organismal development; P:transport; P:response to external stimulus; C:cell; P:regulation of biological process; F:receptor activity; C:plasma membrane; C:cytoplasm; P:metabolic process | IPR002172 (G3DSA:4.10.400.GENE3D); IPR023415 (PROSITE); PR00261 (PRINTS), PTHR10529 (PANTHER) | |
| 3326 | lib_dib_Filter15_IDBA_contig_78560_plus2 | 20 | upf0364 protein c6orf211 homolog | 142 | 5.37E-47 | 65.75% | 3 | F:molecular_function; P:biological_process; C:cellular_component | IPR002791 (PFAM); PTHR12260 (PANTHER) | |
| 3327 | lib_dib_Filter15_IDBA_contig_78640_plus3 | 15 | zinc finger protein 84-like | 142 | 3.90E-24 | 60.35% | 3 | F:binding; F:nucleic acid binding; C:intracellular | IPR007087 (PROSITE); IPR013087 (G3DSA:3.30.160.GENE3D); IPR015880 (SMART); PTHR24377 (PANTHER), PTHR24377:SF0 (PANTHER), PF13465 (PFAM), SSF57667 (SUPERFAMILY) | |
| 3328 | lib_dib_Filter15_IDBA_contig_78643_minus1 | 12 | zinc finger protein 271-like | 142 | 8.73E-34 | 71.20% | 4 | F:nucleic acid binding; C:intracellular; F:protein binding; F:binding | IPR007087 (PROSITE); IPR013087 (G3DSA:3.30.160.GENE3D); IPR015880 (SMART); PTHR24387 (PANTHER), PF13465 (PFAM), SSF57667 (SUPERFAMILY) | |
| 3329 | lib_dib_Filter15_IDBA_contig_78707_minus1 | 47 | ---NA--- | 257 | | | 0 | - | SignalP-NN(euk) (SIGNALP) | yes |
| 3330 | lib_dib_Filter15_IDBA_contig_78707_minus2 | 47 | ---NA--- | 257 | | | 0 | - | SignalP-NN(euk) (SIGNALP), tmhmm (TMHMM) | yes |
| 3331 | lib_dib_Filter15_IDBA_contig_78707_minus3 | 47 | ---NA--- | 256 | | | 0 | - | SignalP-NN(euk) (SIGNALP), tmhmm (TMHMM) | |
| 3332 | lib_dib_Filter15_IDBA_contig_78708_minus1 | 45 | ---NA--- | 257 | | | 0 | - | SignalP-NN(euk) (SIGNALP) | yes |
| 3333 | lib_dib_Filter15_IDBA_contig_78708_minus3 | 45 | ---NA--- | 256 | | | 0 | - | SignalP-NN(euk) (SIGNALP), tmhmm (TMHMM) | |
| 3334 | lib_dib_Filter15_IDBA_contig_78743_plus2 | 14 | low-density lipoprotein receptor-related protein 2 | 142 | 3.02E-33 | 62.80% | 17 | P:protein transport; P:response to abiotic stimulus; P:biological_process; P:transport; P:cellular component organization; C:plasma membrane; P:cell-cell signaling; P:anatomical structure morphogenesis; P:multicellular organismal development; P:response to external stimulus; C:cell; P:metabolic process; C:extracellular space; F:protein binding; C:endosome; F:receptor activity; F:transporter activity | IPR002172 (G3DSA:4.10.400.GENE3D); IPR023415 (PROSITE); PR00261 (PRINTS), PTHR10529 (PANTHER) | |
| 3335 | lib_dib_Filter15_IDBA_contig_78789_minus3 | 11 | laccase- partial | 141 | 6.67E-28 | 56.00% | 3 | F:binding; F:catalytic activity; P:metabolic process | IPR002355 (PROSITE); IPR008972 (G3DSA:2.60.40.GENE3D); IPR011706 (PFAM); PTHR11709 (PANTHER), PTHR11709:SF8 (PANTHER), tmhmm (TMHMM) | |
| 3336 | lib_dib_Filter15_IDBA_contig_78859_plus3 | 9 | tx31_conte ame: full=substrate-specific endoprotease tex31 flags: precursor | 141 | 8.39E-06 | 51.00% | 4 | P:proteolysis; F:hydrolase activity; F:peptidase activity; C:extracellular region | IPR001283 (PANTHER); IPR014044 (G3DSA:3.40.33.GENE3D); tmhmm (TMHMM) | |
| 3337 | lib_dib_Filter15_IDBA_contig_78937_plus1 | 12 | collagen alpha-3 chain | 142 | 5.38E-38 | 60.15% | 2 | F:protein binding; F:calcium ion binding | IPR000152 (PROSITE); IPR000742 (PFAM); IPR001881 (SMART); IPR002035 (PFAM); IPR018097 (PROSITE); PR00010 (PRINTS), G3DSA:2.10.25.10 (GENE3D), G3DSA:3.40.50.410 (GENE3D), PTHR22992 (PANTHER), SSF53300 (SUPERFAMILY), SSF57196 (SUPERFAMILY) | |

| | | | | | | | | | | |
|---|---|---|---|---|---|---|---|---|---|---|
| 3338 | lib_dib_Filter15_IDBA_contig_78957_plus2 | 16 | low quality protein: cubilin-like | 142 | 3.87E-10 | 51.00% | 18 | P:single fertilization; F:metal ion binding; P:proteolysis; F:metallopeptidase activity; F:calcium ion binding; F:hydrolase activity; F:zinc ion binding; F:peptidase activity; F:metalloendopeptidase activity; C:membrane; P:axon guidance; C:axon; P:negative chemotaxis; P:heart development; P:semaphorin-plexin signaling pathway; P:neural crest cell migration; F:semaphorin receptor activity; P:cell adhesion | IPR000859 (G3DSA:2.60.120.GENE3D); PTHR10127 (PANTHER), PTHR10127:SF436 (PANTHER) | |
| 3339 | lib_dib_Filter15_IDBA_contig_79199_plus2 | 19 | choline dehydrogenase | 141 | 9.13E-20 | 73.25% | 2 | P:metabolic process; F:catalytic activity | IPR007867 (PFAM); G3DSA:3.50.50.60 (GENE3D), PTHR11552 (PANTHER), PTHR11552:SF10 (PANTHER), tmhmm (TMHMM), SSF51905 (SUPERFAMILY) | |
| 3340 | lib_dib_Filter15_IDBA_contig_79404_minus2 | 17 | gastrula zinc finger | 141 | 3.56E-24 | 53.90% | 3 | F:binding; F:nucleic acid binding; C:intracellular | IPR007087 (PFAM); IPR013087 (G3DSA:3.30.160.GENE3D); IPR015880 (SMART); IPR022755 (PFAM); PTHR11389 (PANTHER), PF13465 (PFAM), SSF57667 (SUPERFAMILY) | |
| 3341 | lib_dib_Filter15_IDBA_contig_79432_minus1 | 21 | prolow-density lipoprotein receptor-related protein 1 | 141 | 3.32E-09 | 52.95% | 3 | F:receptor activity; C:cell; F:protein binding | IPR001190 (PFAM); IPR002172 (G3DSA:4.10.400.GENE3D); IPR017448 (SUPERFAMILY); IPR023415 (PROSITE); PR00261 (PRINTS), G3DSA:3.10.250.10 (GENE3D), PTHR19331 (PANTHER) | |
| 3342 | lib_dib_Filter15_IDBA_contig_79436_plus1 | 20 | thrombospondin type-1 domain-containing protein 7b | 141 | 2.15E-27 | 48.30% | 0 | - | IPR000884 (PFAM); G3DSA:2.20.100.10 (GENE3D), PTHR11311 (PANTHER), PTHR11311:SF2 (PANTHER) | |
| 3343 | lib_dib_Filter15_IDBA_contig_79520_plus1 | 11 | fibrinogen-related protein | 141 | 9.05E-35 | 62.90% | 3 | F:receptor binding; C:extracellular space; P:signal transduction | IPR002181 (PFAM); IPR014715 (G3DSA:4.10.530.GENE3D); IPR014716 (G3DSA:3.90.215.GENE3D); PTHR19143 (PANTHER) | |
| 3344 | lib_dib_Filter15_IDBA_contig_79600_plus3 | 1193 | carboxypeptidase a2 | 294 | 2.92E-33 | 50.45% | 4 | F:peptidase activity; P:protein metabolic process; P:catabolic process; F:binding | IPR000834 (PRINTS); IPR003146 (G3DSA:3.30.70.GENE3D); IPR009020 (SUPERFAMILY); G3DSA:3.40.630.10 (GENE3D), PTHR11705 (PANTHER), SignalP-NN(euk) (SIGNALP), SSF53187 (SUPERFAMILY) | yes |
| 3345 | lib_dib_Filter15_IDBA_contig_79601_plus1 | 1106 | carboxypeptidase a2 | 280 | 2.67E-31 | 50.80% | 4 | F:peptidase activity; P:protein metabolic process; P:catabolic process; F:binding | IPR000834 (PRINTS); IPR003146 (G3DSA:3.30.70.GENE3D); IPR009020 (SUPERFAMILY); G3DSA:3.40.630.10 (GENE3D), PTHR11705 (PANTHER), SSF53187 (SUPERFAMILY) | |
| 3346 | lib_dib_Filter15_IDBA_contig_79668_minus1 | 9 | lipocalin precursor | 141 | 9.38E-100 | 85.75% | 6 | C:extracellular region; F:lipid binding; F:catalytic activity; P:transport; F:transporter activity; P:lipid metabolic process | IPR000566 (PFAM); IPR002972 (PRINTS); IPR011038 (SUPERFAMILY); IPR012674 (G3DSA:2.40.128.GENE3D); PTHR11430 (PANTHER), PTHR11430:SF32 (PANTHER) | |
| 3347 | lib_dib_Filter15_IDBA_contig_7968_minus1 | 138 | peptidyl-prolyl cis-trans isomerase-like 3 | 508 | 1.20E-86 | 86.35% | 6 | P:protein metabolic process; F:protein binding; P:cellular protein modification process; P:nucleobase-containing compound metabolic process; F:catalytic activity; C:nucleus | IPR002130 (PRINTS); IPR020892 (PROSITE); G3DSA:2.40.100.10 (GENE3D), PTHR11071 (PANTHER), tmhmm (TMHMM) | |
| 3348 | lib_dib_Filter15_IDBA_contig_79703_minus1 | 7 | protein suppressor of hairy wing | 141 | 5.37E-17 | 59.75% | 9 | F:DNA binding; F:chromatin binding; C:chromosome; F:protein binding; C:nucleus; P:regulation of gene expression, epigenetic; P:organelle organization; ; F:binding | IPR007087 (PFAM); IPR013087 (G3DSA:3.30.160.GENE3D); IPR015880 (SMART); PTHR11389 (PANTHER), PF13465 (PFAM), SignalP-NN(euk) (SIGNALP), tmhmm (TMHMM), SSF57667 (SUPERFAMILY) | |

| | | | | | | | | | |
|---|---|---|---|---|---|---|---|---|---|
| 3349 | lib_dib_Filter15_IDBA_contig_79735_minus3 | 12 | matrix metalloproteinase 9 | 140 | 1.81E-13 | 45.80% | 18 | F:carbohydrate binding; P:proteolysis; C:extracellular matrix; F:zinc ion binding; F:metalloendopeptidase activity; P:extracellular matrix organization; P:positive regulation of apoptotic process; P:positive regulation of keratinocyte migration; P:skeletal system development; F:hydrolase activity; P:embryo implantation; F:metallopeptidase activity; F:peptidase activity; P:collagen catabolic process; F:metal ion binding; P:metabolic process; C:extracellular space; C:proteinaceous extracellular matrix | IPR000562 (G3DSA:2.10.10.GENE3D); IPR013806 (SUPERFAMILY); PTHR22918 (PANTHER), PTHR22918:SF0 (PANTHER) |
| 3350 | lib_dib_Filter15_IDBA_contig_79744_plus1 | 14 | mitogen-activated protein kinase 7 | 141 | 5.12E-66 | 82.70% | 20 | P:regulation of biological process; P:protein transport; P:response to external stimulus; P:response to stress; C:nucleoplasm; P:signal transduction; F:protein binding; P:cell death; P:anatomical structure morphogenesis; P:embryo development; C:cytosol; P:cellular protein modification process; F:protein kinase activity; F:signal transducer activity; P:cell differentiation; P:protein metabolic process; P:multicellular organismal development; ; F:nucleotide binding; P:cell cycle | IPR000719 (PFAM); IPR002290 (SMART); IPR008271 (PROSITE); IPR011009 (SUPERFAMILY); G3DSA:1.10.510.10 (GENE3D), PTHR24055 (PANTHER), PTHR24055:SF17 (PANTHER) |
| 3351 | lib_dib_Filter15_IDBA_contig_7985_plus2 | 532 | ww domain-containing oxidoreductase | 841 | 1.44E-172 | 75.45% | 20 | P:cell differentiation; P:multicellular organismal development; P:biosynthetic process; P:lipid metabolic process; C:cell; F:catalytic activity; F:protein binding; C:Golgi apparatus; C:cytosol; P:signal transduction; F:binding; P:regulation of biological process; P:cell death; ; P:metabolic process; P:anatomical structure morphogenesis; C:plasma membrane; C:nucleus; C:mitochondrion; F:nucleotide binding | IPR001202 (PFAM); IPR002198 (PFAM); IPR002347 (PRINTS); IPR016040 (G3DSA:3.40.50.GENE3D); G3DSA:2.20.70.10 (GENE3D), PTHR24316 (PANTHER), PTHR24316:SF43 (PANTHER), SSF51735 (SUPERFAMILY) |
| 3352 | lib_dib_Filter15_IDBA_contig_79902_plus1 | 13 | protein wnt-7b | 140 | 2.58E-60 | 71.05% | 19 | P:signal transduction; C:cell; P:cell differentiation; P:multicellular organismal development; P:regulation of biological process; F:receptor binding; P:biological_process; P:metabolic process; P:anatomical structure morphogenesis; P:growth; P:embryo development; P:cell proliferation; P:response to stress; F:signal transducer activity; P:cellular component organization; C:proteinaceous extracellular matrix; P:cell-cell signaling; ; P:cell death | IPR005817 (PRINTS) |
| 3353 | lib_dib_Filter15_IDBA_contig_7995_plus1 | 1591 | protein acr-11 | 286 | 1.33E-29 | 51.05% | 3 | C:cell; P:ion transport; F:ion channel activity | IPR006201 (PANTHER); IPR006202 (G3DSA:2.70.170.GENE3D) |
| 3354 | lib_dib_Filter15_IDBA_contig_79967_plus2 | 14 | dnaj homolog subfamily c member 22-like | 140 | 4.20E-28 | 66.45% | 7 | C:endosome; P:multicellular organismal development; C:plasma membrane; C:organelle; C:cytoplasm; P:cellular component organization; P:transport | IPR001623 (PRINTS); PTHR24078 (PANTHER) |
| 3355 | lib_dib_Filter15_IDBA_contig_80121_plus2 | 52 | proopiomelanocortin | 140 | 1.84E-27 | 82.25% | 3 | C:extracellular region; P:signal transduction; F:receptor binding | IPR013593 (PFAM); PTHR11416 (PANTHER), PTHR11416:SF6 (PANTHER), tmhmm (TMHMM) |

| | | | | | | | | | | |
|---|---|---|---|---|---|---|---|---|---|---|
| 3356 | lib_dib_Filter15_IDBA_contig_80137_plus1 | 1123 | ankyrin repeat protein | 304 | 2.52E-19 | 50.30% | 1 | F:protein binding | IPR002110 (SMART); IPR020683 (G3DSA:1.25.40.GENE3D); PTHR24198 (PANTHER) | |
| 3357 | lib_dib_Filter15_IDBA_contig_80138_plus1 | 2325 | ankyrin repeat protein | 480 | 1.80E-36 | 52.65% | 2 | F:catalytic activity; F:protein binding | IPR002110 (PFAM); IPR020683 (G3DSA:1.25.40.GENE3D); PTHR24198 (PANTHER) | |
| 3358 | lib_dib_Filter15_IDBA_contig_8016_minus1 | 461 | stromal cell-derived factor 2 | 353 | 1.70E-97 | 71.65% | 1 | C:cell | IPR016093 (PFAM); IPR027005 (PANTHER); G3DSA:2.80.10.50 (GENE3D), SignalP-NN(euk) (SIGNALP) | |
| 3359 | lib_dib_Filter15_IDBA_contig_80161_minus3 | 14 | carbonic anhydrase 4-like | 139 | 4.95E-25 | 55.65% | 11 | F:metal ion binding; P:one-carbon metabolic process; F:zinc ion binding; F:carbonate dehydratase activity; C:cellular_component; F:lyase activity; P:secretion; C:apical part of cell; P:carbon dioxide transport; C:cytosol; P:morphogenesis of an epithelium | IPR001148 (G3DSA:3.10.200.GENE3D); IPR023561 (PANTHER) | |
| 3360 | lib_dib_Filter15_IDBA_contig_80173_minus2 | 15 | prolow-density lipoprotein receptor-related protein 1 | 140 | 1.20E-47 | 71.15% | 17 | P:protein transport; P:metabolic process; C:organelle; C:intracellular; P:transport; P:regulation of biological process; F:calcium ion binding; F:receptor binding; P:signal transduction; P:cytoskeleton organization; P:biological_process; F:protein binding; P:cellular component organization; F:receptor activity; F:transporter activity; C:plasma membrane; C:cytoplasm | IPR000033 (PFAM); IPR011042 (G3DSA:2.120.10.GENE3D); PTHR10529 (PANTHER), PTHR10529:SF116 (PANTHER), SSF63825 (SUPERFAMILY) | |
| 3361 | lib_dib_Filter15_IDBA_contig_80201_minus3 | 12 | variable lymphocyte receptor c | 139 | 6.23E-14 | 53.35% | 14 | P:anatomical structure morphogenesis; P:cellular component organization; P:cell differentiation; P:multicellular organismal development; P:cell recognition; F:receptor binding; P:cell-cell signaling; P:reproduction; P:embryo development; F:carbohydrate binding; P:regulation of biological process; P:behavior; P:response to external stimulus; C:extracellular region | IPR000372 (PFAM); IPR001611 (PROFILE); IPR003591 (SMART); G3DSA:3.80.10.10 (GENE3D), PTHR24365 (PANTHER), PF13855 (PFAM), SSF52058 (SUPERFAMILY) | |
| 3362 | lib_dib_Filter15_IDBA_contig_80282_minus1 | 9749 | nicotinic acetylcholine receptor subunit alpha 6 transcript variant partial | 371 | 4.52E-30 | 49.25% | 3 | P:ion transport; C:cell; F:ion channel activity | IPR006201 (PANTHER); IPR006202 (G3DSA:2.70.170.GENE3D); PTHR18945:SF66 (PANTHER) | |
| 3363 | lib_dib_Filter15_IDBA_contig_80318_plus1 | 15 | a disintegrin and metalloproteinase with thrombospondin motifs 6 | 140 | 1.55E-12 | 51.30% | 3 | F:peptidase activity; P:protein metabolic process; P:catabolic process | IPR001590 (PROFILE); IPR024079 (G3DSA:3.40.390.GENE3D); PTHR13723 (PANTHER), PF13574 (PFAM), SSF55486 (SUPERFAMILY) | |
| 3364 | lib_dib_Filter15_IDBA_contig_80466_plus3 | 17135 | cubilin | 683 | 1.58E-56 | 45.10% | 4 | F:calcium ion binding; F:transferase activity; F:hydrolase activity; F:transmembrane receptor protein tyrosine kinase activity | IPR000859 (G3DSA:2.60.120.GENE3D); PTHR10127 (PANTHER), SignalP-NN(euk) (SIGNALP), tmhmm (TMHMM) | yes |
| 3365 | lib_dib_Filter15_IDBA_contig_80467_plus2 | 277 | low quality protein: cubilin | 136 | 8.22E-20 | 61.70% | 4 | C:organelle; C:intracellular; C:cytoplasm; C:cell | IPR000859 (G3DSA:2.60.120.GENE3D); PTHR10127 (PANTHER) | |
| 3366 | lib_dib_Filter15_IDBA_contig_80468_plus2 | 399 | extracellular serine proteinase-like | 126 | 3.86E-10 | 56.30% | 1 | F:peptidase activity | IPR000859 (G3DSA:2.60.120.GENE3D); PTHR10127 (PANTHER) | |
| 3367 | lib_dib_Filter15_IDBA_contig_80476_minus3 | 517 | venom dipeptidyl peptidase 4-like | 256 | 2.06E-11 | 58.95% | 3 | P:protein metabolic process; P:catabolic process; C:cell | IPR002469 (PFAM); G3DSA:2.140.10.30 (GENE3D), PTHR11731 (PANTHER), PTHR11731:SF24 (PANTHER), tmhmm (TMHMM), SSF82171 (SUPERFAMILY) | |

| | | | | | | | | | |
|---|---|---|---|---|---|---|---|---|---|
| 3368 | lib_dib_Filter15_IDBA_contig_80494_plus1 | 12 | zinc finger protein 271-like | 139 | 1.10E-28 | 59.15% | 3 | F:binding; F:nucleic acid binding; C:intracellular | IPR007087 (PROSITE); IPR013087 (G3DSA:3.30.160.GENE3D); IPR015880 (SMART); PTHR11389 (PANTHER), PF13465 (PFAM), PF13894 (PFAM), SSF57667 (SUPERFAMILY) |
| 3369 | lib_dib_Filter15_IDBA_contig_80588_minus2 | 15 | g protein-coupled receptor kinase 4 | 139 | 5.32E-67 | 89.65% | 13 | C:cell; C:cytosol; P:transport; P:cellular component organization; P:signal transduction; P:metabolic process; P:cellular protein modification process; F:protein kinase activity; P:regulation of biological process; P:ion transport; F:receptor activity; F:nucleotide binding; C:cytoplasm | IPR000719 (PFAM); IPR002290 (SMART); IPR008271 (PROSITE); IPR011009 (SUPERFAMILY); G3DSA:1.10.510.10 (GENE3D), PTHR24355 (PANTHER), PTHR24355:SF4 (PANTHER) |
| 3370 | lib_dib_Filter15_IDBA_contig_80597_plus2 | 12 | zinc finger protein 135- partial | 139 | 7.94E-50 | 55.00% | 3 | F:binding; F:nucleic acid binding; C:intracellular | IPR007087 (PFAM); IPR013087 (G3DSA:3.30.160.GENE3D); IPR015880 (SMART); PTHR11389 (PANTHER), PF13465 (PFAM), PF13894 (PFAM), SSF57667 (SUPERFAMILY) |
| 3371 | lib_dib_Filter15_IDBA_contig_80605_plus1 | 21 | paramyosin | 139 | 3.86E-34 | 70.95% | 3 | C:cytoskeleton; C:protein complex; F:motor activity | IPR002928 (PFAM); PTHR13140 (PANTHER), PTHR13140:SF106 (PANTHER) |
| 3372 | lib_dib_Filter15_IDBA_contig_80744_minus1 | 19 | protocadherin fat 4 | 139 | 5.16E-47 | 53.65% | 3 | F:calcium ion binding; P:biological_process; C:cell | IPR002126 (G3DSA:2.60.40.GENE3D); IPR015919 (SUPERFAMILY); PTHR24027 (PANTHER), PTHR24027:SF110 (PANTHER) |
| 3373 | lib_dib_Filter15_IDBA_contig_80827_minus1 | 15 | zinc finger protein 271 (zinc finger protein 7) (zinc finger protein znfphex133) (epstein-barr virus-induced zinc finger protein) (znf-eb) (ct-zfp48) (zinc finger protein | 139 | 6.83E-22 | 60.35% | 4 | F:protein binding; F:binding; F:nucleic acid binding; C:intracellular | IPR004172 (PROFILE); IPR007087 (PROSITE); IPR013087 (G3DSA:3.30.160.GENE3D); IPR015880 (SMART); IPR022755 (PFAM); PTHR11389 (PANTHER), PF13465 (PFAM), PF13912 (PFAM), SSF57667 (SUPERFAMILY) |
| 3374 | lib_dib_Filter15_IDBA_contig_80934_plus3 | 39 | insulin-degrading enzyme | 138 | 2.29E-54 | 73.80% | 23 | P:protein metabolic process; P:catabolic process; P:multicellular organismal development; P:cellular component organization; F:binding; F:receptor binding; P:signal transduction; P:response to endogenous stimulus; C:cytosol; C:protein complex; C:extracellular space; F:protein binding; C:cell; P:regulation of biological process; F:peptidase activity; P:metabolic process; F:hydrolase activity; P:nucleobase-containing compound metabolic process; P:cellular homeostasis; C:nucleus; C:peroxisome; F:nucleotide binding; C:mitochondrion | IPR007863 (PFAM); IPR011237 (G3DSA:3.30.830.GENE3D); IPR011249 (SUPERFAMILY); PTHR11851 (PANTHER), PTHR11851:SF64 (PANTHER) |
| 3375 | lib_dib_Filter15_IDBA_contig_81002_plus3 | 15 | matrix metalloproteinase-9 | 138 | 2.08E-12 | 45.60% | 18 | F:carbohydrate binding; P:extracellular matrix organization; P:positive regulation of apoptotic process; P:positive regulation of keratinocyte migration; F:metalloendopeptidase activity; P:skeletal system development; F:zinc ion binding; P:embryo implantation; C:proteinaceous extracellular matrix; C:extracellular matrix; P:proteolysis; P:collagen catabolic process; C:extracellular space; F:hydrolase activity; F:metallopeptidase activity; F:peptidase activity; F:metal ion binding; P:metabolic process | IPR000562 (G3DSA:2.10.10.GENE3D); IPR013806 (SUPERFAMILY); PTHR22918 (PANTHER), PTHR22918:SF0 (PANTHER) |
| 3376 | lib_dib_Filter15_IDBA_contig_8102_minus1 | 85 | ---NA--- | 178 | | | 0 | - | no IPS match |

| | | | | | | | | | |
|---|---|---|---|---|---|---|---|---|---|
| 3377 | lib_dib_Filter15_IDBA_contig_81042_plus3 | 15 | beta-( )-xylosyltransferase | 138 | 6.31E-49 | 60.05% | 2 | F:transferase activity, transferring glycosyl groups; F:transferase activity | IPR007657 (PFAM); PTHR20961 (PANTHER), PTHR20961:SF0 (PANTHER) | |
| 3378 | lib_dib_Filter15_IDBA_contig_81054_plus3 | 23 | cysteine-rich secretory protein mr30 | 138 | 1.36E-17 | 63.80% | 2 | C:cell; P:metabolic process | IPR001283 (PANTHER); IPR014044 (G3DSA:3.40.33.GENE3D); SignalP-NN(euk) (SIGNALP), tmhmm (TMHMM) | |
| 3379 | lib_dib_Filter15_IDBA_contig_81164_minus1 | 11 | low-density lipoprotein receptor-related protein 2 | 138 | 1.27E-11 | 58.65% | 5 | P:biological_process; P:multicellular organismal development; P:transport; P:cellular component organization; F:protein binding | IPR002172 (G3DSA:4.10.400.GENE3D); IPR023415 (PROSITE); PR00261 (PRINTS), PTHR10529 (PANTHER) | |
| 3380 | lib_dib_Filter15_IDBA_contig_81197_plus2 | 43 | neurotransmitter-gated ion-channel ligand binding domain protein | 235 | 1.83E-45 | 58.20% | 3 | P:ion transport; C:cell; F:ion channel activity | IPR006201 (PRINTS); IPR006202 (G3DSA:2.70.170.GENE3D); IPR018000 (PROSITE); PTHR18945:SF92 (PANTHER), SignalP-NN(euk) (SIGNALP), tmhmm (TMHMM) | yes |
| 3381 | lib_dib_Filter15_IDBA_contig_81414_plus2 | 22 | l-xylulose reductase | 111 | 1.28E-18 | 68.15% | 7 | P:carbohydrate metabolic process; C:cytoplasm; F:catalytic activity; C:organelle; C:intracellular; P:cellular component organization; F:nucleotide binding | IPR002198 (PRINTS); IPR002347 (PRINTS); IPR016040 (G3DSA:3.40.50.GENE3D); PTHR24311 (PANTHER), PTHR24311:SF0 (PANTHER), PF13561 (PFAM), SSF51735 (SUPERFAMILY) | |
| 3382 | lib_dib_Filter15_IDBA_contig_81469_plus1 | 14 | protein | 138 | 1.65E-34 | 58.00% | 1 | F:calcium ion binding | IPR001881 (PFAM); IPR018097 (PROSITE); G3DSA:2.10.25.10 (GENE3D), PTHR10529 (PANTHER), SSF57196 (SUPERFAMILY) | |
| 3383 | lib_dib_Filter15_IDBA_contig_81473_minus3 | 13 | cytoplasmic tyrosine-protein kinase bmx | 137 | 2.64E-14 | 63.25% | 3 | F:binding; F:protein kinase activity; P:cellular protein modification process | IPR001245 (PFAM); IPR011009 (SUPERFAMILY); G3DSA:1.10.510.10 (GENE3D), PTHR24418 (PANTHER) | |
| 3384 | lib_dib_Filter15_IDBA_contig_81475_minus2 | 11 | torsin family member a (torsin a) | 138 | 5.96E-21 | 52.10% | 5 | C:organelle; C:intracellular; C:endoplasmic reticulum; F:nucleotide binding; P:protein metabolic process | IPR010448 (PANTHER) | |
| 3385 | lib_dib_Filter15_IDBA_contig_81478_plus2 | 12 | arylsulfatase j | 138 | 1.26E-36 | 66.60% | 2 | P:metabolic process; F:hydrolase activity | IPR000917 (PFAM); IPR017849 (G3DSA:3.40.720.GENE3D); IPR017850 (SUPERFAMILY); PTHR10342 (PANTHER), PTHR10342:SF19 (PANTHER), tmhmm (TMHMM) | |
| 3386 | lib_dib_Filter15_IDBA_contig_81703_plus3 | 16 | netrin receptor unc5c | 137 | 1.31E-44 | 75.25% | 9 | P:anatomical structure morphogenesis; P:cellular component organization; P:cell differentiation; P:multicellular organismal development; F:receptor activity; P:regulation of biological process; P:cell death; F:protein binding; C:plasma membrane | IPR000884 (PFAM); IPR013783 (G3DSA:2.60.40.GENE3D); PR01705 (PRINTS), G3DSA:2.20.100.10 (GENE3D), PTHR12582 (PANTHER), SSF48726 (SUPERFAMILY) | |
| 3387 | lib_dib_Filter15_IDBA_contig_81978_minus3 | 230 | tissue factor pathway inhibitor | 366 | 2.44E-21 | 50.95% | 1 | F:enzyme regulator activity | IPR002223 (PRINTS); IPR020901 (PROSITE); PTHR10083 (PANTHER), SignalP-NN(euk) (SIGNALP), tmhmm (TMHMM) | |
| 3388 | lib_dib_Filter15_IDBA_contig_81979_minus1 | 52 | tissue factor pathway inhibitor | 137 | 4.63E-27 | 53.25% | 2 | C:extracellular region; F:enzyme regulator activity | IPR002223 (PRINTS); IPR020901 (PROSITE); PTHR10083 (PANTHER) | |
| 3389 | lib_dib_Filter15_IDBA_contig_82014_minus2 | 17 | zinc finger protein 782- partial | 137 | 1.64E-37 | 59.60% | 3 | F:binding; F:nucleic acid binding; C:intracellular | IPR007087 (PROSITE); IPR013087 (G3DSA:3.30.160.GENE3D); IPR015880 (SMART); PTHR11389 (PANTHER), PF13465 (PFAM), SSF57667 (SUPERFAMILY) | |
| 3390 | lib_dib_Filter15_IDBA_contig_82047_plus3 | 15 | testis-expressed sequence 264 protein | 136 | 2.68E-49 | 71.95% | 3 | F:molecular_function; P:biological_process; C:cellular_component | IPR010499 (PFAM); G3DSA:3.20.80.10 (GENE3D), PTHR15949 (PANTHER), PTHR15949:SF0 (PANTHER) | |
| 3391 | lib_dib_Filter15_IDBA_contig_82123_plus1 | 3726 | protein-glutamine gamma-glutamyltransferase k | 263 | 6.80E-45 | 56.15% | 3 | P:biological_process; F:transferase activity; P:cellular protein modification process | IPR001102 (PFAM); IPR013783 (G3DSA:2.60.40.GENE3D); IPR014756 (SUPERFAMILY); IPR023608 (PANTHER); G3DSA:3.90.260.10 (GENE3D), SSF54001 (SUPERFAMILY) | |

| | | | | | | | | | | |
|---|---|---|---|---|---|---|---|---|---|---|
| 3392 | lib_dib_Filter15_IDBA_contig_82125_plus2 | 57 | reelin | 167 | 2.18E-34 | 63.90% | 17 | P:multicellular organismal development; F:receptor binding; F:catalytic activity; P:anatomical structure morphogenesis; P:cellular component organization; P:cell differentiation; P:signal transduction; P:behavior; C:extracellular space; P:metabolic process; P:response to stress; C:cytoplasm; C:proteinaceous extracellular matrix; P:regulation of biological process; ; P:cell-cell signaling; C:cell | PTHR11841 (PANTHER), PTHR11841:SF0 (PANTHER) | |
| 3393 | lib_dib_Filter15_IDBA_contig_82126_plus1 | 258 | reelin isoform 1 | 379 | 6.76E-20 | 62.00% | 16 | P:multicellular organismal development; F:receptor binding; P:anatomical structure morphogenesis; P:cellular component organization; P:cell differentiation; P:signal transduction; P:behavior; C:extracellular space; P:metabolic process; P:response to stress; C:cytoplasm; C:proteinaceous extracellular matrix; P:regulation of biological process; ; P:cell-cell signaling; C:cell | PTHR11841 (PANTHER), PTHR11841:SF0 (PANTHER) | |
| 3394 | lib_dib_Filter15_IDBA_contig_82126_plus2 | 258 | reelin | 378 | 7.93E-48 | 55.85% | 20 | F:receptor binding; F:catalytic activity; P:multicellular organismal development; P:anatomical structure morphogenesis; P:cellular component organization; P:cell differentiation; P:response to external stimulus; P:response to abiotic stimulus; P:signal transduction; P:biological_process; P:behavior; C:extracellular space; C:cell; P:response to endogenous stimulus; P:metabolic process; P:response to stress; C:cytoplasm; P:regulation of biological process; ; P:cell-cell signaling | IPR013032 (PROSITE); G3DSA:2.10.25.10 (GENE3D), PTHR11841 (PANTHER), PTHR11841:SF0 (PANTHER) | |
| 3395 | lib_dib_Filter15_IDBA_contig_82127_plus1 | 43 | reelin isoform 1 | 137 | 2.04E-36 | 62.70% | 17 | P:multicellular organismal development; F:receptor binding; F:catalytic activity; P:anatomical structure morphogenesis; P:cellular component organization; P:cell differentiation; P:signal transduction; P:behavior; C:extracellular space; P:metabolic process; P:response to stress; C:cytoplasm; C:proteinaceous extracellular matrix; P:regulation of biological process; ; P:cell-cell signaling; C:cell | PTHR11841 (PANTHER), PTHR11841:SF0 (PANTHER) | |
| 3396 | lib_dib_Filter15_IDBA_contig_82179_plus2 | 17 | multiple epidermal growth factor-like domains protein 6-like | 137 | 2.09E-42 | 62.95% | 4 | P:multicellular organismal development; P:cell differentiation; P:biological_process; F:protein binding | IPR000742 (SMART); IPR002049 (SMART); IPR013032 (PFAM); PR00011 (PRINTS), G3DSA:2.170.300.10 (GENE3D), PTHR24035 (PANTHER), PTHR24035:SF4 (PANTHER), SSF57196 (SUPERFAMILY) | |
| 3397 | lib_dib_Filter15_IDBA_contig_82241_plus3 | 20 | group xiia secretory phospholipase a2-like | 180 | 7.95E-29 | 54.60% | 5 | F:hydrolase activity; F:calcium ion binding; C:extracellular region; P:catabolic process; P:lipid metabolic process | IPR010711 (PANTHER); IPR013090 (PROSITE); IPR016090 (G3DSA:1.20.90.GENE3D); SignalP-NN(euk) (SIGNALP) | yes |
| 3398 | lib_dib_Filter15_IDBA_contig_82291_minus3 | 13 | short-chain dehydrogenase reductase 3 | 136 | 1.76E-48 | 72.90% | 6 | F:catalytic activity; F:electron carrier activity; P:lipid metabolic process; P:secondary metabolic process; P:biological_process; F:nucleotide binding | IPR002198 (PFAM); IPR002347 (PRINTS); IPR016040 (G3DSA:3.40.50.GENE3D); PTHR24316 (PANTHER), PTHR24316:SF1 (PANTHER), SSF51735 (SUPERFAMILY) | |

| | | | | | | | | | |
|---|---|---|---|---|---|---|---|---|---|
| 3399 | lib_dib_Filter15_IDBA_contig_82415_minus1 | 12 | heparanase isoform 3 | 137 | 5.16E-35 | 61.75% | 7 | P:multicellular organismal development; P:biological_process; P:regulation of biological process; P:response to external stimulus; P:response to stress; F:catalytic activity; C:cell | IPR005199 (PANTHER); IPR017853 (SUPERFAMILY); PTHR14363:SF1 (PANTHER), SignalP-NN(euk) (SIGNALP) |
| 3400 | lib_dib_Filter15_IDBA_contig_82651_plus1 | 22 | signal cub egf-like 1 | 136 | 2.35E-45 | 64.15% | 13 | P:signal transduction; C:cytoplasmic membrane-bounded vesicle; C:plasma membrane; F:calcium ion binding; P:response to external stimulus; P:response to stress; P:multicellular organismal development; P:cellular component organization; F:protein binding; C:extracellular space; P:cell differentiation; P:embryo development; P:biological_process | IPR009030 (SUPERFAMILY); IPR011641 (PFAM); PTHR24046 (PANTHER), PTHR24046:SF0 (PANTHER) |
| 3401 | lib_dib_Filter15_IDBA_contig_82668_minus1 | 16 | hypotherical protein | 136 | 5.15E-46 | 68.85% | 6 | C:lysosome; F:binding; F:peptidase activity; P:biological_process; P:protein metabolic process; P:catabolic process | IPR000668 (PFAM); IPR013128 (PANTHER); G3DSA:3.90.70.10 (GENE3D), PTHR12411:SF17 (PANTHER), SSF54001 (SUPERFAMILY) |
| 3402 | lib_dib_Filter15_IDBA_contig_8269_plus1 | 3421 | ---NA--- | 187 | | | 0 | - | IPR008160 (PFAM); PTHR24023 (PANTHER), PTHR24023:SF164 (PANTHER) |
| 3403 | lib_dib_Filter15_IDBA_contig_82806_plus1 | 18 | protein kinase c-binding protein nell1 | 136 | 4.39E-45 | 66.45% | 2 | F:protein binding; F:calcium ion binding | IPR000152 (PROSITE); IPR000742 (SMART); IPR001881 (PFAM); IPR013032 (PFAM); IPR018097 (PROSITE); IPR023413 (G3DSA:2.40.155.GENE3D); IPR024731 (PFAM); G3DSA:2.10.25.10 (GENE3D), PTHR24042 (PANTHER), SSF57196 (SUPERFAMILY) |
| 3404 | lib_dib_Filter15_IDBA_contig_83127_plus3 | 15 | deleted in malignant brain tumors 1 | 135 | 1.32E-09 | 56.75% | 5 | F:scavenger receptor activity; C:membrane; F:carbohydrate binding; P:cell adhesion; F:calcium ion binding | IPR000859 (G3DSA:2.60.120.GENE3D); PTHR10127 (PANTHER) |
| 3405 | lib_dib_Filter15_IDBA_contig_83134_plus3 | 12 | fibrinogen c domain-containing protein 1-like | 135 | 7.29E-28 | 60.15% | 3 | F:receptor binding; C:extracellular space; P:signal transduction | IPR002181 (PFAM); IPR014715 (G3DSA:4.10.530.GENE3D); IPR014716 (G3DSA:3.90.215.GENE3D); PTHR19143 (PANTHER) |
| 3406 | lib_dib_Filter15_IDBA_contig_83168_plus1 | 161 | corticosteroid 11-beta-dehydrogenase isozyme 1 | 339 | 1.50E-21 | 57.40% | 6 | F:catalytic activity; P:biosynthetic process; P:lipid metabolic process; C:endoplasmic reticulum; C:cell; F:nucleotide binding | IPR002198 (PFAM); IPR016040 (G3DSA:3.40.50.GENE3D); IPR020904 (PROSITE); PTHR24322 (PANTHER), PTHR24322:SF32 (PANTHER), SSF51735 (SUPERFAMILY) |
| 3407 | lib_dib_Filter15_IDBA_contig_83169_plus2 | 398 | corticosteroid 11-beta-dehydrogenase isozyme 1 | 578 | 2.11E-53 | 57.20% | 6 | F:catalytic activity; P:biosynthetic process; P:lipid metabolic process; C:endoplasmic reticulum; C:cell; F:nucleotide binding | IPR002198 (PFAM); IPR002347 (PRINTS); IPR016040 (G3DSA:3.40.50.GENE3D); IPR020904 (PROSITE); PTHR24322 (PANTHER), PTHR24322:SF32 (PANTHER), tmhmm (TMHMM), SSF51735 (SUPERFAMILY) |
| 3408 | lib_dib_Filter15_IDBA_contig_83170_plus3 | 422 | corticosteroid 11-beta-dehydrogenase isozyme 1 | 577 | 6.95E-54 | 56.90% | 6 | F:catalytic activity; P:biosynthetic process; P:lipid metabolic process; C:endoplasmic reticulum; C:cell; F:nucleotide binding | IPR002198 (PFAM); IPR002347 (PRINTS); IPR016040 (G3DSA:3.40.50.GENE3D); IPR020904 (PROSITE); PTHR24322 (PANTHER), PTHR24322:SF32 (PANTHER), tmhmm (TMHMM), SSF51735 (SUPERFAMILY) |
| 3409 | lib_dib_Filter15_IDBA_contig_83199_minus3 | 25 | insulin-like growth factor-binding protein 4 precursor | 135 | 2.33E-12 | 53.50% | 8 | P:signal transduction; P:primary metabolic process; C:extracellular region; P:cell proliferation; P:metabolic process; P:regulation of biological process; P:cell growth; F:protein binding | IPR000867 (PFAM); IPR009030 (SUPERFAMILY); IPR009168 (PANTHER); G3DSA:4.10.40.20 (GENE3D), SignalP-NN(euk) (SIGNALP), tmhmm (TMHMM) |
| 3410 | lib_dib_Filter15_IDBA_contig_83395_plus1 | 21 | zinc finger protein | 135 | 2.24E-34 | 63.80% | 3 | F:binding; F:nucleic acid binding; C:intracellular | IPR007087 (PROSITE); IPR013087 (G3DSA:3.30.160.GENE3D); IPR015880 (SMART); PTHR11389 (PANTHER), PF13465 (PFAM), SSF57667 (SUPERFAMILY) |

| | | | | | | | | | |
|---|---|---|---|---|---|---|---|---|---|
| 3411 | lib_dib_Filter15_IDBA_contig_83412_plus1 | 18855 | heat shock protein 70 | 135 | 2.87E-88 | 97.30% | 2 | F:nucleotide binding; P:response to stress | IPR013126 (PRINTS); IPR018181 (PROSITE); G3DSA:3.30.420.40 (GENE3D), PTHR19375 (PANTHER), PTHR19375:SF1 (PANTHER), SSF53067 (SUPERFAMILY) |
| 3412 | lib_dib_Filter15_IDBA_contig_83413_plus1 | 23844 | heat shock protein 70 | 131 | 1.02E-82 | 97.35% | 2 | F:nucleotide binding; P:response to stress | IPR013126 (PFAM); IPR018181 (PROSITE); G3DSA:3.30.420.40 (GENE3D), G3DSA:3.90.640.10 (GENE3D), PTHR19375 (PANTHER), SSF53067 (SUPERFAMILY) |
| 3413 | lib_dib_Filter15_IDBA_contig_83414_plus1 | 7484 | heat shock protein 70 | 116 | 1.06E-25 | 97.75% | 24 | C:cell; F:receptor binding; P:cellular component organization; P:transport; P:cell-cell signaling; C:cytoplasmic membrane-bounded vesicle; F:nucleotide binding; P:regulation of biological process; ; C:nucleolus; C:cytosol; P:cell cycle; F:protein binding; C:organelle; C:extracellular region; P:protein metabolic process; C:protein complex; F:hydrolase activity; C:plasma membrane; P:nucleobase-containing compound metabolic process; C:intracellular; P:response to stress; P:response to biotic stimulus; P:biological_process | IPR013126 (PRINTS); IPR018181 (PROSITE); G3DSA:3.30.420.40 (GENE3D), PTHR19375 (PANTHER), SSF53067 (SUPERFAMILY) |
| 3414 | lib_dib_Filter15_IDBA_contig_83415_plus1 | 17733 | heat shock protein 70 | 115 | 1.19E-24 | 90.20% | 25 | C:cell; F:receptor binding; P:cellular component organization; P:transport; P:cell-cell signaling; C:cytoplasmic membrane-bounded vesicle; F:nucleotide binding; P:regulation of biological process; ; C:nucleolus; C:cytosol; P:cell cycle; F:protein binding; C:organelle; C:extracellular region; P:protein metabolic process; C:protein complex; F:hydrolase activity; P:nucleobase-containing compound metabolic process; P:catabolic process; C:plasma membrane; C:intracellular; P:response to stress; P:response to biotic stimulus; P:biological_process | IPR013126 (PFAM); G3DSA:1.20.1270.10 (GENE3D), PTHR19375 (PANTHER), SSF100934 (SUPERFAMILY) |
| 3415 | lib_dib_Filter15_IDBA_contig_83455_plus3 | 9 | uncharacterized aarf domain-containing protein kinase 5 | 134 | 3.27E-63 | 82.90% | 5 | P:cellular protein modification process; F:nucleotide binding; C:cell; F:protein kinase activity; C:mitochondrion | IPR004147 (PFAM); PTHR10566 (PANTHER), PTHR10566:SF25 (PANTHER) |
| 3416 | lib_dib_Filter15_IDBA_contig_835_plus1 | 75 | tissue alpha-l-fucosidase | 153 | 4.08E-15 | 61.55% | 3 | C:cytoplasm; P:carbohydrate metabolic process; F:hydrolase activity | IPR000933 (PANTHER); PTHR10030:SF2 (PANTHER), SignalP-NN(euk) (SIGNALP) |
| 3417 | lib_dib_Filter15_IDBA_contig_83505_plus2 | 12 | zinc finger protein 84 | 135 | 1.01E-33 | 62.00% | 4 | ; F:nucleic acid binding; C:intracellular; F:binding | IPR007087 (PROSITE); IPR013087 (G3DSA:3.30.160.GENE3D); IPR015880 (SMART); PTHR24375 (PANTHER), PTHR24375:SF0 (PANTHER), PF13465 (PFAM), SSF57667 (SUPERFAMILY) |
| 3418 | lib_dib_Filter15_IDBA_contig_83508_minus2 | 12 | ankyrin domain protein | 135 | 1.86E-28 | 58.25% | 1 | F:protein binding | IPR002110 (PFAM); IPR020683 (G3DSA:1.25.40.GENE3D); PTHR24198 (PANTHER) |
| 3419 | lib_dib_Filter15_IDBA_contig_836_plus1 | 62 | tissue alpha-l-fucosidase | 153 | 1.51E-14 | 61.55% | 3 | C:cytoplasm; P:carbohydrate metabolic process; F:hydrolase activity | IPR000933 (PANTHER); PTHR10030:SF2 (PANTHER), SignalP-NN(euk) (SIGNALP) |
| 3420 | lib_dib_Filter15_IDBA_contig_83621_minus1 | 12 | oocyte zinc finger protein 6-like | 135 | 7.60E-43 | 66.50% | 3 | C:nucleus; F:binding; F:nucleic acid binding | IPR007087 (PROSITE); IPR013087 (G3DSA:3.30.160.GENE3D); IPR015880 (SMART); PTHR24402 (PANTHER), PF13465 (PFAM), SSF57667 (SUPERFAMILY) |

| | | | | | | | | | |
|---|---|---|---|---|---|---|---|---|---|
| 3421 | lib_dib_Filter15_IDBA_contig_83649_plus2 | 17 | torsin family member b (torsin b) | 134 | 4.04E-60 | 77.90% | 8 | P:protein metabolic process; P:cellular component organization; F:nucleotide binding; C:endoplasmic reticulum; F:protein binding; P:response to stress; P:response to biotic stimulus; C:nuclear envelope | IPR010448 (PANTHER); G3DSA:3.40.50.300 (GENE3D), PTHR10760:SF2 (PANTHER), SSF52540 (SUPERFAMILY) |
| 3422 | lib_dib_Filter15_IDBA_contig_83738_plus3 | 27 | low-density lipoprotein receptor | 188 | 4.36E-54 | 61.00% | 7 | F:calcium ion binding; F:receptor activity; C:integral to membrane; C:membrane; F:transferase activity; F:hydrolase activity; F:transmembrane receptor protein tyrosine kinase activity | IPR000033 (PFAM); IPR011042 (G3DSA:2.120.10.GENE3D); PTHR10529 (PANTHER), PTHR10529:SF113 (PANTHER), SSF63825 (SUPERFAMILY) |
| 3423 | lib_dib_Filter15_IDBA_contig_8376_plus3 | 67 | matrix metallo protein ase-17 | 218 | 1.26E-23 | 56.45% | 2 | F:hydrolase activity; C:cell | IPR000585 (G3DSA:2.110.10.GENE3D); IPR018487 (PFAM); PTHR10201 (PANTHER), PTHR10201:SF75 (PANTHER), tmhmm (TMHMM) |
| 3424 | lib_dib_Filter15_IDBA_contig_83800_minus3 | 22 | pappalysin-2 | 134 | 8.70E-29 | 65.25% | 6 | P:biological_process; C:extracellular region; P:protein metabolic process; P:catabolic process; F:peptidase activity; F:binding | IPR006558 (SMART); IPR008985 (SUPERFAMILY); IPR013320 (G3DSA:2.60.120.GENE3D); IPR026918 (PTHR19325:PANTHER); PTHR19325 (PANTHER), PF13385 (PFAM) |
| 3425 | lib_dib_Filter15_IDBA_contig_83950_minus2 | 23 | microfibrillar-associated protein 1 | 134 | 2.82E-17 | 88.75% | 1 | C:proteinaceous extracellular matrix | IPR009730 (PANTHER); PTHR15327:SF0 (PANTHER), tmhmm (TMHMM) |
| 3426 | lib_dib_Filter15_IDBA_contig_83954_plus3 | 164 | nadp-dependent aldehyde dehydrogenase | 242 | 4.57E-114 | 79.95% | 2 | F:catalytic activity; P:metabolic process | IPR015590 (PFAM); IPR016161 (SUPERFAMILY); IPR016162 (G3DSA:3.40.605.GENE3D); PTHR11699 (PANTHER), PTHR11699:SF81 (PANTHER) |
| 3427 | lib_dib_Filter15_IDBA_contig_83961_plus2 | 14 | tyrosine-protein kinase btk29a | 134 | 2.60E-57 | 81.05% | 22 | P:multicellular organismal development; P:behavior; P:anatomical structure morphogenesis; F:protein kinase activity; P:reproduction; F:lipid binding; P:regulation of biological process; P:cytoskeleton organization; C:cytoplasm; C:extracellular region; P:embryo development; P:signal transduction; P:response to stress; P:cellular protein modification process; P:DNA metabolic process; P:biosynthetic process; P:cell cycle; P:transport; P:cellular component organization; P:organelle organization; C:plasma membrane; F:nucleotide binding | IPR000719 (PROFILE); IPR001245 (PRINTS); IPR002290 (SMART); IPR008266 (PROSITE); IPR011009 (SUPERFAMILY); IPR020635 (SMART); G3DSA:1.10.510.10 (GENE3D), G3DSA:3.30.200.20 (GENE3D), PTHR24418 (PANTHER), PTHR24418:SF65 (PANTHER) |
| 3428 | lib_dib_Filter15_IDBA_contig_84135_minus2 | 102 | lactase-phlorizin hydrolase-like | 134 | 2.11E-14 | 68.95% | 2 | F:hydrolase activity; P:carbohydrate metabolic process | IPR001360 (PANTHER); IPR013781 (G3DSA:3.20.20.GENE3D); IPR017853 (SUPERFAMILY); IPR018120 (PROSITE); tmhmm (TMHMM) |

| | | | | | | | | | |
|---|---|---|---|---|---|---|---|---|---|
| 3429 | lib_dib_Filter15_IDBA_contig_84247_plus1 | 10 | matrix metalloproteinase-9 | 134 | 5.27E-09 | 50.30% | 28 | F:metalloendopeptidase activity; F:hydrolase activity; F:zinc ion binding; F:metallopeptidase activity; C:extracellular matrix; F:peptidase activity; P:proteolysis; F:metal ion binding; P:metabolic process; P:positive regulation of apoptotic process; P:tissue remodeling; P:skeletal system development; P:collagen catabolic process; C:protein complex; P:response to drug; P:positive regulation of angiogenesis; F:fibronectin binding; P:response to oxidative stress; P:positive regulation of keratinocyte migration; P:positive regulation of synaptic plasticity; F:protein complex binding; C:extracellular space; P:extracellular matrix organization; P:transformation of host cell by virus; P:cellular response to cell-matrix adhesion; P:embryo implantation; C:proteinaceous | IPR000562 (G3DSA:2.10.10.GENE3D); IPR013806 (SUPERFAMILY); PTHR22803 (PANTHER), PTHR22803:SF10 (PANTHER) |
| 3430 | lib_dib_Filter15_IDBA_contig_84516_plus3 | 578 | collagen alpha-3 chain | 412 | 4.49E-85 | 57.00% | 2 | F:protein binding; F:calcium ion binding | IPR000152 (PROSITE); IPR000742 (PFAM); IPR001881 (SMART); IPR002035 (PFAM); IPR013032 (PROSITE); G3DSA:2.10.25.10 (GENE3D), G3DSA:3.40.50.410 (GENE3D), PTHR22992 (PANTHER), SSF53300 (SUPERFAMILY) |
| 3431 | lib_dib_Filter15_IDBA_contig_84610_plus3 | 15 | protocadherin fat 4 | 133 | 1.81E-29 | 63.15% | 3 | F:calcium ion binding; P:biological_process; C:plasma membrane | IPR002126 (PRINTS); IPR015919 (SUPERFAMILY); IPR020894 (PROSITE); PTHR24027 (PANTHER) |
| 3432 | lib_dib_Filter15_IDBA_contig_84991_plus3 | 10 | zinc finger protein 225 | 132 | 8.44E-76 | 61.75% | 11 | F:binding; C:cytoplasm; P:multicellular organismal development; F:protein binding; P:biological_process; P:signal transduction; F:sequence-specific DNA binding transcription factor activity; P:cellular protein modification process; P:regulation of biological process; ; C:nucleus | IPR007087 (PFAM); IPR013087 (G3DSA:3.30.160.GENE3D); IPR015880 (SMART); PTHR11389 (PANTHER), PF13465 (PFAM), SSF57667 (SUPERFAMILY) |
| 3433 | lib_dib_Filter15_IDBA_contig_85035_plus2 | 17 | histone h2a-like | 133 | 4.76E-63 | 98.55% | 5 | C:chromosome; F:DNA binding; P:organelle organization; C:nucleus; F:protein binding | IPR002119 (PRINTS); IPR007125 (PFAM); IPR009072 (G3DSA:1.10.20.GENE3D); PTHR23430 (PANTHER) |
| 3434 | lib_dib_Filter15_IDBA_contig_85039_minus2 | 8 | subtilisin inhibitor | 133 | 1.02E-11 | 63.25% | 3 | F:enzyme regulator activity; P:response to external stimulus; P:response to stress | IPR000864 (PRODOM); G3DSA:3.30.10.10 (GENE3D) |
| 3435 | lib_dib_Filter15_IDBA_contig_85076_minus1 | 12 | signal cub and egf-like domain-containing protein 1-like | 133 | 6.77E-08 | 59.30% | 1 | F:calcium ion binding | IPR000152 (PROSITE); IPR001881 (PFAM); IPR018097 (PROSITE); G3DSA:2.10.25.10 (GENE3D), PTHR24838 (PANTHER), PTHR24838:SF13 (PANTHER), SSF57196 (SUPERFAMILY) |
| 3436 | lib_dib_Filter15_IDBA_contig_85198_minus3 | 33 | | 215 | 0 | 100% | 1 | F:protein binding | IPR003598 (SMART); IPR003599 (SMART); IPR007110 (PROFILE); IPR013098 (PFAM); IPR013783 (G3DSA:2.60.40.GENE3D); PTHR10489 (PANTHER), SSF48726 (SUPERFAMILY) |
| 3437 | lib_dib_Filter15_IDBA_contig_852_plus3 | 898 | arylacetamide deacetylase-like | 521 | 2.75E-76 | 56.35% | 2 | F:hydrolase activity; P:metabolic process | IPR013094 (PFAM); G3DSA:3.40.50.1820 (GENE3D), PTHR23024 (PANTHER), tmhmm (TMHMM), SSF53474 (SUPERFAMILY) |
| 3438 | lib_dib_Filter15_IDBA_contig_85215_plus3 | 16 | delta and notch-like epidermal growth factor-related receptor | 132 | 1.15E-05 | 51.35% | 2 | F:protein binding; F:calcium ion binding | IPR000152 (PROSITE); IPR000742 (PROFILE); IPR001881 (PFAM); IPR018097 (PROSITE); G3DSA:2.10.25.10 (GENE3D), PTHR24838 (PANTHER), SSF57196 (SUPERFAMILY) |

| | | | | | | | | | |
|---|---|---|---|---|---|---|---|---|---|
| 3439 | lib_dib_Filter15_IDBA_contig_85317_plus1 | 11 | ---NA--- | 132 | | | 2 | F:catalytic activity; P:protein metabolic process | IPR002130 (PFAM); G3DSA:2.40.100.10 (GENE3D), PTHR11071 (PANTHER), PTHR11071:SF56 (PANTHER) |
| 3440 | lib_dib_Filter15_IDBA_contig_85338_plus3 | 27 | zinc finger protein 180-like | 217 | 1.96E-79 | 68.40% | 3 | F:binding; F:nucleic acid binding; C:intracellular | IPR007087 (PROSITE); IPR013087 (G3DSA:3.30.160.GENE3D); IPR015880 (SMART); PTHR24402 (PANTHER), PF13465 (PFAM), PF13912 (PFAM), PF13913 (PFAM), SSF57667 (SUPERFAMILY) |
| 3441 | lib_dib_Filter15_IDBA_contig_85485_plus3 | 15 | von willebrand factor type egf and pentraxin domain-containing protein 1 | 132 | 2.74E-29 | 57.10% | 1 | F:calcium ion binding | IPR000436 (PFAM); G3DSA:2.10.70.10 (GENE3D), PTHR19325 (PANTHER) |
| 3442 | lib_dib_Filter15_IDBA_contig_85565_plus1 | 22 | tankyrase-1 isoform 2 | 132 | 3.46E-81 | 95.95% | 21 | P:cellular protein modification process; P:transport; C:Golgi apparatus; F:protein binding; F:binding; C:nuclear chromosome; P:cytoskeleton organization; P:cell cycle; C:nuclear envelope; P:regulation of biological process; P:DNA metabolic process; P:organelle organization; P:biosynthetic process; C:cell; P:protein transport; P:biological_process; F:transferase activity; C:protein complex; C:chromosome; C:microtubule organizing center; | IPR002110 (PRINTS); IPR020683 (G3DSA:1.25.40.GENE3D); PTHR24180 (PANTHER), PTHR24180:SF3 (PANTHER) |
| 3443 | lib_dib_Filter15_IDBA_contig_85584_minus3 | 50 | fibrinogen c domain-containing protein 1-like | 233 | 9.09E-45 | 56.60% | 8 | F:receptor binding; C:extracellular space; P:signal transduction; C:collagen; P:cell-matrix adhesion; F:G-protein coupled receptor activity; C:membrane; P:G-protein coupled receptor signaling pathway | IPR002181 (PFAM); IPR014715 (G3DSA:4.10.530.GENE3D); IPR014716 (G3DSA:3.90.215.GENE3D); PTHR19143 (PANTHER) |
| 3444 | lib_dib_Filter15_IDBA_contig_85597_minus2 | 8 | fukutin-related protein | 132 | 5.51E-31 | 80.00% | 9 | C:Golgi apparatus; C:extracellular space; C:plasma membrane; P:biosynthetic process; P:protein metabolic process; C:protein complex; F:transferase activity; C:cell; C:endoplasmic reticulum | PTHR13627 (PANTHER), PTHR13627:SF2 (PANTHER) |
| 3445 | lib_dib_Filter15_IDBA_contig_85747_plus1 | 12 | retinol dehydrogenase 14 | 132 | 1.38E-28 | 72.40% | 4 | C:endoplasmic reticulum; F:catalytic activity; P:metabolic process; F:nucleotide binding | IPR002424 (PRINTS); IPR016040 (G3DSA:3.40.50.GENE3D); PTHR24320 (PANTHER), PTHR24320:SF0 (PANTHER), SSF51735 (SUPERFAMILY) |
| 3446 | lib_dib_Filter15_IDBA_contig_85787_minus2 | 11 | zinc finger protein 135-like | 132 | 7.61E-50 | 74.40% | 3 | F:binding; F:nucleic acid binding; C:intracellular | IPR007087 (PROSITE); IPR013087 (G3DSA:3.30.160.GENE3D); IPR015880 (SMART); PTHR24402 (PANTHER), PF13465 (PFAM), SSF57667 (SUPERFAMILY) |
| 3447 | lib_dib_Filter15_IDBA_contig_85882_minus1 | 3517 | cathepsin l | 520 | 3.95E-141 | 74.60% | 3 | P:protein metabolic process; P:catabolic process; F:peptidase activity | IPR000169 (PROSITE); IPR000668 (PRINTS); IPR013128 (PANTHER); IPR013201 (PFAM); IPR025660 (PROSITE); IPR025661 (PROSITE); G3DSA:3.90.70.10 (GENE3D), PTHR12411:SF149 (PANTHER), SignalP-NN(euk) (SIGNALP), tmhmm (TMHMM), SSF54001 (SUPERFAMILY) |
| 3448 | lib_dib_Filter15_IDBA_contig_86080_minus2 | 22 | cysteine-rich secretory protein 2-like | 229 | 2.34E-09 | 48.20% | 5 | F:ion channel inhibitor activity; C:extracellular region; F:calcium channel inhibitor activity; P:cell-cell adhesion; P:spermatogenesis | IPR001283 (PANTHER); IPR014044 (G3DSA:3.40.33.GENE3D) |
| 3449 | lib_dib_Filter15_IDBA_contig_86198_minus1 | 10 | zinc finger protein 233-like | 131 | 1.55E-21 | 57.10% | 3 | F:binding; F:nucleic acid binding; C:intracellular | IPR007087 (PFAM); IPR013087 (G3DSA:3.30.160.GENE3D); IPR015880 (SMART); PTHR24375 (PANTHER), PTHR24375:SF0 (PANTHER), PF13465 (PFAM), SSF57667 (SUPERFAMILY) |

| | | | | | | | | | |
|---|---|---|---|---|---|---|---|---|---|
| 3450 | lib_dib_Filter15_IDBA_contig_86218_minus1 | 11 | fukutin related protein | 131 | 2.43E-22 | 56.10% | 13 | P:anatomical structure morphogenesis; P:cell differentiation; P:multicellular organismal development; P:regulation of biological process; P:biosynthetic process; P:cellular protein modification process; P:carbohydrate metabolic process; C:extracellular space; P:protein metabolic process; C:plasma membrane; C:protein complex; P:cellular component organization; C:Golgi apparatus | PTHR13627 (PANTHER), PTHR13627:SF2 (PANTHER) |
| 3451 | lib_dib_Filter15_IDBA_contig_86339_minus3 | 36 | lysosomal pro-x carboxypeptidase precursor | 130 | 3.48E-27 | 63.75% | 3 | P:protein metabolic process; P:catabolic process; F:peptidase activity | IPR008758 (PANTHER); PTHR11010:SF10 (PANTHER), tmhmm (TMHMM) |
| 3452 | lib_dib_Filter15_IDBA_contig_86340_plus3 | 17233 | peptidyl-prolyl cis-trans isomerase | 428 | 8.89E-85 | 77.90% | 8 | C:extracellular space; P:protein metabolic process; C:cytoskeleton; C:protein complex; P:reproduction; C:lipid particle; P:cellular protein modification process; F:catalytic activity | IPR002130 (PRINTS); IPR020892 (PROSITE); G3DSA:2.40.100.10 (GENE3D), PTHR11071 (PANTHER), SignalP-NN(euk) (SIGNALP), tmhmm (TMHMM) |
| 3453 | lib_dib_Filter15_IDBA_contig_86341_plus1 | 14044 | peptidyl-prolyl cis-trans isomerase b-like | 387 | 4.35E-85 | 80.00% | 8 | C:extracellular space; P:protein metabolic process; C:cytoskeleton; C:protein complex; P:reproduction; C:lipid particle; P:cellular protein modification process; F:catalytic activity | IPR002130 (PRINTS); IPR020892 (PROSITE); G3DSA:2.40.100.10 (GENE3D), PTHR11071 (PANTHER) |
| 3454 | lib_dib_Filter15_IDBA_contig_86446_plus2 | 15 | oocyte zinc finger protein 6-like | 131 | 1.71E-32 | 74.30% | 3 | F:binding; F:nucleic acid binding; C:intracellular | IPR007087 (PROSITE); IPR013087 (G3DSA:3.30.160.GENE3D); IPR015880 (SMART); PTHR24382 (PANTHER), PTHR24382:SF0 (PANTHER), PF13465 (PFAM), SSF57667 (SUPERFAMILY) |
| 3455 | lib_dib_Filter15_IDBA_contig_86516_plus3 | 10 | protein | 130 | 3.78E-12 | 48.50% | 1 | C:cytoskeleton | no IPS match |
| 3456 | lib_dib_Filter15_IDBA_contig_8655_plus3 | 281 | ankyrin-3 isoform 1 | 541 | 6.27E-20 | 67.00% | 17 | P:anatomical structure morphogenesis; P:cellular component organization; P:cell differentiation; P:multicellular organismal development; P:cellular homeostasis; C:cellular_component; C:endoplasmic reticulum; P:regulation of biological process; P:cell communication; C:cell; C:cytoplasm; C:plasma membrane; F:cytoskeletal protein binding; P:cell cycle; P:protein transport; P:ion transport; F:structural molecule activity | IPR002110 (PRINTS); IPR020683 (G3DSA:1.25.40.GENE3D); PTHR24149 (PANTHER), PTHR24149:SF1 (PANTHER) |
| 3457 | lib_dib_Filter15_IDBA_contig_86581_minus3 | 43 | matrix metalloproteinase | 234 | 1.17E-58 | 66.35% | 11 | C:organelle; C:intracellular; P:multicellular organismal development; P:protein metabolic process; C:proteinaceous extracellular matrix; F:peptidase activity; F:protein binding; C:cytoplasm; P:metabolic process; P:catabolic process; F:binding | IPR001818 (PFAM); IPR006026 (SMART); IPR021190 (PRINTS); IPR024079 (G3DSA:3.40.390.GENE3D); PTHR10201 (PANTHER), SignalP-NN(euk) (SIGNALP), SSF55486 (SUPERFAMILY) |
| 3458 | lib_dib_Filter15_IDBA_contig_86742_plus1 | 10 | palmitoyltransferase zdhhc17 | 131 | 3.24E-75 | 90.00% | 13 | F:transferase activity; P:ion transport; F:binding; F:signal transducer activity; P:protein transport; C:cell; F:transporter activity; C:Golgi apparatus; C:cytoplasmic membrane-bounded vesicle; P:signal transduction; P:biosynthetic process; P:cellular protein modification process; F:protein binding | IPR002110 (PRINTS); IPR020683 (G3DSA:1.25.40.GENE3D); PTHR24161 (PANTHER), PTHR24161:SF4 (PANTHER) |

| | | | | | | | | |
|---|---|---|---|---|---|---|---|---|
| 3459 | lib_dib_Filter15_IDBA_contig_86831_minus1 | 14 | glutamyl aminopeptidase-like | 130 | 6.31E-44 | 69.05% | 5 | P:transport; F:peptidase activity; P:protein metabolic process; P:catabolic process; F:binding | IPR001930 (PANTHER); IPR014782 (PFAM); G3DSA:1.10.390.10 (GENE3D), PTHR11533:SF38 (PANTHER), SSF55486 (SUPERFAMILY) |
| 3460 | lib_dib_Filter15_IDBA_contig_86833_plus1 | 8 | kelch-like protein 26 | 130 | 2.90E-38 | 62.15% | 2 | P:biological_process; F:protein binding | IPR000210 (SMART); IPR011333 (G3DSA:3.30.710.GENE3D); IPR013069 (PFAM); PTHR24412 (PANTHER), PTHR24412:SF51 (PANTHER) |
| 3461 | lib_dib_Filter15_IDBA_contig_86836_minus3 | 20 | zinc finger protein gfi-1-like | 130 | 2.07E-40 | 93.15% | 19 | P:regulation of biological process; ; F:protein binding; F:binding; P:signal transduction; C:nucleoplasm; C:protein complex; P:cell differentiation; F:DNA binding; P:anatomical structure morphogenesis; P:embryo development; P:behavior; P:response to external stimulus; P:response to abiotic stimulus; P:biological_process; C:nucleus; P:cellular component organization; P:multicellular organismal development; P:response to stress | IPR007087 (PROSITE); IPR013087 (G3DSA:3.30.160.GENE3D); IPR015880 (SMART); PTHR23228 (PANTHER), PTHR23228:SF42 (PANTHER), PF13465 (PFAM), SSF57667 (SUPERFAMILY) |
| 3462 | lib_dib_Filter15_IDBA_contig_87043_plus3 | 10 | neurogenic locus notch | 130 | 4.78E-42 | 71.20% | 8 | C:proteinaceous extracellular matrix; P:biological_process; C:cell; C:organelle; C:cytoplasm; P:regulation of biological process; F:protein binding; F:calcium ion binding | IPR000152 (PROSITE); IPR000742 (PFAM); IPR001881 (SMART); IPR013032 (PROSITE); IPR018097 (PROSITE); PR00010 (PRINTS), G3DSA:2.10.25.10 (GENE3D), PTHR24044 (PANTHER), SSF57196 (SUPERFAMILY) |
| 3463 | lib_dib_Filter15_IDBA_contig_871_minus3 | 2036 | cysteine proteinase inhibitor precursor | 126 | 7.40E-63 | 77.90% | 4 | F:enzyme regulator activity; P:protein metabolic process; P:catabolic process; F:peptidase activity | IPR000668 (PFAM); IPR013128 (PANTHER); IPR025660 (PROSITE); IPR025661 (PROSITE); G3DSA:3.90.70.10 (GENE3D), PTHR12411:SF43 (PANTHER), SSF54001 (SUPERFAMILY) |
| 3464 | lib_dib_Filter15_IDBA_contig_87121_minus1 | 471 | trans-l-3-hydroxyproline dehydratase | 423 | 3.33E-149 | 75.60% | 1 | F:catalytic activity | IPR008794 (PFAM); G3DSA:3.10.310.10 (GENE3D), PTHR32298 (PANTHER), PTHR32298:SF0 (PANTHER), tmhmm (TMHMM), SSF54506 (SUPERFAMILY) |
| 3465 | lib_dib_Filter15_IDBA_contig_87414_plus2 | 1538 | fibrinogen-like protein 1-like | 374 | 8.02E-43 | 54.25% | 13 | P:cellular component organization; P:regulation of biological process; C:cell; P:signal transduction; F:receptor binding; P:cell death; P:multicellular organismal development; P:cellular protein modification process; P:cell proliferation; P:response to external stimulus; P:biological_process; P:anatomical structure morphogenesis; C:extracellular region | IPR002181 (PFAM); IPR014715 (G3DSA:4.10.530.GENE3D); IPR014716 (G3DSA:3.90.215.GENE3D); IPR020837 (PROSITE); PTHR19143 (PANTHER) |
| 3466 | lib_dib_Filter15_IDBA_contig_87415_plus1 | 222 | angiopoietin-like protein 4 | 130 | 5.40E-09 | 58.55% | 1 | P:regulation of biological process | IPR002181 (PFAM); IPR014715 (G3DSA:4.10.530.GENE3D); IPR020837 (PROSITE); PTHR19143 (PANTHER), PTHR19143:SF42 (PANTHER) |
| 3467 | lib_dib_Filter15_IDBA_contig_87551_plus2 | 27 | vitellogenin receptor | 129 | 1.51E-15 | 53.00% | 8 | P:biological_process; P:transport; C:cytoplasmic membrane-bounded vesicle; C:cell; P:regulation of biological process; P:growth; P:anatomical structure morphogenesis; F:protein binding | IPR002172 (G3DSA:4.10.400.GENE3D); IPR023415 (PROSITE); PR00261 (PRINTS), G3DSA:2.10.25.10 (GENE3D), PTHR10529 (PANTHER), PTHR10529:SF113 (PANTHER) |

| | | | | | | | | | | |
|---|---|---|---|---|---|---|---|---|---|---|
| 3468 | lib_dib_Filter15_IDBA_contig_87628_minus2 | 12 | agrin precursor | 129 | 1.29E-27 | 60.00% | 9 | P:metabolic process; P:signal transduction; P:regulation of biological process; P:primary metabolic process; P:cellular component organization; P:cell differentiation; P:multicellular organismal development; F:protein binding; C:proteinaceous extracellular matrix | IPR000742 (PROFILE); IPR002350 (PFAM); IPR003645 (SMART); G3DSA:3.30.60.30 (GENE3D), PTHR10913 (PANTHER), PTHR10913:SF9 (PANTHER), SSF100895 (SUPERFAMILY) | |
| 3469 | lib_dib_Filter15_IDBA_contig_87675_plus3 | 12 | protein toll | 129 | 8.40E-13 | 48.65% | 10 | F:transmembrane signaling receptor activity; C:integral to membrane; C:membrane; F:phosphoprotein phosphatase activity; F:hydrolase activity; C:intrinsic to membrane; P:signal transduction; C:intracellular; P:innate immune response; F:calcium ion binding | PTHR24369 (PANTHER), PTHR24369:SF17 (PANTHER), SSF52058 (SUPERFAMILY) | |
| 3470 | lib_dib_Filter15_IDBA_contig_87923_plus1 | 64 | neudesin precursor | 179 | 3.25E-39 | 75.95% | 1 | F:binding | IPR001199 (G3DSA:3.10.120.GENE3D); PTHR10281 (PANTHER), SignalP-NN(euk) (SIGNALP), tmhmm (TMHMM) | yes |
| 3471 | lib_dib_Filter15_IDBA_contig_87936_minus1 | 1353 | von willebrand factor type egf and pentraxin domain-containing protein 1 | 352 | 1.85E-12 | 52.40% | 2 | F:protein binding; F:calcium ion binding | IPR000742 (PFAM); IPR001881 (SMART); IPR013032 (PROSITE); G3DSA:2.10.25.10 (GENE3D), G3DSA:2.170.300.10 (GENE3D), PTHR24044 (PANTHER), PTHR24044:SF49 (PANTHER), tmhmm (TMHMM), SSF57196 (SUPERFAMILY) | |
| 3472 | lib_dib_Filter15_IDBA_contig_87937_minus1 | 170 | protein | 103 | 5.63E-08 | 66.15% | 2 | F:protein binding; F:calcium ion binding | IPR000742 (PFAM); IPR001881 (SMART); IPR013032 (PROSITE); PR00010 (PRINTS), G3DSA:2.10.25.10 (GENE3D), PTHR24044 (PANTHER), PTHR24044:SF49 (PANTHER), SignalP-NN(euk) (SIGNALP), tmhmm (TMHMM), SSF57196 (SUPERFAMILY) | |
| 3473 | lib_dib_Filter15_IDBA_contig_87948_minus1 | 25 | histone | 129 | 3.02E-58 | 99.75% | 9 | C:chromosome; P:response to stress; P:response to biotic stimulus; F:DNA binding; C:nucleoplasm; C:protein complex; P:organelle organization; C:lipid particle; F:protein binding | IPR000558 (PRINTS); IPR007125 (PFAM); IPR009072 (G3DSA:1.10.20.GENE3D) | |
| 3474 | lib_dib_Filter15_IDBA_contig_87999_minus2 | 12 | calcium calmodulin-dependent protein kinase kinase 1-like | 129 | 2.64E-61 | 86.05% | 10 | P:cellular protein modification process; F:protein binding; P:metabolic process; P:regulation of biological process; F:calcium ion binding; F:protein kinase activity; F:nucleotide binding; ; C:cytoplasm; P:signal transduction | IPR000719 (PFAM); IPR002290 (SMART); IPR008271 (PROSITE); IPR011009 (SUPERFAMILY); IPR020636 (PANTHER); G3DSA:1.10.510.10 (GENE3D), G3DSA:3.30.200.20 (GENE3D), PTHR24347:SF105 (PANTHER) | |
| 3475 | lib_dib_Filter15_IDBA_contig_880_plus1 | 3784 | lipophorin precursor | 227 | 3.69E-41 | 48.75% | 2 | F:transporter activity; P:transport | IPR009454 (PFAM); IPR015255 (PFAM); IPR015818 (G3DSA:2.20.80.GENE3D); IPR015819 (SUPERFAMILY) | |
| 3476 | lib_dib_Filter15_IDBA_contig_88017_plus2 | 8 | ankyrin repeat protein | 129 | 3.73E-28 | 64.30% | 1 | F:protein binding | IPR002110 (SMART); IPR020683 (G3DSA:1.25.40.GENE3D); PTHR24203 (PANTHER) | |
| 3477 | lib_dib_Filter15_IDBA_contig_88030_minus1 | 17 | cub and sushi domain-containing protein 3 isoform 2 | 129 | 2.87E-13 | 56.50% | 1 | F:hydrolase activity | IPR000859 (G3DSA:2.60.120.GENE3D); PTHR10127 (PANTHER), SignalP-NN(euk) (SIGNALP), tmhmm (TMHMM) | yes |

| | | | | | | | | | | |
|---|---|---|---|---|---|---|---|---|---|---|
| 3478 | lib_dib_Filter15_IDBA_contig_88153_minus1 | 37 | angiopoietin-related protein 3 | 176 | 7.86E-12 | 58.65% | 13 | P:lipid metabolic process; P:biological_process; P:catabolic process; P:carbohydrate metabolic process; P:anatomical structure morphogenesis; P:regulation of biological process; P:multicellular organismal development; C:extracellular space; F:receptor binding; P:signal transduction; F:binding; F:enzyme regulator activity; P:biosynthetic process | IPR002181 (PFAM); IPR014716 (G3DSA:3.90.215.GENE3D); PTHR19143 (PANTHER) | |
| 3479 | lib_dib_Filter15_IDBA_contig_88180_minus3 | 181 | gamma-interferon-inducible lysosomal thiol reductase | 367 | 1.77E-88 | 65.65% | 0 | - | IPR003119 (PFAM); IPR004911 (PANTHER); PTHR13234:SF7 (PANTHER), SignalP-NN(euk) (SIGNALP), tmhmm (TMHMM) | yes |
| 3480 | lib_dib_Filter15_IDBA_contig_88262_plus3 | 982 | annexin a7 | 437 | 4.38E-74 | 63.25% | 8 | P:cellular homeostasis; P:behavior; F:lipid binding; P:biological_process; P:cellular component organization; F:protein binding; C:nucleus; F:calcium ion binding | IPR001464 (PRINTS); IPR018252 (PROSITE); IPR018502 (G3DSA:1.10.220.GENE3D) | |
| 3481 | lib_dib_Filter15_IDBA_contig_88402_plus3 | 67 | thrombospondin type 1 domain-containing protein | 218 | 9.47E-24 | 60.95% | 4 | F:hydrolase activity; F:serine-type endopeptidase inhibitor activity; F:arylesterase activity; P:cell adhesion | IPR000884 (PFAM); G3DSA:2.20.100.10 (GENE3D), PTHR11311 (PANTHER), PTHR11311:SF2 (PANTHER) | |
| 3482 | lib_dib_Filter15_IDBA_contig_88417_minus1 | 29 | oocyte zinc finger protein 6-like | 252 | 3.84E-35 | 51.95% | 3 | F:binding; F:nucleic acid binding; C:intracellular | IPR007087 (PROSITE); IPR013087 (G3DSA:3.30.160.GENE3D); IPR015880 (SMART); PTHR11389 (PANTHER), PF13465 (PFAM), SignalP-NN(euk) (SIGNALP), SSF57667 (SUPERFAMILY) | yes |
| 3483 | lib_dib_Filter15_IDBA_contig_88418_minus1 | 20 | zinc finger protein 26 | 178 | 3.06E-30 | 62.85% | 3 | F:binding; F:nucleic acid binding; C:intracellular | IPR007087 (PROSITE); IPR013087 (G3DSA:3.30.160.GENE3D); IPR015880 (SMART); PTHR24402 (PANTHER), PTHR24402:SF54 (PANTHER), PF13465 (PFAM), SignalP-NN(euk) (SIGNALP), SSF57667 (SUPERFAMILY) | yes |
| 3484 | lib_dib_Filter15_IDBA_contig_88421_plus3 | 12 | achain crystal structure of engineered northeast structural genomics consortium target | 128 | 3.81E-41 | 56.50% | 1 | F:protein binding | IPR002110 (SMART); IPR020683 (G3DSA:1.25.40.GENE3D); PTHR24193 (PANTHER) | |
| 3485 | lib_dib_Filter15_IDBA_contig_88447_plus2 | 8 | zinc finger protein 572-like | 128 | 2.88E-18 | 53.40% | 3 | F:binding; F:nucleic acid binding; C:intracellular | IPR007087 (PFAM); IPR013087 (G3DSA:3.30.160.GENE3D); IPR015880 (SMART); PTHR11389 (PANTHER), PF13465 (PFAM), SSF57667 (SUPERFAMILY) | |
| 3486 | lib_dib_Filter15_IDBA_contig_88575_minus2 | 18 | paramyosin | 128 | 9.68E-23 | 69.60% | 4 | C:cytoskeleton; C:protein complex; C:cytoplasm; F:motor activity | IPR002928 (PFAM); IPR015650 (PTHR13140:PANTHER); PTHR13140 (PANTHER) | |
| 3487 | lib_dib_Filter15_IDBA_contig_88578_minus2 | 12 | low-density lipoprotein receptor-related protein 2 | 128 | 4.49E-45 | 71.70% | 7 | C:organelle; C:intracellular; F:receptor activity; F:transferase activity; C:cell; C:cytoplasm; F:protein binding | IPR002172 (G3DSA:4.10.400.GENE3D); IPR023415 (PROSITE); PR00261 (PRINTS), PTHR10529 (PANTHER), PTHR10529:SF98 (PANTHER) | |
| 3488 | lib_dib_Filter15_IDBA_contig_88628_minus3 | 38 | aminopeptidase pils (m01 family) | 135 | 7.55E-29 | 47.75% | 3 | F:peptidase activity; P:protein metabolic process; P:catabolic process | IPR001254 (PFAM); IPR009003 (SUPERFAMILY); IPR018114 (PROSITE); G3DSA:2.40.10.10 (GENE3D), PTHR24268 (PANTHER) | |
| 3489 | lib_dib_Filter15_IDBA_contig_88630_minus1 | 21 | serine protease 30-like | 107 | 6.25E-17 | 51.55% | 3 | F:peptidase activity; P:protein metabolic process; P:catabolic process | IPR001254 (PFAM); IPR009003 (SUPERFAMILY); IPR018114 (PROSITE); G3DSA:2.40.10.10 (GENE3D), PTHR24260 (PANTHER) | |
| 3490 | lib_dib_Filter15_IDBA_contig_88684_minus3 | 37 | fucl_angan ame: full=fucolectin | 224 | 3.43E-07 | 53.05% | 5 | C:extracellular space; F:binding; P:regulation of biological process; P:response to stress; F:carbohydrate binding | IPR008979 (SUPERFAMILY); G3DSA:2.60.120.260 (GENE3D), PTHR32066 (PANTHER) | |
| 3491 | lib_dib_Filter15_IDBA_contig_88685_minus3 | 41 | fucl_angan ame: full=fucolectin | 218 | 1.99E-07 | 53.05% | 5 | C:extracellular space; F:binding; P:regulation of biological process; P:response to stress; F:carbohydrate binding | IPR008979 (SUPERFAMILY); G3DSA:2.60.120.260 (GENE3D), PTHR32066 (PANTHER) | |

| | | | | | | | | | | |
|---|---|---|---|---|---|---|---|---|---|---|
| 3492 | lib_dib_Filter15_IDBA_contig_88715_plus1 | 11 | plasminogen precursor | 128 | 1.24E-28 | 57.40% | 10 | P:cellular component organization; F:peptidase activity; C:extracellular space; P:biological_process; P:response to external stimulus; P:regulation of biological process; P:response to stress; F:protein binding; F:binding; C:plasma membrane | IPR000001 (G3DSA:2.40.20.GENE3D); IPR013806 (SUPERFAMILY); IPR018056 (PROSITE); PTHR24259 (PANTHER), PTHR24259:SF2 (PANTHER) | |
| 3493 | lib_dib_Filter15_IDBA_contig_88822_minus1 | 11 | isoform e | 128 | 3.62E-27 | 61.50% | 1 | F:transferase activity | PTHR11675 (PANTHER), SignalP-NN(euk) (SIGNALP) | |
| 3494 | lib_dib_Filter15_IDBA_contig_88831_plus1 | 26 | neutral cholesterol ester hydrolase 1 isoform 3 | 128 | 2.31E-17 | 55.35% | 2 | P:metabolic process; F:hydrolase activity | IPR013094 (PFAM); G3DSA:3.40.50.1820 (GENE3D), PTHR23024 (PANTHER), SSF53474 (SUPERFAMILY) | |
| 3495 | lib_dib_Filter15_IDBA_contig_88862_minus1 | 159 | adp-ribosylation factor | 232 | 1.48E-81 | 75.30% | 4 | F:nucleotide binding; C:intracellular; P:signal transduction; P:protein transport | IPR003579 (SMART); IPR005225 (TIGRFAMs); IPR006687 (SMART); IPR006689 (PRINTS); IPR024156 (SMART); G3DSA:3.40.50.300 (GENE3D), PTHR11711 (PANTHER), SSF52540 (SUPERFAMILY) | |
| 3496 | lib_dib_Filter15_IDBA_contig_88872_plus2 | 21 | cyclin-g-associated kinase | 127 | 1.15E-62 | 86.65% | 8 | C:Golgi apparatus; C:plasma membrane; F:protein binding; P:cell cycle; P:cellular protein modification process; F:nucleotide binding; F:protein kinase activity; C:cytoplasm | IPR000719 (PFAM); IPR008271 (PROSITE); IPR011009 (SUPERFAMILY); G3DSA:1.10.510.10 (GENE3D), PTHR23172 (PANTHER), PTHR23172:SF20 (PANTHER) | |
| 3497 | lib_dib_Filter15_IDBA_contig_88905_plus3 | 9 | protocadherin gamma-b5 | 127 | 1.65E-28 | 63.15% | 3 | F:calcium ion binding; P:biological_process; C:plasma membrane | IPR002126 (PRINTS); IPR015919 (SUPERFAMILY); IPR020894 (PROSITE); PTHR24028 (PANTHER) | |
| 3498 | lib_dib_Filter15_IDBA_contig_88956_minus2 | 99 | retinol dehydrogenase 12 | 261 | 1.39E-76 | 70.20% | 3 | P:metabolic process; F:catalytic activity; F:nucleotide binding | IPR002198 (PRINTS); IPR002347 (PRINTS); IPR016040 (G3DSA:3.40.50.GENE3D); PTHR24320 (PANTHER), PTHR24320:SF0 (PANTHER), SignalP-NN(euk) (SIGNALP), tmhmm (TMHMM), SSF51735 (SUPERFAMILY) | yes |
| 3499 | lib_dib_Filter15_IDBA_contig_89004_plus1 | 13 | matrilin-like protein | 128 | 1.17E-15 | 50.75% | 2 | F:protein binding; F:calcium ion binding | IPR000152 (PROSITE); IPR000742 (PROFILE); IPR001881 (PFAM); IPR013032 (PROSITE); IPR018097 (PROSITE); G3DSA:2.10.25.10 (GENE3D), PTHR24838 (PANTHER), SSF57196 (SUPERFAMILY) | |
| 3500 | lib_dib_Filter15_IDBA_contig_89010_plus1 | 25 | zinc finger protein 787 | 195 | 3.99E-13 | 57.30% | 3 | F:binding; F:nucleic acid binding; C:intracellular | IPR007087 (PFAM); IPR013087 (G3DSA:3.30.160.GENE3D); IPR015880 (SMART); PTHR11389 (PANTHER), SSF57667 (SUPERFAMILY) | |
| 3501 | lib_dib_Filter15_IDBA_contig_891_minus1 | 1605 | a disintegrin and metalloproteinase with thrombospondin motifs partial | 288 | 1.30E-12 | 44.35% | 3 | F:peptidase activity; P:protein metabolic process; P:catabolic process | IPR001590 (PROFILE); IPR024079 (G3DSA:3.40.390.GENE3D); PTHR11905 (PANTHER), PF13574 (PFAM), SSF55486 (SUPERFAMILY) | |
| 3502 | lib_dib_Filter15_IDBA_contig_89120_minus1 | 12 | frizzled-7 | 128 | 1.37E-16 | 66.45% | 15 | P:signal transduction; P:anatomical structure morphogenesis; P:embryo development; C:plasma membrane; P:reproduction; P:multicellular organismal development; P:biological_process; F:protein binding; P:cell differentiation; P:regulation of biological process; P:cellular component organization; C:cytoplasm; P:metabolic process; F:receptor activity; C:cell | IPR015526 (PANTHER); IPR020067 (G3DSA:1.10.2000.GENE3D); IPR026550 (PTHR11309:PANTHER) | |
| 3503 | lib_dib_Filter15_IDBA_contig_89145_minus2 | 853 | deleted in malignant brain tumors 1 | 131 | 3.87E-20 | 66.30% | 3 | C:plasma membrane; F:protein binding; F:receptor activity | IPR001190 (PRINTS); IPR017448 (SMART); G3DSA:3.10.250.10 (GENE3D), PTHR19331 (PANTHER), PTHR19331:SF118 (PANTHER), SignalP-NN(euk) (SIGNALP) | |

| | | | | | | | | | | |
|---|---|---|---|---|---|---|---|---|---|---|
| 3504 | lib_dib_Filter15_IDBA_contig_89146_minus2 | 2243 | deleted in malignant brain tumors 1 | 233 | 6.97E-31 | 64.65% | 10 | P:regulation of biological process; P:protein transport; F:protein binding; P:transport; P:cell differentiation; C:cytoplasm; C:extracellular region; P:biological_process; C:plasma membrane; F:receptor activity | IPR001190 (PRINTS); IPR017448 (SMART); G3DSA:3.10.250.10 (GENE3D), PTHR19331 (PANTHER), PTHR19331:SF118 (PANTHER), SignalP-NN(euk) (SIGNALP) | |
| 3505 | lib_dib_Filter15_IDBA_contig_89163_plus2 | 17 | kielin chordin-like | 127 | 6.06E-14 | 53.60% | 11 | F:metal ion binding; P:oxidation-reduction process; F:oxidoreductase activity; P:metabolic process; F:peptidase inhibitor activity; F:catalytic activity; P:negative regulation of peptidase activity; C:extracellular region; F:chitin binding; P:chitin metabolic process; P:cell adhesion | IPR001846 (PFAM); PTHR11339 (PANTHER) | |
| 3506 | lib_dib_Filter15_IDBA_contig_89187_minus3 | 14 | endothelin-converting enzyme isoform cra_a | 127 | 3.89E-19 | 82.20% | 9 | F:binding; C:Golgi apparatus; P:protein metabolic process; C:cytoplasmic membrane-bounded vesicle; F:peptidase activity; P:metabolic process; C:cell; P:catabolic process; F:transferase activity | IPR000718 (PANTHER); IPR018497 (PFAM); IPR024079 (G3DSA:3.40.390.GENE3D); PTHR11733:SF31 (PANTHER), SSF55486 (SUPERFAMILY) | |
| 3507 | lib_dib_Filter15_IDBA_contig_89194_minus2 | 16 | uncharacterized aarf domain-containing protein kinase 1 | 127 | 1.27E-15 | 57.70% | 1 | F:transferase activity | PTHR10566 (PANTHER), PTHR10566:SF24 (PANTHER) | |
| 3508 | lib_dib_Filter15_IDBA_contig_89217_minus1 | 17 | matrix metalloproteinase-28 | 127 | 4.88E-13 | 50.75% | 11 | F:metalloendopeptidase activity; F:hydrolase activity; F:zinc ion binding; F:metallopeptidase activity; C:extracellular matrix; F:peptidase activity; P:proteolysis; F:metal ion binding; P:metabolic process; F:calcium ion binding; C:collagen | IPR000585 (G3DSA:2.110.10.GENE3D); IPR018486 (PROSITE); IPR018487 (PFAM); PTHR10201 (PANTHER), PTHR10201:SF16 (PANTHER) | |
| 3509 | lib_dib_Filter15_IDBA_contig_89268_minus1 | 24 | low-density lipoprotein receptor-related protein 2 | 127 | 4.40E-11 | 52.55% | 2 | C:cell; F:protein binding | IPR002172 (G3DSA:4.10.400.GENE3D); PR00261 (PRINTS), PTHR10529 (PANTHER), PTHR10529:SF113 (PANTHER) | |
| 3510 | lib_dib_Filter15_IDBA_contig_89381_minus2 | 15 | protocadherin fat 3 | 127 | 3.25E-25 | 63.15% | 3 | F:calcium ion binding; P:biological_process; C:cell | IPR002126 (PRINTS); IPR015919 (SUPERFAMILY); PTHR24027 (PANTHER), PTHR24027:SF110 (PANTHER) | |
| 3511 | lib_dib_Filter15_IDBA_contig_89533_minus3 | 12 | glyceraldehyde-3-phosphate dehydrogenase | 126 | 9.85E-79 | 93.90% | 19 | P:cell death; C:cytosol; F:nucleotide binding; F:cytoskeletal protein binding; P:metabolic process; C:cytoskeleton; C:cell; C:mitochondrion; P:regulation of biological process; P:cytoskeleton organization; F:catalytic activity; P:carbohydrate metabolic process; P:generation of precursor metabolites and energy; P:catabolic process; P:biosynthetic process; P:multicellular organismal development; C:lipid particle; P:biological_process; C:nucleus | IPR016040 (G3DSA:3.40.50.GENE3D); IPR020828 (PFAM); IPR020829 (PFAM); IPR020830 (PROSITE); IPR020831 (PRINTS); G3DSA:3.30.360.10 (GENE3D), SSF51735 (SUPERFAMILY), SSF55347 (SUPERFAMILY) | |
| 3512 | lib_dib_Filter15_IDBA_contig_89634_minus1 | 10 | low quality protein: sco-spondin | 127 | 8.97E-15 | 51.80% | 1 | F:protein binding | IPR002172 (G3DSA:4.10.400.GENE3D); IPR023415 (PROSITE); PR00261 (PRINTS), PTHR24652 (PANTHER), PTHR24652:SF0 (PANTHER) | |
| 3513 | lib_dib_Filter15_IDBA_contig_89667_minus2 | 98 | 3-oxoacyl-acyl-carrier-protein reductase | 366 | 1.99E-101 | 70.60% | 3 | P:metabolic process; F:catalytic activity; F:nucleotide binding | IPR002198 (PRINTS); IPR002347 (PRINTS); IPR016040 (G3DSA:3.40.50.GENE3D); PTHR24322 (PANTHER), PTHR24322:SF0 (PANTHER), tmhmm (TMHMM), SSF51735 (SUPERFAMILY) | |
| 3514 | lib_dib_Filter15_IDBA_contig_89702_minus2 | 17 | fibrillin-2 | 126 | 8.26E-17 | 56.25% | 3 | P:regulation of biological process; P:multicellular organismal development; P:anatomical structure morphogenesis | IPR013032 (PROSITE); PTHR24039 (PANTHER), SignalP-NN(euk) (SIGNALP), tmhmm (TMHMM) | yes |

| | | | | | | | | | | |
|---|---|---|---|---|---|---|---|---|---|---|
| 3515 | lib_dib_Filter15_IDBA_contig_89727_plus1 | 14 | protein wnt-7a | 127 | 4.68E-44 | 67.20% | 20 | P:signal transduction; C:cell; P:cell differentiation; P:multicellular organismal development; P:regulation of biological process; F:receptor binding; P:biological_process; P:reproduction; P:anatomical structure morphogenesis; P:growth; P:embryo development; P:cellular component organization; P:cell-cell signaling; P:response to stress; ; F:signal transducer activity; P:cell proliferation; P:response to external stimulus; C:extracellular region; P:cell death | IPR005817 (PRINTS); IPR018161 (PROSITE) | |
| 3516 | lib_dib_Filter15_IDBA_contig_89779_minus3 | 31 | isoform a | 208 | 2.17E-07 | 81.55% | 0 | - | SignalP-NN(euk) (SIGNALP), tmhmm (TMHMM) | |
| 3517 | lib_dib_Filter15_IDBA_contig_8995_plus1 | 27 | matrix metalloproteinase-17-like | 130 | 4.47E-29 | 58.90% | 1 | F:hydrolase activity | IPR000585 (G3DSA:2.110.10.GENE3D); IPR018487 (PFAM); PTHR10201 (PANTHER) | |
| 3518 | lib_dib_Filter15_IDBA_contig_9_plus1 | 21244 | supervillin | 5207 | 0 | 61.40% | 7 | C:cytoplasm; P:multicellular organismal development; C:plasma membrane; F:actin binding; C:nucleus; C:cytoskeleton; P:cytoskeleton organization | IPR003128 (G3DSA:1.10.950.GENE3D); IPR007122 (PANTHER); IPR007123 (PFAM); IPR015628 (PTHR11977:PANTHER); G3DSA:3.40.20.10 (GENE3D), tmhmm (TMHMM), SSF55753 (SUPERFAMILY) | |
| 3519 | lib_dib_Filter15_IDBA_contig_90363_minus2 | 10 | low-density lipoprotein receptor-related protein 2 | 126 | 7.40E-48 | 68.30% | 14 | P:regulation of biological process; P:growth; F:transporter activity; F:receptor activity; P:biological_process; C:cytoplasmic membrane-bounded vesicle; P:transport; P:anatomical structure morphogenesis; P:multicellular organismal development; F:protein binding; C:plasma membrane; P:cellular component organization; C:Golgi apparatus; P:protein transport | IPR002172 (G3DSA:4.10.400.GENE3D); IPR023415 (PROSITE); PR00261 (PRINTS), G3DSA:2.10.25.10 (GENE3D), PTHR10529 (PANTHER), PTHR10529:SF113 (PANTHER) | |
| 3520 | lib_dib_Filter15_IDBA_contig_90532_plus3 | 21 | serine carboxypeptidase-like 51 | 167 | 4.38E-25 | 57.40% | 6 | P:lipid metabolic process; P:secondary metabolic process; C:cytosol; F:peptidase activity; P:protein metabolic process; P:catabolic process | IPR001563 (PANTHER); G3DSA:3.40.50.1820 (GENE3D), PTHR11802:SF3 (PANTHER), SSF53474 (SUPERFAMILY) | |
| 3521 | lib_dib_Filter15_IDBA_contig_90533_plus1 | 16 | serine carboxypeptidase-like 51 | 126 | 1.10E-12 | 62.20% | 6 | C:cytosol; F:peptidase activity; P:lipid metabolic process; P:secondary metabolic process; P:protein metabolic process; P:catabolic process | IPR001563 (PANTHER); G3DSA:3.40.50.1820 (GENE3D), PTHR11802:SF3 (PANTHER), SSF53474 (SUPERFAMILY) | |
| 3522 | lib_dib_Filter15_IDBA_contig_90601_minus3 | 12 | gastrula zinc finger protein - partial | 125 | 2.17E-18 | 63.10% | 3 | F:binding; F:nucleic acid binding; C:intracellular | IPR007087 (PROSITE); IPR013087 (G3DSA:3.30.160.GENE3D); IPR015880 (SMART); PTHR24382 (PANTHER), PTHR24382:SF0 (PANTHER), PF13465 (PFAM), PF13894 (PFAM), SSF57667 (SUPERFAMILY) | |
| 3523 | lib_dib_Filter15_IDBA_contig_90652_minus1 | 7 | angiopoietin-related protein 1 | 126 | 4.51E-33 | 66.20% | 1 | C:extracellular space | IPR002181 (PFAM); IPR014715 (G3DSA:4.10.530.GENE3D); IPR014716 (G3DSA:3.90.215.GENE3D); PTHR19143 (PANTHER) | |
| 3524 | lib_dib_Filter15_IDBA_contig_90746_plus1 | 832 | metalloproteinase inhibitor 3 | 506 | 7.68E-19 | 46.50% | 2 | F:protein binding; F:enzyme regulator activity | IPR001134 (PROFILE); IPR001820 (PANTHER); IPR008993 (SUPERFAMILY); G3DSA:2.40.50.120 (GENE3D), G3DSA:3.90.370.10 (GENE3D), PTHR11844:SF9 (PANTHER) | |
| 3525 | lib_dib_Filter15_IDBA_contig_90830_minus2 | 32 | mitochondrial import inner membrane translocase subunit tim8 a | 125 | 9.01E-31 | 78.90% | 7 | F:binding; C:cell; C:mitochondrion; P:mitochondrion organization; P:protein transport; C:protein complex; F:protein binding | IPR004217 (G3DSA:1.10.287.GENE3D); PTHR21535 (PANTHER) | yes |

| | | | | | | | | | | |
|---|---|---|---|---|---|---|---|---|---|---|
| 3526 | lib_dib_Filter15_IDBA_contig_90852_minus2 | 16 | a chain design and synthesis of thiophene dihydroisoquinolins as novel bace-1 inhibitors | 125 | 1.85E-41 | 76.60% | 8 | P:protein metabolic process; P:catabolic process; F:peptidase activity; C:endosome; F:protein binding; P:metabolic process; C:Golgi apparatus; C:plasma membrane | IPR001461 (PANTHER); IPR001969 (PROSITE); IPR009119 (PRINTS); IPR021109 (G3DSA:2.40.70.GENE3D); PTHR13683:SF87 (PANTHER) | |
| 3527 | lib_dib_Filter15_IDBA_contig_90896_minus2 | 11 | zinc finger protein 596-like | 125 | 9.28E-22 | 57.30% | 3 | F:binding; F:nucleic acid binding; C:intracellular | IPR007087 (PROSITE); IPR013087 (G3DSA:3.30.160.GENE3D); IPR015880 (SMART); PTHR11389 (PANTHER), PF13465 (PFAM), PF13894 (PFAM), SSF57667 (SUPERFAMILY) | |
| 3528 | lib_dib_Filter15_IDBA_contig_91007_minus1 | 18 | polypeptide n-acetylgalactosaminyltransferase 9-like | 125 | 8.58E-31 | 58.55% | 7 | F:carbohydrate binding; F:transferase activity; C:integral to membrane; C:membrane; C:Golgi apparatus; F:transferase activity, transferring glycosyl groups; P:carbohydrate metabolic process | IPR000772 (PFAM); G3DSA:2.80.10.50 (GENE3D), PTHR11675 (PANTHER) | |
| 3529 | lib_dib_Filter15_IDBA_contig_91042_plus3 | 15 | leucine-rich repeat-containing protein 38 | 124 | 2.05E-15 | 56.70% | 3 | F:calcium ion binding; P:negative regulation of axon extension; C:extracellular region | PR00019 (PRINTS), G3DSA:3.80.10.10 (GENE3D), PTHR24367 (PANTHER), PF13855 (PFAM), SignalP-NN(euk) (SIGNALP), SSF52058 (SUPERFAMILY) | yes |
| 3530 | lib_dib_Filter15_IDBA_contig_91089_minus2 | 35 | crumbs homolog 1 | 208 | 2.82E-40 | 59.00% | 3 | P:biological_process; F:protein binding; F:calcium ion binding | IPR000152 (PROSITE); IPR000742 (PFAM); IPR001881 (SMART); IPR013032 (PROSITE); IPR018097 (PROSITE); PR00010 (PRINTS), G3DSA:2.10.25.10 (GENE3D), PTHR24044 (PANTHER), SSF57196 (SUPERFAMILY) | |
| 3531 | lib_dib_Filter15_IDBA_contig_91117_plus1 | 9 | transcription factor e4f1 | 125 | 3.38E-38 | 72.50% | 13 | P:regulation of biological process; ; F:protein binding; F:transcription regulator activity; P:cell proliferation; P:cell cycle; C:cytoskeleton; F:sequence-specific DNA binding transcription factor activity; P:embryo development; C:nucleus; P:DNA metabolic process; P:biosynthetic process; F:binding | IPR007087 (PROSITE); IPR013087 (G3DSA:3.30.160.GENE3D); IPR015880 (SMART); PTHR24387 (PANTHER), PTHR24387:SF3 (PANTHER), PF13465 (PFAM), SSF57667 (SUPERFAMILY) | |
| 3532 | lib_dib_Filter15_IDBA_contig_91173_minus3 | 17 | protocadherin fat 3 | 124 | 3.73E-40 | 69.05% | 4 | F:catalytic activity; F:calcium ion binding; P:biological_process; C:plasma membrane | IPR002126 (PRINTS); IPR015919 (SUPERFAMILY); IPR020894 (PROSITE); PTHR24027 (PANTHER), PTHR24027:SF110 (PANTHER), SignalP-NN(euk) (SIGNALP) | |
| 3533 | lib_dib_Filter15_IDBA_contig_91351_minus2 | 9 | transmembrane protease serine 9-like | 124 | 1.47E-29 | 61.20% | 3 | F:peptidase activity; P:protein metabolic process; P:catabolic process | IPR001254 (PFAM); IPR001314 (PRINTS); IPR009003 (SUPERFAMILY); IPR018114 (PROSITE); G3DSA:2.40.10.10 (GENE3D), PTHR24256 (PANTHER) | |
| 3534 | lib_dib_Filter15_IDBA_contig_91426_minus1 | 9 | fucl3_angja ame: full=fucolectin-3 flags: precursor | 125 | 1.73E-14 | 51.75% | 5 | C:extracellular space; P:regulation of biological process; P:response to stress; F:carbohydrate binding; P:biological_process | IPR000421 (PFAM); IPR006585 (SMART); IPR008979 (SUPERFAMILY); G3DSA:2.60.120.260 (GENE3D), PTHR32066 (PANTHER) | |
| 3535 | lib_dib_Filter15_IDBA_contig_91505_minus2 | 11 | fibrinogen c domain-containing protein 1-like | 124 | 8.31E-28 | 62.35% | 3 | F:receptor binding; C:extracellular space; P:signal transduction | IPR002181 (PFAM); IPR014715 (G3DSA:4.10.530.GENE3D); IPR014716 (G3DSA:3.90.215.GENE3D); IPR020837 (PROSITE); PTHR19143 (PANTHER) | |
| 3536 | lib_dib_Filter15_IDBA_contig_91574_plus3 | 6 | ring finger and spry domain-containing protein 1 | 124 | 1.07E-37 | 85.75% | 2 | C:extracellular region; F:binding | PTHR13363 (PANTHER), PTHR13363:SF0 (PANTHER) | |
| 3537 | lib_dib_Filter15_IDBA_contig_91724_plus2 | 40 | microfibril-associated glycoprotein 4 | 178 | 4.17E-10 | 64.65% | 1 | F:protein binding | IPR000742 (PFAM); IPR002181 (PFAM); IPR013032 (PROSITE); IPR014716 (G3DSA:3.90.215.GENE3D); G3DSA:2.10.25.10 (GENE3D), PTHR19143 (PANTHER), PTHR19143:SF46 (PANTHER), SSF57196 (SUPERFAMILY) | |
| 3538 | lib_dib_Filter15_IDBA_contig_91888_minus3 | 49 | enterin partial | 280 | 8.68E-21 | 63.25% | 0 | - | no IPS match | |
| 3539 | lib_dib_Filter15_IDBA_contig_91941_plus2 | 2636 | chymotrypsin inhibitor-2 | 328 | 2.97E-17 | 62.90% | 3 | F:enzyme regulator activity; P:response to external stimulus; P:response to stress | IPR000864 (PRODOM); G3DSA:3.30.10.10 (GENE3D) | |

| | | | | | | | | | |
|---|---|---|---|---|---|---|---|---|---|
| 3540 | lib_dib_Filter15_IDBA_contig_92047_minus1 | 9 | nardilysin isoform 2 | 124 | 2.84E-34 | 70.85% | 8 | C:mitochondrion; F:binding; P:regulation of biological process; P:protein metabolic process; P:catabolic process; F:peptidase activity; P:biological_process; F:protein binding | IPR007863 (PFAM); IPR011237 (G3DSA:3.30.830.GENE3D); IPR011249 (SUPERFAMILY); PTHR11851 (PANTHER), PTHR11851:SF65 (PANTHER) |
| 3541 | lib_dib_Filter15_IDBA_contig_92133_minus1 | 10 | dnaj homolog subfamily c member 7-like | 124 | 1.04E-22 | 60.20% | 4 | C:cytoplasm; C:nucleolus; F:protein binding; P:protein metabolic process | IPR001623 (PRINTS); IPR011990 (G3DSA:1.25.40.GENE3D); PTHR24078 (PANTHER), PTHR24078:SF0 (PANTHER), PF13414 (PFAM), SSF48452 (SUPERFAMILY) |
| 3542 | lib_dib_Filter15_IDBA_contig_92238_minus3 | 9 | zinc finger | 123 | 9.80E-27 | 73.75% | 3 | F:binding; F:nucleic acid binding; C:intracellular | IPR007087 (PROSITE); IPR013087 (G3DSA:3.30.160.GENE3D); IPR015880 (SMART); PTHR11389 (PANTHER), PF13465 (PFAM), SSF57667 (SUPERFAMILY) |
| 3543 | lib_dib_Filter15_IDBA_contig_92246_minus1 | 8 | cdc2 kinase | 124 | 5.04E-78 | 93.25% | 7 | F:protein kinase activity; P:organelle organization; P:cell cycle; P:cellular protein modification process; F:nucleotide binding; C:nucleus; P:biological_process | IPR000719 (PFAM); IPR002290 (SMART); IPR008271 (PROSITE); IPR011009 (SUPERFAMILY); G3DSA:1.10.510.10 (GENE3D), PTHR24056 (PANTHER) |
| 3544 | lib_dib_Filter15_IDBA_contig_92281_plus1 | 15 | a chain targeted polypharmacology: crystal structure of the c-src kinase domain in complex with a multitargeted kinase inhibitor | 124 | 2.64E-60 | 83.35% | 27 | C:cytoskeleton; P:cellular component organization; P:signal transduction; P:reproduction; P:biological_process; C:plasma membrane; C:lysosome; P:anatomical structure morphogenesis; P:cell differentiation; P:multicellular organismal development; P:cellular protein modification process; P:regulation of biological process; C:cytosol; F:receptor binding; F:protein binding; F:binding; P:response to external stimulus; P:response to stress; P:protein metabolic process; C:endosome; F:nucleotide binding; F:signal transducer activity; P:cell cycle; P:response to endogenous stimulus; C:mitochondrion; F:protein kinase activity; C:nucleus | IPR000719 (PROFILE); IPR001245 (PRINTS); IPR011009 (SUPERFAMILY); IPR020635 (SMART); G3DSA:1.10.510.10 (GENE3D), PTHR24418 (PANTHER), PTHR24418:SF35 (PANTHER) |
| 3545 | lib_dib_Filter15_IDBA_contig_92305_minus1 | 9 | ---NA--- | 127 | | | 0 | - | no IPS match |
| 3546 | lib_dib_Filter15_IDBA_contig_92305_minus2 | 9 | ---NA--- | 127 | | | 0 | - | no IPS match |
| 3547 | lib_dib_Filter15_IDBA_contig_92427_minus3 | 13 | ---NA--- | 123 | | | 0 | - | IPR008160 (PFAM); PTHR24023 (PANTHER) |
| 3548 | lib_dib_Filter15_IDBA_contig_92427_plus1 | 13 | ---NA--- | 124 | | | 0 | - | no IPS match |
| 3549 | lib_dib_Filter15_IDBA_contig_92462_minus3 | 12 | arylsulfatase i | 123 | 8.34E-36 | 67.05% | 2 | F:catalytic activity; P:metabolic process | IPR017849 (G3DSA:3.40.720.GENE3D); IPR017850 (SUPERFAMILY); G3DSA:3.30.1120.10 (GENE3D), PTHR10342 (PANTHER), PTHR10342:SF19 (PANTHER) |
| 3550 | lib_dib_Filter15_IDBA_contig_92681_minus2 | 12 | carboxypeptidase b-like | 136 | 5.75E-24 | 54.80% | 4 | F:peptidase activity; P:protein metabolic process; P:catabolic process; F:binding | IPR000834 (PFAM); G3DSA:3.40.630.10 (GENE3D), PTHR11705 (PANTHER), SignalP-NN(euk) (SIGNALP), SSF53187 (SUPERFAMILY) |
| 3551 | lib_dib_Filter15_IDBA_contig_92712_plus1 | 11 | neutral ceramidase | 123 | 2.22E-21 | 59.95% | 6 | C:endoplasmic reticulum membrane; C:apical plasma membrane; P:ceramide metabolic process; C:Golgi membrane; F:hydrolase activity; F:ceramidase activity | IPR006823 (PANTHER) |
| 3552 | lib_dib_Filter15_IDBA_contig_92739_plus3 | 20 | oocyte zinc finger protein 6-like | 129 | 3.69E-42 | 64.90% | 3 | F:binding; F:nucleic acid binding; C:intracellular | IPR007087 (PROSITE); IPR013087 (G3DSA:3.30.160.GENE3D); IPR015880 (SMART); PTHR24402 (PANTHER), PF13465 (PFAM), SSF57667 (SUPERFAMILY) |

| | | | | | | | | | |
|---|---|---|---|---|---|---|---|---|---|
| 3553 | lib_dib_Filter15_IDBA_contig_92925_plus2 | 8 | neurogenic locus protein delta | 123 | 2.18E-08 | 69.58% | 2 | F:protein binding; F:calcium ion binding | IPR000152 (PROSITE); IPR000742 (PFAM); IPR000859 (G3DSA:2.60.120.GENE3D); IPR001881 (SMART); IPR013032 (PROSITE); G3DSA:2.10.25.10 (GENE3D), PTHR22991 (PANTHER), PTHR22991:SF0 (PANTHER), SSF57196 (SUPERFAMILY) |
| 3554 | lib_dib_Filter15_IDBA_contig_92979_minus2 | 277 | dehydrogenase reductase sdr family member 11 | 399 | 2.63E-94 | 73.35% | 3 | P:metabolic process; F:catalytic activity; F:nucleotide binding | IPR002198 (PRINTS); IPR002347 (PRINTS); IPR016040 (G3DSA:3.40.50.GENE3D); IPR020904 (PROSITE); PTHR24322 (PANTHER), PTHR24322:SF7 (PANTHER), SignalP-NN(euk) (SIGNALP), SSF51735 (SUPERFAMILY) |
| 3555 | lib_dib_Filter15_IDBA_contig_92980_minus2 | 292 | dehydrogenase reductase sdr family member 11 | 441 | 1.64E-93 | 73.30% | 3 | P:metabolic process; F:catalytic activity; F:nucleotide binding | IPR002198 (PRINTS); IPR002347 (PRINTS); IPR016040 (G3DSA:3.40.50.GENE3D); IPR020904 (PROSITE); PTHR24322 (PANTHER), PTHR24322:SF7 (PANTHER), SignalP-NN(euk) (SIGNALP), SSF51735 (SUPERFAMILY) |
| 3556 | lib_dib_Filter15_IDBA_contig_93005_minus2 | 22 | low quality protein: pappalysin-1 | 123 | 2.26E-31 | 66.00% | 5 | C:extracellular region; F:peptidase activity; P:protein metabolic process; P:catabolic process; F:binding | IPR026918 (PTHR19325:PANTHER); PTHR19325 (PANTHER) |
| 3557 | lib_dib_Filter15_IDBA_contig_9304_minus1 | 1681 | pregnancy zone protein | 201 | 7.01E-43 | 62.80% | 1 | C:extracellular region | IPR009048 (G3DSA:2.60.40.GENE3D); PTHR11412 (PANTHER), PTHR11412:SF36 (PANTHER) |
| 3558 | lib_dib_Filter15_IDBA_contig_93049_minus3 | 23 | ficolin 3 | 122 | 2.62E-32 | 65.25% | 5 | F:receptor binding; C:extracellular space; P:signal transduction; F:scavenger receptor activity; C:membrane | IPR002181 (PFAM); IPR014715 (G3DSA:4.10.530.GENE3D); IPR014716 (G3DSA:3.90.215.GENE3D); PTHR19143 (PANTHER) |
| 3559 | lib_dib_Filter15_IDBA_contig_93050_plus3 | 11 | ---NA--- | 122 | | | 0 | - | no IPS match |
| 3560 | lib_dib_Filter15_IDBA_contig_93051_minus1 | 9 | carboxylesterase type b | 123 | 5.57E-24 | 59.30% | 1 | F:hydrolase activity | IPR002018 (PFAM); IPR019819 (PROSITE); G3DSA:3.40.50.1820 (GENE3D), PTHR11559 (PANTHER), SSF53474 (SUPERFAMILY) |
| 3561 | lib_dib_Filter15_IDBA_contig_931_minus3 | 161 | cell adhesion molecule | 231 | 6.45E-45 | 50.00% | 1 | F:protein binding | IPR003598 (SMART); IPR007110 (PROFILE); IPR013783 (G3DSA:2.60.40.GENE3D); PTHR10489 (PANTHER), PF13895 (PFAM), SSF48726 (SUPERFAMILY) |
| 3562 | lib_dib_Filter15_IDBA_contig_93253_plus3 | 8 | para-nitrobenzyl esterase-like | 122 | 1.83E-25 | 60.50% | 1 | F:hydrolase activity | IPR002018 (PFAM); IPR019826 (PROSITE); G3DSA:3.40.50.1820 (GENE3D), PTHR11559 (PANTHER), SSF53474 (SUPERFAMILY) |
| 3563 | lib_dib_Filter15_IDBA_contig_93370_minus2 | 8 | gamma-aminobutyric acid type b receptor subunit 1-like | 122 | 9.68E-53 | 72.40% | 4 | P:behavior; P:signal transduction; C:cell; F:receptor activity | G3DSA:3.40.50.2300 (GENE3D), PTHR10519 (PANTHER), PTHR10519:SF3 (PANTHER) |
| 3564 | lib_dib_Filter15_IDBA_contig_93377_plus3 | 7 | tyrosine-protein kinase csk-like | 122 | 1.59E-66 | 85.75% | 12 | P:regulation of biological process; P:growth; P:cellular protein modification process; P:anatomical structure morphogenesis; P:cellular component organization; P:reproduction; P:cell death; P:cell proliferation; P:biological_process; F:nucleotide binding; P:signal transduction; F:protein kinase activity | IPR000719 (PROFILE); IPR001245 (PRINTS); IPR002290 (SMART); IPR008266 (PROSITE); IPR011009 (SUPERFAMILY); IPR020635 (SMART); G3DSA:1.10.510.10 (GENE3D), PTHR24418 (PANTHER) |
| 3565 | lib_dib_Filter15_IDBA_contig_93388_minus2 | 14 | von willebrand factor type egf and pentraxin domain-containing protein 1 | 122 | 7.25E-15 | 53.55% | 2 | F:carbohydrate binding; F:calcium ion binding | IPR000436 (PFAM); G3DSA:2.10.70.10 (GENE3D), PTHR19325 (PANTHER) |
| 3566 | lib_dib_Filter15_IDBA_contig_93400_plus1 | 9 | thrombospondin type-1 domain-containing protein 7a | 122 | 1.04E-12 | 46.80% | 0 | - | IPR000884 (PFAM); G3DSA:2.20.100.10 (GENE3D), PTHR11311 (PANTHER), PTHR11311:SF2 (PANTHER) |
| 3567 | lib_dib_Filter15_IDBA_contig_93418_plus2 | 26 | pregnancy-associated plasma protein-e | 170 | 1.16E-34 | 51.00% | 2 | P:metabolic process; F:peptidase activity | PTHR19325 (PANTHER), PTHR19325:SF35 (PANTHER) |

| | | | | | | | | | | |
|---|---|---|---|---|---|---|---|---|---|---|
| 3568 | lib_dib_Filter15_IDBA_contig_93440_minus3 | 240 | isoform a | 292 | 1.19E-22 | 46.80% | 3 | P:biological_process; P:cell differentiation; F:protein binding | IPR003598 (SMART); IPR003599 (SMART); IPR007110 (PROFILE); IPR013098 (PFAM); IPR013783 (G3DSA:2.60.40.GENE3D); PTHR10075 (PANTHER), PTHR10075:SF3 (PANTHER), SignalP-NN(euk) (SIGNALP), tmhmm (TMHMM), SSF48726 (SUPERFAMILY) | yes |
| 3569 | lib_dib_Filter15_IDBA_contig_93491_plus3 | 18 | zinc finger protein 271-like | 122 | 2.62E-48 | 78.00% | 3 | F:binding; F:nucleic acid binding; C:intracellular | IPR007087 (PROSITE); IPR013087 (G3DSA:3.30.160.GENE3D); IPR015880 (SMART); PTHR24387 (PANTHER), PTHR24387:SF0 (PANTHER), PF13465 (PFAM), SSF57667 (SUPERFAMILY) | |
| 3570 | lib_dib_Filter15_IDBA_contig_93629_plus1 | 6 | proto-oncogene tyrosine-protein kinase ros | 122 | 2.90E-16 | 62.25% | 8 | P:cellular protein modification process; P:cell growth; C:cell; F:protein kinase activity; F:protein binding; P:cell differentiation; P:signal transduction; F:nucleotide binding | IPR000719 (PROFILE); IPR001245 (PFAM); IPR011009 (SUPERFAMILY); G3DSA:1.10.510.10 (GENE3D), PTHR24418 (PANTHER), SignalP-NN(euk) (SIGNALP) | yes |
| 3571 | lib_dib_Filter15_IDBA_contig_93649_plus1 | 42 | ankyrin repeat | 264 | 5.92E-21 | 46.30% | 1 | F:protein binding | IPR002110 (SMART); IPR020683 (G3DSA:1.25.40.GENE3D); PTHR24193 (PANTHER) | |
| 3572 | lib_dib_Filter15_IDBA_contig_93664_plus2 | 10 | isoform a | 122 | 2.74E-19 | 54.85% | 26 | P:regulation of biological process; P:growth; ; P:response to stress; P:anatomical structure morphogenesis; P:cell differentiation; P:multicellular organismal development; P:biological_process; P:cell proliferation; P:signal transduction; P:cytoskeleton organization; C:protein complex; P:reproduction; C:plasma membrane; P:cellular component organization; P:behavior; P:cell-cell signaling; P:embryo development; P:cell communication; C:organelle; C:intracellular; P:cell cycle; P:metabolic process; F:protein binding; F:DNA binding; F:calcium ion binding | IPR000152 (PROSITE); IPR000742 (PFAM); IPR001881 (SMART); IPR013032 (PROSITE); IPR018097 (PROSITE); PR00010 (PRINTS), G3DSA:2.10.25.10 (GENE3D), PTHR24033 (PANTHER), SignalP-NN(euk) (SIGNALP), SSF57196 (SUPERFAMILY) | yes |
| 3573 | lib_dib_Filter15_IDBA_contig_93693_plus3 | 10 | gamma-glutamyl hydrolase | 121 | 1.16E-06 | 62.75% | 2 | ; F:peptidase activity | IPR015527 (PANTHER); PTHR11315:SF0 (PANTHER) | |
| 3574 | lib_dib_Filter15_IDBA_contig_93723_plus1 | 33 | oxidoreductase partial | 214 | 5.89E-40 | 63.45% | 3 | P:metabolic process; F:catalytic activity; F:nucleotide binding | IPR002198 (PRINTS); IPR002347 (PRINTS); IPR016040 (G3DSA:3.40.50.GENE3D); PTHR24322 (PANTHER), PTHR24322:SF57 (PANTHER), SSF51735 (SUPERFAMILY) | |
| 3575 | lib_dib_Filter15_IDBA_contig_93876_minus1 | 16 | zinc finger protein | 122 | 1.36E-31 | 72.70% | 3 | F:binding; F:nucleic acid binding; C:intracellular | IPR007087 (PROSITE); IPR013087 (G3DSA:3.30.160.GENE3D); IPR015880 (SMART); PTHR24402 (PANTHER), PF13465 (PFAM), SSF57667 (SUPERFAMILY) | |
| 3576 | lib_dib_Filter15_IDBA_contig_93931_plus3 | 8 | proteinase inhibitor i4 serpin | 121 | 1.88E-28 | 67.65% | 1 | F:enzyme regulator activity | IPR000215 (PANTHER); IPR023795 (PROSITE); IPR023796 (PFAM); G3DSA:2.30.39.10 (GENE3D), G3DSA:3.30.497.10 (GENE3D) | |
| 3577 | lib_dib_Filter15_IDBA_contig_94030_minus1 | 15 | low quality protein: cadherin-related tumor suppressor-like | 122 | 1.89E-30 | 67.30% | 4 | F:protein kinase activity; F:calcium ion binding; P:biological_process; C:plasma membrane | IPR002126 (PRINTS); IPR015919 (SUPERFAMILY); IPR020894 (PROSITE); PTHR24027 (PANTHER) | |
| 3578 | lib_dib_Filter15_IDBA_contig_94052_plus3 | 132 | hypothetical protein CAPTEDRAFT_228674 | 375 | 3.17E-113 | 54.05% | 0 | - | PF13383 (PFAM), tmhmm (TMHMM) | |

| | | | | | | | | | | |
|---|---|---|---|---|---|---|---|---|---|---|
| 3579 | lib_dib_Filter15_IDBA_contig_94082_minus2 | 14 | discoidin domain-containing receptor 2-like | 121 | 6.82E-28 | 70.40% | 11 | P:biological_process; P:cellular protein modification process; F:nucleotide binding; P:anatomical structure morphogenesis; P:multicellular organismal development; F:receptor activity; F:protein kinase activity; P:cellular component organization; P:cell differentiation; F:protein binding; P:signal transduction | IPR000421 (PFAM); IPR008979 (SUPERFAMILY); IPR027146 (PTHR10127:PANTHER); G3DSA:2.60.120.260 (GENE3D), PTHR10127 (PANTHER), SignalP-NN(euk) (SIGNALP) | yes |
| 3580 | lib_dib_Filter15_IDBA_contig_94103_minus2 | 17 | ectonucleotide pyrophosphatase phosphodiesterase family member 7 | 121 | 3.87E-20 | 68.50% | 3 | P:biological_process; P:metabolic process; F:catalytic activity | IPR017849 (G3DSA:3.40.720.GENE3D); IPR017850 (SUPERFAMILY); IPR024873 (PANTHER); tmhmm (TMHMM) | |
| 3581 | lib_dib_Filter15_IDBA_contig_94133_plus2 | 12 | zinc finger protein 502-like | 121 | 1.81E-23 | 59.50% | 3 | F:binding; F:nucleic acid binding; C:intracellular | IPR007087 (PFAM); IPR013087 (G3DSA:3.30.160.GENE3D); IPR015880 (SMART); PTHR11389 (PANTHER), PF13465 (PFAM), SSF57667 (SUPERFAMILY) | |
| 3582 | lib_dib_Filter15_IDBA_contig_94196_plus3 | 7 | cre-sul-2 protein | 121 | 2.30E-16 | 50.05% | 2 | F:catalytic activity; P:metabolic process | IPR017850 (SUPERFAMILY); G3DSA:3.30.1120.10 (GENE3D), PTHR10342 (PANTHER), PTHR10342:SF28 (PANTHER) | |
| 3583 | lib_dib_Filter15_IDBA_contig_94367_plus2 | 12 | n-acetylglucosamine-6-sulfatase precursor | 121 | 4.95E-47 | 73.75% | 7 | P:carbohydrate metabolic process; P:catabolic process; F:hydrolase activity; C:lysosome; F:protein binding; F:binding; F:carbohydrate binding | IPR000917 (PFAM); IPR015981 (PTHR10342:PANTHER); IPR017849 (G3DSA:3.40.720.GENE3D); IPR017850 (SUPERFAMILY); PTHR10342 (PANTHER) | |
| 3584 | lib_dib_Filter15_IDBA_contig_94389_minus3 | 12 | chondroitin beta- -n-acetyl galactosaminyl transferase 1 | 121 | 3.98E-44 | 73.80% | 11 | P:biosynthetic process; P:carbohydrate metabolic process; P:cellular component organization; C:Golgi apparatus; P:multicellular organismal development; P:cell proliferation; F:transferase activity; F:binding; P:cell recognition; P:nucleobase-containing compound metabolic process; P:anatomical structure morphogenesis | IPR008428 (PANTHER); G3DSA:3.90.550.10 (GENE3D), SSF53448 (SUPERFAMILY) | |
| 3585 | lib_dib_Filter15_IDBA_contig_94441_minus1 | 36 | insulin-like growth factor-binding protein complex acid labile chain | 282 | 3.72E-14 | 49.80% | 2 | F:catalytic activity; F:protein binding | IPR001611 (PROFILE); IPR003591 (SMART); G3DSA:3.80.10.10 (GENE3D), PTHR24367 (PANTHER), PF13855 (PFAM), SSF52058 (SUPERFAMILY) | |
| 3586 | lib_dib_Filter15_IDBA_contig_94457_minus2 | 38 | deleted in malignant brain tumors 1 | 163 | 1.62E-25 | 54.75% | 2 | F:receptor activity; C:cell | IPR001190 (PRINTS); IPR017448 (SMART); G3DSA:3.10.250.10 (GENE3D), PTHR19331 (PANTHER) | |
| 3587 | lib_dib_Filter15_IDBA_contig_94531_plus3 | 11 | aldehyde reductase | 120 | 2.73E-19 | 63.90% | 3 | P:oxidation-reduction process; F:oxidoreductase activity; F:alditol:NADP+ 1-oxidoreductase activity | IPR001395 (PANTHER); IPR023210 (G3DSA:3.20.20.GENE3D); PTHR11732:SF34 (PANTHER) | |
| 3588 | lib_dib_Filter15_IDBA_contig_9462_minus3 | 1480 | lpxtg-motif cell wall anchor domain partial | 1455 | 1.94E-05 | 51.80% | 3 | C:cell wall; C:membrane; C:extracellular region | SignalP-NN(euk) (SIGNALP) | |
| 3589 | lib_dib_Filter15_IDBA_contig_9462_plus3 | 1480 | collagen alpha-2 chain | 1455 | 9.22E-98 | 63.75% | 10 | P:anatomical structure morphogenesis; P:cellular component organization; P:cell differentiation; P:multicellular organismal development; P:regulation of biological process; F:protein binding; P:response to endogenous stimulus; ; F:structural molecule activity; C:proteinaceous extracellular matrix | IPR001442 (G3DSA:2.170.240.GENE3D); IPR008160 (PFAM); IPR016187 (SUPERFAMILY); PTHR24023 (PANTHER), SignalP-NN(euk) (SIGNALP) | |
| 3590 | lib_dib_Filter15_IDBA_contig_94696_plus2 | 9 | zinc finger protein 271 (zinc finger protein 7) (zinc finger protein znfphex133) (epstein-barr virus-induced zinc finger protein) (znf-eb) (ct-zfp48) (zinc finger protein | 121 | 1.05E-17 | 53.65% | 3 | F:binding; F:nucleic acid binding; C:intracellular | IPR007087 (PROSITE); IPR013087 (G3DSA:3.30.160.GENE3D); IPR015880 (SMART); PTHR11389 (PANTHER), PTHR11389:SF316 (PANTHER), PF13465 (PFAM), PF13912 (PFAM), SSF57667 (SUPERFAMILY) | |

| | | | | | | | | | |
|---|---|---|---|---|---|---|---|---|---|
| 3591 | lib_dib_Filter15_IDBA_contig_94714_minus3 | 10 | protocadherin fat 4 | 120 | 2.35E-31 | 61.70% | 9 | P:multicellular organismal development; P:response to external stimulus; P:response to abiotic stimulus; P:cell differentiation; P:transport; P:cellular component organization; F:calcium ion binding; P:biological_process; C:plasma membrane | IPR002126 (PRINTS); IPR015919 (SUPERFAMILY); IPR020894 (PROSITE); PTHR24027 (PANTHER) |
| 3592 | lib_dib_Filter15_IDBA_contig_94784_plus3 | 12 | er degradation-enhancing alpha-mannosidase-like 2 | 120 | 1.88E-61 | 89.20% | 10 | F:calcium ion binding; P:biosynthetic process; P:cellular protein modification process; P:carbohydrate metabolic process; P:protein metabolic process; F:hydrolase activity; C:endoplasmic reticulum; P:response to stress; P:response to biotic stimulus; C:extracellular region | IPR001382 (PRINTS); PTHR11742:SF10 (PANTHER) |
| 3593 | lib_dib_Filter15_IDBA_contig_94811_plus3 | 28 | alpha-galactosidase alpha-n-acetylgalactosaminidase | 120 | 6.14E-53 | 70.05% | 2 | F:hydrolase activity; P:carbohydrate metabolic process | IPR002241 (PRINTS); IPR013780 (G3DSA:2.60.40.GENE3D); IPR013785 (G3DSA:3.20.20.GENE3D); IPR017853 (SUPERFAMILY); PTHR11452 (PANTHER), SSF51011 (SUPERFAMILY) |
| 3594 | lib_dib_Filter15_IDBA_contig_9482_plus2 | 31 | matrix metalloproteinase-24-like | 132 | 1.10E-33 | 75.00% | 5 | F:peptidase activity; P:protein metabolic process; P:catabolic process; C:extracellular region; F:binding | IPR001818 (PFAM); IPR006026 (SMART); IPR021190 (PRINTS); IPR024079 (G3DSA:3.40.390.GENE3D); PTHR10201 (PANTHER), SSF55486 (SUPERFAMILY) |
| 3595 | lib_dib_Filter15_IDBA_contig_9483_plus2 | 108 | matrix metalloproteinase-24-like | 267 | 1.55E-67 | 62.55% | 5 | F:peptidase activity; P:protein metabolic process; P:catabolic process; F:binding; C:extracellular region | IPR001818 (PFAM); IPR002477 (PFAM); IPR006026 (SMART); IPR021190 (PRINTS); IPR024079 (G3DSA:3.40.390.GENE3D); PTHR10201 (PANTHER), SSF55486 (SUPERFAMILY) |
| 3596 | lib_dib_Filter15_IDBA_contig_94831_minus3 | 11 | beta- -galactosyltransferase | 120 | 2.29E-14 | 54.80% | 1 | P:carbohydrate metabolic process | IPR003859 (PRINTS); G3DSA:3.90.550.10 (GENE3D), SSF53448 (SUPERFAMILY) |
| 3597 | lib_dib_Filter15_IDBA_contig_94980_minus3 | 27 | af355375_1 reverse transcriptase | 120 | 9.76E-22 | 51.80% | 8 | F:zinc ion binding; C:intracellular; F:GTP binding; P:translational initiation; F:GTPase activity; P:GTP catabolic process; F:translation initiation factor activity; F:nucleotide binding | IPR000305 (PFAM); IPR027299 (PROFILE) |
| 3598 | lib_dib_Filter15_IDBA_contig_95046_minus1 | 12 | fibrillin-1- partial | 121 | 5.63E-23 | 62.20% | 2 | F:protein binding; F:calcium ion binding | IPR000152 (PROSITE); IPR000742 (SMART); IPR001881 (PFAM); IPR013032 (PROSITE); IPR018097 (PROSITE); IPR026823 (PFAM); G3DSA:2.10.25.10 (GENE3D), PTHR24034 (PANTHER), PTHR24034:SF0 (PANTHER), SSF57196 (SUPERFAMILY) |
| 3599 | lib_dib_Filter15_IDBA_contig_95120_minus1 | 15 | aldose reductase-like | 120 | 9.12E-33 | 63.55% | 2 | F:catalytic activity; P:metabolic process | IPR001395 (PANTHER); IPR018170 (PROSITE); IPR020471 (PRINTS); IPR023210 (G3DSA:3.20.20.GENE3D); PTHR11732:SF34 (PANTHER) |
| 3600 | lib_dib_Filter15_IDBA_contig_95142_plus2 | 16 | agrin | 120 | 1.06E-17 | 49.85% | 7 | P:metabolic process; P:multicellular organismal development; P:regulation of biological process; P:cellular component organization; P:cell communication; C:extracellular region; F:protein binding | IPR000742 (PROFILE); IPR001791 (PFAM); IPR008985 (SUPERFAMILY); IPR013320 (G3DSA:2.60.120.GENE3D); PTHR10574 (PANTHER) |
| 3601 | lib_dib_Filter15_IDBA_contig_95359_plus2 | 20 | epididymal sperm-binding protein 1 | 120 | 5.07E-07 | 43.80% | 2 | P:single fertilization; C:extracellular region | IPR000562 (G3DSA:2.10.10.GENE3D); IPR013806 (SUPERFAMILY); PTHR22918 (PANTHER), PTHR22918:SF0 (PANTHER) |
| 3602 | lib_dib_Filter15_IDBA_contig_95362_plus1 | 11 | bone morphogenetic protein 6 | 120 | 4.19E-21 | 65.95% | 8 | P:metabolic process; P:regulation of biological process; P:primary metabolic process; P:signal transduction; P:cell differentiation; P:multicellular organismal development; F:receptor binding; P:growth | IPR001111 (PFAM); IPR015615 (PANTHER); PTHR11848:SF55 (PANTHER) |

| | | | | | | | | | | |
|---|---|---|---|---|---|---|---|---|---|---|
| 3603 | lib_dib_Filter15_IDBA_contig_95389_plus3 | 26 | dehydrogenase reductase sdr family member 13-like | 120 | 8.96E-17 | 71.50% | 3 | P:metabolic process; F:catalytic activity; F:nucleotide binding | IPR002198 (PFAM); IPR016040 (G3DSA:3.40.50.GENE3D); PTHR24320 (PANTHER), PTHR24320:SF0 (PANTHER), SignalP-NN(euk) (SIGNALP), tmhmm (TMHMM), SSF51735 (SUPERFAMILY) | |
| 3604 | lib_dib_Filter15_IDBA_contig_95401_minus1 | 39 | af355375_1 reverse transcriptase | 197 | 3.13E-31 | 51.70% | 1 | F:binding | PTHR21301 (PANTHER) | |
| 3605 | lib_dib_Filter15_IDBA_contig_95443_plus2 | 9 | zinc finger protein 271-like | 120 | 2.33E-38 | 72.65% | 3 | F:binding; F:nucleic acid binding; C:intracellular | IPR007087 (PROSITE); IPR013087 (G3DSA:3.30.160.GENE3D); IPR015880 (SMART); PTHR24387 (PANTHER), PF13465 (PFAM), SSF57667 (SUPERFAMILY) | |
| 3606 | lib_dib_Filter15_IDBA_contig_95658_minus2 | 14 | fibrinogen c domain-containing protein 1-like | 120 | 5.07E-23 | 66.40% | 3 | F:receptor binding; C:extracellular space; P:signal transduction | IPR002181 (PFAM); IPR014715 (G3DSA:4.10.530.GENE3D); IPR020837 (PROSITE); PTHR19143 (PANTHER) | |
| 3607 | lib_dib_Filter15_IDBA_contig_95678_plus3 | 8 | low quality protein: papilin-like | 119 | 2.61E-27 | 61.50% | 1 | F:peptidase activity | IPR000884 (PFAM); G3DSA:2.20.100.10 (GENE3D), PTHR13723 (PANTHER), PTHR13723:SF87 (PANTHER) | |
| 3608 | lib_dib_Filter15_IDBA_contig_95919_plus2 | 12 | ankyrin repeat-containing protein | 119 | 5.50E-19 | 56.70% | 1 | F:protein binding | IPR002110 (PFAM); IPR020683 (G3DSA:1.25.40.GENE3D); PTHR24188 (PANTHER), SignalP-NN(euk) (SIGNALP) | |
| 3609 | lib_dib_Filter15_IDBA_contig_95936_minus3 | 12 | glycoside hydrolase | 119 | 3.62E-35 | 67.50% | 2 | F:hydrolase activity; P:carbohydrate metabolic process | IPR002772 (G3DSA:3.40.50.GENE3D); IPR026891 (PFAM); IPR026892 (PANTHER); PTHR30620:SF0 (PANTHER) | |
| 3610 | lib_dib_Filter15_IDBA_contig_96292_minus2 | 8 | low-density lipoprotein receptor-related protein 2 | 119 | 1.01E-36 | 68.40% | 3 | F:catalytic activity; F:protein binding; F:calcium ion binding | IPR000152 (PROSITE); IPR000742 (PFAM); IPR001881 (PFAM); IPR013032 (PROSITE); IPR018097 (PROSITE); G3DSA:2.10.25.10 (GENE3D), PTHR10529 (PANTHER), PTHR10529:SF112 (PANTHER), SSF57196 (SUPERFAMILY) | |
| 3611 | lib_dib_Filter15_IDBA_contig_96418_minus1 | 45 | low quality protein: hemicentin-1 | 234 | 3.79E-92 | 72.05% | 6 | P:biological_process; F:receptor activity; P:anatomical structure morphogenesis; P:multicellular organismal development; C:cell; F:calcium ion binding | IPR000152 (PROSITE); IPR001881 (PFAM); IPR006605 (PFAM); IPR009017 (SUPERFAMILY); IPR018097 (PROSITE); IPR023413 (G3DSA:2.40.155.GENE3D); G3DSA:2.10.25.10 (GENE3D), PTHR24046 (PANTHER), PTHR24046:SF0 (PANTHER), SignalP-NN(euk) (SIGNALP), SSF57196 (SUPERFAMILY) | yes |
| 3612 | lib_dib_Filter15_IDBA_contig_96419_minus1 | 46 | low quality protein: hemicentin-1 | 234 | 3.79E-92 | 72.05% | 6 | P:biological_process; F:receptor activity; P:anatomical structure morphogenesis; P:multicellular organismal development; C:cell; F:calcium ion binding | IPR000152 (PROSITE); IPR001881 (PFAM); IPR006605 (PFAM); IPR009017 (SUPERFAMILY); IPR018097 (PROSITE); IPR023413 (G3DSA:2.40.155.GENE3D); G3DSA:2.10.25.10 (GENE3D), PTHR24046 (PANTHER), PTHR24046:SF0 (PANTHER), SignalP-NN(euk) (SIGNALP), SSF57196 (SUPERFAMILY) | yes |
| 3613 | lib_dib_Filter15_IDBA_contig_96538_plus1 | 37 | macroglobulin complement-related | 211 | 1.33E-65 | 57.60% | 7 | P:response to biotic stimulus; P:cell differentiation; P:cell-cell signaling; F:protein binding; F:binding; P:transport; P:cellular component organization | IPR011625 (PFAM); PTHR11412 (PANTHER), PTHR11412:SF22 (PANTHER) | |
| 3614 | lib_dib_Filter15_IDBA_contig_96568_plus3 | 18 | alpha-mannosidase 2x | 118 | 1.81E-56 | 85.10% | 6 | F:hydrolase activity; C:Golgi apparatus; F:carbohydrate binding; P:carbohydrate metabolic process; C:cell; F:binding | IPR000602 (PFAM); IPR011330 (SUPERFAMILY); IPR027291 (G3DSA:3.20.110.GENE3D); PTHR11607 (PANTHER), PTHR11607:SF4 (PANTHER) | |
| 3615 | lib_dib_Filter15_IDBA_contig_96585_minus1 | 138 | integrin beta subunit | 309 | 3.11E-14 | 53.95% | 9 | P:cell-matrix adhesion; P:integrin-mediated signaling pathway; F:receptor binding; F:receptor activity; P:cell adhesion; C:integrin complex; C:integral to membrane; C:membrane; P:multicellular organismal development | IPR015812 (PANTHER); G3DSA:2.10.25.10 (GENE3D), tmhmm (TMHMM), SSF57196 (SUPERFAMILY) | |

| | | | | | | | | | | |
|---|---|---|---|---|---|---|---|---|---|---|
| 3616 | lib_dib_Filter15_IDBA_contig_96673_minus1 | 16 | low-density lipoprotein receptor-related protein 1-like | 119 | 1.10E-32 | 68.45% | 1 | C:cell | IPR000033 (PFAM); IPR011042 (G3DSA:2.120.10.GENE3D); PTHR10529 (PANTHER), PTHR10529:SF116 (PANTHER), SSF63825 (SUPERFAMILY) | |
| 3617 | lib_dib_Filter15_IDBA_contig_96701_plus1 | 2852 | retinoid-inducible serine carboxypeptidase | 327 | 1.85E-33 | 48.25% | 3 | F:peptidase activity; P:protein metabolic process; P:catabolic process | IPR001563 (PANTHER); G3DSA:3.40.50.1820 (GENE3D), PTHR11802:SF3 (PANTHER), SSF53474 (SUPERFAMILY) | |
| 3618 | lib_dib_Filter15_IDBA_contig_96751_plus2 | 25 | #NAME? | 118 | 1.05E-19 | 57.70% | 1 | F:protein binding | IPR002110 (SMART); IPR020683 (G3DSA:1.25.40.GENE3D); PTHR24198 (PANTHER) | |
| 3619 | lib_dib_Filter15_IDBA_contig_96832_minus3 | 15 | protein gly- isoform b | 118 | 9.68E-53 | 76.90% | 5 | P:carbohydrate metabolic process; C:cell; F:carbohydrate binding; C:Golgi apparatus; F:transferase activity | PTHR11675 (PANTHER), SSF53448 (SUPERFAMILY) | |
| 3620 | lib_dib_Filter15_IDBA_contig_96910_minus1 | 282 | peptidyl-dipeptidase a | 195 | 1.02E-19 | 68.60% | 4 | P:protein metabolic process; P:catabolic process; F:peptidase activity; C:cell | IPR001548 (PANTHER); PTHR10514:SF16 (PANTHER), tmhmm (TMHMM), SSF55486 (SUPERFAMILY) | |
| 3621 | lib_dib_Filter15_IDBA_contig_96938_minus1 | 820 | thioredoxin-1 | 370 | 5.25E-28 | 71.05% | 5 | P:metabolic process; F:electron carrier activity; F:catalytic activity; P:cellular homeostasis; P:regulation of biological process | IPR005746 (PRINTS); IPR012336 (G3DSA:3.40.30.GENE3D); IPR013766 (PFAM); IPR017937 (PROSITE) | |
| 3622 | lib_dib_Filter15_IDBA_contig_96963_plus2 | 11 | protein | 118 | 8.99E-14 | 56.95% | 3 | F:catalytic activity; F:protein binding; F:calcium ion binding | IPR000152 (PROSITE); IPR000742 (PROFILE); IPR001881 (PFAM); IPR013032 (PROSITE); IPR018097 (PROSITE); G3DSA:2.10.25.10 (GENE3D), PTHR24034 (PANTHER), SSF57196 (SUPERFAMILY) | |
| 3623 | lib_dib_Filter15_IDBA_contig_97084_minus3 | 21 | tolloid-like protein 2-like | 118 | 1.03E-11 | 59.60% | 1 | F:hydrolase activity | IPR000859 (G3DSA:2.60.120.GENE3D); PTHR10127 (PANTHER) | |
| 3624 | lib_dib_Filter15_IDBA_contig_97144_plus2 | 20 | low quality protein: slit homolog 1 protein | 118 | 1.62E-49 | 68.80% | 11 | F:receptor binding; F:protein binding; C:extracellular space; C:cytoplasm; P:anatomical structure morphogenesis; P:cellular component organization; P:cell differentiation; P:multicellular organismal development; P:behavior; P:response to external stimulus; P:regulation of biological process | IPR000483 (SMART); G3DSA:3.80.10.10 (GENE3D), PTHR26342 (PANTHER), PTHR26342:SF40 (PANTHER), PF13855 (PFAM), SSF52058 (SUPERFAMILY) | |
| 3625 | lib_dib_Filter15_IDBA_contig_97214_minus3 | 18 | inter-alpha-trypsin inhibitor heavy chain h3-like | 118 | 4.83E-21 | 63.25% | 4 | C:cytoplasm; F:carbohydrate binding; C:plasma membrane; F:calcium ion binding | IPR013694 (PFAM); PTHR10338 (PANTHER), PTHR10338:SF24 (PANTHER) | |
| 3626 | lib_dib_Filter15_IDBA_contig_97248_plus2 | 15 | protocadherin-16 | 137 | 1.01E-24 | 57.65% | 3 | F:calcium ion binding; P:biological_process; C:plasma membrane | IPR002126 (PRINTS); IPR015919 (SUPERFAMILY); IPR020894 (PROSITE); PTHR24027 (PANTHER) | |
| 3627 | lib_dib_Filter15_IDBA_contig_97316_plus2 | 3147 | fibrinogen c domain-containing protein 1-like | 318 | 2.01E-25 | 51.65% | 3 | F:receptor binding; C:extracellular space; P:signal transduction | IPR002181 (PFAM); IPR014715 (G3DSA:4.10.530.GENE3D); IPR014716 (G3DSA:3.90.215.GENE3D); PTHR19143 (PANTHER), tmhmm (TMHMM) | |
| 3628 | lib_dib_Filter15_IDBA_contig_97317_plus1 | 2475 | fibrinogen c domain-containing protein 1-like | 280 | 7.40E-24 | 52.50% | 3 | F:receptor binding; C:extracellular space; P:signal transduction | IPR002181 (PFAM); IPR014715 (G3DSA:4.10.530.GENE3D); IPR014716 (G3DSA:3.90.215.GENE3D); PTHR19143 (PANTHER), SignalP-NN(euk) (SIGNALP), tmhmm (TMHMM) | yes |
| 3629 | lib_dib_Filter15_IDBA_contig_97425_plus1 | 133 | d-dopachrome decarboxylase | 269 | 3.03E-18 | 53.75% | 3 | P:multicellular organismal development; P:regulation of biological process; P:cell death | IPR001398 (PRODOM); IPR014347 (SUPERFAMILY); G3DSA:3.30.429.10 (GENE3D) | |
| 3630 | lib_dib_Filter15_IDBA_contig_97437_plus2 | 11 | ankyrin repeat protein | 118 | 1.59E-17 | 57.60% | 1 | F:protein binding | IPR002110 (PRINTS); IPR020683 (G3DSA:1.25.40.GENE3D); PTHR24198 (PANTHER) | |
| 3631 | lib_dib_Filter15_IDBA_contig_97479_plus3 | 10 | ankyrin repeat and socs box protein 8 | 117 | 1.07E-41 | 72.15% | 4 | C:cytoplasm; P:biological_process; C:nucleus; F:protein binding | IPR002110 (SMART); IPR020683 (G3DSA:1.25.40.GENE3D); PTHR24134 (PANTHER), PTHR24134:SF1 (PANTHER) | |

| | | | | | | | | | | |
|---|---|---|---|---|---|---|---|---|---|---|
| 3632 | lib_dib_Filter15_IDBA_contig_97480_minus3 | 8 | zinc finger protein 23 isoform 2 | 117 | 2.57E-14 | 49.45% | 3 | F:binding; F:nucleic acid binding; C:intracellular | IPR007087 (PFAM); IPR013087 (G3DSA:3.30.160.GENE3D); IPR015880 (SMART); PTHR11389 (PANTHER), PTHR11389:SF357 (PANTHER), PF13465 (PFAM), SSF57667 (SUPERFAMILY) | |
| 3633 | lib_dib_Filter15_IDBA_contig_9754_minus2 | 607 | pregnancy zone partial | 137 | 3.51E-39 | 59.70% | 1 | C:extracellular region | IPR009048 (G3DSA:2.60.40.GENE3D); PTHR11412 (PANTHER), PTHR11412:SF36 (PANTHER) | |
| 3634 | lib_dib_Filter15_IDBA_contig_9755_minus2 | 1208 | pregnancy zone partial | 137 | 7.02E-39 | 59.05% | 1 | C:extracellular region | IPR009048 (G3DSA:2.60.40.GENE3D); PTHR11412 (PANTHER), PTHR11412:SF36 (PANTHER) | |
| 3635 | lib_dib_Filter15_IDBA_contig_97646_plus3 | 7 | meteorin-like protein | 117 | 1.20E-16 | 51.10% | 2 | C:extracellular region; F:molecular_function | IPR024821 (PANTHER) | |
| 3636 | lib_dib_Filter15_IDBA_contig_97797_minus3 | 25 | supporter of activation of yellow protein | 117 | 4.72E-21 | 79.50% | 3 | C:extracellular region; F:binding; C:cytoplasm | PTHR10615 (PANTHER) | |
| 3637 | lib_dib_Filter15_IDBA_contig_97828_minus2 | 8 | multiple epidermal growth factor-like domains protein 6-like | 117 | 5.32E-11 | 53.95% | 2 | F:calcium ion binding; P:biological_process | PTHR24035 (PANTHER), PTHR24035:SF4 (PANTHER) | |
| 3638 | lib_dib_Filter15_IDBA_contig_97883_minus1 | 46 | l-xylulose reductase | 225 | 4.89E-50 | 67.90% | 5 | P:carbohydrate metabolic process; F:catalytic activity; P:cellular component organization; C:cell; F:nucleotide binding | IPR002198 (PRINTS); IPR002347 (PRINTS); IPR016040 (G3DSA:3.40.50.GENE3D); PTHR24311 (PANTHER), PTHR24311:SF0 (PANTHER), SSF51735 (SUPERFAMILY) | |
| 3639 | lib_dib_Filter15_IDBA_contig_97884_minus1 | 29 | l-xylulose reductase | 178 | 6.76E-36 | 68.15% | 7 | C:organelle; C:intracellular; P:carbohydrate metabolic process; F:catalytic activity; C:cytoplasm; P:cellular component organization; F:nucleotide binding | IPR002198 (PRINTS); IPR002347 (PRINTS); IPR016040 (G3DSA:3.40.50.GENE3D); PTHR24311 (PANTHER), PTHR24311:SF0 (PANTHER), SSF51735 (SUPERFAMILY) | |
| 3640 | lib_dib_Filter15_IDBA_contig_97899_minus1 | 11 | egf-like domain-containing | 117 | 9.95E-26 | 59.45% | 2 | F:protein binding; F:calcium ion binding | IPR000742 (SMART); IPR001881 (PFAM); IPR013032 (PROSITE); IPR018097 (PROSITE); IPR026823 (PFAM); G3DSA:2.10.25.10 (GENE3D), PTHR24048 (PANTHER), SSF57196 (SUPERFAMILY) | |
| 3641 | lib_dib_Filter15_IDBA_contig_97910_plus2 | 15 | aminopeptidase pils (m01 family) | 117 | 1.21E-21 | 52.65% | 4 | C:cell; F:peptidase activity; P:protein metabolic process; P:catabolic process | IPR001254 (PFAM); IPR009003 (SUPERFAMILY); IPR018114 (PROSITE); G3DSA:2.40.10.10 (GENE3D), PTHR24268 (PANTHER), PTHR24268:SF1 (PANTHER) | |
| 3642 | lib_dib_Filter15_IDBA_contig_97981_plus2 | 10 | thioredoxin domain-containing protein 16 | 117 | 3.97E-21 | 61.55% | 5 | P:cell redox homeostasis; F:molecular_function; P:biological_process; C:extracellular region; C:cellular_component | PTHR22699 (PANTHER) | |
| 3643 | lib_dib_Filter15_IDBA_contig_98052_plus3 | 18 | lysosomal acid lipase isoform cra_a | 103 | 7.74E-11 | 70.63% | 11 | P:multicellular organismal development; C:lysosome; P:lipid metabolic process; F:hydrolase activity; P:biological_process; P:response to external stimulus; P:response to stress; P:anatomical structure morphogenesis; P:cellular component organization; P:biosynthetic process; P:cell proliferation | IPR006693 (PFAM); G3DSA:3.40.50.1820 (GENE3D), PTHR11005 (PANTHER), PTHR11005:SF0 (PANTHER), SignalP-NN(euk) (SIGNALP), tmhmm (TMHMM) | yes |
| 3644 | lib_dib_Filter15_IDBA_contig_98065_minus1 | 13 | plxna2 partial | 117 | 6.22E-44 | 77.75% | 8 | P:signal transduction; C:intracellular; P:multicellular organismal development; P:regulation of biological process; P:biological_process; F:receptor activity; F:enzyme regulator activity; C:plasma membrane | IPR013548 (PFAM); G3DSA:3.10.20.90 (GENE3D), PTHR22625 (PANTHER) | |
| 3645 | lib_dib_Filter15_IDBA_contig_98070_minus2 | 15 | zinc finger protein 271-like | 117 | 4.35E-36 | 64.90% | 3 | F:binding; F:nucleic acid binding; C:intracellular | IPR007087 (PROSITE); IPR013087 (G3DSA:3.30.160.GENE3D); IPR015880 (SMART); PTHR24402 (PANTHER), PF13465 (PFAM), SSF57667 (SUPERFAMILY) | |

| | | | | | | | | | | |
|---|---|---|---|---|---|---|---|---|---|---|
| 3646 | lib_dib_Filter15_IDBA_contig_98148_minus1 | 12 | lysyl oxidase homolog 4 | 117 | 4.15E-10 | 60.20% | 2 | F:receptor activity; C:cell | IPR000859 (G3DSA:2.60.120.GENE3D); IPR001190 (PFAM); IPR017448 (SMART); G3DSA:3.10.250.10 (GENE3D), PTHR19331 (PANTHER), PTHR19331:SF118 (PANTHER) | |
| 3647 | lib_dib_Filter15_IDBA_contig_98196_plus3 | 16 | xanthine dehydrogenase oxidase | 116 | 8.69E-36 | 78.00% | 5 | F:nucleotide binding; F:binding; F:catalytic activity; F:electron carrier activity; P:metabolic process | IPR002888 (G3DSA:1.10.150.GENE3D); PTHR11908 (PANTHER), PTHR11908:SF5 (PANTHER) | |
| 3648 | lib_dib_Filter15_IDBA_contig_98205_minus3 | 6 | von willebrand factor type egf and pentraxin domain-containing protein 1-like | 116 | 2.42E-18 | 53.80% | 1 | F:calcium ion binding | IPR000436 (PFAM); IPR001881 (PFAM); G3DSA:2.10.25.10 (GENE3D), G3DSA:2.10.70.10 (GENE3D), PTHR19325 (PANTHER), SSF57196 (SUPERFAMILY) | |
| 3649 | lib_dib_Filter15_IDBA_contig_9840_minus2 | 124 | sulfhydryl oxidase 1 | 368 | 4.29E-56 | 57.60% | 5 | P:cellular protein modification process; C:extracellular space; F:catalytic activity; C:Golgi apparatus; P:metabolic process | IPR006863 (G3DSA:1.20.120.GENE3D); IPR017905 (PROFILE); PTHR22897 (PANTHER) | |
| 3650 | lib_dib_Filter15_IDBA_contig_98420_minus3 | 23 | paramyosin | 116 | 1.01E-35 | 75.70% | 5 | C:cytoplasm; C:extracellular region; C:cytoskeleton; C:protein complex; F:motor activity | IPR002928 (PFAM); PTHR13140 (PANTHER), PTHR13140:SF106 (PANTHER) | |
| 3651 | lib_dib_Filter15_IDBA_contig_98458_minus2 | 2277 | transmembrane serine protease 8 precursor | 453 | 5.12E-54 | 51.35% | 3 | F:peptidase activity; P:protein metabolic process; P:catabolic process | IPR001254 (PFAM); IPR001314 (PRINTS); IPR009003 (SUPERFAMILY); IPR018114 (PROSITE); G3DSA:2.40.10.10 (GENE3D), PTHR24268 (PANTHER), SignalP-NN(euk) (SIGNALP), tmhmm (TMHMM) | yes |
| 3652 | lib_dib_Filter15_IDBA_contig_98459_minus2 | 2355 | transmembrane serine protease 8 precursor | 472 | 7.74E-54 | 51.35% | 3 | F:peptidase activity; P:protein metabolic process; P:catabolic process | IPR001254 (PFAM); IPR001314 (PRINTS); IPR009003 (SUPERFAMILY); IPR018114 (PROSITE); G3DSA:2.40.10.10 (GENE3D), PTHR24268 (PANTHER), SignalP-NN(euk) (SIGNALP), tmhmm (TMHMM) | yes |
| 3653 | lib_dib_Filter15_IDBA_contig_98460_minus1 | 160 | transmembrane serine protease 8 precursor | 117 | 6.82E-27 | 60.20% | 3 | F:peptidase activity; P:protein metabolic process; P:catabolic process | IPR001254 (PFAM); IPR009003 (SUPERFAMILY); G3DSA:2.40.10.10 (GENE3D), PTHR24268 (PANTHER) | |
| 3654 | lib_dib_Filter15_IDBA_contig_98523_minus2 | 18 | hypothetical protein EAI_16186 | 116 | 3.33E-05 | 84.71% | 0 | - | no IPS match | |
| 3655 | lib_dib_Filter15_IDBA_contig_9855_plus3 | 151 | glycoprotein-n-acetylgalactosamine 3-beta-galactosyltransferase 1 | 463 | 3.29E-69 | 60.05% | 7 | P:anatomical structure morphogenesis; P:multicellular organismal development; C:cell; F:transferase activity; P:biosynthetic process; P:cellular protein modification process; P:carbohydrate metabolic process | IPR003378 (PFAM); IPR026842 (PTHR23033:PANTHER); PTHR23033 (PANTHER), tmhmm (TMHMM) | |
| 3656 | lib_dib_Filter15_IDBA_contig_98597_plus1 | 8 | leukocyte elastase inhibitor-like | 117 | 1.99E-27 | 64.75% | 5 | P:metabolic process; P:regulation of biological process; P:protein metabolic process; P:catabolic process; F:enzyme regulator activity | IPR000215 (PANTHER); IPR023796 (PFAM); G3DSA:3.30.497.10 (GENE3D) | |
| 3657 | lib_dib_Filter15_IDBA_contig_98720_minus2 | 19 | beta-mannosidase | 116 | 1.77E-23 | 62.50% | 5 | F:binding; C:lysosome; F:hydrolase activity; P:catabolic process; P:carbohydrate metabolic process | IPR006102 (PFAM); IPR013812 (G3DSA:2.60.40.GENE3D); PTHR10066 (PANTHER), PTHR10066:SF12 (PANTHER) | |
| 3658 | lib_dib_Filter15_IDBA_contig_98748_minus1 | 3532 | loc397853 partial | 474 | 5.71E-53 | 56.55% | 3 | F:peptidase activity; P:protein metabolic process; P:catabolic process | IPR001254 (PFAM); IPR001314 (PRINTS); IPR009003 (SUPERFAMILY); IPR018114 (PROSITE); G3DSA:2.40.10.10 (GENE3D), PTHR24275 (PANTHER), SignalP-NN(euk) (SIGNALP) | yes |
| 3659 | lib_dib_Filter15_IDBA_contig_98749_minus3 | 36 | elastase 4 precursor | 109 | 5.58E-20 | 61.45% | 3 | F:peptidase activity; P:protein metabolic process; P:catabolic process | IPR001254 (PFAM); IPR009003 (SUPERFAMILY); IPR018114 (PROSITE); G3DSA:2.40.10.10 (GENE3D), PTHR24250 (PANTHER) | |
| 3660 | lib_dib_Filter15_IDBA_contig_98750_minus1 | 243 | elastase 4 precursor | 114 | 1.57E-23 | 63.00% | 3 | F:peptidase activity; P:protein metabolic process; P:catabolic process | IPR001254 (PFAM); IPR001314 (PRINTS); IPR009003 (SUPERFAMILY); IPR018114 (PROSITE); G3DSA:2.40.10.10 (GENE3D), PTHR24250 (PANTHER) | |

| | | | | | | | | | |
|---|---|---|---|---|---|---|---|---|---|
| 3661 | lib_dib_Filter15_IDBA_contig_98803_minus1 | 21 | protein gly- isoform b | 117 | 2.47E-37 | 78.35% | 8 | P:biosynthetic process; P:cellular protein modification process; P:carbohydrate metabolic process; C:cell; F:transferase activity; P:lipid metabolic process; F:carbohydrate binding; C:Golgi apparatus | IPR001173 (PFAM); G3DSA:3.90.550.10 (GENE3D), PTHR11675 (PANTHER), SSF53448 (SUPERFAMILY) |
| 3662 | lib_dib_Filter15_IDBA_contig_98994_plus1 | 10 | dna methylase n-4 n-6 domain protein | 116 | 3.06E-09 | 62.36% | 2 | P:proteolysis; F:cysteine-type peptidase activity | no IPS match |
| 3663 | lib_dib_Filter15_IDBA_contig_98994_plus2 | 10 | dna methylase n-4 n-6 domain protein | 116 | 4.35E-10 | 62.42% | 4 | F:transferase activity; F:3-deoxy-7-phosphoheptulonate synthase activity; F:phosphotransferase activity, alcohol group as acceptor; F:transferase activity, transferring phosphorus-containing groups | no IPS match |
| 3664 | lib_dib_Filter15_IDBA_contig_99009_plus2 | 105 | ankyrin repeat protein | 338 | 6.99E-15 | 50.00% | 1 | F:protein binding | IPR002110 (SMART); IPR020683 (G3DSA:1.25.40.GENE3D); PTHR24154 (PANTHER) |
| 3665 | lib_dib_Filter15_IDBA_contig_99010_plus3 | 120 | ankyrin repeat protein | 341 | 6.81E-15 | 50.05% | 1 | F:protein binding | IPR002110 (SMART); IPR020683 (G3DSA:1.25.40.GENE3D); PTHR24154 (PANTHER) |
| 3666 | lib_dib_Filter15_IDBA_contig_99152_minus1 | 6 | zinc finger protein 501 | 116 | 3.38E-16 | 55.85% | 3 | F:binding; F:nucleic acid binding; C:intracellular | IPR007087 (PROSITE); IPR013087 (G3DSA:3.30.160.GENE3D); IPR015880 (SMART); PTHR11389 (PANTHER), PF13465 (PFAM), SSF57667 (SUPERFAMILY) |
| 3667 | lib_dib_Filter15_IDBA_contig_99242_plus3 | 8 | a chain x-ray structure of c-met with triazolopyridazine | 115 | 3.85E-10 | 52.05% | 7 | F:protein kinase activity; C:plasma membrane; P:signal transduction; P:multicellular organismal development; P:anatomical structure morphogenesis; F:nucleotide binding; P:cellular protein modification process | IPR000719 (PROFILE); IPR001245 (PFAM); IPR011009 (SUPERFAMILY); G3DSA:1.10.510.10 (GENE3D), PTHR24416 (PANTHER), PTHR24416:SF85 (PANTHER) |
| 3668 | lib_dib_Filter15_IDBA_contig_99261_plus2 | 11 | zinc finger protein 271 (zinc finger protein 7) (zinc finger protein znfphex133) (epstein-barr virus-induced zinc finger protein) (znf-eb) (ct-zfp48) (zinc finger protein | 116 | 1.69E-30 | 63.35% | 3 | F:binding; F:nucleic acid binding; C:intracellular | IPR007087 (PROSITE); IPR013087 (G3DSA:3.30.160.GENE3D); IPR015880 (SMART); PTHR11389 (PANTHER), PF13465 (PFAM), SSF57667 (SUPERFAMILY) |
| 3669 | lib_dib_Filter15_IDBA_contig_99272_minus2 | 9 | von willebrand factor type egf and pentraxin domain-containing protein 1 | 116 | 3.82E-13 | 56.20% | 1 | F:chromatin binding | IPR003410 (PFAM); PTHR19325 (PANTHER), PTHR19325:SF26 (PANTHER) |
| 3670 | lib_dib_Filter15_IDBA_contig_99314_minus3 | 17 | krueppel-like factor 15 | 115 | 7.99E-50 | 92.55% | 9 | F:binding; C:nucleolus; P:transport; P:cell differentiation; P:multicellular organismal development; F:DNA binding; F:sequence-specific DNA binding transcription factor activity; P:regulation of biological process; | IPR007087 (PROSITE); IPR013087 (G3DSA:3.30.160.GENE3D); IPR015880 (SMART); PTHR23223 (PANTHER), PTHR23223:SF19 (PANTHER), PF13465 (PFAM), SSF57667 (SUPERFAMILY) |
| 3671 | lib_dib_Filter15_IDBA_contig_99351_minus3 | 9 | dehydrogenase reductase sdr family member 11-like | 115 | 1.24E-27 | 66.20% | 3 | P:metabolic process; F:catalytic activity; F:nucleotide binding | IPR002198 (PFAM); IPR016040 (G3DSA:3.40.50.GENE3D); PTHR24322 (PANTHER), PTHR24322:SF7 (PANTHER), SSF51735 (SUPERFAMILY) |

| | | | | | | | | | | |
|---|---|---|---|---|---|---|---|---|---|---|
| 3672 | lib_dib_Filter15_IDBA_contig_99396_minus2 | 33 | 72 kda type iv collagenase | 116 | 1.02E-12 | 65.90% | 20 | P:multicellular organismal development; P:reproduction; P:cellular component organization; P:response to stress; P:biological_process; F:peptidase activity; P:response to external stimulus; P:response to abiotic stimulus; P:anatomical structure morphogenesis; C:extracellular space; P:regulation of biological process; P:cell differentiation; P:cell death; P:metabolic process; P:protein metabolic process; P:catabolic process; P:response to endogenous stimulus; F:protein binding; C:plasma membrane; C:cytoplasm | IPR000562 (G3DSA:2.10.10.GENE3D); IPR013806 (SUPERFAMILY); PR00013 (PRINTS), PTHR22803 (PANTHER), PTHR22803:SF17 (PANTHER) | |
| 3673 | lib_dib_Filter15_IDBA_contig_99412_minus2 | 9 | dbh-like 1 | 116 | 1.11E-27 | 60.00% | 4 | F:binding; P:metabolic process; F:catalytic activity; | IPR000323 (G3DSA:2.60.120.GENE3D); IPR000945 (PANTHER); IPR008977 (SUPERFAMILY); IPR014784 (G3DSA:2.60.120.GENE3D); PF03712 (PFAM) | |
| 3674 | lib_dib_Filter15_IDBA_contig_99472_plus3 | 12 | fmrfamide partial | 115 | 6.05E-18 | 64.55% | 1 | P:signal transduction | IPR002544 (PFAM); PTHR20986 (PANTHER) | |
| 3675 | lib_dib_Filter15_IDBA_contig_99597_plus1 | 12 | heat shock protein 70 cognate | 116 | 3.31E-16 | 86.55% | 25 | C:cell; F:receptor binding; P:cellular component organization; P:transport; P:cell-cell signaling; C:cytoplasmic membrane-bounded vesicle; F:nucleotide binding; P:regulation of biological process; ; C:nucleolus; C:cytosol; P:cell cycle; F:protein binding; C:organelle; C:extracellular region; P:protein metabolic process; C:protein complex; F:hydrolase activity; P:nucleobase-containing compound metabolic process; P:catabolic process; C:plasma membrane; C:intracellular; P:response to stress; P:response to biotic stimulus; P:biological_process | G3DSA:1.20.1270.10 (GENE3D), PTHR25941 (PANTHER), PTHR25941:SF1 (PANTHER), SSF100934 (SUPERFAMILY) | |
| 3676 | lib_dib_Filter15_IDBA_contig_99608_plus2 | 20 | zinc finger protein 366 | 115 | 1.99E-40 | 64.25% | 5 | P:biological_process; P:regulation of biological process; C:intracellular; F:binding; F:nucleic acid binding | IPR007087 (PFAM); IPR013087 (G3DSA:3.30.160.GENE3D); IPR015880 (SMART); PTHR24390 (PANTHER), PTHR24390:SF13 (PANTHER), PF13894 (PFAM), SSF57667 (SUPERFAMILY) | |
| 3677 | lib_dib_Filter15_IDBA_contig_99620_plus3 | 8 | beta-( )-xylosyltransferase | 115 | 1.14E-20 | 54.40% | 2 | F:transferase activity, transferring glycosyl groups; C:extracellular region | IPR007657 (PFAM); PTHR20961 (PANTHER), PTHR20961:SF0 (PANTHER) | |
| 3678 | lib_dib_Filter15_IDBA_contig_997_plus1 | 327 | plasma alpha-l-fucosidase | 166 | 4.46E-69 | 72.95% | 2 | F:hydrolase activity; P:carbohydrate metabolic process | IPR000933 (PANTHER); IPR013781 (G3DSA:3.20.20.GENE3D); IPR016286 (PRINTS); IPR017853 (SUPERFAMILY); PTHR10030:SF3 (PANTHER) | |
| 3679 | lib_dib_Filter15_IDBA_contig_99727_minus1 | 15 | fibrinogen c domain-containing protein 1-like | 116 | 4.05E-26 | 62.55% | 3 | F:receptor binding; C:extracellular space; P:signal transduction | IPR002181 (PFAM); IPR014715 (G3DSA:4.10.530.GENE3D); IPR014716 (G3DSA:3.90.215.GENE3D); PTHR19143 (PANTHER) | |
| 3680 | lib_dib_Filter15_IDBA_contig_99826_minus1 | 9 | arylsulfatase j | 115 | 1.04E-21 | 58.60% | 2 | F:hydrolase activity; P:metabolic process | IPR017850 (SUPERFAMILY); G3DSA:3.30.1120.10 (GENE3D), PTHR10342 (PANTHER), PTHR10342:SF19 (PANTHER), SignalP-NN(euk) (SIGNALP) | yes |
| 3681 | lib_dib_Filter15_IDBA_contig_99928_minus1 | 14 | chitinase domain-containing protein 1-like | 115 | 1.31E-42 | 65.75% | 2 | F:hydrolase activity; P:carbohydrate metabolic process | IPR001223 (PFAM); IPR013781 (G3DSA:3.20.20.GENE3D); IPR017853 (SUPERFAMILY); PTHR11177 (PANTHER) | |

**Supplementary Table 7**

**ProtTest 3 Model Selection results**

| | **Best Fitting Model** | | | | **Scond Best Fitting Model** | | | | **Third Best Fitting Model** | | | | | | |
|---|---|---|---|---|---|---|---|---|---|---|---|---|---|---|---|
| **Data set** | ***BIC*** | ***.-lnl*** | ***LnL*** | ***.-lnl*** | ***BIC*** | ***.-lnl*** | ***LnL*** | ***.-lnl*** | ***BIC*** | ***.-lnl*** | ***LnL*** | ***.-lnl*** | BIC - LnL | Criterion | Model Selected |
| Actinoporin-like | WAG+I+G | 10515.01 | WAG+I+G | 10515.01 | LG+I+G | 10561.37 | WAG+I+G+F | 10523.73 | WAG+I+G+F | 10523.73 | LG+I+G | 10561.37 | 0 | both | WAG+I+G |
| CAP | WAG+I+G | 18034.58 | WAG+I+G | 18034.58 | WAG+I+G+F | 18037.27 | WAG+I+G+F | 18037.27 | LG+I+G | 18096.47 | LG+I+G+F | 18096.47 | 0 | both | WAG+I+G |
| Chitinase | LG+I+G | 20788 | LG+I+G | 20788 | WAG+I+G | 20826.78 | RtRev+I+G+F | 20812.9 | RtRev+I+G+F | 20812.9 | LG+I+G+F | 20819.99 | 0 | both | LG+I+G |
| C-type Lectin | WAG+I+G | 18232.91 | WAG+I+G+F | 18227.42 | Blosum62+I+G | 18251.24 | WAG+I+G | 18232.91 | LG+I+G | 18272.65 | Blosum62+I+G+F | 18236.44 | 5.49 | LnL | WAG+I+G+F |
| Cystatin | WAG+I+G | 7284.9 | WAG+I+G+F | 7270.38 | LG+I+G | 7317.74 | WAG+I+G | 7284.9 | WAG+I+G+F | 7270.38 | LG+I+G+F | 7305.7 | 14.52 | LnL | WAG+I+G+F |
| Hyaluronidase | LG+I+G | 21850.32 | WAG+I+G+F | 21831.39 | WAG+I+G+F | 21831.39 | LG+I+G+F | 21841.82 | WAG+I+G | 21889.99 | LG+I+G | 21850.32 | 18.93 | LnL | WAG+I+G+F |
| Kazal | WAG+I+G+F | 16486.19 | WAG+I+G+F | 16486.19 | VT+I+G+F | 16522.51 | VT+I+G+F | 16522.51 | Blosum62+I+G+F | 16531.04 | Blosum62+I+G+F | 16531.04 | 0 | both | WAG+I+G+F |
| Kunitz | WAG+I+G | 7411.68 | WAG+I+G | 7411.68 | Blosum62+I+G | 7452.18 | Blosum62+I+G | 7452.18 | LG+I+G | 7476.41 | LG+I+G | 7476.41 | 0 | both | WAG+I+G |
| Lipocalin | WAG+I+G+F | 19935.54 | WAG+I+G+F | 19935.54 | WAG+I+G | 20005.22 | Blosum62+I+G+F | 19976.14 | BLOSUM62+I+G+F | 19976.14 | WAG+I+G | 20005.22 | 0 | both | WAG+I+G+F |
| Metallopeptidase M12 | WAG+I+G | 22038.95 | Blosum62+I+G+F | 22009.7 | Blosum62+I+G+F | 22009.7 | WAG+I+F+G | 22013.79 | WAG+I+G+F | 22013.79 | WAG+I+G | 22038.95 | 29.25 | LnL | Blosum62+I+G+F |
| Peptidase S1 | LG+I+G | 43267.74 | LG+I+G | 43267.74 | WAG+I+G | 43335.95 | WAG+I+G | 43335.95 | CpREV+I+G | 43464.1 | WAG+I+G+F | 43443.26 | 0 | both | LG+I+G |
| Peptidase S10 | LG+I+G | 37406.7 | LG+I+G | 37406.7 | WAG+I+G | 37439.91 | WAG+I+G | 37439.91 | WAG+I+G+F | 37445.09 | WAG+I+G+F | 37445.09 | 0 | both | LG+I+G |
| Phospholipase A2 | WAG+I+G | 5102.6 | WAG+I+G | 5102.6 | Blosum62+I+G | 5120.64 | VT+I+G+F | 5115.76 | LG+I+G | 5144.46 | Blosum62+I+G | 5120.64 | 0 | both | WAG+I+G |
| Serpin | LG+I+G | 25121.39 | LG+I+G+F | 25086.74 | LG+I+G+F | 25086.74 | LG+I+G | 25121.39 | WAG+I+G+F | 25160.5 | WAG+I+G+F | 25160.5 | 34.65 | LnL | LG+I+G+F |
| ShKT | VT+I+G+F | 4944.28 | VT+I+G+F | 4944.28 | VT+F | 4984.1 | VT+F | 4984.1 | WAG+I+G | 5059.18 | WAG+I+G+F | 5013.35 | 0 | both | VT+I+G+F |
| Sphingomylinase | WAG+I+G | 19251.5 | WAG+I+G+F | 19226.81 | WAG+I+G+F | 19226.81 | WAG+I+G | 19251.5 | Blosum62+I+G | 19291.74 | Blosum62+I+G+F | 19257.69 | 24.69 | LnL | WAG+I+G+F |
| Stonustoxin-like | JTT+I+G | 10197.02 | JTT+I+G+F | 10153.62 | WAG+I+G | 10207.79 | WAG+I+G+F | 10181.81 | JTT+I+G+F | 10153.62 | JTT+I+G | 10197.02 | 43.4 | LnL | JTT+I+G+F |

## Supplementary table S8

| Toxin family | Figure No. | Venom contig | Body contig | Notes on branch length. |
|---|---|---|---|---|
| Cystatin | Supp_Figure S10 | lib_tri_Filter15_IDBA_contig_7467 | lib3_tri_Filter15_IDBA_contig_947 | 99% Pairwise identity, Contig lengths differ, Body contig and G. fallax contig (2199_minus3) similar in length. |
| Kazal | Supp_Figure S12 | lib_tri_Filter15_IDBA_contig_1248 | lib3_tri_Filter15_IDBA_contig_1235 | Identical sequences. Venom contig has longer length. |
| | | lib_tri_Filter15_IDBA_contig_673 | lib3_tri_Filter15_IDBA_contig_652 | 98.9% Pairwise identity, Body contig has longer length. |
| | | lib_tri_Filter15_IDBA_contig_4060 | lib3_tri_Filter15_IDBA_contig_2710 | 90.9% Pairwise identity, Body contig has longer length. |
| | | | lib3_tri_Filter15_IDBA_contig_1892 | |
| | | | lib3_tri_Filter15_IDBA_contig_1893 | 93.4% Pairwise identity, Contig lengths differ. |
| | | | lib3_tri_Filter15_IDBA_contig_3351 | |
| | | | lib3_tri_Filter15_IDBA_contig_3353 | 95.1% Pairwise identity, Contig lengths differ. |
| Peptidase S1 | Supp_Figure S18 | lib_tri_Filter15_IDBA_contig_1066 | lib3_tri_Filter15_IDBA_contig_8198 | |
| | | lib_tri_Filter15_IDBA_contig_1067 | | Venom contigs 100% Identical, all contigs have unique length. Body contig has 91% and 93.2% Identity to venom contigs. |
| | | lib_tri_Filter15_IDBA_contig_7573 | lib3_tri_Filter15_IDBA_contig_1905 | 100% Identical, Contig lengths differ. |
| | | lib_tri_Filter15_IDBA_contig_3398 | lib3_tri_Filter15_IDBA_contig_1886 | 98.5% Pairwise Identiy, Contig lengths differ. |
| | | lib_tri_Filter15_IDBA_contig_7956 | lib3_tri_Filter15_IDBA_contig_280 | 100% Identical, Contig lengths differ. |
| Pep S10 | Supp_Figure S19 | lib_tri_Filter15_IDBA_contig_7540 | lib3_tri_Filter15_IDBA_contig_5709 | 100% Identical, Contig lengths differ, Body contig unpaired (extends N-terminally), Venom contig unpaired (extends C-terminally). |
| Serpin | Supp_Figure S20 | lib_tri_Filter15_IDBA_contig_6362 | lib3_tri_Filter15_IDBA_contig_3072 | 100% Identical, Contig lengths differ, Venom contig unpaired (extends N-terminally), Body contig unpaired (extends C-terminally). |
