# Supplementary material for: A Polychaete’s Powerful Punch: Venom Gland Transcriptomics of Glycera Reveals a Complex Cocktail of Toxin Homologs
Source: Genome Biol Evol. 2014 Sep 5;6(9):2406–23. doi: 10.1093/gbe/evu190 (PMC4202326; doi:10.1093/gbe/evu190)
Supplement: Supplementary Data [file supp_evu190_suppl_data.zip › Supp_file1_final.pdf]

Supplementary Material

A polychaete's powerful punch: venom gland transcriptomics of *Glycera* reveals a complex cocktail of toxin homologs

Björn M. von Reumont, Lahcen Campbell, Sandy Richter, Lars Hering, Dan Sykes, Jörg Hetmank, Ronald A. Jenner, Christoph Bleidorn

Supplementary Material includes:

Supplementary_file1: Supplementary Text, References, Supplementary Figures S1-S20

Supplementary _file2: Supplementary Tables 1-8

Supplementary_file3: Alignments, datasets, scripts

Supplementary Text

CAP proteins

The CAP protein superfamily includes cysteine-rich secretory proteins (CRISPs), antigen 5 (Ag5), and pathogenesis-related 1 (Pr-1) proteins. All these proteins were initially named differently and an evolutionary relationship was recognized later due to sequence similarity (Gibbs et al. 2008). CAP proteins have been identified in the venoms of many

taxa, including cnidarians, cone snails, cephalopods, arthropods, monotremes, bats, and toxicoferans (Fry et al. 2009; Low et al. 2013; Moran et al. 2013; Wong et al. 2013). Especially in the latter taxon, which unites snakes and lizards, it has been shown that CAP proteins play an important role as an active component of the venom (Sunagar et al. 2012). The activities known for CAP proteins include blocking of ion channels and inhibition of muscle contraction (Yamazaki and Morita 2004).

Our analysis pipeline could identify several transcripts representing CAP protein homologs. They were found in all three investigated species and the majority of these transcripts form a clade, which clusters together with homologs from cone snails (Supplementary Figure S8). Within this clade several *G. dibranchiata* paralogs can be found. However, it remains to be seen if this relative abundance of transcripts in *G. dibranchiata* is an artifact of the uneven sequencing depth of the different libraries. Some more transcripts cluster outside this major clade, grouping with mollusc and fish sequences. The placement of a single transcript deeply nested within hexapod sequences seems to be an artifact due to its very short sequence length. Mapping of reads shows that CAP transcripts belong to the second most abundant toxin class within the deeply sequenced venom gland library of *G. dibranchiata* (Supplementary Figure S1). The biological function of this venom protein family remains to be clarified for *Glycera*.

Chitinase

Chitinases are part of the glycoside hydrolase 18 family and can be found in archaeans, bacteria and eukaryotes (Funkhouser and Aronson 2007). These proteins specifically

hydrolyse glycosidic linkages in chitin. Chitinase activity is known for several investigated venoms, and chitinases have been found to be expressed in the venom glands of cephalopods and arthropods (Fernandes-Pedrosa et al. 2008; Fry et al. 2009; von Reumont et al. 2014). We found several chitinase paralogs in the venom gland transcriptome of *Glycera dibranchiata*, which together form a monophyletic group (Supplementary Figure S9). We included one chitinase paralog from the polychaete *Capitella teleta* in our analysis. Although several more copies can be found in its genome none of these cluster between the glycerid copies (data not shown). It can be speculated that chitinase activity in the venom might assist in dealing with arthropod prey since chitin is the main component of the exoskeleton of arthropods. Quite fittingly, amphipods have been identified as a glycerid prey in both feeding experiments (Ockelmann and Vahl 1970) and glycerid gut content inspection (Retiere 1967; Michel 1970).

Cystatin

Cystatins are functional inhibitors of cysteine proteases that can protect host tissue from proteolytic digestion (Bobek and Levine 1992). Cystatins have been found as part of several venoms, e.g., in toxicoferans and insects (Fry et al. 2009). We found cystatin contigs in *G. tridactyla* and *G. fallax,* with identical contigs present in the venom gland and body tissue libraries of *G. tridactyla*. The *Glycera* sequences form a clade in the cystatin tree (Supplementary Figure S10). Moreover, as cystatin transcripts could not be recovered in the deeply sequenced *G. dibranchiata* library it remains unclear if this protein plays a role at all in the venom.

Hyaluronidase

Hyaluronic acid is a key component of extracellular matrix and hyaluronidases are involved in the breakdown of such barriers (Fox 2013). Hyaluronidases have been detected in the venom cocktail of cephalopods, stonefish, arthropods and toxicoferans (Fry et al. 2009). Vonk et al. (2013) demonstrated that hyaluronidases did not evolve under positive selection in the king cobra genome, while also showing lower levels of expression than key components of its venom. They hypothesized that this corresponded with hyaluronidase's ancillary role as a spreading factor that facilitates the actions of specific venom components. We found two hyaluronidase paralogs expressed in the venom gland of *Glycera dibranchiata*, which group together in a clade in the phylogenetic tree of hyaluronidase (Supplementary Figure S11). As for snakes, it is not unimaginable that hyaluronidases in *Glycera* may play a role in tissue disintegration, facilitating the spread of toxins. But evidence remains to be found to support a primary role of hyaluronidases in glycerid venom toxicity.

Kazal inhibitors including turripeptide-like toxin

Kazal serine protease inhibitors are characterized by the possession of Kazal domains, which show six cysteine residues pairing as three disulfide bridges (Laskowski and Kato 1980). Kazal inhibitors are known to inhibit chymotrypsin, elastase, plasmin, proteinase K, subtilisin, trypsin, and thrombin. Moreover, bacteriostatic activity has been reported for Kazal inhibitors of different organisms (Kim et al. 2013). Kazal-type inhibitors have been reported from the venom of insects, snakes, and the oral secretions of hematophagous bats and leeches (De Graaf et al. 2010; Durban et al. 2011). We found

several transcripts including Kazal domains expressed in the venom gland of all three investigated *Glycera* species. Kazal transcripts are relatively highly expressed in the venom glands of both *G. tridactyla* and *G. fallax* (22% and 32%, respectively, of the total number of transcripts) (Supplementary Figure S1). Kazal transcripts were also highly expressed in the *G. tridactyla* body tissue library, and some Kazal transcripts are expressed both in the body tissue library and the venom gland library. The *Glycera* transcripts vary enormously in length, featuring different numbers of Kazal domain repeats. This made aligning the sequences difficult. Consequently, there is very little robustly supported structure in the Kazal tree (Supplementary Figure S12).

Close inspection of our Kazal alignment combined with additional BLAST analyses (data not shown), revealed sequence similarity of two *G. dibranchiata* sequences (40507_minus2 and 71773minus3) to turripeptides. Turripeptides are secreted peptides expressed in the venom ducts of turrids, which are venomous toxoglossate molluscs related to cone snails and auger snails (Watkins et al. 2006). Supplementary Figure S13 shows a structural alignment of putative *Glycera* and turrid turripeptides. First characterized in the genus *Gemmula* by combined transcriptomic and proteomic analyses (Heralde III et al. 2008), turripeptides of the Pg-superfamily are short peptides of about 70 AA residues, containing a Kazal-like domain displaying a conserved cysteine pattern (C-C-C-C-C-C), which is seen also in the cone snail P-like superfamily conopeptides (Watkins, Hillyard, Olivera 2006; Cabang et al. 2011; Olivera et al. 2012). Although sharing a conserved cysteine pattern similar to P-like conotoxins, turripeptides are currently considered to be a unique superfamily broadly expressed across Turridae

(Olivera et al. 2012). Pg-like turripeptides have strikingly conserved N-terminal signal peptides, fully conserved cysteine residues, and they show accelerated evolution of non-cysteine residues within the C-terminal mature peptide. This is an evolutionary signature of toxoglossan venom peptide gene superfamilies in general (Olivera et al. 2012).

Expression of turripeptide-like sequences in *G. dibranchiata* comprises only a tiny fraction of overall reads expressed, 627 reads and 306 reads for contigs 40507_minus2 and 71773_minus3, respectively. These two sequences have a single Kazal domain located within the longest ORF, a feature characteristic of turripeptides. Furthermore, the signal peptide sequences of these *Glycera* transcripts share between 31.8% and 40.0% sequence identity with similarly conserved signal peptide regions of turripeptides, and they show a relatively low sequence conservation of non-cysteine residues. The two turripeptide-like transcripts for *G. dibranchiata* cluster together with high support in our phylogenetic analysis of Kazal sequences, and some distance away from the clade that contains the majority of *Glycera* Kazal sequences (Supplementary Figure S12). Based on their similarity to conotoxins, turripeptides are suspected to function as ion channel modulators (Heralde III et al. 2008). However, both proteomic and functional studies are required to confirm this both for turrids and *Glycera*.

Kunitz

Kunitz-domain proteins are a large family of protease inhibitors that were initially described by Kunitz and Northrop (1936). The peptides are usually quite short (around 60 amino acids) and stabilized by three disulfide bridges (Mourao and Schwartz 2013).

Kunitz peptides have been reported in analyses of venom composition in cnidarians, insects, chelicerates, molluscs, mammals, and snakes (Fry et al. 2009). Ticks show an especially great variety of Kunitz peptides, which have been categorized into different groups (Schwarz et al. 2014). Some members of this protein family are able to block or modulate ion channels. In blood feeding tick Kunitz peptides are involved in the inhibition of blood coagulation and the regulation of host blood supply (Mans and Neitz 2004a; Dai et al. 2012). We found Kunitz transcripts in the venom gland transcriptome of *G. dibranchiata*, as well as the body transcriptome of *G. tridactyla* (Supplementary Figure S1). The *Glycera* peptides show the highly conserved motif of six cysteine residues typical of this family. Phylogenetic analyses reveal no clear pattern of the evolution of this protein family, with *Glycera* sequences clustering with different taxa with low support (Supplementary Figure S14). It has been demonstrated for the green Mamba (*Dendroaspis angusticeps*) that a Kunitz-type venom peptide called calcicludine blocks different Ca^{2+} channels of L-, P-, and N-type (Schweitz et al. 1994; Stotz et al. 2000). The latter type of calcium channel is known to be activated specifically by glycerotoxin (Meunier et al. 2002). However, without functional studies a putative role of Kunitz peptides in the glycerid venom gland remains uncertain.

C-type lectin

C-type lectin-like domains are found in a large group of extracellular proteins that are characterized by a double-loop, which is in-turn stabilized by two highly conserved disulfide bridges located at the bases of the loops (Zelensky and Gready 2005). C-type lectins have been found in the venom of many taxa, including cnidarians, snakes, insects,

and stonefish. The effects of venom lectins is diverse, including anticoagulant activity and myotoxic effects (Fry et al. 2009). Moreover, lectin domains might be associated with other toxins and involved in the mediation of their activity. Several potent neurotoxins bear lectin-like domains, as is known for ricin or botolinum toxin (Olsnes et al. 1974; Lacy et al. 1998). We found c-type lectin domain transcripts expressed in the venom glands of all investigated *Glycera* species and especially the *G. dibranchiata* library revealed a greater diversity of paralogs that group in various places in our phylogenetic tree, although the tree has very few supported clades (Supplementary Figure S15). A putative synergy of the effect of glycerotoxin and c-type lectins can be hypothesized. It might be possible that the carbohydrate binding lectin domain binds to oligosaccharides of the presynaptic membrane and is used for guiding glycerotoxin.

Lipocalin

Lipocalins belong to the large calycin 'structural superfamily' including fatty-acid binding proteins (FABPs) and metalloproteinase inhibitors (Mans and Neitz 2004b). They display a wide range of physiological roles that include retinol transport, olfaction, pheromone transport, prostaglandin synthesis, metabolism, and cellular homoeostasis and are typically involved in the binding of small hydrophobic ligands (Flower 1996; Flower et al. 2000; Gutierrez et al. 2000; Pugalenthi et al. 2010).

In terms of their role as proteinaceous venom components, lipocalins have been recruited into a wide range of animal venoms and oral secretions. To date the most common species investigated for the recruitment of lipocalin protein members as active toxin components are species of haematophagous arthropods such as hard/soft ticks,

triatomines (kissing bugs) and blood feeding dipterans (Fry et al. 2009), but also caterpillars of the non-haematophagous silk moth *Lonomia obliqua* (Reis et al. 2006). Furthermore, lipocalins have also been described as active toxins within vertebrate oral secretions, present within Chiroptera (*Desmodus* allergen-related lipocalins, also known as 'Desmallipins') and snakes (Fry et al. 2012; Wei and Chen 2012; Low et al. 2013). Interestingly, distant lipocalin homologs have also been identified in gram-negative bacteria (e.g. *Escherichia. coli*), while a putative homolog has been further suggested in the slime mold *Dictyostelium* (Bishop 2000; Gutierrez, Ganfornina, Sanchez 2000). Recurrent recruitment of lipocalin family members into venom cocktails/salivary secretions is best highlighted when considering their role as antihemostatic mediators in haematophagous arthropods (Waxman and Connolly 1993).

Lipocalins only make up a small fraction of the overall toxin transcripts expressed across our individual NGS libraries. Three contigs, comprising less than 1% of total toxin transcripts overall were identified within *G. dibranchiata*. A single lipocalin contig was identified for *G. fallax,* but in contrast to *G. dibranchiata* this contig represents the second most abundantly expressed transcript, comprising a total of 21% of overall expressed venom gland transcripts in this species. Contigs generated from *G. dibranchiata* and *G. fallax* venom glands lack any sequence specific functional motifs, a feature of the majority of calycin members, which are typically identified on the basis of secondary structure (Flower, North, Sansom 2000). These contigs, however, do contain two significant structurally conserved amino acid residues implicated in maintaining the signature calycin β-barrel structure, composed of antiparallel β -sheets (FABP contain 10 Beta-barrels) that form the enclosed ligand binding site (Flower et al. 1993; Flower,

North, Sansom 2000). Three out of four *Glycera* contigs (*fallax*: (1505_plus2); *dibranchiata*: (39599_plus1, 39596_plus1) group together in a clade (Supplementary Figure S16) and contain the amino acid tryptophan (Trp/W) at position 49 of the lipocalin MSA, but this is absent from the *G. dibranchiata* contig 79668_minus1 due to a partially truncated sequence which begins at position 52 of the MSA. This latter contig is the only *Glycera* contig that contains the second conserved residue Arginine (Arg/R) at position 264 of the MSA. From our data we infer that in *Glycera* lipocalins have not undergone a substantial expansion. This fact, and the low expression values obtained, may suggest that lipocalin toxin recruitment occurred recently in the evolution of the *Glycera* venom complement and/or lipocalins do not confer substantial toxicity during envenomation of prey or during defense from predation.

Metalloproteinase M12

Metalloproteinases of the M12 family have been widely recruited into animal venoms and hematophagous secretions, including in arthropods, cephalopods, cnidarians, toxicoferans, and the platypus (Fry et al. 2009; Wong et al. 2012). Metalloproteinases are involved in many cellular processes, and they can have various effects as venom components, including skin damage, edema, and inflammation. Snake venom metalloproteinases of the M12 family can degrade extracellular matrix, and prevent blood clot formation, while spider variants can be haemorrhagic. This toxin family has diversified substantially in bloodworms. About three dozen different M12 contigs are expressed in the venom glands of *G. dibranchiata*, representing both the astacin (M12a) and reprolysin (M12b; best known for housing the snake venom metalloproteinases)

subfamilies. Most *Glycera* sequences have the conserved sequence motif His-Glu-x-x-His-x-x-Gly-x-x-His, with Glu representing the catalytic site, and with the three His residues functioning as ligands for zinc atoms needed for the enzyme to be active. The *G. tridactyla* body library also contains two M12 transcripts with these active sites. M12 metalloproteases are the fourth highest expressed toxin in the venom glands of *G. dibranchiata*. The strong swelling and inflammation that can occur after a *Glycera* bite (Klawe and Dickie 1957) are consistent with the expression of M12 metalloproteinases in *Glycera* venom glands. In our tree (Supplementary Figure S17) the bloodworm astacin-like sequences group together, while the reprolysin-like sequences are found in several clusters elsewhere in the tree.

Peptidase S1

The S1 family of serine proteases is the largest peptidase family. It is among the most widely recruited venom toxins, and is found expressed in the venom glands of a broad range of taxa, including molluscs, insects, mammals, reptiles, centipedes, and chelicerates (Aminetzach et al. 2009; Fry et al. 2009; Whittington et al. 2010; Liu et al. 2012; Ruder et al. 2013). S1 peptidases are both diverse and highly expressed in the venom glands of several taxa, for instance the crural glands of male platypuses, the posterior venom glands of cephalopods, and especially the venom glands of the remipede crustacean *Speleonectes tulumensis* (Whittington et al. 2010; Ruder et al. 2013; von Reumont et al. 2014). Venom serine proteases have a variety of activities, such as the prevention of blood coagulation, and the causing of vasodilation, smooth muscle contraction, pain, immunosuppression and inflammation. S1 peptidases represent the most diverse putative toxin expressed of

the venom glands of all three species of *Glycera*. Moreover, S1 peptidases are the most abundantly expressed putative toxins in the venom glands of both *G. dibranchiata* and *G. tridactyla*—accounting for 29% and 33% of the total number of toxin transcripts, respectively—and the second most abundantly expressed toxin in *G. fallax* (21% of the total number of transcripts). The phylogenetic distribution of the transcripts in the three *Glycera* species (Supplementary Figure S18) suggests that the venom glands of their last common ancestor already expressed a substantial diversity of S1 peptidases. This toxin family seems to have diversified especially in *G. dibranchiata*, although the discrepancy in the size of the transcriptome libraries makes it difficult to establish this with certainty. Most S1 transcripts probably code for active enzymes, as they contain the catalytic triad His-Asp-Ser that is characteristic for this peptidase family.

Peptidase S10 (venom serine carboxypeptidase)

Members of the S10 serine peptidase family have not been widely recruited into venoms. As far as we are aware they have only been identified in the venoms of hymenopterans and remipede crustaceans. The majority of taxa included in our peptidase S10 tree are either non-venomous, or the sequences from venomous or hematophagous organisms are derived from non-venom gland tissues or whole animals including venom glands, leaving the tissue source of the sequences undetermined. Only the S10 peptidases from the honeybee and remipede crustaceans are specifically known to be expressed in the animal's venom glands (Li et al. 2013; von Reumont et al. 2014). The venom glands of both *G. dibranchiata* and *G. tridactyla* express a high diversity of peptidase S10 transcripts. They are the fourth most diverse putative toxin transcripts in the venom

glands of *G. dibranchiata*, but they are not highly expressed, together only representing about 3% of the total number of putative toxin transcripts. All the *Glycera* transcripts that are sufficiently long have the catalytic triad (Ser-Asp-His) characteristic of this peptidase family. The biological function of this putative toxin is unknown, although the S10 peptidases in honeybee venom are recognized allergens. Members of this peptidase family are only active in an acidic environment. Although the pH of *Glycera* is unknown, if it is acidic peptidase S10 might function by modifying other venom components before the venom is injected into prey. The clustering of the vast majority of *Glycera* sequences in one large clade (Supplementary Figure S19) suggests a lineage specific radiation of this venom toxin.

Serpin

Serpins irreversibly inhibit serine and cysteine endopeptidases. Although some authors label Kunitz and Kazal type protease inhibitors as families or types of serpins (Min et al. 2010; Kvist et al. 2014), strictly speaking this is incorrect. Serpins are classified as MEROPS family I4, Kunitz as family I2, and Kazal as family I1 (http://merops.sanger.ac.uk/inhibitors/index.shtml). Kunitz and Kazal strictly inhibit serine proteases, while serpins can also inhibit cysteine proteases. Serpins have been recruited into the salivary secretions of blood-feeders and the venoms of several taxa, including the crural glands of male platypus (Wong et al. 2012), the venom glands of remipede crustaceans (von Reumont et al. 2014), tick salivary glands (Chmelar et al. 2011), snake venom glands (Rokyta et al. 2012), mosquito salivary glands (Chagas et al. 2013), and the venom glands of parasitoid wasps (Colinet et al. 2013; Dorémus et al.

2013). In addition serpins have also been characterized from the defensive skin secretions of a frog and the defensive bristles of the *Lonomia obliqua* caterpillar (Veiga et al. 2005; Wu et al. 2011). Serpins may affect the blood pressure of envenomed organisms, as well as affect blood coagulation and disrupt the immune system. The serpins found in platypus venom, for instance, have been proposed to be able to both decrease (Wong et al. 2011) and increase blood pressure (Wong et al. 2012). The presence of serpins in the sialomes of mosquitos and ticks suggests that they may play a role in inhibiting blood coagulation, while the serpins in the venom of parasitoid wasps can inhibit the phenoloxidase cascade of the host, thereby disrupting immune system function (Colinet et al. 2013).

Serpin transcripts are expressed at a low level in the venom glands of both *G. dibranchiata* and *G. tridactyla*, and they are also expressed in the non-venom gland tissue of the latter (Supplementary Figure S1). All bloodworm serpin sequences fall in one clade, albeit a very weakly supported one (Supplementary Figure S20). The deep structure of the serpin phylogeny is very poorly supported throughout. The fact that seven different serpin transcript are expressed in *G. dibranchiata* suggests that this putative toxin may play an important role, although it is unclear what that would be.

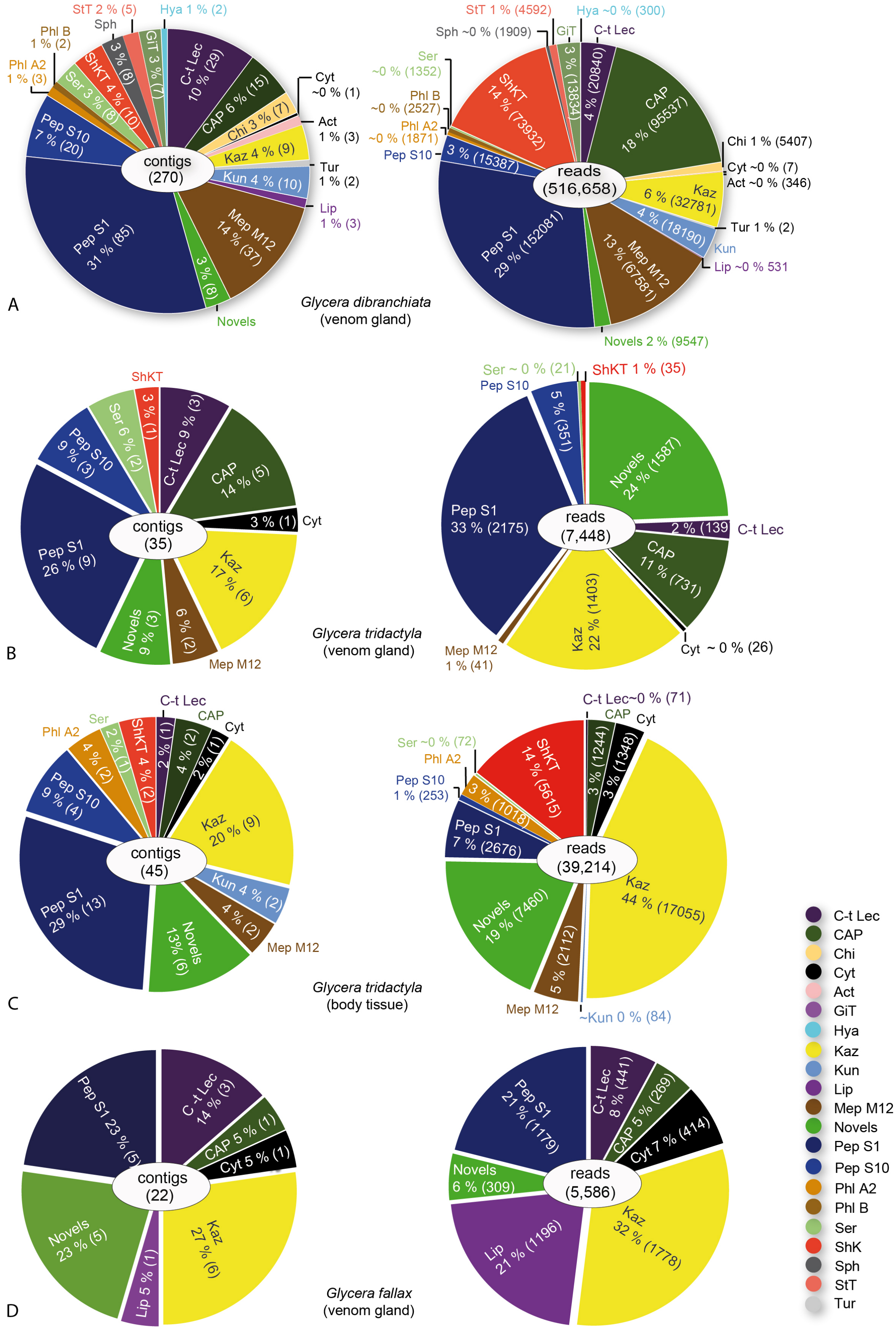
A
Glycera dibranchiata
(venom gland)
contigs
(270)
StT 2 % (5)
Hya 1 % (2)
Sph
Phl B
1 % (2)
Phl A2
1 % (3)
GiT 3 % (7)
3 % (8)
ShKT 4 % (10)
Ser 3 % (8)
Pep S10
7 % (20)
C-t Lec
10 % (29)
CAP 6 % (15)
Chi 3 % (7)
Cyt
~0 % (1)
Act
1 % (3)
Kaz 4 % (9)
Tur
1 % (2)
Kun 4 % (10)
Lip
1 % (3)
Mep M12
14 % (37)
3 % (8)
Novels
Pep S1
31 % (85)
reads
(516,658)
StT 1 % (4592)
Hya ~0 % (300)
Sph ~0 % (1909)
GiT
C-t Lec
3 % (13834)
4 % (20840)
Ser
~0 % (1352)
Phl B
~0 % (2527)
Phl A2
~0 % (1871)
Pep S10
3 % (15387)
ShKT
14 % (73932)
CAP
18 % (95537)
Chi 1 % (5407)
Cyt ~0 % (7)
Act ~0 % (346)
Kaz
6 % (32781)
4 % (18190)
Tur 1 % (2)
Kun
Lip ~0 % 531
Mep M12
13 % (67581)
Pep S1
29 % (152081)
Novels 2 % (9547)
B
Glycera tridactyla
(venom gland)
contigs
(35)
ShKT
3 % (1)
Ser 6 % (2)
Pep S10
9 % (3)
C-t Lec 9 % (3)
CAP
14 % (5)
3 % (1)
Cyt
Kaz
17 % (6)
6 % (2)
Mep M12
Novels
9 % (3)
Pep S1
26 % (9)
reads
(7,448)
Ser ~ 0 % (21)
ShKT 1 % (35)
Pep S10
5 % (351)
Novels
24 % (1587)
2 % (139)
C-t Lec
CAP
11 % (731)
Cyt ~ 0 % (26)
Kaz
22 % (1403)
Mep M12
1 % (41)
Pep S1
33 % (2175)
C
Glycera tridactyla
(body tissue)
contigs
(45)
C-t Lec
CAP
Cyt
Ser
Phl A2
2 % (1)
4 % (2)
2 % (1)
ShKT 4 % (2)
2 % (1)
4 % (2)
Pep S10
9 % (4)
Kaz
20 % (9)
Kun 4 % (2)
4 % (2)
Mep M12
Novels
13% (6)
Pep S1
29 % (13)
reads
(39,214)
C-t Lec~0 % (71)
CAP
Cyt
Ser ~0 % (72)
Phl A2
Pep S10
1 % (253)
3 % (1018)
ShKT
14 % (5615)
3 % (1244)
3 % (1348)
Pep S1
7 % (2676)
Kaz
44 % (17055)
Novels
19 % (7460)
5 % (2112)
Mep M12
~Kun 0 % (84)
D
Glycera fallax
(venom gland)
contigs
(22)
Pep S1 23 % (5)
C-t Lec
14 % (3)
CAP 5 % (1)
Cyt 5 % (1)
Kaz
27 % (6)
Lip 5 % (1)
Novels
23 % (5)
reads
(5,586)
Pep S1
21 % (1179)
C-t Lec
8 % (441)
CAP 5 % (269)
Cyt 7 % (414)
Kaz
32 % (1778)
Lip
21 % (1196)
Novels
6 % (309)
C-t Lec
CAP
Chi
Cyt
Act
GiT
Hya
Kaz
Kun
Lip
Mep M12
Novels
Pep S1
Pep S10
Phl A2
Phl B
Ser
ShK
Sph
StT
Tur

Supplementary figure S1. Transcriptomic profiles of toxin genes expressed in all four libraries. (A) Contig diversity (left) and read abundance (right) of the different toxins expressed in the venom glands of *Glycera dibranchiata*. (B) Contig diversity (left) and read abundance (right) of the different toxins expressed in the venom glands of *Glycera tridactyla*. (C) Contig diversity (left) and read abundance (right) of the different toxins expressed in the body tissue of *Glycera tridactyla*. (D) Contig diversity (left) and read abundance (right) of the different toxins expressed in the venom glands of *Glycera fallax*. Relative contig diversity and relative abundance of reads are expressed as percentages followed by the total number of contigs in parentheses.

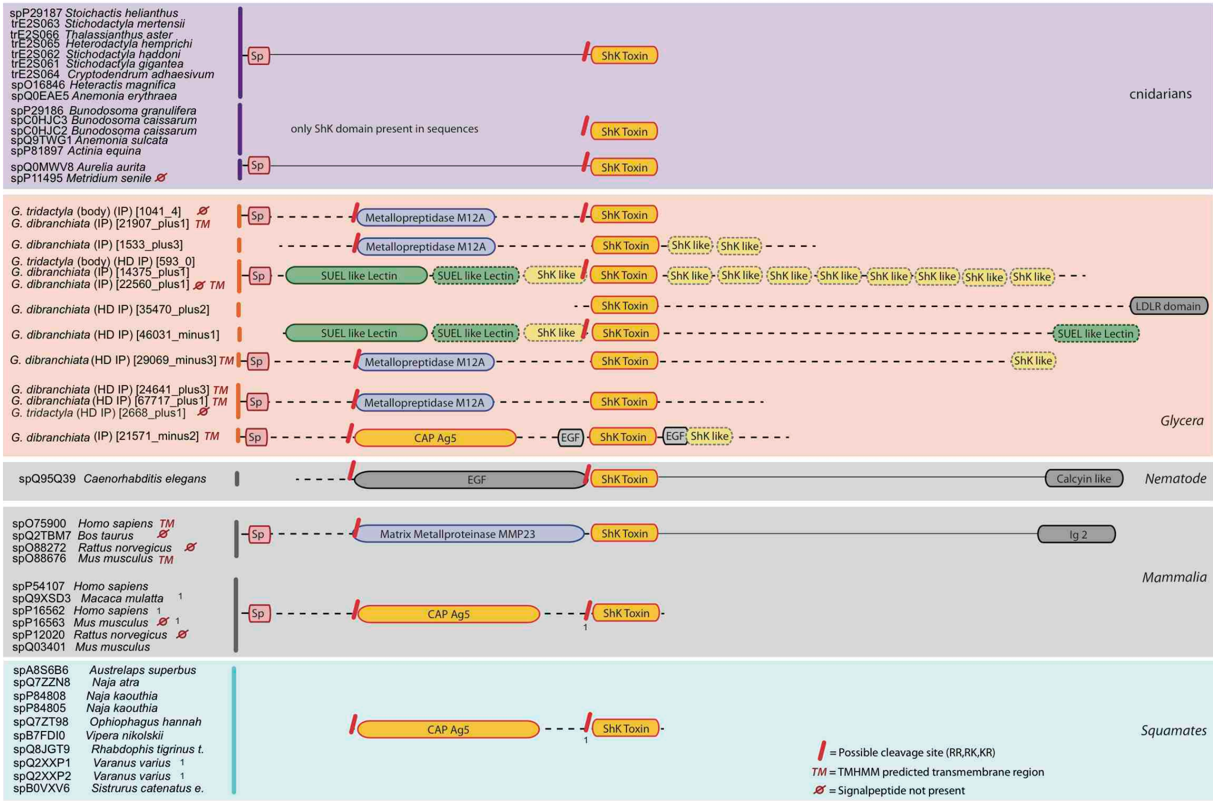

Supplementary figure S2. Overview of the domain organization of contigs with ShKT domains. Geneious R6 was used to predict domains via InterProScan. Transmembrane regions are indicated by a red TM label. Signal peptides are indicated by pink boxes. Different transcripts with similar domain patterns are grouped, marking transcripts without signal peptide by a red "ø". Potential cleavage sites (RR, RK, KR) between domains are marked by a red slash. Transcripts with potential cleavage sites are labeled by a ‘1’.

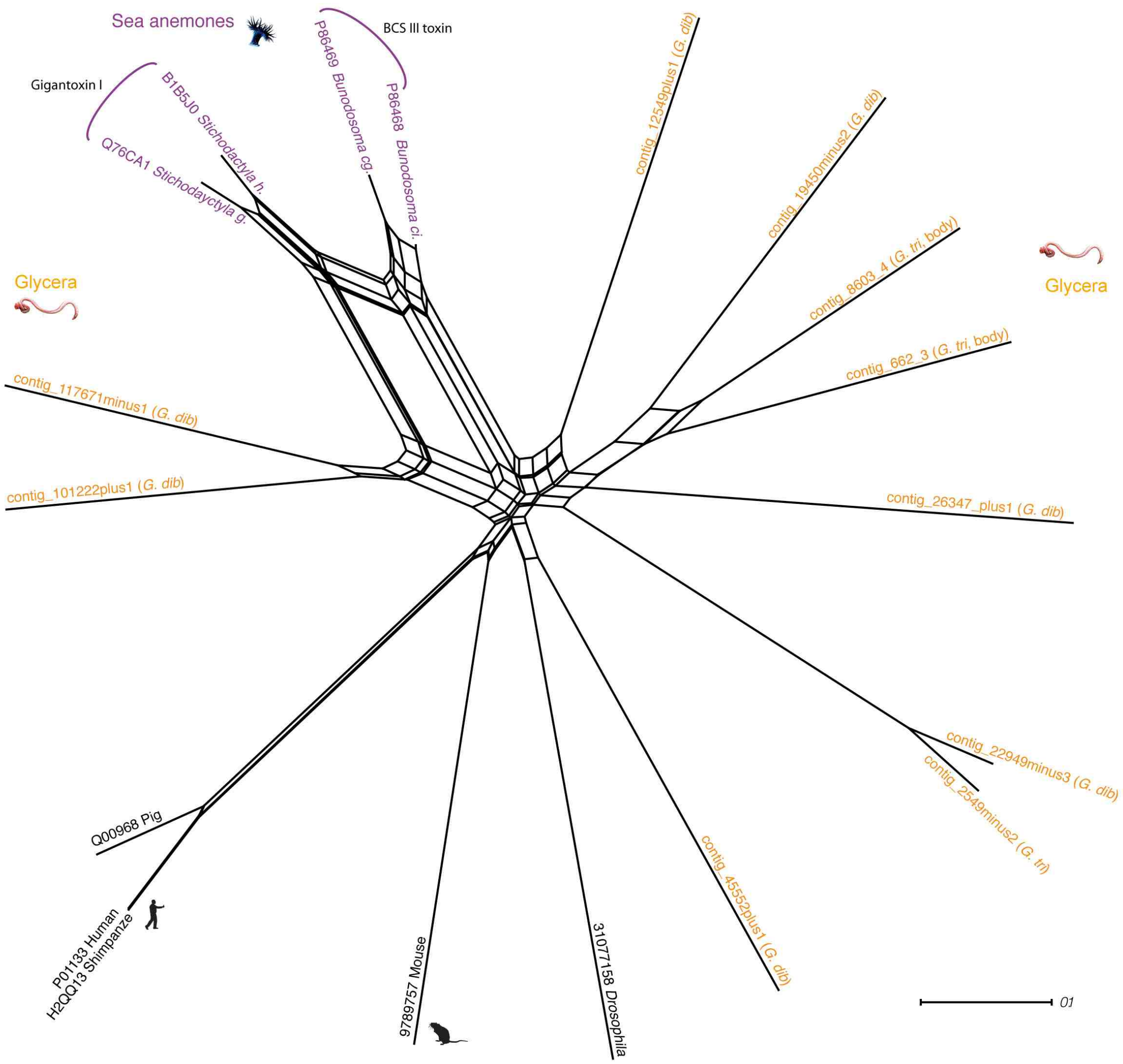

Supplementary figure S3. Neighbour joining network of gigantoxin I sequences produced by Splitstree (Huson and Bryant 2006). *Glycera* sequences are indicated by orange branches, and sea anemone sequences by purple branches. Parallel edges indicate support for the same splits, while non-parallel edges indicate conflicting splits.

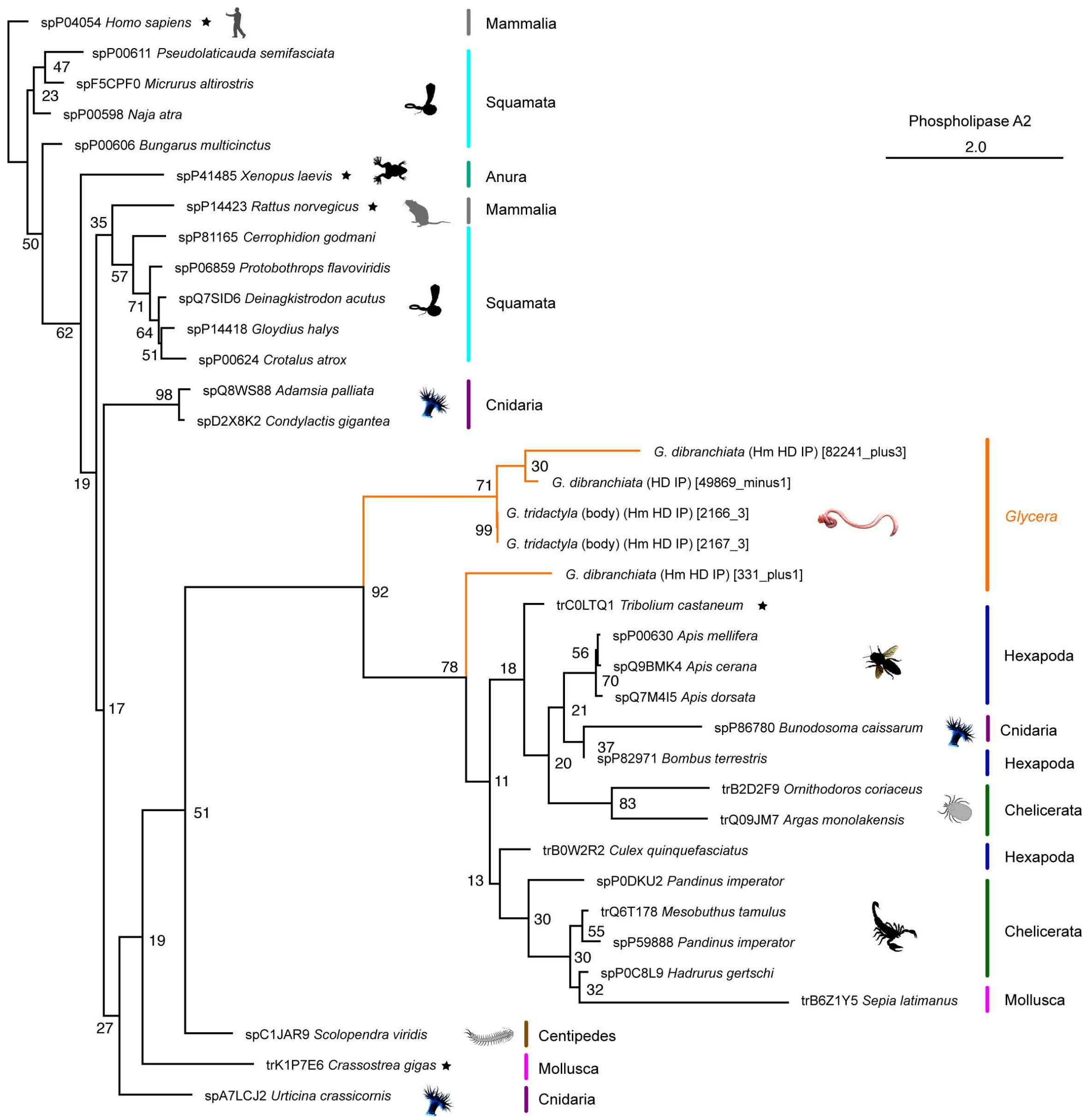

Supplementary figure S4. Phylogenetic tree of phospholipase A_2 sequences. Phylogenetic reconstruction was performed with RAxML-PTHREADS-SSE3 v7.4.2 (Stamatakis and Alachiotis 2010) by using the -f a and PROTGAMMAIWAG option and calculating bootstrap support on 1,000 pseudoreplicates. Bootstrap support values are given for all nodes. See the legend of Fig. 3 for further information.

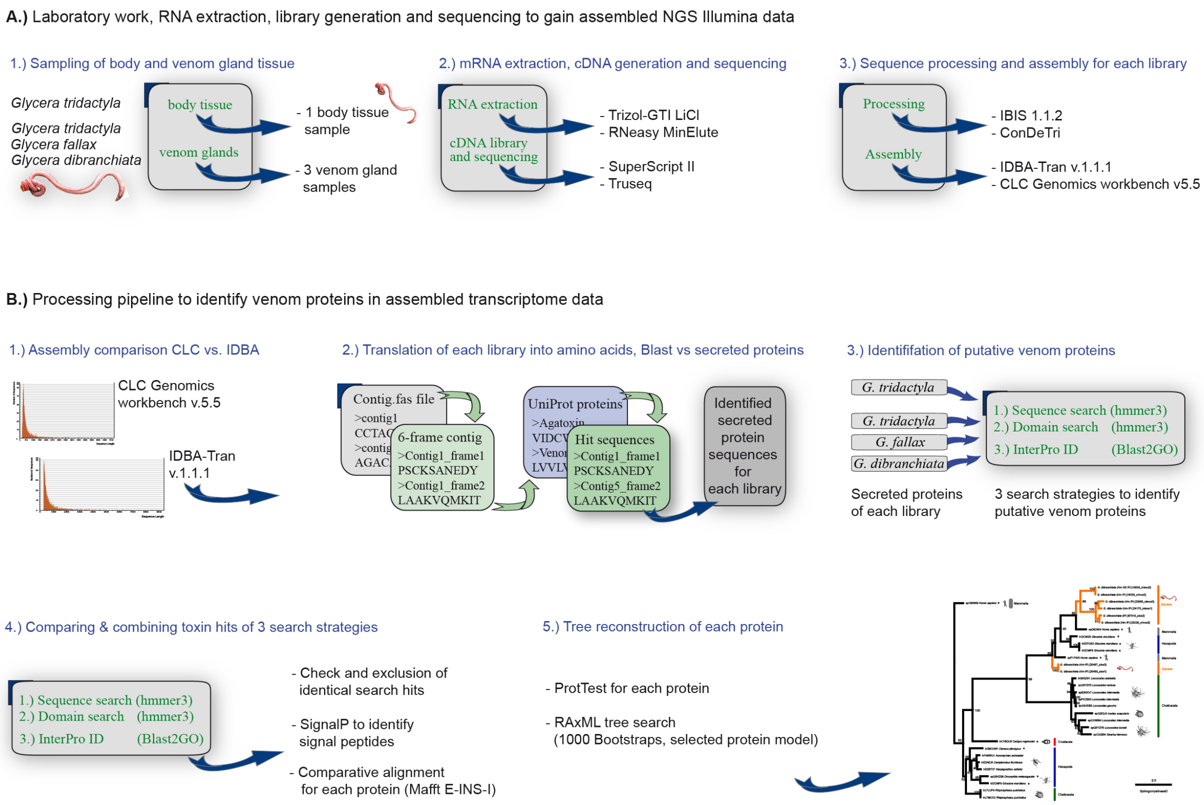

Supplementary figure S5. Flow chart of our procedures to identify putative venom proteins. (A) Kits used for molecular work on dissected venom glands and body tissue, and software used to build the final library assemblies. (B) Processing pipeline used for identifying venom proteins. Scripts were used to translate contigs from two different assembly methods (IDBA-tran and CLC Genomics Workbench) into amino acids, and to search for secreted proteins (reference sequences obtained from UniProt) using BLAST. For each library three different search strategies based on InterProScan, HMMER and HMMER-domain matches were compared. Only unique non-redundant hits were finally analyzed. All contigs that matched toxins were checked for signalpeptides and aligned. Trees were reconstructed with RAxML after selecting the best fitting model using

ProtTest. For details of further analyses specific to each dataset see the respective figure legends and Supplementary table 7.

All contigs

CLC Assembler

IDBA-Tran Assembler

Glycera dibranchiata (Venom gland)

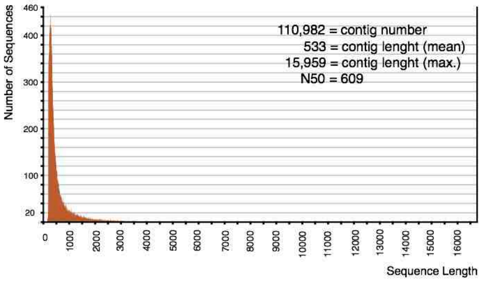

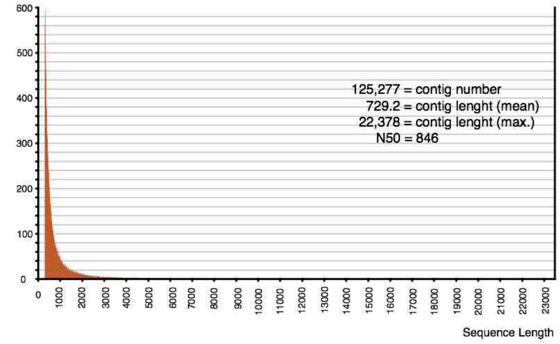

Glycera fallax (Venom gland)

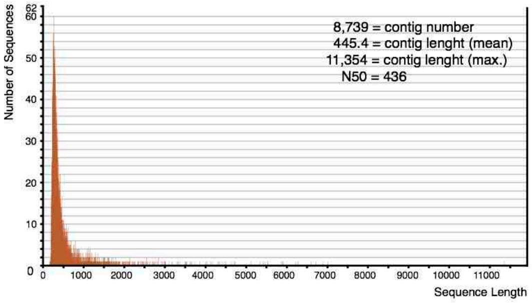

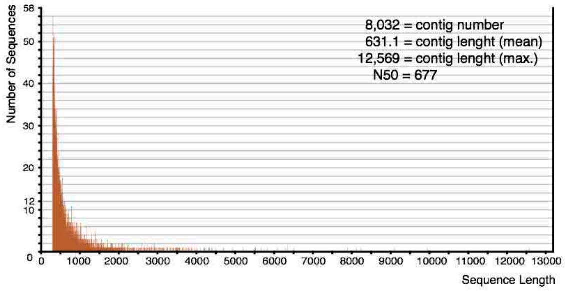

Glycera tridactyla (Venom gland)

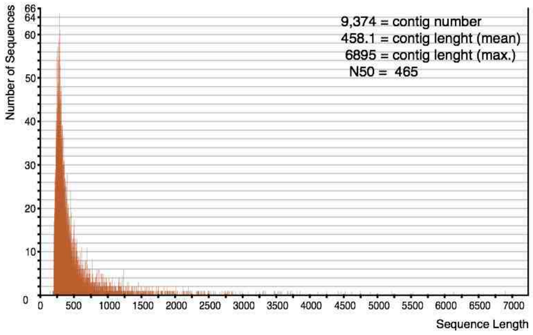

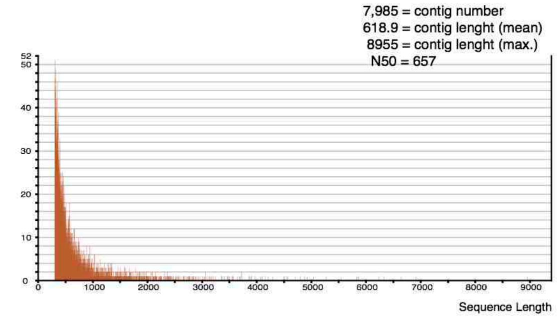

Glycera tridactyla (Body tissue)

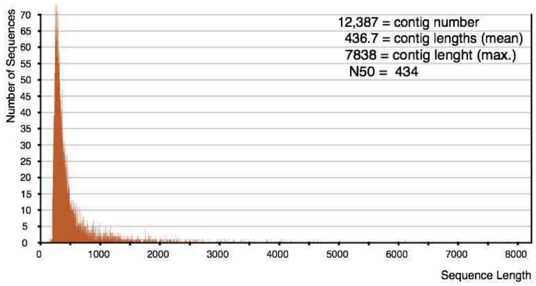

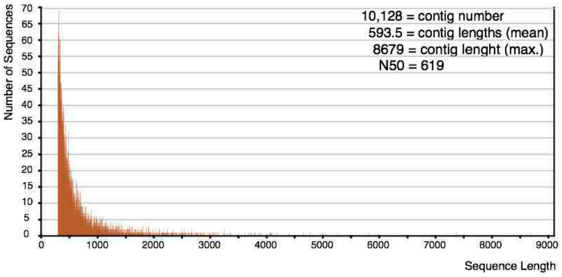

Supplementary figure S6. Assembly statistics for all four libraries for all contigs.

Contigs of secreted proteins

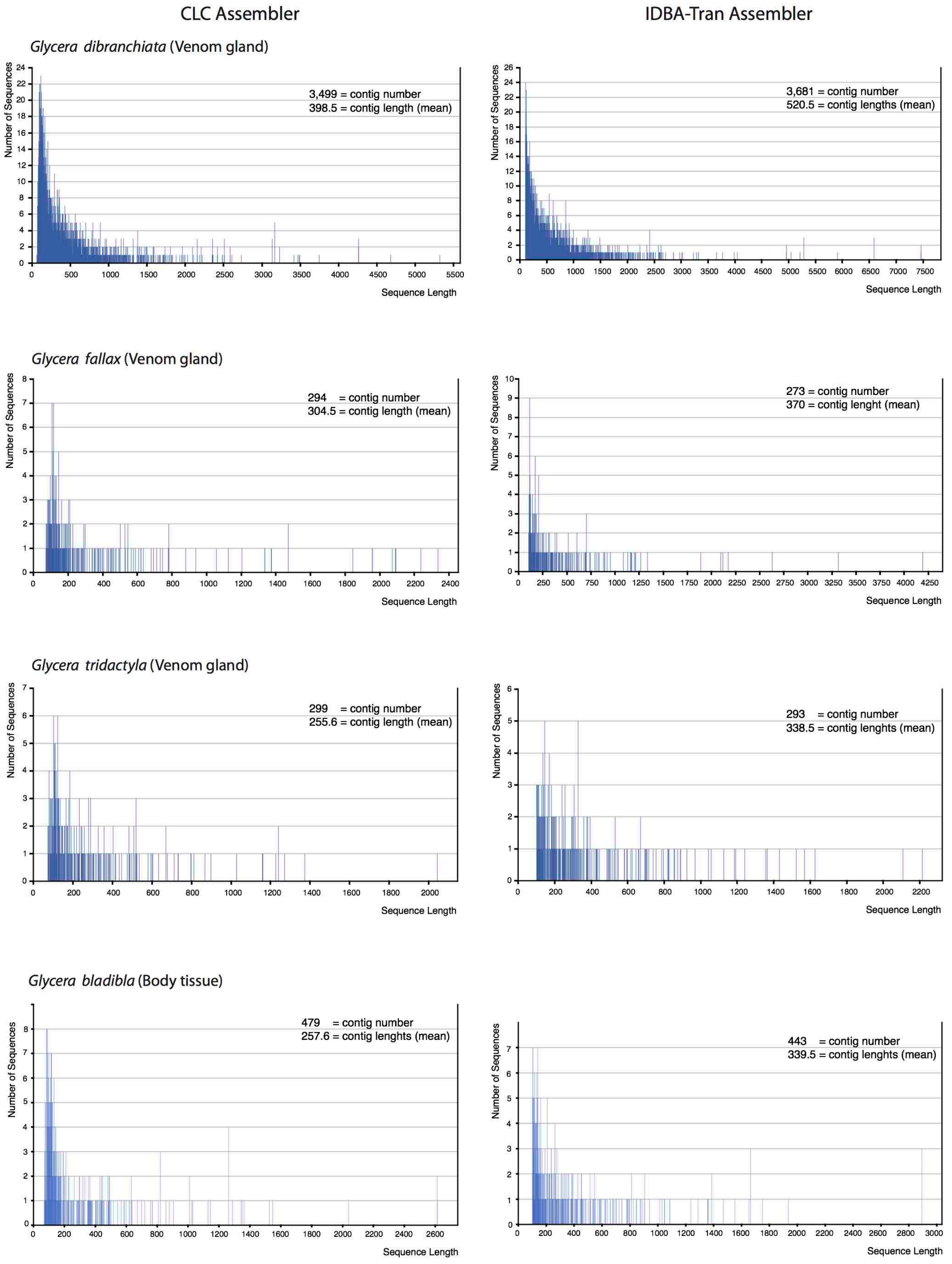

Supplementary figure S7. Assembly statistics for all four libraries for contigs of secreted proteins.

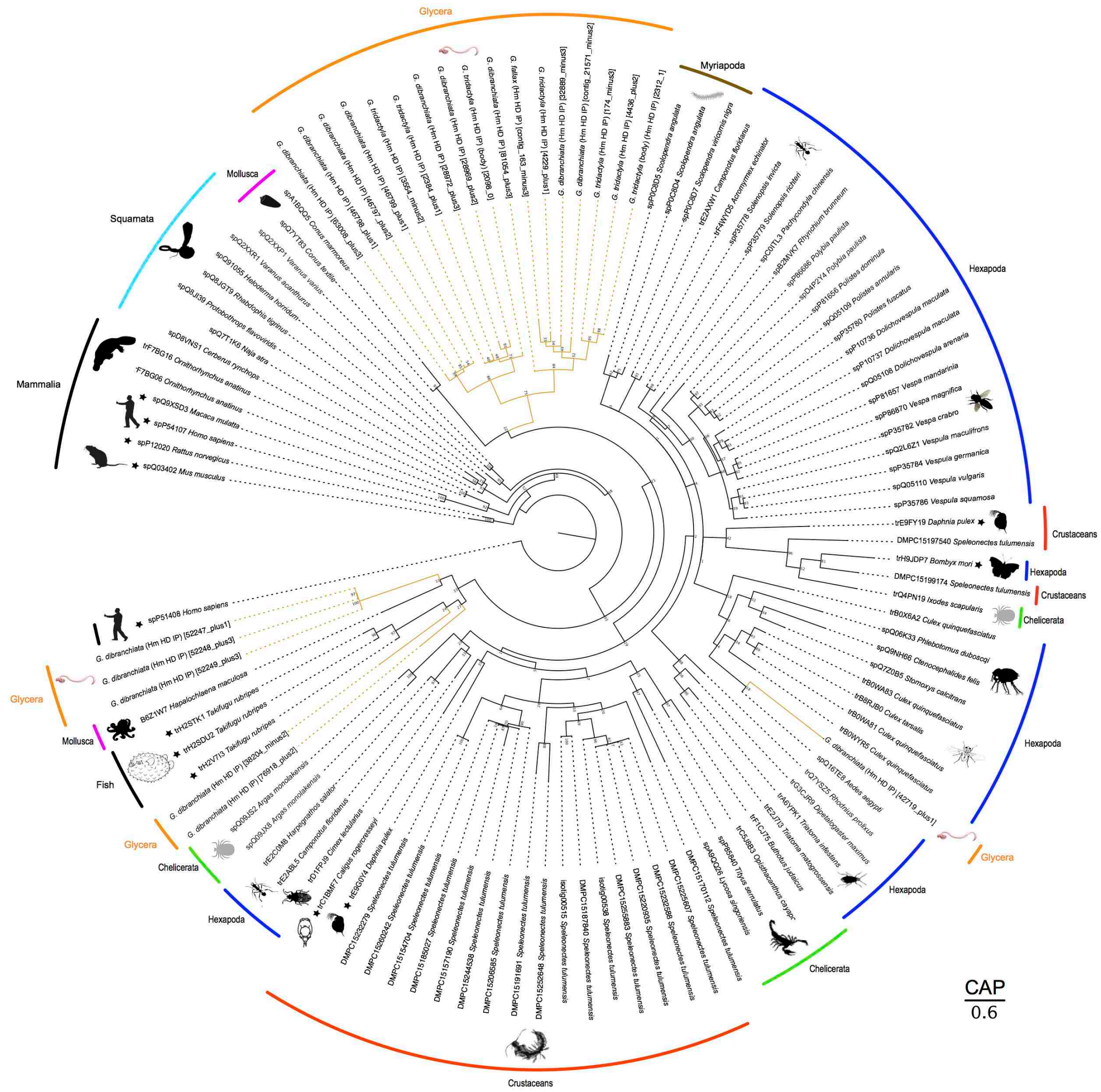

Supplementary figure S8. Phylogenetic tree of CAP sequences. Phylogenetic reconstruction was performed with RAxML-PTHREADS-SSE3 v7.4.2 (Stamatakis, Alachiotis 2010) by using the -f a and PROTGAMMAIWAG option and calculating bootstrap support on 1,000 pseudoreplicates. Bootstrap support values are given for all nodes. See the legend of Fig. 3 for further information.

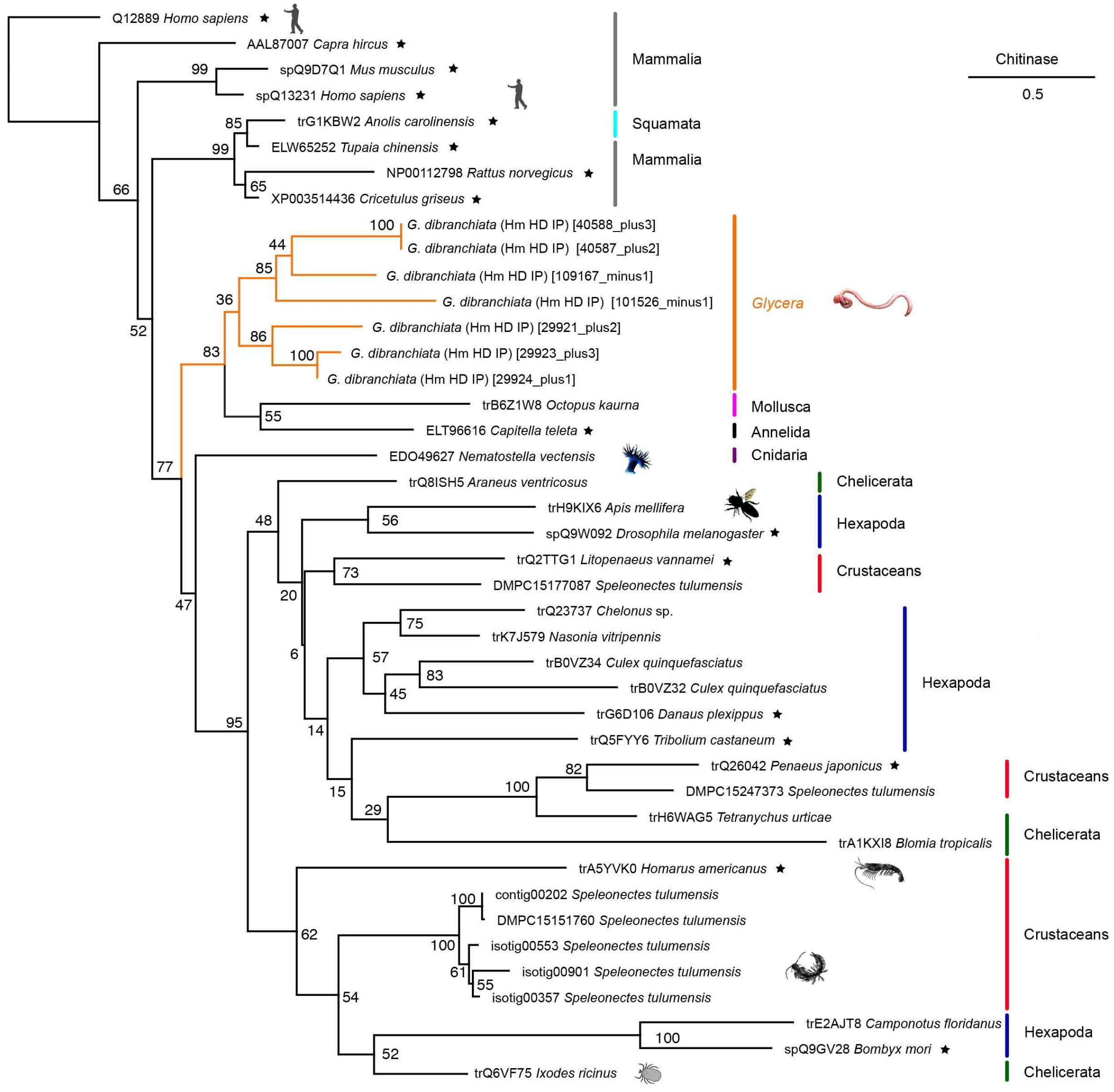

Supplementary figure S9. Phylogenetic tree of chitinase sequences. Phylogenetic reconstruction was performed with RAxML-PTHREADS-SSE3 v7.4.2 (Stamatakis, Alachiotis 2010) by using the -f a and PROTGAMMAILG option and calculating bootstrap support on 1,000 pseudoreplicates. Bootstrap support values are given for all nodes. See the legend of Fig. 3 for further information.

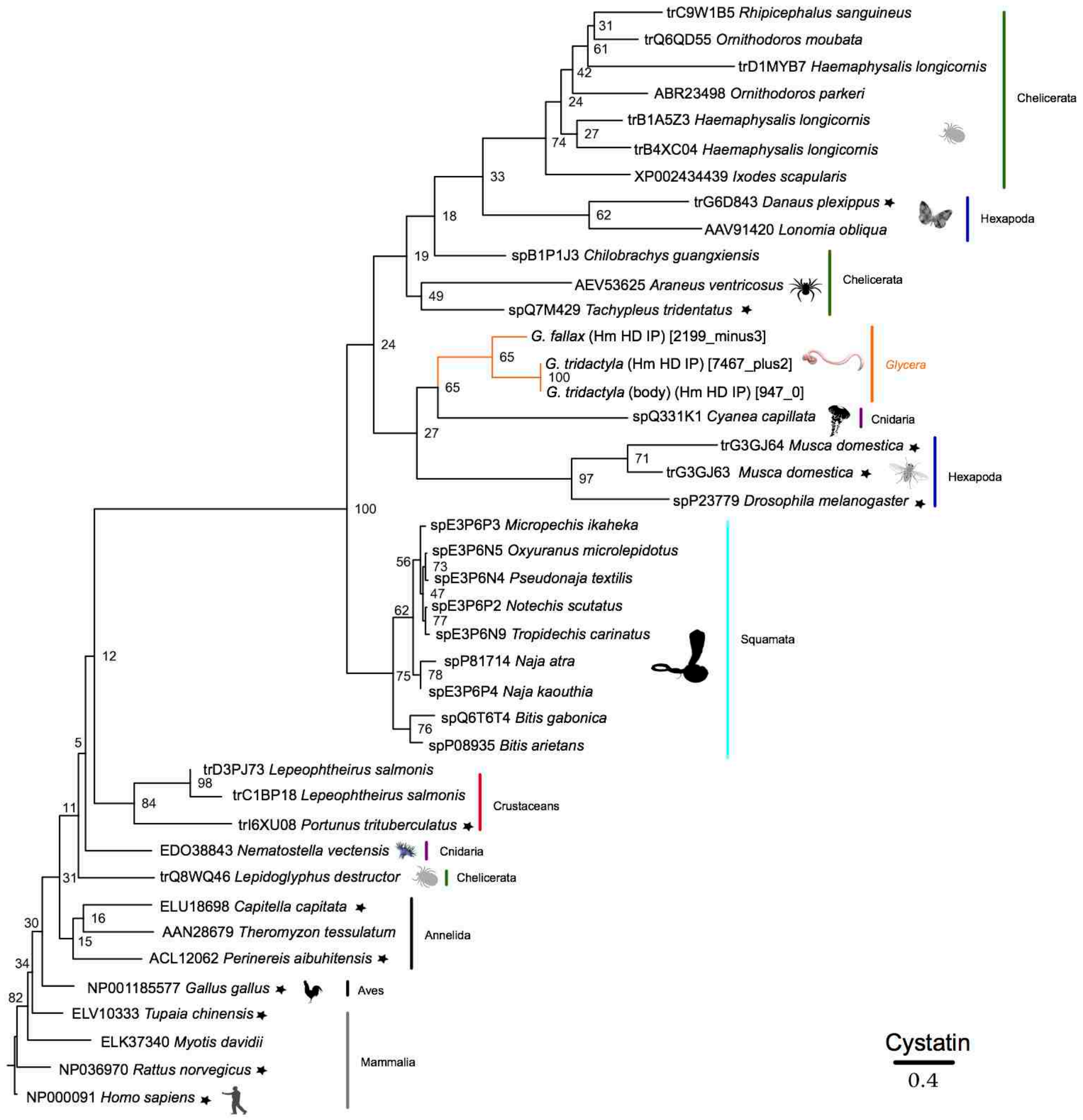

Supplementary figure S10. Phylogenetic tree of cystatin sequences. Phylogenetic reconstruction was performed with RAxML-PTHREADS-SSE3 v7.4.2 (Stamatakis, Alachiotis 2010) by using the -f a and PROTGAMMAIWAGF option and calculating bootstrap support on 1,000 pseudoreplicates. Bootstrap support values are given for all nodes. See the legend of Fig. 3 for further information.

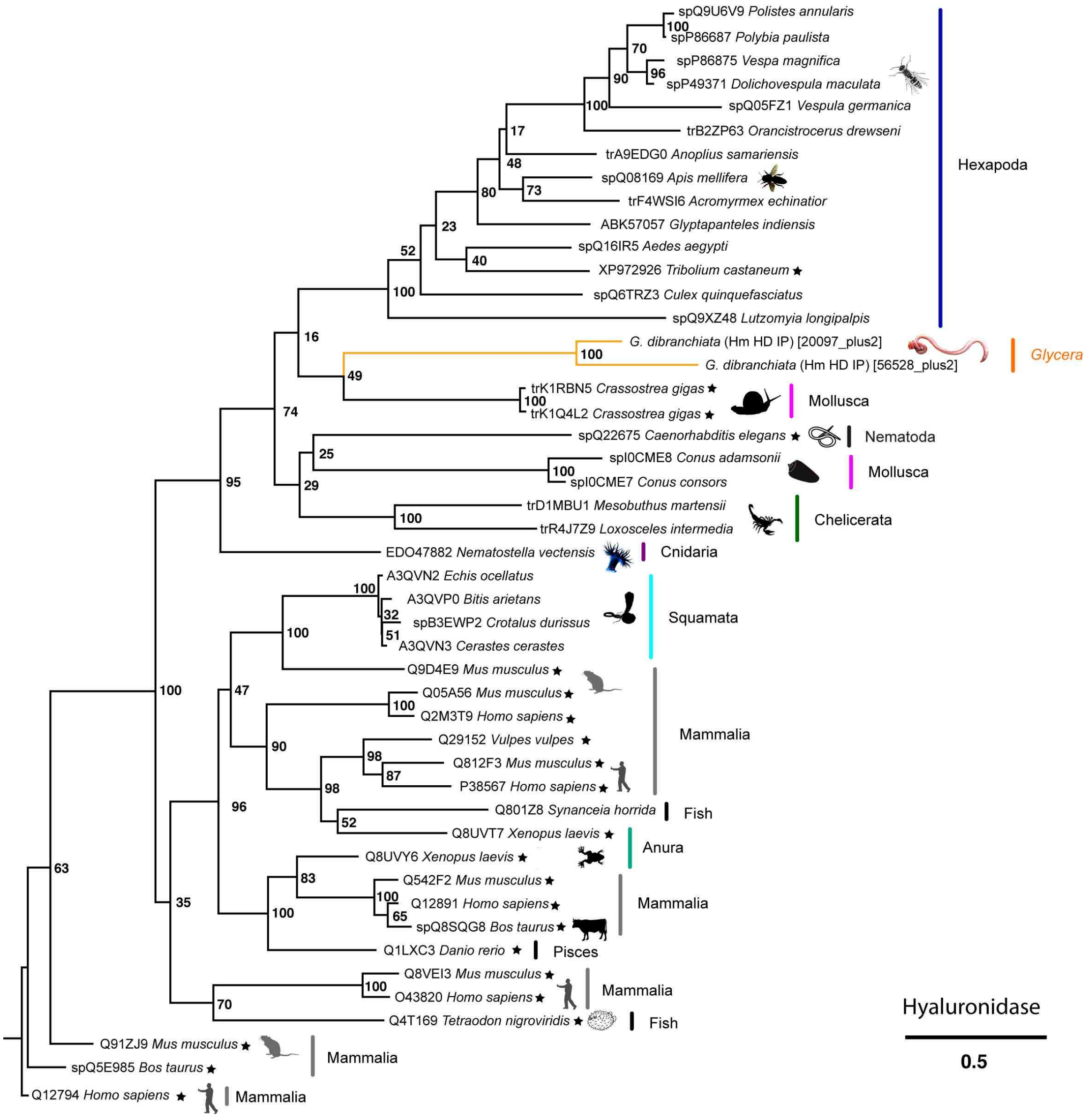

Supplementary figure S11. Phylogenetic tree of hyaluronidase sequences. Phylogenetic reconstruction was performed with RAxML-PTHREADS-SSE3 v7.4.2 (Stamatakis, Alachiotis 2010) by using the -f a and PROTGAMMAIWAGF option and calculating bootstrap support on 1,000 pseudoreplicates. Bootstrap support values are given for all nodes. See the legend of Fig. 3 for further information.

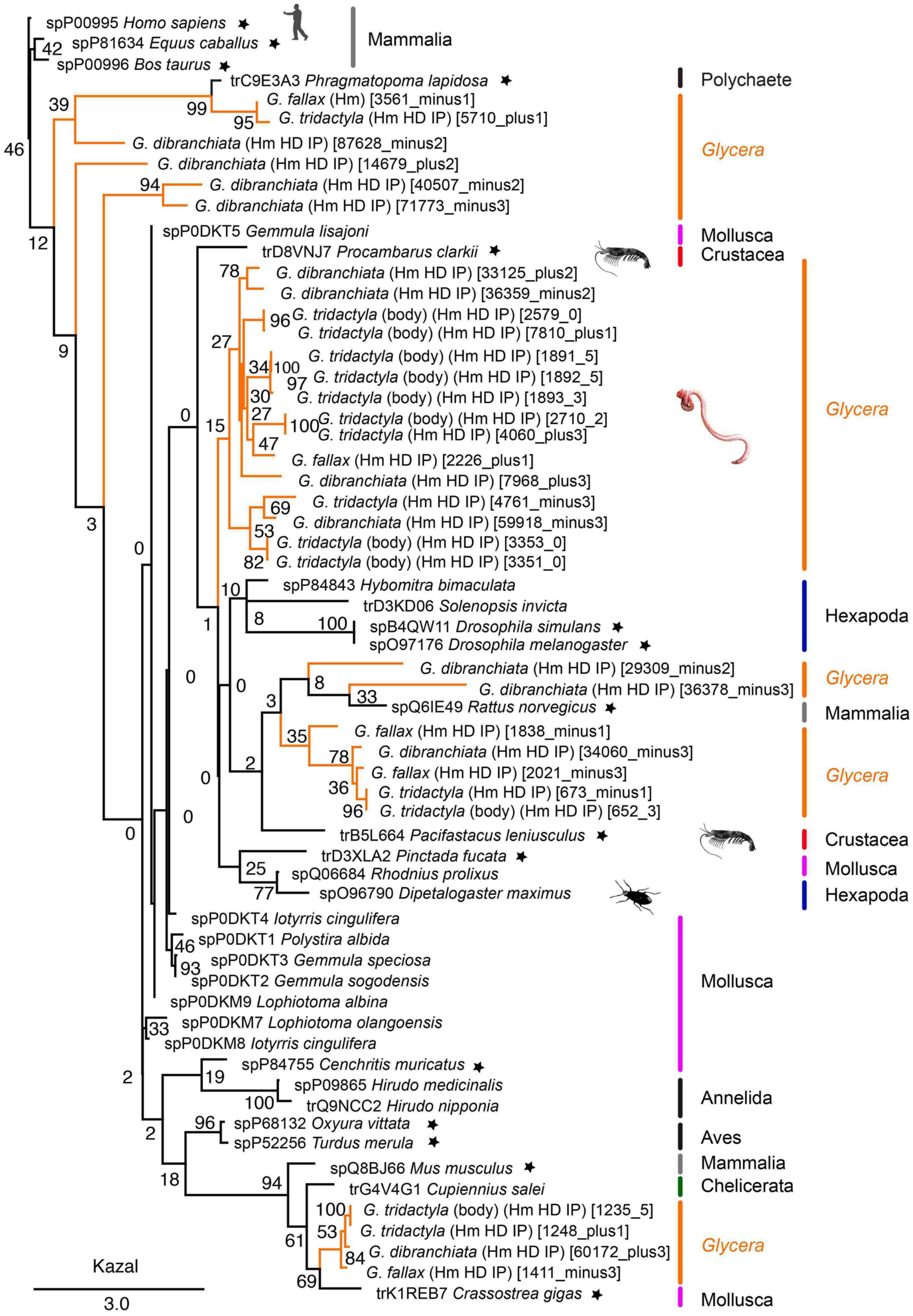
spP00995 Homo sapiens
spP81634 Equus caballus
spP00996 Bos taurus
Mammalia
trC9E3A3 Phragmatopoma lapidosa
Polychaete
G. fallax (Hm) [3561_minus1]
G. tridactyla (Hm HD IP) [5710_plus1]
G. dibranchiata (Hm HD IP) [87628_minus2]
G. dibranchiata (Hm HD IP) [14679_plus2]
G. dibranchiata (Hm HD IP) [40507_minus2]
G. dibranchiata (Hm HD IP) [71773_minus3]
Glycera
spP0DKT5 Gemmula lisajoni
Mollusca
trD8VNJ7 Procambarus clarkii
Crustacea
G. dibranchiata (Hm HD IP) [33125_plus2]
G. dibranchiata (Hm HD IP) [36359_minus2]
G. tridactyla (body) (Hm HD IP) [2579_0]
G. tridactyla (body) (Hm HD IP) [7810_plus1]
G. tridactyla (body) (Hm HD IP) [1891_5]
G. tridactyla (body) (Hm HD IP) [1892_5]
G. tridactyla (body) (Hm HD IP) [1893_3]
G. tridactyla (body) (Hm HD IP) [2710_2]
G. tridactyla (Hm HD IP) [4060_plus3]
G. fallax (Hm HD IP) [2226_plus1]
G. dibranchiata (Hm HD IP) [7968_plus3]
G. tridactyla (Hm HD IP) [4761_minus3]
G. dibranchiata (Hm HD IP) [59918_minus3]
G. tridactyla (body) (Hm HD IP) [3353_0]
G. tridactyla (body) (Hm HD IP) [3351_0]
Glycera
spP84843 Hybomitra bimaculata
trD3KD06 Solenopsis invicta
spB4QW11 Drosophila simulans
spO97176 Drosophila melanogaster
Hexapoda
G. dibranchiata (Hm HD IP) [29309_minus2]
G. dibranchiata (Hm HD IP) [36378_minus3]
Glycera
spQ6IE49 Rattus norvegicus
Mammalia
G. fallax (Hm HD IP) [1838_minus1]
G. dibranchiata (Hm HD IP) [34060_minus3]
G. fallax (Hm HD IP) [2021_minus3]
G. tridactyla (Hm HD IP) [673_minus1]
G. tridactyla (body) (Hm HD IP) [652_3]
Glycera
trB5L664 Pacifastacus leniusculus
Crustacea
trD3XLA2 Pinctada fucata
Mollusca
spQ06684 Rhodnius prolixus
spO96790 Dipetalogaster maximus
Hexapoda
spP0DKT4 Iotyrris cingulifera
spP0DKT1 Polystira albida
spP0DKT3 Gemmula speciosa
spP0DKT2 Gemmula sogodensis
spP0DKM9 Lophiotoma albina
spP0DKM7 Lophiotoma olangoensis
spP0DKM8 Iotyrris cingulifera
Mollusca
spP84755 Cenchritis muricatus
spP09865 Hirudo medicinalis
trQ9NCC2 Hirudo nipponia
Annelida
spP68132 Oxyura vittata
spP52256 Turdus merula
Aves
spQ8BJ66 Mus musculus
Mammalia
trG4V4G1 Cupiennius salei
Chelicerata
G. tridactyla (body) (Hm HD IP) [1235_5]
G. tridactyla (Hm HD IP) [1248_plus1]
G. dibranchiata (Hm HD IP) [60172_plus3]
G. fallax (Hm HD IP) [1411_minus3]
Glycera
trK1REB7 Crassostrea gigas
Mollusca
Kazal
3.0

Supplementary figure S12. Phylogenetic tree of Kazal sequences. Phylogenetic reconstruction was performed with RAxML-PTHREADS-SSE3 v7.4.2 (Stamatakis, Alachiotis 2010) by using the -f a and PROTGAMMAIWAGF option and calculating bootstrap support on 1,000 pseudoreplicates. Bootstrap support values are given for all nodes. See the legend of Fig. 3 for further information.

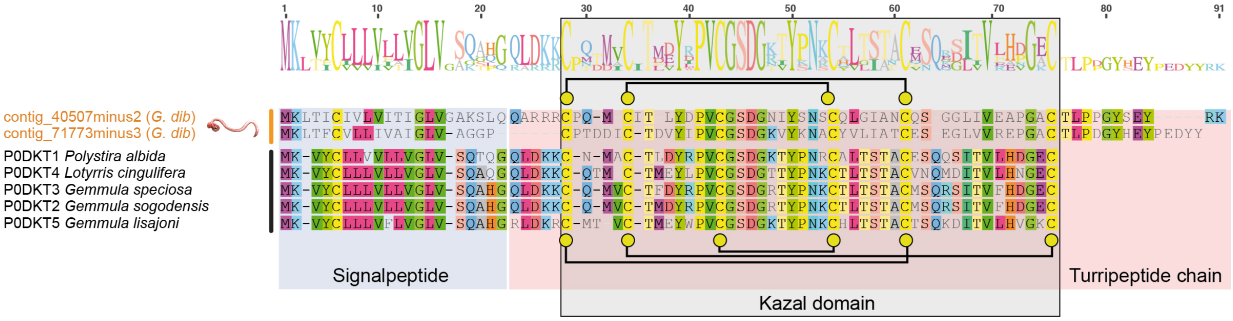

Supplementary figure S13. Multiple sequence alignment of turripeptide-like toxin sequences generated by MAFFT-L-INS-i (Katoh, Standley 2013). The alignment contains two *Glycera* sequences and five sequences from turrid molluscs. The predicted pattern of disulfide bonds for the *Glycera* peptides is indicated above the alignment, those for the mollusc sequences below it.

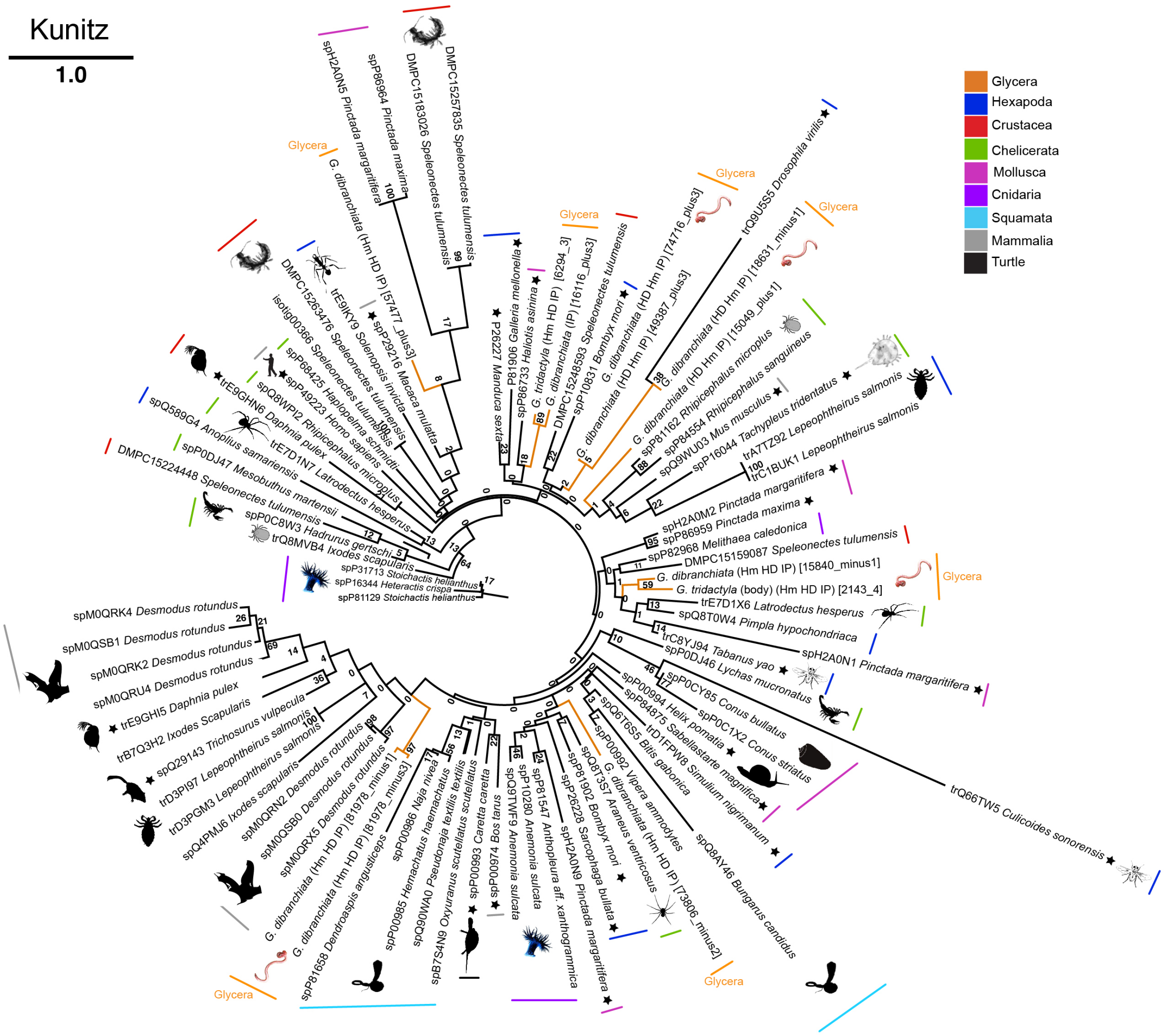

Supplementary figure S14. Phylogenetic tree of Kunitz sequences. Phylogenetic reconstruction was performed with RAxML-PTHREADS-SSE3 v7.4.2 (Stamatakis, Alachiotis 2010) by using the -f a and PROTGAMMAIWAG option and calculating bootstrap support on 1,000 pseudoreplicates. Bootstrap support values are given for all nodes. See the legend of Fig. 3 for further information.

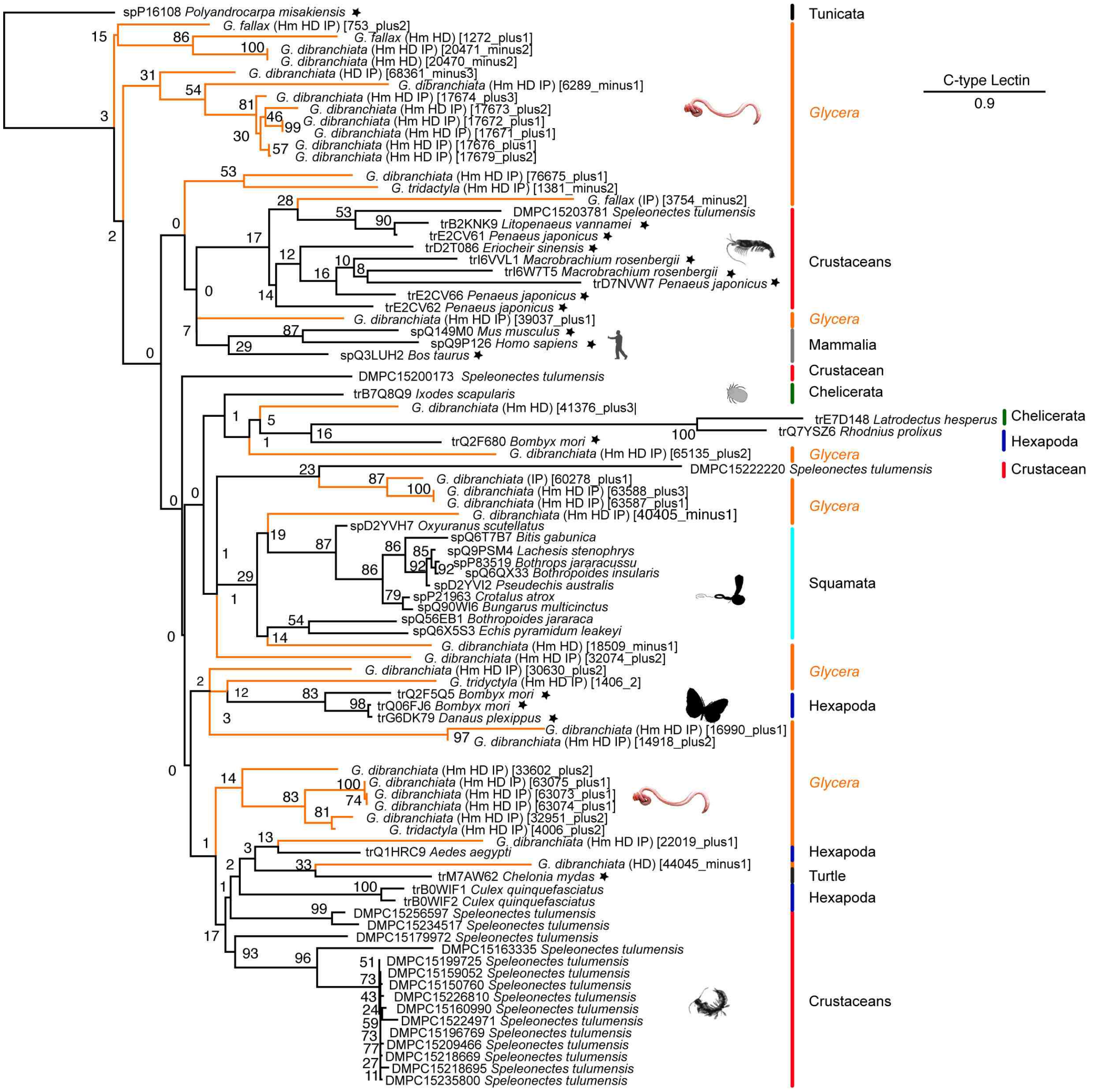

Supplementary figure S15. Phylogenetic tree of C-type lectin sequences. Phylogenetic reconstruction was performed with RAxML-PTHREADS-SSE3 v7.4.2 (Stamatakis, Alachiotis 2010) by using the -f a and PROTGAMMAIWAGF option and calculating bootstrap support on 1,000 pseudoreplicates. Bootstrap support values are given for all nodes. See the legend of Fig. 3 for further information.

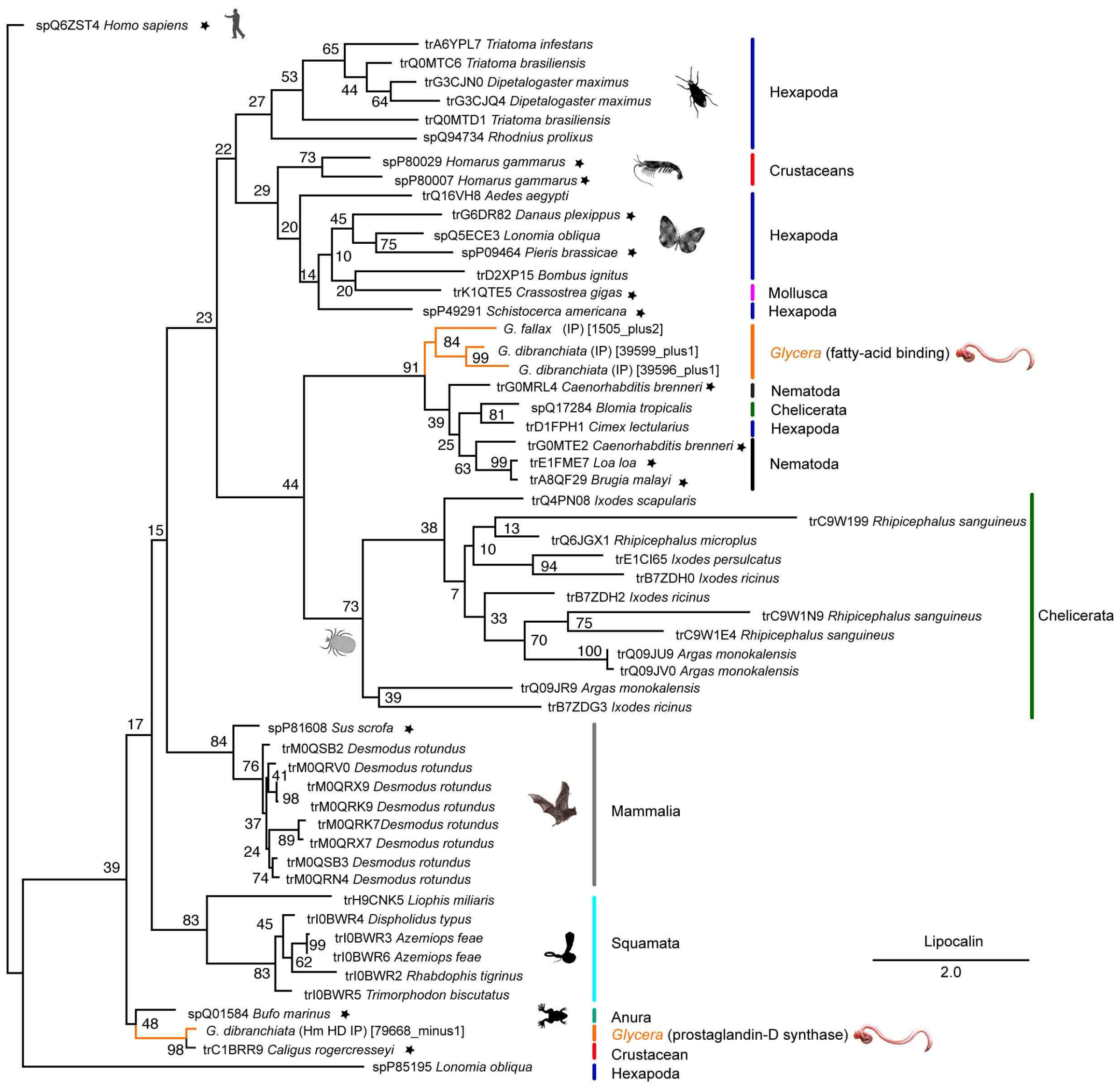

Supplementary figure S16. Phylogenetic tree of lipocalin sequences. Phylogenetic reconstruction was performed with RAxML-PTHREADS-SSE3 v7.4.2 (Stamatakis, Alachiotis 2010) by using the -f a and PROTGAMMAIWAGF option and calculating bootstrap support on 1,000 pseudoreplicates. Bootstrap support values are given for all nodes. See the legend of Fig. 3 for further information.

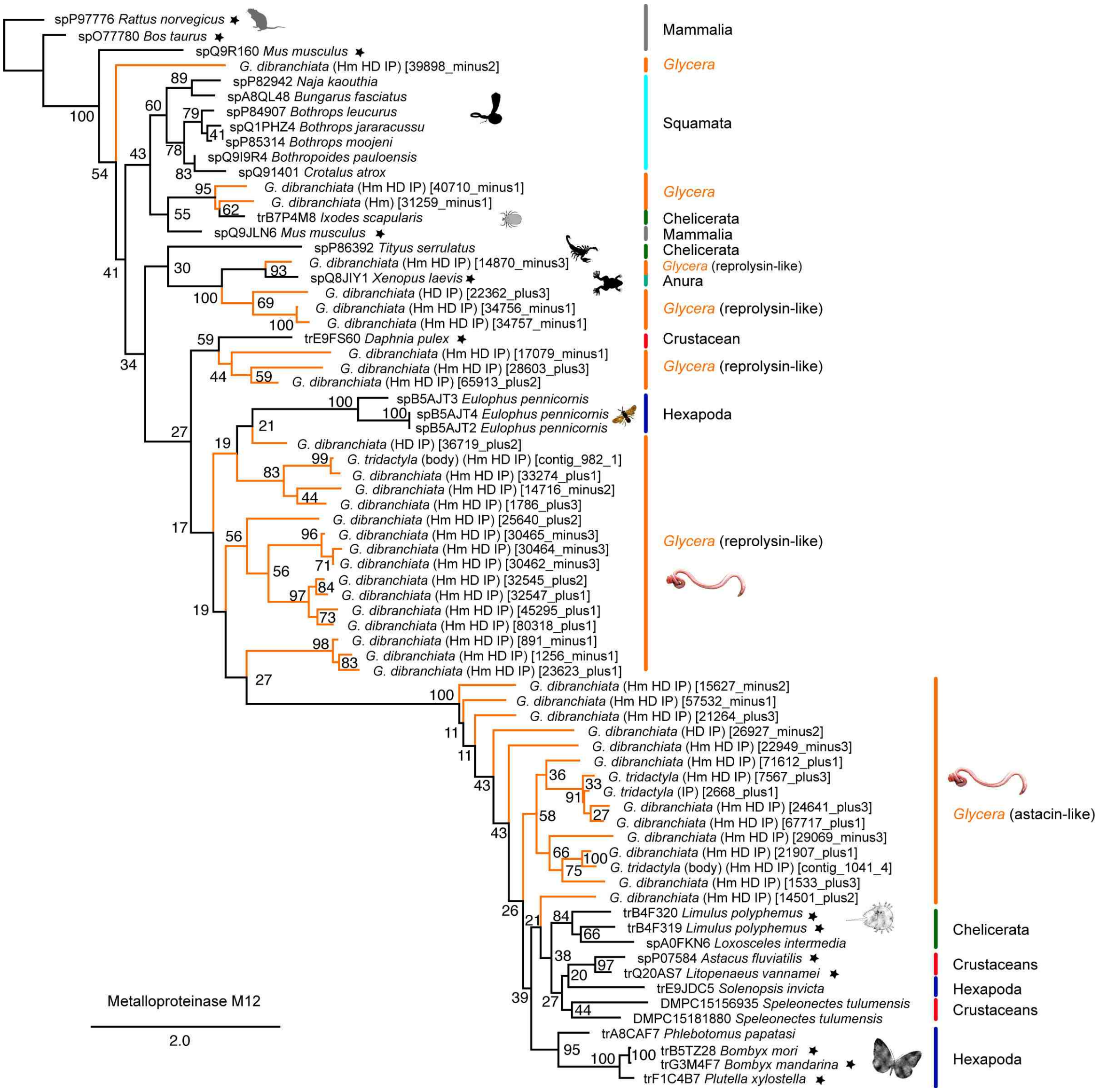

Supplementary figure S17. Phylogenetic tree of metalloproteinase M12 sequences. Phylogenetic reconstruction was performed with RAxML-PTHREADS-SSE3 v7.4.2 (Stamatakis, Alachiotis 2010) by using the -f a and PROTGAMMAIBLOSUM62F option and calculating bootstrap support on 1,000 pseudoreplicates. Bootstrap support values are given for all nodes. See the legend of Fig. 3 for further information.

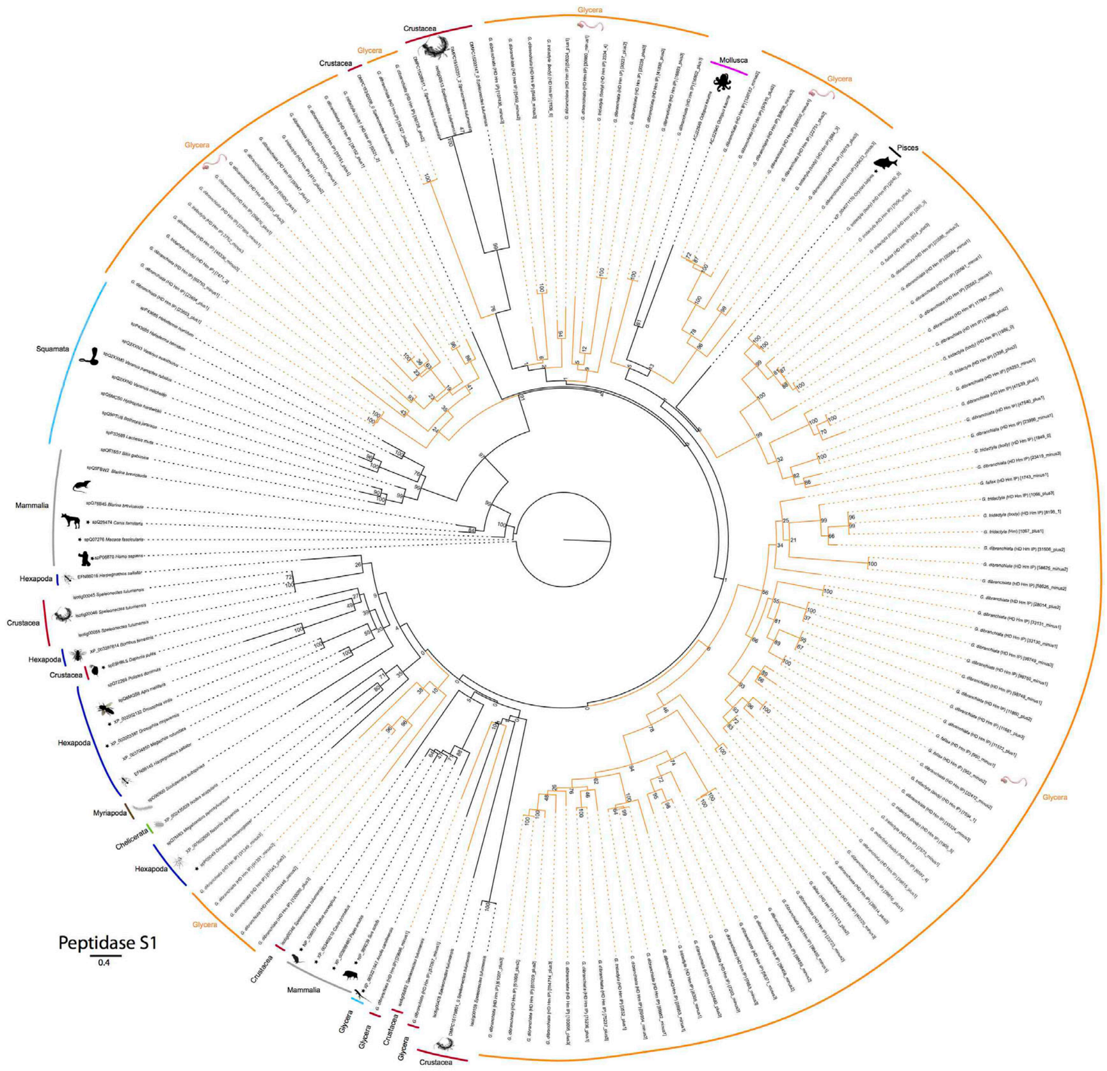

Supplementary figure S18. Phylogenetic tree of peptidase S1 sequences. Phylogenetic reconstruction was performed with RAxML-PTHREADS-SSE3 v7.4.2 (Stamatakis, Alachiotis 2010) by using the -f a and PROTGAMMAILG option and calculating bootstrap support on 1,000 pseudoreplicates. Bootstrap support values are given for all nodes. See the legend of Fig. 3 for further information.

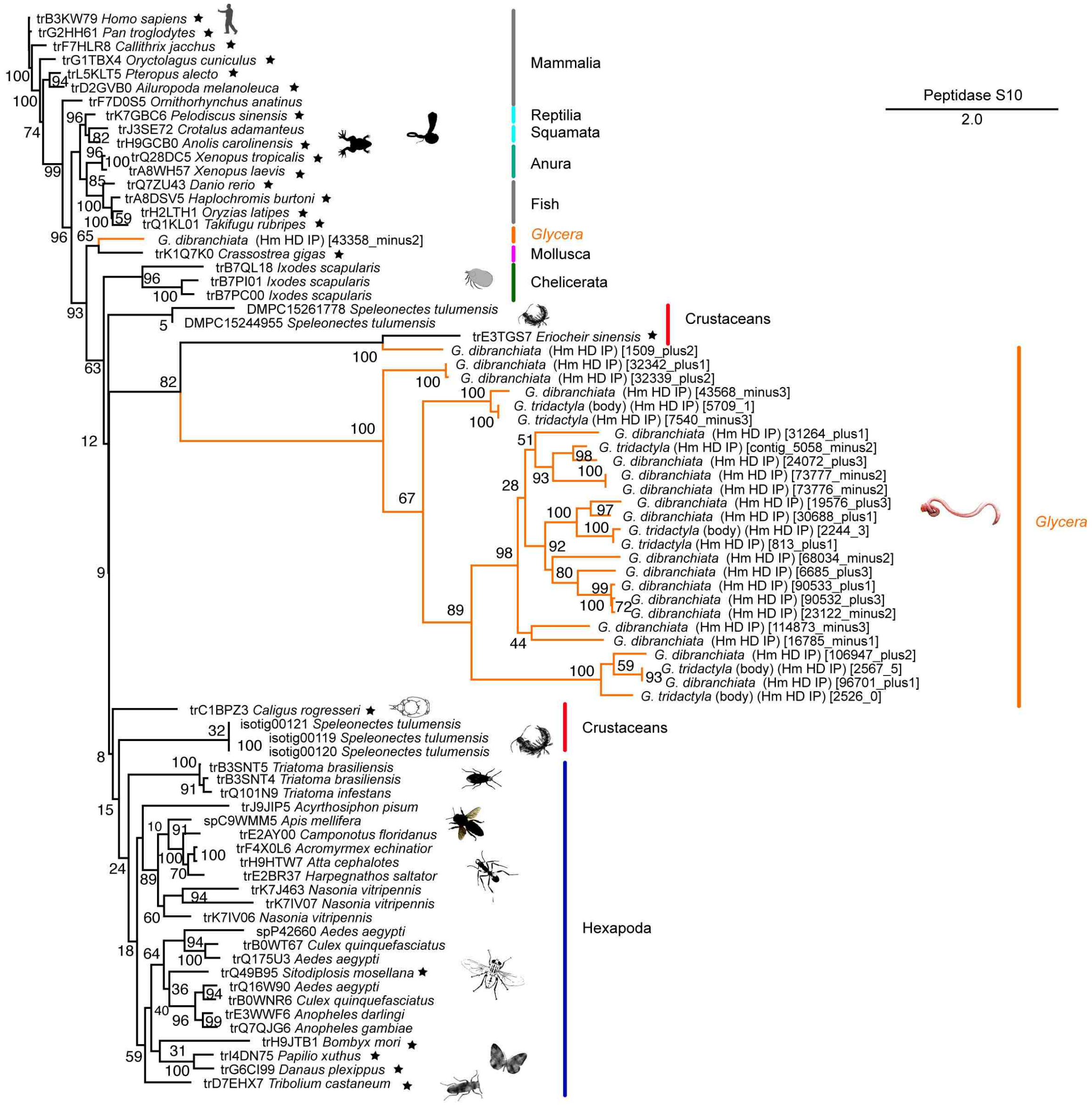

Supplementary figure S19. Phylogenetic tree of peptidase S10 sequences. Phylogenetic reconstruction was performed with RAxML-PTHREADS-SSE3 v7.4.2 (Stamatakis, Alachiotis 2010) by using the -f a and PROTGAMMAILG option and calculating bootstrap support on 1,000 pseudoreplicates. Bootstrap support values are given for all nodes. See the legend of Fig. 3 for further information.

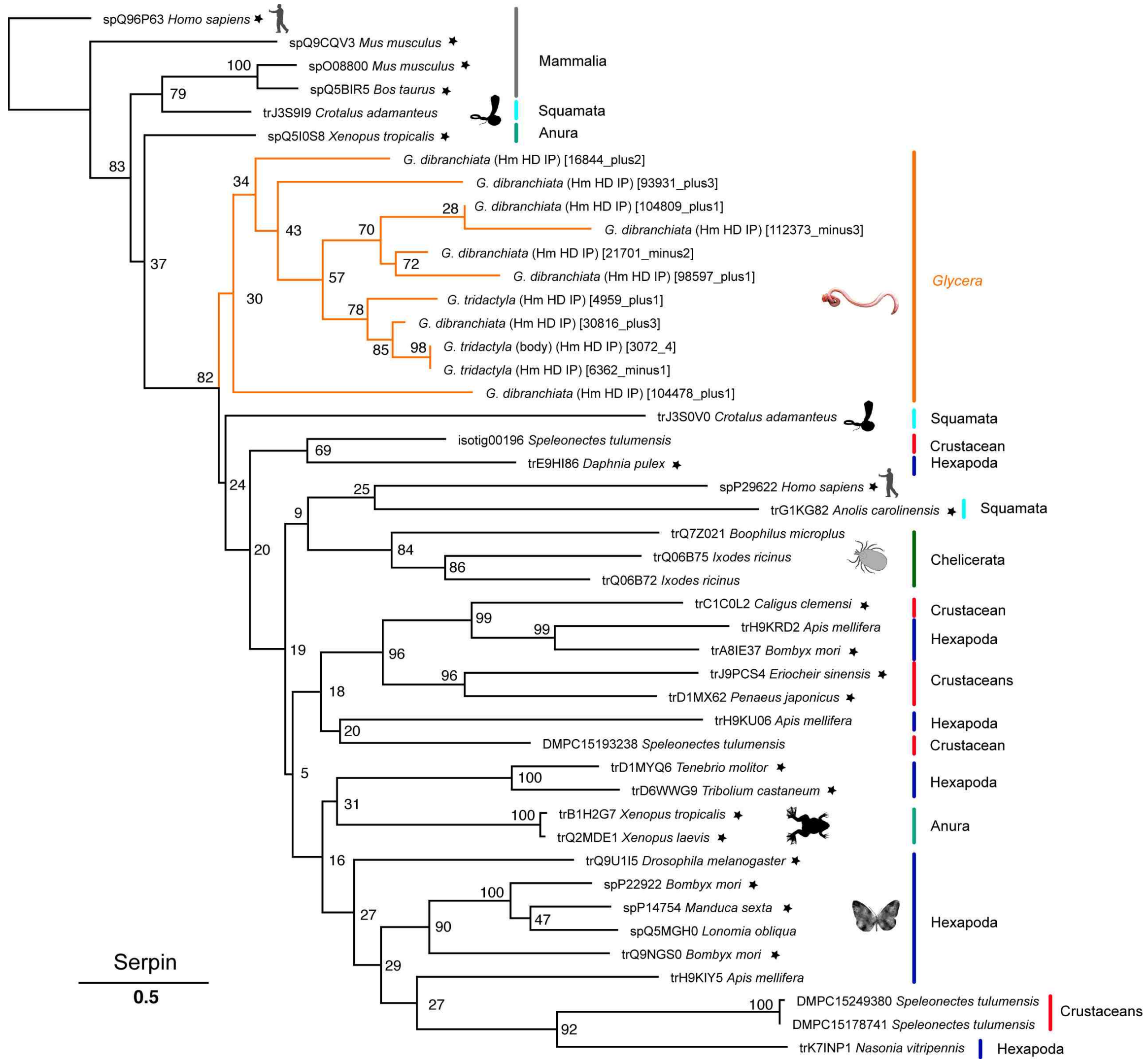

Supplementary figure S20. Phylogenetic tree of serpin sequences. Phylogenetic reconstruction was performed with RAxML-PTHREADS-SSE3 v7.4.2 (Stamatakis, Alachiotis 2010) by using the -f a and PROTGAMMAILGF option and calculating bootstrap support on 1,000 pseudoreplicates. Bootstrap support values are given for all nodes. See the legend of Fig. 3 for further information.

References

Aminetzach Y, Srouji J, Kong C, Hoekstra H. 2009. Convergent evolution of novel protein function in shrew and lizard venom. Curr Biol. 19:1925-1931.
Bishop RE. 2000. The bacterial lipocalins. BBA-Protein Struct M 1482:73-83.
Bobek LA, Levine MJ. 1992. Cystatins - Inhibitors of cysteine proteinases. Crit Rev Oral Biol M 3:307-332.
Cabang AB, et al. 2011. Characterization of a venom peptide from a crassispirid gastropod. Toxicon 58:672-680.
Chagas AC, et al. 2013. A deep insight into the sialotranscriptome of the mosquito, Psorophora albipes. BMC Genomics 14:875.
Chmelar J, et al. 2011. A tick salivary protein targets cathepsin G and chymase and inhibits host inflammation and platelet aggregation. Blood 117:736-744.
Colinet D, et al. 2013. Extensive inter- and intraspecific venom variation in closely related parasites targeting the same host: The case of *Leptopilina* parasitoids of *Drosophila*. Insect Biochem Molec 43:601-611.
Dai S-X, Zhang A-D, Huang J-F. 2012. Evolution, expansion and expression of the Kunitz/BPTI gene family associated with long-term blood feeding in *Ixodes scapularis*. BMC Evol Biol. 12.
De Graaf DC, et al. 2010. Insights into the venom composition of the ectoparasitoid wasp *Nasonia vitripennis* from bioinformatic and proteomic studies. Insect Mol Biol. 19:11-26.
Dorémus T, et al. 2013. Venom gland extract is not required for successful parasitism in the polydnavirus-associated endoparasitoid *Hyposoter didymator* (Hym. Ichneumonidae) despite the presence of numerous novel and conserved venom proteins. Insect Mol Biol 43:292-307.
Durban J, et al. 2011. Profiling the venom gland transcriptomes of Costa Rican snakes by 454 pyrosequencing. BMC Genomics 12.
Fernandes-Pedrosa M, et al. 2008. Transcriptome analysis of *Loxosceles laeta* (Araneae, Sicariidae) spider venomous gland using expressed sequence tags. BMC Genomics 9:279.
Flower DR. 1996. The lipocalin protein family: structure and function. Biochem J. 318:1-14.
Flower DR, North ACT, Attwood TK. 1993. Structure and sequence relationships in the lipocalins and related proteins. Protein Sci. 2:753-761.
Flower DR, North ACT, Sansom CE. 2000. The lipocalin protein family: structural and sequence overview. BBA-Protein Struct M 1482:9-24.
Fox JW. 2013. A brief review of the scientific history of several lesser-known snake venom proteins: L-amino acid oxidases, hyaluronidases and phosphodiesterases. Toxicon 62:75-82.
Fry B, et al. 2009. The toxicogenomic multiverse: convergent recruitment of proteins into animal venoms Annu Rev Genom Hum G. 10:483-511.
Fry BG, Scheib H, Junqueira de Azevedo IdLM, Silva DA, Casewell NR. 2012. Novel transcripts in the maxillary venom glands of advanced snakes. Toxicon 59:696-708.
Funkhouser JD, Aronson NN, Jr. 2007. Chitinase family GH18: evolutionary insights from the genomic history of a diverse protein family. BMC Evol Biol. 7.

Gibbs GM, Roelants K, O'Bryan MK. 2008. The CAP Superfamily: cysteine-rich secretory proteins, antigen 5, and pathogenesis-related 1 proteins-roles in reproduction, cancer, and immune defense. Endocr Rev. 29:865-897.

Gutierrez G, Ganfornina MD, Sanchez D. 2000. Evolution of the lipocalin family as inferred from a protein sequence phylogeny. BBA-Protein Struct M 1482:35-45.

Heralde III F, et al. 2008. A rapidly diverging superfamily of peptide toxins in venomous *Gemmula* species. Toxicon 51:890-897.

Huson D, Bryant D. 2006. Application of phylogenetic networks in evolutionary studies. Mol Biol Evol. 23:254-267.

Kim BY, et al. 2013. Antimicrobial activity of a honeybee (*Apis cerana*) venom Kazal-type serine protease inhibitor. Toxicon 76:110-117.

Klawe WL, Dickie LM. 1957. Biology of the bloodworm, *Glycera dibranchiata* Ehlers, and its relation to the bloodworm fishery of the Maritime Provinces. B Fish Res Board Can. 115.

Kunitz M, Northrop JH. 1936. Isolation from beef pancreas of crystalline trypsinogen, trypsin, a trypsin inhibitor, and an inhibitor-trypsin compound. J Gen Physiol. 19:991-1007.

Kvist S, Brugler MR, Goh TG, Giribet G, Siddall ME. 2014. Pyrosequencing the salivary transcriptome of *Haemadipsa interrupta* (Annelida: Clitellata: Haemadipsidae): anticoagulant diversity and insight into the evolution of anticoagulation capabilities in leeches. Invertebr Biol 133:74-98.

Lacy DB, Tepp W, Cohen AC, DasGupta BR, Stevens RC. 1998. Crystal structure of botulinum neuro-toxin type A and implications for toxicity. Nat Struct Biol. 5:898-902.

Laskowski MJ, Kato I. 1980. Protein inhibitors of proteinases. Annu Rev Biochem. 49:593-626.

Li R, et al. 2013. Proteome and phosphoproteome analysis of honeybee (*Apis mellifera*) venom collected from electrical stimulation and manual extraction of the venom gland. BMC Genomics 14:766.

Liu Z-C, et al. 2012. Venomic and transcriptomic analysis of centipede *Scolopendra subspinipes dehaani*. J Proteome Res. 11:6197-6212.

Low DHW, et al. 2013. Dracula's children: molecular evolution of vampire bat venom. J Proteomics 89:95-111.

Mans BJ, Neitz AWH. 2004a. Adaptation of ticks to a blood-feeding environment: evolution from a functional perspective. Insect Biochem Molec. 34:1-17.

Mans BJ, Neitz AWH. 2004b. Exon-intron structure of outlier tick lipocalins indicate a monophyletic origin within the larger lipocalin family. Insect Biochem Molec. 34:585-594.

Meunier FA, Feng ZP, Molgo J, Zamponi GW, Schiavo G. 2002. Glycerotoxin from *Glycera convoluta* stimulates neurosecretion by up-regulating N-type Ca2+ channel activity. EMBO J. 21:6733-6743.

Michel C. 1970. Rôle physiologique de la trompe chez quatre annelides polychètes appartenant aux genres: *Eulalia*, *Phyllodoce*, *Glycera* et *Notomastus*. Cah. Biol. Mar. 11:209-228.

Min G-S, Sarkar IN, Siddall ME. 2010. Salivary transcriptome of the North American medicinal leech, *Macrobdella decora*. J Parasitol. 96:1211-1221.

Moran Y, et al. 2013. Analysis of soluble protein contents from the nematocysts of a model sea anemone sheds light on venom evolution. Mar Biotechnol. 15:329-339.

Mourao CBF, Schwartz EF. 2013. Protease inhibitors from marine venomous animals and their counterparts in terrestrial venomous animals. Mar Drugs 11:2069-2112.

Ockelmann KW, Vahl O. 1970. On the biology of the polychaete *Glycera alba*, especially its burrowing and feeding. Ophelia 8:275-294.

Olivera BM, et al. 2012. Adaptive radiation of venomous marine snail lineages and the accelerated evolution of venom peptide genes. AnnNY Acad Sci. 1267:61-70.

Olsnes S, Refsnes K, Pihl A. 1974. Mechanism of action of toxic lectins abrin and ricin. Nature 249:627-631.

Pugalenthi G, Kandaswamy KK, Suganthan PN, Archunan G, Sowdhamini R. 2010. Identification of functionally diverse lipocalin proteins from sequence information using support vector machine. Amino Acids 39:777-783.

Reis CV, et al. 2006. Lopap, a prothrombin activator from *Lonomia obliqua* belonging to the lipocalin family: recombinant production, biochemical characterization and structure-function insights. Biochem J. 398:295-302.

Retiere C. 1967. Place du Spionidae *Nerine cirratulus* (Delle Chiaje) dans les sables medio-littoraux de la plage de Lancieux (Coted-du-Nord). Interactions alimentaires des differents especes du groupement anelidien. Bull Soc Scient Bretagne 42:39-47.

Rokyta DR, Lemmon AR, Margres MJ, Aronow K. 2012. The venom-gland transcriptome of the eastern diamondback rattlesnake (*Crotalus adamanteus*). BMC Genomics 13:312.

Ruder T, et al. 2013. Molecular phylogeny and evolution of the proteins encoded by coleoid (cuttlefish, octopus, and squid) posterior venom glands. J Mol Evol. 76:192-204.

Schwarz A, Cabezas-Cruz A, Kopecky J, Valdes JJ. 2014. Understanding the evolutionary structural variability and target specificity of tick salivary Kunitz peptides using next generation transcriptome data. BMC Evol Biol. 14.

Schweitz H, et al. 1994. Calcicludine, a venom peptide of the Kunitz-type protease inhibitor family, is a potent blocker of high-threshold Ca^{2+} channels with a high affinity for L-type channels in cerebellar granule neurons. P Natl Acad Sci USA 91:878-882.

Stamatakis A, Alachiotis N. 2010. Time and memory efficient likelihood-based tree searches on phylogenomic alignments with missing data. Bioinformatics 26:i132-i139.

Stotz SC, Spaetgens RL, Zamponi GW. 2000. Block of voltage-dependent calcium channel by the green mamba toxin calcicludine. J Membr Biol. 174:157-165.

Sunagar K, Johnson WE, O'Brien SJ, Vasconcelos V, Antunes A. 2012. Evolution of CRISPs associated with toxicoferan-reptilian venom and mammalian reproduction. Mol Biol Evol. 29:1807-1822.

Veiga ABG, Ribeiro JMC, Guimarães JA, Francischetti IMB. 2005. A catalog for the transcripts from the venomous structures of the caterpillar *Lonomia obliqua*: Identification of the proteins potentially involved in the coagulation disorder and hemorrhagic syndrome. Gene 355:11-27.

von Reumont BM, et al. 2014. The first venomous crustacean revealed by transcriptomics and functional morphology: remipede venom glands express a unique toxin cocktail dominated by enzymes and a neurotoxin. Mol Biol Evol. 31:48-58.

Vonk FJ, et al. 2013. The king cobra genome reveals dynamic gene evolution and adaptation in the snake venom system. P Natl Acad Sci USA 110:20651-20656.

Watkins M, Hillyard DR, Olivera BM. 2006. Genes expressed in a turrid venom duct: divergence and similarity to conotoxins. J Mol Evol. 62:247-256.

Waxman L, Connolly TM. 1993. Isolation of an inhibitor selective for collagen-stimulated platelet-aggregation from the soft tick *Ornithodoros moubata*. J Biol Chem. 268:5445-5449.

Wei CB, Chen J. 2012. A novel lipocalin homologue from the venom gland of *Deinagkistrodon acutus* similar to mammalian lipocalins. J Venom Anim Toxins 18:16-23.

Whittington CM, et al. 2010. Novel venom gene discovery in the platypus. Genome Biol. 11:R95.

Wong ESW, et al. 2012. Proteomics and deep sequencing comparison of seasonally active venom glands in the platypus reveals novel venom peptides and distinct expression profiles. Mol Cell Proteomics 11:1354-1364.

Wong ESW, Nicol S, Warren WC, Belov K. 2013. Echidna venom gland transcriptome provides insights into the evolution of monotreme venom. Plos One 8.

Wong ESW, Papenfuss AT, Whittington CM, Warren WC, Belov K. 2011. A limited role for gene duplications in the evolution of platypus venom. Mol Biol Evol. 29:167-177.

Wu J, et al. 2011. Proteomic analysis of skin defensive factors of tree frog *Hyla simplex*. J Proteome Res 10:4230-4240.

Yamazaki Y, Morita T. 2004. Structure and function of snake venom cysteine-rich secretory proteins. Toxicon 44:227-231.

Zelensky AN, Gready JE. 2005. The C-type lectin-like domain superfamily. FEBS J. 272:6179-6217.
